# Synthesis and Vibrational Circular Dichroism Analysis of *N*-Heterocyclic Carbene Precursors Containing Remote Chirality Centers

**DOI:** 10.3390/ijms23073471

**Published:** 2022-03-23

**Authors:** Zita Szabó, Attila Paczal, Tibor Kovács, Attila Mándi, Andras Kotschy, Tibor Kurtán

**Affiliations:** 1 Servier Research Institute of Medicinal Chemistry, Záhony u. 7., 1031 Budapest, Hungary; szabo.zita.2109@gmail.com (Z.S.); attila.paczal@servier.com (A.P.); 2 Department of Organic Chemistry, University of Debrecen, P.O. Box 400, 4002 Debrecen, Hungary; kovacs.tibor@science.unideb.hu (T.K.); mandi.attila@science.unideb.hu (A.M.)

**Keywords:** DFT calculations, characteristic VCD transitions, electronic circular dichroism, conformational analysis, NHC precursors

## Abstract

VCD analysis of 16 diastereomeric pairs of NHC precursors containing two isolated chirality centers and different substitution patterns identified VCD transitions characteristic of the chirality center in the imidazolium ring or in the side chain, which, in contrast to ECD and OR, could be utilized to assign the two chirality centers separately by simple comparison, regardless of the type and position of achiral aromatic substituents. While the ECD and OR data showed great dependence on the position of an achiral substituent such as a methoxy group, characteristic experimental VCD transitions remained consistent and they could be used to determine the absolute configuration of all the regio- and stereoisomers and substituted analogues. VCD, ECD and OR approaches were evaluated, and several carbene precursors were found, for which only the VCD method could distinguish the four stereoisomers. With *t*-butyl, phenyl or 2-naphthyl substituents at the C-1′ chirality center, the ECD spectra of the C-1′ epimers were near-identical, and hence it was only the VCD approach that showed distinct differences suitable for the configurational assignment. The chiroptical characterization of our diastereomeric pairs of NHC precursors enables the future application of related derivatives having different substitution patterns in stereoselective transformations.

## 1. Introduction

**Figure 1:**
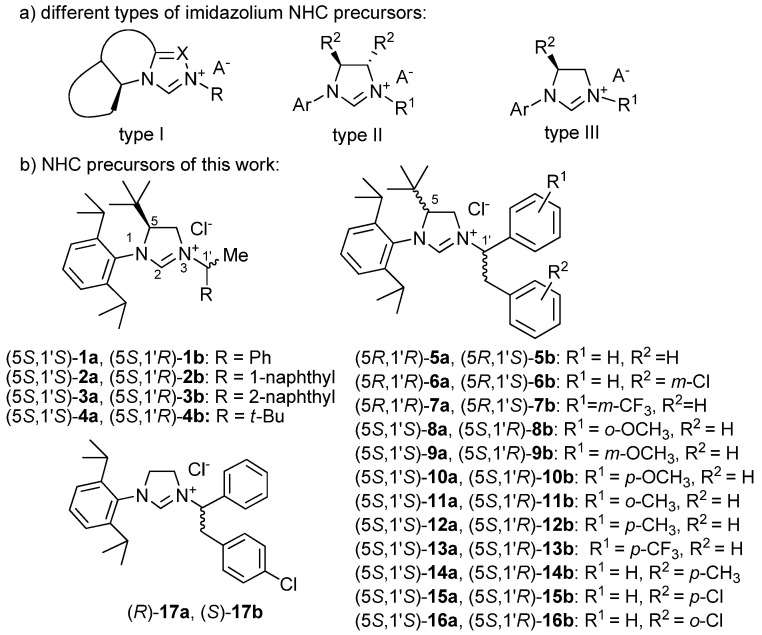
(**a**) Different types of chiral non-racemic imidazolium NHC precursors. (**b**) Structures of the NHC precursors included in the present study.

Chiral *N*-heterocyclic carbenes (NHCs) have received considerable attention in asymmetric synthesis due to the stability and catalytic activity of their transition metal complexes [1,2,3,4], and their ability to act as organocatalyst [5]. The first report of an imidazole-based NHC by Arduengo [6,7] initiated a rapid development aided by the easily tunable electronic and steric properties of NHC ligands through structural modifications [8,9,10,11,12,13,14,15,16,17,18]. The majority of early chiral NHC precursors contained the central chirality element in the NHC core (type I, Figure 1), sometimes embedded into a condensed ring system where triazolium and thiazolium rings frequently replaced the imidazolium unit [19,20,21,22,23]. The 4,5-disubstituted 4,5-dihydro-1*H*-imidazol-3-ium salts (type II, Figure 1) are the most prominent representatives of the class, in which the C-4 and C-5 substituents show a *C*_2_-symmetry, and further asymmetry can be introduced through the different substituents of the ring nitrogens [24,25,26,27,28]. Although less common, 4,5-dihydro-1*H*-imidazol-3-ium salts bearing different substituents at C-4 and C-5 (type III, Figure 1) were also reported [29,30,31,32,33]. Herein, we report the stereoselective synthesis of NHC stereoisomers **6–17a**,**b** and the chiroptical analysis of **1–17a,b** containing remote chirality centers at C-5 of the imidazolium core and C-1′ of the side chain, the relative configuration of which could not be determined by NMR experiments (Figure 1). The absolute configuration (AC) had been determined for some of these NHC precursors such as (5*R*,1′*R*)-**5a** and (5*R*,1′*S*)-**5b**, retrospectively, on the basis of single-crystal X-ray diffraction analysis of their gold(I) complexes [34]. However, chiroptical data (electronic and vibrational circular dichroism (ECD, VCD), optical rotation) of the NHC precursors were not measured and correlated with the stereochemistry, which would have enabled direct configurational assignment of the synthetic samples. Although there are a few reports on the ECD analysis of NHC precursors and their metal complexes [35,36,37,38], the VCD approach has not been applied yet to investigate their stereochemistry. Due to the relatively large number of characteristic vibrational transitions associated with the different stereogenic elements, vibrational circular dichroism assisted by DFT calculations was found to be a powerful tool to distinguish more than two stereoisomers for compounds of natural [39,40,41,42] or synthetic origin [43,44,45,46].

DFT VCD calculations allowed us to correlate the distinct VCD transitions of diastereomeric NHC precursors having different substitution patterns of the 1,2-disubstituted-*N*-ethyl side chain with the chirality centers of the dihydroimidazolium unit and the side chain, on the basis of which simple comparison of the experimental VCD transitions could be utilized to distinguish the diastereomers. VCD analysis usually requires the DFT calculation of the experimental curve and there are quite a limited number of examples where the recorded VCD curves of structurally related derivatives can be used for configurational assignment through simple comparison [47,48,49,50]. In contrast, experimental ECD spectra of structurally similar derivatives are frequently compared and utilized for the determination of AC, which was also promoted by semiempirical ECD rules such as the helicity rules of the condensed benzene chromophore [51]. We identified several NHC precursors where near-identical ECD and optical rotation (OR) data could not distinguish the diastereomers even with the aid of calculations, while the characteristic VCD transitions can be used efficiently. We found that the different positions of an achiral aromatic substituent with large spectroscopic moment such as a methoxy group can alter significantly both the ECD and OR values of the homochiral regioisomers, while the VCD transitions remain consistent and they can be used for the assignment by simple comparison. We performed a systematic VCD, ECD and OR analysis of stereoisomeric NHC precursors having versatile substitution patterns to evaluate the scope of the different methods and VCD was found suitable to distinguish and assign all the studied isomers.

## 2. Results and Discussion

**Scheme 1:**
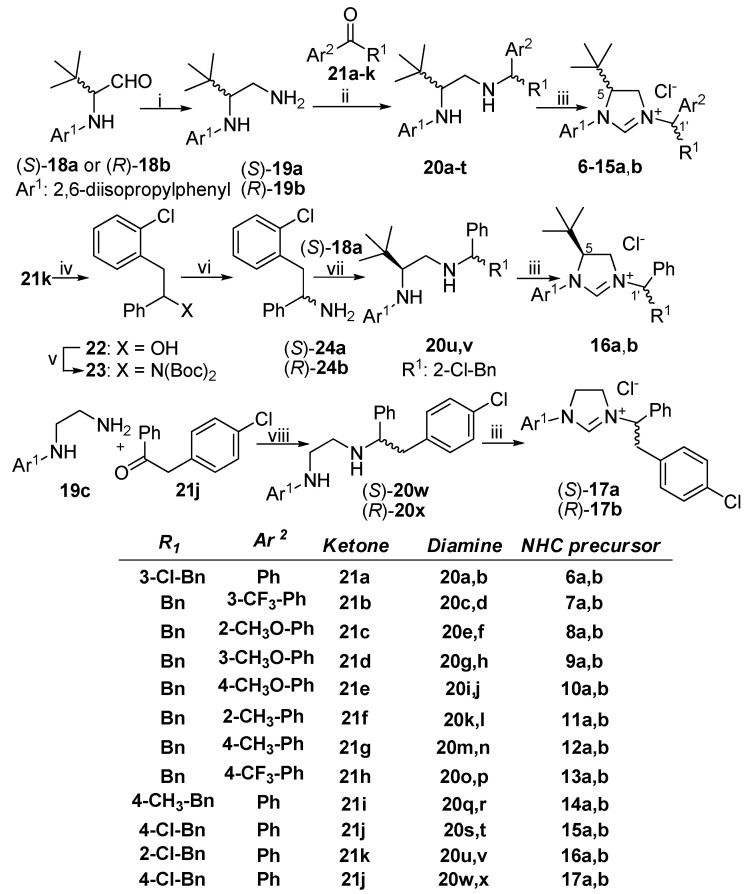
Synthesis of optically active NHC precursors. i: (a) NH_2_OH·HCl, DIPEA, THF, rt; (b) LAH (73%); ii: (a) cat. AcOH, PhMe, reflux; (b) NaBH_4_, MeOH, rt (5–88%); (c) separation of diastereomers; iii: HC(OEt)_3_, NH_4_Cl (40–98%); iv: LAH, THF, 0 °C, (92%); v: Boc_2_NH, DTAD, PPh_3_, toluene, 50 °C, (48%); vi: (a) TFA, DCM, rt (68%); (b) separation of enantiomers; vii: (a) cat. AcOH, EtOH, 60 °C; (b) NaBH_4_, rt (41–88%); viii: (a) cat. AcOH, PhMe, reflux; (b) NaBH_4_, MeOH, rt; (c) separation of enantiomers (22–24%).

The synthesis of optically active NHC precursors **1–2a,b**, **4–5a,b** and **15a,b**, as well as their conversion to gold(I) NHC complexes was reported earlier [34]. These procedures utilized the optically active diamines (*S*)-**19a** and (*R*)-**19b** as key intermediates (Scheme 1), which were obtained previously from (*S*)-**18a** and (*R*)-**18b** in three steps with an overall yield of 53% using 1-(4-methoxyphenyl)methanamine as an ammonia surrogate [34]. For the synthesis of NHC precursors **6–16a,b** having different substitution of the C-1′ chirality center, we simplified and improved the transformation of **18** to **19** by employing hydroxylamine and reduction of the oxime intermediate without isolation. The introduction of the 1,2-disubstituted ethyl side chain was achieved through reductive amination with selected aryl benzyl ketones (**21a–k**), prepared in the reaction of the appropriate Weinreb amides and Grignard reagents. Schiff base formation with the ketones **21a–k** followed by reduction with sodium borohydride afforded the chiral diamines **20a–v** as a mixture of diastereoisomers that were separated by flash chromatography (**20a–h,k,l,q–t**) or chiral preparative HPLC (**20i–j,m–p**).

At this stage, the AC of the ethylenediamine moiety was defined by the AC of the **18a,b** precursor, but the relative configuration of the side chain could not be determined by experimental NMR methods. Since we were unable to separate the diastereoisomers **20u,v** even by chiral preparative HPLC, we converted the ketone **21k** to the racemic amine **24** in 3 steps (Scheme 1) through a reduction-Mitsunobu amination–deprotection sequence (**21k** → **22** → **23** → **24a,b**). After the chiral HPLC separation of the enantiomers (*S*)-**24a** and (*R*)-**24b**, they were condensed separately with (*S*)-**18a** and reduced with NaBH_4_ to produce the diastereoisomeric ethylenediamine pair **20u,v**. For the synthesis of the enantiomeric NHC precursors (*S*)-**17a**, (*R*)-**17b** lacking the C-5 chirality center, the racemic mixture **20w**,**x** was prepared from diamine **19c** and ketone **21j** and enantiomers were separated by chiral preparative HPLC. The final step of the synthesis was the cyclization of the optically active diamines **20a-x** with triethyl orthoformate to afford 12 diastereomeric pairs of NHC precursors (**6a,b–17a,b**).

The (5*R*,1′*R*)-**5a** and (5*R*,1′*S*)-**5b** epimeric NHC precursors containing C-1′ phenyl and benzyl substituents served as reference compounds for the VCD analysis of the analogues **6–16a,b** (Figure 1), which had different substitution patterns in the phenyl and benzyl groups and different AC at the C-5 and C-1′ chirality centers.

**Figure 2:**
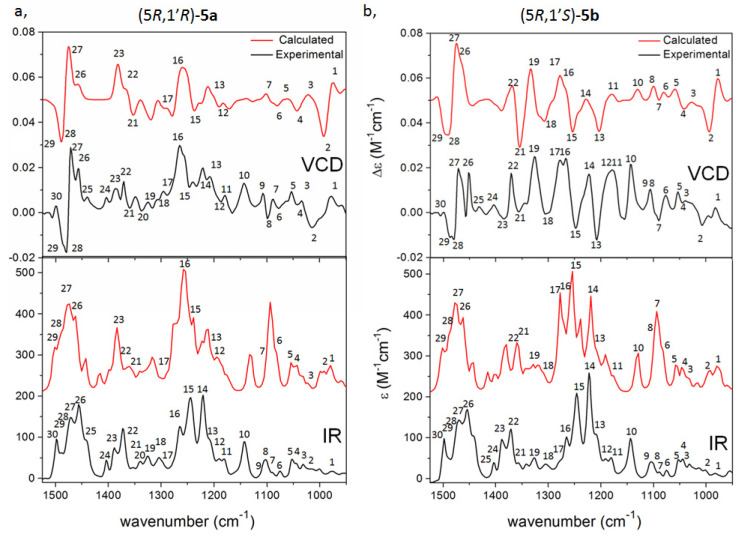
Experimental VCD (upper black, CDCl_3_) and IR spectra (lower black, CDCl_3_) of (**a**) (5*R*,1′*R*)-**5a** and (**b**) (5*R*,1′*S*)-**5b** compared with the B3LYP/TZPV PCM/CHCl_3_ VCD (upper red) and IR (lower red) spectra.

**Figure 3:**
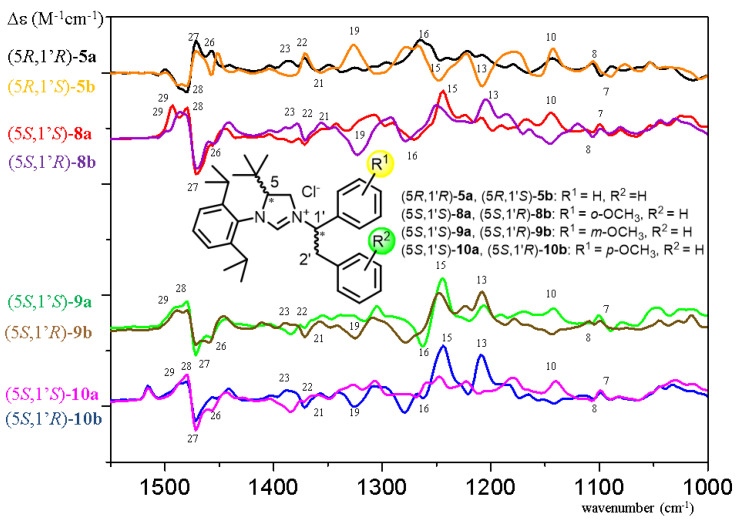
Overlapped experimental VCD spectra of the C-1′ epimeric reference compounds (5*R*,1′*R*)-**5a** and (5*R*,1′*S*)-**5b** and their methoxy-substituted analogues (R^1^ = OMe) (5*S*,1′*S*)-**8–10a** and (5*S*,1′*R*)-**8–10b** containing the methoxy group in different positions.

**Figure 4:**
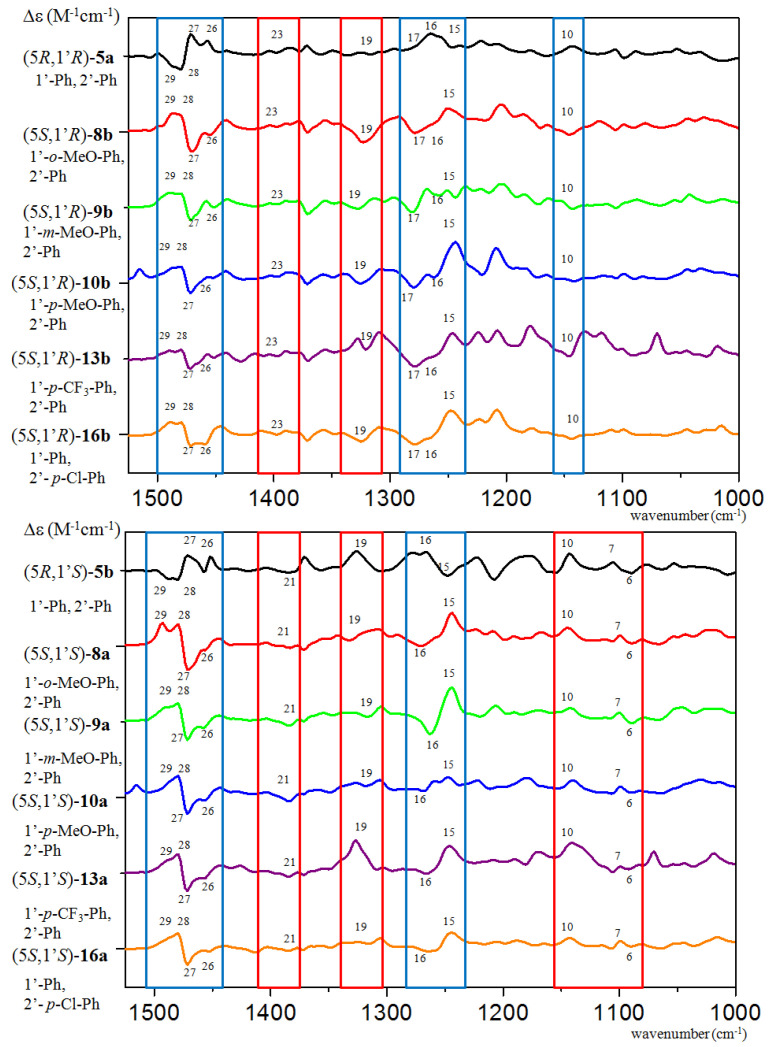
Experimental VCD spectra a) of (5*R*,1′*R*)-**5a** compared with those of (5*S*,1′*R*)-**8–10b**, **13b** and **16b** in CDCl_3_, and b) of (5*R*,1′*S*)-**5b** compared with those of (5*S*,1′*S*)-**8–10a**, **13a** and **16a** in CDCl_3_.

The VCD spectra of (5*R*,1′*R*)-**5a** and (5*R*,1′*S*)-**5b** showed significant differences in the sign and shape of several transitions in the range 1200–1400 cm^−^^1^ (transitions 11–23), which could be reproduced well by the VCD calculations and allowed configurational assignment of the C-1′ epimers (Figure 2 and Figure 3). In contrast, both epimers had a similar positive VCD couplet in the range of 1450–1500 cm^−^^1^ deriving from overlapping C-H deformation vibrations of the *t*-butyl group and the C-5 methine (transitions 26–29). Similarly, the transitions 15 and 16 constructed a negative VCD couplet for both epimers at about 1250 cm^−^^1^ (Figure 2 and Figure 3). Since these vibrations appear in the vicinity of the C-5 chirality center, they are governed by the AC of the imidazolium ring. Interestingly, the ECD spectra of (5*R*,1′*R*)-**5a** and (5*R*,1′*S*)-**5b** showed much less differences; both of them had an intense positive Cotton effect (CE) above 240 nm, positive CEs in the range 225–200 nm and a negative one below 200 nm. (5*R*,1′*S*)-**5b** had a weak negative CE at about 229 nm, which were missing from the ECD spectrum of (5*R*,1′*R*)-**5a** (Figure S153). This difference as well as the different shape and relative intensities of the bands could be reproduced well by the TDDFT-ECD calculations (Figures S181 and S182), which could also confirm the AC. The (5*R*,1′*R*)-**5a** and (5*R*,1′*S*)-**5b** epimers had oppositely signed specific rotation values (${\left[\alpha \right]}_{D}^{20}$ +61 versus −61, MeOH), the sign of which could be utilized to distinguish and assign them by OR calculations (Tables S5 and S6). The VCD spectra of (5*R*,1′*R*)-**5a** and (5*R*,1′*S*)-**5b** were compared with those of (5*S*,1′*S*)- and (5*S*,1′*R*)-**8–10a**, **13a** and **16a** having different substitution pattern in the C-1′ phenyl or benzyl group (Figure 4).

On the basis of the highlighted characteristic transitions, the AC could be determined by simple comparison, and there was no need to use the computed VCD spectra (Figure S167). The 26–29 VCD transitions (blue highlight, Figure 4) composed a VCD couplet characteristic of the C-5 chirality center and they gave a positive VCD couplet for the (5*R*) and a negative one for the (5S) AC. Moreover, VCD transitions associated with the vibrations of the 1′,2′-diarylethyl side chain (red highlights, Figure 4) reflected the AC of the C-1′ chirality center. When comparing ECD and OR data of **5a**,**b** with those of methoxy-substituted analogues **8–10a**,**b** (R^1^ = OMe, R^2^ = H), the **8a**,**b** derivatives having an *o*-methoxy substitution were found to show anomalous behavior (Tables S1 and S2, Figures S173 and S174), since their ECD spectra did not have a mirror-image relationship with those of the corresponding reference compounds **5a,b** and specific rotations of (5*S*,1′*S*)-**8a** and (5*S*,1′*R*)-**8b** had the same negative sign (${\left[\alpha \right]}_{D}^{20}$ −52 and −32, MeOH).

The presence of the *o*-methoxy substituent changed the direction of the electric transition moment of the C-1′ aryl group, and this was manifested in ECD spectra markedly different from those of the reference compound. While negative computed OR values (−146–−180 vs. −52, Tables S2 and S7) were obtained with four methods for (5*S*,1′*S*)-**8a**, the negative specific rotation values of (5*S*,1′*R*)-**8b** could not be reproduced by the OR calculations (+12–+29 vs. −32, Tables S2 and S8), since the population of a low-energy conformer with large negative OR value was clearly underestimated. The ECD spectra of the *m*- and *p*-methoxy derivatives **9a**,**b** and **10a**,**b** were found to be the mirror image of those of the corresponding **5a**,**b** reference compounds, and their ACs could be determined by simple comparison (Figures S173 and S174). In contrast to ECD and OR values, which were sensitive to the position of an achiral substituent such as a methoxy group and failed to assign the AC of **8a**,**b**, the simple comparison of the highlighted characteristic VCD transitions could be used to determine the AC of all the regio- and stereoisomers and substituted analogues.

**Figure 5:**
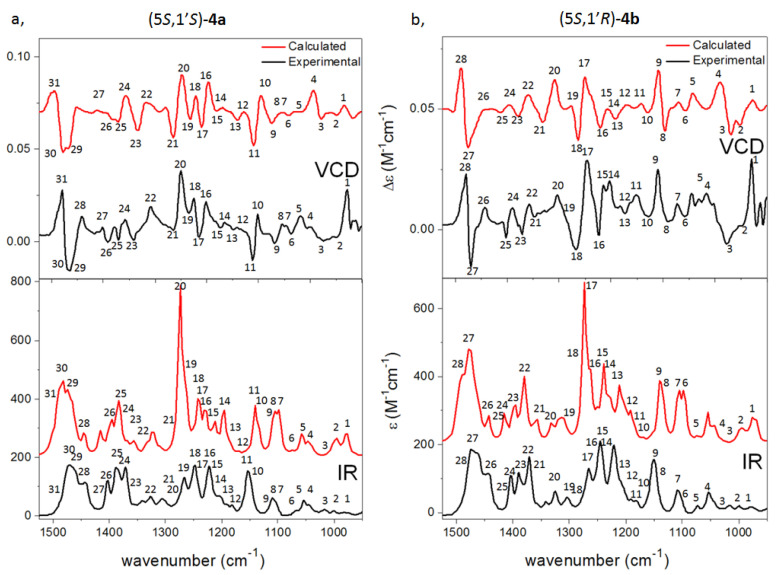
Experimental VCD (upper black) and IR spectra (lower black) of (**a**) (5*S*,1′*S*)-**4a** and (**b**) (5*S*,1′*R*)-**4b** compared with the B3LYP/TZPV PCM/CHCl_3_ VCD (upper red) and IR (lower red) spectra.

In (5*S*,1′*S*)-**4a** and (5*S*,1′*R*)-**4b**, the N-3 side chain, and hence the C-1′ chirality center, did not carry any chromophores resulting in near-identical ECD spectra (Figure S168d), which could not be used to distinguish the C-1 epimers (Figures S179 and S180). The similar negative VCD couplet of (5*S*,1′*S*)-**4a** and (5*S*,1′*R*)-**4b** at 1450–1500 cm^−^^1^ indicated their (5*S*) AC, while VCD calculation was utilized to determine the AC of the C-1′ chirality center based on the different transitions in the range of 1100–1400 cm^−^^1^ (Figure 5).

**Figure 6:**
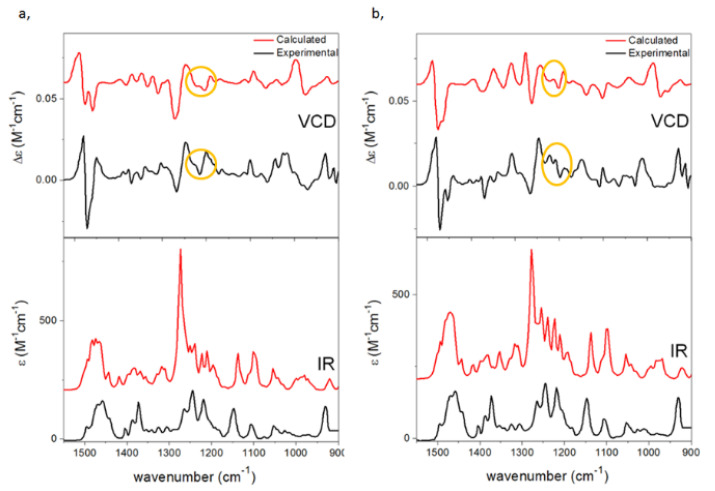
Experimental VCD (upper black, in CDCl_3_) and IR spectra (lower black, in CDCl_3_) of (**a**) (5*S*,1′*S*)-**1a** and (**b**) (5*S*,1′*R*)-**1b** compared with the B3LYP/TZPV PCM/CHCl_3_ VCD (upper red) and IR (lower red) spectra. Characteristic VCD transitions reflecting the different C-1′ ACs were highlighted with yellow circles.

**Figure 7:**
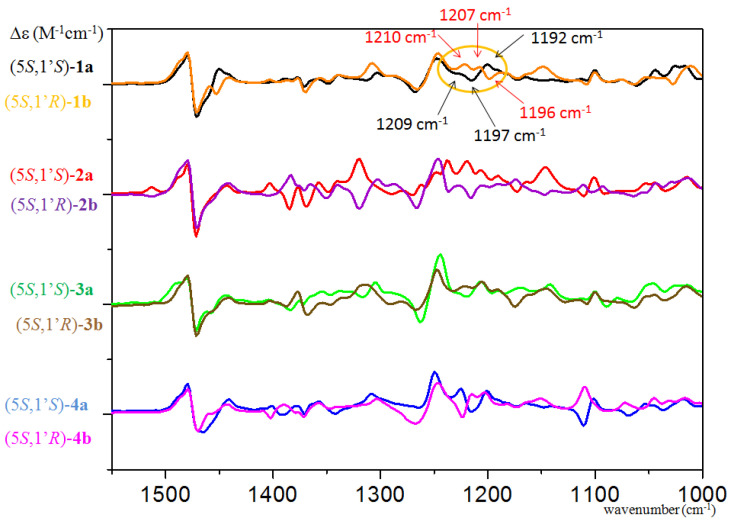
Experimental VCD curves of (5*S*,1′*S*)-**1–4a** overlapped with those of the epimeric (5*S*,1′*R*)-**1–4a** (CDCl_3)_.

Although either a phenyl or a 2-naphthyl group was attached directly to the C-1′ chirality center in the NHC precursors **1a**,**b** and **3a**,**b**, respectively, their (5*S*,1′*S*) and (5*S*,1′*R*) epimers provided near-identical ECD spectra, which was dictated by the C-5 chirality center, and it could not be used to distinguish the C-1′ epimers even with the aid of calculations (Figures S175 and S176). The specific rotation of (5*S*,1′*S*)-**1a** and (5*S*,1′*R*)-**1b** had also the same negative sign with relatively small amplitude (${\left[\alpha \right]}_{D}^{20}$ −27 and −46, MeOH), and thus it was only the slightly different VCD spectra (e.g.*,* three oppositely signed VCD transitions in the range of 1192–1210 cm^−^^1^) aided by DFT calculation that could determine the AC of C-1′ in the epimers (Figure 6 and Figure 7). The VCD configurational assignment was also confirmed by the maximum enantiomeric similarity index (ESI_max_) [52,53], which gave 0.739 value vs. 0.565 for (5*S*,1′*R*)-**1b** and 0.614 vs. 0.453 for (5*S*,1′*S*)-**1a** in the range of 1150–1500 cm^−1^.

**Figure 8:**
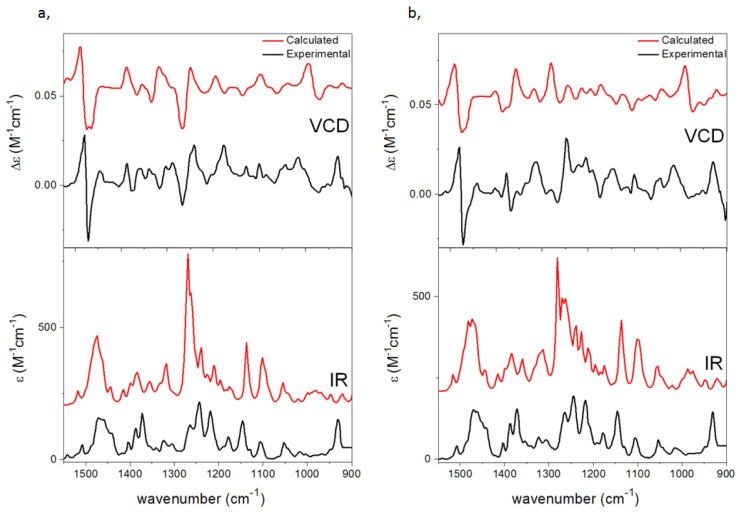
Experimental VCD (upper black, in CDCl_3_) and IR spectra (lower black, in CDCl_3_) of (**a**) (5*S*,1′*S*)-**3a** and (**b**) (5*S*,1′*R*)-**3b** compared with the B3LYP/TZPV PCM/CHCl_3_ VCD (upper red) and IR (lower red) spectra.

VCD calculations of the 2-napthyl derivatives (5*S*,1′*S*)-**3a** and (5*S*,1′*R*)-**3b** could efficiently distinguish the four stereoisomers (Figure 8). The observed negative VCD couplet at 1450–1500 cm^−^^1^ defined the (5*S*) AC from the experimental spectra, and VCD calculations allowed assigning the AC of the C-1′.

Significantly different VCD, ECD spectra and OR values were recorded for the 1-naphthyl analogues (5*S*,1′*S*)-**2a** and (5*S*,1′*R*)-**2b** (Figures S168, S144 and S145), and both ECD and VCD calculations were suitable to distinguish the stereoisomers (Figures S146, S177 and S178). The overlapped VCD spectra of the C-1′ epimeric **1–4a**,**b** clearly showed that, similarly to **5a**,**b** and **8–10a**,**b**, a negative VCD couplet centered at 1475 cm^−^^1^ and a positive couplet centered at 1260 cm^−^^1^ uniformly indicate the (5*S*) AC of the heteroring (Figure 7). The C-1′ epimers gave oppositely signed transitions in the 1100–1250 and 1300–1400 cm^−^^1^ ranges, but the exact position of the characteristic transitions changed significantly with the different types of C-1′ substituents.

## 3. Materials and Methods

### 3.1. General Procedures

All reagents obtained from commercial sources were used without further purification. Anhydrous solvents were obtained from commercial sources and used without further drying. The reactions were monitored using LCMS and GCMS instruments. Analytical LC-MS: Agilent HP1200 LC with Agilent 6140 quadrupole MS, operating in positive or negative ion electrospray ionisation mode. Molecular weight scan range was 100 to 1350 *m*/*z*. Parallel UV detection was done at 210 nm and 254 nm. Samples were supplied as a 1 mM solution in MeCN or in THF/water (1:1) with 5 µL loop injection. LCMS analyses were performed on two instruments, one of which was operated with basic, and the other with acidic eluents. Basic LCMS: Gemini-NX, 3 µm, C18, 50 mm × 3.00 mm i.d. column at 23 °C, at a flow rate of 1 mL min^−1^ using 5 mM aq. NH_4_HCO_3_ solution and MeCN as eluents. Acidic LCMS: ZORBAX Eclipse XDB-C18, 1.8 µm, 50 mm × 4.6 mm i.d. column at 40 °C, at a flow rate of 1 mL min^−1^ using water and MeCN as eluents, both containing 0.02 *v/v*% formic acid. Combination gas chromatography and low-resolution mass spectrometry were performed on Agilent 6850 gas chromatograph and Agilent 5975C mass spectrometer using a 15 m × 0.25 mm column with 0.25 µm HP-5MS coating and helium as carrier gas. Ion source: EI^+^, 70 eV, 230 °C, quadrupole: 150 °C, interface: 300 °C. Flash chromatography was performed on ISCO CombiFlash Rf 200i with pre-packed silica-gel cartridges (Redi*Sep*^®^*R*_f_ Gold High Performance). Chiral separations were performed on a KNAUER Smartline Preparative HPLC system with a (*R*,*R*) WHELKO O-1 50 mm × 500 mm, 10 µm column running at a flow rate of 50 mL min^−1^ with UV diode array detection (210–285 nm). Chiral purity was determined on an Agilent 1100 HPLC system with a WHELKO O-1, 250 mm × 4.6 mm, 10 µm column running at a flow rate of 1 mL min^−1^ with UV diode array detection (210–285 nm). ^1^H NMR and proton-decoupled ^13^C NMR measurements were performed on Bruker Avance III 500 MHz spectrometer and Bruker Avance III 400 MHz spectrometer, using DMSO-d_6_ or CDCl_3_ as solvent. ^1^H and ^13^C NMR data are in the form of delta values, given in part per million (ppm), using the residual peak of the solvent as internal standard [DMSO-d_6_: 2.50 ppm (^1^H)/39.5 ppm (^13^C); CDCl_3_: 7.26 ppm (^1^H)/77.0 ppm (^13^C)]. Splitting patterns are designated as: s (singlet), d (doublet), t (triplet), q (quartet), sp (septet), m (multiplet), br s (broad singlet), dd (doublet of doublets), td (triplet of doublets), qd (quartet of doublets). In some cases, two sets of signals appear in the spectra due to hindered rotation. HRMS were determined on a Shimadzu IT-TOF, ion source temperature 200 °C, ESI +/−, ionization voltage: (+/−) 4.5 kV. Mass resolution min. 10,000. Melting points were determined by OptiMelt melting view apparatus at ramp rates of 2 °C∙min^–1^ in sealed glass capillaries and are uncorrected. All products had an LC purity above 95% that was corroborated by their ^1^H NMR spectrum unless specifically mentioned otherwise. Specific rotation was measured on JASCO P-2000 polarimeter, while ECD spectra on a JASCO J-810 spectropolarimeter. VCD spectra were recorded on a BioTools ChiralIR-2X at a resolution of 4 cm^−1^ under ambient temperature for 18 × 3000 scans, respectively. Samples were dissolved in CDCl_3_ and DMSO-d_6_, and the concentrations were between 0.09 M and 0.30 M, and the solution was placed in a 100 μm BaF_2_ cell.

### 3.2. Computational Section

Mixed torsional/low-frequency mode conformational searches were carried out by means of the Macromodel 10.8.011 software, using the Merck Molecular Force Field (MMFF) with an implicit solvent model for CHCl_3_ [54]. All quantum chemical calculations were carried out with the Gaussian 09 software package [55]. The B3LYP and ωB97X [56] functionals with the TZVP basis set and the PCM solvent model for CHCl_3_, DMSO, MeCN and MeOH were used to re-optimize the MMFF geometries. TDDFT-ECD and OR calculations were performed at the B3LYP/TZVP, BH&HLYP/TZVP, CAM-B3LYP/TZVP and the PBE0/TZVP levels of theory with the PCM solvent model. ECD spectra were generated as sums of Gaussians with 3000 cm^−1^ widths at half-height, using dipole-velocity-computed rotational strength values [57]. VCD calculations were performed at the B3LYP/TZVP PCM/CHCl_3_ level, while the spectra were gained by applying a 8 cm^−1^ half-height width and scaled by a factor of 0.98 [58]. The CDSpecTech package [52,59] was applied to compute the maximum enantiomeric similarity index (ESI_max_) [53] values. Boltzmann distributions were estimated from the B3LYP and ωB97X energies. The MOLEKEL software package was used for visualization of the results [60].

## 4. Conclusions

The VCD approach has been found efficient to distinguish the four stereoisomers and determine the ACs of the studied NHC precursors, either by simple comparison of the experimental spectra or by means of DFT calculations, which provided good agreement with the experimental data. ECD and OR characterization and calculations were also carried out, but in several cases these methods were not suitable to determine the AC of the flexible N-3 side chain. The configurational assignment and chiroptical characterization of 16 diastereomeric pairs of NHC precursors enables the future application of closely related type III derivatives having different substitution patterns in stereoselective transformations.

## Data Availability

Not applicable.

## Supplementary Materials

The following supporting information can be downloaded at: https://www.mdpi.com/article/10.3390/ijms23073471/s1.
