# Supplementary material for: Synthesis and Vibrational Circular Dichroism Analysis of N-Heterocyclic Carbene Precursors Containing Remote Chirality Centers
Source: Int J Mol Sci. 2022 Mar 23;23(7):3471. doi: 10.3390/ijms23073471 (PMC8999040; doi:10.3390/ijms23073471)
Supplement: Supplementary file 1 [file ijms-23-03471-s001.zip › ijms-1643199-supplementary.pdf]

Supporting Information

Synthesis and vibrational circular dichroism analysis of *N*-heterocyclic carbene precursors containing remote chirality centers

Zita Szabó,† Attila Paczal,† Tibor Kovács,‡ Attila Mándi,‡ András Kotschy,*† Tibor Kurtán*‡

† Servier Research Institute of Medicinal Chemistry, Záhony u. 7., H-1031 Budapest, Hungary

‡ Department of Organic Chemistry, University of Debrecen, P. O. Box 400, 4002 Debrecen, Hungary

Table of Contents

1. Experimental Section for the Synthesis of NHC Precursors 9

1.1. General Procedure for the Preparation of *N*,*N*-Disubstituted 4,5-Dihydro-1*H*-imidazol-3-ium Chloride Derivatives (**1-17**) 9

1.2. General Procedure for Preparation of Aldehyde (**18a-b**) 23

1.3. General Procedure for Preparation of Butane-1,2-diamine (**19a-c**) 23

1.4. General Procedure for Preparation of Substituated Diamine Derivatives (**20a-z**) 24

1.5. General Procedure for Preparation of Ketone Derivatives (**21a-k**) 33

1.6. Synthesis of of Substituted 1-Phenylethanamine (**22-24a**,**b**) 36

1.7. References for Known Compounds 37

2. NMR Spectra (^{1}H and ^{13}C) 38

Figure S1. ^{1}H NMR (500 MHz) spectrum of **3a** in DMSO-d6 38

Figure S2. ^{13}C NMR (125 MHz) spectrum of **3a** in DMSO-d6 38

Figure S3. ^{1}H NMR (500 MHz) spectrum of **3b** in DMSO-d6 39

Figure S4. ^{13}C NMR (125 MHz) spectrum of **3b** in DMSO-d6 39

Figure S5. ^{1}H NMR (500 MHz) spectrum of **6a** in DMSO-d6 40

Figure S6. ^{13}C NMR (125 MHz) spectrum of **6a** in DMSO-d6 40

Figure S7. ^{1}H NMR (500 MHz) spectrum of **6b** in DMSO-d6 41

Figure S8. ^{13}C NMR (125 MHz) spectrum of **6b** in DMSO-d6 41

Figure S9. ^{1}H NMR (500 MHz) spectrum of **7a** in DMSO-d6 42

Figure S10. ^{13}C NMR (125 MHz) spectrum of **7a** in DMSO-d6 42

Figure S11. ^{1}H NMR (500 MHz) spectrum of **7b** in DMSO-d6 43

Figure S12. ^{13}C NMR (125 MHz) spectrum of **7b** in DMSO-d6 43
Figure S13. ^{1}H NMR (500 MHz) spectrum of **8a** in DMSO-d6 44
Figure S14. ^{13}C NMR (125 MHz) spectrum of **8a** in DMSO-d6 44
Figure S15. ^{1}H NMR (500 MHz) spectrum of **8b** in DMSO-d6 45
Figure S16. ^{13}C NMR (125 MHz) spectrum of **8b** in DMSO-d6 45
Figure S17. ^{1}H NMR (400 MHz) spectrum of **9a** in DMSO-d6 46
Figure S18. ^{13}C NMR (100 MHz) spectrum of **9a** in DMSO-d6 46
Figure S19. ^{1}H NMR (400 MHz) spectrum of **9b** in DMSO-d6 47
Figure S20. ^{13}C NMR (100 MHz) spectrum of **9b** in DMSO-d6 47
Figure S21. ^{1}H NMR (500 MHz) spectrum of **10a** in DMSO-d6 48
Figure S22. ^{13}C NMR (125 MHz) spectrum of **10a** in DMSO-d6 48
Figure S23. ^{1}H NMR (500 MHz) spectrum of **10b** in DMSO-d6 49
Figure S24. ^{13}C NMR (125 MHz) spectrum of **10b** in DMSO-d6 49
Figure S25. ^{1}H NMR (500 MHz) spectrum of **11a** in DMSO-d6 50
Figure S26. ^{13}C NMR (125 MHz) spectrum of **11a** in DMSO-d6 50
Figure S27. ^{1}H NMR (500 MHz) spectrum of **11b** in DMSO-d6 51
Figure S28. ^{13}C NMR (125 MHz) spectrum of **11b** in DMSO-d6 51
Figure S29. ^{1}H NMR (500 MHz) spectrum of **12a** in DMSO-d6 52
Figure S30. ^{13}C NMR (125 MHz) spectrum of **12a** in DMSO-d6 52
Figure S31. ^{1}H NMR (500 MHz) spectrum of **12b** in DMSO-d6 53
Figure S32. ^{13}C NMR (125 MHz) spectrum of **12b** in DMSO-d6 53
Figure S33. ^{1}H NMR (500 MHz) spectrum of **13a** in DMSO-d6 54
Figure S34. ^{13}C NMR (125 MHz) spectrum of **13a** in DMSO-d6 54
Figure S35. ^{1}H NMR (500 MHz) spectrum of **13b** in DMSO-d6 55
Figure S36. ^{13}C NMR (125 MHz) spectrum of **13b** in DMSO-d6 55
Figure S37. ^{1}H NMR (500 MHz) spectrum of **14a** in DMSO-d6 56
Figure S38. ^{13}C NMR (125 MHz) spectrum of **14a** in DMSO-d6 56
Figure S39. ^{1}H NMR (500 MHz) spectrum of **14b** in DMSO-d6 57
Figure S40. ^{13}C NMR (125 MHz) spectrum of **14b** in DMSO-d6 57
Figure S41. ^{1}H NMR (400 MHz) spectrum of **15a** in DMSO-d6 58
Figure S42. ^{13}C NMR (100 MHz) spectrum of **15a** in DMSO-d6 58
Figure S43. ^{1}H NMR (400 MHz) spectrum of **15b** in DMSO-d6 59
Figure S44. ^{13}C NMR (100 MHz) spectrum of **15b** in DMSO-d6 59
Figure S45. ^{1}H NMR (500 MHz) spectrum of **16a** in DMSO-d6 60
Figure S46. ^{13}C NMR (125 MHz) spectrum of **16a** in DMSO-d6 60

Figure S47. ^{1}H NMR (500 MHz) spectrum of **16b** in DMSO-d6 61
Figure S48. ^{13}C NMR (125 MHz) spectrum of **16b** in DMSO-d6 61
Figure S49. ^{1}H NMR (500 MHz) spectrum of **17a** in DMSO-d6 62
Figure S50. ^{13}C NMR (125 MHz) spectrum of **17a** in DMSO-d6 62
Figure S51. ^{1}H NMR (500 MHz) spectrum of **17b** in DMSO-d6 63
Figure S52. ^{13}C NMR (125 MHz) spectrum of **17b** in DMSO-d6 63
Figure S53. ^{1}H NMR (500 MHz) spectrum of **19a** in DMSO-d6 64
Figure S54. ^{13}C NMR (125 MHz) spectrum of **19a** in DMSO-d6 64
Figure S55. ^{1}H NMR (500 MHz) spectrum of **20a** in DMSO-d6 65
Figure S56. ^{13}C NMR (125 MHz) spectrum of **20a** in DMSO-d6 65
Figure S57. ^{1}H NMR (400 MHz) spectrum of **20b** in DMSO-d6 66
Figure S58. ^{13}C NMR (100 MHz) spectrum of **20b** in DMSO-d6 66
Figure S59. ^{1}H NMR (500 MHz) spectrum of **20c** in DMSO-d6 67
Figure S60. ^{13}C NMR (125 MHz) spectrum of **20c** in DMSO-d6 67
Figure S61. ^{1}H NMR (500 MHz) spectrum of **20d** in DMSO-d6 68
Figure S62. ^{13}C NMR (125 MHz) spectrum of **20d** in DMSO-d6 68
Figure S63. ^{1}H NMR (500 MHz) spectrum of **20e** in DMSO-d6 69
Figure S64. ^{13}C NMR (125 MHz) spectrum of **20e** in DMSO-d6 69
Figure S65. ^{1}H NMR (500 MHz) spectrum of **20f** in DMSO-d6 70
Figure S66. ^{13}C NMR (125 MHz) spectrum of **20f** in DMSO-d6 70
Figure S67. ^{1}H NMR (400 MHz) spectrum of **20g** in DMSO-d6 71
Figure S68. ^{13}C NMR (100 MHz) spectrum of **20g** in DMSO-d6 71
Figure S69. ^{1}H NMR (400 MHz) spectrum of **20h** in DMSO-d6 72
Figure S70. ^{13}C NMR (100 MHz) spectrum of **20h** in DMSO-d6 72
Figure S71. ^{1}H NMR (500 MHz) spectrum of **20i** in DMSO-d6 73
Figure S72. ^{13}C NMR (125 MHz) spectrum of **20i** in DMSO-d6 73
Figure S73. ^{1}H NMR (500 MHz) spectrum of **20j** in DMSO-d6 74
Figure S74. ^{13}C NMR (125 MHz) spectrum of **20j** in DMSO-d6 74
Figure S75. ^{1}H NMR (500 MHz) spectrum of **20l** in DMSO-d6 75
Figure S76. ^{13}C NMR (125 MHz) spectrum of **20l** in DMSO-d6 75
Figure S77. ^{1}H NMR (500 MHz) spectrum of **20m** in DMSO-d6 76
Figure S78. ^{13}C NMR (125 MHz) spectrum of **20m** in DMSO-d6 76
Figure S79. ^{1}H NMR (500 MHz) spectrum of **20n** in DMSO-d6 77
Figure S80. ^{13}C NMR (125 MHz) spectrum of **20n** in DMSO-d6 77
Figure S81. ^{1}H NMR (500 MHz) spectrum of **20o** in DMSO-d6 78

Figure S82. ^{13}C NMR (125 MHz) spectrum of **20o** in DMSO-d6 78
Figure S83. ^{1}H NMR (500 MHz) spectrum of **20p** in DMSO-d6 79
Figure S84. ^{13}C NMR (125 MHz) spectrum of **20p** in DMSO-d6 79
Figure S85. ^{1}H NMR (500 MHz) spectrum of **20q** in DMSO-d6 80
Figure S86. ^{13}C NMR (125 MHz) spectrum of **20q** in DMSO-d6 80
Figure S87. ^{1}H NMR (400 MHz) spectrum of **20r** in DMSO-d6 81
Figure S88. ^{13}C NMR (100 MHz) spectrum of **20r** in DMSO-d6 81
Figure S89. ^{1}H NMR (500 MHz) spectrum of **20s** in DMSO-d6 82
Figure S90. ^{13}C NMR (125 MHz) spectrum of **20s** in DMSO-d6 82
Figure S91. ^{1}H NMR (500 MHz) spectrum of **20t** in DMSO-d6 83
Figure S92. ^{13}C NMR (125 MHz) spectrum of **20t** in DMSO-d6 83
Figure S93. ^{1}H NMR (500 MHz) spectrum of **20u** in DMSO-d6 84
Figure S94. ^{13}C NMR (125 MHz) spectrum of **20u** in DMSO-d6 84
Figure S95. ^{1}H NMR (500 MHz) spectrum of **20v** in DMSO-d6 85
Figure S96. ^{13}C NMR (125 MHz) spectrum of **20v** in DMSO-d6 85
Figure S97. ^{1}H NMR (500 MHz) spectrum of **20w** in DMSO-d6 86
Figure S98. ^{13}C NMR (125 MHz) spectrum of **20w** in DMSO-d6 86
Figure S99. ^{1}H NMR (500 MHz) spectrum of **20x** in DMSO-d6 87
Figure S100. ^{13}C NMR (125 MHz) spectrum of **20x** in DMSO-d6 87
Figure S101. ^{1}H NMR (500 MHz) spectrum of **20y** in DMSO-d6 88
Figure S102. ^{13}C NMR (125 MHz) spectrum of **20y** in DMSO-d6 88
Figure S103. ^{1}H NMR (400 MHz) spectrum of **20z** in DMSO-d6 89
Figure S104. ^{13}C NMR (100 MHz) spectrum of **20z** in DMSO-d6 89
Figure S105. ^{1}H NMR (500 MHz) spectrum of **21a** in DMSO-d6 90
Figure S106. ^{13}C NMR (125 MHz) spectrum of **21a** in DMSO-d6 90
Figure S107. ^{1}H NMR (500 MHz) spectrum of **21b** in DMSO-d6 91
Figure S108. ^{13}C NMR (125 MHz) spectrum of **21b** in DMSO-d6 91
Figure S109. ^{1}H NMR (500 MHz) spectrum of **21c** in DMSO-d6 92
Figure S110. ^{13}C NMR (125 MHz) spectrum of **21c** in DMSO-d6 92
Figure S111. ^{1}H NMR (400 MHz) spectrum of **21d** in DMSO-d6 93
Figure S112. ^{13}C NMR (100 MHz) spectrum of **21d** in DMSO-d6 93
Figure S113. ^{1}H NMR (500 MHz) spectrum of **21e** in DMSO-d6 94
Figure S114. ^{13}C NMR (125 MHz) spectrum of **21e** in DMSO-d6 94
Figure S115. ^{1}H NMR (500 MHz) spectrum of **21f** in DMSO-d6 95
Figure S116. ^{13}C NMR (125 MHz) spectrum of **21f** in DMSO-d6 95

Figure S117. ^{1}H NMR (500 MHz) spectrum of **21g** in DMSO-d6 96
Figure S118. ^{13}C NMR (125 MHz) spectrum of **21g** in DMSO-d6 96
Figure S119. ^{1}H NMR (500 MHz) spectrum of **21h** in DMSO-d6 97
Figure S120. ^{13}C NMR (125 MHz) spectrum of **21h** in DMSO-d6 97
Figure S121. ^{1}H NMR (500 MHz) spectrum of **21i** in DMSO-d6 98
Figure S122. ^{13}C NMR (125 MHz) spectrum of **21i** in DMSO-d6 98
Figure S123. ^{1}H NMR (400 MHz) spectrum of **21j** in DMSO-d6 99
Figure S124. ^{13}C NMR (100 MHz) spectrum of **21j** in DMSO-d6 99
Figure S125. ^{1}H NMR (500 MHz) spectrum of **21k** in DMSO-d6 100
Figure S126. ^{13}C NMR (125 MHz) spectrum of **21k** in DMSO-d6 100
Figure S127. ^{1}H NMR (500 MHz) spectrum of **22** in DMSO-d6 101
Figure S128. ^{13}C NMR (125 MHz) spectrum of **22** in DMSO-d6 101
Figure S129. ^{1}H NMR (500 MHz) spectrum of **23** in DMSO-d6 102
Figure S130. ^{13}C NMR (125 MHz) spectrum of **23** in DMSO-d6 102
Figure S131. ^{1}H NMR (500 MHz) spectrum of **24a** in DMSO-d6 103
Figure S132. ^{13}C NMR (125 MHz) spectrum of **24a** in DMSO-d6 103
Figure S133. ^{1}H NMR (500 MHz) spectrum of **24b** in DMSO-d6 104
Figure S134. ^{13}C NMR (125 MHz) spectrum of **24b** in DMSO-d6 104
3. HPLC chromatograms performed with optically active stationary phase 105
Figure S135. Chiral separation of **20i**,**j** 105
Figure S136. Chiral separation of **20m**,**n** 105
Figure S137. Chiral separation of **20o**,**p** 106
Figure S138. Chiral separation of **20w**,**x** 106
Figure S139. Chiral separation of **24a**,**b** 107
4. VCD analysis 108
Figure S140. Experimental and computed VCD spectra of (5*S*,1’*S*)**-1a** 108
Figure S141. Experimental and computed VCD spectra of (5*S*,1’*R*)**-1b** 108
Figure S142. Overlapped VCD spectra of (5*S*,1’*S*)**-1a** and (5*S*,1’*R*)- **1b** 109
Figure S143. Overlapped VCD spectra of (5*S*,1’*S*)**-1a** and (5*S*,1’*R*)- **1b** 110
Figure S144. Experimental and computed VCD spectra of (5*S*,1’*S*)**-2a** 111
Figure S145. Experimental and computed VCD spectra of (5*S*,1’*R*)**-2b** 111
Figure S146. Experimental and computed VCD and IR spectra (5*S*,1’*S*)**-2a** and (5*S*,1’*R*)**-2b** 112
Figure S147. Experimental and computed VCD spectra of (5*S*,1’*S*)**-3a** 113
Figure S148. Experimental and computed VCD spectra of (5*S*,1’*R*)**-3b** 113
Figure S149. Experimental and computed VCD spectra of (5*S*,1’*S*)**-4a** 114

Figure S150. Experimental and computed VCD spectra of (5*S*,1’*R*)-**4b** 114
Figure S151. Experimental and computed VCD spectra of (5*S*,1’*S*)-**5a** 115
Figure S152. Experimental and computed VCD spectra of (5*S*,1’*R*)-**5b** 115
Figure S153. Experimental ECD and VCD spectra of (5*R*,1’*R*)-**5a** and (5*R*,1’*S*)-**5b** 116
Figure S154. Experimental and computed VCD spectra of (5*S*,1’*S*)-**8a** 117
Figure S155. Experimental and computed VCD spectra of (5*S*,1’*R*)-**8b** 117
Figure S156. VCD and IR spectra of (5*S*,1’*S*)-**8a** and (5*S*,1’*R*)-**8b** 118
Figure S157. Experimental and computed VCD spectra of (5*S*,1’*S*)-**9a** 119
Figure S158. Experimental and computed VCD spectra of (5*S*,1’*R*)-**9b** 119
Figure S159. VCD and IR spectra of (5*S*,1’*S*)-**9a** and (5*S*,1’*R*)-**9b** 120
Figure S160. Experimental and computed VCD spectra of (5*S*,1’*S*)-**10a** 120
Figure S161. Experimental and computed VCD spectra of (5*S*,1’*R*)-**10b** 121
Figure S162. VCD and IR spectra of (5*S*,1’*S*)-**10a** and (5*S*,1’*R*)-**10b** 122
Figure S163. Experimental VCD spectra of (5*S*,1’*R*)-**15b** recorded in DMSO-d_6 and $CDCl_3$ 122
Figure S164. Experimental and computed VCD spectra of (*R*)-**17a** 123
Figure S165. Experimental VCD spectra of (*S*)-**17b** and (*R*)-**17a** 124
Figure S166. Experimental VCD spectra of (5*S*,1’*S*)-**15a**, (5*S*,1’*R*)-**15b**, (*S*)-**17b** and (*R*)-**17b** 125
Figure S167. Experimental VCD spectra of **5**, **8**, **9, 10**, **13** and **16** 126
5. ECD analysis **127**
Figure S168. Experimental ECD spectra of **1**, **2**, **3**, **4** compared with reference spectra **5** 127
Figure S169. Experimental ECD spectra of **6**, **7**, **8**, **9** compared with reference spectra **5** 128
Figure S170. Experimental ECD spectra of **10**, **11**, **12**, **13** compared with reference spectra **5** 129
Figure S171. Experimental ECD spectra of **14**, **15**, **16**, **17** compared with reference spectra **5** 130
Figure S172. Experimental ECD spectra of **5** compared with **8**, **9** and **10** 131
Figure S173. ECD spectra of (5*R*,1’*R*)-**5a**, (5*S*,1’*S*)-**8a**, (5*S*,1’*S*)-**9a** and (5*S*,1’*S*)-**10a** 132
Figure S174. ECD spectra of (5*R*,1’*S*)-**5b**, (5*S*,1’*R*)-**8b**, (5*S*,1’*R*)-**9b** and (5*S*,1’*R*)-**10b** 133
Figure S175. Experimental and computed ECD spectrum of (5*S*,1’*S*)-**1a** 134
Figure S176. Experimental and computed ECD spectrum of (5*S*,1’*R*)-**1b** 134
Figure S177. Experimental and computed ECD spectrum of (5*S*,1’*S*)-**2a** 135
Figure S178. Experimental and computed ECD spectrum of (5*S*,1’*R*)-**2b** 135
Figure S179. Experimental and computed ECD spectra of (5*S*,1’*S*)-**4a** 136
Figure S180. Experimental and computed ECD spectra of (5*S*,1’*R*)-**4b** 136
Figure S181. Experimental and computed ECD spectra of (5*R*,1’*R*)-**5a** 137
Figure S182. Experimental and computed ECD spectra of (5*R*,1’*S*)-**5b** 137
Figure S183. Experimental and computed ECD spectra of (5*S*,1’*S*)-**8a** 138
Figure S184. Experimental and computed ECD spectra of (5*S*,1’*R*)-**8b** 138

Figure S185. Experimental and computed ECD spectra of (5*S*,1'*S*)-**9a** 139
Figure S186. Experimental and computed ECD spectra of (5*S*,1'*R*)-**9b** 139
Figure S187. Experimental and computed ECD spectra of (5*S*,1'*S*)-**10a** 140
Figure S188. Experimental and computed ECD spectra of (5*S*,1'*R*)-**10b** 140
Figure S189. Experimental and computed ECD spectra of (5*S*,1'*S*)-**13a** 141
6. OR analysis **142**
Table S1. Measured specific rotation of compounds at the sodium D line in MeOH 142
Table S2. Measured specific rotation compared with the four computed values 143
Table S3. OR calculation details of (5*S*,1'*S*)-**2a** 143
Table S4. OR calculation details of (5*S*,1'*R*)-**2b** 143
Table S5. OR calculation details of (5*R*,1'*R*)-**5a** 144
Table S6. OR calculation details of (5*R*,1'*S*)-**5b** 144
Table S7. OR calculation details of (5*S*,1'*S*)-**8a** 144
Table S8. OR calculation details of (5*S*,1'*R*)-**8b** 145
7. Low-energy conformers **146**
Figure S190. Structure and population of B3LYP low-energy conformers of (5*S*,1'*S*)-**1a** 146
Figure S191. Structure and population of ωB97X low-energy conformers of (5*S*,1'*S*)-**1a** 146
Figure S192. Structure and population of B3LYP low-energy conformers of (5*S*,1'*R*)-**1b** 147
Figure S193. Structure and population of ωB97X low-energy conformers of (5*S*,1'*R*)-**1b** 147
Figure S194. Structure and population of B3LYP low-energy conformers of (5*S*,1'*S*)-**2a** 148
Figure S195. Structure and population of ωB97X low-energy conformers of (5*S*,1'*S*)-**2a** 148
Figure S196. Structure and population of B3LYP low-energy conformers of (5*S*,1'*R*)-**2b** 149
Figure S197. Structure and population of ωB97X low-energy conformers of (5*S*,1'*R*)-**2b** 149
Figure S198. Structure and population of B3LYP low-energy conformers of (5*S*,1'*S*)-**3a** 150
Figure S199. Structure and population of ωB97X low-energy conformers of (5*S*,1'*S*)-**3a** 151
Figure S200. Structure and population of B3LYP low-energy conformers of (5*S*,1'*R*)-**3b** 152
Figure S201. Structure and population of ωB97X low-energy conformers of (5*S*,1'*R*)-**3b** 153
Figure S202. Structure and population of B3LYP low-energy conformers of (5*S*,1'*S*)-**4a** 153
Figure S203. Structure and population of ωB97X low-energy conformers of (5*S*,1'*S*)-**4a** 154
Figure S204. Structure and population of B3LYP low-energy conformers of (5*S*,1'*R*)-**4b** 154
Figure S205. Structure and population of ωB97X low-energy conformers of (5*S*,1'*R*)-**4b** 155
Figure S206. Structure and population of B3LYP low-energy conformers of (5*R*,1'*R*)-**5a** 155
Figure S207. Structure and population of ωB97X low-energy conformers of (5*R*,1'*R*)-**5a** 156
Figure S208. Structure and population of B3LYP low-energy conformers of (5*R*,1'*S*)-**5b** 156
Figure S209. Structure and population of ωB97X low-energy conformers of (5*R*,1'*S*)-**5b** 157
Figure S210. Structure and population of B3LYP low-energy conformers of (5*S*,1'*S*)-**8a** 157

Figure S211. Structure and population of ωB97X low-energy conformers of (5*S*,1’*S*)-**8a** 158
Figure S212. Structure and population of ωB97X low-energy conformers of (5*S*,1’*S*)-**8a** 158
Figure S213. Structure and population of B3LYP low-energy conformers of (5*S*,1’*R*)-**8b** 159
Figure S214. Structure and population of ωB97X low-energy conformers of (5*S*,1’*R*)-**8b** 159
Figure S215. Structure and population of ωB97X low-energy conformers of (5*S*,1’*R*)-**8b** 160
Figure S216. Structure and population of B3LYP low-energy conformers of (5*S*,1’*S*)-**9a** 160
Figure S217. Structure and population of ωB97X low-energy conformers of (5*S*,1’*S*)-**9a** 161
Figure S218. Structure and population of B3LYP low-energy conformers of (5*S*,1’*R*)-**9b** 162
Figure S219. Structure and population of ωB97X low-energy conformers of (5*S*,1’*R*)-**9b** 163
Figure S220. Structure and population of B3LYP low-energy conformers of (5*S*,1’*S*)-**10a** 163
Figure S221. Structure and population of ωB97X low-energy conformers of (5*S*,1’*S*)-**10a** 164
Figure S222. Structure and population of B3LYP low-energy conformers of (5*S*,1’*R*)-**10b** 165
Figure S223. Structure and population of ωB97X low-energy conformers of (5*S*,1’*R*)-**10b** 166
Figure S224. Structure and population of B3LYP low-energy conformers of (*R*)-**17a** 166
Figure S225. Structure and population of ωB97X low-energy conformers of (*R*)-**17a** 167
8. Cartesian coordinates 168
Table S9. Cartesian coordinates and energies at the B3LYP/TZVP PCM level 168
Table S10. Cartesian coordinates and energies at the ωB97X/TZVP PCM/MeCN level 272
Table S11. Cartesian coordinates and energies at the ωB97X/TZVP PCM/MeOH level 365

1 Experimental Section for the Synthesis of NHC Precursors

1.1 General Procedure for the Preparation of *N,N*-Disubstituted 4,5-Dihydro-1*H*-imidazol-3-ium Chloride Derivatives (1-17)

Example for compound **3a:**

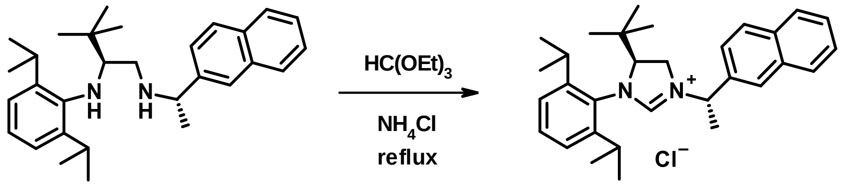

20y **3a**

2.10 g (2*S*)-*N*^{2}-(2,6-di(propan-2-yl)phenyl)-3,3-dimethyl-*N*^{1}-[(1'*S*)-1-(2-naphthyl)ethyl] butane-1,2-diamine [(2*S*,1'*S*)-**20y**] (4.88 mmol) was dissolved in 24.3 mL $HC(OEt)_3$ (146 mmol) and 287 mg NH_4Cl (5.63 mmol) was added in one portion. The mixture was heated to reflux temperature and stirred for 2 h, when complete conversion was observed by HPLC-MS. The volatiles were removed under reduced pressure and the residue was purified by column chromatography using DCM with 1.2% methanolic ammonia as eluent. The crude product was recrystallized from DCM-Et_2O.

(5*S*,1'*S*)-5-*tert*-butyl-1-(2,6-di(propan-2-yl)phenyl)-3-[1-phenylethyl]-4,5-dihydro-1*H*-imidazol-3-ium chloride [(5*S*,1'*S*)-1a]

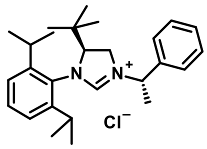

(5*S*,1'*S*)-**1a** was prepared by following published procedures.[1] $[\alpha]_D^{20}$ = –27 (*c* 0.01, MeOH). ECD (MeCN, λ nm (Δε) c = 1.94 x 10^{-4} M): 254 (–8.12), 224 (–0.24), 196 (2.24), 190 (–17.66).**(5*S*,1'*R*)-5-*tert*-butyl-1-(2,6-di(propan-2-yl)phenyl)-3-[1-phenylethyl]-4,5-dihydro-1*H*-imidazol-3-ium chloride [(5*S*,1'*R*)-1b]**

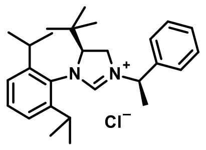

(5*S*,1'*R*)-**1b** was prepared by following published procedures.[1] $[\alpha]_D^{20}$ = –46 (*c* 0.01, MeOH). ECD (MeCN, λ nm (Δε) c = 1.92 x 10^{-4} M): 253 (–7.51), 225 (–0.97), 205 (–6.44), 197 (3.59), 191 (15.15).

(5*S*,1'*S*)-5-*tert*-butyl-1-(2,6-di(propan-2-yl)phenyl)-3-[1-(1-naphthyl)ethyl]-4,5-dihydro-1*H*-imidazol-3-ium chloride [(5*S*,1'*S*)-2a]

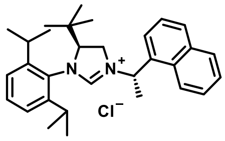

(5*S*,1'*S*)-**2a** was prepared by following published procedures.[1] $[\alpha]_D^{20}$ = +50 (*c* 0.01, MeOH). ECD (MeCN, λ nm (Δε) c = 1.62 x 10^{-4} M): 284 (–2.50), 254 (6.19), 245 (5.92), 236 (7.40), 221 (–28.97), 190 (21.03).

(5*S*,1'*R*)-4-*tert*-butyl-1-(2,6-di(propan-2-yl)phenyl)-3-[1-(1-naphthyl)ethyl]-4,5-dihydro-1*H*-imidazol-3-ium chloride [(5*S*,1'*R*)-2b]

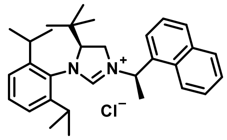

(5*S*,1'*R*)-**2b** was prepared by following published procedures.[1] $[\alpha]_D^{20}$ = −188 (*c* 0.01, MeOH). ECD (MeCN, λ nm (Δε) c = 1.86 x 10^{-4} M): 294 (1.03), 248 (–29.92), 232 (–27.73), 227 (–30.53), 213 (9.02), 210 (8.11), 199 (22.43), 198 (21.12), 190 (53.28).

(5*S*,1'*S*)-5-*tert*-butyl-1-(2,6-di(propan-2-yl)phenyl)-3-[1-(2-naphthyl)ethyl]-4,5-dihydro-1*H*-imidazol-3-ium chloride [(5*S*,1'*S*)-3a]

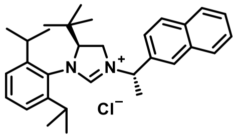

(5*S*,1'*S*)-**3a** was collected as white solid (1.87 g, 3.93 mmol, 80.6%). mp 238-240 °C. $[\alpha]_D^{20}$ = –35 (*c* 0.01, MeOH). ECD (MeCN, λ nm (Δε) c = 1.48 x 10^{-4} M): 253 (–8.75), 224 (–29.92), 205 (–9.75), 190 (20.23). ^{1}H NMR (500 MHz, DMSO-d_6) δ 9.13 (s, CH, 1 H), 8.06 (d, *J* = 8.6 Hz, Ar-H, 1 H), 7.99 (dd, *J* = 6.7, 1.8 Hz, Ar-H, 1 H), 7.97 (br, Ar-H, 1 H), 7.94 (dd, *J* = 6.7, 1.8 Hz, Ar-H, 1 H), 7.61 (td, *J* = 6.7, 1.8 Hz, Ar-H, 1 H), 7.59 (td, *J* = 6.7, 1.8 Hz, Ar-H, 1 H), 7.55 (dd, *J* = 8.6, 1.9 Hz, Ar-H, 1 H), 7.49 (t, *J* = 7.7 Hz, Ar-H, 1 H), 7.40 (dd, *J* = 7.7, 1.5 Hz, Ar-H, 1 H), 7.38 (dd, *J* = 7.7, 1.5 Hz, Ar-H, 1 H), 5.20 (q, *J* = 7.0 Hz, CH, 1 H), 4.44 (dd, *J* = 12.4, 9.3 Hz, CH, 1 H), 4.17/4.01 (t+dd, *J* = 12.4 Hz, *J* = 12.4, 9.3 Hz, CH_2, 2 H), 3.25 (sp, *J* = 6.7 Hz, CH, 1 H), 2.92 (sp, *J* = 6.7 Hz, CH, 1 H), 1.83 (d, *J* = 7.0 Hz, CH_3, 3 H), 1.30 (d, *J* = 6.7 Hz, CH_3, 3 H), 1.30 (d, *J* = 6.7 Hz, CH_3, 3 H), 1.24 (d, *J* = 6.7 Hz, CH_3, 3 H), 1.22 (d, *J* = 6.7 Hz, CH_3, 3 H), 0.74 (s, CH_3, 9 H). ^{13}C NMR (125 MHz, DMSO-d_6) δ 158.6, 145.5, 145.2, 135.8, 132.9, 132.8, 132.2, 130.4, 129.0, 127.9, 127.7, 126.9, 126.8, 126.0, 125.5, 125.3, 123.8, 73.9, 64.9, 57.8, 49.7, 28.3, 28.0, 25.8, 25.6, 25.2, 23.2, 22.6, 19.2. HRMS calcd for $C_{31}H_{41}N_2$ [M – Cl$^-$]$^+$ 441.3270, found 441.3261.

(5*S*,1'*R*)-5-*tert*-butyl-1-(2,6-di(propan-2-yl)phenyl)-3-[1-(2-naphthyl)ethyl]-4,5-dihydro-1*H*-imidazol-3-ium chloride [(5*S*,1'*R*)-3b]

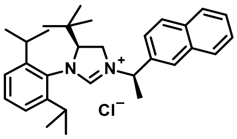

By following the synthetic procedure described for (5*S*,1'*S*)-**3a**, the reaction was carried out with 2.71 g (2*S*,1'*R*)-**20z** (6.31 mmol). The product (5*S*,1'*R*)-**3b** was collected as white solid (2.49 g, 5.22 mmol, 82.8%). mp 264-266 °C. $[\alpha]_D^{20}$ = –29 (*c* 0.01, MeOH). ECD (MeCN, λ nm (Δε) c = 1.81 x 10^{-4} M): 250 (–9.63), 224 (6.33), 205 (–7.89), 190 (19.83). ^{1}H NMR (500 MHz, DMSO-d_6) δ 8.21 (s, CH, 1 H), 8.19-7.52 (m, Ar-H, 7 H), 7.47 (t, *J* = 7.7 Hz, Ar-H, 1 H), 7.38 (dd, *J* = 7.7, 1.5 Hz, Ar-H, 1 H), 7.35 (dd, *J* = 7.7, 1.5 Hz, Ar-H, 1 H), 5.47 (q, *J* = 7.0 Hz, CH, 1 H), 4.37 (dd, *J* = 12.4, 8.9 Hz, CH, 1 H), 4.08/4.00 (t+dd, *J* = 12.4 Hz, *J* = 12.4, 8.9 Hz, CH_2, 2 H), 3.28 (sp, *J* = 6.7 Hz, CH, 1 H), 2.84 (sp, *J* = 6.7 Hz, CH, 1 H), 1.87 (d, *J* = 7.0 Hz, CH_3, 3 H), 1.31 (d, *J* = 6.7 Hz, CH_3, 3 H), 1.27 (d, *J* = 6.7 Hz, CH_3, 3 H), 1.22 (d, *J* = 6.7 Hz, CH_3, 3 H), 1.13 (d, *J* = 6.7 Hz, CH_3, 3 H), 0.77 (s, CH_3, 9 H). ^{13}C NMR (125 MHz, DMSO-d_6) δ 159.5, 145.5, 145.3, 135.0, 132.8, 132.8, 132.2, 130.3, 128.8, 128.1, 127.7, 126.8, 126.1, 125.4, 125.3, 124.6, 74.2, 56.9, 47.7, 28.3, 28.0, 26.0, 25.5, 25.3, 23.2, 22.6, 17.5. HRMS calcd for $C_{31}H_{41}N_2$[M–Cl$^-$]$^+$ 441.3270, found 441.3255.

(5*S*,1'*S*)-5-*tert*-butyl-1-(2,6-di(propan-2-yl)phenyl)-3-[1,2,2-trimethylpropyl]-4,5-dihydro-1*H*-imidazol-3-ium chloride [(5*S*,1'*S*)-4a]

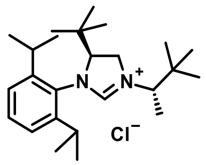

(5*S*,1'*S*)-**4a** was prepared by following published procedures.[1] $[\alpha]_D^{20}$ = –10 (*c* 0.01, MeOH). ECD (MeCN, λ nm (Δε) c = 2.17 x 10^{-4} M): 249 (–5.83), 227 (–0.69), 208 (–3.75), 190 (22.05).

(5*S*,1'*R*)-5-*tert*-butyl-1-(2,6-di(propan-2-yl)phenyl)-3-[1,2,2-trimethylpropyl]-4,5-dihydro-1*H*-imidazol-3-ium chloride [(5*S*,1'*R*)-4b]

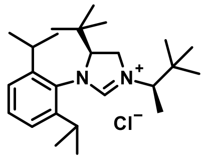

(5*S*,1'*R*)-**4b** was prepared by following published procedures.[1] $[\alpha]_D^{20}$ = –55 (*c* 0.01, MeOH). ECD (MeCN, λ nm (Δε) c = 2.17 x 10^{-4} M): 250 (–8.30), 223 (–1.78), 208 (–3.89), 190 (16.52).

(5*R*,1'*R*)-5-*tert*-butyl-1-(2,6-di(propan-2-yl)phenyl)-3-[1,2-diphenylethyl]-4,5-dihydro-1*H*-imidazol-3-ium chloride [(5*R*,1'*R*)-5a]

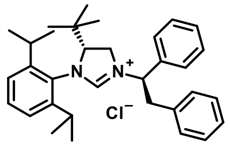

(5*R*,1'*R*)-**5a** was prepared by following published procedures.[1] $[\alpha]_D^{20}$ = +61 (*c* 0.01, MeOH). ECD (MeCN, λ nm (Δε) c = 1.50 x 10^{-4} M): 255 (10.69), 228 (0.46), 208 (6.40), 194 (–8.27), 191 (–1.56).

(5*R*,1'*S*)-5-*tert*-butyl-1-(2,6-di(propan-2-yl)phenyl)-3-[1,2-diphenylethyl]-4,5-dihydro-1*H*-imidazol-3-ium chloride [(5*R*,1'*S*)-5b]

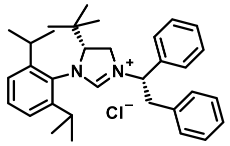

(5*R*,1'*S*)-**5b** was prepared by following published procedures.[1] $[\alpha]_D^{20}$ = –61 (*c* 0.01, MeOH). ECD (MeCN, λ nm (Δε) c = 2.14 x 10^{-4} M): 257 (4.63), 229 (–2.67), 217 (0.99), 195 (–18.32), 190 (13.23).

(5*R*,1'*R*)-5-*tert*-butyl-3-[2-(3-chlorophenyl)-1-phenylethyl]-1-(2,6-di(propan-2-yl)phenyl)-4,5-dihydro-1*H*-imidazol-3-ium chloride [(5*R*,1'*R*)-6a]

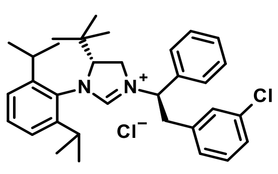

By following the synthetic procedure described for (5*S*,1'*S*)-**3a**, the reaction was performed with 1.13 g (2*R*,1'*R*)-**20a** (2.29 mmol). The product (5*R*,1'*R*)-**6a** was collected as white solid (804 mg, 1.50 mmol, 65.3%). mp 255-257 °C. $[\alpha]_D^{20}$ = +58 (*c* 0.01, MeOH). ECD (MeCN, λ nm (Δε) c = 1.66 x 10^{-4} M): 256 (4.63), 227 (–0.03), 207 (7.23), 190 (–11.87). ^{1}H NMR (500 MHz, DMSO-d_6) δ 9.21 (s, CH, 1 H), 7.58-7.33 (m, Ar- H, 4 H), 7.58-7.42 (m, Ar-H, 5 H), 7.46 (t, *J* = 7.8 Hz, Ar-H, 1 H), 7.35 (d, *J* = 7.8 Hz, Ar-H, 2 H), 5.52 (dd, *J* = 11.7, 5.1 Hz, CH, 1 H), 4.24 (m, CH, 1 H), 4.23/3.92 (m+m, CH_2, 2 H), 3.64/3.45 (dd+dd,

J = 15.4, 11.7 Hz, J = 15.4, 5.1 Hz, CH_2, 2 H), 2.77 (sp, J = 6.7 Hz, CH, 1 H), 2.66 (sp, J = 6.7 Hz, CH, 1 H), 1.26 (d, J = 6.7 Hz, CH_3, 3 H), 1.20 (d, J = 6.7 Hz, CH_3, 3 H), 1.14 (d, J = 6.7 Hz, CH_3, 3 H), 1.05 (d, J = 6.7 Hz, CH_3, 3 H), 0.61 (s, CH_3, 9 H). ^{13}C NMR (125 MHz, DMSO-d_6) δ 158.9, 145.3, 144.9, 138.9, 136.9, 133.6, 131.8, 130.5, 130.5, 129.2, 129.1, 128.3, 127.7, 127.1, 126.9, 125.5, 125.3, 73.8, 61.5, 50.3, 37.4, 35.0, 28.4, 28.0, 25.8, 25.1, 25.0, 23.2, 22.5. HRMS calcd for $C_{33}H_{42}ClN_2$ [M-Cl$^-$]$^+$ 501.3037, found 501.3032.

(5*R*,1'*S*)-5-*tert*-butyl-3-[2-(3-chlorophenyl)-1-phenylethyl]-1-(2,6-di(propan-2-yl)phenyl)-4,5-dihydro-1*H*-imidazol-3-ium chloride [(5*R*,1'*S*)-6b]

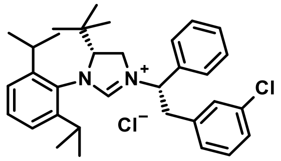

By following the synthetic procedure described for (5*S*,1'*S*)-**3a**, the reaction was performed with 1.62 g (2*R*,1'*S*)-**20b** (3.30 mmol). The product (5*R*,1'*S*)-**6b** was collected as white solid (1.42 g, 2.64 mmol, 80.1%). mp 262-264°C. $[\alpha]_D^{20}$ = –50 (*c* 0.01, MeOH). ECD (MeCN, λ nm (Δε) c = 1.52 x 10^{-4} M): 262 (4.53), 229 (–3.79), 207 (1.60), 200 (–4.31), 191 (13.12). ^{1}H NMR (500 MHz, DMSO-d_6) δ 9.02 (s, CH, 1 H), 7.65 (brs, Ar-H, 1 H), 7.61-7.43 (m, Ar-H, 8 H), 7.43 (t, J = 7.8 Hz, Ar-H, 1 H), 7.30 (dm, J = 7.8 Hz, Ar-H, 2 H), 5.70 (dd, J = 12.1, 4.0 Hz, CH, 1 H), 4.44 (dd, J = 12.0, 10.3 Hz, 1 H), 4.14/3.79 (dd+dd, J = 12.0, 10.3 Hz, J = 12.0, 10.3 Hz, CH_2, 2 H), 3.79/3.41 (dd+dd, J = 14.3, 12.1 Hz, J = 14.3, 4.0 Hz, CH_2, 2 H), 2.72 (sp, J = 6.7 Hz, CH, 1 H), 2.41 (sp, J = 6.7 Hz, CH, 1 H), 1.24 (d, J = 6.7 Hz, CH_3, 3 H), 1.17 (d, J = 6.7 Hz, CH_3, 3 H), 1.05 (d, J = 6.7 Hz, CH_3, 3 H), 0.93 (d, J = 6.7 Hz, CH_3, 3 H), 0.66 (s, CH_3, 9 H). ^{13}C NMR (125 MHz, DMSO-d_6) δ 159.4, 145.4, 145.2, 139.5, 135.7, 133.4, 131.3, 130.6, 130.5, 129.4, 129.2, 127.9, 127.7, 127.1, 125.3, 125.2, 74.0, 62.0, 46.2, 34.7, 34.3, 28.3, 27.8, 26.0, 25.6, 25.2, 22.7, 22.3. HRMS calcd for $C_{33}H_{42}ClN_2$ [M-Cl$^-$]$^+$ 501.3037, found 501.3038.

(5*R*,1'*R*)-5-*tert*-butyl-1-(2,6-di(propan-2-yl)phenyl)-3-[2-phenyl-1-[3-(trifluoromethyl)phenyl]ethyl]-4,5-dihydro-1*H*-imidazol-3-ium chloride [(5*R*,1'*R*)-7a]

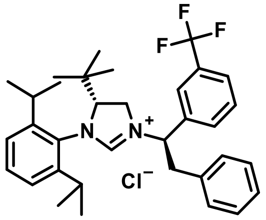

By following the synthetic procedure described for (5*S*,1'*S*)-**3a**, the reaction was performed with 815 mg (2*R*,1'*R*)-**20c** (1.53 mmol). The product (5*R*,1'*R*)-**7a** was collected as white solid (547 mg, 0.958 mmol, 61.7%). mp 225-227 °C. $[\alpha]_D^{20}$ = +63 (*c* 0.01, MeOH). ECD (MeCN, λ nm (Δε) c = 1.47 x 10^{-4} M): 252 (10.92), 220 (0.53), 205 (7.35), 196 (–6.14), 194 (–4.73), 190 (–10.61). ^{1}H NMR (500 MHz, DMSO-d_6) δ 9.29 (s, CH, 1 H), 7.94 (s, Ar-H, 1 H), 7.89 (dm, J = 7.8 Hz, Ar-H, 1 H), 7.84 (dm, J = 7.8 Hz, Ar-H, 1 H), 7.76 (t, J = 7.8 Hz, Ar-H, 1 H), 7.47 (t, J = 7.7 Hz, Ar-H, 1 H), 7.45-7.28 (m, Ar-H, 7 H), 5.60 (dd, J = 11.6, 5.4 Hz, CH, 1 H), 4.26/3.91 (m+m, CH_2, 2 H), 4.23 (m, CH, 1 H), 3.62/3.48 (dd+dd, J = 15.3, 11.6 Hz, J = 15.3, 5.4 Hz, CH_2, 2 H), 2.74 (sp, J = 6.7 Hz, CH, 1 H), 2.67 (sp, J = 6.7 Hz, CH, 1 H), 1.24 (d, J = 6.7 Hz, CH_3, 3 H), 1.18 (d, J = 6.7 Hz, CH_3, 3 H), 1.18 (d, J = 6.7 Hz, CH_3, 3 H), 1.04 (d, J = 6.7 Hz, CH_3, 3 H), 0.57 (s, CH_3, 9 H). ^{13}C NMR (125 MHz, DMSO-d_6) δ 158.9, 145.2, 144.8, 138.8, 136.0, 131.7, 131.7, 130.4, 130.3, 128.8, 128.7, 127.1, 125.8, 125.5, 125.4, 123.5, 73.7, 61.5, 50.9, 38.0, 34.9, 28.3, 27.8, 26.1, 25.0, 25.0, 23.2, 22.5. HRMS calcd for $C_{34}H_{42}F_3N_2$ [M-Cl$^-$]$^+$ 535.3300, found 535.3303.

(5*R*,1'*S*)-5-*tert*-butyl-1-(2,6-di(propan-2-yl)phenyl)-3-[2-phenyl-1-[3-(trifluoromethyl)phenyl]ethyl]-4,5-dihydro-1*H*-imidazol-3-ium chloride [(5*R*,1'*S*)-7b]

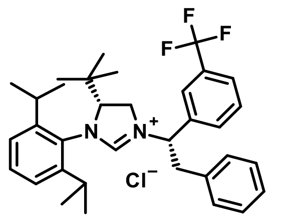

By following the synthetic procedure described for (5*S*,1'*S*)-**3a**, the reaction was performed with 660 mg (2*R*,1'*S*)-**20d** (1.26 mmol). The product (5*R*,1'*S*)-**7b** was collected as a white solid (294 mg, 0.514 mmol, 40.9%). mp 267-269°C. $[\alpha]_D^{20}$ = –39 (*c* 0.01, MeOH). ECD (MeCN, λ nm (Δε) c = 1.45 x 10^{-4} M): 255 (4.24), 230 (–2.41), 209 (2.44), 195 (–19.86), 190 (–0.29). ^{1}H NMR (500 MHz, DMSO-d_6) δ 9.24 (s, CH, 1 H), 7.99 (dm, *J* = 7.8 Hz, Ar-H, 1 H), 7.98 (s, Ar-H, 1 H), 7.88 (dm, *J* = 7.8 Hz, Ar-H, 1 H), 7.82 (t, *J* = 7.8 Hz, Ar-H, 1 H), 7.56-7.32 (m, Ar-H, 5 H), 7.43 (t, *J* = 7.8 Hz, Ar-H, 1 H), 7.30 (d, *J* = 7.8 Hz, Ar-H, 2 H), 5.98 (dd, *J* = 12.5, 4.2 Hz, CH, 1 H), 4.41 (dd, *J* = 12.6, 10.1 Hz, CH, 1 H), 4.05/3.80 (dd+dm, *J* = 12.6, 10.1 Hz, *J* = 12.6 Hz, CH_2, 2 H), 3.83/3.47 (dd+dd, *J* = 14.4, 12.5 Hz, *J* = 14.4, 4.2 Hz, CH_2, 2 H), 2.73 (sp, *J* = 6.7 Hz, CH, 1 H), 2.41 (sp, *J* = 6.7 Hz, CH, 1 H), 1.22 (d, *J* = 6.7 Hz, CH_3, 3 H), 1.13 (d, *J* = 6.7 Hz, CH_3, 3 H), 1.04 (d, *J* = 6.7 Hz, CH_3, 3 H), 0.98 (d, *J* = 6.7 Hz, CH_3, 3 H), 0.59 (s, CH_3, 9 H). ^{13}C NMR (125 MHz, DMSO-d_6) δ 159.7, 145.3, 145.1, 137.5, 136.5, 131.8, 131.2, 130.4, 130.3, 129.1, 128.8, 127.1, 125.9, 125.3, 125.2, 124.8, 73.9, 60.8, 46.4, 34.6, 34.3, 28.2, 27.6, 26.1, 25.6, 25.1, 22.7, 22.2. HRMS calcd for $C_{34}H_{42}F_3N_2$ $[M\text{-}Cl^-]^+$ 535.3300, found 535.3294.

(5*S*,1'*S*)-5-*tert*-butyl-1-(2,6-di(propan-2-yl)phenyl)-3-[1-(2-methoxyphenyl)-2-phenylethyl]-4,5-dihydro-1*H*-imidazol-3-ium chloride [(5*S*,1'*S*)-8a]

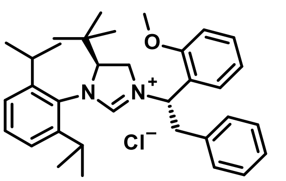

By following the synthetic procedure described for (5*S*,1'*S*)-**3a**, the reaction was performed with 753 mg (2*S*,1'*S*)-**20e** (1.55 mmol). The product (5*S*,1'*S*)-**8a** was collected as white solid (673 mg, 1.26 mmol, 81.6%). mp 208-210°C. $[\alpha]_D^{20}$ = –52 (*c* 0.01, MeOH). ECD (MeCN, λ nm (Δε) c = 1.76 x 10^{-4} M): 282 (12.44), 254 (–14.75), 229 (0.67), 206 (–14.93), 195 (8.70), 193 (–0.69), 190 (12.88). ^{1}H NMR (500 MHz, DMSO-d_6) δ 9.05 (s, CH, 1 H), 7.57 (d, *J* = 7.8 Hz, Ar-H, 1 H), 7.48 (t, *J* = 7.8 Hz, Ar-H, 1 H), 7.45 (d, *J* = 7.3 Hz, Ar-H, 2 H), 7.44 (t, *J* = 7.7 Hz, Ar-H, 1 H), 7.37 (t, *J* = 7.3 Hz, Ar-H, 2 H), 7.34 (d, *J* = 7.7 Hz, Ar-H, 1 H), 7.31 (d, *J* = 7.7 Hz, Ar-H, 1 H), 7.28 (t, *J* = 7.3 Hz, Ar-H, 1 H), 7.18 (d, *J* = 7.8 Hz, Ar-H, 1 H), 7.12 (t, *J* = 7.8 Hz, Ar-H, 1 H), 5.60 (dd, *J* = 11.5, 4.6 Hz, CH, 1 H), 4.37/3.45 (t+dd, *J* = 12.5 Hz, *J* = 12.5, 7.9 Hz, CH_2, 2 H), 4.20 (dd, *J* = 12.5, 7.9 Hz, CH, 1 H), 3.87 (s, CH_3, 3 H), 3.60/3.36 (dd+dd, *J* = 14.6, 11.5 Hz, *J* = 14.6, 4.6 Hz, CH_2, 2 H), 2.96 (sp, *J* = 6.7 Hz, CH, 1 H), 2.56 (sp, *J* = 6.7 Hz, CH, 1 H), 1.28 (d, *J* = 6.7 Hz, CH_3, 3 H), 1.25 (d, *J* = 6.7 Hz, CH_3, 3 H), 1.23 (d, *J* = 6.7 Hz, CH_3, 3 H), 0.92 (d, *J* = 6.7 Hz, CH_3, 3 H), 0.52 (s, CH_3, 9 H). ^{13}C NMR (125 MHz, DMSO-d_6) δ 159.8, 157.1, 145.3, 145.0, 136.7, 131.6, 130.7, 130.3, 129.0, 128.6, 127.5, 127.0, 125.5, 125.3, 123.2, 120.7, 111.6, 73.4, 56.9, 55.8, 47.2, 34.8, 34.8, 28.3, 27.8, 26.0, 25.4, 24.9, 23.5, 22.7. HRMS calcd for $C_{34}H_{45}N_2O$ $[M\text{-}Cl^-]^+$ 497.3532, found 497.3533.

(5*S*,1'*R*)-5-*tert*-butyl-1-(2,6-di(propan-2-yl)phenyl)-3-[1-(2-methoxyphenyl)-2-phenylethyl]-4,5-dihydro-1*H*-imidazol-3-ium chloride [(5*S*,1'*R*)-8b]

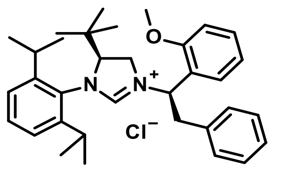

By following the synthetic procedure described for (5*S*,1'*S*)-**3a**, the reaction was performed with 485 mg (2*S*,1'*R*)-**20f** (1.00 mmol). The product (5*S*,1'*R*)-**8b** was collected as white solid (441 mg, 0.827 mmol, 82.7%). mp 232-234°C. $[\alpha]_D^{20}$ = –32 (*c* 0.01, MeOH). ECD (MeCN, λ nm (Δε) c = 1.54 x 10^{-4} M): 276 (–15.39), 253 (0.54), 229 (–1.89), 225 (–0.86), 206 (9.93), 195 (33.67). ^{1}H NMR (500 MHz, DMSO-*d*$_6$) *δ* 8.70 (s, CH, 1 H), 7.62 (d, *J* = 7.7 Hz, Ar-H, 1 H), 7.50 (t, *J* = 7.7 Hz, Ar-H, 1 H), 7.49 (d, *J* = 7.2 Hz, Ar-H, 2 H), 7.41 (t, *J* = 7.7 Hz, Ar-H, 1 H), 7.39 (t, *J* = 7.2 Hz, Ar-H, 2 H), 7.36 (t, *J* = 7.2 Hz, Ar-H, 1 H), 7.28 (d, *J* = 7.7 Hz, Ar-H, 1 H), 7.26 (d, *J* = 7.7 Hz, Ar-H, 1 H), 7.18 (d, *J* = 7.7 Hz, Ar-H, 1 H), 7.13 (t, *J* = 7.7 Hz, Ar-H, 1 H), 5.58 (dd, *J* = 12.1, 3.9 Hz, CH, 1 H), 4.36 (dd, *J* = 12.3, 10.6 Hz, CH, 1 H), 4.00/3.61 (dd+t, *J* = 12.3, 10.6 Hz, *J* = 12.3 Hz, CH_2, 2 H), 3.82 (s, CH_3, 3 H), 3.80/3.31 (dd+dd, *J* = 14.0, 12.1 Hz, *J* = 14.0, 3.9 Hz, CH_2, 2 H), 2.74 (sp, *J* = 6.7 Hz, CH, 1 H), 2.32 (sp, *J* = 6.7 Hz, CH, 2 H), 1.24 (d, *J* = 6.7 Hz, CH_3, 3 H), 1.13 (d, *J* = 6.7 Hz, CH_3, 3 H), 1.10 (d, *J* = 6.7 Hz, CH_3, 3 H), 0.90 (d, *J* = 6.7 Hz, CH_3, 3 H), 0.62 (s, CH_3, 9 H). ^{13}C NMR (125 MHz, DMSO-*d*$_6$) *δ* 159.6, 157.7, 145.4, 145.4, 136.8, 131.3, 130.8, 130.2, 129.4, 128.6, 128.0, 127.1, 125.3, 125.1, 122.9, 120.5, 111.6, 73.6, 57.2, 55.8, 46.2, 34.6, 34.2, 28.2, 27.5, 25.9, 25.7, 25.5, 23.1, 22.6. HRMS calcd for $C_{34}H_{45}N_2O$ [M-Cl$^-$]$^+$ 497.3532, found 497.3530.

(5*S*,1'*S*)-5-*tert*-butyl-1-(2,6-di(propan-2-yl)phenyl)-3-[1-(3-methoxyphenyl)-2-phenylethyl]-4,5-dihydro-1*H*-imidazol-3-ium chloride [(5*S*,1'*S*)-9a]

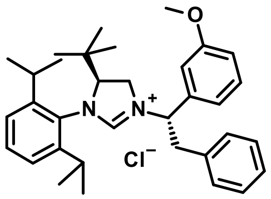

By following the synthetic procedure described for (5*S*,1'*S*)-**3a**, the reaction was performed with 350 mg (2*S*,1'*S*)-**20g** (0.719 mmol). The product (5*S*,1'*S*)-**9a** was collected as white solid (292 mg, 0.548 mmol, 76.2%). mp 240-242°C. $[\alpha]_D^{20}$ = –70 (*c* 0.01, MeOH). ECD (MeCN, λ nm (Δε) c = 1.68 x 10^{-4} M): 254 (–10.38), 228 (–1.30), 205 (–6.72), 195 (8.25), 193 (–0.02), 191 (4.27). ^{1}H NMR (400 MHz, DMSO-*d*$_6$) *δ* 9.23 (s, CH, 1 H), 7.46 (t, *J* = 7.7 Hz, Ar-H, 1 H), 7.43 (d, *J* = 7.3 Hz, Ar-H, 2 H), 7.42 (t, *J* = 8.0 Hz, Ar-H, 1 H), 7.39 (t, *J* = 7.3 Hz, Ar-H, 2 H), 7.36 (d, *J* = 7.7 Hz, Ar-H, 1 H), 7.34 (d, *J* = 7.7 Hz, Ar-H, 1 H), 7.31 (t, *J* = 7.3 Hz, Ar-H, 1 H), 7.14 (br, Ar-H, 1 H), 7.10 (d, *J* = 8.0 Hz, Ar-H, 1 H), 7.02 (dd, *J* = 8.0, 2.2 Hz, Ar-H, 1 H), 5.41 (dd, *J* = 11.6, 5.2 Hz, CH, 1 H), 4.23 (m, CH, 1 H), 4.23/3.89 (m+m, CH_2, 2 H), 3.80 (s, CH_3, 3 H), 3.60/3.40 (dd+dd, *J* = 15.2, 11.6 Hz, *J* = 15.2, 5.2 Hz, CH_2, 2 H), 2.76 (sp, *J* = 6.7 Hz, CH, 1 H), 2.67 (sp, *J* = 6.7 Hz, CH, 1 H), 1.25 (d, *J* = 6.7 Hz, CH_3, 3 H), 1.18 (d, *J* = 6.7 Hz, CH_3, 3 H), 1.17 (d, *J* = 6.7 Hz, CH_3, 3 H), 1.06 (d, *J* = 6.7 Hz, CH_3, 3 H), 0.57 (s, CH_3, 9 H). ^{13}C NMR (100 MHz, DMSO-*d*$_6$) *δ* 159.9, 158.8, 145.3, 144.9, 138.8, 136.3, 131.8, 130.4, 130.2, 128.7, 128.7, 127.0, 125.5, 125.3, 119.0, 114.2, 112.7, 73.6, 62.0, 55.3, 50.6, 38.0, 34.9, 28.3, 27.8, 26.0, 25.2, 25.0, 23.2, 22.5. HRMS calcd for $C_{34}H_{45}N_2O$ [M-Cl$^-$]$^+$ 497.3532, found 497.3528.

(5*S*,1'*R*)-5-*tert*-butyl-1-(2,6-di(propan-2-yl)phenyl)-3-[1-(3-methoxyphenyl)-2-phenylethyl]-4,5-dihydro-1*H*-imidazol-3-ium chloride [(5*S*,1'*R*)-9b]

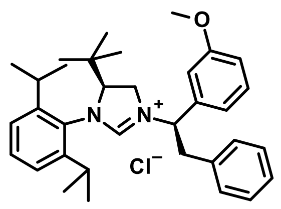

By following the synthetic procedure described for (5*S*,1'*S*)-**3a**, the reaction was performed with 470 mg (2*S*,1'*R*)-**20h** (0.966 mmol). The product (5*S*,1'*R*)-**9b** was collected as white solid (410 mg, 0.769 mmol, 79.6%). mp 252-254°C. $[\alpha]_D^{20}$ = +49 (*c* 0.01, MeOH). ECD (MeCN, λ nm (Δε) c = 1.68 x 10^{-4} M): 257 (–5.41), 229 (3.66), 215 (–4.31), 194 (19.52). ^{1}H NMR (400 MHz, DMSO-d_6) δ 8.96 (s, CH, 1 H), 7.54-7.32 (m, Ar-H, 5 H), 7.47 (t, *J* = 8.0 Hz, Ar-H, 1 H), 7.42 (t, *J* = 7.7 Hz, Ar-H, 1 H), 7.30 (d, *J* = 7.7 Hz, Ar-H, 2 H), 7.18 (d, *J* = 8.0 Hz, Ar-H, 1 H), 7.17 (s, Ar-H, 1 H), 7.06 (dd, *J* = 8.0, 2.3 Hz, Ar-H, 1 H), 5.64 (dd, *J* = 12.0, 4.2 Hz, CH, 1 H), 4.43 (dd, *J* = 12.4, 10.3 Hz, CH, 1 H), 4.05/3.82 (dd+m, *J* = 12.4, 10.3 Hz, CH_2, 2 H), 3.82 (s, CH_3, 3 H), 3.73/3.41 (dd+dd, *J* = 14.4, 12.0 Hz, *J* = 14.4, 4.2 Hz, CH_2, 2 H), 2.75 (sp, *J* = 6.7 Hz, CH, 1 H), 2.40 (sp, *J* = 6.7 Hz, CH, 1 H), 1.24 (d, *J* = 6.7 Hz, CH_3, 3 H), 1.13 (d, *J* = 6.7 Hz, CH_3, 3 H), 1.05 (d, *J* = 6.7 Hz, CH_3, 3 H), 0.95 (d, *J* = 6.7 Hz, CH_3, 3 H), 0.61 (s, CH_3, 9 H). ^{13}C NMR (100 MHz, DMSO-d_6) δ 159.7, 159.4, 145.3, 145.2, 137.5, 136.7, 131.3, 130.4, 130.3, 129.2, 128.7, 127.0, 125.3, 125.2, 119.6, 114.6, 113.4, 73.8, 61.8, 55.2, 46.5, 34.6, 28.3, 27.6, 26.1, 25.6, 25.2, 22.7, 22.3. HRMS calcd for $C_{34}H_{45}N_2O$ [M-Cl$^-$]$^+$ 497.3532, found 497.3529.

(5*S*,1'*S*)-5-*tert*-butyl-1-(2,6-di(propan-2-yl)phenyl)-3-[1-(4-methoxyphenyl)-2-phenylethyl]-4,5-dihydro-1*H*-imidazol-3-ium chloride [(5*S*,1'*S*)-10a]

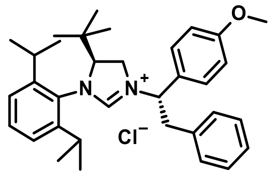

By following the synthetic procedure described for (5*S*,1'*S*)-**3a**, the reaction was performed with 150 mg (2*S*,1'*S*)-**20j** (0.308 mmol). The product (5*S*,1'*S*)-**10a** was collected as white solid (116 mg, 0.218 mmol, 70.6%). mp 249-251°C. $[\alpha]_D^{20}$ = –76 (*c* 0.01, MeOH). ECD (MeCN, λ nm (Δε) c = 1.50 x 10^{-4} M): 254 (–5.60), 211 (–2.53), 205 (–5.18), 196 (8.95), 192 (4.72), 190 (12.38). ^{1}H NMR (500 MHz, DMSO-d_6) δ 9.23 (s, CH, 1 H), 7.49 (d, *J* = 8.7 Hz, Ar-H, 2 H), 7.45-7.26 (m, Ar-H, 5 H), 7.45 (t, *J* = 7.8 Hz, Ar-H, 1 H), 7.35 (dm, *J* = 7.8 Hz, Ar-H, 1 H), 7.33 (dm, *J* = 7.8 Hz, Ar-H, 1 H), 7.05 (d, *J* = 8.7 Hz, Ar-H, 2 H), 5.40 (dd, *J* = 11.5, 5.4 Hz, CH, 1 H), 4.20 (m, CH, 1 H), 4.20/3.84 (m+dd, *J* = 8.9, 3.9 Hz, CH_2, 2 H), 3.79 (s, CH_3, 3 H), 3.60/3.36 (dd+dd, *J* = 15.0, 11.5 Hz, *J* = 15.0, 5.4 Hz, CH_2, 2 H), 2.80 (sp, *J* = 6.7 Hz, CH, 1 H), 2.62 (sp, *J* = 6.7 Hz, CH, 1 H), 1.25 (d, *J* = 6.7 Hz, CH_3, 3 H), 1.19 (d, *J* = 6.7 Hz, CH_3, 3 H), 1.19 (d, *J* = 6.7 Hz, CH_3, 3 H), 1.04 (d, *J* = 6.7 Hz, CH_3, 3 H), 0.57 (s, CH_3, 9 H). ^{13}C NMR (125 MHz, DMSO-d_6) δ 159.6, 158.7, 145.3, 145.0, 136.5, 131.9, 130.4, 129.0, 128.8, 128.6, 128.4, 127.0, 125.4, 125.3, 114.4, 73.6, 61.7, 55.3, 50.2, 38.0, 34.9, 28.3, 27.8, 26.0, 25.2, 25.0, 23.2, 22.5. HRMS calcd for $C_{34}H_{45}N_2O$ [M-Cl$^-$]$^+$ 497.3532, found 497.3531.

(5*S*,1'*R*)-5-*tert*-butyl-1-(2,6-di(propan-2-yl)phenyl)-3-[1-(4-methoxyphenyl)-2-phenylethyl]-4,5-dihydro-1*H*-imidazol-3-ium chloride [(5*S*,1'*R*)-10b]

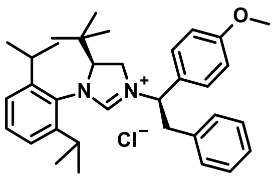

By following the synthetic procedure described for (5*S*,1'*S*)-**3a**, the reaction was performed with 1.35 g (2*S*,1'*R*)-**20i** (2.76 mmol). The product (5*S*,1'*R*)-**10b** was collected as white solid (1.20 g, 2.26 mmol, 81.7%). mp 248-250 °C. $[\alpha]_D^{20}$ = +42 (*c* 0.01, MeOH). ECD (MeCN, λ nm (Δε) c = 1.57 x 10^{-4} M): 256 (–5.65), 232 (7.55), 211 (–1.72), 197

(14.55), 190 (1.19). ^{1}H NMR (500 MHz, DMSO-*d$_6$*) δ 9.01 (s, CH, 1 H), 7.54 (d, *J* = 8.7 Hz, Ar-H, 2 H), 7.53-7.31 (m, Ar-H, 5 H), 7.42 (t, *J* = 7.9 Hz, Ar-H, 1 H), 7.29 (dm, *J* = 7.9 Hz, Ar-H, 2 H), 7.10 (d, *J* = 8.7 Hz, Ar-H, 2 H), 5.69 (dd, *J* = 12.0, 4.3 Hz, CH, 1 H), 4.39 (dd, *J* = 12.7, 10.1 Hz, CH, 1 H), 4.03/3.78 (dd+t, *J* = 12.7, 10.1 Hz, *J* = 12.7 Hz, CH_2, 2 H), 3.81 (s, CH_3, 3 H), 3.73/3.35 (dd+dd, *J* = 14.1, 12.0 Hz, *J* = 14.1, 4.3 Hz, CH_2, 2 H), 2.72 (sp, *J* = 6.7 Hz, CH, 1 H), 2.41 (sp, *J* = 6.7 Hz, CH, 1 H), 1.23 (d, *J* = 6.7 Hz, CH_3, 3 H), 1.13 (d, *J* = 6.7 Hz, CH_3, 3 H), 1.05 (d, *J* = 6.7 Hz, CH_3, 3 H), 0.95 (d, *J* = 6.7 Hz, CH_3, 3 H), 0.60 (s, CH_3, 9 H). ^{13}C NMR (125 MHz, DMSO-*d$_6$*) δ 159.7, 159.2, 145.4, 145.2, 136.9, 131.4, 130.4, 129.2, 129.1, 128.7, 127.8, 127.0, 125.3, 125.2, 114.4, 73.8, 61.3, 55.3, 46.2, 35.0, 34.6, 28.3, 27.6, 26.1, 25.6, 25.2, 22.7, 22.2. HRMS calcd for $C_{34}H_{45}N_2O$ [M-Cl$^-$]$^+$ 497.3532, found 497.3528.

(5*S*,1'*S*)-5-*tert*-butyl-1-(2,6-di(propan-2-yl)phenyl)-3-[1-(o-tolyl)-2-phenylethyl]-4,5-dihydro-1*H*-imidazol-3-ium chloride [(5*S*,1'*S*)-11a]

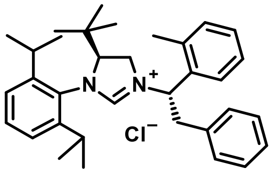

By following the synthetic procedure described for (5*S*,1'*S*)-**3a**, the reaction was performed with 188 mg (2*S*,1'*S*)-**20k** (0.399 mmol). The product (5*S*,1'*S*)-**11a** was collected as white solid (120 mg, 0.232 mmol, 58.1%). mp 247-249°C. $[\alpha]_D^{20}$ = –80 (*c* 0.01, MeOH). ECD (MeCN, λ nm ($\Delta\varepsilon$) c = 1.27 x 10^{-4} M): 282 (–2.44), 276 (–2.02), 255 (–11.64), 227 (0.79), 206 (–7.02), 196 (7.93), 194 (4.50), 190 (13.73). ^{1}H NMR (500 MHz, DMSO-*d$_6$*) δ 9.22 (s, CH, 1 H), 7.49-7.27 (m, Ar-H, 12 H), 5.55 (dd, *J* = 11.2, 5.0 Hz, CH, 1 H), 4.19 (m, CH, 1 H), 4.19/3.98 (m+m, CH_2, 2 H), 3.53/3.34 (dd+dd, *J* = 15.3, 11.2 Hz, *J* = 15.3, 5.0 Hz, CH_2, 2 H), 2.80 (sp, *J* = 6.7 Hz, CH, 1 H), 2.72 (sp, *J* = 6.7 Hz, CH, 1 H), 2.44 (s, CH_3, 3 H), 1.28 (d, *J* = 6.7 Hz, CH_3, 3 H), 1.20 (d, *J* = 6.7 Hz, CH_3, 3 H), 1.15 (d, *J* = 6.7 Hz, CH_3, 3 H), 1.13 (d, *J* = 6.7 Hz, CH_3, 3 H), 0.60 (s, CH_3, 9 H). ^{13}C NMR (125 MHz, DMSO-*d$_6$*) δ 159.0, 145.3, 144.9, 136.3, 136.2, 134.8, 131.9, 131.2, 130.4, 128.8, 128.7, 128.6, 127.0, 126.7, 125.5, 125.3, 125.2, 73.7, 58.8, 50.2, 37.8, 35.1, 28.4, 27.9, 25.9, 25.2, 25.0, 23.4, 22.5, 18.6. HRMS calcd for $C_{34}H_{45}N_2$ [M-Cl$^-$]$^+$ 481.3583, found 481.3584.

(5*S*,1'*R*)-5-*tert*-butyl-1-(2,6-di(propan-2-yl)phenyl)-3-[1-(o-tolyl)-2-phenylethyl]-4,5-dihydro-1*H*-imidazol-3-ium chloride [(5*S*,1'*R*)-11b]

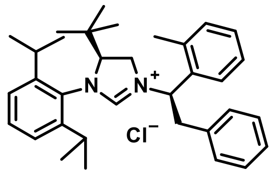

By following the synthetic procedure described for (5*S*,1'*S*)-**3a**, the reaction was performed with 188 mg (2*S*,1'*R*)-**20l** (0.399 mmol). The product (5*S*,1'*R*)-**11b** was collected as white solid (159 mg, 0.307 mmol, 77.0%). mp 213-215°C. $[\alpha]_D^{20}$ = +31 (*c* 0.01, MeOH). ECD (MeCN, λ nm ($\Delta\varepsilon$) c = 1.60 x 10^{-4} M): 272 (–7.37), 269 (–6.34), 266 (–6.87), 228 (1.37), 206 (–8.03), 194 (25.93). ^{1}H NMR (500 MHz, DMSO-*d$_6$*) δ 8.81 (s, CH, 1 H), 7.77-7.32 (m, Ar-H, 4 H), 7.56-7.32 (m, Ar-H, 5 H), 7.40 (t, *J* = 7.7 Hz, Ar-H, 1 H), 7.29 (dd, *J* = 7.7, 1.1 Hz, Ar-H, 1 H), 7.26 (dd, *J* = 7.7, 1.1 Hz, Ar-H, 1 H), 5.56 (dd, *J* = 11.2, 5.1 Hz, CH, 1 H), 4.38 (m, CH, 1 H), 4.13/3.64 (m+t, *J* = 12.5 Hz, CH_2, 2 H), 3.89/3.29 (dd+dd, *J* = 15.3, 11.2 Hz, *J* = 15.3, 5.1 Hz, CH_2, 2 H), 2.75 (sp, *J* = 6.7 Hz, CH, 1 H), 2.36 (s, CH_3, 3 H), 2.31 (sp, *J* = 6.7 Hz, CH, 1 H), 1.24 (d, *J* = 6.7 Hz, CH_3, 3 H), 1.15 (d, *J* = 6.7 Hz, CH_3, 3 H), 1.04 (d, *J* = 6.7 Hz, CH_3, 3 H), 0.83 (d, *J* = 6.7 Hz, CH_3, 3 H), 0.68 (s, CH_3, 9 H). ^{13}C NMR (125 MHz, DMSO-*d$_6$*) δ 159.6, 145.4, 145.4, 137.8, 136.7, 133.1, 131.3, 131.1, 130.3, 129.6,

129.2, 128.6, 127.3, 127.3, 126.4, 125.4, 125.1, 74.1, 59.6, 46.0, 35.4, 34.6, 28.2, 27.5, 26.2, 25.7, 25.4, 23.1, 22.4, 18.7. HRMS calcd for $C_{34}H_{45}N_2$ $[M-Cl^-]^+$ 481.3583, found 481.3580.

(5*S*,1'*S*)-5-*tert*-butyl-1-(2,6-di(propan-2-yl)phenyl)-3-[2-phenyl-1-(p-tolyl)ethyl]-4,5-dihydro-1*H*-imidazol-3-ium chloride [(5*S*,1'*S*)-12a]

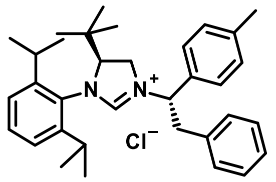

By following the synthetic procedure described for (5*S*,1'*S*)-**3a**, the reaction was performed with 310 mg (2*S*,1'*S*)-**20n** (0.660 mmol). The product (5*S*,1'*S*)-**12a** was collected as white solid (278 mg, 0.538 mmol, 81.6%). mp 260-262°C. $[\alpha]_D^{20}$ = –87 (*c* 0.01, MeOH). ECD (MeCN, λ nm (Δε) c = 1.52 x 10^{-4} M): 254 (–10.15), 229 (–0.77), 206 (–5.99), 191 (12.30). ^{1}H NMR (500 MHz, DMSO-d_6) δ 9.24 (s, CH, 1 H), 7.46-7.27 (m, Ar-H, 5 H), 7.46 (t, *J* = 7.9 Hz, Ar-H, 1 H), 7.43 (m, Ar-H, 2 H), 7.36 (m, Ar-H, 1 H), 7.34 (m, Ar-H, 1 H), 7.31 (m, Ar-H, 2 H), 5.41 (dd, *J* = 11.6, 5.3 Hz, CH, 1 H), 4.20/3.86 (m+m, CH_2, 2 H), 4.19 (m, CH, 1 H), 3.57/3.37 (dd+dd, *J* = 15.2, 11.6 Hz, *J* = 15.2, 5.3 Hz, CH_2, 2 H), 2.79 (sp, *J* = 6.7 Hz, CH, 1 H), 2.65 (sp, *J* = 6.7 Hz, CH, 1 H), 2.35 (s, CH_3, 3 H), 1.25 (d, *J* = 6.7 Hz, CH_3, 3 H), 1.19 (d, *J* = 6.7 Hz, CH_3, 3 H), 1.19 (d, *J* = 6.7 Hz, CH_3, 3 H), 1.06 (d, *J* = 6.7 Hz, CH_3, 3 H), 0.57 (s, CH_3, 9 H). ^{13}C NMR (125 MHz, DMSO-d_6) δ 158.8, 145.3, 145.0, 138.4, 136.4, 134.3, 131.9, 130.4, 129.6, 129.2, 128.7, 128.6, 127.0, 126.9, 125.4, 125.3, 73.7, 62.0, 50.4, 38.2, 38, 34.9, 28.3, 27.8, 25.9, 25.2, 25.0, 23.2, 22.5, 20.7. HRMS calcd for $C_{34}H_{45}N_2$ $[M-Cl^-]^+$ 481.3583, found 481.3581.

(5*S*,1'*R*)-5-*tert*-butyl-1-(2,6-di(propan-2-yl)phenyl)-3-[2-phenyl-1-(p-tolyl)ethyl]-4,5-dihydro-1*H*-imidazol-3-ium chloride [(5*S*,1'*R*)-12b]

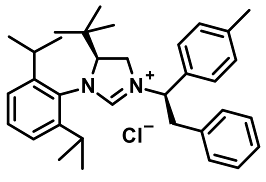

By following the synthetic procedure described for (5*S*,1'*S*)-**3a**, the reaction was performed with 430 mg (2*S*,1'*R*)-**20m** (0.915 mmol). The product (5*S*,1'*R*)-**12b** was collected as white solid (358 mg, 0.692 mmol, 75.8%). mp 265-267°C. $[\alpha]_D^{20}$ = +50 (*c* 0.01, MeOH). ECD (MeCN, λ nm (Δε) c = 1.51 x 10^{-4} M): 263 (–4.95), 228 (5.06), 210 (–1.33), 196 (15.40), 190 (–6.25). ^{1}H NMR (500 MHz, DMSO-d_6) δ 8.99 (s, CH, 1 H), 7.53-7.31 (m, Ar-H, 5 H), 7.49 (d, *J* = 8.0 Hz, Ar-H, 2 H), 7.42 (t, *J* = 7.9 Hz, Ar-H, 1 H), 7.36 (d, *J* = 8.0 Hz, Ar-H, 2 H), 7.30 (dd, *J* = 7.9, 1.8 Hz, Ar-H, 1 H), 7.29 (dd, *J* = 7.9, 1.8 Hz, Ar-H, 1 H), 5.67 (dd, *J* = 12.0, 4.0 Hz, CH, 1 H), 4.40 (dd, *J* = 12.5, 10.5 Hz, CH, 1 H), 4.03/3.78 (dd+t, *J* = 12.5, 10.5 Hz, *J* = 12.5 Hz, CH_2, 2 H), 3.73/3.37 (dd+dd, *J* = 14.2, 12.0 Hz, *J* = 14.2, 4.0 Hz, CH_2, 2 H), 2.72 (sp, *J* = 6.7 Hz, CH, 1 H), 2.42 (sp, *J* = 6.7 Hz, CH, 1 H), 2.37 (s, CH_3, 3 H), 1.23 (d, *J* = 6.7 Hz, CH_3, 3 H), 1.13 (d, *J* = 6.7 Hz, CH_3, 3 H), 1.05 (d, *J* = 6.7 Hz, CH_3, 3 H), 0.95 (d, *J* = 6.7 Hz, CH_3, 3 H), 0.60 (s, CH_3, 9 H). ^{13}C NMR (125 MHz, DMSO-d_6) δ 159.3, 145.4, 145.2, 138.6, 136.8, 133.0, 131.3, 130.4, 129.7, 129.5, 129.2, 128.7, 127.6, 127.0, 125.3, 125.2, 73.8, 61.5, 46.3, 34.8, 34.6, 28.2, 27.6, 26.1, 25.6, 25.2, 22.7, 22.2, 20.8. HRMS calcd for $C_{34}H_{45}N_2$ $[M-Cl^-]^+$ 481.3583, found 481.3580.

(5*S*,1'*S*)-5-*tert*-butyl-1-(2,6-di(propan-2-yl)phenyl)-3-[2-phenyl-1-[4-(trifluoromethyl)phenyl]ethyl]-4,5-dihydro-1*H*-imidazol-3-ium chloride [(5*S*,1'*S*)-13a]

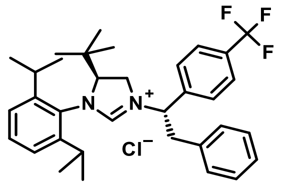

By following the synthetic procedure described for (5*S*,1'*S*)-**3a**, the reaction was performed with 1.05 g (2*S*,1'*S*)-**20p** (2.00 mmol). The product (5*S*,1'*S*)-**13a** was collected as white solid (598 mg, 1.05 mmol, 52.3%). mp 271-273°C. $[\alpha]_D^{20}$ = –70 (*c* 0.01, MeOH). ECD (MeCN, λ nm (Δε) c = 1.48 x 10^{-4} M): 255 (–11.18), 221 (0.79), 207 (–7.65), 196 (6.28). ^{1}H NMR (500 MHz, DMSO-d_6) δ 9.37 (s, CH, 1 H), 7.91 (d, *J* = 8.2 Hz, Ar-H, 2 H), 7.80 (d, *J* = 8.2 Hz, Ar-H, 2 H), 7.49-7.29 (m, Ar-H, 8 H), 5.61 (dd, *J* = 11.5, 5.4 Hz, CH, 1 H), 4.25/3.90 (dd+dd, *J* = 11.9, 7.1 Hz, *J* = 11.9, 7.1 Hz, CH_2, 2 H), 4.22 (dd, *J* = 11.9, 7.1 Hz, CH, 1 H), 3.64/3.46 (dd+dd, *J* = 15.1, 11.5 Hz, *J* = 15.1, 5.4 Hz, CH_2, 2 H), 2.78 (sp, *J* = 6.7 Hz, CH, 1 H), 2.67 (sp, *J* = 6.7 Hz, CH, 1 H), 1.25 (d, *J* = 6.7 Hz, CH_3, 3 H), 1.19 (d, *J* = 6.7 Hz, CH_3, 6 H), 1.07 (d, *J* = 6.7 Hz, CH_3, 3 H), 0.57 (s, CH_3, 9 H). ^{13}C NMR (125 MHz, DMSO-d_6) δ 159.2, 145.3, 144.9, 142.0, 136.0, 131.8, 130.4, 128.8, 128.7, 128.0, 127.1, 126.1, 126.1, 125.5, 125.3, 73.8, 61.5, 50.9, 38.0, 34.9, 28.3, 27.8, 26.0, 25.2, 25.0, 23.2, 22.5. HRMS calcd for $C_{34}H_{42}F_3N_2$ $[M-Cl^-]^+$ 535.3300, found 535.3302.

(5*S*,1'*R*)-5-*tert*-butyl-1-(2,6-di(propan-2-yl)phenyl)-3-[2-phenyl-1-[4-(trifluoromethyl)phenyl]ethyl]-4,5-dihydro-1*H*-imidazol-3-ium chloride [(5*S*,1'*R*)-13b]

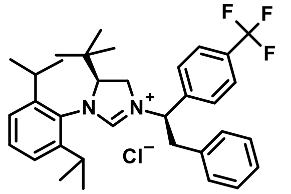

By following the synthetic procedure described for (5*S*,1'*S*)-**3a**, the reaction was performed with 1.35 g (2*S*,1'*R*)-**20o** (2.57 mmol). The product (5*S*,1'*R*)-**13b** was collected as white solid (1.31 g, 2.30 mmol, 89.3%). mp 262-264°C. $[\alpha]_D^{20}$ = +33 (*c* 0.01, MeOH). ECD (MeCN, λ nm (Δε) c = 1.50 x 10^{-4} M): 270 (–5.72), 263 (–5.24), 263 (–6.11), 230 (2.87), 222 (0.26), 213 (2.54), 207 (–2.04), 194 (23.15). ^{1}H NMR (500 MHz, DMSO-d_6) δ 9.21 (s, CH, 1 H), 7.96 (d, *J* = 8.2 Hz, Ar-H, 2 H), 7.88 (d, *J* = 8.2 Hz, Ar-H, 2 H), 7.59-7.30 (m, Ar-H, 5 H), 7.43 (t, *J* = 7.7 Hz, Ar-H, 1 H), 7.29 (d, *J* = 7.7 Hz, Ar-H, 1 H), 7.29 (d, *J* = 7.7 Hz, Ar-H, 1 H), 5.97 (dd, *J* = 12.1, 4.3 Hz, CH, 1 H), 4.43 (dd, *J* = 12.3, 10.5 Hz, CH, 1 H), 4.07/3.84 (dd+t, *J* = 12.3, 10.5 Hz, *J* = 12.3 Hz, CH_2, 2 H), 3.80/3.45 (dd+dd, *J* = 14.0, 12.1 Hz, *J* = 14.0, 4.3 Hz, CH_2, 2 H), 2.75 (sp, *J* = 6.8 Hz, CH, 1 H), 2.37 (sp, *J* = 6.8 Hz, CH, 1 H), 1.23 (d, *J* = 6.8 Hz, CH_3, 3 H), 1.13 (d, *J* = 6.8 Hz, CH_3, 3 H), 1.05 (d, *J* = 6.8 Hz, CH_3, 3 H), 0.96 (d, *J* = 6.8 Hz, CH_3, 3 H), 0.60 (s, CH_3, 9 H). ^{13}C NMR (125 MHz, DMSO-d_6) δ 159.7, 145.4, 145.2, 140.6, 136.4, 131.2, 130.4, 129.2, 128.8, 128.7, 128.5, 127.3, 127.1, 126.1, 126.1, 126.0, 125.3, 125.2, 73.9, 60.8, 46.4, 34.6, 34.6, 28.2, 27.6, 26.2, 25.6, 25.3, 22.6, 22.2. HRMS calcd for $C_{34}H_{42}F_3N_2$ $[M-Cl^-]^+$ 535.3300, found 535.3300.

(5*S*,1'*S*)-5-*tert*-butyl-1-(2,6-di(propan-2-yl)phenyl)-3-[1-phenyl-2-(p-tolyl)ethyl]-4,5-dihydro-1*H*-imidazol-3-ium chloride [(5*S*,1'*S*)-14a]

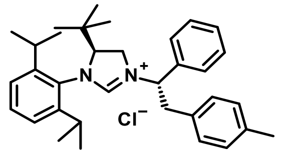

By following the synthetic procedure described for (5*S*,1'*S*)-**3a**, the reaction was performed with 195 mg (2*S*,1'*S*)-**20q** (0.414 mmol). The product (5*S*,1'*S*)-**14a** was collected as white solid (211 mg, 0.408 mmol, 98.5%). mp 268-270°C. $[\alpha]_D^{20}$ = –88 (*c* 0.01, MeOH). ECD (MeCN, λ nm (Δε) c = 1.53 x 10^{-4} M): 253 (–10.46), 225 (–0.60), 207 (–5.61), 198 (3.76), 195 (–2.59). ^{1}H NMR (500 MHz, DMSO-d_6) δ 9.17 (s, CH, 1 H), 7.57-7.32 (m, Ar-H, 8 H), 7.29 (d, *J* = 7.8 Hz, Ar-H, 2 H), 7.19 (d, *J* = 7.8 Hz, Ar-H, 2 H), 5.41 (dd, *J* = 11.5, 5.5 Hz, CH, 1

H), 4.22 (m, CH, 1 H), 4.22/3.83 (m+m, CH_2, 2 H), 3.52/3.37 (dd+dd, *J* = 15.2, 11.5 Hz, *J* = 15.2, 5.5 Hz, CH_2, 2 H), 2.79 (sp, *J* = 6.7 Hz, CH, 1 H), 2.66 (sp, *J* = 6.7 Hz, CH, 1 H), 2.30 (s, CH_3, 3 H), 1.25 (d, *J* = 6.7 Hz, CH_3, 3 H), 1.20 (d, *J* = 6.7 Hz, CH_3, 3 H), 1.18 (d, *J* = 6.7 Hz, CH_3, 3 H), 1.05 (d, *J* = 6.7 Hz, CH_3, 3 H), 0.57 (s, CH_3, 9 H). ^{13}C NMR (125 MHz, DMSO-d_6) δ 158.9, 145.3, 145.0, 137.3, 136.1, 133.2, 131.8, 130.4, 129.2, 129.1, 128.9, 128.6, 126.9, 125.5, 125.3, 73.7, 64.9, 62.2, 50.4, 37.7, 34.9, 28.3, 27.8, 25.9, 25.1, 25.0, 23.2, 22.5, 20.6. HRMS calcd for $C_{34}H_{45}N_2$ [M-Cl$^-$]$^+$ 481.3583, found 481.3577.

(5*S*,1'*R*)-5-*tert*-butyl-1-(2,6-di(propan-2-yl)phenyl)-3-[1-phenyl-2-(p-tolyl)ethyl]-4,5-dihydro-1*H*-imidazol-3-ium chloride [(5*S*,1'*R*)-14b]

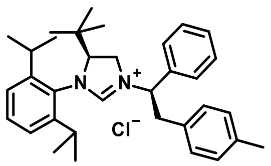

By following the synthetic procedure described for (5*S*,1'*S*)-**3a**, the reaction was performed with 110 mg (2*S*,1'*R*)-**20r** (0.234 mmol). The product (5*S*,1'*R*)-**14b** was collected as white solid (84 mg, 0.162 mmol, 69.5%). mp 279-281 °C. $[\alpha]_D^{20}$ = +45 (*c* 0.01, MeOH). ECD (MeCN, λ nm (Δε) c = 1.67 x 10^{-4} M): 262 (–3.77), 231 (2.32), 221 (–3.69), 199 (11.96), 191 (–27.46). ^{1}H NMR (500 MHz, DMSO-d_6) δ 8.93 (s, CH, 1 H), 7.62-7.46 (m, Ar-H, 5 H), 7.43 (t, *J* = 7.7 Hz, Ar-H, 1 H), 7.38 (d, *J* = 7.8 Hz, Ar-H, 2 H), 7.30 (d, *J* = 7.7 Hz, Ar-H, 2 H), 7.21 (d, *J* = 7.8 Hz, Ar-H, 2 H), 5.61 (dd, *J* = 12.0, 4.2 Hz, CH, 1 H), 4.44 (dd, *J* = 12.6, 10.4 Hz, CH, 1 H), 4.04/3.80 (dd+t, *J* = 12.6, 10.4 Hz, *J* = 12.6 Hz, CH_2, 2 H), 3.69/3.35 (dd+dd, *J* = 14.5, 12.0 Hz, *J* = 14.5, 4.2 Hz, CH_2, 2 H), 2.74 (sp, *J* = 6.7 Hz, CH, 1 H), 2.42 (sp, *J* = 6.7 Hz, CH, 1 H), 2.34 (s, CH_3, 3 H), 1.24 (d, *J* = 6.7 Hz, CH_3, 3 H), 1.14 (d, *J* = 6.7 Hz, CH_3, 3 H), 1.05 (d, *J* = 6.7 Hz, CH_3, 3 H), 0.94 (d, *J* = 6.7 Hz, CH_3, 3 H), 0.61 (s, CH_3, 9 H). ^{13}C NMR (125 MHz, DMSO-d_6) δ 159.3, 145.4, 145.2, 136.0, 133.6, 131.3, 130.4, 129.3, 129.2, 129.1, 129.1, 127.7, 125.3, 125.2, 73.8, 62.0, 46.4, 34.6, 34.4, 28.3, 27.7, 25.9, 25.6, 25.2, 22.7, 22.2, 20.8. HRMS calcd for $C_{34}H_{45}N_2$ [M-Cl$^-$]$^+$ 481.3583, found 481.3580.

(5*S*,1'*S*)-5-*tert*-butyl-3-[2-(4-chlorophenyl)-1-phenylethyl]-1-(2,6-di(propan-2-yl)phenyl)-4,5-dihydro-1*H*-imidazol-3-ium chloride [(5*S*,1'*S*)-15a]

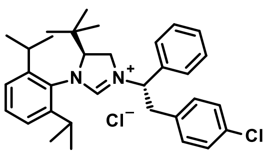

By following the synthetic procedure described for (5*S*,1'*S*)-**3a**, the reaction was performed with 250 mg (2*S*,1'*S*)-**20s** (0.509 mmol). The product (5*S*,1'*S*)-**15a** was collected as white solid (219 mg, 0.407 mmol, 80.0%). mp 284-286 °C. $[\alpha]_D^{20}$ = –82 (*c* 0.01, MeOH). ECD (MeCN, λ nm (Δε) c = 1.62 x 10^{-4} M): 253 (–11.34), 225 (2.47), 206 (–7.99), 197 (8.17). ^{1}H NMR (400 MHz, DMSO-d_6) δ 9.26 (s, CH, 1 H), 7.58-7.30 (m, Ar-H, 12 H), 5.50 (dd, *J* = 11.4, 5.4 Hz, CH, 1 H), 4.24 (m, CH, 1 H), 4.24/3.86 (m+m, CH_2, 2 H), 3.64/3.42 (dd+dd, *J* = 15.3, 11.4 Hz, *J* = 15.3, 5.4 Hz, CH_2, 2 H), 2.78 (sp, *J* = 6.7 Hz, CH, 1 H), 2.64 (sp, *J* = 6.7 Hz, CH, 1 H), 1.26 (d, *J* = 6.7 Hz, CH_3, 3 H), 1.21 (d, *J* = 6.7 Hz, CH_3, 3 H), 1.17 (d, *J* = 6.7 Hz, CH_3, 3 H), 1.05 (d, *J* = 6.7 Hz, CH_3, 3 H), 0.60 (s, CH_3, 9 H). ^{13}C NMR (100 MHz, DMSO-d_6) δ 159.0, 145.3, 144.9, 136.9, 135.5, 131.7, 131.7, 130.7, 130.4, 129.2, 129.0, 128.6, 127.0, 125.5, 125.3, 73.7, 64.9, 61.8, 49.9, 36.9, 34.9, 28.4, 27.9, 25.9, 25.1, 25.1, 23.2, 22.5. HRMS calcd for $C_{33}H_{42}ClN_2$ [M-Cl$^-$]$^+$ 501.3037, found 501.3036.

(5*S*,1'*R*)-5-*tert*-butyl-3-[2-(4-chlorophenyl)-1-phenylethyl]-1-(2,6-di(propan-2-yl)phenyl)-4,5-dihydro-1*H*-imidazol-3-ium chloride [(5*S*,1'*R*)-15b]

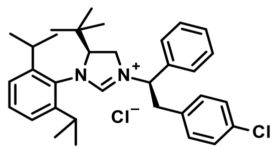

By following the synthetic procedure described for (5*S*,1'*S*)-**3a**, the reaction was performed with 280 mg (2*S*,1'*R*)-**20t** (0.570 mmol). The product (5*S*,1'*R*)-**15b** was collected as white solid (264 mg, 0.491 mmol, 86.1%). mp 282-284 °C. $[\alpha]_D^{20}$ = +36 (*c* 0.01, MeOH). ECD (MeCN, λ nm (Δε) c = 1.19 x 10^{-4} M): 263 (–3.33), 233 (3.65), 222 (–6.52), 212 (0.50), 209 (–0.08), 198 (17.12), 190 (–12.15). ^{1}H NMR (400 MHz, DMSO-d_6) δ 9.06 (s, CH, 1 H), 7.64-7.46 (m, Ar-H, 9 H), 7.43 (t, *J* = 7.8 Hz, Ar-H, 1 H), 7.30 (d, *J* = 7.8 Hz, Ar-H, 2 H), 5.71 (dd, *J* = 12.1, 4.1 Hz, CH, 1 H), 4.45 (dd, *J* = 12.1, 10.4 Hz, CH, 1 H), 4.06/3.80 (dd+dd, *J* = 12.1, 10.4 Hz, *J* = 12.1, 4.7 Hz, CH_2, 2 H), 3.77/3.40 (dd+dm, *J* = 12.1, 6.1 Hz, *J* = 4.1 Hz, CH_2, 2 H), 2.74 (sp, *J* = 6.7 Hz, CH, 1 H), 2.37 (sp, *J* = 6.7 Hz, CH, 1 H), 1.24 (d, *J* = 6.7 Hz, CH_3, 3 H), 1.17 (d, *J* = 6.7 Hz, CH_3, 3 H), 1.05 (d, *J* = 6.7 Hz, CH_3, 3 H), 0.98 (d, *J* = 6.7 Hz, CH_3, 3 H), 0.62 (s, CH_3, 9 H). ^{13}C NMR (100 MHz, DMSO-d_6) δ 159.4, 145.3, 145.1, 135.9, 135.7, 131.8, 131.2, 130.5, 129.2, 128.7, 127.7, 125.3, 125.2, 73.8, 61.8, 46.3, 34.6, 34.1, 28.3, 27.8, 25.9, 25.6, 25.2, 22.6, 22.2. HRMS calcd for $C_{33}H_{42}ClN_2$ $[M-Cl^-]^+$ 501.3037, found 501.3036.

(5*S*,1'*S*)-5-*tert*-butyl-3-[2-(2-chlorophenyl)-1-phenylethyl]-1-(2,6-di(propan-2-yl)phenyl)-4,5-dihydro-1*H*-imidazol-3-ium chloride [(5*S*,1'*S*)-16a]

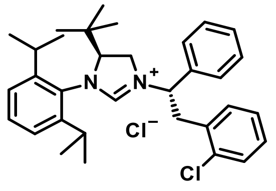

By following the synthetic procedure described for (5*S*,1'*S*)-**3a**, the reaction was performed with 2.02 g (2*S*,1'*S*)-**20u** (4.11 mmol). The product (5*S*,1'*S*)-**16a** was collected as white solid (1. 39 g, 2.58 mmol, 62.7%). mp 278-280 °C. $[\alpha]_D^{20}$ = –97 (*c* 0.01, MeOH). ECD (MeCN, λ nm (Δε) c = 1.59 x 10^{-4} M): 254 (–10.34), 227 (–0.92), 207 (–8.45), 191 (18.80). ^{1}H NMR (500 MHz, DMSO-d_6) δ 9.32 (s, CH, 1 H), 7.60-7.31 (m, Ar-H, 12 H), 5.50 (dd, *J* = 10.7, 5.1 Hz, CH, 1 H), 4.34 (m, CH, 1 H), 4.33/3.95 (m+dd, *J* = 10.3, 6.1 Hz, CH_2, 2 H), 3.80/3.47 (dd+dd, *J* = 15.5, 10.7 Hz, *J* = 15.5, 5.1 Hz, CH_2, 2 H), 2.89 (sp, *J* = 6.7 Hz, CH, 1 H), 2.73 (sp, *J* = 6.7 Hz, CH, 1 H), 1.28 (d, *J* = 6.7 Hz, CH_3, 3 H), 1.21 (d, *J* = 6.7 Hz, CH_3, 3 H), 1.10 (d, *J* = 6.7 Hz, CH_3, 3 H), 1.06 (d, *J* = 6.7 Hz, CH_3, 3 H), 0.65 (s, CH_3, 9 H). ^{13}C NMR (125 MHz, DMSO-d_6) δ 159.1, 145.4, 145.0, 136.7, 133.8, 133.6, 131.8, 130.7, 130.4, 129.8, 129.2, 129.1, 129.1, 127.3, 127.0, 125.5, 125.2, 73.8, 60.7, 49.7, 35.3, 28.3, 28.0, 25.5, 25.3, 25.2, 23.2, 22.5. HRMS calcd for $C_{33}H_{42}ClN_2$ $[M-Cl^-]^+$ 501.3037, found 501.3011.

(5*S*,1'*R*)-5-*tert*-butyl-3-[2-(2-chlorophenyl)-1-phenylethyl]-1-(2,6-di(propan-2-yl)phenyl)-4,5-dihydro-1*H*-imidazol-3-ium chloride [(5*S*,1'*R*)-16b]

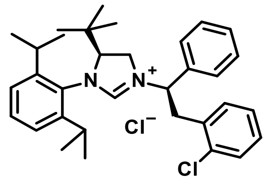

By following the synthetic procedure described for (5*S*,1'*S*)-**3a**, the reaction was performed with 941 mg (2*S*,1'*R*)-**20v** (1.92 mmol). The product (5*S*,1'*R*)-**16b** was collected as white solid (625 mg, 1.16 mmol, 60.7%). mp 267-269 °C. $[\alpha]_D^{20}$ = +70 (*c* 0.01, MeOH). ECD (MeCN, λ nm (Δε) c = 1.81 x 10^{-4} M): 259 (–4.17), 229 (4.03), 207 (–5.37), 196 (6.59), 191 (–9.05). ^{1}H NMR (500 MHz, DMSO-d_6) δ 9.06 (s, CH, 1 H), 7.68-7.26 (m, Ar-H, 12 H), 5.66 (dd, *J* = 11.2, 4.1 Hz, CH, 1 H), 4.51 (dd, *J* = 12.3, 10.4 Hz, CH, 1 H), 4.17/3.88

(dd+dd, *J* = 12.6, 10.4 Hz, *J* = 12.6, 12.3 Hz, CH_2, 2 H), 3.88/3.49 (dd+dd, *J* = 14.5, 11.2 Hz, *J* = 14.5, 4.1 Hz, CH_2, 2 H), 2.77 (sp, *J* = 6.7 Hz, CH, 1 H), 2.53 (sp, *J* = 6.7 Hz, CH, 1 H), 1.25 (d, *J* = 6.7 Hz, CH_3, 3 H), 1.18 (d, *J* = 6.7 Hz, CH_3, 3 H), 1.07 (d, *J* = 6.7 Hz, CH_3, 3 H), 0.91(d, *J* = 6.7 Hz, CH_3, 3 H), 0.69 (s, CH_3, 9 H). ^{13}C NMR (125 MHz, DMSO-d_6) δ 159.7, 145.4, 145.1, 135.7, 134.1, 133.5, 131.7, 131.2, 130.5, 130.0, 129.4, 129.3, 127.6, 127.4, 125.4, 125.2, 74.0, 60.4, 47.0, 33.5, 28.3, 27.8, 25.8, 25.7, 25.2, 22.7, 22.2. HRMS calcd for $C_{33}H_{42}ClN_2$ [M-Cl$^-$]$^+$ 501.3037, found 501.3020.

(1'*R*)-3-[2-(4-chlorophenyl)-1-phenylethyl]-1-(2,6-di(propan-2-yl)phenyl)-4,5-dihydro-1*H*-imidazol-3-ium chloride [(*R*)-17a]

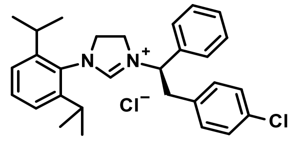

By following the synthetic procedure described for (5*S*,1'*S*)-**3a**, the reaction was performed with 713 mg (*R*)-**20x** (1.64 mmol). The product (*R*)-**17a** was collected as a white solid (309 mg, 0.641 mmol, 39.2%). mp 87-89 °C. $[\alpha]_D^{20}$ = –51 (*c* 0.01, MeOH). ECD (MeCN, λ nm (Δε) c = 2.91 x 10^{-4} M): 247 (– 4.32), 221 (5.74), 209 (– 0.80), 207 (1.12), 204 (0.13), 197 (7.06), 194 (4.91), 192 (8.64). ^{1}H NMR (500 MHz, DMSO-d_6) δ 9.24 (s, CH, 1 H), 7.56-7.42 (m, Ar-H, 10 H), 7.33 (d, *J* = 7.8 Hz, Ar-H, 2 H), 5.42 (dd, *J* = 11.0, 5.1 Hz, CH, 1 H), 4.29-3.81 (m, CH_2, 4 H), 3.63/3.38 (dd+dd, *J* = 14.5, 11.0 Hz, *J* = 14.5, 5.1 Hz, CH_2, 2 H), 2.69 (sp, *J* = 6.7 Hz, CH, 1 H), 2.35 (sp, *J* = 6.7 Hz, CH, 1 H), 1.17 (d, *J* = 6.7 Hz, CH_3, 3 H), 1.16 (d, *J* = 6.7 Hz, CH_3, 3 H), 1.08 (d, *J* = 6.7 Hz, CH_3, 3 H), 1.02 (d, *J* = 6.7 Hz, CH_3, 3 H). ^{13}C NMR (125 MHz, DMSO-d_6) δ 157.9, 146.1, 136.5, 135.9, 131.7, 130.9, 130.8, 130.1, 129.2, 129.0, 128.7, 127.3, 124.7, 124.7, 62.0, 52.7, 46.2, 35.8, 28.0, 28.0, 24.6, 24.5, 23.5, 23.3. HRMS calcd for $C_{29}H_{34}ClN_2$ [M-Cl$^-$]$^+$ 445.2411, found 445.2405.

(1'*S*)-3-[2-(4-chlorophenyl)-1-phenylethyl]-1-(2,6-di(propan-2-yl)phenyl)-4,5-dihydro-1*H*-imidazol-3-ium chloride [(*S*)-17b]

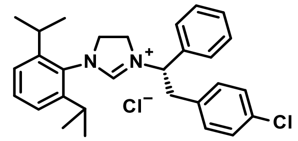

By following the synthetic procedure described for (5*S*,1'*S*)-**3a**, the reaction was performed with 669 mg (*S*)-**20w** (1.53 mmol). The product (*S*)-**17b** was collected as off-white solid (369 mg, 0.766 mmol, 49.8%). mp 83-85 °C. $[\alpha]_D^{20}$ = +40 (*c* 0.01, MeOH). ECD (MeCN, λ nm (Δε) c = 2.83 x 10^{-4} M): 241 (5.37), 222 (–4.96), 207 (0.95), 194 (11.35). ^{1}H NMR (500 MHz, DMSO-d_6) δ 9.24 (s, CH, 1 H), 7.56-7.42 (m, Ar-H, 10 H), 7.33 (d, *J* = 7.8 Hz, Ar-H, 2 H), 5.42 (dd, *J* = 11.0, 5.1 Hz, CH, 1 H), 4.29-3.81 (m, CH_2, 4 H), 3.63/3.38 (dd+dd, *J* = 14.5, 11.0 Hz, *J* = 14.5, 5.1 Hz, CH_2, 2 H), 2.69 (sp, *J* = 6.7 Hz, CH, 1 H), 2.35 (sp, *J* = 6.7 Hz, CH, 1 H), 1.17 (d, *J* = 6.7 Hz, CH_3, 3 H), 1.16 (d, *J* = 6.7 Hz, CH_3, 3 H), 1.08 (d, *J* = 6.7 Hz, CH_3, 3 H), 1.02 (d, *J* = 6.7 Hz, CH_3, 3 H). ^{13}C NMR (125 MHz, DMSO-d_6) δ 157.9, 146.1, 136.5, 135.9, 131.7, 130.9, 130.8, 130.1, 129.2, 129.0, 128.7, 127.3, 124.7, 124.7, 62.0, 52.7, 46.2, 35.8, 28.0, 28.0, 24.6, 24.5, 23.5, 23.3. HRMS calcd for $C_{29}H_{34}ClN_2$ [M-Cl$^-$]$^+$ 445.2411, found 445.2405.

1.2 General Procedure for Preparation of Aldehyde (18a-b)

(*S*)-2-(2,6-di(propan-2-yl)anilino)-3,3-dimethylbutanal [(*S*)-18a]

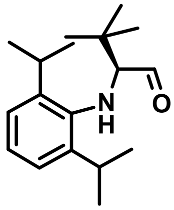

(*S*)-**18a** was prepared by following published procedures.[1]

(2*R*)-2-(2,6-di(propan-2-yl)anilino)-3,3-dimethylbutanal [(2*R*)-18b]

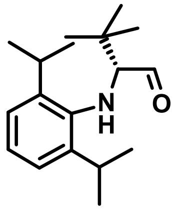

(*R*)-**18b** was prepared by following published procedures.[1]

1.3 General Procedure for Preparation of Butane-1,2-diamine (19a-c)

Example for compound **19a:**

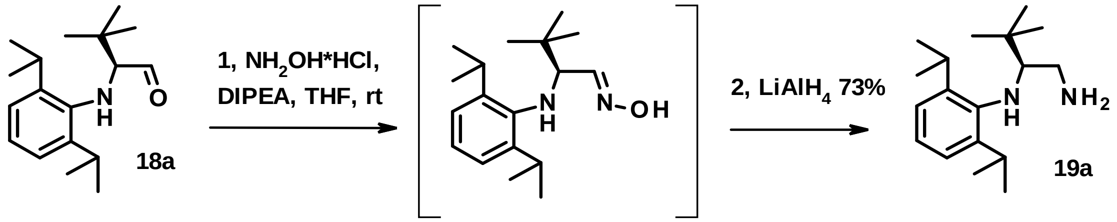

15.9 g (*S*)-**18a** (57.77 mmol) was dissolved in 115 mL THF (2 mL/mmol) under inert atmosphere then 6.02 g, hydroxylamine hydrogen chloride (86.66 mmol) and 30.2 mL DIPEA (173.3 mmol) were added at room temperature. The reaction mixture was stirred for 5 hours, the expected oxime intermediate formed. In the next step 8.77 g $LiAlH_4$ (231.1 mmol) was suspended in 288 mL THF (5 mL/mmol) at 0°C and the previous mixture was added slowly to the reaction mixture after filtration off the DIPEA hydrochloride, then it was stirred for 16 hours at room temperature. Reaction progress was monitored by HPLC.

The reaction mixture was cooled to 0°C, 8.7 ml water, 17.4 ml 2M NaOH and 17.4 ml water was added. 80 mL MTBE was added to the mixture at the previous temperature and stirred for 10 min. The mixture was filtered, washed with MTBE. The crude product was purified *via* column chromatography using DCM and 1.2% methanolic ammonia as eluents.

(2*S*)-*N*-(2,6-di(propan-2-yl)phenyl)-3,3-dimethylbutane-1,2-diamine [(*S*)-19a]

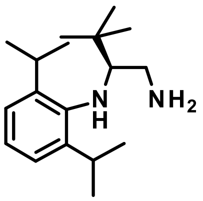

(*S*)-**19a** was collected as a yellow oil (12.0 g, 43.4 mmol, 75.2%).^{1}H NMR (500 MHz, DMSO-d_6) δ 7.00 (d, J = 7.6 Hz, Ar-H, 2 H), 6.87 (t, J = 7.6 Hz, Ar-H, 1 H), 3.38 (sp, J = 6.8 Hz, CH, 2 H), 3.38 (d, J = 5.0 Hz, NH, 1 H), 2.85 (m, CH, 1 H), 2.66/2.58 (dd+dd, J = 13.1, 5.0 Hz, J = 13.1 Hz, J = 5.0 Hz, CH_2, 2 H), 1.20 (brs, NH_2, 2 H), 1.16 (d, J = 6.8 Hz, CH_3, 6 H), 1.15 (d, J = 6.8 Hz, CH_3, 6 H), 0.99 (s, CH_3, 9 H). ^{13}C NMR (125 MHz, DMSO-d_6) δ 142.7, 140.0, 123.4, 121.6, 69.3, 42.8, 35.3, 27.4, 26.9, 24.1, 24.0. HRMS calcd for $C_{18}H_{33}N_2$ $[M + H]^+$ 277.2638, found 277.2628.

(2*R*)-*N*-(2,6-di(propan-2-yl)phenyl)-3,3-dimethylbutane-1,2-diamine [(*R*)-19b]

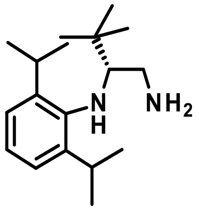

(*R*)-**19b** was prepared by following published procedures.[1]

***N*-(2,6-di(propan-2-yl)phenyl)ethane-1,2-diamine (19c)**

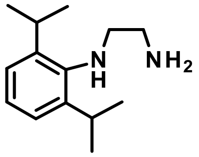

19c was prepared by following published procedures.[2]

1.4 General Procedure for Preparation of Substituated Diamine Derivatives (20a-z)

Method A (20a-t,w-x)

Example for Compound **20a-b:**

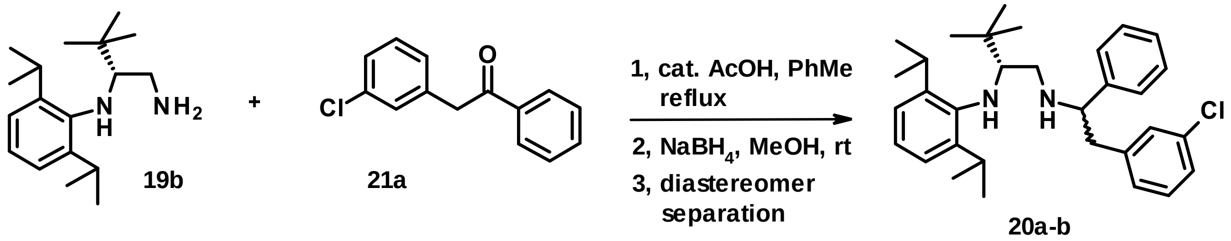

3.23 g **21a** (14.0 mmol) was dissolved in 70 mL toluene (5 mL/mmol) under inert atmosphere, then 3.87 g (*R*)-**19b** (14.0 mmol) was added in one portion and 74 µL acetic acid (1.40 mmol) was also added. The mixture was stirred at 120°C. The reaction mixture was stirred for 4h, until all the **21a** was reacted, as monitored by GC-MS. When the starting material was reacted, the mixture was allowed to cool down at room temperature and the volatiles were removed under reduced pressure. The resulting Schiff-base was dissolved in 140 mL MeOH (10 mL/mmol) and 2.12 g $NaBH_4$ (55.9 mmol) was added carefully. The mixture was stirred for 16 h at room temperature, and monitored by LC-MS. The reaction mixture was quenched with the addition of saturated aqueous NH_4Cl solution, and then it was extracted with EtOAc. The combined organic layers were dried over $MgSO_4$. The volatiles were removed under reduced pressure and the residue was purified via flash column chromatography using a mixture of heptane and EtOAc as eluents. (2*R*,1'*R*)-**20a** and (2*R*,1'*S*)-**20b** were collected as a diastereomeric mixture (2.80 g, 5.67 mmol, 40.6%). Diastereomers were separated by column chromatography using a mixture of heptane and EtOAc as eluents.

(2*R*)-*N*^{1}-[(1*R*)-2-(3-chlorophenyl)-1-phenylethyl]-*N*^{2}-(2,6-di(propan-2-yl)phenyl)-3,3-dimethylbutane-1,2-diamine [(2*R*,1'*R*)-20a] and (2*R*)-*N*^{1}-[(1*S*)-2-(3-chlorophenyl)-1-phenylethyl]-*N*^{2}-(2,6-di(propan-2-yl)phenyl)-3,3-dimethylbutane-1,2-diamine [(2*R*,1'*S*)-20b]

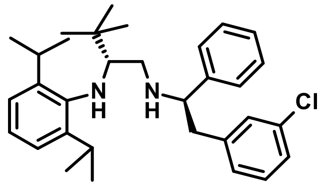

(2*R*,1'*R*)-**20a** was obtained as a single diastereomer, eluted first, and collected as yellow oil (1.13 g, 2.29 mmol, 16.4%). ^{1}H NMR (500 MHz, DMSO-d_6) δ 7.18 (m, Ar-H, 1 H), 7.17 (m, Ar-H, 2 H), 7.13 (m, Ar-H, 1 H), 7.00 (m, Ar-H, 1 H), 6.99 (m, Ar-H, 2 H), 6.98 (s, Ar-H, 1 H), 6.94 (m, Ar-H, 2 H), 6.93 (m, Ar-H, 1 H), 6.85 (m, Ar-H, 1 H), 3.39 (dd, J = 7.5, 6.3 Hz, CH, 1 H), 3.19 (sp, J = 6.7 Hz, CH, 2 H), 3.08 (d, J = 11.6 Hz, NH, 1 H), 2.78 (m, CH, 1 H), 2.62/2.58 (m+m, CH_2, 2 H), 2.45/2.14 (dd+dd, J = 11.6, 4.6 Hz, J = 11.6, 4.6 Hz, CH_2, 2 H), 1.44 (brs, NH, 1 H), 1.12 (d, J = 6.7 Hz, CH_3, 6 H), 1.02 (d, J = 6.7 Hz, CH_3, 6 H), 0.85 (s, CH_3, 9 H). ^{13}C NMR (125 MHz, DMSO-d_6) δ 143.7, 141.9, 141.3, 140.5, 132.6, 129.7, 128.9, 127.9, 127.8, 127.0, 126.6, 126.0, 123.4, 122.2, 67.2, 63.5, 47.4, 43.8, 35.2, 35.1, 27.0, 26.9, 24.0, 23.8. HRMS calcd for $C_{32}H_{44}ClN_2$ $[M + H]^+$ 491.3187, found 491.3167.

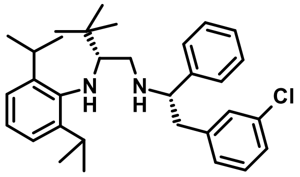

(2*R*,1'*S*)-**20b** was found a single diastereomer, eluted second, and collected as yellow oil (1.62 g, 3.30 mmol, 23.6%). ^{1}H NMR (400 MHz, DMSO-d_6) δ 7.16 (m, Ar-H, 1 H), 7.16 (m, Ar-H, 1 H), 7.14 (m, Ar-H, 2 H), 7.12 (m, Ar-H, 1 H), 6.99 (d, J = 7.7 Hz, Ar-H, 2 H), 6.99 (s, Ar-H, 1 H), 6.96 (d, J = 8.0 Hz, Ar-H, 2 H), 6.90 (t, J = 7.7 Hz, Ar-H, 1 H), 6.85 (m, Ar-H, 1 H), 3.55 (t, J = 6.8 Hz, CH, 1 H), 3.24 (sp, J = 6.8 Hz, CH, 2 H), 3.22 (d, J = 11.6 Hz, NH, 1 H), 2.92 (m, CH, 1 H), 2.64/2.58 (dd+dd, J = 13.2, 6.8 Hz, J = 13.2, 6.8 Hz, CH_2, 2 H), 2.35 (m, CH_2, 2 H), 1.38 (brs, NH, 1 H), 1.13 (d, J = 6.8 Hz, CH_3, 6 H), 1.07 (d, J = 6.8 Hz, CH_3, 6 H), 0.89 (s, CH_3, 9 H). ^{13}C NMR (100 MHz, DMSO-d_6) δ 143.7, 141.9, 141.3, 140.5, 132.6, 129.7, 128.9, 127.9, 127.8, 127.0, 126.6, 126.0, 123.4, 122.2, 67.2, 63.5, 47.4, 43.8, 35.2, 35.1, 27.0, 26.9, 24.0, 23.8. HRMS calcd for $C_{32}H_{44}ClN_2$ $[M + H]^+$ 491.3187, found 491.3175.

(2*R*)-*N*^{2}-(2,6-di(propan-2-yl)phenyl)-3,3-dimethyl-*N*^{1}-[(1'*R*)-2-phenyl-1-[3-(trifluoromethyl)phenyl]ethyl]butane-1,2-diamine [(2*R*,1'*R*)-20c] and (2*R*)-*N*^{2}-(2,6-di(propan-2-yl)phenyl)-3,3-dimethyl-*N*^{1}-[(1'*S*)-2-phenyl-1-[3-(trifluoromethyl)phenyl]ethyl]butane-1,2-diamine [(2*R*,1'*S*)-20d]

By following the synthetic procedure described for (2*R*,1'*R*)-**20a** and (2*R*,1'*S*)-**20b**, the reaction was performed with 2.92 g **21b** (11.0 mmol) and 3.05 g (*R*)-**19b** (11.0 mmol). The reaction time of the first step was 4 h and that of the second step was 16 h. (2*R*,1'*R*)-**20c** and (2*R*,1'*S*)-**20d** were collected as a diastereomeric mixture (1490 mg, 2.83 mmol, 25.8%). Diastereomers were separated by column chromatography using a mixture of heptane and DCM as eluents.

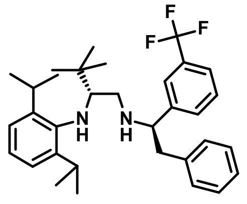

(2*R*,1'*R*)-**20c** was obtained as a single diastereomer, eluted first, and collected as yellow oil (815 mg, 1.55 mmol, 14.1%). ^{1}H NMR (500 MHz, DMSO-d_6) δ 7.49 (d, J = 7.8 Hz, Ar-H, 1 H), 7.39 (t, J = 7.8 Hz, Ar-H, 1 H), 7.35 (s, Ar-H, 1 H), 7.22-6.86 (m, Ar-H, 5 H), 7.20 (d, J = 7.8 Hz, Ar-H, 1 H), 6.98 (d, J = 7.4 Hz, Ar-H, 2 H), 6.92 (m, Ar-H, 1 H), 3.54 (t, J = 6.0 Hz, CH, 1 H), 3.22 (sp, J = 6.7 Hz, CH, 2 H), 3.10 (d, J = 11.6 Hz, NH, 1 H), 2.80 (m, CH, 1 H), 2.63/2.57 (dd+dd, J = 13.3, 6.0 Hz, J = 13.3, 6.0 Hz, CH_2, 2 H), 2.44/2.17 (m+m, CH_2, 2 H), 1.55 (brs, NH, 1 H), 1.14 (d, J = 6.7 Hz, CH_3, 6 H), 1.01 (d, J = 6.7 Hz, CH_3, 6 H), 0.82 (s, CH_3, 9 H). ^{13}C NMR (125 MHz, DMSO-d_6) δ 145.6, 142.0, 140.5, 138.0, 131.4, 129.1, 128.8, 128.0, 126.2, 123.4, 122.2, 67.3, 63.2, 47.5, 44.4, 35.1, 27.0, 26.8, 24.0, 23.7. HRMS calcd for $C_{33}H_{44}F_3N_2$ $[M + H]^+$ 525.3451, found 525.3454.

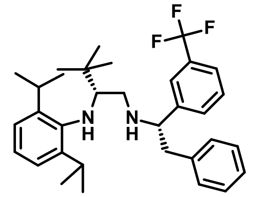

(2*R*,1'*S*)-**20d** was found a single diastereomer, eluted second, and collected as yellow oil (660 mg, 1.26 mmol, 11.4%). ^{1}H NMR (500 MHz, DMSO-d_6) δ 7.44 (dm, *J* = 7.7 Hz, Ar-H, 1 H), 7.32 (m, Ar-H, 1 H), 7.31 (t, *J* = 7.7 Hz, Ar-H, 1 H), 7.21-6.89 (m, A-H, 5 H), 7.21 (dm, *J* = 7.7 Hz, Ar-H, 1 H), 6.95 (d, *J* = 7.5 Hz, Ar-H, 2 H), 6.87 (t, *J* = 7.5 Hz, Ar-H, 1 H), 3.69 (m, CH, 1 H), 3.22 (sp, *J* = 6.7 Hz, CH, 2 H), 3.17 (d, *J* = 11.5 Hz, NH, 1 H), 2.88 (m, CH, 1 H), 2.67/2.63 (dd+dd, *J* = 13.2, 8.6 Hz, *J* = 13.2, 7.4 Hz, CH_2, 2 H), 2.36/2.27 (m+m, CH_2, 2 H), 1.50 (brs, NH, 1 H), 1.13 (d, *J* = 6.7 Hz, CH_3, 6 H), 1.04 (d, *J* = 6.7 Hz, CH_3, 6 H), 0.88 (s, CH_3, 9 H). ^{13}C NMR (125 MHz, DMSO-d_6) δ 145.0, 142.0, 140.3, 138.2, 130.9, 129.2, 128.9, 128.0, 126.0, 123.3, 122.1, 66.5, 63.7, 48.0, 43.9, 35.0, 26.9, 24.1, 23.7. HRMS calcd for $C_{33}H_{44}F_3N_2$ $[M + H]^+$ 525.3451, found 525.3444.

(2*S*)-*N*^{2}-(2,6-di(propan-2-yl)phenyl)-*N*^{1}-[(1'*S*)-1-(2-methoxyphenyl)-2-phenylethyl]-3,3-dimethylbutane-1,2-diamine [(2*S*,1'*S*)-20e] and (2*S*)-*N*^{2}-(2,6-di(propan-2-yl)phenyl)-*N*^{1}-[(1'*R*)-1-(2-methoxyphenyl)-2-phenylethyl]-3,3-dimethylbutane-1,2-diamine [(2*S*,1'*R*)-20f]

By following the synthetic procedure described for (2*R*,1'*R*)-**20a** and (2*R*,1'*S*)-**20b**, the reaction was performed with 930 mg **21c** (4.11 mmol) and 1.13 g (*S*)-**19a** (4.11 mmol). The reaction time of the first step was 5 h and that of the second was 16 h. (2*S*,1'*S*)-**20e** and (2*S*,1'*R*)-**20f** were collected as a diastereomeric mixture (1.26 g, 2.59 mmol, 63.0%). Diastereomers were separated bycolumn chromatography using a mixture of heptane and DCM as eluents.

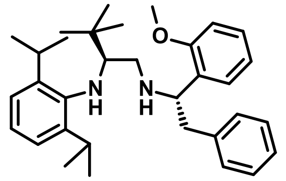

(2*S*,1'*S*)-**20e** was a obtained as a single diastereomer, eluted first, and collected as yellow oil (760 mg, 1.56 mmol, 38.0%). ^{1}H NMR (500 MHz, DMSO-d_6) δ 7.20-6.84 (m, Ar-H, 5 H), 7.10 (m, Ar-H, 1 H), 7.00 (d, *J* = 7.5 Hz, Ar-H, 2 H), 6.90 (m, Ar-H, 1 H), 6.88 (m, Ar-H, 1 H), 6.84 (m, Ar-H, 1 H), 6.80 (t, *J* = 7.5 Hz, Ar-H, 1 H), 3.90 (m, CH, 1 H), 3.68 (s, CH_3, 3 H), 3.25 (sp, *J* = 6.7 Hz, CH, 2 H), 3.14 (d, *J* = 11.4 Hz, NH, 1 H), 2.88 (m, CH, 1 H), 2.67/2.45 (dd+m, *J* = 13.3, 4.7 Hz, CH_2, 2 H), 2.48/2.20 (m+dd, *J* = 11.8, 4.6 Hz, CH_2, 2 H), 1.37 (brs, NH, 1 H), 1.12 (d, *J* = 6.7 Hz, CH_3, 6 H), 1.06 (d, *J* = 6.7 Hz, CH_3, 6 H), 0.83 (s, CH_3, 9 H). ^{13}C NMR (125 MHz, DMSO-d_6) δ 156.8, 142.1, 140.0, 139.0, 131.2, 129.0, 127.9, 127.3, 126.7, 125.8, 123.4, 121.9, 120.0, 110.6, 67.2, 57.5, 55.2, 48.3, 42.6, 35.2, 27.0, 27.0, 24, 23.8. HRMS calcd for $C_{33}H_{47}N_2O$ $[M + H]^+$ 487.3683, found 487.3685.

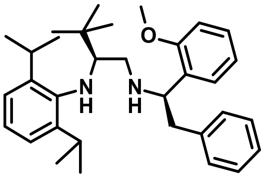

(2*S*,1'*R*)-**20f** was found a single diastereomer, eluted second, and collected as yellow oil (490 mg, 1.01 mmol, 24.5%). ^{1}H NMR (500 MHz, DMSO-d_6) δ 7.20-6.86 (m, Ar-H, 5 H), 7.08 (td, *J* = 7.4, 1.5 Hz, Ar-H, 1 H), 7.00 (d, *J* = 7.6 Hz, Ar-H, 2 H), 6.90 (m, Ar-H, 1 H), 6.83 (dm, *J* = 7.4 Hz, Ar-H, 1 H), 6.81 (dd, *J* = 7.4, 1.5 Hz, Ar-H, 1 H), 6.68 (tm, *J* = 7.4 Hz, Ar-H, 1 H), 3.95 (m, CH, 1 H), 3.61 (s, CH_3, 3 H), 3.27 (sp, *J* = 6.8 Hz, 2 H), 3.21 (d, *J* = 11.5 Hz, NH, 1 H), 2.90 (m, CH, 1 H), 2.71/2.53 (dd+dm, *J* = 13.3, 6.3 Hz, *J* = 6.3 Hz, CH_2, 2 H), 2.33/2.23 (m+m, CH_2, 2 H), 1.42 (brs, NH, 1 H), 1.14 (d, *J* = 6.8 Hz, CH_3, 6 H), 1.07 (d, *J* = 6.8 Hz, CH_3, 6 H), 0.88 (s, CH_3, 9 H). ^{13}C NMR (125 MHz, DMSO-d_6) δ 156.9, 142.4, 140.1, 139.2, 130.7, 129.1, 127.7, 127.3, 127.0, 125.7, 123.3, 121.8, 120.2, 110.6, 66.6, 55.2, 48.2, 43.1, 42.5, 42.0, 35.0, 27.1, 26.9, 24.0, 23.8. HRMS calcd for $C_{33}H_{47}N_2O$ $[M + H]^+$ 487.3683, found 487.3684.

(2*S*)-*N*^{2}-(2,6-di(propan-2-yl)phenyl)-*N*^{1}-[(1'*S*)-1-(3-methoxyphenyl)-2-phenylethyl]-3,3-dimethylbutane-1,2-diamine [(2*S*,1'*S*)-20g] and (2*S*)-*N*^{2}-(2,6-di(propan-2-yl)phenyl)-*N*^{1}-

[(1'*R*)-1-(3-methoxyphenyl)-2-phenylethyl]-3,3-dimethylbutane-1,2-diamine [(2*S*,1'*R*)-20h]

By following the synthetic procedure described for (2*R*,1'*R*)-**20a** and (2*R*,1'*S*)-**20b**, the reaction was performed with 1.19 g **21d** (5.25 mmol) and 1.32 g (*S*)-**19a** (4.77 mmol). The reaction time of the first step was 3.5 h and that of the second was 16 h. (2*S*,1'*S*)-**20g** and (2*S*,1'*R*)-**20h** were collected as a diastereomeric mixture (1.04 g, 2.14 mmol, 44.8%). Diastereomers were separated by column chromatography using heptane and acetone as eluents.

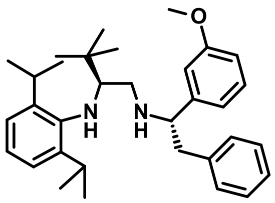

(2*S*,1'*S*)-**20g** was obtained as a single diastereomer, eluted first, and collected as yellow oil (373 mg, 0.766 mmol, 16.1%). ^{1}H NMR (400 MHz, DMSO-d_6) 7.16 (m, Ar-H, 2 H), 7.13 (m, Ar-H, 1 H), 7.08 (t, *J* = 7.8 Hz, Ar-H, 1 H), 6.99 (d, *J* = 7.5 Hz, Ar-H, 2 H), 6.91 (dd, *J* = 7.5, 1.5 Hz, Ar-H, 1 H), 6.89 (m, Ar-H, 2 H), 6.70 (dd, *J* = 7.8, 2,5 Hz, Ar-H, 1 H), 6.66 (brs, Ar-H, 1 H), 6.55 (d, *J* = 7.8 Hz, Ar-H, 1 H), 3.66 (s, CH_3, 3 H), 3.44 (dd, *J* = 7.8, 5.6 Hz, CH, 1 H), 3.24 (sp, *J* = 6.7 Hz, CH, 2 H), 3.12 (d, *J* = 11.5 Hz, NH, 1 H), 2.84 (sp, *J* = 6.7 Hz, CH, 1 H), 2.62/2.53 (dd+dd, *J* = 13.2, 6.6 Hz, *J* = 13.2, 7.8 Hz, CH_2, 2 H), 2.47/2.19 (br+dd, *J* = 12.2, 5.0 Hz, CH_2, 2 H), 1.39 (brs, NH, 1 H), 1.14 (d, *J* = 6.7 Hz, CH_3, 6 H), 1.05 (d, *J* = 6.7 Hz, CH_3, 6 H), 0.93 (s, CH_3, 9 H). ^{13}C NMR (100 MHz, DMSO-d_6) δ 159.1, 145.8, 142.1, 140.2, 138.5, 129.0, 128.8, 128.0, 126.0, 123.4, 122.1, 119.4, 112.2, 112.1, 67.3, 64.0, 54.8, 48.0, 44.6, 27.0, 26.9, 24.0, 23.8. HRMS calcd for $C_{33}H_{47}N_2O$ [M + H]$^+$ 487.3683, found 487.3688.

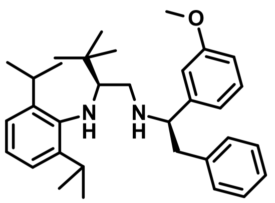

(2*S*,1'*R*)-**20h** was obtained as a single diastereomer, eluted second, and collected as yellow oil (498 mg, 1.02 mmol, 21.4%). ^{1}H NMR (400 MHz, DMSO-d_6) 7.16 (m, Ar-H, 2 H), 7.10 (m, Ar-H, 1 H), 7.03 (t, *J* = 7.8 Hz, Ar-H, 1 H), 6.99 (d, *J* = 7.5 Hz, Ar-H, 2 H), 6.93 (m, Ar-H, 2 H), 6.89 (dd, *J* = 8.3, 7.1 Hz, Ar-H, 1 H), 6.66 (dd, *J* = 7.8, 2.3 Hz, Ar-H, 1 H), 6.59 (brs, Ar-H, 1 H), 6.53 (d, *J* = 7.8 Hz, Ar-H, 1 H), 3.61 (s, CH_3, 3 H), 3.54 (t, *J* = 6.8 Hz, CH, 1 H), 3.26 (sp, *J* = 6.7 Hz, CH, 2 H), 3.21 (d, *J* = 11.1 Hz, NH, 1 H), 2.91 (m, CH, 1 H), 2.66/2.58 (dd+dd, *J* = 13.1, 6.8 Hz, *J* = 13.1, 6.8 Hz, CH_2, 2 H), 2.34 (m, CH_2, 2 H), 1.39 (brs, NH, 1 H), 1.13 (d, *J* = 6.7 Hz, CH_3, 6 H), 1.08 (d, *J* = 6.7 Hz, CH_3, 6 H), 0.88 (s, CH_3, 9 H). ^{13}C NMR (100 MHz, DMSO-d_6) δ 159.0, 145.2, 142.4, 140.0, 138.8, 129.2, 128.8, 127.9, 125.9, 123.3, 121.9, 119.2, 112.2, 112.1, 66.3, 64.1, 54.7, 48.3, 44.1, 27.1, 27.0, 24.1, 23.8. HRMS calcd for $C_{33}H_{47}N_2O$ [M + H]$^+$ 487.3683, found 487.3682.

(2*S*)-*N*^{2}-(2,6-di(propan-2-yl)phenyl)-*N*^{1}-[(1'*R*)-1-(4-methoxyphenyl)-2-phenylethyl]-3,3-dimethylbutane-1,2-diamine [(2*S*,1'*R*)-20i] and (2*S*)-*N*^{2}-(2,6-di(propan-2-yl)phenyl)-*N*^{1}-[(1'*S*)-1-(4-methoxyphenyl)-2-phenylethyl]-3,3-dimethylbutane-1,2-diamine [(2*S*,1'*S*)-20j]

By following the synthetic procedure described for (2*R*,1'*R*)-**20a** and (2*R*,1'*S*)-**20b**, the reaction was performed with 2.00 g **21e** (8.84 mmol) and 2.44 g (*S*)-**19a** (8.84 mmol). Reaction time of the first step was 5 h and that of the second was 16 h. (2*S*,1'*R*)-**20i** and (2*S*,1'*S*)-**20j** were collected as a diastereoisomeric mixture (2.34 g, 4.81 mmol, 54.4%). Diastereomers were separated by preparative chiral HPLC, on OD-I (50×500 mm, 20 µm, Flow rate: 50 mL/min) column.

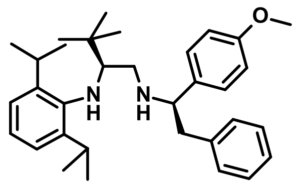

By chiral HPLC analysis, (2*S*,1'*R*)-**20i** was found a single enantiomer and eluted first on the Chiralcel OD-I column using MTBE/heptane (2:98) and 0.1% diethylamine as eluents (t_R = 14.75 min). (2*S*,1'*R*)-**20i** was collected as yellow oil (1.34 g, 2.76 mmol, 31.2%). ^{1}H NMR (500 MHz, DMSO-d_6) δ 7.18-6.85 (m, Ar-H, 5 H), 7.00 (d, *J* = 7.6 Hz, Ar-H, 2 H), 6.91 (t, *J* = 7.6 Hz, Ar-H, 1 H), 6.86 (d, *J* = 8.6 Hz, Ar-H, 2 H), 6.67 (d, *J* = 8.6 Hz, Ar-H, 2 H), 3.68 (s, CH_3, 3 H), 3.50 (t, *J* = 6.7 Hz, CH, 1 H), 3.25 (sp, *J* = 6.7 Hz, CH, 2 H), 3.21 (d, *J* = 11.5 Hz, NH, 1 H), 2.91 (m, CH, 1 H), 2.60/2.53 (dd+dd, *J* = 13.0, 6.7 Hz, *J* = 13.0, 6.7 Hz, CH_2, 2 H), 2.33 (m, CH_2, 2 H), 1.28 (brs, NH, 1 H), 1.14 (d, *J* = 6.7 Hz, CH_3, 6 H), 1.08 (d, *J* = 6.7 Hz, CH_3, 6 H), 0.88 (s, CH_3, 9 H). ^{13}C NMR (125 MHz, DMSO-d_6) δ 157.8, 142.4, 140.1, 138.8, 135.1, 129.2, 127.9, 127.9, 125.8, 123.4, 121.9, 113.3, 66.3, 63.2, 54.9, 48.2, 44.3, 35.0, 27.0, 27.0, 24.1, 23.8. HRMS calcd for $C_{33}H_{47}N_2O$ $[M + H]^+$ 487.3683, found 487.3685.

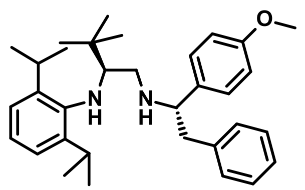

By chiral HPLC analysis, (2*S*,1'*S*)-**20j** was found a single enantiomer and eluted second on the OD-I column using MTBE/heptane (2:98) and 0.1% diethylamine as eluents (t_R = 21.19 min). (2*S*,1'*S*)-**20j** was collected as yellow oil (974 mg, 2.00 mmol, 22.6%). ^{1}H NMR (500 MHz, DMSO-d_6) δ 7.20-6.84 (m, Ar-H, 5 H), 7.00 (d, *J* = 7.2 Hz, Ar-H, 2 H), 6.94 (m, Ar-H, 1 H), 6.87 (d, *J* = 8.6 Hz, Ar-H, 2 H), 6.72 (d, *J* = 8.6 Hz, Ar-H, 2 H), 3.69 (s, CH_3, 3 H), 3.38 (t, *J* = 6.7 Hz, CH, 1 H), 3.23 (sp, *J* = 6.7 Hz, 2 H), 3.10 (d, *J* = 11.7 Hz, NH, 1 H), 2.81 (m, CH, 1 H), 2.58/2.53 (dd+dm, *J* = 13.5, 5.8 Hz, *J* = 7.6 Hz, CH_2, 2 H), 2.45/2.16 (m+m, CH_2, 2 H), 1.34 (brs, NH, 1 H), 1.14 (d, *J* = 6.7 Hz, CH_3, 6 H), 1.05 (d, *J* = 6.7 Hz, CH_3, 6 H), 0.83 (s, CH_3, 9 H). ^{13}C NMR (125 MHz, DMSO-d_6) δ 157.9, 142.1, 140.3, 138.7, 135.8, 129.0, 128.0, 128.0, 125.9, 123.5, 122.1, 113.2, 67.1, 63.3, 54.9, 47.7, 44.6, 35.2, 27.0, 26.9, 24.0, 23.8. HRMS calcd for $C_{33}H_{47}N_2O$ $[M + H]^+$ 487.3683, found 487.3686.

(2*S*)-*N*^{2}-(2,6-di(propan-2-yl)phenyl)-3,3-dimethyl-*N*^{1}-[(1'*S*)-1-(o-tolyl)-2-phenylethyl]butane-1,2-diamine [(2*S*,1'*S*)-20k] and (2*S*)-*N*^{2}-(2,6-di(propan-2-yl)phenyl)-3,3-dimethyl-*N*^{1}-[(1'*R*)-1-(o-tolyl)-2-phenylethyl]butane-1,2-diamine [(2*S*,1'*R*)-20l]

By following the synthetic procedure described for (2*R*,1'*R*)-**20a** and (2*R*,1'*S*)-**20b**, the reaction was performed with 783 mg **21f** (3.72 mmol) and 1.03 g (*S*)-**19a** (3.72 mmol). The reaction time of the first step was 4 h and that of the second was 16 h. (2*S*,1'*S*)-**20k** and (2*S*,1'*R*)-**20l** were collected as a diastereomeric mixture (590 mg, 1.25 mmol, 33.6%). Diastereomers were separated by column chromatography using a mixture of heptane and EtOAc as eluents.

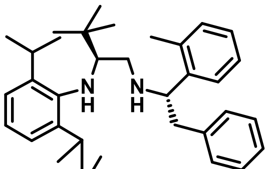

(2*S*,1'*S*)-**20k** was obtained a single diastereomer, eluted first, and collected as yellow oil (195 mg, 0.414 mmol, 11.1%). The product was decomposed during the structure measurents.

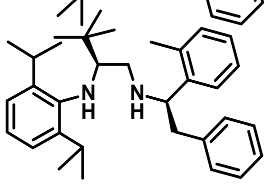

(2*S*,1'*R*)-**20l** was obtained as a single diastereomer, eluted second, and collected as yellow oil (236 mg, 0.501 mmol, 13.5%). ^{1}H NMR (500 MHz, DMSO-d_6) δ 7.20-6.84 (m, Ar-H, 9 H), 7.00 (d, *J* = 7.5 Hz, Ar-H, 2 H), 6.90 (d, *J* = 7.5 Hz, Ar-H, 1 H), 3.83 (t, *J* = 6.6 Hz, CH, 1 H), 3.25 (sp, *J* = 6.7 Hz, CH, 2 H), 3.22 (d, *J* = 11.5 Hz, NH, 1 H), 2.90 (m, CH, 1 H), 2.62/2.53 (dd+dd, *J* = 13.1, 6.6 Hz, *J* = 13.1, 6.6 Hz, CH_2, 2 H), 2.35/2.27 (m+m, CH_2, 2 H), 1.96 (s, CH_3, 3 H), 1.37 (brs, NH, 1 H), 1.14 (d, *J* = 6.7 Hz, CH_3, 6 H), 1.08 (d, *J* = 6.7 Hz, CH_3, 6 H), 0.87 (s, CH_3, 9 H). ^{13}C NMR (125 MHz, DMSO-d_6) δ 142.4, 141.3, 140.0, 138.7, 135.2, 129.7, 129.2, 127.9, 126.0, 125.9, 125.9, 125.6, 123.4,

121.9, 66.5, 59.3, 48.1, 43.5, 27.0, 27.0, 24.0, 23.8, 18.7. HRMS calcd for $C_{33}H_{47}N_2$ [M + H]$^+$ 471.3734, found 471.3739.

(2*S*)-*N²*-(2,6-di(propan-2-yl)phenyl)-3,3-dimethyl-*N¹*-[(1'*R*)-2-phenyl-1-(p-tolyl)ethyl]butane-1,2-diamine [(2*S*,1'*R*)-20m] and (2*S*)-*N²*-(2,6-di(propan-2-yl)phenyl)-3,3-dimethyl-*N¹*-[(1'*S*)-2-phenyl-1-(p-tolyl)ethyl]butane-1,2-diamine [(2*S*,1'*S*)-20n]

By following the synthetic procedure described for (2*R*,1'*R*)-**20a** and (2*R*,1'*S*)-**20b**, the reaction was performed with 956 mg **21g** (4.55 mmol) and 1.26 g (*S*)-19a (4.55 mmol). The reaction time of the first step was 4 h and that of the second was 16 h. (2*S*,1'*R*)-**20m** and (2*S*,1'*S*)-**20n** were collected as a diastereomeric mixture (1.11 g, 2.36 mmol, 52.0%). Diastereomers were separated by chiral preparative chromatography, on OD (50×500 mm, 20 μm, Flow rate: 50 mL/min) column.

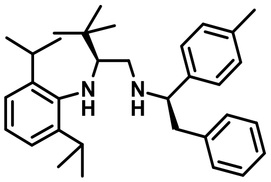

By chiral HPLC analysis, (2*S*,1'*R*)-**20m** was found to be a single enantiomer and eluted first on the OD column using heptane and 0.1% diethylamine as eluents (t_R = 6.45 min). (2*S*,1'*R*)-**20m** was collected as yellow oil (438 mg, 0.931 mmol, 20.5%). ^{1}H NMR (500 MHz, DMSO-*d₆*) δ 7.20-6.85 (m, Ar-H, 5 H), 7.00 (d, *J* = 7.5 Hz, Ar-H, 2 H), 6.92 (m, Ar-H, 2 H), 6.91 (m, Ar-H, 1 H), 6.84 (d, *J* = 8.0 Hz, Ar-H, 2 H), 3.52 (m, 1 H), 3.25 (sp, *J* = 6.7 Hz, CH, 2 H), 3.21 (d, *J* = 11.5 Hz, NH, 1 H), 2.91 (m, CH, 1 H), 2.62/2.56 (dd+dd, *J* = 13.6, 7.1 Hz, *J* = 12.9, 7.1 Hz, CH_2, 2 H), 2.32 (m, CH_2, 2 H), 2.21 (s, CH_3, 3 H), 1.32 (brs, NH, 1 H), 1.14 (d, *J* = 6.7 Hz, CH_3, 6 H), 1.08 (d, *J* = 6.7 Hz, CH_3, 6 H), 0.87 (s, CH_3, 9 H). ^{13}C NMR (125 MHz, DMSO-*d₆*) δ 142.5, 140.3, 140.0, 138.8, 135.3, 129.2, 128.5, 127.9, 126.8, 125.8, 123.4, 121.8, 66.4, 63.6, 48.2, 44.2, 35.1, 27.1, 27.0, 24.1, 23.8, 20.7. HRMS calcd for $C_{33}H_{47}N_2$ [M + H]$^+$ 471.3734, found 471.3736.

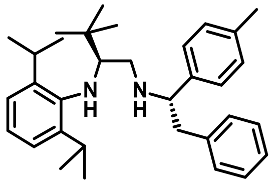

By chiral HPLC analysis, (2*S*,1'*S*)-**20n** was found to be a single enantiomer and eluted second on the OD column using heptane with 0.1% diethylamine as eluents (t_R = 8.30 min). (2*S*,1'*S*)-**20n** was collected as yellow oil (319 mg, 0.678 mmol, 14.9%). ^{1}H NMR (500 MHz, DMSO-*d₆*) δ 7.20-6.84 (m, Ar-H, 5 H), 7.00 (d, *J* = 7.4 Hz, Ar-H, 2 H), 6.98 (d, *J* = 8.1 Hz, Ar-H, 2 H), 6.93 (dm, *J* = 7.4 Hz, Ar-H, 1 H), 6.86 (d, *J* = 8.1 Hz, Ar-H, 2 H), 3.41 (m, CH, 1 H), 3.23 (sp, *J* = 6.7 Hz, CH, 2 H), 3.11 (d, *J* = 11.4 Hz, NH, 1 H), 2.82 (m, CH, 1 H), 2.60/2.53 (dd+dd, *J* = 13.4, 5.4 Hz, *J* = 13.4, 5.4 Hz, CH_2, 2 H), 2.44/2.17 (m+dd, *J* = 12.2, 4.4 Hz, CH_2, 2 H), 2.23 (s, CH_3, 3 H), 1.35 (brs, NH, 1 H), 1.13 (d, *J* = 6.7 Hz, CH_3, 6 H), 1.05 (d, *J* = 6.7 Hz, CH_3, 6 H), 0.82 (s, CH_3, 9 H). ^{13}C NMR (125 MHz, DMSO-*d₆*) δ 142.1, 140.9, 140.3, 138.7, 135.4, 129.0, 128.5, 128.0, 126.9, 125.9, 123.4, 122.0, 67.2, 63.7, 47.9, 44.6, 35.2, 27.0, 26.9, 24.0, 23.8, 20.7. HRMS calcd for $C_{33}H_{47}N_2$ [M + H]$^+$ 471.3734, found 471.3736.

(2*S*)-*N²*-(2,6-di(propan-2-yl)phenyl)-3,3-dimethyl-*N¹*-[(1'*R*)-2-phenyl-1-[4-(trifluoromethyl)phenyl]ethyl]butane-1,2-diamine [(2*S*,1'*R*)-20o] and (2*S*)-*N²*-(2,6-di(propan-2-yl)phenyl)-3,3-dimethyl-*N¹*-[(1'*S*)-2-phenyl-1-[4-(trifluoromethyl)phenyl]ethyl]butane-1,2-diamine [(2*S*,1'*S*)-20p]

By following the synthetic procedure described for (2*R*,1'*R*)-**20a** and (2*R*,1'*S*)-**20b**, the reaction was performed with 2.50 g **21h** (9.46 mmol) and 2.62 g (*S*)-**19a** (9.46 mmol). The reaction time of the first step was 4 h and thst of the second was 16 h. (2*S*,1'*R*)-**20o** and (2*S*,1'*S*)-**20p** were collected as a diastereomeric mixture (2.43 g, 4.63 mmol, 48.9%). Diastereomers were separated by chiral preparative chromatography, on OD (50×500mm, 20μm, Flow rate: 50 mL/min) column.

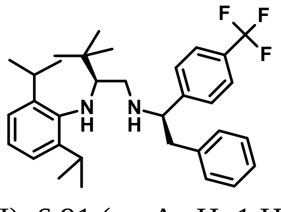

By chiral HPLC analysis, (2S,1'R)-**20o** was obtained as a single diastereoisomer and eluted first on the OD column using heptane and 0.1% diethylamine as eluents (t_R = 5.80 min). (2S,1'R)-**20o** was collected as yellow oil (1.36 g, 2.60 mmol, 27.4%). ^{1}H NMR (500 MHz, DMSO-d_6) δ 7.39 (d, J = 7.0 Hz, Ar-H, 2 H), 7.24-6.92 (m, Ar-H, 5 H), 7.10 (d, J = 7.0 Hz, Ar-H, 2 H), 6.95 (d, J = 6.6 Hz, Ar-H, 2 H), 6.91 (m, Ar-H, 1 H), 3.69 (t, J = 6.6 Hz, CH, 1 H), 3.16 (sp, J = 6.7 Hz, CH, 2 H), 3.11 (d, J = 11.5 Hz, NH, 1 H), 2.85 (m, CH, 1 H), 2.68/2.60 (dd+dd, J = 13.3, 6.6 Hz, J = 13.3, 7.6 Hz, CH_2, 2 H), 2.31/2.18 (m+m, CH_2, 2 H), 1.43 (brs, NH, 1 H), 1.14 (d, J = 6.7 Hz, CH_3, 6 H), 0.99 (d, J = 6.7 Hz, CH_3, 6 H), 0.89 (s, CH_3, 9 H). ^{13}C NMR (125 MHz, DMSO-d_6) δ 148.4, 141.8, 140.7, 138.2, 129.1, 128.0, 127.5, 127.1, 126.1, 124.7, 124.7, 123.3, 122.3, 67.0, 63.6, 47.6, 44.0, 34.9, 26.9, 26.8, 24.1, 23.6. HRMS calcd for $C_{33}H_{44}F_3N_2$ $[M + H]^+$ 525.3451, found 525.3452.

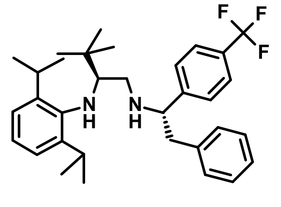

By chiral HPLC analysis, (2S,1'S)-**20p** was obtained as a single diastereoisomer and eluted second on the OD column using heptane and 0.1% diethylamine as eluents (t_R = 7.92 min). (2S,1'S)-**20p** was collected as yellow oil (1.06 g, 2.02 mmol, 21.3%). ^{1}H NMR (500 MHz, DMSO-d_6) δ 7.50 (d, J = 8.0 Hz, Ar-H, 2 H), 7.23-6.89 (m, Ar-H, 5 H), 7.10 (d, J = 8.0 Hz, Ar-H, 2 H), 6.99 (m, Ar-H, 2 H), 6.97 (m, Ar-H, 1 H), 3.51 (t, J = 6.6 Hz, CH, 1 H), 3.18 (sp, J = 6.7 Hz, CH, 2 H), 3.06 (d, J = 11.5 Hz, NH, 1 H), 2.76 (m, CH, 1 H), 2.64/2.60 (dd+dd, J = 13.4, 6.6 Hz, J = 13.4, 8.1 Hz, CH_2, 2 H), 2.45/2.09 (m+dd, J = 11.6, 3.2 Hz, CH_2, 2 H), 1.58 (brs, NH, 1 H), 1.14 (d, J = 6.7 Hz, CH_3, 6 H), 0.99 (d, J = 6.7 Hz, CH_3, 6 H), 0.83 (s, CH_3, 9 H). ^{13}C NMR (125 MHz, DMSO-d_6) δ 149.0, 141.9, 140.7, 138.2, 129.1, 128.1, 127.8, 126.2, 124.7, 124.7, 123.5, 122.4, 67.0, 63.3, 47.1, 44.0, 35.1, 27.0, 26.8, 24.0, 23.7. HRMS calcd for $C_{33}H_{44}F_3N_2$ $[M + H]^+$ 525.3451, found 525.3454.

(2*S*)-*N*^{2}-(2,6-di(propan-2-yl)phenyl)-3,3-dimethyl-*N*^{1}-[(1'*S*)-1-phenyl-2-(p-tolyl)ethyl]butane-1,2-diamine [(2*S*,1'*S*)-20q] and (2*S*)-*N*^{2}-(2,6-di(propan-2-yl)phenyl)-3,3-dimethyl-*N*^{1}-[(1'*R*)-1-phenyl-2-(p-tolyl)ethyl]butane-1,2-diamine [(2*S*,1'*R*)-20r]

By following the synthetic procedure described for (2R,1'R)-**20a** and (2R,1'S)-**20b**, the reaction was performed with 950 mg **21i** (4.52 mmol) and 1.25 g (S)-**19a** (4.52 mmol). The reaction time of the first step was 1 h and thst of the second was 16 h. (2S,1'S)-**20q** and (2S,1'R)-**20r** were collected as a diastereomeric mixture (323 mg, 0.687 mmol, 15.2%). Diastereoisomers were separated by column chromatography using a mixture of heptane and DCM as eluents.

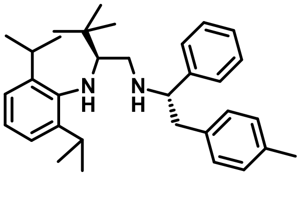

(2S,1'S)-**20q** was obtained as a single diastereoisomer, eluted first, and collected as yellow oil (198 mg, 0.421 mmol, 9.31%). ^{1}H NMR (500 MHz, DMSO-d_6) δ 7.18 (t, J = 7.1 Hz, Ar-H, 2 H), 7.13 (t, J = 7.1 Hz, Ar-H, 1 H), 7.00 (d, J = 7.1 Hz, Ar-H, 2 H), 7.00 (d, J = 7.0 Hz, Ar-H, 2 H), 6.99 (dd, J = 7.0, 1.2 Hz, Ar-H, 1 H), 6.96 (d, J = 7.8 Hz, Ar-H, 2 H), 6.77 (d, J = 7.8 Hz, Ar-H, 2 H), 3.42 (t, J = 6.8 Hz, CH, 1 H), 3.24 (sp, J = 6.7 Hz, CH, 2 H), 3.11 (d, J = 11.5 Hz, NH, 1 H), 2.84 (m, CH, 1 H), 2.59/2.51 (dd+m, J = 13.1, 5.5 Hz, CH_2, 2 H), 2.45/2.18 (m+dd, J = 12.6, 5.5 Hz, CH_2, 2 H), 2.22 (s, CH_3, 3 H), 1.38 (brs, NH, 1 H), 1.15 (d, J = 6.7 Hz, CH_3, 6 H), 1.05 (d, J = 6.7 Hz, CH_3, 6 H), 0.83 (s, CH_3, 9 H). ^{13}C NMR (125 MHz, DMSO-d_6) δ 144.1, 142.1, 140.3, 135.4, 134.8, 128.9, 128.6, 127.9, 127.0, 126.5, 123.4, 122.1, 67.2, 64.1, 47.9, 44.2, 35.2, 27.0, 26.9, 24.0, 23.8, 20.6. HRMS calcd for $C_{33}H_{47}N_2$ $[M + H]^+$ 471.3734, found 471.3739.

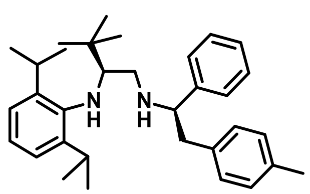

(2*S*,1'*R*)-**20r** was obtained as a single diastereoisomer, eluted second, and collected as yellow oil (115 mg, 0.245 mmol, 5.41%). ^{1}H NMR (400 MHz, DMSO-d_6) δ 7.12 (m, Ar-H, 1 H), 7.12 (m, Ar-H, 2 H), 7.01 (d, *J* = 7.0 Hz, Ar-H, 2 H), 6.95 (d, *J* = 7.9 Hz, Ar-H, 2 H), 6.95 (d, *J* = 7.3 Hz, Ar-H, 2 H), 6.91 (dd, *J* = 8.2, 7.0 Hz, Ar-H, 1 H), 6.77 (d, *J* = 7.9 Hz, Ar-H, 2 H), 3.53 (q, *J* = 5.5 Hz, CH, 1 H), 3.26 (sp, *J* = 6.9 Hz, CH, 2 H), 3.22 (d, *J* = 11.7 Hz, NH, 1 H), 2.92 (dt, *J* = 11.7, 5.2 Hz, CH, 1 H), 2.60/2.51 (dd+m, *J* = 12.9, 7.1 Hz, CH_2, 2 H), 2.35/2.33 (m+m, CH_2, 2 H), 2.20 (s, CH_3, 3 H), 1.34 (q, *J* = 5.5 Hz, NH, 1 H), 1.15 (d, *J* = 6.9 Hz, CH_3, 6 H), 1.08 (d, *J* = 6.9 Hz, CH_3, 6 H), 0.88 (s, CH_3, 9 H). ^{13}C NMR (100 MHz, DMSO-d_6) δ 143.4, 142.4, 140, 135.5, 134.7, 129.0, 128.5, 127.9, 126.9, 126.5, 123.4, 121.9, 66.3, 63.9, 48.3, 43.9, 35.0, 27.0, 27.0, 24.0, 23.8, 20.6. HRMS calcd for $C_{33}H_{47}N_2$ $[M + H]^+$ 471.3734, found 471.3739.

(2*S*)-*N*^{1}-[(1'*S*)-2-(4-chlorophenyl)-1-phenylethyl]-*N*^{2}-(2,6-di(propan-2-yl)phenyl)-3,3-dimethylbutane-1,2-diamine [(2*S*,1'*S*)-20s] and (2*S*)-*N*^{1}-[(1'*R*)-2-(4-chlorophenyl)-1-phenylethyl]-*N*^{2}-(2,6-di(propan-2-yl)phenyl)-3,3-dimethylbutane-1,2-diamine [(2*S*,1'*R*)-20t]

By following the synthetic procedure described for (2*R*,1'*R*)-**20a** and (2*R*,1'*S*)-**20b**, the reaction was performed with 890 mg **21j** (3.86 mmol) and 1.07 g (*S*)-**19a** (3.86 mmol). The reaction time of the first step was 1 h and that of the second was 16 h. (2*S*,1'*S*)-**20s** and (2*S*,1'*R*)-**20t** were collected as a diastereomeric mixture (545 mg, 1.11 mmol, 28.9%). Diastereomers were separated by column chromatography using a mixture of heptane and DCM as eluents.

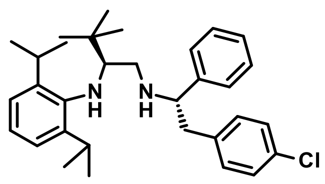

(2*S*,1'*S*)-**20s** was obtained as a single diastereomer, eluted first, and collected as yellow oil (255 mg, 0.519 mmol, 13.5%). ^{1}H NMR (500 MHz, DMSO-d_6) δ 7.23-7.10 (m, Ar-H, 5 H), 6.99 (m, Ar-H, 2 H), 6.99 (m, Ar-H, 2 H), 6.93 (dd, *J* = 8.1, 6.7 Hz, Ar-H, 1 H), 6.87 (dm, *J* = 8.4 Hz, Ar-H, 2 H), 3.41 (dd, *J* = 7.7, 6.4 Hz, CH, 1 H), 3.21 (sp, *J* = 6.7 Hz, CH, 2 H), 3.11 (d, *J* = 12.4 Hz, NH, 1 H), 2.83 (m, CH, 1 H), 2.60/2.56 (dd+dd, *J* = 13.5, 6.4 Hz, *J* = 13.5, 7.7 Hz, CH_2, 2 H), 2.45/2.17 (dm+dd, *J* = 12.4 Hz, *J* = 12.4, 5.0 Hz, CH_2, 2 H), 1.37 (brs, NH, 1 H), 1.13 (d, *J* = 6.7 Hz, CH_3, 6 H), 1.05 (d, *J* = 6.7 Hz, CH_3, 6 H), 0.85 (s, CH_3, 9 H). ^{13}C NMR (125 MHz, DMSO-d_6) δ 143.7, 142.0, 140.4, 137.6, 130.9, 130.6, 127.9, 127.9, 127.0, 126.6, 123.4, 122.2, 67.3, 63.9, 48.0, 43.6, 35.1, 27.0, 26.9, 24.0, 23.8. HRMS calcd for $C_{32}H_{44}ClN_2$ $[M + H]^+$ 491.3187, found 491.3191.

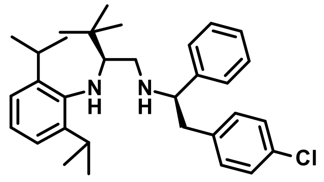

(2*S*,1'*R*)-**20t** was obtained as a single diastereomer, eluted second, and collected as yellow oil (281 mg, 0.572 mmol, 14.8%). ^{1}H NMR (500 MHz, DMSO-d_6) δ 7.19 (dm, *J* = 8.4 Hz, Ar-H, 2 H), 7.16-6.92 (m, Ar-H, 5 H), 7.00 (d, *J* = 7.0 Hz, Ar-H, 2 H), 6.91 (m, Ar-H, 1 H), 6.89 (d, *J* = 8.4 Hz, Ar-H, 2 H), 3.54 (m, CH, 1 H), 3.25 (sp, *J* = 6.8 Hz, CH, 2 H), 3.22 (d, *J* = 11.5 Hz, NH, 1 H), 2.92 (m, CH, 1 H), 2.63/2.58 (dd+dd, *J* = 13.2, 7.0 Hz, *J* = 13.2, 7.0 Hz, CH_2, 2 H), 2.35 (m, CH_2, 2 H), 1.36 (brs, NH, 1 H), 1.14 (d, *J* = 6.7 Hz, CH_3, 6 H), 1.08 (d, *J* = 6.7 Hz, CH_3, 6 H), 0.89 (s, CH_3, 9 H). ^{13}C NMR (125 MHz, DMSO-d_6) δ 143.1, 142.5, 140.0, 137.8, 131.0, 130.5, 127.9, 127.7, 126.9, 126.6, 123.4, 121.9, 66.3, 63.7, 48.3, 43.3, 35.1, 27.1, 27, 24.0, 23.8. HRMS calcd for $C_{32}H_{44}ClN_2$ $[M + H]^+$ 491.3187, found 491.3190.

N^1-[(1'*S*)-2-(4-chlorophenyl)-1-phenylethyl]-N^2-(2,6-di(propan-2-yl)phenyl)ethane-1,2-diamine [(1'*S*)-20w] and N^1-[(1'*R*)-2-(4-chlorophenyl)-1-phenylethyl]-N^2-(2,6-di(propan-2-yl)phenyl)ethane-1,2-diamine [(1'*R*)-20x]

By following the synthetic procedure described for (2*R*,1'*R*)-**20a** and (2*R*,1'*S*)-**20b**, the reaction was performed with 1.57 g **21j** (6.81 mmol) and 1.50 g **19c** (6.81 mmol). The reaction time of the first step was 6 h and that of the second was 16 h. (*S*)-**20w** and (*R*)-**20x** were collected as an enantiomeric mixture (1.14 g, 3.32 mmol, 48.8%). The enantiomers were separated by chiral chromatography on IG (100×500 mm, 20 μm, flow rate: 50 mL/min) column.

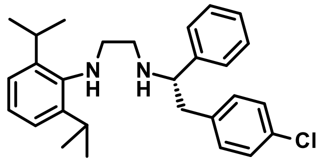

By chiral HPLC analysis, (*S*)-**20w** was found to be a single enantiomer, and eluted first on the IG column using 2:98 THF/heptane with 0.1% diethylamine as eluent. (*S*)-**20w** was collected as yellow oil (669 mg, 1.54 mmol, 22.6%). ^{1}H NMR (500 MHz, DMSO-d_6) δ 7.27 (m, Ar-H, 2 H), 7.27 (m, Ar-H, 2 H), 7.25 (d, J = 8.4 Hz, Ar-H, 2 H), 7.20 (m, Ar-H, 1 H), 7.10 (d, J = 8.4 Hz, Ar-H, 2 H), 6.99 (d, J = 7.6 Hz, Ar-H, 2 H), 6.92 (dd, J = 8.3, 6.8 Hz, Ar-H, 1 H), 3.82 (t, J = 7.0 Hz, CH, 1 H), 3.67 (brs, NH, 1 H), 3.21 (sp, J = 6.7 Hz, 2 H), 2.97/2.83 (dd+dd, J = 13.4, 7.0 Hz, J = 13.4, 7.0 Hz, CH_2, 2 H), 2.74 (m, CH_2, 2 H), 2.49 (m, CH_2, 2 H), 1.10 (d, J = 6.7 Hz, CH_3, 12 H). ^{13}C NMR (125 MHz, DMSO-d_6) δ 141.9, 131.4, 131.1, 128.1, 127.9, 127.8, 127.7, 127.2, 126.0, 123.1, 123.0, 63.8, 51.1, 47.0, 44.8, 26.8, 24.3. HRMS calcd for $C_{28}H_{36}ClN_2$ $[M + H]^+$ 435.2562, found 435.2565.

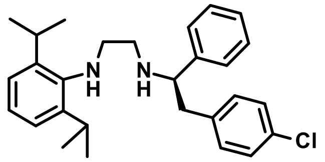

By chiral HPLC analysis, (*R*)-**20x** was found to be a single enantiomer, and eluted second on the IG column using 2:98 THF/heptane with 0.1% diethylamine as eluent. (*R*)-**20x** was collected as yellow oil (713 mg, 1.64 mmol, 24.1%). ^{1}H NMR (500 MHz, DMSO-d_6) δ 7.27 (m, Ar-H, 2 H), 7.27 (m, Ar-H, 2 H), 7.25 (d, J = 8.4 Hz, Ar-H, 2 H), 7.20 (m, Ar-H, 1 H), 7.10 (d, J = 8.4 Hz, Ar-H, 2 H), 6.99 (d, J = 7.6 Hz, Ar-H, 2 H), 6.92 (dd, J = 8.3, 6.8 Hz, Ar-H, 1 H), 3.82 (t, J = 7.0 Hz, CH, 1 H), 3.67 (brs, NH, 1 H), 3.21 (sp, J = 6.7 Hz, CH, 2 H), 2.97/2.83 (dd+dd, J = 13.4, 7.0 Hz, J = 13.4, 7.0 Hz, CH_2, 2 H), 2.74 (m, CH_2, 2 H), 2.49 (m, CH_2, 2 H), 1.10 (d, J = 6.7 Hz, CH_3, 12 H). ^{13}C NMR (125 MHz, DMSO-d_6) δ 141.9, 131.4, 131.1, 128.1, 127.9, 127.8, 127.7, 127.2, 126.0, 123.1, 123.0, 63.8, 51.1, 47.0, 44.8, 26.8, 24.3. HRMS calcd for $C_{28}H_{36}ClN_2$ $[M + H]^+$ 435.2562, found 435.2567.

Method B (20u-v,y-z)

Example for compound **20u:**

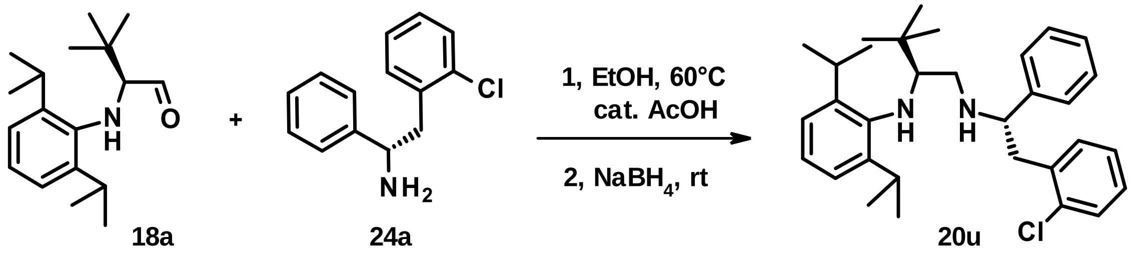

1.08 g of (*S*)-**24a** (4.66 mmol) was dissolved in 23 mL of EtOH under inert atmosphere, and then 662 mg of (*S*)-**18a** (4.66 mmol) was added in one portion and 27 μL of acetic acid (0.47 mmol) was also added. The mixture was stirred at 60°C. The mixture in the first reaction step was stirred for 2 h, until all amount of the (*S*)-**18a** had reacted, as monitoredd by GC-MS. When the starting material was consumed, the mixture was cooled to room temperature and 705 mg

$NaBH_4$ (18.6 mmol) was added carefully. The mixture was stirred for 16 h at room temperature and monitored by LC-MS. The reaction mixture was quenched with the addition of saturated aqueous NH_4Cl solution, and then it was extracted with EtOAc. The combined organic layers were dried over $MgSO_4$. The volatiles were removed under reduced pressure, and the residue was purified *via* column chromatography using a mixture of heptane and EtOAc as eluents.

(2*S*)-*N*^1-[(1'*S*)-2-(2-chlorophenyl)-1-phenylethyl]-*N*^2-(2,6-di(propan-2-yl)phenyl)-3,3-dimethylbutane-1,2-diamine [(2*S*,1'*S*)-20u]

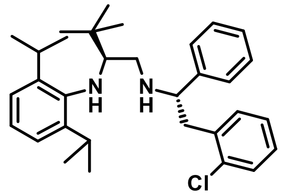

(2*S*,1'*S*)-**20u** was collected as yellow oil (2.02 g, 4.11 mmol, 88.2%). ^{1}H NMR (500 MHz, DMSO-*d*$_6$) δ 7.35-6.88 (m, Ar-H, 4 H), 7.22-6.93 (m, Ar-H, 5 H), 6.97 (m, Ar-H, 2 H), 6.91 (m, Ar-H, 1 H), 3.51 (m, CH, 1 H), 3.20 (sp, *J* = 6.7 Hz, CH, 2 H), 3.11 (d, *J* = 11.6 Hz, NH, 1 H), 2.82 (dt, *J* = 11.6, 4.8 Hz, CH, 1 H), 2.75/2.70 (dd+dd, *J* = 13.4, 8.0 Hz, *J* = 13.4, 5.8 Hz, CH_2, 2 H), 2.46/2.17 (m+m, CH_2, 2 H), 1.52 (brs, NH, 1 H), 1.12 (d, *J* = 6.7 Hz, CH_3, 6 H), 1.02 (d, *J* = 6.7 Hz, CH_3, 6 H), 0.84 (s, CH_3, 9 H). ^{13}C NMR (125 MHz, DMSO-*d*$_6$) δ 143.9, 142.0, 140.4, 136.3, 133.2, 131.7, 129.0, 128.0, 126.8, 126.7, 126.6, 123.4, 122.2, 67.1, 62.1, 47.5, 41.9, 27.0, 24.0, 23.8. $C_{32}H_{44}ClN_2$ $[M + H]^+$ 491.3187, found 491.3190.

(2*S*)-*N*^1-[(1'*R*)-2-(2-chlorophenyl)-1-phenylethyl]-*N*^2-(2,6-di(propan-2-yl)phenyl)-3,3-dimethylbutane-1,2-diamine [(2*S*,1'*R*)-20v]

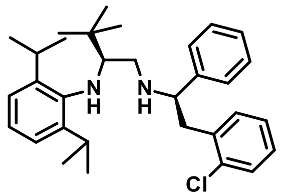

This compound was prepared by following the synthetic procedure described for (2*S*,1'*S*)-**20u** starting from 1.07 g of (*R*)-**24b** (4.62 mmol) and 1.27 g of (*S*)-**18a** (4.62 mmol). The reaction time of the first step was 2 h and thst of the second was 16 h. (2*S*,1'*R*)-**20v** was collected as yellow oil (941 mg, 1.92 mmol, 41.5%). ^{1}H NMR (500 MHz, DMSO-*d*$_6$) δ 7.36-6.86 (m, Ar-H, 12 H), 3.67 (q, *J* = 6.3 Hz, CH, 1 H), 3.21 (sp, *J* = 6.7 Hz, CH, 2 H), 3.19 (d, *J* = 11.4 Hz, NH, 1 H), 2.89 (dt, *J* = 11.4, 4.8 Hz, CH, 1 H), 2.84-2.77 (m, CH_2, 2 H), 2.34 (dd, *J* = 6.9, 4.8 Hz, CH_2, 2 H), 1.43 (brs, NH, 1 H), 1.12 (d, *J* = 6.7 Hz, CH_3, 6 H), 1.04 (d, *J* = 6.7 Hz, CH_3, 6 H), 0.88 (s, CH_3, 9 H). ^{13}C NMR (125 MHz, DMSO-*d*$_6$) δ 143.4, 142.3, 140.1, 136.5, 133.2, 131.9, 129.0, 128.0, 127.9, 126.6, 126.6, 123.3, 121.9, 66.9, 62.6, 48.3, 41.5, 35.1, 27.0, 27.0, 24.0, 23.7. $C_{32}H_{44}ClN_2$ $[M + H]^+$ 491.3187, found 491.3189.

(2*S*)-*N*^2-(2,6-di(propan-2-yl)phenyl)-3,3-dimethyl-*N*^1-[(1'*S*)-1-(2-naphthyl)ethyl]butane-1,2-diamine [(2*S*,1'*S*)-20y]

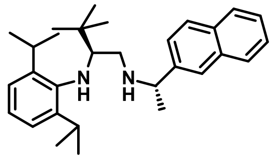

By following the synthetic procedure described for (2*S*,1'*S*)-**20u**, the reaction was performed with 855 mg of (1*S*)-1-(2-naphthyl)ethanamine (4.99 mmol) and 1.25 g of (*S*)-**18a** (4.54 mmol). The reaction time of the first step was 1 h and that of the second step was 4 h. (2*S*,1'*S*)-**20y** was collected as yellow oil (939 mg, 2.18 mmol, 48.0%).^{1}H NMR (500 MHz, DMSO-d$_6$) δ 7.87-7.38 (m, Ar-H, 4 H), 7.79 (d, *J* = 8.4 Hz, Ar-H, 1 H), 7.44 (br, Ar-H, 1 H), 7.34 (dd, *J* = 8.4 Hz, *J* = 1.3 Hz, Ar-H, 1 H), 7.04 (d, *J* = 7.6 Hz, Ar-H, 2 H), 6.96 (t, *J* = 7.6 Hz, Ar-H, 1 H), 3.49 (q, *J* = 6.7 Hz, CH, 1 H), 3.38-3.29 (sp, *J* = 6.7 Hz, CH, 2 H), 3.25 (d, *J* =12.2 Hz, NH, 1 H), 3.00 (m, CH, 1 H), 2.51/2.37 (m+m, CH_2, 2 H), 1.34 (brs, NH, 1 H), 1.18 (d, *J* = 6.7 Hz, CH_3, 6 H), 1.10 (d, *J* = 6.7 Hz, CH_3, 3 H), 1.09 (d, *J* = 6.7 Hz, CH_3, 3 H), 1.05 (d, *J* = 6.7 Hz, CH_3, 3 H), 0.94 (s, CH_3, 9 H). ^{13}C NMR (125 MHz, DMSO-d$_6$) δ 143.6, 142.4, 140.5, 132.9, 132.2, 127.7, 127.5, 127.4,

125.8, 125.3, 124.8, 124.6, 123.5, 122.2, 67.5, 57.7, 48.8, 35.2, 27.1, 27, 24.1, 23.9. HRMS calcd for $C_{30}H_{43}N_2$ [M+H]$^+$ 431.3421, found 431.3419.

(2*S*)-*N*^{2}-(2,6-di(propan-2-yl)phenyl)-3,3-dimethyl-*N*^{1}-[(1'*R*)-1-(2-naphthyl)ethyl]butane-1,2-diamine [(2*S*,1'*R*)-20z]

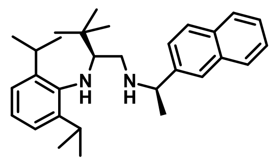

By following the synthetic procedure described for (2*S*,1'*S*)-**20u**, the reaction was performed with 1.57 g of (*R*)-1-(2-naphthyl)ethanamine (9.15 mmol) and 2.29 g of (*S*)-**18a** (8.32 mmol). The reaction time of the first step was 1 h and that of the second was 4 h. (2*S*,1'*R*)-**20z** was collected as yellow oil (2.71 g, 6.30 mmol, 75.8%).^{1}H NMR (400 MHz, DMSO-d$_6$) δ 7.84-7.74 (m, Ar-H, 2 H), 7.76 (d, J = 8.5 Hz, Ar-H, 1 H), 7.57 (brs, Ar-H, 1 H), 7.44 (m, Ar-H, 2 H), 7.31 (dd, J = 8.5, 1.7 Hz, Ar-H, 1 H), 6.98 (d, J = 7.6 Hz, Ar-H, 2 H), 6.86 (t, J = 7.6 Hz, Ar-H, 1 H), 3.63 (q, J = 6.7 Hz, CH, 1 H), 3.37 (sp, J = 6.8 Hz, CH, 2 H), 3.05 (m, CH, 1 H), 2.48/2.40 (m+dd, J = 12.8, 6.0 Hz, CH_2, 2 H), 1.24 (br, NH, 1 H), 1.17 (d, J = 6.8 Hz, CH_3, 6 H), 1.11 (d, J = 6.8 Hz, CH_3, 6 H), 1.11 (d, J = 6.7 Hz, CH_3, 3 H), 0.95 (s, CH_3, 9 H). ^{13}C NMR (100 MHz, DMSO-d$_6$) δ 143.5, 142.6, 139.9, 132.9, 132.2, 127.8, 127.5, 127.4, 125.8, 125.3, 124.7, 124.7, 123.4, 121.8, 66.0, 57.3, 48.5, 35.2, 27.2, 27.0, 24.0, 23.9. HRMS calcd for $C_{30}H_{43}N_2$ [M + H]$^+$ 431.3421, found 431.3420.

1.5 General Procedure for Preparation of Ketone Derivatives (21a-k)

Example for Compound **21a:**

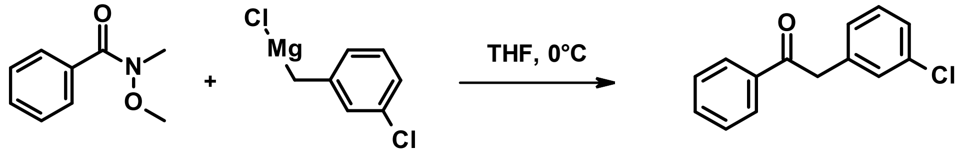

"Weinreb amide" "Grignard reagent" 21a

A solution of 7.60 mL *N*-methoxy-*N*-methyl-benzamide (50.0 mmol) in 200 mL THF (5 mL/mmol) was cooled to 0 °C under inert atmosphere, and then 200 mL chloro-[(3-chlorophenyl)methyl]magnesium (50.0 mmol) was added dropwise over 10 min. The resulting mixture was stirred at 0 °C for 30 minutes when full conversion was observed by GC-MS. The reaction mixture was quenched by the addition of saturated aqueous NH_4Cl solution, then it was allowed to warm up to room temperature. Then it was extracted with EtOAc. The combined organic layers were dried over $MgSO_4$ and the volatiles were removed under reduced pressure. The crude product was purified by column chromatography using a mixture of heptane and EtOAc as eluents.

2-(3-chlorophenyl)-1-phenyl-ethanone (21a)

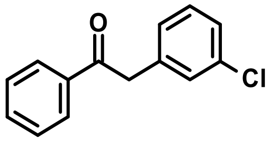

21a was collected as light yellow oil (7.46 g, 32.3 mmol, 65.0%). ^{1}H NMR (500 MHz, DMSO-*d$_6$*) δ 8.05 (m, Ar-H, 2 H), 7.66 (m, Ar-H, 1 H), 7.55 (m, Ar-H, 2 H), 7.36 (m, Ar-H, 1 H), 7.34 (m, Ar-H, 1 H), 7.31 (dm, J = 7.6 Hz, Ar-H, 1 H), 7.23 (dm, J = 7.6 Hz, Ar-H, 1 H), 4.46 (s, CH_2, 2 H). ^{13}C NMR (125 MHz, DMSO-*d$_6$*) δ 197.2, 137.7, 136.3, 133.4, 132.8, 130.0, 129.8, 128.8, 128.7, 128.3, 126.5, 44.1. HRMS calcd for $C_{14}H_{11}ClO$ [M]$^+$ 230.0498, found 230.0493.

2-phenyl-1-[3-(trifluoromethyl)phenyl]ethanone (21b)

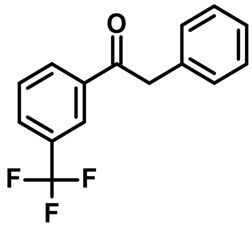

By following the synthetic procedure described for **21a**, the reaction was performed with 3.08 mL *N*-methoxy-*N*-methyl-3-(trifluoromethyl)benzamide (17.2 mmol) in 85 mL THF and 12.9 mL benzyl(chloro)magnesium (25.7 mmol). **21b** was collected as yellow oil (3.37 g, 12.8 mmol, 74.3%). ^{1}H NMR (500 MHz, DMSO-d_6) δ 8.35 (d, J = 7.8 Hz, Ar-H, 1 H), 8.29 (brs, Ar-H, 1 H), 8.02 (d, J = 7.8 Hz, Ar-H, 1 H), 7.79 (t, J = 7.8 Hz, Ar-H, 1 H), 7.38-7.19 (m, Ar-H, 5 H), 4.50 (s, CH_2, 2 H). ^{13}C NMR (125 MHz, DMSO-d_6) δ 196.8, 137.1, 134.7, 132.4, 130.2, 129.8, 128.3, 126.6, 44.8. HRMS calcd for $C_{15}H_{11}F_3O$ $[M]^+$ 264.0762, found 264.0748.

1-(2-methoxyphenyl)-2-phenyl-ethanone (21c)

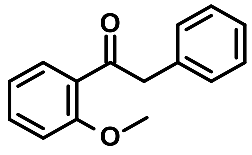

By following the synthetic procedure described for **21a**, the reaction was performed with 0.94 mL *N*,2-dimethoxy-*N*-methyl-benzamide (5.12 mmol) in 25 mL THF and 3.84 mL benzyl(chloro)magnesium (7.68 mmol). **21c** was collected as colorless oil (949 mg, 4.19 mmol, 81.9%). ^{1}H NMR (500 MHz, DMSO-d_6) δ 7.53 (dm, J = 7.7 Hz, Ar-H, 1 H), 7.52 (td, J = 7.7, 1.2 Hz, Ar-H, 1 H), 7.34-7.15 (m, Ar-H, 5 H), 7.16 (dm, J = 7.7 Hz, Ar-H, 1 H), 7.01 (td, J = 7.7, 1.2 Hz, Ar-H, 1 H), 4.25 (s, CH_2, 2 H), 3.90 (s, CH_3, 3 H). ^{13}C NMR (125 MHz, DMSO-d_6) δ 199.7, 158.0, 135.3, 133.7, 129.7, 129.7, 128.2, 127.9, 126.4, 120.5, 112.4, 55.7, 49.4. HRMS calcd for $C_{15}H_{15}O_2$ $[M + H]^+$ 227.1067, found 227.1067.

1-(3-methoxyphenyl)-2-phenyl-ethanone (21d)

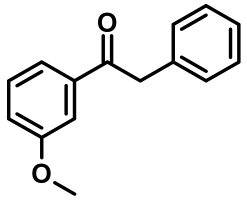

By following the synthetic procedure described for **21a**, the reaction was performed with 2.50 mg *N*,3-dimethoxy-*N*-methyl-benzamide (12.8 mmol) in 25 mL THF and 27.4 mL benzyl(chloro)magnesium (38.4 mmol). **21d** was collected as yellow oil (2.73 g, 12.1 mmol, 94.1%).^{1}H NMR (400 MHz, DMSO-d_6) δ 7.66 (dm, J = 7.9 Hz, Ar-H, 1 H), 7.52 (dd, J = 2.6, 1.5 Hz, Ar-H, 1 H), 7.45 (t, J = 7.9 Hz, Ar-H, 1 H), 7.36-7.17 (m, Ar-H, 5 H), 7.21 (dm, J = 7.9 Hz, Ar-H, 1 H), 4.38 (s, CH_2, 2 H), 3.81 (s, CH_3, 3 H). ^{13}C NMR (100 MHz, DMSO-d_6) δ 197.5, 159.4, 137.8, 135.1, 129.9, 129.7, 128.3, 126.5, 120.9, 119.2, 112.9, 55.3, 44.9. HRMS calcd for $C_{15}H_{15}O_2$ $[M + H]^+$ 227.1067, found 227.1065.

1-(4-methoxyphenyl)-2-phenyl-ethanone (21e)

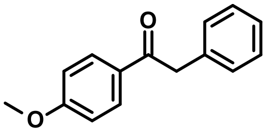

By following the synthetic procedure described for **21a**, the reaction was performed with 1.80 mL *N*,4-dimethoxy-*N*-methyl-benzamide (10.2 mmol) in 51 mL THF and 7.68 mL benzyl(chloro)magnesium (15.4 mmol). **21e** was collected as white solid (2.31 g, 10.2 mmol, 99.8%). Mp: 115-117°C. ^{1}H NMR (500 MHz, DMSO-d_6) δ 8.03 (m, Ar-H, 2 H), 7.34-7.18 (m, Ar-H, 5 H), 7.04 (m, Ar-H, 2 H), 4.31 (s, CH_2, 2 H), 3.84 (s, CH_3, 3 H). ^{13}C NMR (125 MHz, DMSO-d_6) δ 196.1, 163.2, 135.5, 130.8, 129.6, 129.2, 128.3, 126.4, 114.0, 55.6, 44.4. HRMS calcd for $C_{15}H_{15}O_2$ $[M + H]^+$ 227.1067, found 227.1067.

1-(o-tolyl)-2-phenyl-ethanone (21f)

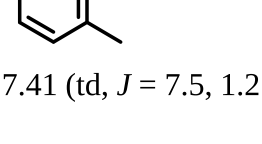

By following the synthetic procedure described for **21a**, the reaction was performed with 0.94 mL *N*-methoxy-*N*,2-dimethyl-benzamide (55.8 mmol) in 28 mL THF and 4.18 mL benzyl(chloro)magnesium (8.37 mmol). **21f** was collected as yellow oil (863 mg, 4.10 mmol, 73.5%). ^{1}H NMR (500 MHz, DMSO-d_6) δ 7.90 (dd, J = 7.5, 1.2 Hz, Ar-H, 1 H), 7.41 (td, J = 7.5, 1.2 Hz, Ar-H, 1 H), 7.32 (t, J = 7.5 Hz, Ar-H, 1 H), 7.30 (m, Ar-H, 2 H), 7.27

(d, J = 7.5 Hz, Ar-H, 1 H), 7.23 (dm, J = 7.2 Hz, Ar-H, 2 H), 7.22 (m, Ar-H, 1 H), 4.29 (s, CH_2, 2 H), 2.33 (s, CH_3, 3 H). ^{13}C NMR (125 MHz, DMSO-d_6) δ 201.6, 137.7, 137.1, 135.0, 131.6, 131.2, 129.7, 128.8, 128.3, 126.5, 125.8, 47.8, 20.6. HRMS calcd for $C_{15}H_{14}O$ $[M]^+$ 210.1045, found 210.1019.

2-phenyl-1-(p-tolyl)ethanone (21g)

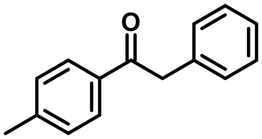

By following the synthetic procedure described for **21a**, the reaction was performed with 0.94 mL *N*-methoxy-*N*,4-dimethyl-benzamide (5.58 mmol) in 28 mL THF and 4.18 mL benzyl(chloro)magnesium (8.37 mmol). **21g** was collected as white solid (956 mg, 4.55 mmol, 81.5%). mp 110-112 °C. ^{1}H NMR (500 MHz, DMSO-d_6) δ 7.95 (d, J = 8.1 Hz, Ar-H, 2 H), 7.33 (d, J = 8.1 Hz, Ar-H, 2 H), 7.30 (t, J = 7.2 Hz, Ar-H, 2 H), 7.25 (d, J = 7.2 Hz, Ar-H, 2 H), 7.22 (t, J = 7.2 Hz, Ar-H, 1 H), 4.34 (s, CH_2, 2 H), 2.37 (s, CH_3, 3 H). ^{13}C NMR (125 MHz, DMSO-d_6) δ 197.2, 143.7, 135.3, 133.9, 129.7, 129.3, 128.6, 128.3, 126.4, 44.6, 21.2. HRMS calcd for $C_{15}H_{15}O$ $[M + H]^+$ 211.1117, found 211.1118.

2-phenyl-1-[4-(trifluoromethyl)phenyl]ethanone (21h)

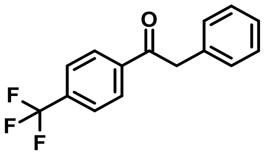

By following the synthetic procedure described for **21a**, the reaction was performed with 2.31 mL N-methoxy-N-methyl-4-(trifluoromethyl)benzamide (12.9 mmol) in 65 mL THF and 9.65 mL benzyl(chloro)magnesium (19.3 mmol). **21h** was collected as white solid (2.63 g, 9.95 mmol, 77.4%). mp 127-129 °C. ^{1}H NMR (500 MHz, DMSO-d_6) δ 8.23 (dm, J = 8.2 Hz, Ar-H, 2 H), 7.91 (dm, J = 8.2 Hz, Ar-H, 2 H), 7.37-7.20 (m, Ar-H, 5 H), 4.47 (s, CH_2, 2 H). ^{13}C NMR (125 MHz, DMSO-d_6) δ 197.2, 139.6, 134.6, 132.7, 132.4, 129.8, 129.2, 128.4, 126.6, 125.8, 125.8, 45.0. HRMS calcd for $C_{15}H_{12}F_3O$ $[M + H]^+$ 265.0835, found 265.0835.

1-phenyl-2-(p-tolyl)ethanone (21i)

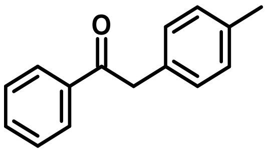

By following the synthetic procedure described for **21a**, the reaction was performed with 0.92 mL *N*-methoxy-*N*-methyl-benzamide (6.05 mmol) in 30 mL THF and 20 mL chloro(p-tolylmethyl)magnesium (9.10 mmol). **21i** was collected as white solid (974 mg, 4.63 mmol, 76.5%). mp 94-96 °C. ^{1}H NMR (500 MHz, DMSO-d_6) δ 8.03 (dm, J = 7.2 Hz, Ar-H, 2 H), 7.63 (tm, J = 7.2 Hz, Ar-H, 1 H), 7.52 (tm, J = 7.2 Hz, Ar-H, 2 H), 7.15 (dm, J = 8.0 Hz, Ar-H, 2 H), 7.11 (dm, J = 8.0 Hz, Ar-H, 2 H), 4.32 (s, CH_2, 2 H), 2.26 (s, CH_3, 3 H). ^{13}C NMR (125 MHz, DMSO-d_6) δ 197.8, 136.3, 135.5, 132.0, 129.5, 128.9, 128.8, 128.4, 44.4, 20.7. HRMS calcd for $C_{15}H_{15}O$ $[M + H]^+$ 211.1117, found 211.1115.

2-(4-chlorophenyl)-1-phenyl-ethanone (21j)

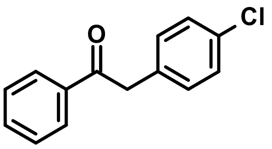

By following the synthetic procedure described for **21a**, the reaction was performed with 1.38 mL *N*-methoxy-*N*-methyl-benzamide (9.08 mmol) in 45 mL THF and 54 mL collected chloro-[(4-chlorophenyl)methyl]magnesium (13.6 mmol). **21j** was as white solid (902 mg, 3.91 mmol, 43.1%). mp 128-130 °C. ^{1}H NMR (400 MHz, DMSO-d_6) δ 8.05 (dm, J = 7.3 Hz, Ar-H, 2 H), 7.65 (tm, J = 7.3 Hz, Ar-H, 1 H), 7.54 (tm, J = 7.3 Hz, Ar-H, 2 H), 7.38 (dm, J = 8.5 Hz, Ar-H, 2 H), 7.29 (dm, J = 8.5 Hz, Ar-H, 2 H), 4.43 (s, CH_2, 2 H). ^{13}C NMR (100 MHz, DMSO-d_6) δ 197.4, 136.3, 134.2, 133.4, 131.7, 131.2, 128.8, 128.3, 128.2, 43.9. HRMS calcd for $C_{14}H_{12}ClO$ $[M + H]^+$ 231.0571, found 231.0559.

2-(2-chlorophenyl)-1-phenyl-ethanone (21k)

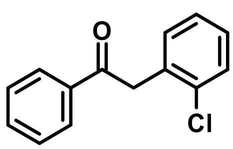

By following the synthetic procedure described for **21a**, the reaction was performed with 1.38 mL *N*-methoxy-*N*-methyl-benzamide (9.08 mmol) in 45 mL THF and 54 mL chloro-[(2-chlorophenyl)methyl]magnesium (13.6 mmol). **21k** was collected as white solid (1.82 g, 7.89 mmol, 86.8%). mp 71-73 °C. ^{1}H NMR (500 MHz, DMSO-d_6) δ 8.08 (dm, J = 7.6 Hz, Ar-H, 2 H), 7.68 (tm, J = 7.6 Hz, Ar-H, 1 H), 7.57 (tm, J = 7.6 Hz, Ar-H, 2 H), 7.50-7.28 (m, Ar-H, 4 H), 4.57 (s, CH_2, 2 H). ^{13}C NMR (125 MHz, DMSO-d_6) δ 196.2, 136.3, 133.9, 133.5, 132.6, 129.0, 128.9, 128.7, 128.1, 127.1, 43.1. HRMS calcd for $C_{14}H_{12}ClO$ [M + H]$^+$ 231.0571, found 231.0575.

1.6 Synthesis of of Substituted 1-phenylethanamine (22-24a,b)

2-(2-chlorophenyl)-1-phenyl-ethanol (22)

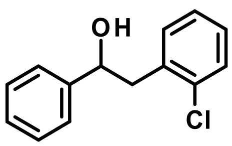

3.44 g $LiAlH_4$ (90.6 mmol) was dissolved in 300 mL THF at 0°C and 6.97 g **21k** (30.2 mmol) was added in small portions. The reaction mixture was stirred at room temperature for 60 min, when complete conversion was observed by LC-MS. The reaction mixture was cooled to 0°C and 1.5 mL water, 3 mL 2N NaOH and 3 mL water was added slowly to the mixture. MTBE was added to the mixture at the previous temperature and stirred for 10 min. The mixture was filtered, washed with MTBE. **22** was collected as white solid (6.47 g, 27.8 mmol, 92.0%). mp 71-73 °C. ^{1}H NMR (500 MHz, DMSO-d_6) δ 7.42-7.17 (m, Ar-H, 9 H), 5.36 (d, J = 4.8 Hz, OH, 1 H), 4.80 (m, CH, 1 H), 3.01-2.93 (m, CH_2, 2 H). ^{13}C NMR (125 MHz, DMSO-d_6) δ 145.6, 136.5, 133.3, 132.3, 129.0, 128.0, 128.0, 126.9, 126.7, 125.8, 71.9, 43.3. HRMS calcd for $C_{14}H_{12}Cl$ [M - OH]$^+$ 215.0622, found 215.0622.

***tert*-butyl N-*tert*-butoxycarbonyl-N-[2-(2-chlorophenyl)-1-phenyl-ethyl]carbamate (23)**

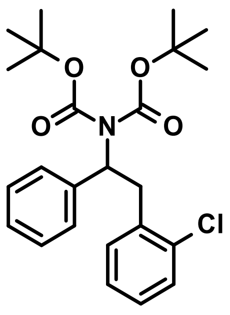

6.46 g **22** (27.8 mmol) was dissolved in 139 mL toluene under inert atmosphere, then 6.03 g di*tert*-butyl-iminodicarboxylate (27.8 mmol), 7.82 g triphenylphosphine (27.8 mmol) and 6.39 g di*tert*-butyl-azodicarboxylate (27.8 mmol) were added. The mixture was stirred at room temperature for 60 min, when a complete conversion was observed by HPLC-MS. The volatiles were removed under reduced pressure and the residue was purified by column chromatography using a mixture of heptane and EtOAc as eluent. **23** was collected as white solid (5.76 g, 13.3 mmol, 48.1%).mp 88-90 °C. ^{1}H NMR (500 MHz, DMSO-d_6) δ 7.46-7.18 (m, Ar-H, 9 H), 5.66 (dd, J = 11.4, 4.4 Hz, CH, 1 H), 3.63/3.53 (dd+dd, J = 13.3, 11.4 Hz, J = 13.3, 4.4 Hz, CH_2, 2 H), 1.18 (s, CH_3, 18 H). ^{13}C NMR (125 MHz, DMSO-d_6) δ 152.0, 140.2, 135.7, 133.7, 133.3, 133.2, 132.0, 129.2, 128.8, 128.8, 128.5, 128.3, 127.2, 127.1, 126.7, 81.5, 57.3, 34.4, 27.2. HRMS calcd for $C_{24}H_{30}ClO_4Na$ [M + Na]$^+$ 454.1756, found 454.1766.

(*S*)-2-(2-chlorophenyl)-1-phenyl-ethanamine [(*S*)-24a] and (*R*)-2-(2-chlorophenyl)-1-phenyl-ethanamine [(*R*)-24b]

5.76 g **23** (13.3 mmol) was dissolved in 67 mL DCM and 33.3 mL 2,2,2-triflouracetic acid (436 mmol) was added. The mixture was stirred at room temperature for 60 min, when a complete conversion was observed by HPLC-MS. The volatiles were removed under reduced pressure and pH was set to 7 with saturated aqueous $NaHCO_3$ solution. Then it was extracted with DCM. The combined organic layers were dried over $MgSO_4$ and the volatiles were removed under reduced pressure. The crude product was purified by column chromatography using DCM with

1.2% methanolic ammonia as an eluent. (*S*)-**24a** and (*R*)-**24b** were collected as a racemic mixture (2.11 g, 9.11 mmol, 68.3%). The enantiomers were separated by chiral preparative chromatography on OD-H (100×500mm, 20µm, Flow rate: 20 mL/min) column.

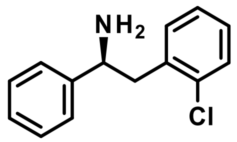

By chiral HPLC analysis, (*S*)-**24a** was found to be a single enantiomer and eluted first on the OD-H column using 1-PrOH/heptane (15:85) with 0.1% diethylamine as an eluent (t_R = 5.10 min). (*S*)-**24a** was collected as yellow oil (1052 mg, 4.54 mmol, 34.0%). ^{1}H NMR (500 MHz, DMSO-d_6) δ 7.42-7.14 (m, Ar-H, 9 H), 4.09 (dd, J = 7.9, 6.3 Hz, CH, 1 H), 2.96/2.91 (dd+dd, J = 13.3, 6.3 Hz, J = 13.3, 7.9 Hz, CH_2, 2 H), 1.98 (brs, NH, 2 H). ^{13}C NMR (125 MHz, DMSO-d_6) δ 146.6, 137.0, 132.1, 129.1, 128.0, 127.9, 126.8, 126.5, 126.3, 55.5, 43.6. HRMS calcd for $C_{14}H_{15}ClN$ $[M + H]^+$ 232.0886, found 232.0887.

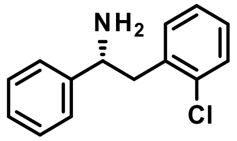

By chiral HPLC analysis, (*R*)-**24b** was obtained as a single enantiomer and eluted second on the OD-H column using 1-PrOH/heptane (15:85) with 0.1% diethylamine as an eluent (t_R = 8.48 min). (*R*)-**24b** was collected as yellow oil (1041 mg, 4.49 mmol, 33.7%). ^{1}H NMR (500 MHz, DMSO-d_6) δ 7.41-7.15 (m, Ar-H, 9 H), 4.09 (dd, J = 7.9, 6.2 Hz, CH, 1 H), 2.96/2.91 (dd+dd, J = 13.3, 6.2 Hz, J = 13.3, 7.9 Hz, CH_2, 2 H), 1.97 (brs, NH, 2 H). ^{13}C NMR (125 MHz, DMSO-d_6) δ 146.6, 137.0, 132.1, 129.1, 128.0, 127.9, 126.8, 126.5, 126.3, 55.5, 43.6. HRMS calcd for $C_{14}H_{15}ClN$ $[M + H]^+$ 232.0886, found 232.0887.

1.7 References for Known Compounds

1. Szabó, Z.; Timári, M.; Kassai, R.; Szokol, B.; Bényei, A. Cs.; Gáti, T.; Paczal, A.; Kotschy, A. *Organometallics* **2020**, *39*, 3572−3589.
2. Paczal, A.; Bényei, A. Cs.; Kotschy, A. *J. Org. Chem.* **2006**, *71*, 16, 5969–5979.

2 NMR Spectra (^{1}H and ^{13}C)

(5*S*,1'*S*)-5-*tert*-butyl-1-(2,6-di(propan-2-yl)phenyl)-3-[1-(2-naphthyl)ethyl]-4,5-dihydro-1*H*-imidazol-3-ium chloride [(5*S*,1'*S*)-3a]

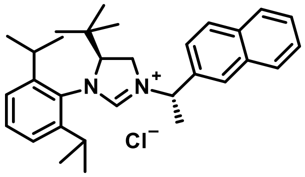

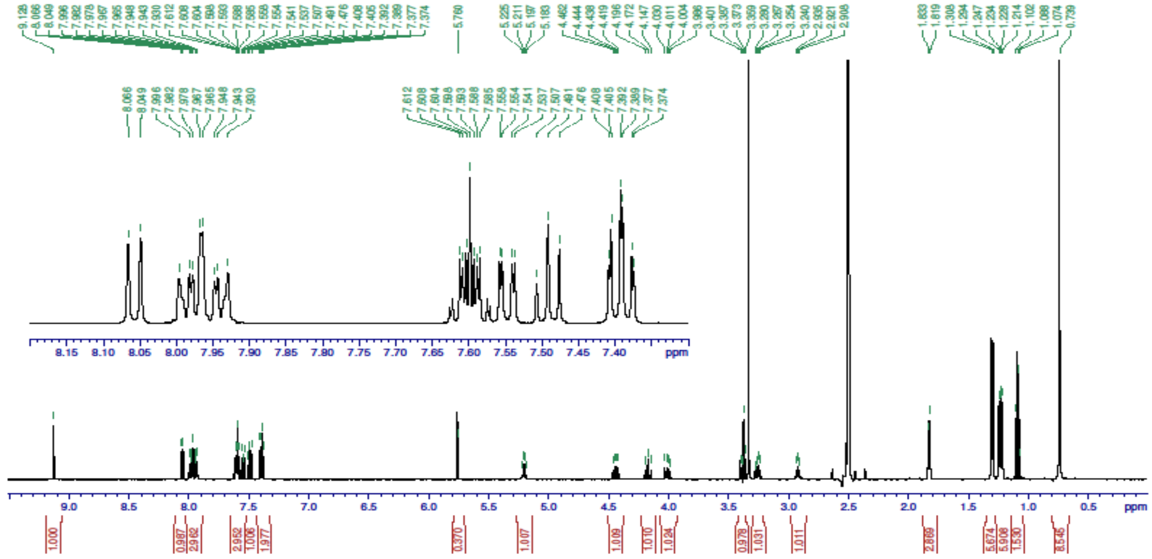

Figure S1. ^{1}H NMR (500 MHz) spectrum of **3a** in DMSO-d_6

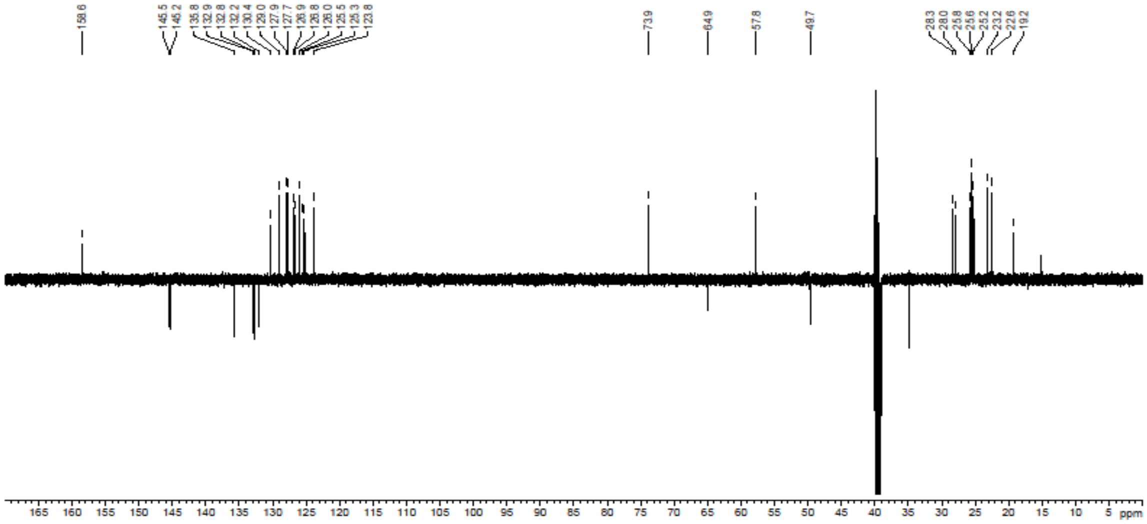

Figure S2. ^{13}C NMR (125 MHz) spectrum of 3a in DMSO-d_6

(5*S*,1’*R*)-5-*tert*-butyl-1-(2,6-di(propan-2-yl)phenyl)-3-[1-(2-naphthyl)ethyl]-4,5-dihydro-1*H*-imidazol-3-ium chloride [(5*S*,1’*R*)-3b]

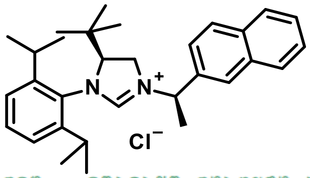

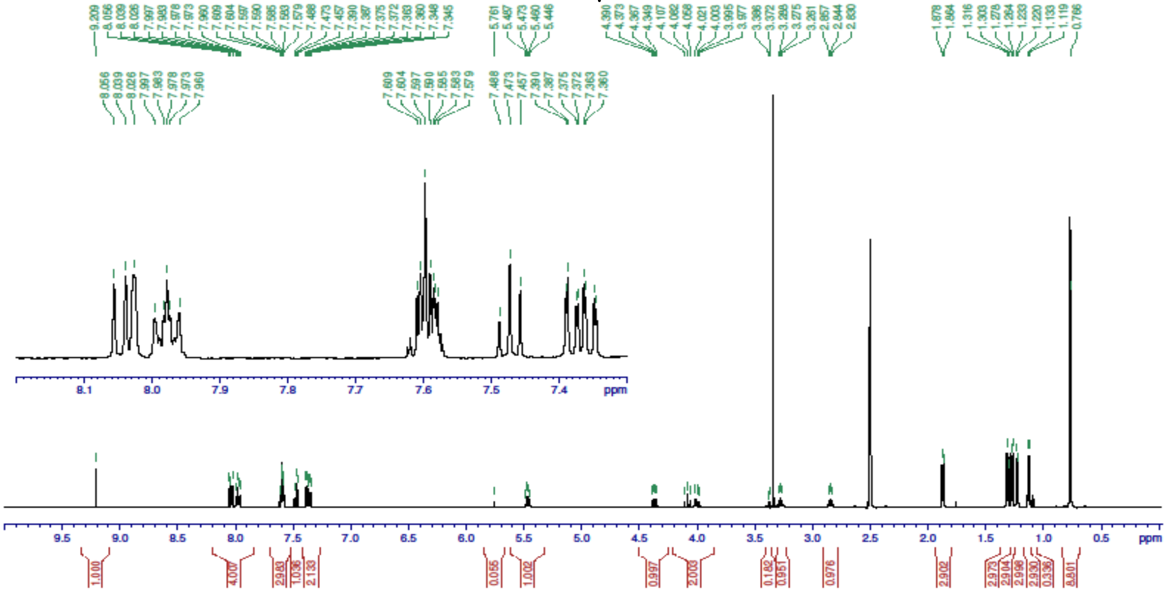

Figure S3. ^{1}H NMR (500 MHz) spectrum of 3b in DMSO-d_6

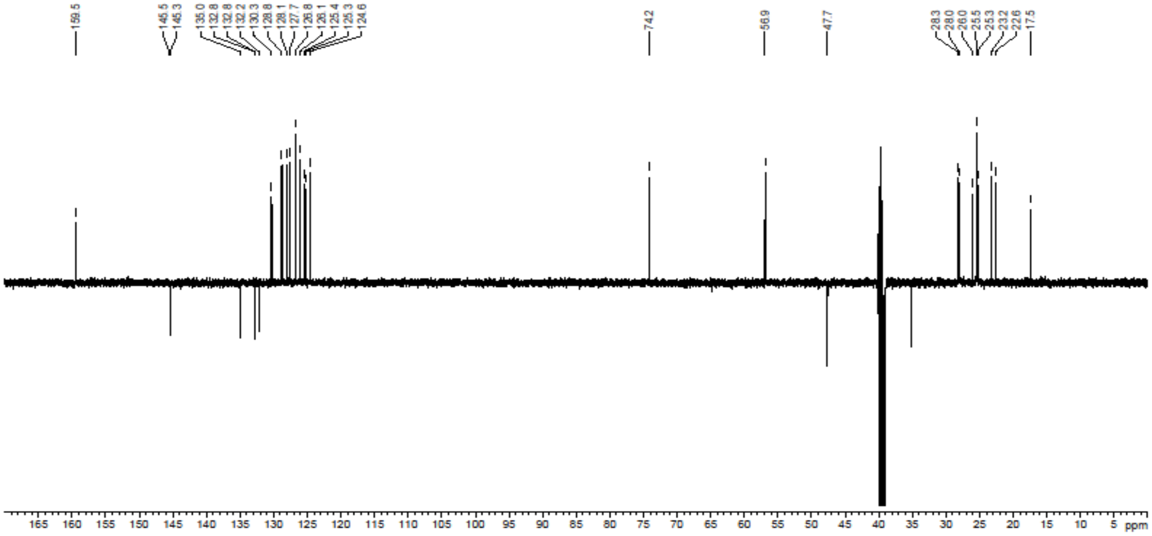

Figure S4. ^{13}C NMR (125 MHz) spectrum of 3b in DMSO-d_6

(5*R*,1'*R*)-5-*tert*-butyl-3-[2-(3-chlorophenyl)-1-phenylethyl]-1-(2,6-di(propan-2-yl)phenyl)-4,5-dihydro-1*H*-imidazol-3-ium chloride [(5*R*,1'*R*)-6a]

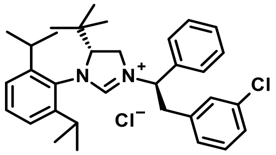

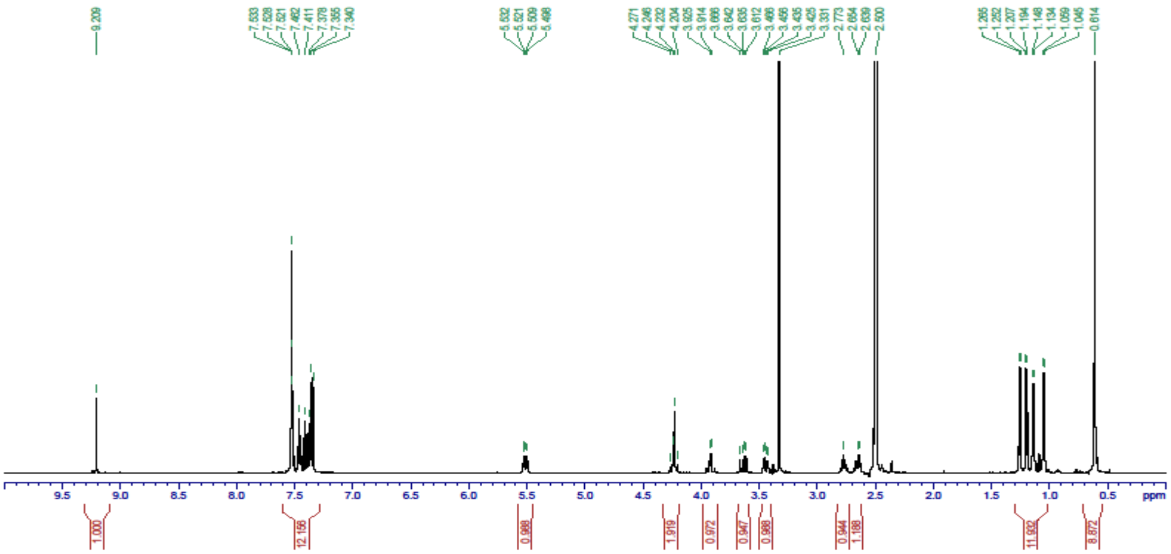

Figure S5. ^{1}H NMR (500 MHz) spectrum of **6a** in DMSO-d_6

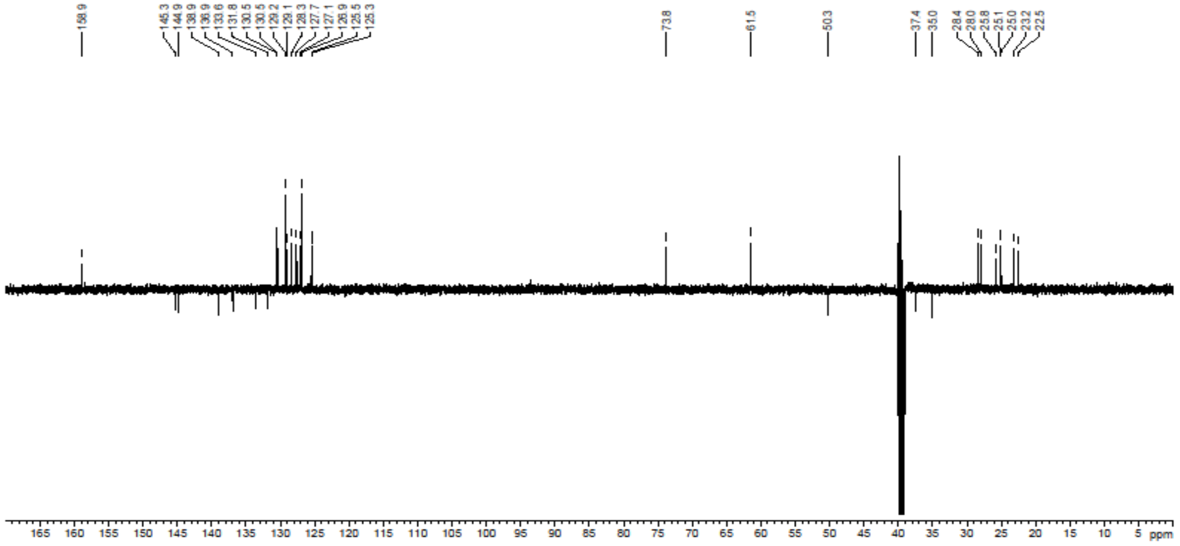

Figure S6. ^{13}C NMR (125 MHz) spectrum of **6a** in DMSO-d_6

(5*R*,1'*S*)-5-*tert*-butyl-3-[2-(3-chlorophenyl)-1-phenylethyl]-1-(2,6-di(propan-2-yl)phenyl)-4,5-dihydro-1*H*-imidazol-3-ium chloride [(5*R*,1'*S*)-6b]

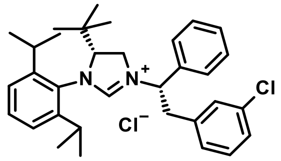

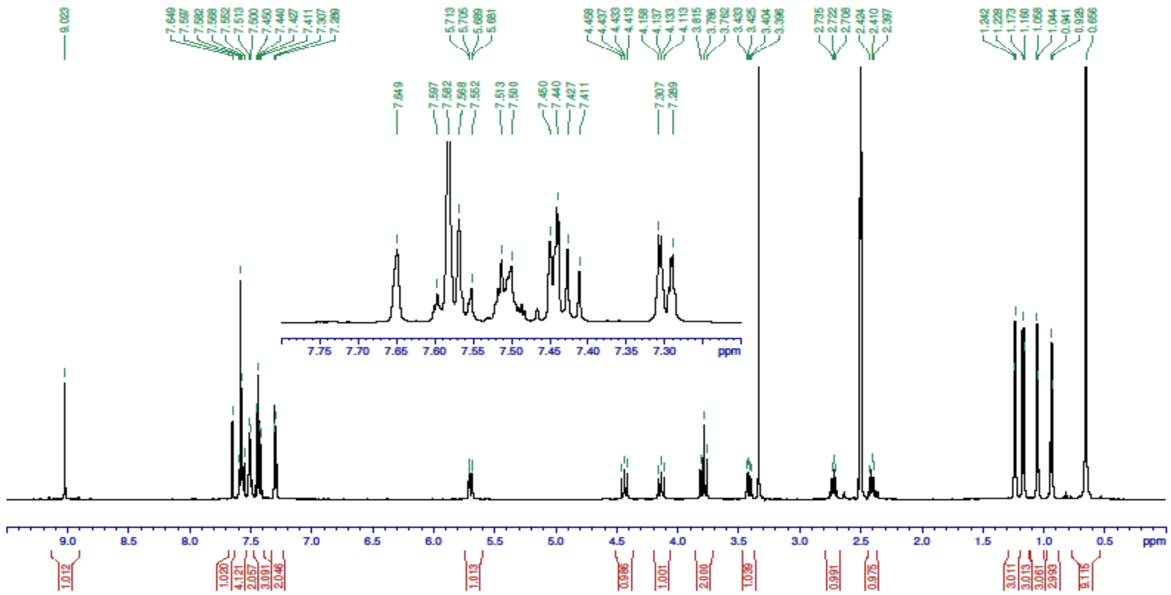

Figure S7. ^{1}H NMR (500 MHz) spectrum of **6b** in DMSO-d_6

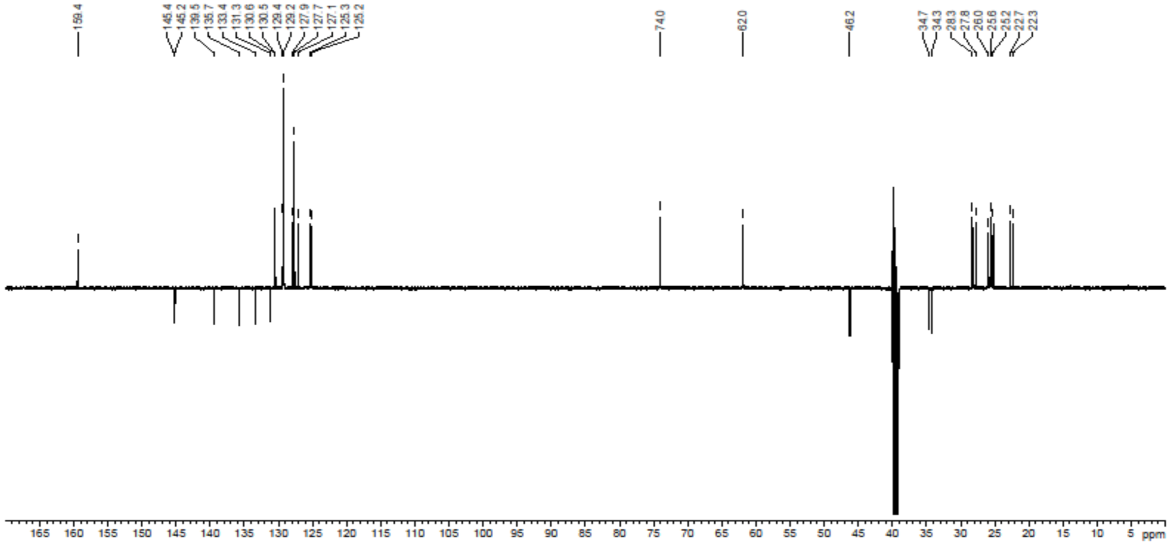

Figure S8. ^{13}C NMR (125 MHz) spectrum of **6b** in DMSO-d_6

(5*R*,1'*R*)-5-*tert*-butyl-1-(2,6-di(propan-2-yl)phenyl)-3-[2-phenyl-1-[3-(trifluoromethyl)phenyl]ethyl]-4,5-dihydro-1*H*-imidazol-3-ium chloride [(5*R*,1'*R*)-7a]

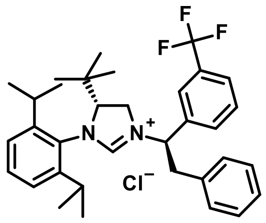

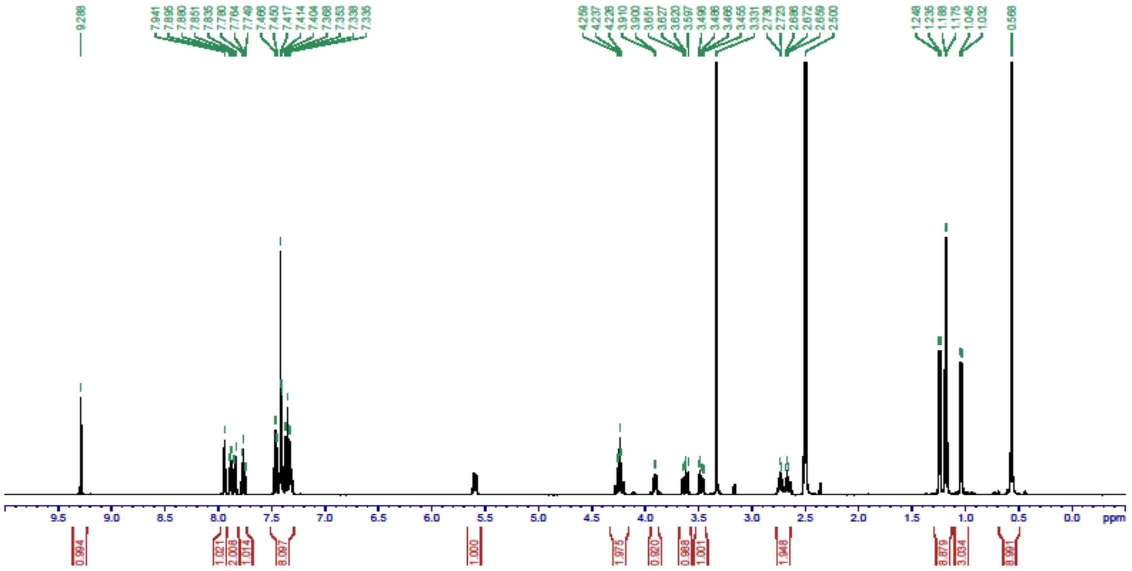

Figure S9. ^{1}H NMR (500 MHz) spectrum of **7a** in DMSO-d_6

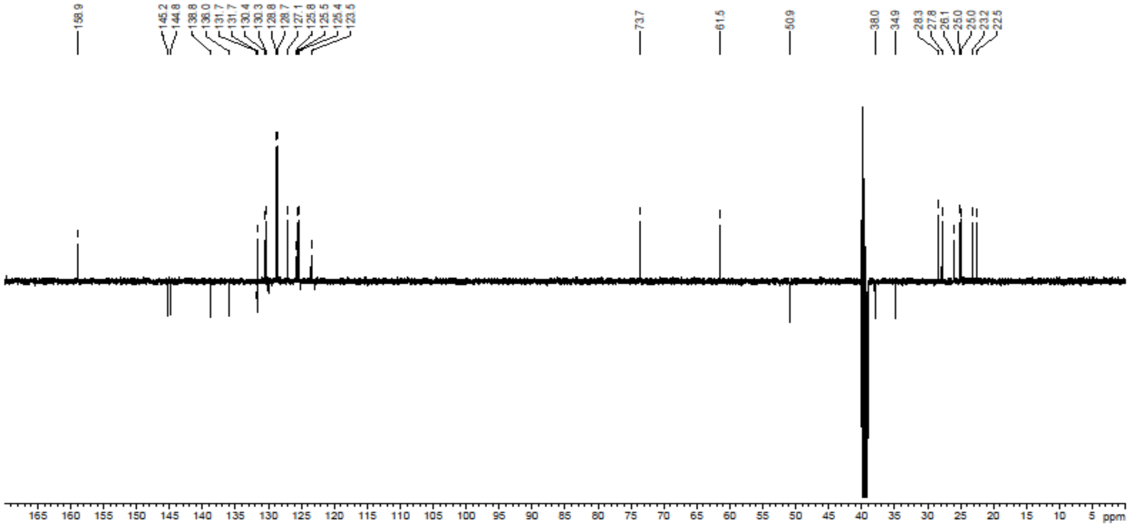

Figure S10. ^{13}C NMR (125 MHz) spectrum of **7a** in DMSO-d_6

(5*R*,1’*S*)-5-*tert*-butyl-1-(2,6-di(propan-2-yl)phenyl)-3-[2-phenyl-1-[3-(trifluoromethyl)phenyl]ethyl]-4,5-dihydro-1*H*-imidazol-3-ium chloride [(5*R*,1’*S*)-7b]

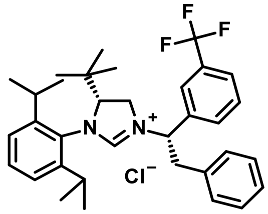

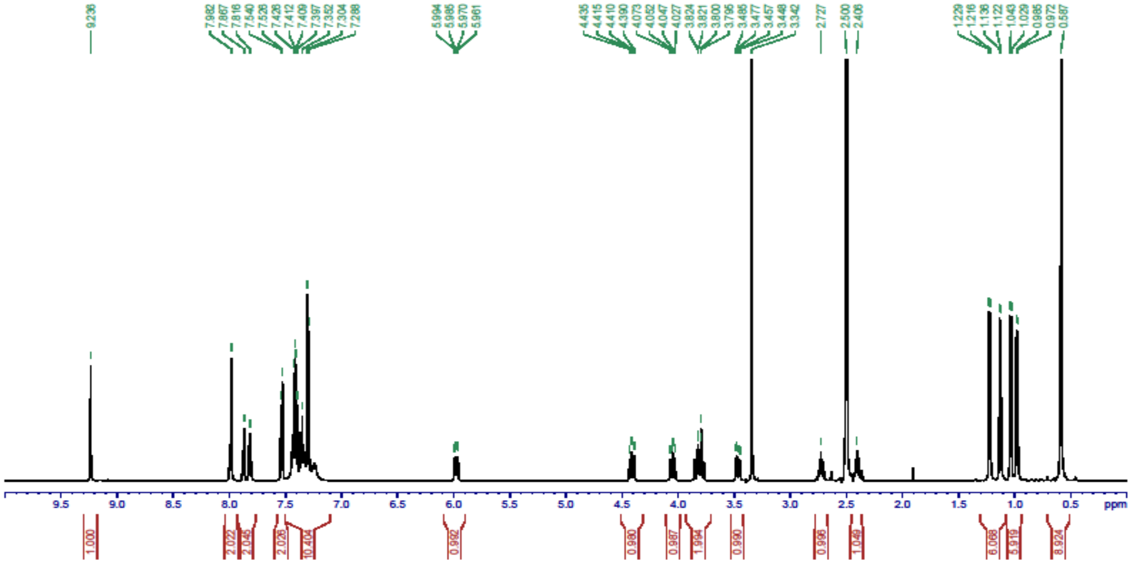

Figure S11. ^{1}H NMR (500 MHz) spectrum of **7b** in DMSO-d_6

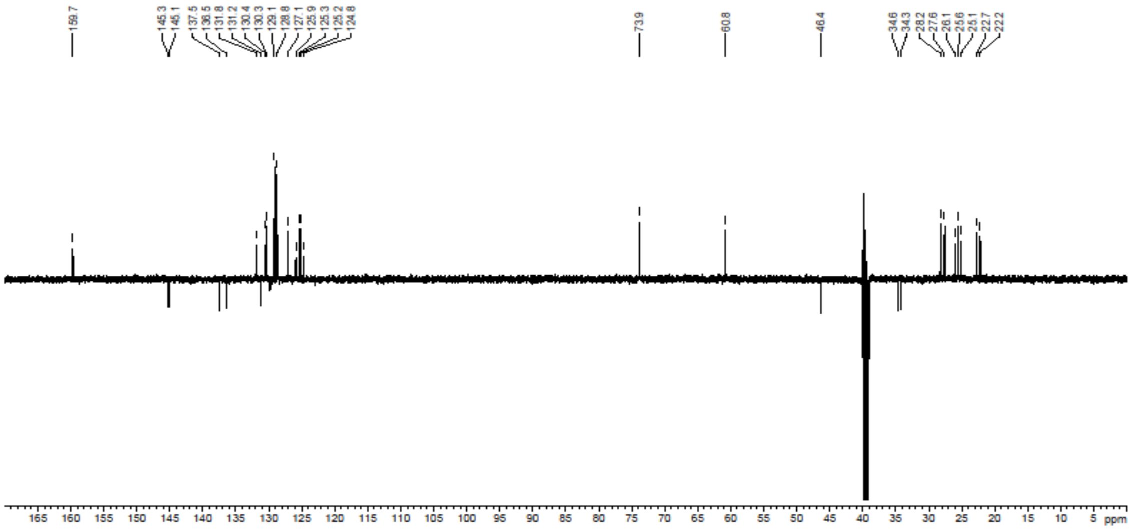

Figure S12. ^{13}C NMR (125 MHz) spectrum of **7b** in DMSO-d_6

(5*S*,1'*S*)-5-*tert*-butyl-1-(2,6-di(propan-2-yl)phenyl)-3-[1-(2-methoxyphenyl)-2-phenylethyl]-4,5-dihydro-1*H*-imidazol-3-ium chloride [(5*S*,1'*S*)-8a]

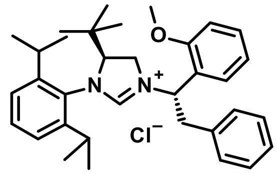

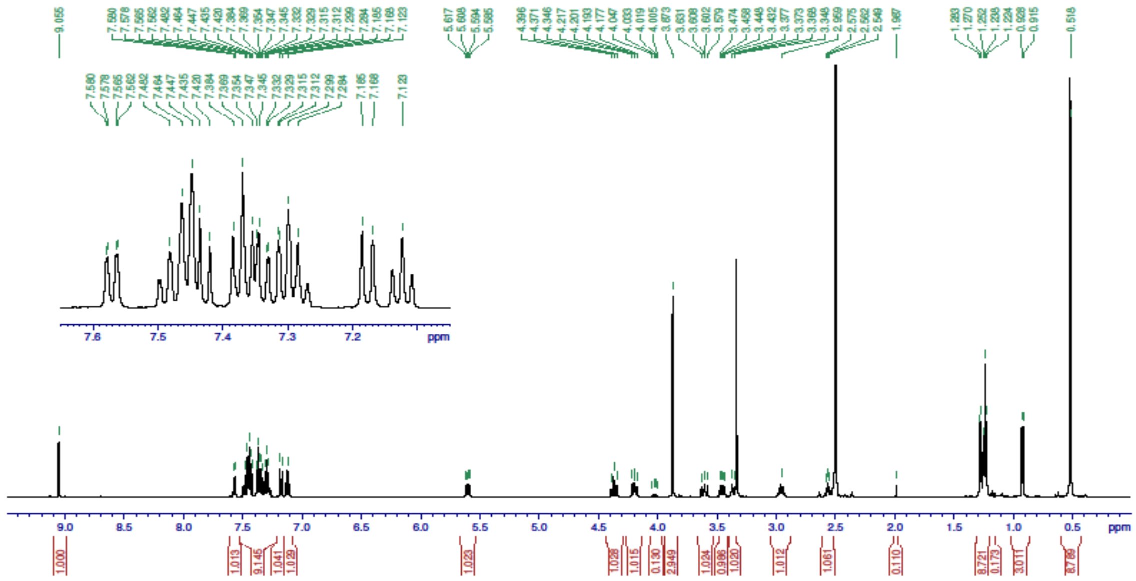

Figure S13. ^{1}H NMR (500 MHz) spectrum of **8a** in DMSO-d_6

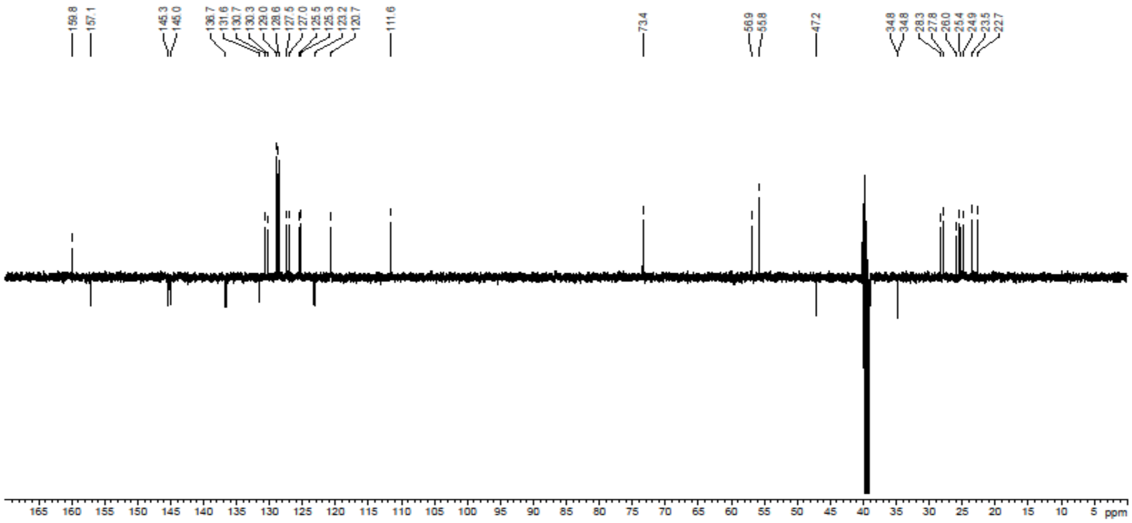

Figure S14. ^{13}C NMR (125 MHz) spectrum of **8a** in DMSO-d_6

(5*S*,1'*R*)-5-*tert*-butyl-1-(2,6-di(propan-2-yl)phenyl)-3-[1-(2-methoxyphenyl)-2-phenylethyl]-4,5-dihydro-1*H*-imidazol-3-ium chloride [(5*S*,1'*R*)-8b]

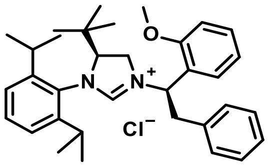

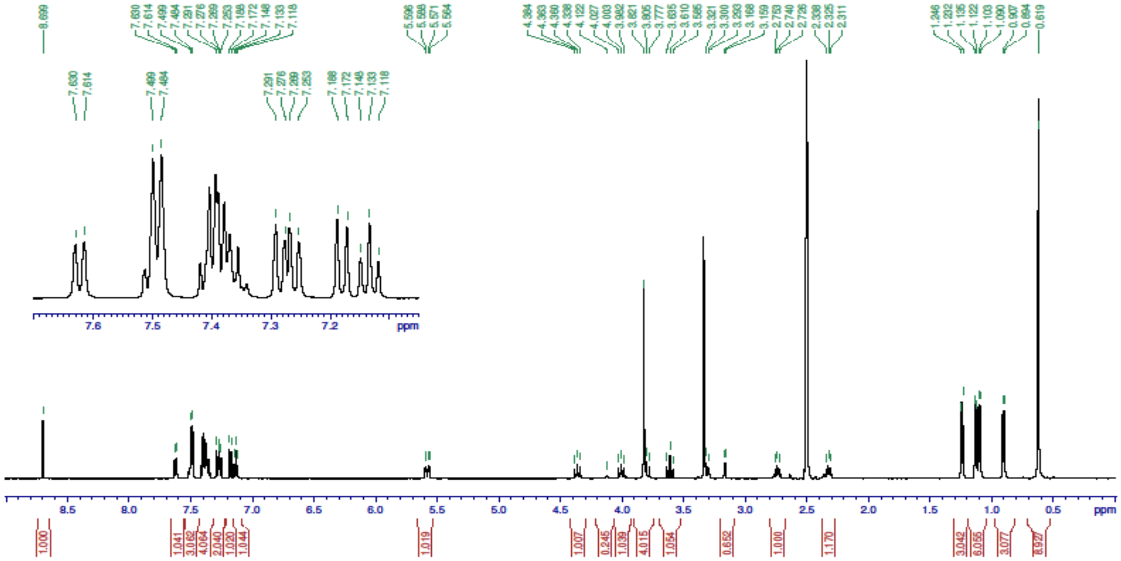

Figure S15. ^{1}H NMR (500 MHz) spectrum of **8b** in DMSO-d_6

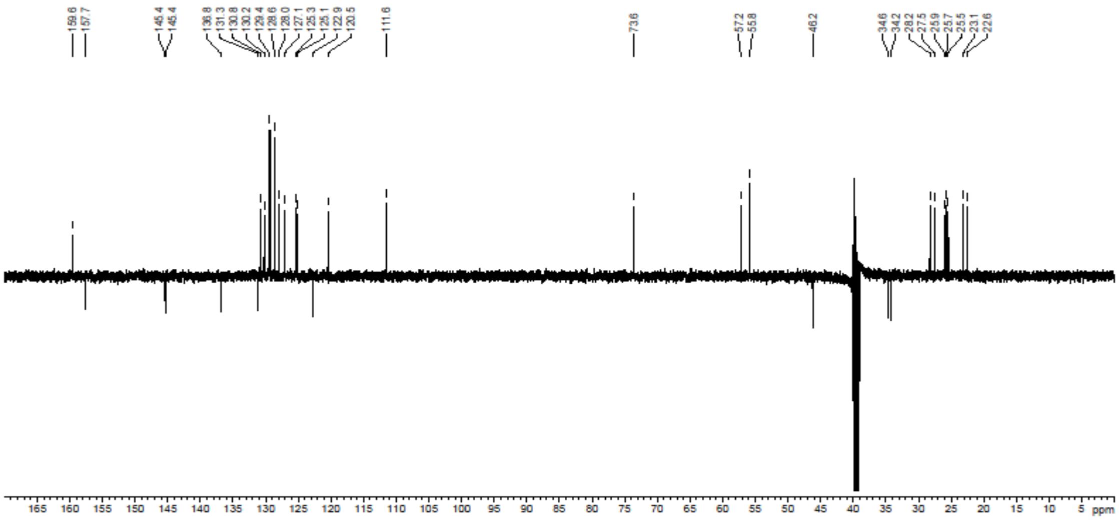

Figure S16. ^{13}C NMR (125 MHz) spectrum of **8b** in DMSO-d_6

(5*S*,1'*S*)-5-*tert*-butyl-1-(2,6-di(propan-2-yl)phenyl)-3-[1-(3-methoxyphenyl)-2-phenylethyl]-4,5-dihydro-1*H*-imidazol-3-ium chloride [(5*S*,1'*S*)-9a]

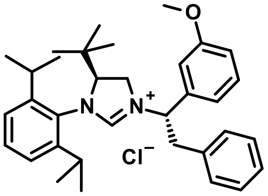

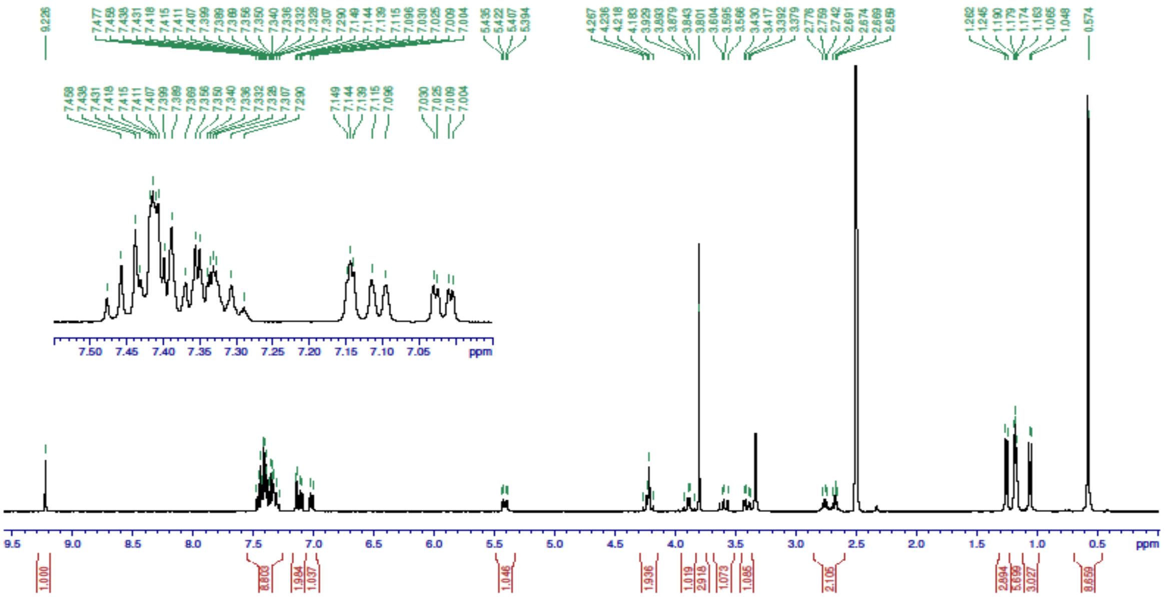

Figure S17. ^{1}H NMR (400 MHz) spectrum of **9a** in DMSO-d_6

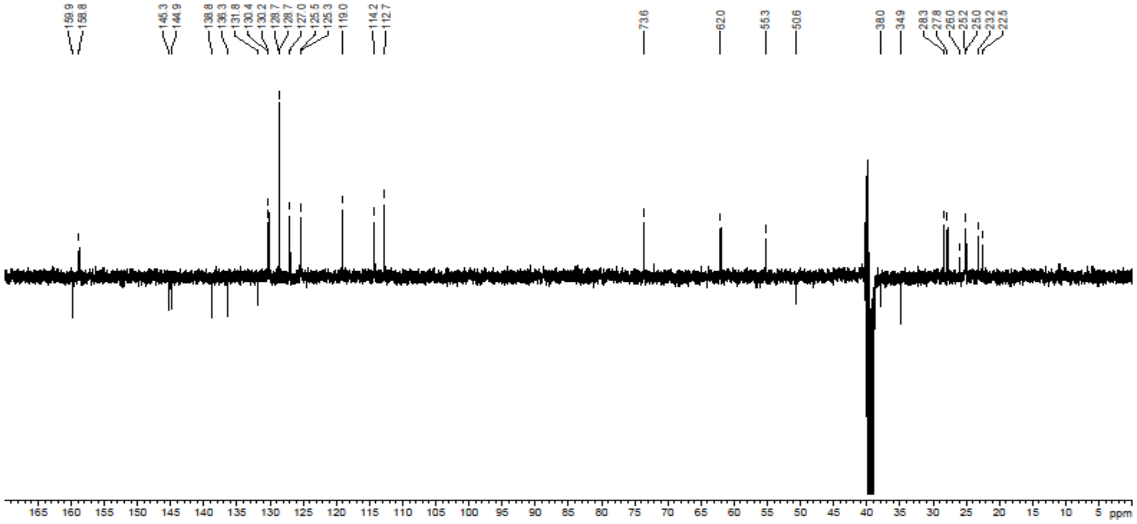

Figure S18. ^{13}C NMR (100 MHz) spectrum of **9a** in DMSO-d_6

(5*S*,1'*R*)-5-*tert*-butyl-1-(2,6-di(propan-2-yl)phenyl)-3-[1-(3-methoxyphenyl)-2-phenylethyl]-4,5-dihydro-1*H*-imidazol-3-ium chloride [(5*S*,1'*R*)-9b]

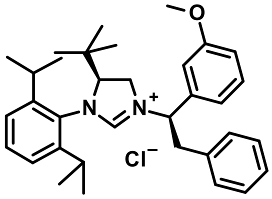

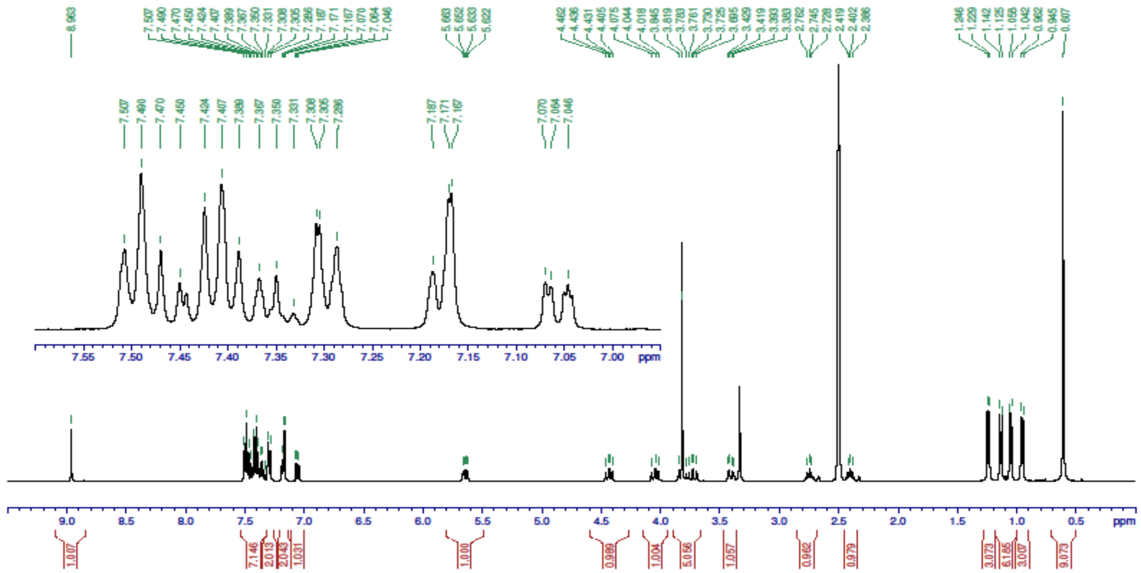

Figure S19. ^{1}H NMR (400 MHz) spectrum of **9b** in DMSO-d_6

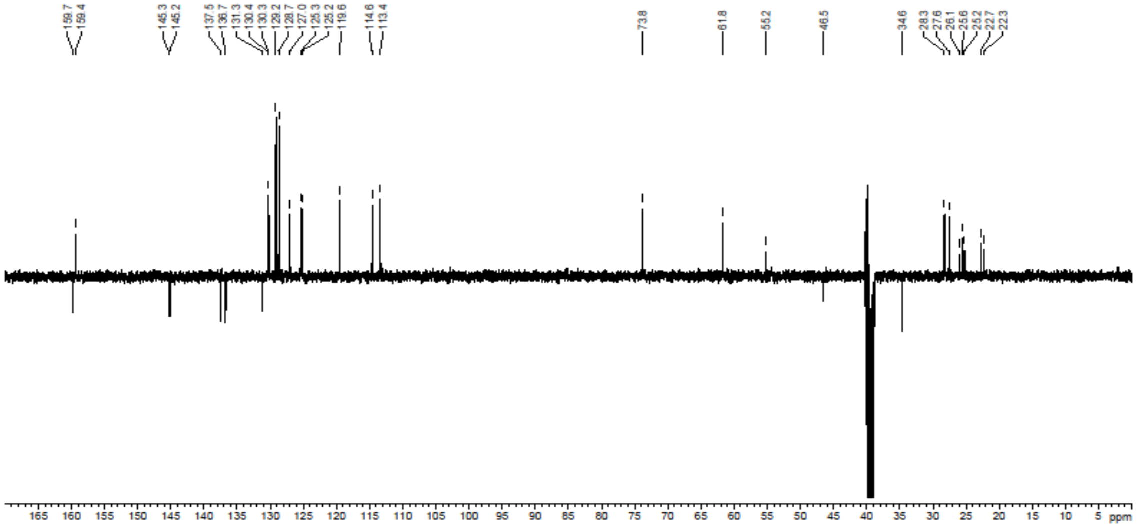

Figure S20. ^{13}C NMR (100 MHz) spectrum of **9b** in DMSO-d_6

(5*S*,1'*R*)-5-*tert*-butyl-1-(2,6-di(propan-2-yl)phenyl)-3-[1-(4-methoxyphenyl)-2-phenylethyl]-4,5-dihydro-1*H*-imidazol-3-ium chloride [(5*S*,1'*R*)-10a]

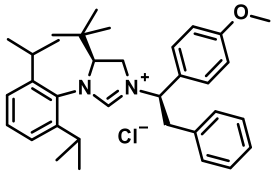

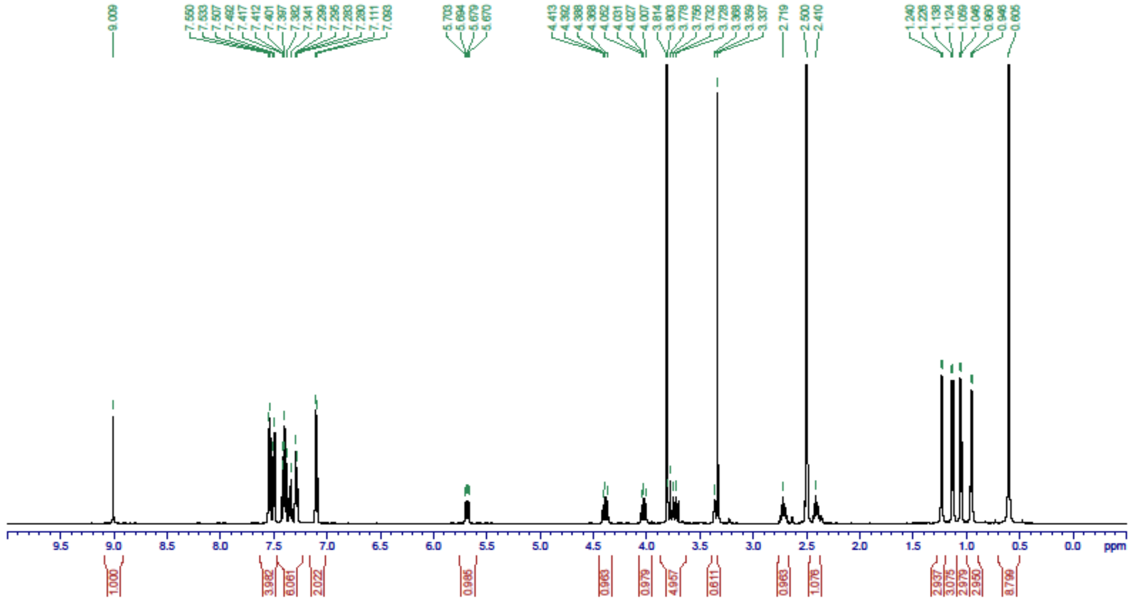

Figure S21. ^{1}H NMR (500 MHz) spectrum of **10a** in DMSO-d_6

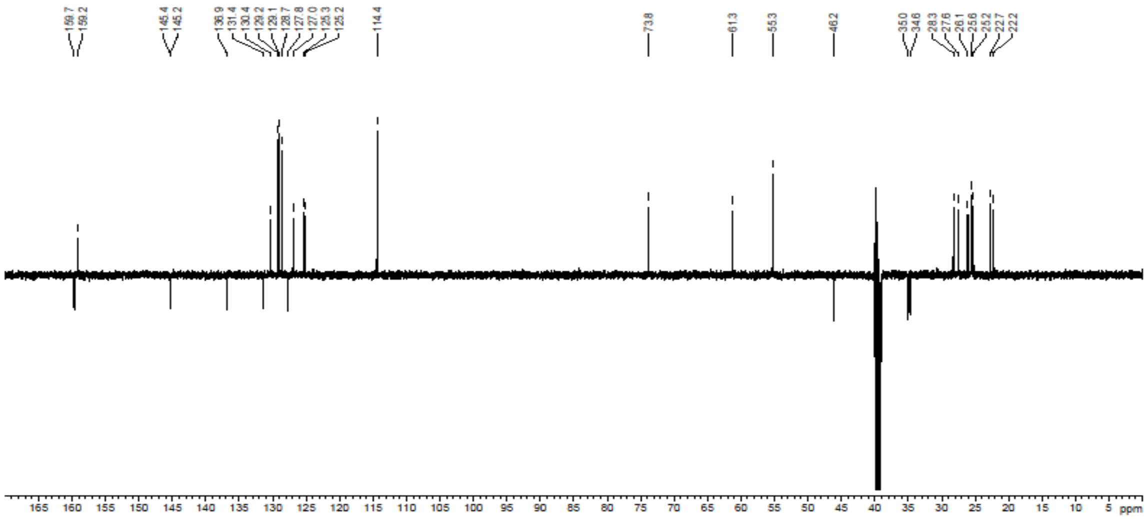

Figure S22. ^{13}C NMR (125 MHz) spectrum of **10a** in DMSO-d_6

(5*S*,1'*S*)-5-*tert*-butyl-1-(2,6-di(propan-2-yl)phenyl)-3-[1-(4-methoxyphenyl)-2-phenylethyl]-4,5-dihydro-1*H*-imidazol-3-ium chloride [(5*S*,1'*S*)-10b]

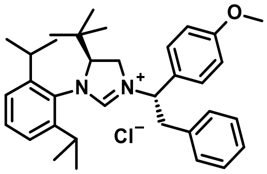

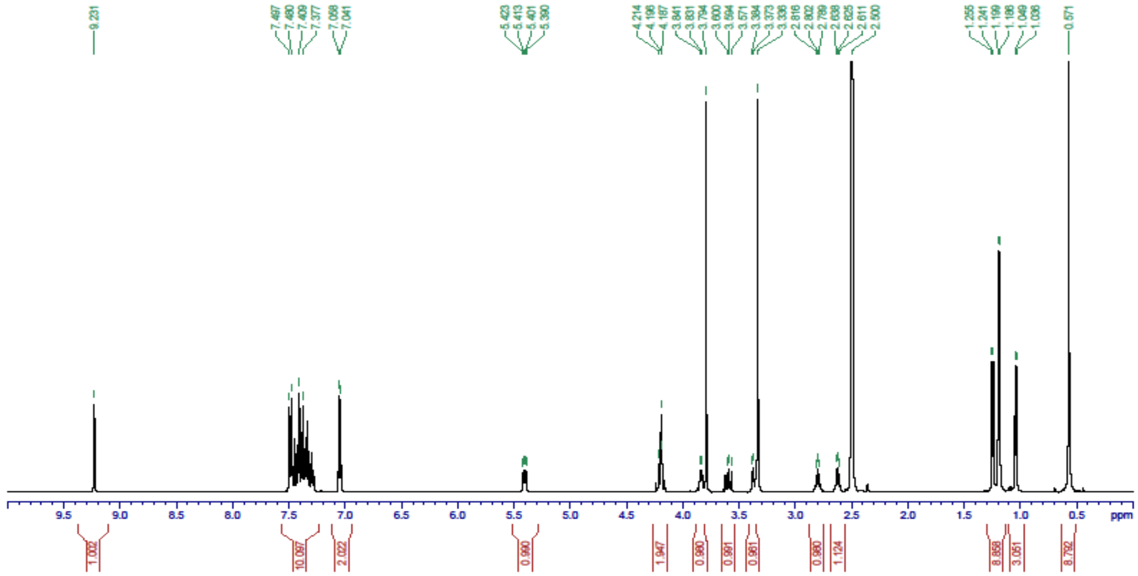

Figure S23. ^{1}H NMR (500 MHz) spectrum of **10b** in DMSO-d_6

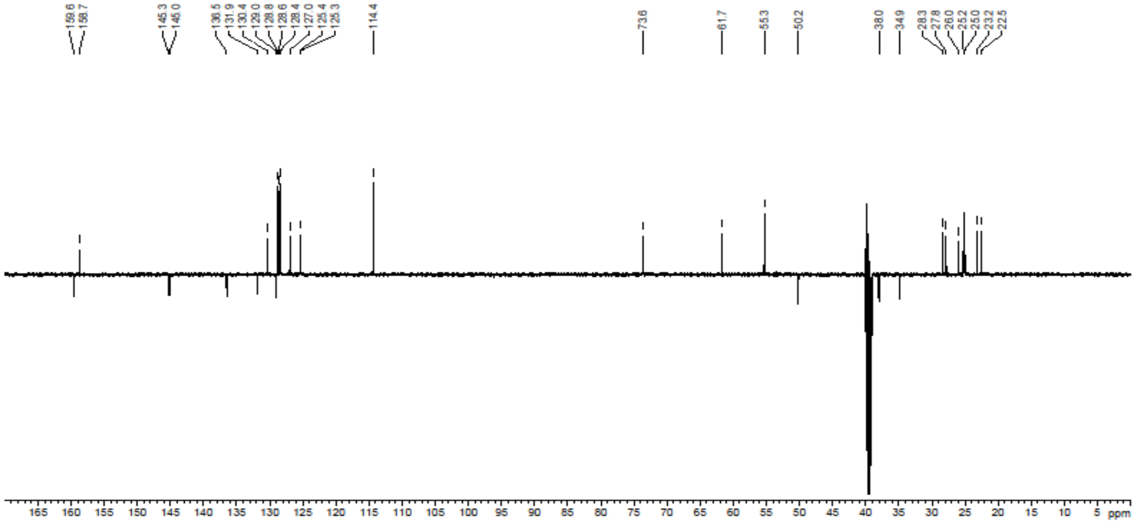

Figure S24. ^{13}C NMR (125 MHz) spectrum of **10b** in DMSO-d_6

(5*S*,1'*S*)-5-*tert*-butyl-1-(2,6-di(propan-2-yl)phenyl)-3-[1-(o-tolyl)-2-phenylethyl]-4,5-dihydro-1*H*-imidazol-3-ium chloride [(5*S*,1'*S*)-11a]

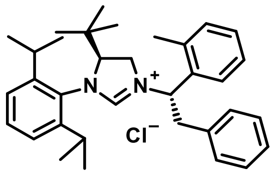

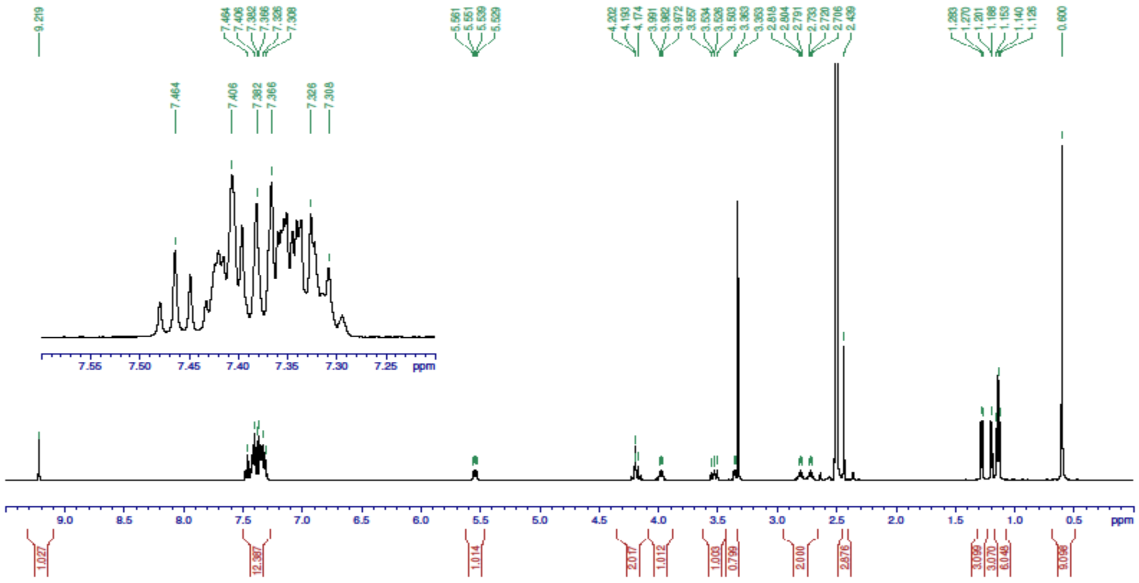

Figure S25. ^{1}H NMR (500 MHz) spectrum of **11a** in DMSO-d_6

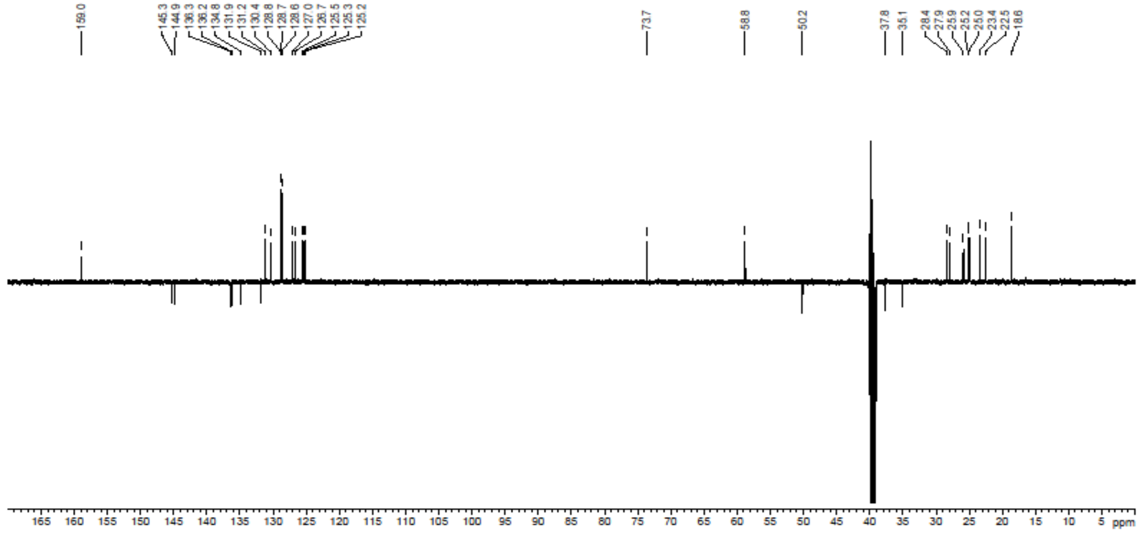

Figure S26. ^{13}C NMR (125 MHz) spectrum of **11a** in DMSO-d_6

(5*S*,1'*R*)-5-*tert*-butyl-1-(2,6-di(propan-2-yl)phenyl)-3-[1-(o-tolyl)-2-phenylethyl]-4,5-dihydro-1*H*-imidazol-3-ium chloride [(5*S*,1'*R*)-11b]

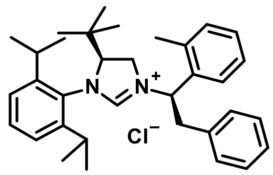

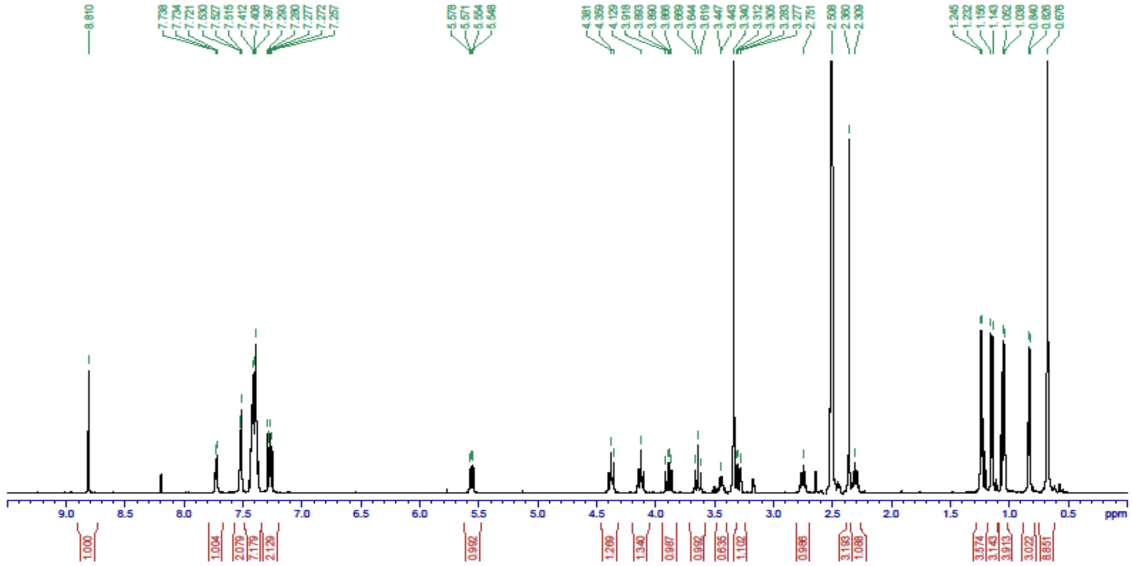

Figure S27. ^{1}H NMR (500 MHz) spectrum of **11b** in DMSO-*d*$_6$

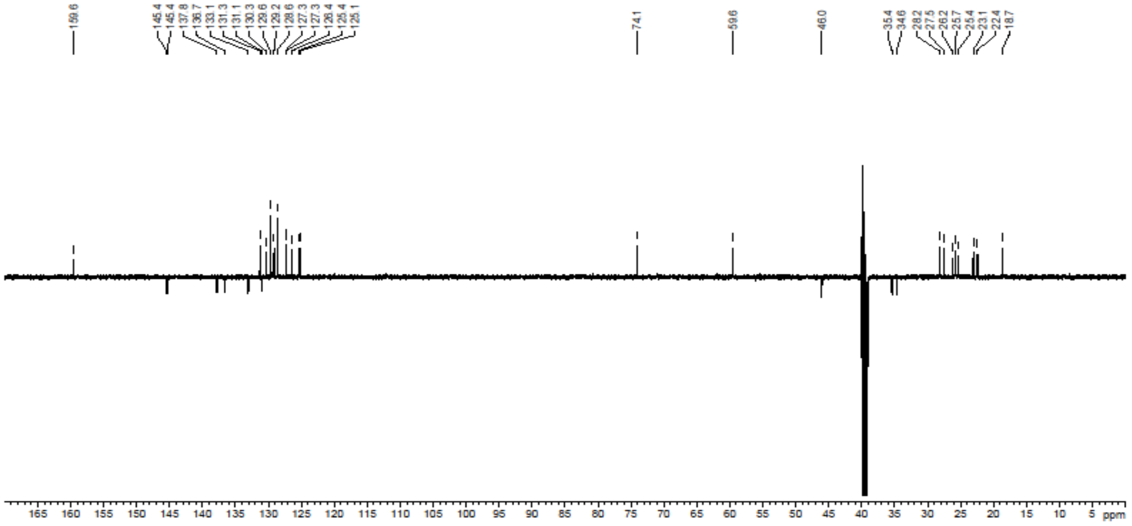

Figure S28. ^{13}C NMR (125 MHz) spectrum of **11b** in DMSO-*d*$_6$

(5*S*,1'*R*)-5-*tert*-butyl-1-(2,6-di(propan-2-yl)phenyl)-3-[2-phenyl-1-(p-tolyl)ethyl]-4,5-dihydro-1*H*-imidazol-3-ium chloride [(5*S*,1'*R*)-12a]

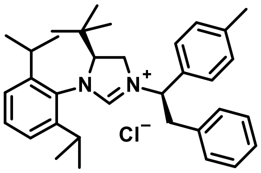

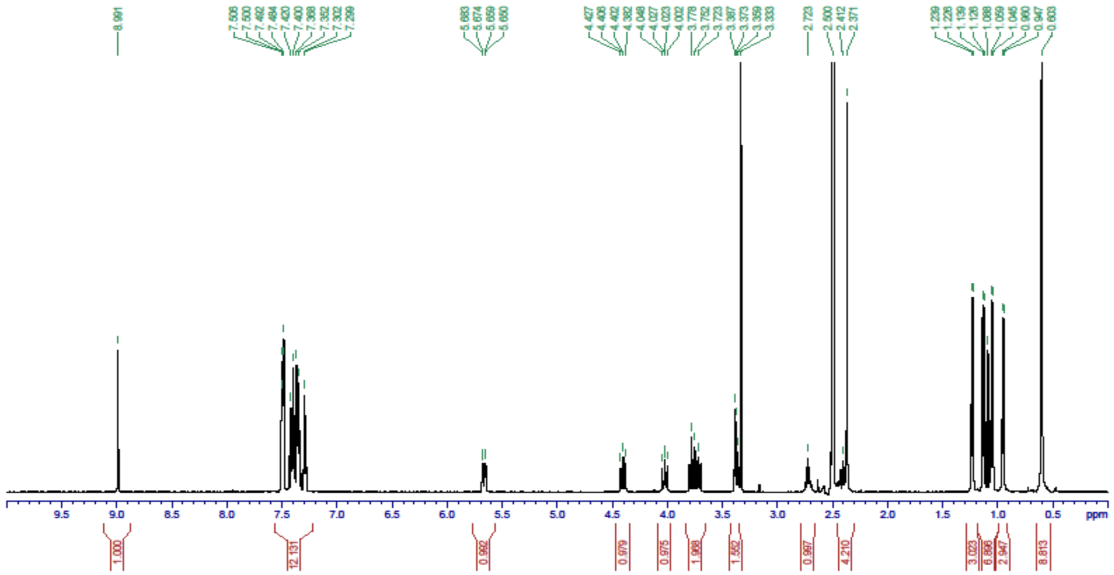

Figure S29. ^{1}H NMR (500 MHz) spectrum of **12a** in DMSO-d_6

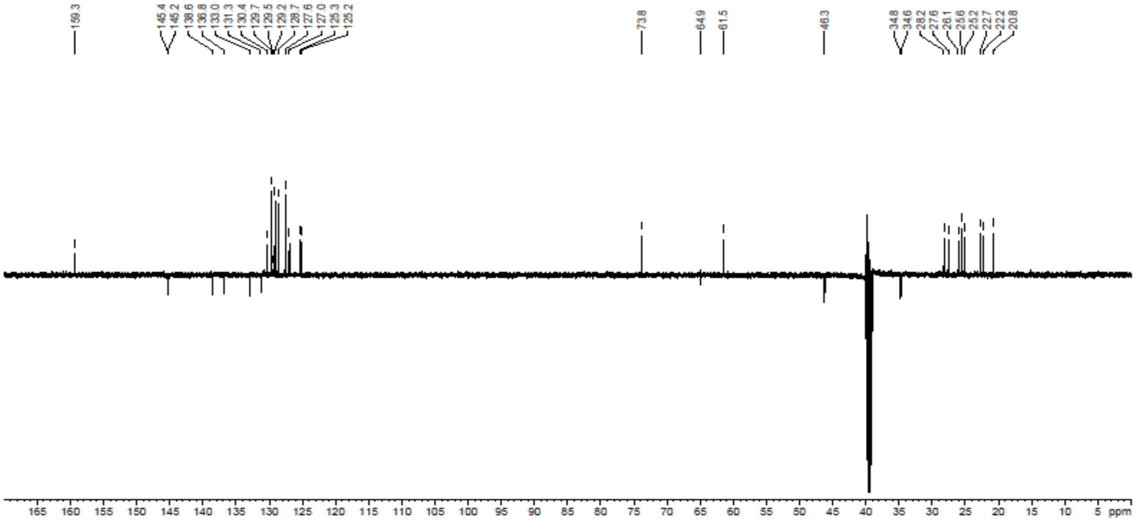

Figure S30. ^{13}C NMR (125 MHz) spectrum of **12a** in DMSO-d_6

(5*S*,1'*S*)-5-*tert*-butyl-1-(2,6-di(propan-2-yl)phenyl)-3-[2-phenyl-1-(p-tolyl)ethyl]-4,5-dihydro-1*H*-imidazol-3-ium chloride [(5*S*,1'*S*)-12b]

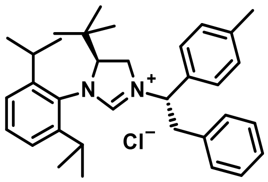

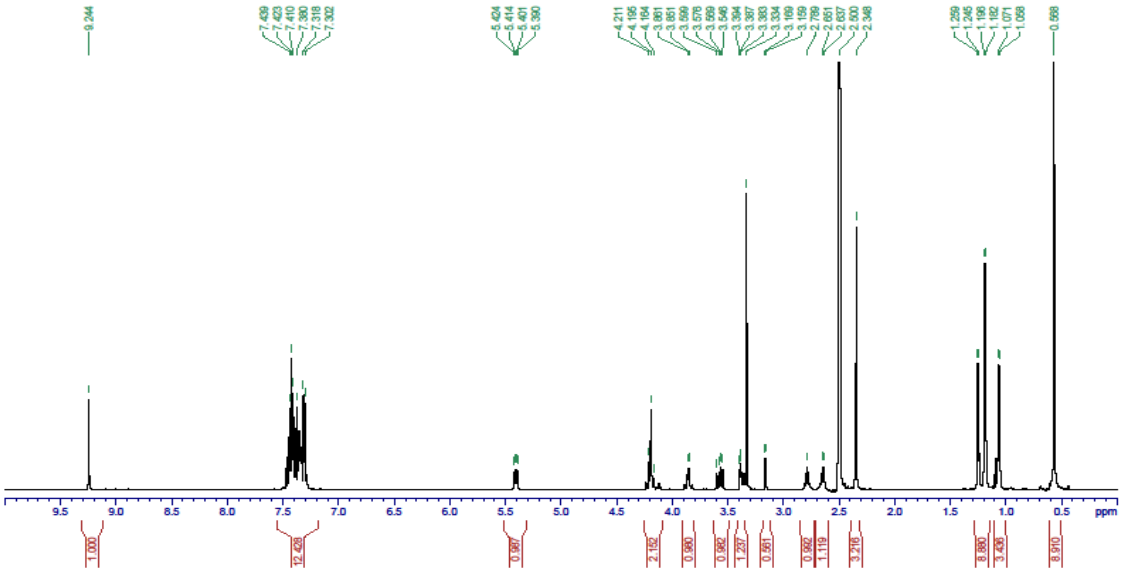

Figure S31. ^{1}H NMR (500 MHz) spectrum of **12b** in DMSO-d_6

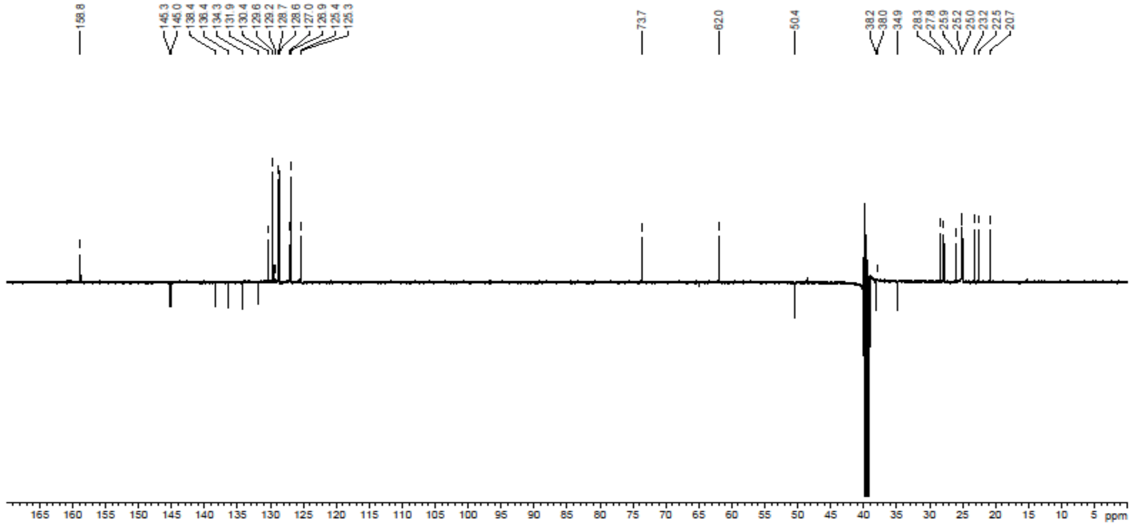

Figure S32. ^{13}C NMR (125 MHz) spectrum of **12b** in DMSO-d_6

(5*S*,1’*R*)-5-*tert*-butyl-1-(2,6-di(propan-2-yl)phenyl)-3-[2-phenyl-1-[4-(trifluoromethyl)phenyl]ethyl]-4,5-dihydro-1*H*-imidazol-3-ium chloride [(5*S*,1’*R*)-13a]

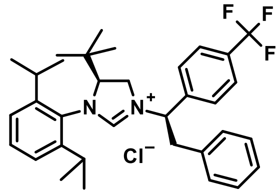

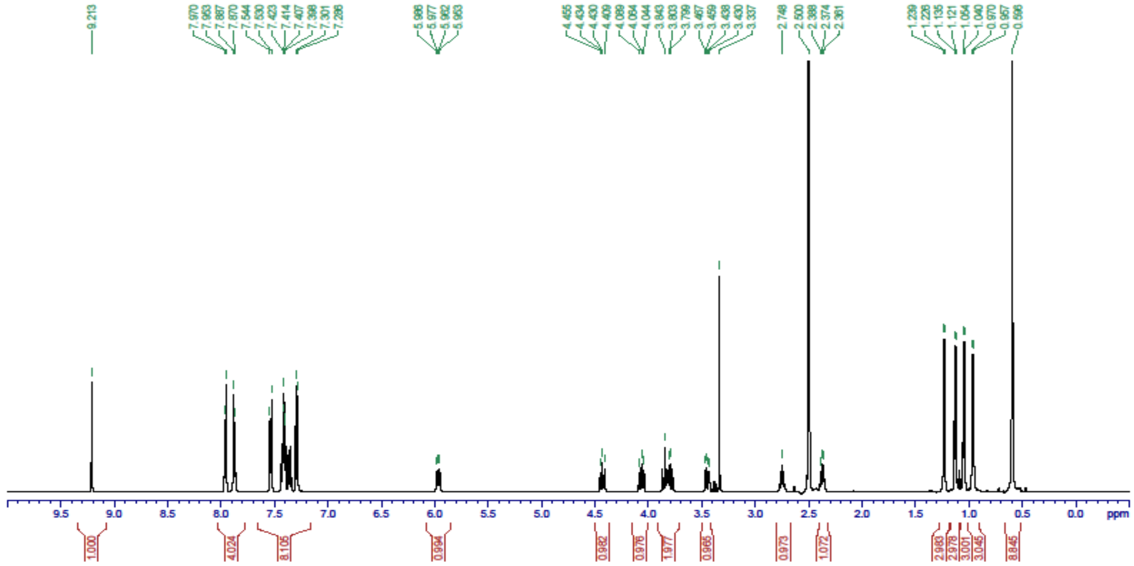

Figure S33. ^{1}H NMR (500 MHz) spectrum of **13a** in DMSO-d_6

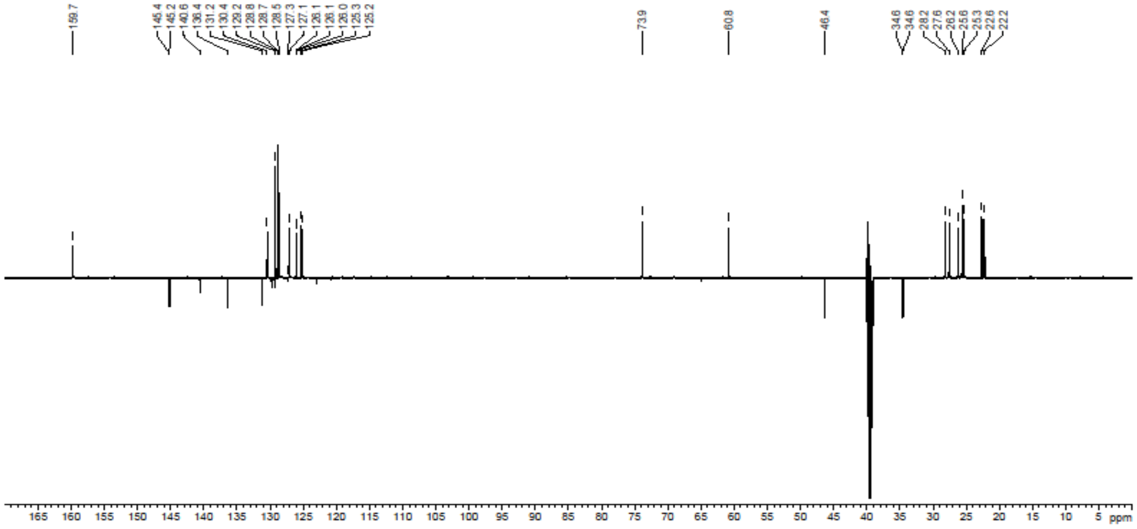

Figure S34. ^{13}C NMR (125 MHz) spectrum of **13a** in DMSO-d_6

(5*S*,1’*S*)-5-*tert*-butyl-1-(2,6-di(propan-2-yl)phenyl)-3-[2-phenyl-1-[4-(trifluoromethyl)phenyl]ethyl]-4,5-dihydro-1*H*-imidazol-3-ium chloride [(5*S*,1’*S*)-13b]

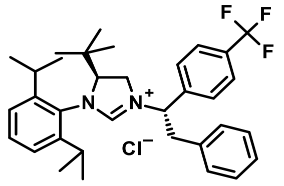

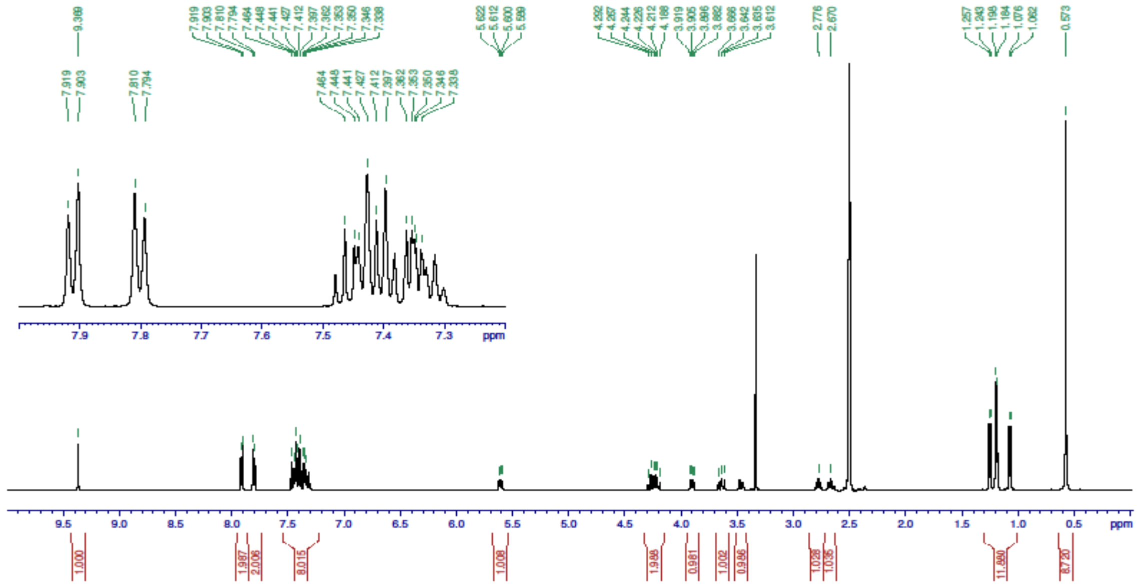

Figure S35. ^{1}H NMR (500 MHz) spectrum of **13b** in DMSO-*d*$_6$

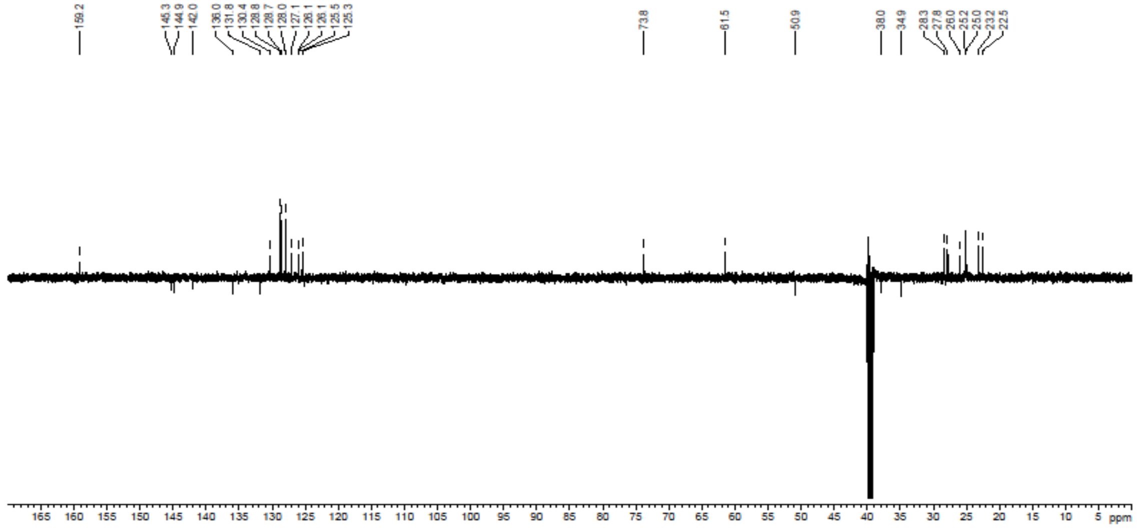

Figure S36. ^{13}C NMR (125 MHz) spectrum of **13b** in DMSO-*d*$_6$

(5*S*,1'*S*)-5-*tert*-butyl-1-(2,6-di(propan-2-yl)phenyl)-3-[1-phenyl-2-(p-tolyl)ethyl]-4,5-dihydro-1*H*-imidazol-3-ium chloride [(5*S*,1'*S*)-14a]

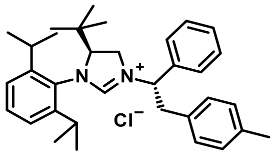

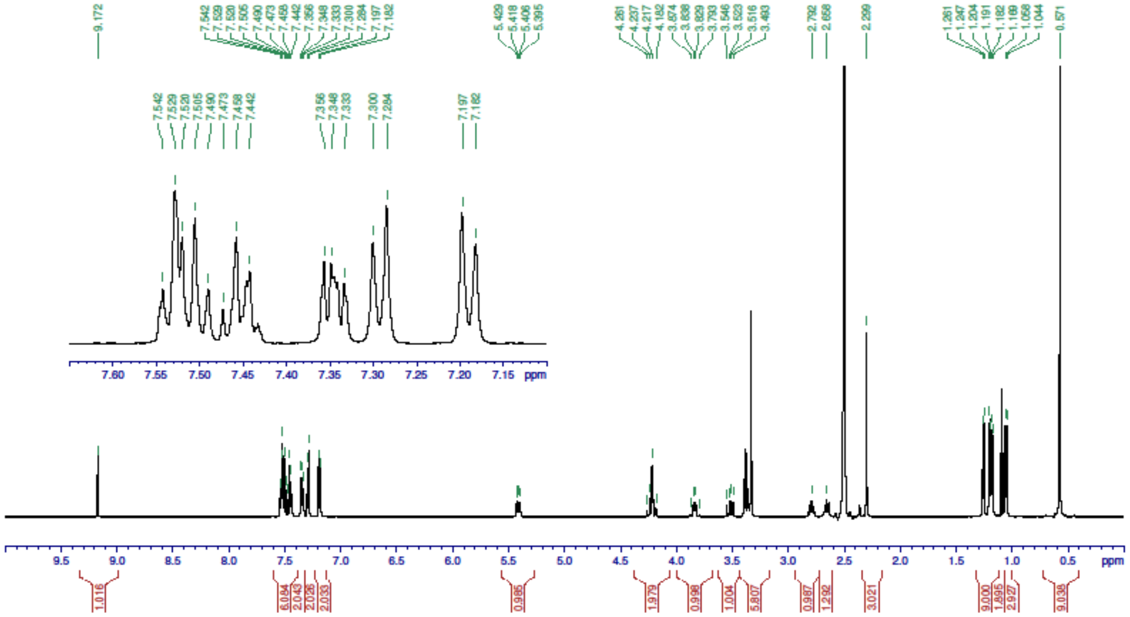

Figure S37. ^{1}H NMR (500 MHz) spectrum of **14a** in DMSO-d_6

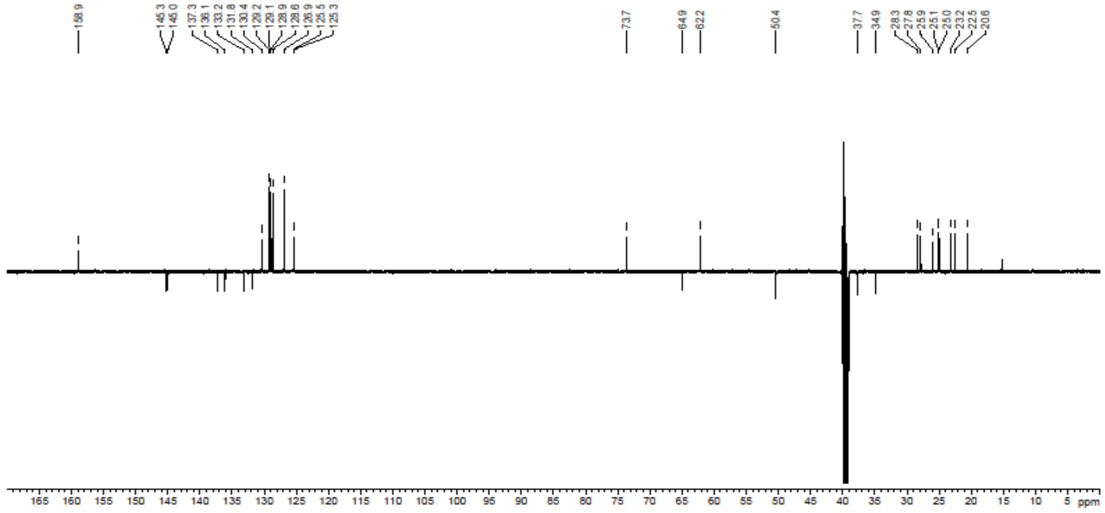

Figure S38. ^{13}C NMR (125 MHz) spectrum of **14a** in DMSO-d_6

(5*S*,1'*R*)-5-*tert*-butyl-1-(2,6-di(propan-2-yl)phenyl)-3-[1-phenyl-2-(p-tolyl)ethyl]-4,5-dihydro-1*H*-imidazol-3-ium chloride [(5*S*,1'*R*)-14b]

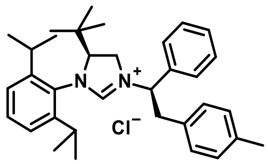

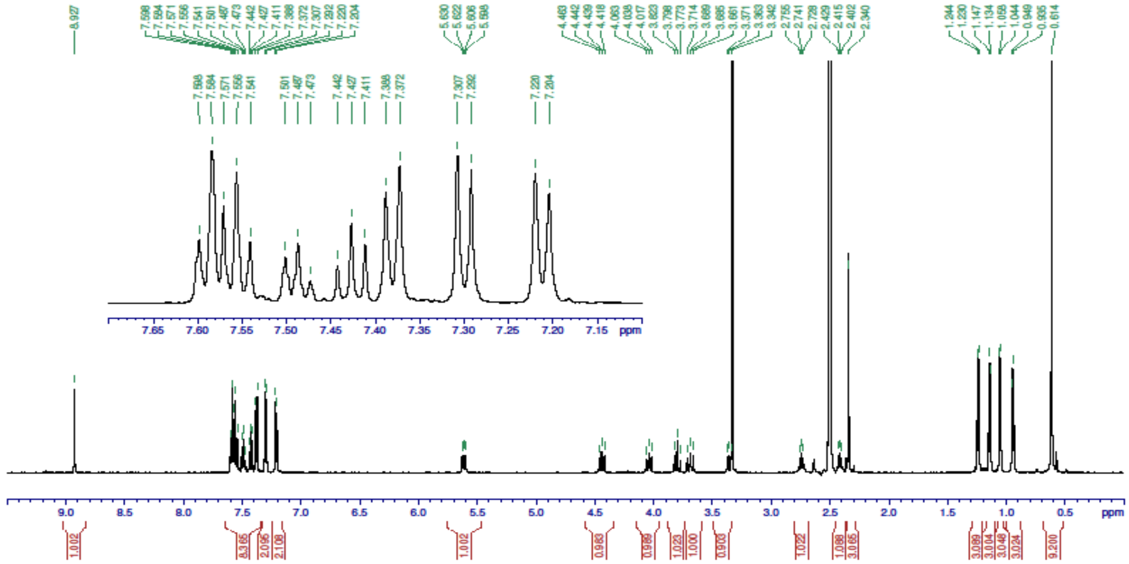

Figure S39. ^{1}H NMR (500 MHz) spectrum of **14b** in DMSO-d_6

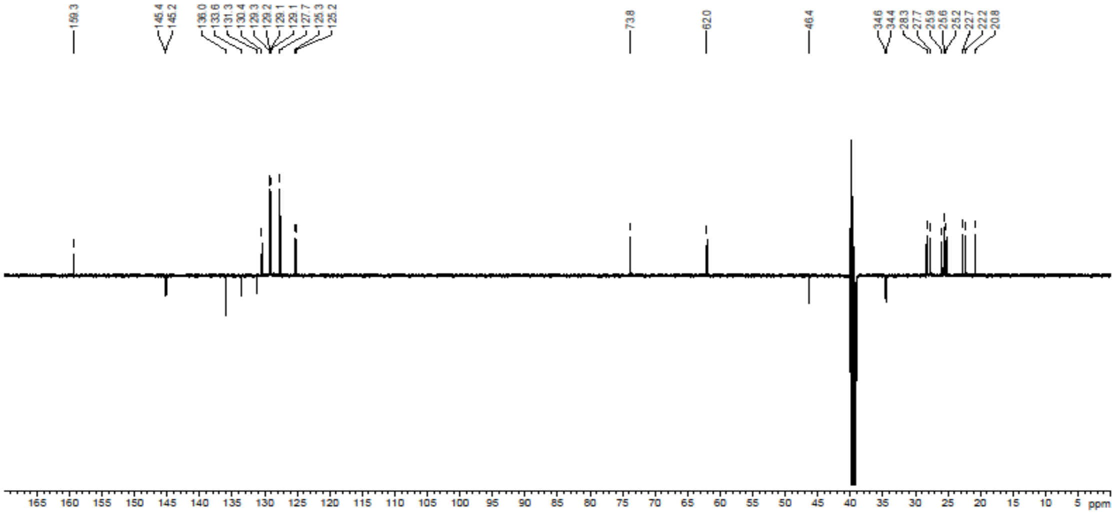

Figure S40. ^{13}C NMR (125 MHz) spectrum of **14b** in DMSO-d_6

(5*S*,1'*S*)-5-*tert*-butyl-3-[2-(4-chlorophenyl)-1-phenylethyl]-1-(2,6-di(propan-2-yl)phenyl)-4,5-dihydro-1*H*-imidazol-3-ium chloride [(5*S*,1'*S*)-15a]

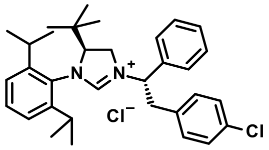

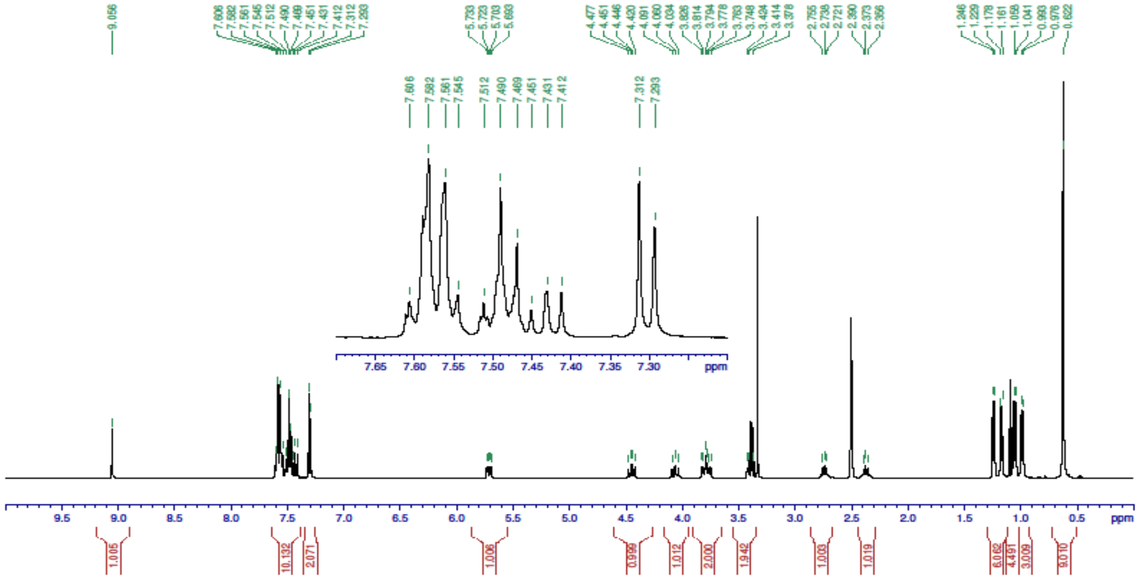

Figure S41. ^{1}H NMR (400 MHz) spectrum of **15a** in DMSO-d_6

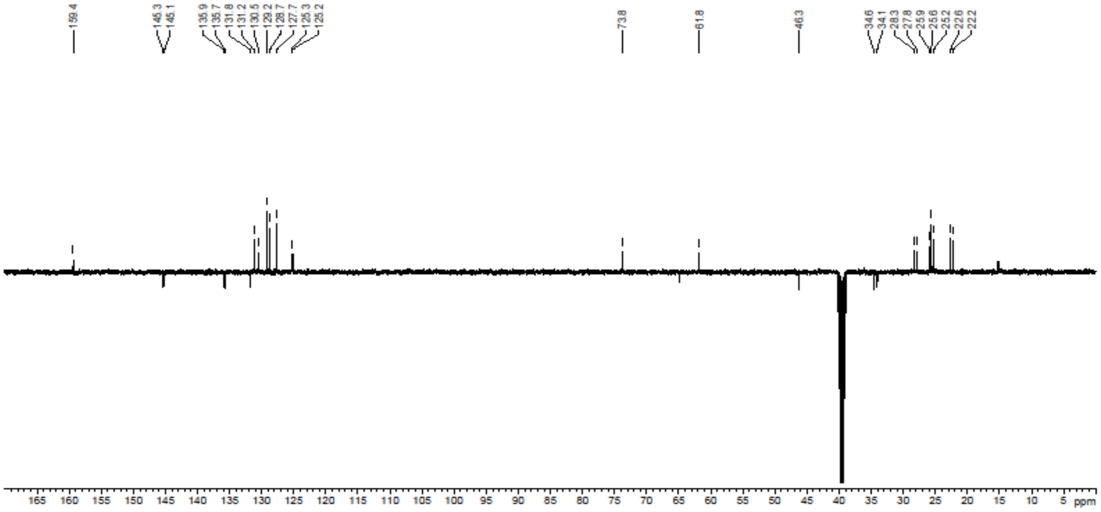

Figure S42. ^{13}C NMR (100 MHz) spectrum of **15a** in DMSO-d_6

(5*S*,1'*R*)-5-*tert*-butyl-3-[2-(4-chlorophenyl)-1-phenylethyl]-1-(2,6-di(propan-2-yl)phenyl)-4,5-dihydro-1*H*-imidazol-3-ium chloride [(5*S*,1'*R*)-15b]

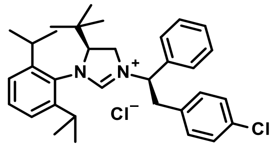

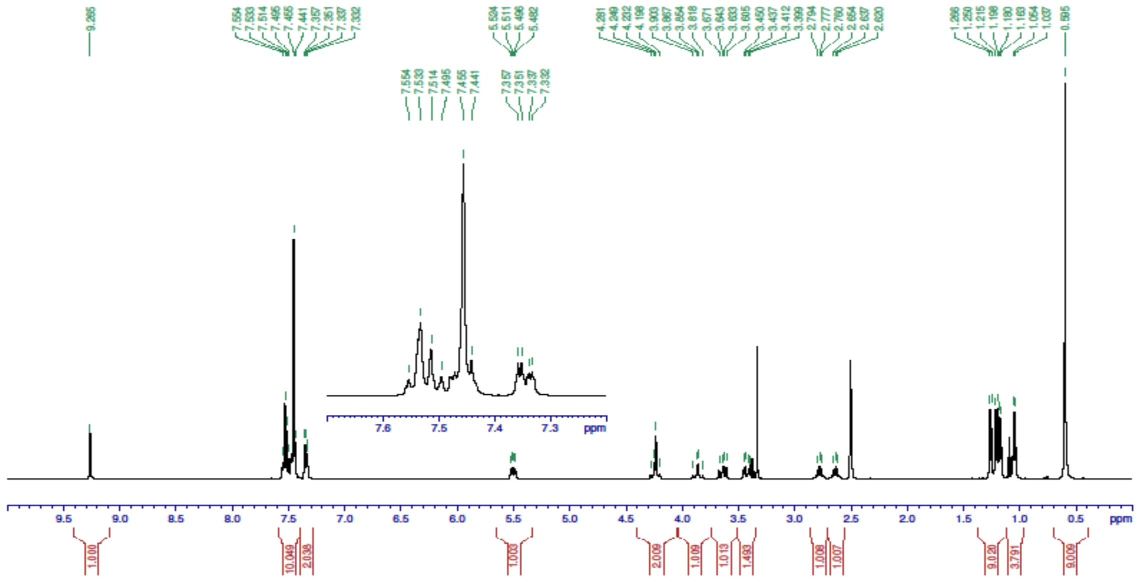

Figure S43. ^{1}H NMR (400 MHz) spectrum of **15b** in DMSO-d_6

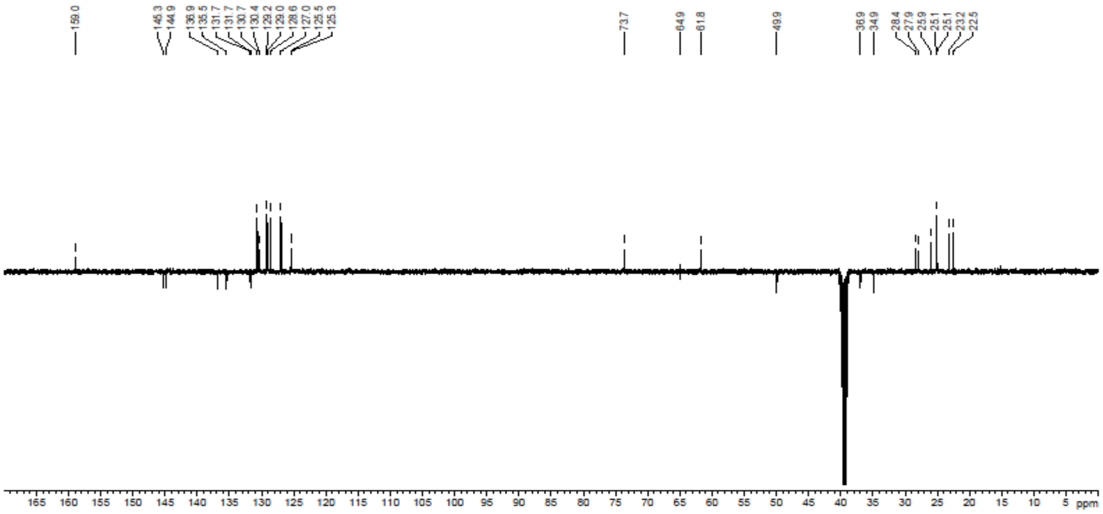

Figure S44. ^{13}C NMR (100 MHz) spectrum of **15b** in DMSO-d_6

(5*S*,1'*S*)-5-*tert*-butyl-3-[2-(2-chlorophenyl)-1-phenylethyl]-1-(2,6-di(propan-2-yl)phenyl)-4,5-dihydro-1*H*-imidazol-3-ium chloride [(5*S*,1'*S*)-16a]

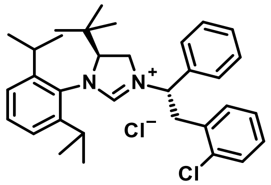

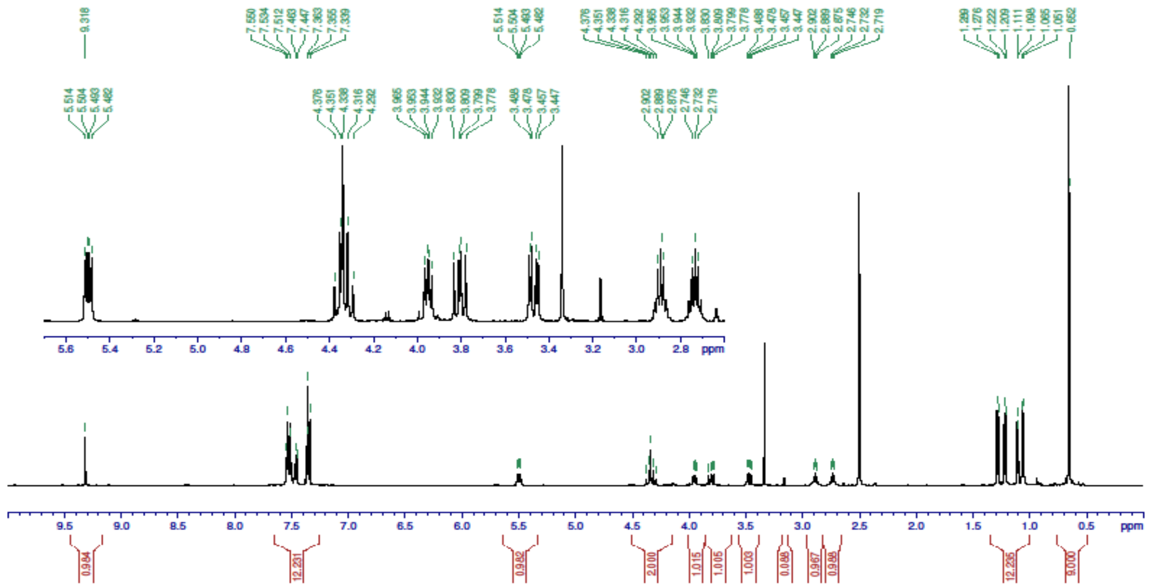

Figure S45. ^{1}H NMR (500 MHz) spectrum of **16a** in DMSO-d_6

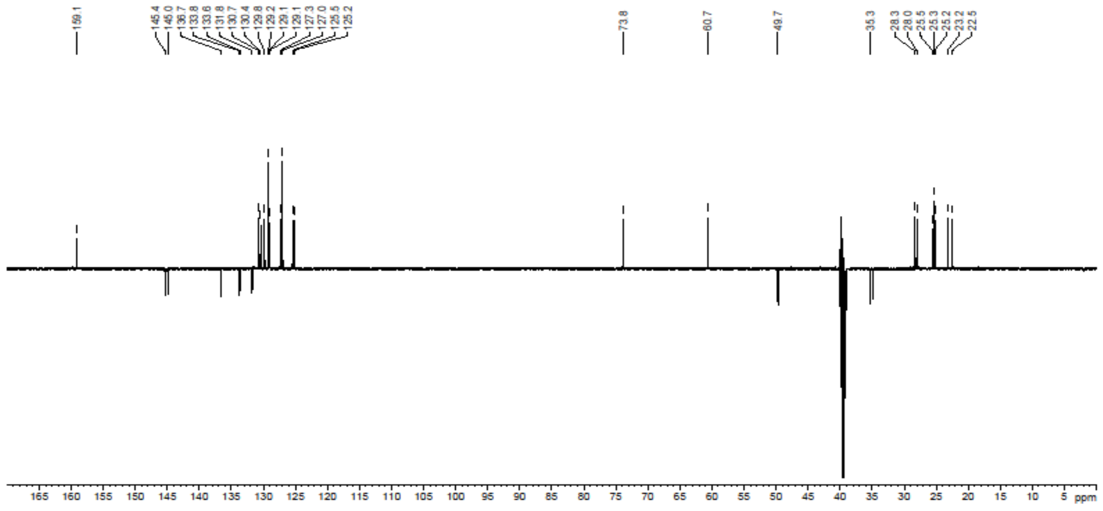

Figure S46. ^{13}C NMR (125 MHz) spectrum of **16a** in DMSO-d_6

(5*S*,1'*R*)-5-*tert*-butyl-3-[2-(2-chlorophenyl)-1-phenylethyl]-1-(2,6-di(propan-2-yl)phenyl)-4,5-dihydro-1*H*-imidazol-3-ium chloride [(5*S*,1'*R*)-16b]

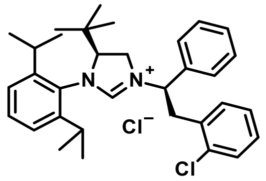

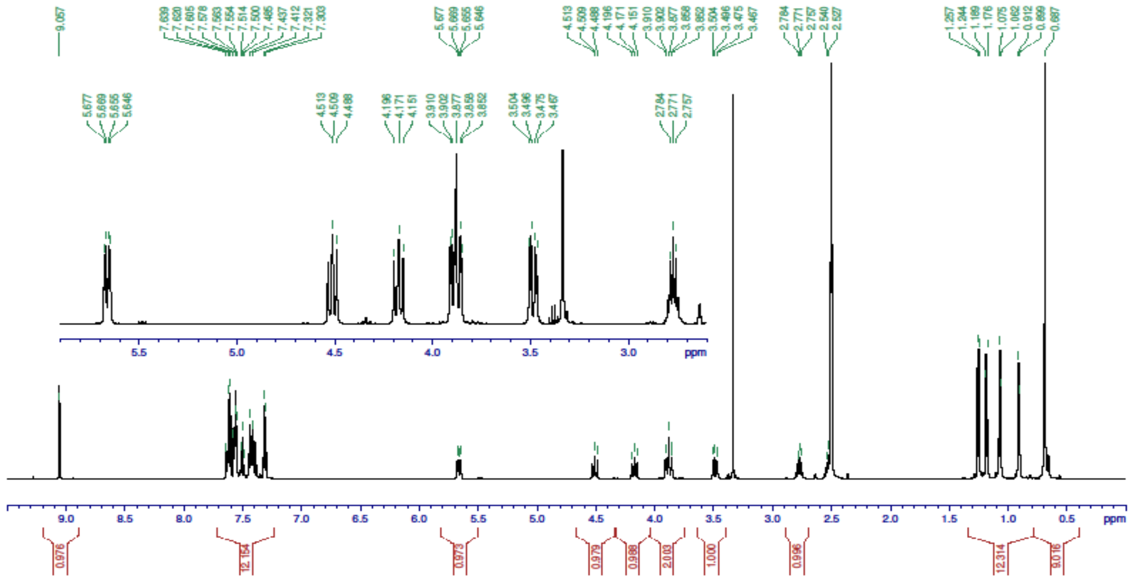

Figure S47. ^{1}H NMR (500 MHz) spectrum of **16b** in DMSO-d_6

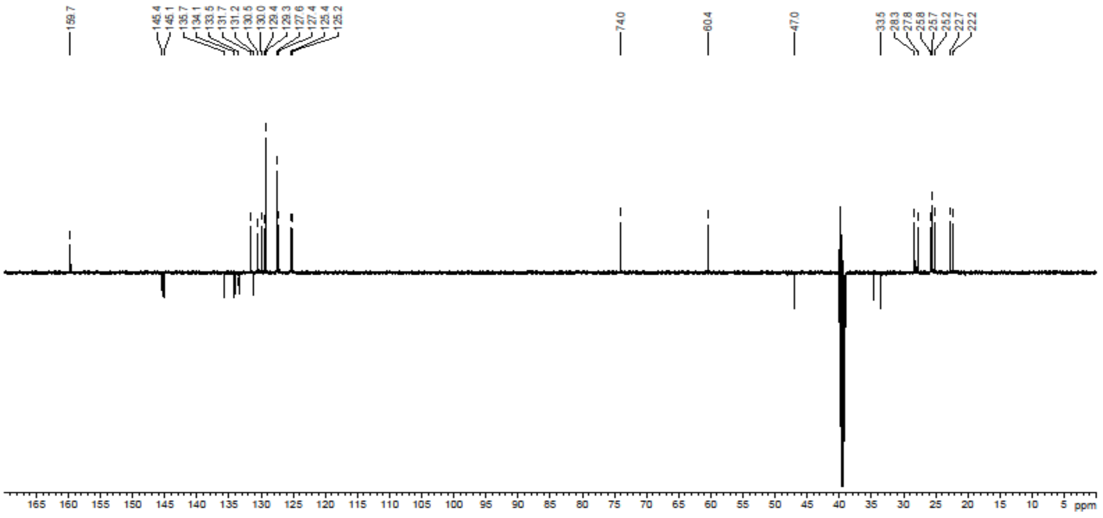

Figure S48. ^{13}C NMR (125 MHz) spectrum of **16b** in DMSO-d_6

(1’*S*)-3-[2-(4-chlorophenyl)-1-phenylethyl]-1-(2,6-di(propan-2-yl)phenyl)-4,5-dihydro-1*H*-imidazol-3-ium chloride [(1’*S*)-17a]

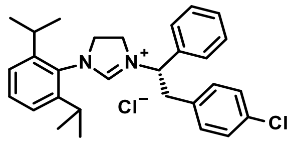

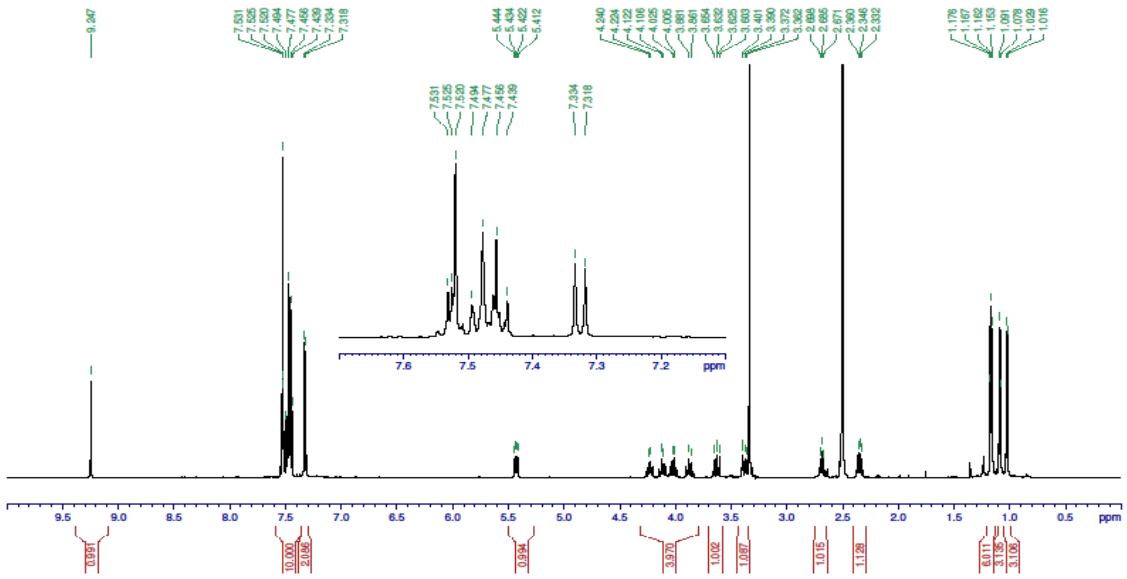

Figure S49. ^{1}H NMR (500 MHz) spectrum of **17a** in DMSO-d_6

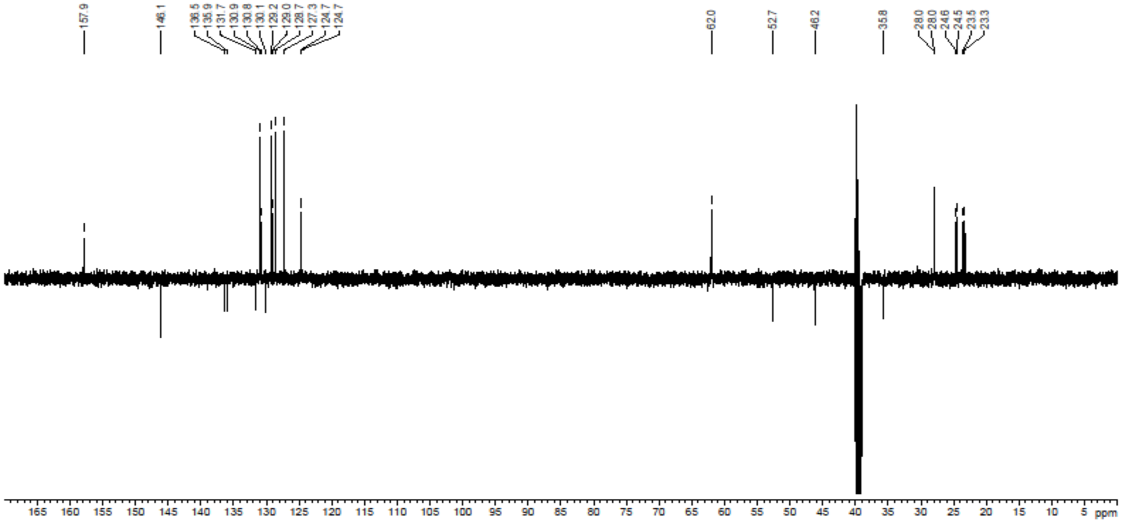

Figure S50. ^{13}C NMR (125 MHz) spectrum of **17a** in DMSO-d_6

(1’*R*)-3-[2-(4-chlorophenyl)-1-phenylethyl]-1-(2,6-di(propan-2-yl)phenyl)-4,5-dihydro-1*H*-imidazol-3-ium chloride [(1’*R*)-17b]

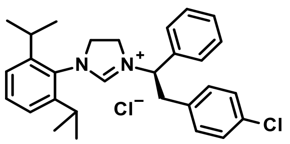

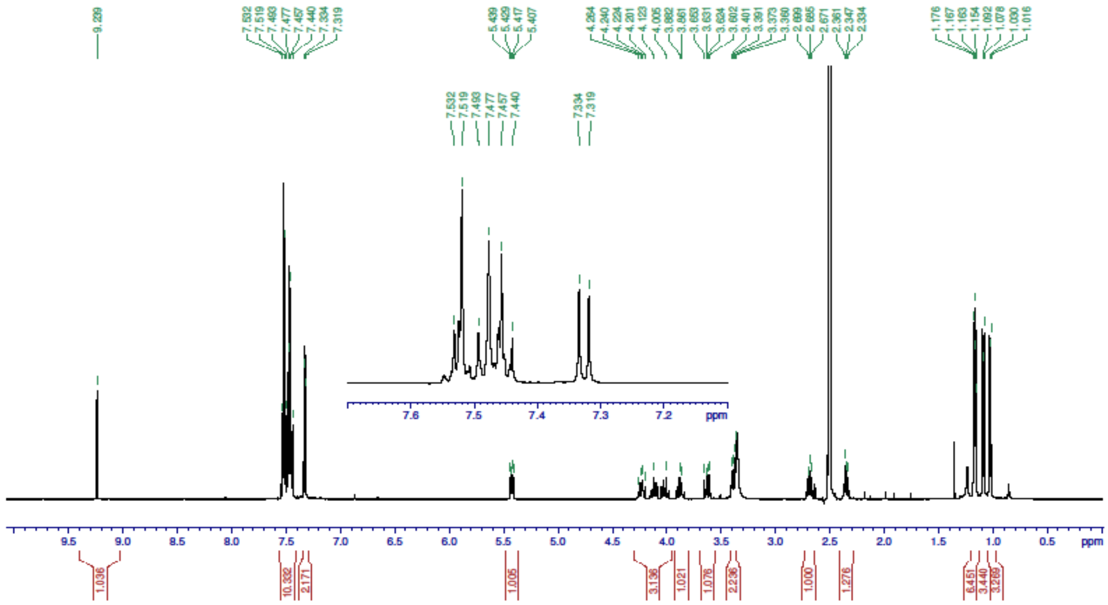

Figure S51. ^{1}H NMR (500 MHz) spectrum of **17b** in DMSO-d_6

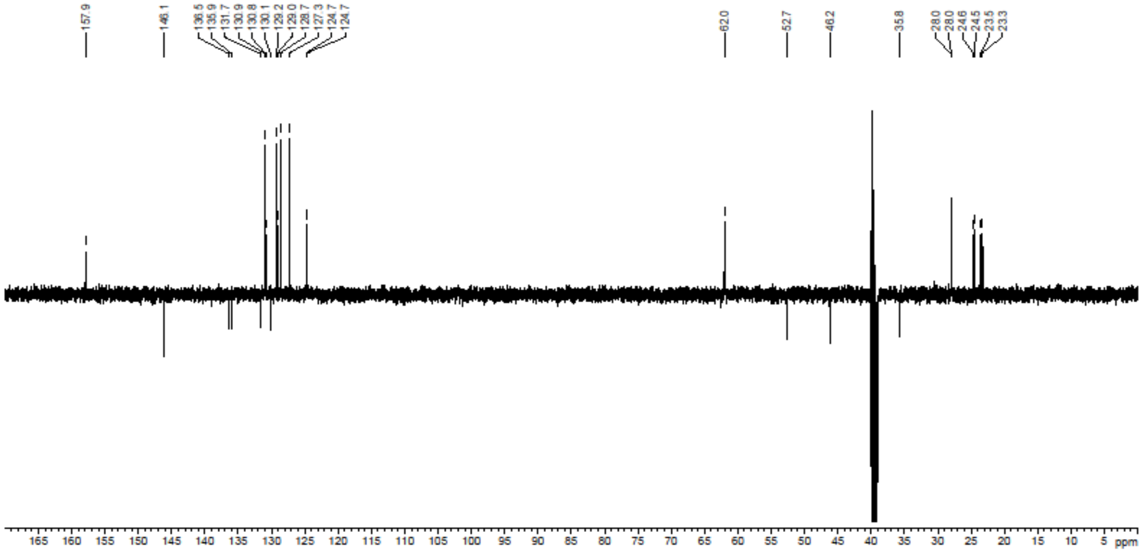

Figure S52. ^{13}C NMR (125 MHz) spectrum of **17b** in DMSO-d_6

(2*S*)-*N*-(2,6-di(propan-2-yl)phenyl)-3,3-dimethylbutane-1,2-diamine (19a)

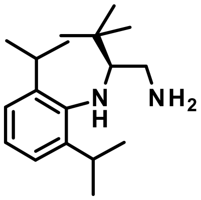

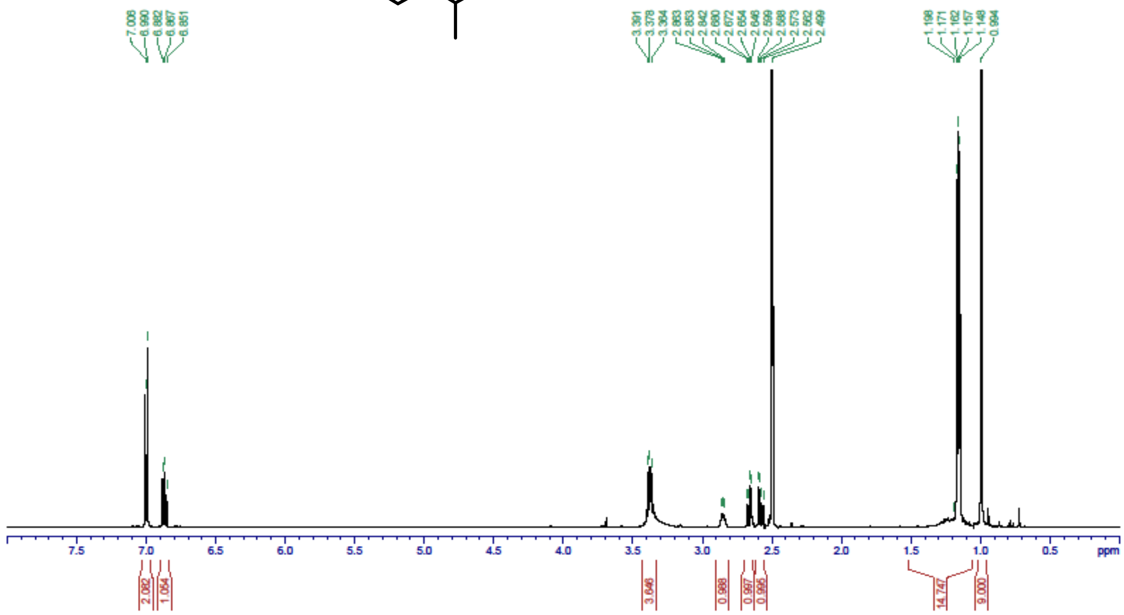

Figure S53. ^{1}H NMR (500 MHz) spectrum of **19a** in DMSO-d_6

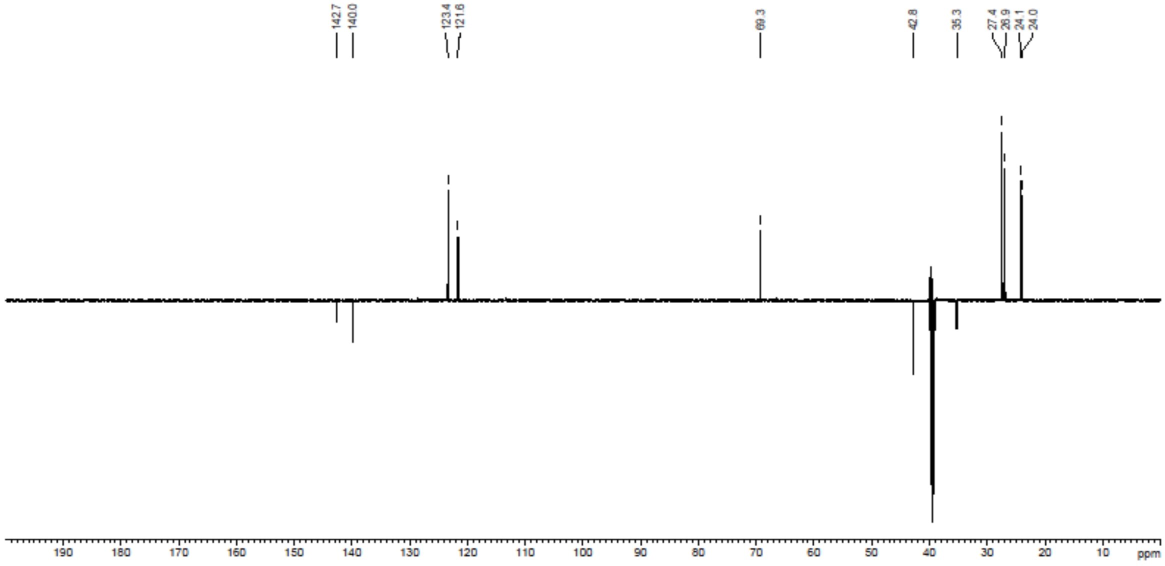

Figure S54. ^{13}C NMR (125 MHz) spectrum of **19a** in DMSO-d_6

(2*R*)-*N*[1]-[(1*R*)-2-(3-chlorophenyl)-1-phenylethyl]-*N*[2]-(2,6-di(propan-2-yl)phenyl)-3,3-dimethylbutane-1,2-diamine [(2*R*,1'*R*)-20a]

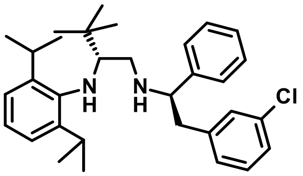

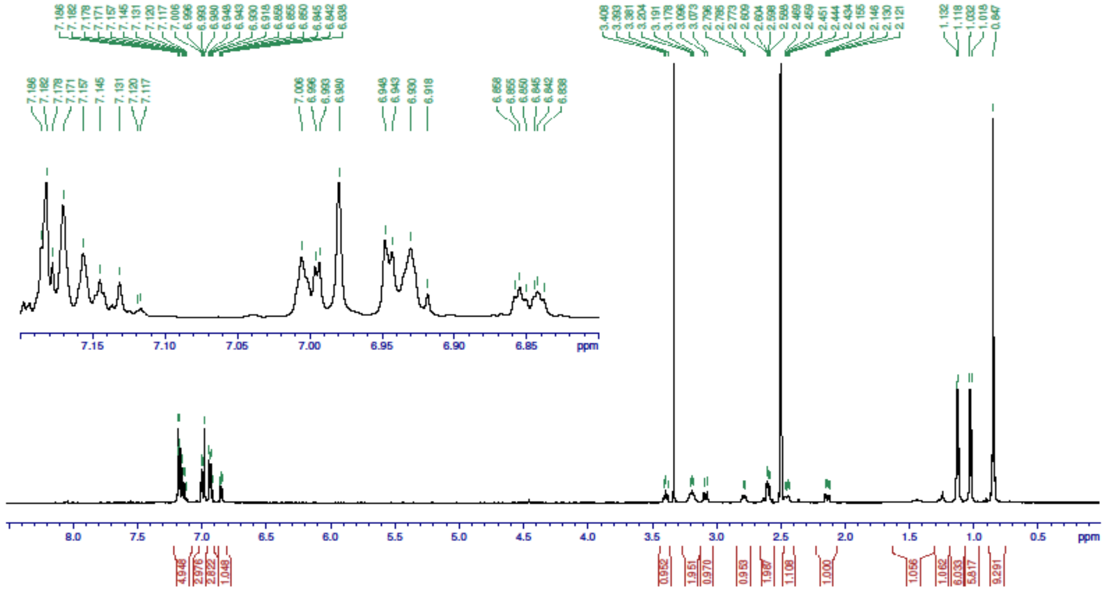

Figure S55. ^{1}H NMR (500 MHz) spectrum of **20a** in DMSO-d_6

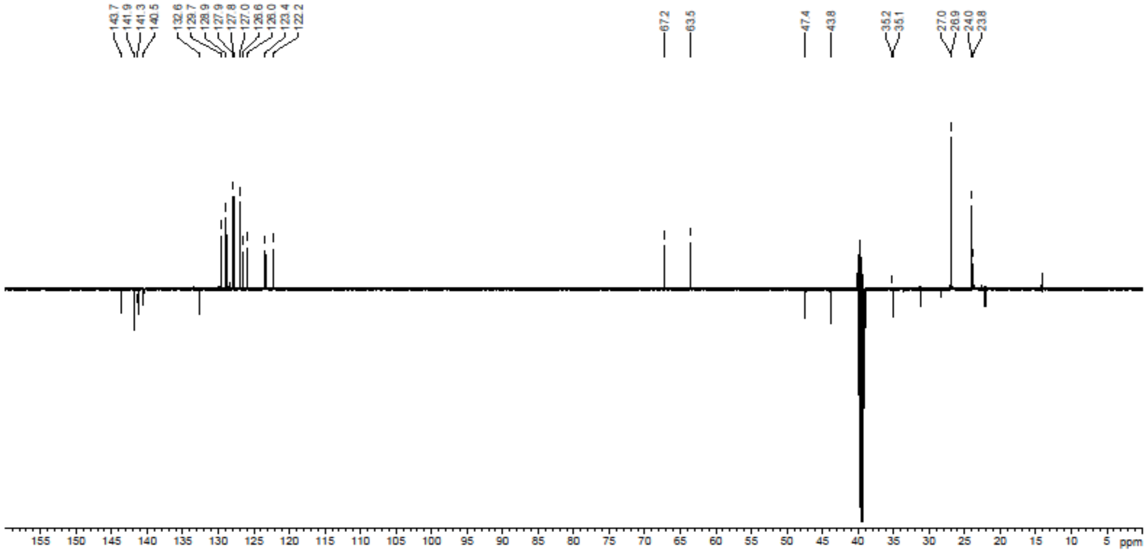

Figure S56. ^{13}C NMR (125 MHz) spectrum of **20a** in DMSO-d_6

(2*R*)-*N*[1]-[(1*S*)-2-(3-chlorophenyl)-1-phenylethyl]-*N*[2]-(2,6-di(propan-2-yl)phenyl)-3,3-dimethylbutane-1,2-diamine [(2*R*,1'*S*)-20b]

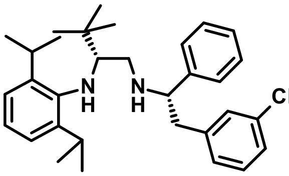

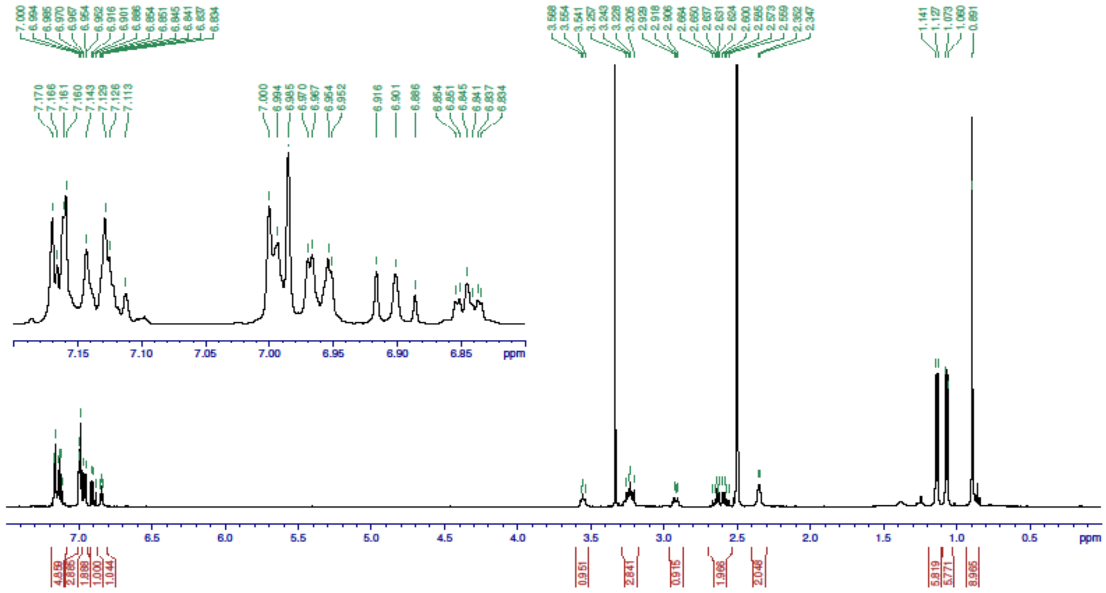

Figure S57. ^{1}H NMR (400 MHz) spectrum of **20b** in DMSO-d_6

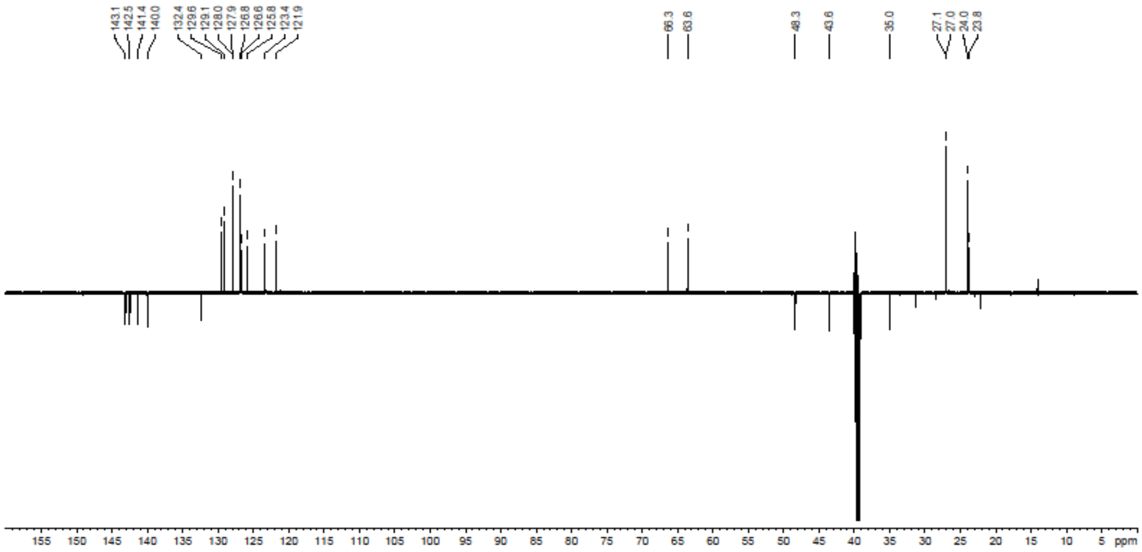

Figure S58. ^{13}C NMR (100 MHz) spectrum of **20b** in DMSO-d_6

(2*R*)-*N*²-(2,6-di(propan-2-yl)phenyl)-3,3-dimethyl-*N*¹-[(1'*R*)-2-phenyl-1-[3-(trifluoromethyl)phenyl]ethyl]butane-1,2-diamine [(2*R*,1'*R*)-20c]

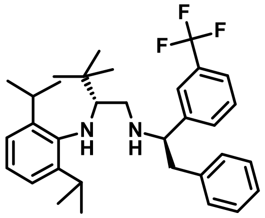

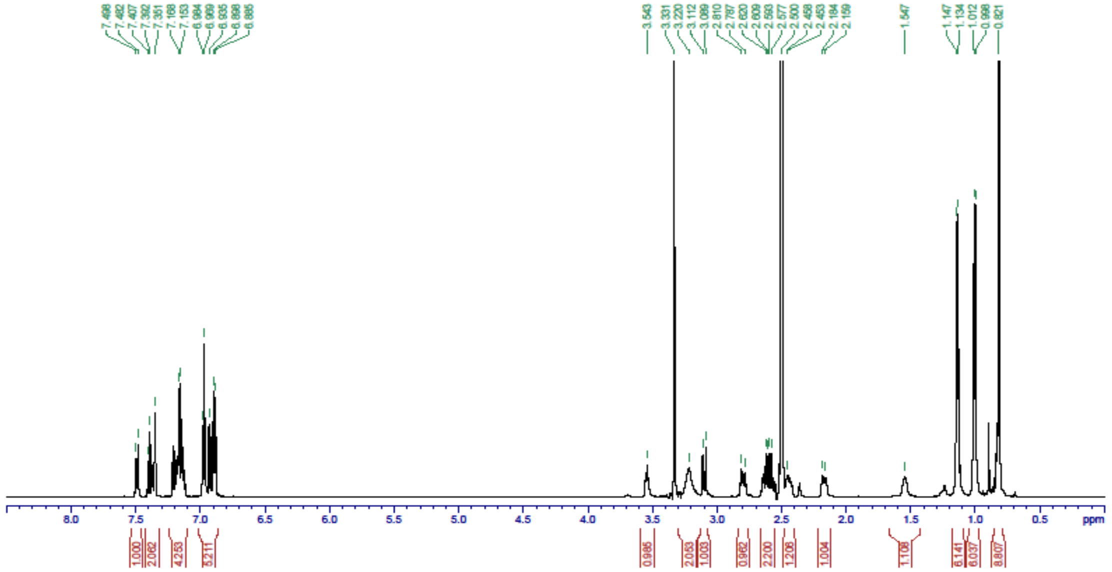

Figure S59. ^{1}H NMR (500 MHz) spectrum of **20c** in DMSO-d_6

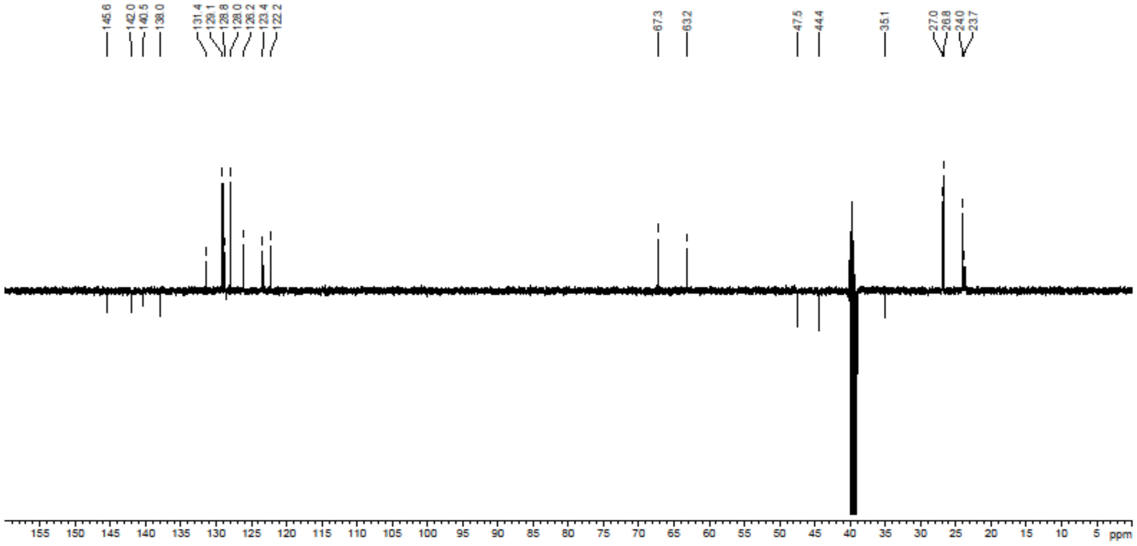

Figure S60. ^{13}C NMR (125 MHz) spectrum of **20c** in DMSO-d_6

(2*R*)-*N*²-(2,6-di(propan-2-yl)phenyl)-3,3-dimethyl-*N*¹-[(1'*S*)-2-phenyl-1-[3-(trifluoromethyl)phenyl]ethyl]butane-1,2-diamine [(2*R*,1'*S*)-20d]

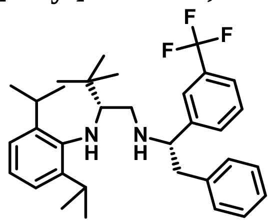

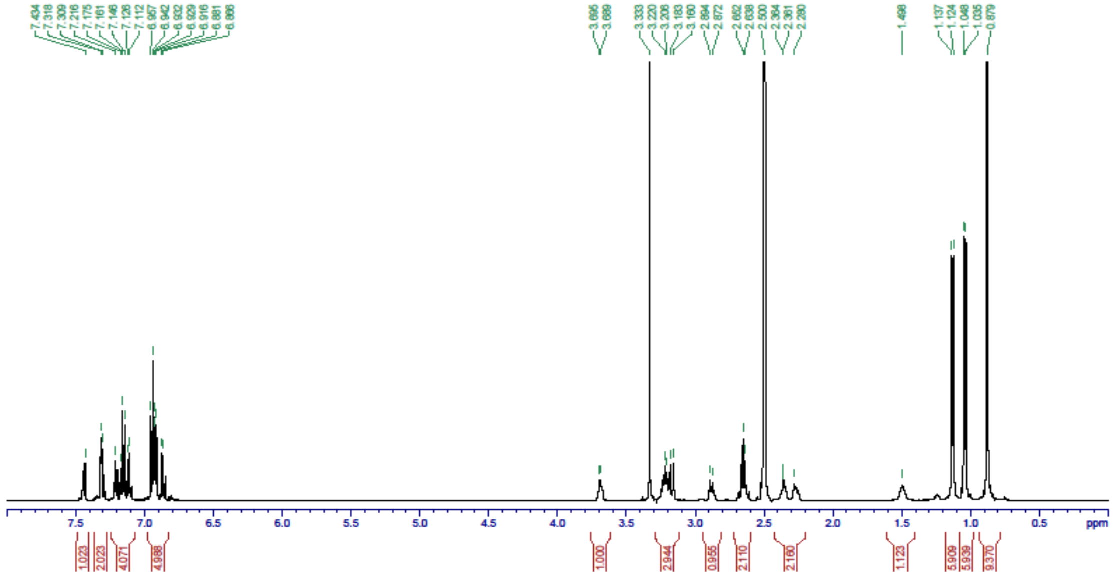

Figure S61. ^{1}H NMR (500 MHz) spectrum of **20d** in DMSO-d_6

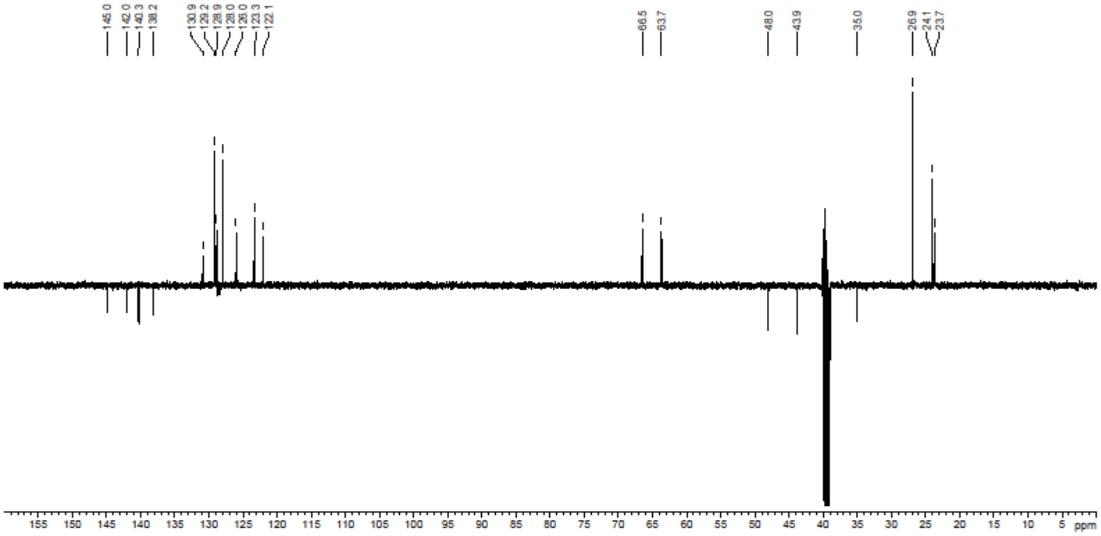

Figure S62. ^{13}C NMR (125 MHz) spectrum of **20d** in DMSO-d_6

(2*S*)-*N*²-(2,6-di(propan-2-yl)phenyl)-*N*¹-[(1'*S*)-1-(2-methoxyphenyl)-2-phenylethyl]-3,3-dimethylbutane-1,2-diamine [(2*S*,1'*S*)-20e]

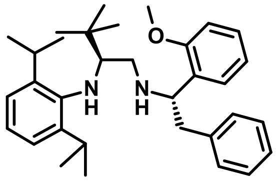

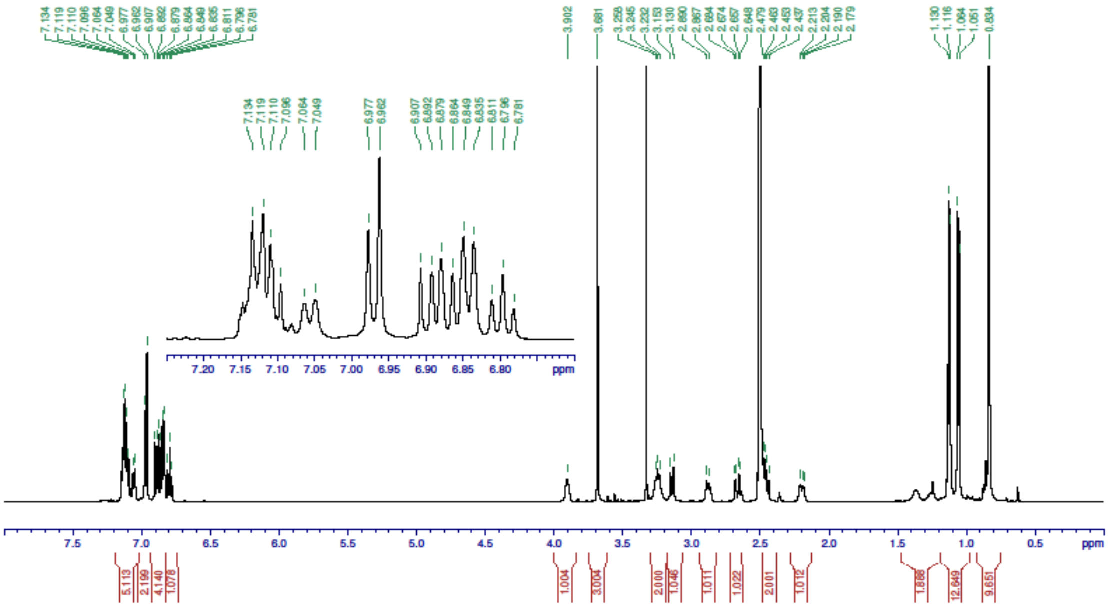

Figure S63. ^{1}H NMR (500 MHz) spectrum of **20e** in DMSO-d_6

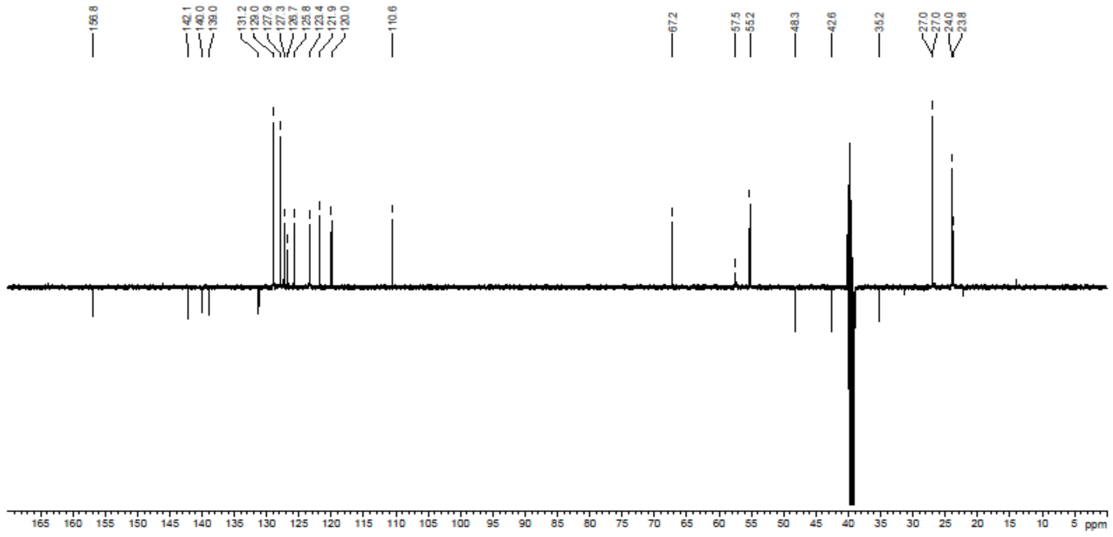

Figure S64. ^{13}C NMR (125 MHz) spectrum of **20e** in DMSO-d_6

(2*S*)-*N*²-(2,6-di(propan-2-yl)phenyl)-*N*¹-[(1'*R*)-1-(2-methoxyphenyl)-2-phenylethyl]-3,3-dimethylbutane-1,2-diamine [(2*S*,1'*R*)-20f]

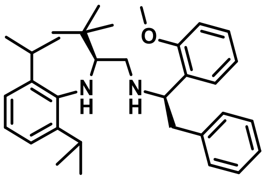

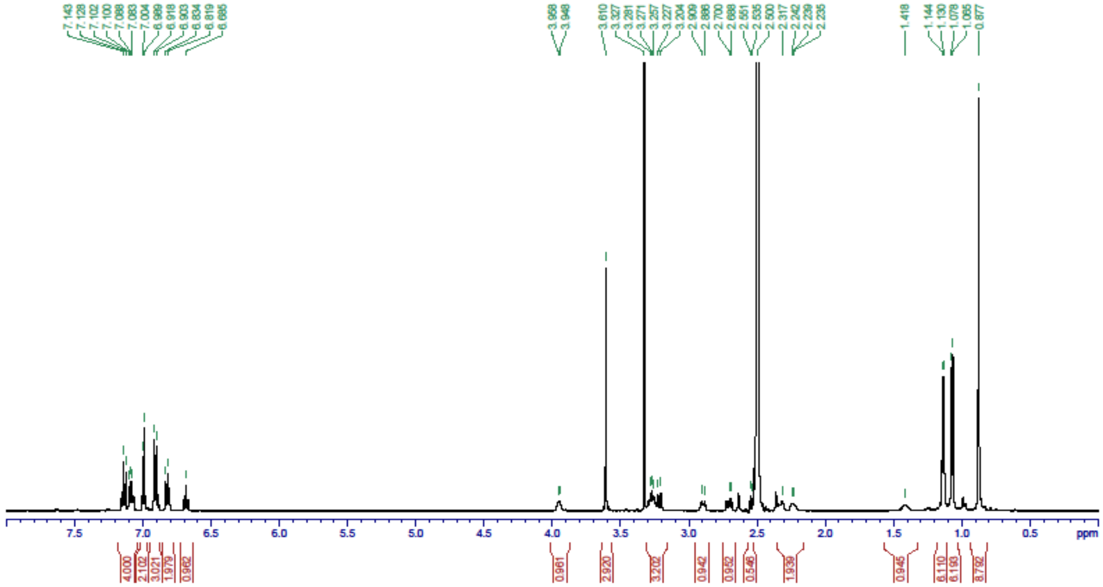

Figure S65. ^{1}H NMR (500 MHz) spectrum of **20f** in DMSO-d_6

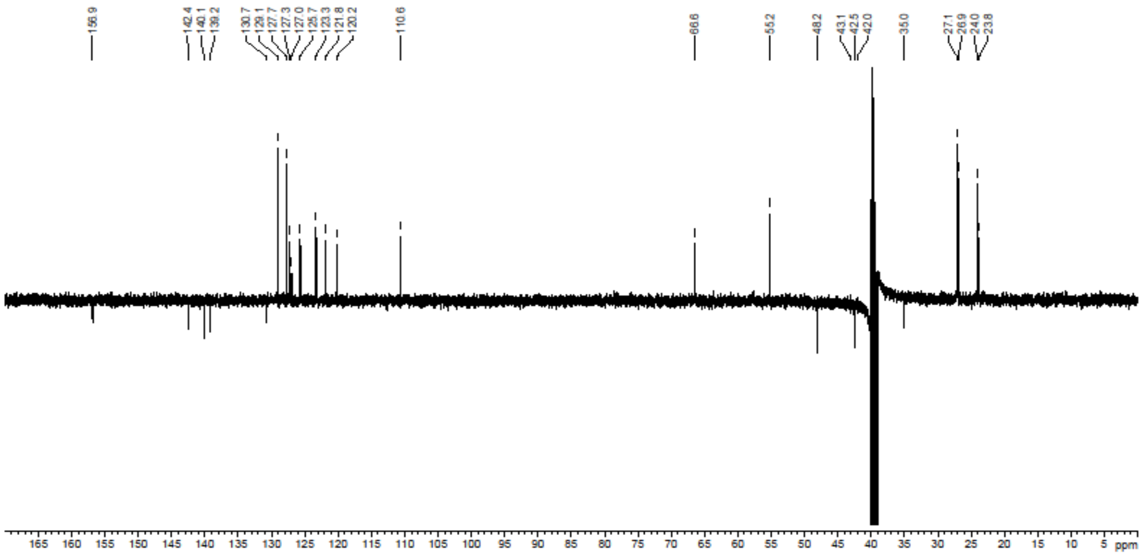

Figure S66. ^{13}C NMR (125 MHz) spectrum of **20f** in DMSO-d_6

(2*S*)-*N*²-(2,6-di(propan-2-yl)phenyl)-*N*¹-[(1'*S*)-1-(3-methoxyphenyl)-2-phenylethyl]-3,3-dimethylbutane-1,2-diamine [(2*S*,1'*S*)-20g]

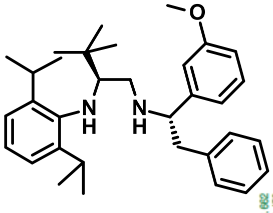

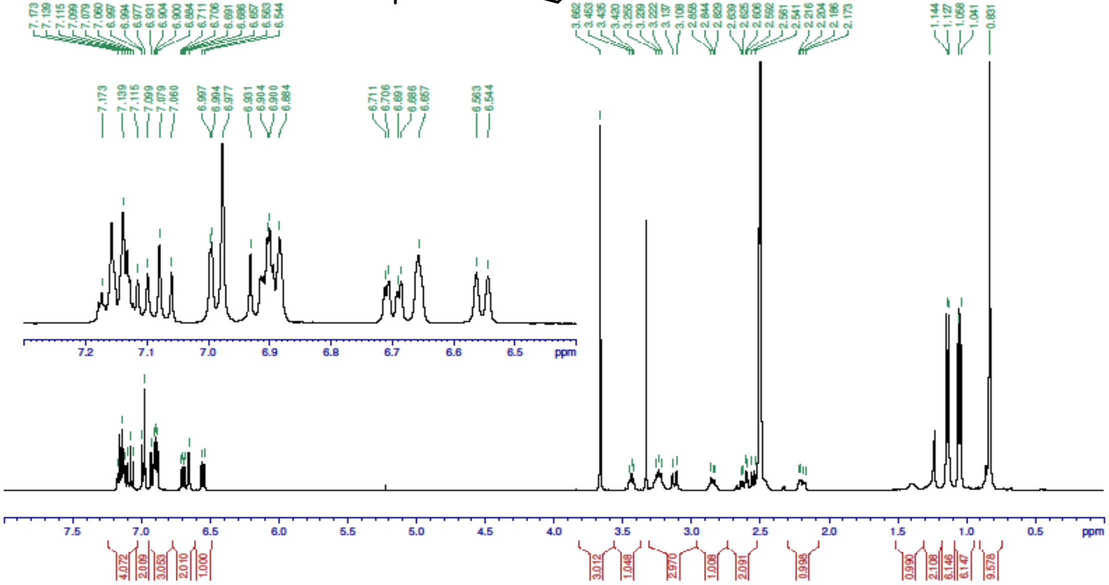

Figure S67. ^{1}H NMR (400 MHz) spectrum of **20g** in DMSO-d_6

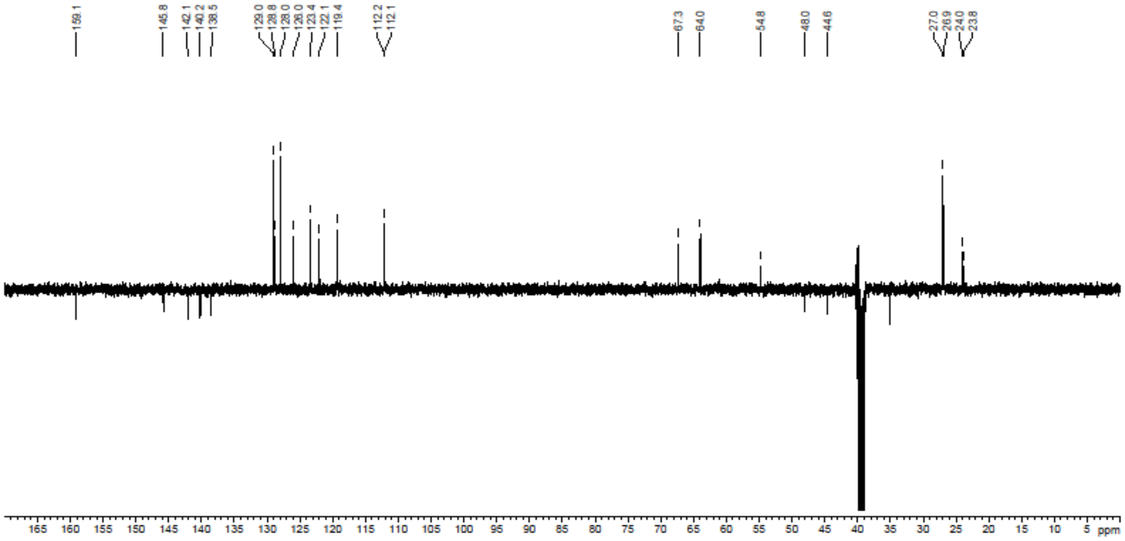

Figure S68. ^{13}C NMR (100 MHz) spectrum of **20g** in DMSO-d_6

(2*S*)-*N*^{2}-(2,6-di(propan-2-yl)phenyl)-*N*^{1}-[(1'*R*)-1-(3-methoxyphenyl)-2-phenylethyl]-3,3-dimethylbutane-1,2-diamine [(2*S*,1'*R*)-20h]

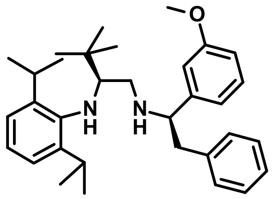

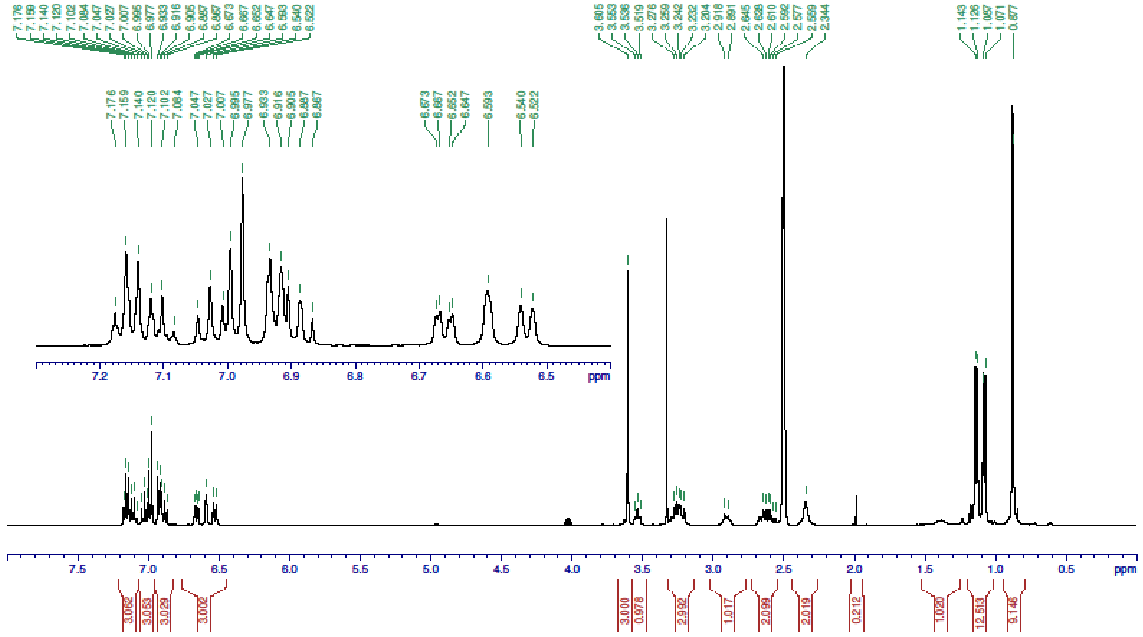

Figure S69. ^{1}H NMR (400 MHz) spectrum of **20h** in DMSO-d_6

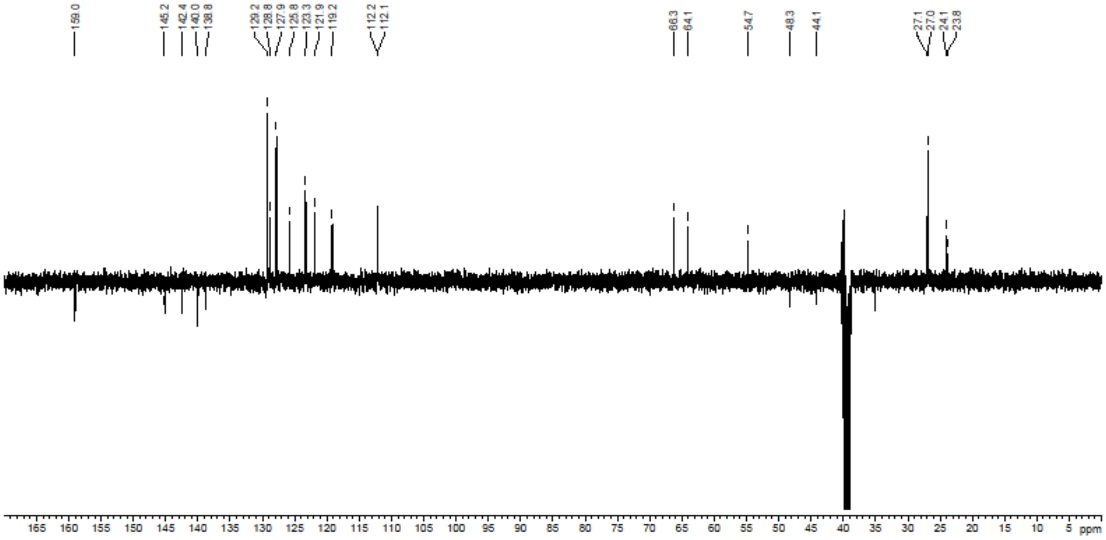

Figure S70. ^{13}C NMR (100 MHz) spectrum of **20h** in DMSO-d_6

(2*S*)-*N*[2]-(2,6-di(propan-2-yl)phenyl)-*N*[1]-[(1’*R*)-1-(4-methoxyphenyl)-2-phenylethyl]-3,3-dimethylbutane-1,2-diamine [(2*S*,1’*R*)-20i]

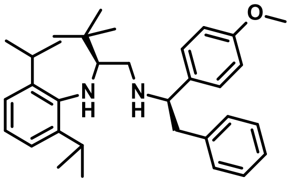

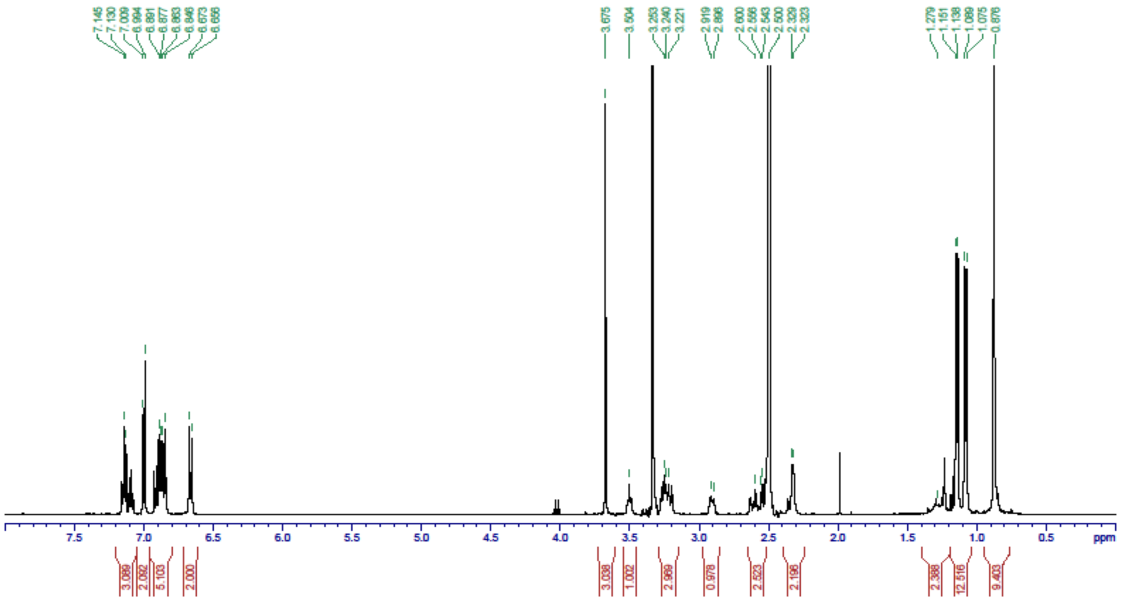

Figure S71. ^{1}H NMR (500 MHz) spectrum of **20i** in DMSO-d_6

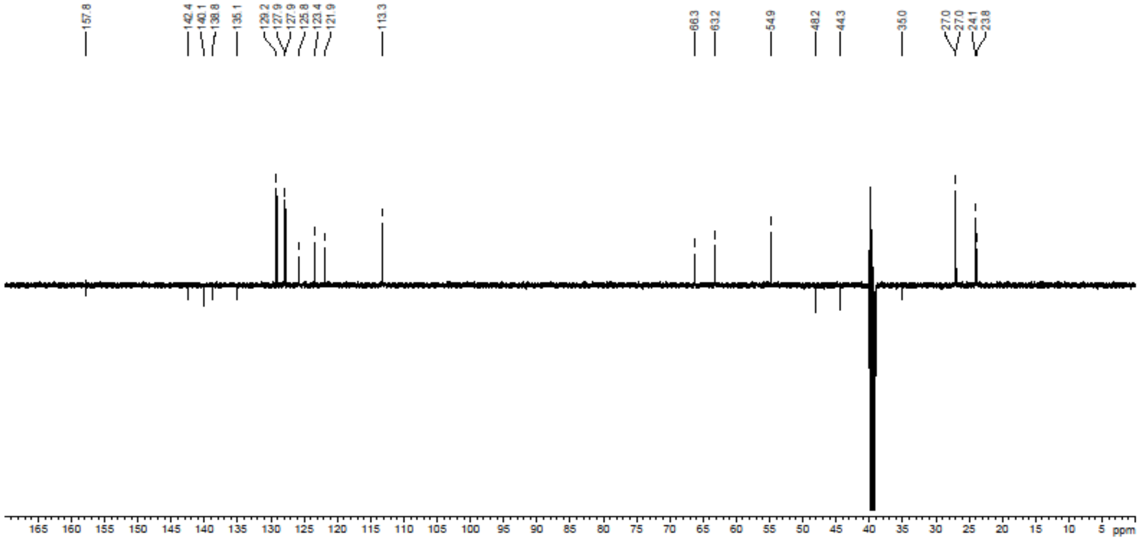

Figure S72. ^{13}C NMR (125 MHz) spectrum of **20i** in DMSO-d_6

(2*S*)-*N*^{2}-(2,6-di(propan-2-yl)phenyl)-*N*^{1}-[(1'*S*)-1-(4-methoxyphenyl)-2-phenylethyl]-3,3-dimethylbutane-1,2-diamine [(2*S*,1'*S*)-20j]

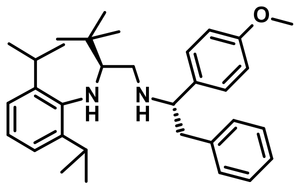

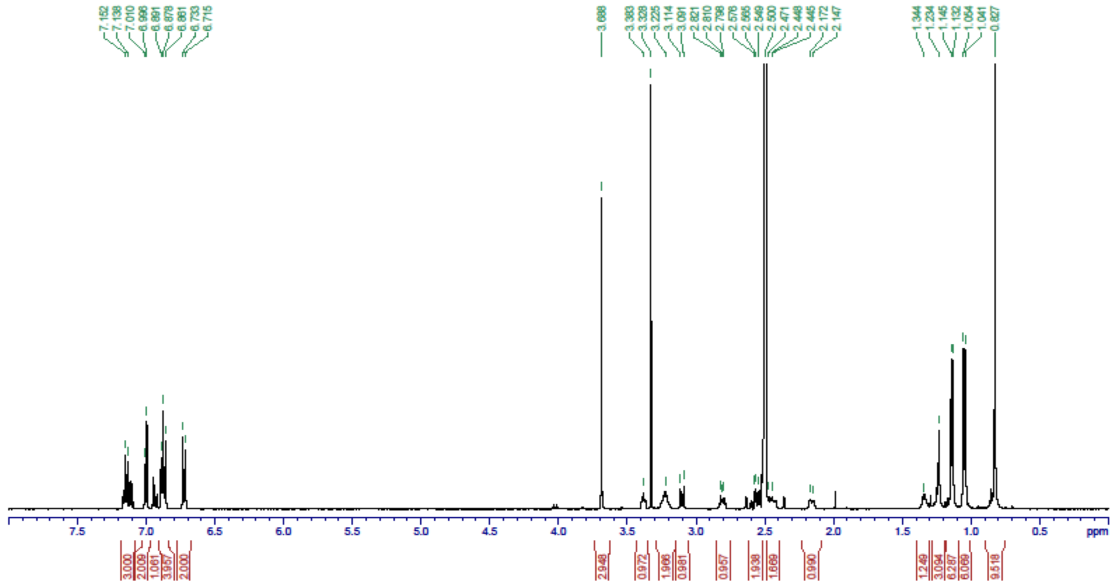

Figure S73. ^{1}H NMR (500 MHz) spectrum of **20j** in DMSO-d_6

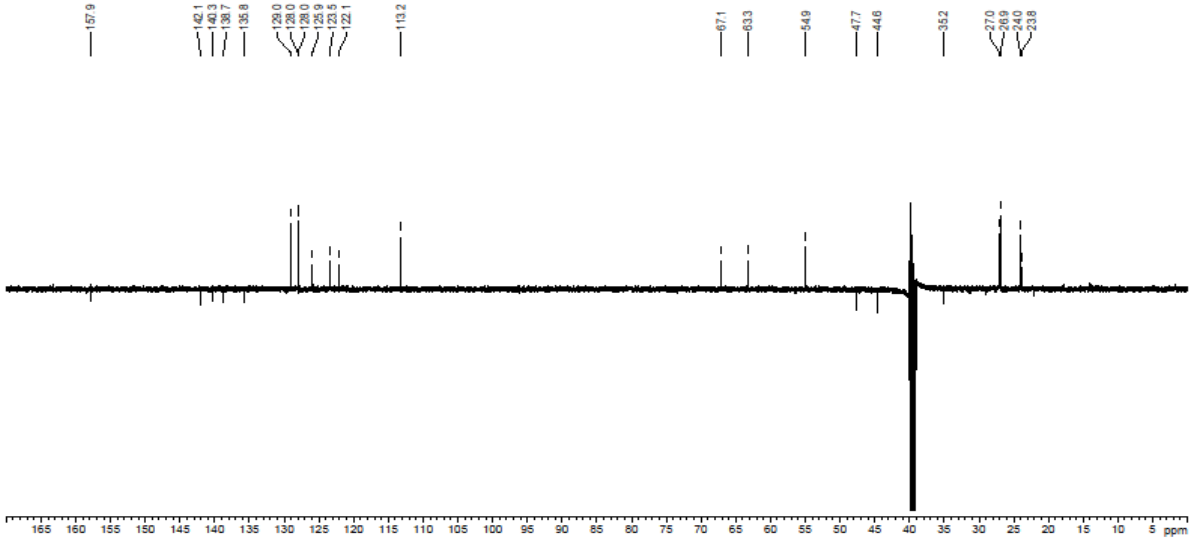

Figure S74. ^{13}C NMR (125 MHz) spectrum of **20j** in DMSO-d_6

(2*S*)-*N*[2]-(2,6-di(propan-2-yl)phenyl)-3,3-dimethyl-*N*[1]-[(1'*R*)-1-(o-tolyl)-2-phenylethyl]butane-1,2-diamine [(2*S*,1'*R*)-20l]

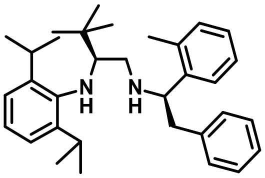

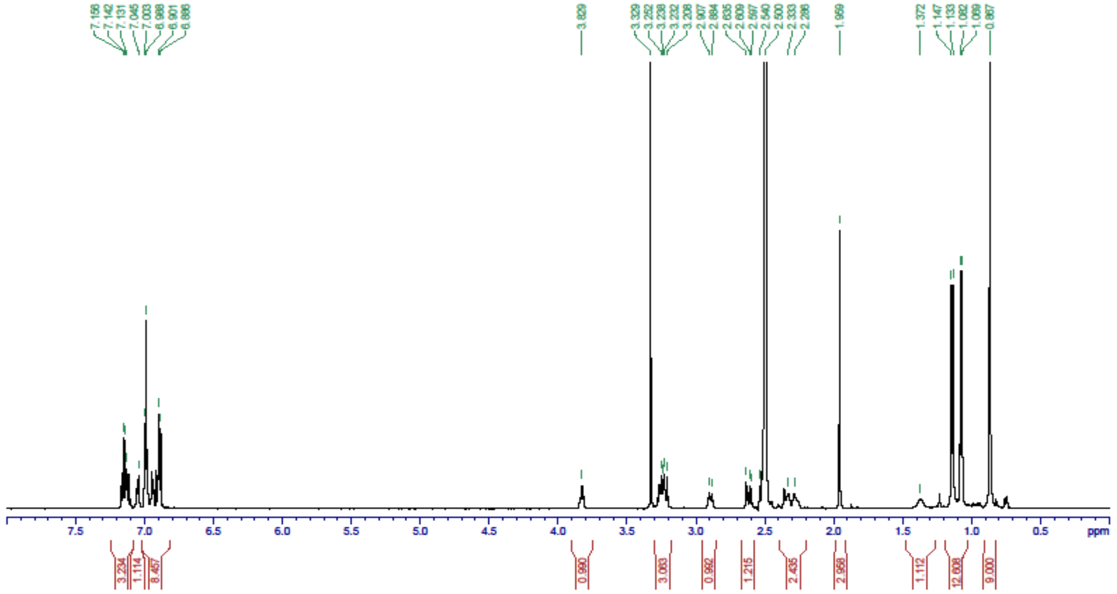

Figure S75. ^{1}H NMR (500 MHz) spectrum of **20l** in DMSO-d_6

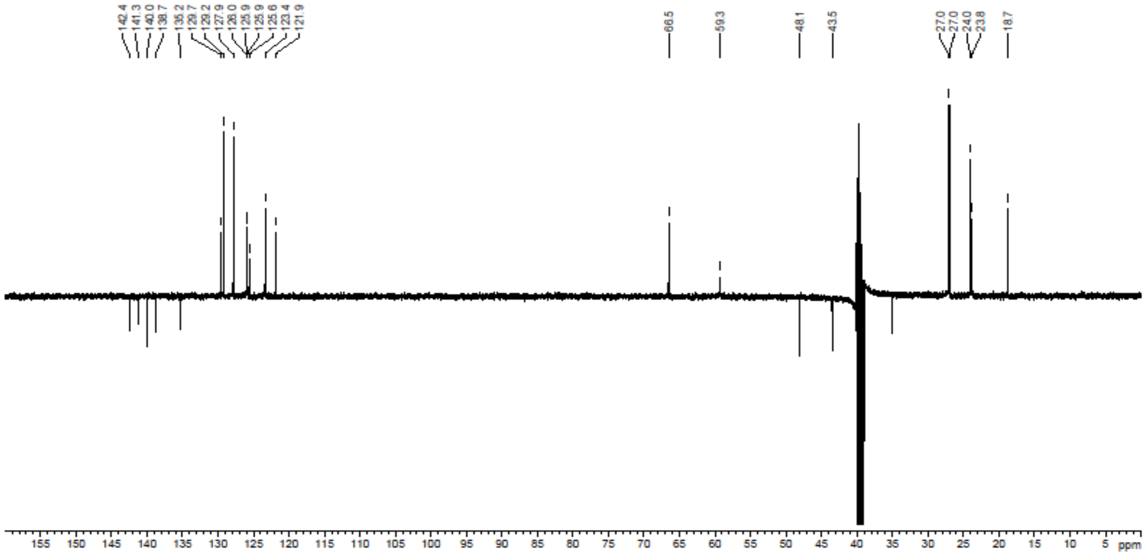

Figure S76. ^{13}C NMR (125 MHz) spectrum of **20l** in DMSO-d_6

(2*S*)-*N*^{2}-(2,6-di(propan-2-yl)phenyl)-3,3-dimethyl-*N*^{1}-[(1’*R*)-2-phenyl-1-(p-tolyl)ethyl]butane-1,2-diamine [(2*S*,1’*R*)-20m]

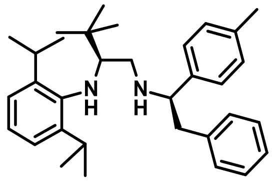

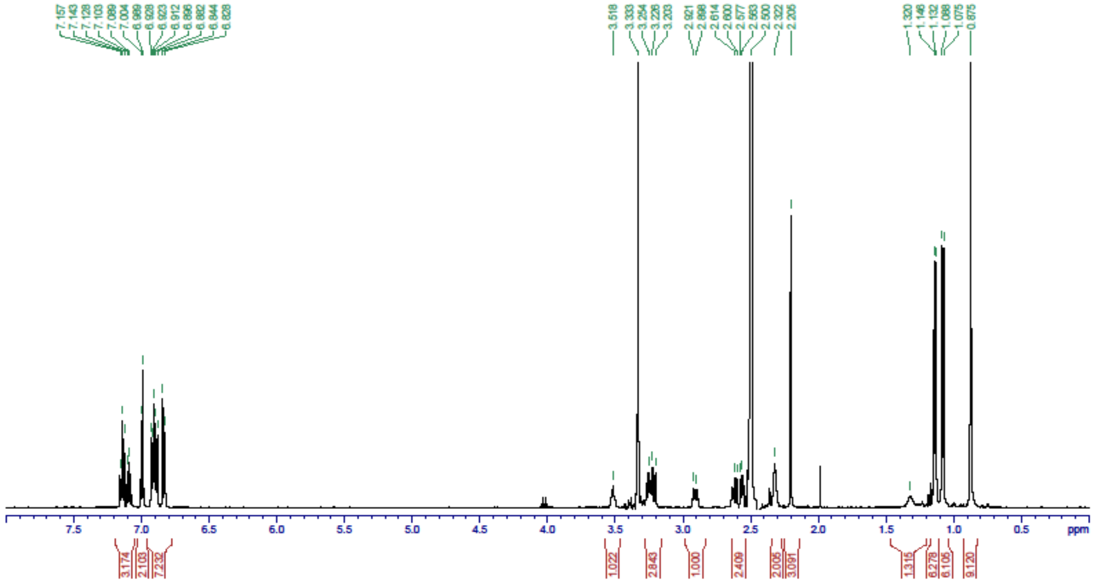

Figure S77. ^{1}H NMR (500 MHz) spectrum of **20m** in DMSO-*d*$_6$

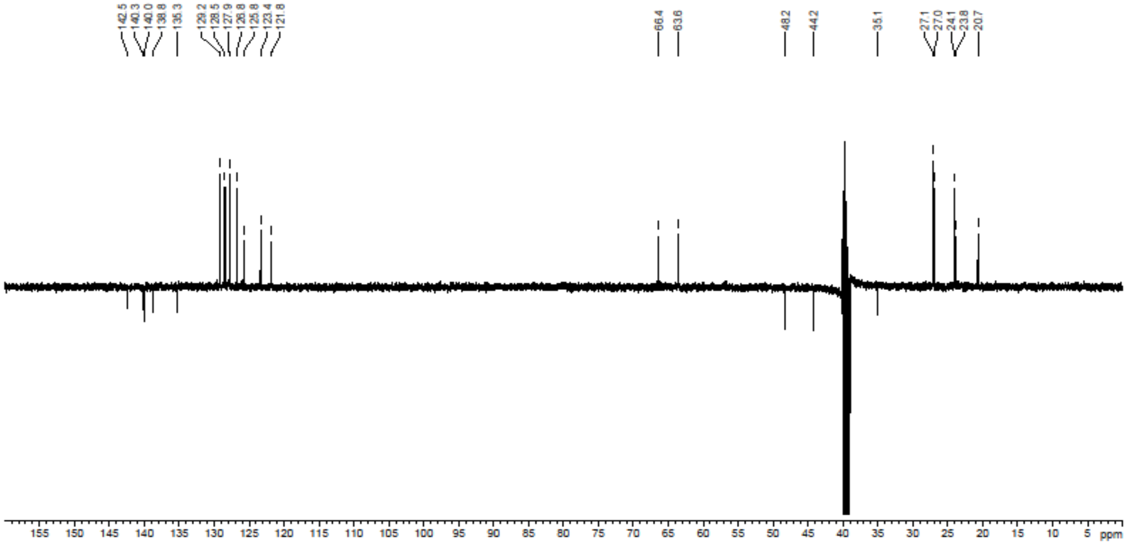

Figure S78. ^{13}C NMR (125 MHz) spectrum of **20m** in DMSO-*d*$_6$

(2*S*)-*N*²-(2,6-di(propan-2-yl)phenyl)-3,3-dimethyl-*N*¹-[(1'*S*)-2-phenyl-1-(p-tolyl)ethyl]butane-1,2-diamine [(2*S*,1'*S*)-20n]

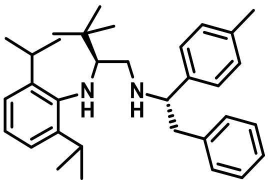

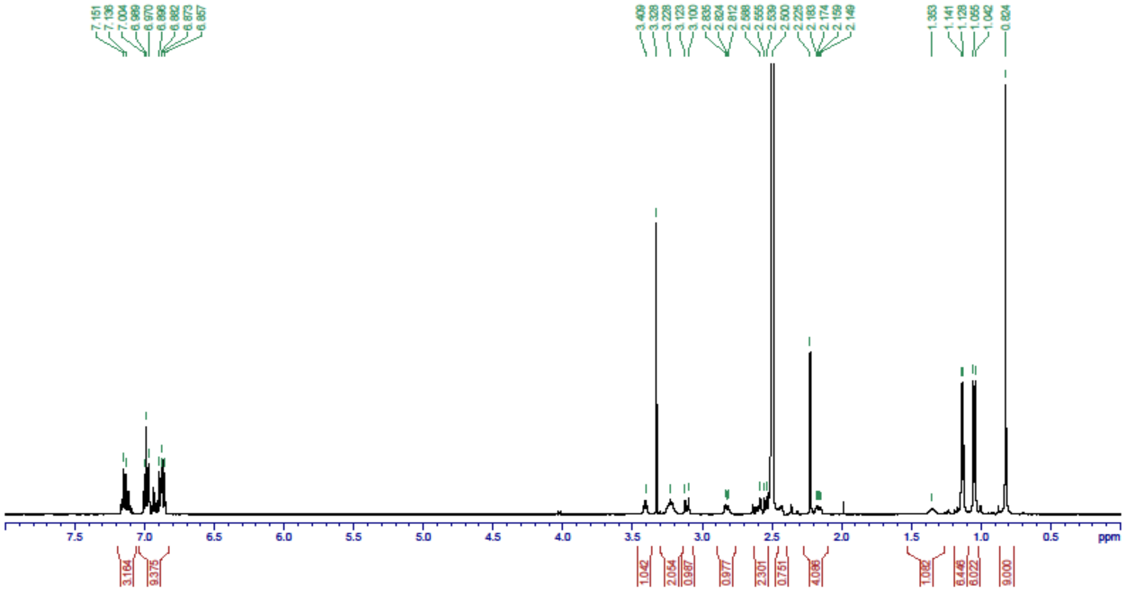

Figure S79. ^{1}H NMR (500 MHz) spectrum of **20n** in DMSO-d_6

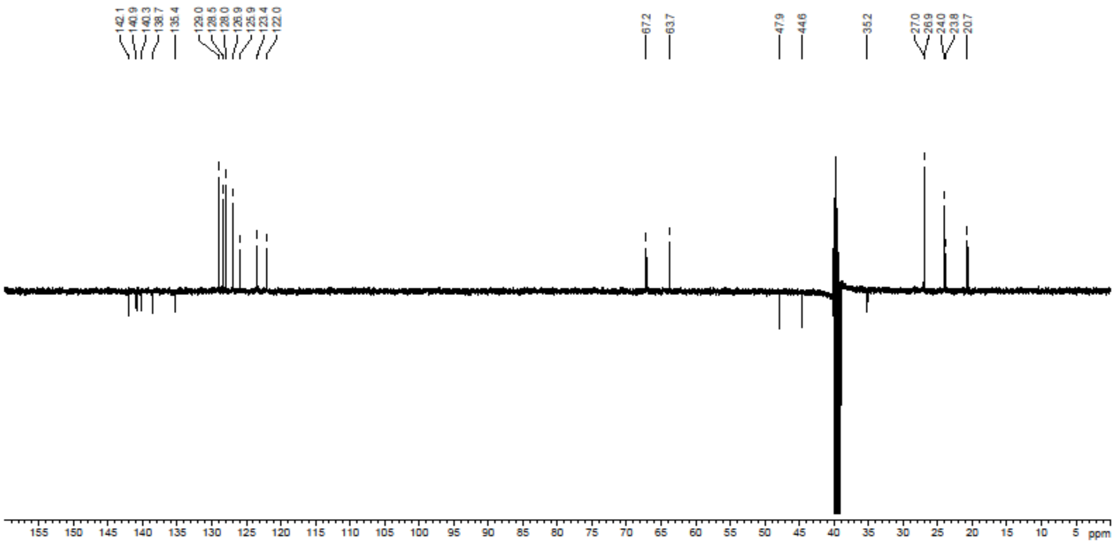

Figure S80. ^{13}C NMR (125 MHz) spectrum of **20n** in DMSO-d_6

(2*S*)-*N*[2]-(2,6-di(propan-2-yl)phenyl)-3,3-dimethyl-*N*[1]-[(1'*R*)-2-phenyl-1-[4-(trifluoromethyl)phenyl]ethyl]butane-1,2-diamine [(2*S*,1'*R*)-20o]

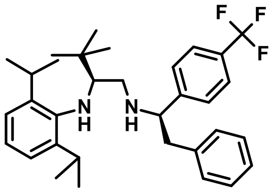

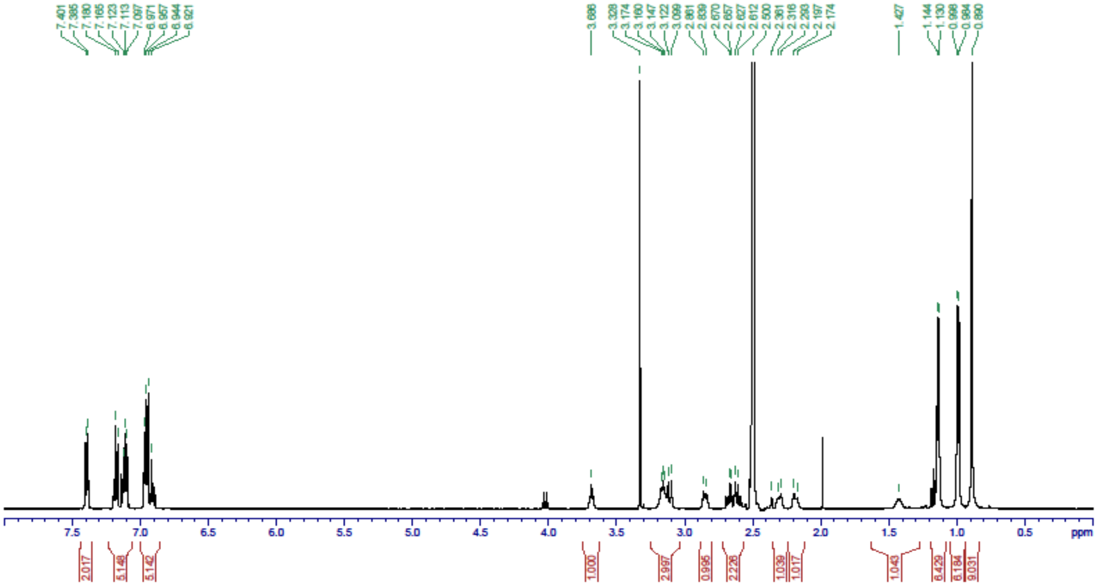

Figure S81. ^{1}H NMR (500 MHz) spectrum of **20o** in DMSO-d_6

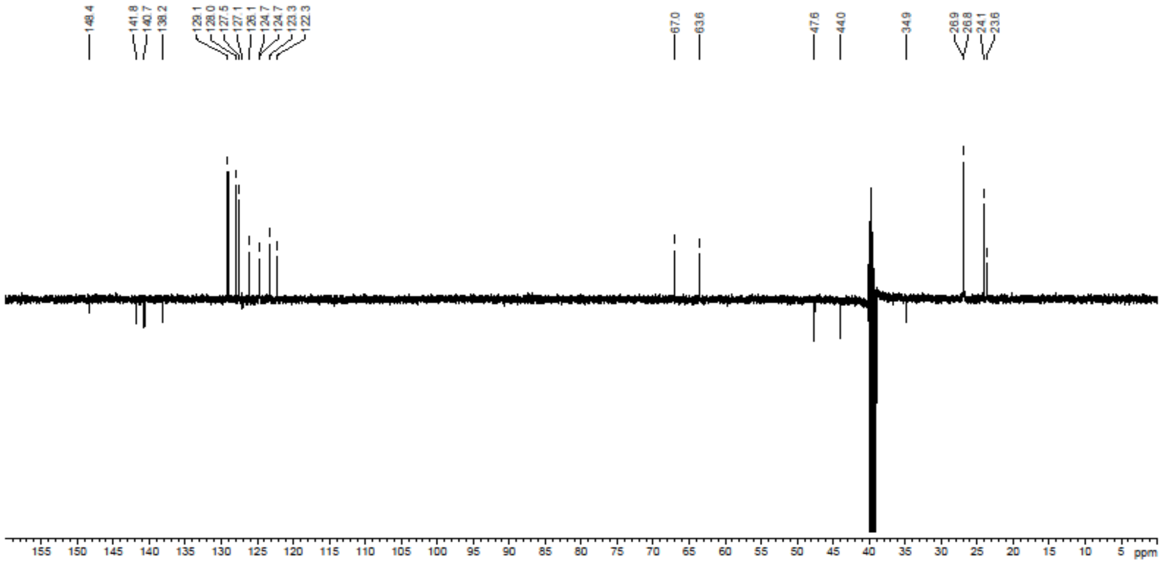

Figure S82. ^{13}C NMR (125 MHz) spectrum of **20o** in DMSO-d_6

(2*S*)-*N*^{2}-(2,6-di(propan-2-yl)phenyl)-3,3-dimethyl-*N*^{1}-[(1'*S*)-2-phenyl-1-[4-(trifluoromethyl)phenyl]ethyl]butane-1,2-diamine [(2*S*,1'*S*)-20p]

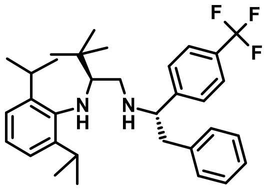

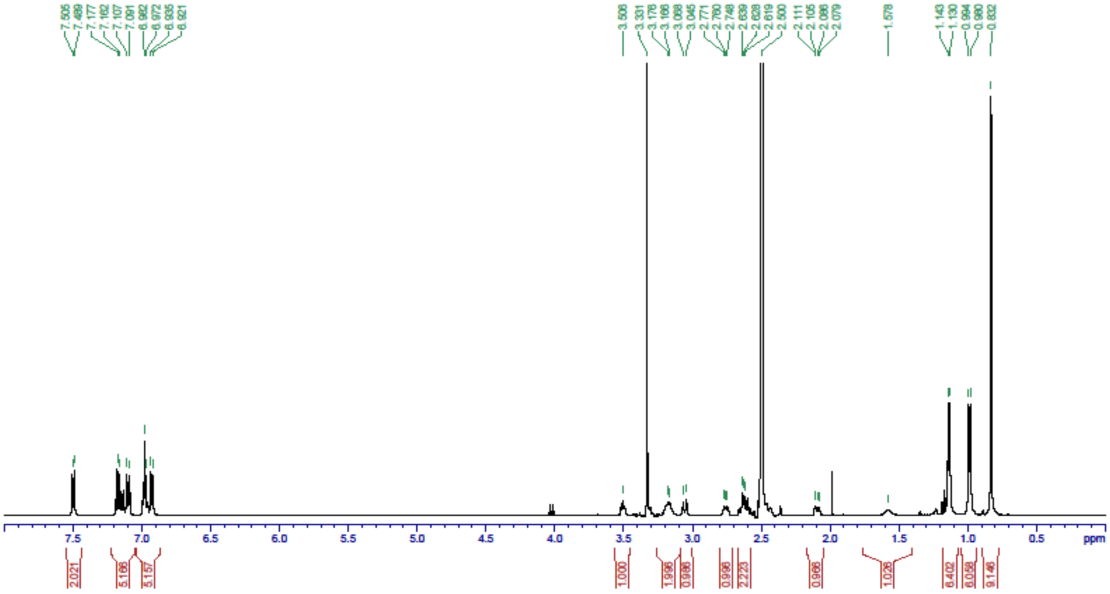

Figure S83. ^{1}H NMR (500 MHz) spectrum of **20p** in DMSO-d_6

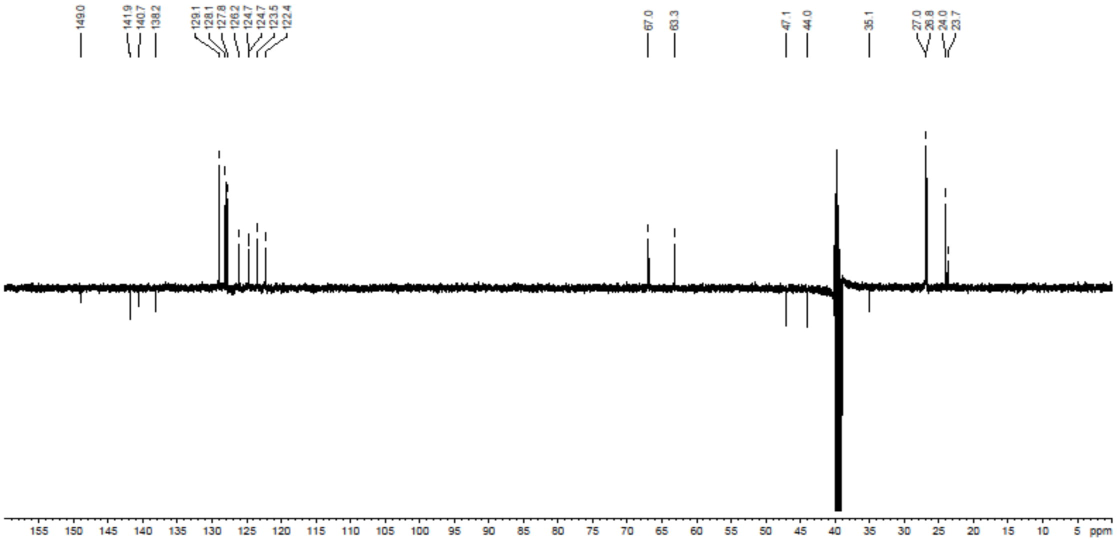

Figure S84. ^{13}C NMR (125 MHz) spectrum of **20p** in DMSO-d_6

(2*S*)-*N*²-(2,6-di(propan-2-yl)phenyl)-3,3-dimethyl-*N*¹-[(1'*S*)-1-phenyl-2-(p-tolyl)ethyl]butane-1,2-diamine [(2*S*,1'*S*)-20q]

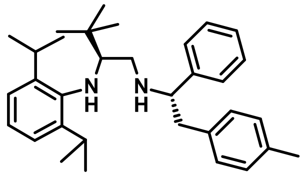

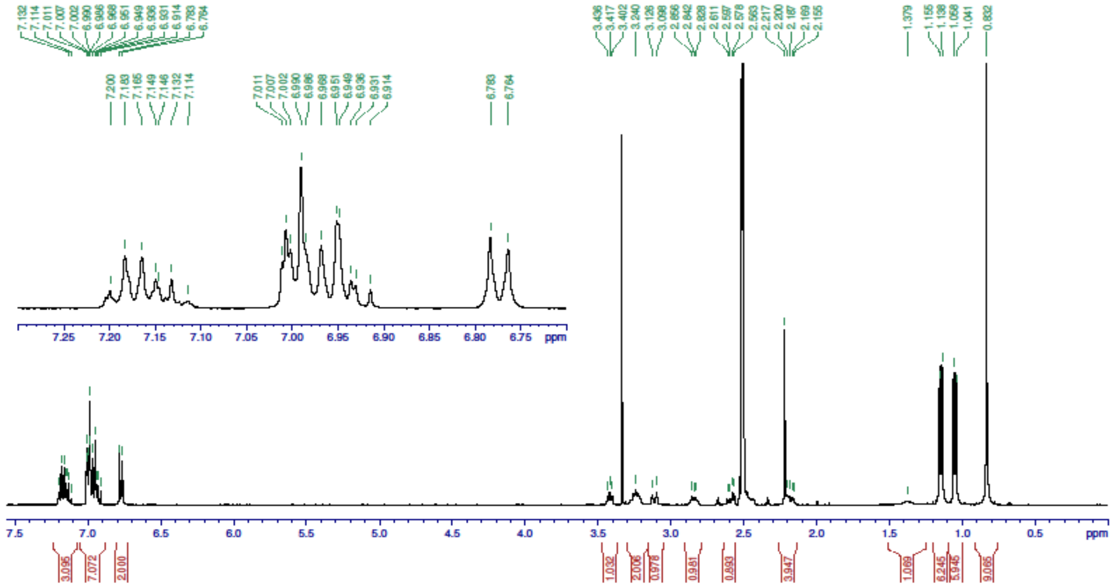

Figure S85. ^{1}H NMR (500 MHz) spectrum of **20q** in DMSO-d_6

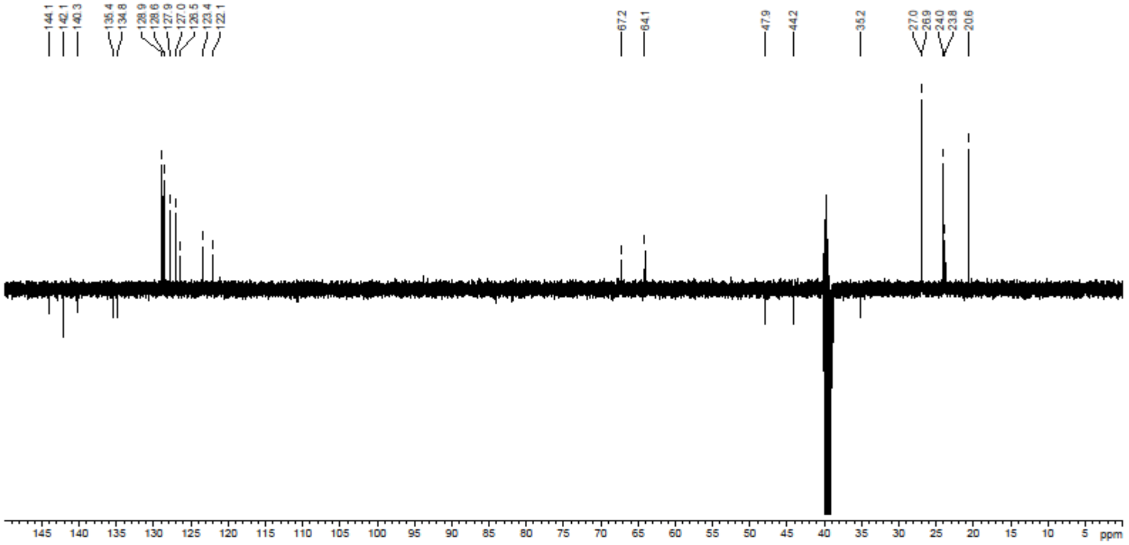

Figure S86. ^{13}C NMR (125 MHz) spectrum of **20q** in DMSO-d_6

(2*S*)-*N*²-(2,6-di(propan-2-yl)phenyl)-3,3-dimethyl-*N*¹-[(1'*R*)-1-phenyl-2-(p-tolyl)ethyl]butane-1,2-diamine [(2*S*,1'*R*)-20r]

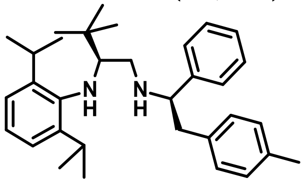

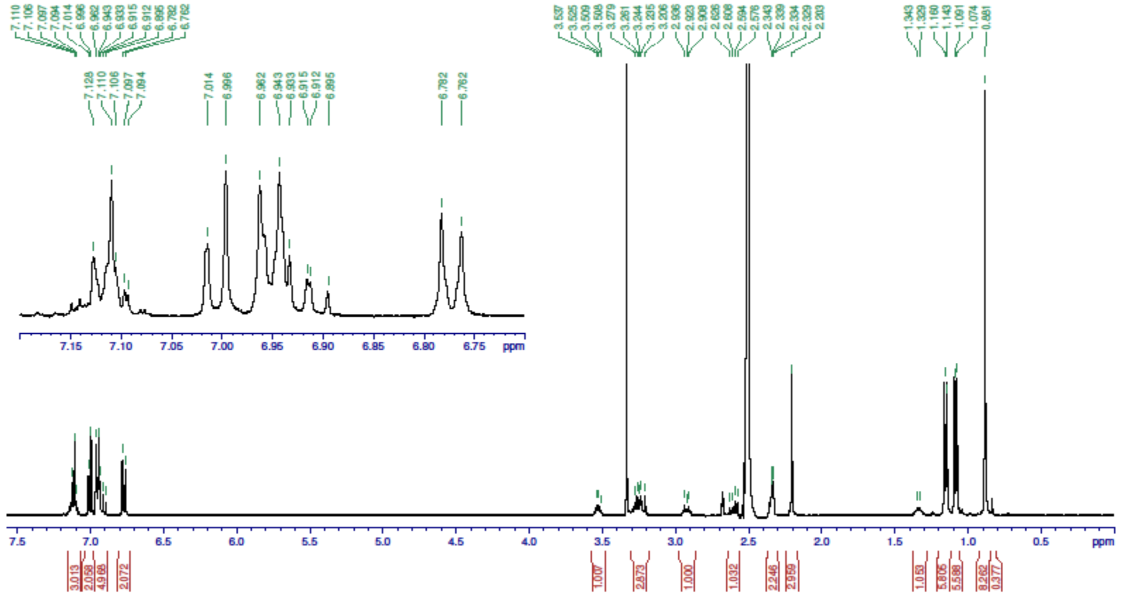

Figure S87. ¹H NMR (400 MHz) spectrum of **20r** in DMSO-d_6

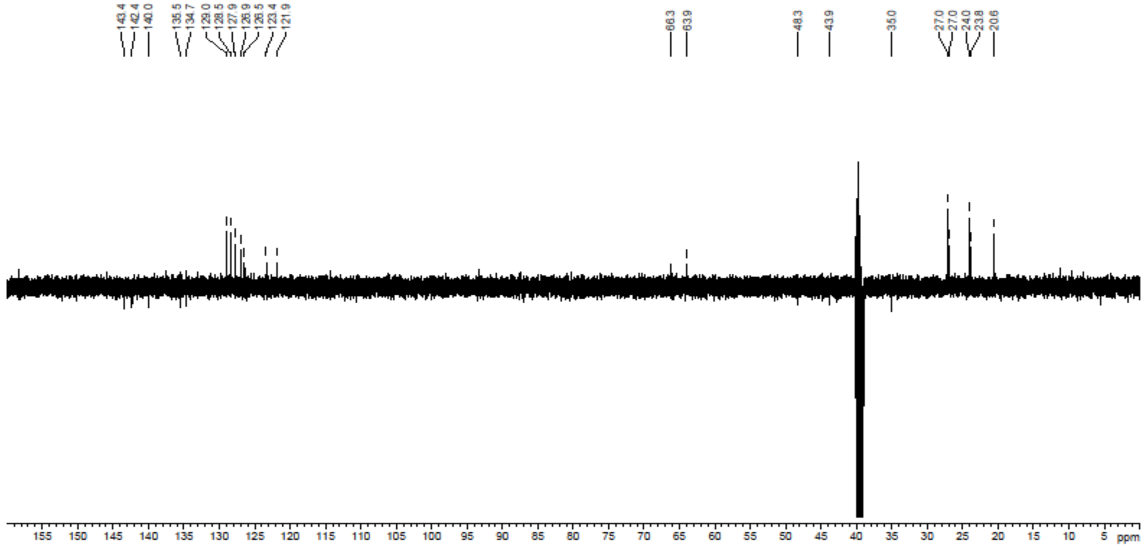

Figure S88. ¹³C NMR (100 MHz) spectrum of **20r** in DMSO-d_6

(2*S*)-*N*[1]-[(1'*S*)-2-(4-chlorophenyl)-1-phenylethyl]-*N*[2]-(2,6-di(propan-2-yl)phenyl)-3,3-dimethylbutane-1,2-diamine [(2*S*,1'*S*)-20s]

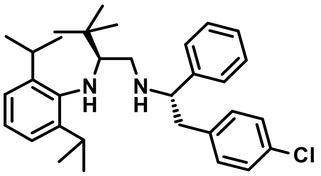

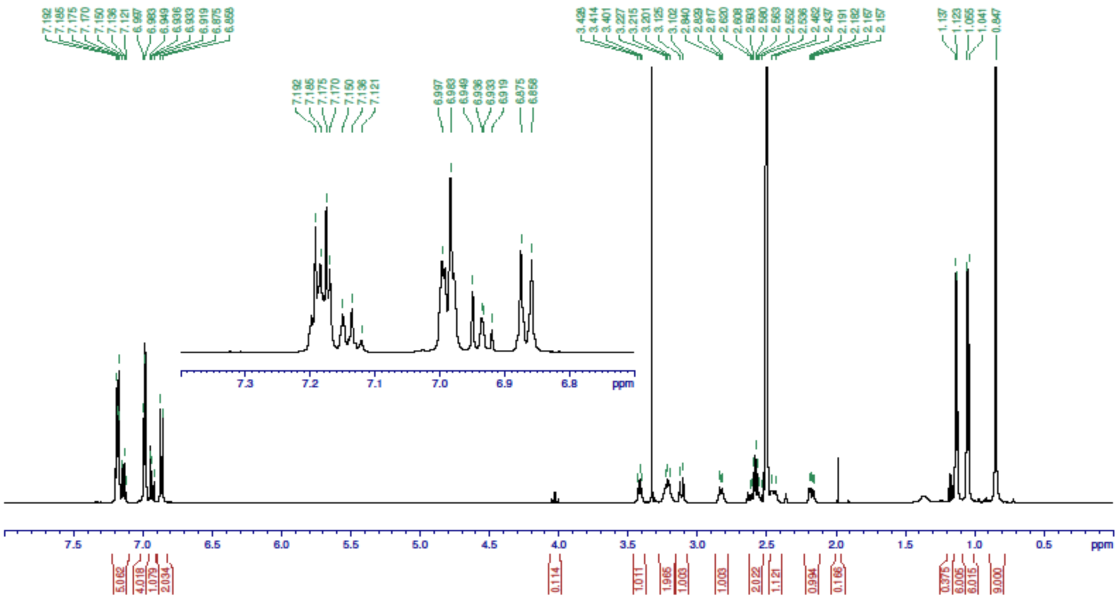

Figure S89. ^{1}H NMR (500 MHz) spectrum of **20s** in DMSO-d_6

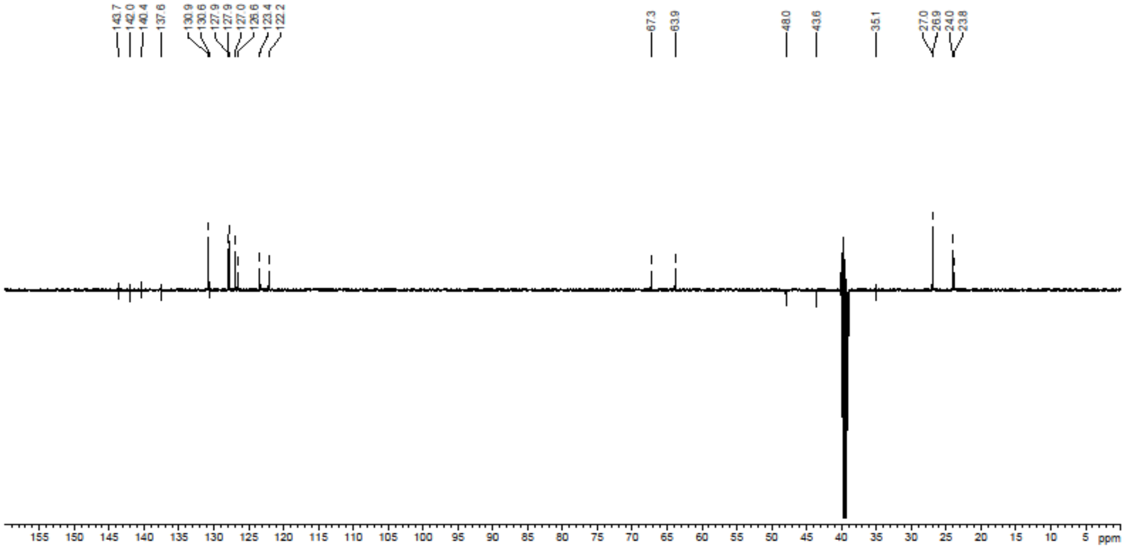

Figure S90. ^{13}C NMR (125 MHz) spectrum of **20s** in DMSO-d_6

(2*S*)-*N*[1]-[(1'*R*)-2-(4-chlorophenyl)-1-phenylethyl]-*N*[2]-(2,6-di(propan-2-yl)phenyl)-3,3-dimethylbutane-1,2-diamine [(2*S*,1'*R*)-20t]

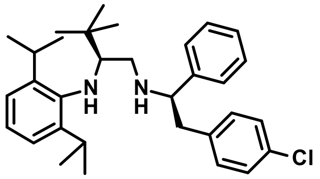

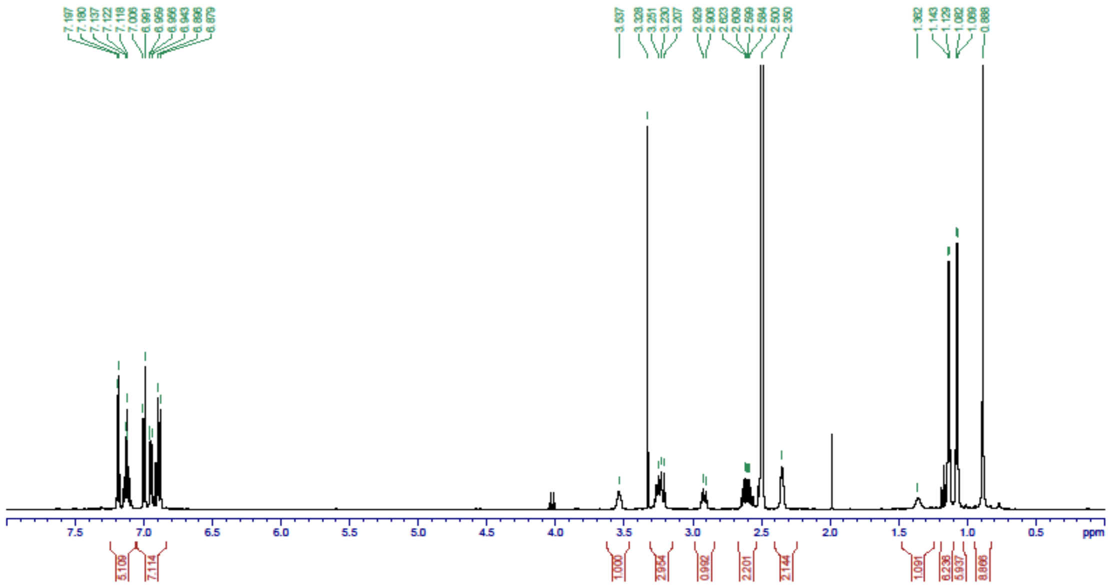

Figure S91. ^{1}H NMR (500 MHz) spectrum of **20t** in DMSO-d_6

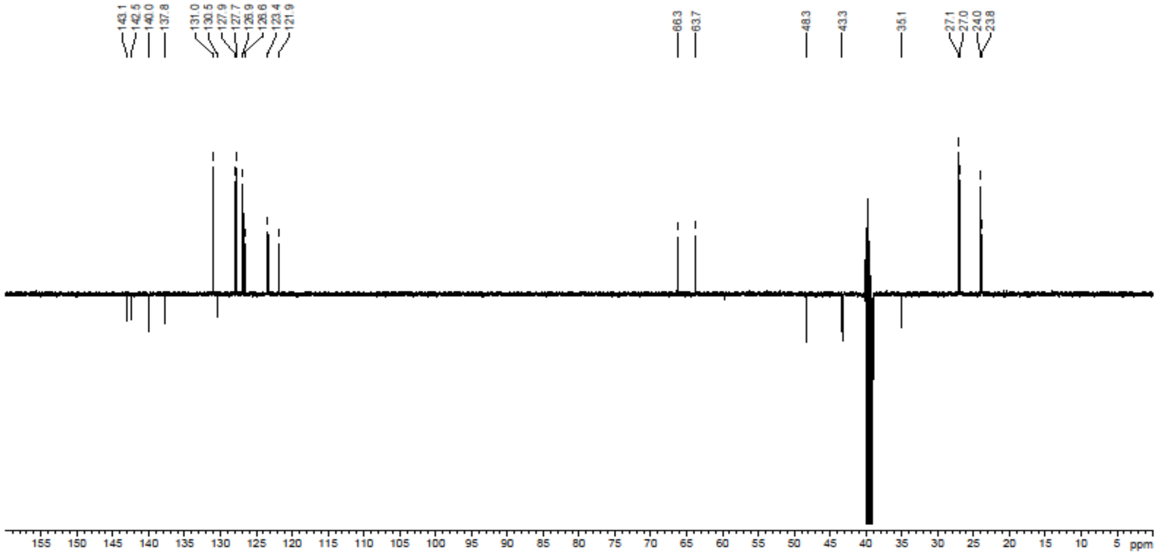

Figure S92. ^{13}C NMR (125 MHz) spectrum of **20t** in DMSO-d_6

(2*S*)-*N*[1]-[(1’*S*)-2-(2-chlorophenyl)-1-phenylethyl]-*N*[2]-(2,6-di(propan-2-yl)phenyl)-3,3-dimethylbutane-1,2-diamine [(2*S*,1’*S*)-20u]

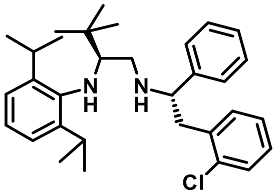

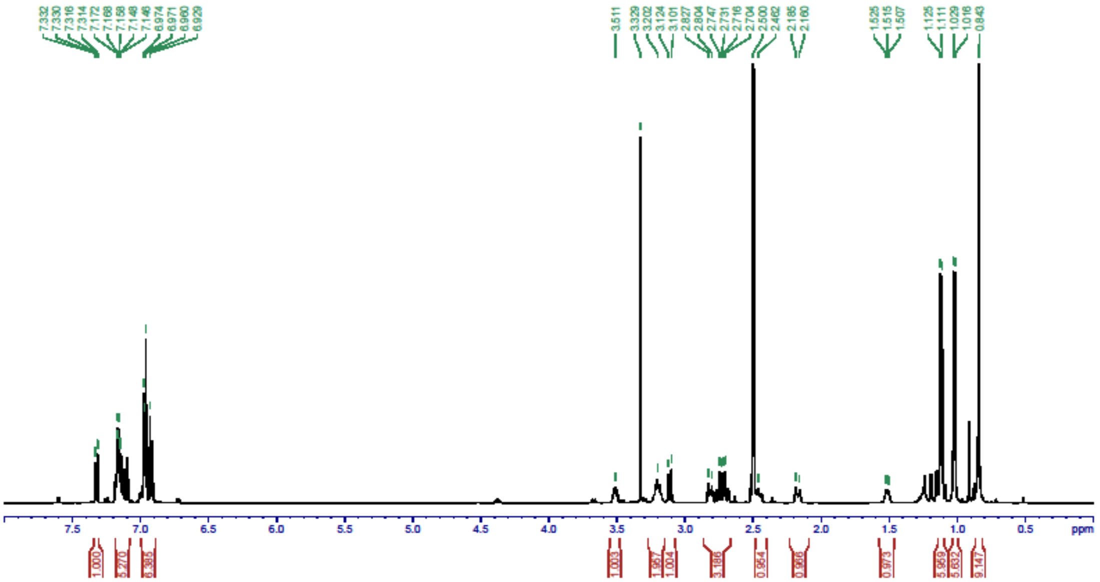

Figure S93. ^{1}H NMR (500 MHz) spectrum of **20u** in DMSO-d_6

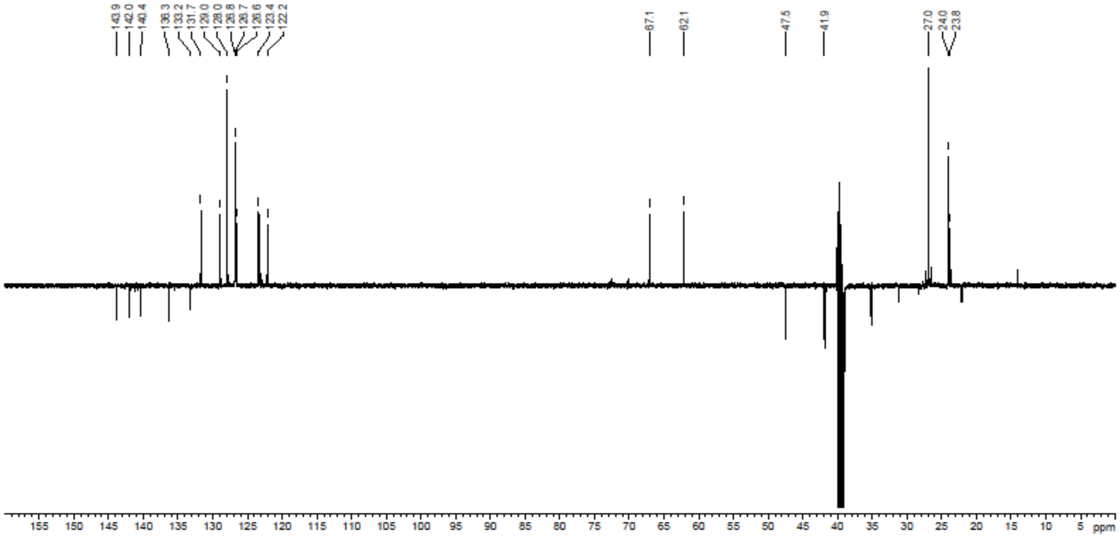

Figure S94. ^{13}C NMR (125 MHz) spectrum of **20u** in DMSO-d_6

(2*S*)-*N*[1]-[(1'*R*)-2-(2-chlorophenyl)-1-phenylethyl]-*N*[2]-(2,6-di(propan-2-yl)phenyl)-3,3-dimethylbutane-1,2-diamine [(2*S*,1'*R*)-20v]

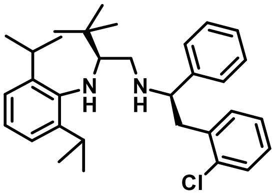

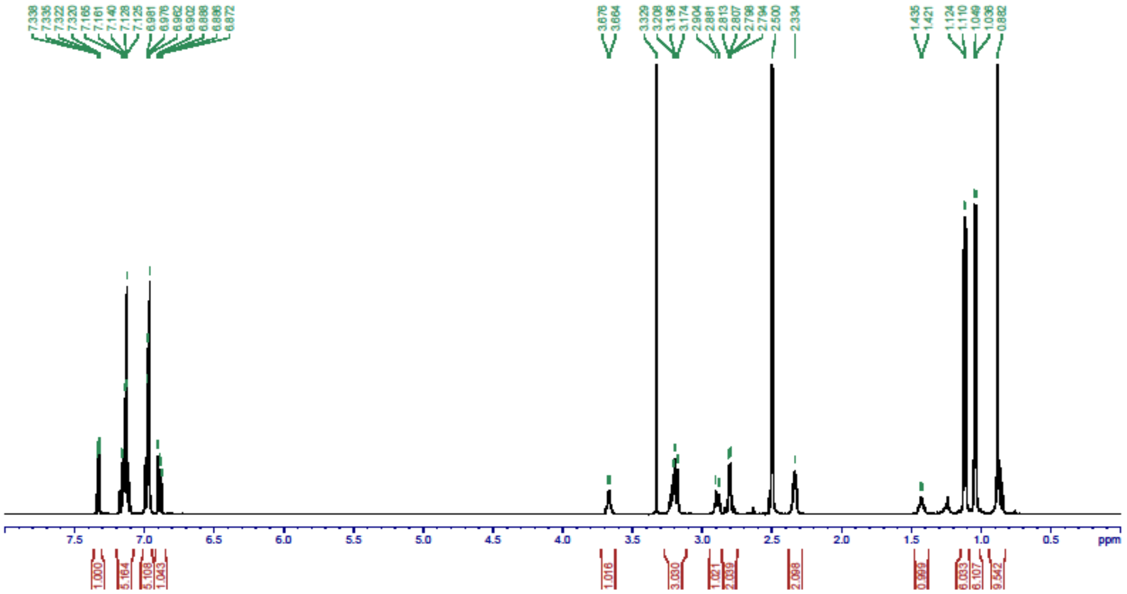

Figure S95. ^{1}H NMR (500 MHz) spectrum of **20v** in DMSO-d_6

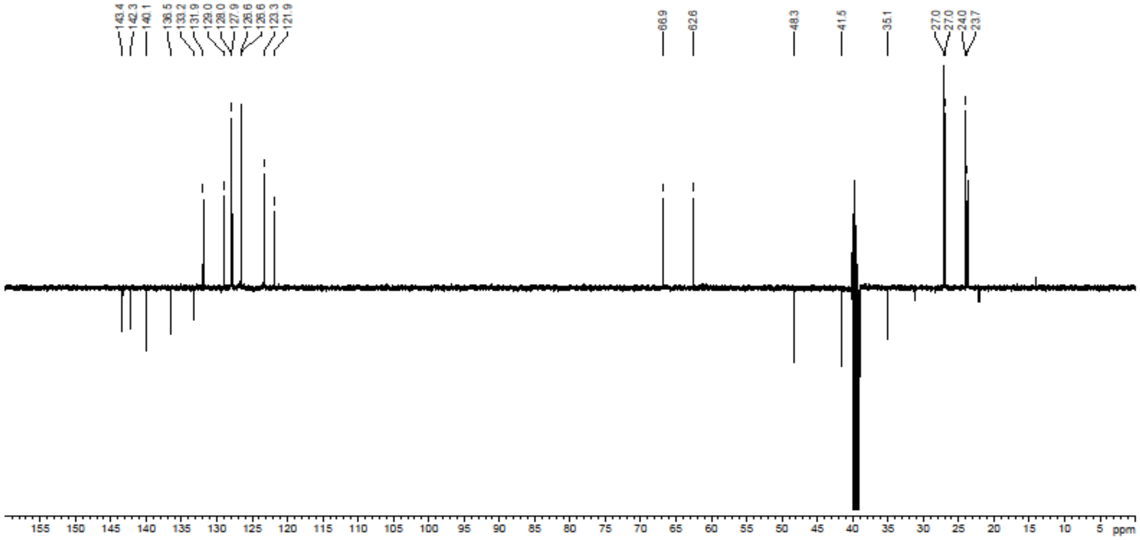

Figure S96. ^{13}C NMR (125 MHz) spectrum of **20v** in DMSO-d_6

N^1-[(1’*S*)-2-(4-chlorophenyl)-1-phenylethyl]-N^2-(2,6-di(propan-2-yl)phenyl)ethane-1,2-diamine [(1’*S*)-20w]

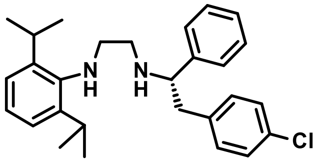

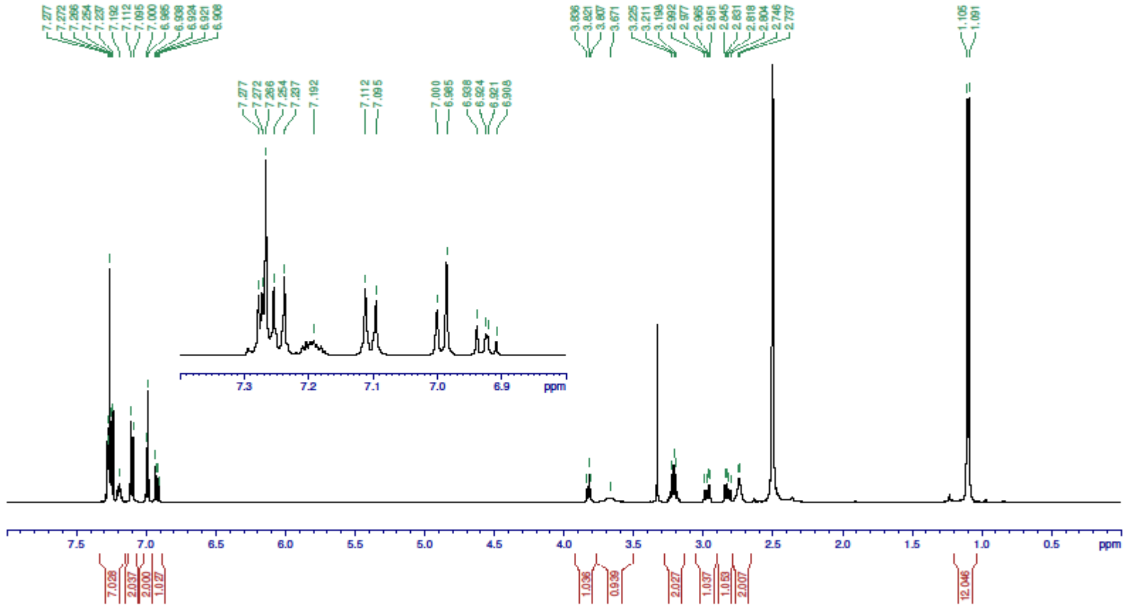

Figure S97. ^{1}H NMR (500 MHz) spectrum of **20w** in DMSO-d_6

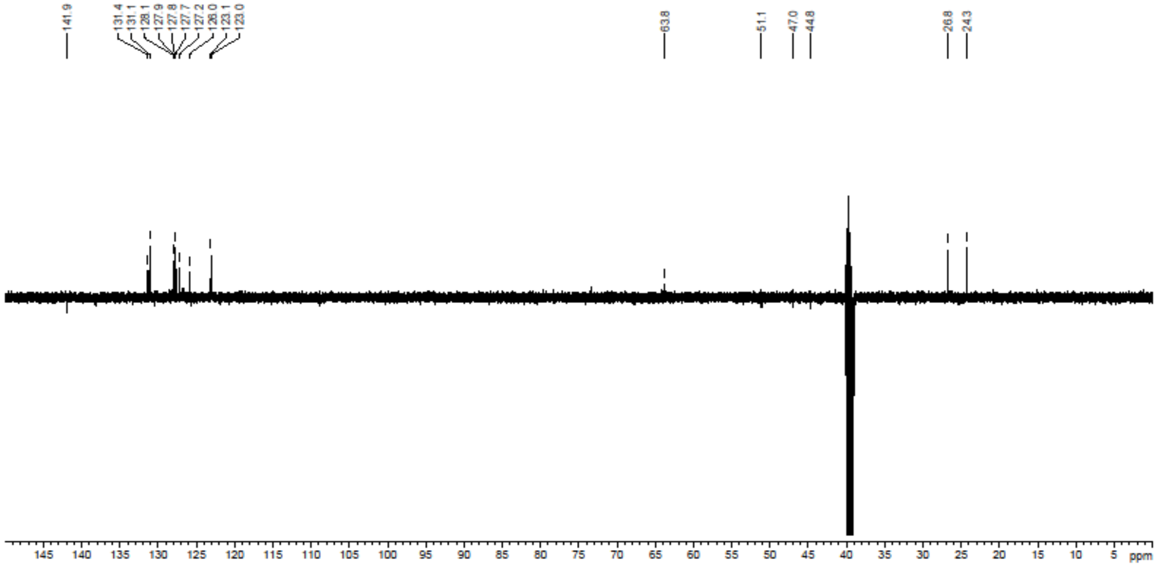

Figure S98. ^{13}C NMR (125 MHz) spectrum of **20w** in DMSO-d_6

***N*[1]-[(1'*R*)-2-(4-chlorophenyl)-1-phenylethyl]-*N*[2]-(2,6-di(propan-2-yl)phenyl)ethane-1,2-diamine [(1'*R*)-20x]**

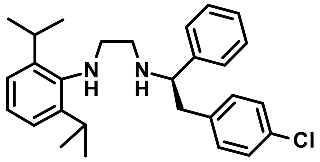

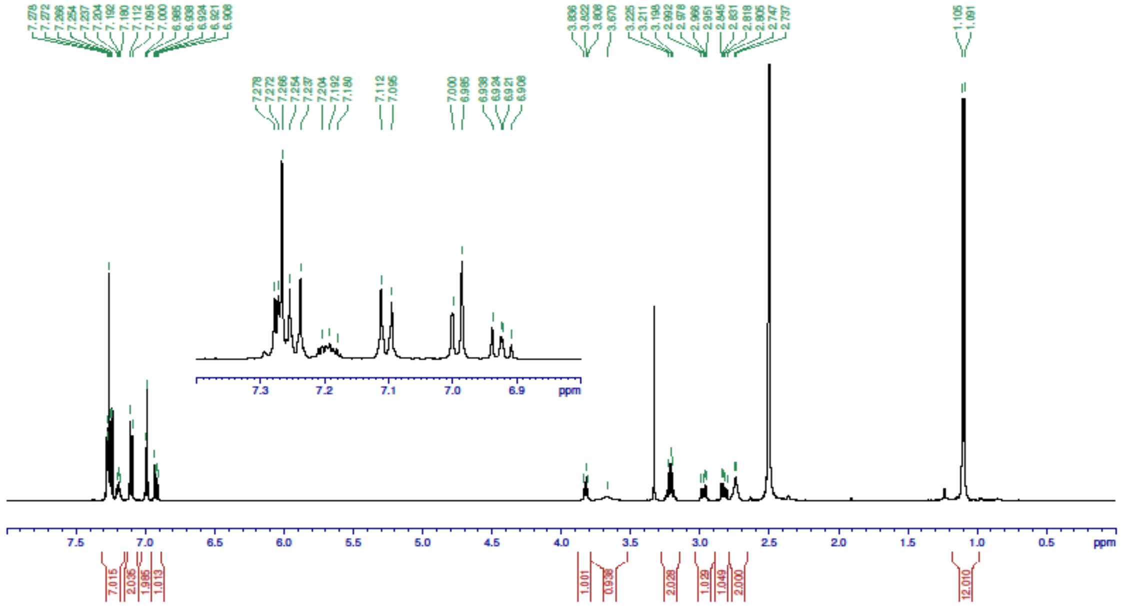

Figure S99. ^{1}H NMR (500 MHz) spectrum of **20x** in DMSO-d_6

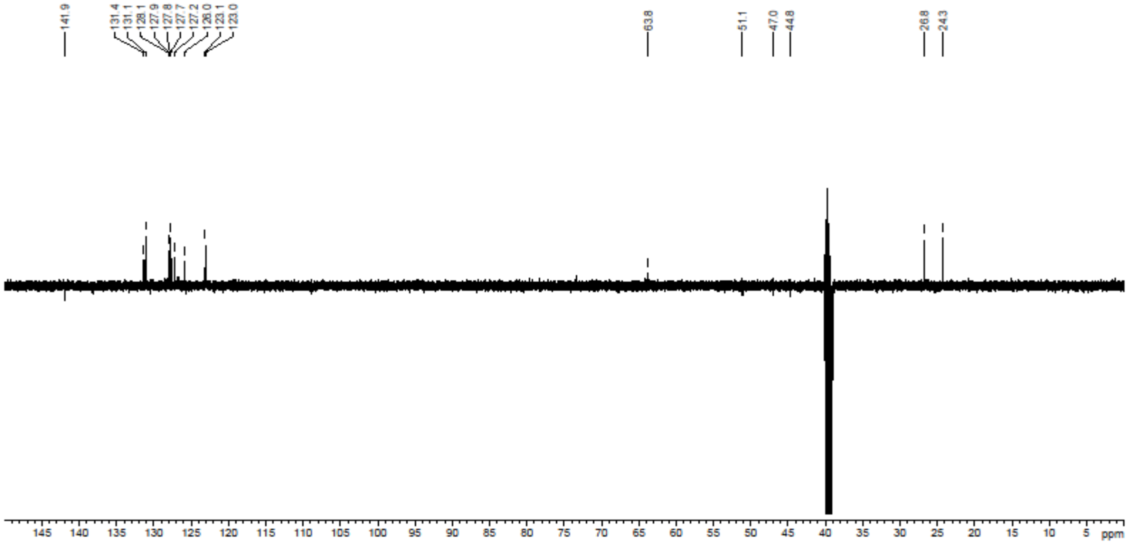

Figure S100. ^{13}C NMR (125 MHz) spectrum of **20x** in DMSO-d_6

(2*S*)-*N*²-(2,6-di(propan-2-yl)phenyl)-3,3-dimethyl-*N*¹-[(1’*S*)-1-(2-naphthyl)ethyl]butane-1,2-diamine [(2*S*,1’*S*)-20y]

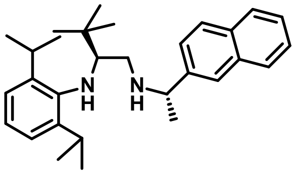

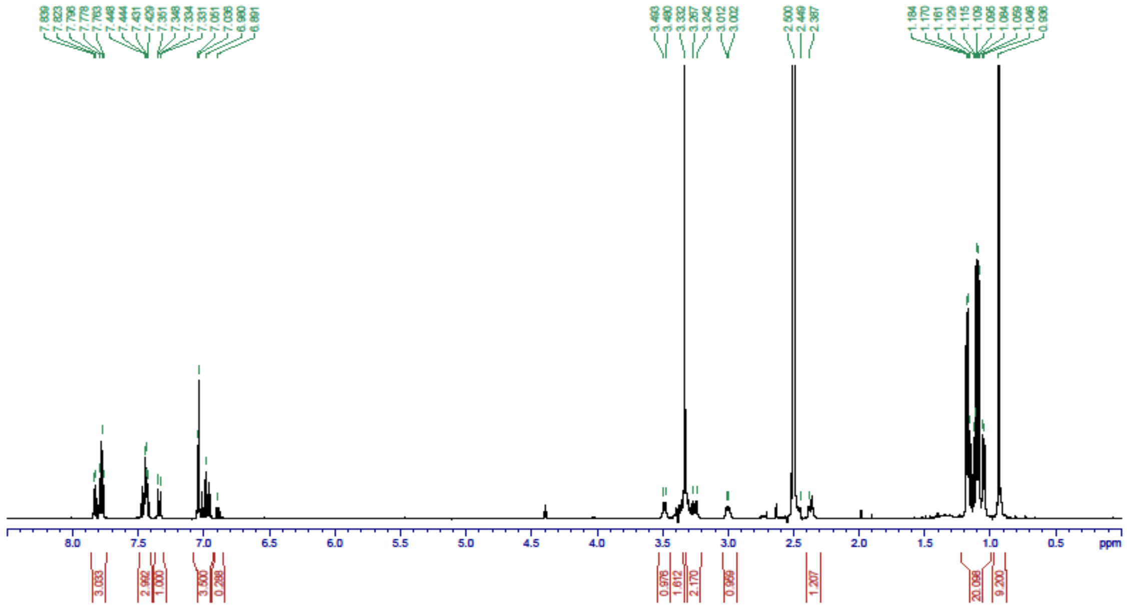

Figure S101. ^{1}H NMR (500 MHz) spectrum of **20y** in DMSO-d_6

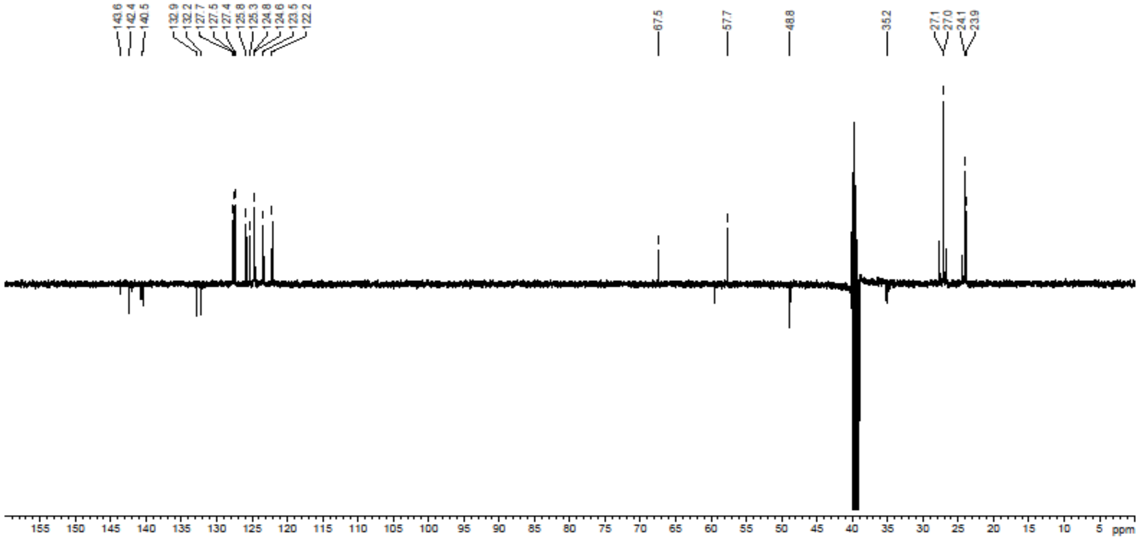

Figure S102. ^{13}C NMR (125 MHz) spectrum of **20y** in DMSO-d_6

(2*S*)-*N*²-(2,6-di(propan-2-yl)phenyl)-3,3-dimethyl-*N*¹-[(1'*R*)-1-(2-naphthyl)ethyl]butane-1,2-diamine [(2*S*,1'*R*)-20z]

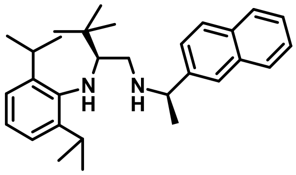

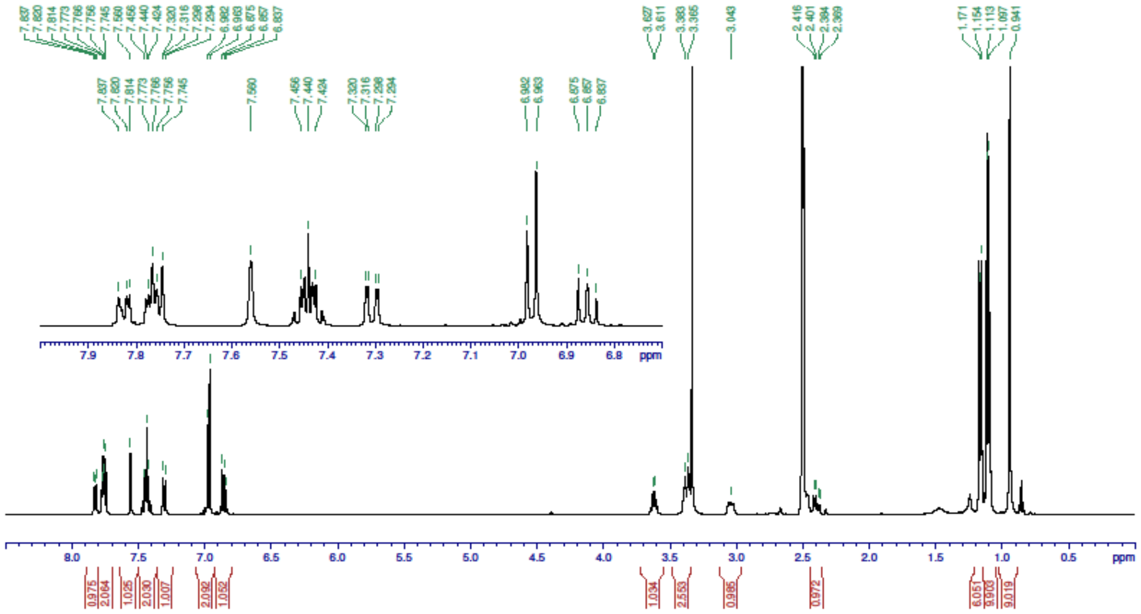

Figure S103. ^{1}H NMR (400 MHz) spectrum of **20z** in DMSO-d_6

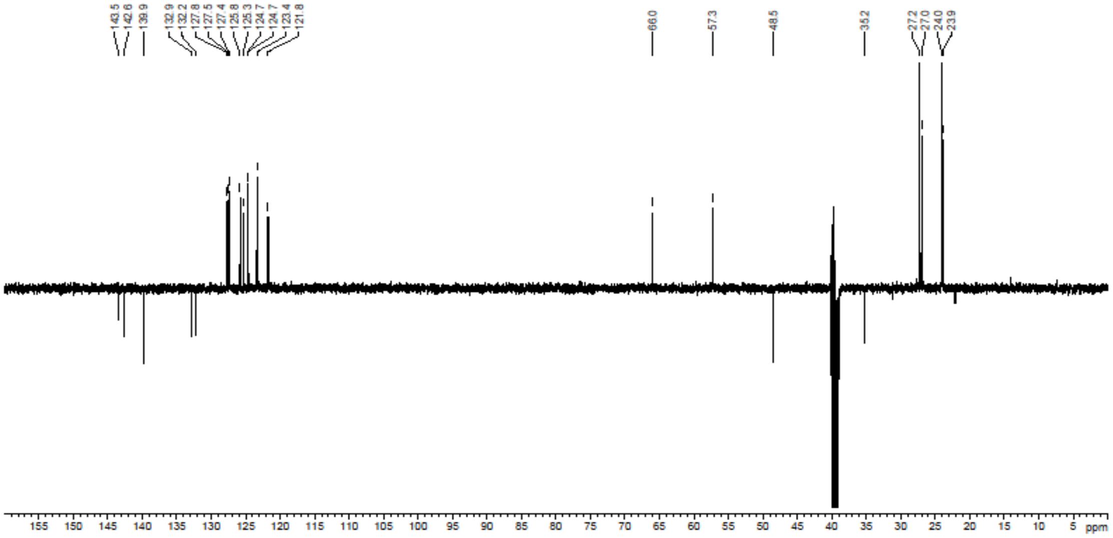

Figure S104. ^{13}C NMR (100 MHz) spectrum of **20z** in DMSO-d_6

2-(3-chlorophenyl)-1-phenyl-ethanone (21a)

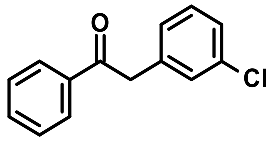

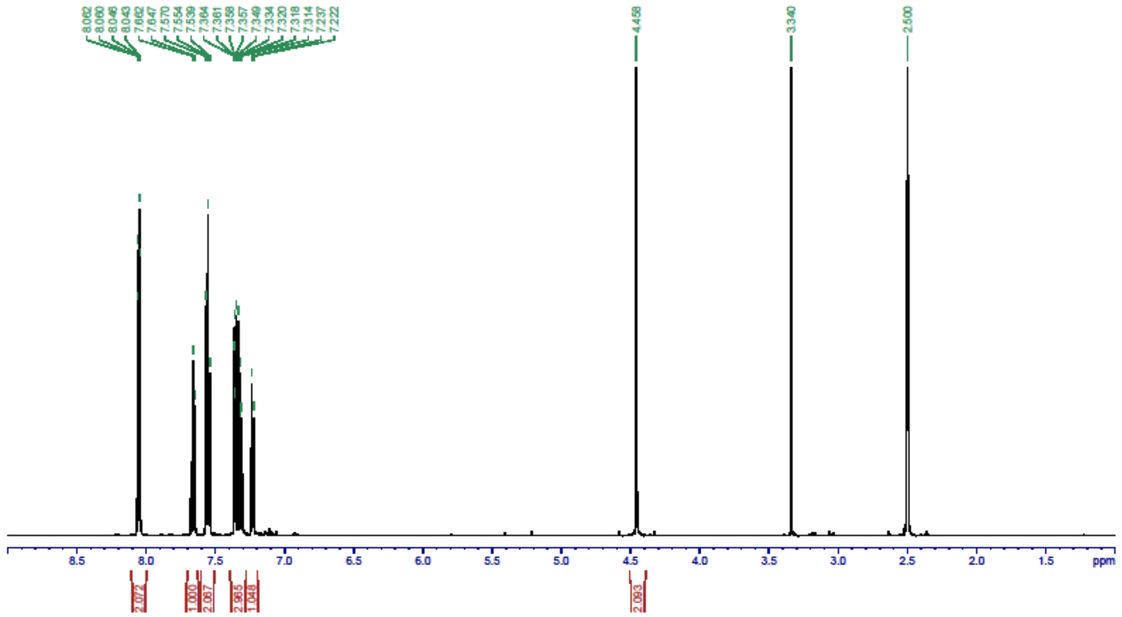

Figure S105. ^{1}H NMR (500 MHz) spectrum of **21a** in DMSO-d_6

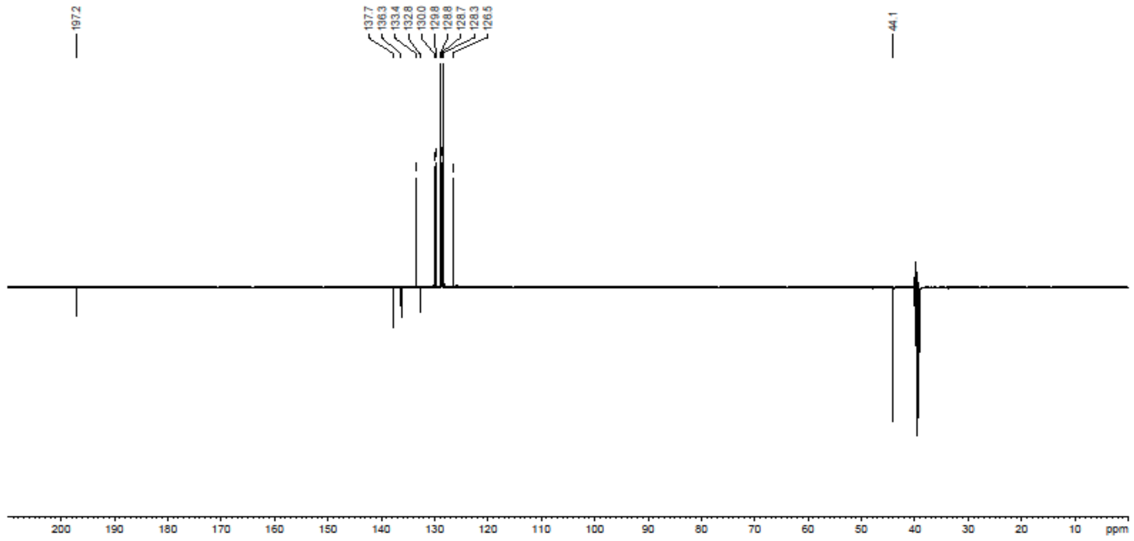

Figure S106. ^{13}C NMR (125 MHz) spectrum of **21a** in DMSO-d_6

2-phenyl-1-[3-(trifluoromethyl)phenyl]ethanone (21b)

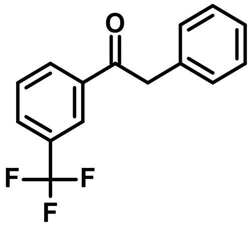

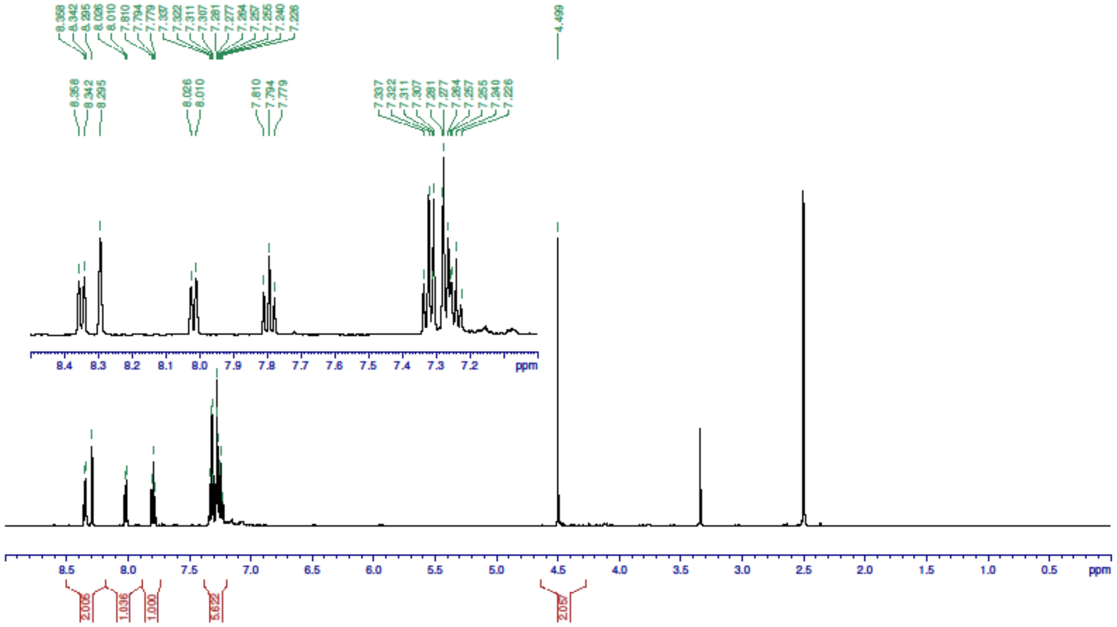

Figure S107. ^{1}H NMR (500 MHz) spectrum of **21b** in DMSO-d_6

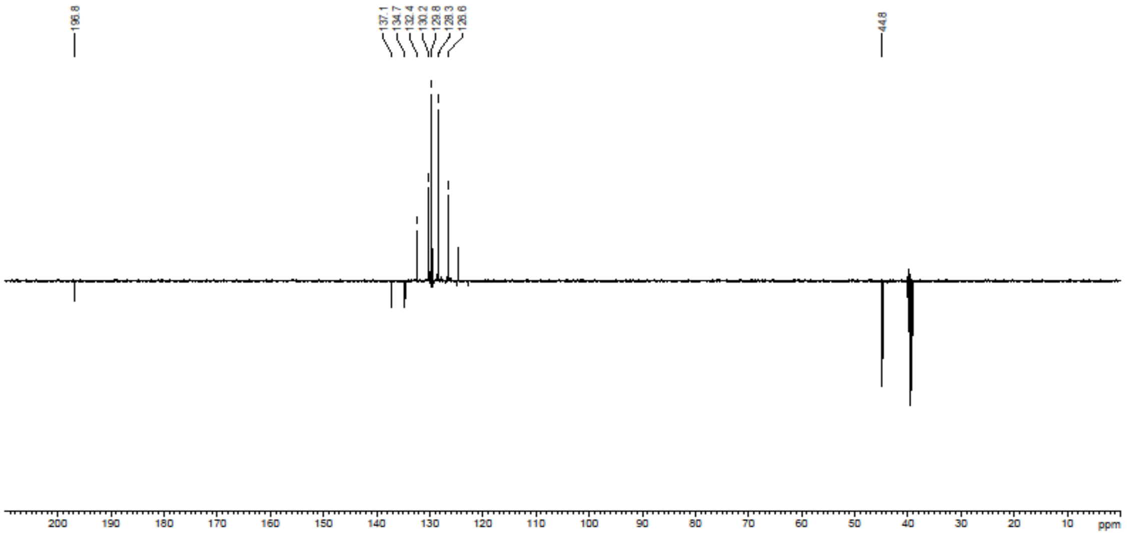

Figure S108. ^{13}C NMR (125 MHz) spectrum of **21b** in DMSO-d_6

1-(2-methoxyphenyl)-2-phenyl-ethanone (21c)

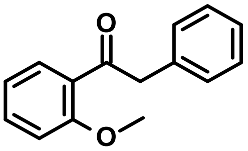

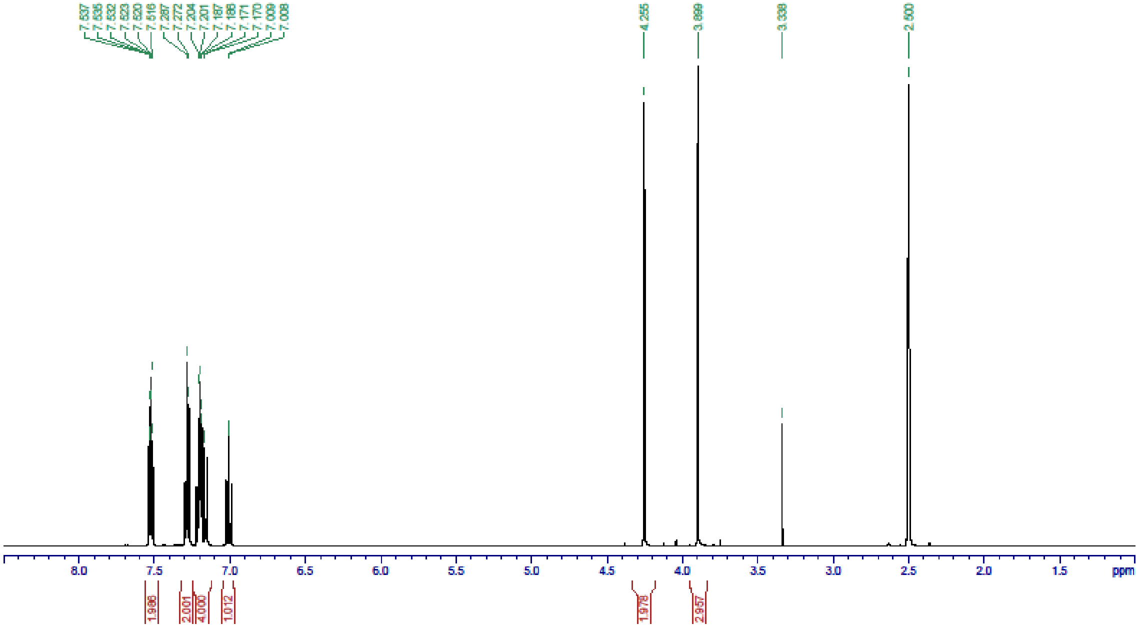

Figure S109. ^{1}H NMR (500 MHz) spectrum of **21c** in DMSO-d_6

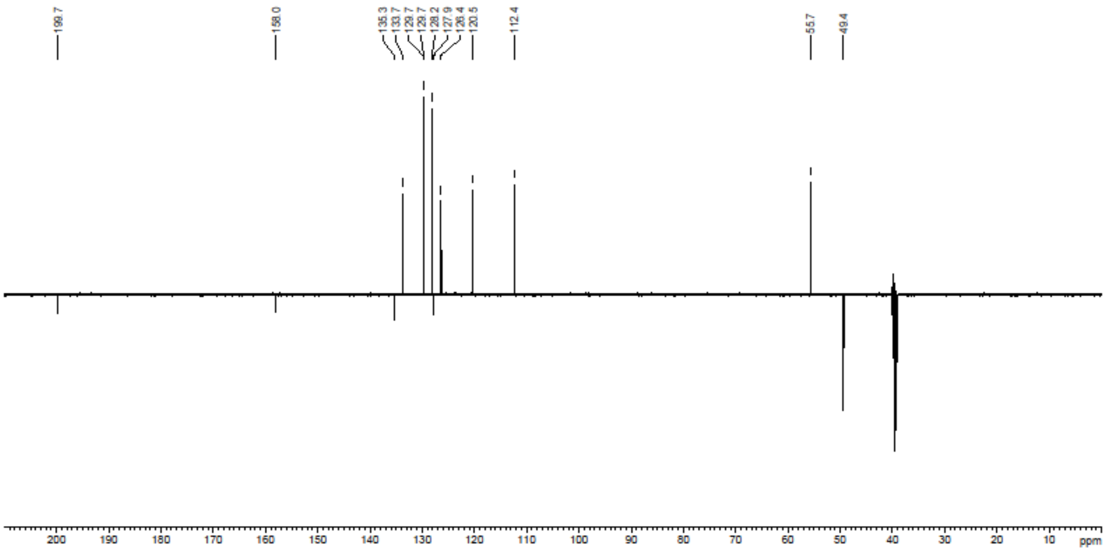

Figure S110. ^{13}C NMR (125 MHz) spectrum of **21c** in DMSO-d_6

1-(3-methoxyphenyl)-2-phenyl-ethanone (21d)

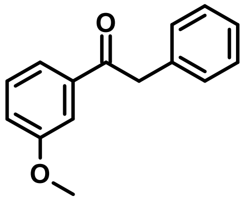

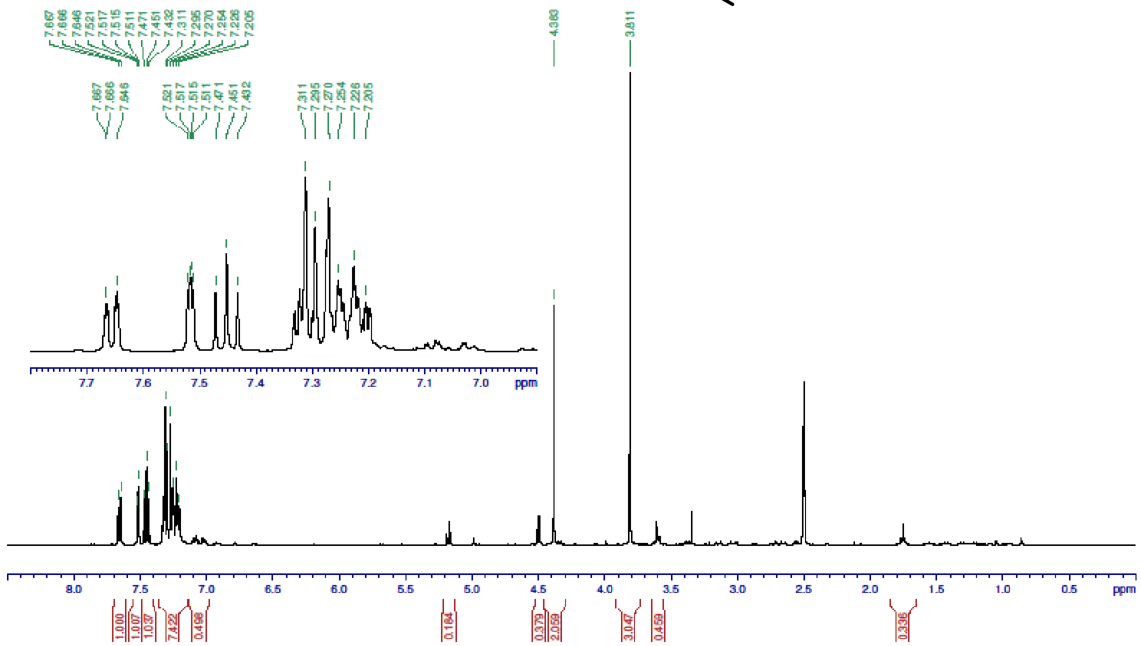

Figure S111. ^{1}H NMR (400 MHz) spectrum of **21d** in DMSO-d_6

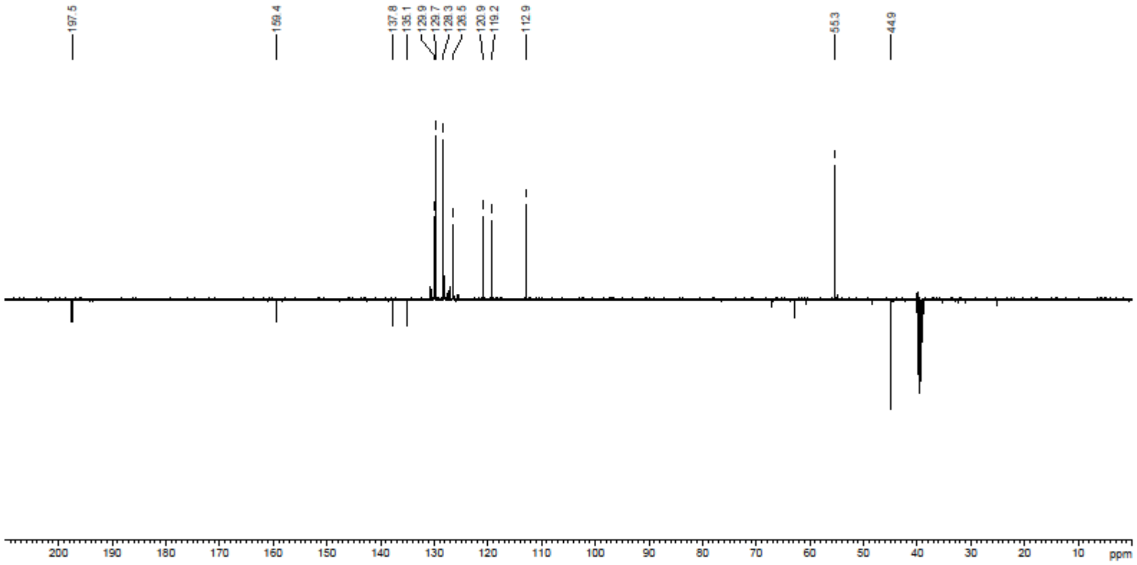

Figure S112. ^{13}C NMR (100 MHz) spectrum of **21d** in DMSO-d_6

1-(4-methoxyphenyl)-2-phenyl-ethanone (21e)

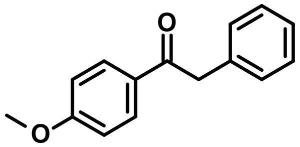

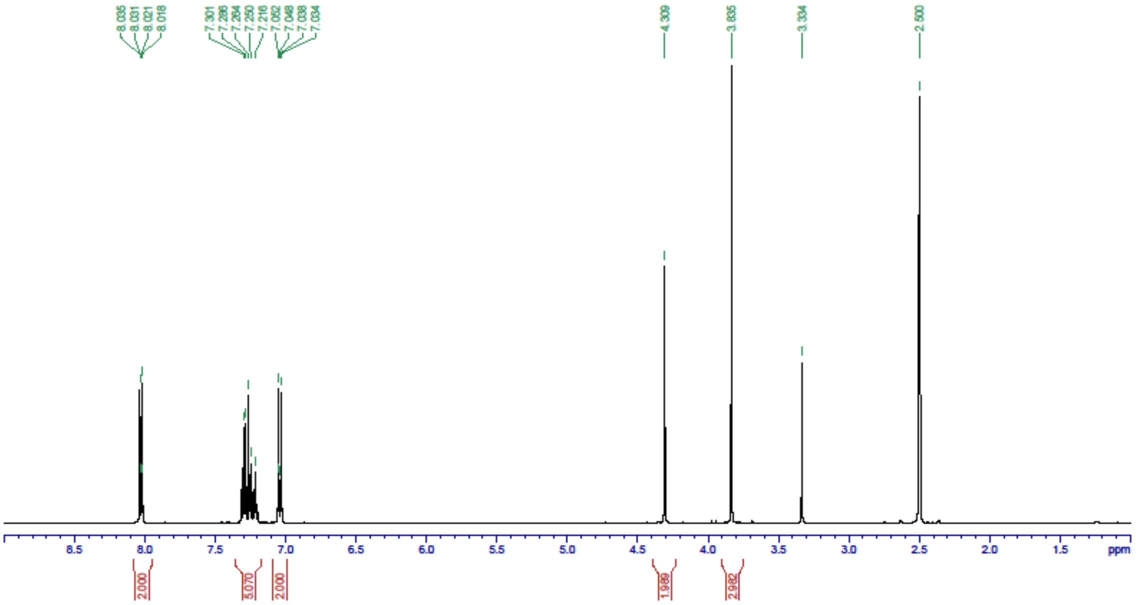

Figure S113. ^{1}H NMR (500 MHz) spectrum of **21e** in DMSO-d_6

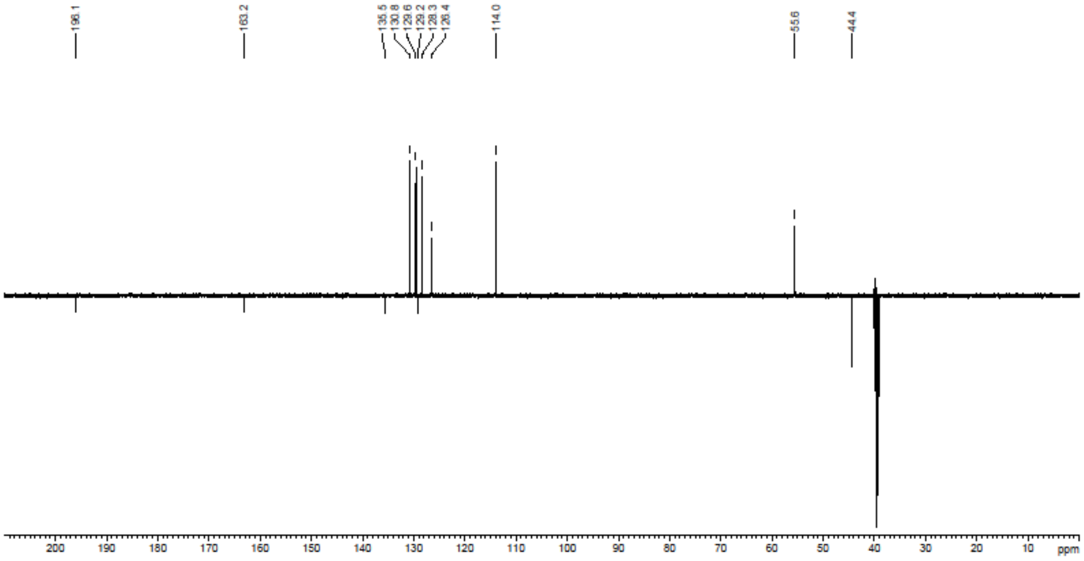

Figure S114. ^{13}C NMR (125 MHz) spectrum of **21e** in DMSO-d_6

1-(o-tolyl)-2-phenyl-ethanone (21f)

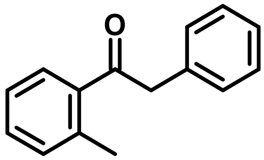

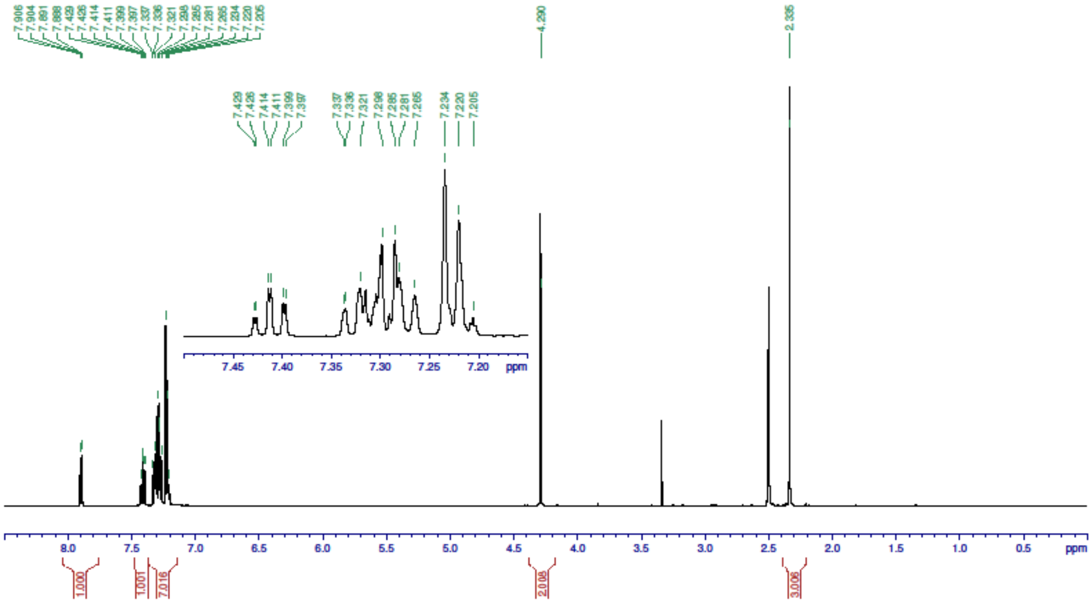

Figure S115. ^{1}H NMR (500 MHz) spectrum of **21f** in DMSO-d_6

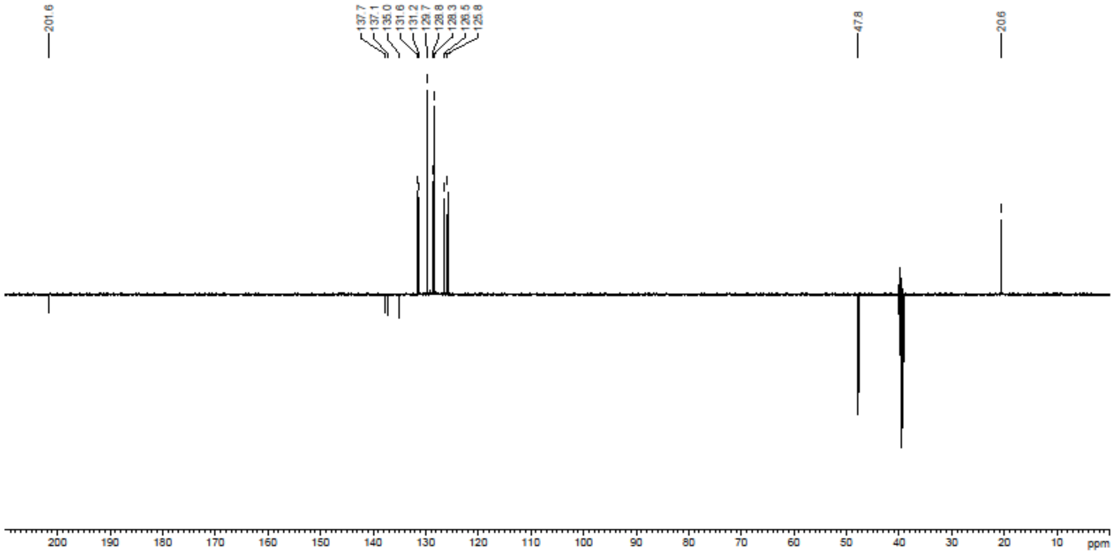

Figure S116. ^{13}C NMR (125 MHz) spectrum of **21f** in DMSO-d_6

2-phenyl-1-(p-tolyl)ethanone (21g)

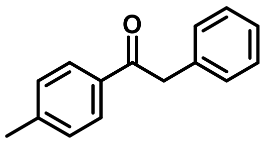

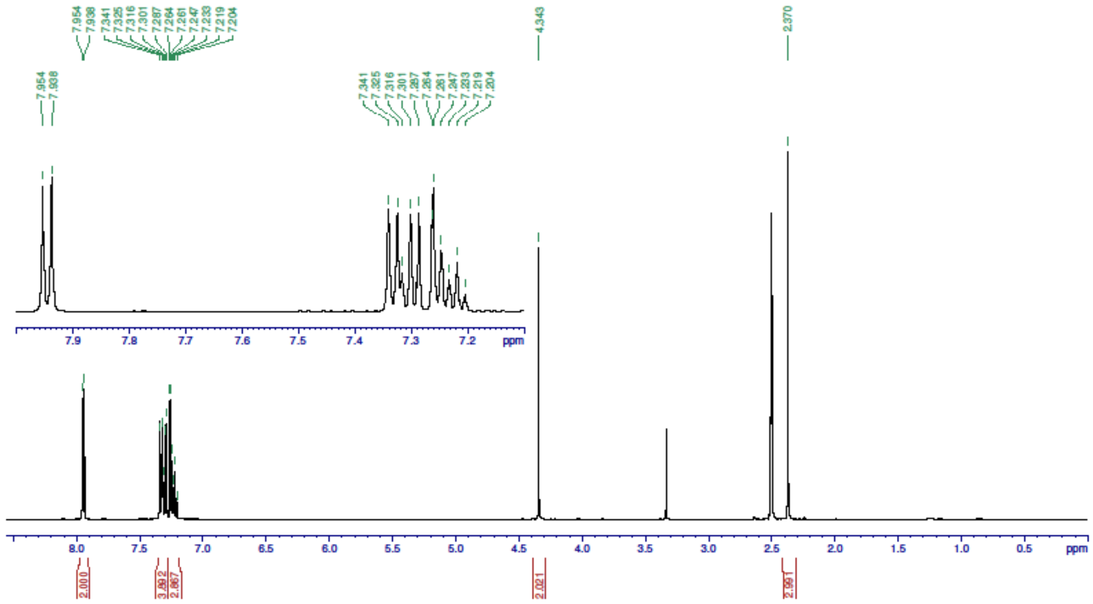

Figure S117. ^{1}H NMR (500 MHz) spectrum of **21g** in DMSO-d_6

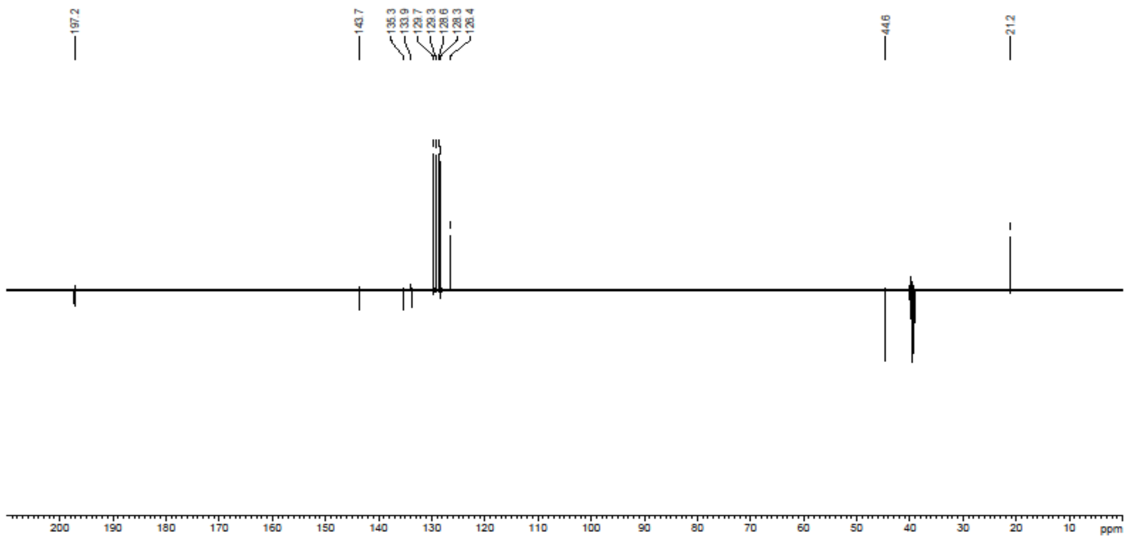

Figure S118. ^{13}C NMR (125 MHz) spectrum of **21g** in DMSO-d_6

2-phenyl-1-[4-(trifluoromethyl)phenyl]ethanone (21h)

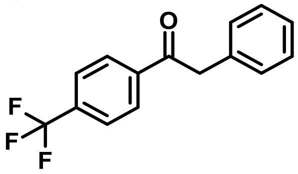

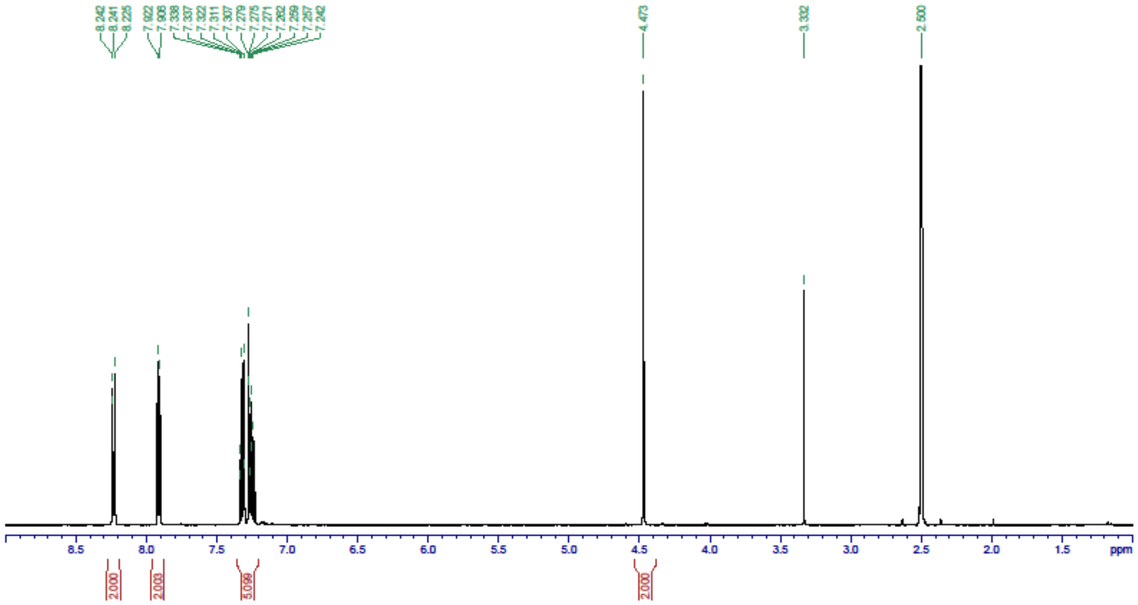

Figure S119. ^{1}H NMR (500 MHz) spectrum of **21h** in DMSO-d_6

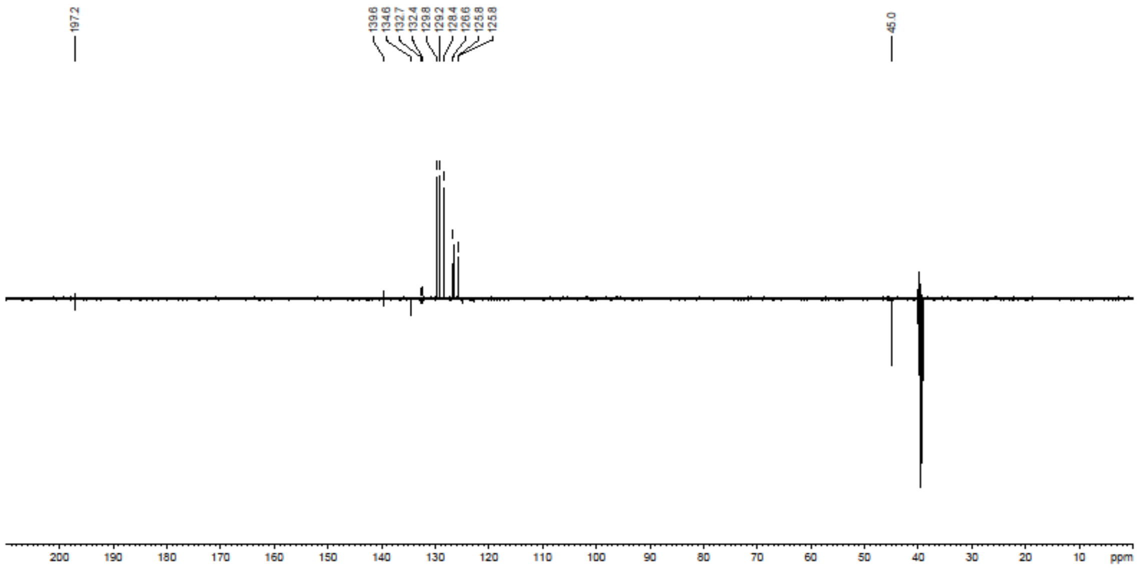

Figure S120. ^{13}C NMR (125 MHz) spectrum of **21h** in DMSO-d_6

1-phenyl-2-(p-tolyl)ethanone (21i)

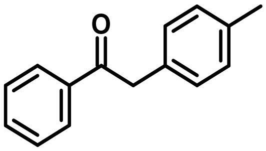

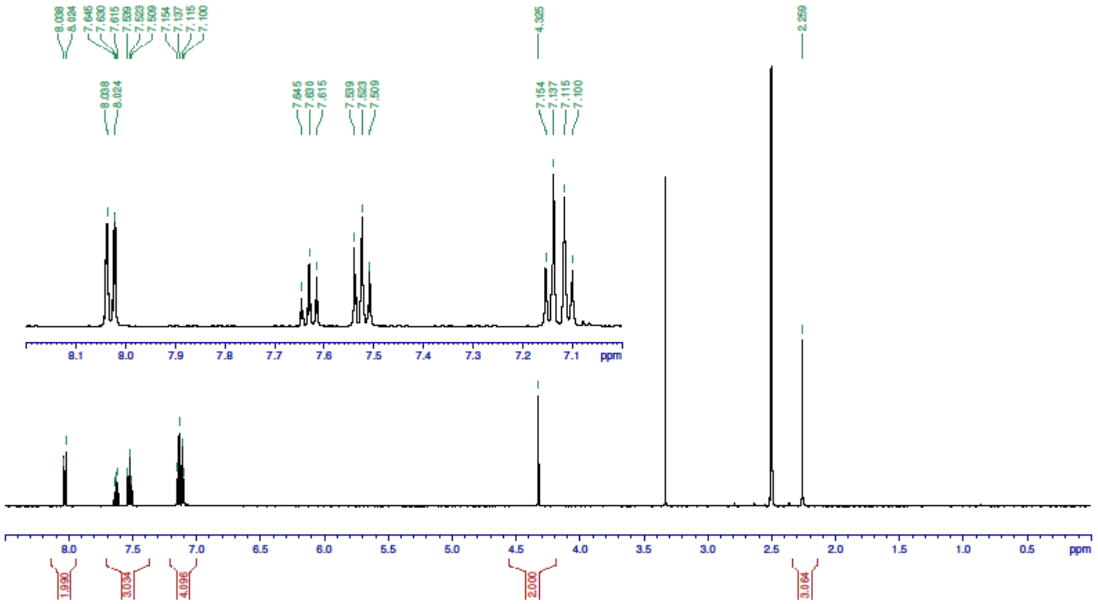

Figure S121. ^{1}H NMR (500 MHz) spectrum of **21i** in DMSO-d_6

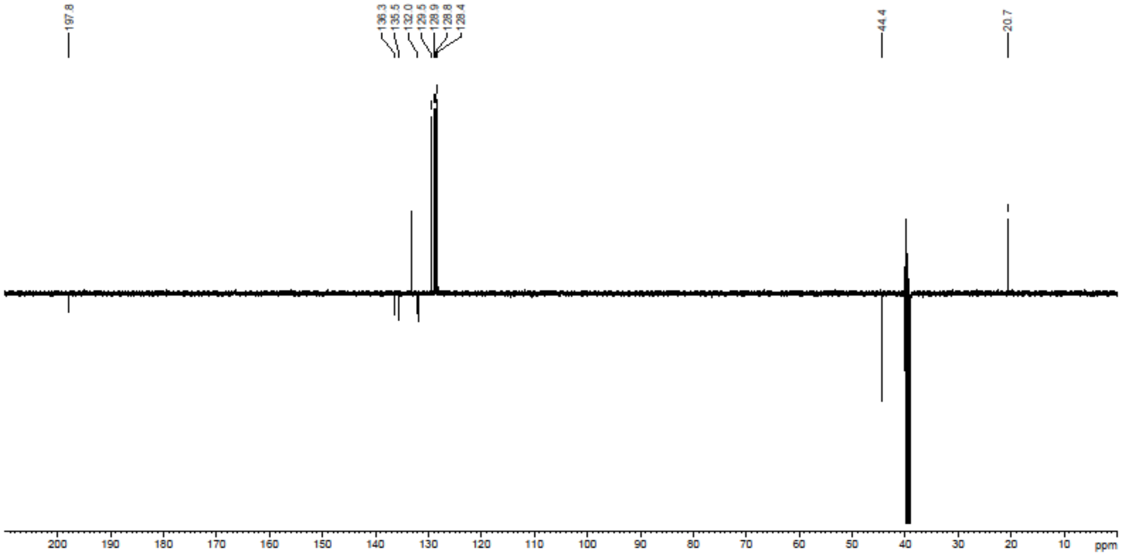

Figure S122. ^{13}C NMR (125 MHz) spectrum of **21i** in DMSO-d_6

2-(4-chlorophenyl)-1-phenyl-ethanone (21j)

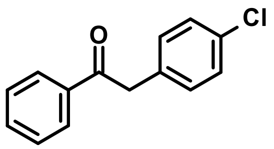

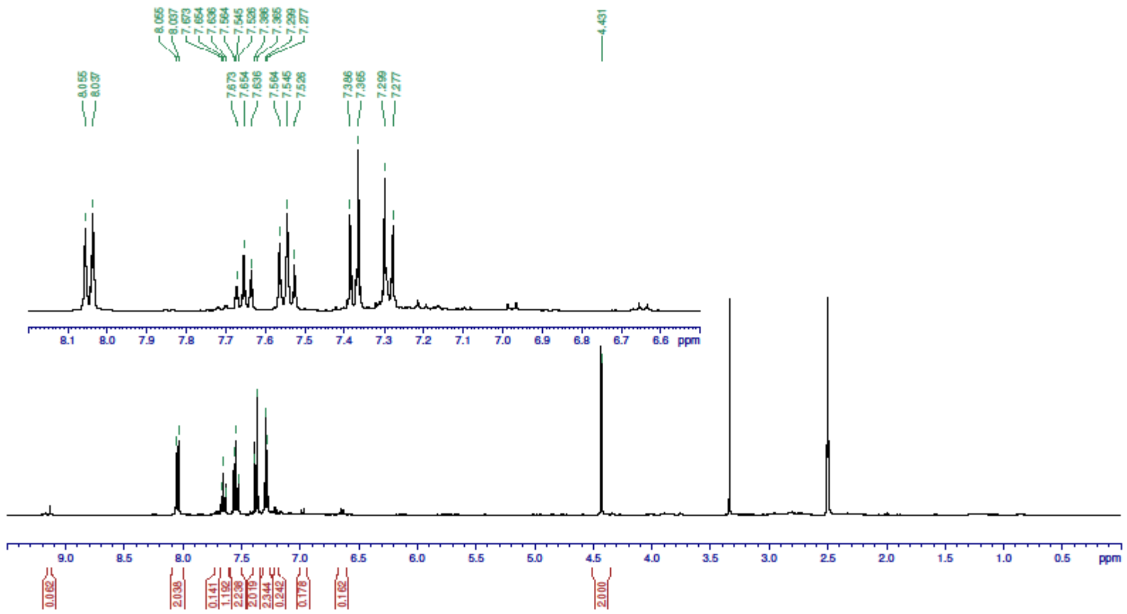

Figure S123. ^{1}H NMR (400 MHz) spectrum of **21j** in DMSO-d_6

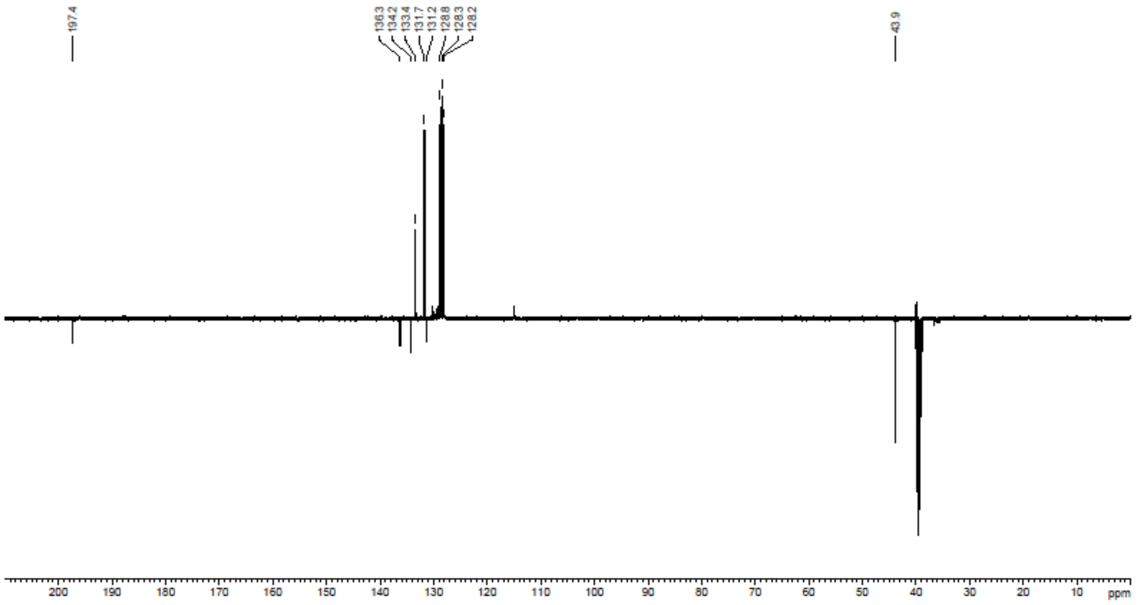

Figure S124. ^{13}C NMR (100 MHz) spectrum of **21j** in DMSO-d_6

2-(2-chlorophenyl)-1-phenyl-ethanone (21k)

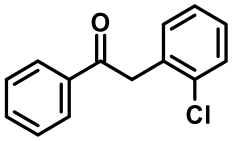

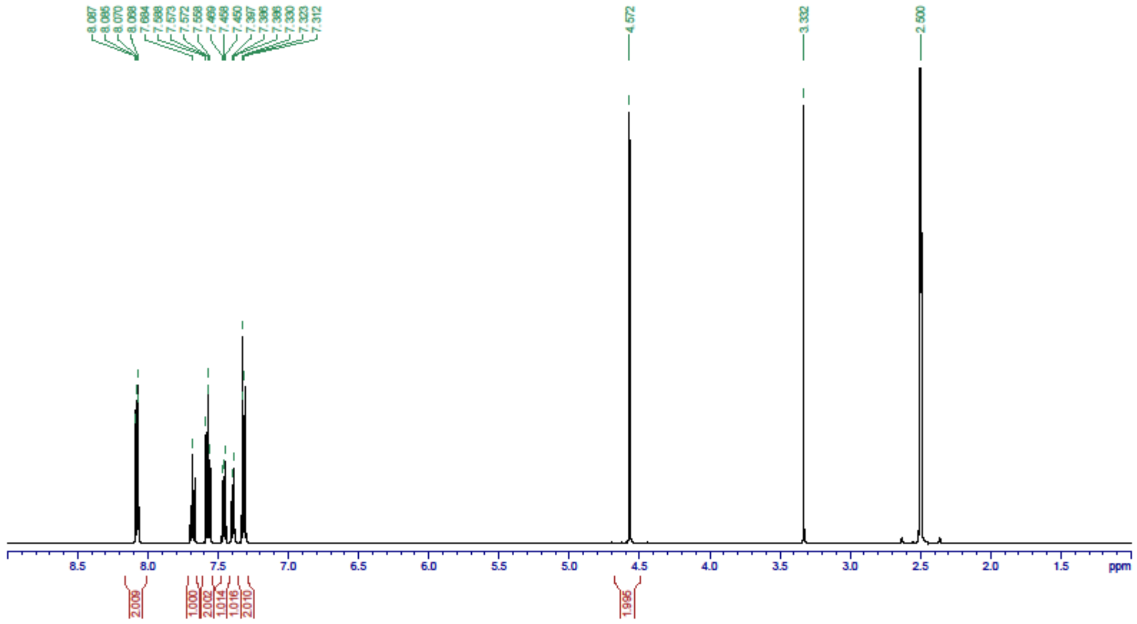

Figure S125. ^{1}H NMR (500 MHz) spectrum of **21k** in DMSO-d_6

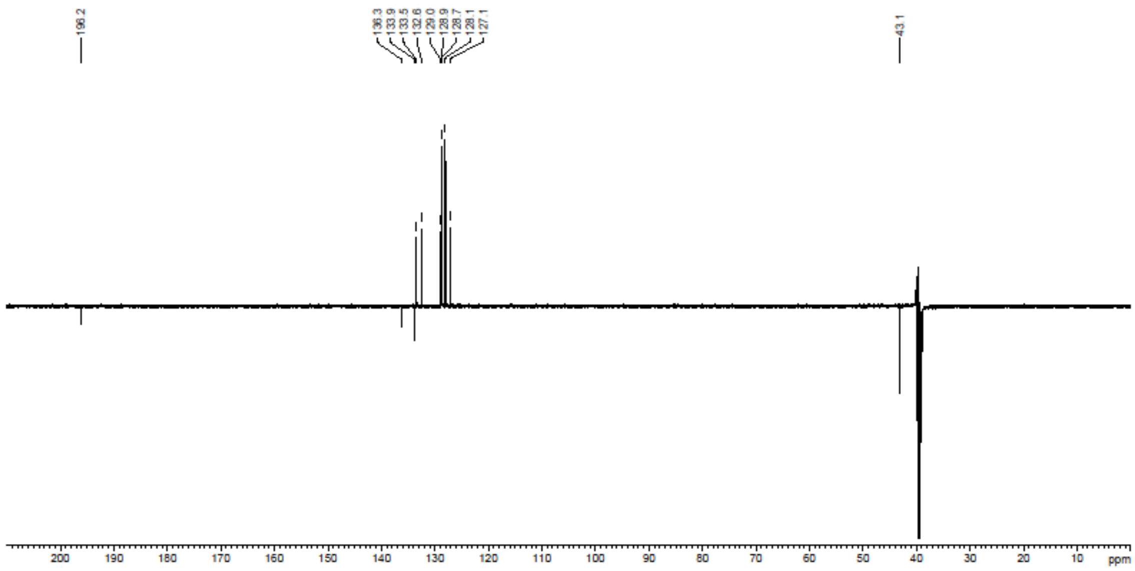

Figure S126. ^{13}C NMR (125 MHz) spectrum of **21k** in DMSO-d_6

2-(2-chlorophenyl)-1-phenyl-ethanol (22)

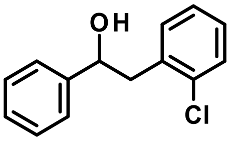

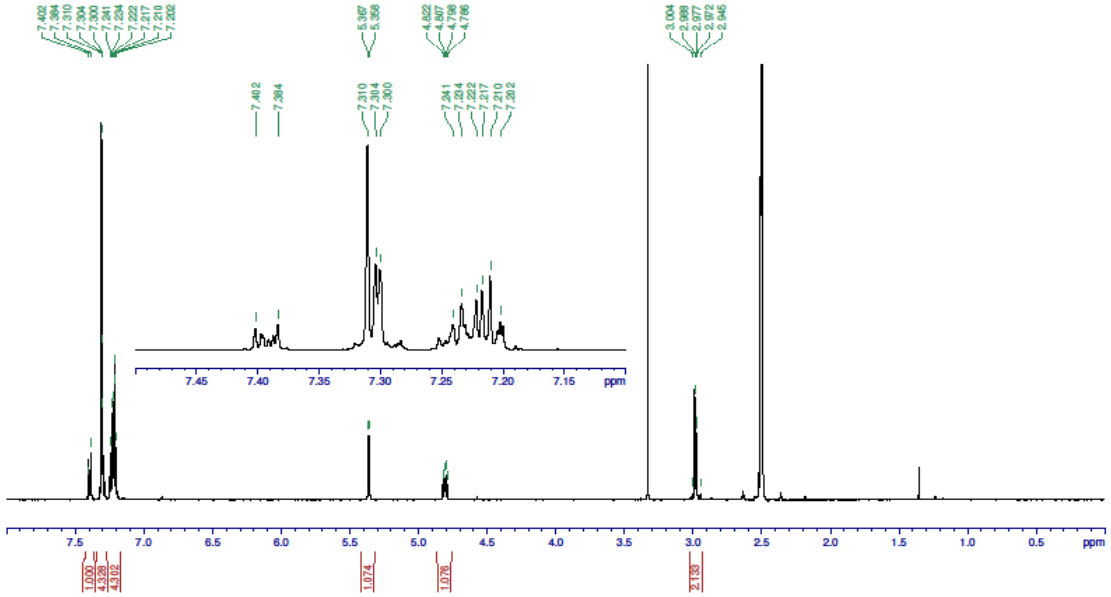

Figure S127. ^{1}H NMR (500 MHz) spectrum of **22** in DMSO-d_6

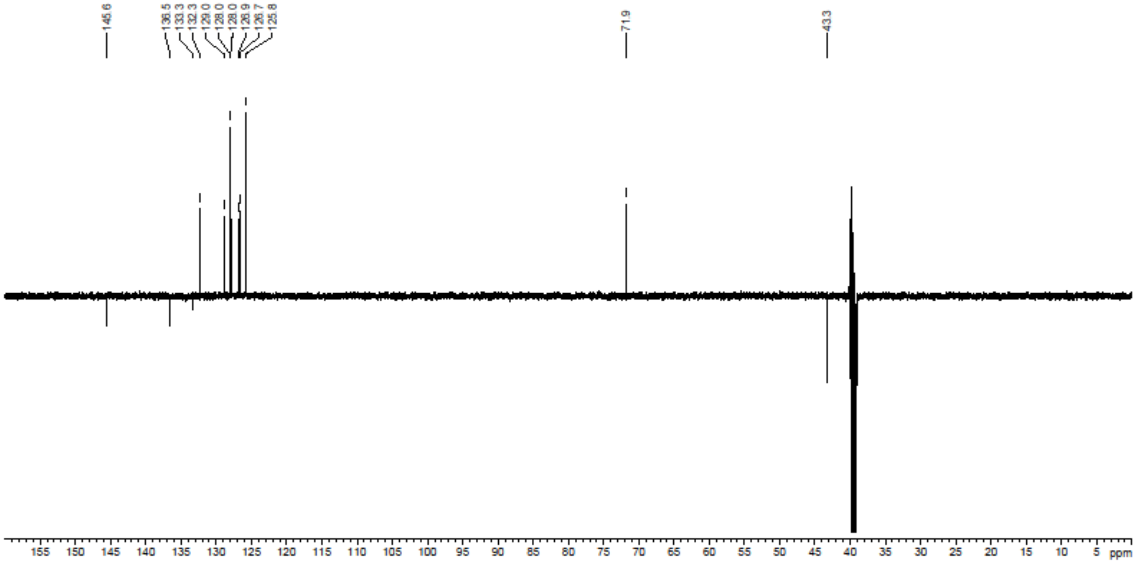

Figure S128. ^{13}C NMR (125 MHz) spectrum of **22** in DMSO-d_6

***tert*-butyl-[*tert*-butoxycarbonyl-[2-(2-chlorophenyl)-1-phenylethyl]-$l^{3}-oxidanyl]formate (23)**

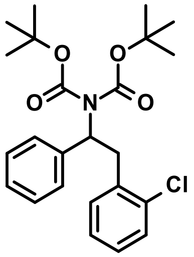

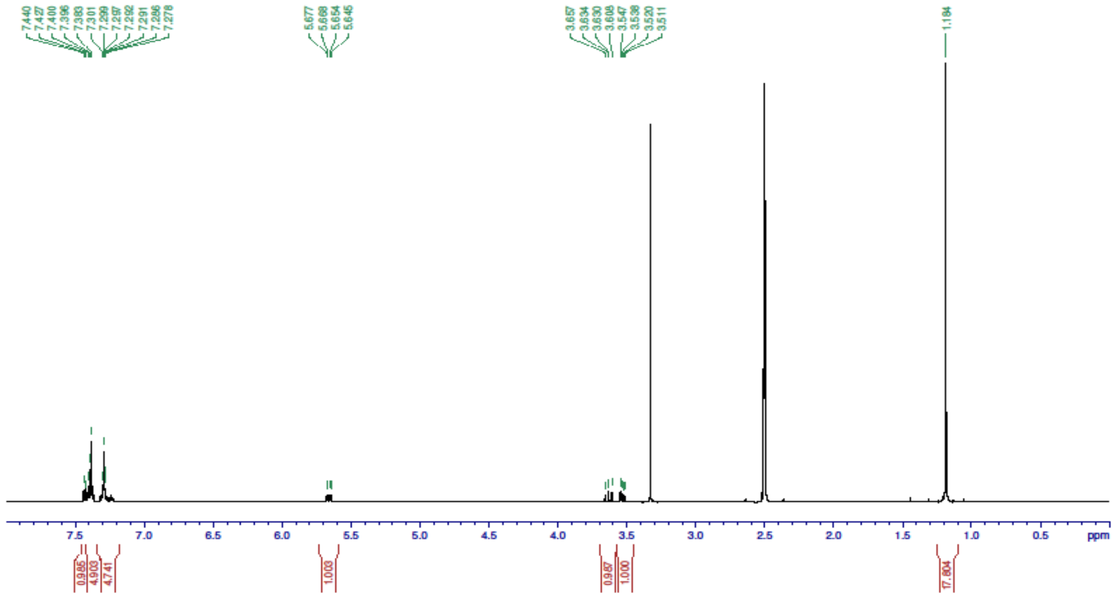

Figure S129. ^{1}H NMR (500 MHz) spectrum of **23** in DMSO-*d*$_6$

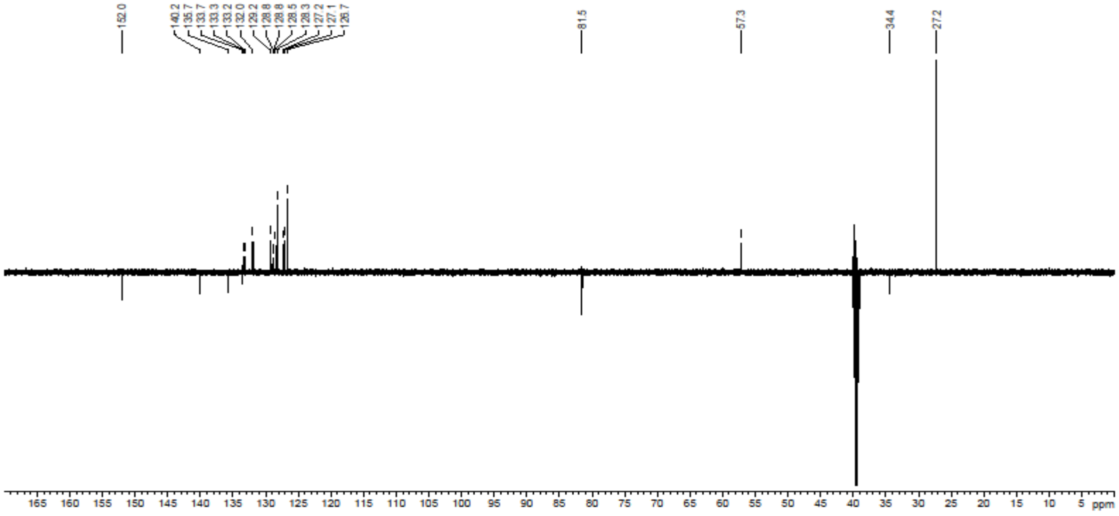

Figure S130. ^{13}C NMR (125 MHz) spectrum of **23** in DMSO-*d*$_6$

(1*S*)-2-(2-chlorophenyl)-1-phenyl-ethanamine [(1*S*)-24a]

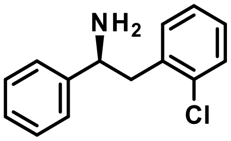

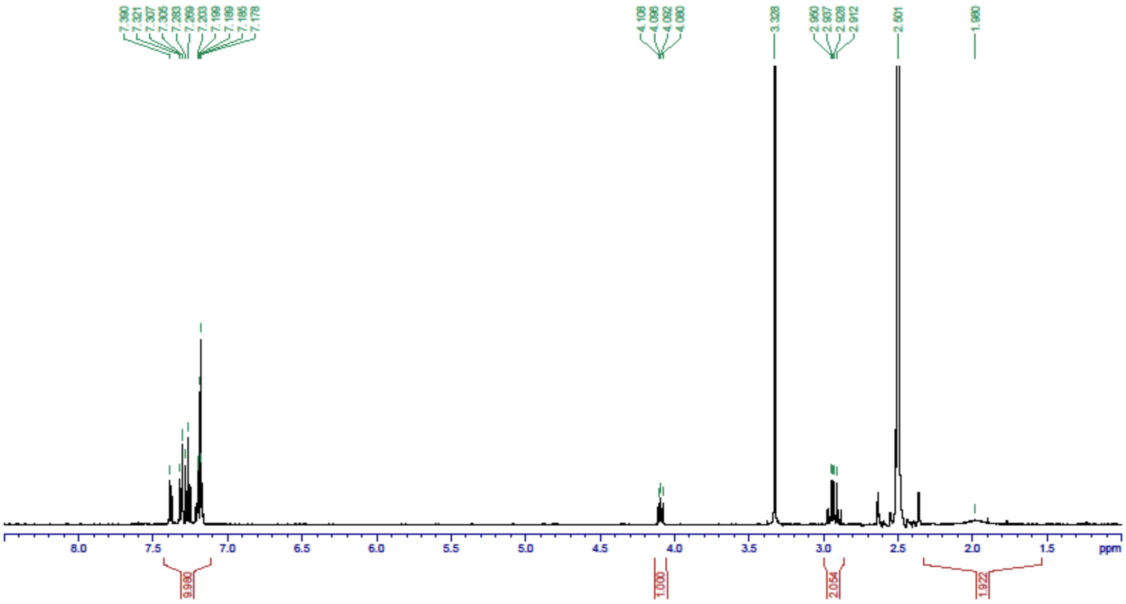

Figure S131. ^{1}H NMR (500 MHz) spectrum of **24a** in DMSO-d_6

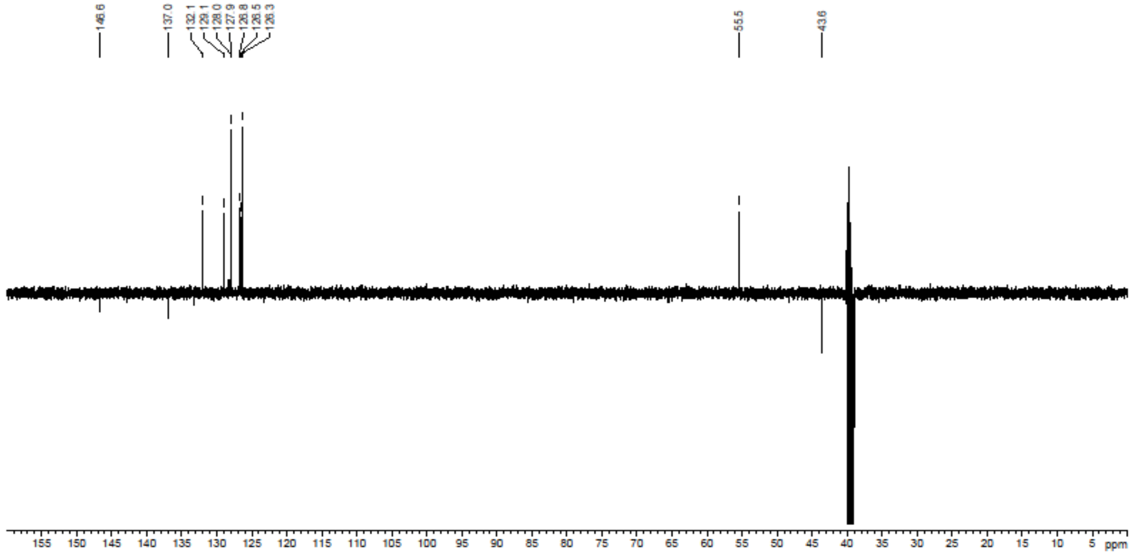

Figure S132. ^{13}C NMR (125 MHz) spectrum of **24a** in DMSO-d_6

(1*R*)-2-(2-chlorophenyl)-1-phenyl-ethanamine [(1*R*)-24b]

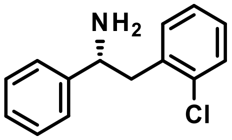

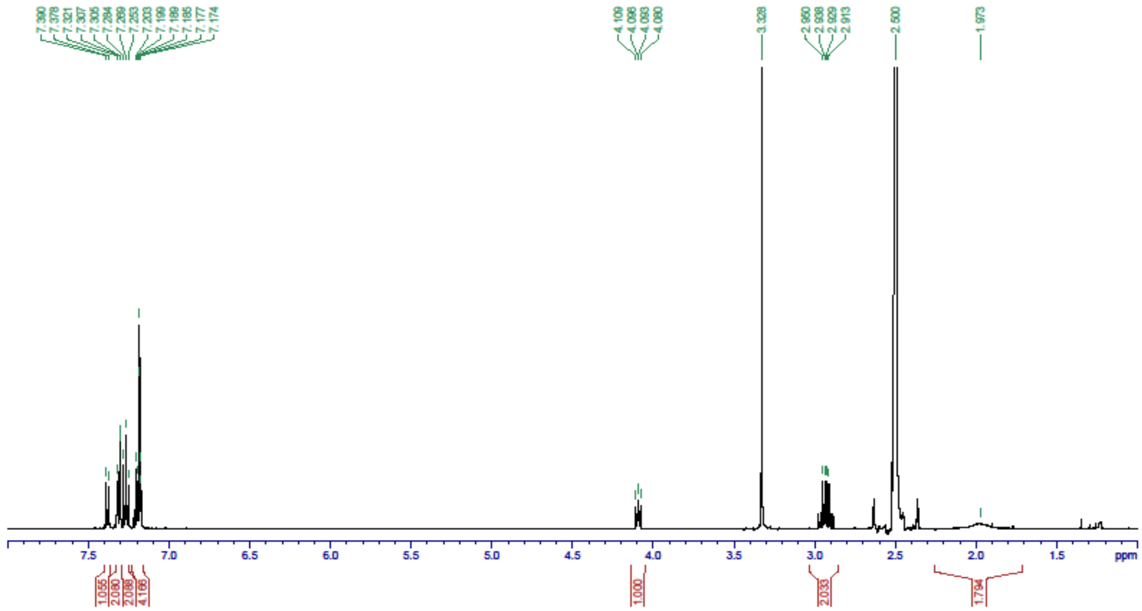

Figure S133. ^{1}H NMR (500 MHz) spectrum of **24b** in DMSO-d_6

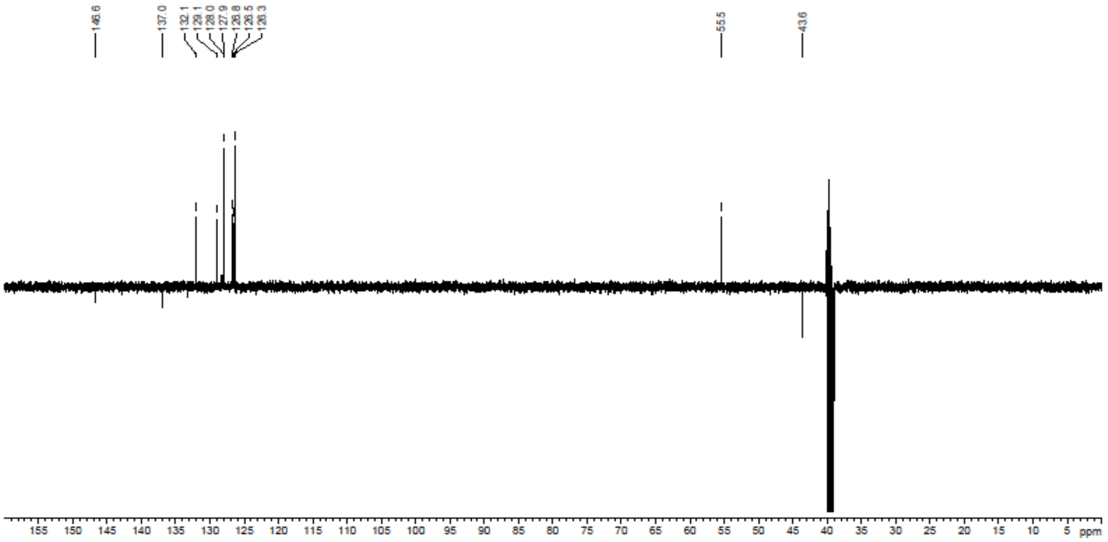

Figure S134. ^{13}C NMR (125 MHz) spectrum of **24b** in DMSO-d_6

3 HPLC chromatograms performed with optically active stationary phase

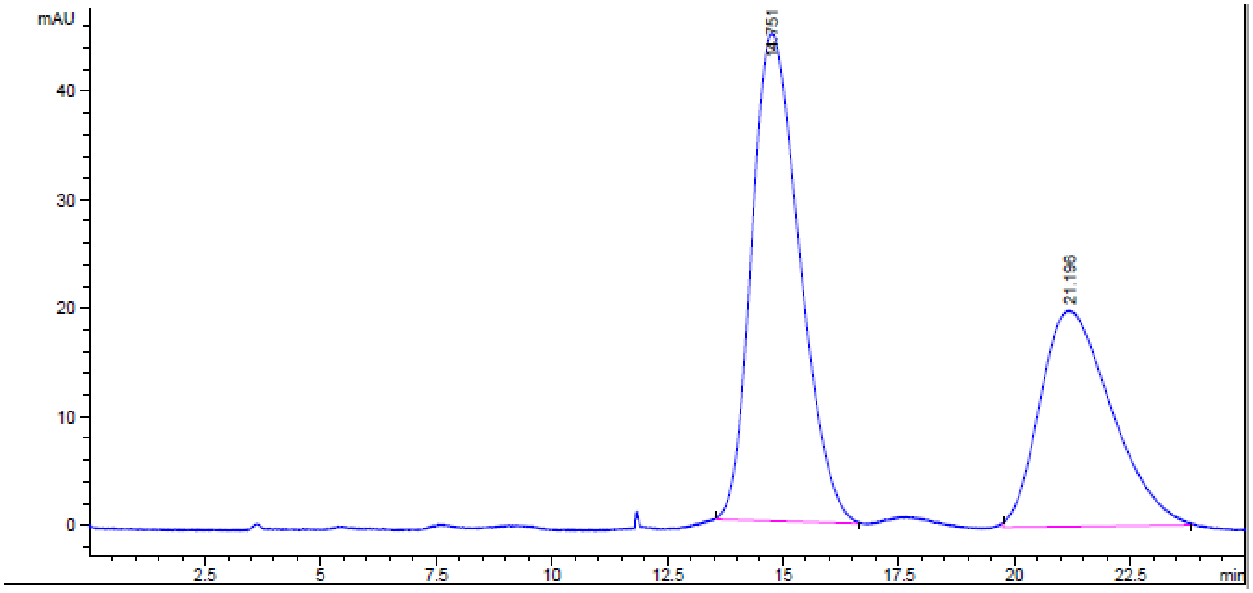

Figure S135. Chiral HPLC separation of (2*S*,1'*R*)-**20i** and (2*S*,1'*S*)-**20j** on Chiralcel OD-I column using MTBE/heptane (2:98) and 0.1% diethylamine as eluents.

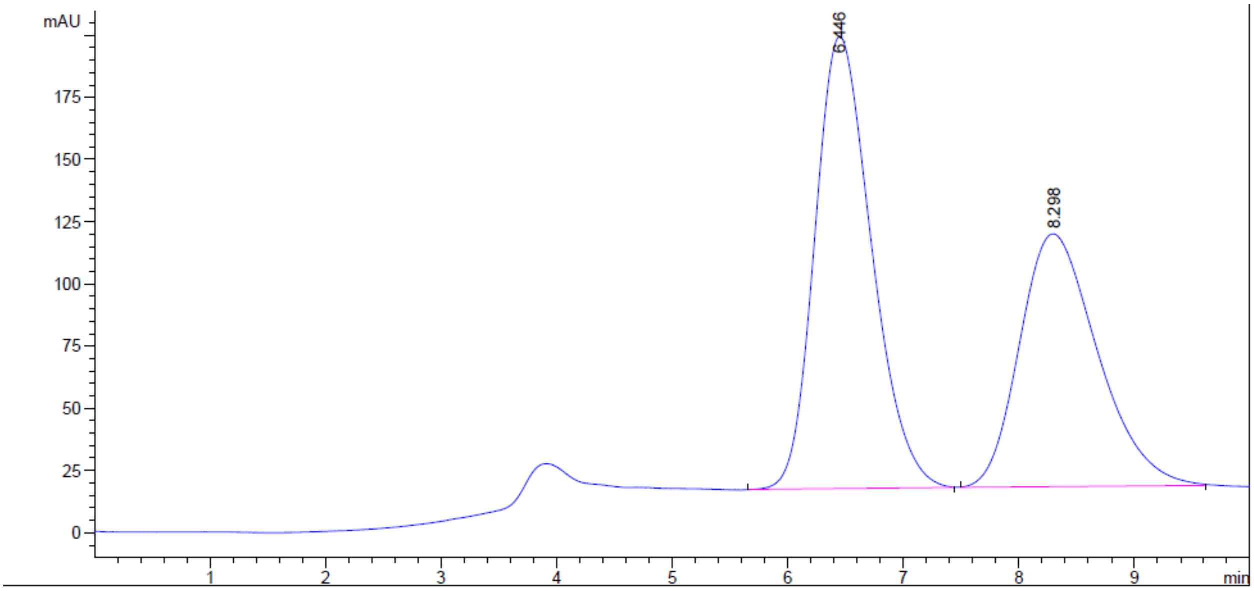

Figure S136. Chiral HPLC separation of (2*S*,1'*R*)-**20m** and (2*S*,1'*S*)-**20n** on Chiralcel OD column using heptane and 0.1% diethylamine as eluents.

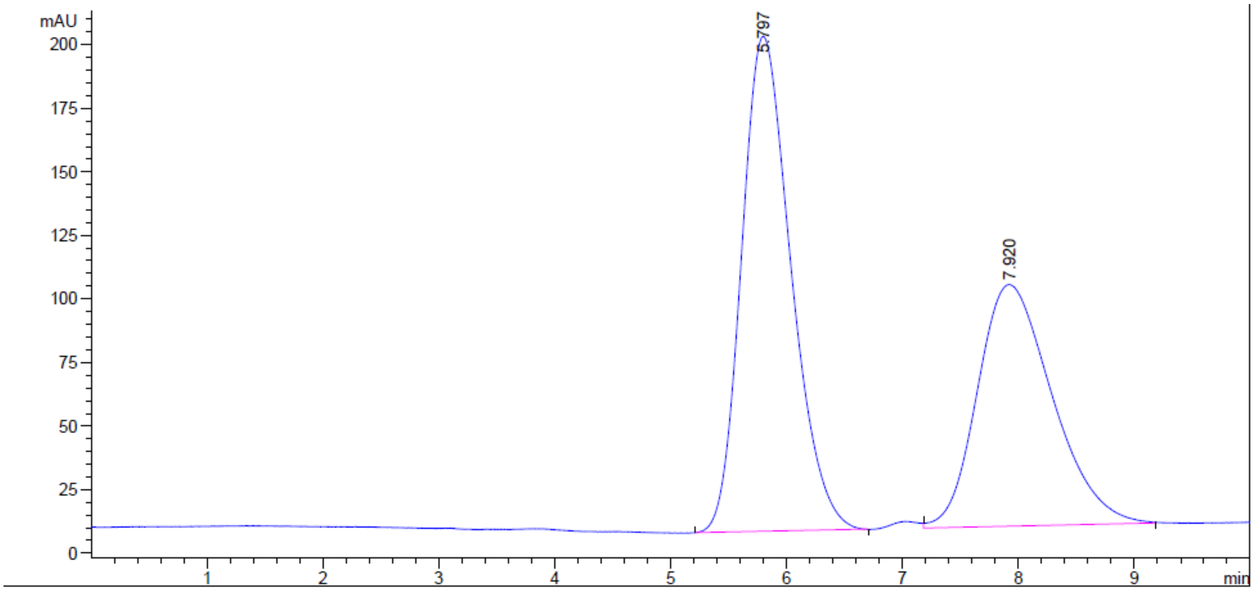

Figure S137. Chiral HPLC separation of (2*S*,1’*R*)-**20o** and (2*S*,1’*S*)-**20p** on Chiralcel OD column using heptane and 0.1% diethylamine as eluents.

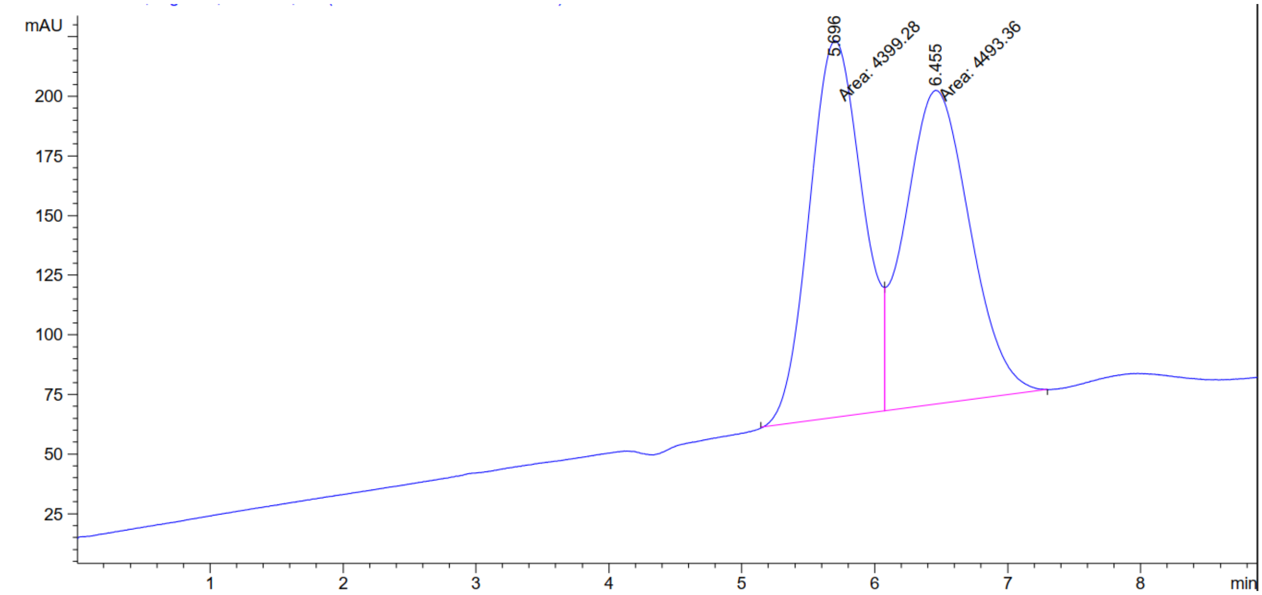

Figure S138. Chiral HPLC separation of (2*S*,1’*R*)-**20w** and (2*S*,1’*S*)-**20x** on Chiralcel OD column using heptane and 0.1% diethylamine as eluents.

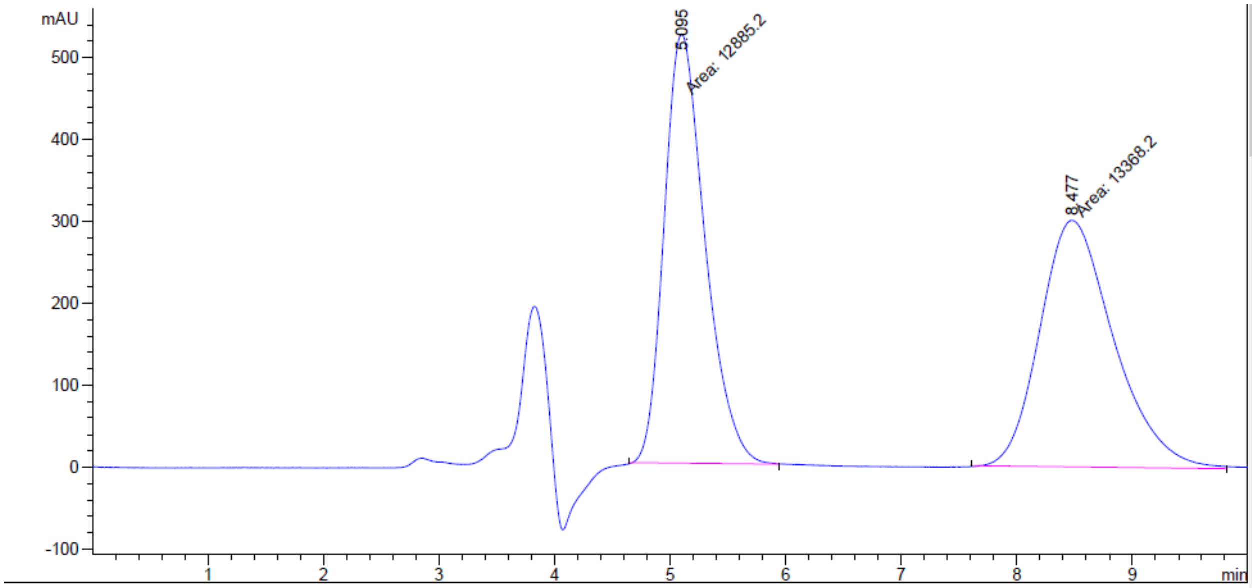

Figure S139. Chiral HPLC separation of (*S*)-**24a** and (*R*)-**24b** on Chiralcel OD-H column using 1-PrOH/heptanes (15:85) and 0.1% diethylamine as eluents.

4. VCD analysis

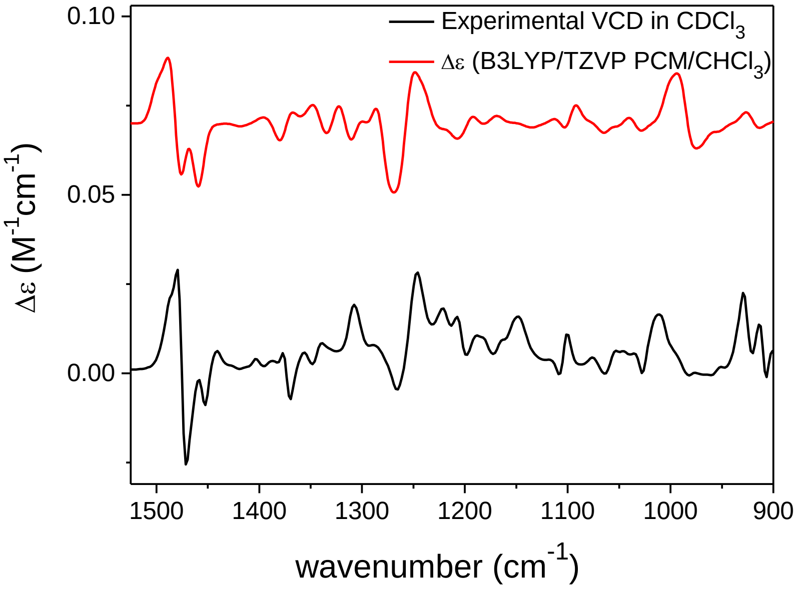

Figure S140. Experimental VCD spectrum of (5*S*,1’*S*)-**1a** in $CDCl_3$ compared with the B3LYP/TZVP PCM/$CHCl_3$ // B3LYP/TZVP PCM/$CHCl_3$ VCD spectrum of (5*S*,1’*S*)-**1a**.

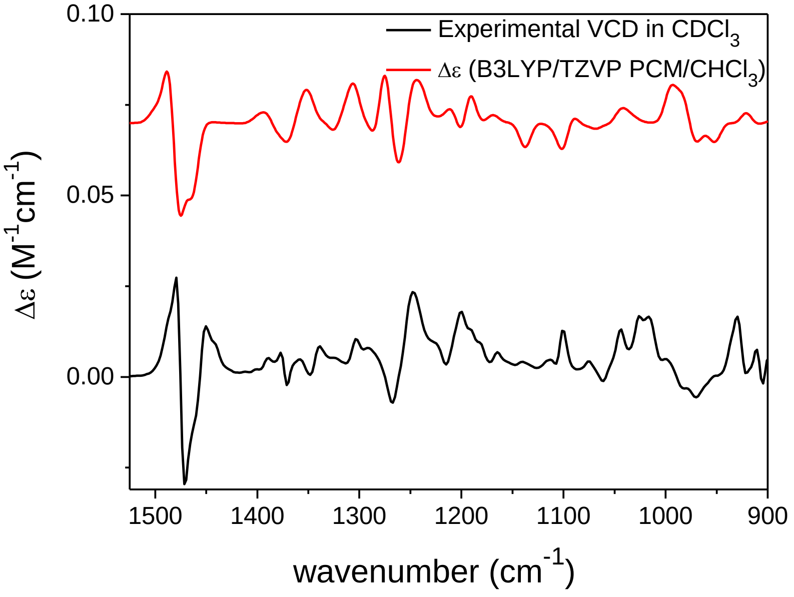

Figure S141. Experimental VCD spectrum of (5*S*,1’*R*)-**1b** in $CDCl_3$ compared with the B3LYP/TZVP PCM/$CHCl_3$ // B3LYP/TZVP PCM/$CHCl_3$ VCD spectrum of (5*S*,1’*R*)-**1b**.

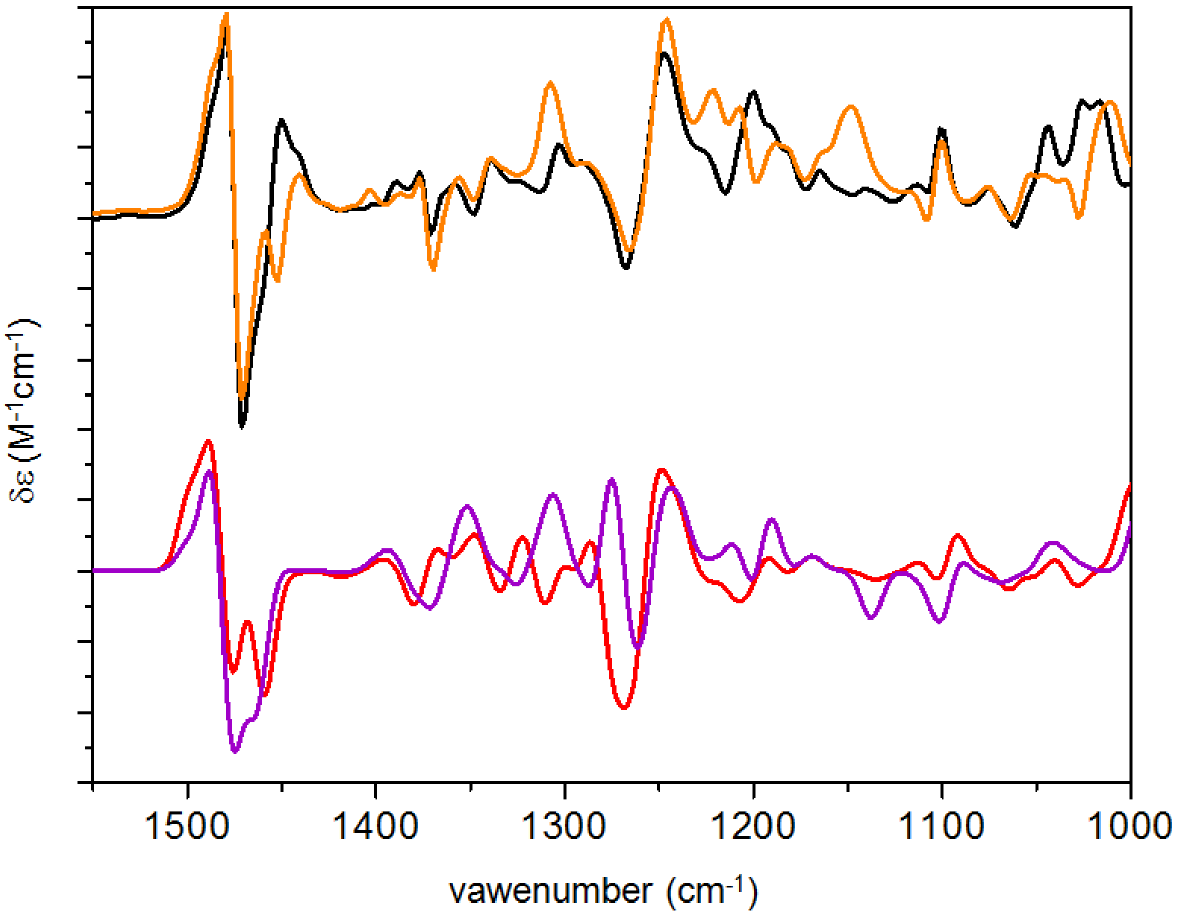

Figure S142. Experimental VCD spectra of (5*S*,1'*S*)-**1a** (black) and (5*S*,1'*R*)-**1b** (orange) in $CDCl_3$ compared with the B3LYP/TZVP PCM/$CHCl_3$ // B3LYP/TZVP PCM/$CHCl_3$ VCD spectra of (5*S*,1'*S*)-**1a** (red) and (5*S*,1'*R*)-**1b** (purple).

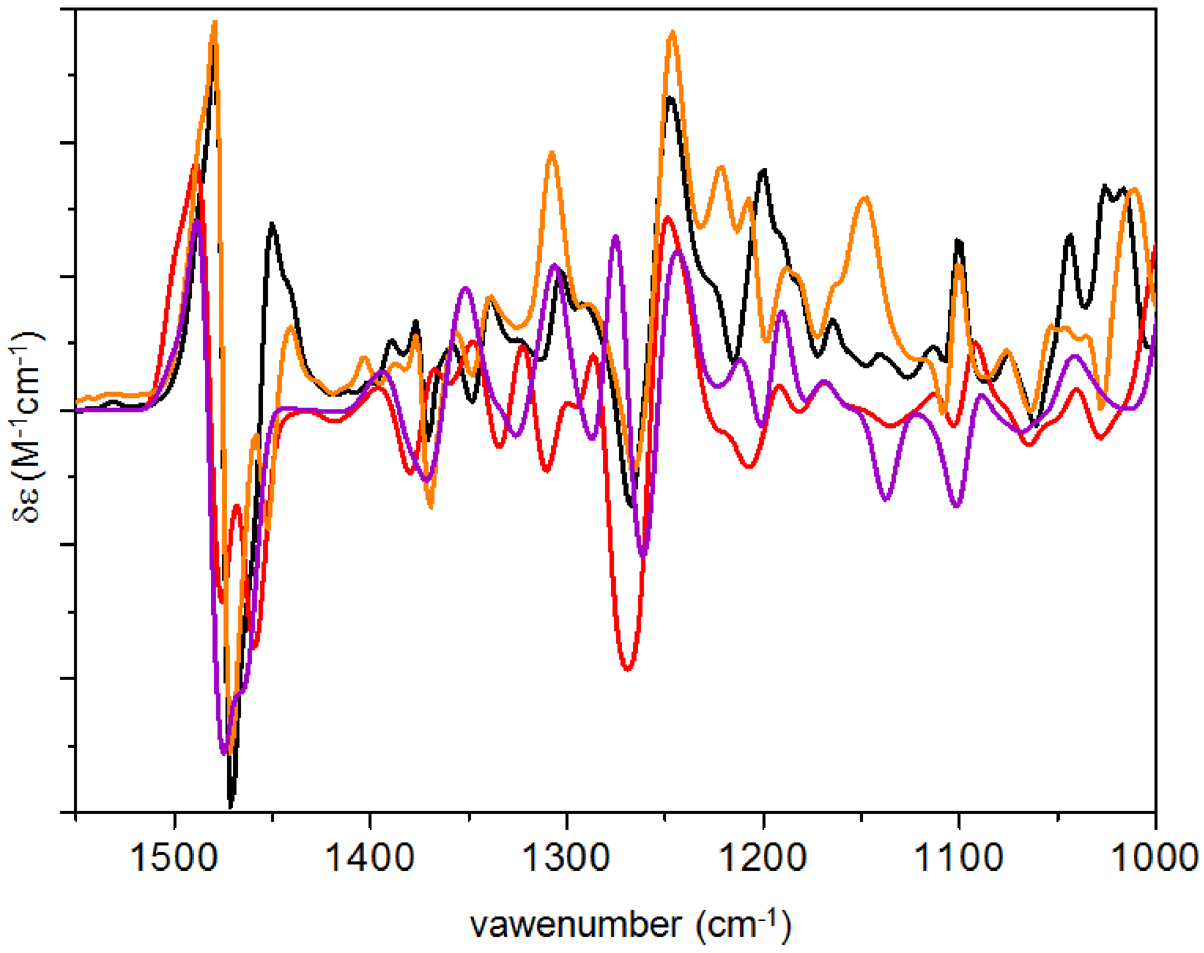

Figure S143. Experimental VCD spectra of (5*S*,1'*S*)-**1a** (black) and (5*S*,1'*R*)-**1b** (orange) in $CDCl_3$ compared with the B3LYP/TZVP PCM/$CHCl_3$ // B3LYP/TZVP PCM/$CHCl_3$ VCD spectra of (5*S*,1'*S*)-**1a** (red) and (5*S*,1'*R*)-**1b** (purple).

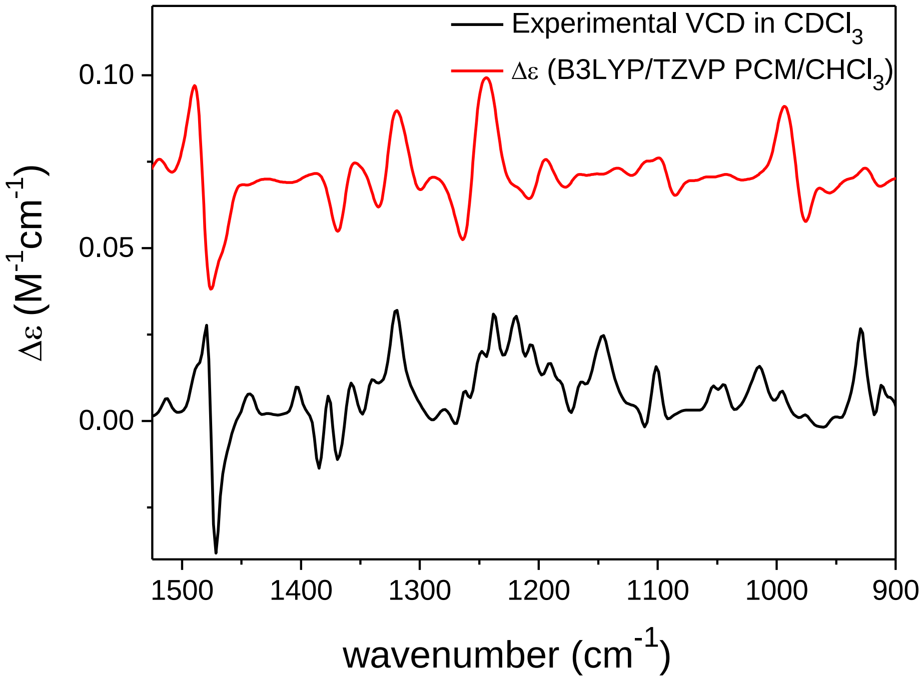

Figure S144. Experimental VCD spectrum of (5*S*,1'*S*)-**2a** in $CDCl_3$ compared with the B3LYP/TZVP PCM/$CHCl_3$ // B3LYP/TZVP PCM/$CHCl_3$ VCD spectrum of (5*S*,1'*S*)-**2a**.

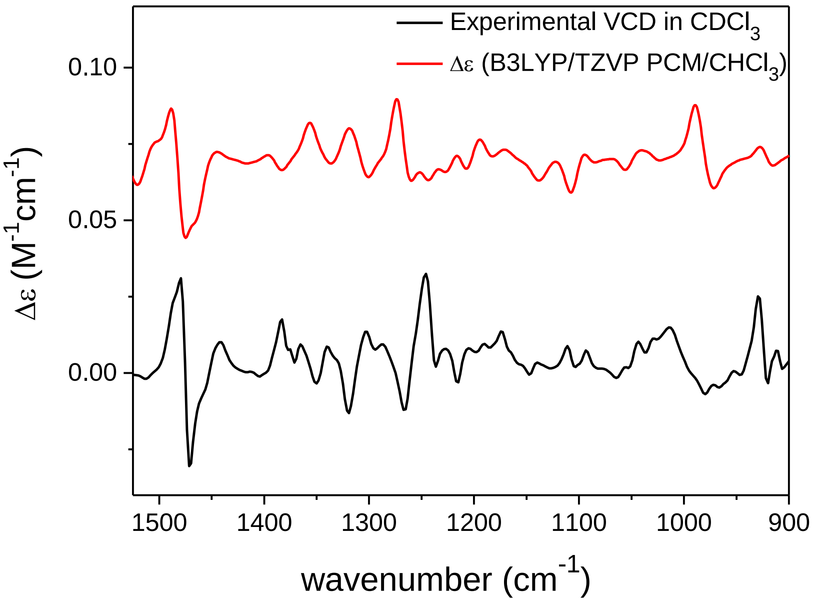

Figure S145. Experimental VCD spectrum of (5*S*,1'*R*)-**2b** in $CDCl_3$ compared with the B3LYP/TZVP PCM/$CHCl_3$ // B3LYP/TZVP PCM/$CHCl_3$ VCD spectrum of (5*S*,1'*R*)-**2b**.

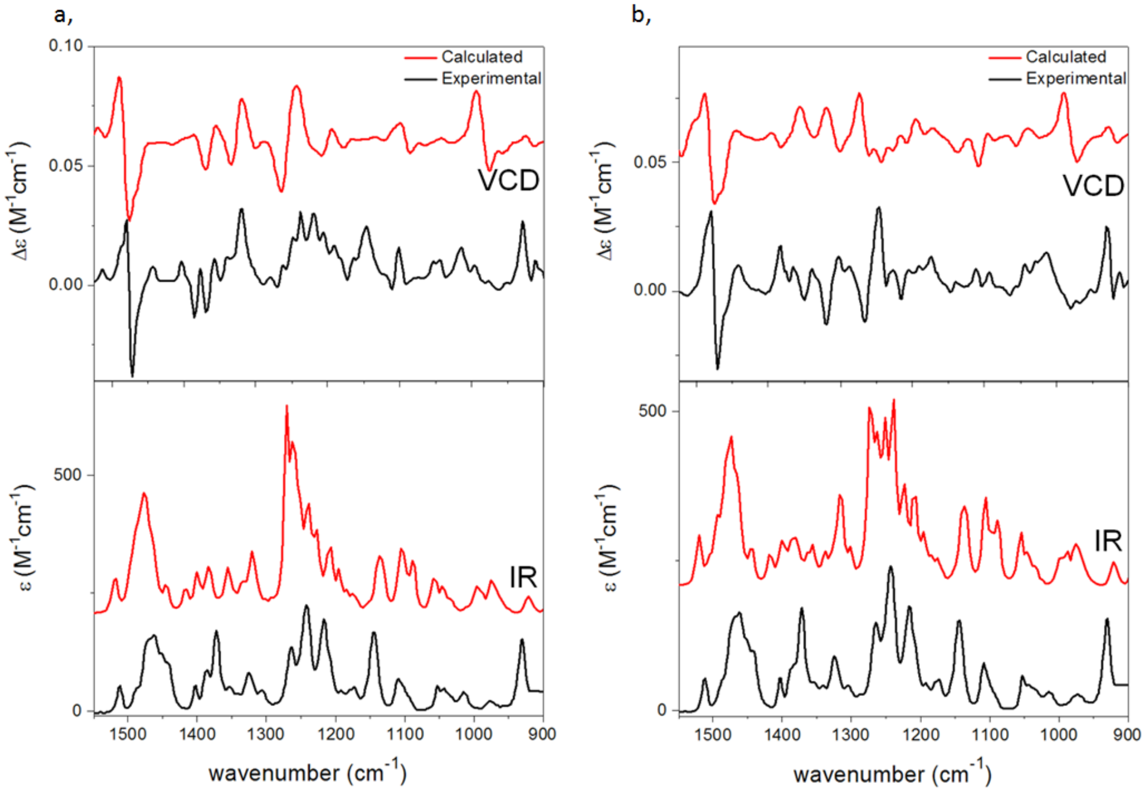

Figure S146. Experimental VCD (upper black, $CDCl_3$) and IR spectra (lower black, $CDCl_3$) of a) (5*S*,1'*S*)-**2a** and b) (5*S*,1'*R*)-**2b** compared with the B3LYP/TZPV VCD (upper red) and IR (lower red) spectra.

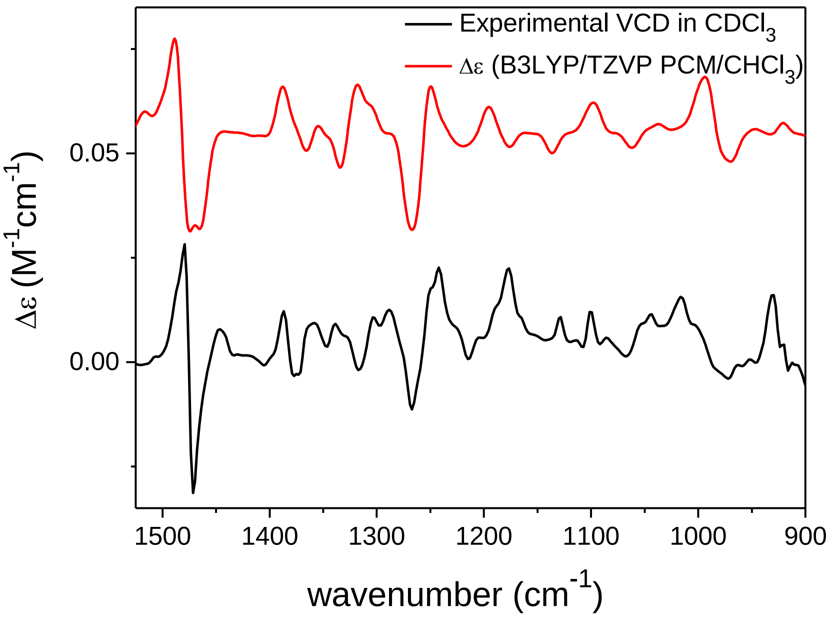

Figure S147. Experimental VCD spectrum of (5*S*,1'*S*)-**3a** in $CDCl_3$ compared with the B3LYP/TZVP PCM/$CHCl_3$ // B3LYP/TZVP PCM/$CHCl_3$ VCD spectrum of (5*S*,1'*S*)-**3a**.

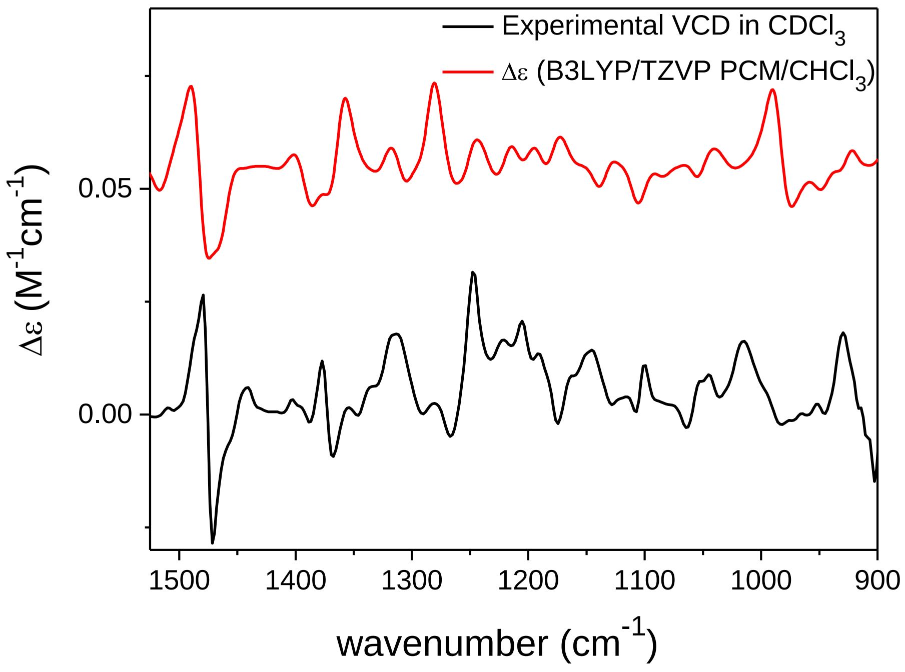

Figure S148. Experimental VCD spectrum of (5*S*,1'*R*)-**3b** in $CDCl_3$ compared with the B3LYP/TZVP PCM/$CHCl_3$ // B3LYP/TZVP PCM/$CHCl_3$ VCD spectrum of (5*S*,1'*R*)-**3b**.

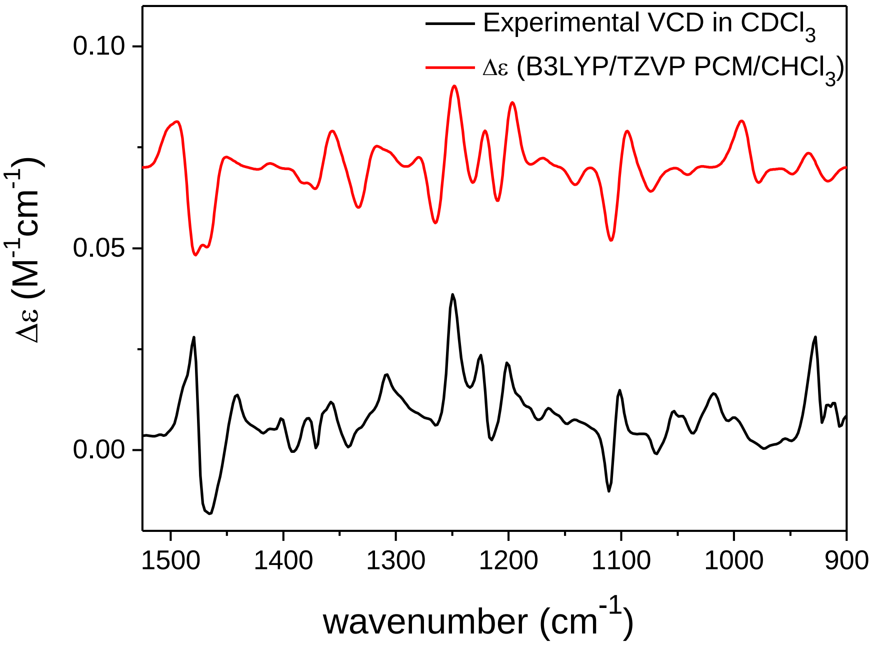

Figure S149. Experimental VCD spectrum of (5*S*,1'*S*)-**4a** in $CDCl_3$ compared with the B3LYP/TZVP PCM/$CHCl_3$ // B3LYP/TZVP PCM/$CHCl_3$ VCD spectrum of (5*S*,1'*S*)-**4a**.

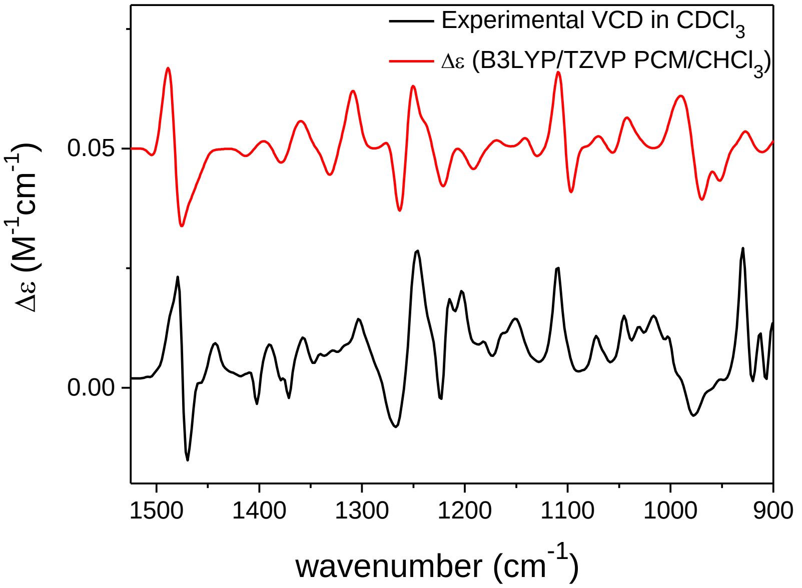

Figure S150. Experimental VCD spectrum of (5*S*,1'*R*)-**4b** in $CDCl_3$ compared with the B3LYP/TZVP PCM/$CHCl_3$ // B3LYP/TZVP PCM/$CHCl_3$ VCD spectrum of (5*S*,1'*R*)-**4b**.

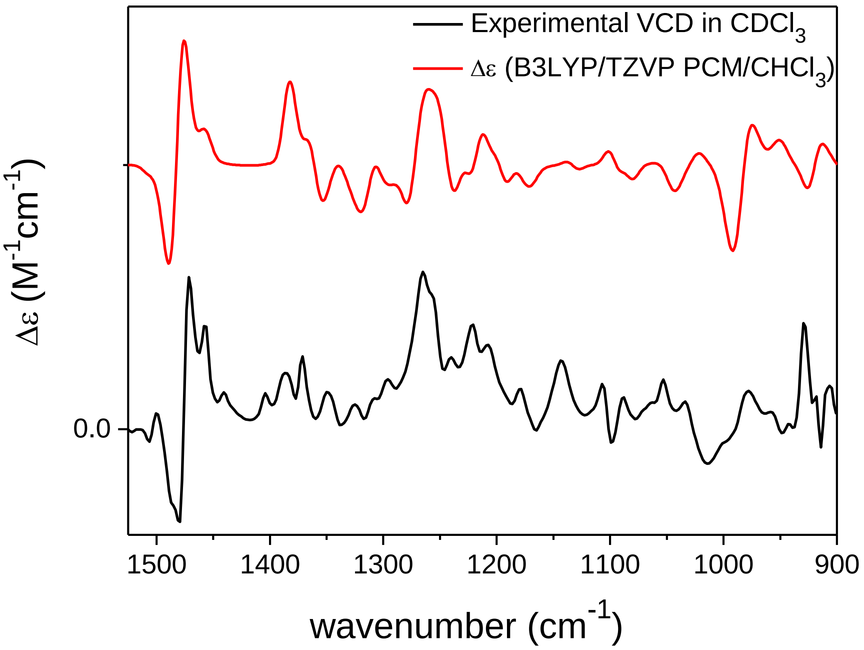

Figure S151. Experimental VCD spectrum of (5*R*,1'*R*)-**5a** in $CDCl_3$ compared with the B3LYP/TZVP PCM/$CHCl_3$ // B3LYP/TZVP PCM/$CHCl_3$ VCD spectrum of (5*R*,1'*R*)-**5a**.

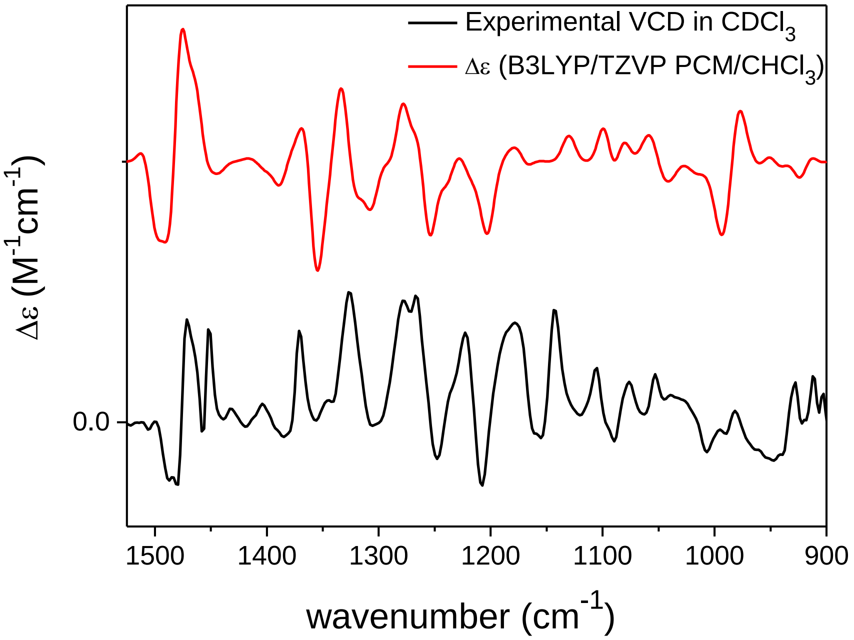

Figure S152. Experimental VCD spectrum of (5*R*,1'*S*)-**5b** in $CDCl_3$ compared with the B3LYP/TZVP PCM/$CHCl_3$ // B3LYP/TZVP PCM/$CHCl_3$ VCD spectrum of (5*R*,1'*S*)-**5b**.

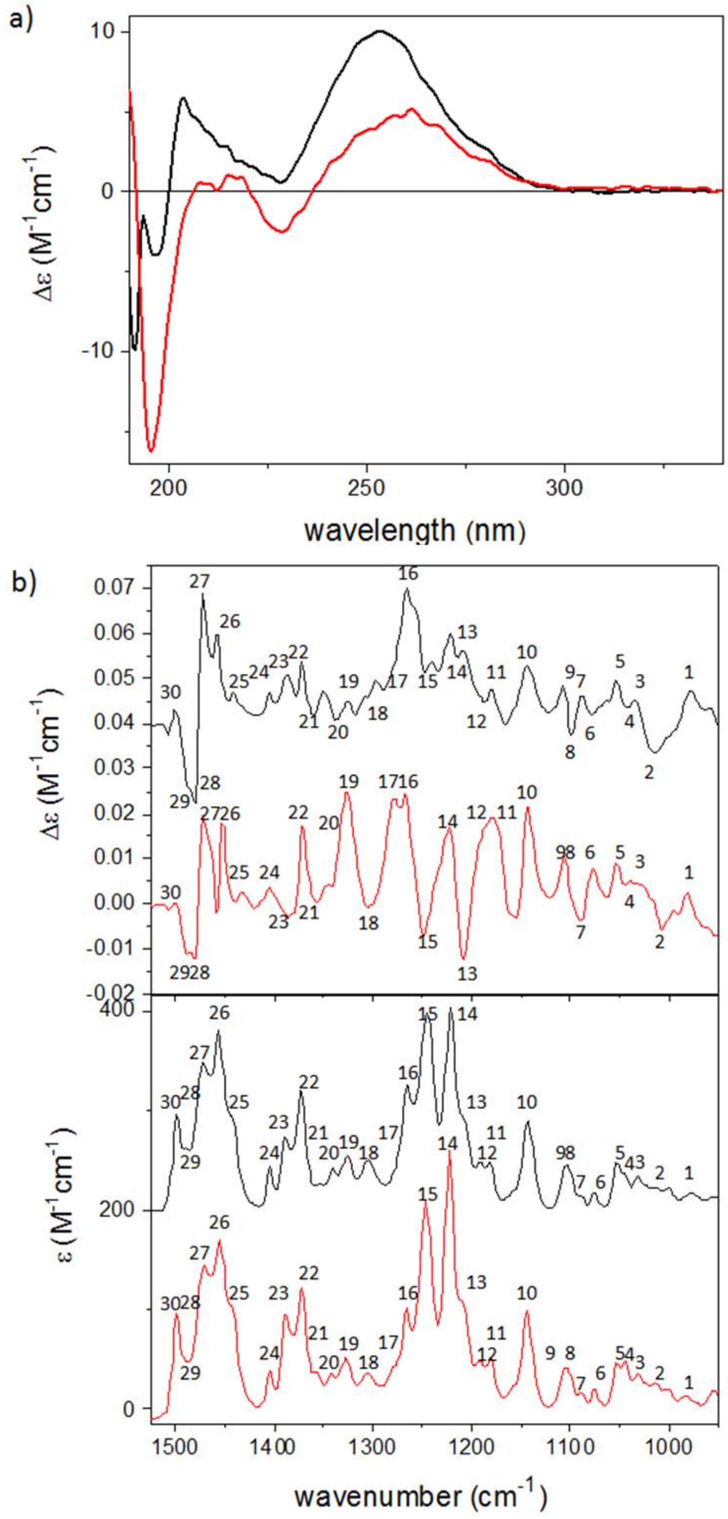

Figure S153. a) Overlapped experimental ECD spectra of (5*R*,1’*R*)-**5a** (black) and (5*R*,1’*S*)-**5b** (red) in MeCN. b) Experimental VCD spectra of (5*R*,1’*R*)-**5a** (black) and (5*R*,1’*S*)-**5b** (red) in $CDCl_3$.

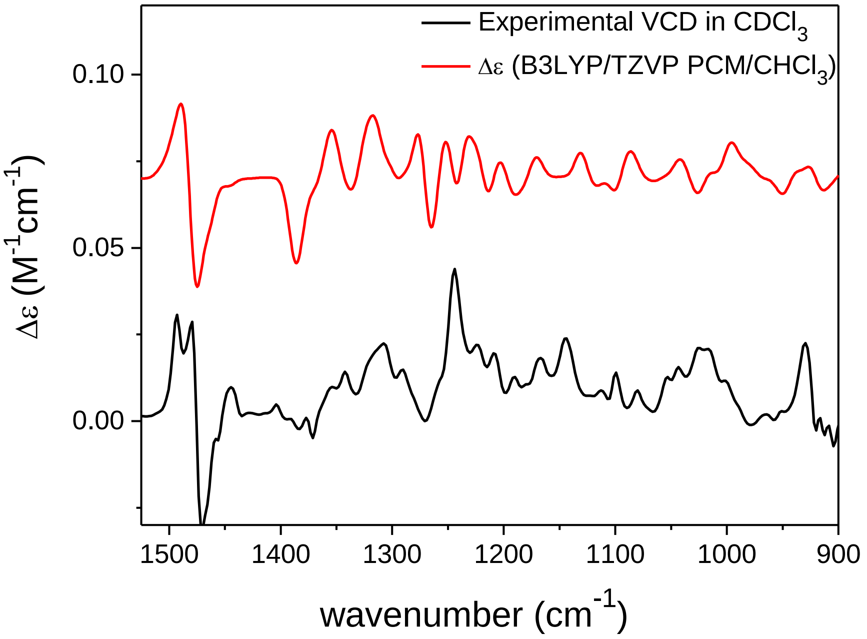

Figure S154. Experimental VCD spectrum of (5*S*,1'*S*)-**8a** in $CDCl_3$ compared with the B3LYP/TZVP PCM/$CHCl_3$ // B3LYP/TZVP PCM/$CHCl_3$ VCD spectrum of (5*S*,1'*S*)-**8a**.

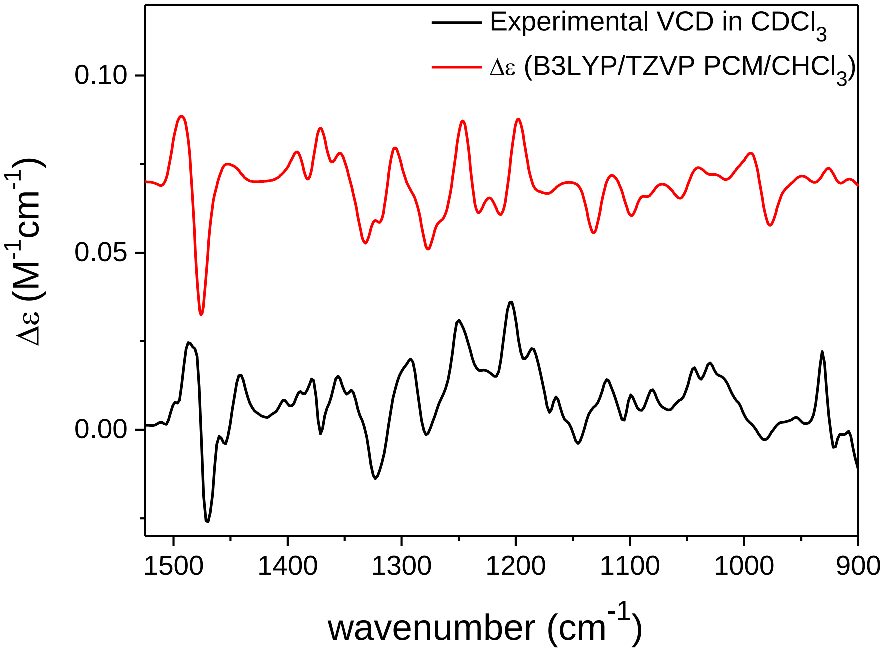

Figure S155. Experimental VCD spectrum of (5*S*,1'*R*)-**8b** in $CDCl_3$ compared with the B3LYP/TZVP PCM/$CHCl_3$ // B3LYP/TZVP PCM/$CHCl_3$ VCD spectrum of (5*S*,1'*R*)-**8b**.

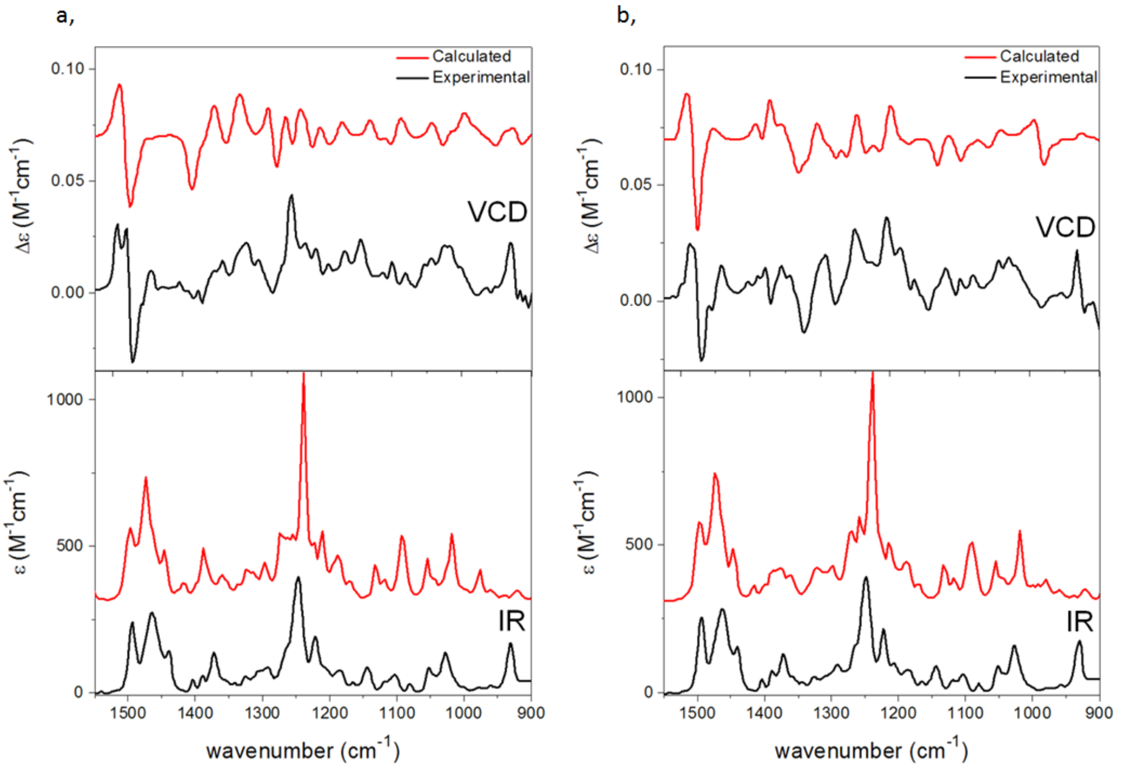

Figure S156. Experimental VCD (upper black, $CDCl_3$) and IR spectra (lower black, $CDCl_3$) of a) (5*S*,1'*S*)-**8a** and b) (5*S*,1'*R*)-**8b** compared with the B3LYP/TZPV VCD (upper red) and IR (lower red) spectra.

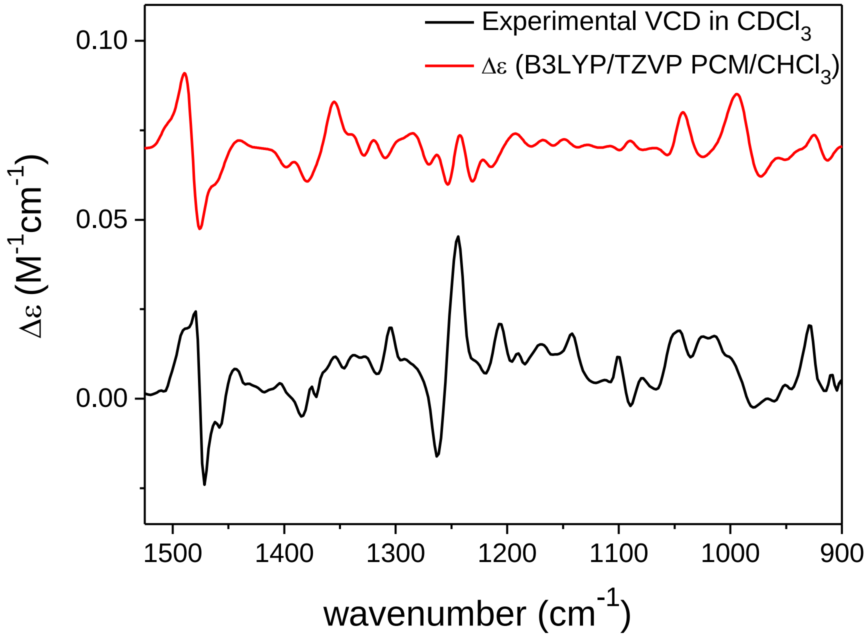

Figure S157. Experimental VCD spectrum of (5*S*,1'*S*)-**9a** in $CDCl_3$ compared with the B3LYP/TZVP PCM/$CHCl_3$ // B3LYP/TZVP PCM/$CHCl_3$ VCD spectrum of (5*S*,1'*S*)-**9a**.

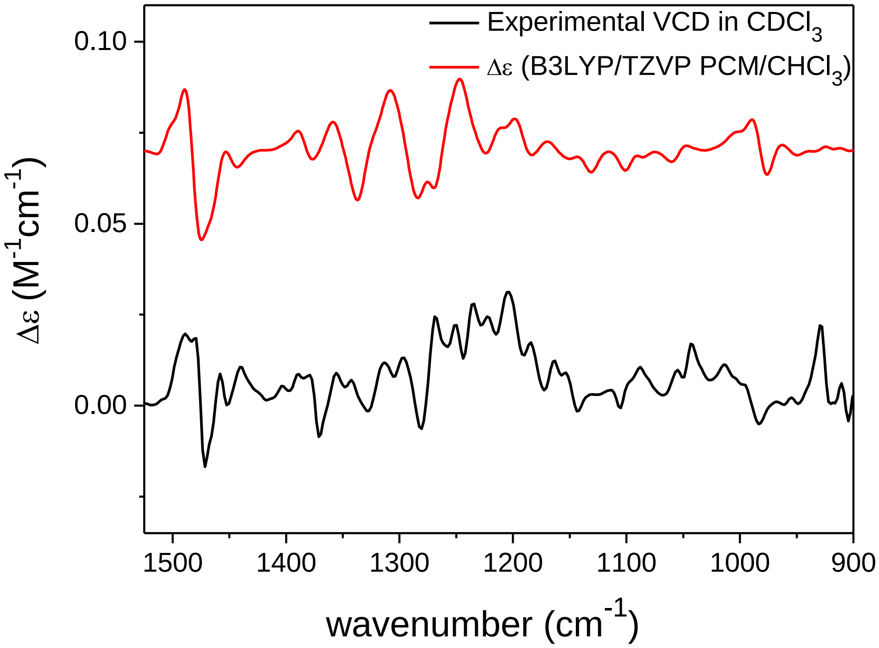

Figure S158. Experimental VCD spectrum of (5*S*,1'*R*)-**9b** in $CDCl_3$ compared with the B3LYP/TZVP PCM/$CHCl_3$ // B3LYP/TZVP PCM/$CHCl_3$ VCD spectrum of (5*S*,1'*R*)-**9b**.

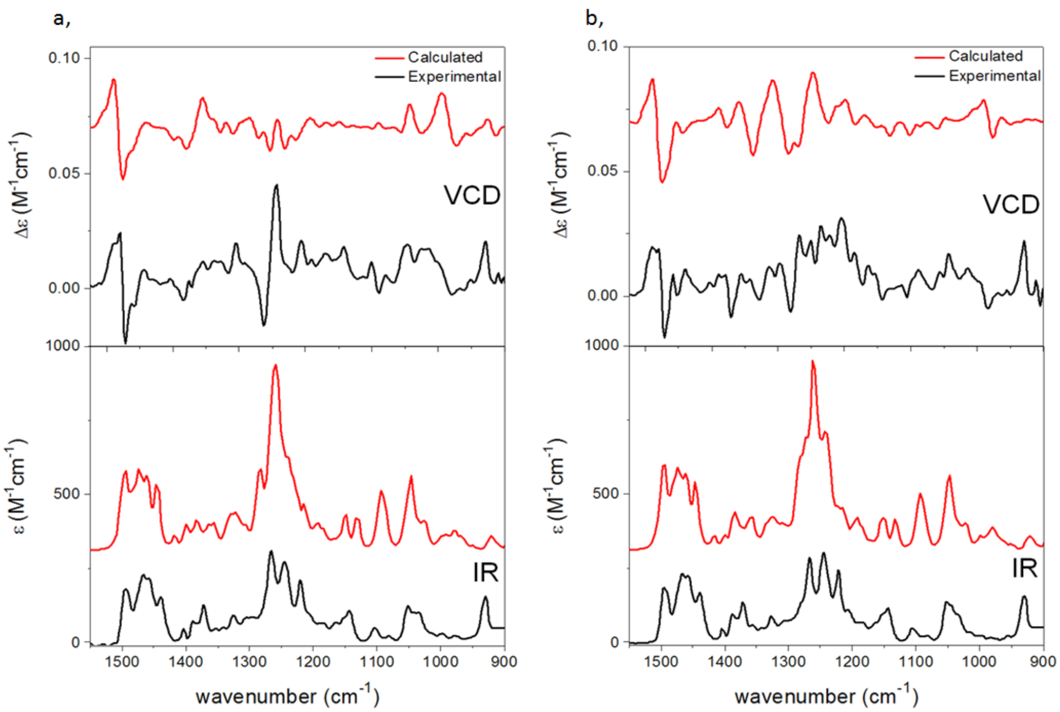

Figure S159. Experimental VCD (upper black, $CDCl_3$) and IR spectra (lower black, $CDCl_3$) of a) (5*S*,1'*S*)-**9a** and b) (5*S*,1'*R*)-**9b** compared with the B3LYP/TZPV VCD (upper red) and IR (lower red) spectra.

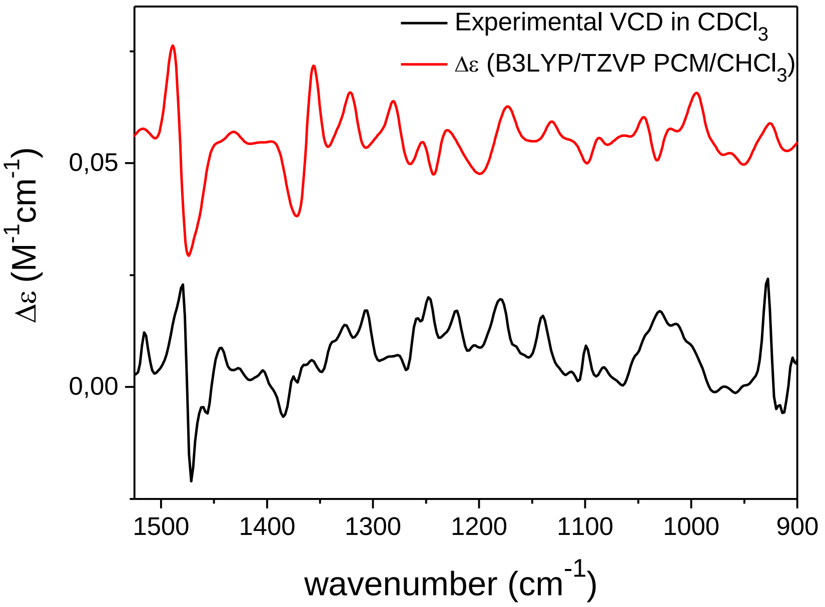

Figure S160. Experimental VCD spectrum of (5*S*,1'*S*)-**10a** in $CDCl_3$ compared with the B3LYP/TZVP PCM/$CHCl_3$ // B3LYP/TZVP PCM/$CHCl_3$ VCD spectrum of (5*S*,1'*S*)-**10a**.

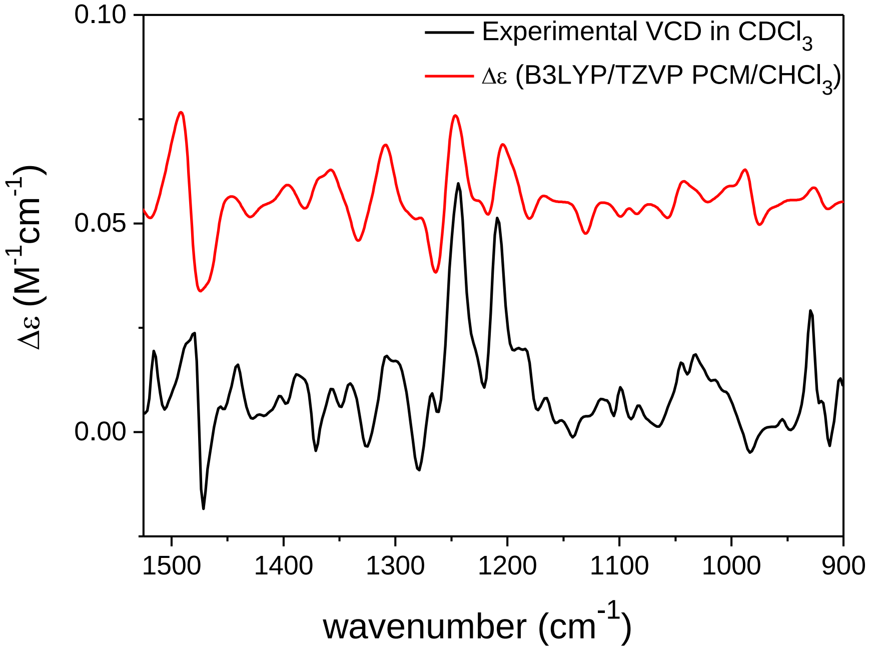

Figure S161. Experimental VCD spectrum of (5*S*,1'*R*)-**10b** in $CDCl_3$ compared with the B3LYP/TZVP PCM/$CHCl_3$ // B3LYP/TZVP PCM/$CHCl_3$ VCD spectrum of (5*S*,1'*R*)-**10b**.

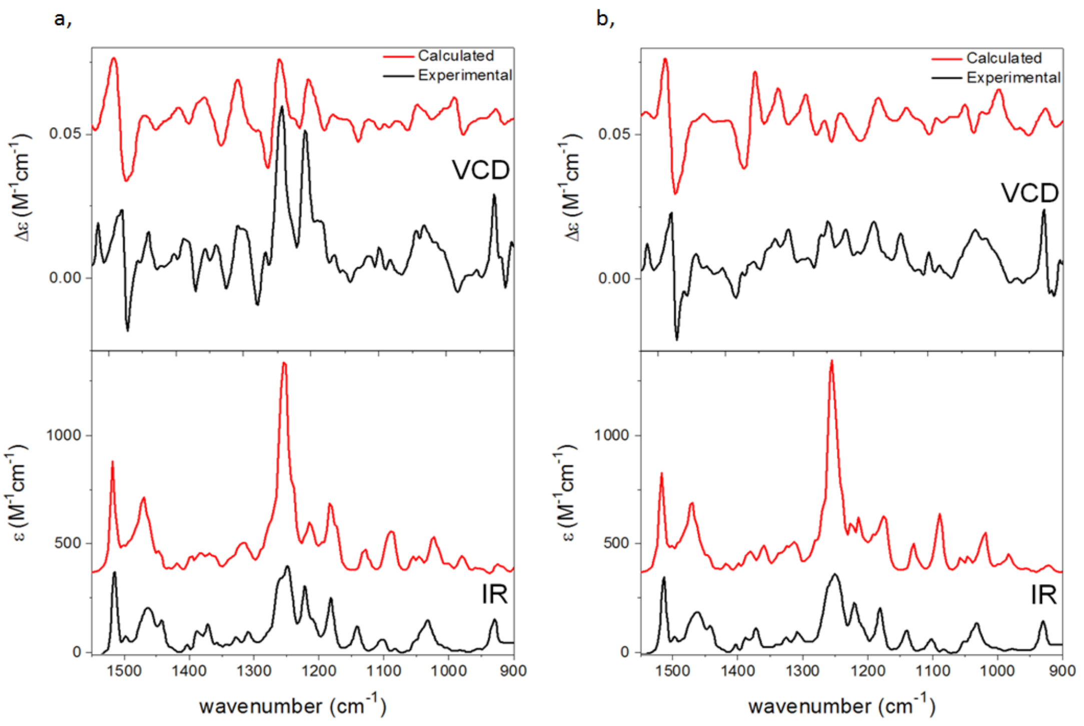

Figure S162. Experimental VCD (upper black, $CDCl_3$) and IR spectra (lower black, $CDCl_3$) of a) (5*S*,1'*S*)-**10a** and b) (5*S*,1'*R*)-**10b** compared with the B3LYP/TZPV VCD (upper red) and IR (lower red) spectra.

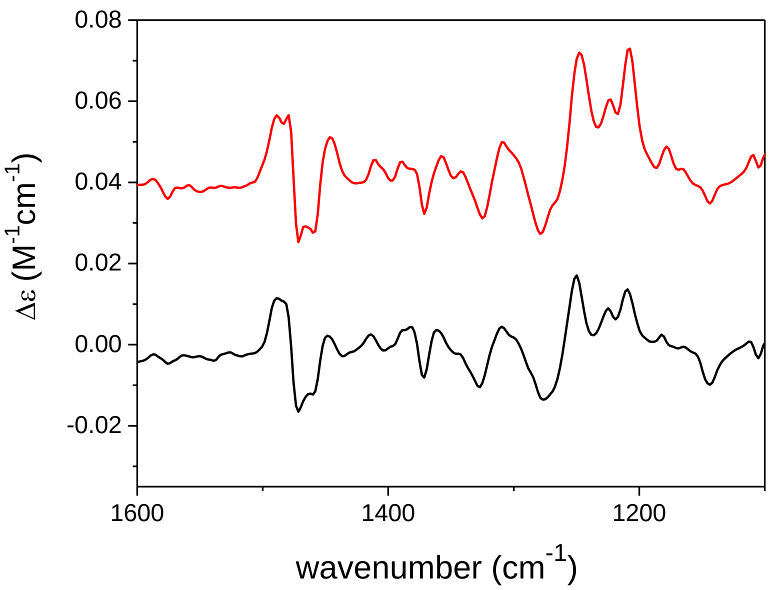

Figure S163. Experimental VCD spectra of (5*S*,1'*R*)-**15b** recorded in DMSO-d6 (black) and $CDCl_3$ (red).

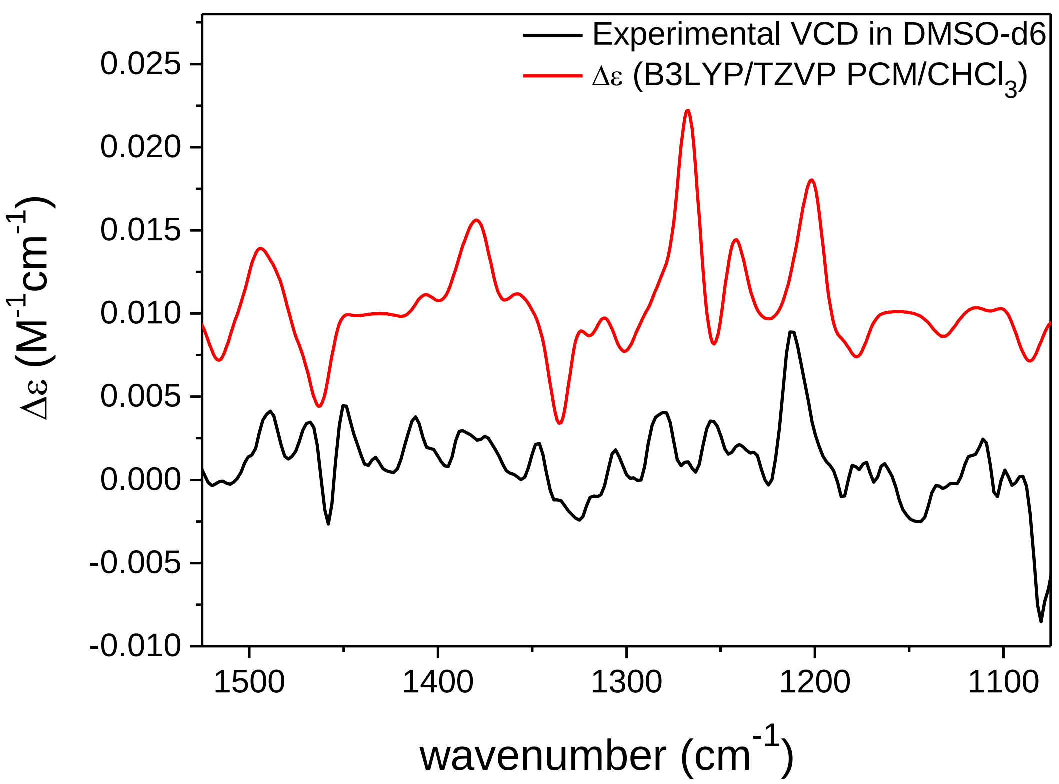

Figure S164. Experimental VCD spectrum of (*R*)-**17a** in DMSO-d6 compared with the B3LYP/TZVP PCM/DMSO // B3LYP/TZVP PCM/$CHCl_3$ VCD spectrum of (*R*)-**17a**.

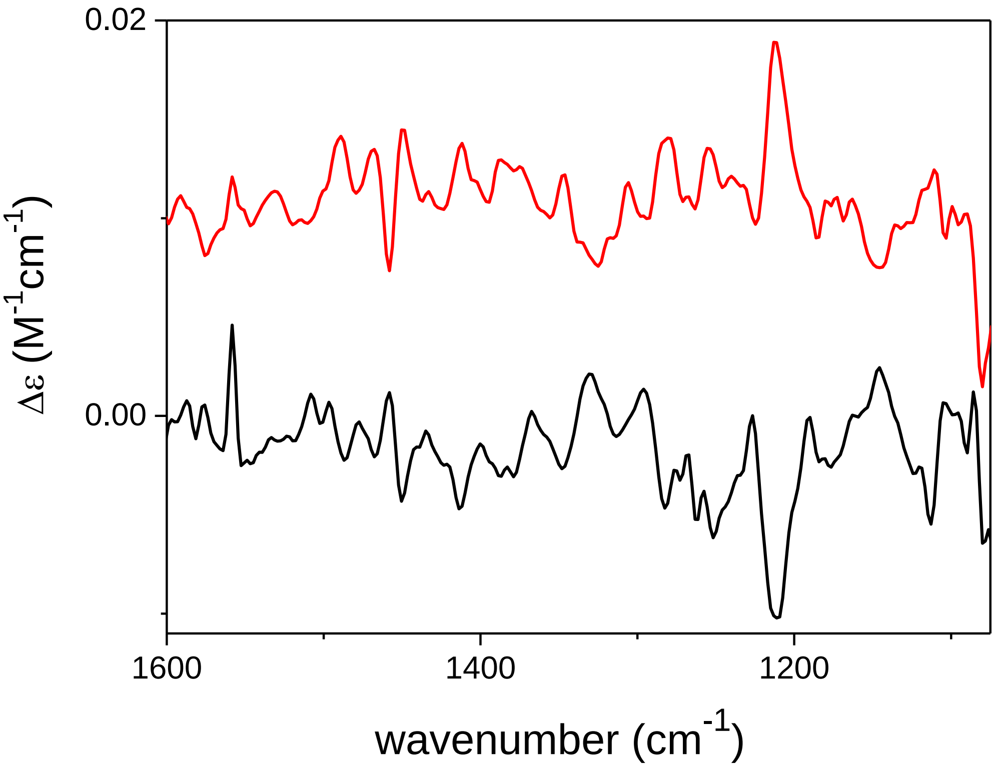

Figure S165. Experimental VCD spectra of (*S*)-**17b** (red) and (*R*)-**17a** (black) in DMSO-d6.

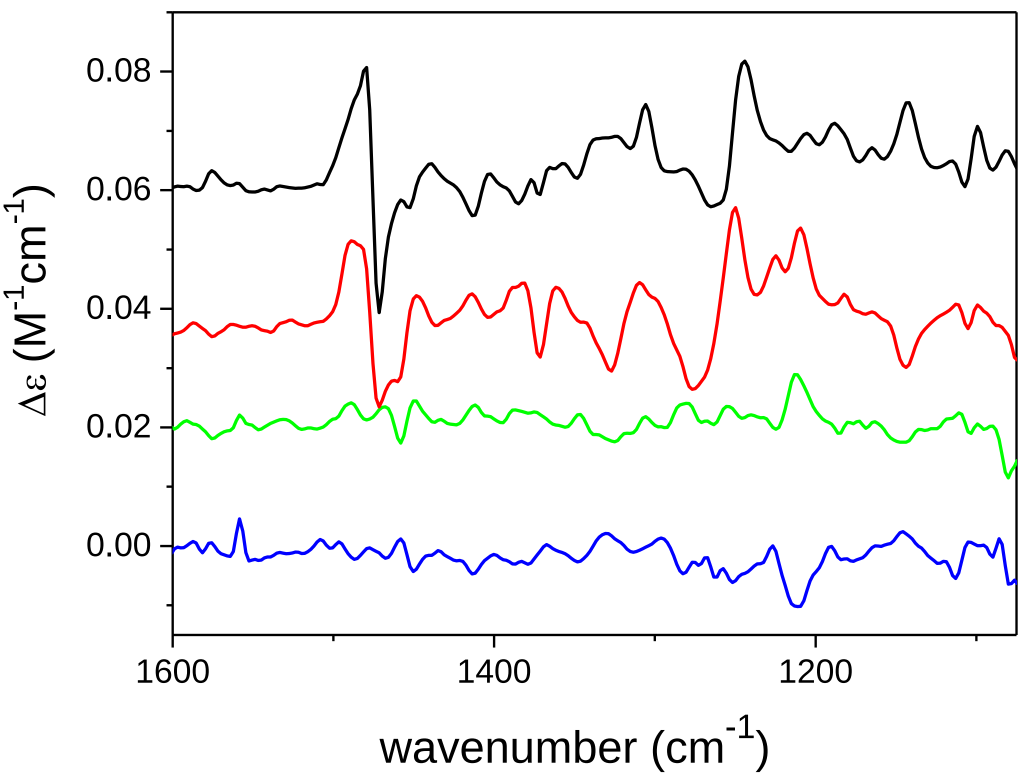

Figure S166. Experimental VCD spectra of (5*S*,1'*S*)-**15a** ($CDCl_3$, black), (5*S*,1'*R*)-**15b** (DMSO d-6, red), (*S*)-**17b** (DMSO-d6, green) and (*R*)-**17b** (DMSO-d6, blue).

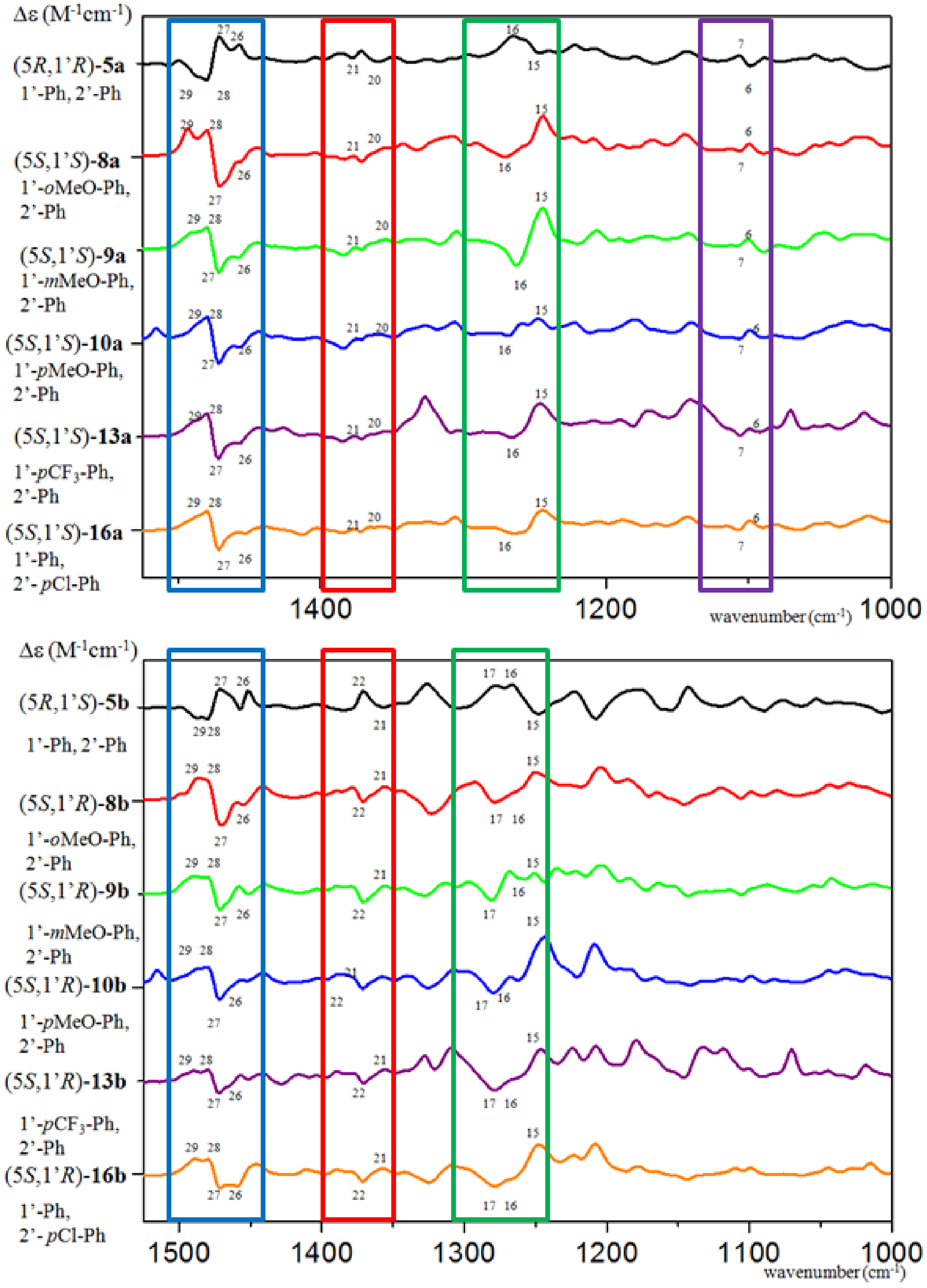

Figure S167. a) Experimental VCD spectra of (5*R*,1'*R*)-**5a** (black), (5*S*,1'*S*)-**8a** (red), (5*S*,1'*S*)-**9a** (green) (5*S*,1'*S*)-**10a** (blue), (5*S*,1'*S*)-**13a** and (5*S*,1'*S*)-**16a** (orange). b) Experimental VCD spectra of (5*R*,1'*S*)-**5a** (black), (5*S*,1'*R*)-**8a** (red), (5*S*,1'*R*)-**9a** (green) (5*S*,1'*R*)-**10a** (blue), (5*S*,1'*R*)-**13a** and (5*S*,1'*R*)-**16a** (orange).

5. ECD analysis

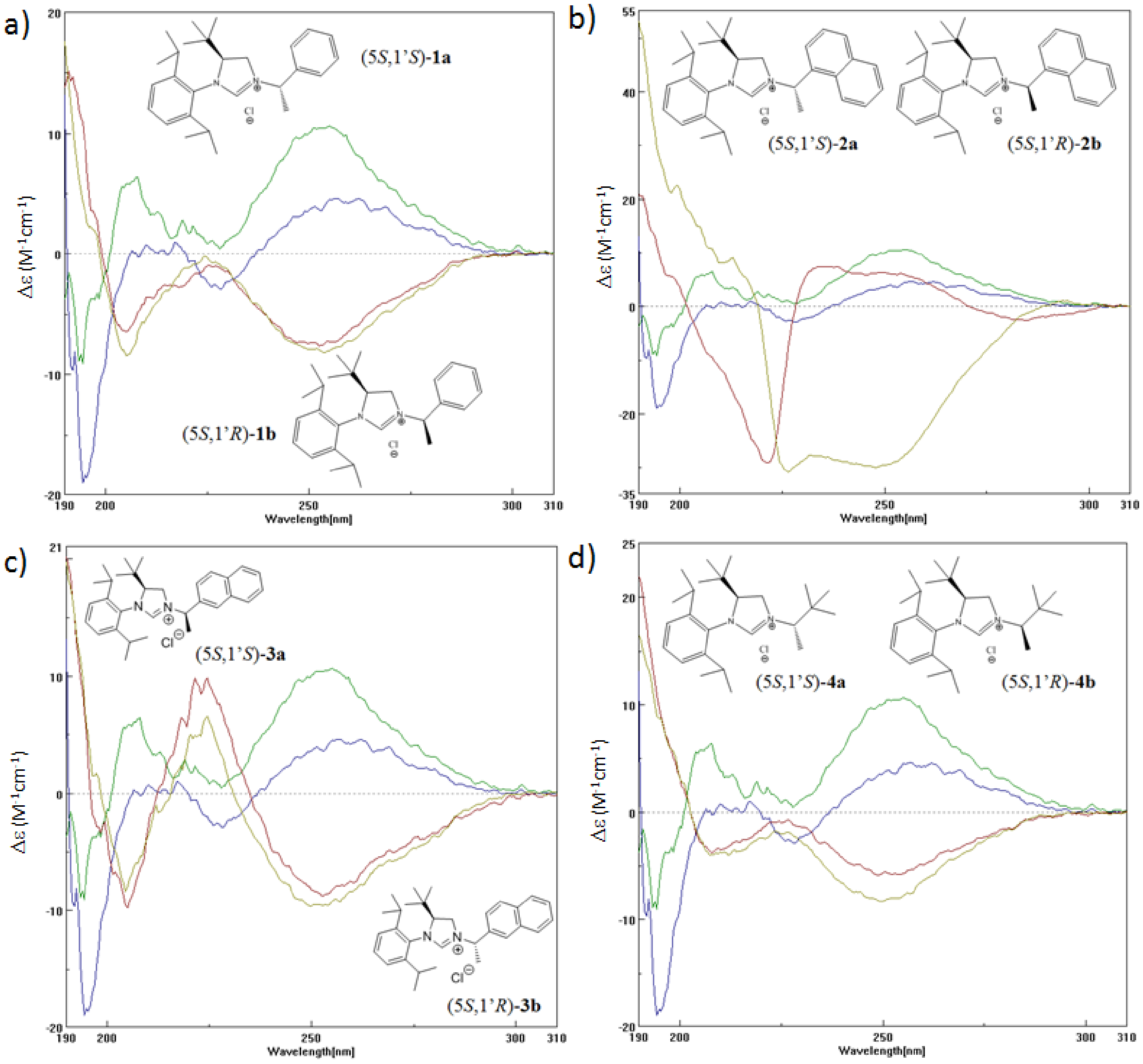

Figure S168. a) Experimental ECD spectra of (5*S*,1'*S*)-**1a** (brown) and (5*S*,1'*R*)-**1b** (yellow) compared with the reference ECD spectra of (5*R*,1'*R*)-**5a** (green) and (5*R*,1'*S*)-**5b** (blue). b) Experimental ECD spectra of (5*S*,1'*S*)-**2a** (brown) and (5*S*,1'*R*)-**2b** (yellow) compared with the reference ECD spectra of (5*R*,1'*R*)-**5a** (green) and (5*R*,1'*S*)-**5b** (blue). c) Experimental ECD spectra of (5*S*,1'*S*)-**3a** (brown) and (5*S*,1'*R*)-**3b** (yellow) compared with the reference ECD spectra of (5*R*,1'*R*)-**5a** (green) and (5*R*,1'*S*)-**5b** (blue). d) Experimental ECD spectra of (5*S*,1'*S*)-**4a** (brown) and (5*S*,1'*R*)-**4b** (yellow) compared with the reference ECD spectra of (5*R*,1'*R*)-**5a** (green) and (5*R*,1'*S*)-**5b** (blue).

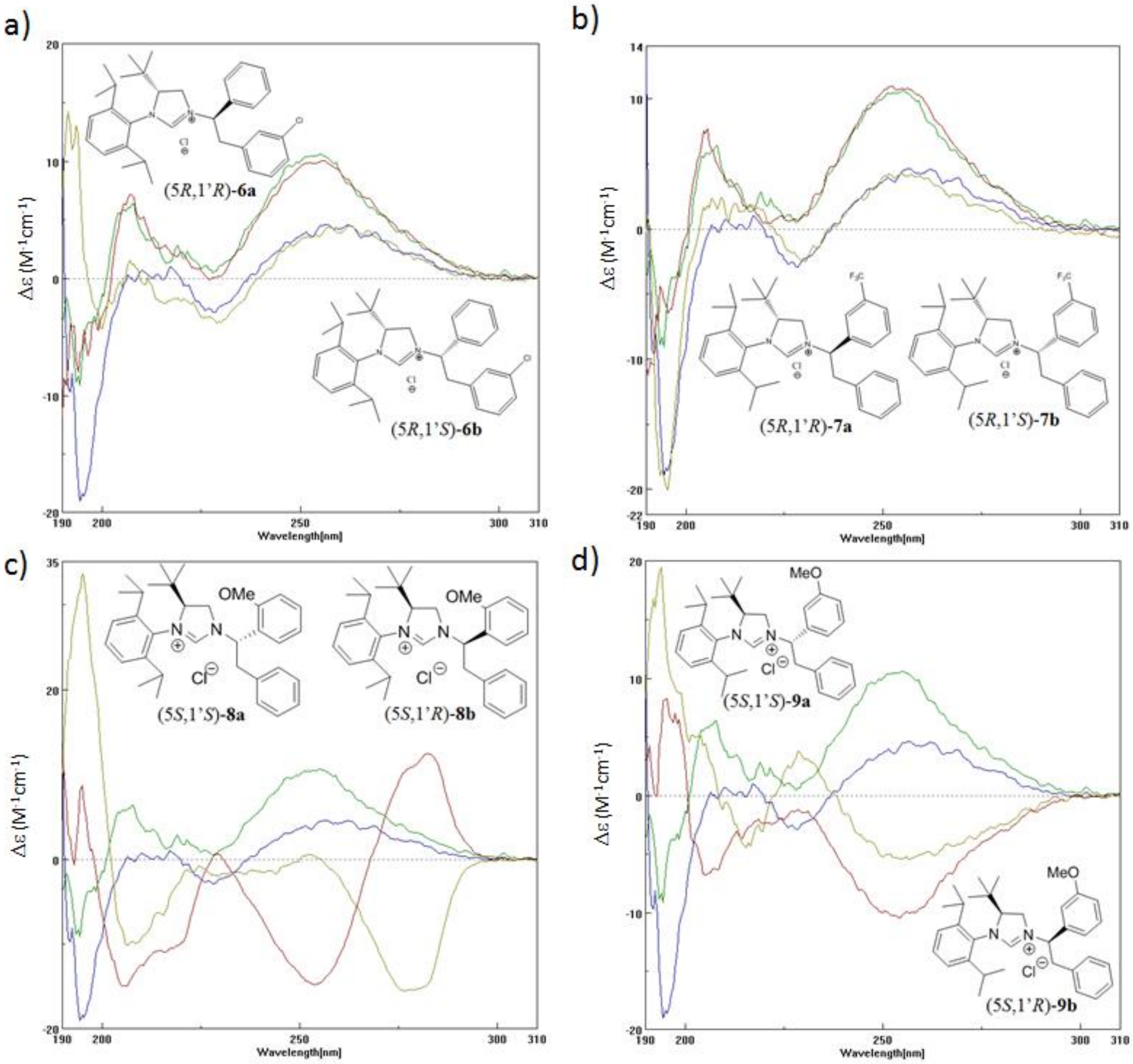

Figure S169. a) Experimental ECD spectra of (5*R*,1'*R*)-**6a** (brown) and (5*R*,1'*S*)-**6b** (yellow) compared with the reference ECD spectra of (5*R*,1'*R*)-**5a** (green) and (5*R*,1'*S*)-**5b** (blue). b) Experimental ECD spectra of (5*R*,1'*R*)-**7a** (brown) and (5*R*,1'*S*)-**7b** (yellow) compared with reference the ECD spectra of (5*R*,1'*R*)-**5a** (green) and (5*R*,1'*S*)-**5b** (blue). c) Experimental ECD spectra of (5*S*,1'*S*)-**8a** (brown) and (5*S*,1'*R*)-**8b** (yellow) compared with the reference ECD spectra of (5*R*,1'*R*)-**5a** (green) and (5*R*,1'*S*)-**5b** (blue). d) Experimental ECD spectra of (5*S*,1'*S*)-**9a** (brown) and (5*S*,1'*R*)-**9b** (yellow) compared with the reference ECD spectra of (5*R*,1'*R*)-**5a** (green) and (5*R*,1'*S*)-**5b** (blue).

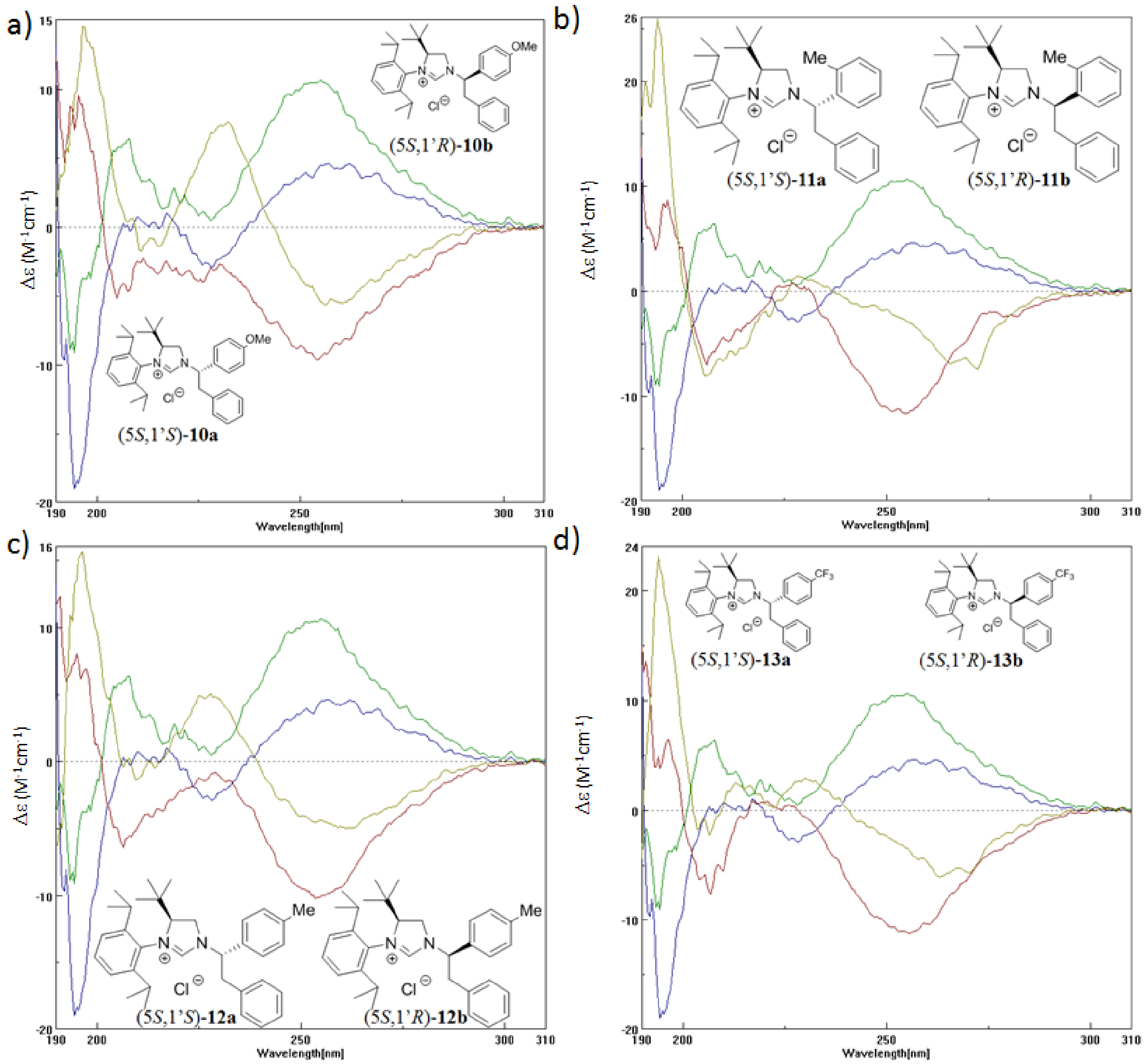

Figure S170. a) Experimental ECD spectra of (5*S*,1'*S*)-**10a** (brown) and (5*S*,1'*R*)-**10b** (yellow) compared with the reference ECD spectra of (5*R*,1'*R*)-**5a** (green) and (5*R*,1'*S*)-**5b** (blue). b) Experimental ECD spectra of (5*S*,1'*S*)-**11a** (brown) and (5*S*,1'*R*)-**11b** (yellow) compared with the reference ECD spectra of (5*R*,1'*R*)-**5a** (green) and (5*R*,1'*S*)-**5b** (blue). c) Experimental ECD spectra of (5*S*,1'*S*)-**12a** (brown) and (5*S*,1'*R*)-**12b** (yellow) compared with the reference ECD spectra of (5*R*,1'*R*)-**5a** (green) and (5*R*,1'*S*)-**5b** (blue). d) Experimental ECD spectra of (5*S*,1'*S*)-**13a** (brown) and (5*S*,1'*R*)-**13b** (yellow) compared with the reference ECD spectra of (5*R*,1'*R*)-**5a** (green) and (5*R*,1'*S*)-**5b** (blue).

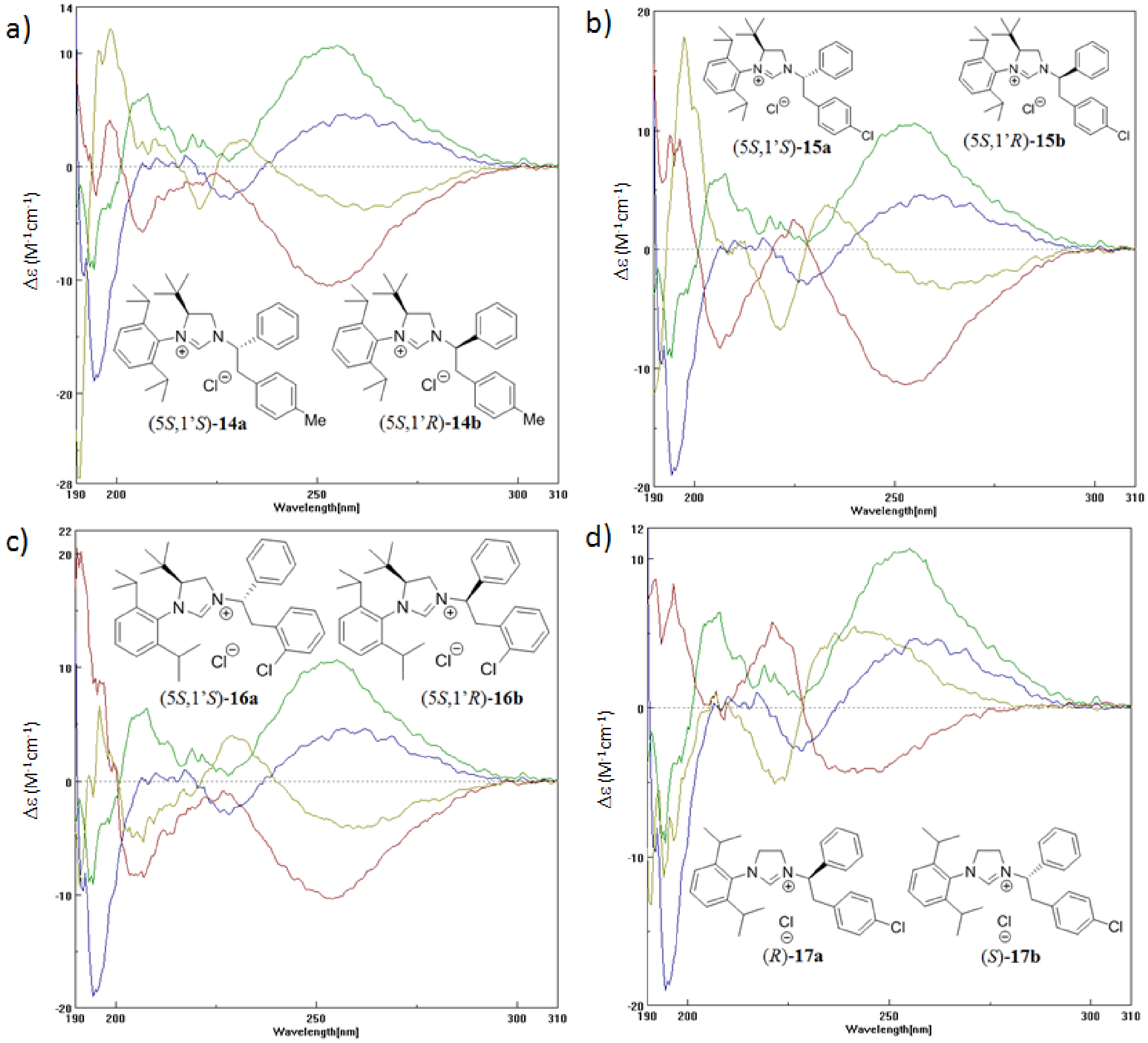

Figure S171. a) Experimental ECD spectra of (5*S*,1'*S*)-**14a** (brown) and (5*S*,1'*R*)-**14b** (yellow) compared with the reference ECD spectra of (5*R*,1'*R*)-**5a** (green) and (5*R*,1'*S*)-**5b** (blue). b) Experimental ECD spectra of (5*S*,1'*S*)-**15a** (brown) and (5*S*,1'*R*)-**15b** (yellow) compared with the reference ECD spectra of (5*R*,1'*R*)-**5a** (green) and (5*R*,1'*S*)-**5b** (blue). c) Experimental ECD spectra of (5*S*,1'*S*)-**16a** (brown) and (5*S*,1'*R*)-**16b** (yellow) compared with the reference ECD spectra of (5*R*,1'*R*)-**5a** (green) and (5*R*,1'*S*)-**5b** (blue). d) Experimental ECD spectra of (*R*)-**17a** (brown) and (*S*)-**17b** (yellow) compared with the reference ECD spectra of (5*R*,1'*R*)-**5a** (green) and (5*R*,1'*S*)-**5b** (blue).

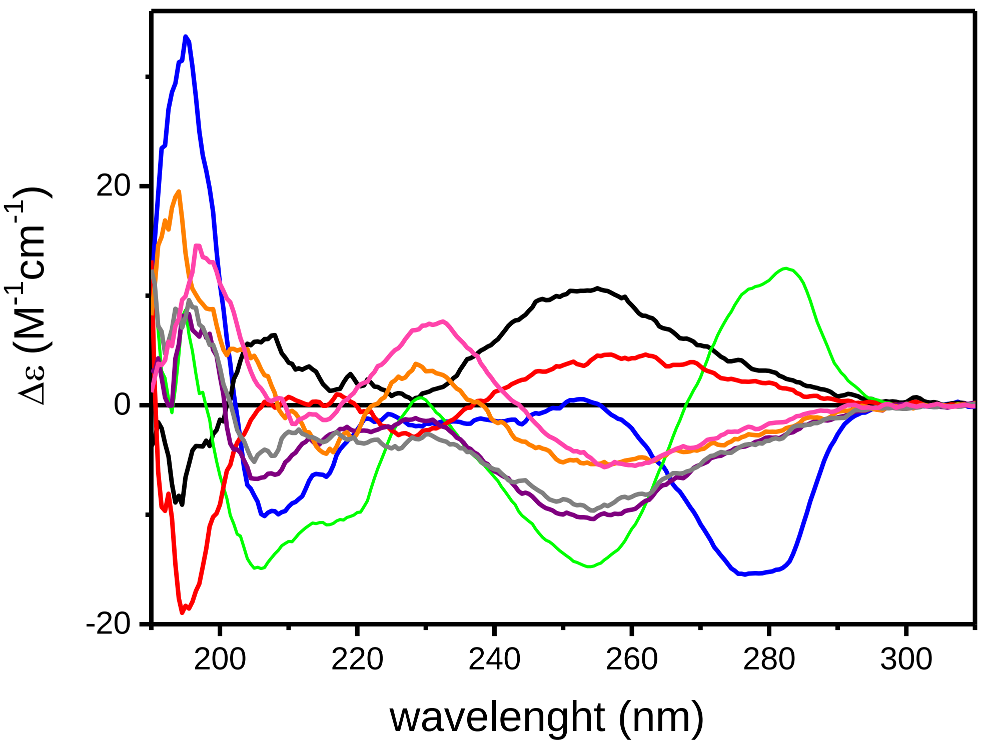

Figure S172. Experimental ECD spectra of (5*R*,1'*R*)-**5a** (black) and (5*R*,1'*S*)-**5b** (red) compared with those of (5*S*,1'*S*)-**8a** (green), (5*S*,1'*R*)-**8b** (blue), (5*S*,1'*S*)-**9a** (purple), (5*S*,1'*R*)-**9b** (orange), (5*S*,1'*S*)-**10a** (grey) and (5*S*,1'*R*)-**10b** (pink).

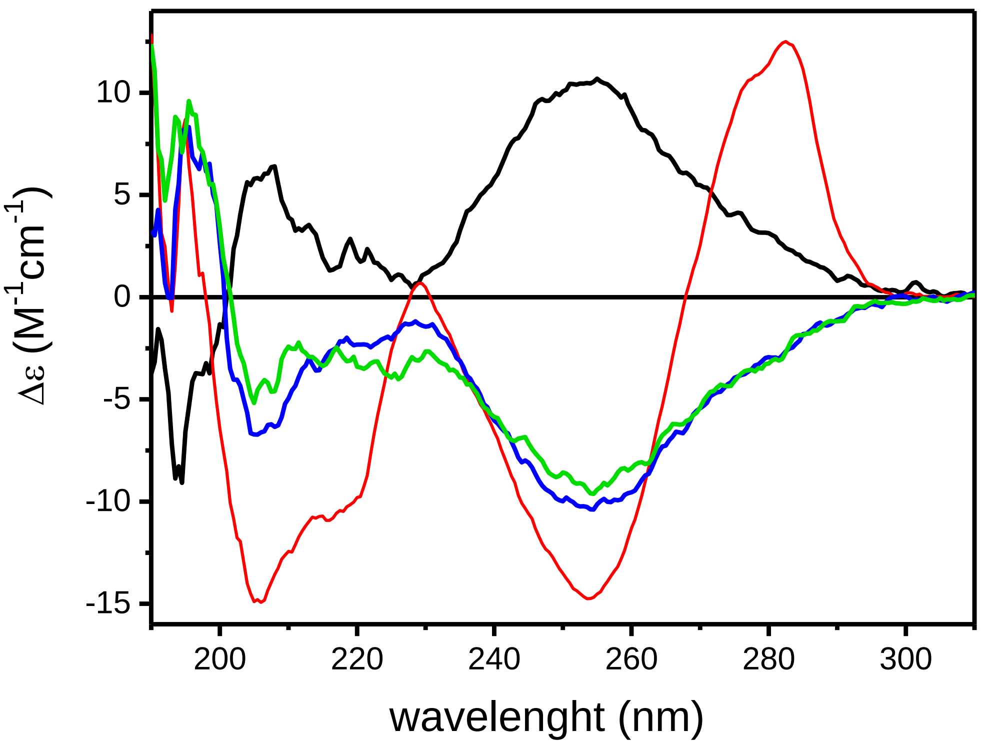

Figure S173. Experimental ECD spectra of (5*R*,1’*R*)-**5a** (black), (5*S*,1’*S*)-**8a** (red), (5*S*,1’*S*)-**9a** (blue) and (5*S*,1’*S*)-**10a** (green).

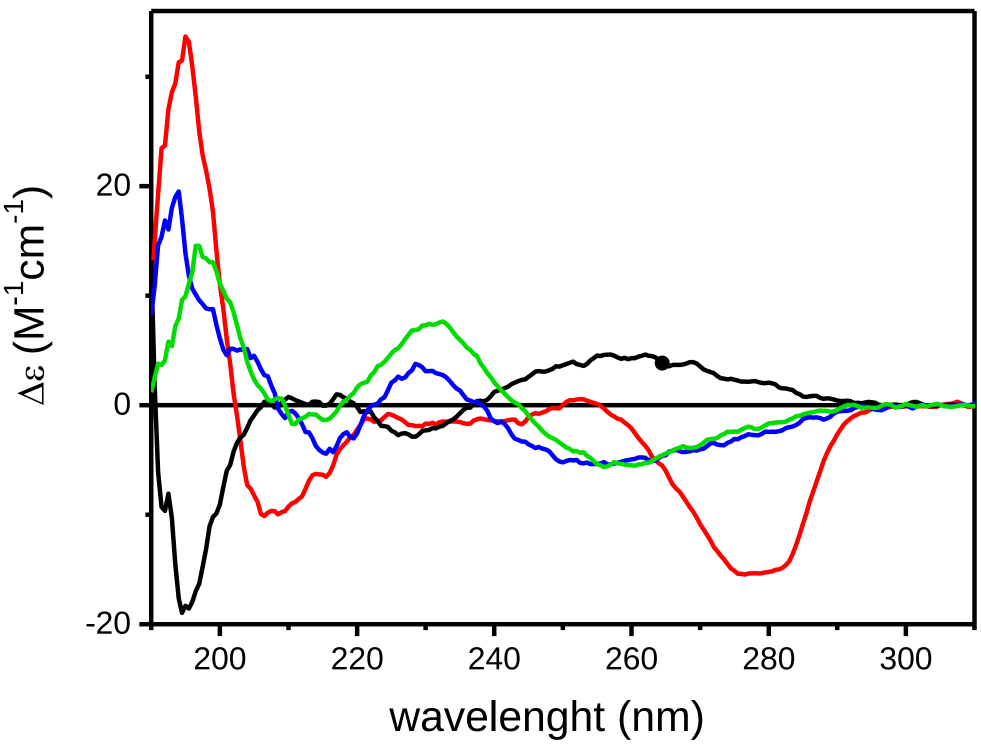

Figure S174. Experimental ECD spectra of (5*R*,1'*S*)-**5b** (black), (5*S*,1'*R*)-**8b** (red), (5*S*,1'*R*)-**9b** (blue) and (5*S*,1'*R*)-**10b** (green).

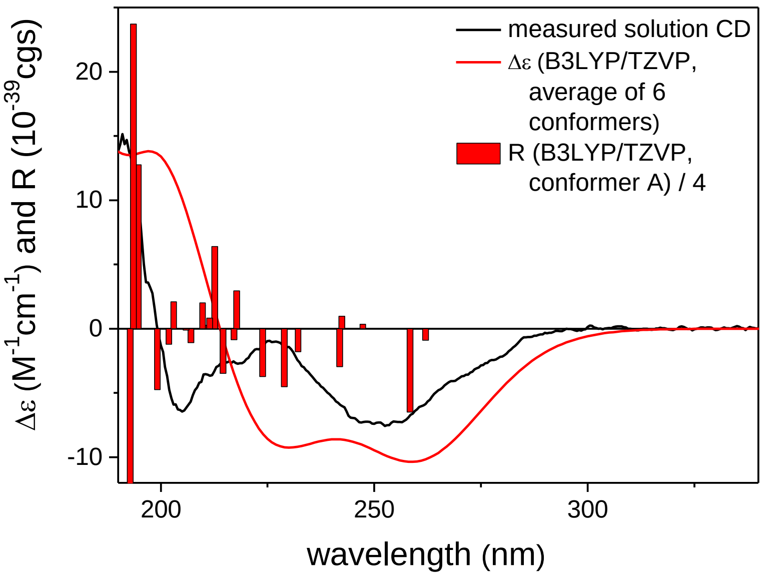

Figure S175. Experimental ECD spectrum of (5*S*,1'*S*)-**1a** in acetonitrile compared with its B3LYP/TZVP PCM/MeCN // ωB97X/TZVP PCM/MeCN ECD spectrum. Bars represent the computed rotarory strength values.

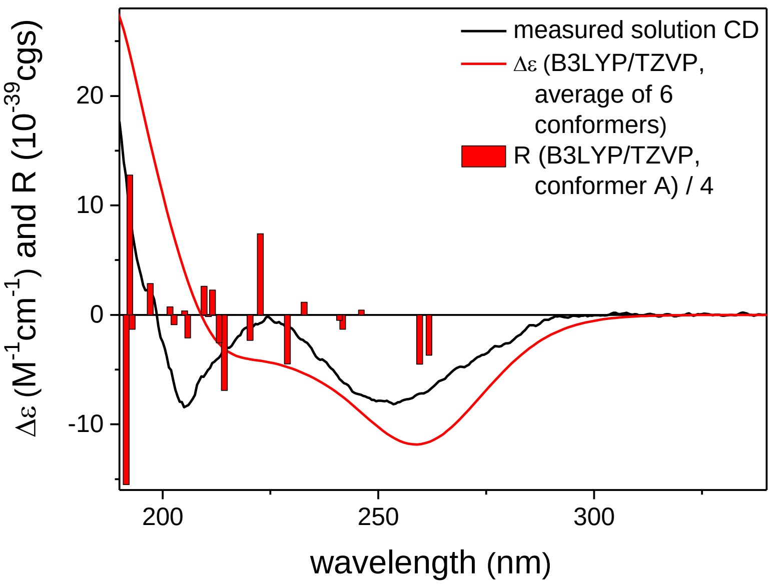

Figure S176. Experimental ECD spectrum of (5*S*,1'*R*)-**1b** in acetonitrile compared with its B3LYP/TZVP PCM/MeCN // ωB97X/TZVP PCM/MeCN ECD spectrum. Bars represent the computed rotarory strength values.

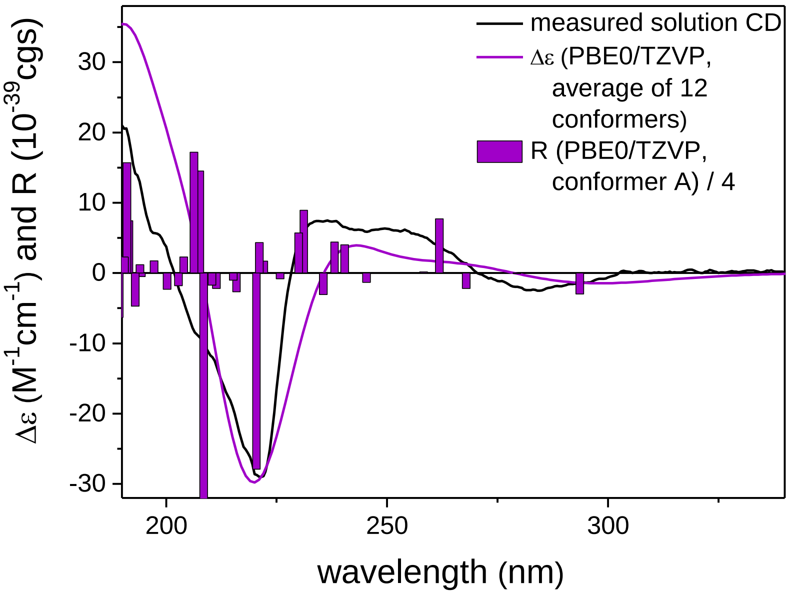

Figure S177. Experimental ECD spectrum of (5*S*,1'*S*)-**2a** in acetonitrile compared with its PBE0/TZVP PCM/MeCN // ωB97X/TZVP PCM/MeCN ECD spectrum. Bars represent the computed rotarory strength values.

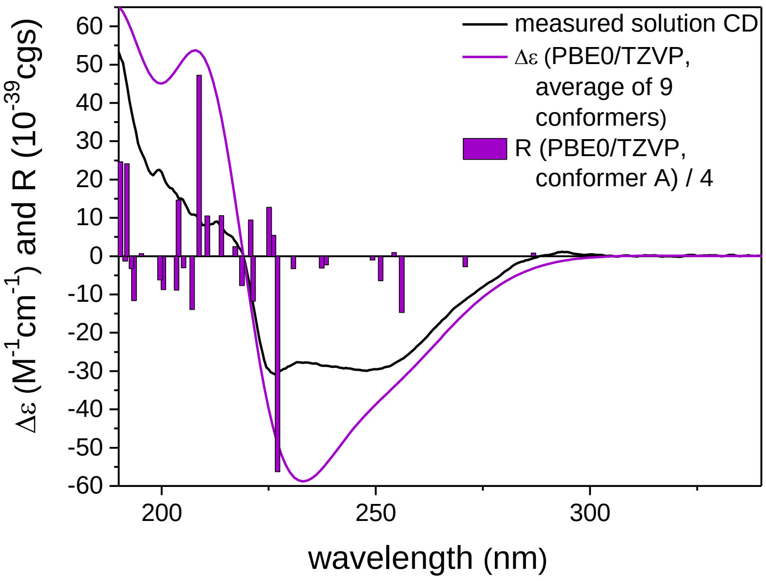

Figure S178. Experimental ECD spectrum of (5*S*,1'*R*)-**2b** in acetonitrile compared with its PBE0/TZVP PCM/MeCN // ωB97X/TZVP PCM/MeCN ECD spectrum. Bars represent the computed rotarory strength values.

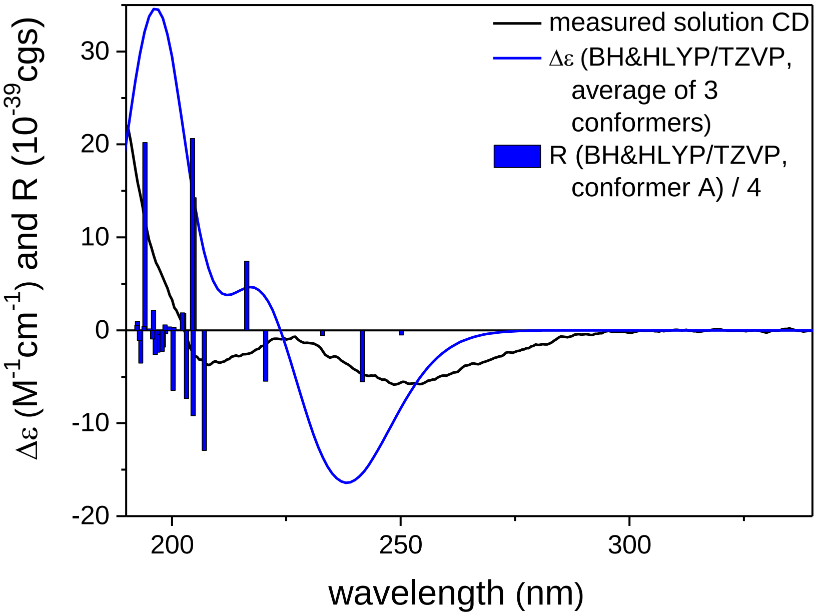

Figure S179. Experimental ECD spectrum of (5*S*,1'*S*)-**4a** in acetonitrile compared with its BH&HLYP/TZVP PCM/MeCN // ωB97X/TZVP PCM/MeCN ECD spectrum. Bars represent the computed rotarory strength values.

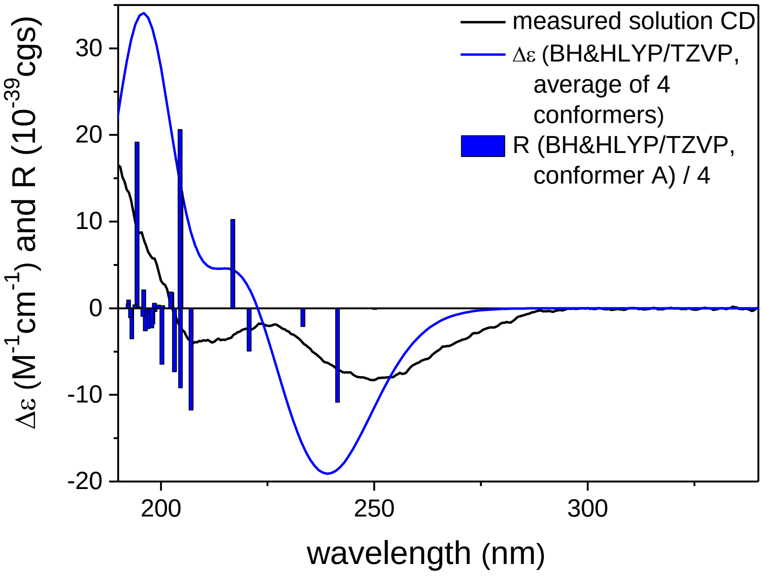

Figure S180. Experimental ECD spectrum of (5*S*,1'*R*)-**4b** in acetonitrile compared with its BH&HLYP/TZVP PCM/MeCN // ωB97X/TZVP PCM/MeCN spectrum. Bars represent the computed rotarory strength values.

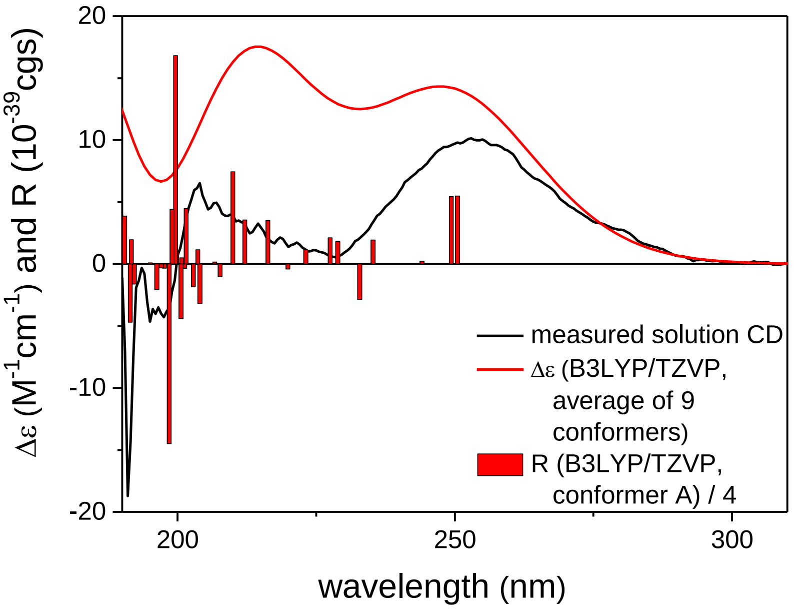

Figure S181. Experimental ECD spectrum of (5*R*,1'*R*)-**5a** in acetonitrile compared with its B3LYP/TZVP PCM/MeCN // ωB97X/TZVP PCM/MeCN ECD spectrum. Bars represent the computed rotarory strength values.

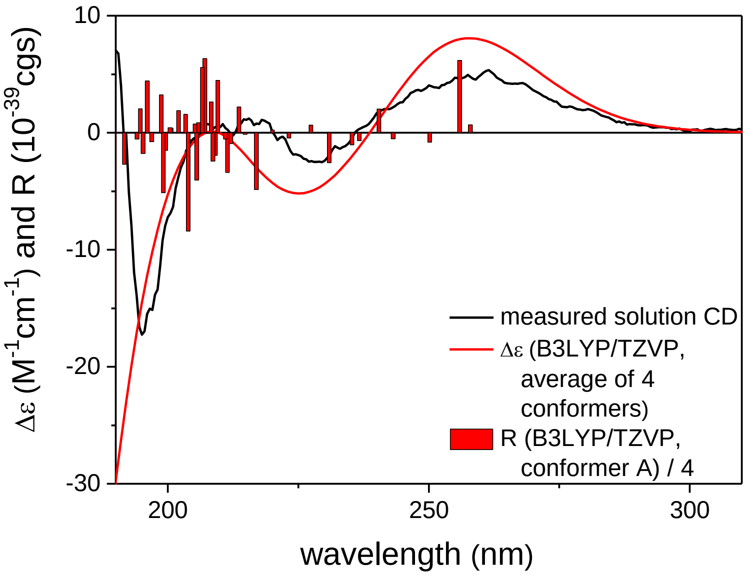

Figure S182. Experimental ECD spectrum of (5*R*,1'*S*)-**5b** in acetonitrile compared with its B3LYP/TZVP PCM/MeCN // ωB97X/TZVP PCM/MeCN ECD spectrum. Bars represent the computed rotarory strength values.

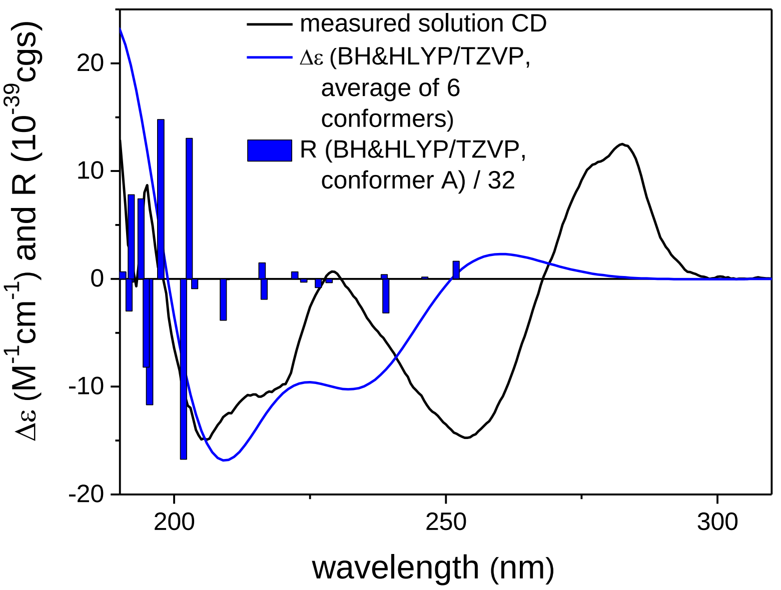

Figure S183. Experimental ECD spectrum of (5*S*,1'*S*)-**8a** in acetonitrile compared with its BH&HLYP/TZVP PCM/MeCN // ωB97X/TZVP PCM/MeCN ECD spectrum. Bars represent the computed rotarory strength values.

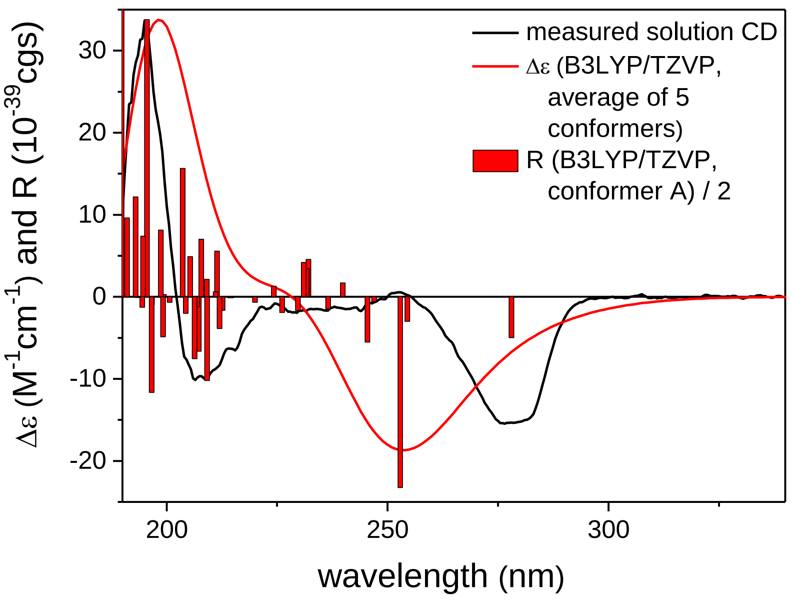

Figure S184. Experimental ECD spectrum of (5*S*,1'*R*)-**8b** in acetonitrile compared with its B3LYP/TZVP PCM/MeCN // ωB97X/TZVP PCM/MeCN ECD spectrum. Bars represent the computed rotarory strength values.

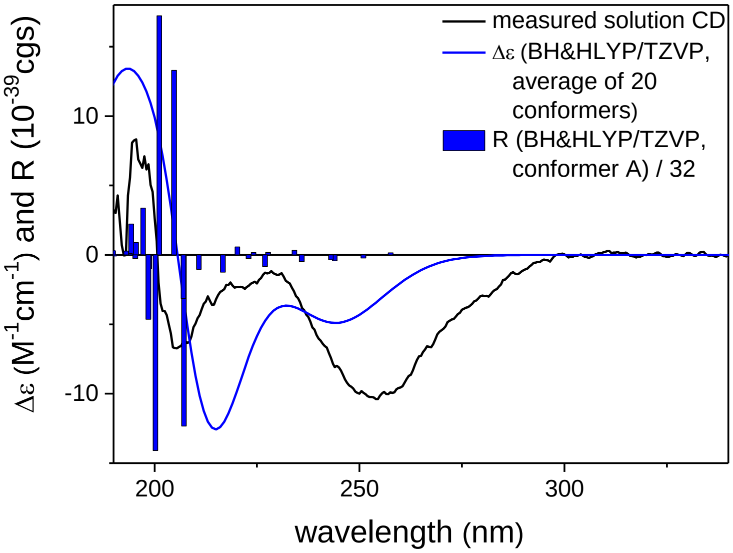

Figure S185. Experimental ECD spectrum of (5*S*,1'*S*)-**9a** in acetonitrile compared with its BH&HLYP/TZVP PCM/MeCN // ωB97X/TZVP PCM/MeCN ECD spectrum. Bars represent the computed rotarory strength values.

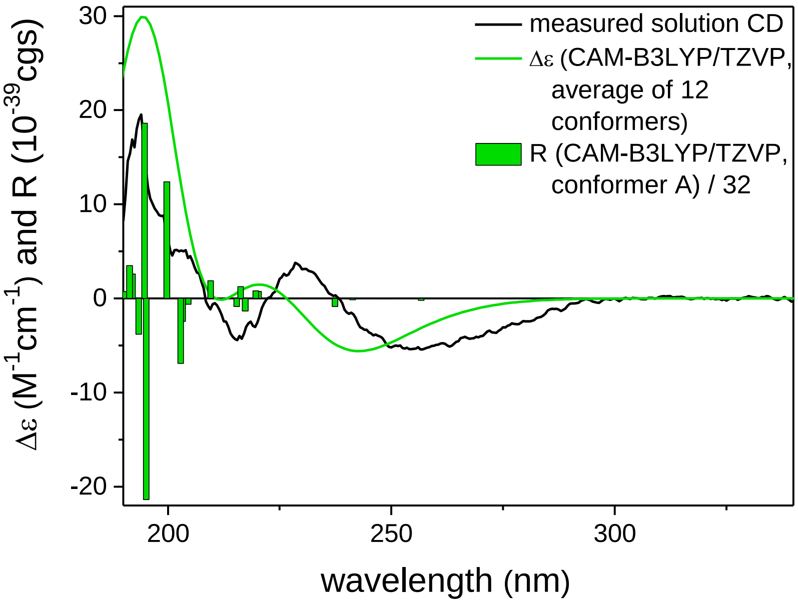

Figure S186. Experimental ECD spectrum of (5*S*,1'*R*)-**9b** in acetonitrile compared with its CAM-B3LYP/TZVP PCM/MeCN // ωB97X/TZVP PCM/MeCN ECD spectrum. Bars represent the computed rotarory strength values.

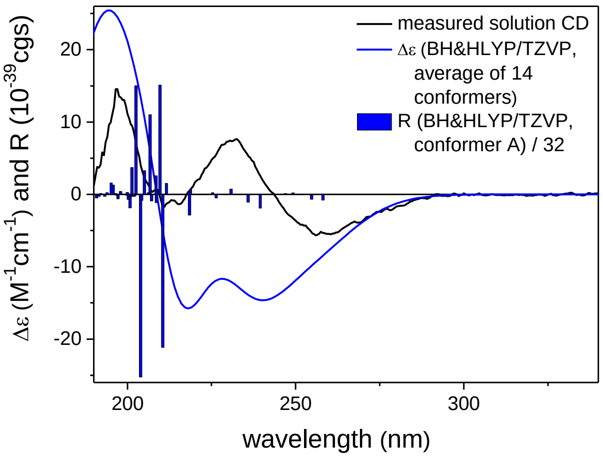

Figure S187. Experimental ECD spectrum of (5*S*,1'*S*)-**10a** in acetonitrile compared with its BH&HLYP/TZVP PCM/MeCN // ωB97X/TZVP PCM/MeCN ECD spectrum. Bars represent the computed rotarory strength values.

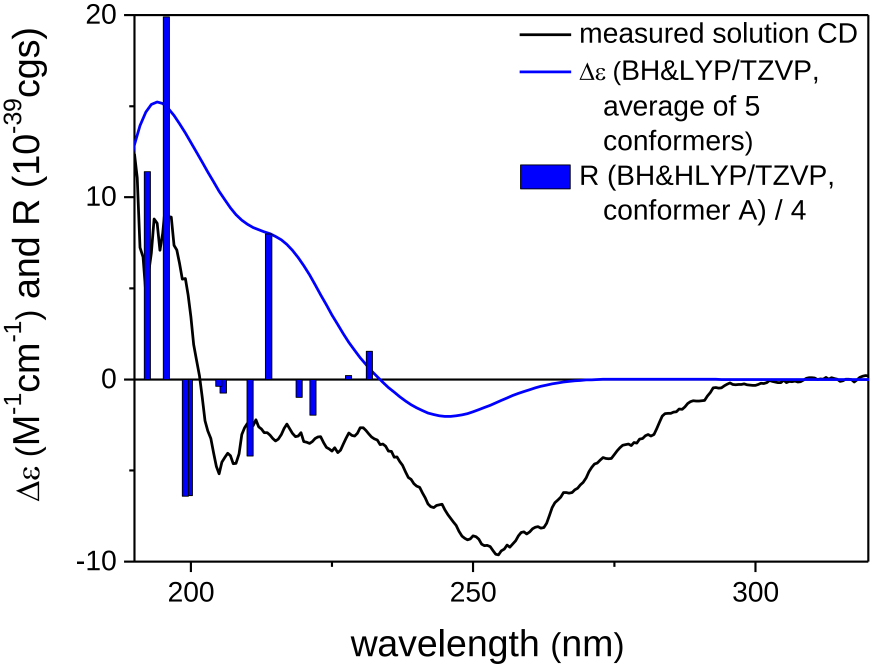

Figure S188. Experimental ECD spectrum of (5*S*,1'*R*)-**10b** in acetonitrile compared with its BH&HLYP/TZVP PCM/MeCN // ωB97X/TZVP PCM/MeCN ECD spectrum. Bars represent the computed rotarory strength values.

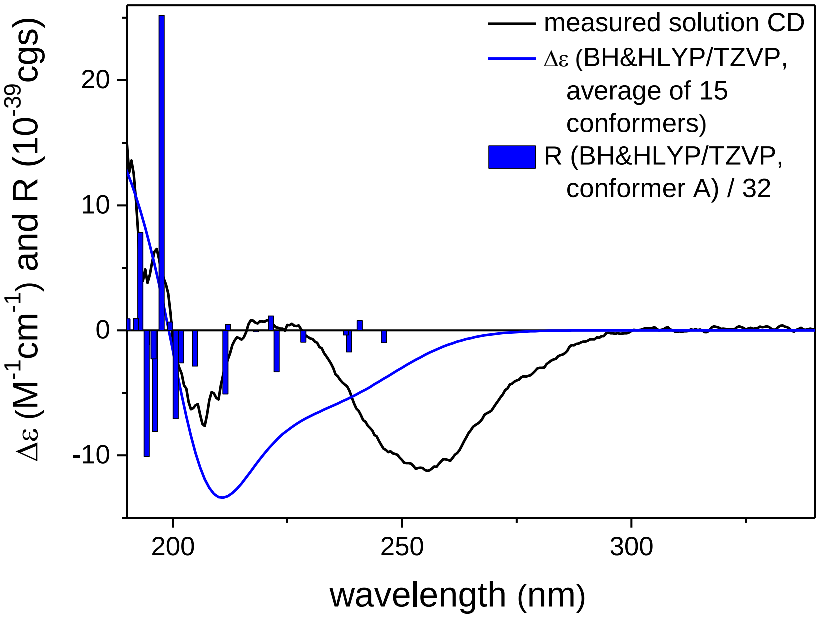

Figure S189. Experimental ECD spectrum of (5*S*,1’*S*)-**13a** in acetonitrile compared with its BH&HLYP/TZVP PCM/MeCN // ωB97X/TZVP PCM/MeCN ECD spectrum. Bars represent the computed rotarory strength values.

6. OR analysis

Table S1: Measured specific rotation $\{[\alpha]_D^{20}\}$of compounds at the sodium D line (589.3 nm) in MeOH.

Compound	Specific rotation $\{[\alpha]_D^{20}\}$in MeOH
(5*S*,1'*S*)-**1a**	–27
(5*S*,1'*R*)-**1b**	–46
(5*S*,1'S)-**2a**	–29
(5*S*,1'*R*)-**2b**	–188
(5*S*,1'S)-**3a**	–35
(5*S*,1'*R*)-**3b**	+50
(5*S*,1'S)-**4a**	–10
(5*S*,1'*R*)-**4b**	–55
(5*R*,1'*R*)-**5a**	+61
(5*R*,1'*S*)-**5b**	–61
(5*R*,1'*R*)-**6a**	+58
(5*R*,1'*S*)-**6b**	–50
(5*R*,1'*R*)-**7a**	+63
(5*R*,1'*S*)-**7b**	–39
(5*S*,1'*S*)-**8a**	–52
(5*S*,1'*R*)-**8b**	–32
(5*S*,1'*S*)-**9a**	–70
(5*S*,1'*R*)-**9b**	+49
(5*S*,1'*S*)-**10a**	–76
(5*S*,1'*R*)-**10b**	+42
(5*S*,1'*S*)-**11a**	–80
(5*S*,1'*R*)-**11b**	+31
(5*S*,1'*S*)-**12a**	–87
(5*S*,1'*R*)-**12b**	+50
(5*S*,1'*S*)-**13a**	–70
(5*S*,1'*R*)-**13b**	+33
(5*S*,1'*S*)-**14a**	–88
(5*S*,1'*R*)-**14b**	+45
(5*S*,1'*S*)-**15a**	–82
(5*S*,1'*R*)-**15b**	+36
(5*S*,1'*S*)-**16a**	–97
(5*S*,1'*R*)-**16b**	+70
(*R*)-**17a**	–51
(*S*)-**17b**	+40

Table S2: Measured specific rotation $\{[\alpha]^{20}_{D}\}$ in MeOH compared with the four computed average values (basis set: TZVP, solvent model: PCM/MeOH).

Compound	Experimental $[\alpha]^{20}_{D}$	B3LYP	BH&HLYP	CAM-B3LYP	PBE0
(5*R*,1'*R*)-**5a**	+61	+196	+148	+159	+191
(5*R*,1'*S*)-**5b**	–61	–91	–93	–94	–91
(5*S*,1'S)-**8a**	–52	–180	–137	–145	–174
(5*S*,1'*R*)-**8b**	–32	+12	+29	+24	+13
(5*S*,1'*S*)-**2a**	–29	–97.18	–67.25	–74.00	–91.42
(5*S*,1'*R*)-**2b**	–188	–153.58	–103.94	–109.76	–148.50

Table S3. Boltzmann populations and optical rotations of the low-energy conformers of (5*S*,1'*S*)-**2a** computed at various levels for the ωB97X/TZVP PCM/MeCN optimized MMFF conformers (basis set: TZVP, solvent model: PCM/MeOH).

Conformer	Boltzmann population	B3LYP	BH&HLYP	CAM-B3LYP	PBE0
Conf. A	36.95 %	–149.48	–109.15	–115.44	–143.43
Conf. B	34.90 %	–8.98	+0.10	–6.66	–3.36
Conf. C	13.84 %	–224.49	–161.15	–169.44	–216.5
Conf. D	8.93 %	–76.34	–54.45	–61.39	–73.72
Conf. E	5.37 %	–16.81	+4.22	–1.36	–12.66
Average	N/A	–97.18	–67.25	–74.00	–91.42

Table S4. Boltzmann populations and optical rotations of the low-energy conformers of (5*S*,1'*R*)-**2b** computed at various levels for the ωB97X/TZVP PCM/MeCN optimized MMFF conformers (basis set: TZVP, solvent model: PCM/MeOH).

Conformer	Boltzmann population	B3LYP	BH&HLYP	CAM-B3LYP	PBE0
Conf. A	78.18 %	–174.47	–120.01	–125.77	–168.9
Conf. B	9.77 %	–31.64	–9.97	–13.89	–29.37
Conf. C	7.02 %	–101.78	–56.13	–65.05	–97.82
Conf. D	3.83 %	–170.37	–128.31	–134.44	–165.08
Conf. E	0.98 %	–8.74	–5.9	–12.01	–6.77
Average	N/A	–153.58	–103.94	–109.76	–148.50

Table S5. Boltzmann populations and optical rotations of the low-energy conformers of (5*R*,1'*R*)-**5a** computed at various levels for the ωB97X/TZVP PCM/MeCN optimized MMFF conformers (basis set: TZVP, solvent model: PCM/MeOH).

Conformer	Boltzmann population	B3LYP	BH&HLYP	CAM-B3LYP	PBE0
Conf. A	33.06 %	+274.02	+214.79	+228.96	+266.24
Conf. B	31.43 %	+275.18	+216.65	+230.98	+267.75
Conf. C	13.73 %	+85.94	+52.18	+59.12	+83.35
Conf. D	9.76 %	–23.19	–39.26	–37.44	–23.23
Conf. E	3.57 %	+203.52	+164.47	+173.33	+200.25
Conf. F	3.43 %	–116.95	–126.39	–122.21	–116.63
Conf. G	2.23 %	+199.01	+162.88	+173.23	+195.56
Conf. H	1.34 %	+96.99	+49.97	+55.45	+91.66
Conf. I	1.17 %	+.74	–1.43	+6.04	+37.44
Average	N/A	+196.68	+148.67	+159.87	+191.06

Table S6. Boltzmann populations and optical rotations of the low-energy conformers of (5*R*,1'*S*)-**5b** computed at various levels for the ωB97X/TZVP PCM/MeCN optimized MMFF conformers (basis set: TZVP, solvent model: PCM/MeOH).

Conformer	Boltzmann population	B3LYP	BH&HLYP	CAM-B3LYP	PBE0
Conf. A	48.52 %	–113.4	–112.29	–113.57	–112.43
Conf. B	42.97 %	–99.14	–101.30	–101.61	–98.92
Conf. C	3.84 %	+278.49	+227.00	+235.73	+271.71
Conf. D	3.56 %	–93.12	–97.75	–98.79	–94.52
Average	N/A	–91.27	–93.83	–94.29	–91.01

Table S7. Boltzmann populations and optical rotations of the low-energy conformers of (5*S*,1'*S*)-**8a** computed at various levels for the ωB97X/TZVP PCM/MeOH optimized MMFF conformers (basis set: TZVP, solvent model: PCM/MeOH).

Conformer	Boltzmann population	B3LYP	BH&HLYP	CAM-B3LYP	PBE0
Conf. A	82.68 %	–191.28	–147.85	–155.87	–184.66
Conf. B	8.41 %	–124.43	–82.62	–89.86	–120.79
Conf. C	4.28 %	–163.22	–133.17	–142.33	–160.77
Conf. D	1.40 %	–79.98	–44.90	–46.52	–73.08
Conf. E	1.04 %	+22.02	+51.62	+42.48	+24.18
Average	N/A	–180.43	–137.99	–145.92	–174.29

Table S8. Boltzmann populations and optical rotations of the low-energy conformers of (5*S*,1'*R*)-**8b** computed at various levels for the ωB97X/TZVP PCM/MeOH optimized MMFF conformers (basis set: TZVP, solvent model: PCM/MeOH).

Conformer	Boltzmann population	B3LYP	BH&HLYP	CAM-B3LYP	PBE0
Conf. A	72.73 %	+34.54	+49.65	+44.55	+35.02
Conf. B	12.18 %	+73.84	+81.52	+80.73	+74.98
Conf. C	9.34 %	–243.56	–196.02	–203.09	–235.76
Average	N/A	+12.05	+29.41	+24.67	+13.34

7. Low-energy conformers

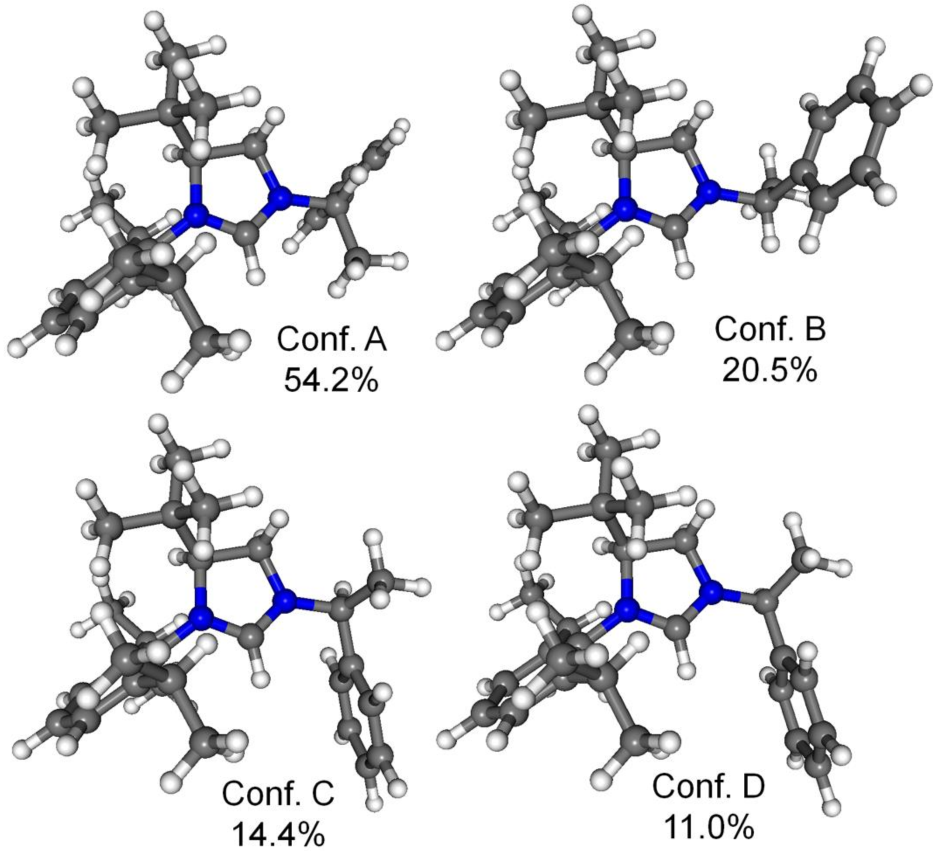

Figure S190. Structure and population of the low-energy B3LYP/TZVP PCM (solvent: $CHCl_3$) conformers (≥ 1%) of (5*S*,1'*S*)-**1a**.

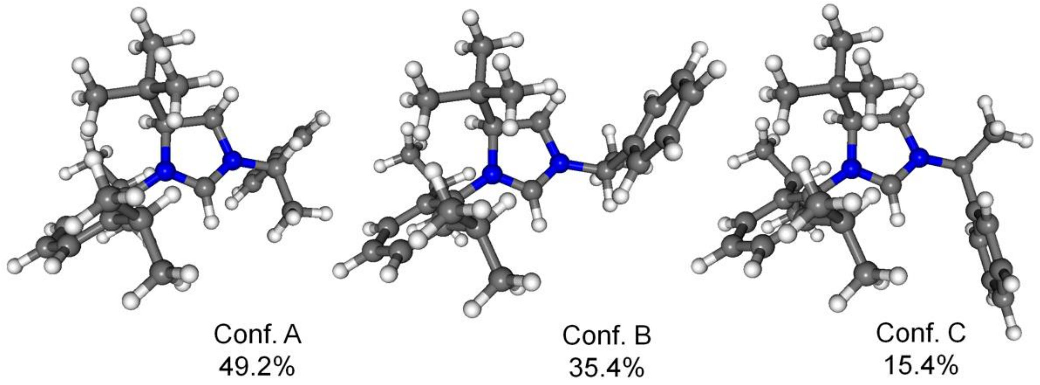

Figure S191. Structure and population of the low-energy ωB97X/TZVP PCM (solvent: acetonitrile) conformers (≥ 1%) of (5*S*,1'*S*)-**1a**.

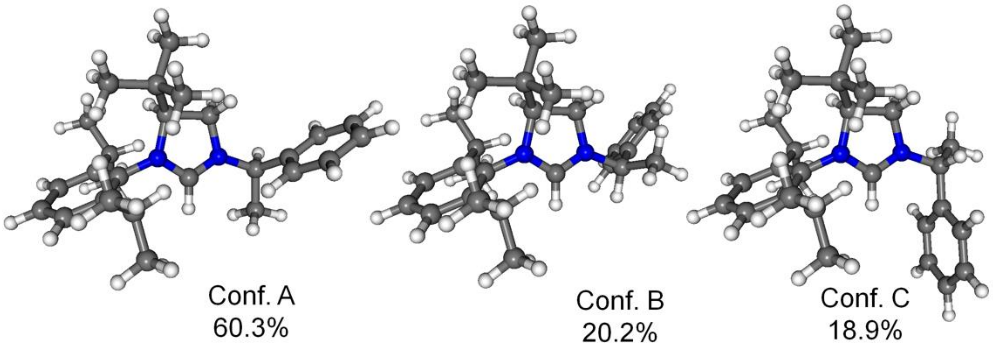

Figure S192. Structure and population of the low-energy B3LYP/TZVP PCM (solvent: $CHCl_3$) conformers (≥ 1%) of (5*S*,1'*R*)-**1b**.

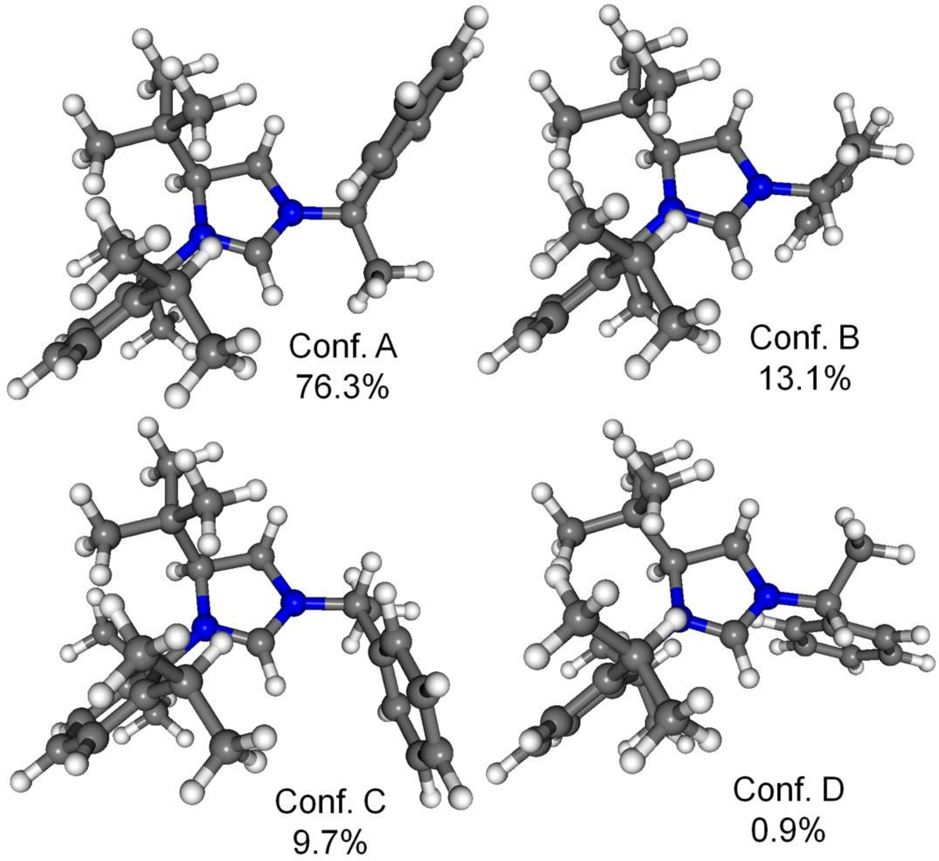

Figure S193. Structure and population of the low-energy ωB97X/TZVP PCM (solvent: acetonitrile) conformers (≥ 1%) of (5*S*,1'*R*)-**1b**.

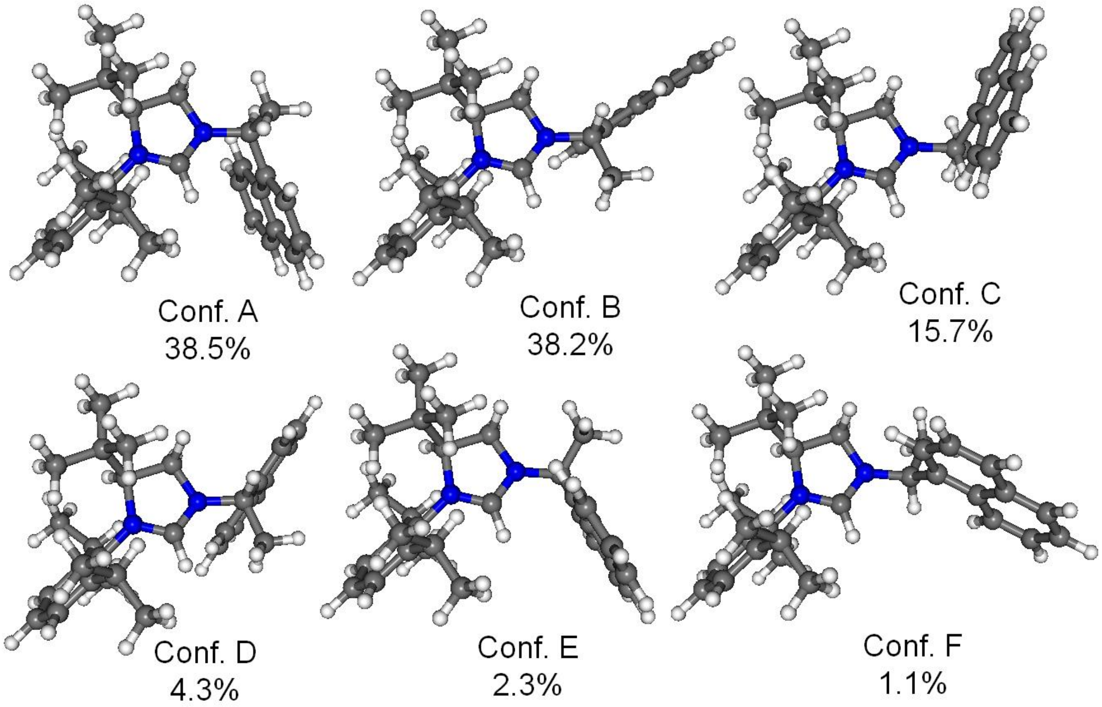

Figure S194. Structure and population of the low-energy B3LYP/TZVP PCM (solvent: $CHCl_3$) conformers (≥ 1%) of (5*S*,1'*S*)-**2a**.

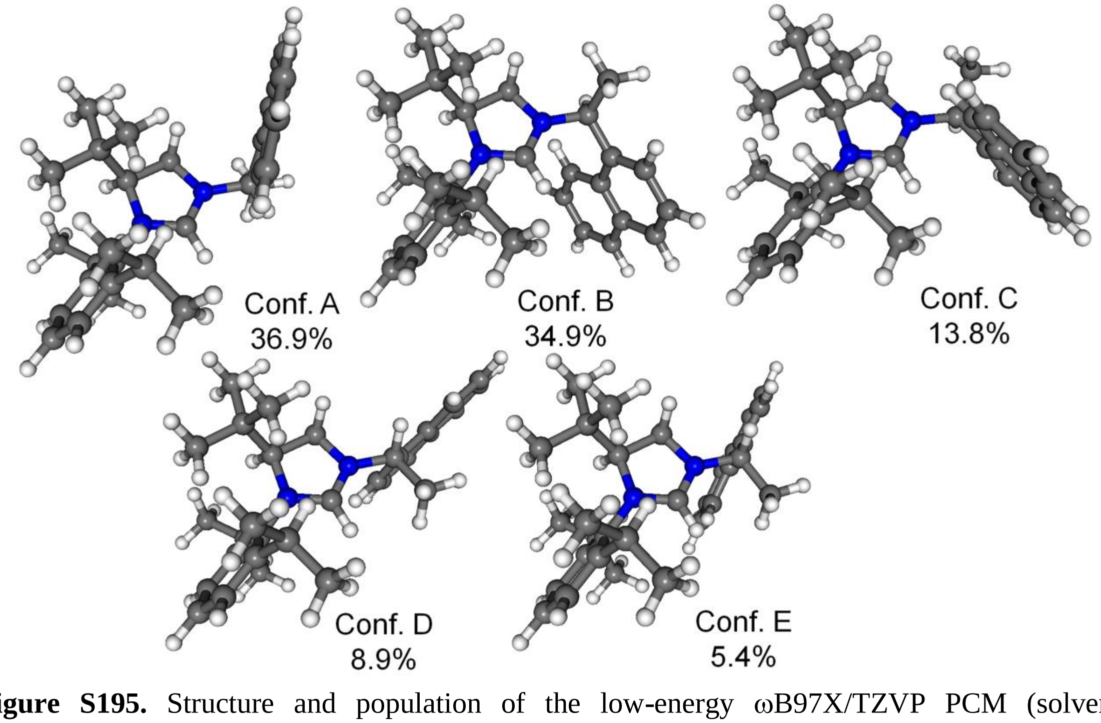

Figure S195. Structure and population of the low-energy ωB97X/TZVP PCM (solvent: acetonitrile) conformers (≥ 1%) of (5*S*,1'*S*)-**2a**.

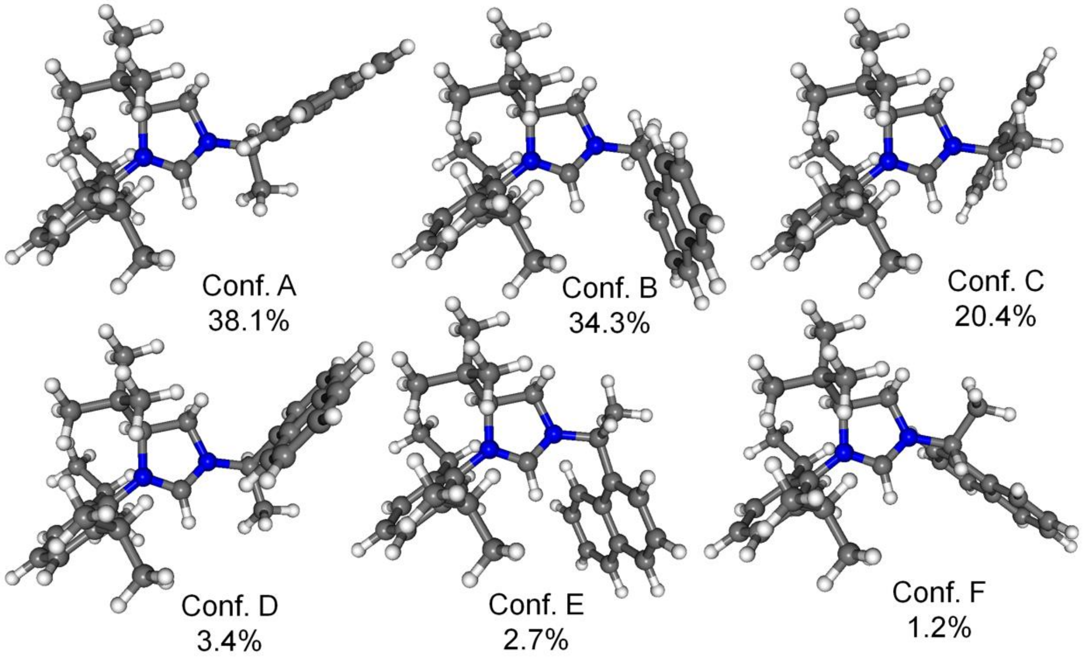

Figure S196. Structure and population of the low-energy B3LYP/TZVP PCM (solvent: $CHCl_3$) conformers (≥ 1%) of (5*S*,1'*R*)-**2b**.

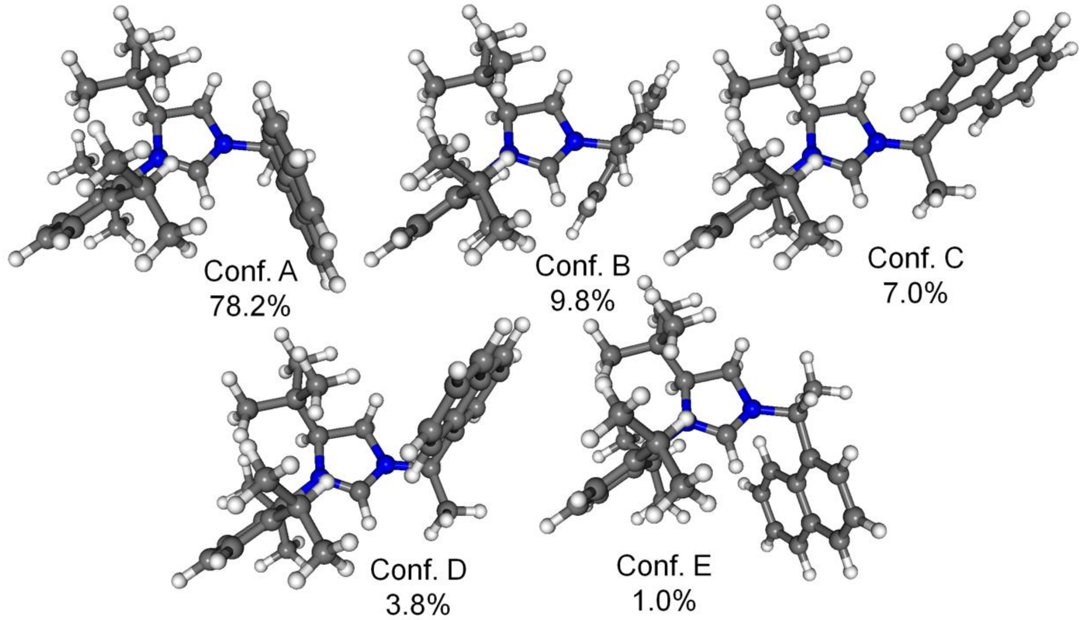

Figure S197. Structure and population of the low-energy ωB97X/TZVP PCM (solvent: acetonitrile) conformers (≥ 1%) of (5*S*,1'*R*)-**2b**.

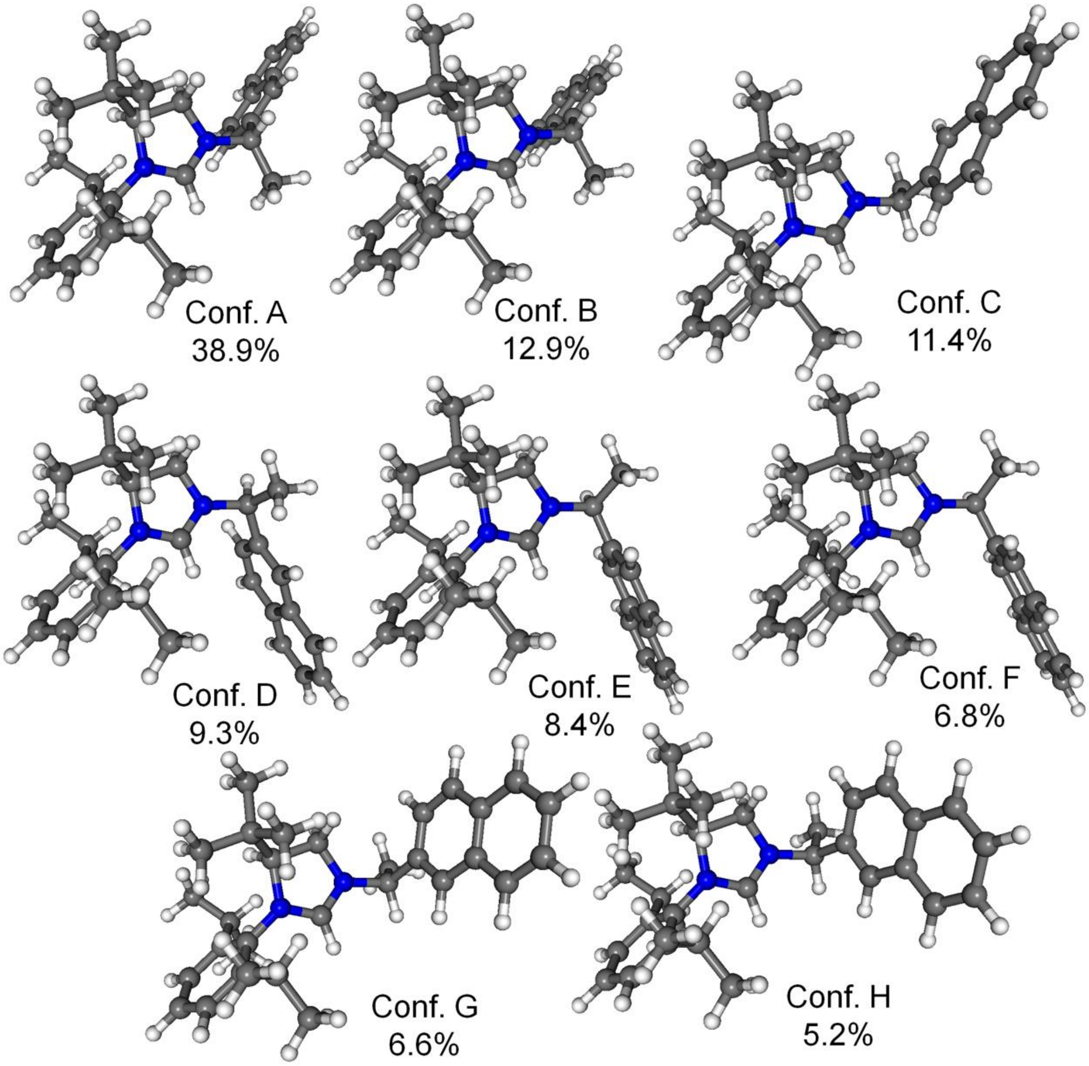

Figure S198. Structure and population of the low-energy B3LYP/TZVP PCM (solvent: $CHCl_3$) conformers (≥ 1%) of (5*S*,1'*S*)-**3a**.

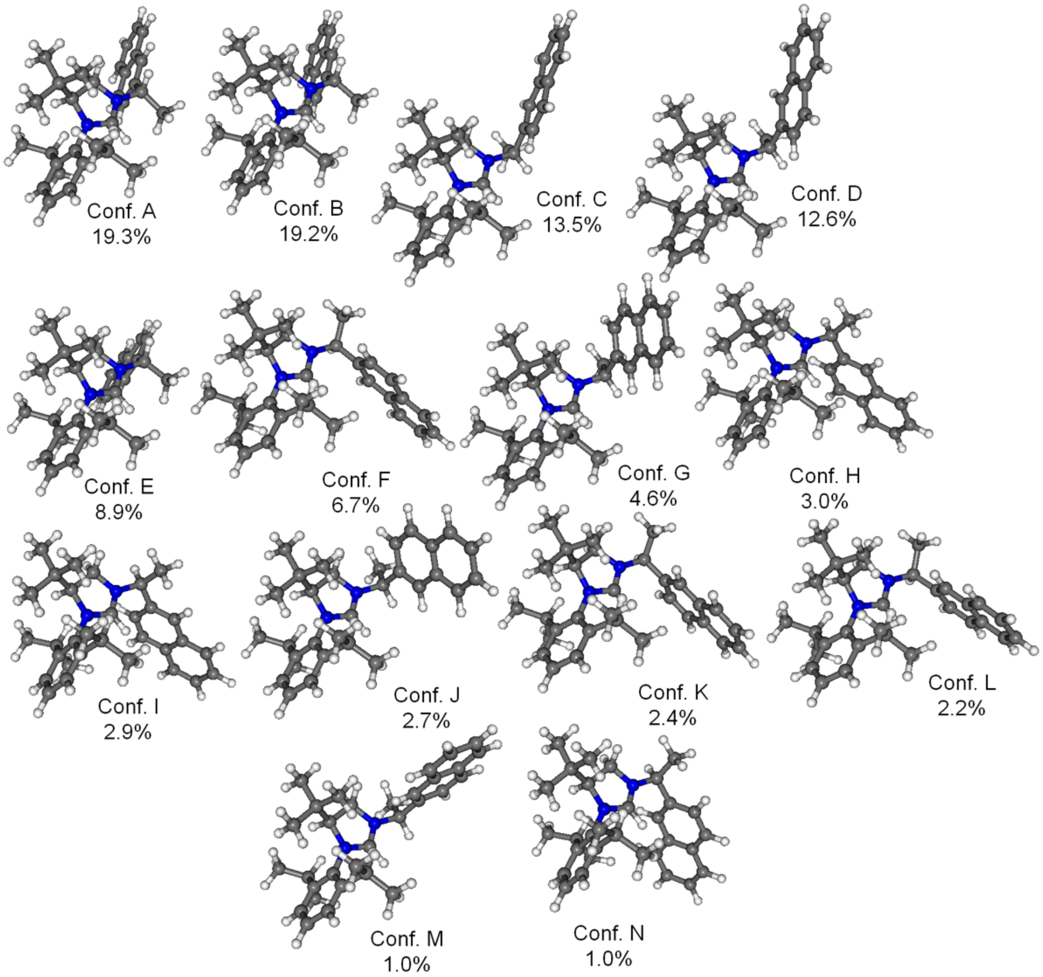

Figure S199. Structure and population of the low-energy ωB97X/TZVP PCM (solvent: acetonitrile) conformers (≥ 1%) of (5*S*,1'*S*)-**3a**.

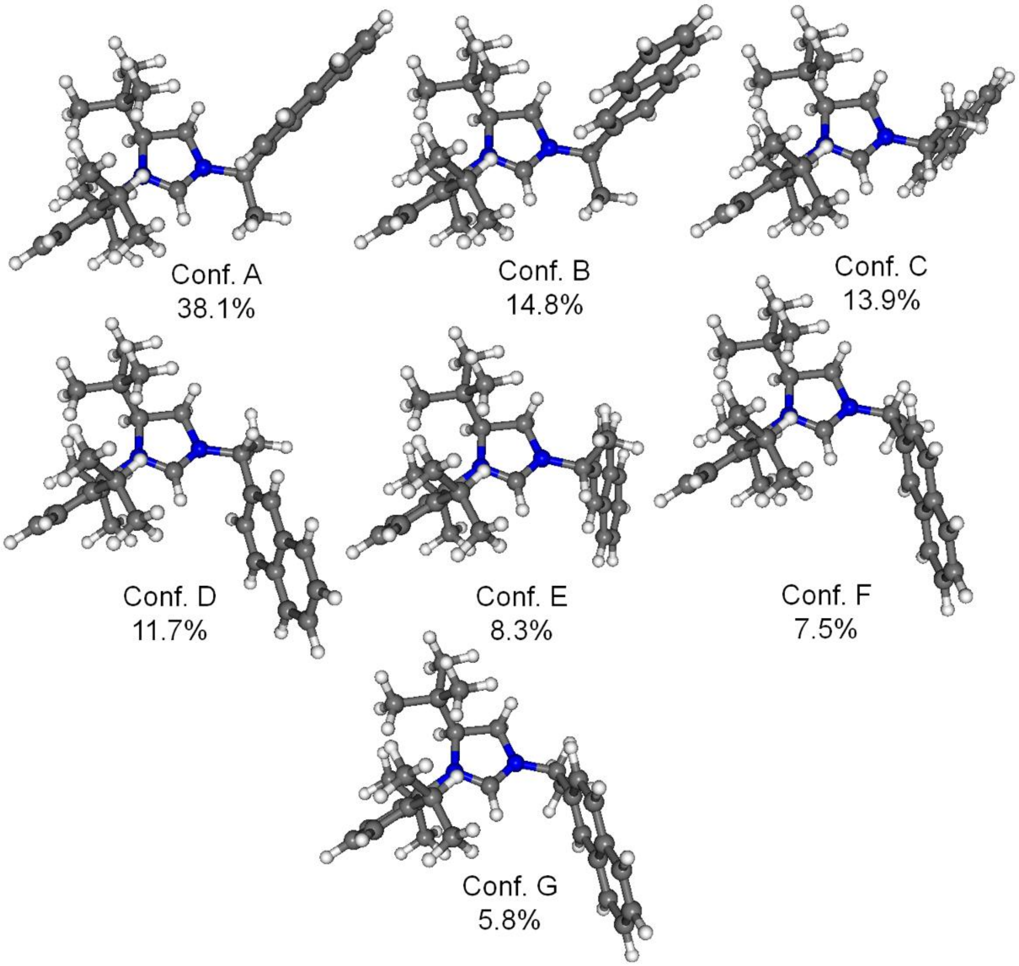

Figure S200. Structure and population of the low-energy B3LYP/TZVP PCM (solvent: $CHCl_3$) conformers (≥ 1%) of (5*S*,1'*R*)-**3b**.

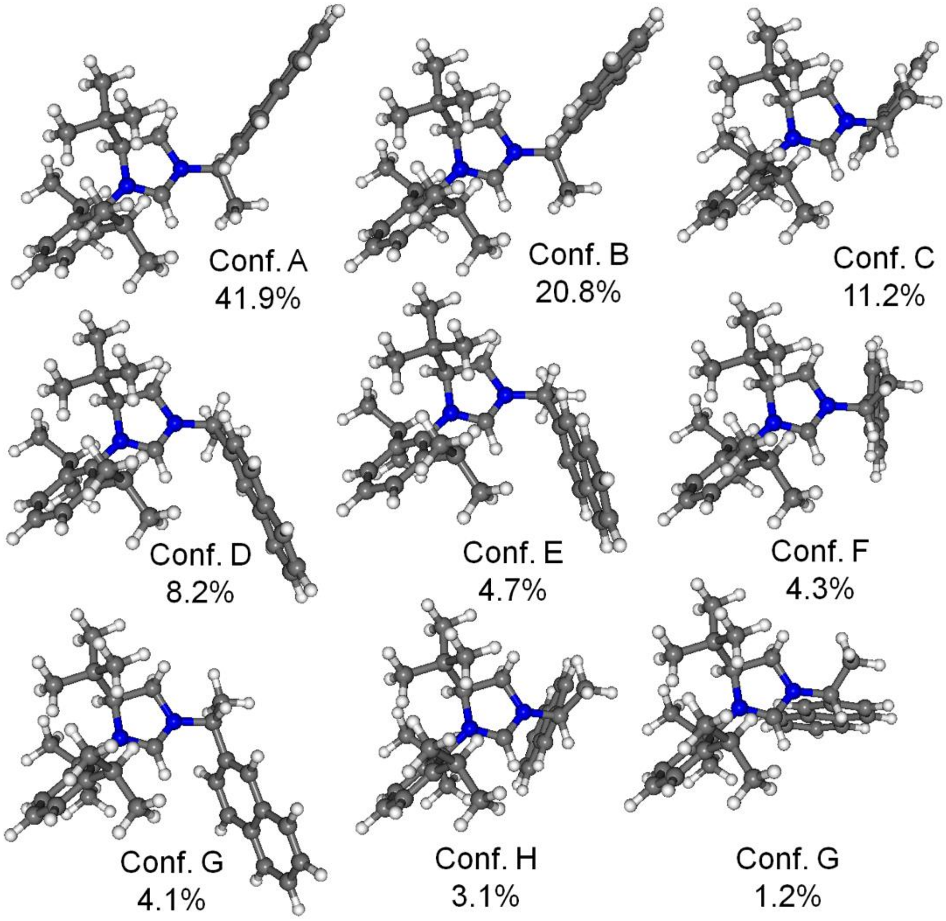

Figure S201. Structure and population of the low-energy ωB97X/TZVP PCM (solvent: acetonitrile) conformers (≥ 1%) of (5*S*,1'*R*)-**3b**.

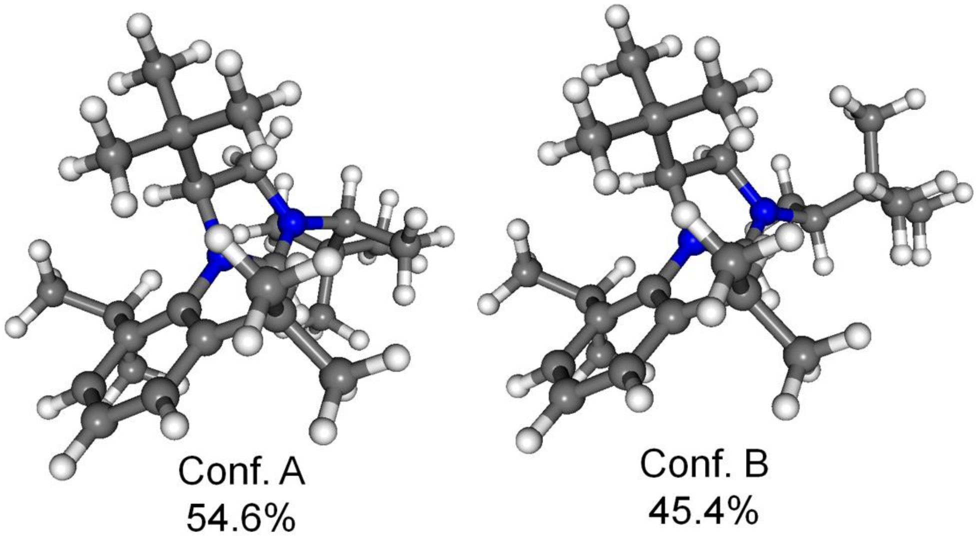

Figure S202. Structure and population of the low-energy B3LYP/TZVP PCM (solvent: $CHCl_3$) conformers (≥ 1%) of (5*S*,1'*S*)-**4a**.

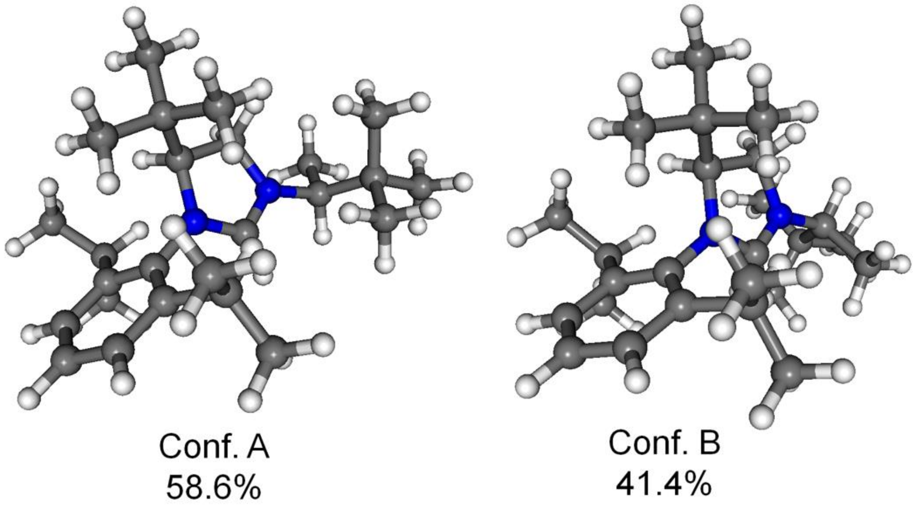

Figure S203. Structure and population of the low-energy ωB97X/TZVP PCM (solvent: acetonitrile) conformers (≥ 1%) of (5*S*,1'*S*)-**4a**.

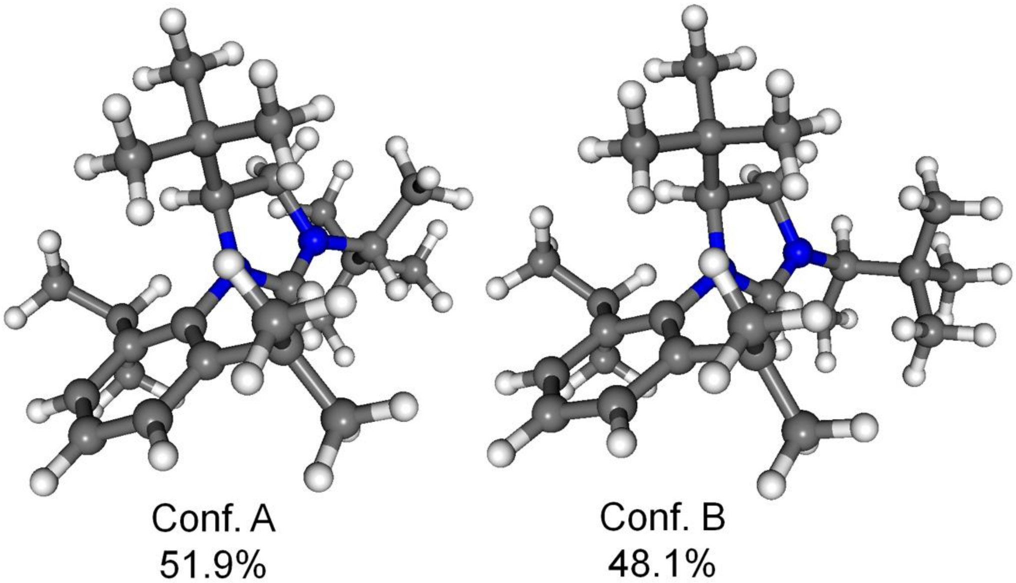

Figure S204. Structure and population of the low-energy B3LYP/TZVP PCM (solvent: $CHCl_3$) conformers (≥ 1%) of (5*S*,1'*R*)-**4b**.

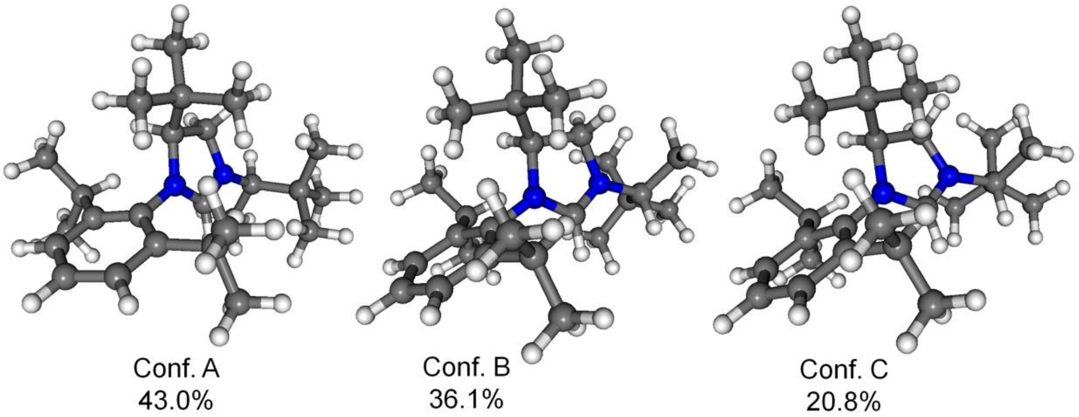

Figure S205. Structure and population of the low-energy ωB97X/TZVP PCM (solvent: acetonitrile) conformers (≥ 1%) of (5*S*,1’*R*)-**4b**.

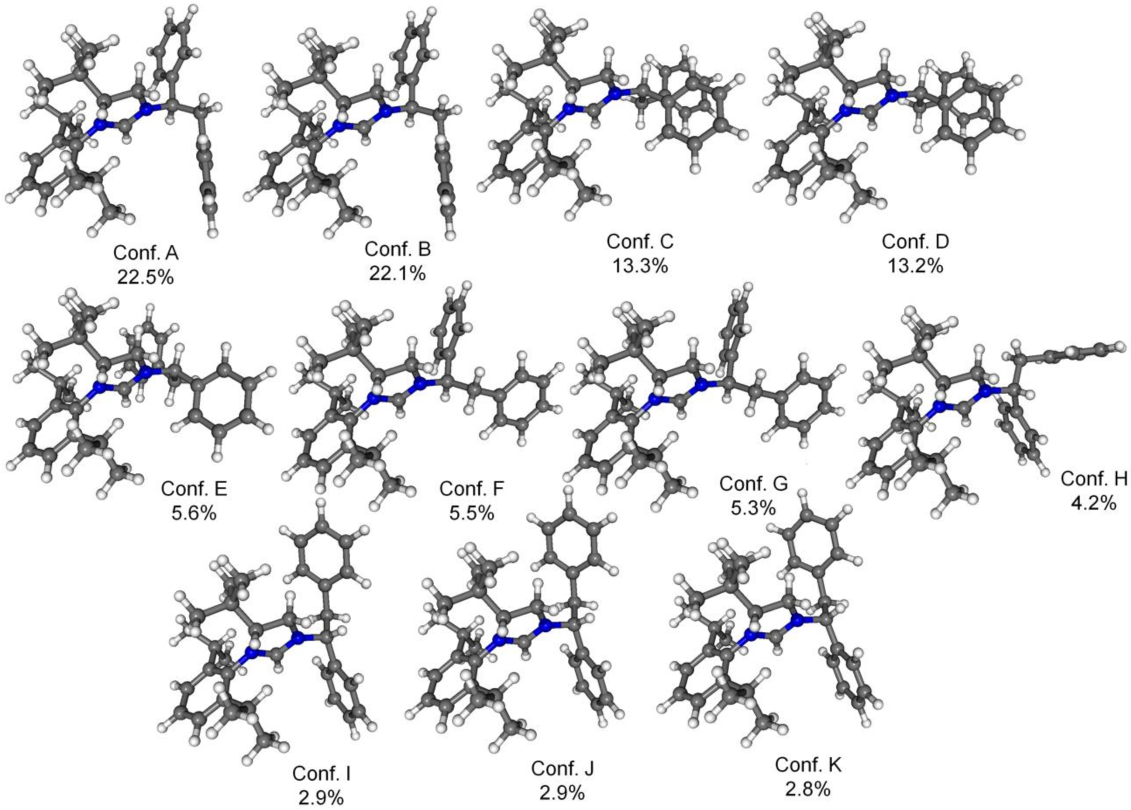

Figure S206. Structure and population of the low-energy B3LYP/TZVP PCM (solvent: $CHCl_3$) conformers (≥ 1%) of (5*R*,1’*R*)-**5a**.

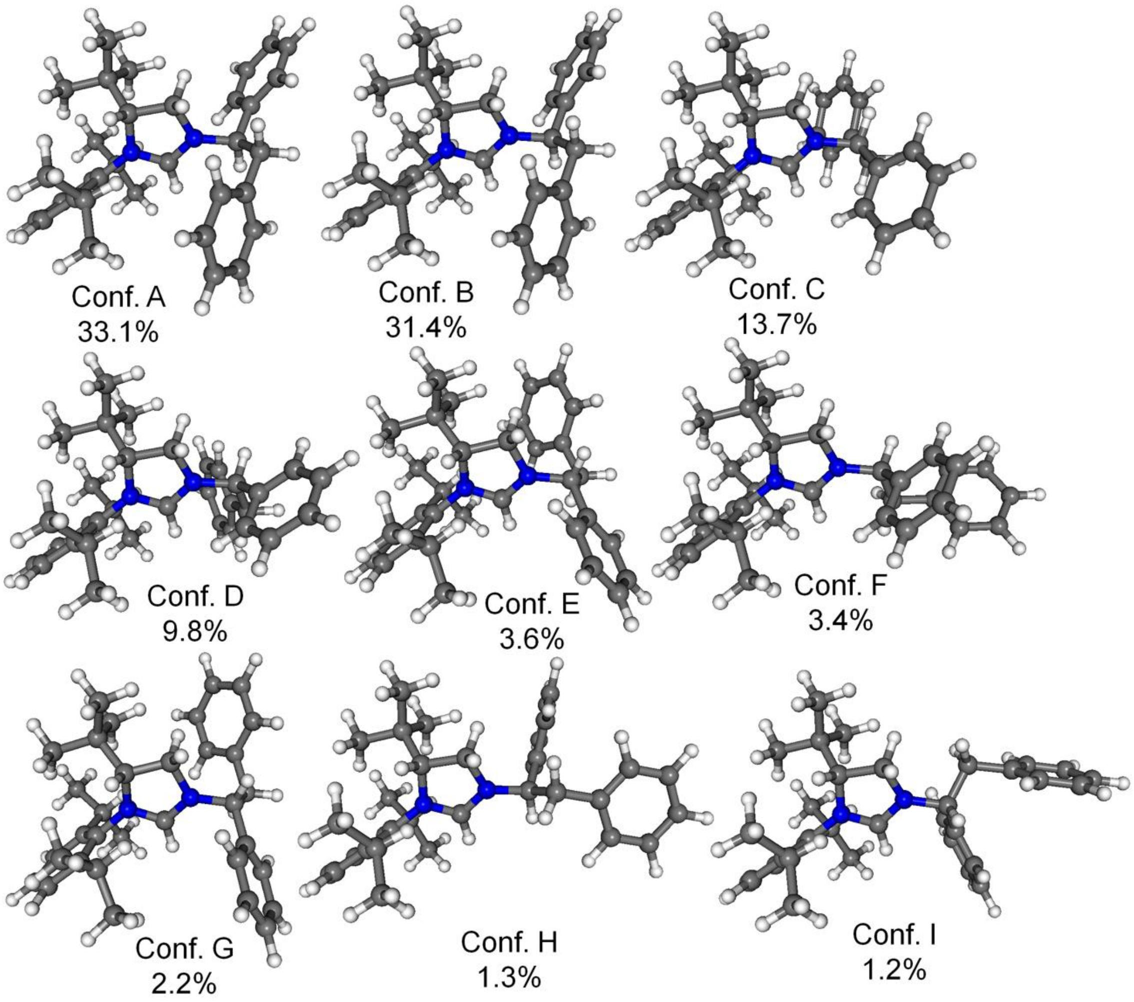

Figure S207. Structure and population of the low-energy ωB97X/TZVP PCM (solvent: acetonitrile) conformers (≥ 1%) of (5*R*,1'*R*)-**5a**.

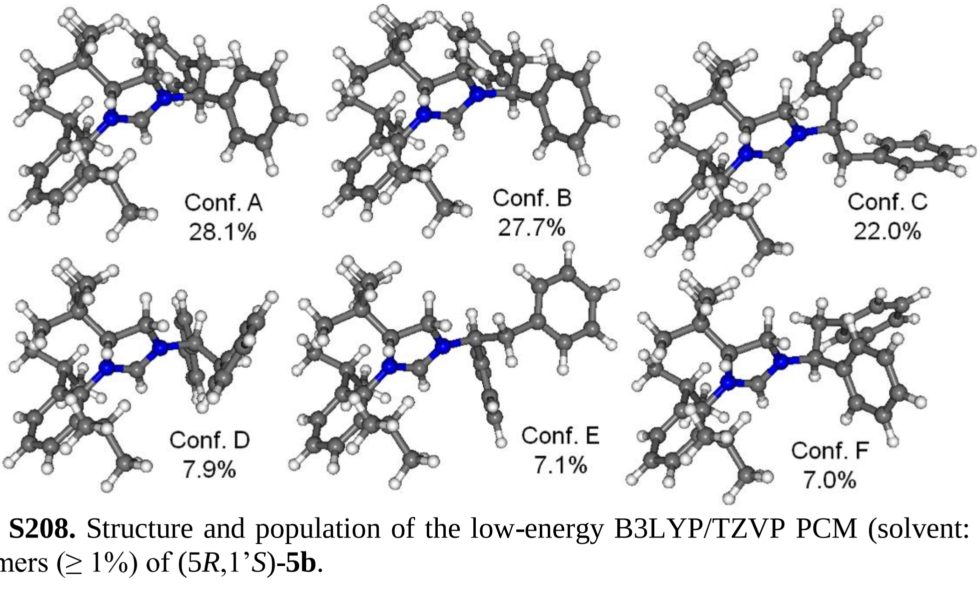

Figure S208. Structure and population of the low-energy B3LYP/TZVP PCM (solvent: $CHCl_3$) conformers (≥ 1%) of (5*R*,1'*S*)-**5b**.

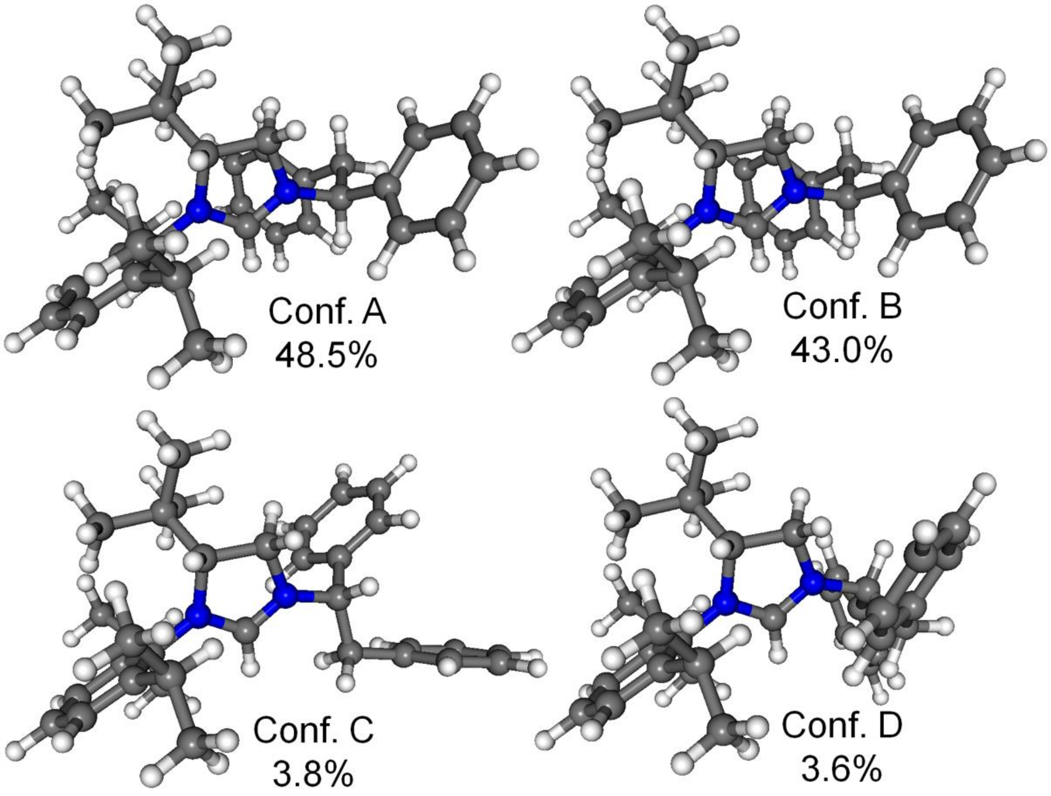

Figure S209. Structure and population of the low-energy ωB97X/TZVP PCM (solvent: acetonitrile) conformers (≥ 1%) of (5*R*,1'*S*)-**5b**.

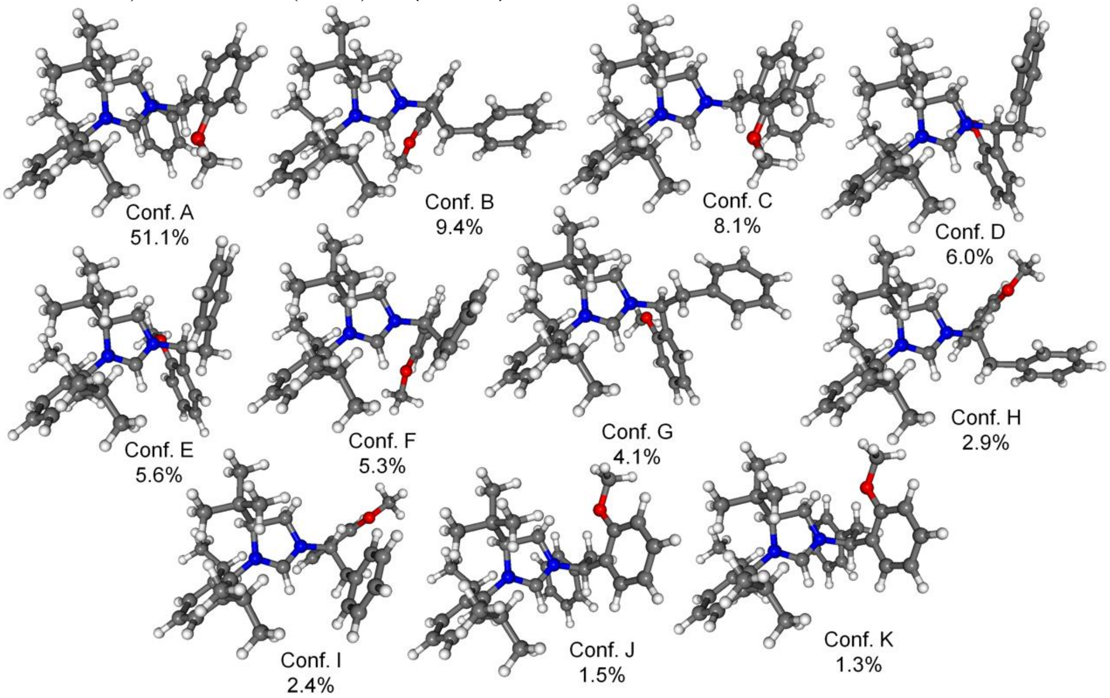

Figure S210. Structure and population of the low-energy B3LYP/TZVP PCM (solvent: $CHCl_3$) conformers (≥ 1%) of (5*S*,1'*S*)**-8a**.

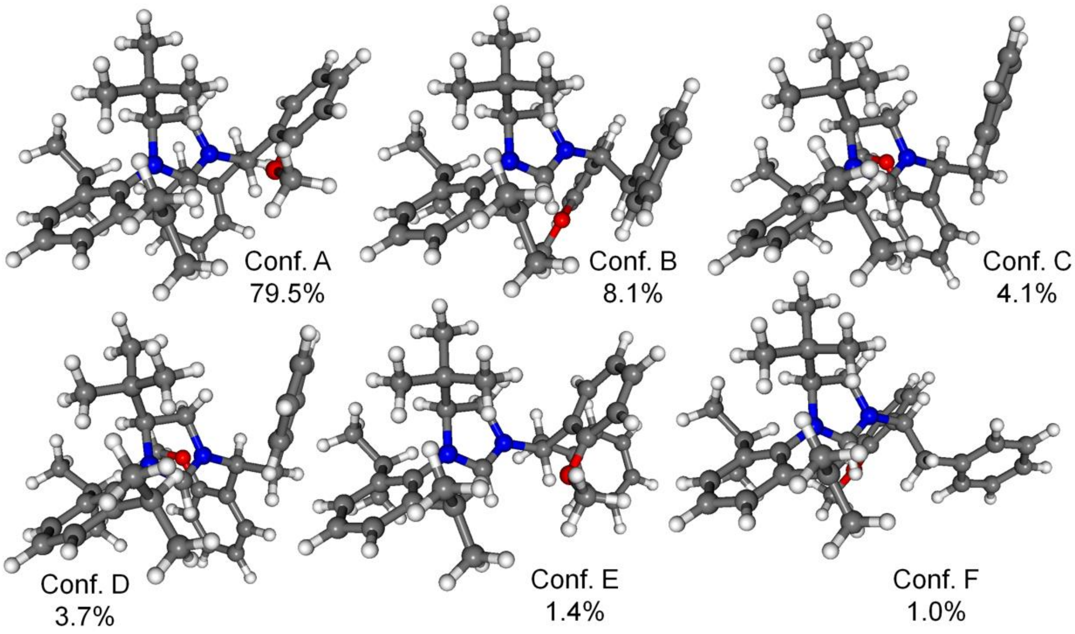

Figure S211. Structure and population of the low-energy ωB97X/TZVP PCM (solvent: acetonitrile) conformers (≥ 1%) of (5*S*,1'*S*)-**8a**.

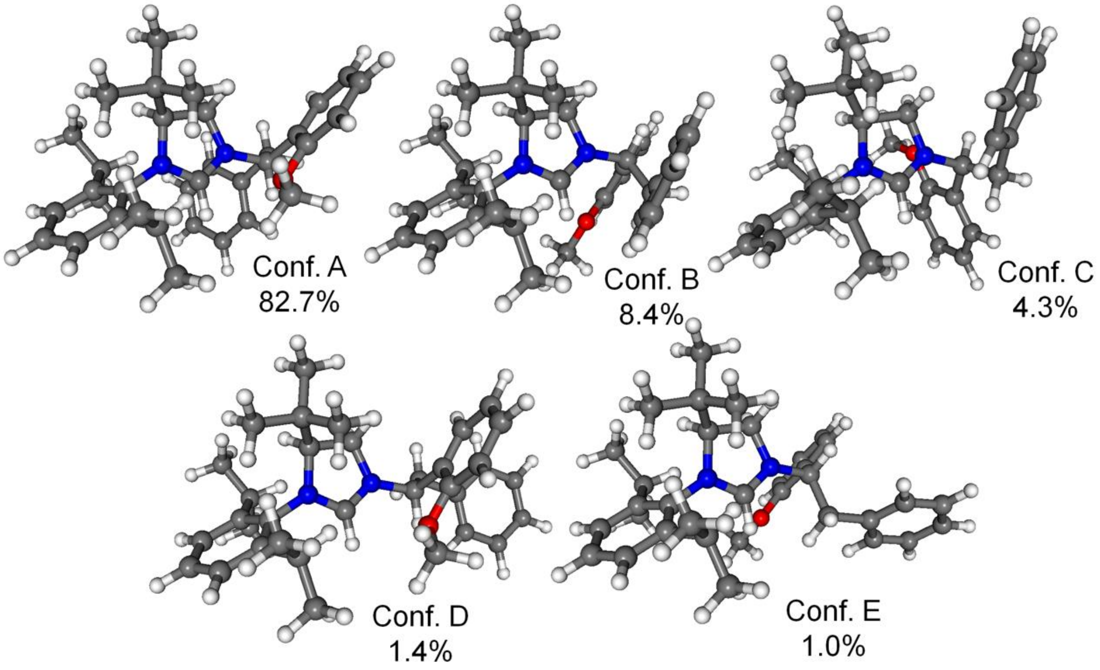

Figure S212. Structure and population of the low-energy ωB97X/TZVP PCM (solvent: methanol) conformers (≥ 1%) of (5*S*,1'*S*)-**8a**.

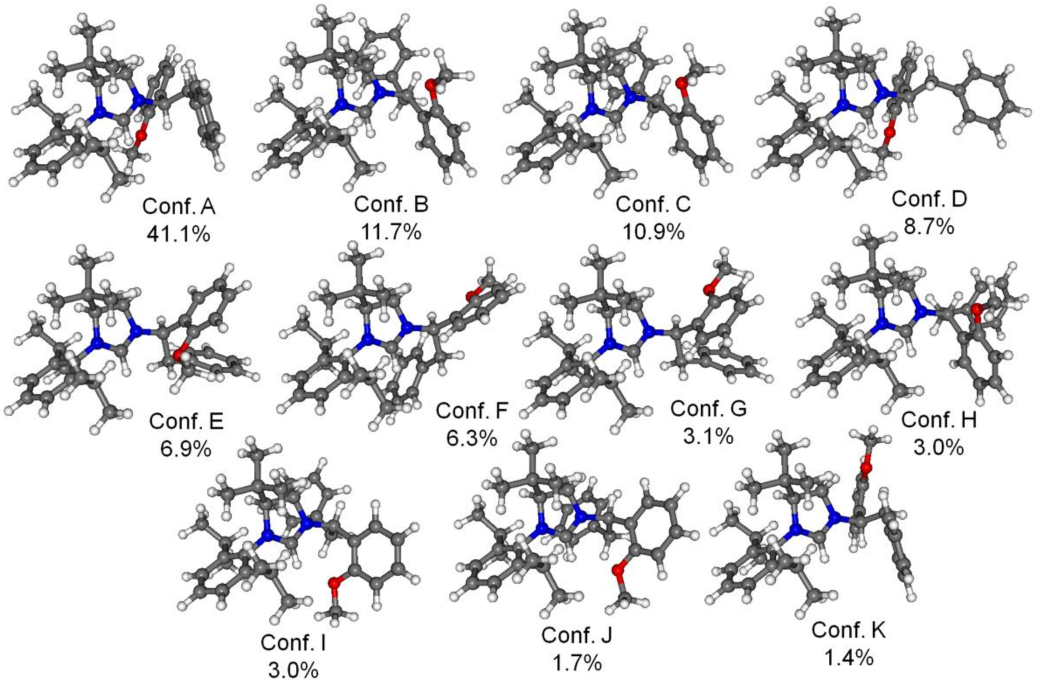

Figure S213. Structure and population of the low-energy B3LYP/TZVP PCM (solvent: $CHCl_3$) conformers (≥ 1%) of (5*S*,1'*R*)-**8b**.

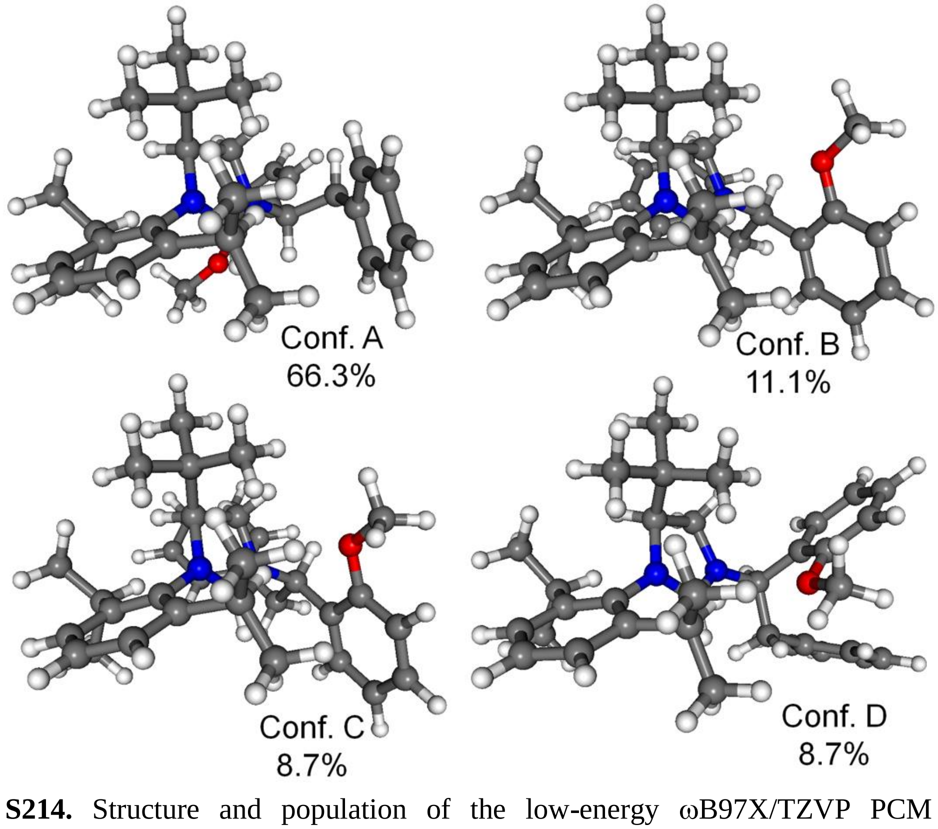

Figure S214. Structure and population of the low-energy ωB97X/TZVP PCM (solvent: acetonitrile) conformers (≥ 1%) of (5*S*,1'*R*)-**8b**.

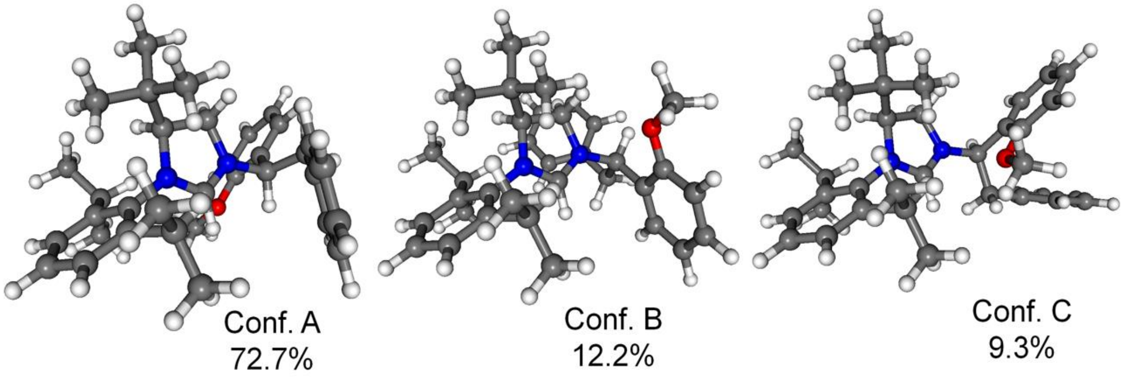

Figure S215. Structure and population of the low-energy ωB97X/TZVP PCM (solvent: methanol) conformers (≥ 1%) of (5*S*,1'*R*)-**8b**.

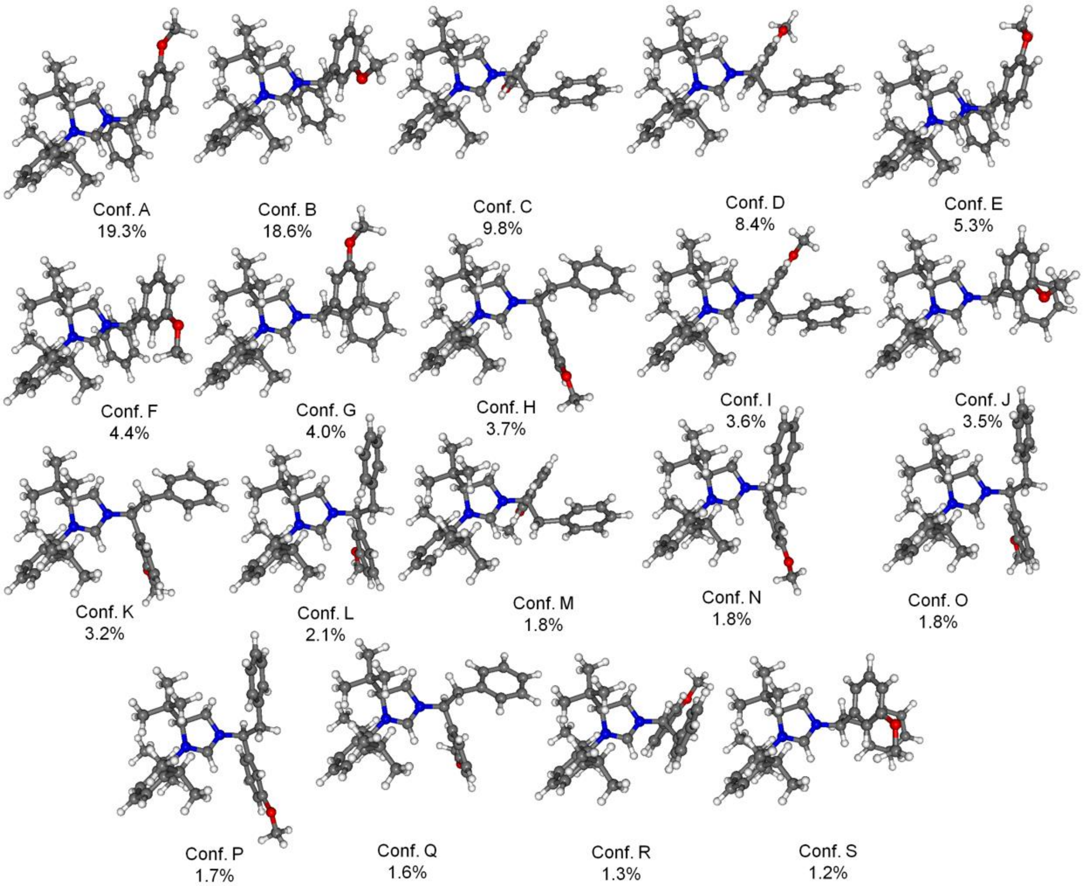

Figure S216. Structure and population of the low-energy B3LYP/TZVP PCM (solvent: $CHCl_3$) conformers (≥ 1%) of (5*S*,1'*S*)-**9a**.

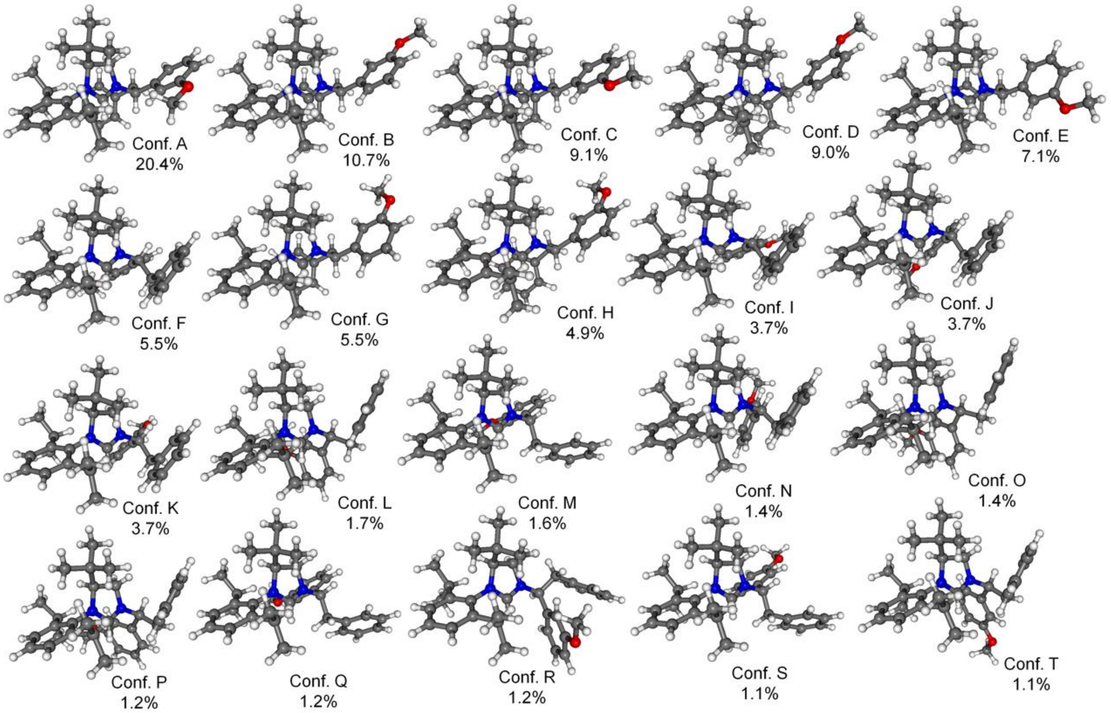

Figure S217. Structure and population of the low-energy ωB97X/TZVP PCM (solvent: acetonitrile) conformers (≥ 1%) of (5*S*,1'*S*)-**9a**.

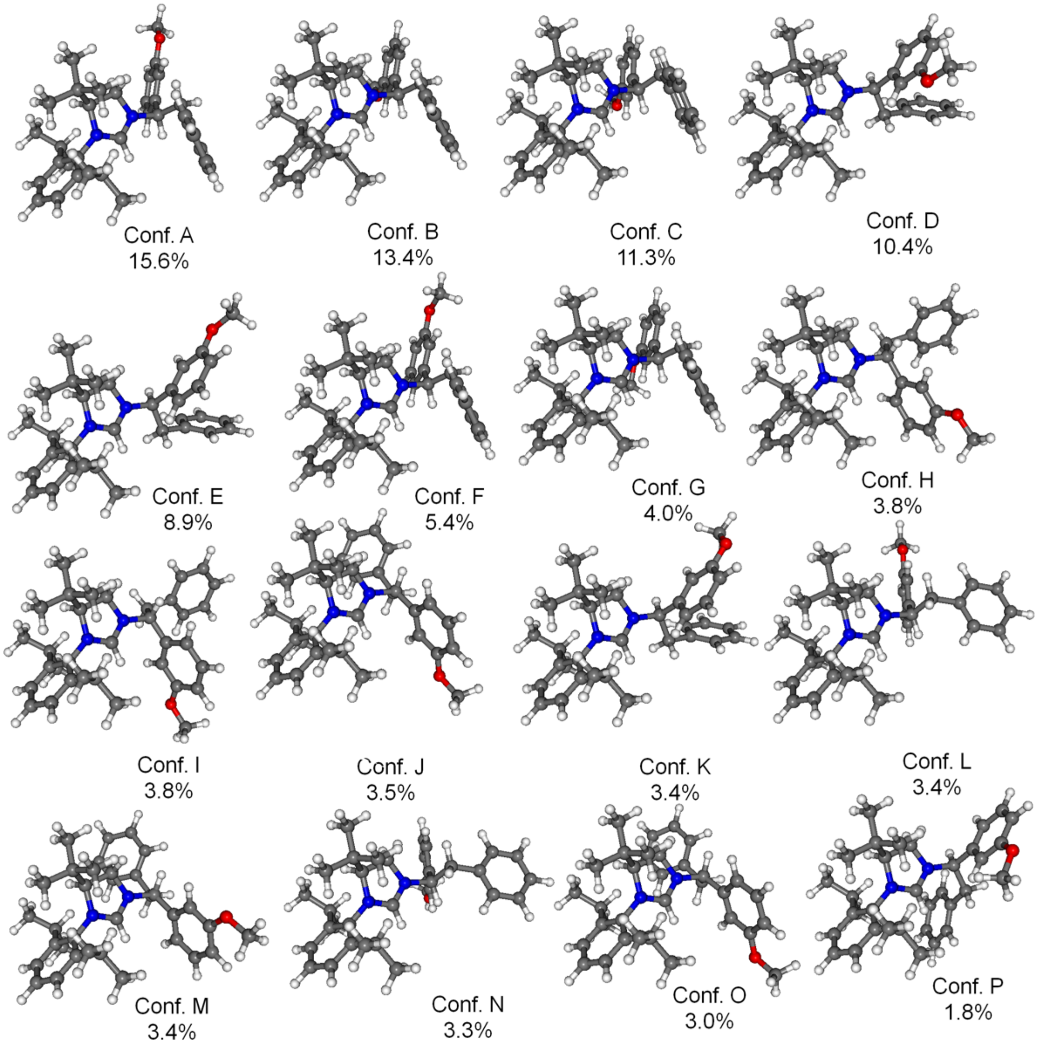

Figure S218. Structure and population of the low-energy B3LYP/TZVP PCM (solvent: $CHCl_3$) conformers (≥ 1%) of (5*S*,1'*R*)-**9b**.

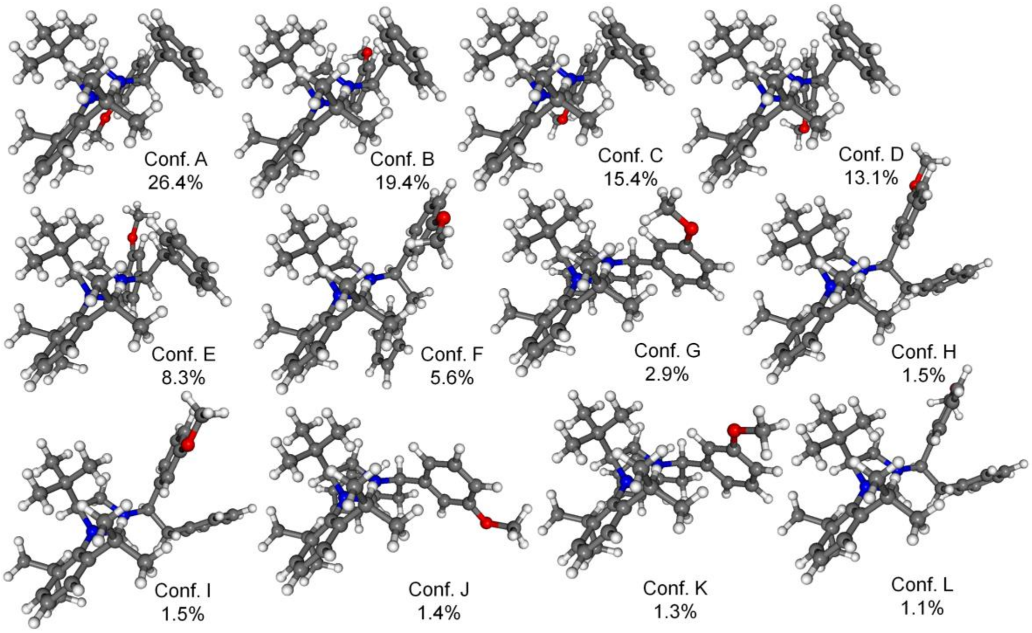

Figure S219. Structure and population of the low-energy ωB97X/TZVP PCM (solvent: acetonitrile) conformers (≥ 1%) of (5*S*,1'*R*)-**9b**.

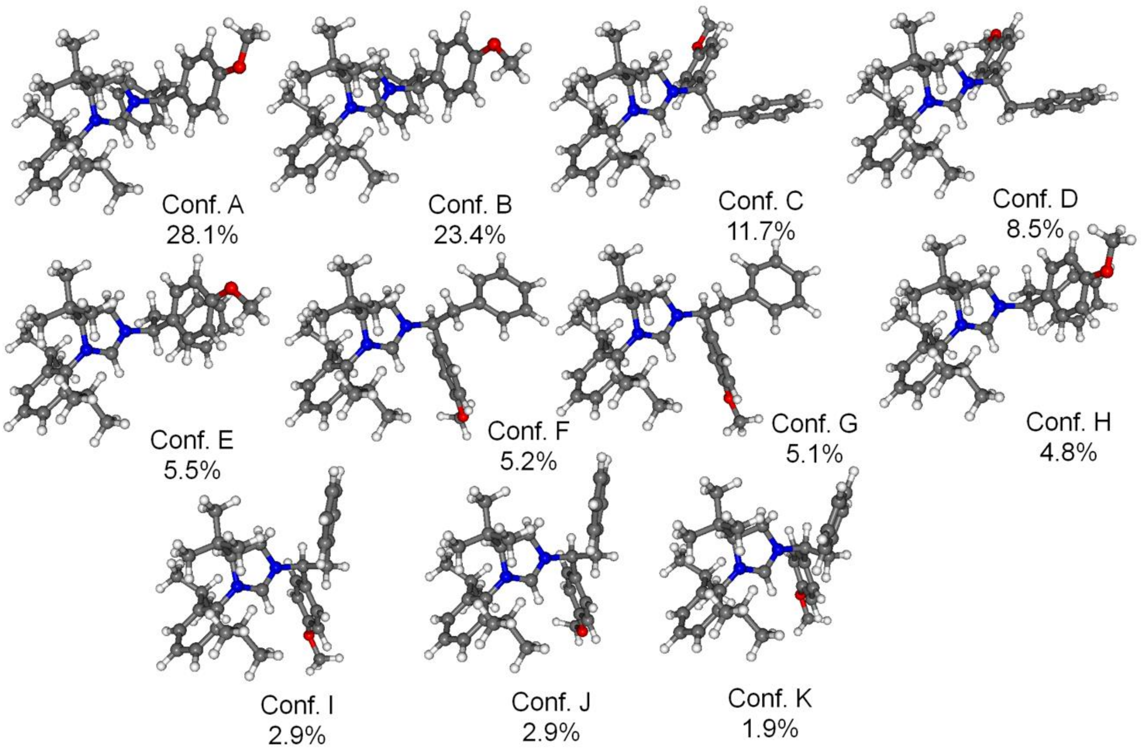

Figure S220. Structure and population of the low-energy B3LYP/TZVP PCM (solvent: $CHCl_3$) conformers (≥ 1%) of (5*S*,1'*S*)-**10a**.

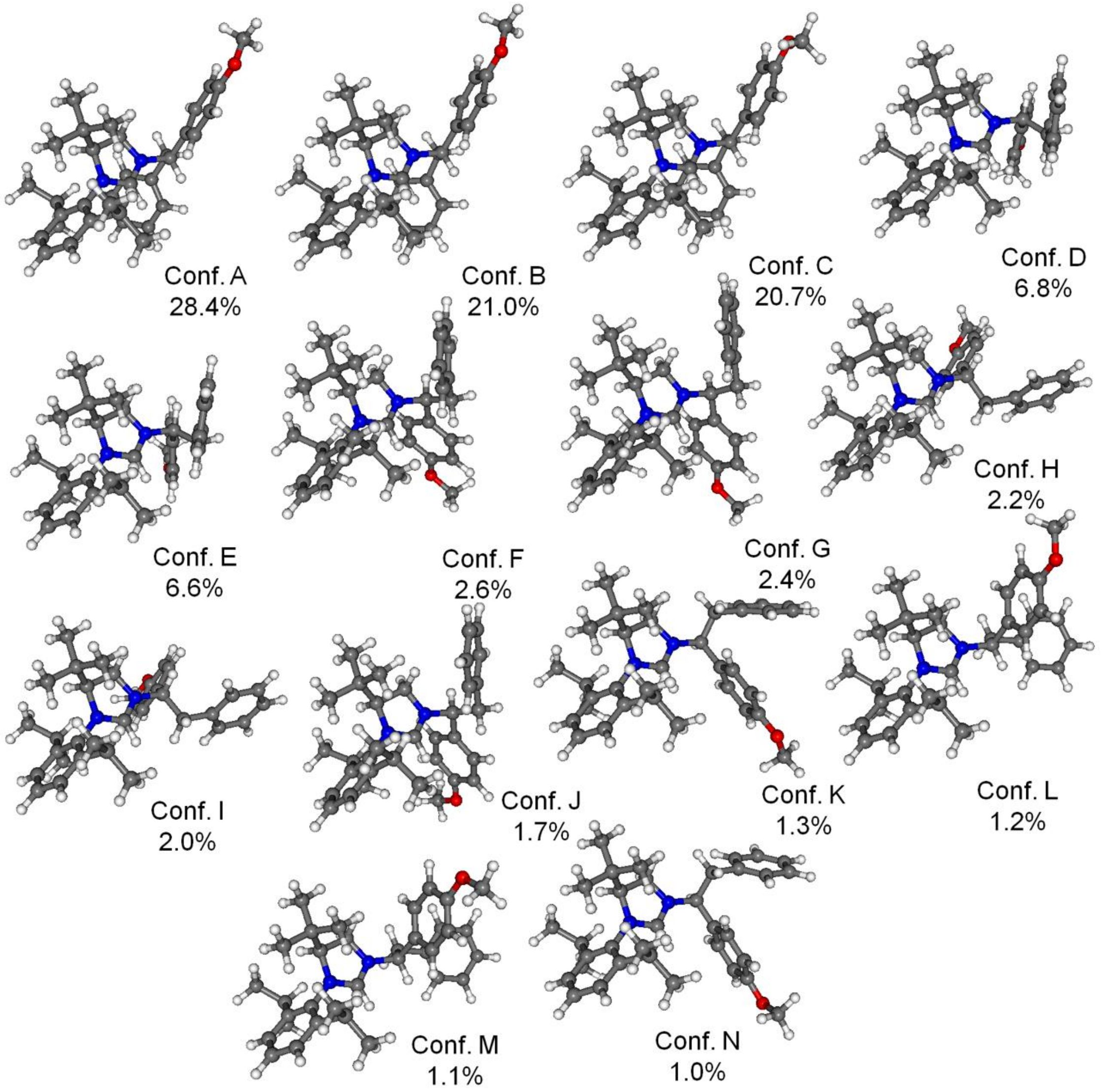

Figure S221. Structure and population of the low-energy ωB97X/TZVP PCM (solvent: acetonitrile) conformers (≥ 1%) of (5*S*,1'*S*)**-10a**.

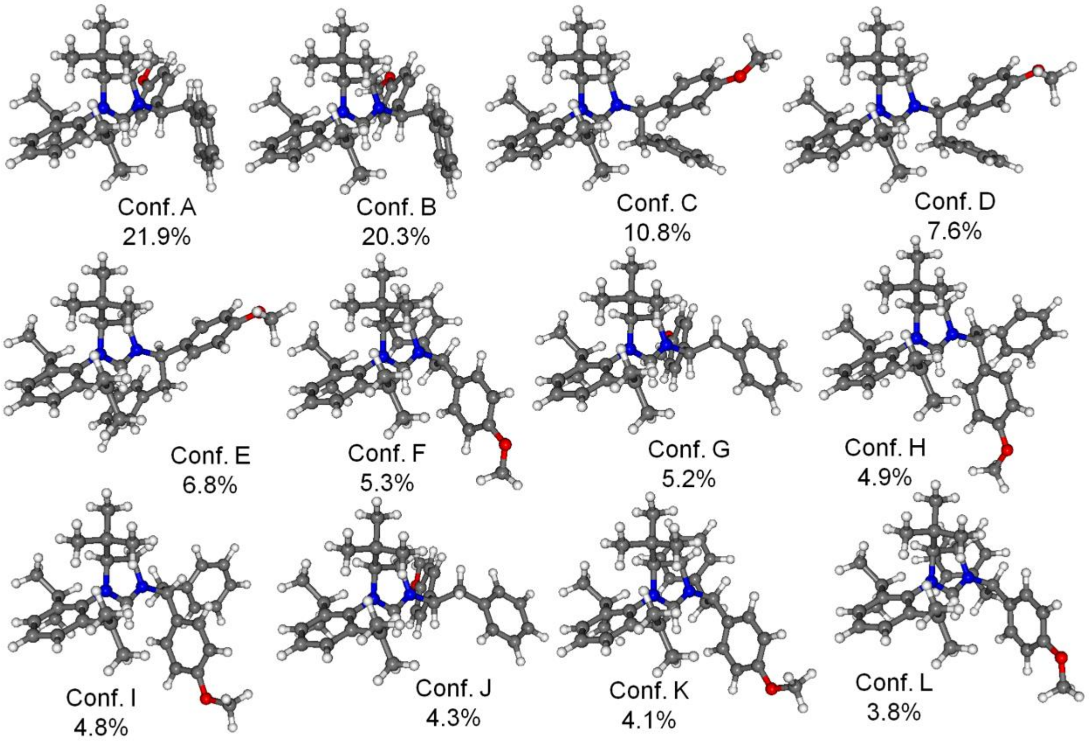

Figure S222. Structure and population of the low-energy B3LYP/TZVP PCM (solvent: $CHCl_3$) conformers (≥ 1%) of (5*S*,1'*R*)-**10b**.

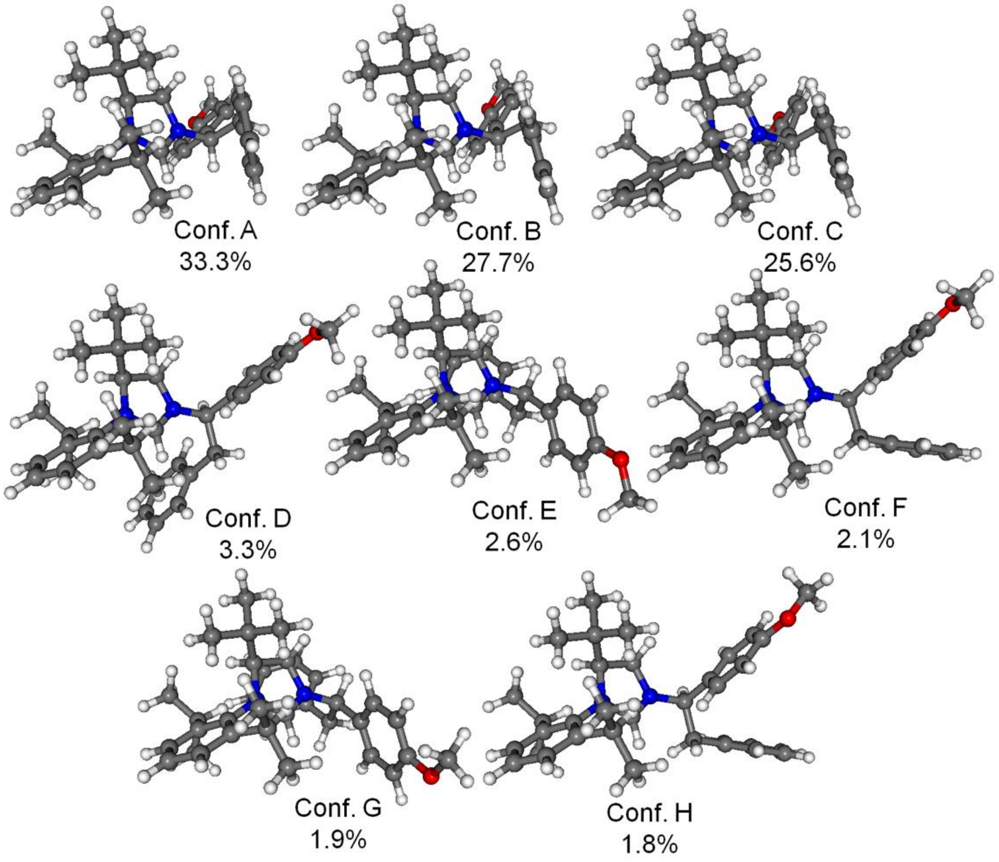

Figure S223. Structure and population of the low-energy ωB97X/TZVP PCM (solvent: acetonitrile) conformers (≥ 1%) of (5*S*,1'*R*)-**10b**.

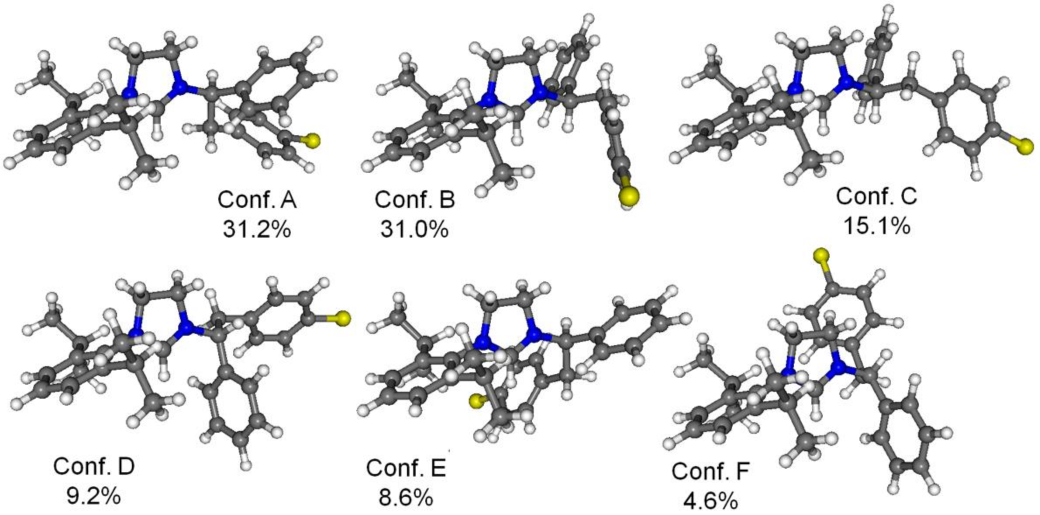

Figure S224. Structure and population of the low-energy B3LYP/TZVP PCM (solvent: DMSO) conformers (≥ 1%) of (*R*)-**17a**.

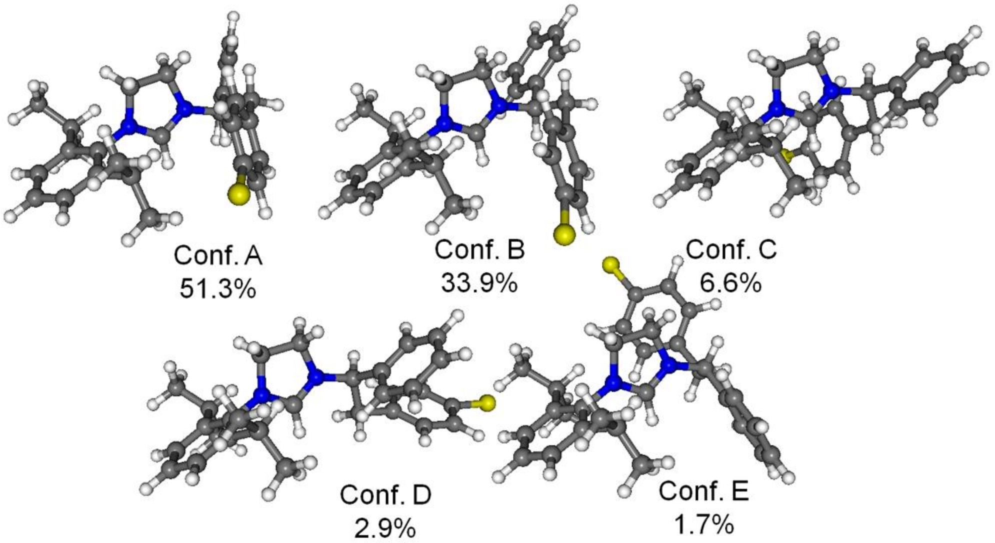

Figure S225. Structure and population of the low-energy ωB97X/TZVP PCM (solvent: acetonitrile) conformers (≥ 1%) of (*R*)-**17a**.

8. Cartesian coordinates

Table S9. Cartesian coordinates and energies of the low-energy conformers of (5*S*,1’*S*)-**1a,** (5*S*,1’R)-**1b,** (5*S*,1’*S*)-**2a**, (5*S*,1’*R*)-**2b**, (5*S*,1’*R*)-**3a**, (5*S*,1’*R*)-**3b**, (5*S*,1’*S*)-**4a**, (5*S*,1’*R*)-**4b**, (5*R*,1’*R*)-**5a**, (5*R*,1’*S*)-**5b**, (5*S*,1’*S*)-**8a**, (5*S*,1’*R*)-**8b**, (5*S*,1’*S*)-**9a**, (5*S*,1’*R*)-**9b**, (5*S*,1’*S*)-**10a**, (5*S*,1’*R*)-**10b** and (*R*)-**17a**, calculated at the B3LYP/TZVP PCM (solvent: $CHCl_3$ or DMSO in case of (*R*)-17a.) level. Charge: +1.

(5*S*,1’*S*)-**1a**, Conf. A

C	-0.166200	-0.529468	1.414755
N	-0.683318	-0.204136	0.028641
C	0.264949	-0.434787	-0.862960
N	1.384320	-0.899340	-0.372629
C	1.278457	-1.009758	1.094371
C	-0.993056	-1.516346	2.288819
C	-1.875963	0.558941	-0.300893
C	2.605895	-1.315646	-1.097082
C	-2.938465	-0.077014	-0.973153
C	-4.079033	0.682017	-1.240781
C	-4.165082	2.013287	-0.865155
C	-3.087112	2.629099	-0.248865
C	-1.912199	1.928570	0.027370
C	-0.241215	-1.633661	3.632585
C	-1.129223	-2.913022	1.664567
C	-2.388447	-0.938261	2.581365
C	-0.726015	2.691963	0.607698
C	-2.883610	-1.516583	-1.469592
C	-4.079065	-2.356260	-0.991218
C	-2.774338	-1.562572	-3.005092
C	-0.253366	3.794960	-0.356970
C	-1.030475	3.273402	1.998213
C	3.789383	-0.455233	-0.677685
C	2.392038	-1.337683	-2.609303
C	4.931530	-1.058322	-0.153175
C	6.037619	-0.290461	0.202289
C	6.006951	1.090308	0.041804
C	4.867498	1.700593	-0.477782
C	3.766196	0.932054	-0.836917
H	-0.125842	0.409857	1.963411
H	0.116798	-0.238556	-1.914348
H	2.040984	-0.390550	1.563477
H	1.449032	-2.045195	1.383487
H	2.799993	-2.338343	-0.764576
H	-4.913301	0.222740	-1.754598
H	-5.065687	2.577701	-1.072663
H	-3.151123	3.679765	0.003158
H	-0.120748	-0.657831	4.109187
H	0.746917	-2.084757	3.523618
H	-0.812883	-2.266619	4.312851
H	-0.163337	-3.386542	1.477511
H	-1.675010	-3.563510	2.350063
H	-1.682448	-2.891341	0.726983
H	-3.001476	-0.853613	1.686083
H	-2.321782	0.051475	3.038489
H	-2.912179	-1.590890	3.282269
H	0.112370	2.004454	0.716059
H	-1.982678	-1.980696	-1.070816
H	-3.948892	-3.396372	-1.297084
H	-4.181886	-2.333321	0.094619
H	-5.016682	-2.002910	-1.423506
H	-2.672831	-2.595551	-3.344601
H	-1.912063	-0.999634	-3.368896
H	-3.664967	-1.141178	-3.475366
H	0.639867	4.280984	0.041087
H	-0.009687	3.386702	-1.339947
H	-1.016579	4.562872	-0.493913
H	-0.143564	3.764226	2.404124
H	-1.341888	2.498613	2.701421
H	-1.828452	4.016802	1.951110
H	3.300314	-1.713993	-3.077675
H	1.570351	-1.999858	-2.888372
H	2.205236	-0.341340	-3.012660
H	4.960509	-2.134231	-0.023024
H	6.919098	-0.772062	0.606645
H	6.864710	1.689623	0.320036
H	4.838587	2.775447	-0.605994
H	2.888294	1.421258	-1.243015

B3LYP energy = -1162.10128820 a.u.

(5*S*,1’*S*)-**1a**, Conf. B

C	0.226919	-0.877516	1.221233
N	0.692730	-0.195253	-0.048983
C	-0.218010	-0.358956	-0.992322
N	-1.289957	-1.010763	-0.625025
C	-1.172989	-1.405573	0.791235
C	0.223643	-0.052746	2.539914
C	2.045893	0.240655	-0.354064
C	-2.499665	-1.226362	-1.462303
C	2.309380	1.615305	-0.514645
C	3.629608	1.993759	-0.765546
C	4.644498	1.054897	-0.861247
C	4.350185	-0.295171	-0.751691
C	3.047090	-0.736715	-0.519208
C	1.656701	0.360743	2.916147
C	-0.296739	-1.005372	3.638628
C	-0.680125	1.187045	2.479027
C	2.772168	-2.237343	-0.530602

C	1.232594	2.693446	-0.485563
C	1.538923	3.809895	0.526309
C	1.012427	3.288399	-1.889105
C	3.039147	-2.828869	-1.927280
C	3.569706	-2.991139	0.546179
C	-3.669398	-0.409561	-0.927214
C	-2.771015	-2.720673	-1.627536
C	-3.840867	0.901600	-1.378423
C	-4.884487	1.688246	-0.904384
C	-5.774443	1.171534	0.032864
C	-5.615789	-0.133813	0.486081
C	-4.571726	-0.920961	0.007673
H	0.898948	-1.718270	1.386610
H	-0.077544	0.008464	-1.999292
H	-1.251001	-2.487259	0.882886
H	-1.981750	-0.948540	1.357542
H	-2.230716	-0.819176	-2.437643
H	3.865625	3.042386	-0.891097
H	5.662917	1.374535	-1.043880
H	5.143668	-1.021498	-0.870529
H	2.087336	1.064138	2.206077
H	2.317582	-0.506907	2.974829
H	1.652651	0.839353	3.897083
H	0.313443	-1.909430	3.705433
H	-0.251316	-0.502196	4.605455
H	-1.334215	-1.304254	3.477152
H	-1.718497	0.939559	2.250604
H	-0.334668	1.909045	1.741237
H	-0.676457	1.685075	3.450100
H	1.714794	-2.402792	-0.327694
H	0.294536	2.230169	-0.182742
H	0.700823	4.508127	0.573695
H	1.706420	3.414610	1.529051
H	2.424678	4.379810	0.240184
H	0.207375	4.025865	-1.862444
H	0.745958	2.522551	-2.620601
H	1.912969	3.789191	-2.249840
H	2.777238	-3.889027	-1.942047
H	2.448408	-2.321467	-2.692845
H	4.091266	-2.740692	-2.203788
H	3.298564	-4.048833	0.543063
H	3.377377	-2.594151	1.544732
H	4.644054	-2.926085	0.364926
H	-3.620548	-2.859771	-2.295845
H	-1.903261	-3.216115	-2.063898
H	-3.006345	-3.209709	-0.682579
H	-3.159064	1.310437	-2.115683
H	-5.006355	2.699725	-1.271170
H	-6.590127	1.780371	0.402121
H	-6.308907	-0.545364	1.209099
H	-4.477048	-1.938312	0.364854

B3LYP energy = -1162.10036783 a.u.

(5*S*,1'*S*)-**1a**, Conf. C

C	1.009728	-1.479951	-1.026530
N	0.592814	-0.319487	-0.148705
C	-0.720084	-0.335882	0.008133
N	-1.334801	-1.339739	-0.559929
C	-0.355771	-2.190443	-1.262738
C	2.137866	-2.420099	-0.514212
C	1.383195	0.864632	0.151810
C	-2.790716	-1.658867	-0.517726
C	1.800643	1.099513	1.476673
C	2.590618	2.225885	1.713982
C	2.948360	3.086285	0.688009
C	2.482430	2.861066	-0.597714
C	1.672752	1.763826	-0.893858
C	3.456342	-1.643964	-0.354513
C	2.345860	-3.480469	-1.617412
C	1.785573	-3.124649	0.803586
C	1.097740	1.641085	-2.301541
C	1.392612	0.229207	2.659565
C	2.598107	-0.260770	3.478448
C	0.391797	0.968585	3.567261
C	0.177240	2.833100	-2.622333
C	2.190726	1.488882	-3.371661
C	-3.602247	-0.434311	-0.130365
C	-3.042654	-2.899348	0.341974
C	-3.924787	-0.141738	1.196741
C	-4.659087	0.999106	1.509895
C	-5.083295	1.857793	0.501158
C	-4.771936	1.571397	-0.825000
C	-4.036660	0.433270	-1.136193
H	1.365982	-1.051921	-1.962684
H	-1.248303	0.436632	0.546930
H	-0.619086	-2.252826	-2.318204
H	-0.373603	-3.192913	-0.841732
H	-3.041487	-1.903860	-1.552080
H	2.929494	2.433844	2.720466
H	3.573195	3.946281	0.894893
H	2.732861	3.563578	-1.382216
H	3.411011	-0.903386	0.441471
H	3.730450	-1.129130	-1.277731
H	4.260987	-2.341129	-0.113740
H	2.606156	-3.016951	-2.572039
H	3.166153	-4.141698	-1.334802
H	1.463191	-4.104414	-1.769682
H	0.890855	-3.744811	0.722415
H	1.634891	-2.417743	1.617911
H	2.605122	-3.785102	1.091631
H	0.468720	0.752739	-2.346186
H	0.882372	-0.652006	2.273345
H	2.264375	-0.949301	4.257436
H	3.326336	-0.782889	2.856369
H	3.111668	0.566825	3.970631
H	0.072440	0.317548	4.383952
H	-0.497919	1.285398	3.019465
H	0.842993	1.860006	4.007448
H	-0.287325	2.694099	-3.600966
H	-0.616461	2.932796	-1.879477
H	0.732612	3.772272	-2.648071

H	1.737874	1.364799	-4.357448
H	2.827250	0.623295	-3.179250
H	2.833262	2.370243	-3.410521
H	-4.105636	-3.139078	0.323947
H	-2.500960	-3.761463	-0.047479
H	-2.740015	-2.744543	1.377604
H	-3.613822	-0.801769	1.996165
H	-4.904731	1.211210	2.542898
H	-5.659328	2.741317	0.745928
H	-5.107884	2.228857	-1.616986
H	-3.805898	0.210944	-2.171805

B3LYP energy = -1162.10003349 a.u.

(5*S*,1'*S*)-**1a**, Conf. D

C	1.026283	1.771103	0.375914
N	0.602062	0.344511	0.107931
C	-0.710736	0.244892	0.215298
N	-1.331825	1.361812	0.495390
C	-0.340182	2.433686	0.715715
C	1.833339	2.501687	-0.736505
C	1.466050	-0.822492	0.048599
C	-2.784278	1.536978	0.786753
C	1.592077	-1.532618	-1.162205
C	2.467913	-2.619348	-1.184587
C	3.186435	-2.992232	-0.059871
C	3.005949	-2.309413	1.132627
C	2.127993	-1.228496	1.224654
C	2.132418	3.918345	-0.200115
C	1.060582	2.614283	-2.058483
C	3.180480	1.798061	-0.974979
C	1.890743	-0.599259	2.594130
C	0.792845	-1.216107	-2.420962
C	1.687422	-1.007513	-3.654327
C	-0.253496	-2.311716	-2.697837
C	1.303968	-1.628242	3.577833
C	3.163439	0.042096	3.172206
C	-3.563051	0.274922	0.466191
C	-3.323751	2.785141	0.085209
C	-3.886320	-0.072905	-0.849079
C	-4.611555	-1.229243	-1.114246
C	-5.026775	-2.050730	-0.069126
C	-4.711290	-1.711922	1.242142
C	-3.981987	-0.555991	1.506349
H	1.658438	1.754651	1.261494
H	-1.227838	-0.694922	0.097184
H	-0.396032	2.769629	1.752019
H	-0.557430	3.276997	0.067480
H	-2.843319	1.702191	1.866077
H	2.588095	-3.183545	-2.100100
H	3.870579	-3.830383	-0.106733
H	3.539884	-2.636451	2.015563
H	2.665607	3.881194	0.752892
H	1.229335	4.516099	-0.062832
H	2.765427	4.448542	-0.913201
H	0.109728	3.138661	-1.944740
H	1.652351	3.180747	-2.779525
H	0.858721	1.638753	-2.498053
H	3.062784	0.797341	-1.385436
H	3.757300	1.716540	-0.051130
H	3.773495	2.379378	-1.683318
H	1.141523	0.185758	2.494427
H	0.248631	-0.287390	-2.256241
H	1.078956	-0.701514	-4.507824
H	2.441659	-0.237807	-3.484691
H	2.205183	-1.926678	-3.933782
H	-0.855849	-2.044733	-3.568841
H	-0.928456	-2.455238	-1.851689
H	0.226434	-3.270412	-2.904161
H	1.066577	-1.143814	4.527336
H	0.389328	-2.077198	3.185926
H	2.010572	-2.433933	3.783382
H	2.944711	0.514220	4.132219
H	3.575444	0.803016	2.506467
H	3.941271	-0.705029	3.340458
H	-4.382974	2.887347	0.319329
H	-2.819963	3.687354	0.432225
H	-3.216171	2.720924	-0.997684
H	-3.576694	0.557546	-1.673507
H	-4.858625	-1.485258	-2.136893
H	-5.596661	-2.947595	-0.277021
H	-5.035404	-2.342463	2.060485
H	-3.745361	-0.294582	2.531398

B3LYP energy = -1162.09977901 a.u.

(5*S*,1'*R*)-**1b**, Conf. A

C	0.214935	-0.848585	1.266013
N	0.691077	-0.218772	-0.027367
C	-0.224546	-0.392801	-0.965558
N	-1.314458	-0.996963	-0.569732
C	-1.201404	-1.344481	0.858864
C	0.244462	0.012376	2.561272
C	2.052319	0.178975	-0.349610
C	-2.539632	-1.295460	-1.343533
C	2.342581	1.541950	-0.558306
C	3.669109	1.886224	-0.824439
C	4.666069	0.925770	-0.887689
C	4.346420	-0.413504	-0.728784
C	3.035531	-0.822076	-0.480210
C	-0.301696	-0.892974	3.687301
C	-0.620181	1.278219	2.469803
C	1.690882	0.391937	2.922212
C	2.736408	-2.316903	-0.423596
C	1.288539	2.642819	-0.560055
C	1.623676	3.782324	0.416777
C	1.076605	3.201852	-1.979377
C	3.087034	-3.002980	-1.756840
C	3.450374	-3.011296	0.748347
C	-3.732456	-0.535911	-0.778482
C	-2.339302	-1.057751	-2.838701

C	-3.726731	0.857992	-0.693348
C	-4.838098	1.539230	-0.210404
C	-5.970301	0.834153	0.191323
C	-5.982965	-0.553964	0.111360
C	-4.866993	-1.235086	-0.368244
H	0.862465	-1.704297	1.451072
H	-0.065127	-0.070060	-1.983221
H	-1.320818	-2.420471	0.980069
H	-1.994574	-0.844798	1.410690
H	-2.716491	-2.362400	-1.187707
H	3.924471	2.925152	-0.986908
H	5.690069	1.219205	-1.082609
H	5.126343	-1.158237	-0.820996
H	0.278523	-1.814764	3.775915
H	-1.348977	-1.161955	3.537333
H	-0.235427	-0.366066	4.640191
H	-0.252043	1.971018	1.715145
H	-1.665776	1.057767	2.246882
H	-0.600402	1.799292	3.428473
H	1.703416	0.904570	3.885779
H	2.143363	1.055922	2.187970
H	2.323917	-0.493610	3.011955
H	1.664305	-2.453451	-0.284308
H	0.341949	2.208738	-0.240583
H	2.522756	4.320350	0.111357
H	0.803361	4.502447	0.443557
H	1.783855	3.414404	1.431048
H	0.288572	3.957889	-1.971870
H	0.790969	2.422513	-2.688873
H	1.986743	3.672113	-2.356678
H	4.159555	-2.968051	-1.955077
H	2.789355	-4.053227	-1.725234
H	2.575863	-2.529286	-2.597343
H	3.173371	-4.066831	0.786418
H	3.193417	-2.558214	1.707695
H	4.534982	-2.957163	0.637593
H	-1.506716	-1.645436	-3.228873
H	-3.243940	-1.369540	-3.358701
H	-2.177905	-0.003595	-3.068729
H	-2.853978	1.420824	-1.003241
H	-4.822253	2.620293	-0.149945
H	-6.836195	1.365570	0.565701
H	-6.858435	-1.109107	0.424114
H	-4.882733	-2.317653	-0.424934

B3LYP energy = -1162.10129004 a.u.

(5*S*,1’*R*)-**1b**, Conf. B

C	-0.115700	-0.470016	1.339543
N	-0.689918	-0.161287	-0.027585
C	0.238222	-0.358629	-0.947125
N	1.389470	-0.780036	-0.494882
C	1.340058	-0.888277	0.975361
C	-0.875109	-1.496705	2.228441
C	-1.919875	0.559768	-0.311450
C	2.576750	-1.110775	-1.325356
C	-2.978841	-0.108747	-0.957282
C	-4.158663	0.606081	-1.170553
C	-4.286410	1.925511	-0.766197
C	-3.212591	2.575904	-0.178809
C	-1.999665	1.921689	0.040646
C	-0.075178	-1.602101	3.544962
C	-0.981717	-2.888315	1.587964
C	-2.280670	-0.974979	2.573762
C	-0.824914	2.724544	0.590190
C	-2.881351	-1.535533	-1.482999
C	-4.017522	-2.436993	-0.973253
C	-2.832044	-1.547435	-3.022281
C	-0.459905	3.887927	-0.350098
C	-1.087521	3.241193	2.014862
C	3.771976	-0.283685	-0.868810
C	2.797270	-2.622507	-1.371354
C	4.754053	-0.794503	-0.019806
C	5.821418	0.002838	0.386211
C	5.920849	1.317976	-0.053891
C	4.948120	1.835381	-0.905486
C	3.883337	1.038314	-1.308630
H	-0.098101	0.467041	1.893219
H	0.059805	-0.169432	-1.996714
H	2.087021	-0.229354	1.413906
H	1.562093	-1.909739	1.274616
H	2.310695	-0.775521	-2.328889
H	-4.990898	0.120997	-1.663522
H	-5.216929	2.454795	-0.929591
H	-3.311062	3.618503	0.094688
H	0.031948	-0.626446	4.025246
H	0.921818	-2.022030	3.398916
H	-0.603837	-2.257574	4.238633
H	-1.571607	-2.873387	0.672979
H	-0.006643	-3.321409	1.356293
H	-1.475739	-3.569528	2.282889
H	-2.757476	-1.656388	3.280644
H	-2.924659	-0.900774	1.699545
H	-2.235956	0.009614	3.044220
H	0.051698	2.078107	0.627614
H	-1.944878	-1.965228	-1.129973
H	-4.988123	-2.115441	-1.354600
H	-3.856940	-3.463524	-1.309137
H	-4.071872	-2.442887	0.116388
H	-2.694270	-2.567318	-3.387586
H	-2.013465	-0.935326	-3.407007
H	-3.760001	-1.161166	-3.448533
H	-1.264846	4.622181	-0.411159
H	0.430108	4.400868	0.020315
H	-0.252313	3.533500	-1.361569
H	-1.917763	3.949789	2.029880
H	-0.204444	3.756698	2.398005
H	-1.333542	2.431013	2.703806
H	1.917348	-3.116398	-1.784388
H	3.649667	-2.845988	-2.012903
H	2.996706	-3.046532	-0.387365
H	4.702997	-1.818338	0.326458

H	6.577814	-0.408777	1.042685
H	6.753890	1.934942	0.258784
H	5.023071	2.855245	-1.261501
H	3.135553	1.446915	-1.979389

B3LYP energy = -1162.10028630 a.u.

(5*S*,1'*R*)-**1b**, Conf. C

C	1.022744	-1.801431	0.122831
N	0.594696	-0.352111	0.048676
C	-0.721626	-0.285008	-0.049475
N	-1.340444	-1.437632	-0.034583
C	-0.353465	-2.526694	0.106912
C	1.959374	-2.229116	1.288507
C	1.447047	0.783866	-0.265302
C	-2.808119	-1.682701	0.062349
C	1.675029	1.770412	0.714199
C	2.542498	2.814193	0.387140
C	3.150929	2.886502	-0.856019
C	2.865760	1.934025	-1.821544
C	1.993042	0.876774	-1.560848
C	2.211331	-3.741624	1.105145
C	1.346493	-1.983250	2.674262
C	3.315816	-1.510899	1.183381
C	1.627493	-0.065072	-2.704187
C	0.985410	1.791886	2.072999
C	1.977818	1.902436	3.242155
C	-0.051040	2.930076	2.140996
C	0.895816	0.697281	-3.824726
C	2.847195	-0.817098	-3.260571
C	-3.594593	-0.388438	-0.021083
C	-3.236485	-2.729110	-0.969202
C	-4.150597	0.151821	1.139726
C	-4.891158	1.329389	1.091439
C	-5.079523	1.983209	-0.121422
C	-4.528717	1.452575	-1.285156
C	-3.794679	0.272464	-1.236803
H	1.558035	-2.017355	-0.800872
H	-1.243516	0.654106	-0.152944
H	-0.440959	-3.222041	-0.724690
H	-0.553559	-3.066885	1.031796
H	-2.962107	-2.102949	1.060115
H	2.743129	3.585512	1.119037
H	3.830194	3.699535	-1.080301
H	3.311555	2.024753	-2.803624
H	2.634320	-3.960115	0.121511
H	1.302997	-4.335089	1.225479
H	2.924242	-4.084132	1.856473
H	0.393593	-2.498433	2.809518
H	2.024147	-2.360100	3.442182
H	1.190493	-0.923794	2.869720
H	3.784400	-1.684429	0.212279
H	3.991072	-1.896936	1.949190
H	3.231446	-0.435628	1.327984
H	0.923431	-0.810303	-2.335284
H	0.443028	0.855057	2.195217
H	2.726299	1.109305	3.215735
H	2.503964	2.858589	3.233479
H	1.443312	1.830984	4.191639
H	-0.584104	2.897372	3.093573
H	-0.787800	2.860603	1.338214
H	0.432375	3.905825	2.061232
H	0.573471	0.001654	-4.602427
H	0.013382	1.214159	-3.441977
H	1.544064	1.441133	-4.291013
H	2.535813	-1.512315	-4.042933
H	3.360470	-1.387712	-2.484433
H	3.571905	-0.128666	-3.699238
H	-2.731758	-3.681197	-0.805432
H	-4.308092	-2.900196	-0.872143
H	-3.031340	-2.398740	-1.987605
H	-4.011726	-0.355492	2.087463
H	-5.321978	1.731549	1.999742
H	-5.656169	2.898711	-0.162121
H	-4.677311	1.953714	-2.233425
H	-3.383234	-0.130651	-2.154000

B3LYP energy = -1162.10019331 a.u.

(5*S*,1'*S*)-**2a**, Conf. A

C	1.501625	-0.575375	-1.666057
N	1.123564	-0.188236	-0.251575
C	-0.080487	-0.655537	0.028026
N	-0.644558	-1.342778	-0.930766
C	0.245231	-1.382302	-2.106146
C	2.849881	-1.320214	-1.886068
C	1.766786	0.823111	0.571873
C	-1.929565	-2.094043	-0.863845
C	1.674515	2.172673	0.178136
C	2.352840	3.118951	0.947576
C	3.053056	2.753881	2.086575
C	3.068277	1.429093	2.493169
C	2.428067	0.433372	1.753350
C	4.033408	-0.430677	-1.468437
C	2.961624	-1.566804	-3.406670
C	2.924549	-2.665557	-1.149342
C	2.433531	-0.993139	2.289708
C	0.826834	2.658941	-0.992992
C	-0.313124	3.570202	-0.502478
C	1.666477	3.366427	-2.069460
C	3.851187	-1.514524	2.575936
C	1.552495	-1.107191	3.547770
C	-2.875915	-1.452156	0.140389
C	-1.651881	-3.583947	-0.650170
C	-3.571812	-0.244278	-0.199572
C	-4.459028	0.333009	0.765386
C	-4.629334	-0.296257	2.023759
C	-3.957096	-1.449880	2.322255
C	-3.082962	-2.024214	1.375185
C	-3.432779	0.417184	-1.447976
C	-4.128486	1.567865	-1.728024

C	-5.000690	2.130438	-0.773842
C	-5.160012	1.522719	0.444618
H	1.561255	0.349230	-2.238010
H	-0.570935	-0.465778	0.971767
H	-0.259823	-0.935506	-2.962060
H	0.483478	-2.414867	-2.350106
H	-2.360851	-1.983185	-1.860078
H	2.313037	4.162200	0.662187
H	3.568884	3.506127	2.670328
H	3.586752	1.161021	3.404239
H	4.969902	-0.925286	-1.732593
H	4.052178	-0.236680	-0.397874
H	4.011300	0.530686	-1.986058
H	2.906302	-0.630378	-3.966857
H	3.924558	-2.029049	-3.628412
H	2.186251	-2.236618	-3.783593
H	2.132449	-3.353623	-1.450974
H	2.874436	-2.543975	-0.068423
H	3.873596	-3.152978	-1.379106
H	1.996603	-1.643950	1.533600
H	0.348649	1.798582	-1.461046
H	-0.949489	3.857876	-1.341893
H	-0.935741	3.065001	0.237922
H	0.073403	4.484219	-0.048428
H	1.035983	3.643944	-2.916761
H	2.472333	2.730149	-2.439625
H	2.119595	4.280698	-1.681636
H	3.809196	-2.564812	2.871799
H	4.496178	-1.435380	1.699681
H	4.325146	-0.964001	3.390293
H	1.514916	-2.143597	3.889977
H	0.529743	-0.775220	3.357592
H	1.950234	-0.499435	4.363032
H	-2.590484	-4.137564	-0.657459
H	-1.030316	-3.974757	-1.455872
H	-1.140210	-3.773032	0.293124
H	-5.304750	0.150507	2.743646
H	-4.093018	-1.933538	3.281073
H	-2.577942	-2.942920	1.639413
H	-2.776900	0.013500	-2.206763
H	-4.009285	2.047084	-2.691823
H	-5.543145	3.037523	-1.008391
H	-5.829776	1.944257	1.184784

B3LYP energy = -1315.78590055 a.u.

(5*S*,1'*S*)-**2a**, Conf. B

C	-0.940397	-0.293196	1.502279
N	-1.424225	-0.178395	0.072363
C	-0.408913	-0.353904	-0.754590
N	0.739471	-0.598180	-0.178420
C	0.573661	-0.583277	1.288657
C	-1.661733	-1.307911	2.436395
C	-2.691632	0.375238	-0.377183
C	2.030983	-0.923973	-0.815315
C	-2.930327	1.750794	-0.187173
C	-4.175455	2.250095	-0.571682
C	-5.127579	1.434696	-1.162943
C	-4.841020	0.100443	-1.404639
C	-3.622082	-0.462718	-1.023214
C	-3.139711	-0.921050	2.616461
C	-0.970978	-1.190606	3.812646
C	-1.562845	-2.759085	1.943956
C	-3.345399	-1.918934	-1.378166
C	-1.887178	2.728770	0.344670
C	-1.504206	3.759758	-0.733016
C	-2.343724	3.428299	1.635199
C	-4.443769	-2.872118	-0.879969
C	-3.140873	-2.080630	-2.896543
C	3.078605	0.142809	-0.507587
C	1.878655	-1.162664	-2.318684
C	4.449743	-0.224303	-0.322529
C	5.410047	0.814766	-0.090627
C	4.985964	2.165651	-0.052501
C	3.668103	2.487479	-0.236520
C	2.719342	1.470062	-0.464343
C	4.922331	-1.563653	-0.365391
C	6.251328	-1.857643	-0.185455
C	7.189862	-0.831160	0.047713
C	6.774045	0.473992	0.093923
H	-1.064222	0.689098	1.955268
H	-0.530473	-0.284544	-1.824806
H	1.212601	0.184594	1.720846
H	0.875918	-1.549671	1.688346
H	2.331994	-1.862770	-0.348920
H	-4.394069	3.300221	-0.426738
H	-6.086012	1.844989	-1.455770
H	-5.577455	-0.517303	-1.901636
H	-3.599531	-1.578492	3.356498
H	-3.708557	-1.013193	1.693272
H	-3.243685	0.104817	2.976052
H	-1.020997	-0.169567	4.198233
H	-1.476564	-1.840637	4.528120
H	0.077451	-1.493000	3.784197
H	-0.530871	-3.103459	1.850659
H	-2.056138	-2.899593	0.983526
H	-2.053747	-3.418808	2.661448
H	-2.416169	-2.218699	-0.895369
H	-0.975003	2.180513	0.577892
H	-0.706780	4.408543	-0.364455
H	-1.152809	3.272347	-1.644731
H	-2.352003	4.393592	-0.999022
H	-1.549915	4.075206	2.014278
H	-2.597320	2.708873	2.415784
H	-3.222838	4.051011	1.460422
H	-4.157876	-3.906314	-1.081945
H	-4.611534	-2.771291	0.193258
H	-5.393699	-2.690912	-1.385587
H	-2.887416	-3.115724	-3.135039
H	-2.338624	-1.440055	-3.269918
H	-4.049248	-1.823036	-3.444792
H	2.847043	-1.451926	-2.723647

H	1.172022	-1.967714	-2.528104
H	1.566560	-0.256583	-2.840539
H	5.723500	2.939752	0.122347
H	3.345959	3.520706	-0.209733
H	1.683541	1.753367	-0.606823
H	4.237237	-2.379925	-0.548250
H	6.582917	-2.887792	-0.224140
H	8.234629	-1.078025	0.187968
H	7.486370	1.271259	0.270967

B3LYP energy = -1315.78589325 a.u.

(5*S*,1'*S*)-**2a**, Conf. C

C	-0.838508	1.076709	1.172736
N	-1.230395	0.241151	-0.029373
C	-0.314583	0.375712	-0.972364
N	0.702314	1.134785	-0.658915
C	0.510811	1.688830	0.695425
C	-0.763649	0.374514	2.559100
C	-2.535688	-0.342553	-0.295001
C	1.861570	1.453072	-1.537902
C	-2.674137	-1.744327	-0.326839
C	-3.952016	-2.262333	-0.544326
C	-5.046498	-1.432742	-0.729207
C	-4.875655	-0.057467	-0.744540
C	-3.619888	0.519979	-0.551465
C	-2.145954	-0.160767	2.970326
C	-0.362080	1.468466	3.572677
C	0.268895	-0.760629	2.606477
C	-3.484106	2.031210	-0.706961
C	-1.506672	-2.714914	-0.195253
C	-1.727868	-3.761969	0.908881
C	-1.218460	-3.408394	-1.540002
C	-3.784982	2.456411	-2.156222
C	-4.364372	2.808254	0.285373
C	3.167041	1.184687	-0.797723
C	1.705482	2.862748	-2.112765
C	3.670514	-0.152868	-0.675552
C	4.881692	-0.366832	0.060533
C	5.554887	0.738029	0.637996
C	5.060122	2.005670	0.496400
C	3.866426	2.221791	-0.222698
C	3.041540	-1.289941	-1.248198
C	3.569024	-2.549958	-1.104096
C	4.756393	-2.752889	-0.372042
C	5.396456	-1.680961	0.193807
H	-1.585500	1.863652	1.263705
H	-0.415069	-0.095455	-1.940224
H	0.477067	2.776314	0.650058
H	1.347081	1.394701	1.323883
H	1.781553	0.755369	-2.371091
H	-4.090712	-3.335233	-0.570711
H	-6.030005	-1.859015	-0.883268
H	-5.729756	0.580559	-0.931742
H	-2.488722	-0.968962	2.327283
H	-2.900823	0.628283	2.952370
H	-2.096548	-0.546697	3.990159
H	-1.074062	2.297325	3.568464
H	-0.352967	1.044027	4.577568
H	0.634311	1.872836	3.383128
H	1.280328	-0.420735	2.376607
H	0.020026	-1.568697	1.920744
H	0.295684	-1.183435	3.612225
H	-2.449545	2.312759	-0.515776
H	-0.616967	-2.145625	0.069976
H	-0.835904	-4.382230	1.017362
H	-1.934870	-3.297324	1.873815
H	-2.561852	-4.424025	0.670616
H	-1.021407	-2.687872	-2.336712
H	-2.063456	-4.024121	-1.854510
H	-0.346307	-4.059204	-1.448383
H	-3.617131	3.528933	-2.275250
H	-3.143369	1.930234	-2.866082
H	-4.822053	2.248792	-2.425883
H	-4.200173	3.881747	0.172123
H	-4.144898	2.538451	1.319975
H	-5.424582	2.616303	0.111270
H	2.539504	3.080641	-2.779467
H	0.780862	2.924750	-2.687213
H	1.676507	3.632660	-1.342709
H	6.470421	0.561556	1.190043
H	5.578612	2.850451	0.931566
H	3.506615	3.237245	-0.315352
H	2.136048	-1.180189	-1.827355
H	3.070085	-3.395918	-1.560394
H	5.160417	-3.751608	-0.265441
H	6.313706	-1.822019	0.753190

B3LYP energy = -1315.78505447 a.u.

(5*S*,1'*S*)-**2a**, Conf. D

C	-0.756950	-0.763795	1.388461
N	-1.226235	-0.260526	0.040165
C	-0.303813	-0.513584	-0.871598
N	0.776581	-1.096300	-0.420275
C	0.669633	-1.277160	1.040478
C	-1.653546	-1.809981	2.112939
C	-2.343826	0.632997	-0.217833
C	1.918730	-1.645021	-1.192226
C	-3.439140	0.171905	-0.975939
C	-4.505783	1.051140	-1.169828
C	-4.493357	2.331389	-0.639426
C	-3.385118	2.776441	0.063317
C	-2.277039	1.953496	0.269610
C	-3.034688	-1.212836	2.432053
C	-0.955822	-2.122481	3.454503
C	-1.820668	-3.109129	1.311448
C	-1.052036	2.546043	0.959372
C	-3.498571	-1.202520	-1.633413
C	-4.777823	-1.977086	-1.276306
C	-3.351682	-1.086406	-3.162381
C	-0.497350	3.748557	0.174275

C	-1.343858	2.940337	2.417312
C	3.260298	-1.221016	-0.602464
C	1.783851	-1.385633	-2.692997
C	3.696565	0.140448	-0.498687
C	5.010125	0.397783	0.017146
C	5.840113	-0.683681	0.404096
C	5.397838	-1.973910	0.298080
C	4.105952	-2.232255	-0.202132
C	2.907452	1.262019	-0.869044
C	3.381896	2.545278	-0.748895
C	4.675850	2.789130	-0.246314
C	5.468198	1.735021	0.127424
H	-0.687239	0.100318	2.045407
H	-0.437297	-0.237482	-1.906387
H	1.454601	-0.707416	1.535024
H	0.805497	-2.329570	1.281093
H	1.854047	-2.726646	-1.048513
H	-5.361172	0.726076	-1.747258
H	-5.339910	2.989455	-0.791058
H	-3.369307	3.790401	0.441468
H	-3.605476	-0.979856	1.535444
H	-2.946678	-0.299020	3.023643
H	-3.613525	-1.929686	3.017349
H	-0.826240	-1.218709	4.054509
H	-1.569871	-2.817198	4.029418
H	0.023191	-2.586909	3.323635
H	-0.866666	-3.594857	1.096064
H	-2.336877	-2.942773	0.366924
H	-2.417266	-3.818314	1.887552
H	-0.258266	1.799765	0.969277
H	-2.656050	-1.790786	-1.272493
H	-4.733926	-2.982011	-1.700838
H	-4.906696	-2.071721	-0.197216
H	-5.668147	-1.490852	-1.678496
H	-4.180313	-0.521723	-3.594170
H	-3.348659	-2.078966	-3.617862
H	-2.426223	-0.580899	-3.447342
H	0.419599	4.108542	0.645099
H	-0.265374	3.479915	-0.858188
H	-1.207166	4.577180	0.153137
H	-2.092247	3.733539	2.467105
H	-0.435546	3.308661	2.898337
H	-1.719207	2.097087	2.999893
H	2.622165	-1.868422	-3.192983
H	0.866740	-1.824168	-3.089395
H	1.815012	-0.327543	-2.947490
H	6.831344	-0.469478	0.785651
H	6.030717	-2.800436	0.594798
H	3.775771	-3.261901	-0.278701
H	1.912496	1.118799	-1.260687
H	2.755757	3.378087	-1.043637
H	5.038455	3.805505	-0.158888
H	6.464926	1.908856	0.515357

B3LYP energy = -1315.78383032 a.u.

(5*S*,1’*S*)-**2a**, Conf. E

C	1.644345	-1.426574	1.026786
N	1.154316	-0.337892	0.097187
C	-0.085087	-0.600295	-0.275684
N	-0.603013	-1.686597	0.240046
C	0.432456	-2.402932	1.011250
C	2.108995	-0.997101	2.448257
C	1.969144	0.673889	-0.555485
C	-1.893657	-2.319610	-0.158140
C	2.915335	0.252308	-1.511216
C	3.738895	1.224409	-2.080431
C	3.601324	2.564794	-1.756028
C	2.615393	2.962067	-0.867113
C	1.775803	2.035175	-0.246356
C	3.320530	-0.053048	2.355941
C	2.576770	-2.281766	3.165233
C	0.989981	-0.336879	3.265537
C	0.678093	2.553226	0.676042
C	3.042540	-1.185741	-2.003522
C	2.716742	-1.282274	-3.505133
C	4.426902	-1.785577	-1.706432
C	1.225372	3.433315	1.812121
C	-0.395982	3.314586	-0.123458
C	-2.856126	-1.326826	-0.791604
C	-2.497550	-3.124488	0.998588
C	-3.435668	-0.219340	-0.087238
C	-4.407665	0.586355	-0.767185
C	-4.762630	0.283559	-2.105449
C	-4.181227	-0.767450	-2.760745
C	-3.229289	-1.566877	-2.096502
C	-3.099426	0.141173	1.245317
C	-3.698346	1.206599	1.871664
C	-4.666311	1.982884	1.202832
C	-5.007078	1.677795	-0.089173
H	2.500994	-1.895009	0.547156
H	-0.625686	0.029876	-0.966408
H	0.664038	-3.345181	0.511646
H	0.065407	-2.622970	2.009156
H	-1.628258	-3.039775	-0.936750
H	4.484952	0.928464	-2.806899
H	4.248875	3.302791	-2.212901
H	2.493224	4.015008	-0.649587
H	3.076991	0.897807	1.887142
H	4.136626	-0.508231	1.790640
H	3.692584	0.157333	3.360397
H	3.373058	-2.780687	2.607695
H	2.972574	-2.022448	4.148254
H	1.768545	-2.998632	3.320848
H	1.371294	-0.060057	4.249803
H	0.142629	-1.005926	3.428154
H	0.619666	0.571226	2.791687
H	0.186217	1.698814	1.138672
H	2.301576	-1.798224	-1.491131
H	2.736639	-2.326010	-3.825640
H	1.727262	-0.876686	-3.724279
H	3.443339	-0.735047	-4.108131

H	4.463740	-2.827651	-2.030765
H	4.664303	-1.752069	-0.641536
H	5.213535	-1.245374	-2.236316
H	0.412739	3.723713	2.481188
H	1.978860	2.911621	2.403720
H	1.677557	4.349219	1.428229
H	-1.204984	3.628802	0.538895
H	-0.827776	2.697682	-0.914079
H	0.021010	4.208504	-0.591725
H	-3.419526	-3.584710	0.644830
H	-1.829040	-3.926887	1.311982
H	-2.737454	-2.509773	1.863094
H	-5.502026	0.901571	-2.600896
H	-4.449344	-0.995934	-3.784236
H	-2.790797	-2.402982	-2.628620
H	-2.355572	-0.424398	1.786353
H	-3.424787	1.452584	2.890216
H	-5.133240	2.817626	1.710063
H	-5.744326	2.271318	-0.616601

B3LYP energy = -1315.78322334 a.u.

(5*S*,1'*S*)-**2a**, Conf. F

C	-1.540053	0.542437	1.611723
N	-1.373293	0.187848	0.148455
C	-0.159719	0.522872	-0.245203
N	0.618535	1.015266	0.685233
C	-0.122426	1.085989	1.963268
C	-2.068861	-0.554654	2.579006
C	-2.438785	-0.100539	-0.800243
C	2.020335	1.448590	0.446983
C	-2.529657	-1.387522	-1.366007
C	-3.592446	-1.634584	-2.237114
C	-4.521666	-0.652251	-2.539770
C	-4.381075	0.620200	-2.008578
C	-3.328609	0.935271	-1.148089
C	-3.490553	-0.986689	2.178955
C	-2.150858	0.105807	3.972510
C	-1.155555	-1.785887	2.661847
C	-3.167469	2.383641	-0.695976
C	-1.512440	-2.495479	-1.121547
C	-2.161638	-3.797552	-0.624794
C	-0.676145	-2.760925	-2.387548
C	-2.883640	3.303284	-1.897935
C	-4.379992	2.889476	0.102955
C	3.012414	0.289810	0.467391
C	2.400366	2.563459	1.426314
C	4.224239	0.384890	-0.292179
C	5.160060	-0.698979	-0.229002
C	4.871277	-1.829461	0.574608
C	3.704782	-1.895326	1.286734
C	2.779775	-0.832533	1.227171
C	4.554795	1.496055	-1.114068
C	5.728440	1.534056	-1.825986
C	6.645416	0.465190	-1.757479
C	6.362777	-0.624955	-0.976141
H	-2.260432	1.358116	1.650400
H	0.163533	0.416164	-1.271474
H	-0.155761	2.113408	2.317796
H	0.375592	0.480687	2.716743
H	2.005685	1.873167	-0.556748
H	-3.692509	-2.613541	-2.687369
H	-5.345156	-0.872508	-3.207656
H	-5.090249	1.389837	-2.284562
H	-3.516336	-1.507863	1.223975
H	-4.164296	-0.129864	2.112472
H	-3.890262	-1.662773	2.936827
H	-2.789423	0.992173	3.959007
H	-2.579426	-0.601630	4.683744
H	-1.170708	0.396442	4.355227
H	-0.161450	-1.544785	3.042762
H	-1.040457	-2.280264	1.698573
H	-1.589088	-2.511500	3.352055
H	-2.295296	2.454973	-0.047229
H	-0.823317	-2.161974	-0.347158
H	-1.388284	-4.528619	-0.380153
H	-2.766419	-3.634712	0.268420
H	-2.803616	-4.242571	-1.386726
H	-0.181306	-1.855842	-2.745225
H	-1.301105	-3.138351	-3.199465
H	0.092562	-3.508392	-2.180946
H	-2.700723	4.323466	-1.553810
H	-2.006164	2.969020	-2.454816
H	-3.728433	3.330810	-2.588152
H	-4.203110	3.909961	0.448684
H	-4.581329	2.265455	0.975683
H	-5.282387	2.900353	-0.510844
H	3.405486	2.911492	1.195279
H	1.719614	3.411791	1.344059
H	2.406894	2.204251	2.455832
H	5.588798	-2.640321	0.613777
H	3.482929	-2.759585	1.899868
H	1.866988	-0.921232	1.799322
H	3.883255	2.340248	-1.190281
H	5.952759	2.393779	-2.445056
H	7.567938	0.509433	-2.322481
H	7.059785	-1.452634	-0.917006

B3LYP energy = -1315.78252752 a.u.

(5*S*,1'*R*)-**2b**, Conf. A

C	-0.941578	0.534210	1.437657
N	-1.411797	0.181307	0.041162
C	-0.419665	0.354093	-0.815100
N	0.712790	0.731488	-0.281485
C	0.551274	0.884672	1.176976
C	-1.171267	-0.505111	2.571121
C	-2.791146	0.048110	-0.400578
C	2.001545	0.994962	-0.951486
C	-3.261226	-1.207239	-0.834131
C	-4.603153	-1.299696	-1.207597

C	-5.443890	-0.198966	-1.161385
C	-4.944531	1.036274	-0.779423
C	-3.607543	1.196337	-0.413245
C	-2.675845	-0.743192	2.787213
C	-0.607593	0.132449	3.859859
C	-0.462004	-1.842563	2.316725
C	-3.099678	2.603136	-0.112459
C	-2.378621	-2.441880	-0.974462
C	-2.929920	-3.655683	-0.208581
C	-2.167836	-2.793022	-2.459311
C	-3.223034	3.509307	-1.351265
C	-3.809929	3.232065	1.097737
C	3.048348	-0.050580	-0.575397
C	1.838956	1.129197	-2.466166
C	4.410647	0.332268	-0.360726
C	5.366501	-0.690318	-0.049685
C	4.947666	-2.041033	0.028505
C	3.639380	-2.379003	-0.192354
C	2.694657	-1.377628	-0.494923
C	4.879236	1.670955	-0.448070
C	6.199419	1.980318	-0.232709
C	7.132506	0.970882	0.082708
C	6.721017	-0.333457	0.170972
H	-1.480934	1.433877	1.730032
H	-0.549702	0.206708	-1.876397
H	0.797795	1.907254	1.461685
H	1.236322	0.209437	1.684930
H	2.307477	1.962727	-0.553925
H	-4.994989	-2.250668	-1.543822
H	-6.484244	-0.299123	-1.444759
H	-5.599523	1.898041	-0.786324
H	-3.148196	-1.230906	1.936740
H	-3.204454	0.193946	2.975384
H	-2.819788	-1.383889	3.659070
H	-1.088174	1.090283	4.073583
H	-0.799069	-0.530383	4.705002
H	0.470956	0.293723	3.812176
H	0.619702	-1.732353	2.222829
H	-0.831910	-2.336138	1.419485
H	-0.642851	-2.514111	3.157722
H	-2.036315	2.551377	0.120381
H	-1.399111	-2.212600	-0.555678
H	-2.230390	-4.491046	-0.280773
H	-3.081409	-3.433172	0.848505
H	-3.884052	-3.990164	-0.619457
H	-1.473434	-3.630449	-2.554794
H	-1.764288	-1.949534	-3.023489
H	-3.108431	-3.083069	-2.931567
H	-2.794258	4.491346	-1.140501
H	-2.696891	3.085040	-2.208610
H	-4.265622	3.656102	-1.638180
H	-3.383303	4.212686	1.318144
H	-3.715157	2.611656	1.990777
H	-4.875121	3.370096	0.903574
H	2.803250	1.398424	-2.894945
H	1.123473	1.911662	-2.725383
H	1.532959	0.187416	-2.923596
H	5.681755	-2.802542	0.262941
H	3.321650	-3.412364	-0.135964
H	1.666332	-1.673549	-0.663839
H	4.198447	2.473937	-0.694986
H	6.528429	3.009335	-0.306844
H	8.170096	1.230011	0.251353
H	7.429858	-1.117766	0.409277

B3LYP energy = -1315.78591338 a.u.

(5*S*,1'*R*)-**2b**, Conf. B

C	1.572797	-0.810988	1.575121
N	1.131816	-0.235013	0.246495
C	-0.049233	-0.729218	-0.080014
N	-0.570296	-1.546106	0.797624
C	0.359164	-1.720103	1.929655
C	1.978727	0.181839	2.702810
C	1.961629	0.476974	-0.711632
C	-1.889809	-2.235524	0.723879
C	2.991464	-0.228813	-1.364320
C	3.822450	0.487991	-2.226199
C	3.613530	1.836061	-2.471892
C	2.545199	2.489187	-1.877859
C	1.692130	1.831547	-0.989862
C	0.834490	1.116479	3.118713
C	3.203098	1.012357	2.280785
C	2.391238	-0.686507	3.911519
C	0.494815	2.590299	-0.429613
C	3.195455	-1.734885	-1.238200
C	2.929664	-2.434389	-2.584094
C	4.587688	-2.097252	-0.697165
C	0.893945	3.906526	0.257120
C	-0.548881	2.851763	-1.531995
C	-2.810691	-1.540224	-0.267845
C	-1.678219	-3.732504	0.485429
C	-3.494843	-0.335674	0.106089
C	-4.363203	0.286124	-0.848756
C	-4.524043	-0.295304	-2.130985
C	-3.858583	-1.443610	-2.463827
C	-3.005234	-2.062749	-1.526601
C	-3.359288	0.283088	1.376793
C	-4.042702	1.433349	1.687388
C	-4.899436	2.037444	0.744507
C	-5.054042	1.472374	-0.494902
H	2.445918	-1.430759	1.375301
H	-0.541809	-0.482302	-1.008767
H	0.643043	-2.766497	2.023302
H	-0.136909	-1.414623	2.848990
H	-2.315843	-2.124240	1.722711
H	4.632564	-0.022961	-2.730308
H	4.269620	2.372301	-3.146205
H	2.366715	3.530906	-2.110268
H	1.175439	1.775013	3.919483
H	-0.031902	0.573437	3.500988
H	0.502794	1.747366	2.295828

H	2.984380	1.689893	1.457734
H	3.539638	1.615757	3.125867
H	4.035290	0.372321	1.980529
H	3.201379	-1.372162	3.651960
H	1.561378	-1.273819	4.309797
H	2.747597	-0.042127	4.716341
H	0.014080	1.965829	0.321999
H	2.459517	-2.130282	-0.539168
H	3.009683	-3.517139	-2.465891
H	1.930426	-2.204505	-2.959530
H	3.651197	-2.127667	-3.343424
H	4.672502	-3.178205	-0.567946
H	4.782625	-1.623604	0.266567
H	5.375119	-1.784012	-1.385021
H	0.015626	4.370264	0.710660
H	1.634286	3.745665	1.042239
H	1.311159	4.620958	-0.454487
H	-1.421410	3.355727	-1.111813
H	-0.888001	1.924417	-1.998523
H	-0.138976	3.487934	-2.318923
H	-2.642221	-4.240164	0.469839
H	-1.088429	-4.167634	1.292168
H	-1.162714	-3.928095	-0.454487
H	-5.185615	0.184437	-2.842413
H	-3.985627	-1.889291	-3.442076
H	-2.505157	-2.976028	-1.817899
H	-2.714187	-0.151388	2.127979
H	-3.925113	1.879827	2.666989
H	-5.433485	2.942722	1.004080
H	-5.710868	1.926616	-1.227327

B3LYP energy = -1315.78581352 a.u.

(5*S*,1’*R*)-**2b**, Conf. C

C	0.766724	0.847664	1.293291
N	1.223266	0.220967	-0.008448
C	0.277791	0.370406	-0.918997
N	-0.783043	1.023270	-0.522194
C	-0.624653	1.419113	0.890503
C	1.706726	1.882546	1.975447
C	2.327648	-0.708493	-0.188776
C	-1.927426	1.429637	-1.384520
C	3.432094	-0.322999	-0.974876
C	4.486308	-1.230250	-1.095468
C	4.451656	-2.466248	-0.470066
C	3.334060	-2.840002	0.259189
C	2.239074	-1.986363	0.399054
C	0.977340	2.343110	3.256181
C	2.000119	3.106321	1.095365
C	3.027908	1.217776	2.398063
C	1.000472	-2.507596	1.121393
C	3.516445	0.995441	-1.735577
C	4.790752	1.788289	-1.400511
C	3.413489	0.763302	-3.254789
C	0.421202	-3.740716	0.404063
C	1.275157	-2.820543	2.602190
C	-3.243855	1.122337	-0.680656
C	-1.746636	2.876396	-1.850213
C	-3.747395	-0.220316	-0.642763
C	-4.978372	-0.473685	0.046265
C	-5.669333	0.598671	0.662836
C	-5.172992	1.872117	0.603055
C	-3.960883	2.127201	-0.071465
C	-3.099627	-1.324811	-1.257044
C	-3.628851	-2.590877	-1.195810
C	-4.836913	-2.832791	-0.510784
C	-5.494874	-1.792985	0.093341
H	0.634508	0.031931	2.001929
H	0.373906	-0.032513	-1.917533
H	-1.435787	1.001459	1.482498
H	-0.660878	2.503168	0.971234
H	-1.851324	0.795401	-2.267630
H	5.348761	-0.963995	-1.692310
H	5.287759	-3.147385	-0.568661
H	3.300654	-3.823529	0.709524
H	0.733559	1.498194	3.904626
H	0.053971	2.884128	3.040444
H	1.624443	3.017134	3.819165
H	1.094339	3.635765	0.793310
H	2.611507	3.816050	1.655289
H	2.552434	2.840238	0.195760
H	3.615510	0.879604	1.546566
H	2.851247	0.358782	3.049013
H	3.633335	1.934588	2.955987
H	0.224931	-1.743706	1.084357
H	2.666372	1.612449	-1.447007
H	4.768966	2.757524	-1.902880
H	4.888167	1.965167	-0.328418
H	5.688312	1.265570	-1.735516
H	3.422328	1.719417	-3.782359
H	2.496977	0.235189	-3.526347
H	4.254898	0.171708	-3.620710
H	-0.508553	-4.049502	0.885926
H	0.205666	-3.526804	-0.644426
H	1.111023	-4.585692	0.438769
H	0.356141	-3.144735	3.094687
H	1.658521	-1.950637	3.138750
H	2.008427	-3.622338	2.707269
H	-2.577084	3.161508	-2.495775
H	-0.822722	2.964965	-2.422239
H	-1.700058	3.583935	-1.023059
H	-6.599894	0.392556	1.178263
H	-5.705017	2.692350	1.067794
H	-3.603161	3.147169	-0.103089
H	-2.177345	-1.183645	-1.802398
H	-3.115386	-3.411301	-1.681570
H	-5.242389	-3.835783	-0.470056
H	-6.427552	-1.963969	0.617788

B3LYP energy = -1315.78532294 a.u.

(5*S*,1’*R*)-**2b**, Conf. D

C	0.765087	-0.886586	1.281716
N	1.239373	-0.249725	-0.008840
C	0.351097	-0.474637	-0.961684
N	-0.728564	-1.104206	-0.578221
C	-0.621549	-1.446287	0.852746
C	0.734122	-0.013817	2.568320
C	2.591505	0.190055	-0.314986
C	-1.862840	-1.578125	-1.410380
C	2.843010	1.559964	-0.527037
C	4.161765	1.942267	-0.780177
C	5.187319	1.011609	-0.828862
C	4.905960	-0.336030	-0.668007
C	3.605204	-0.782132	-0.431167
C	0.198178	-0.927051	3.692719
C	-0.172823	1.219071	2.445019
C	2.158357	0.420446	2.954878
C	3.348151	-2.285111	-0.379795
C	1.756979	2.629210	-0.548420
C	2.043427	3.781957	0.427791
C	1.549340	3.174069	-1.973896
C	3.697181	-2.947569	-1.725375
C	4.097748	-2.969141	0.775438
C	-3.210945	-1.268563	-0.766151
C	-1.736114	-1.136419	-2.869430
C	-3.695648	0.055713	-0.510065
C	-5.005093	0.205458	0.056577
C	-5.785421	-0.942378	0.342251
C	-5.298493	-2.195640	0.090022
C	-4.009716	-2.348565	-0.459347
C	-2.956969	1.239878	-0.772027
C	-3.473822	2.483533	-0.503390
C	-4.763392	2.622192	0.048288
C	-5.508360	1.504382	0.320368
H	1.446112	-1.711483	1.487042
H	0.522570	-0.166396	-1.981800
H	-0.692733	-2.527047	0.972309
H	-1.445041	-0.985827	1.393344
H	-1.770342	-2.667216	-1.398363
H	4.388078	2.987524	-0.945329
H	6.204068	1.334696	-1.014685
H	5.708164	-1.057859	-0.750748
H	0.805528	-1.829291	3.798636
H	-0.837603	-1.229951	3.529092
H	0.233013	-0.390300	4.641779
H	-1.202126	0.961421	2.189357
H	-0.201895	1.743581	3.401733
H	0.194110	1.922389	1.699545
H	2.603736	1.089834	2.221215
H	2.818439	-0.442150	3.069786
H	2.132490	0.945491	3.911387
H	2.282651	-2.453507	-0.225069
H	0.818780	2.168990	-0.241499
H	1.199690	4.474890	0.442876
H	2.203938	3.422904	1.445162
H	2.927606	4.348755	0.130802
H	0.736287	3.903119	-1.983232
H	1.301891	2.380898	-2.682351
H	2.449081	3.672773	-2.339492
H	3.428421	-4.005816	-1.702283
H	3.160723	-2.477065	-2.551892
H	4.765262	-2.880449	-1.938902
H	3.853713	-4.032997	0.805209
H	3.838075	-2.534303	1.742407
H	5.178792	-2.881073	0.652915
H	-2.576280	-1.555067	-3.421496
H	-0.820655	-1.523887	-3.320155
H	-1.766064	-0.054792	-2.993963
H	-6.773963	-0.808588	0.765103
H	-5.892879	-3.073257	0.309659
H	-3.642901	-3.350850	-0.648261
H	-1.967150	1.176449	-1.195616
H	-2.885853	3.367169	-0.718520
H	-5.159771	3.608556	0.253342
H	-6.501091	1.596714	0.744766

B3LYP energy = -1315.78362839 a.u.

(5*S*,1’*R*)-**2b**, Conf. E

C	1.704511	-1.161689	-1.280005
N	1.127534	-0.297290	-0.180921
C	-0.165420	-0.537956	-0.060608
N	-0.645715	-1.429522	-0.890801
C	0.442531	-1.944043	-1.745965
C	2.924593	-2.064229	-0.933546
C	1.759502	0.855044	0.441589
C	-1.974018	-2.099201	-0.805123
C	2.003854	1.998492	-0.344412
C	2.663578	3.069380	0.260073
C	3.024079	3.031896	1.597671
C	2.710809	1.922574	2.366973
C	2.073229	0.810371	1.814775
C	3.295593	-2.800705	-2.238917
C	2.618436	-3.097808	0.160454
C	4.132793	-1.208410	-0.515854
C	1.710464	-0.353478	2.729626
C	1.542535	2.151747	-1.790623
C	0.574011	3.338811	-1.941209
C	2.724438	2.286257	-2.766037
C	2.917543	-0.887848	3.517654
C	0.570817	0.036402	3.688992
C	-2.957177	-1.343974	0.075858
C	-2.512391	-2.437814	-2.200589
C	-3.460282	-0.034898	-0.225911
C	-4.449364	0.532127	0.644509
C	-4.903125	-0.201478	1.768837
C	-4.399057	-1.444054	2.040435
C	-3.425804	-2.006038	1.190390
C	-3.028396	0.750245	-1.328314
C	-3.547293	1.999607	-1.565501
C	-4.528791	2.544444	-0.713027
C	-4.965304	1.823342	0.367679
H	2.028930	-0.489550	-2.072174

H	-0.782971	-0.023102	0.660049
H	0.209986	-1.767496	-2.793762
H	0.538379	-3.017834	-1.591394
H	-1.775439	-3.050334	-0.303804
H	2.877785	3.956967	-0.321057
H	3.531382	3.876873	2.046390
H	2.965713	1.919788	3.418627
H	3.518684	-2.097204	-3.044602
H	2.504815	-3.471156	-2.580537
H	4.186393	-3.408112	-2.073166
H	1.807023	-3.774239	-0.115254
H	3.501570	-3.715197	0.332924
H	2.360775	-2.626500	1.107748
H	3.967255	-0.681579	0.421935
H	4.381005	-0.468202	-1.279401
H	5.004463	-1.852294	-0.384661
H	1.343332	-1.171982	2.111969
H	0.979468	1.263084	-2.075725
H	0.202043	3.387766	-2.966842
H	-0.283101	3.240197	-1.272753
H	1.065113	4.288097	-1.721491
H	2.360896	2.352161	-3.793590
H	3.404696	1.434871	-2.701747
H	3.304662	3.187691	-2.560594
H	2.623807	-1.767132	4.094686
H	3.737296	-1.174828	2.857495
H	3.299328	-0.147114	4.222284
H	0.284638	-0.821046	4.301759
H	-0.315528	0.378696	3.150850
H	0.879431	0.839989	4.360452
H	-3.461239	-2.959619	-2.080881
H	-1.834180	-3.106087	-2.731851
H	-2.686301	-1.556350	-2.813814
H	-5.655291	0.241242	2.410868
H	-4.743456	-2.003368	2.900769
H	-3.046551	-2.995319	1.417971
H	-2.272946	0.368771	-1.998694
H	-3.198902	2.572176	-2.416027
H	-4.931194	3.529299	-0.913324
H	-5.715862	2.232974	1.033191

B3LYP energy = -1315.78340168 a.u.

(5*S*,1'*R*)-**2b**, Conf. F

C	-1.538981	0.575789	-1.606106
N	-1.366750	0.192989	-0.151041
C	-0.127757	0.443149	0.224025
N	0.647767	0.950204	-0.701076
C	-0.105017	1.066495	-1.968361
C	-2.667035	1.589009	-1.959414
C	-2.285671	-0.592744	0.656627
C	2.058248	1.356542	-0.467779
C	-2.485345	-1.946387	0.320972
C	-3.427667	-2.663416	1.059334
C	-4.108621	-2.081309	2.116840
C	-3.837713	-0.770377	2.475541
C	-2.921986	0.005238	1.762424
C	-2.612896	1.776128	-3.491253
C	-2.486359	2.952107	-1.276632
C	-4.047344	1.007612	-1.607966
C	-2.620296	1.416187	2.252821
C	-1.681683	-2.681310	-0.746997
C	-0.826749	-3.796167	-0.116404
C	-2.567670	-3.244470	-1.870162
C	-3.885590	2.273492	2.418008
C	-1.819336	1.375399	3.567932
C	3.023386	0.175354	-0.517858
C	2.453928	2.480234	-1.430845
C	4.237905	0.226094	0.241517
C	5.148248	-0.877647	0.155507
C	4.832954	-1.983443	-0.671884
C	3.665269	-2.006470	-1.384831
C	2.764113	-0.924645	-1.302197
C	4.594675	1.312236	1.085692
C	5.768401	1.308005	1.798253
C	6.659724	0.219200	1.708062
C	6.351922	-0.847539	0.904250
H	-1.762195	-0.340501	-2.149352
H	0.223495	0.233772	1.224839
H	0.355973	0.453370	-2.740640
H	-0.098094	2.099430	-2.303730
H	2.062340	1.764840	0.542293
H	-3.614540	-3.701450	0.816397
H	-4.834637	-2.657401	2.676898
H	-4.347179	-0.339303	3.327258
H	-2.730262	0.823831	-4.013680
H	-1.681742	2.236600	-3.826513
H	-3.428064	2.429589	-3.805002
H	-1.541312	3.431219	-1.539797
H	-3.284229	3.625480	-1.594062
H	-2.537799	2.875750	-0.191802
H	-4.189835	0.885412	-0.535989
H	-4.203183	0.035946	-2.081532
H	-4.826679	1.680106	-1.971047
H	-1.994001	1.910098	1.511152
H	-0.981189	-1.980420	-1.199726
H	-0.201571	-4.264907	-0.879323
H	-0.172990	-3.404009	0.664729
H	-1.450120	-4.574671	0.327011
H	-1.949011	-3.714443	-2.637427
H	-3.165213	-2.465525	-2.347122
H	-3.255384	-4.002471	-1.490672
H	-3.609839	3.295429	2.685987
H	-4.470890	2.312544	1.498068
H	-4.531747	1.890552	3.209606
H	-1.555823	2.387943	3.880816
H	-0.896469	0.799894	3.465729
H	-2.403024	0.918082	4.369179
H	3.468579	2.801817	-1.202679
H	1.793517	3.342271	-1.326481
H	2.443334	2.140190	-2.466884
H	5.530875	-2.810267	-0.728783

H	3.423716	-2.852200	-2.016096
H	1.849749	-0.982668	-1.877007
H	3.943329	2.170268	1.178941
H	6.012821	2.149489	2.434553
H	7.582651	0.229870	2.274033
H	7.029190	-1.689990	0.827966

B3LYP energy = -1315.78265601 a.u.

(5*S*,1'*S*)-**3a**, Conf. A

C	0.452857	-1.106928	0.930008
C	-0.937953	-0.555281	1.355318
N	-1.547720	-0.219617	0.010627
C	-0.666782	-0.460359	-0.945149
N	0.472321	-0.956038	-0.537353
C	-1.733157	-1.489833	2.310852
C	-0.861463	-1.645259	3.576593
C	-2.008293	-2.877646	1.714462
C	-3.058015	-0.829385	2.729572
C	1.633291	-1.390151	-1.348021
C	-2.737216	0.581767	-0.230597
C	-3.868161	-0.020571	-0.816130
C	-5.002577	0.773466	-0.993380
C	-5.018307	2.106072	-0.612629
C	-3.875817	2.688466	-0.086527
C	-2.704308	1.952230	0.094326
C	-1.447807	2.682043	0.557694
C	-1.007531	3.721322	-0.489607
C	-1.619197	3.332837	1.939818
C	-3.893457	-1.460236	-1.314951
C	-5.082417	-2.261112	-0.760093
C	-3.884635	-1.507311	-2.854786
C	1.340964	-1.323765	-2.845859
C	2.872265	-0.602655	-0.952927
C	3.998549	-1.262712	-0.526040
C	5.184132	-0.561364	-0.189430
C	5.194809	0.863006	-0.290288
C	4.017043	1.521244	-0.728275
C	2.890507	0.812113	-1.052398
C	6.355979	-1.227508	0.248596
C	7.485285	-0.516790	0.570361
C	7.495683	0.892448	0.470521
C	6.376407	1.566872	0.050254
H	1.277992	-0.549159	1.368036
H	0.579291	-2.160162	1.176711
H	-0.809853	0.388164	1.884174
H	-0.880652	-0.245402	-1.981040
H	-0.636350	-0.675504	4.027078
H	0.082661	-2.155440	3.376067
H	-1.400206	-2.238375	4.316912
H	-1.094321	-3.398243	1.421734
H	-2.662501	-2.827309	0.845920
H	-2.504414	-3.500293	2.460926
H	-2.887596	0.153904	3.173751
H	-3.744981	-0.707499	1.894034
H	-3.553048	-1.449257	3.479301
H	1.798440	-2.435967	-1.077145
H	-5.888374	0.340446	-1.439231
H	-5.915419	2.697483	-0.747132
H	-3.886028	3.740552	0.167440
H	-0.632284	1.963806	0.633663
H	-0.064822	4.182470	-0.187658
H	-0.864578	3.259630	-1.468646
H	-1.747583	4.515836	-0.600184
H	-0.683340	3.798842	2.254886
H	-1.906207	2.602014	2.698165
H	-2.385643	4.109758	1.921350
H	-2.983949	-1.953046	-0.974460
H	-5.006255	-3.304565	-1.072730
H	-5.114115	-2.236312	0.330185
H	-6.034282	-1.877248	-1.130847
H	-3.851505	-2.543025	-3.199391
H	-3.022626	-0.983373	-3.273652
H	-4.783413	-1.044799	-3.267357
H	2.203462	-1.721571	-3.378443
H	0.473772	-1.929791	-3.112829
H	1.186928	-0.300593	-3.191516
H	3.997182	-2.344440	-0.443612
H	4.020840	2.602152	-0.806799
H	2.005969	1.342401	-1.385306
H	6.347763	-2.308695	0.324029
H	8.376219	-1.035003	0.902506
H	8.394385	1.439245	0.727368
H	6.383164	2.647825	-0.026893

B3LYP energy = -1315.79105788 a.u.

(5*S*,1'*S*)-**3a**, Conf. B

C	-0.285257	-1.461713	-1.087326
C	1.031962	-0.701172	-1.410622
N	1.453647	-0.239295	-0.030708
C	0.570446	-0.652332	0.862386
N	-0.418578	-1.356553	0.377633
C	2.057078	-1.518933	-2.248665
C	1.355160	-1.852108	-3.583672
C	2.490443	-2.823631	-1.565179
C	3.294150	-0.663367	-2.571658
C	-1.525755	-2.016362	1.102563
C	2.450830	0.772959	0.279069
C	3.619867	0.405297	0.974887
C	4.568621	1.401415	1.212702
C	4.371469	2.705060	0.786221
C	3.191533	3.051805	0.147085
C	2.195813	2.106036	-0.100271
C	0.876649	2.578714	-0.702681
C	0.172450	3.579292	0.231804
C	1.056721	3.179897	-2.106529
C	3.878447	-0.993395	1.522115
C	5.220652	-1.575973	1.049528
C	3.802120	-1.004846	3.060392
C	-1.315105	-1.990655	2.615350

C	-2.867648	-1.426337	0.687663
C	-3.121202	-0.078774	0.793076
C	-4.383198	0.463054	0.448631
C	-5.409631	-0.416973	-0.015667
C	-5.123244	-1.800386	-0.115215
C	-3.888172	-2.291154	0.224851
C	-4.664554	1.849759	0.553650
C	-5.899349	2.340867	0.213983
C	-6.913533	1.470471	-0.247637
C	-6.674467	0.124463	-0.359677
H	-1.155322	-1.014560	-1.565023
H	-0.242376	-2.513666	-1.363894
H	0.805371	0.194183	-1.986177
H	0.661726	-0.407659	1.909605
H	1.014287	-0.947478	-4.092895
H	0.497174	-2.514201	-3.453880
H	2.058906	-2.359395	-4.245191
H	1.647875	-3.479972	-1.338468
H	3.034206	-2.638396	-0.640320
H	3.156222	-3.377771	-2.229029
H	3.015377	0.261110	-3.082040
H	3.862982	-0.398487	-1.682625
H	3.957023	-1.220248	-3.236267
H	-1.500083	-3.057956	0.772666
H	5.478117	1.150703	1.742729
H	5.129426	3.456193	0.970653
H	3.030535	4.081072	-0.146419
H	0.207448	1.724075	-0.794228
H	-0.803575	3.849327	-0.176951
H	0.020647	3.156235	1.226793
H	0.751237	4.497693	0.344037
H	0.087341	3.459954	-2.523765
H	1.530434	2.475123	-2.792187
H	1.676489	4.077973	-2.075530
H	3.092958	-1.653586	1.156983
H	5.320984	-2.606768	1.395710
H	5.302989	-1.575442	-0.038138
H	6.065558	-1.012450	1.449055
H	3.934338	-2.021709	3.435991
H	2.842613	-0.631050	3.423264
H	4.584769	-0.382260	3.498424
H	-2.125282	-2.548386	3.083046
H	-0.373353	-2.464297	2.898639
H	-1.347335	-0.975551	3.013664
H	-2.352185	0.600104	1.146355
H	-5.896536	-2.472350	-0.468334
H	-3.688108	-3.353092	0.140631
H	-3.888993	2.517381	0.910888
H	-6.104087	3.400832	0.299223
H	-7.883305	1.873713	-0.511337
H	-7.451759	-0.543892	-0.711079

B3LYP energy = -1315.79001824 a.u.

(5*S*,1’*S*)-**3a**, Conf. C

C	-0.379609	-1.390813	0.661186
C	0.953011	-0.794018	1.203689
N	1.543991	-0.189505	-0.053282
C	0.707879	-0.368546	-1.060215
N	-0.396922	-0.999197	-0.761468
C	0.782768	0.137629	2.437899
C	2.152445	0.640778	2.924502
C	0.177754	-0.734734	3.558990
C	-0.146453	1.328348	2.165939
C	-1.518442	-1.274245	-1.696258
C	2.922572	0.225747	-0.254587
C	3.209080	1.584345	-0.493892
C	4.549875	1.945624	-0.636031
C	5.563998	1.004402	-0.553394
C	5.252174	-0.333871	-0.372346
C	3.929387	-0.759377	-0.242165
C	3.648971	-2.257012	-0.168703
C	4.065770	-2.953112	-1.477431
C	4.319304	-2.921127	1.044970
C	2.138170	2.653498	-0.669825
C	2.359409	3.878278	0.231696
C	2.038113	3.079958	-2.146588
C	-1.651872	-2.777102	-1.932677
C	-2.775755	-0.559386	-1.219130
C	-3.852417	-1.224163	-0.684859
C	-5.008409	-0.527271	-0.246841
C	-5.047891	0.894850	-0.364151
C	-3.925544	1.558870	-0.922347
C	-2.826993	0.854667	-1.336325
C	-6.127152	-1.201500	0.303411
C	-7.231570	-0.500534	0.719359
C	-7.269745	0.906870	0.603455
C	-6.203063	1.588953	0.072894
H	-0.409728	-2.475977	0.745423
H	-1.256174	-0.981330	1.157090
H	1.617785	-1.598890	1.510856
H	0.927858	-0.027479	-2.061971
H	2.640580	1.288497	2.199038
H	2.826248	-0.190861	3.142379
H	2.022848	1.212130	3.845489
H	0.806929	-1.602203	3.772458
H	-0.825700	-1.091396	3.319827
H	0.101907	-0.145907	4.474247
H	0.251918	1.992624	1.400377
H	-1.146880	1.018728	1.858443
H	-0.260099	1.915788	3.078640
H	-1.210975	-0.809870	-2.635442
H	4.803624	2.981783	-0.817133
H	6.597668	1.311450	-0.653100
H	6.049445	-1.065717	-0.353244
H	2.574420	-2.409190	-0.072753
H	3.799799	-4.011715	-1.440379
H	3.566646	-2.506143	-2.339682
H	5.142724	-2.885093	-1.640848
H	4.049912	-3.978204	1.092247
H	4.016206	-2.450716	1.982359
H	5.407192	-2.860306	0.981107

H	1.176977	2.222241	-0.393918
H	1.522745	4.572284	0.127966
H	2.436563	3.598023	1.283256
H	3.268028	4.419023	-0.038204
H	1.219745	3.790423	-2.281178
H	1.860116	2.226726	-2.805104
H	2.959442	3.562867	-2.478399
H	-2.429736	-2.962898	-2.672936
H	-0.712924	-3.178119	-2.315141
H	-1.911958	-3.322108	-1.025263
H	-3.848480	-2.302815	-0.592565
H	-3.952036	2.637301	-1.025352
H	-1.985846	1.384129	-1.769692
H	-6.098765	-2.281584	0.388950
H	-8.081410	-1.025255	1.137749
H	-8.148313	1.446200	0.934942
H	-6.232385	2.668438	-0.018650

B3LYP energy = -1315.78990121 a.u.

(5*S*,1’*S*)-**3a**, Conf. D

C	1.104516	-2.466901	-0.993159
C	2.204934	-1.403481	-0.709085
N	1.385795	-0.284245	-0.101838
C	0.114859	-0.643238	-0.051329
N	-0.138849	-1.843375	-0.503561
C	3.419745	-1.936947	0.104006
C	4.055143	-3.053018	-0.753303
C	3.020789	-2.509699	1.471473
C	4.472462	-0.830428	0.288973
C	-1.445874	-2.558448	-0.490107
C	1.812916	1.090703	0.097231
C	1.915374	1.599462	1.407432
C	2.379097	2.907537	1.557205
C	2.722111	3.682919	0.460950
C	2.561960	3.177708	-0.819556
C	2.082578	1.884885	-1.034805
C	1.815890	1.437571	-2.468432
C	0.736708	2.317262	-3.126152
C	3.094124	1.412945	-3.322955
C	1.491773	0.826987	2.651027
C	2.589344	0.779005	3.726413
C	0.189970	1.407636	3.235143
C	-1.496372	-3.549374	0.672697
C	-2.593161	-1.564730	-0.557460
C	-3.327754	-1.204535	0.547092
C	-4.385131	-0.262671	0.456147
C	-4.688027	0.319993	-0.811566
C	-3.919315	-0.070895	-1.937764
C	-2.905665	-0.983134	-1.815099
C	-5.151058	0.117286	1.586846
C	-6.168570	1.030601	1.467300
C	-6.468191	1.606301	0.212478
C	-5.745272	1.258835	-0.901492
H	1.002142	-2.691578	-2.054624
H	1.283424	-3.398239	-0.461110
H	2.601872	-1.028189	-1.649977
H	-0.658899	0.014510	0.317169
H	4.345564	-2.681980	-1.739324
H	3.389435	-3.906910	-0.892264
H	4.955363	-3.421662	-0.259477
H	2.294614	-3.321658	1.393738
H	2.606022	-1.745643	2.127157
H	3.903622	-2.918653	1.965849
H	4.784083	-0.414104	-0.671516
H	4.112650	-0.012447	0.909939
H	5.358052	-1.248420	0.770883
H	-1.447159	-3.125720	-1.424022
H	2.472795	3.326244	2.550605
H	3.093034	4.690173	0.604198
H	2.792986	3.806357	-1.669732
H	1.412334	0.425985	-2.449851
H	0.501302	1.937363	-4.122666
H	-0.183120	2.324574	-2.538311
H	1.073478	3.349574	-3.234763
H	2.870175	1.039663	-4.324421
H	3.862869	0.772598	-2.886294
H	3.518344	2.413152	-3.429603
H	1.281950	-0.201603	2.360146
H	2.268081	0.147394	4.557161
H	3.522880	0.372713	3.334371
H	2.800125	1.770153	4.131419
H	-0.138710	0.811927	4.089444
H	-0.616341	1.419880	2.498690
H	0.336807	2.433450	3.578891
H	-2.429028	-4.111822	0.634283
H	-0.674212	-4.262029	0.608896
H	-1.432461	-3.044657	1.636679
H	-3.120541	-1.639201	1.516877
H	-4.152230	0.356730	-2.905857
H	-2.338008	-1.276502	-2.690880
H	-4.922553	-0.325650	2.549290
H	-6.748505	1.313584	2.336884
H	-7.274816	2.324461	0.133770
H	-5.975466	1.698682	-1.864734

B3LYP energy = -1315.78970624 a.u.

(5*S*,1’*S*)-**3a**, Conf. E

C	1.107044	-2.457558	-1.012097
C	2.214363	-1.401524	-0.727235
N	1.398225	-0.266724	-0.148097
C	0.117937	-0.593603	-0.157589
N	-0.144778	-1.800907	-0.586270
C	3.415721	-1.940479	0.102064
C	4.049096	-3.066500	-0.743723
C	3.001658	-2.504779	1.468695
C	4.476061	-0.841824	0.290664
C	-1.486928	-2.414835	-0.798283
C	1.840015	1.102080	0.061798
C	1.924528	1.608108	1.374069
C	2.397546	2.911773	1.533566

C	2.765318	3.685643	0.444335
C	2.622796	3.183530	-0.839581
C	2.136287	1.895057	-1.064562
C	1.891087	1.450629	-2.502941
C	0.822079	2.332217	-3.174533
C	3.182392	1.425264	-3.337278
C	1.475906	0.837004	2.609632
C	2.563103	0.767281	3.694552
C	0.179085	1.435681	3.186338
C	-1.516135	-3.837944	-0.234068
C	-2.589347	-1.531069	-0.251170
C	-3.406352	-0.844617	-1.118028
C	-4.465704	-0.027450	-0.649528
C	-4.677712	0.085894	0.758184
C	-3.820683	-0.629212	1.633241
C	-2.810008	-1.416527	1.147102
C	-5.319515	0.679824	-1.532616
C	-6.335905	1.462042	-1.044178
C	-6.545758	1.574033	0.348538
C	-5.735625	0.902180	1.229640
H	1.040378	-2.717777	-2.068302
H	1.253837	-3.370327	-0.441102
H	2.627096	-1.040633	-1.667680
H	-0.652759	0.094958	0.154107
H	4.356261	-2.702031	-1.727111
H	3.374637	-3.912717	-0.887987
H	4.938657	-3.444200	-0.237541
H	2.283353	-3.323068	1.386480
H	2.572559	-1.739187	2.113123
H	3.880812	-2.903686	1.977613
H	4.796732	-0.430519	-0.669038
H	4.118994	-0.019324	0.907261
H	5.355311	-1.265607	0.779022
H	-1.608562	-2.473728	-1.883406
H	2.478295	3.328143	2.529135
H	3.142167	4.689580	0.595294
H	2.873744	3.811385	-1.684690
H	1.487134	0.439048	-2.493310
H	0.603238	1.956766	-4.176516
H	-0.107367	2.336027	-2.601604
H	1.159250	3.365449	-3.272676
H	2.973505	1.054661	-4.342977
H	3.941461	0.781343	-2.889054
H	3.611260	2.424357	-3.434939
H	1.251682	-0.186255	2.310712
H	2.225090	0.136247	4.519117
H	3.494496	0.349841	3.309371
H	2.784231	1.753370	4.106415
H	-0.162309	0.843393	4.038005
H	-0.623347	1.458669	2.446324
H	0.338537	2.458799	3.532621
H	-2.503416	-4.264056	-0.409670
H	-0.785530	-4.478955	-0.727654
H	-1.321843	-3.850787	0.838575
H	-3.253522	-0.929207	-2.188605
H	-3.981294	-0.552066	2.702111
H	-2.178225	-1.958341	1.840337
H	-5.159047	0.593067	-2.600930
H	-6.984003	1.997761	-1.726447
H	-7.352758	2.194192	0.718256
H	-5.896940	0.986909	2.297866

B3LYP energy = -1315.78961438 a.u.

(5*S*,1’*S*)-**3a**, Conf. F

C	-1.123193	2.469336	-0.947945
C	-2.238300	1.492084	-0.479679
N	-1.424012	0.262268	-0.146266
C	-0.137409	0.542311	-0.250658
N	0.134230	1.772227	-0.608197
C	-3.159684	2.059060	0.640040
C	-3.893832	3.272514	0.030189
C	-2.379578	2.508983	1.884223
C	-4.225878	1.023036	1.034749
C	1.479009	2.329733	-0.931820
C	-1.923332	-1.095611	-0.012161
C	-1.836168	-1.752457	1.231463
C	-2.375327	-3.037125	1.320773
C	-2.971333	-3.649291	0.229579
C	-2.997447	-3.002685	-0.995910
C	-2.455569	-1.726488	-1.154355
C	-2.403638	-1.127156	-2.556309
C	-1.528317	-1.986562	-3.487550
C	-3.803177	-0.923374	-3.159848
C	-1.137887	-1.167559	2.453807
C	-2.020945	-1.183025	3.712204
C	0.190872	-1.899345	2.723178
C	1.575792	3.784593	-0.466164
C	2.582470	1.455237	-0.373150
C	3.398810	0.754618	-1.228560
C	4.465645	-0.045364	-0.746492
C	4.684479	-0.125830	0.662370
C	3.826439	0.603011	1.525393
C	2.808850	1.372723	1.026498
C	5.319781	-0.767247	-1.617147
C	6.343328	-1.532038	-1.115881
C	6.559880	-1.611525	0.277928
C	5.749202	-0.925034	1.147278
H	-1.148183	2.651813	-2.023824
H	-1.172151	3.424960	-0.436279
H	-2.883735	1.234609	-1.316564
H	0.630344	-0.193847	-0.067052
H	-4.458148	2.988148	-0.861052
H	-3.216714	4.084698	-0.239523
H	-4.602185	3.670757	0.758104
H	-1.650065	3.289826	1.660265
H	-1.855464	1.680518	2.359876
H	-3.071908	2.921076	2.620277
H	-4.798560	0.691439	0.165895
H	-3.798398	0.143131	1.510320
H	-4.926647	1.473236	1.740149
H	1.550923	2.313214	-2.022849

H	-2.326286	-3.567646	2.262547
H	-3.394735	-4.641026	0.328755
H	-3.425340	-3.506996	-1.852735
H	-1.922493	-0.150990	-2.503283
H	-1.438475	-1.506228	-4.464286
H	-0.524384	-2.120456	-3.079916
H	-1.961274	-2.976317	-3.642159
H	-3.725345	-0.448792	-4.140189
H	-4.431533	-0.293845	-2.527001
H	-4.319153	-1.876092	-3.293525
H	-0.895438	-0.126396	2.244729
H	-1.502250	-0.686656	4.534872
H	-2.968055	-0.666575	3.550302
H	-2.245242	-2.201404	4.033772
H	0.709891	-1.439149	3.566544
H	0.857374	-1.868920	1.858789
H	0.016802	-2.948867	2.968597
H	2.568303	4.162537	-0.708984
H	0.847111	4.417113	-0.973600
H	1.428613	3.873928	0.610501
H	3.239872	0.813772	-2.299957
H	3.991627	0.549420	2.595033
H	2.174686	1.924789	1.709401
H	5.154226	-0.705454	-2.686429
H	6.991887	-2.078765	-1.788925
H	7.372266	-2.218286	0.657941
H	5.915469	-0.984886	2.216445

B3LYP energy = -1315.78941546 a.u.

(5*S*,1'*S*)-**3a**, Conf. G

C	-0.117685	-1.781871	0.651407
C	1.164620	-1.049385	1.145350
N	1.497175	-0.186184	-0.053724
C	0.605189	-0.396647	-1.005567
N	-0.335772	-1.253685	-0.708560
C	1.006355	-0.345229	2.523472
C	0.677402	-1.458903	3.541551
C	-0.117205	0.701397	2.539501
C	2.332850	0.304283	2.953355
C	-1.482750	-1.611830	-1.585290
C	2.754763	0.496376	-0.312196
C	2.780514	1.904338	-0.360567
C	4.015612	2.520514	-0.568583
C	5.175350	1.778794	-0.728757
C	5.115203	0.394166	-0.730661
C	3.906981	-0.279718	-0.547600
C	3.894430	-1.799523	-0.678206
C	4.323972	-2.232433	-2.091898
C	4.761436	-2.486214	0.390157
C	1.534777	2.776640	-0.264031
C	1.654980	3.871492	0.808330
C	1.200739	3.399163	-1.633074
C	-1.404648	-3.084691	-1.988514
C	-2.801411	-1.190781	-0.953241
C	-3.356471	0.019482	-1.299677
C	-4.577739	0.466639	-0.738446
C	-5.243477	-0.365035	0.213246
C	-4.656904	-1.609552	0.551470
C	-3.475545	-2.014124	-0.015365
C	-5.161776	1.709856	-1.091086
C	-6.345536	2.111403	-0.525533
C	-7.003220	1.288914	0.417293
C	-6.465908	0.078873	0.777422
H	0.010745	-2.862229	0.614721
H	-0.985946	-1.555838	1.265231
H	1.976483	-1.766674	1.252255
H	0.662715	0.096321	-1.966063
H	1.454048	-2.227185	3.555348
H	-0.280549	-1.943246	3.342105
H	0.619488	-1.028107	4.542102
H	-1.088586	0.279137	2.275378
H	0.085336	1.530875	1.863810
H	-0.211711	1.116250	3.544551
H	3.146109	-0.424730	2.966813
H	2.627444	1.124502	2.301420
H	2.230676	0.703963	3.963882
H	-1.340666	-1.004781	-2.479450
H	4.068559	3.600572	-0.609282
H	6.123618	2.280700	-0.875758
H	6.019948	-0.175184	-0.900384
H	2.871496	-2.152500	-0.551899
H	4.247208	-3.317260	-2.190777
H	3.692144	-1.775866	-2.855941
H	5.357632	-1.951374	-2.300111
H	4.677822	-3.571517	0.303745
H	4.462622	-2.201634	1.400684
H	5.814691	-2.225043	0.271908
H	0.693686	2.143066	0.013930
H	0.710757	4.413217	0.893464
H	1.894667	3.455137	1.787787
H	2.428282	4.598462	0.555009
H	0.277532	3.978878	-1.568733
H	1.070468	2.637265	-2.405052
H	1.995945	4.070166	-1.964008
H	-2.218398	-3.310759	-2.677478
H	-0.458817	-3.290257	-2.490693
H	-1.493834	-3.757475	-1.136012
H	-2.861119	0.655334	-2.026286
H	-5.161951	-2.249293	1.265600
H	-3.066786	-2.979355	0.254294
H	-4.659102	2.339225	-1.816188
H	-6.783471	3.062658	-0.801103
H	-7.937439	1.619370	0.853936
H	-6.970961	-0.554089	1.497675

B3LYP energy = -1315.78938679 a.u.

(5*S*,1'*S*)-**3a**, Conf. H

C	0.120044	-2.042480	0.659046
C	1.400807	-1.253566	1.057559
N	1.454086	-0.199769	-0.029447

C	0.501575	-0.441196	-0.913129
N	-0.282888	-1.448217	-0.629824
C	1.428994	-0.792806	2.542618
C	1.346674	-2.081800	3.390372
C	0.259915	0.129782	2.916835
C	2.764277	-0.102016	2.868226
C	-1.485813	-1.836822	-1.416469
C	2.588898	0.654441	-0.344667
C	2.482254	2.044112	-0.143327
C	3.609505	2.822339	-0.412319
C	4.788156	2.252733	-0.868121
C	4.849993	0.889160	-1.109536
C	3.750458	0.061782	-0.877346
C	3.844301	-1.408195	-1.274411
C	4.043656	-1.550358	-2.794626
C	4.945599	-2.154722	-0.504132
C	1.195150	2.735351	0.287722
C	1.393989	3.665290	1.495078
C	0.568692	3.505994	-0.889457
C	-1.504395	-3.347550	-1.645407
C	-2.760507	-1.277552	-0.803823
C	-3.342974	-0.166721	-1.368914
C	-4.534481	0.396493	-0.849400
C	-5.137024	-0.205562	0.296809
C	-4.522131	-1.350756	0.861510
C	-3.374192	-1.876522	0.326725
C	-5.149378	1.533980	-1.431260
C	-6.303574	2.052454	-0.900433
C	-6.898554	1.457887	0.235280
C	-6.329624	0.354309	0.819695
H	0.312676	-3.106042	0.537498
H	-0.683870	-1.911882	1.380722
H	2.276459	-1.885635	0.912768
H	0.391871	0.144309	-1.815410
H	2.153237	-2.775901	3.141833
H	0.395793	-2.603862	3.268227
H	1.442748	-1.826848	4.446785
H	-0.714111	-0.320688	2.717199
H	0.304846	1.081847	2.391584
H	0.298671	0.345164	3.986001
H	3.613787	-0.746344	2.630918
H	2.890675	0.835019	2.329151
H	2.806448	0.120970	3.935834
H	-1.345553	-1.348767	-2.381431
H	3.561976	3.893214	-0.264660
H	5.653423	2.876056	-1.056637
H	5.762178	0.461779	-1.505458
H	2.896770	-1.895398	-1.044727
H	4.040934	-2.605472	-3.076438
H	3.247731	-1.047503	-3.347817
H	4.995088	-1.122029	-3.114425
H	4.948922	-3.211074	-0.780352
H	4.800505	-2.086447	0.575647
H	5.933387	-1.749967	-0.731899
H	0.481105	1.967428	0.581920
H	0.430976	4.065576	1.818465
H	1.843128	3.141561	2.340308
H	2.035144	4.513757	1.249257
H	-0.381794	3.949539	-0.585603
H	0.380093	2.854176	-1.744948
H	1.223443	4.312329	-1.226319
H	-2.335094	-3.594042	-2.306034
H	-0.577865	-3.676149	-2.117776
H	-1.639884	-3.908145	-0.720560
H	-2.892966	0.294375	-2.241889
H	-4.980200	-1.819157	1.724687
H	-2.950513	-2.768007	0.771418
H	-4.695650	1.988302	-2.304445
H	-6.766207	2.921120	-1.352078
H	-7.810107	1.878079	0.641527
H	-6.788332	-0.104252	1.687866

B3LYP energy = -1315.78916452 a.u.

(5*S*,1’*R*)-**3b**, Conf. A

C	0.362392	1.390244	0.822783
C	-1.012728	0.827693	1.282487
N	-1.532247	0.225420	-0.007748
C	-0.676085	0.472655	-0.984489
N	0.408866	1.106258	-0.623580
C	-0.944004	-0.078371	2.544084
C	-0.357543	0.798610	3.671901
C	-0.053554	-1.314091	2.353513
C	-2.356565	-0.511472	2.972702
C	1.585286	1.465593	-1.447657
C	-2.895124	-0.204348	-0.279074
C	-3.910825	0.770732	-0.335335
C	-5.219419	0.331017	-0.538983
C	-5.507262	-1.012075	-0.724892
C	-4.481538	-1.943911	-0.735604
C	-3.154550	-1.568375	-0.518012
C	-2.070075	-2.636261	-0.601411
C	-2.326595	-3.813894	0.353070
C	-1.906536	-3.145420	-2.045716
C	-3.649298	2.271825	-0.258101
C	-4.020474	2.958900	-1.585030
C	-4.375954	2.935245	0.923200
C	1.296576	1.329435	-2.941478
C	2.808343	0.677043	-1.006699
C	3.943565	1.336923	-0.603674
C	5.115249	0.631313	-0.228956
C	5.101779	-0.796146	-0.265511
C	3.914550	-1.453695	-0.678131
C	2.802063	-0.740768	-1.039659
C	6.296663	1.296446	0.184234
C	7.412543	0.581824	0.542209
C	7.399301	-0.830424	0.505126
C	6.270064	-1.503942	0.110925
H	0.450944	2.464176	0.983140
H	1.201500	0.897123	1.309037
H	-1.680426	1.652012	1.530107

H	-0.878797	0.184157	-2.004662
H	-0.950423	1.703694	3.824304
H	0.675013	1.095100	3.478634
H	-0.363653	0.236232	4.606658
H	0.968226	-1.056386	2.068446
H	-0.453748	-1.993598	1.603491
H	0.006686	-1.865532	3.293531
H	-3.001740	0.353511	3.141863
H	-2.838271	-1.152050	2.236227
H	-2.298026	-1.068035	3.909728
H	1.767852	2.520024	-1.228884
H	-6.023342	1.054822	-0.575114
H	-6.530142	-1.330282	-0.883518
H	-4.714644	-2.984592	-0.919028
H	-1.122885	-2.182634	-0.312118
H	-1.482675	-4.506373	0.325503
H	-2.457179	-3.481314	1.383621
H	-3.219019	-4.373645	0.067673
H	-1.089901	-3.868461	-2.099258
H	-1.687647	-2.334097	-2.742742
H	-2.814961	-3.641335	-2.393390
H	-2.580833	2.435544	-0.119554
H	-3.754604	4.017415	-1.546255
H	-3.493889	2.505017	-2.426838
H	-5.091365	2.891285	-1.784133
H	-4.126057	3.997102	0.970323
H	-4.101310	2.479411	1.876164
H	-5.459403	2.855144	0.817977
H	2.158701	1.705379	-3.490417
H	0.426692	1.919220	-3.235167
H	1.147711	0.291361	-3.241675
H	3.960725	2.421246	-0.571293
H	3.899714	-2.536941	-0.706592
H	1.909505	-1.270857	-1.351327
H	6.306532	2.379896	0.211531
H	8.310977	1.099266	0.854797
H	8.287735	-1.380339	0.789552
H	6.258618	-2.587232	0.082015

B3LYP energy = -1315.79097302 a.u.

(5*S*,1'*R*)-**3b**, Conf. B

C	-0.185422	-1.685118	0.979979
C	1.070760	-0.846810	1.355941
N	1.462286	-0.281446	0.006313
C	0.618113	-0.709724	-0.915632
N	-0.344137	-1.475424	-0.471897
C	0.848967	0.152459	2.528080
C	-0.273269	1.163510	2.252995
C	2.153541	0.899171	2.857045
C	0.471665	-0.701302	3.758205
C	-1.463975	-2.069814	-1.228134
C	2.725408	0.348488	-0.340283
C	2.740484	1.707092	-0.713472
C	3.980140	2.285022	-0.992910
C	5.153725	1.552041	-0.912977
C	5.106413	0.203663	-0.595449
C	3.895712	-0.435806	-0.325930
C	3.902342	-1.944468	-0.100202
C	4.402102	-2.684673	-1.354591
C	4.723302	-2.348953	1.135156
C	1.483836	2.552893	-0.885385
C	1.530538	3.857818	-0.073997
C	1.226661	2.854292	-2.374144
C	-1.247217	-1.969898	-2.738223
C	-2.800310	-1.481602	-0.793872
C	-2.988416	-0.126977	-0.654052
C	-4.252279	0.408675	-0.302538
C	-5.348468	-0.484276	-0.094433
C	-5.127491	-1.875691	-0.244204
C	-3.889969	-2.360643	-0.580557
C	-4.466284	1.802767	-0.151407
C	-5.703862	2.288580	0.186851
C	-6.787648	1.404834	0.392890
C	-6.613791	0.050858	0.256164
H	-0.054300	-2.748824	1.177702
H	-1.083793	-1.346647	1.490699
H	1.877312	-1.510734	1.662428
H	0.734140	-0.453629	-1.957649
H	-1.234902	0.683176	2.063159
H	-0.042318	1.807473	1.405817
H	-0.404134	1.806022	3.125303
H	2.471391	1.557596	2.050912
H	2.970028	0.204891	3.068183
H	2.005503	1.512848	3.747388
H	-0.475808	-1.227548	3.629587
H	1.244460	-1.439442	3.985136
H	0.365629	-0.052882	4.629200
H	-1.460180	-3.127190	-0.952953
H	4.026388	3.327174	-1.280466
H	6.104420	2.027253	-1.120442
H	6.024789	-0.368921	-0.577328
H	2.878216	-2.278785	0.062334
H	4.328194	-3.764065	-1.205830
H	3.812297	-2.420794	-2.234676
H	5.445934	-2.449042	-1.568484
H	4.673142	-3.429570	1.283423
H	4.355987	-1.864653	2.041776
H	5.774471	-2.078745	1.018332
H	0.632492	1.977540	-0.523037
H	0.580759	4.388234	-0.167535
H	1.709400	3.670269	0.985627
H	2.315216	4.526062	-0.432268
H	0.295928	3.413753	-2.490025
H	1.149563	1.940952	-2.967982
H	2.032914	3.455501	-2.799009
H	-2.065810	-2.486459	-3.237260
H	-0.312887	-2.445781	-3.042132
H	-1.259104	-0.934346	-3.081883
H	-2.165705	0.563853	-0.807208
H	-5.953229	-2.558731	-0.083449
H	-3.739659	-3.429249	-0.684286

H	-3.636832	2.482105	-0.310042
H	-5.856424	3.355004	0.297235
H	-7.758451	1.803663	0.659438
H	-7.443939	-0.627702	0.413877

B3LYP energy = -1315.79007995 a.u.

(5*S*,1’*R*)-**3b**, Conf. C

C	0.462744	-1.109459	0.863629
C	-0.921549	-0.546111	1.301645
N	-1.536726	-0.185957	-0.035506
C	-0.666520	-0.436392	-0.997640
N	0.465630	-0.957119	-0.603307
C	-1.723347	-1.484227	2.247619
C	-0.849923	-1.667704	3.508395
C	-2.016120	-2.859194	1.630706
C	-3.038669	-0.814391	2.681628
C	1.588244	-1.366201	-1.484474
C	-2.714793	0.637657	-0.256403
C	-3.860909	0.061737	-0.839510
C	-4.985314	0.875177	-0.991026
C	-4.978318	2.200910	-0.587409
C	-3.821851	2.757626	-0.064140
C	-2.658879	2.002177	0.090874
C	-1.389032	2.708994	0.554488
C	-0.953139	3.770823	-0.471963
C	-1.537067	3.328072	1.954060
C	-3.919319	-1.369555	-1.360051
C	-5.091111	-2.168229	-0.765871
C	-3.982235	-1.390824	-2.899426
C	1.719455	-2.887306	-1.509139
C	2.844119	-0.593750	-1.101488
C	3.950855	-1.196117	-0.555094
C	5.108213	-0.448027	-0.215531
C	5.117278	0.960491	-0.446823
C	3.963627	1.559887	-1.014633
C	2.865059	0.807391	-1.332216
C	6.257736	-1.058252	0.345872
C	7.362940	-0.308793	0.664065
C	7.371020	1.085070	0.435043
C	6.273739	1.705600	-0.108657
H	1.295474	-0.555044	1.291706
H	0.579410	-2.160554	1.118151
H	-0.780822	0.388868	1.841671
H	-0.870905	-0.210883	-2.034872
H	-0.609968	-0.707017	3.970508
H	0.086445	-2.188604	3.299256
H	-1.394960	-2.262662	4.242631
H	-1.108448	-3.387599	1.332763
H	-2.666405	-2.785582	0.760835
H	-2.523266	-3.486156	2.366036
H	-2.854327	0.159702	3.140253
H	-3.727949	-0.670767	1.851583
H	-3.538024	-1.439666	3.423966
H	1.287618	-1.036674	-2.481363
H	-5.881551	0.462060	-1.434977
H	-5.868366	2.807059	-0.701740
H	-3.814015	3.804953	0.208878
H	-0.578520	1.982666	0.601383
H	-0.001910	4.214652	-0.170948
H	-0.827558	3.333550	-1.464494
H	-1.686559	4.575121	-0.551731
H	-0.593400	3.778908	2.267865
H	-1.821293	2.582843	2.699315
H	-2.297273	4.111282	1.963117
H	-3.000588	-1.876990	-1.068256
H	-5.046171	-3.204730	-1.106525
H	-5.069111	-2.169870	0.324780
H	-6.053751	-1.761283	-1.079761
H	-3.959142	-2.420295	-3.262961
H	-3.145703	-0.852378	-3.349949
H	-4.903052	-0.928731	-3.260890
H	2.498133	-3.177259	-2.214154
H	0.780514	-3.334934	-1.834585
H	1.976041	-3.300086	-0.533393
H	3.970001	-2.263512	-0.375882
H	3.966254	2.626854	-1.204359
H	1.999721	1.287325	-1.775381
H	6.252561	-2.128138	0.519356
H	8.236435	-0.784868	1.091817
H	8.250582	1.662985	0.690019
H	6.279908	2.774512	-0.287155

B3LYP energy = -1315.79002238 a.u.

(5*S*,1’*R*)-**3b**, Conf. D

C	1.110978	-2.643266	0.244388
C	2.221794	-1.554568	0.277703
N	1.408367	-0.295803	0.074296
C	0.131983	-0.609753	-0.059417
N	-0.137247	-1.888352	0.014038
C	3.151927	-1.628077	1.522628
C	3.837785	-3.010845	1.466799
C	2.392036	-1.493826	2.849946
C	4.250816	-0.554611	1.440958
C	-1.484740	-2.519569	0.112074
C	1.922572	1.019212	-0.272413
C	2.529884	1.194482	-1.531249
C	3.084806	2.443148	-1.814234
C	3.002605	3.489159	-0.908620
C	2.332387	3.313384	0.291876
C	1.772453	2.082840	0.639297
C	0.979838	1.977465	1.936278
C	1.774740	2.451924	3.163207
C	-0.349169	2.748448	1.822544
C	2.549201	0.123360	-2.617019
C	1.723767	0.570092	-3.838030
C	3.976579	-0.266560	-3.034900
C	-1.531037	-3.792942	-0.735799
C	-2.581903	-1.532405	-0.230959
C	-3.420321	-1.072066	0.756816
C	-4.480196	-0.174552	0.471412

C	-4.668646	0.263407	-0.874784
C	-3.788142	-0.221239	-1.875748
C	-2.778624	-1.094901	-1.567459
C	-5.356914	0.301445	1.478308
C	-6.372428	1.170435	1.166514
C	-6.558727	1.603753	-0.165211
C	-5.726696	1.160782	-1.162999
H	1.265905	-3.363287	-0.556083
H	1.030421	-3.185179	1.185580
H	2.861407	-1.661025	-0.596877
H	-0.629326	0.135870	-0.231684
H	4.383029	-3.148275	0.530019
H	3.130767	-3.835808	1.574173
H	4.556970	-3.092265	2.283110
H	1.620447	-2.256739	2.971735
H	1.923662	-0.516629	2.955447
H	3.089685	-1.611987	3.680784
H	4.818834	-0.637643	0.511840
H	3.851988	0.456201	1.502554
H	4.950396	-0.686182	2.268446
H	-1.602111	-2.801731	1.162029
H	3.570861	2.604750	-2.767726
H	3.440081	4.450160	-1.149137
H	2.238656	4.149458	0.972350
H	0.728898	0.929943	2.098359
H	1.192971	2.282003	4.071528
H	2.721211	1.918568	3.263420
H	1.996152	3.519276	3.111430
H	-0.942752	2.612651	2.729036
H	-0.945208	2.409054	0.972618
H	-0.172821	3.818262	1.694263
H	2.062762	-0.772289	-2.232462
H	1.681236	-0.234139	-4.575636
H	0.701285	0.827029	-3.553651
H	2.165922	1.442642	-4.322109
H	3.945658	-1.064360	-3.779849
H	4.567731	-0.616887	-2.186446
H	4.504692	0.579457	-3.478366
H	-2.522905	-4.235270	-0.649532
H	-0.806885	-4.530182	-0.388567
H	-1.338281	-3.583317	-1.788193
H	-3.283700	-1.402016	1.781146
H	-3.929601	0.104728	-2.899510
H	-2.128147	-1.455376	-2.354823
H	-5.215375	-0.033153	2.499357
H	-7.038232	1.527671	1.942144
H	-7.365194	2.288647	-0.395629
H	-5.870584	1.491509	-2.184872

B3LYP energy = -1315.78985693 a.u.

(5*S*,1’*R*)-**3b**, Conf. E

C	0.181786	1.583277	-0.931266
C	-1.163511	0.883480	-1.287356
N	-1.479984	0.169446	0.010512
C	-0.542140	0.440972	0.900302
N	0.403800	1.241886	0.486363
C	-2.248541	1.833890	-1.872055
C	-2.594811	3.000563	-0.936335
C	-3.523867	1.047211	-2.219918
C	-1.670069	2.394546	-3.189579
C	1.548064	1.712504	1.311779
C	-2.456437	-0.888634	0.206333
C	-2.247922	-2.125117	-0.436019
C	-3.233754	-3.102727	-0.293668
C	-4.356215	-2.885617	0.489776
C	-4.501663	-1.687745	1.171549
C	-3.561038	-0.663355	1.051831
C	-3.750087	0.602283	1.879651
C	-5.127514	1.251849	1.668396
C	-3.515717	0.317336	3.375530
C	-0.982691	-2.472865	-1.213828
C	-0.226568	-3.633558	-0.541000
C	-1.269002	-2.794803	-2.690101
C	1.478757	3.226445	1.516207
C	2.866212	1.204886	0.747312
C	3.429346	0.066318	1.275981
C	4.653259	-0.454458	0.788811
C	5.310713	0.221547	-0.283996
C	4.715003	1.394113	-0.810965
C	3.533102	1.875935	-0.309491
C	5.247575	-1.622142	1.331441
C	6.432836	-2.099662	0.831767
C	7.081629	-1.432107	-0.231833
C	6.534592	-0.297543	-0.776151
H	1.012840	1.213171	-1.529053
H	0.127339	2.663175	-1.043694
H	-0.987744	0.114665	-2.037168
H	-0.551232	0.020115	1.895846
H	-1.729897	3.627393	-0.710643
H	-3.021240	2.657665	0.005260
H	-3.336335	3.642305	-1.415192
H	-4.026828	0.654890	-1.338439
H	-3.305728	0.208904	-2.885041
H	-4.225805	1.704149	-2.736464
H	-0.783142	3.010983	-3.032177
H	-1.409302	1.592921	-3.884511
H	-2.418334	3.023221	-3.674343
H	1.397128	1.230992	2.278463
H	-3.108931	-4.057817	-0.787541
H	-5.107276	-3.659613	0.587392
H	-5.362611	-1.543787	1.811064
H	-3.000657	1.329152	1.569188
H	-5.182771	2.192498	2.219990
H	-5.317494	1.465996	0.615700
H	-5.933644	0.611663	2.030544
H	-3.595864	1.241448	3.951936
H	-2.529310	-0.113442	3.560969
H	-4.257654	-0.384016	3.762424
H	-0.310049	-1.616373	-1.187046
H	0.715836	-3.816844	-1.061304
H	-0.000673	-3.409646	0.503374

H	-0.806550	-4.557741	-0.567350
H	-0.334551	-2.987854	-3.220962
H	-1.782068	-1.974138	-3.195092
H	-1.894401	-3.684273	-2.786725
H	2.291573	3.536138	2.173013
H	0.532553	3.503523	1.982191
H	1.576965	3.779177	0.582131
H	2.937402	-0.452165	2.092405
H	5.213153	1.917638	-1.618452
H	3.118668	2.785052	-0.725708
H	4.752293	-2.133045	2.148954
H	6.878897	-2.992193	1.252492
H	8.016925	-1.821241	-0.614421
H	7.033876	0.217092	-1.588776

B3LYP energy = -1315.78953337 a.u.

(5*S*,1’*R*)-**3b**, Conf. F

C	-1.131666	2.670694	0.033735
C	-2.213921	1.563249	0.194777
N	-1.387868	0.310107	-0.007306
C	-0.131249	0.643174	-0.245868
N	0.114930	1.926839	-0.223361
C	-3.055772	1.679512	1.497957
C	-3.773789	3.045171	1.426890
C	-2.202738	1.624140	2.773462
C	-4.135119	0.583983	1.543021
C	1.429562	2.604447	-0.412043
C	-1.893552	-1.030651	-0.251638
C	-2.572745	-1.280950	-1.460590
C	-3.113651	-2.553181	-1.648838
C	-2.949420	-3.552357	-0.702215
C	-2.212734	-3.302891	0.444655
C	-1.664243	-2.044526	0.699431
C	-0.810968	-1.860913	1.949097
C	-1.550988	-2.261324	3.236106
C	0.512462	-2.640253	1.829053
C	-2.682708	-0.266008	-2.593852
C	-1.935604	-0.762022	-3.845668
C	-4.141324	0.085544	-2.929624
C	1.493558	3.256089	-1.793729
C	2.571599	1.661261	-0.076583
C	3.285031	0.986723	-1.038776
C	4.341342	0.105056	-0.692846
C	4.664660	-0.083213	0.685193
C	3.916871	0.626060	1.659461
C	2.903853	1.470372	1.290884
C	5.087061	-0.594756	-1.674626
C	6.103643	-1.441047	-1.308834
C	6.423143	-1.626744	0.054846
C	5.721028	-0.962497	1.029420
H	-1.346638	3.339775	-0.797647
H	-1.008594	3.269264	0.934625
H	-2.916695	1.615018	-0.635491
H	0.634689	-0.090187	-0.450524
H	-4.386207	3.128667	0.525861
H	-3.078960	3.887032	1.446168
H	-4.435191	3.151728	2.287913
H	-1.446167	2.410671	2.806566
H	-1.700280	0.665378	2.888460
H	-2.844472	1.763057	3.645123
H	-4.763723	0.608156	0.650192
H	-3.712479	-0.415177	1.629600
H	-4.781639	0.746758	2.407236
H	1.431670	3.398415	0.338975
H	-3.652492	-2.771214	-2.561827
H	-3.375854	-4.533564	-0.869908
H	-2.057224	-4.101998	1.157395
H	-0.553753	-0.805955	2.036484
H	-0.933158	-2.032068	4.106744
H	-2.497073	-1.729007	3.343313
H	-1.765316	-3.331276	3.259122
H	1.137582	-2.455410	2.705062
H	1.079448	-2.349274	0.942750
H	0.331510	-3.715194	1.767391
H	-2.184931	0.653584	-2.288038
H	-1.958892	0.003996	-4.623673
H	-0.891411	-0.988513	-3.620762
H	-2.393008	-1.664996	-4.254191
H	-4.174649	0.847614	-3.710999
H	-4.677008	0.468529	-2.059015
H	-4.685364	-0.787143	-3.295444
H	2.426119	3.811609	-1.891628
H	0.671578	3.959528	-1.927006
H	1.442062	2.517965	-2.594286
H	3.062180	1.120941	-2.089928
H	4.164953	0.498424	2.706517
H	2.351146	2.011878	2.050193
H	4.844449	-0.449818	-2.720890
H	6.668205	-1.970123	-2.066406
H	7.229078	-2.296535	0.327588
H	5.967335	-1.102004	2.075510

B3LYP energy = -1315.78943625 a.u.

(5*S*,1’*R*)-**3b**, Conf. G

C	1.068007	-2.649285	0.213352
C	2.161687	-1.559971	0.415284
N	1.410837	-0.308509	0.009090
C	0.164136	-0.621319	-0.298360
N	-0.132744	-1.888888	-0.178645
C	2.829009	-1.573220	1.821568
C	3.513013	-2.950072	1.966651
C	1.820319	-1.387386	2.964024
C	3.923752	-0.495769	1.909571
C	-1.440388	-2.540709	-0.471139
C	1.993094	0.984118	-0.313018
C	1.702553	2.100260	0.496562
C	2.323539	3.308692	0.175244
C	3.189991	3.412841	-0.901220
C	3.417789	2.313282	-1.713779
C	2.809165	1.084101	-1.457498

C	3.006949	-0.049713	-2.457955
C	2.443102	0.331250	-3.839318
C	4.478938	-0.479218	-2.569945
C	0.710438	2.074732	1.652922
C	1.313796	2.599643	2.966002
C	-0.563772	2.861282	1.293203
C	-1.438416	-3.107154	-1.891095
C	-2.589631	-1.606278	-0.131230
C	-3.363074	-0.999553	-1.091136
C	-4.430468	-0.132022	-0.741096
C	-4.701524	0.112349	0.638960
C	-3.893468	-0.530627	1.612045
C	-2.872230	-1.362533	1.240005
C	-5.235700	0.500299	-1.721197
C	-6.260409	1.335625	-1.351780
C	-6.528254	1.576825	0.014006
C	-5.767101	0.978286	0.987215
H	1.331276	-3.358296	-0.570809
H	0.857418	-3.206930	1.123439
H	2.958752	-1.702251	-0.312020
H	-0.552998	0.115579	-0.630012
H	4.230536	-3.127161	1.162085
H	2.799360	-3.775906	1.975364
H	4.058610	-2.984643	2.910763
H	1.046893	-2.157958	2.968397
H	1.330807	-0.415769	2.922680
H	2.339333	-1.450886	3.921839
H	4.666661	-0.615464	1.117680
H	3.522123	0.513362	1.843691
H	4.443964	-0.584313	2.865051
H	-1.481499	-3.377290	0.230456
H	2.122972	4.184116	0.779091
H	3.670376	4.358233	-1.120750
H	4.062096	2.416204	-2.577363
H	2.434542	-0.914222	-2.123072
H	2.519839	-0.517970	-4.521709
H	1.393065	0.623260	-3.775122
H	2.994912	1.163092	-4.280658
H	4.574280	-1.328553	-3.249517
H	4.891745	-0.769514	-1.602021
H	5.097309	0.330027	-2.962638
H	0.412110	1.041721	1.825482
H	0.596036	2.477770	3.779802
H	2.225233	2.064562	3.236254
H	1.556714	3.661491	2.899897
H	-1.294806	2.785850	2.100771
H	-1.028874	2.485897	0.379661
H	-0.341028	3.918993	1.139486
H	-2.360988	-3.658978	-2.070275
H	-0.605798	-3.797548	-2.027054
H	-1.351335	-2.319315	-2.640176
H	-3.181641	-1.178237	-2.143354
H	-4.102721	-0.361823	2.661866
H	-2.274556	-1.852618	2.000609
H	-5.031992	0.313319	-2.769211
H	-6.870429	1.813307	-2.108258
H	-7.340899	2.237082	0.290092
H	-5.973105	1.161029	2.035342

B3LYP energy = -1315.78919185 a.u.

(5*S*,1’*S*)-**4a**, Conf. A

C	-0.051687	-0.564797	1.573417
N	-0.328958	-0.193450	0.131461
C	0.798325	-0.245315	-0.557694
N	1.848936	-0.637871	0.115395
C	1.448906	-0.965040	1.498506
C	-0.964165	-1.625602	2.252459
C	-1.505096	0.491648	-0.381367
C	3.210115	-0.914583	-0.417657
C	-1.716876	1.834421	-0.011947
C	-2.883297	2.453813	-0.462438
C	-3.780113	1.789320	-1.284394
C	-3.510925	0.493517	-1.695902
C	-2.370833	-0.185603	-1.262348
C	-0.416838	-1.804840	3.685302
C	-0.952002	-2.984739	1.538638
C	-2.407201	-1.103094	2.360261
C	-2.095925	-1.580160	-1.811165
C	-0.711860	2.662451	0.782176
C	-0.127705	3.786991	-0.092494
C	-1.310266	3.228724	2.079814
C	-3.275628	-2.543427	-1.602203
C	-1.713840	-1.512961	-3.301942
C	4.093309	0.373736	-0.508152
C	3.107210	-1.740694	-1.701468
C	3.572417	1.369843	-1.555270
C	4.139260	1.066129	0.865563
C	5.525711	-0.054202	-0.882006
H	-0.155412	0.349809	2.156923
H	0.833797	0.027382	-1.601536
H	2.061175	-0.414431	2.208353
H	1.603154	-2.031045	1.661708
H	3.662484	-1.545899	0.349803
H	-3.080573	3.480649	-0.182556
H	-4.678239	2.290261	-1.623723
H	-4.196687	-0.001148	-2.371187
H	-0.386469	-0.854426	4.223150
H	0.585238	-2.237757	3.697331
H	-1.069000	-2.480210	4.240826
H	-1.565759	-3.692270	2.098717
H	-1.361674	-2.922804	0.532551
H	0.049239	-3.414477	1.472768
H	-2.442883	-0.146193	2.886250
H	-3.010951	-1.814150	2.926986
H	-2.876851	-0.970715	1.387280
H	-1.239885	-1.992663	-1.278814
H	0.128279	2.026959	1.060108
H	-0.899819	4.493904	-0.400819
H	0.631226	4.340966	0.464056
H	0.338200	3.383969	-0.994031

H	-0.543772	3.761055	2.646743
H	-1.717102	2.439804	2.715200
H	-2.116277	3.934376	1.871354
H	-2.999834	-3.547160	-1.932068
H	-3.571359	-2.600866	-0.553572
H	-4.150128	-2.237967	-2.179046
H	-1.464197	-2.509366	-3.672522
H	-0.854403	-0.861398	-3.473542
H	-2.541724	-1.129622	-3.901703
H	2.474577	-2.613284	-1.534494
H	4.091031	-2.096404	-1.999969
H	2.694830	-1.170763	-2.534751
H	4.236834	2.234807	-1.597925
H	3.538371	0.938853	-2.557144
H	2.578895	1.748984	-1.305224
H	4.816774	1.920709	0.827776
H	4.505527	0.389473	1.641625
H	3.160215	1.443188	1.168135
H	6.193537	0.806497	-0.818572
H	5.904717	-0.818995	-0.199367
H	5.589074	-0.441590	-1.899194

B3LYP energy = -1088.28161310 a.u.

(5*S*,1’*S*)-**4a**, Conf. B

C	-0.016122	-0.776079	1.344792
N	0.380526	-0.143846	0.029090
C	-0.657881	-0.146228	-0.787393
N	-1.767954	-0.625210	-0.289411
C	-1.518112	-1.102313	1.088620
C	0.251965	0.025926	2.650796
C	1.734559	0.088937	-0.447341
C	-3.037643	-0.766489	-1.052183
C	2.552993	-1.023848	-0.725000
C	3.869815	-0.777488	-1.115492
C	4.345360	0.515175	-1.270201
C	3.498906	1.592575	-1.061937
C	2.177418	1.410885	-0.650258
C	-0.226755	-0.871712	3.812645
C	-0.497979	1.364634	2.702401
C	1.760112	0.268207	2.835114
C	1.280705	2.634334	-0.503042
C	2.060033	-2.467272	-0.689595
C	2.109265	-3.095496	-2.094287
C	2.830760	-3.329896	0.323315
C	1.877229	3.699953	0.430372
C	0.958735	3.242733	-1.881202
C	-4.102720	0.335428	-0.729010
C	-3.524903	-2.212077	-0.942981
C	-3.457146	1.726604	-0.845315
C	-5.211892	0.229917	-1.796148
C	-4.741993	0.184193	0.661538
H	0.550628	-1.702124	1.428556
H	-0.585398	0.209459	-1.805295
H	-1.724816	-2.168608	1.152182
H	-2.171729	-0.584142	1.783225
H	-2.738627	-0.593859	-2.088414
H	4.526951	-1.612002	-1.323093
H	5.370612	0.682587	-1.576102
H	3.870353	2.595957	-1.223885
H	0.283566	-1.837778	3.806327
H	-1.302801	-1.054240	3.787664
H	-0.003030	-0.385076	4.763019
H	-1.580726	1.242766	2.639778
H	-0.288362	1.862334	3.650809
H	-0.187557	2.038169	1.904905
H	2.322359	-0.667331	2.798864
H	1.937944	0.722721	3.811417
H	2.169730	0.936299	2.080110
H	0.336161	2.313725	-0.065545
H	1.012266	-2.469887	-0.391571
H	3.134161	-3.177885	-2.460125
H	1.682278	-4.100309	-2.070393
H	1.543625	-2.501448	-2.814793
H	2.421026	-4.341860	0.343706
H	2.772188	-2.920227	1.333497
H	3.886823	-3.405963	0.058186
H	1.160328	4.510573	0.575925
H	2.124088	3.287224	1.409601
H	2.786009	4.137385	0.013345
H	0.263349	4.077559	-1.772216
H	0.507155	2.509884	-2.553858
H	1.862272	3.619528	-2.364648
H	-2.733026	-2.902200	-1.237092
H	-4.366938	-2.372609	-1.612801
H	-3.847792	-2.468721	0.065605
H	-4.217291	2.499122	-0.717875
H	-2.693639	1.894255	-0.083641
H	-3.002266	1.875593	-1.828414
H	-5.909773	1.060933	-1.681545
H	-5.786306	-0.692392	-1.704695
H	-4.800923	0.278906	-2.807708
H	-5.539881	0.920800	0.773869
H	-5.187765	-0.800745	0.806750
H	-4.031489	0.363403	1.469377

B3LYP energy = -1088.28143897 a.u.

(5*S*,1’*R*)-**4b**, Conf. A

C	0.052710	-0.563181	1.451124
N	-0.355833	-0.145739	0.054930
C	0.714480	-0.102692	-0.718011
N	1.841311	-0.437963	-0.143861
C	1.579913	-0.799886	1.266675
C	-0.701316	-1.755291	2.107454
C	-1.621284	0.450402	-0.343748
C	3.136372	-0.600896	-0.860909
C	-1.938090	1.740241	0.126623
C	-3.186194	2.264789	-0.211918
C	-4.066221	1.564622	-1.021979
C	-3.700301	0.329706	-1.533512

C	-2.474670	-0.256841	-1.213932
C	-0.569068	-3.063102	1.313707
C	-2.187818	-1.413412	2.306248
C	-0.071484	-1.946459	3.504712
C	-2.110268	-1.586808	-1.862627
C	-0.970734	2.616340	0.916472
C	-0.580634	3.865493	0.104344
C	-1.525356	3.014136	2.294069
C	-3.179105	-2.669182	-1.638450
C	-1.837566	-1.407581	-3.368189
C	4.092114	0.627144	-0.698565
C	3.739211	-1.962829	-0.511018
C	5.336535	0.373599	-1.573086
C	3.389317	1.891137	-1.222246
C	4.538776	0.863645	0.753639
H	-0.106889	0.299959	2.096095
H	0.658464	0.197746	-1.754543
H	2.173286	-0.181403	1.934088
H	1.855810	-1.839270	1.425598
H	2.855843	-0.625986	-1.916086
H	-3.462882	3.247499	0.147031
H	-5.029046	1.991771	-1.273431
H	-4.379350	-0.190602	-2.196049
H	-1.080909	-3.864792	1.848586
H	0.469363	-3.375762	1.188142
H	-1.020829	-2.990201	0.326111
H	-2.719577	-1.298035	1.363684
H	-2.672674	-2.215738	2.865377
H	-2.309824	-0.491585	2.878930
H	0.976816	-2.247040	3.454656
H	-0.608330	-2.731291	4.039527
H	-0.137631	-1.032957	4.100304
H	-1.186556	-1.944943	-1.410267
H	-0.049082	2.059780	1.081563
H	0.166617	4.447396	0.648069
H	-0.160504	3.595240	-0.866409
H	-1.442351	4.511449	-0.072599
H	-0.781469	3.592499	2.845846
H	-1.790228	2.141345	2.893515
H	-2.419255	3.633045	2.197694
H	-2.833170	-3.622917	-2.042142
H	-3.398225	-2.809970	-0.579071
H	-4.114318	-2.420634	-2.143010
H	-1.528532	-2.357299	-3.809970
H	-1.050077	-0.675036	-3.557135
H	-2.733749	-1.070166	-3.892585
H	4.565212	-2.184770	-1.182910
H	2.995355	-2.751458	-0.634254
H	4.119654	-2.004693	0.509822
H	5.943257	1.279572	-1.615391
H	5.966137	-0.422963	-1.175291
H	5.059870	0.112374	-2.597900
H	4.064011	2.746397	-1.157655
H	2.498622	2.138920	-0.642108
H	3.099091	1.780750	-2.270510
H	5.303317	1.642563	0.775776
H	3.717575	1.207992	1.384369
H	4.972888	-0.029439	1.205801

B3LYP energy = -1088.28142369 a.u.

(5*S*,1’*R*)-**4b**, Conf. B

C	0.088347	-0.382840	1.636942
N	0.335490	-0.116472	0.167363
C	-0.764599	-0.380100	-0.517458
N	-1.802243	-0.724059	0.199836
C	-1.415884	-0.782912	1.624798
C	0.451453	0.732031	2.658601
C	1.632719	-0.014344	-0.482764
C	-3.140297	-1.144589	-0.297313
C	2.436014	-1.169293	-0.561548
C	3.702935	-1.038098	-1.131751
C	4.139921	0.173999	-1.643117
C	3.301844	1.277665	-1.619351
C	2.030324	1.213482	-1.046581
C	1.963334	1.014362	2.627813
C	0.106360	0.164660	4.052901
C	-0.329508	2.035413	2.439214
C	1.128737	2.439582	-1.121614
C	1.968820	-2.554952	-0.126633
C	1.845883	-3.494934	-1.340634
C	2.872777	-3.168596	0.954715
C	1.788331	3.701985	-0.544054
C	0.669498	2.686516	-2.570890
C	-4.083029	0.059666	-0.627305
C	-2.971895	-2.177912	-1.414893
C	-5.468397	-0.506384	-0.995275
C	-4.240182	0.949206	0.618228
C	-3.561908	0.915575	-1.792644
H	0.696679	-1.247600	1.900013
H	-0.783505	-0.332418	-1.595926
H	-1.581731	-1.792165	2.000341
H	-2.033213	-0.097105	2.199648
H	-3.580285	-1.654847	0.561534
H	4.349541	-1.903860	-1.195613
H	5.126933	0.252606	-2.081732
H	3.639511	2.207695	-2.057742
H	2.220174	1.708179	3.430258
H	2.285325	1.460212	1.688967
H	2.541706	0.100686	2.781923
H	0.636363	-0.771078	4.247166
H	0.405206	0.880385	4.820169
H	-0.962947	-0.016654	4.177754
H	-0.135147	2.471671	1.460722
H	-0.030423	2.768765	3.189989
H	-1.407524	1.898557	2.541772
H	0.234328	2.240607	-0.532651
H	0.968697	-2.470914	0.297377
H	1.451112	-4.463486	-1.026393
H	1.173840	-3.082874	-2.096702
H	2.815212	-3.666763	-1.811750
H	2.482659	-4.140404	1.263929

H	2.934013	-2.530684	1.838607
H	3.888235	-3.322199	0.585397
H	1.076045	4.529557	-0.550283
H	2.121964	3.551781	0.483587
H	2.652916	4.009482	-1.134726
H	-0.023653	3.529455	-2.609195
H	0.164214	1.813263	-2.988568
H	1.517328	2.919287	-3.218188
H	-3.927287	-2.647689	-1.638800
H	-2.282585	-2.961888	-1.099263
H	-2.594548	-1.739508	-2.339098
H	-6.184873	0.311747	-1.084880
H	-5.457507	-1.037641	-1.947256
H	-5.839234	-1.188290	-0.225824
H	-4.953028	1.750509	0.415981
H	-3.299375	1.421689	0.905516
H	-4.617426	0.380131	1.471652
H	-4.284390	1.702626	-2.015926
H	-2.619625	1.413278	-1.552125
H	-3.426018	0.334481	-2.706382

B3LYP energy = -1088.28135287 a.u.

(5*R*,1’*R*)-**5a**, Conf. A

C	-1.933522	3.577182	0.820611
C	-1.302592	4.786423	0.537707
C	-1.283886	5.275734	-0.763784
C	-1.903550	4.551597	-1.779560
C	-2.534610	3.346026	-1.492676
C	-2.555195	2.840066	-0.188728
C	-3.259180	1.537213	0.116461
C	-2.567515	0.295428	-0.480052
N	-1.223701	0.087291	0.114586
C	-0.102649	0.023871	-0.557438
N	0.955596	-0.334765	0.144771
C	2.308704	-0.226145	-0.374421
C	3.090915	0.881851	0.005897
C	2.556936	2.059418	0.814753
C	3.298903	2.240508	2.149131
C	2.604633	3.355918	-0.014697
C	4.411234	0.927060	-0.443774
C	4.922984	-0.055804	-1.276015
C	4.109669	-1.095613	-1.698153
C	2.787807	-1.208658	-1.264222
C	1.932710	-2.343744	-1.815593
C	1.615305	-2.115586	-3.305572
C	2.575898	-3.725252	-1.611131
C	0.551674	-0.553155	1.588202
C	0.903244	-1.918561	2.246850
C	2.427706	-2.119433	2.303170
C	0.246437	-3.115295	1.543572
C	0.386103	-1.837043	3.699998
C	-0.974328	-0.248086	1.529466
C	-3.388556	-0.984848	-0.397524
C	-4.203117	-1.282388	0.697080
C	-4.923810	-2.472819	0.738828
C	-4.842968	-3.378307	-0.313889
C	-4.039655	-3.087544	-1.412668
C	-3.318232	-1.899683	-1.451313
H	-1.958430	3.215629	1.842647
H	-0.834070	5.348653	1.336141
H	-0.798424	6.217980	-0.985170
H	-1.905581	4.932050	-2.793684
H	-3.032553	2.803928	-2.289445
H	-3.375347	1.425040	1.195191
H	-4.268692	1.555252	-0.299956
H	-2.372643	0.496153	-1.533850
H	-0.043086	0.258176	-1.610792
H	1.505880	1.881558	1.040636
H	2.857088	3.061476	2.717601
H	3.256486	1.340288	2.764665
H	4.351376	2.479769	1.985965
H	2.143321	4.173539	0.542372
H	2.066553	3.244453	-0.957788
H	3.631348	3.645484	-0.245272
H	5.041726	1.758109	-0.155248
H	5.950040	0.000695	-1.614600
H	4.508735	-1.835886	-2.379061
H	0.983091	-2.348359	-1.281948
H	0.959816	-2.906298	-3.676629
H	1.120261	-1.156880	-3.474500
H	2.526315	-2.125611	-3.907072
H	1.890192	-4.507393	-1.943290
H	2.815588	-3.909326	-0.562990
H	3.496173	-3.828904	-2.188158
H	1.055568	0.214125	2.173626
H	2.922355	-1.281657	2.799017
H	2.653243	-3.021031	2.875521
H	2.871395	-2.235958	1.316320
H	0.601492	-3.232886	0.521239
H	0.493551	-4.031749	2.082265
H	-0.842646	-3.043997	1.517632
H	-0.700996	-1.752691	3.755365
H	0.822211	-0.987830	4.231662
H	0.667844	-2.744209	4.236265
H	-1.580264	-1.107278	1.806723
H	-1.248970	0.591555	2.165003
H	-4.294704	-0.587034	1.521597
H	-5.553433	-2.687733	1.593164
H	-5.408235	-4.301250	-0.282079
H	-3.979832	-3.781263	-2.241978
H	-2.701991	-1.678365	-2.315701

B3LYP energy = -1393.22259218 a.u.

(5*R*,1’*R*)-**5a**, Conf. B

C	2.551156	-3.336270	-1.480792
C	1.916677	-4.542548	-1.756999
C	1.297717	-5.257314	-0.734268
C	1.321180	-4.758409	0.563558
C	1.955926	-3.548753	0.835788

C	2.576140	-2.820417	-0.180898
C	3.280703	-1.514935	0.110984
C	2.578460	-0.276582	-0.481471
N	1.232457	-0.081531	0.112650
C	0.115662	-0.004001	-0.565262
N	-0.950120	0.326986	0.138939
C	-2.295499	0.250483	-0.405973
C	-3.091453	-0.868865	-0.094131
C	-2.579146	-2.089918	0.662311
C	-3.354757	-2.342565	1.965612
C	-2.605653	-3.338617	-0.238168
C	-4.404285	-0.880895	-0.567391
C	-4.895367	0.146922	-1.356797
C	-4.068015	1.200236	-1.713208
C	-2.752522	1.280008	-1.253526
C	-1.876312	2.428193	-1.739810
C	-1.524509	2.249111	-3.228948
C	-2.513638	3.807128	-1.502738
C	-0.561915	0.474398	1.596341
C	-0.981244	1.771056	2.346421
C	-2.513706	1.890690	2.421264
C	-0.394071	3.046781	1.725988
C	-0.449628	1.618991	3.788878
C	0.977068	0.249155	1.527380
C	3.392795	1.007161	-0.396605
C	4.161820	1.331932	0.723264
C	4.880865	2.523087	0.765069
C	4.844242	3.401898	-0.312638
C	4.085463	3.084605	-1.435352
C	3.364767	1.896050	-1.474120
H	3.048006	-2.800843	-2.282764
H	1.915250	-4.930758	-2.768170
H	0.809287	-6.199979	-0.947204
H	0.853417	-5.313554	1.367418
H	1.984873	-3.180050	1.855169
H	3.412038	-1.398289	1.187419
H	4.284525	-1.530758	-0.319010
H	2.385485	-0.476689	-1.535690
H	0.065500	-0.204864	-1.625899
H	-1.534597	-1.926611	0.924941
H	-2.927109	-3.194261	2.498451
H	-3.326141	-1.477291	2.629977
H	-4.403133	-2.570498	1.764687
H	-2.164881	-4.187784	0.287575
H	-2.037784	-3.177510	-1.156282
H	-3.625930	-3.609267	-0.515780
H	-5.045396	-1.720533	-0.331982
H	-5.917203	0.115703	-1.713966
H	-4.450077	1.976473	-2.363104
H	-0.940334	2.405266	-1.182869
H	-0.843091	3.038285	-3.553690
H	-1.046736	1.285863	-3.421417
H	-2.419639	2.301294	-3.851920
H	-1.809347	4.594091	-1.780263
H	-2.788746	3.952743	-0.457193
H	-3.411862	3.943768	-2.106991
H	-1.027474	-0.356380	2.125406
H	-2.962867	1.004162	2.873946
H	-2.777939	2.746992	3.044405
H	-2.969698	2.039756	1.444188
H	-0.758369	3.214097	0.714220
H	-0.689554	3.909064	2.326117
H	0.696864	3.037915	1.696394
H	0.640809	1.588113	3.833086
H	-0.836809	0.715052	4.265208
H	-0.773896	2.473105	4.385007
H	1.538879	1.142946	1.790379
H	1.299913	-0.567600	2.169044
H	4.217257	0.657963	1.568707
H	5.475168	2.759479	1.638816
H	5.409163	4.325047	-0.280830
H	4.059634	3.758051	-2.282905
H	2.782669	1.653318	-2.356309

B3LYP energy = -1393.22257293 a.u.

(5*R*,1'*R*)-**5a**, Conf. C

C	-4.351980	-2.482793	0.273196
C	-5.693704	-2.848259	0.296464
C	-6.589717	-2.270449	-0.599011
C	-6.134267	-1.329048	-1.516483
C	-4.791056	-0.964085	-1.536092
C	-3.885210	-1.533695	-0.640466
C	-2.425119	-1.138243	-0.647982
C	-2.081397	-0.137453	0.476449
N	-0.618422	0.018373	0.628797
C	0.332691	-0.332601	-0.197075
N	1.560364	-0.082547	0.224153
C	2.703810	-0.131269	-0.674140
C	3.146427	1.070152	-1.262172
C	2.417217	2.401732	-1.118358
C	3.308863	3.499953	-0.516830
C	1.831687	2.848405	-2.470675
C	4.278208	1.013949	-2.076513
C	4.920161	-0.186942	-2.335111
C	4.422645	-1.365425	-1.801675
C	3.305407	-1.371352	-0.964625
C	2.767366	-2.709043	-0.472028
C	2.132497	-3.499931	-1.631286
C	3.836693	-3.557989	0.234800
C	1.502442	0.595831	1.577395
C	2.312616	-0.038927	2.743616
C	3.817903	-0.011963	2.428331
C	1.871155	-1.471852	3.074909
C	2.073702	0.863540	3.973747
C	-0.032488	0.658571	1.822149
C	-2.742949	1.223619	0.315013
C	-2.368213	2.094052	-0.711396
C	-2.998512	3.324457	-0.857089
C	-4.013038	3.698966	0.020861
C	-4.390833	2.838317	1.045320
C	-3.755515	1.608138	1.192517

H	-3.660152	-2.950208	0.965884
H	-6.037671	-3.589196	1.007708
H	-7.634032	-2.556589	-0.584785
H	-6.823411	-0.878806	-2.220273
H	-4.446313	-0.230858	-2.255785
H	-1.814061	-2.034064	-0.509182
H	-2.169359	-0.707945	-1.618095
H	-2.421577	-0.568555	1.420763
H	0.142860	-0.781900	-1.160230
H	1.568663	2.266897	-0.448953
H	2.732473	4.416268	-0.374120
H	3.719911	3.202474	0.449595
H	4.147065	3.736096	-1.174844
H	1.249622	3.763615	-2.343702
H	1.176641	2.083133	-2.892357
H	2.619655	3.051773	-3.198134
H	4.649095	1.922567	-2.533000
H	5.795722	-0.207522	-2.972051
H	4.908587	-2.301588	-2.043155
H	1.978148	-2.512683	0.252239
H	1.702976	-4.433199	-1.261153
H	1.338943	-2.933361	-2.123222
H	2.876663	-3.750540	-2.389709
H	3.384432	-4.467315	0.635999
H	4.301381	-3.019243	1.061787
H	4.627869	-3.861670	-0.452700
H	1.893806	1.602499	1.441555
H	4.157338	1.001539	2.203037
H	4.377677	-0.363019	3.297099
H	4.080170	-0.649057	1.586082
H	2.044177	-2.156447	2.246126
H	2.445028	-1.840449	3.926823
H	0.816468	-1.531753	3.350867
H	1.032892	0.858513	4.302774
H	2.362349	1.897907	3.771863
H	2.679133	0.506729	4.808160
H	-0.339620	0.104553	2.707595
H	-0.403053	1.678880	1.906570
H	-1.581634	1.820861	-1.405997
H	-2.699919	3.990621	-1.656873
H	-4.504515	4.656846	-0.094261
H	-5.178260	3.122022	1.732213
H	-4.054703	0.941238	1.992628

B3LYP energy = -1393.22209504 a.u.

(5*R*,1'*R*)-**5a**, Conf. D

C	-4.382043	-2.483728	0.321316
C	-5.727367	-2.836551	0.330212
C	-6.601999	-2.276310	-0.597043
C	-6.121777	-1.364370	-1.531417
C	-4.775221	-1.011698	-1.536272
C	-3.890484	-1.564483	-0.609575
C	-2.426923	-1.181912	-0.606444
C	-2.081593	-0.175849	0.512708
N	-0.619088	-0.004118	0.647969
C	0.329132	-0.384521	-0.168086
N	1.557142	-0.089745	0.223230
C	2.688834	-0.175404	-0.686988
C	3.086483	0.986113	-1.378262
C	2.317802	2.303013	-1.335874
C	3.178678	3.468311	-0.821968
C	1.718624	2.628876	-2.716578
C	4.212595	0.897752	-2.197678
C	4.890215	-0.299993	-2.363544
C	4.434950	-1.445269	-1.729751
C	3.326392	-1.416060	-0.881782
C	2.837246	-2.723216	-0.270458
C	2.216350	-3.630161	-1.349703
C	3.942801	-3.473580	0.489650
C	1.498820	0.691894	1.520289
C	2.382569	0.213742	2.707509
C	3.874553	0.328793	2.349493
C	2.065818	-1.218635	3.159951
C	2.099643	1.187669	3.872330
C	-0.028466	0.682154	1.813040
C	-2.754043	1.179648	0.348042
C	-2.366359	2.060428	-0.664326
C	-3.007408	3.284387	-0.817650
C	-4.045798	3.641887	0.039077
C	-4.435704	2.771199	1.050562
C	-3.789538	1.547709	1.205623
H	-3.707593	-2.937472	1.039673
H	-6.090868	-3.554346	1.055333
H	-7.648930	-2.553067	-0.593972
H	-6.793991	-0.927563	-2.259645
H	-4.410875	-0.300916	-2.268689
H	-1.823948	-2.082141	-0.459734
H	-2.161579	-0.757997	-1.576964
H	-2.410495	-0.604619	1.462063
H	0.137350	-0.890223	-1.102303
H	1.473489	2.196639	-0.656126
H	2.578811	4.378058	-0.753682
H	3.592226	3.259054	0.166332
H	4.013562	3.672197	-1.494680
H	1.118396	3.539426	-2.659235
H	1.077494	1.819664	-3.072523
H	2.498901	2.789566	-3.462638
H	4.551031	1.776585	-2.731194
H	5.760898	-0.345754	-3.005857
H	4.948493	-2.382674	-1.898451
H	2.050081	-2.489898	0.445343
H	1.825341	-4.543656	-0.896597
H	1.396927	-3.134858	-1.875267
H	2.959715	-3.917308	-2.095982
H	3.522864	-4.348311	0.990383
H	4.412937	-2.844040	1.246387
H	4.725073	-3.826559	-0.184639
H	1.817430	1.707028	1.285457
H	4.132489	1.343134	2.037118
H	4.478698	0.090513	3.226755
H	4.165511	-0.352614	1.552277

H	2.292788	-1.950156	2.386661
H	2.674003	-1.465810	4.031770
H	1.022138	-1.346728	3.453440
H	1.072077	1.120957	4.234912
H	2.293575	2.223254	3.583388
H	2.755069	0.948858	4.710999
H	-0.280378	0.123375	2.713412
H	-0.444794	1.683964	1.898267
H	-1.558146	1.800706	-1.338700
H	-2.698071	3.958901	-1.606304
H	-4.545733	4.594696	-0.081604
H	-5.241014	3.042232	1.721657
H	-4.098035	0.873930	1.996342

B3LYP energy = -1393.22208944 a.u.

(5*R*,1’*R*)-**5a**, Conf. E

C	2.643852	3.425098	0.354461
C	2.173815	4.719444	0.545950
C	1.325704	5.303465	-0.391944
C	0.955050	4.583456	-1.521671
C	1.424086	3.285268	-1.707685
C	2.270594	2.686363	-0.773265
C	2.820606	1.295637	-1.017410
C	2.669116	0.297003	0.144046
N	1.260677	-0.012396	0.469549
C	0.195950	0.075550	-0.284682
N	-0.924954	-0.345812	0.278359
C	-2.123177	-0.629473	-0.498037
C	-2.328798	-1.947549	-0.953610
C	-1.299480	-3.065174	-0.817975
C	-1.846979	-4.281234	-0.053338
C	-0.765997	-3.480954	-2.201460
C	-3.519508	-2.221175	-1.627858
C	-4.450971	-1.225983	-1.878845
C	-4.191954	0.076907	-1.484773
C	-3.025597	0.410726	-0.793823
C	-2.768647	1.875494	-0.464458
C	-2.523956	2.683054	-1.752247
C	-3.904251	2.510318	0.354965
C	-0.623051	-0.882906	1.663000
C	-1.460300	-0.341632	2.856864
C	-2.938170	-0.740681	2.701348
C	-1.343710	1.178664	3.033906
C	-0.914596	-1.043459	4.119663
C	0.901099	-0.601877	1.772459
C	3.456443	-0.985135	-0.095938
C	3.059296	-1.911765	-1.061701
C	3.814783	-3.055744	-1.293583
C	4.977744	-3.285977	-0.562770
C	5.380564	-2.366899	0.400007
C	4.621246	-1.223422	0.633002
H	3.321671	2.999254	1.085306
H	2.478370	5.276808	1.423292
H	0.964518	6.313669	-0.245374
H	0.303725	5.030328	-2.262370
H	1.138736	2.740499	-2.600992
H	2.393664	0.875345	-1.930757
H	3.895377	1.358968	-1.204696
H	3.065778	0.762526	1.047476
H	0.218755	0.447463	-1.297607
H	-0.441425	-2.691162	-0.260521
H	-2.672165	-4.752464	-0.590474
H	-1.062974	-5.030887	0.071384
H	-2.212222	-4.004363	0.937225
H	0.013175	-4.238131	-2.091134
H	-0.339645	-2.628651	-2.735163
H	-1.556926	-3.903319	-2.823746
H	-3.710857	-3.226487	-1.980005
H	-5.368581	-1.461725	-2.403415
H	-4.907686	0.853520	-1.721595
H	-1.858558	1.937710	0.130865
H	-2.268567	3.715109	-1.505459
H	-1.706292	2.264602	-2.342233
H	-3.415003	2.698430	-2.382987
H	-3.639713	3.534854	0.624653
H	-4.101229	1.959815	1.275761
H	-4.834002	2.550410	-0.215357
H	-0.796614	-1.957533	1.624557
H	-3.048193	-1.821673	2.589174
H	-3.490835	-0.443622	3.594499
H	-3.411560	-0.264135	1.845339
H	-1.732832	1.721257	2.174067
H	-1.922494	1.487547	3.906056
H	-0.315208	1.503711	3.201946
H	0.119184	-0.769292	4.338033
H	-0.967220	-2.130913	4.027649
H	-1.515740	-0.753916	4.982747
H	1.140491	0.112036	2.558969
H	1.483858	-1.507005	1.937201
H	2.159402	-1.748942	-1.642957
H	3.497401	-3.766772	-2.046052
H	5.565047	-4.177410	-0.743510
H	6.282534	-2.539398	0.973647
H	4.939957	-0.511049	1.385614

B3LYP energy = -1393.22128141 a.u.

(5*R*,1’*R*)-**5a**, Conf. F

C	-5.359111	-1.113473	-0.689742
C	-6.569976	-1.509405	-0.127957
C	-6.586306	-2.386599	0.951277
C	-5.384647	-2.869604	1.463002
C	-4.177329	-2.473080	0.898345
C	-4.148042	-1.587373	-0.183283
C	-2.824347	-1.165201	-0.783977
C	-2.073979	-0.138083	0.092223
N	-0.653328	-0.046500	-0.334867
C	0.379717	-0.277858	0.432661
N	1.557730	-0.065232	-0.126531
C	2.785966	-0.571919	0.466525
C	3.264632	-1.830046	0.050244

C	2.504703	-2.759928	-0.891519
C	3.299995	-3.080004	-2.167788
C	2.090384	-4.053736	-0.166321
C	4.477135	-2.268500	0.584180
C	5.164183	-1.516497	1.524267
C	4.635462	-0.315662	1.970610
C	3.436325	0.185363	1.460328
C	2.875250	1.478497	2.038624
C	2.378588	1.265252	3.480951
C	3.882520	2.638663	1.983810
C	1.357585	0.342724	-1.571571
C	2.088880	1.614962	-2.086643
C	3.613479	1.415340	-2.038299
C	1.704932	2.888418	-1.319733
C	1.680568	1.772333	-3.568014
C	-0.194650	0.420023	-1.657298
C	-2.699081	1.246537	0.174145
C	-3.216370	1.904129	-0.944229
C	-3.747201	3.185390	-0.827976
C	-3.772428	3.824153	0.407736
C	-3.263333	3.175788	1.528727
C	-2.729840	1.897103	1.409275
H	-5.359870	-0.433562	-1.533190
H	-7.499917	-1.133311	-0.536449
H	-7.527665	-2.696608	1.387629
H	-5.388036	-3.560742	2.296999
H	-3.248842	-2.867929	1.297132
H	-2.178576	-2.040560	-0.888583
H	-2.983092	-0.767596	-1.786834
H	-2.028644	-0.540054	1.104727
H	0.282869	-0.636748	1.447778
H	1.578354	-2.273291	-1.196052
H	2.702343	-3.701116	-2.838070
H	3.583453	-2.173957	-2.706251
H	4.215381	-3.628287	-1.937348
H	1.490409	-4.679823	-0.830174
H	1.498254	-3.837827	0.725305
H	2.960433	-4.635046	0.143581
H	4.879469	-3.224482	0.274792
H	6.102596	-1.877832	1.926209
H	5.163079	0.244061	2.731845
H	2.010900	1.768876	1.443073
H	1.935330	2.186116	3.866009
H	1.625055	0.477193	3.539406
H	3.200049	0.985962	4.143657
H	3.404575	3.562415	2.316143
H	4.261367	2.799413	0.973498
H	4.738104	2.459154	2.637152
H	1.716032	-0.487223	-2.179329
H	3.915457	0.526810	-2.597149
H	4.107635	2.275627	-2.493204
H	3.989321	1.316897	-1.021640
H	2.005542	2.842954	-0.274679
H	2.210996	3.745450	-1.767381
H	0.633982	3.096356	-1.355832
H	0.615012	1.976196	-3.689468
H	1.926026	0.878892	-4.147174
H	2.223023	2.611336	-4.006056
H	-0.548781	1.435774	-1.819174
H	-0.597797	-0.219209	-2.440200
H	-3.220988	1.424012	-1.914641
H	-4.146625	3.681331	-1.703795
H	-4.191093	4.818641	0.497243
H	-3.286803	3.661553	2.496123
H	-2.341117	1.396068	2.288689

B3LYP energy = -1393.22126745 a.u.

(5*R*,1’*R*)-**5a**, Conf. G

C	5.328522	-1.102816	0.710291
C	6.545227	-1.498222	0.161094
C	6.573420	-2.385977	-0.909328
C	5.377833	-2.879888	-1.424768
C	4.164466	-2.483754	-0.872701
C	4.123398	-1.587892	0.199997
C	2.794436	-1.161381	0.785138
C	2.060647	-0.129973	-0.101424
N	0.641068	-0.012680	0.323043
C	-0.392634	-0.254274	-0.440667
N	-1.570867	-0.044407	0.119063
C	-2.795904	-0.564610	-0.468993
C	-3.258145	-1.828583	-0.051573
C	-2.482682	-2.750020	0.885794
C	-3.268067	-3.082630	2.164958
C	-2.053808	-4.036908	0.156827
C	-4.467520	-2.280730	-0.581040
C	-5.167103	-1.535750	-1.517540
C	-4.654271	-0.328441	-1.965022
C	-3.458756	0.185904	-1.459389
C	-2.914190	1.485487	-2.039225
C	-2.417085	1.277293	-3.482166
C	-3.935407	2.633270	-1.983302
C	-1.370082	0.364469	1.563411
C	-2.116421	1.625036	2.085176
C	-3.638236	1.402879	2.046162
C	-1.756709	2.905415	1.318087
C	-1.700421	1.785833	3.564004
C	0.181085	0.463685	1.641907
C	2.722160	1.236259	-0.189268
C	3.162474	1.925405	0.943702
C	3.740090	3.185413	0.824799
C	3.894298	3.769889	-0.428785
C	3.470550	3.086509	-1.563876
C	2.887168	1.829448	-1.442058
H	5.319320	-0.413818	1.546367
H	7.470413	-1.113259	0.572126
H	7.519354	-2.695405	-1.336122
H	5.390581	-3.578876	-2.252105
H	3.240724	-2.886204	-1.274754
H	2.143707	-2.033371	0.886303
H	2.945260	-0.758427	1.787191
H	2.008205	-0.538523	-1.110803

H	-0.296263	-0.620077	-1.453417
H	-1.561895	-2.251626	1.187881
H	-2.658527	-3.695402	2.832216
H	-3.562389	-2.181164	2.705312
H	-4.176265	-3.644135	1.938049
H	-1.441810	-4.655425	0.816807
H	-1.469382	-3.811454	-0.737461
H	-2.917241	-4.629896	-0.149497
H	-4.857483	-3.241832	-0.271493
H	-6.102877	-1.907690	-1.915929
H	-5.191662	0.225566	-2.723595
H	-2.052358	1.786681	-1.445270
H	-1.988393	2.204056	-3.869531
H	-1.651724	0.500675	-3.540658
H	-3.235446	0.984415	-4.142862
H	-3.469017	3.562789	-2.315901
H	-4.315155	2.789196	-0.972539
H	-4.789473	2.443730	-2.635814
H	-1.714185	-0.471695	2.171203
H	-3.923736	0.508420	2.604212
H	-4.142070	2.254450	2.506763
H	-4.019138	1.301838	1.031574
H	-2.063127	2.857227	0.274883
H	-2.272734	3.753917	1.770569
H	-0.688798	3.129282	1.347788
H	-0.637021	2.004590	3.678399
H	-1.929057	0.888008	4.143291
H	-2.251739	2.616251	4.007312
H	0.520341	1.486672	1.788707
H	0.595735	-0.159083	2.431864
H	3.071348	1.483909	1.928344
H	4.076438	3.707073	1.712091
H	4.349455	4.748047	-0.520224
H	3.597761	3.527942	-2.544391
H	2.568908	1.300169	-2.332943

B3LYP energy = -1393.22122622 a.u.

(5*R*,1’*R*)-**5a**, Conf. H

C	5.300417	-0.283644	1.123123
C	6.665488	-0.202278	0.861827
C	7.206297	-0.855147	-0.241004
C	6.374575	-1.593802	-1.078429
C	5.012055	-1.675862	-0.812035
C	4.455927	-1.018250	0.289482
C	2.971769	-1.116489	0.566329
C	2.126017	-0.247501	-0.394284
N	0.684192	-0.607065	-0.280204
C	-0.323874	0.189594	-0.030609
N	-1.514034	-0.382127	-0.070099
C	-2.742850	0.394834	-0.080189
C	-3.364413	0.650061	-1.318829
C	-2.752165	0.281687	-2.666876
C	-3.637647	-0.684529	-3.470937
C	-2.436983	1.545229	-3.488871
C	-4.579323	1.336336	-1.303160
C	-5.132765	1.794691	-0.117897
C	-4.464402	1.595268	1.079633
C	-3.256792	0.896747	1.131591
C	-2.543590	0.769330	2.472649
C	-1.997862	2.134433	2.932554
C	-3.434561	0.154272	3.564277
C	-1.367259	-1.836939	-0.455841
C	-1.979197	-2.906045	0.494948
C	-3.505778	-2.738052	0.582868
C	-1.368685	-2.877022	1.903613
C	-1.693937	-4.278080	-0.154064
C	0.174948	-1.947596	-0.634373
C	2.336107	1.246505	-0.241125
C	2.314039	1.871619	1.009172
C	2.502205	3.245516	1.114779
C	2.721987	4.011736	-0.026631
C	2.749479	3.398361	-1.274550
C	2.554826	2.024534	-1.379340
H	4.891023	0.226526	1.986830
H	7.305689	0.369300	1.522566
H	8.268252	-0.794163	-0.444028
H	6.788214	-2.113933	-1.933758
H	4.378289	-2.269189	-1.462561
H	2.665222	-2.157826	0.451236
H	2.757913	-0.835703	1.598237
H	2.399523	-0.518759	-1.417023
H	-0.197249	1.241776	0.173088
H	-1.797040	-0.213362	-2.494449
H	-3.137466	-0.969488	-4.398763
H	-3.858752	-1.595021	-2.911165
H	-4.589408	-0.221272	-3.737701
H	-1.939399	1.270178	-4.421351
H	-1.780594	2.223220	-2.939913
H	-3.345543	2.092610	-3.745430
H	-5.088778	1.535895	-2.237013
H	-6.075171	2.328027	-0.130426
H	-4.887012	1.991362	1.993613
H	-1.687576	0.108185	2.344132
H	-1.433325	2.021118	3.860565
H	-1.338325	2.582313	2.186595
H	-2.810657	2.839089	3.119510
H	-2.854891	0.005990	4.477719
H	-3.839258	-0.812132	3.260827
H	-4.274355	0.805939	3.811343
H	-1.860837	-1.959797	-1.418386
H	-3.968372	-2.766835	-0.406029
H	-3.928081	-3.556656	1.168626
H	-3.795746	-1.804591	1.060977
H	-1.561754	-1.935239	2.415362
H	-1.809161	-3.672199	2.507334
H	-0.289763	-3.043581	1.895235
H	-0.627451	-4.503703	-0.211927
H	-2.110666	-4.336808	-1.162335
H	-2.158623	-5.063992	0.443003
H	0.606703	-2.696232	0.023415
H	0.463819	-2.180811	-1.659551

H	2.155040	1.291956	1.910422
H	2.485049	3.716221	2.089685
H	2.875429	5.080187	0.057812
H	2.926829	3.986099	-2.166374
H	2.582842	1.552539	-2.354620

B3LYP energy = -1393.22101132 a.u.

(5*R*,1’*R*)-**5a**, Conf. I

C	3.798206	-1.498973	1.166145
C	4.511079	-2.639574	0.802782
C	5.394757	-2.597801	-0.270105
C	5.567305	-1.407158	-0.972641
C	4.856756	-0.270139	-0.605033
C	3.956511	-0.302063	0.465541
C	3.201639	0.944007	0.873817
C	2.189222	1.472072	-0.165720
N	1.053612	0.529755	-0.379358
C	-0.105072	0.547641	0.226521
N	-0.960233	-0.380460	-0.165622
C	-2.366875	-0.333908	0.201552
C	-2.770209	-0.838350	1.453711
C	-1.799683	-1.335483	2.518388
C	-1.743606	-0.354353	3.704913
C	-2.134001	-2.752502	3.012334
C	-4.135088	-0.824603	1.746232
C	-5.062241	-0.328666	0.843490
C	-4.634938	0.211673	-0.359178
C	-3.282094	0.245440	-0.699900
C	-2.873405	0.958902	-1.984913
C	-3.477214	0.302588	-3.237719
C	-3.239620	2.453260	-1.921831
C	-0.353049	-1.174627	-1.304124
C	-0.380948	-2.726366	-1.218156
C	-1.830972	-3.241589	-1.225445
C	0.358058	-3.277115	0.008356
C	0.308224	-3.238847	-2.501784
C	1.075263	-0.559394	-1.377304
C	1.675156	2.873008	0.131073
C	1.431466	3.742822	-0.935231
C	0.952347	5.028190	-0.708951
C	0.708991	5.462398	0.591057
C	0.951601	4.606255	1.659885
C	1.434048	3.320114	1.432090
H	3.119545	-1.539495	2.010707
H	4.378591	-3.557524	1.362159
H	5.952509	-3.482080	-0.552049
H	6.263951	-1.361898	-1.800769
H	5.016248	0.654759	-1.148643
H	2.692866	0.768203	1.822978
H	3.913945	1.754715	1.045034
H	2.684997	1.505467	-1.136656
H	-0.357728	1.282678	0.976596
H	-0.802062	-1.371195	2.081655
H	-0.991633	-0.678587	4.427539
H	-1.494393	0.659423	3.384656
H	-2.704789	-0.308180	4.220942
H	-1.368022	-3.093563	3.711843
H	-2.183369	-3.467668	2.190135
H	-3.091072	-2.779163	3.535868
H	-4.476711	-1.206922	2.699139
H	-6.117303	-0.342394	1.087173
H	-5.363621	0.635379	-1.038152
H	-1.789277	0.915424	-2.080645
H	-3.116308	0.805768	-4.137109
H	-3.213461	-0.754131	-3.310852
H	-4.566599	0.371817	-3.234596
H	-2.879615	2.964759	-2.816966
H	-2.792692	2.936111	-1.050873
H	-4.320029	2.597139	-1.867461
H	-0.911699	-0.906680	-2.200253
H	-2.383487	-2.866878	-2.089810
H	-1.828134	-4.331561	-1.282774
H	-2.378299	-2.958719	-0.328147
H	-0.114016	-2.968364	0.939423
H	0.342287	-4.368201	-0.018815
H	1.405121	-2.972918	0.040453
H	1.364238	-2.966701	-2.547779
H	-0.183768	-2.855961	-3.399220
H	0.252692	-4.327969	-2.532801
H	1.852238	-1.272777	-1.114275
H	1.299998	-0.145955	-2.359502
H	1.627393	3.416168	-1.950260
H	0.776329	5.691904	-1.546155
H	0.339650	6.464356	0.769702
H	0.773185	4.939330	2.674573
H	1.628972	2.675855	2.279722

B3LYP energy = -1393.22067027 a.u.

(5*R*,1’*R*)-**5a**, Conf. J

C	-3.800833	-1.482734	-1.163901
C	-4.518283	-2.621847	-0.804852
C	-5.401547	-2.580742	0.268390
C	-5.569157	-1.392200	0.975661
C	-4.854094	-0.256655	0.612339
C	-3.954251	-0.287953	-0.458611
C	-3.195136	0.956973	-0.862359
C	-2.180401	1.476951	0.179099
N	-1.048495	0.529266	0.388744
C	0.109575	0.544183	-0.218542
N	0.962322	-0.386195	0.171725
C	2.365596	-0.347393	-0.206873
C	3.294164	0.215001	0.691079
C	2.902585	0.922043	1.984736
C	3.515705	0.256010	3.227676
C	3.274808	2.415113	1.925448
C	4.643187	0.173520	0.335930
C	5.053048	-0.355487	-0.877848
C	4.112284	-0.832006	-1.777235
C	2.750903	-0.839408	-1.469404
C	1.761013	-1.309371	-2.528322

C	1.659434	-0.279348	-3.669365
C	2.097773	-2.701524	-3.086206
C	0.353769	-1.183245	1.306789
C	0.374735	-2.734819	1.211488
C	1.822426	-3.256441	1.208137
C	-0.371721	-3.276195	-0.014747
C	-0.310834	-3.251284	2.495418
C	-1.071639	-0.560966	1.385782
C	-1.660642	2.877059	-0.111753
C	-1.421880	3.330817	-1.410846
C	-0.933331	4.615787	-1.632535
C	-0.682194	5.464102	-0.559496
C	-0.923583	5.023424	0.738723
C	-1.408887	3.739355	0.958870
H	-3.122728	-1.522631	-2.008945
H	-4.389667	-3.538129	-1.367858
H	-5.962926	-3.463825	0.546876
H	-6.265487	-1.347357	1.804081
H	-5.009764	0.666770	1.159545
H	-2.687518	0.783139	-1.812526
H	-3.904733	1.770825	-1.029965
H	-2.676038	1.508284	1.150140
H	0.364773	1.276626	-0.970472
H	1.819304	0.883275	2.091000
H	3.167568	0.757096	4.133260
H	3.245658	-0.799266	3.298734
H	4.605402	0.318296	3.213406
H	2.928665	2.923161	2.828006
H	2.818528	2.904739	1.063079
H	4.355054	2.554511	1.857693
H	5.382629	0.583268	1.011914
H	6.105295	-0.374783	-1.133030
H	4.440444	-1.203867	-2.738836
H	0.775787	-1.376809	-2.068225
H	0.899819	-0.587704	-4.390920
H	1.393974	0.712976	-3.297529
H	2.608901	-0.187276	-4.200601
H	1.315654	-3.024631	-3.776324
H	2.177159	-3.446133	-2.292687
H	3.039511	-2.698744	-3.637536
H	0.915054	-0.923233	2.203579
H	2.382318	-2.888089	2.070498
H	1.814880	-4.346629	1.260787
H	2.364875	-2.972307	0.308131
H	0.103109	-2.972059	-0.946071
H	-0.367558	-4.367408	0.011325
H	-1.415504	-2.960805	-0.045685
H	-1.365176	-2.973847	2.548332
H	0.187588	-2.875948	3.392502
H	-0.260915	-4.340835	2.520030
H	-1.853102	-1.270264	1.124938
H	-1.290388	-0.147462	2.369293
H	-1.623485	2.693166	-2.261884
H	-0.756766	4.954038	-2.645838
H	-0.307917	6.465059	-0.733429
H	-0.741254	5.681160	1.579295
H	-1.603352	3.407837	1.972605

B3LYP energy = -1393.22066930 a.u.

(5*R*,1’*R*)-**5a**, Conf. K

C	3.508657	-1.624905	1.189565
C	4.118199	-2.824977	0.830472
C	5.058092	-2.850582	-0.193892
C	5.389441	-1.668739	-0.853143
C	4.780879	-0.472505	-0.490248
C	3.826966	-0.435302	0.532567
C	3.177926	0.870587	0.933915
C	2.234971	1.473772	-0.126985
N	1.068448	0.589780	-0.412535
C	-0.035397	0.498620	0.282444
N	-0.934604	-0.342996	-0.196358
C	-2.297291	-0.375052	0.311068
C	-3.286222	0.373248	-0.357142
C	-2.992098	1.328209	-1.509471
C	-3.748596	0.950848	-2.793478
C	-3.292326	2.782857	-1.104134
C	-4.597159	0.276081	0.111070
C	-4.910355	-0.490720	1.222381
C	-3.906972	-1.158185	1.906922
C	-2.580199	-1.116580	1.475091
C	-1.514750	-1.814680	2.311389
C	-1.281602	-1.059549	3.633497
C	-1.846372	-3.290240	2.586520
C	-0.444851	-0.899584	-1.519403
C	-0.515802	-2.434474	-1.755028
C	-1.977376	-2.915467	-1.744595
C	0.304075	-3.245291	-0.743327
C	0.050651	-2.674365	-3.171843
C	0.990723	-0.301857	-1.586864
C	1.770746	2.892629	0.173834
C	1.510910	3.752354	-0.897662
C	1.069352	5.051409	-0.677226
C	0.882585	5.512046	0.623500
C	1.144114	4.667687	1.696570
C	1.587678	3.365968	1.474511
H	2.787711	-1.612538	1.999116
H	3.862422	-3.736817	1.356065
H	5.536390	-3.781426	-0.471951
H	6.131016	-1.677501	-1.642516
H	5.062676	0.443485	-0.998111
H	2.640581	0.734132	1.873726
H	3.949952	1.620616	1.121312
H	2.772038	1.501324	-1.074968
H	-0.219565	1.085438	1.170512
H	-1.926779	1.289429	-1.733574
H	-4.828646	1.029102	-2.655885
H	-3.469313	1.624770	-3.605952
H	-3.527897	-0.070613	-3.108431
H	-4.354689	2.930704	-0.901986
H	-3.008579	3.461849	-1.910961
H	-2.737149	3.068147	-0.208637

H	-5.381939	0.827691	-0.390118
H	-5.934580	-0.549658	1.569102
H	-4.157653	-1.721424	2.796150
H	-0.576968	-1.791013	1.757482
H	-0.478323	-1.532249	4.202723
H	-1.008555	-0.016028	3.462559
H	-2.180431	-1.066657	4.253298
H	-1.013498	-3.768537	3.106041
H	-2.031634	-3.842473	1.663934
H	-2.728946	-3.392658	3.220254
H	-1.065708	-0.443789	-2.290866
H	-2.585981	-2.358170	-2.460214
H	-2.014403	-3.968358	-2.029657
H	-2.440765	-2.822273	-0.763776
H	-0.075437	-3.133559	0.270892
H	0.247298	-4.304899	-0.998921
H	1.359973	-2.971696	-0.741850
H	1.106913	-2.409544	-3.249284
H	-0.500810	-2.107050	-3.925592
H	-0.037418	-3.732360	-3.422758
H	1.764997	-1.062333	-1.511436
H	1.156009	0.277261	-2.494556
H	1.664519	3.404754	-1.913133
H	0.879706	5.705807	-1.518834
H	0.543131	6.525184	0.798401
H	1.011408	5.021589	2.711259
H	1.800455	2.735291	2.328165

B3LYP energy = -1393.22043024 a.u.

(5*R*,1'*S*)-**5b**, Conf. A

C	2.563685	-3.169579	1.492858
C	1.944662	-4.374830	1.805308
C	1.307188	-5.112328	0.810228
C	1.298529	-4.636983	-0.496499
C	1.918507	-3.428193	-0.805101
C	2.554651	-2.675629	0.183487
C	3.246255	-1.371408	-0.146913
C	2.586447	-0.130082	0.490395
N	1.245540	0.140308	-0.085617
C	0.109631	0.028374	0.553047
N	-0.963905	0.355990	-0.143423
C	-2.257839	0.566833	0.486426
C	-3.076060	-0.543623	0.772217
C	-2.639754	-1.988090	0.563054
C	-2.367555	-2.676200	1.914125
C	-3.649404	-2.802369	-0.261567
C	-4.331308	-0.293409	1.329545
C	-4.757810	0.997586	1.598421
C	-3.912880	2.069355	1.356416
C	-2.638594	1.881921	0.819305
C	-1.719496	3.092755	0.684983
C	-2.271183	4.142092	-0.294343
C	-1.441035	3.726443	2.060290
C	-0.543005	0.840752	-1.514967
C	-1.210976	0.192002	-2.760992
C	-0.956780	-1.317817	-2.866533
C	-0.599327	0.898431	-3.990985
C	-2.725026	0.464298	-2.768025
C	1.002539	0.659265	-1.445399
C	3.436161	1.132670	0.443428
C	3.364473	2.032577	1.509836
C	4.114869	3.202975	1.505288
C	4.949233	3.490336	0.428922
C	5.030301	2.599906	-0.636539
C	4.279894	1.427330	-0.629216
H	3.075240	-2.618189	2.274413
H	1.969524	-4.744398	2.823137
H	0.830444	-6.054215	1.051413
H	0.816604	-5.209137	-1.279795
H	1.920148	-3.079758	-1.831485
H	4.273242	-1.396998	0.224608
H	3.313872	-1.251237	-1.229086
H	2.393040	-0.360017	1.538305
H	0.052228	-0.287685	1.585155
H	-1.700327	-1.984481	0.011943
H	-1.984990	-3.685747	1.751585
H	-1.632849	-2.128696	2.508298
H	-3.280553	-2.753845	2.508124
H	-3.255511	-3.803645	-0.446931
H	-3.853550	-2.336145	-1.226549
H	-4.599870	-2.915750	0.262682
H	-4.985311	-1.124862	1.556794
H	-5.742031	1.167075	2.017127
H	-4.240020	3.069621	1.609238
H	-0.755050	2.759295	0.302320
H	-1.565712	4.969234	-0.396478
H	-2.445605	3.721533	-1.286279
H	-3.217350	4.555080	0.060298
H	-0.720129	4.540137	1.957686
H	-1.032678	2.995734	2.760806
H	-2.349606	4.140359	2.501022
H	-0.784702	1.901585	-1.554638
H	-1.374430	-1.863619	-2.021914
H	-1.429186	-1.702797	-3.771915
H	0.105130	-1.559592	-2.933042
H	-0.758712	1.978788	-3.952389
H	0.472453	0.715725	-4.090345
H	-1.076945	0.525177	-4.897969
H	-3.241818	-0.031232	-1.948491
H	-3.154198	0.096241	-3.701674
H	-2.939909	1.533043	-2.705879
H	1.367557	-0.053961	-2.180831
H	1.537677	1.597718	-1.577123
H	2.725507	1.812440	2.357724
H	4.053986	3.884913	2.344233
H	5.537648	4.399243	0.424183
H	5.682675	2.812832	-1.474108
H	4.368659	0.743985	-1.463897

B3LYP energy = -1393.22245278 a.u.

(5*R*,1’*S*)-**5b**, Conf. B

C	2.564218	3.180621	-1.502755
C	1.938451	4.384576	-1.806926
C	1.299301	5.113243	-0.806370
C	1.294742	4.630426	0.497697
C	1.921563	3.423162	0.797741
C	2.560183	2.679919	-0.196128
C	3.257338	1.377331	0.127632
C	2.587067	0.133688	-0.493485
N	1.246857	-0.121447	0.090394
C	0.112364	-0.039950	-0.555292
N	-0.961675	-0.348148	0.149153
C	-2.250643	-0.582841	-0.483294
C	-2.613829	-1.906083	-0.802276
C	-1.680885	-3.103414	-0.646992
C	-2.262295	-4.189091	0.272900
C	-1.312809	-3.688999	-2.022705
C	-3.879342	-2.114091	-1.352319
C	-4.733082	-1.054521	-1.616731
C	-4.324491	0.244263	-1.357522
C	-3.077693	0.514993	-0.790807
C	-2.658412	1.966297	-0.594937
C	-2.383216	2.642671	-1.951297
C	-3.683467	2.777667	0.213270
C	-0.539950	-0.797105	1.533209
C	-1.236725	-0.153029	2.765563
C	-0.611170	-0.823905	4.008618
C	-2.741091	-0.475003	2.765581
C	-1.032945	1.365630	2.856744
C	1.000481	-0.573979	1.472905
C	3.429471	-1.133352	-0.436422
C	4.256255	-1.432982	0.648075
C	4.999690	-2.609846	0.665677
C	4.928658	-3.499408	-0.401298
C	4.111419	-3.206954	-1.489348
C	3.367626	-2.032386	-1.504106
H	3.076517	2.635378	-2.288143
H	1.959371	4.760224	-2.822604
H	0.817698	6.054239	-1.041277
H	0.810979	5.195491	1.284982
H	1.927043	3.066383	1.821340
H	4.278105	1.400151	-0.260549
H	3.343193	1.262693	1.209069
H	2.390542	0.354408	-1.542865
H	0.056397	0.237780	-1.598412
H	-0.746190	-2.764775	-0.201692
H	-1.539570	-4.997177	0.402875
H	-2.510446	-3.793999	1.259629
H	-3.170888	-4.623603	-0.148003
H	-0.598098	-4.505893	-1.903226
H	-0.860222	-2.932487	-2.666775
H	-2.190735	-4.084499	-2.536375
H	-4.192504	-3.120706	-1.597574
H	-5.710355	-1.239859	-2.044918
H	-4.985532	1.065593	-1.601008
H	-1.722794	1.978723	-0.037428
H	-2.015157	3.658862	-1.796703
H	-1.635927	2.098104	-2.532353
H	-3.291674	2.702129	-2.554290
H	-3.302042	3.784921	0.392355
H	-3.890872	2.318291	1.180865
H	-4.630576	2.875591	-0.320116
H	-0.751351	-1.864224	1.587308
H	0.453011	-0.603649	4.113268
H	-1.108080	-0.455450	4.907171
H	-0.734376	-1.909382	3.982832
H	-3.268078	-0.005112	1.937402
H	-2.919757	-1.550991	2.713123
H	-3.188251	-0.111735	3.692584
H	-1.459364	1.889766	2.003019
H	0.019493	1.643424	2.932627
H	-1.527460	1.743280	3.753399
H	1.331967	0.191290	2.171305
H	1.561458	-1.485845	1.666685
H	4.336942	-0.750037	1.484093
H	5.638933	-2.826828	1.512290
H	5.511790	-4.411641	-0.388586
H	4.058524	-3.888400	-2.329230
H	2.741395	-1.808564	-2.360539

B3LYP energy = -1393.22243743 a.u.

(5*R*,1’*S*)-**5b**, Conf. C

C	4.320843	2.589424	-0.457636
C	5.657183	2.971556	-0.507083
C	6.565136	2.454992	0.413611
C	6.127046	1.557702	1.382160
C	4.789107	1.176059	1.428046
C	3.871395	1.684614	0.508122
C	2.416491	1.270897	0.546450
C	2.075014	0.223004	-0.534350
N	0.611542	0.050142	-0.671416
C	-0.335241	0.395637	0.161827
N	-1.556428	0.043518	-0.201594
C	-2.738334	0.602086	0.434981
C	-3.183647	0.064914	1.658817
C	-2.430348	-1.010588	2.431399
C	-1.781147	-0.417106	3.696226
C	-3.315821	-2.213024	2.796380
C	-4.348457	0.599341	2.211920
C	-5.039746	1.625433	1.587266
C	-4.550736	2.173135	0.411681
C	-3.380761	1.694773	-0.179557
C	-2.836641	2.418102	-1.407849
C	-3.795261	2.338164	-2.607703
C	-2.498383	3.883586	-1.077617
C	-1.503707	-0.660923	-1.540530
C	-2.165511	-2.064191	-1.649896
C	-3.681362	-1.967529	-1.403995
C	-1.549863	-3.097165	-0.695400
C	-1.952349	-2.527574	-3.107650

C	0.024202	-0.647902	-1.831463
C	2.755751	-1.121683	-0.325202
C	2.431151	-1.933925	0.763549
C	3.077282	-3.149876	0.953262
C	4.058345	-3.567535	0.056911
C	4.386405	-2.764871	-1.029884
C	3.734928	-1.549117	-1.220575
H	3.619510	3.008641	-1.171245
H	5.987722	3.677888	-1.258745
H	7.605226	2.754425	0.378789
H	6.825524	1.155364	2.105509
H	4.457616	0.478134	2.187820
H	2.177861	0.873013	1.534531
H	1.791912	2.153117	0.382792
H	2.400637	0.619147	-1.498462
H	-0.149392	0.942764	1.073744
H	-1.623935	-1.381141	1.799509
H	-2.539794	-0.069410	4.400158
H	-1.177506	-1.173819	4.201881
H	-1.136423	0.433144	3.462343
H	-2.713114	-2.986676	3.276551
H	-3.788205	-2.650534	1.915739
H	-4.105771	-1.932175	3.495122
H	-4.718587	0.206853	3.149986
H	-5.948321	2.014361	2.029873
H	-5.075361	3.002982	-0.043880
H	-1.898642	1.948605	-1.702921
H	-3.352119	2.827723	-3.477377
H	-4.022713	1.305418	-2.878571
H	-4.740872	2.838717	-2.391652
H	-2.040806	4.365667	-1.944085
H	-1.800689	3.952714	-0.240748
H	-3.392554	4.451595	-0.815331
H	-2.016976	-0.017694	-2.253074
H	-4.150013	-1.246425	-2.077214
H	-4.142133	-2.939448	-1.589555
H	-3.920122	-1.678475	-0.382193
H	-0.477885	-3.230096	-0.852376
H	-1.709376	-2.832177	0.348698
H	-2.020024	-4.068077	-0.860440
H	-2.369349	-1.810993	-3.819398
H	-2.458096	-3.481691	-3.262484
H	-0.898596	-2.676487	-3.350634
H	0.446373	-1.648235	-1.896861
H	0.275270	-0.106656	-2.743133
H	1.671886	-1.624539	1.472878
H	2.817598	-3.770968	1.801431
H	4.562796	-4.513861	0.206362
H	5.147914	-3.082277	-1.731128
H	3.995934	-0.926514	-2.068441

B3LYP energy = -1393.22221945 a.u.

(5*R*,1'*S*)-**5b**, Conf. D

C	4.870009	-0.627069	0.353153
C	5.414417	-1.848177	0.735837
C	4.939514	-3.031839	0.176050
C	3.921983	-2.983829	-0.771307
C	3.378505	-1.758957	-1.151596
C	3.840487	-0.566102	-0.591287
C	3.245478	0.763435	-0.995709
C	2.301992	1.387704	0.056016
N	1.130943	0.506352	0.330483
C	-0.024387	0.522670	-0.281541
N	-0.933007	-0.302080	0.209430
C	-2.160819	-0.613433	-0.506444
C	-3.261043	0.261081	-0.407776
C	-3.217344	1.598835	0.320227
C	-3.245045	2.765542	-0.685357
C	-4.347217	1.752220	1.351381
C	-4.436533	-0.104708	-1.066182
C	-4.521361	-1.280110	-1.795540
C	-3.408464	-2.096257	-1.922029
C	-2.199483	-1.774282	-1.304213
C	-0.984107	-2.653366	-1.579823
C	-1.178484	-4.096159	-1.085671
C	-0.622801	-2.634652	-3.076587
C	-0.325088	-1.098599	1.346972
C	-1.097063	-1.162418	2.695956
C	-1.305474	0.217061	3.337117
C	-0.239849	-2.034766	3.639066
C	-2.455619	-1.861388	2.515307
C	1.087565	-0.454723	1.450570
C	1.853738	2.804551	-0.268653
C	1.676725	3.711495	0.779929
C	1.250883	5.011406	0.532234
C	0.995299	5.424050	-0.772469
C	1.172206	4.531215	-1.823864
C	1.601153	3.230074	-1.574781
H	5.258513	0.288786	0.784994
H	6.216583	-1.875367	1.463086
H	5.367389	-3.982576	0.468565
H	3.556787	-3.898391	-1.222337
H	2.594875	-1.730929	-1.900612
H	4.043140	1.489659	-1.165941
H	2.714912	0.649549	-1.942160
H	2.833271	1.422307	1.008011
H	-0.225171	1.162982	-1.128088
H	-2.273688	1.663124	0.860778
H	-3.141730	3.717768	-0.161093
H	-2.437145	2.691489	-1.416011
H	-4.187391	2.786439	-1.236593
H	-4.220564	2.684420	1.905698
H	-4.355313	0.930775	2.069068
H	-5.326939	1.786684	0.871968
H	-5.300438	0.544159	-1.007057
H	-5.449369	-1.548885	-2.284718
H	-3.472957	-2.989828	-2.529381
H	-0.125544	-2.237562	-1.053735
H	-0.268880	-4.676979	-1.251967
H	-1.415247	-4.132175	-0.020883
H	-1.990279	-4.591286	-1.621597

H	0.294004	-3.202669	-3.247783
H	-0.465286	-1.615799	-3.436135
H	-1.410197	-3.085075	-3.683410
H	-0.231154	-2.124032	0.991324
H	-1.921220	0.867177	2.718168
H	-1.815384	0.098108	4.294541
H	-0.364649	0.732185	3.540662
H	-0.065708	-3.027370	3.216936
H	0.729011	-1.583556	3.861701
H	-0.763033	-2.164909	4.587490
H	-3.136315	-1.293622	1.884026
H	-2.931626	-1.984923	3.489777
H	-2.338362	-2.855294	2.078476
H	1.230089	0.090404	2.382635
H	1.886561	-1.184403	1.346277
H	1.882715	3.401330	1.798024
H	1.125705	5.702762	1.356141
H	0.667159	6.437080	-0.968383
H	0.984167	4.847007	-2.842350
H	1.744531	2.558525	-2.411423

B3LYP energy = -1393.22125304 a.u.

(5*R*,1'*S*)-**5b**, Conf. E

C	5.318950	-0.167446	-1.212735
C	6.674647	-0.102038	-0.902853
C	7.195776	-0.883233	0.123547
C	6.354792	-1.735184	0.834568
C	5.001692	-1.800536	0.519713
C	4.465343	-1.014406	-0.504357
C	2.989118	-1.090745	-0.826365
C	2.123346	-0.313196	0.193017
N	0.683320	-0.648770	0.002795
C	-0.329166	0.182284	0.005089
N	-1.522217	-0.383403	0.037224
C	-2.733209	0.358968	-0.277283
C	-3.339381	1.149331	0.718676
C	-2.738827	1.373480	2.100794
C	-2.176064	2.802332	2.223128
C	-3.733202	1.086395	3.237188
C	-4.524895	1.807445	0.386445
C	-5.082698	1.696771	-0.877457
C	-4.436842	0.957843	-1.856286
C	-3.239360	0.292616	-1.590250
C	-2.515283	-0.397871	-2.741318
C	-3.355503	-1.515305	-3.380744
C	-2.072128	0.629920	-3.799013
C	-1.369789	-1.887792	0.051574
C	-2.098088	-2.688313	1.169034
C	-3.622700	-2.533900	1.033310
C	-1.658413	-2.289640	2.584839
C	-1.749963	-4.173415	0.927197
C	0.178066	-2.036789	0.059484
C	2.348222	1.185886	0.170575
C	2.226504	1.927495	-1.008827
C	2.444502	3.300410	-1.004127
C	2.794700	3.949418	0.177434
C	2.921535	3.219492	1.353963
C	2.695875	1.845740	1.349546
H	4.923383	0.444209	-2.014675
H	7.323135	0.558421	-1.465311
H	8.250457	-0.833899	0.364296
H	6.754028	-2.354710	1.628315
H	4.358545	-2.479542	1.069500
H	2.797136	-0.712099	-1.831298
H	2.686239	-2.139109	-0.817231
H	2.376177	-0.678604	1.191775
H	-0.202075	1.253504	-0.031057
H	-1.901222	0.688480	2.224115
H	-2.970395	3.546626	2.137925
H	-1.693912	2.935699	3.193852
H	-1.438409	3.016400	1.446724
H	-3.231924	1.181589	4.202541
H	-4.146227	0.078920	3.168642
H	-4.567026	1.790342	3.230691
H	-5.016867	2.420843	1.129764
H	-6.009778	2.207165	-1.107156
H	-4.858940	0.915560	-2.852075
H	-1.602468	-0.853701	-2.358732
H	-2.777150	-2.027623	-4.152387
H	-3.670824	-2.256307	-2.643852
H	-4.254403	-1.115423	-3.853479
H	-1.484284	0.138641	-4.577361
H	-1.460317	1.418095	-3.355376
H	-2.929839	1.104358	-4.278894
H	-1.764184	-2.252895	-0.895353
H	-3.964434	-2.821688	0.036587
H	-4.120064	-3.184786	1.754676
H	-3.956110	-1.515057	1.221813
H	-0.582502	-2.396113	2.737682
H	-1.934016	-1.264102	2.824306
H	-2.149487	-2.937753	3.312594
H	-2.030088	-4.490283	-0.080385
H	-2.299794	-4.794602	1.635561
H	-0.687918	-4.383715	1.067790
H	0.546991	-2.511619	0.967809
H	0.535715	-2.603145	-0.797001
H	1.961444	1.438613	-1.939012
H	2.347754	3.863000	-1.924169
H	2.970748	5.017702	0.178297
H	3.198648	3.715859	2.275404
H	2.801185	1.281853	2.268967

B3LYP energy = -1393.22115871 a.u.

(5*R*,1'*S*)-**5b**, Conf. F

C	-4.189507	-2.343947	-0.944700
C	-5.399321	-2.726588	-1.513670
C	-6.596872	-2.225373	-1.009952
C	-6.574053	-1.343886	0.065701
C	-5.360789	-0.962002	0.631862
C	-4.153641	-1.454221	0.133370

C	-2.827684	-1.046342	0.738554
C	-2.066454	-0.020259	-0.130056
N	-0.646642	0.062805	0.300220
C	0.387452	-0.206578	-0.453405
N	1.561373	-0.094486	0.141160
C	2.810735	-0.087711	-0.601748
C	3.365719	-1.307125	-1.038643
C	2.680537	-2.659070	-0.874965
C	2.204889	-3.201909	-2.235908
C	3.571279	-3.695500	-0.170116
C	4.591905	-1.255190	-1.703855
C	5.238978	-0.051444	-1.933889
C	4.645462	1.138392	-1.542503
C	3.411216	1.153700	-0.891609
C	2.758152	2.500429	-0.595496
C	3.596264	3.361399	0.363779
C	2.462913	3.264598	-1.899406
C	1.359214	0.349144	1.574515
C	1.962054	-0.544178	2.696239
C	1.389022	-1.968857	2.697546
C	1.617243	0.145799	4.034044
C	3.495007	-0.594327	2.578057
C	-0.190024	0.494808	1.634959
C	-2.679902	1.370015	-0.204728
C	-2.711101	2.024562	-1.437950
C	-3.233180	3.308545	-1.550893
C	-3.729766	3.958563	-0.425261
C	-3.704165	3.315860	0.808382
C	-3.185439	2.029054	0.918079
H	-3.264486	-2.753154	-1.336924
H	-5.407936	-3.421305	-2.344645
H	-7.540176	-2.524654	-1.449581
H	-7.500829	-0.953593	0.468034
H	-5.356587	-0.278647	1.472507
H	-2.985805	-0.650915	1.742477
H	-2.189078	-1.927278	0.841852
H	-2.021642	-0.416624	-1.144743
H	0.293808	-0.485119	-1.493626
H	1.794034	-2.520271	-0.257252
H	1.658260	-4.137197	-2.097983
H	1.547852	-2.497043	-2.749454
H	3.050904	-3.403426	-2.895844
H	3.007752	-4.614887	0.001644
H	3.930420	-3.334788	0.794490
H	4.442124	-3.953401	-0.775364
H	5.046706	-2.174323	-2.048917
H	6.196356	-0.038806	-2.439820
H	5.139268	2.075029	-1.766717
H	1.792815	2.324942	-0.121770
H	3.069647	4.290092	0.592795
H	3.796867	2.844439	1.304080
H	4.558093	3.626939	-0.078992
H	1.933627	4.193379	-1.676696
H	1.841466	2.673424	-2.574992
H	3.381652	3.522961	-2.428624
H	1.824298	1.328199	1.671210
H	1.638247	-2.511437	1.786837
H	1.807120	-2.529293	3.535375
H	0.303228	-1.983120	2.813513
H	1.999402	1.168974	4.064057
H	0.543726	0.177140	4.229001
H	2.079096	-0.405359	4.854363
H	3.826604	-1.093421	1.669833
H	3.907438	-1.141543	3.427707
H	3.928270	0.408358	2.588480
H	-0.634114	-0.142054	2.396005
H	-0.504850	1.521494	1.812856
H	-2.332429	1.522410	-2.321074
H	-3.257363	3.797169	-2.516842
H	-4.139226	4.957333	-0.509640
H	-4.094096	3.813068	1.687757
H	-3.190435	1.545926	1.886969

B3LYP energy = -1393.22114490 a.u.

(5*S*,1'*S*)-**8a**, Conf. A

C	-3.501481	-1.294052	0.803072
C	-4.461046	-2.280691	0.579633
C	-5.303596	-2.190232	-0.524687
C	-5.200190	-1.122467	-1.404965
C	-4.247340	-0.134582	-1.170742
C	-3.389465	-0.197355	-0.075428
C	-2.345472	0.867728	0.217315
C	-2.715538	2.279861	-0.275268
C	-1.772548	3.363668	0.198241
C	-0.967765	4.059785	-0.705130
C	-0.117557	5.074501	-0.271647
C	-0.059757	5.407509	1.077125
C	-0.859375	4.722418	1.988795
C	-1.708391	3.711572	1.551552
N	-1.021485	0.446433	-0.304146
C	-0.004298	0.067230	0.425352
N	1.027576	-0.406239	-0.252398
C	2.333575	-0.615561	0.352262
C	3.323286	0.373915	0.184551
C	3.071930	1.727164	-0.472201
C	3.310432	2.874852	0.526200
C	3.921244	1.928422	-1.739126
C	4.592856	0.116025	0.703224
C	4.863524	-1.049407	1.402137
C	3.853976	-1.973544	1.618046
C	2.569828	-1.783456	1.104614
C	1.497248	-2.814804	1.433762
C	1.901429	-4.241916	1.028537
C	1.137859	-2.763472	2.930152
C	0.726763	-0.338376	-1.735253
C	0.899316	-1.636244	-2.576171
C	2.365588	-2.100701	-2.560345
C	0.541971	-1.255824	-4.029394
C	-0.015230	-2.779573	-2.113454
C	-0.720998	0.231928	-1.731181

C	-2.799695	-2.301339	2.872776
O	-2.626635	-1.313657	1.851497
H	-4.559844	-3.118115	1.254825
H	-6.043659	-2.963449	-0.689019
H	-5.857026	-1.049681	-2.261744
H	-4.187481	0.702789	-1.853744
H	-2.212605	0.911464	1.296921
H	-2.781735	2.304208	-1.363700
H	-3.722737	2.479476	0.097099
H	-1.017359	3.821889	-1.761806
H	0.492426	5.608816	-0.989692
H	0.596617	6.199300	1.415879
H	-0.830559	4.983785	3.039548
H	-2.341037	3.201311	2.269640
H	-0.004684	0.145508	1.502954
H	2.023382	1.789016	-0.761184
H	3.056458	3.830285	0.063489
H	2.695166	2.759577	1.420371
H	4.354862	2.921929	0.839534
H	4.986451	1.942774	-1.500510
H	3.674794	2.882218	-2.210113
H	3.758280	1.135799	-2.472071
H	5.376283	0.851726	0.575685
H	5.855735	-1.227188	1.797936
H	4.065438	-2.861853	2.198788
H	0.595438	-2.562177	0.877647
H	1.070813	-4.928170	1.206223
H	2.170037	-4.303289	-0.026828
H	2.752196	-4.598973	1.611331
H	0.337932	-3.473064	3.150746
H	0.803503	-1.768684	3.231287
H	1.997520	-3.024879	3.550196
H	1.406434	0.399062	-2.158885
H	3.038636	-1.306009	-2.889213
H	2.487038	-2.941306	-3.245921
H	2.688327	-2.427951	-1.574156
H	-0.502505	-0.958179	-4.139675
H	1.170744	-0.439755	-4.393947
H	0.704878	-2.116137	-4.680296
H	-1.074301	-2.524909	-2.185171
H	0.146086	-3.652662	-2.748184
H	0.192111	-3.078994	-1.087429
H	-0.791993	1.172138	-2.274253
H	-1.444894	-0.464586	-2.148352
H	-3.798024	-2.240340	3.310665
H	-2.055790	-2.075712	3.631773
H	-2.627409	-3.306991	2.483064

B3LYP energy = -1507.78938887 a.u.

(5*S*,1’*S*)-**8a**, Conf. B

C	2.421439	2.047250	-0.392582
C	3.063808	3.283152	-0.329394
C	3.928537	3.566640	0.724746
C	4.157803	2.627676	1.719999
C	3.513273	1.396219	1.650446
C	2.647409	1.080592	0.606437
C	1.992401	-0.289326	0.575681
C	2.365177	-1.179116	-0.632862
C	3.827026	-1.567189	-0.625454
C	4.751750	-0.904561	-1.434086
C	6.096870	-1.262690	-1.418184
C	6.536271	-2.291864	-0.591536
C	5.622461	-2.962522	0.217093
C	4.279137	-2.602478	0.197702
N	0.523973	-0.182383	0.742967
C	-0.407409	-0.289095	-0.167349
N	-1.643623	-0.166232	0.284672
C	-2.795426	-0.071572	-0.593874
C	-3.257173	-1.224253	-1.262834
C	-2.561504	-2.580181	-1.207365
C	-3.507880	-3.713194	-0.776361
C	-1.904036	-2.916773	-2.559336
C	-4.398750	-1.096383	-2.055888
C	-5.058292	0.115469	-2.185568
C	-4.556664	1.244210	-1.557299
C	-3.405816	1.186223	-0.770425
C	-2.847657	2.486987	-0.202821
C	-2.490008	3.470651	-1.331641
C	-3.806151	3.144733	0.804060
C	-1.615675	0.076800	1.779316
C	-2.356541	-0.946908	2.689320
C	-3.860646	-0.970912	2.368238
C	-2.195442	-0.434781	4.137303
C	-1.778390	-2.365603	2.585544
C	-0.084894	0.173076	2.038150
C	1.388758	2.588080	-2.501063
O	1.554532	1.699313	-1.391270
H	2.898642	4.028122	-1.093726
H	4.421278	4.530273	0.758319
H	4.830626	2.844618	2.539082
H	3.693869	0.655939	2.421206
H	2.324155	-0.818830	1.470673
H	1.756303	-2.085997	-0.581315
H	2.125427	-0.666395	-1.562906
H	4.418300	-0.104545	-2.084537
H	6.800091	-0.739447	-2.054434
H	7.581936	-2.573312	-0.580605
H	5.954379	-3.771068	0.856891
H	3.573874	-3.140454	0.822379
H	-0.192573	-0.454661	-1.210512
H	-1.765328	-2.529623	-0.465610
H	-2.947436	-4.643790	-0.665882
H	-3.993023	-3.495914	0.176163
H	-4.289406	-3.887256	-1.517769
H	-1.196459	-2.146248	-2.873216
H	-2.653646	-3.013910	-3.347123
H	-1.364902	-3.863974	-2.491239
H	-4.778820	-1.963939	-2.579153
H	-5.951614	0.184468	-2.793776
H	-5.055769	2.194525	-1.696792
H	-1.914918	2.268300	0.316096

H	-2.011838	4.359694	-0.915097
H	-1.803219	3.018495	-2.048857
H	-3.377247	3.795543	-1.877802
H	-3.354289	4.047054	1.221296
H	-4.052534	2.476029	1.630815
H	-4.743156	3.434281	0.324335
H	-2.079398	1.044808	1.954455
H	-4.299907	0.026372	2.440974
H	-4.375059	-1.610863	3.087476
H	-4.069096	-1.358012	1.372918
H	-1.155942	-0.429146	4.470044
H	-2.593614	0.576697	4.248950
H	-2.748755	-1.085839	4.815728
H	-0.724191	-2.408518	2.866055
H	-2.316351	-3.029473	3.264260
H	-1.878744	-2.776027	1.581652
H	0.227112	1.177801	2.322069
H	0.258657	-0.522448	2.800875
H	0.948789	3.536644	-2.186812
H	0.711044	2.084312	-3.185406
H	2.341456	2.771362	-3.002143

B3LYP energy = -1507.78780482 a.u.

(5*S*,1'*S*)-**8a**, Conf. C

C	-2.413742	2.044769	-0.619487
C	-2.967277	3.302179	-0.382354
C	-3.672156	3.536828	0.794860
C	-3.830847	2.528454	1.735060
C	-3.285623	1.271909	1.486401
C	-2.578905	1.004438	0.317680
C	-1.962491	-0.349527	0.012715
C	-2.690173	-1.580919	0.588842
C	-4.059445	-1.806445	-0.017100
C	-5.212417	-1.727994	0.765315
C	-6.469326	-1.954807	0.211081
C	-6.591499	-2.265117	-1.138996
C	-5.448301	-2.352221	-1.929408
C	-4.194705	-2.127558	-1.371283
N	-0.535762	-0.367508	0.429028
C	0.488081	-0.321211	-0.382336
N	1.668383	-0.222898	0.206778
C	2.910005	-0.500417	-0.498432
C	3.526545	0.520581	-1.247728
C	2.908803	1.894758	-1.474275
C	3.851801	3.044798	-1.084545
C	2.452361	2.049788	-2.937045
C	4.745852	0.225014	-1.860151
C	5.326211	-1.028278	-1.746548
C	4.671915	-2.032848	-1.050807
C	3.443453	-1.802986	-0.429956
C	2.722215	-2.979941	0.219851
C	2.332519	-4.029512	-0.837244
C	3.540791	-3.619084	1.353574
C	1.479235	-0.235061	1.709706
C	2.149151	0.895649	2.540851
C	3.680757	0.810614	2.423715
C	1.772337	0.627073	4.014291
C	1.671280	2.300100	2.146096
C	-0.071405	-0.284259	1.826358
C	-1.604215	2.698836	-2.790576
O	-1.689179	1.735998	-1.735391
H	-2.853446	4.099703	-1.101934
H	-4.097004	4.517436	0.969474
H	-4.381149	2.710088	2.648804
H	-3.428485	0.488165	2.218200
H	-1.928700	-0.458623	-1.069210
H	-2.057106	-2.448862	0.385035
H	-2.778091	-1.509913	1.673196
H	-5.130511	-1.492469	1.820021
H	-7.351547	-1.890482	0.836135
H	-7.568110	-2.442275	-1.572046
H	-5.532255	-2.603254	-2.979726
H	-3.314035	-2.215281	-1.998180
H	0.376683	-0.371922	-1.455920
H	2.019822	1.975988	-0.850084
H	3.333080	4.000581	-1.184518
H	4.198387	2.955355	-0.054177
H	4.730532	3.080234	-1.731027
H	3.302637	2.009804	-3.620791
H	1.955792	3.012279	-3.076246
H	1.755401	1.262135	-3.230262
H	5.246968	0.989305	-2.439597
H	6.278827	-1.229414	-2.220723
H	5.114120	-3.019800	-1.006495
H	1.788256	-2.624481	0.654175
H	1.752899	-4.831479	-0.375089
H	1.728503	-3.586509	-1.631714
H	3.214104	-4.478224	-1.298293
H	2.964867	-4.413865	1.832040
H	3.811534	-2.888005	2.117713
H	4.464592	-4.061938	0.976995
H	1.901309	-1.172641	2.068634
H	4.046129	-0.180917	2.700664
H	4.138675	1.534079	3.100621
H	4.034043	1.028826	1.417834
H	0.700046	0.722021	4.195262
H	2.085950	-0.370279	4.332118
H	2.274712	1.352814	4.655528
H	0.592869	2.425249	2.260240
H	2.148428	3.039729	2.791335
H	1.932819	2.545869	1.118252
H	-0.414713	-1.152190	2.386184
H	-0.482221	0.611514	2.287386
H	-2.597590	2.971088	-3.152621
H	-1.046297	2.215353	-3.587859
H	-1.070349	3.594455	-2.465588

B3LYP energy = -1507.78764834 a.u.

(5*S*,1'*S*)-**8a**, Conf. D

C	1.612598	3.340664	0.125770

C	1.304796	4.631217	-0.301288
C	1.308193	4.929897	-1.661515
C	1.618859	3.954071	-2.597421
C	1.935866	2.668434	-2.164491
C	1.940082	2.339114	-0.812163
C	2.295664	0.954949	-0.297335
C	3.151149	0.110662	-1.262806
C	3.684112	-1.176174	-0.671976
C	3.252775	-2.414819	-1.149450
C	3.757866	-3.600694	-0.620664
C	4.704003	-3.562857	0.397629
C	5.145882	-2.332733	0.880098
C	4.642013	-1.151450	0.347412
N	1.064247	0.235304	0.135457
C	-0.085190	0.227045	-0.485066
N	-1.048946	-0.444508	0.122321
C	-2.440084	-0.312355	-0.281190
C	-3.242855	0.649546	0.363143
C	-2.707783	1.663860	1.368787
C	-2.836624	3.097802	0.822178
C	-3.385383	1.540236	2.743710
C	-4.590746	0.710866	0.006415
C	-5.113402	-0.112287	-0.978716
C	-4.286242	-0.998902	-1.650039
C	-2.934062	-1.119341	-1.324687
C	-2.061477	-2.058052	-2.147783
C	-2.619601	-3.488975	-2.208210
C	-1.854462	-1.500983	-3.568864
C	-0.542643	-0.935311	1.464956
C	-0.794730	-2.418496	1.856274
C	-2.303304	-2.693325	1.981376
C	-0.162338	-2.604696	3.252976
C	-0.163319	-3.421095	0.880555
C	0.959718	-0.534246	1.389373
C	1.436020	3.945686	2.449016
O	1.615185	2.953846	1.433460
H	1.064546	5.405669	0.412465
H	1.066326	5.935606	-1.981763
H	1.624852	4.186715	-3.654059
H	2.195043	1.919089	-2.900469
H	2.854176	1.085697	0.628874
H	2.581705	-0.115307	-2.165790
H	3.989298	0.741488	-1.568846
H	2.524764	-2.454716	-1.951856
H	3.415203	-4.551911	-1.009192
H	5.101321	-4.483032	0.807502
H	5.892136	-2.294088	1.664194
H	5.008453	-0.201196	0.720250
H	-0.248608	0.754112	-1.414571
H	-1.642144	1.488197	1.510085
H	-2.392538	3.806391	1.524718
H	-2.326644	3.205737	-0.137040
H	-3.881480	3.379996	0.681370
H	-3.279268	0.536681	3.159711
H	-4.452571	1.760815	2.681321
H	-2.941798	2.247872	3.447084
H	-5.236615	1.430148	0.493209
H	-6.162329	-0.046067	-1.239794
H	-4.696879	-1.607062	-2.445499
H	-1.080637	-2.108078	-1.676386
H	-1.915680	-4.141874	-2.728455
H	-2.790435	-3.900541	-1.212414
H	-3.564876	-3.527682	-2.752218
H	-1.184044	-2.150962	-4.135329
H	-1.421980	-0.498556	-3.551240
H	-2.800824	-1.442233	-4.110013
H	-1.043777	-0.329742	2.220136
H	-2.820948	-2.616737	1.026811
H	-2.778594	-2.000859	2.679758
H	-2.458128	-3.704438	2.362405
H	0.922053	-2.480506	3.239644
H	-0.579513	-1.901162	3.977653
H	-0.369700	-3.613401	3.612980
H	-0.332756	-4.435768	1.245423
H	-0.602535	-3.355182	-0.113353
H	0.915199	-3.290563	0.782818
H	1.266735	0.094087	2.223368
H	1.620058	-1.397124	1.336218
H	2.200823	4.721754	2.378058
H	1.538041	3.420587	3.394997
H	0.443881	4.398270	2.389090

B3LYP energy = -1507.78735901 a.u.

(5*S*,1’*S*)-**8a**, Conf. E

C	2.042488	3.183258	0.149408
C	1.922755	4.506212	-0.271173
C	1.993317	4.810075	-1.628608
C	2.184561	3.806554	-2.566977
C	2.311438	2.486437	-2.140118
C	2.242621	2.151293	-0.790834
C	2.393627	0.728086	-0.279598
C	3.074847	-0.240991	-1.265848
C	3.416630	-1.591959	-0.677784
C	4.414030	-1.712817	0.295888
C	4.744510	-2.954215	0.827322
C	4.084873	-4.100679	0.389648
C	3.096494	-3.994621	-0.582560
C	2.766034	-2.748358	-1.110405
N	1.080873	0.202303	0.188499
C	-0.046281	0.245452	-0.469891
N	-1.084378	-0.284570	0.154156
C	-2.440005	-0.116564	-0.343418
C	-3.230053	0.930876	0.170086
C	-2.707653	1.994833	1.129904
C	-2.657459	3.370159	0.437930
C	-3.524602	2.070620	2.430158
C	-4.544827	1.034088	-0.286256
C	-5.045461	0.167111	-1.244798
C	-4.226314	-0.810060	-1.787336
C	-2.908582	-0.975838	-1.357287
C	-2.044317	-2.027872	-2.042128

C	-2.691117	-3.422546	-2.029995
C	-1.707812	-1.605454	-3.484826
C	-0.663845	-0.701037	1.551742
C	-1.061248	-2.113619	2.066018
C	-0.486772	-2.220237	3.495828
C	-0.496736	-3.255840	1.211535
C	-2.590956	-2.243799	2.164281
C	0.870804	-0.446937	1.495549
C	1.892689	3.793816	2.472887
O	1.972210	2.792258	1.454390
H	1.776602	5.301456	0.445092
H	1.898474	5.841412	-1.944544
H	2.243068	4.042625	-3.621260
H	2.475754	1.712640	-2.877963
H	2.993650	0.768829	0.628738
H	2.445265	-0.378973	-2.147120
H	3.990646	0.247845	-1.605727
H	4.947705	-0.831071	0.633506
H	5.524460	-3.028858	1.575197
H	4.346038	-5.068750	0.798586
H	2.584123	-4.881314	-0.935186
H	2.001539	-2.677684	-1.875910
H	-0.135885	0.704101	-1.444065
H	-1.681617	1.751251	1.402284
H	-2.227018	4.113766	1.112147
H	-2.048875	3.339665	-0.467918
H	-3.656229	3.710878	0.159184
H	-4.548820	2.395497	2.238340
H	-3.073813	2.791756	3.115117
H	-3.572668	1.105012	2.936393
H	-5.180198	1.820769	0.099464
H	-6.068435	0.267687	-1.585607
H	-4.615791	-1.455546	-2.563607
H	-1.101938	-2.100826	-1.500218
H	-1.997937	-4.159289	-2.441148
H	-2.958815	-3.736108	-1.019938
H	-3.596663	-3.449605	-2.638611
H	-1.040296	-2.336140	-3.946669
H	-1.220166	-0.629094	-3.520106
H	-2.610255	-1.543926	-4.096311
H	-1.124636	0.013718	2.233726
H	0.604304	-2.189675	3.510747
H	-0.791506	-3.169606	3.938529
H	-0.861369	-1.418772	4.137220
H	-0.903111	-3.248473	0.201941
H	0.591155	-3.230121	1.136971
H	-0.765324	-4.211880	1.664551
H	-3.076862	-2.207759	1.190740
H	-2.845131	-3.199334	2.626768
H	-3.017651	-1.453285	2.785568
H	1.212036	0.219319	2.285049
H	1.446517	-1.369321	1.535759
H	2.753925	4.464038	2.432656
H	1.895857	3.255510	3.416945
H	0.970678	4.372884	2.386926

B3LYP energy = -1507.78730329 a.u.

(5*S*,1’*S*)-**8a**, Conf. F

C	-3.105169	1.880259	0.783539
C	-3.868410	3.046860	0.803547
C	-4.815208	3.279230	-0.190770
C	-5.008857	2.355666	-1.207719
C	-4.245404	1.192211	-1.219525
C	-3.292795	0.928592	-0.237217
C	-2.532521	-0.387222	-0.290287
C	-2.765633	-1.313557	0.923696
C	-2.273534	-2.730527	0.723401
C	-1.232921	-3.245974	1.497848
C	-0.789842	-4.555045	1.322325
C	-1.384560	-5.370241	0.365818
C	-2.429754	-4.871200	-0.408309
C	-2.869692	-3.564599	-0.228652
N	-1.095383	-0.183758	-0.597781
C	-0.103657	0.035761	0.224788
N	1.073259	0.209921	-0.354960
C	2.220829	0.743670	0.360865
C	2.489666	2.124190	0.263570
C	1.568468	3.124142	-0.429427
C	1.028244	4.158284	0.574992
C	2.253657	3.826655	-1.613483
C	3.639714	2.607173	0.889110
C	4.467256	1.770149	1.620674
C	4.141996	0.430719	1.762927
C	3.015936	-0.116302	1.144668
C	2.689683	-1.584048	1.394257
C	3.846719	-2.520874	1.009185
C	2.284675	-1.814379	2.862620
C	0.908948	0.159996	-1.859807
C	1.812735	-0.819320	-2.662609
C	1.408049	-0.660684	-4.144908
C	1.633237	-2.285543	-2.244388
C	3.291951	-0.414013	-2.537428
C	-0.613941	-0.123943	-1.989295
C	-2.041720	2.427530	2.876747
O	-2.149702	1.592347	1.718595
H	-3.732955	3.778285	1.586644
H	-5.400310	4.189803	-0.159874
H	-5.744444	2.532225	-1.981308
H	-4.400889	0.462437	-2.006040
H	-2.903634	-0.918054	-1.166758
H	-2.329782	-0.879792	1.822210
H	-3.846495	-1.320409	1.083425
H	-0.772880	-2.625391	2.258301
H	0.015517	-4.937630	1.937328
H	-1.044687	-6.389353	0.229766
H	-2.908989	-5.503864	-1.145392
H	-3.698753	-3.197778	-0.823984
H	-0.236712	0.097577	1.292318
H	0.699158	2.593193	-0.816290
H	1.828383	4.781099	0.978472
H	0.308782	4.816487	0.083465

H	0.527960	3.671606	1.413724
H	2.618622	3.113282	-2.354611
H	3.106650	4.420368	-1.279370
H	1.552527	4.501632	-2.108464
H	3.879007	3.660362	0.818118
H	5.354399	2.166359	2.098959
H	4.775357	-0.204843	2.367949
H	1.833260	-1.853469	0.777704
H	3.538516	-3.561614	1.128124
H	4.157423	-2.378204	-0.026457
H	4.719676	-2.362122	1.644822
H	1.992640	-2.855772	3.013252
H	1.446130	-1.181065	3.159519
H	3.115163	-1.600381	3.538317
H	1.129315	1.158993	-2.231973
H	0.386054	-0.991722	-4.338448
H	2.068354	-1.266619	-4.767095
H	1.500350	0.376863	-4.475138
H	1.935895	-2.456378	-1.212895
H	0.603841	-2.630565	-2.356332
H	2.254212	-2.921875	-2.877333
H	3.669484	-0.517038	-1.522091
H	3.898953	-1.050394	-3.184072
H	3.448264	0.620457	-2.852075
H	-1.140676	0.663538	-2.527067
H	-0.821408	-1.075513	-2.475235
H	-1.741816	3.442293	2.608557
H	-1.271435	1.975766	3.496473
H	-2.984413	2.453610	3.426759

B3LYP energy = -1507.78725047 a.u.

(5*S*,1'*S*)-**8a**, Conf. G

C	2.213777	2.342627	0.100943
C	2.436957	3.493960	-0.653405
C	2.688271	3.389938	-2.018727
C	2.719565	2.148670	-2.638138
C	2.505321	1.001758	-1.878274
C	2.252087	1.071831	-0.511196
C	2.018667	-0.152958	0.351125
C	2.679007	-1.463166	-0.130544
C	4.190429	-1.446907	-0.042025
C	4.979591	-1.575481	-1.185859
C	6.369182	-1.578053	-1.099072
C	6.991866	-1.451040	0.137924
C	6.215803	-1.328341	1.287592
C	4.828297	-1.330275	1.196972
N	0.562939	-0.372353	0.571010
C	-0.421259	-0.031958	-0.217046
N	-1.623789	-0.384656	0.204085
C	-2.836946	0.153958	-0.386839
C	-3.315108	-0.384322	-1.598096
C	-2.573879	-1.444534	-2.403319
C	-3.455133	-2.656897	-2.745077
C	-1.976423	-0.835171	-3.685939
C	-4.507140	0.132888	-2.107715
C	-5.194831	1.143132	-1.454024
C	-4.676031	1.692139	-0.292040
C	-3.478225	1.230963	0.255778
C	-2.903286	1.959351	1.466420
C	-2.583601	3.425827	1.122561
C	-3.826545	1.876035	2.693223
C	-1.506098	-1.091715	1.536837
C	-2.112285	-2.519697	1.651906
C	-3.634521	-2.476003	1.431519
C	-1.860892	-2.981882	3.104054
C	-1.474156	-3.525887	0.682955
C	0.029641	-1.020992	1.782797
C	1.999494	3.594363	2.145971
O	1.939896	2.353768	1.435881
H	2.417958	4.468995	-0.188915
H	2.860244	4.291104	-2.593943
H	2.919206	2.067343	-3.698377
H	2.546060	0.038003	-2.366908
H	2.400106	0.074580	1.346483
H	2.298875	-2.274011	0.496227
H	2.360972	-1.692814	-1.148614
H	4.506845	-1.680450	-2.155413
H	6.963418	-1.680621	-1.998787
H	8.072529	-1.451650	0.207444
H	6.691454	-1.238011	2.256546
H	4.239356	-1.249083	2.104072
H	-0.268481	0.518181	-1.133761
H	-1.741368	-1.808555	-1.802734
H	-2.859318	-3.418695	-3.252006
H	-3.889133	-3.107226	-1.851190
H	-4.273750	-2.382828	-3.412906
H	-1.328445	0.016819	-3.468491
H	-2.763452	-0.486868	-4.357862
H	-1.387058	-1.583532	-4.220145
H	-4.902246	-0.260921	-3.034947
H	-6.124387	1.518557	-1.863521
H	-5.199175	2.510009	0.186570
H	-1.953819	1.497742	1.734436
H	-2.103386	3.912431	1.974279
H	-1.911066	3.495800	0.265410
H	-3.488552	3.987863	0.885289
H	-3.361271	2.369014	3.549339
H	-4.040926	0.842369	2.971500
H	-4.780569	2.371598	2.504016
H	-2.022924	-0.474471	2.269075
H	-4.115217	-1.771472	2.113815
H	-4.058456	-3.463264	1.623831
H	-3.900620	-2.193608	0.414816
H	-0.799052	-3.086446	3.335360
H	-2.299866	-2.287185	3.824261
H	-2.323228	-3.957769	3.259693
H	-0.399545	-3.636409	0.840567
H	-1.920398	-4.509975	0.835831
H	-1.638494	-3.252780	-0.358659
H	0.289136	-0.418309	2.653030
H	0.474677	-2.005834	1.901942

H	2.988259	4.049235	2.058910
H	1.803879	3.347889	3.186173
H	1.238453	4.291215	1.788104

B3LYP energy = -1507.78700822 a.u.

(5*S*,1’*S*)-**8a**, Conf. H

C	3.576772	1.572430	0.586111
C	4.238693	2.735906	0.186177
C	3.868250	3.378732	-0.990005
C	2.839249	2.876030	-1.777091
C	2.187161	1.714976	-1.376662
C	2.539521	1.047606	-0.206783
C	1.830811	-0.233837	0.205813
C	2.056133	-1.407201	-0.770359
C	3.499457	-1.861302	-0.805486
C	4.347454	-1.481644	-1.846909
C	5.673557	-1.904411	-1.874074
C	6.169201	-2.715403	-0.858293
C	5.330710	-3.103309	0.183601
C	4.006541	-2.679415	0.207416
N	0.386730	0.032669	0.407698
C	-0.636625	-0.446333	-0.248299
N	-1.820031	-0.045099	0.186973
C	-3.034488	-0.241528	-0.588177
C	-3.686009	-1.490266	-0.552414
C	-3.139247	-2.711654	0.177122
C	-4.150500	-3.311529	1.167710
C	-2.672483	-3.782895	-0.826403
C	-4.875511	-1.619344	-1.271870
C	-5.397788	-0.562551	-2.000330
C	-4.711171	0.640005	-2.061517
C	-3.506658	0.823379	-1.381550
C	-2.747412	2.130789	-1.583320
C	-2.365728	2.325105	-3.062212
C	-3.527006	3.348527	-1.059200
C	-1.633763	0.916220	1.341318
C	-2.379507	0.608974	2.671432
C	-1.981964	-0.740847	3.286226
C	-3.902566	0.658781	2.460755
C	-2.002205	1.744283	3.647859
C	-0.083858	0.944032	1.468455
C	4.952406	1.343154	2.547927
O	3.871261	0.892828	1.728903
H	5.040428	3.144045	0.783997
H	4.391456	4.279118	-1.287013
H	2.550124	3.376474	-2.691880
H	1.386917	1.320256	-1.992729
H	2.207865	-0.529263	1.183994
H	1.430490	-2.246788	-0.455067
H	1.743774	-1.120187	-1.777072
H	3.969445	-0.853461	-2.644710
H	6.317138	-1.602705	-2.691327
H	7.199647	-3.047829	-0.880252
H	5.705945	-3.741676	0.974115
H	3.359464	-2.995914	1.018201
H	-0.537141	-1.108502	-1.094279
H	-2.265315	-2.404522	0.750284
H	-3.687241	-4.129598	1.723118
H	-4.501668	-2.570753	1.887259
H	-5.022819	-3.717033	0.652522
H	-1.929848	-3.391527	-1.524811
H	-3.510405	-4.160395	-1.415619
H	-2.228691	-4.628169	-0.296191
H	-5.399489	-2.566089	-1.261800
H	-6.329301	-0.683089	-2.539247
H	-5.105923	1.445708	-2.666949
H	-1.809653	2.077114	-1.031210
H	-1.750402	3.220251	-3.174465
H	-1.800414	1.472780	-3.444388
H	-3.249215	2.448732	-3.691139
H	-2.932714	4.256635	-1.179282
H	-3.780154	3.246267	-0.002263
H	-4.459468	3.486727	-1.609799
H	-1.990775	1.887404	1.002841
H	-2.248501	-1.576777	2.641502
H	-2.506525	-0.878268	4.233311
H	-0.913366	-0.805155	3.500927
H	-4.212621	1.609445	2.021204
H	-4.262099	-0.141933	1.817409
H	-4.405981	0.561611	3.424346
H	-2.259808	2.724089	3.238642
H	-2.553624	1.618499	4.580761
H	-0.939460	1.747827	3.896789
H	0.332917	1.936431	1.305355
H	0.265101	0.574894	2.431341
H	5.895519	1.328029	1.997588
H	5.005602	0.641189	3.375813
H	4.765242	2.347751	2.934437

B3LYP energy = -1507.78668441 a.u.

(5*S*,1’*S*)-**8a**, Conf. I

C	-4.396728	-1.228014	-0.026595
C	-5.117190	-2.382682	-0.333345
C	-4.512686	-3.407021	-1.056413
C	-3.195146	-3.290797	-1.477693
C	-2.480931	-2.137277	-1.165190
C	-3.056776	-1.100717	-0.440101
C	-2.307418	0.187257	-0.126797
C	-2.416167	1.202530	-1.281429
C	-1.958427	2.611955	-0.966213
C	-2.478783	3.305582	0.131910
C	-2.093653	4.615019	0.395223
C	-1.184709	5.259988	-0.440604
C	-0.670014	4.585924	-1.541858
C	-1.054862	3.272125	-1.800028
N	-0.918138	-0.097168	0.285356
C	0.195342	0.030510	-0.388198
N	1.287942	-0.340595	0.260781
C	2.558603	-0.566203	-0.412397
C	3.396411	0.527640	-0.707506

C	3.012214	1.983231	-0.472800
C	4.029107	2.729951	0.406637
C	2.824832	2.717794	-1.813355
C	4.631065	0.255307	-1.299687
C	5.021662	-1.040207	-1.596633
C	4.158502	-2.094761	-1.344864
C	2.904300	-1.885832	-0.770396
C	1.971866	-3.084458	-0.618311
C	1.628422	-3.695703	-1.989369
C	2.546871	-4.155581	0.323893
C	0.910385	-0.845634	1.638224
C	1.604197	-0.189437	2.866071
C	1.342057	1.319784	2.966467
C	3.118148	-0.460004	2.836711
C	1.026614	-0.891395	4.114525
C	-0.634376	-0.679512	1.610744
C	-6.265648	-0.234782	1.120744
O	-4.912867	-0.170500	0.661711
H	-6.144597	-2.489399	-0.017417
H	-5.083307	-4.297402	-1.288852
H	-2.725698	-4.084932	-2.043054
H	-1.453261	-2.051445	-1.495450
H	-2.771922	0.630686	0.752530
H	-1.905115	0.820863	-2.168534
H	-3.476504	1.220624	-1.543981
H	-3.206823	2.829063	0.778123
H	-2.511485	5.136982	1.247367
H	-0.888535	6.281717	-0.238448
H	0.028794	5.080841	-2.204749
H	-0.658743	2.763909	-2.672165
H	0.233477	0.394373	-1.403557
H	2.053171	2.008026	0.043173
H	3.677389	3.745095	0.601419
H	4.178527	2.235306	1.367035
H	5.001060	2.806507	-0.083892
H	2.090942	2.218185	-2.448693
H	3.763373	2.771235	-2.368860
H	2.480067	3.738159	-1.636797
H	5.296660	1.075760	-1.533390
H	5.990288	-1.226242	-2.043631
H	4.455571	-3.099125	-1.617680
H	1.029290	-2.745033	-0.191238
H	0.910548	-4.509232	-1.865900
H	1.190335	-2.954573	-2.660498
H	2.514124	-4.105253	-2.477748
H	1.827872	-4.966820	0.454898
H	2.781558	-3.747693	1.308780
H	3.463732	-4.587880	-0.081096
H	1.166273	-1.902826	1.666476
H	1.748740	1.863482	2.115288
H	1.820550	1.713196	3.865022
H	0.278797	1.555467	3.041348
H	3.329947	-1.529914	2.780862
H	3.613120	0.023438	1.997154
H	3.572952	-0.081030	3.753705
H	1.181011	-1.972424	4.073567
H	1.532572	-0.518194	5.006025
H	-0.040618	-0.703803	4.245389
H	-1.160703	-1.628284	1.707756
H	-0.997470	0.000644	2.378993
H	-6.961003	-0.325804	0.283438
H	-6.444732	0.702769	1.640108
H	-6.408416	-1.068150	1.812308

B3LYP energy = -1507.78648897 a.u.

(5*S*,1’*S*)-**8a**, Conf. J

C	-3.927133	-1.129378	-0.146642
C	-4.780307	-2.212040	0.071540
C	-4.841116	-2.817749	1.322833
C	-4.057261	-2.350096	2.367582
C	-3.213353	-1.267001	2.145086
C	-3.126691	-0.636991	0.903620
C	-2.196577	0.565608	0.784403
C	-2.851888	1.851244	0.236534
C	-2.012799	3.096980	0.421291
C	-1.532395	3.805783	-0.681596
C	-0.775664	4.963637	-0.515697
C	-0.487364	5.431292	0.761717
C	-0.965805	4.737856	1.871020
C	-1.722179	3.583655	1.700396
N	-0.932883	0.245180	0.071255
C	0.199036	-0.020976	0.670342
N	1.188868	-0.386613	-0.123518
C	2.562465	-0.478198	0.342966
C	2.964561	-1.606182	1.086857
C	2.016793	-2.719214	1.518196
C	2.518149	-4.114431	1.110460
C	1.756210	-2.664063	3.035206
C	4.300978	-1.674437	1.484050
C	5.202805	-0.672540	1.162504
C	4.772374	0.447717	0.469456
C	3.443703	0.585423	0.065300
C	3.015889	1.892465	-0.594127
C	3.248726	3.088133	0.347396
C	3.716525	2.120694	-1.944049
C	0.712441	-0.369479	-1.559621
C	0.876197	-1.671881	-2.396286
C	0.330986	-1.347703	-3.804505
C	0.099478	-2.862828	-1.816786
C	2.362675	-2.038748	-2.543087
C	-0.762753	0.098757	-1.389484
C	-4.671829	-0.900960	-2.428729
O	-3.812271	-0.505401	-1.355536
H	-5.399493	-2.589189	-0.728893
H	-5.509428	-3.656506	1.472271
H	-4.103473	-2.813126	3.344396
H	-2.611022	-0.890210	2.964232
H	-1.870730	0.785932	1.800525
H	-3.115085	1.720927	-0.810418
H	-3.795246	1.958962	0.777580
H	-1.767641	3.462574	-1.682882

H	-0.419496	5.502906	-1.385005
H	0.096786	6.333478	0.893644
H	-0.758696	5.102803	2.869613
H	-2.105633	3.068095	2.574411
H	0.323636	0.060913	1.740805
H	1.059651	-2.563137	1.022855
H	1.766771	-4.866650	1.359072
H	2.717108	-4.176568	0.039590
H	3.435959	-4.381541	1.637157
H	2.674525	-2.838491	3.599385
H	1.035270	-3.432643	3.321711
H	1.358506	-1.695264	3.344844
H	4.640834	-2.528276	2.055253
H	6.237809	-0.755556	1.470133
H	5.476182	1.242088	0.256847
H	1.942773	1.855715	-0.778577
H	2.868454	4.002945	-0.111289
H	2.736545	2.950065	1.301336
H	4.310363	3.234777	0.553825
H	3.351792	3.040073	-2.406639
H	3.540294	1.297897	-2.639333
H	4.796168	2.218944	-1.816129
H	1.284163	0.401904	-2.071935
H	-0.736610	-1.120117	-3.798639
H	0.475779	-2.209791	-4.457352
H	0.858226	-0.500895	-4.250765
H	0.468156	-3.153441	-0.834437
H	-0.970203	-2.663682	-1.730359
H	0.213387	-3.725352	-2.475778
H	2.822508	-2.305670	-1.593542
H	2.461059	-2.895322	-3.212465
H	2.935098	-1.214926	-2.974962
H	-0.946408	1.055315	-1.874487
H	-1.481771	-0.620432	-1.765973
H	-4.488844	-1.937108	-2.722000
H	-4.428956	-0.241598	-3.257898
H	-5.722056	-0.773660	-2.158483

B3LYP energy = -1507.78607673 a.u.

(5*S*,1'*S*)-**8a**, Conf. K

C	-3.727639	1.490507	0.082918
C	-4.466047	2.650397	-0.154340
C	-4.508666	3.208095	-1.428816
C	-3.820306	2.615673	-2.477190
C	-3.088780	1.457258	-2.234691
C	-3.024201	0.872566	-0.970190
C	-2.227435	-0.418902	-0.827403
C	-3.028645	-1.615237	-0.276542
C	-2.319206	-2.948150	-0.385080
C	-1.942990	-3.469398	-1.627541
C	-1.311021	-4.704553	-1.721801
C	-1.046987	-5.445916	-0.572499
C	-1.425078	-4.944693	0.668256
C	-2.055489	-3.705702	0.757915
N	-0.942798	-0.233641	-0.100341
C	0.183138	0.070890	-0.691971
N	1.202541	0.308920	0.112412
C	2.565845	0.408965	-0.382987
C	3.387982	-0.733786	-0.336813
C	2.891173	-2.117400	0.067264
C	2.959275	-3.089589	-1.125037
C	3.648548	-2.685142	1.278737
C	4.712174	-0.596660	-0.755142
C	5.191921	0.608385	-1.243679
C	4.342452	1.698458	-1.348810
C	3.013316	1.629251	-0.927353
C	2.118108	2.845487	-1.133165
C	2.704155	4.123683	-0.511109
C	1.828642	3.063895	-2.630383
C	0.769304	0.076981	1.545204
C	1.078309	1.179407	2.597186
C	2.596852	1.370566	2.754296
C	0.528759	0.651066	3.940463
C	0.415141	2.526496	2.275774
C	-0.747398	-0.223811	1.364710
C	-4.430125	1.417888	2.387953
O	-3.637257	0.904514	1.312920
H	-5.010324	3.125840	0.648331
H	-5.088211	4.107977	-1.592500
H	-3.853139	3.041096	-3.471471
H	-2.559324	0.985629	-3.055270
H	-1.915266	-0.684315	-1.836825
H	-3.321169	-1.426506	0.753897
H	-3.954426	-1.643101	-0.856936
H	-2.159292	-2.916161	-2.535086
H	-1.033673	-5.094001	-2.693739
H	-0.560187	-6.410375	-0.646428
H	-1.237431	-5.519388	1.567029
H	-2.361911	-3.332692	1.729094
H	0.278903	0.114150	-1.767775
H	1.839503	-2.046106	0.340860
H	2.533062	-4.054992	-0.844855
H	2.399810	-2.707566	-1.981168
H	3.989237	-3.257377	-1.444993
H	3.588339	-2.020831	2.142768
H	4.705348	-2.836864	1.051117
H	3.229359	-3.652141	1.563861
H	5.371127	-1.454645	-0.719884
H	6.222664	0.691365	-1.565311
H	4.717298	2.622398	-1.769408
H	1.162719	2.653654	-0.646453
H	1.991667	4.945486	-0.607475
H	2.928737	3.993616	0.548517
H	3.624446	4.426678	-1.013137
H	1.134940	3.896841	-2.762485
H	1.387594	2.178821	-3.093194
H	2.744275	3.299626	-3.176483
H	1.283092	-0.823385	1.881276
H	2.792050	2.061695	3.576369
H	3.061283	1.780565	1.859427
H	3.094012	0.427343	2.990875

H	-0.556311	0.532183	3.930171
H	0.976097	-0.310833	4.202633
H	0.771406	1.357684	4.735444
H	0.780732	2.950411	1.342417
H	-0.671982	2.453947	2.210455
H	0.641414	3.240468	3.069679
H	-1.024062	-1.189883	1.779813
H	-1.384651	0.537667	1.802278
H	-4.141783	2.440601	2.640293
H	-4.233260	0.766897	3.235759
H	-5.493346	1.383612	2.141855

B3LYP energy = -1507.78590085 a.u.

(5*S*,1’*R*)-**8b**, Conf. A

C	-3.430915	1.595669	0.834938
C	-4.345975	2.630247	0.644923
C	-5.239005	2.577306	-0.421914
C	-5.229143	1.500606	-1.297309
C	-4.320440	0.465012	-1.095732
C	-3.415370	0.488779	-0.037899
C	-2.422145	-0.630344	0.225173
C	-2.867776	-2.017189	-0.274557
C	-1.986418	-3.154249	0.193227
C	-1.309876	-3.960276	-0.723498
C	-0.518032	-5.023114	-0.294859
C	-0.390660	-5.294610	1.062923
C	-1.064868	-4.501073	1.988331
C	-1.856792	-3.443176	1.555756
N	-1.087425	-0.260538	-0.307215
C	-0.029372	-0.003504	0.415704
N	1.045525	0.352313	-0.266356
C	2.221204	0.912626	0.378582
C	2.371568	2.313200	0.410851
C	1.300155	3.290458	-0.061863
C	0.804160	4.164853	1.104471
C	1.777824	4.169047	-1.229638
C	3.544987	2.826055	0.965004
C	4.507405	1.992180	1.512598
C	4.300576	0.622111	1.538157
C	3.158743	0.047461	0.976684
C	2.968402	-1.460005	1.089673
C	4.158625	-2.249503	0.520240
C	2.700163	-1.869690	2.549930
C	0.699521	0.432545	-1.739432
C	1.613358	-0.315791	-2.751346
C	1.675457	-1.829848	-2.509363
C	3.035457	0.271496	-2.732778
C	1.013048	-0.048833	-4.148661
C	-0.781882	-0.047457	-1.733449
C	-2.571810	2.585825	2.854673
O	-2.511805	1.573567	1.844616
H	-4.370549	3.475066	1.317575
H	-5.944408	3.387102	-0.560501
H	-5.924807	1.457970	-2.124971
H	-4.332297	-0.377580	-1.774709
H	-2.276730	-0.691903	1.302437
H	-3.884463	-2.164424	0.097341
H	-2.935156	-2.032841	-1.363117
H	-1.414261	-3.768216	-1.785402
H	-0.006700	-5.640755	-1.023000
H	0.220952	-6.122804	1.398515
H	-0.983085	-4.713808	3.047219
H	-2.393427	-2.849358	2.287597
H	-0.037998	-0.060109	1.494260
H	0.436249	2.721342	-0.404935
H	-0.019952	4.799553	0.771731
H	0.450348	3.554528	1.937049
H	1.594913	4.816833	1.479565
H	2.605183	4.813180	-0.925757
H	0.965429	4.812656	-1.573176
H	2.119023	3.571896	-2.076873
H	3.697477	3.897418	0.988345
H	5.409279	2.412481	1.940107
H	5.040759	-0.015616	2.003585
H	2.087911	-1.738891	0.512555
H	3.940129	-3.319085	0.540376
H	4.374748	-1.968134	-0.511484
H	5.064440	-2.088484	1.107245
H	2.496858	-2.940829	2.608401
H	1.842843	-1.339152	2.969753
H	3.563743	-1.656533	3.183395
H	0.739643	1.486636	-2.011191
H	2.307401	-2.291726	-3.270008
H	0.696516	-2.306923	-2.575017
H	2.102818	-2.072515	-1.538096
H	3.023206	1.348311	-2.913921
H	3.549728	0.094376	-1.789881
H	3.628270	-0.189073	-3.525210
H	0.945170	1.021708	-4.356170
H	1.653894	-0.494829	-4.910591
H	0.018315	-0.482581	-4.267333
H	-1.461090	0.693835	-2.150527
H	-0.912938	-0.981832	-2.274711
H	-1.795371	2.333101	3.571937
H	-3.543305	2.584197	3.353025
H	-2.372012	3.573958	2.435401

B3LYP energy = -1507.78916878 a.u.

(5*S*,1’*R*)-**8b**, Conf. B

C	1.946179	3.145635	-0.079021
C	1.776734	4.462584	0.344480
C	1.833984	4.765940	1.702509
C	2.059461	3.768170	2.639677
C	2.235393	2.454623	2.210557
C	2.182067	2.119872	0.860365
C	2.389757	0.705801	0.348046
C	3.142770	-0.229277	1.317616
C	3.536160	-1.557320	0.709591
C	2.879846	-2.736257	1.068545

C	3.237942	-3.955716	0.497851
C	4.262427	-4.012603	-0.441157
C	4.931920	-2.844884	-0.799793
C	4.572327	-1.629765	-0.227373
N	1.097004	0.104318	-0.084787
C	-0.066066	0.261577	0.488533
N	-1.084560	-0.298110	-0.143067
C	-2.384314	-0.448437	0.490909
C	-2.676634	-1.654833	1.157247
C	-1.663155	-2.776242	1.362817
C	-1.350778	-2.965372	2.858846
C	-2.116659	-4.101486	0.728451
C	-3.949887	-1.797587	1.709977
C	-4.881375	-0.773072	1.647082
C	-4.546060	0.429958	1.046271
C	-3.295357	0.625773	0.457487
C	-2.971010	1.991772	-0.135377
C	-4.008759	2.443541	-1.176603
C	-2.827858	3.051944	0.972154
C	-0.565254	-1.060594	-1.346619
C	-1.274902	-0.869171	-2.716521
C	-1.242393	0.579062	-3.224926
C	-2.730805	-1.362200	-2.649631
C	-0.516496	-1.771638	-3.715113
C	0.934868	-0.651690	-1.340642
C	1.775318	3.755009	-2.400903
O	1.893848	2.755223	-1.384239
H	1.604543	5.253886	-0.370367
H	1.701489	5.792582	2.020204
H	2.107651	4.004384	3.694434
H	2.425905	1.685397	2.946837
H	2.965661	0.774232	-0.574784
H	4.040276	0.304770	1.636808
H	2.539001	-0.401737	2.210689
H	2.089905	-2.704570	1.810220
H	2.721426	-4.860814	0.793295
H	4.544959	-4.959917	-0.883167
H	5.740868	-2.882319	-1.519082
H	5.110787	-0.729922	-0.504839
H	-0.191726	0.805720	1.413613
H	-0.724563	-2.491514	0.888479
H	-0.574732	-3.723069	2.986766
H	-0.998294	-2.037175	3.313163
H	-2.231494	-3.294568	3.413068
H	-3.034011	-4.470552	1.190930
H	-1.347989	-4.865259	0.862924
H	-2.304622	-3.995555	-0.341590
H	-4.208350	-2.718605	2.216215
H	-5.862820	-0.905054	2.085390
H	-5.269116	1.235201	1.035468
H	-2.008624	1.922991	-0.641219
H	-3.687100	3.377536	-1.642124
H	-4.143236	1.701622	-1.964966
H	-4.983098	2.624399	-0.719375
H	-2.552186	4.015340	0.538182
H	-2.061760	2.778841	1.700101
H	-3.767311	3.184033	1.512517
H	-0.652820	-2.118712	-1.100648
H	-1.710667	0.625287	-4.209654
H	-0.226735	0.962735	-3.333819
H	-1.790311	1.254531	-2.570786
H	-2.785815	-2.395507	-2.300622
H	-3.348635	-0.751572	-1.993742
H	-3.172013	-1.326745	-3.647535
H	-0.504714	-2.812822	-3.383632
H	-1.014580	-1.737674	-4.685151
H	0.515742	-1.450800	-3.868261
H	1.599793	-1.511376	-1.347874
H	1.194468	0.004436	-2.170346
H	1.804486	3.219648	-3.346040
H	2.607275	4.460843	-2.355701
H	0.828855	4.293466	-2.317090

B3LYP energy = -1507.78797889 a.u.

(5*S*,1’*R*)-**8b**, Conf. C

C	2.807094	-2.537535	0.089276
C	3.017824	-3.848537	-0.333972
C	3.146405	-4.125444	-1.692902
C	3.069617	-3.106102	-2.630514
C	2.866476	-1.796336	-2.201129
C	2.729930	-1.488600	-0.850720
C	2.525615	-0.073041	-0.339834
C	2.948324	1.038231	-1.322406
C	2.857957	2.432949	-0.745312
C	1.856698	3.311868	-1.162363
C	1.761868	4.594264	-0.626992
C	2.673086	5.016924	0.335146
C	3.681546	4.151956	0.754000
C	3.772695	2.872292	0.217023
N	1.124157	0.129135	0.123945
C	0.035398	-0.229197	-0.501895
N	-1.091012	0.011051	0.148234
C	-2.391106	-0.085352	-0.494724
C	-2.999997	-1.348594	-0.634222
C	-2.329160	-2.659389	-0.242299
C	-3.199817	-3.511925	0.695124
C	-1.935582	-3.467579	-1.493208
C	-4.270371	-1.393916	-1.211496
C	-4.912338	-0.241601	-1.636118
C	-4.271549	0.983165	-1.534067
C	-2.992317	1.090294	-0.986926
C	-2.301289	2.450090	-1.015639
C	-2.115556	2.940186	-2.463634
C	-3.043292	3.504468	-0.177679
C	-0.775465	0.716359	1.452614
C	-1.403439	0.162784	2.763675
C	-0.988150	-1.282482	3.074391
C	-2.938092	0.261112	2.718621
C	-0.904467	1.087122	3.896131
C	0.780499	0.708736	1.435142
C	2.860201	-3.161257	2.413720

O	2.660345	-2.175506	1.395898
H	3.086525	-4.654654	0.381829
H	3.309453	-5.147806	-2.010280
H	3.174974	-3.319662	-3.685954
H	2.828247	-1.006310	-2.938991
H	3.115762	0.035513	0.569526
H	3.978055	0.825575	-1.617819
H	2.340620	0.984515	-2.227410
H	1.152235	2.994947	-1.923076
H	0.981607	5.264016	-0.967402
H	2.605420	6.015220	0.749149
H	4.403426	4.477730	1.492839
H	4.569818	2.213416	0.543768
H	0.047201	-0.687017	-1.480652
H	-1.408829	-2.425820	0.291274
H	-2.645539	-4.395934	1.017016
H	-3.499041	-2.957641	1.585760
H	-4.107072	-3.857343	0.196657
H	-2.818627	-3.764310	-2.062833
H	-1.402684	-4.375666	-1.203554
H	-1.288476	-2.894777	-2.160399
H	-4.764192	-2.349542	-1.329935
H	-5.904303	-0.300455	-2.066541
H	-4.764895	1.871308	-1.907087
H	-1.299707	2.343828	-0.600318
H	-3.074577	3.132224	-2.947897
H	-1.546983	3.872700	-2.473179
H	-1.577376	2.206734	-3.067293
H	-3.159077	3.191247	0.861408
H	-4.040434	3.698121	-0.577607
H	-2.493427	4.447758	-0.187129
H	-1.134589	1.739328	1.345757
H	-1.438916	-1.592511	4.018705
H	0.091920	-1.394633	3.181275
H	-1.324958	-1.978140	2.307879
H	-3.267164	1.276947	2.490624
H	-3.377750	-0.408755	1.982052
H	-3.346562	-0.006189	3.694973
H	-1.180952	2.128144	3.712574
H	-1.360381	0.783692	4.839782
H	0.178216	1.040125	4.027851
H	1.202147	1.707404	1.521564
H	1.206988	0.075903	2.211168
H	3.860554	-3.594342	2.349173
H	2.109767	-3.952071	2.350596
H	2.753315	-2.635559	3.358847

B3LYP energy = -1507.78791330 a.u.

(5*S*,1’*R*)-**8b**, Conf. D

C	-2.360190	2.150698	0.645651
C	-2.883200	3.429134	0.457037
C	-3.582056	3.725188	-0.709951
C	-3.763064	2.757763	-1.688305
C	-3.249056	1.479453	-1.487760
C	-2.552140	1.149833	-0.329065
C	-1.969652	-0.229115	-0.072209
C	-2.725370	-1.421112	-0.694381
C	-4.072173	-1.665293	-0.047195
C	-5.261727	-1.426811	-0.736419
C	-6.495205	-1.664285	-0.135755
C	-6.556540	-2.145375	1.167599
C	-5.376507	-2.391612	1.864921
C	-4.146928	-2.155190	1.260260
N	-0.541533	-0.256378	-0.478465
C	0.475292	-0.275901	0.342415
N	1.663078	-0.261957	-0.236874
C	2.888259	-0.011055	0.501872
C	3.445646	1.282192	0.458918
C	2.766093	2.475781	-0.204782
C	2.396034	3.543676	0.840558
C	3.617094	3.083469	-1.331965
C	4.658151	1.486541	1.119134
C	5.271269	0.468161	1.831742
C	4.665343	-0.775185	1.920479
C	3.463099	-1.047027	1.265212
C	2.812959	-2.410860	1.462966
C	3.762020	-3.578902	1.151218
C	2.247022	-2.541061	2.890080
C	1.489454	-0.215297	-1.740354
C	2.140639	-1.357723	-2.572374
C	1.594154	-2.746712	-2.212587
C	3.670107	-1.336834	-2.408286
C	1.823013	-1.054176	-4.052636
C	-0.060004	-0.131607	-1.867070
C	-1.525787	2.703776	2.836219
O	-1.639750	1.783441	1.746399
H	-2.750617	4.195777	1.206452
H	-3.983520	4.721519	-0.846790
H	-4.306418	2.987813	-2.595309
H	-3.408909	0.729166	-2.251060
H	-1.947281	-0.381152	1.005085
H	-2.850786	-1.288413	-1.769286
H	-2.094639	-2.304112	-0.560941
H	-5.227835	-1.055120	-1.753533
H	-7.407071	-1.474775	-0.688699
H	-7.515111	-2.331714	1.635658
H	-5.413687	-2.774951	2.877276
H	-3.235900	-2.366076	1.810024
H	0.352984	-0.282214	1.415757
H	1.826647	2.144052	-0.645571
H	1.840224	4.355430	0.366582
H	1.775004	3.122885	1.633461
H	3.285053	3.974695	1.304316
H	4.551665	3.495041	-0.946274
H	3.073770	3.895979	-1.818795
H	3.871317	2.341726	-2.091521
H	5.118670	2.465652	1.093519
H	6.210899	0.650350	2.338314
H	5.133983	-1.550333	2.512484
H	1.971728	-2.491814	0.775978
H	4.594485	-3.623091	1.855556

H	3.221995	-4.525129	1.223870
H	4.178176	-3.504197	0.145544
H	1.725039	-3.493575	3.003809
H	1.543916	-1.738613	3.124764
H	3.045353	-2.505388	3.634087
H	1.938072	0.714581	-2.083718
H	2.053294	-3.496574	-2.859040
H	0.513730	-2.821839	-2.352843
H	1.821868	-3.021470	-1.183416
H	4.083556	-0.357903	-2.661249
H	3.984690	-1.582583	-1.396026
H	4.118367	-2.069294	-3.082037
H	2.185967	-0.065761	-4.344271
H	2.318236	-1.790601	-4.687159
H	0.755051	-1.103792	-4.273026
H	-0.395176	0.817177	-2.284621
H	-0.467637	-0.938341	-2.470529
H	-2.510160	2.982242	3.217617
H	-0.975998	3.599806	2.540626
H	-0.972119	2.178576	3.609988

B3LYP energy = -1507.78769920 a.u.

(5*S*,1’*R*)-**8b**, Conf. E

C	-2.518126	-1.868127	0.314851
C	-3.221693	-3.067461	0.205131
C	-4.096561	-3.268739	-0.859164
C	-4.274783	-2.284566	-1.820671
C	-3.566622	-1.092031	-1.707395
C	-2.688823	-0.858176	-0.651998
C	-1.958714	0.471923	-0.580115
C	-2.295264	1.350149	0.645283
C	-3.743107	1.788344	0.651554
C	-4.164931	2.848842	-0.155150
C	-5.495558	3.254014	-0.160945
C	-6.426362	2.604189	0.645249
C	-6.016599	1.550674	1.456291
C	-4.684325	1.147272	1.458517
N	-0.496665	0.283552	-0.733512
C	0.445665	0.436432	0.157340
N	1.664663	0.126807	-0.258015
C	2.853194	0.595909	0.439806
C	3.360374	-0.141578	1.527049
C	2.668626	-1.363606	2.117682
C	3.589542	-2.592711	2.187453
C	2.097937	-1.040641	3.511529
C	4.534685	0.316741	2.127364
C	5.176211	1.460905	1.680923
C	4.626318	2.201271	0.646166
C	3.445986	1.803062	0.017303
C	2.834334	2.725401	-1.032711
C	2.394924	4.058834	-0.399892
C	3.776544	2.969189	-2.222818
C	1.606507	-0.314822	-1.704269
C	2.243182	-1.686623	-2.066218
C	2.008797	-1.888870	-3.578903
C	1.619396	-2.858654	-1.295941
C	3.762231	-1.647350	-1.828937
C	0.081752	-0.219265	-1.993245
C	-1.507841	-2.553849	2.386667
O	-1.644044	-1.598811	1.329970
H	-3.097572	-3.847554	0.941744
H	-4.637246	-4.204448	-0.927715
H	-4.955350	-2.437411	-2.647780
H	-3.705495	-0.317161	-2.452450
H	-2.248902	1.038271	-1.468005
H	-2.070581	0.808592	1.562774
H	-1.657514	2.237965	0.610781
H	-3.445841	3.371015	-0.777427
H	-5.804038	4.081326	-0.788371
H	-7.462053	2.920558	0.644675
H	-6.732951	1.043423	2.090850
H	-4.373930	0.328252	2.096606
H	0.261925	0.807888	1.153424
H	1.826772	-1.620544	1.475991
H	3.029759	-3.457602	2.549586
H	4.004338	-2.845263	1.210813
H	4.423163	-2.431190	2.873153
H	2.891540	-0.769015	4.210290
H	1.581160	-1.911999	3.919229
H	1.388880	-0.210535	3.477035
H	4.949850	-0.231929	2.962573
H	6.092814	1.789027	2.155195
H	5.111067	3.116759	0.333099
H	1.929873	2.261105	-1.423369
H	3.248618	4.616855	-0.011137
H	1.900624	4.683026	-1.147435
H	1.697410	3.897194	0.424532
H	3.279037	3.582066	-2.977216
H	4.084503	2.033528	-2.693335
H	4.680092	3.497150	-1.912802
H	2.139336	0.436055	-2.285695
H	2.507369	-2.803322	-3.903690
H	2.421287	-1.061446	-4.161303
H	0.951422	-1.989676	-3.829752
H	1.788673	-2.777515	-0.223407
H	0.543994	-2.943836	-1.463378
H	2.070979	-3.794414	-1.629759
H	4.017414	-1.529942	-0.777629
H	4.210711	-2.579235	-2.178198
H	4.227321	-0.827878	-2.381613
H	-0.151146	0.472565	-2.802192
H	-0.361056	-1.184983	-2.226877
H	-1.094083	-3.494859	2.017700
H	-2.467037	-2.739618	2.874239
H	-0.820146	-2.109761	3.100460

B3LYP energy = -1507.78748250 a.u.

(5*S*,1’*R*)-**8b**, Conf. F

C	4.547361	-0.647204	0.103465
C	5.432254	-1.680906	-0.205180

C	5.009280	-2.743628	-0.998408
C	3.710797	-2.786307	-1.487951
C	2.831673	-1.753606	-1.174168
C	3.225142	-0.682361	-0.379250
C	2.290942	0.476503	-0.062127
C	2.266762	1.507714	-1.206715
C	1.540831	2.808012	-0.924078
C	0.606201	3.307272	-1.834011
C	-0.030468	4.526196	-1.612912
C	0.259525	5.266617	-0.472510
C	1.193316	4.782766	0.440300
C	1.829367	3.566847	0.214911
N	0.949841	-0.007917	0.317156
C	-0.179924	0.088331	-0.334067
N	-1.215616	-0.484459	0.255834
C	-2.582143	-0.251521	-0.180643
C	-3.363844	0.689280	0.519306
C	-2.827335	1.576222	1.638456
C	-2.948956	3.066501	1.272656
C	-3.515523	1.293132	2.984889
C	-4.693231	0.846247	0.124540
C	-5.215792	0.140210	-0.947517
C	-4.406650	-0.727389	-1.663952
C	-3.076025	-0.945775	-1.303196
C	-2.226148	-1.868954	-2.168230
C	-2.856653	-3.259011	-2.352405
C	-1.937275	-1.223951	-3.536567
C	-0.781300	-1.084446	1.576023
C	-1.073612	-2.593367	1.822735
C	-2.588730	-2.859349	1.826171
C	-0.538386	-2.907070	3.236899
C	-0.386122	-3.516736	0.807117
C	0.727923	-0.709779	1.596548
C	6.202457	0.531166	1.395118
O	4.880492	0.438388	0.858334
H	6.447556	-1.663796	0.163415
H	5.706521	-3.538667	-1.231357
H	3.382433	-3.611307	-2.106302
H	1.817658	-1.792043	-1.553935
H	2.671651	0.968409	0.832119
H	3.317187	1.721803	-1.418360
H	1.876930	1.044261	-2.116790
H	0.384107	2.748329	-2.736689
H	-0.748366	4.896184	-2.334515
H	-0.232449	6.215228	-0.297851
H	1.434466	5.358309	1.325618
H	2.569163	3.219676	0.926972
H	-0.278339	0.609668	-1.273212
H	-1.762056	1.380597	1.758802
H	-2.492268	3.678974	2.052787
H	-2.445763	3.289034	0.330458
H	-3.992457	3.372261	1.179745
H	-4.580449	1.528928	2.937813
H	-3.073682	1.908733	3.771135
H	-3.422846	0.246525	3.280914
H	-5.323270	1.550745	0.651937
H	-6.249417	0.281897	-1.237837
H	-4.815169	-1.246484	-2.521158
H	-1.267432	-2.014279	-1.672225
H	-3.788429	-3.206900	-2.917947
H	-2.173490	-3.907019	-2.905397
H	-3.073409	-3.735563	-1.395317
H	-1.280135	-1.866601	-4.126338
H	-1.455603	-0.249281	-3.433599
H	-2.858639	-1.076644	-4.103596
H	-1.304472	-0.535362	2.356284
H	-2.773423	-3.897859	2.107145
H	-3.043280	-2.694062	0.851411
H	-3.104007	-2.225513	2.551002
H	0.544546	-2.792444	3.310953
H	-1.002584	-2.265786	3.989900
H	-0.773141	-3.941478	3.492240
H	0.697450	-3.387792	0.788148
H	-0.580348	-4.557099	1.073560
H	-0.763848	-3.364518	-0.202946
H	0.982695	-0.041616	2.418947
H	1.376999	-1.581303	1.645112
H	6.948829	0.565760	0.598622
H	6.414970	-0.304348	2.065947
H	6.227823	1.462018	1.955311

B3LYP energy = -1507.78740473 a.u.

(5*S*,1'*R*)-**8b**, Conf. G

C	3.505098	-1.528388	0.591326
C	4.109353	-2.702809	0.135392
C	3.689003	-3.286103	-1.054934
C	2.665936	-2.713261	-1.800641
C	2.070715	-1.542965	-1.343410
C	2.474131	-0.933275	-0.159015
C	1.824606	0.359512	0.313695
C	2.069281	1.561602	-0.623697
C	3.525250	1.971052	-0.673329
C	4.066674	2.773405	0.334383
C	5.403996	3.153415	0.300129
C	6.221593	2.736369	-0.746964
C	5.691920	1.940679	-1.757803
C	4.352857	1.562173	-1.720418
N	0.377065	0.151632	0.550519
C	-0.632019	0.486922	-0.209270
N	-1.812078	0.052929	0.201646
C	-3.052405	0.581291	-0.341875
C	-3.537409	0.077666	-1.565126
C	-2.782356	-0.931562	-2.421517
C	-3.639253	-2.151230	-2.798026
C	-2.220345	-0.258294	-3.688010
C	-4.751106	0.582245	-2.034610
C	-5.452461	1.548407	-1.330693
C	-4.928268	2.065184	-0.156399
C	-3.710061	1.614298	0.354177
C	-3.137157	2.305995	1.587484
C	-2.858815	3.793483	1.304106

C	-4.042595	2.146222	2.819643
C	-1.645926	-0.713578	1.497103
C	-2.232993	-2.151560	1.576158
C	-3.763222	-2.120956	1.419892
C	-1.914175	-2.666166	2.997178
C	-1.622908	-3.111582	0.544627
C	-0.105322	-0.640195	1.697397
C	4.924583	-1.433399	2.532603
O	3.852184	-0.903045	1.750324
H	4.904766	-3.164537	0.701528
H	4.168186	-4.195593	-1.395302
H	2.337031	-3.168087	-2.725703
H	1.273708	-1.095844	-1.927003
H	2.232475	0.606304	1.292150
H	1.728852	1.324269	-1.633450
H	1.473356	2.404238	-0.263012
H	3.436146	3.112786	1.149001
H	5.805827	3.780416	1.086664
H	7.262200	3.034771	-0.777123
H	6.319102	1.616844	-2.579331
H	3.948673	0.945642	-2.514611
H	-0.527719	1.089848	-1.098869
H	-1.932387	-1.296715	-1.846362
H	-3.032160	-2.880270	-3.338547
H	-4.052521	-2.643896	-1.916694
H	-4.471682	-1.872543	-3.446596
H	-3.025978	0.100887	-4.331451
H	-1.625299	-0.971008	-4.263014
H	-1.585988	0.597766	-3.447198
H	-5.152367	0.214665	-2.969956
H	-6.398160	1.915243	-1.709879
H	-5.464281	2.849450	0.362504
H	-2.173909	1.856495	1.826964
H	-3.780359	4.341115	1.099486
H	-2.382183	4.256259	2.170924
H	-2.197481	3.918402	0.444456
H	-3.578014	2.611039	3.691613
H	-4.228286	1.096321	3.052642
H	-5.010358	2.626565	2.664135
H	-2.144444	-0.131791	2.270916
H	-4.164156	-3.123945	1.577324
H	-4.074718	-1.790900	0.430583
H	-4.225800	-1.461708	2.157715
H	-0.842396	-2.780272	3.169977
H	-2.314376	-1.997851	3.763568
H	-2.371419	-3.646449	3.139768
H	-0.539487	-3.201143	0.642240
H	-2.039940	-4.109771	0.688480
H	-1.846123	-2.810109	-0.477676
H	0.176606	-0.141779	2.624055
H	0.366914	-1.620181	1.674856
H	5.857453	-1.444847	1.964948
H	4.694858	-2.440908	2.887355
H	5.026253	-0.764314	3.382842

B3LYP) energy = -1507.78674298 a.u.

(5*S*,1’*R*)-**8b**, Conf. H

C	2.298140	-2.255153	0.001066
C	2.550017	-3.378547	-0.785135
C	2.794700	-3.229643	-2.147659
C	2.790766	-1.971285	-2.732211
C	2.546963	-0.852039	-1.940306
C	2.299727	-0.967280	-0.575731
C	2.031232	0.223232	0.323239
C	2.625287	1.575806	-0.125283
C	4.137165	1.628093	-0.066854
C	4.807583	1.479127	1.151232
C	6.195278	1.540328	1.214029
C	6.938692	1.759400	0.057132
C	6.283032	1.919212	-1.158823
C	4.893475	1.853451	-1.217917
N	0.571043	0.381684	0.567010
C	-0.405362	0.107546	-0.255739
N	-1.619669	0.315554	0.226785
C	-2.794259	0.352579	-0.630568
C	-3.420152	-0.853443	-1.003106
C	-2.878877	-2.232495	-0.647304
C	-3.927031	-3.120432	0.043315
C	-2.315661	-2.938872	-1.894916
C	-4.571884	-0.768335	-1.787548
C	-5.081299	0.454987	-2.193010
C	-4.417760	1.624672	-1.857643
C	-3.251082	1.603298	-1.092234
C	-2.507440	2.915652	-0.864871
C	-2.009300	3.497699	-2.200549
C	-3.352165	3.948421	-0.100436
C	-1.513950	0.876703	1.629641
C	-2.317956	0.176643	2.762317
C	-1.923211	-1.292883	2.967325
C	-3.829081	0.278460	2.493681
C	-2.013716	0.966826	4.054240
C	0.027243	0.875534	1.844484
C	2.109582	-3.568571	2.009463
O	2.031633	-2.308680	1.336466
H	2.559587	-4.366323	-0.348124
H	2.989761	-4.109371	-2.748163
H	2.985568	-1.855039	-3.790120
H	2.559480	0.125882	-2.401633
H	2.434662	-0.017019	1.306172
H	2.275948	1.824034	-1.128569
H	2.218105	2.345438	0.536251
H	4.243809	1.322801	2.064291
H	6.696282	1.423542	2.167238
H	8.019407	1.808396	0.104963
H	6.851371	2.095995	-2.063728
H	4.394928	1.985579	-2.171198
H	-0.236883	-0.257409	-1.258111
H	-2.051712	-2.103955	0.049182
H	-3.472944	-4.066689	0.344718
H	-4.339184	-2.644564	0.934373
H	-4.757537	-3.353781	-0.624977

H	-3.103266	-3.135202	-2.625297
H	-1.871625	-3.896791	-1.616098
H	-1.547323	-2.341610	-2.390057
H	-5.076074	-1.677453	-2.087880
H	-5.983617	0.495762	-2.790470
H	-4.800221	2.571595	-2.216338
H	-1.617310	2.715735	-0.269918
H	-1.420452	4.399630	-2.020399
H	-1.381449	2.782136	-2.735356
H	-2.840807	3.767158	-2.854177
H	-2.764297	4.848943	0.088848
H	-3.697768	3.561124	0.859901
H	-4.232564	4.243807	-0.674069
H	-1.875370	1.902789	1.583434
H	-2.490207	-1.706208	3.803237
H	-0.865138	-1.413641	3.206608
H	-2.141611	-1.901991	2.091688
H	-4.138800	1.315766	2.349784
H	-4.135993	-0.288059	1.616677
H	-4.377715	-0.114655	3.351540
H	-2.268314	2.023929	3.946036
H	-2.610039	0.564290	4.874339
H	-0.965395	0.896775	4.350933
H	0.414965	1.870330	2.058384
H	0.341245	0.202866	2.640777
H	3.105291	-4.005695	1.910882
H	1.359543	-4.266021	1.630044
H	1.908807	-3.356207	3.056162

B3LYP energy = -1507.78669495 a.u.

(5*S*,1’*R*)-**8b**, Conf. I

C	-2.422756	2.593002	-0.869609
C	-2.725328	3.954459	-0.876900
C	-3.320097	4.545927	0.233244
C	-3.617212	3.788018	1.356702
C	-3.320439	2.429320	1.354062
C	-2.727056	1.803613	0.257281
C	-2.503972	0.299204	0.329928
C	-3.142908	-0.511670	-0.824821
C	-3.174147	-2.001307	-0.570264
C	-4.099397	-2.552705	0.322419
C	-4.121354	-3.920476	0.573312
C	-3.217210	-4.763441	-0.068515
C	-2.298661	-4.229281	-0.966364
C	-2.280178	-2.858790	-1.215177
N	-1.079690	-0.074374	0.545886
C	-0.037948	0.296339	-0.152534
N	1.125583	-0.124167	0.314936
C	2.351720	-0.011839	-0.457494
C	3.040597	1.217967	-0.484297
C	2.529939	2.489377	0.183420
C	3.565319	3.115577	1.132004
C	2.076952	3.518925	-0.868446
C	4.236036	1.274612	-1.202943
C	4.731441	0.165340	-1.869969
C	4.011684	-1.018994	-1.868443
C	2.798775	-1.131000	-1.187473
C	2.005978	-2.426726	-1.326498
C	1.626117	-2.682716	-2.796284
C	2.754409	-3.635404	-0.740369
C	0.897763	-0.995925	1.532705
C	1.687090	-0.671813	2.833769
C	3.200715	-0.831721	2.609258
C	1.250341	-1.730767	3.869727
C	1.392832	0.728630	3.391850
C	-0.647425	-0.905889	1.685936
C	-1.661127	2.664806	-3.161729
O	-1.810437	1.963519	-1.920962
H	-2.500385	4.559933	-1.742696
H	-3.549383	5.603848	0.209515
H	-4.081337	4.241828	2.222369
H	-3.568598	1.831934	2.223808
H	-2.986877	-0.026626	1.249966
H	-4.161719	-0.133770	-0.937491
H	-2.618257	-0.293826	-1.752359
H	-4.818780	-1.908626	0.816739
H	-4.850089	-4.330192	1.262057
H	-3.236653	-5.829254	0.122215
H	-1.601849	-4.879868	-1.480662
H	-1.572611	-2.453925	-1.929838
H	-0.134260	0.897624	-1.045461
H	1.655749	2.233095	0.779872
H	3.123258	3.965537	1.655955
H	3.914924	2.402169	1.879542
H	4.436695	3.483281	0.587552
H	1.309707	3.112686	-1.530239
H	2.915352	3.838598	-1.490593
H	1.666790	4.403629	-0.377122
H	4.787961	2.204873	-1.240573
H	5.668244	0.230136	-2.409402
H	4.386852	-1.868044	-2.425160
H	1.067551	-2.323597	-0.782571
H	2.509072	-2.849350	-3.415886
H	0.996424	-3.571886	-2.871543
H	1.075845	-1.839245	-3.217877
H	2.138482	-4.533833	-0.816193
H	3.007287	-3.486427	0.310807
H	3.684490	-3.823871	-1.280056
H	1.175458	-2.009528	1.246345
H	3.723054	-0.710188	3.560053
H	3.600755	-0.094124	1.916106
H	3.444169	-1.824722	2.225349
H	0.193005	-1.650445	4.129440
H	1.436510	-2.744325	3.506551
H	1.821583	-1.596794	4.789567
H	0.336281	0.876836	3.622811
H	1.945031	0.870176	4.322539
H	1.702510	1.515252	2.706246
H	-1.128495	-1.880549	1.632336
H	-0.946351	-0.419901	2.613325
H	-0.983137	3.514272	-3.057472

H	-2.628922	3.005485	-3.534185
H	-1.236049	1.945456	-3.856665

B3LYP energy = -1507.78668404 a.u.

(5*S*,1'*R*)-**8b**, Conf. J

C	2.845031	-2.136027	-0.854242
C	3.392494	-3.418503	-0.854317
C	4.130545	-3.864698	0.238281
C	4.330211	-3.039049	1.335236
C	3.785942	-1.758565	1.325887
C	3.043039	-1.280937	0.246881
C	2.523630	0.147923	0.302638
C	2.956233	1.051615	-0.872981
C	2.729452	2.526161	-0.625777
C	1.788796	3.241155	-1.368698
C	1.594914	4.604312	-1.156373
C	2.340493	5.273394	-0.192002
C	3.284332	4.572135	0.555156
C	3.476873	3.212522	0.337474
N	1.059591	0.212602	0.563640
C	0.072808	-0.091187	-0.240330
N	-1.125364	-0.083588	0.322213
C	-2.355800	-0.116194	-0.451873
C	-3.016965	1.099717	-0.719931
C	-2.450199	2.472366	-0.369060
C	-2.237325	3.320971	-1.635569
C	-3.330406	3.227759	0.641494
C	-4.239145	1.038806	-1.390955
C	-4.766395	-0.168109	-1.821791
C	-4.064108	-1.343105	-1.607576
C	-2.847699	-1.350343	-0.922315
C	-2.108708	-2.674480	-0.770413
C	-2.966375	-3.760003	-0.099219
C	-1.592803	-3.169825	-2.133767
C	-1.001728	0.323607	1.775893
C	-1.637219	-0.600441	2.854913
C	-1.348135	0.070730	4.215790
C	-1.048501	-2.018947	2.858376
C	-3.164341	-0.669534	2.681971
C	0.536255	0.495021	1.912777
C	2.093344	-2.355726	-3.133957
O	2.094773	-1.646568	-1.888840
H	3.247808	-4.074693	-1.699864
H	4.548552	-4.863379	0.221400
H	4.906057	-3.379578	2.185624
H	3.953034	-1.105091	2.174639
H	2.949988	0.585791	1.203790
H	4.023071	0.863328	-1.016543
H	2.463548	0.739550	-1.790929
H	1.211532	2.731505	-2.132180
H	0.866910	5.142793	-1.750396
H	2.195265	6.334005	-0.028791
H	3.878558	5.087191	1.300056
H	4.229595	2.684556	0.913333
H	0.228098	-0.336242	-1.280163
H	-1.466792	2.341722	0.082037
H	-1.771663	4.272342	-1.372103
H	-1.590554	2.813609	-2.353430
H	-3.183012	3.540073	-2.133934
H	-4.313194	3.449462	0.220828
H	-2.863062	4.177123	0.910729
H	-3.485578	2.654669	1.557196
H	-4.777234	1.954834	-1.597355
H	-5.715506	-0.190474	-2.342851
H	-4.468149	-2.275664	-1.979198
H	-1.237355	-2.510738	-0.137893
H	-3.813237	-4.046650	-0.725431
H	-2.365862	-4.656153	0.070363
H	-3.359886	-3.430815	0.863188
H	-1.008101	-4.083269	-2.005512
H	-0.959597	-2.425982	-2.619959
H	-2.420351	-3.394097	-2.809709
H	-1.488814	1.292611	1.870890
H	-1.832887	-0.498144	5.010476
H	-1.740667	1.089904	4.249169
H	-0.281990	0.107811	4.447328
H	-1.252575	-2.551065	1.931058
H	0.031000	-2.025283	3.019881
H	-1.496202	-2.594797	3.670204
H	-3.457581	-1.157316	1.754515
H	-3.597833	-1.237012	3.507684
H	-3.613449	0.325961	2.694734
H	0.821585	1.503224	2.209798
H	0.970684	-0.211267	2.618084
H	1.600995	-3.325142	-3.039572
H	3.111691	-2.491048	-3.503109
H	1.534154	-1.733826	-3.828078

B3LYP energy = -1507.78618413 a.u.

(5*S*,1'*R*)-**8b**, Conf. K

C	-4.147635	0.924391	-0.146628
C	-5.117918	1.905658	0.066027
C	-5.228032	2.527788	1.305483
C	-4.376773	2.179607	2.344573
C	-3.415098	1.198761	2.128011
C	-3.277043	0.554285	0.898592
C	-2.214696	-0.531444	0.782600
C	-2.701112	-1.879231	0.201579
C	-1.746892	-3.027969	0.438678
C	-1.095033	-3.650770	-0.627462
C	-0.234533	-4.725003	-0.412397
C	-0.012052	-5.193217	0.878054
C	-0.658009	-4.583480	1.950978
C	-1.517854	-3.513008	1.731165
N	-0.994163	-0.032268	0.099774
C	0.196532	-0.002347	0.639530
N	1.158601	0.471335	-0.132658
C	2.456175	0.844583	0.408238
C	2.667967	2.183598	0.792164
C	1.574029	3.247348	0.803860

C	1.262520	3.690912	2.245220
C	1.922404	4.458338	-0.077085
C	3.940063	2.532374	1.247878
C	4.948317	1.588061	1.362430
C	4.693354	0.262817	1.046952
C	3.446564	-0.144084	0.568334
C	3.208977	-1.626497	0.310053
C	4.246828	-2.236475	-0.646018
C	3.172011	-2.409481	1.635962
C	0.560586	0.933179	-1.444548
C	1.225123	0.459152	-2.768103
C	2.660859	1.002021	-2.875793
C	0.397556	1.085160	-3.911984
C	1.232233	-1.066752	-2.931611
C	-0.924346	0.497203	-1.277133
C	-4.863229	0.595814	-2.424982
O	-3.980894	0.289380	-1.342026
H	-5.791816	2.189128	-0.728994
H	-5.988694	3.284630	1.450991
H	-4.460613	2.656697	3.312047
H	-2.756136	0.915314	2.940843
H	-1.879652	-0.728450	1.800747
H	-3.656347	-2.088766	0.689704
H	-2.915692	-1.774457	-0.858609
H	-1.274184	-3.305974	-1.639637
H	0.254415	-5.199494	-1.254473
H	0.651904	-6.031733	1.047457
H	-0.501392	-4.950196	2.958084
H	-2.031422	-3.063470	2.574554
H	0.383698	-0.323196	1.654568
H	0.654285	2.813604	0.413321
H	0.437752	4.406605	2.246828
H	0.977481	2.841558	2.869374
H	2.124896	4.173039	2.709075
H	2.803029	4.982377	0.298405
H	1.092939	5.168409	-0.084602
H	2.126784	4.163549	-1.107961
H	4.137244	3.556221	1.538307
H	5.927659	1.881409	1.719789
H	5.476335	-0.472629	1.178266
H	2.230997	-1.739295	-0.155159
H	5.246369	-2.235529	-0.207839
H	3.985778	-3.273758	-0.865488
H	4.296079	-1.693577	-1.591158
H	2.942540	-3.459821	1.446458
H	2.414182	-2.017151	2.317344
H	4.135420	-2.359901	2.147475
H	0.621047	2.021292	-1.443688
H	3.070939	0.749871	-3.855474
H	3.323844	0.583137	-2.121005
H	2.682840	2.089670	-2.778298
H	-0.624887	0.703685	-3.943733
H	0.354782	2.173620	-3.826226
H	0.863104	0.846021	-4.869192
H	0.227126	-1.491373	-2.934745
H	1.691441	-1.326193	-3.887106
H	1.804481	-1.560405	-2.148467
H	-1.614829	1.328394	-1.387895
H	-1.212914	-0.281538	-1.978399
H	-4.774785	1.642962	-2.723396
H	-5.899756	0.372344	-2.164271
H	-4.551250	-0.042905	-3.247124

B3LYP energy = -1507.78596434 a.u.

(5*S*,1’*S*)-**9a**, Conf. A

C	-0.755867	0.093927	-1.324629
C	0.580954	-0.683381	-1.506493
N	1.180968	-0.573604	-0.119939
C	0.374599	0.130601	0.652337
N	-0.729635	0.538427	0.081079
C	0.416449	-2.101501	-2.125052
C	1.785102	-2.780641	-2.308471
C	-0.192215	-1.885356	-3.528141
C	-0.509916	-3.015021	-1.309254
C	-1.851965	1.214702	0.775967
C	2.540335	-0.907345	0.270523
C	2.757120	-1.966846	1.174704
C	4.079214	-2.291571	1.482851
C	5.142229	-1.599325	0.924669
C	4.901267	-0.527831	0.079411
C	3.601267	-0.139675	-0.248112
C	3.407329	1.122632	-1.082434
C	3.967806	2.353962	-0.347566
C	4.024820	0.999659	-2.485084
C	1.636784	-2.732487	1.868425
C	1.770371	-4.255417	1.704793
C	1.562591	-2.359759	3.361219
C	-2.134359	2.605161	0.171788
C	-3.069576	0.301607	0.840402
C	-3.256034	-0.497896	1.973675
C	-4.354483	-1.341939	2.051580
C	-5.281905	-1.412098	1.014673
C	-5.099893	-0.614539	-0.115835
C	-3.993607	0.239520	-0.195826
C	-0.990784	3.585396	0.304958
C	-0.617215	4.076405	1.560316
C	0.422056	4.991536	1.687887
C	1.105978	5.435051	0.558543
C	0.740244	4.959487	-0.695956
C	-0.300482	4.041920	-0.819495
O	-5.935895	-0.590821	-1.187763
C	-7.105241	-1.412452	-1.166033
H	-0.819630	0.955775	-1.986122
H	-1.630081	-0.532182	-1.484888
H	1.234219	-0.125508	-2.175417
H	0.616230	0.363001	1.679694
H	2.267403	-3.012644	-1.360735
H	2.466890	-2.156786	-2.890463
H	1.653323	-3.718606	-2.850848
H	0.441320	-1.240774	-4.142171

H	-1.191349	-1.447106	-3.490334
H	-0.279719	-2.846386	-4.036890
H	-0.111631	-3.221888	-0.317368
H	-1.513005	-2.601156	-1.191886
H	-0.617521	-3.971771	-1.823061
H	-1.494761	1.356772	1.795791
H	4.278854	-3.101631	2.172078
H	6.159505	-1.880919	1.166826
H	5.738051	0.033075	-0.316633
H	2.339384	1.302250	-1.203476
H	3.755705	3.258837	-0.920248
H	3.518355	2.466851	0.640548
H	5.049416	2.284615	-0.219663
H	3.823920	1.902486	-3.065404
H	3.622612	0.146687	-3.034320
H	5.107893	0.875677	-2.430095
H	0.690033	-2.440813	1.415627
H	0.897247	-4.753868	2.130723
H	1.847077	-4.545850	0.656009
H	2.651094	-4.637835	2.223302
H	0.722875	-2.871385	3.836341
H	1.433324	-1.285466	3.509453
H	2.474501	-2.655582	3.883647
H	-3.014553	2.989605	0.691978
H	-2.417138	2.511390	-0.877493
H	-2.551077	-0.451208	2.794813
H	-4.501571	-1.954061	2.932694
H	-6.131300	-2.073837	1.101097
H	-3.898997	0.853131	-1.082011
H	-1.155034	3.756386	2.446236
H	0.690827	5.367504	2.667488
H	1.911400	6.152172	0.656281
H	1.258053	5.307974	-1.581175
H	-0.590102	3.694560	-1.805207
H	-7.617051	-1.221023	-2.105374
H	-6.844486	-2.471843	-1.103633
H	-7.760509	-1.144741	-0.333639

B3LYP energy = -1507.78847808 a.u.

(5*S*,1’*S*)-**9a**, Conf. B

C	-0.401179	0.481838	-1.709309
C	0.773225	-0.539937	-1.683088
N	1.030150	-0.663135	-0.195156
C	0.210965	0.140039	0.457870
N	-0.621927	0.817184	-0.290401
C	0.499918	-1.843626	-2.487359
C	0.285218	-1.406669	-3.953272
C	-0.742588	-2.602724	-1.999351
C	1.725333	-2.773042	-2.451373
C	-1.740021	1.650741	0.215601
C	2.179532	-1.285891	0.441905
C	3.448176	-0.697032	0.271177
C	4.546261	-1.352450	0.829855
C	4.392856	-2.513889	1.570129
C	3.126059	-3.031900	1.786974
C	1.991040	-2.436185	1.234175
C	0.625666	-3.028268	1.564156
C	0.524662	-4.519812	1.204562
C	0.279031	-2.809722	3.049076
C	3.677559	0.636522	-0.432142
C	4.299854	1.666337	0.528358
C	4.540075	0.487810	-1.696913
C	-1.621793	3.107798	-0.273419
C	-3.076879	0.989826	-0.095789
C	-3.758393	1.211235	-1.297391
C	-4.964957	0.564219	-1.530268
C	-5.514593	-0.302435	-0.589217
C	-4.837687	-0.522563	0.611650
C	-3.621447	0.126635	0.847655
C	-0.397992	3.832530	0.239625
C	0.644393	4.183283	-0.620003
C	1.763144	4.864783	-0.146735
C	1.855380	5.204338	1.198635
C	0.820787	4.862722	2.066385
C	-0.295764	4.184623	1.589489
O	-5.274190	-1.338219	1.607756
C	-6.521981	-2.015709	1.442649
H	-0.150957	1.378761	-2.272600
H	-1.313611	0.056159	-2.120274
H	1.662197	-0.085516	-2.117290
H	0.234569	0.238638	1.533837
H	1.145596	-0.851429	-4.334662
H	-0.605043	-0.788093	-4.082278
H	0.157484	-2.291144	-4.579143
H	-1.653172	-2.004578	-2.065577
H	-0.635476	-2.942069	-0.970664
H	-0.894352	-3.486286	-2.621588
H	2.623617	-2.262479	-2.804686
H	1.927852	-3.160773	-1.454957
H	1.551281	-3.625937	-3.109767
H	-1.613352	1.650308	1.298224
H	5.535528	-0.932630	0.701906
H	5.259187	-3.003192	1.997682
H	3.013927	-3.916924	2.399698
H	-0.127278	-2.502898	0.978110
H	-0.491026	-4.877489	1.385267
H	0.765536	-4.700813	0.156202
H	1.199428	-5.125618	1.811634
H	-0.726612	-3.179859	3.259078
H	0.317567	-1.754026	3.325233
H	0.975272	-3.344336	3.698194
H	2.712306	1.044471	-0.731636
H	4.369755	2.638740	0.037414
H	3.697384	1.786068	1.430163
H	5.306159	1.372773	0.831839
H	4.647179	1.452886	-2.196475
H	4.104247	-0.215931	-2.408295
H	5.541102	0.129282	-1.449494
H	-2.524801	3.618890	0.067255
H	-1.642231	3.144601	-1.363324
H	-3.365636	1.886362	-2.045348

H	-5.495118	0.737069	-2.458572
H	-6.457238	-0.787111	-0.797521
H	-3.120863	-0.052947	1.791830
H	0.574891	3.939994	-1.674379
H	2.557237	5.136569	-0.831345
H	2.721758	5.738551	1.568094
H	0.878240	5.135344	3.113076
H	-1.103546	3.943684	2.272151
H	-6.666525	-2.592386	2.352315
H	-7.344941	-1.306219	1.327384
H	-6.494716	-2.691602	0.584367

B3LYP energy = -1507.78844475 a.u.

(5*S*,1'*S*)-**9a**, Conf. C

C	-0.164523	0.242208	1.911806
C	-1.697736	0.320505	1.664441
N	-1.784876	-0.136916	0.224189
C	-0.586624	-0.498933	-0.199601
N	0.371678	-0.373575	0.681872
C	-2.561240	-0.442971	2.710642
C	-2.200011	-1.932014	2.814756
C	-4.057312	-0.288535	2.388613
C	-2.301239	0.240575	4.070439
C	1.802910	-0.727792	0.555909
C	-2.910626	0.032908	-0.679038
C	-3.279527	1.340543	-1.052298
C	-4.401211	1.488429	-1.868951
C	-5.102557	0.388350	-2.336409
C	-4.677066	-0.890336	-2.013039
C	-3.575567	-1.103448	-1.182630
C	-3.126962	-2.536714	-0.920628
C	-4.254467	-3.421932	-0.364687
C	-2.530170	-3.163411	-2.195061
C	-2.487381	2.586675	-0.670109
C	-1.924419	3.285345	-1.921500
C	-3.314620	3.570229	0.174474
C	2.065009	-1.561167	-0.716624
C	2.664749	0.522400	0.661048
C	3.655930	0.602923	1.640875
C	4.457641	1.735076	1.715178
C	4.285294	2.797213	0.832437
C	3.289699	2.718322	-0.144615
C	2.485031	1.578726	-0.223961
C	3.463011	-2.138255	-0.757722
C	4.464507	-1.541606	-1.525021
C	5.748247	-2.078442	-1.559261
C	6.047481	-3.221740	-0.825102
C	5.055012	-3.827250	-0.059100
C	3.772595	-3.289383	-0.028418
O	3.025987	3.691691	-1.058481
C	3.829889	4.873180	-1.053037
H	0.288626	1.223529	2.047952
H	0.095383	-0.378440	2.766132
H	-2.021163	1.358696	1.690647
H	-0.424258	-0.858697	-1.203742
H	-1.158376	-2.091903	3.101063
H	-2.380613	-2.464103	1.881649
H	-2.816805	-2.403080	3.581919
H	-4.335102	-0.771627	1.454060
H	-4.342810	0.763503	2.322218
H	-4.649695	-0.741880	3.185426
H	-1.265048	0.145061	4.399849
H	-2.551300	1.303489	4.035035
H	-2.928152	-0.222217	4.834001
H	2.017759	-1.358415	1.421661
H	-4.716082	2.481390	-2.162209
H	-5.968542	0.527041	-2.971638
H	-5.209499	-1.742160	-2.415252
H	-2.337172	-2.518453	-0.170633
H	-3.862466	-4.410849	-0.118395
H	-4.694296	-2.998638	0.539418
H	-5.055329	-3.559242	-1.093263
H	-2.149529	-4.164352	-1.981392
H	-1.709245	-2.565958	-2.597985
H	-3.285128	-3.252230	-2.978709
H	-1.625682	2.284322	-0.076205
H	-1.284687	4.120124	-1.627881
H	-1.330130	2.598919	-2.527640
H	-2.722547	3.682288	-2.551203
H	-4.168665	3.952035	-0.387993
H	-2.700828	4.425022	0.465817
H	-3.699816	3.104114	1.083369
H	1.902923	-0.943071	-1.602061
H	1.344545	-2.383022	-0.747685
H	3.804757	-0.215526	2.334192
H	5.230008	1.798115	2.471539
H	4.920284	3.667557	0.912781
H	1.726062	1.547228	-0.996331
H	4.240340	-0.651579	-2.100988
H	6.512904	-1.604553	-2.162397
H	7.045202	-3.641799	-0.853424
H	5.276868	-4.722865	0.508191
H	3.003618	-3.777694	0.560473
H	3.451612	5.490905	-1.863221
H	3.733292	5.413834	-0.108313
H	4.880800	4.637017	-1.236249

B3LYP energy = -1507.78784080 a.u.

(5*S*,1'*S*)-**9a**, Conf. D

C	-0.115707	0.877720	1.523988
C	-1.663040	0.958846	1.387816
N	-1.901483	0.055797	0.195894
C	-0.754201	-0.465218	-0.201863
N	0.289264	-0.104023	0.498990
C	-2.440682	0.647452	2.699870
C	-2.119954	-0.738961	3.277547
C	-3.957077	0.780315	2.477242
C	-2.018272	1.731477	3.715307
C	1.698742	-0.526021	0.342584
C	-3.119482	-0.049745	-0.589208

C	-3.535899	1.066811	-1.340717
C	-4.744183	0.967982	-2.031449
C	-5.486935	-0.202036	-2.021661
C	-5.019694	-1.310778	-1.333775
C	-3.830551	-1.267033	-0.604186
C	-3.346390	-2.539724	0.081024
C	-4.408288	-3.160685	1.002949
C	-2.868345	-3.570820	-0.958602
C	-2.715378	2.344033	-1.490563
C	-2.325376	2.580180	-2.961283
C	-3.438689	3.573350	-0.915897
C	1.814049	-1.760577	-0.576090
C	2.551720	0.651939	-0.103912
C	2.283004	1.319814	-1.303974
C	3.091065	2.377046	-1.693662
C	4.169429	2.787595	-0.910656
C	4.437215	2.121042	0.284779
C	3.619563	1.055593	0.682973
C	3.196539	-2.374579	-0.550710
C	3.579476	-3.202499	0.508078
C	4.849708	-3.767827	0.545561
C	5.756310	-3.514958	-0.480359
C	5.383458	-2.696033	-1.541428
C	4.111971	-2.130395	-1.575277
O	5.455575	2.428626	1.130666
C	6.336898	3.498063	0.782566
H	0.372976	1.830885	1.325680
H	0.202077	0.524952	2.502666
H	-1.951727	1.963496	1.086870
H	-0.694593	-1.131928	-1.048919
H	-1.057505	-0.868804	3.494138
H	-2.431521	-1.541791	2.610844
H	-2.654922	-0.872747	4.219320
H	-4.348397	0.024318	1.799243
H	-4.215960	1.762195	2.075150
H	-4.474594	0.669051	3.431782
H	-0.959373	1.675456	3.973961
H	-2.223614	2.734948	3.335053
H	-2.584828	1.603489	4.638779
H	2.021515	-0.820867	1.343402
H	-5.096993	1.815401	-2.604713
H	-6.420129	-0.256767	-2.568299
H	-5.589754	-2.230188	-1.363218
H	-2.489557	-2.288662	0.704741
H	-3.984095	-4.014454	1.535213
H	-4.769352	-2.445843	1.743711
H	-5.269245	-3.522350	0.438358
H	-2.470161	-4.455907	-0.458144
H	-2.087162	-3.166392	-1.606270
H	-3.692460	-3.889827	-1.599575
H	-1.779549	2.224103	-0.945386
H	-1.670609	3.450742	-3.038289
H	-1.797617	1.719392	-3.376596
H	-3.201781	2.767716	-3.584111
H	-4.362487	3.777935	-1.460519
H	-2.802765	4.457300	-0.996524
H	-3.699480	3.437959	0.135306
H	1.558490	-1.481448	-1.599983
H	1.088190	-2.507807	-0.243977
H	1.455105	1.016695	-1.933426
H	2.888653	2.895899	-2.622498
H	4.782974	3.613285	-1.240358
H	3.847206	0.551257	1.614037
H	2.877001	-3.416463	1.306402
H	5.128664	-4.411041	1.371134
H	6.744559	-3.956974	-0.454593
H	6.081129	-2.497784	-2.345818
H	3.830607	-1.494395	-2.406237
H	7.063930	3.554770	1.588303
H	6.852898	3.294804	-0.159000
H	5.799157	4.446740	0.710760

B3LYP energy = -1507.78768901 a.u.

(5*S*,1’*S*)-**9a**, Conf. E

C	0.805519	0.010658	1.287208
C	-0.638343	-0.516192	1.530734
N	-1.203949	-0.494915	0.124119
C	-0.331215	0.077790	-0.683447
N	0.806077	0.415482	-0.130912
C	-0.705091	-1.839513	2.344103
C	-0.047733	-1.532575	3.708135
C	0.043321	-3.002154	1.677109
C	-2.165637	-2.245476	2.608686
C	1.995137	0.910048	-0.868305
C	-2.589358	-0.708292	-0.264613
C	-2.921509	-1.845210	-1.028349
C	-4.268802	-2.044472	-1.334844
C	-5.244996	-1.155773	-0.912852
C	-4.885118	-0.015042	-0.212710
C	-3.552675	0.249837	0.106546
C	-3.210875	1.585467	0.758136
C	-3.554000	2.750125	-0.189250
C	-3.893284	1.772418	2.122929
C	-1.893518	-2.825388	-1.580328
C	-2.216331	-4.286225	-1.224649
C	-1.751297	-2.668623	-3.106376
C	2.488712	2.251774	-0.294978
C	3.057877	-0.179666	-0.957718
C	2.987296	-1.091946	-2.007480
C	3.931315	-2.112558	-2.108241
C	4.944289	-2.229869	-1.172410
C	5.026731	-1.313015	-0.119083
C	4.082425	-0.289397	-0.011388
C	1.485078	3.378057	-0.403604
C	0.913293	3.942381	0.737887
C	0.005968	4.994640	0.637933
C	-0.344378	5.497288	-0.610273
C	0.221095	4.944923	-1.757056
C	1.128822	3.896613	-1.652950
O	6.059124	-1.495898	0.746439
C	6.232739	-0.575369	1.823285

H	1.042713	0.859628	1.923590
H	1.561023	-0.759064	1.436129
H	-1.201228	0.226140	2.096412
H	-0.546822	0.262942	-1.726387
H	-0.542059	-0.698782	4.212421
H	1.014227	-1.296727	3.616514
H	-0.130520	-2.407272	4.354751
H	1.098258	-2.781939	1.504721
H	-0.402470	-3.281717	0.725127
H	0.002138	-3.877775	2.327144
H	-2.718800	-1.445198	3.105251
H	-2.698235	-2.508884	1.696690
H	-2.185347	-3.115888	3.266906
H	1.630377	1.094453	-1.878761
H	-4.557950	-2.910026	-1.916368
H	-6.285217	-1.340097	-1.151112
H	-5.650169	0.695628	0.071895
H	-2.134423	1.631718	0.919980
H	-3.235227	3.696986	0.250501
H	-3.052711	2.639049	-1.152602
H	-4.628117	2.809505	-0.374153
H	-3.581483	2.718078	2.570930
H	-3.640701	0.968957	2.817046
H	-4.980123	1.795912	2.024599
H	-0.925211	-2.588925	-1.141070
H	-1.403334	-4.937522	-1.551922
H	-2.349003	-4.424020	-0.150651
H	-3.127572	-4.626300	-1.719401
H	-0.966988	-3.329966	-3.480644
H	-1.497863	-1.645527	-3.391832
H	-2.680999	-2.931234	-3.615094
H	3.394385	2.507202	-0.849650
H	2.790239	2.132467	0.746439
H	2.207653	-1.005133	-2.755063
H	3.879339	-2.816091	-2.929728
H	5.687590	-3.013339	-1.242686
H	4.155545	0.425811	0.794087
H	1.192638	3.572687	1.718227
H	-0.419207	5.425485	1.536163
H	-1.045679	6.318406	-0.690288
H	-0.035664	5.339216	-2.732624
H	1.578499	3.492231	-2.553367
H	7.117396	-0.910521	2.357991
H	6.395564	0.440389	1.454268
H	5.374913	-0.589295	2.500950

B3LYP energy = -1507.78726318 a.u.

(5*S*,1’*S*)-**9a**, Conf. F

C	0.369525	0.544767	1.754713
C	-0.907694	-0.345059	1.709689
N	-1.053379	-0.584265	0.220612
C	-0.113828	0.083817	-0.422494
N	0.724870	0.741320	0.336577
C	-0.856488	-1.576562	2.658531
C	-2.173450	-2.370233	2.595839
C	-0.715949	-1.003515	4.086064
C	0.325279	-2.516080	2.375465
C	1.943692	1.436595	-0.146968
C	-2.193260	-1.170834	-0.465683
C	-3.414607	-0.468788	-0.477761
C	-4.506475	-1.082715	-1.093163
C	-4.385593	-2.316249	-1.713144
C	-3.155798	-2.954251	-1.749049
C	-2.032192	-2.402852	-1.131072
C	-0.697793	-3.127473	-1.256275
C	-0.778224	-4.600363	-0.824563
C	-0.147919	-3.014159	-2.690193
C	-3.590770	0.940260	0.077902
C	-3.966227	1.927313	-1.042551
C	-4.620222	0.996846	1.218777
C	1.964506	2.908147	0.310092
C	3.196823	0.651411	0.217849
C	3.794791	0.748359	1.471371
C	4.931085	-0.010635	1.754850
C	5.476280	-0.854339	0.803587
C	4.887143	-0.948188	-0.462340
C	3.746461	-0.197550	-0.749880
C	0.814679	3.736354	-0.218032
C	-0.150753	4.257808	0.644878
C	-1.198973	5.036106	0.159208
C	-1.295994	5.304221	-1.201800
C	-0.336619	4.793702	-2.073041
C	0.709031	4.018564	-1.583858
O	5.496613	-1.787492	-1.340730
C	4.973720	-1.903542	-2.663641
H	0.181708	1.505353	2.229724
H	1.196509	0.057694	2.266131
H	-1.770684	0.246116	2.012929
H	-0.051578	0.095457	-1.501836
H	-2.325184	-2.849635	1.630321
H	-3.037001	-1.732322	2.796794
H	-2.159542	-3.153512	3.355791
H	-1.537267	-0.324345	4.327222
H	0.223940	-0.467268	4.230104
H	-0.737169	-1.820535	4.808582
H	0.251956	-2.978702	1.392951
H	1.291929	-2.013288	2.442257
H	0.331962	-3.319707	3.113853
H	1.850219	1.424152	-1.232992
H	-5.461696	-0.574197	-1.107332
H	-5.246135	-2.770993	-2.187954
H	-3.065197	-3.899286	-2.268118
H	0.019203	-2.637368	-0.599338
H	0.217966	-5.047335	-0.839911
H	-1.179926	-4.703792	0.184446
H	-1.408815	-5.184190	-1.497199
H	0.833880	-3.487711	-2.756516
H	-0.045104	-1.972747	-3.002676
H	-0.808302	-3.508538	-3.405312
H	-2.636290	1.281931	0.476500
H	-4.008009	2.943560	-0.646062

H	-3.232982	1.909303	-1.850955
H	-4.943273	1.693848	-1.469436
H	-4.674206	2.008387	1.626351
H	-4.364965	0.315986	2.032681
H	-5.617868	0.729135	0.865580
H	2.913082	3.322360	-0.038588
H	1.989675	2.964192	1.398885
H	3.401818	1.413715	2.228154
H	5.397428	0.065222	2.729119
H	6.361648	-1.440563	1.012884
H	3.285192	-0.254193	-1.726687
H	-0.073294	4.073775	1.710743
H	-1.932893	5.439383	0.846177
H	-2.106837	5.913411	-1.581124
H	-0.396265	5.010057	-3.132656
H	1.460934	3.646807	-2.271524
H	5.628022	-2.601132	-3.179528
H	3.955666	-2.302040	-2.655205
H	4.989289	-0.941202	-3.181566

B3LYP energy = -1507.78708226 a.u.

(5*S*,1'*S*)-**9a**, Conf. G

C	-0.032249	-0.360722	-1.517590
C	1.507417	-0.587588	-1.460180
N	1.855617	0.058029	-0.135125
C	0.761958	0.561867	0.407606
N	-0.338007	0.372672	-0.273758
C	1.942353	-2.063338	-1.691456
C	1.324993	-3.043023	-0.683029
C	3.475771	-2.183853	-1.665068
C	1.464814	-2.430152	-3.113379
C	-1.700735	0.757060	0.176718
C	3.184633	0.415257	0.335233
C	3.886449	1.434016	-0.338705
C	5.185728	1.714876	0.085910
C	5.750354	1.051474	1.163839
C	5.011561	0.105850	1.857497
C	3.715498	-0.235022	1.466892
C	2.934286	-1.233945	2.311780
C	3.693683	-2.552304	2.531725
C	2.534586	-0.609931	3.662149
C	3.282612	2.287085	-1.449404
C	3.183976	3.760399	-1.012340
C	4.052753	2.159597	-2.773580
C	-2.335300	1.739003	-0.832520
C	-2.523721	-0.479959	0.504568
C	-2.622653	-0.889957	1.837981
C	-3.341932	-2.032486	2.159017
C	-3.966664	-2.789569	1.170955
C	-3.870281	-2.382613	-0.160510
C	-3.151183	-1.227097	-0.485200
C	-3.549633	2.444592	-0.269044
C	-3.389058	3.516444	0.614366
C	-4.493231	4.170488	1.150124
C	-5.779687	3.763253	0.806454
C	-5.951589	2.702192	-0.076517
C	-4.843983	2.048827	-0.609951
O	-4.437569	-3.033276	-1.211703
C	-5.196394	-4.217509	-0.958253
H	-0.331925	0.232075	-2.379946
H	-0.586668	-1.295561	-1.527947
H	1.994854	0.003559	-2.234411
H	0.783262	1.104256	1.342444
H	0.233569	-3.039146	-0.703953
H	1.644360	-2.835230	0.336717
H	1.644821	-4.058292	-0.924096
H	3.892940	-1.981513	-0.680470
H	3.940574	-1.497614	-2.376368
H	3.765690	-3.197988	-1.945853
H	0.377432	-2.418023	-3.207351
H	1.879894	-1.750081	-3.860735
H	1.800346	-3.438507	-3.359411
H	-1.539549	1.298449	1.108975
H	5.756300	2.481622	-0.422062
H	6.759574	1.287579	1.477596
H	5.448674	-0.377805	2.721136
H	2.011736	-1.477068	1.786788
H	3.056212	-3.263878	3.060414
H	3.996921	-3.007346	1.587708
H	4.590496	-2.405167	3.135904
H	1.936551	-1.316285	4.241778
H	1.947458	0.301412	3.529681
H	3.415783	-0.351123	4.252374
H	2.260820	1.954896	-1.630591
H	2.678697	4.347102	-1.782428
H	2.621508	3.861121	-0.082111
H	4.171395	4.197761	-0.854649
H	3.560072	2.743074	-3.554036
H	4.111255	1.122857	-3.109544
H	5.073497	2.533384	-2.675704
H	-2.600528	1.219490	-1.753900
H	-1.574442	2.478595	-1.091880
H	-2.148781	-0.309926	2.620604
H	-3.425597	-2.343183	3.192983
H	-4.522459	-3.673810	1.446785
H	-3.118966	-0.937301	-1.527246
H	-2.391340	3.852579	0.877037
H	-4.349814	5.002854	1.828248
H	-6.640856	4.273567	1.219579
H	-6.948642	2.383123	-0.354438
H	-4.990594	1.225991	-1.299210
H	-5.546773	-4.556313	-1.929615
H	-4.576639	-4.994628	-0.504117
H	-6.054617	-4.007527	-0.315376

B3LYP energy = -1507.78700092 a.u.

(5*S*,1'*S*)-**9a**, Conf. H

C	0.240826	-2.279498	-0.589420
C	1.747980	-1.993094	-0.327972
N	1.721119	-0.506147	-0.047499

C	0.477585	-0.064223	-0.116262
N	-0.421935	-0.978195	-0.374125
C	2.400938	-2.918134	0.739166
C	2.286044	-4.358598	0.194655
C	1.708391	-2.836762	2.107388
C	3.897203	-2.593431	0.888009
C	-1.894565	-0.807093	-0.510352
C	2.864510	0.392332	-0.056876
C	3.257114	1.030416	1.136480
C	4.393734	1.839827	1.090981
C	5.106595	2.019760	-0.083761
C	4.666243	1.427414	-1.256879
C	3.527538	0.621058	-1.278990
C	3.028590	0.102223	-2.623511
C	2.628463	1.270761	-3.543044
C	4.048954	-0.814768	-3.317853
C	2.480302	0.935229	2.444163
C	3.355777	0.482516	3.624255
C	1.793159	2.275314	2.769092
C	-2.619171	-1.482985	0.677114
C	-2.261388	0.646550	-0.747676
C	-2.496983	1.082857	-2.055306
C	-2.822349	2.411475	-2.291584
C	-2.914023	3.326716	-1.246224
C	-2.677843	2.894709	0.060012
C	-2.353821	1.555086	0.300817
C	-4.104228	-1.646504	0.441297
C	-4.573600	-2.639055	-0.424325
C	-5.935855	-2.799730	-0.653637
C	-6.853509	-1.970110	-0.014535
C	-6.398575	-0.983349	0.854034
C	-5.033924	-0.823241	1.078633
O	-2.739309	3.689262	1.161116
C	-3.089808	5.065719	0.998000
H	0.047994	-2.615940	-1.608253
H	-0.161568	-3.018444	0.098624
H	2.314366	-2.130661	-1.247271
H	0.226273	0.977731	0.010647
H	2.753086	-4.452715	-0.788684
H	1.251409	-4.697126	0.114202
H	2.798173	-5.043663	0.871843
H	0.649319	-3.098131	2.058887
H	1.790964	-1.844720	2.548885
H	2.179229	-3.540466	2.795797
H	4.414795	-2.659075	-0.071570
H	4.071379	-1.599570	1.295103
H	4.360582	-3.315006	1.563208
H	-2.148811	-1.361075	-1.416206
H	4.724038	2.340683	1.991628
H	5.992437	2.642673	-0.090132
H	5.203686	1.613646	-2.177846
H	2.121995	-0.479383	-2.460420
H	2.203312	0.887187	-4.472954
H	1.884597	1.912642	-3.067048
H	3.489967	1.889401	-3.800399
H	3.633367	-1.200850	-4.250964

H	4.324490	-1.665661	-2.691996
H	4.965288	-0.274716	-3.562911
H	1.693048	0.192492	2.321054
H	2.739929	0.354981	4.516898
H	3.854248	-0.466010	3.419805
H	4.124526	1.220540	3.859010
H	1.183805	2.177425	3.670012
H	1.145325	2.609364	1.956026
H	2.529888	3.061087	2.947407
H	-2.437829	-0.913536	1.589197
H	-2.176218	-2.469264	0.830338
H	-2.439363	0.384048	-2.880552
H	-3.014268	2.746377	-3.303265
H	-3.173697	4.353982	-1.456779
H	-2.188299	1.256669	1.327866
H	-3.869715	-3.301838	-0.916427
H	-6.281199	-3.577125	-1.324210
H	-7.914898	-2.096085	-0.188627
H	-7.105518	-0.337653	1.360440
H	-4.691333	-0.052600	1.758787
H	-3.087476	5.490227	1.998299
H	-2.357582	5.589869	0.379035
H	-4.085400	5.170281	0.560637

B3LYP energy = -1507.78691571 a.u.

(5*S*,1’*S*)-**9a**, Conf. I

C	-0.108202	0.958437	1.456593
C	-1.659931	0.979436	1.351968
N	-1.890534	0.027155	0.197327
C	-0.732074	-0.453473	-0.218935
N	0.313059	-0.026583	0.441365
C	-2.398330	0.686409	2.690419
C	-1.983888	1.809660	3.665719
C	-2.025797	-0.673880	3.297884
C	-3.921926	0.772603	2.497109
C	1.734741	-0.394461	0.262715
C	-3.118789	-0.141370	-0.561312
C	-3.793873	-1.378052	-0.523353
C	-4.990836	-1.481681	-1.234408
C	-5.499897	-0.411607	-1.952886
C	-4.793291	0.779375	-2.012043
C	-3.579253	0.936934	-1.342853
C	-2.800225	2.233279	-1.540038
C	-2.442011	2.444158	-3.022343
C	-3.549667	3.455226	-0.982783
C	-3.269857	-2.610225	0.205585
C	-4.295411	-3.195685	1.190271
C	-2.812484	-3.688626	-0.794619
C	1.883066	-1.645950	-0.628325
C	2.531432	0.804363	-0.232428
C	2.199050	1.440088	-1.425215
C	2.962273	2.520323	-1.864878
C	4.048513	2.965801	-1.129381
C	4.387791	2.327318	0.067537
C	3.622655	1.248095	0.516559

C	3.291185	-2.198954	-0.621173
C	3.728615	-2.995247	0.440798
C	5.022741	-3.504889	0.461516
C	5.898720	-3.226676	-0.584399
C	5.471516	-2.438907	-1.648677
C	4.176315	-1.929324	-1.665886
O	5.467670	2.826565	0.724746
C	5.887191	2.203066	1.939080
H	0.340296	1.925109	1.231493
H	0.242073	0.633760	2.434510
H	-1.991201	1.962018	1.022538
H	-0.665420	-1.136124	-1.052390
H	-2.219007	2.796810	3.260925
H	-0.919666	1.785416	3.906823
H	-2.531080	1.695793	4.602619
H	-0.954278	-0.770139	3.485417
H	-2.334310	-1.502565	2.662625
H	-2.529691	-0.794450	4.258346
H	-4.214650	1.732889	2.066881
H	-4.307141	-0.015636	1.853317
H	-4.416667	0.681085	3.465671
H	2.089750	-0.650734	1.263387
H	-5.532769	-2.418274	-1.224160
H	-6.437989	-0.512456	-2.484345
H	-5.179744	1.596415	-2.607472
H	-1.853067	2.154321	-1.007050
H	-1.814164	3.330880	-3.131771
H	-1.896862	1.588943	-3.426468
H	-3.333922	2.591629	-3.633977
H	-2.942034	4.355150	-1.097438
H	-3.787585	3.339473	0.076259
H	-4.488670	3.618213	-1.515284
H	-2.396277	-2.317821	0.786755
H	-3.845986	-4.019298	1.748772
H	-4.640496	-2.449495	1.907176
H	-5.169980	-3.590394	0.670761
H	-2.399636	-4.546818	-0.260243
H	-2.047147	-3.315278	-1.478263
H	-3.649171	-4.042244	-1.400130
H	1.592609	-1.401826	-1.651714
H	1.199107	-2.415970	-0.262171
H	1.358173	1.100517	-2.018053
H	2.707961	3.017414	-2.792764
H	4.648091	3.802226	-1.464366
H	3.872392	0.742515	1.438694
H	3.049438	-3.229451	1.253620
H	5.344020	-4.125697	1.288882
H	6.905279	-3.625906	-0.572162
H	6.145060	-2.222068	-2.468650
H	3.852140	-1.318172	-2.499769
H	6.761011	2.758651	2.268772
H	5.109798	2.263205	2.705319
H	6.161996	1.158463	1.773365

B3LYP energy = -1507.78690449 a.u.

(5*S*,1'*S*)-**9a**, Conf. J

C	0.141423	-0.330014	1.778674
C	1.680732	-0.152316	1.639262
N	1.845295	-0.136637	0.132578
C	0.676486	-0.377150	-0.434156
N	-0.329072	-0.507963	0.391821
C	2.259006	1.035635	2.458647
C	1.652777	2.392375	2.072626
C	3.790260	1.089308	2.321273
C	1.925767	0.735461	3.936807
C	-1.746316	-0.701094	-0.008951
C	3.092317	-0.252976	-0.607421
C	3.793005	-1.474178	-0.560955
C	5.015875	-1.545308	-1.229419
C	5.504760	-0.468849	-1.952582
C	4.762664	0.698308	-2.040978
C	3.541852	0.837249	-1.378320
C	2.745430	2.122013	-1.572036
C	3.563043	3.381156	-1.240694
C	2.183915	2.209471	-3.003777
C	3.261692	-2.732104	0.118857
C	3.002847	-3.844947	-0.913634
C	4.190500	-3.227799	1.239154
C	-2.279507	-2.030970	0.568685
C	-2.574553	0.531039	0.321984
C	-3.031373	0.794799	1.618261
C	-3.762759	1.947651	1.867475
C	-4.059664	2.849470	0.848196
C	-3.612518	2.584210	-0.446647
C	-2.868602	1.426108	-0.698412
C	-3.612035	-2.434436	-0.023954
C	-3.680087	-2.944009	-1.324005
C	-4.898723	-3.316658	-1.880808
C	-6.072038	-3.188733	-1.141929
C	-6.015964	-2.688821	0.154834
C	-4.794448	-2.314359	0.707932
O	-3.845192	3.379943	-1.523970
C	-4.616477	4.569538	-1.344488
H	-0.121699	-1.198609	2.378867
H	-0.339489	0.547151	2.205493
H	2.184817	-1.050237	1.996031
H	0.566383	-0.471640	-1.505522
H	0.566511	2.415599	2.177344
H	1.898388	2.674012	1.050513
H	2.054103	3.165892	2.729658
H	4.109467	1.333040	1.309699
H	4.247609	0.137224	2.599304
H	4.188730	1.855206	2.989067
H	0.851640	0.741647	4.131829
H	2.325121	-0.232548	4.248875
H	2.374724	1.500386	4.571943
H	-1.714810	-0.798261	-1.094003
H	5.583132	-2.466429	-1.199911
H	6.454866	-0.547080	-2.466282
H	5.136959	1.518388	-2.639511
H	1.892176	2.099417	-0.895518

H	2.926611	4.265868	-1.306914
H	3.982068	3.340604	-0.234209
H	4.388679	3.518529	-1.940953
H	1.571941	3.107289	-3.113270
H	1.563674	1.345472	-3.250844
H	2.988547	2.260148	-3.739979
H	2.296081	-2.506473	0.569713
H	2.560002	-4.714801	-0.423970
H	2.318632	-3.508768	-1.695505
H	3.928085	-4.168067	-1.393861
H	3.752476	-4.096382	1.735155
H	4.362421	-2.456807	1.992481
H	5.163385	-3.527380	0.845024
H	-2.365173	-1.964266	1.653787
H	-1.536139	-2.803289	0.354763
H	-2.835095	0.105527	2.428305
H	-4.117803	2.151653	2.870000
H	-4.637720	3.734605	1.070203
H	-2.537770	1.242084	-1.713494
H	-2.771948	-3.061516	-1.905809
H	-4.931713	-3.713090	-2.888260
H	-7.021414	-3.481493	-1.572849
H	-6.922717	-2.591257	0.739153
H	-4.762497	-1.929508	1.720457
H	-4.679158	5.030963	-2.326380
H	-5.622538	4.338192	-0.986437
H	-4.127288	5.257259	-0.650228

B3LYP energy = -1507.78685743 a.u.

(5*S*,1'*S*)-**9a**, Conf. K

C	-0.231564	-1.815482	1.351283
C	-1.757547	-1.616376	1.118768
N	-1.759467	-0.424605	0.186359
C	-0.515580	-0.070520	-0.083481
N	0.405807	-0.814144	0.473286
C	-2.511011	-2.897882	0.657732
C	-1.939835	-3.500020	-0.633954
C	-4.010222	-2.602756	0.481917
C	-2.363761	-3.921767	1.804498
C	1.883872	-0.653875	0.392158
C	-2.892099	0.431505	-0.127745
C	-3.434883	1.232182	0.896740
C	-4.557511	2.002466	0.589715
C	-5.093138	2.018606	-0.688464
C	-4.497380	1.277409	-1.696679
C	-3.386552	0.470029	-1.446737
C	-2.742458	-0.270003	-2.612985
C	-3.744374	-1.127068	-3.402747
C	-2.017204	0.714213	-3.550419
C	-2.824733	1.358463	2.288872
C	-2.287913	2.782876	2.521882
C	-3.806431	0.953466	3.400608
C	2.517255	-1.873913	-0.315996
C	2.257747	0.690897	-0.204342
C	2.234074	0.923254	-1.585216
C	2.566282	2.178979	-2.074849
C	2.930595	3.215528	-1.218569
C	2.958439	2.984877	0.157538
C	2.616130	1.721926	0.654538
C	4.018027	-1.937948	-0.135244
C	4.885973	-1.637184	-1.185960
C	6.265775	-1.696564	-1.009564
C	6.797801	-2.056987	0.224055
C	5.941988	-2.363297	1.278913
C	4.564324	-2.306373	1.098003
O	3.300740	3.910531	1.090513
C	3.694109	5.213450	0.653365
H	0.062714	-1.623912	2.383786
H	0.098748	-2.814894	1.081242
H	-2.230604	-1.295089	2.044489
H	-0.276975	0.779948	-0.704498
H	-0.882747	-3.757279	-0.543291
H	-2.052884	-2.829502	-1.484553
H	-2.473417	-4.421513	-0.872917
H	-4.205304	-1.913914	-0.337352
H	-4.440677	-2.175254	1.390213
H	-4.542243	-3.531474	0.268205
H	-1.328201	-4.227563	1.964941
H	-2.752204	-3.525252	2.745612
H	-2.932471	-4.820880	1.563288
H	2.216670	-0.661586	1.432124
H	-5.005445	2.617982	1.359136
H	-5.962703	2.626952	-0.904144
H	-4.901500	1.327501	-2.699199
H	-1.987832	-0.944518	-2.211420
H	-3.220027	-1.697989	-4.171823
H	-4.269673	-1.832998	-2.757933
H	-4.492371	-0.511557	-3.904820
H	-1.511961	0.170214	-4.351326
H	-1.269247	1.305452	-3.017942
H	-2.720843	1.410698	-4.010767
H	-1.962621	0.696210	2.356630
H	-1.792584	2.841674	3.493424
H	-1.565180	3.065433	1.753889
H	-3.092952	3.519793	2.512776
H	-3.311985	0.997638	4.373150
H	-4.185725	-0.060117	3.257565
H	-4.665749	1.625624	3.437032
H	2.262252	-1.855482	-1.375659
H	2.072224	-2.781792	0.096491
H	1.966603	0.136570	-2.278473
H	2.550800	2.359593	-3.142412
H	3.191817	4.180108	-1.628895
H	2.650285	1.566471	1.725949
H	4.482695	-1.356057	-2.151590
H	6.923893	-1.462471	-1.837376
H	7.870847	-2.103582	0.362415
H	6.347497	-2.653815	2.240377
H	3.908540	-2.561869	1.923661
H	3.932122	5.767787	1.557152
H	4.577807	5.163980	0.012803

H	2.881158	5.715063	0.122629

B3LYP energy = -1507.78678165 a.u.

(5*S*,1’*S*)-**9a**, Conf. L

C	0.085475	-1.310579	-1.383220
C	1.357011	-0.412575	-1.406887
N	1.154594	0.420982	-0.157180
C	-0.031231	0.150984	0.360081
N	-0.700606	-0.796165	-0.243789
C	2.690915	-1.193923	-1.571254
C	3.881165	-0.222761	-1.658757
C	2.586692	-1.935393	-2.922425
C	2.937303	-2.217261	-0.454904
C	-2.069496	-1.277053	0.099134
C	1.895239	1.617104	0.213022
C	1.748263	2.774814	-0.576176
C	2.521540	3.887946	-0.243345
C	3.368352	3.874237	0.853873
C	3.437519	2.748500	1.659465
C	2.705901	1.596912	1.364649
C	2.771742	0.419216	2.328815
C	4.211110	-0.047249	2.601512
C	2.055901	0.757529	3.650191
C	0.749125	2.896662	-1.721517
C	-0.323128	3.953776	-1.398668
C	1.428689	3.199190	-3.066600
C	-2.042654	-2.476584	1.070185
C	-2.939544	-0.118524	0.567033
C	-3.078304	0.217097	1.917903
C	-3.872983	1.298659	2.277859
C	-4.539844	2.056598	1.319821
C	-4.406097	1.720330	-0.029166
C	-3.605141	0.634086	-0.394521
C	-1.415694	-3.737358	0.516233
C	-0.239089	-4.251144	1.064661
C	0.328798	-5.422435	0.568269
C	-0.274917	-6.097446	-0.487095
C	-1.453396	-5.598954	-1.038112
C	-2.019056	-4.431616	-0.537884
O	-5.010834	2.377141	-1.053369
C	-5.874413	3.475800	-0.751570
H	-0.500229	-1.226031	-2.297473
H	0.318117	-2.359358	-1.216286
H	1.300466	0.278616	-2.247537
H	-0.429685	0.694507	1.204537
H	4.044622	0.321186	-0.730284
H	3.743417	0.507649	-2.459062
H	4.790799	-0.784401	-1.878633
H	2.397959	-1.242330	-3.745639
H	1.800162	-2.692170	-2.924836
H	3.528213	-2.446319	-3.128071
H	3.060480	-1.740574	0.515907
H	2.134710	-2.951502	-0.374222
H	3.855314	-2.768210	-0.666739
H	-2.472384	-1.621369	-0.853328
H	2.441802	4.787022	-0.840822
H	3.957453	4.750257	1.095092
H	4.070641	2.763944	2.536822
H	2.238472	-0.416775	1.878224
H	4.200558	-0.943794	3.224584
H	4.742833	-0.284181	1.678887
H	4.786351	0.713344	3.132073
H	2.052823	-0.111433	4.311725
H	1.020714	1.062480	3.484344
H	2.560055	1.573640	4.171417
H	0.220856	1.950239	-1.826580
H	-1.079204	3.972487	-2.186177
H	-0.822045	3.736339	-0.452590
H	0.109898	4.953118	-1.327735
H	0.684988	3.229760	-3.865374
H	2.172299	2.443000	-3.324440
H	1.933475	4.166604	-3.046827
H	-3.082871	-2.677539	1.336967
H	-1.531055	-2.193614	1.991148
H	-2.589043	-0.360763	2.689853
H	-3.984114	1.556246	3.323660
H	-5.155328	2.888037	1.630624
H	-3.526996	0.387147	-1.446505
H	0.234005	-3.736014	1.893179
H	1.239047	-5.808307	1.010390
H	0.163077	-7.009691	-0.872263
H	-1.937561	-6.126070	-1.850922
H	-2.947146	-4.066750	-0.963994
H	-6.243784	3.831095	-1.709672
H	-6.716444	3.157391	-0.132603
H	-5.331475	4.280749	-0.250713

B3LYP energy = -1507.78639136 a.u.

(5*S*,1’*S*)-**9a**, Conf. M

C	-0.126393	0.258825	1.923707
C	-1.662110	0.281526	1.679015
N	-1.734496	-0.162508	0.233029
C	-0.521381	-0.453638	-0.202901
N	0.435625	-0.289193	0.673744
C	-2.491357	-0.525036	2.721056
C	-2.243264	0.149375	4.087779
C	-2.076985	-2.001568	2.803064
C	-3.995234	-0.420980	2.415137
C	1.879340	-0.593083	0.547722
C	-2.880818	-0.062763	-0.654567
C	-3.349360	1.218514	-1.007089
C	-4.496044	1.293689	-1.798636
C	-5.127145	0.150947	-2.263911
C	-4.604431	-1.096898	-1.964252
C	-3.473498	-1.238531	-1.158416
C	-2.919747	-2.638801	-0.921469
C	-3.969724	-3.605450	-0.349981
C	-2.311792	-3.210565	-2.216419
C	-2.638219	2.514438	-0.631750
C	-2.143315	3.251626	-1.889933

C	-3.514652	3.439083	0.229244
C	2.179461	-1.373080	-0.749362
C	2.705048	0.674414	0.715090
C	3.651669	0.744212	1.729014
C	4.429152	1.894795	1.873714
C	4.263297	2.968139	1.018579
C	3.309841	2.902688	-0.005764
C	2.530505	1.756514	-0.155702
C	3.595095	-1.905525	-0.792212
C	3.940374	-3.047432	-0.064392
C	5.238683	-3.545260	-0.096287
C	6.211606	-2.908425	-0.862154
C	5.876914	-1.774103	-1.594669
C	4.577122	-1.277260	-1.558911
O	3.220607	4.004279	-0.799230
C	2.307343	3.996795	-1.894750
H	0.285257	1.251589	2.098431
H	0.158612	-0.384274	2.753508
H	-2.023495	1.307038	1.716680
H	-0.346361	-0.790530	-1.212588
H	-2.528028	1.203789	4.068510
H	-1.202051	0.083439	4.408456
H	-2.848642	-0.343816	4.849638
H	-2.666164	-2.501918	3.573422
H	-1.026657	-2.128164	3.073241
H	-2.252216	-2.529744	1.866873
H	-4.265719	-0.901497	1.476980
H	-4.561897	-0.906313	3.211767
H	-4.320210	0.620495	2.367424
H	2.104864	-1.247964	1.392498
H	-4.888350	2.264085	-2.073921
H	-6.014690	0.233655	-2.878781
H	-5.083936	-1.981069	-2.363386
H	-2.115076	-2.570263	-0.190347
H	-3.504375	-4.566298	-0.121057
H	-4.418664	-3.220607	0.566652
H	-4.773653	-3.792181	-1.063759
H	-1.861776	-4.186655	-2.023664
H	-1.540483	-2.556103	-2.628609
H	-3.076351	-3.340119	-2.984841
H	-1.748367	2.269416	-0.052985
H	-1.572766	4.137843	-1.603980
H	-1.500858	2.613261	-2.499506
H	-2.975113	3.580817	-2.515048
H	-2.951427	4.328264	0.519656
H	-3.858435	2.943537	1.138880
H	-4.397537	3.771474	-0.319802
H	2.006179	-0.732470	-1.616565
H	1.486500	-2.216459	-0.815188
H	3.792306	-0.093171	2.400845
H	5.169416	1.948834	2.661999
H	4.859342	3.865877	1.119016
H	1.794740	1.701173	-0.945804
H	3.187242	-3.559433	0.525040
H	5.488673	-4.433988	0.470126
H	7.221971	-3.297011	-0.891025
H	6.626415	-1.275570	-2.196921
H	4.325597	-0.393433	-2.132985
H	2.544986	3.199890	-2.604467
H	1.274825	3.888791	-1.551859
H	2.423708	4.960351	-2.383691

B3LYP energy = -1507.78625286 a.u.

(5*S*,1’*S*)-**9a**, Conf. N

C	-0.467994	1.193278	-1.711894
C	-1.584777	0.132745	-1.482278
N	-1.075603	-0.567506	-0.237321
C	0.121819	-0.099907	0.067963
N	0.549247	0.874816	-0.691176
C	-3.029095	0.708311	-1.474709
C	-4.062499	-0.421872	-1.325033
C	-3.235157	1.355277	-2.861958
C	-3.258391	1.766323	-0.387821
C	1.866283	1.565087	-0.590373
C	-1.585256	-1.802265	0.336930
C	-1.428256	-3.002049	-0.384727
C	-1.981942	-4.161313	0.160257
C	-2.623709	-4.145942	1.388557
C	-2.701283	-2.966755	2.112609
C	-2.184524	-1.770771	1.611565
C	-2.234926	-0.526283	2.488982
C	-3.648658	-0.221421	3.009746
C	-1.244523	-0.651927	3.662170
C	-0.626790	-3.118856	-1.677674
C	0.627945	-3.984775	-1.459066
C	-1.465035	-3.658473	-2.848181
C	1.904366	2.562398	0.583850
C	2.999757	0.547856	-0.597081
C	3.470822	0.072631	-1.826966
C	4.485449	-0.872340	-1.858975
C	5.050221	-1.362288	-0.682854
C	4.586320	-0.886443	0.543582
C	3.562537	0.068885	0.579232
C	0.904693	3.691963	0.475600
C	-0.186724	3.770549	1.342133
C	-1.097697	4.820531	1.250186
C	-0.929805	5.807233	0.284960
C	0.157162	5.741775	-0.583967
C	1.067026	4.695090	-0.486012
O	5.060912	-1.279166	1.755277
C	6.130709	-2.226065	1.800044
H	-0.027694	1.119960	-2.705256
H	-0.818708	2.212609	-1.560566
H	-1.552583	-0.605037	-2.283658
H	0.710768	-0.516837	0.872160
H	-4.007195	-0.911128	-0.354258
H	-3.934942	-1.184503	-2.096143
H	-5.067669	-0.010059	-1.432613
H	-3.073564	0.634451	-3.667155
H	-2.574313	2.208197	-3.026637
H	-4.260450	1.718879	-2.943516

H	-3.169491	1.348736	0.613357
H	-2.568749	2.607456	-0.465957
H	-4.268342	2.168663	-0.484441
H	1.933274	2.134465	-1.516726
H	-1.887832	-5.096519	-0.376183
H	-3.043319	-5.058902	1.792523
H	-3.168239	-2.974493	3.088719
H	-1.918749	0.326978	1.889806
H	-3.649405	0.725711	3.553020
H	-4.372496	-0.145667	2.196977
H	-3.998815	-0.992660	3.697757
H	-1.232181	0.269202	4.248842
H	-0.227717	-0.849364	3.315484
H	-1.528622	-1.469445	4.327864
H	-0.268828	-2.129968	-1.961598
H	1.231735	-4.007313	-2.368713
H	1.246136	-3.590537	-0.650421
H	0.362952	-5.013477	-1.208060
H	-0.866340	-3.675307	-3.761171
H	-2.347898	-3.043983	-3.032810
H	-1.805408	-4.677989	-2.658228
H	2.917922	2.968604	0.614627
H	1.752615	2.029946	1.524201
H	3.051931	0.449599	-2.752371
H	4.854533	-1.233933	-2.810728
H	5.843180	-2.094071	-0.734756
H	3.247544	0.425861	1.550861
H	-0.321374	3.010791	2.104051
H	-1.934707	4.868473	1.935937
H	-1.634630	6.626316	0.214460
H	0.302594	6.513344	-1.330096
H	1.919934	4.667698	-1.155523
H	6.350330	-2.372526	2.854164
H	5.834519	-3.178789	1.354428
H	7.019074	-1.842393	1.292833

B3LYP energy = -1507.78624517 a.u.

(5*S*,1'*S*)-**9a**, Conf. O

C	-0.879075	-1.199075	-1.199366
C	0.650094	-1.491603	-1.219264
N	1.146158	-0.551463	-0.141073
C	0.137679	0.176841	0.305417
N	-1.030169	-0.114769	-0.205885
C	1.016720	-2.998385	-1.109411
C	0.493830	-3.659023	0.173604
C	2.539733	-3.190535	-1.212518
C	0.371587	-3.685155	-2.332986
C	-2.323869	0.572104	0.068392
C	2.529014	-0.174164	0.106879
C	3.205826	0.586236	-0.867242
C	4.552382	0.875211	-0.641979
C	5.191469	0.470650	0.519595
C	4.483145	-0.210329	1.497022
C	3.139615	-0.545652	1.320682
C	2.397917	-1.229765	2.461741
C	3.095839	-2.512034	2.942704
C	2.189992	-0.257250	3.637910
C	2.532779	1.167220	-2.106486
C	2.509294	2.705596	-2.042136
C	3.184921	0.683171	-3.411648
C	-3.230509	-0.243026	1.017300
C	-2.084625	1.998724	0.538962
C	-1.887682	2.315325	1.888122
C	-1.660831	3.635088	2.256730
C	-1.628863	4.654818	1.309200
C	-1.829571	4.341644	-0.036511
C	-2.054666	3.012486	-0.411294
C	-3.769360	-1.540734	0.455676
C	-3.456470	-2.761940	1.055320
C	-3.969753	-3.956719	0.555650
C	-4.807234	-3.945560	-0.554538
C	-5.135059	-2.732555	-1.156020
C	-4.623374	-1.541845	-0.652238
O	-1.828346	5.246496	-1.049551
C	-1.628691	6.627635	-0.738582
H	-1.251355	-0.857217	-2.164213
H	-1.464354	-2.060367	-0.888770
H	1.077404	-1.140618	-2.158069
H	0.275421	0.973454	1.021797
H	-0.590067	-3.580479	0.271686
H	0.948758	-3.233973	1.066771
H	0.737643	-4.722735	0.158039
H	3.072683	-2.761125	-0.366197
H	2.935957	-2.740228	-2.125313
H	2.770549	-4.256992	-1.243091
H	-0.718800	-3.640627	-2.311205
H	0.714287	-3.235678	-3.268018
H	0.653320	-4.739004	-2.347986
H	-2.808683	0.626553	-0.907564
H	5.103161	1.445141	-1.379125
H	6.236950	0.707328	0.673623
H	4.981747	-0.485148	2.417226
H	1.409409	-1.511113	2.101797
H	2.482126	-3.009864	3.696305
H	3.263001	-3.214253	2.124759
H	4.062901	-2.295138	3.399743
H	1.601944	-0.736175	4.423872
H	1.667219	0.649231	3.326400
H	3.144803	0.046046	4.071915
H	1.491061	0.849293	-2.121877
H	1.968037	3.108376	-2.900868
H	2.015038	3.056248	-1.134097
H	3.518110	3.121723	-2.057704
H	2.642880	1.079240	-4.272767
H	3.187656	-0.405959	-3.483463
H	4.219631	1.022590	-3.488067
H	-4.068087	0.410767	1.272573
H	-2.694037	-0.443370	1.945929
H	-1.917526	1.547943	2.649817
H	-1.511899	3.881618	3.300524
H	-1.456632	5.672918	1.626394

H	-2.216170	2.795843	-1.460450
H	-2.813118	-2.779326	1.927613
H	-3.718251	-4.893541	1.037483
H	-5.209840	-4.872522	-0.943432
H	-5.797619	-2.713365	-2.012649
H	-4.902950	-0.604465	-1.120260
H	-1.679534	7.153077	-1.688424
H	-2.412008	6.999905	-0.074159
H	-0.649171	6.793234	-0.283507

B3LYP energy = -1507.78623115 a.u.

(5*S*,1’*S*)-**9a**, Conf. P

C	0.840073	1.481728	-1.173662
C	-0.668450	1.808083	-0.970629
N	-1.120989	0.618064	-0.150487
C	-0.120659	-0.236783	-0.028208
N	1.012614	0.151116	-0.553558
C	-0.946272	3.235013	-0.418142
C	-0.283805	3.498073	0.940276
C	-2.460391	3.488878	-0.321388
C	-0.369286	4.217493	-1.460755
C	2.284297	-0.624570	-0.621653
C	-2.494533	0.223977	0.124228
C	-3.294681	-0.226966	-0.944194
C	-4.625448	-0.542968	-0.667071
C	-5.132327	-0.463376	0.620608
C	-4.304683	-0.087805	1.666920
C	-2.970281	0.259715	1.449621
C	-2.095624	0.593221	2.651290
C	-2.683398	1.718565	3.518435
C	-1.835819	-0.663501	3.503401
C	-2.771571	-0.458789	-2.358173
C	-2.809516	-1.957189	-2.712659
C	-3.527883	0.371017	-3.408708
C	3.294576	-0.200350	0.467392
C	2.003497	-2.119877	-0.650685
C	1.815215	-2.747302	-1.887666
C	1.556194	-4.109387	-1.938577
C	1.476472	-4.871213	-0.774890
C	1.664045	-4.248828	0.459782
C	1.928954	-2.874874	0.514053
C	3.831462	1.208531	0.341066
C	3.572349	2.164058	1.325067
C	4.081252	3.456546	1.218852
C	4.861355	3.811283	0.123567
C	5.136145	2.863713	-0.859908
C	4.628476	1.574004	-0.749119
O	1.612461	-4.881337	1.661253
C	1.365727	-6.289187	1.687598
H	1.117856	1.430848	-2.225430
H	1.491524	2.196630	-0.678519
H	-1.190178	1.742353	-1.925069
H	-0.240404	-1.197449	0.451015
H	0.800703	3.385676	0.903861
H	-0.668264	2.839353	1.716897
H	-0.489524	4.523912	1.250876
H	-2.944431	2.855531	0.419786
H	-2.952800	3.321353	-1.281721
H	-2.636813	4.527692	-0.036268
H	0.715925	4.141874	-1.550895
H	-0.807930	4.054728	-2.448209
H	-0.599262	5.240900	-1.160700
H	2.700438	-0.357042	-1.594136
H	-5.268911	-0.879045	-1.469840
H	-6.167590	-0.716984	0.812190
H	-4.699773	-0.068050	2.674270
H	-1.129441	0.936042	2.283267
H	-1.984681	1.978043	4.316432
H	-2.880111	2.619230	2.934967
H	-3.619885	1.414449	3.989080
H	-1.158051	-0.427837	4.326755
H	-1.389109	-1.467781	2.915381
H	-2.763930	-1.045885	3.932884
H	-1.723588	-0.164053	-2.399698
H	-2.374134	-2.119535	-3.701036
H	-2.244892	-2.551099	-1.991184
H	-3.832787	-2.336504	-2.732060
H	-3.080582	0.227809	-4.394471
H	-3.505614	1.437355	-3.176735
H	-4.574680	0.068937	-3.474393
H	4.125078	-0.907418	0.401639
H	2.847697	-0.332754	1.453864
H	1.885427	-2.175199	-2.804674
H	1.420709	-4.595629	-2.896594
H	1.278978	-5.931035	-0.842628
H	2.084443	-2.433899	1.489768
H	2.974414	1.894523	2.188443
H	3.871719	4.182368	1.994998
H	5.260700	4.814340	0.040327
H	5.755535	3.126756	-1.708627
H	4.868370	0.841955	-1.512687
H	1.370799	-6.569107	2.737583
H	0.392520	-6.528267	1.251936
H	2.150700	-6.837264	1.161236

B3LYP energy = -1507.78621087 a.u.

(5*S*,1’*S*)-**9a**, Conf. Q

C	0.149950	-1.584497	-1.509406
C	1.684480	-1.443796	-1.293041
N	1.732705	-0.401141	-0.198699
C	0.503242	-0.056939	0.141771
N	-0.446634	-0.687319	-0.500113
C	2.423620	-2.790744	-1.046516
C	2.206547	-3.642809	-2.316133
C	1.892042	-3.555407	0.174577
C	3.936663	-2.555764	-0.895776
C	-1.919703	-0.515921	-0.359381
C	2.892480	0.380660	0.197214
C	3.424414	0.223913	1.492360
C	4.566730	0.958825	1.814036

C	5.156045	1.816167	0.898483
C	4.583165	1.992162	-0.351134
C	3.429232	1.301707	-0.724066
C	2.787434	1.632996	-2.068196
C	2.350965	3.108565	-2.119588
C	3.705678	1.294093	-3.254065
C	2.787708	-0.647265	2.568553
C	3.778333	-1.643842	3.192078
C	2.142190	0.223951	3.662624
C	-2.554599	-1.797251	0.229373
C	-2.254935	0.764122	0.385341
C	-2.204271	0.845575	1.774879
C	-2.496705	2.054462	2.407062
C	-2.845985	3.172816	1.671000
C	-2.906891	3.096205	0.275081
C	-2.604868	1.892487	-0.363995
C	-4.061474	-1.817174	0.094080
C	-4.653228	-2.061111	-1.149011
C	-6.036678	-2.076134	-1.289939
C	-6.853023	-1.851452	-0.184544
C	-6.275811	-1.614790	1.058715
C	-4.890417	-1.597399	1.195353
O	-3.268360	4.236688	-0.367607
C	-3.391005	4.219993	-1.790353
H	-0.164174	-1.265545	-2.503574
H	-0.194870	-2.602510	-1.350290
H	2.140893	-0.996745	-2.174143
H	0.298093	0.702357	0.881465
H	2.564582	-3.125362	-3.209356
H	1.159701	-3.909196	-2.473779
H	2.766431	-4.574833	-2.226093
H	0.821776	-3.762058	0.108867
H	2.077003	-3.018997	1.104018
H	2.398654	-4.519081	0.248523
H	4.345109	-2.024683	-1.758398
H	4.183420	-1.985910	-0.002156
H	4.448445	-3.517528	-0.828238
H	-2.278599	-0.408791	-1.385358
H	5.000794	0.859041	2.800227
H	6.050011	2.365031	1.167374
H	5.026875	2.696436	-1.042884
H	1.877527	1.043569	-2.177711
H	1.830197	3.311592	-3.057830
H	1.677587	3.353104	-1.295861
H	3.207929	3.781925	-2.062919
H	3.196496	1.502983	-4.197148
H	4.000071	0.242899	-3.252574
H	4.618368	1.892417	-3.230944
H	1.990645	-1.229522	2.108252
H	3.256618	-2.294295	3.897026
H	4.249550	-2.274027	2.436482
H	4.570241	-1.132680	3.742273
H	1.628110	-0.405972	4.391755
H	1.416976	0.927280	3.247724
H	2.896661	0.805723	4.195989
H	-2.264604	-1.901645	1.275211
H	-2.142320	-2.659782	-0.298754
H	-1.946802	-0.017167	2.374931
H	-2.459325	2.117912	3.487278
H	-3.084178	4.111653	2.153796
H	-2.650248	1.816050	-1.441383
H	-4.028609	-2.253180	-2.014885
H	-6.477345	-2.271453	-2.259983
H	-7.930563	-1.866208	-0.291354
H	-6.903141	-1.445402	1.925250
H	-4.452608	-1.414155	2.169368
H	-3.704170	5.222019	-2.070771
H	-2.434520	3.992692	-2.268130
H	-4.146251	3.499342	-2.113391

B3LYP energy = -1507.78612557 a.u.

(5*S*,1'*S*)-**9a**, Conf. R

C	-0.632280	-0.208280	1.679686
C	0.837401	-0.712768	1.702723
N	1.262084	-0.450933	0.272684
C	0.258868	0.094176	-0.393694
N	-0.825681	0.299804	0.308408
C	1.709244	-0.098438	2.835135
C	1.043039	-0.504701	4.167428
C	1.798305	1.431680	2.756682
C	3.121933	-0.707727	2.815551
C	-2.148388	0.782933	-0.139883
C	2.416394	-1.033568	-0.394079
C	3.469527	-0.202231	-0.823258
C	4.583917	-0.817300	-1.397027
C	4.652925	-2.192950	-1.548204
C	3.578675	-2.983717	-1.171771
C	2.428554	-2.426621	-0.611223
C	1.241254	-3.342165	-0.327125
C	0.739776	-4.010583	-1.620138
C	1.561522	-4.401435	0.740948
C	3.432404	1.319548	-0.755131
C	4.640450	1.910428	-0.009826
C	3.327357	1.921225	-2.169047
C	-2.063417	1.822621	-1.275943
C	-3.068726	-0.381261	-0.489229
C	-2.683497	-1.364563	-1.394504
C	-3.575628	-2.382468	-1.730012
C	-4.842368	-2.424847	-1.173354
C	-5.235705	-1.437627	-0.263527
C	-4.344102	-0.419524	0.081630
C	-1.369747	3.117537	-0.908663
C	-1.860984	3.920846	0.126296
C	-1.245028	5.124664	0.448679
C	-0.126417	5.551610	-0.263338
C	0.364804	4.768087	-1.301001
C	-0.253499	3.560821	-1.618990
O	-6.494410	-1.556763	0.234496
C	-6.974131	-0.571601	1.149832
H	-1.351411	-1.002692	1.877473
H	-0.806411	0.602873	2.383877

H	0.855258	-1.791456	1.845046
H	0.339709	0.329679	-1.443653
H	0.939310	-1.589457	4.247550
H	0.056129	-0.056908	4.298897
H	1.663811	-0.168694	4.999186
H	2.386767	1.805797	3.596278
H	0.819986	1.913139	2.813007
H	2.286253	1.764626	1.841971
H	3.085507	-1.796781	2.890041
H	3.675702	-0.451326	1.914740
H	3.689629	-0.337083	3.670923
H	-2.561919	1.283891	0.735762
H	5.411456	-0.206155	-1.732952
H	5.535002	-2.647279	-1.982055
H	3.625105	-4.052670	-1.335452
H	0.412347	-2.738677	0.042391
H	-0.157058	-4.597826	-1.412650
H	0.491680	-3.269215	-2.382016
H	1.488846	-4.683733	-2.040241
H	0.675862	-5.004174	0.951751
H	1.895033	-3.949096	1.676704
H	2.349618	-5.076735	0.402498
H	2.536828	1.618588	-0.211954
H	4.531205	2.993480	0.075499
H	4.736629	1.501152	0.996700
H	5.573691	1.714862	-0.540900
H	3.236741	3.007413	-2.109777
H	2.460006	1.536674	-2.710022
H	4.214553	1.690626	-2.761848
H	-3.098720	2.029994	-1.556647
H	-1.606955	1.377981	-2.162836
H	-1.700879	-1.350506	-1.848370
H	-3.277601	-3.148177	-2.435169
H	-5.542547	-3.209748	-1.427932
H	-4.632476	0.346337	0.788396
H	-2.744930	3.616381	0.675810
H	-1.644401	5.735431	1.249033
H	0.350725	6.491642	-0.015974
H	1.226137	5.096756	-1.869334
H	0.131730	2.969961	-2.441912
H	-6.376177	-0.554876	2.064763
H	-6.981686	0.422221	0.694725
H	-7.992215	-0.865047	1.391313

B3LYP energy = -1507.78595536 a.u.

(5*S*,1’*S*)-**9a**, Conf. S

C	0.150474	-0.419747	1.841414
C	1.689635	-0.266048	1.664324
N	1.810482	-0.180550	0.156171
C	0.615505	-0.331724	-0.386074
N	-0.370811	-0.468332	0.461836
C	2.316084	0.873931	2.517757
C	1.723287	2.256459	2.211033
C	3.843223	0.909256	2.335329
C	2.024170	0.516089	3.991630

C	-1.804529	-0.586561	0.091222
C	3.030140	-0.285617	-0.629637
C	3.711649	-1.518442	-0.658631
C	4.909914	-1.579732	-1.371228
C	5.392305	-0.481870	-2.065790
C	4.667708	0.699403	-2.079288
C	3.473172	0.829538	-1.368876
C	2.697943	2.137249	-1.478205
C	3.551718	3.362743	-1.113076
C	2.097212	2.302603	-2.887038
C	3.186167	-2.797207	-0.013910
C	2.889764	-3.865723	-1.081891
C	4.140727	-3.343704	1.060116
C	-2.387925	-1.906715	0.642459
C	-2.573991	0.669446	0.472204
C	-2.951321	0.936184	1.785826
C	-3.636861	2.114037	2.082935
C	-3.956871	3.018798	1.085999
C	-3.591358	2.752103	-0.237733
C	-2.894463	1.580829	-0.539168
C	-3.718749	-2.259088	0.014139
C	-3.766931	-2.798193	-1.275031
C	-4.982780	-3.122445	-1.867295
C	-6.173351	-2.914734	-1.175620
C	-6.137389	-2.383852	0.109559
C	-4.918510	-2.058841	0.698635
O	-3.958958	3.685101	-1.155293
C	-3.650607	3.462951	-2.531309
H	-0.115819	-1.331384	2.373424
H	-0.289224	0.428337	2.360506
H	2.183838	-1.189330	1.964429
H	0.469453	-0.356711	-1.456875
H	0.640812	2.290573	2.347798
H	1.946085	2.581359	1.196654
H	2.154892	2.992603	2.891407
H	4.136088	1.182424	1.323290
H	4.294159	-0.057517	2.569137
H	4.273054	1.646761	3.015574
H	0.957369	0.533205	4.223494
H	2.415571	-0.471031	4.248760
H	2.508021	1.245381	4.642781
H	-1.799533	-0.657371	-0.996375
H	5.462184	-2.510141	-1.399438
H	6.323045	-0.553329	-2.614701
H	5.035314	1.538489	-2.655171
H	1.865623	2.100191	-0.776860
H	2.933700	4.262846	-1.120747
H	3.996160	3.266432	-0.121450
H	4.361513	3.514778	-1.828565
H	1.501908	3.216781	-2.937170
H	1.452516	1.463085	-3.155963
H	2.881126	2.371488	-3.643751
H	2.234831	-2.581645	0.471134
H	2.441145	-4.746339	-0.617374
H	2.198641	-3.489032	-1.838262
H	3.800450	-4.185388	-1.591333

H	3.709701	-4.228323	1.533386
H	4.339359	-2.604416	1.838173
H	5.100192	-3.633419	0.627594
H	-2.495561	-1.849888	1.726176
H	-1.666428	-2.700331	0.433117
H	-2.733586	0.237410	2.582300
H	-3.930575	2.321193	3.104375
H	-4.497807	3.930049	1.305787
H	-2.610450	1.361209	-1.559107
H	-2.845652	-2.978033	-1.819327
H	-5.000507	-3.544353	-2.864752
H	-7.120791	-3.170435	-1.633504
H	-7.058023	-2.224507	0.657395
H	-4.902551	-1.649590	1.701721
H	-4.054274	4.318781	-3.065714
H	-2.570569	3.413163	-2.693174
H	-4.122398	2.549100	-2.901188

B3LYP energy = -1507.78582265 a.u.

(5*S*,1’*R*)-**9b**, Conf. A

C	-0.917431	0.353619	-1.189894
C	0.464888	1.045344	-1.371035
N	1.197751	0.584762	-0.127608
C	0.367909	-0.107173	0.633894
N	-0.827916	-0.288744	0.135613
C	1.127817	0.798377	-2.755333
C	2.441772	1.590601	-2.873621
C	0.145054	1.358449	-3.807226
C	1.389000	-0.685495	-3.048944
C	-1.925785	-1.045848	0.785965
C	2.433815	1.147897	0.394826
C	2.414765	2.466744	0.890545
C	3.626213	3.017303	1.310851
C	4.799593	2.279806	1.284966
C	4.777536	0.959551	0.864171
C	3.600112	0.358658	0.415115
C	3.627246	-1.116799	0.037574
C	4.703149	-1.442957	-1.010815
C	3.814260	-1.995268	1.288960
C	1.141490	3.290545	1.057837
C	0.863762	3.570074	2.546610
C	1.177028	4.600781	0.254458
C	-2.133076	-2.431952	0.137888
C	-3.182412	-0.188657	0.852212
C	-3.382135	0.630238	1.969110
C	-4.518944	1.421386	2.051462
C	-5.471623	1.418120	1.034911
C	-5.274321	0.602696	-0.080081
C	-4.129757	-0.198890	-0.163880
C	-0.977684	-3.387457	0.332358
C	-0.209973	-3.819801	-0.750336
C	0.838182	-4.719491	-0.569906
C	1.133874	-5.200600	0.700799
C	0.372398	-4.781282	1.789210
C	-0.674608	-3.885105	1.604679
O	-6.130158	0.512362	-1.132725
C	-7.333070	1.283526	-1.107468
H	-1.742443	1.061979	-1.199640
H	-1.102246	-0.403631	-1.949016
H	0.345591	2.123975	-1.275420
H	0.654844	-0.479104	1.607341
H	3.205880	1.236260	-2.184385
H	2.284286	2.654887	-2.684791
H	2.836230	1.489910	-3.886442
H	-0.074374	2.413574	-3.627074
H	-0.799706	0.812114	-3.832273
H	0.592114	1.277971	-4.799111
H	2.104101	-1.120576	-2.353325
H	0.478474	-1.285705	-3.016432
H	1.804176	-0.787986	-4.052968
H	-1.584336	-1.201288	1.809381
H	3.647310	4.033544	1.682872
H	5.728471	2.727826	1.615559
H	5.692163	0.381684	0.886644
H	2.661104	-1.375815	-0.393089
H	4.623799	-2.489530	-1.311885
H	4.600321	-0.825846	-1.904644
H	5.708477	-1.291729	-0.613875
H	3.775060	-3.051360	1.015419
H	3.037410	-1.812499	2.034133
H	4.779719	-1.805103	1.762415
H	0.296049	2.706440	0.695598
H	-0.083933	4.101389	2.656790
H	0.804445	2.643638	3.121363
H	1.646509	4.188435	2.989399
H	0.227077	5.129453	0.355623
H	1.354293	4.420927	-0.807362
H	1.965048	5.265178	0.613578
H	-3.034552	-2.849509	0.591909
H	-2.346828	-2.324084	-0.926271
H	-2.657315	0.637375	2.774275
H	-4.677248	2.047977	2.920375
H	-6.350755	2.039471	1.124568
H	-4.021779	-0.827002	-1.038188
H	-0.443161	-3.466307	-1.748143
H	1.416854	-5.049186	-1.424085
H	1.944922	-5.903919	0.842597
H	0.586594	-5.161935	2.780360
H	-1.273617	-3.585282	2.457914
H	-7.853473	1.048057	-2.031951
H	-7.960898	1.008486	-0.256543
H	-7.116505	2.354173	-1.074622

B3LYP energy = -1507.78830373 a.u.

(5*S*,1’*R*)-**9b**, Conf. B

C	-0.657293	0.121247	1.673112
C	0.577223	-0.827182	1.641752
N	1.062192	-0.643634	0.217761
C	0.219825	0.144349	-0.428511

N	-0.762298	0.622251	0.290092
C	1.587770	-0.600294	2.800771
C	2.174068	0.818165	2.826666
C	2.723628	-1.635564	2.731085
C	0.805041	-0.849218	4.108813
C	-1.834357	1.517987	-0.207882
C	1.992050	-1.510089	-0.492580
C	3.248752	-1.008227	-0.882905
C	4.124961	-1.887687	-1.521957
C	3.772294	-3.203693	-1.775086
C	2.509647	-3.658334	-1.429062
C	1.584433	-2.823091	-0.801916
C	0.178635	-3.359024	-0.549716
C	-0.529965	-3.684891	-1.877371
C	0.178718	-4.578615	0.386358
C	3.676497	0.442328	-0.699258
C	4.999004	0.574501	0.074271
C	3.776394	1.160496	-2.058105
C	-1.649808	2.966709	0.292209
C	-3.198008	0.912047	0.097486
C	-3.905101	1.215122	1.265470
C	-5.138686	0.619464	1.493743
C	-5.689656	-0.275399	0.580468
C	-4.986101	-0.577922	-0.586859
C	-3.743163	0.019891	-0.818569
C	-0.433261	3.658665	-0.280771
C	0.666378	3.972323	0.519666
C	1.778090	4.620074	-0.014565
C	1.805478	4.963288	-1.361765
C	0.712894	4.659742	-2.170800
C	-0.395878	4.015574	-1.633343
O	-5.420946	-1.430047	-1.552886
C	-6.698891	-2.050728	-1.395255
H	-1.573854	-0.396884	1.946018
H	-0.518134	0.957809	2.354629
H	0.242898	-1.861065	1.723108
H	0.324610	0.359674	-1.482754
H	1.406635	1.585405	2.940716
H	2.745521	1.042606	1.927770
H	2.850175	0.915129	3.677855
H	3.346069	-1.511535	1.847249
H	2.330726	-2.654740	2.726795
H	3.365642	-1.532377	3.607791
H	0.003721	-0.124119	4.262691
H	0.368512	-1.850641	4.128104
H	1.484476	-0.767433	4.958386
H	-1.709720	1.520847	-1.290834
H	5.099377	-1.532340	-1.830633
H	4.473943	-3.869174	-2.262483
H	2.227259	-4.675316	-1.668765
H	-0.415453	-2.581614	-0.070710
H	-1.559658	-3.993985	-1.686369
H	-0.552826	-2.816150	-2.538491
H	-0.030358	-4.496927	-2.408586
H	-0.845082	-4.906433	0.577816
H	0.647829	-4.350486	1.345217
H	0.719192	-5.418309	-0.054663
H	2.906924	0.953852	-0.122896
H	5.225503	1.628561	0.247624
H	4.955910	0.075181	1.043226
H	5.833544	0.146508	-0.483946
H	4.012623	2.215777	-1.908126
H	2.841595	1.100160	-2.618525
H	4.562785	0.724586	-2.677758
H	-2.551711	3.510107	0.002156
H	-1.612529	2.990607	1.382397
H	-3.510238	1.913369	1.990873
H	-5.689032	0.855845	2.395933
H	-6.653722	-0.718558	0.783401
H	-3.222269	-0.223121	-1.737285
H	0.651018	3.724354	1.574567
H	2.618017	4.862255	0.625015
H	2.666472	5.471365	-1.777840
H	0.719745	4.935466	-3.218218
H	-1.248225	3.805020	-2.270304
H	-7.496760	-1.305649	-1.348215
H	-6.835418	-2.671695	-2.276543
H	-6.727092	-2.677960	-0.500846

B3LYP energy = -1507.78815605 a.u.

(5*S*,1’*R*)-**9b**, Conf. C

C	-0.473214	-0.425296	-1.695296
C	0.678416	0.622868	-1.687038
N	1.035261	0.664189	-0.215207
C	0.191530	-0.098292	0.457745
N	-0.678540	-0.747622	-0.270789
C	1.812286	0.341288	-2.713019
C	2.508380	-1.008717	-2.495199
C	2.852756	1.474444	-2.685694
C	1.144028	0.348620	-4.105666
C	-1.764753	-1.607805	0.259479
C	1.839740	1.679065	0.448516
C	1.329672	2.989902	0.536396
C	2.144387	3.962536	1.117486
C	3.391201	3.643918	1.632761
C	3.837921	2.332282	1.601243
C	3.077160	1.318655	1.015770
C	3.590730	-0.114358	1.079147
C	5.007611	-0.263450	0.502010
C	3.540362	-0.647802	2.523429
C	-0.078178	3.383341	0.099859
C	-0.923658	3.815829	1.312061
C	-0.075187	4.475661	-0.982164
C	-1.610509	-3.072668	-0.191190
C	-3.121024	-0.990475	-0.058922
C	-3.820475	-1.282225	-1.234634
C	-5.046194	-0.673486	-1.471588
C	-5.597235	0.222883	-0.559801
C	-4.901990	0.513459	0.615467
C	-3.666727	-0.096617	0.855254
C	-0.358739	-3.752428	0.319154

C	0.592990	-4.256306	-0.568789
C	1.737271	-4.899975	-0.103313
C	1.946349	-5.049003	1.263309
C	1.000255	-4.558452	2.160314
C	-0.142155	-3.919327	1.691228
O	-5.337511	1.364853	1.581726
C	-6.609287	1.996423	1.417551
H	-1.393081	-0.028845	-2.119865
H	-0.201548	-1.329917	-2.235418
H	0.277165	1.604844	-1.934587
H	0.209213	-0.161195	1.536784
H	1.817929	-1.851797	-2.550193
H	3.019937	-1.055118	-1.535637
H	3.258439	-1.158047	-3.273744
H	3.395769	1.519510	-1.743489
H	2.385595	2.447433	-2.852527
H	3.582778	1.317105	-3.481772
H	0.429230	-0.466990	-4.231326
H	0.624094	1.290664	-4.295452
H	1.908603	0.231433	-4.875037
H	-1.635246	-1.574531	1.340878
H	1.787434	4.981954	1.187133
H	4.005561	4.415085	2.080689
H	4.794744	2.087625	2.043711
H	2.927239	-0.740453	0.484317
H	5.299084	-1.315706	0.498638
H	5.069997	0.107061	-0.522232
H	5.743207	0.278893	1.098637
H	3.854292	-1.693222	2.549185
H	2.534402	-0.587496	2.944116
H	4.206819	-0.079431	3.175406
H	-0.574150	2.508744	-0.319977
H	-1.947768	4.027787	0.998053
H	-0.956466	3.033690	2.073237
H	-0.522930	4.718652	1.776333
H	-1.096323	4.685020	-1.307263
H	0.505889	4.178891	-1.857402
H	0.348813	5.407552	-0.603937
H	-2.494655	-3.599642	0.175719
H	-1.645766	-3.139936	-1.279332
H	-3.427629	-1.982823	-1.958899
H	-5.590454	-0.900857	-2.379765
H	-6.555218	0.675886	-0.769725
H	-3.151443	0.138412	1.779183
H	0.431875	-4.163323	-1.636866
H	2.460384	-5.288733	-0.809777
H	2.833293	-5.551876	1.627976
H	1.146839	-4.683980	3.226102
H	-0.879392	-3.564356	2.403179
H	-6.747637	2.613818	2.301073
H	-6.625654	2.628581	0.526347
H	-7.412556	1.257890	1.360504

B3LYP energy = -1507.78799422 a.u.

(5*S*,1'*R*)-**9b**, Conf. D

C	0.196593	0.046412	1.984587
C	1.703392	0.276549	1.675245
N	1.775942	-0.150124	0.224099
C	0.591633	-0.586989	-0.167572
N	-0.350677	-0.520399	0.736831
C	2.225340	1.694027	2.047685
C	3.737417	1.799583	1.784901
C	1.999656	1.850853	3.567793
C	1.488834	2.820990	1.309763
C	-1.785575	-0.858563	0.608414
C	2.982504	-0.421135	-0.540807
C	3.777674	-1.525623	-0.176311
C	4.965646	-1.727288	-0.880184
C	5.328793	-0.901149	-1.932172
C	4.490934	0.132208	-2.320651
C	3.299255	0.396628	-1.643255
C	2.386229	1.498093	-2.167519
C	3.104581	2.849561	-2.311358
C	1.748752	1.082127	-3.506759
C	3.380986	-2.544415	0.888044
C	3.177254	-3.936797	0.263109
C	4.391096	-2.606908	2.045160
C	-2.050138	-1.702563	-0.656923
C	-2.641245	0.396703	0.702359
C	-3.636567	0.486655	1.677388
C	-4.435459	1.621383	1.740532
C	-4.256672	2.676880	0.851086
C	-3.256707	2.588950	-0.120607
C	-2.454130	1.447009	-0.187896
C	-3.445155	-2.287543	-0.686127
C	-3.741103	-3.440446	0.046118
C	-5.020469	-3.985979	0.027673
C	-6.023737	-3.386327	-0.728803
C	-5.738218	-2.241308	-1.465753
C	-4.457389	-1.696848	-1.443874
O	-2.986581	3.554967	-1.040183
C	-3.786343	4.739311	-1.045179
H	0.037968	-0.656504	2.801736
H	-0.330407	0.970266	2.214367
H	2.304764	-0.434857	2.239387
H	0.432879	-0.982966	-1.158983
H	3.984660	1.719676	0.727812
H	4.290150	1.024643	2.320732
H	4.100144	2.766691	2.137714
H	2.494112	1.055813	4.131203
H	0.941790	1.852390	3.837020
H	2.419089	2.801747	3.899558
H	1.660409	2.785510	0.235529
H	0.411125	2.803584	1.481637
H	1.854324	3.785607	1.666251
H	-2.005419	-1.479017	1.479482
H	5.605884	-2.558817	-0.615268
H	6.254623	-1.077529	-2.465468
H	4.765590	0.744036	-3.169895
H	1.573526	1.636452	-1.454986
H	2.390035	3.620233	-2.607500

H	3.570893	3.162468	-1.376018
H	3.882116	2.811987	-3.076371
H	1.040865	1.843674	-3.840619
H	1.214235	0.133230	-3.423774
H	2.506784	0.964576	-4.283609
H	2.417124	-2.257942	1.308180
H	2.824967	-4.639333	1.021430
H	2.440692	-3.906510	-0.542132
H	4.107723	-4.331004	-0.149055
H	4.045422	-3.305603	2.809676
H	4.530481	-1.631401	2.513975
H	5.368268	-2.949736	1.700296
H	-1.896216	-1.090082	-1.547838
H	-1.324909	-2.520320	-0.684945
H	-3.791019	-0.326876	2.375266
H	-5.211084	1.691510	2.492938
H	-4.890127	3.549115	0.922455
H	-1.690354	1.410034	-0.955133
H	-2.963612	-3.924034	0.627685
H	-5.231505	-4.882884	0.597052
H	-7.019184	-3.812256	-0.747652
H	-6.511428	-1.772054	-2.061556
H	-4.244064	-0.805599	-2.022115
H	-4.837252	4.505693	-1.231761
H	-3.691970	5.285048	-0.103196
H	-3.402272	5.351015	-1.857202

B3LYP energy = -1507.78791457 a.u.

(5*S*,1’*R*)-**9b**, Conf. E

C	0.145529	-0.759921	-1.631666
C	1.670994	-0.950352	-1.396033
N	1.902596	-0.048834	-0.201404
C	0.770256	0.551260	0.123315
N	-0.262415	0.220889	-0.607255
C	2.121471	-2.435067	-1.288059
C	3.649515	-2.524701	-1.134174
C	1.741301	-3.098276	-2.630166
C	1.435942	-3.190647	-0.141009
C	-1.673685	0.637323	-0.448326
C	3.189265	0.430382	0.279194
C	3.926708	1.310772	-0.537524
C	5.187878	1.706055	-0.089414
C	5.681910	1.284486	1.134952
C	4.906538	0.477291	1.951883
C	3.644459	0.034421	1.552101
C	2.817546	-0.788828	2.532300
C	3.549753	-2.054046	3.009046
C	2.389052	0.068196	3.738713
C	3.397638	1.905628	-1.839133
C	3.233933	3.431499	-1.714328
C	4.276361	1.547055	-3.048812
C	-1.786881	1.880257	0.459475
C	-2.524914	-0.536819	0.011799
C	-2.248164	-1.200481	1.212293
C	-3.056958	-2.252607	1.614218
C	-4.144284	-2.662021	0.843310
C	-4.419974	-2.000116	-0.352955
C	-3.601452	-0.940206	-0.763648
C	-3.171036	2.490462	0.439536
C	-3.562176	3.313221	-0.620204
C	-4.833948	3.875482	-0.652288
C	-5.733832	3.624556	0.379997
C	-5.352826	2.810555	1.441983
C	-4.079870	2.248043	1.470423
O	-5.447349	-2.307319	-1.188002
C	-6.329579	-3.371815	-0.827189
H	-0.080277	-0.366403	-2.622224
H	-0.418416	-1.679039	-1.488341
H	2.227093	-0.516286	-2.225268
H	0.718450	1.272606	0.924616
H	3.999327	-2.100339	-0.195116
H	4.162936	-2.009347	-1.948871
H	3.956424	-3.571917	-1.160550
H	2.188025	-2.573535	-3.478269
H	0.661542	-3.140613	-2.784556
H	2.110347	-4.124808	-2.643543
H	1.703667	-2.784177	0.832861
H	0.347700	-3.182644	-0.223891
H	1.750945	-4.235401	-0.156553
H	-1.998991	0.921292	-1.451175
H	5.783375	2.372648	-0.699580
H	6.662953	1.604661	1.463136
H	5.286496	0.187744	2.922913
H	1.907164	-1.109828	2.027443
H	2.894802	-2.643124	3.654360
H	3.856910	-2.685235	2.174120
H	4.442592	-1.806071	3.585612
H	1.730317	-0.505929	4.393808
H	1.859204	0.971498	3.429102
H	3.254541	0.380191	4.326719
H	2.400891	1.508179	-2.029073
H	2.792693	3.836718	-2.627435
H	2.584700	3.693278	-0.876366
H	4.194959	3.925285	-1.560748
H	3.835088	1.944904	-3.964963
H	4.385982	0.467233	-3.165308
H	5.277134	1.971987	-2.952741
H	-1.522709	1.610531	1.483817
H	-1.065077	2.625679	0.115175
H	-1.411584	-0.900543	1.831812
H	-2.847184	-2.768876	2.542848
H	-4.758054	-3.483682	1.182492
H	-3.835470	-0.439384	-1.695037
H	-2.864812	3.525404	-1.423464
H	-5.119315	4.514890	-1.478620
H	-6.723205	4.064275	0.358475
H	-6.045213	2.613906	2.251320
H	-3.792005	1.616055	2.302203
H	-7.064111	-3.429187	-1.626049
H	-5.794848	-4.322245	-0.756184
H	-6.836269	-3.162428	0.118073

B3LYP energy = -1507.78776618 a.u.

(5*S*,1'*R*)-**9b**, Conf. F

C	-0.922411	-0.426278	1.184726
C	0.508716	-1.000381	1.396789
N	1.216386	-0.513373	0.148919
C	0.342265	0.089300	-0.638124
N	-0.868903	0.189309	-0.154758
C	1.135430	-0.664191	2.779429
C	0.196645	-1.282965	3.838481
C	1.262016	0.843866	3.035744
C	2.511707	-1.336511	2.925395
C	-2.026944	0.798457	-0.853849
C	2.503249	-0.977847	-0.347610
C	2.609494	-2.306426	-0.805083
C	3.867917	-2.752386	-1.210930
C	4.966766	-1.907686	-1.209371
C	4.820007	-0.584145	-0.825785
C	3.591024	-0.084104	-0.391498
C	3.480260	1.397600	-0.056531
C	4.509754	1.848717	0.992132
C	3.602398	2.252201	-1.331957
C	1.421316	-3.251817	-0.949943
C	1.177835	-3.596457	-2.431030
C	1.577994	-4.531006	-0.111155
C	-2.404721	2.166603	-0.249496
C	-3.167843	-0.209272	-0.933857
C	-3.144217	-1.154130	-1.956042
C	-4.159120	-2.104656	-2.049076
C	-5.195669	-2.118773	-1.131777
C	-5.229199	-1.168982	-0.105102
C	-4.214666	-0.213726	-0.006627
C	-1.353517	3.236036	-0.447701
C	-1.079609	3.730595	-1.727813
C	-0.127384	4.725385	-1.919718
C	0.567760	5.247279	-0.831334
C	0.300724	4.768784	0.446649
C	-0.652641	3.770455	0.634753
O	-6.286515	-1.254448	0.745219
C	-6.400159	-0.309734	1.808970
H	-1.688166	-1.198506	1.203160
H	-1.175170	0.330577	1.924320
H	0.476419	-2.087172	1.327917
H	0.607805	0.455676	-1.619830
H	0.083511	-2.359657	3.691274
H	-0.797506	-0.831713	3.834146
H	0.618953	-1.126770	4.832179
H	0.299448	1.357727	3.003355
H	1.924964	1.325997	2.319627
H	1.678189	1.008844	4.031043
H	2.446807	-2.413010	2.752899
H	3.251256	-0.928903	2.238869
H	2.883048	-1.186440	3.940736
H	-1.676131	0.968563	-1.871973
H	3.983691	-3.772020	-1.554687
H	5.933556	-2.275215	-1.530378
H	5.675798	0.076762	-0.868617
H	2.489846	1.579399	0.358278
H	4.336240	2.893319	1.258516
H	4.448728	1.252953	1.903969
H	5.529479	1.772834	0.610721
H	3.444596	3.306354	-1.096181
H	2.868522	1.960842	-2.085921
H	4.593711	2.151095	-1.778460
H	0.521926	-2.742587	-0.605298
H	0.278405	-4.207772	-2.530592
H	1.045839	-2.694711	-3.032426
H	2.012269	-4.159921	-2.852354
H	0.683522	-5.150678	-0.201113
H	1.731184	-4.307942	0.946273
H	2.428103	-5.126557	-0.449232
H	-3.334116	2.473141	-0.734689
H	-2.628874	2.062432	0.813238
H	-2.342879	-1.145122	-2.685031
H	-4.143761	-2.834332	-2.848925
H	-5.993092	-2.847770	-1.194538
H	-4.244489	0.527297	0.778112
H	-1.628438	3.349469	-2.582402
H	0.063982	5.101855	-2.917098
H	1.304473	6.027023	-0.979062
H	0.827871	5.176466	1.300502
H	-0.863676	3.418772	1.638264
H	-7.313515	-0.569320	2.337496
H	-5.552889	-0.381032	2.496248
H	-6.480778	0.710967	1.426821

B3LYP energy = -1507.78729554 a.u.

(5*S*,1'*R*)-**9b**, Conf. G

C	-0.700298	0.163818	1.716872
C	0.578956	-0.724847	1.698450
N	1.004719	-0.608839	0.249972
C	0.124358	0.126421	-0.406031
N	-0.850772	0.610316	0.318634
C	1.619559	-0.371322	2.799070
C	2.816571	-1.336205	2.741589
C	0.903920	-0.589152	4.150361
C	2.115471	1.078761	2.716353
C	-1.975963	1.429967	-0.195512
C	1.968862	-1.457265	-0.433537
C	1.654207	-2.818008	-0.620652
C	2.623776	-3.632003	-1.207497
C	3.836445	-3.114323	-1.634717
C	4.090886	-1.757885	-1.509089
C	3.168396	-0.895937	-0.913157
C	3.477070	0.595410	-0.876748
C	4.827783	0.909477	-0.213494
C	3.423213	1.194429	-2.295223
C	0.296438	-3.429135	-0.289178
C	-0.417059	-3.893864	-1.572067
C	0.399584	-4.579136	0.725711

C	-1.868374	2.901275	0.256049
C	-3.301145	0.756256	0.140127
C	-4.034480	1.080255	1.276830
C	-5.232048	0.412192	1.539530
C	-5.701759	-0.566062	0.682363
C	-4.971010	-0.893911	-0.465998
C	-3.772273	-0.232841	-0.731980
C	-0.668315	3.629032	-0.307079
C	0.377348	4.043199	0.519410
C	1.474277	4.725476	-0.002020
C	1.540266	5.004143	-1.362815
C	0.500963	4.601016	-2.198031
C	-0.592827	3.921594	-1.673274
O	-5.509294	-1.858698	-1.257836
C	-4.833012	-2.219477	-2.461825
H	-1.587486	-0.386721	2.022444
H	-0.588709	1.027620	2.367859
H	0.302293	-1.765956	1.858993
H	0.194879	0.298976	-1.470715
H	3.402426	-1.220198	1.831878
H	2.491483	-2.376856	2.804815
H	3.477447	-1.144267	3.588776
H	0.534147	-1.612994	4.245022
H	0.062682	0.091459	4.294205
H	1.607068	-0.413201	4.965662
H	2.634575	1.281827	1.780997
H	1.305976	1.803576	2.817466
H	2.817885	1.268624	3.529689
H	-1.858391	1.405705	-1.279818
H	2.415741	-4.684415	-1.351403
H	4.574008	-3.764642	-2.088342
H	5.023561	-1.357990	-1.884737
H	2.704197	1.090142	-0.290274
H	4.962591	1.990396	-0.139044
H	4.893116	0.491213	0.791941
H	5.662592	0.514399	-0.794870
H	3.570216	2.275310	-2.252881
H	2.464425	1.003126	-2.781827
H	4.205092	0.771934	-2.929762
H	-0.338613	-2.660348	0.149561
H	-1.414812	-4.267106	-1.331515
H	-0.520627	-3.074541	-2.286306
H	0.130455	-4.699062	-2.065220
H	-0.595760	-4.955780	0.969655
H	0.878036	-4.259983	1.653339
H	0.979732	-5.412187	0.324565
H	-2.785851	3.392349	-0.075909
H	-1.861287	2.965931	1.344953
H	-3.697573	1.848077	1.959737
H	-5.804493	0.666339	2.422782
H	-6.632361	-1.083825	0.875432
H	-3.199978	-0.470548	-1.618404
H	0.328639	3.849154	1.584706
H	2.271647	5.045206	0.657594
H	2.389109	5.539533	-1.769467
H	0.536959	4.826557	-3.256800
H	-1.404772	3.633822	-2.332312
H	-5.440310	-2.994833	-2.921005
H	-4.755734	-1.368198	-3.142980
H	-3.836214	-2.616461	-2.252784

B3LYP energy = -1507.78701315 a.u.

(5*S*,1’*R*)-**9b**, Conf. H

C	0.271185	-2.064891	0.956986
C	1.791869	-1.750542	0.869711
N	1.777113	-0.414049	0.162299
C	0.533324	-0.085674	-0.138480
N	-0.377723	-0.943189	0.246813
C	2.549729	-1.856612	2.224533
C	4.049414	-1.576362	2.027572
C	2.398645	-3.322895	2.685169
C	1.985649	-0.927081	3.308676
C	-1.856950	-0.768022	0.231240
C	2.914306	0.246356	-0.458969
C	3.496324	-0.346682	-1.596206
C	4.626917	0.265768	-2.138379
C	5.134329	1.440503	-1.605918
C	4.499254	2.044068	-0.531961
C	3.375083	1.470341	0.064602
C	2.676619	2.222383	1.190664
C	3.628685	2.602015	2.336266
C	1.960853	3.473144	0.645858
C	2.917198	-1.566833	-2.306194
C	2.402603	-1.185184	-3.706657
C	3.916835	-2.731683	-2.388643
C	-2.521350	-1.954440	-0.504903
C	-2.248708	0.595222	-0.305619
C	-2.176890	0.899593	-1.670902
C	-2.544798	2.162879	-2.111621
C	-2.992412	3.136803	-1.221163
C	-3.066341	2.834663	0.138881
C	-2.688004	1.563262	0.587361
C	-4.018568	-2.005338	-0.294661
C	-4.545073	-2.452867	0.920676
C	-5.919557	-2.501158	1.127180
C	-6.791582	-2.106022	0.116123
C	-6.279187	-1.664279	-1.099378
C	-4.902618	-1.613876	-1.301186
O	-3.486399	3.695948	1.101255
C	-3.920294	5.001459	0.712853
H	0.022787	-3.010463	0.480867
H	-0.093594	-2.087894	1.983387
H	2.269007	-2.458129	0.193225
H	0.289524	0.818847	-0.674624
H	4.246593	-0.543814	1.746161
H	4.479262	-2.222904	1.259465
H	4.580729	-1.772668	2.960605
H	2.789230	-4.018643	1.938699
H	1.361088	-3.591802	2.892843
H	2.963263	-3.473500	3.606332
H	2.078062	0.122843	3.035848

H	0.936313	-1.129260	3.531716
H	2.541735	-1.073855	4.236147
H	-2.156955	-0.813702	1.280742
H	5.105136	-0.172348	-3.004958
H	6.012427	1.899166	-2.043305
H	4.882154	2.981840	-0.151096
H	1.909314	1.572362	1.608422
H	3.063875	3.053745	3.154393
H	4.156071	1.731928	2.730077
H	4.376224	3.329015	2.014269
H	1.408710	3.970547	1.446010
H	1.254252	3.222247	-0.148176
H	2.676887	4.188588	0.236618
H	2.049605	-1.920301	-1.749873
H	1.935186	-2.049637	-4.183011
H	1.662443	-0.384084	-3.653161
H	3.215081	-0.846766	-4.352088
H	3.444577	-3.599886	-2.852898
H	4.276523	-3.027790	-1.401612
H	4.787137	-2.467720	-2.992259
H	-2.289056	-1.898374	-1.568785
H	-2.083416	-2.883119	-0.134054
H	-1.843965	0.160681	-2.388368
H	-2.493027	2.399225	-3.167099
H	-3.280516	4.108741	-1.594307
H	-2.759710	1.350552	1.646854
H	-3.876551	-2.777306	1.711089
H	-6.310019	-2.854795	2.073623
H	-7.862186	-2.146855	0.273877
H	-6.950263	-1.360178	-1.893331
H	-4.513496	-1.268618	-2.251677
H	-4.217888	5.498337	1.632276
H	-3.110621	5.563609	0.241168
H	-4.775419	4.947113	0.035062

B3LYP energy = -1507.78697646 a.u.

(5*S*,1'*R*)-**9b**, Conf. I

C	0.226812	-2.333196	0.313879
C	1.741688	-2.017551	0.154148
N	1.718191	-0.508646	0.040022
C	0.466752	-0.084526	0.047906
N	-0.437072	-1.020363	0.185424
C	2.647760	-2.660295	1.243030
C	4.128367	-2.342472	0.971522
C	2.461702	-4.187644	1.108910
C	2.277085	-2.229387	2.669245
C	-1.908920	-0.857272	0.347881
C	2.814708	0.349346	-0.381209
C	3.263105	0.264841	-1.714198
C	4.355729	1.051845	-2.080605
C	4.953474	1.918784	-1.179377
C	4.448467	2.035876	0.105777
C	3.368672	1.263005	0.536878
C	2.820640	1.491885	1.940197
C	3.897870	1.351547	3.028085
C	2.133309	2.866776	2.041031
C	2.577921	-0.575598	-2.787387
C	1.940792	0.326613	-3.860471
C	3.526952	-1.600049	-3.429971
C	-2.642117	-1.482951	-0.862030
C	-2.282094	0.583905	0.641842
C	-2.581716	0.953325	1.956447
C	-2.924727	2.267653	2.243459
C	-2.970912	3.233760	1.242344
C	-2.669272	2.869024	-0.071249
C	-2.327947	1.544194	-0.363467
C	-4.126916	-1.649690	-0.626937
C	-4.598215	-2.669715	0.204869
C	-5.960709	-2.833112	0.431093
C	-6.876482	-1.978866	-0.177658
C	-6.419526	-0.964508	-1.012708
C	-5.054662	-0.801716	-1.233975
O	-2.681580	3.718933	-1.131768
C	-3.047072	5.084166	-0.915077
H	-0.130841	-3.017256	-0.452252
H	-0.012470	-2.751791	1.290657
H	2.091354	-2.398324	-0.804417
H	0.210120	0.958231	-0.059051
H	4.355941	-1.284472	1.086787
H	4.423339	-2.642902	-0.036303
H	4.752510	-2.895015	1.676133
H	2.716878	-4.534477	0.104636
H	1.441902	-4.507813	1.331284
H	3.121687	-4.696263	1.813155
H	2.402376	-1.158275	2.817721
H	1.250910	-2.491244	2.934650
H	2.929307	-2.737170	3.381682
H	-2.156528	-1.446563	1.233595
H	4.730600	1.001597	-3.094711
H	5.799870	2.520677	-1.486129
H	4.899138	2.744457	0.788363
H	2.059102	0.737944	2.135469
H	3.441866	1.436383	4.016587
H	4.408380	0.389064	2.973083
H	4.653394	2.134987	2.947347
H	1.687826	2.993133	3.030019
H	1.342890	2.982552	1.296888
H	2.850165	3.676396	1.890437
H	1.759674	-1.130496	-2.330121
H	1.393756	-0.281471	-4.584101
H	1.243624	1.040296	-3.417312
H	2.698416	0.892586	-4.405459
H	2.983948	-2.213495	-4.151833
H	3.971251	-2.263654	-2.686049
H	4.342340	-1.107750	-3.963200
H	-2.464029	-0.874667	-1.749507
H	-2.204269	-2.463400	-1.059451
H	-2.560511	0.213769	2.747025
H	-3.166688	2.550224	3.260371
H	-3.245734	4.248388	1.491200
H	-2.112802	1.297982	-1.395501

H	-3.895751	-3.351511	0.672335
H	-6.307840	-3.631815	1.075174
H	-7.938025	-2.107073	-0.006122
H	-7.125006	-0.299362	-1.495468
H	-4.710012	-0.009340	-1.887621
H	-4.064064	5.163144	-0.523944
H	-2.350026	5.576897	-0.232907
H	-2.996277	5.560514	-1.890461

B3LYP energy = -1507.78697405 a.u.

(5*S*,1’*R*)-**9b**, Conf. J

C	-0.244347	-1.552977	-1.390820
C	-1.613446	-0.867028	-1.115493
N	-1.195239	0.286495	-0.227388
C	0.100292	0.204358	0.018680
N	0.717013	-0.783410	-0.575919
C	-2.440639	-0.551341	-2.394991
C	-3.801032	0.063396	-2.024271
C	-2.705966	-1.912961	-3.074174
C	-1.708797	0.369997	-3.381953
C	2.179192	-1.069372	-0.547244
C	-2.075758	1.136550	0.557015
C	-2.775732	0.563890	1.637020
C	-3.673038	1.377165	2.330545
C	-3.835820	2.713692	2.001158
C	-3.078229	3.272247	0.983745
C	-2.179182	2.505293	0.240586
C	-1.323865	3.194298	-0.815489
C	-2.157919	3.991458	-1.830985
C	-0.268659	4.102386	-0.154813
C	-2.549219	-0.861205	2.132113
C	-1.953384	-0.852521	3.552317
C	-3.826486	-1.715262	2.081101
C	2.540225	-2.037086	0.600205
C	2.980777	0.224000	-0.555093
C	3.246429	0.841277	-1.783256
C	3.953996	2.033361	-1.818095
C	4.410981	2.633354	-0.646192
C	4.152767	2.017729	0.578457
C	3.438201	0.813252	0.616897
C	1.906712	-3.405431	0.490986
C	0.855223	-3.780477	1.329621
C	0.270520	-5.040253	1.222036
C	0.733452	-5.945568	0.272732
C	1.787455	-5.586015	-0.563911
C	2.369232	-4.327896	-0.452888
O	4.549528	2.500131	1.785465
C	5.293751	3.719823	1.827438
H	-0.234573	-2.597663	-1.091350
H	0.052852	-1.487051	-2.436417
H	-2.231189	-1.522035	-0.502200
H	0.608300	0.905886	0.664209
H	-3.703853	1.050794	-1.577022
H	-4.349006	-0.571257	-1.324639
H	-4.410319	0.164439	-2.924340
H	-3.219373	-2.602065	-2.399277
H	-1.789030	-2.393975	-3.420044
H	-3.344076	-1.764228	-3.946445
H	-1.538277	1.361604	-2.966627
H	-0.746811	-0.034603	-3.702226
H	-2.317144	0.492953	-4.279711
H	2.367459	-1.576320	-1.494653
H	-4.236466	0.962310	3.156280
H	-4.537274	3.326601	2.553429
H	-3.184892	4.325975	0.762047
H	-0.783992	2.427132	-1.369090
H	-1.508354	4.393867	-2.611018
H	-2.915547	3.369587	-2.309947
H	-2.666344	4.834952	-1.360920
H	0.378213	4.542938	-0.916402
H	0.362224	3.551436	0.545938
H	-0.741232	4.917532	0.397291
H	-1.807749	-1.340711	1.493338
H	-1.722623	-1.871582	3.869897
H	-1.033333	-0.266240	3.592960
H	-2.651781	-0.428617	4.275989
H	-3.610287	-2.734303	2.408211
H	-4.242678	-1.764238	1.073345
H	-4.599297	-1.313806	2.739098
H	3.627947	-2.135399	0.593731
H	2.266634	-1.583833	1.554295
H	2.909651	0.382692	-2.704946
H	4.165495	2.506146	-2.769097
H	4.965196	3.558903	-0.700931
H	3.276261	0.359981	1.585702
H	0.494348	-3.085638	2.079677
H	-0.539951	-5.316169	1.885532
H	0.284158	-6.927402	0.191195
H	2.163097	-6.290014	-1.296282
H	3.199498	-4.064765	-1.099217
H	5.499507	3.902903	2.878637
H	6.236561	3.626444	1.283179
H	4.713273	4.551397	1.420341

B3LYP energy = -1507.78690236 a.u.

(5*S*,1’*R*)-**9b**, Conf. K

C	0.128690	-0.710145	-1.641301
C	1.671274	-0.802344	-1.477504
N	1.882893	0.006566	-0.213329
C	0.731179	0.528130	0.173287
N	-0.299956	0.197338	-0.560623
C	2.232144	-2.251358	-1.532399
C	3.768291	-2.241047	-1.447165
C	1.841206	-2.808245	-2.919414
C	1.654265	-3.165565	-0.443175
C	-1.728472	0.515519	-0.350883
C	3.153541	0.483176	0.308835
C	3.866743	1.450983	-0.425327
C	5.114487	1.842692	0.061830
C	5.615385	1.334443	1.250005

C	4.860870	0.439401	1.991944
C	3.615259	-0.006033	1.546801
C	2.803586	-0.933582	2.442189
C	3.587797	-2.184059	2.871828
C	2.279735	-0.176129	3.677012
C	3.324422	2.138765	-1.674510
C	3.131555	3.646280	-1.427152
C	4.208979	1.894805	-2.907972
C	-1.901097	1.761904	0.543680
C	-2.483338	-0.708032	0.150871
C	-2.095677	-1.362743	1.316370
C	-2.826279	-2.462506	1.763470
C	-3.933131	-2.910415	1.061216
C	-4.325554	-2.255682	-0.110500
C	-3.594648	-1.155852	-0.565841
C	-3.315980	2.297000	0.522680
C	-3.752112	3.089632	-0.542713
C	-5.052404	3.582315	-0.576984
C	-5.936359	3.290198	0.458444
C	-5.510849	2.505673	1.525793
C	-4.209197	2.013217	1.556627
O	-5.420099	-2.761050	-0.738132
C	-5.887937	-2.128240	-1.929810
H	-0.167630	-0.294121	-2.603335
H	-0.363909	-1.672066	-1.510795
H	2.157050	-0.250655	-2.281475
H	0.664463	1.190968	1.023489
H	4.131875	-1.883511	-0.485406
H	4.204622	-1.614639	-2.228523
H	4.145945	-3.255235	-1.589169
H	2.215075	-2.172954	-3.725983
H	0.761068	-2.912918	-3.038939
H	2.278615	-3.799353	-3.048524
H	1.930310	-2.835413	0.556538
H	0.566104	-3.237328	-0.487700
H	2.046350	-4.175595	-0.573563
H	-2.103520	0.762451	-1.345776
H	5.692995	2.574596	-0.487037
H	6.585187	1.654118	1.610499
H	5.245499	0.081412	2.937932
H	1.934786	-1.272969	1.879477
H	2.936958	-2.856398	3.434526
H	3.978304	-2.732177	2.013128
H	4.430639	-1.928756	3.516413
H	1.644436	-0.828678	4.279682
H	1.695561	0.703399	3.396928
H	3.103954	0.163983	4.307276
H	2.335181	1.738460	-1.894931
H	2.683499	4.115933	-2.305384
H	2.477241	3.827854	-0.572117
H	4.082791	4.143978	-1.231752
H	3.761837	2.357483	-3.790059
H	4.336584	0.829891	-3.110467
H	5.202422	2.326918	-2.774947
H	-1.621050	1.518004	1.569742
H	-1.221413	2.539739	0.185947
H	-1.233828	-1.028552	1.881767
H	-2.528240	-2.974353	2.670033
H	-4.506656	-3.762801	1.401505
H	-3.886283	-0.637479	-1.468241
H	-3.066876	3.334710	-1.347190
H	-5.372454	4.200799	-1.406585
H	-6.947853	3.676292	0.435686
H	-6.190815	2.278135	2.337514
H	-3.885895	1.404633	2.392837
H	-6.761410	-2.695031	-2.240837
H	-6.176155	-1.091244	-1.740768
H	-5.133374	-2.163394	-2.720094

B3LYP energy = -1507.78687339 a.u.

(5*S*,1'*R*)-**9b**, Conf. L

C	0.048010	0.541483	1.437396
C	-1.504025	0.674158	1.435733
N	-1.861935	-0.020355	0.138284
C	-0.757423	-0.430434	-0.458241
N	0.351747	-0.183476	0.188455
C	-2.189568	0.178913	2.741332
C	-1.895675	-1.294413	3.056740
C	-3.709073	0.407139	2.669647
C	-1.635717	1.064301	3.879231
C	1.708706	-0.596696	-0.252311
C	-3.140645	0.014578	-0.552945
C	-3.578655	1.236562	-1.100566
C	-4.832377	1.258352	-1.713069
C	-5.602392	0.110885	-1.819733
C	-5.118159	-1.093807	-1.335315
C	-3.879837	-1.176425	-0.696185
C	-3.377522	-2.544984	-0.251955
C	-4.378970	-3.279625	0.654296
C	-3.017204	-3.414321	-1.471685
C	-2.734171	2.506291	-1.124078
C	-2.348531	2.877470	-2.567997
C	-3.427679	3.685499	-0.422218
C	2.317533	-1.593524	0.759533
C	2.558961	0.622764	-0.574560
C	2.661374	1.038495	-1.906156
C	3.412794	2.160650	-2.224817
C	4.068753	2.890298	-1.236212
C	3.968180	2.478063	0.093243
C	3.213779	1.344594	0.415790
C	3.508011	-2.340485	0.198112
C	3.312454	-3.417928	-0.671453
C	4.394370	-4.111856	-1.202349
C	5.693732	-3.739365	-0.868131
C	5.900534	-2.672787	0.000576
C	4.815143	-1.979545	0.529329
O	4.565211	3.102059	1.144216
C	5.380082	4.248305	0.890258
H	0.548150	1.507918	1.432081
H	0.410087	-0.033818	2.286619
H	-1.781196	1.720594	1.319917

H	-0.775269	-0.925441	-1.418808
H	-0.826996	-1.499891	3.146152
H	-2.307786	-1.963558	2.303403
H	-2.352263	-1.558919	4.011931
H	-4.189132	-0.207650	1.910630
H	-3.941283	1.452574	2.455064
H	-4.159974	0.158102	3.631905
H	-0.565649	0.921861	4.040883
H	-1.813220	2.124161	3.681193
H	-2.140635	0.812001	4.812826
H	1.541577	-1.134283	-1.185793
H	-5.201756	2.184865	-2.133268
H	-6.571568	0.151154	-2.301206
H	-5.711511	-1.990277	-1.458857
H	-2.463505	-2.405514	0.324429
H	-3.940187	-4.212498	1.014252
H	-4.656040	-2.679547	1.522266
H	-5.294701	-3.534212	0.118071
H	-2.607045	-4.372263	-1.145029
H	-2.277672	-2.928848	-2.112576
H	-3.898513	-3.617577	-2.083072
H	-1.799263	2.316453	-0.598873
H	-1.697608	3.754318	-2.568479
H	-1.817310	2.059835	-3.059481
H	-3.229253	3.114061	-3.167629
H	-2.761304	4.549919	-0.392856
H	-3.709486	3.437377	0.602692
H	-4.333982	3.985595	-0.951263
H	2.602326	-1.077134	1.676810
H	1.534728	-2.307268	1.026882
H	2.165411	0.478027	-2.689343
H	3.498895	2.475595	-3.257287
H	4.651094	3.757846	-1.510259
H	3.176553	1.050781	1.456459
H	2.304524	-3.727776	-0.926530
H	4.223303	-4.948056	-1.869265
H	6.537620	-4.280832	-1.277224
H	6.907701	-2.380517	0.271399
H	4.989742	-1.153581	1.208235
H	5.752803	4.565436	1.860615
H	6.222769	3.998264	0.241203
H	4.797687	5.057448	0.442833

B3LYP energy = -1507.78686433 a.u.

(5*S*,1'*R*)-**9b**, Conf. M

C	-0.298976	-1.263687	-1.401083
C	-1.461422	-0.234214	-1.334299
N	-0.996563	0.663181	-0.204064
C	0.086261	0.143730	0.347831
N	0.536633	-0.941404	-0.226071
C	-1.822950	0.413440	-2.700909
C	-0.654552	1.173741	-3.343245
C	-3.035278	1.349304	-2.552454
C	-2.236340	-0.751899	-3.626428
C	1.767632	-1.701834	0.131218
C	-1.772138	1.694909	0.466373
C	-2.874828	1.307333	1.253613
C	-3.639715	2.317865	1.837515
C	-3.302698	3.654146	1.688155
C	-2.170680	4.005416	0.970085
C	-1.376290	3.041515	0.346351
C	-0.107028	3.489510	-0.367667
C	-0.373323	4.574232	-1.424196
C	0.946959	3.973129	0.646051
C	-3.231085	-0.144454	1.557751
C	-3.034411	-0.450409	3.054266
C	-4.654633	-0.508948	1.106857
C	1.454405	-2.851675	1.114513
C	2.870673	-0.767087	0.604631
C	3.045732	-0.434857	1.952658
C	4.063284	0.438050	2.317221
C	4.918160	0.989839	1.367427
C	4.747935	0.655864	0.022160
C	3.724056	-0.221754	-0.347917
C	0.497558	-3.893829	0.582422
C	-0.812226	-3.972602	1.060059
C	-1.697310	-4.927279	0.564224
C	-1.281161	-5.820067	-0.417899
C	0.025953	-5.757778	-0.895226
C	0.907180	-4.804302	-0.396977
O	5.520092	1.121946	-0.993963
C	6.605864	1.998952	-0.685374
H	-0.649239	-2.290209	-1.341354
H	0.307688	-1.150873	-2.299550
H	-2.365175	-0.731554	-0.981984
H	0.559106	0.586082	1.212763
H	0.213908	0.536370	-3.518207
H	-0.336073	2.021658	-2.740266
H	-0.966457	1.561405	-4.314609
H	-2.821641	2.217025	-1.930649
H	-3.889810	0.824786	-2.119322
H	-3.335885	1.712821	-3.536830
H	-1.408608	-1.432770	-3.834718
H	-3.056782	-1.331391	-3.196528
H	-2.576818	-0.352006	-4.582546
H	2.090965	-2.140644	-0.813606
H	-4.501439	2.053041	2.436231
H	-3.910322	4.421370	2.151726
H	-1.894314	5.048981	0.894849
H	0.316890	2.628365	-0.882645
H	0.545946	4.795398	-1.970418
H	-1.128546	4.260130	-2.146107
H	-0.715070	5.504579	-0.967326
H	1.870768	4.241430	0.129291
H	1.183818	3.203114	1.383035
H	0.596237	4.854077	1.187429
H	-2.542936	-0.796503	1.021050
H	-3.233260	-1.505972	3.251347
H	-2.013445	-0.230742	3.373384
H	-3.712617	0.137876	3.674795
H	-4.844435	-1.569787	1.282445

H	-4.804923	-0.307627	0.044929
H	-5.405412	0.056677	1.661667
H	2.410948	-3.319373	1.358098
H	1.058471	-2.437064	2.042782
H	2.411107	-0.857132	2.719851
H	4.202408	0.691497	3.360796
H	5.704541	1.660467	1.682005
H	3.619392	-0.474999	-1.395921
H	-1.140878	-3.287961	1.834055
H	-2.707735	-4.977862	0.951078
H	-1.965692	-6.566159	-0.801730
H	0.361843	-6.459361	-1.648941
H	1.926566	-4.776285	-0.766207
H	7.079073	2.227594	-1.636496
H	6.249223	2.923627	-0.225098
H	7.329730	1.514089	-0.026130

B3LYP energy = -1507.78686190 a.u.

(5*S*,1'*R*)-**9b**, Conf. N

C	0.142261	-0.073124	-1.789278
C	1.690714	-0.000985	-1.645278
N	1.855899	-0.119193	-0.144668
C	0.671472	-0.287591	0.415194
N	-0.338018	-0.317485	-0.415321
C	2.465041	-1.015139	-2.536087
C	2.064461	-2.473964	-2.272454
C	3.982208	-0.845490	-2.347608
C	2.130495	-0.650407	-3.998558
C	-1.753699	-0.551027	-0.032572
C	3.038519	0.228219	0.625790
C	3.435154	1.579306	0.671375
C	4.612738	1.884193	1.355525
C	5.340997	0.902590	2.009272
C	4.887958	-0.407281	2.013334
C	3.729887	-0.778509	1.328265
C	3.247937	-2.220664	1.430495
C	4.327892	-3.241865	1.038180
C	2.715316	-2.517359	2.845338
C	2.617320	2.721739	0.077158
C	2.148044	3.691988	1.176849
C	3.377995	3.476116	-1.025876
C	-2.261141	-1.877784	-0.643891
C	-2.601615	0.673435	-0.341024
C	-3.034485	0.970509	-1.638498
C	-3.787887	2.113439	-1.866646
C	-4.130114	2.972828	-0.825115
C	-3.704229	2.675983	0.470020
C	-2.939131	1.527329	0.701145
C	-3.548918	-2.355042	-0.009058
C	-3.523685	-2.992372	1.235119
C	-4.698620	-3.433233	1.834588
C	-5.921248	-3.245127	1.195299
C	-5.958199	-2.616631	-0.045132
C	-4.780239	-2.175368	-0.641533
O	-3.977199	3.431755	1.566429
C	-4.763526	4.614287	1.406578
H	-0.285153	0.855888	-2.162080
H	-0.173959	-0.885480	-2.438499
H	2.041023	0.990247	-1.925802
H	0.547603	-0.377179	1.485342
H	0.999255	-2.652484	-2.433346
H	2.311424	-2.790021	-1.259602
H	2.604790	-3.129110	-2.957792
H	4.310977	-1.114649	-1.345953
H	4.293366	0.183991	-2.538666
H	4.511319	-1.487441	-3.054346
H	1.074684	-0.790135	-4.238169
H	2.395879	0.385765	-4.221238
H	2.702220	-1.291376	-4.671095
H	-1.728952	-0.674656	1.049922
H	4.952038	2.911158	1.397649
H	6.250052	1.163141	2.536961
H	5.443298	-1.157497	2.560624
H	2.415961	-2.351913	0.740016
H	3.907805	-4.249644	1.049343
H	4.720788	-3.051628	0.038420
H	5.166987	-3.229271	1.735742
H	2.310272	-3.530478	2.891540
H	1.925713	-1.821957	3.138396
H	3.511634	-2.440941	3.588365
H	1.712095	2.309244	-0.367939
H	1.508307	4.464204	0.744600
H	1.578421	3.172002	1.949470
H	2.991188	4.189395	1.659050
H	2.740686	4.248471	-1.461152
H	3.699553	2.809370	-1.828180
H	4.268227	3.966756	-0.627869
H	-2.396077	-1.771291	-1.720971
H	-1.481836	-2.628679	-0.492053
H	-2.799104	0.316947	-2.467662
H	-4.124545	2.342746	-2.870056
H	-4.725268	3.850553	-1.030737
H	-2.625589	1.318569	1.716840
H	-2.575575	-3.158121	1.736083
H	-4.659186	-3.930454	2.796058
H	-6.836855	-3.590968	1.658709
H	-6.904103	-2.471531	-0.552458
H	-4.821730	-1.690465	-1.609369
H	-4.854760	5.043862	2.400601
H	-4.270634	5.330836	0.744731
H	-5.757838	4.378944	1.019584

B3LYP energy = -1507.78682900 a.u.

(5*S*,1'*R*)-**9b**, Conf. O

C	-0.631677	1.289702	1.561250
C	-1.660153	0.140469	1.351124
N	-0.996805	-0.651769	0.242802
C	0.144820	-0.075468	-0.089693
N	0.443151	0.998412	0.593119
C	-2.049544	-0.617617	2.653247

C	-3.094096	-1.707881	2.357255
C	-2.709264	0.428517	3.578110
C	-0.843505	-1.239395	3.371624
C	1.687110	1.813168	0.493330
C	-1.603278	-1.693380	-0.569800
C	-2.653855	-1.343266	-1.441145
C	-3.262467	-2.368477	-2.166487
C	-2.820932	-3.678720	-2.072278
C	-1.740751	-3.986579	-1.260626
C	-1.105261	-3.009645	-0.492081
C	0.109951	-3.409501	0.336010
C	-0.180441	-4.584276	1.284482
C	1.310171	-3.734666	-0.572928
C	-3.119318	0.089992	-1.678663
C	-2.903843	0.505142	-3.145448
C	-4.585149	0.302736	-1.264793
C	1.608470	2.827142	-0.668271
C	2.918103	0.917781	0.487981
C	3.422533	0.467268	1.714660
C	4.523700	-0.374719	1.741422
C	5.145467	-0.784460	0.563203
C	4.649400	-0.332545	-0.659250
C	3.537019	0.518672	-0.689814
C	0.436058	3.776652	-0.575803
C	-0.672453	3.623784	-1.410883
C	-1.761749	4.486389	-1.316027
C	-1.755071	5.518586	-0.383298
C	-0.650860	5.686850	0.448984
C	0.434827	4.823060	0.351473
O	5.170582	-0.655119	-1.872514
C	6.319352	-1.504137	-1.923414
H	-1.054711	2.270603	1.354824
H	-0.209604	1.296919	2.564574
H	-2.580647	0.549118	0.938895
H	0.778077	-0.469254	-0.871245
H	-2.698953	-2.503915	1.728933
H	-3.975940	-1.294005	1.863748
H	-3.423147	-2.158294	3.295667
H	-3.573015	0.896218	3.099560
H	-2.018529	1.218331	3.879744
H	-3.058439	-0.061771	4.488012
H	-0.369696	-2.016056	2.773818
H	-0.083295	-0.501499	3.634554
H	-1.175389	-1.701465	4.302835
H	1.699718	2.376721	1.426321
H	-4.082717	-2.132838	-2.832203
H	-3.306177	-4.457255	-2.647742
H	-1.381729	-5.006682	-1.222486
H	0.397170	-2.560783	0.954681
H	0.688122	-4.773700	1.918420
H	-1.033919	-4.379374	1.932354
H	-0.391400	-5.503020	0.734781
H	2.189177	-3.965521	0.032477
H	1.562428	-2.898740	-1.228618
H	1.100847	-4.599531	-1.205727
H	-2.505166	0.761464	-1.079895
H	-3.168194	1.556443	-3.278096
H	-1.863392	0.374616	-3.449092
H	-3.525395	-0.081038	-3.824447
H	-4.866817	1.349233	-1.398142
H	-4.755968	0.035113	-0.220598
H	-5.259024	-0.302298	-1.874244
H	2.545868	3.388008	-0.657214
H	1.566778	2.298690	-1.621690
H	2.959335	0.782277	2.642135
H	4.916569	-0.717016	2.690774
H	6.005262	-1.436537	0.611522
H	3.197287	0.859271	-1.658731
H	-0.679111	2.830231	-2.149711
H	-2.610307	4.356788	-1.976615
H	-2.598605	6.193767	-0.311580
H	-0.631602	6.496412	1.168239
H	1.294699	4.970372	0.995553
H	7.166507	-1.054425	-1.400153
H	6.103863	-2.487595	-1.498532
H	6.560213	-1.610609	-2.977700

B3LYP energy = -1507.78675002 a.u.

(5*S*,1’*R*)-**9b**, Conf. P

C	0.695045	-0.110210	1.981068
C	-0.522335	-1.048677	1.746079
N	-0.955320	-0.627281	0.357944
C	-0.147134	0.317164	-0.091914
N	0.820542	0.650497	0.722361
C	-0.226863	-2.557184	1.987244
C	-1.494871	-3.399238	1.765394
C	0.167851	-2.685540	3.474695
C	0.916086	-3.093892	1.113549
C	1.912991	1.628498	0.530781
C	-2.226921	-0.925966	-0.280717
C	-2.246690	-1.720451	-1.444204
C	-3.490803	-2.018127	-2.002612
C	-4.666396	-1.549037	-1.438175
C	-4.617520	-0.729604	-0.321599
C	-3.402919	-0.378117	0.268975
C	-3.417090	0.619974	1.422461
C	-4.010331	1.968691	0.975744
C	-4.164901	0.080760	2.653546
C	-0.991486	-2.220523	-2.148738
C	-1.000262	-3.741957	-2.367984
C	-0.785349	-1.484544	-3.486149
C	1.720103	2.526239	-0.705123
C	3.267554	0.931490	0.522382
C	4.224588	1.294717	1.462014
C	5.483942	0.692397	1.437133
C	5.786466	-0.265602	0.487565
C	4.824773	-0.636078	-0.461318
C	3.564999	-0.038074	-0.442435
C	0.492995	3.416885	-0.711673
C	-0.323506	3.475822	-1.843793
C	-1.425564	4.326042	-1.889454

C	-1.727964	5.133485	-0.798674
C	-0.918956	5.088342	0.333708
C	0.182070	4.239673	0.375269
O	5.214503	-1.583671	-1.355324
C	4.304147	-1.989769	-2.375379
H	0.534020	0.584496	2.805374
H	1.617711	-0.655608	2.164080
H	-1.329606	-0.777492	2.423121
H	-0.299957	0.786836	-1.050681
H	-1.827279	-3.390310	0.729133
H	-2.320522	-3.046181	2.386870
H	-1.295026	-4.436430	2.040541
H	-0.624549	-2.317080	4.130806
H	1.086753	-2.146974	3.713902
H	0.336463	-3.736215	3.715228
H	0.681589	-3.034433	0.051534
H	1.856998	-2.567322	1.283032
H	1.090585	-4.144719	1.350282
H	1.878554	2.258521	1.421942
H	-3.538610	-2.627351	-2.895773
H	-5.620595	-1.804895	-1.881707
H	-5.539390	-0.335951	0.086674
H	-2.389416	0.824358	1.721905
H	-3.940770	2.694025	1.789120
H	-3.477720	2.374208	0.113975
H	-5.063490	1.871818	0.706297
H	-4.108389	0.800016	3.473107
H	-3.748486	-0.865655	3.003179
H	-5.220670	-0.086075	2.431905
H	-0.133801	-1.989632	-1.518005
H	-0.054108	-4.060698	-2.810296
H	-1.134888	-4.284961	-1.431580
H	-1.798019	-4.044794	-3.048105
H	0.146093	-1.807835	-3.955866
H	-0.741083	-0.401541	-3.350986
H	-1.600355	-1.695280	-4.181218
H	2.616822	3.149807	-0.745904
H	1.747988	1.921527	-1.614500
H	3.997533	2.045211	2.209343
H	6.230801	0.974807	2.168393
H	6.757902	-0.741688	0.457526
H	2.818225	-0.319705	-1.171714
H	-0.087652	2.864113	-2.708445
H	-2.042526	4.359184	-2.778913
H	-2.582974	5.796979	-0.831277
H	-1.139999	5.722156	1.183689
H	0.809902	4.236340	1.258785
H	4.023344	-1.148544	-3.014555
H	4.834662	-2.730440	-2.967739
H	3.406886	-2.445271	-1.948002

B3LYP energy = -1507.78625130 a.u.

(5*S*,1’*S*)-**10a**, Conf. A

C	3.898102	0.908331	0.571531
C	5.094507	0.195016	0.604932
C	5.424727	-0.654657	-0.452190
C	4.546064	-0.774796	-1.535741
C	3.361966	-0.061026	-1.550296
C	3.011436	0.792826	-0.496696
C	1.690485	1.542399	-0.558998
C	1.736845	2.965349	0.030369
C	0.485537	3.779893	-0.210749
C	0.138804	4.186014	-1.503699
C	-1.000645	4.950322	-1.729744
C	-1.813722	5.326152	-0.663105
C	-1.477225	4.933583	0.627734
C	-0.336220	4.165963	0.849682
N	0.599379	0.743921	0.060662
C	-0.351868	0.128814	-0.593603
N	-1.121278	-0.659330	0.134880
C	-2.354002	-1.228737	-0.385396
C	-2.291119	-2.383618	-1.191248
C	-0.988487	-3.030285	-1.646978
C	-0.930887	-4.533111	-1.327379
C	-0.753647	-2.792996	-3.150932
C	-3.498004	-2.929340	-1.631949
C	-4.715084	-2.357819	-1.296469
C	-4.747521	-1.192343	-0.547407
C	-3.574013	-0.587020	-0.095687
C	-3.677002	0.758212	0.614926
C	-4.264069	1.825937	-0.326580
C	-4.490797	0.675587	1.917000
C	-0.698422	-0.564410	1.587303
C	-0.452286	-1.881688	2.377287
C	-0.052455	-1.452258	3.806191
C	0.667397	-2.747924	1.782063
C	-1.747222	-2.705503	2.480837
C	0.550891	0.357977	1.482860
C	7.508331	-1.303077	0.543623
O	6.562516	-1.391011	-0.524145
H	3.679294	1.568839	1.400978
H	5.756109	0.317119	1.450191
H	4.815318	-1.425451	-2.357661
H	2.702926	-0.159395	-2.405516
H	1.404333	1.624193	-1.607977
H	1.947715	2.924368	1.099893
H	2.595012	3.458659	-0.431338
H	0.774458	3.920687	-2.341709
H	-1.247592	5.263261	-2.736859
H	-2.697580	5.926901	-0.837458
H	-2.096951	5.230862	1.464879
H	-0.074155	3.881612	1.862842
H	-0.507642	0.261785	-1.654730
H	-0.166160	-2.553550	-1.115222
H	0.053662	-4.930153	-1.582768
H	-1.111445	-4.728887	-0.269562
H	-1.669634	-5.094617	-1.901819
H	0.206995	-3.215728	-3.452728
H	-0.749790	-1.730106	-3.401474
H	-1.532034	-3.269081	-3.750556

H	-3.483530	-3.817735	-2.249468
H	-5.638360	-2.808860	-1.638372
H	-5.701153	-0.730480	-0.326600
H	-2.674134	1.096828	0.872542
H	-4.261640	2.800663	0.164826
H	-3.681165	1.910001	-1.245604
H	-5.293926	1.591256	-0.601690
H	-4.496351	1.645526	2.418532
H	-4.079752	-0.061779	2.608861
H	-5.528317	0.400468	1.718186
H	-1.496202	-0.034308	2.106664
H	0.889921	-0.901429	3.830246
H	-0.824392	-0.831970	4.267915
H	0.075481	-2.339495	4.428123
H	0.434848	-3.080324	0.772304
H	0.798203	-3.639671	2.397363
H	1.629901	-2.233790	1.756318
H	-2.080497	-3.084394	1.516390
H	-2.559320	-2.117870	2.914086
H	-1.577810	-3.563926	3.133499
H	0.460572	1.242497	2.109499
H	1.472053	-0.160425	1.740931
H	7.071430	-1.638842	1.487367
H	8.325615	-1.964122	0.268258
H	7.886476	-0.283799	0.653858

B3LYP energy = -1507.78868219 a.u.

(5*S*,1’*S*)-**10a**, Conf. B

C	-3.821458	1.135705	-0.861398
C	-5.045894	0.503260	-0.989517
C	-5.517572	-0.336651	0.025713
C	-4.740356	-0.531260	1.169679
C	-3.509716	0.109850	1.279220
C	-3.027289	0.948744	0.276622
C	-1.672091	1.613374	0.450188
C	-1.600963	3.056174	-0.088155
C	-0.318612	3.785089	0.243032
C	0.563371	4.176410	-0.765856
C	1.732422	4.870915	-0.464151
C	2.036850	5.183628	0.856144
C	1.164056	4.801218	1.871980
C	-0.003304	4.109814	1.566356
N	-0.592023	0.771924	-0.130187
C	0.315726	0.126990	0.555457
N	1.088916	-0.679892	-0.148501
C	2.287303	-1.285364	0.409399
C	2.164218	-2.427843	1.225821
C	0.829471	-3.026211	1.654557
C	0.727287	-4.527664	1.339880
C	0.569912	-2.774727	3.152214
C	3.339668	-3.010226	1.703014
C	4.584181	-2.486156	1.392002
C	4.676128	-1.331810	0.630895
C	3.536528	-0.690395	0.143368
C	3.707244	0.642098	-0.578306
C	4.320064	1.695372	0.363156
C	4.542085	0.512696	-1.863402
C	0.714991	-0.577757	-1.613313
C	0.432806	-1.891266	-2.397581
C	1.692883	-2.771522	-2.456777
C	0.091181	-1.460914	-3.841097
C	-0.739368	-2.700552	-1.824103
C	-0.493953	0.401663	-1.554034
C	-7.283850	-1.754444	0.817908
O	-6.730753	-0.905610	-0.189921
H	-3.494914	1.788624	-1.660672
H	-5.659006	0.653414	-1.868614
H	-5.080654	-1.165531	1.975223
H	-2.921524	-0.047235	2.177143
H	-1.451259	1.643212	1.517757
H	-1.757280	3.065360	-1.167513
H	-2.451840	3.585244	0.347216
H	0.326331	3.954819	-1.800659
H	2.399377	5.174170	-1.261924
H	2.942365	5.728222	1.092733
H	1.386669	5.052764	2.901740
H	-0.684422	3.838478	2.365611
H	0.435893	0.251969	1.622080
H	0.036096	-2.524038	1.102291
H	-0.276160	-4.889622	1.573226
H	0.926223	-4.734910	0.287618
H	1.432423	-5.110969	1.934430
H	-0.412571	-3.159631	3.433770
H	0.601541	-1.711821	3.400233
H	1.315916	-3.278054	3.770378
H	3.278318	-3.890559	2.329203
H	5.482122	-2.965289	1.761967
H	5.650638	-0.906369	0.429009
H	2.724321	1.019161	-0.858267
H	4.355519	2.666131	-0.134579
H	3.730806	1.805219	1.275277
H	5.338488	1.428047	0.650500
H	4.603276	1.477794	-2.370535
H	4.111265	-0.209223	-2.559674
H	5.561258	0.190573	-1.641444
H	1.551838	-0.091326	-2.112817
H	2.541954	-2.223074	-2.869931
H	1.981173	-3.156620	-1.480318
H	1.505601	-3.627123	-3.108220
H	-0.830988	-0.879322	-3.898012
H	-0.047924	-2.348685	-4.459794
H	0.897101	-0.870637	-4.283703
H	-1.676461	-2.141014	-1.824313
H	-0.545706	-3.035475	-0.806859
H	-0.898207	-3.589857	-2.436418
H	-0.331173	1.289412	-2.162055
H	-1.424975	-0.067263	-1.864621
H	-8.247241	-2.078420	0.433483
H	-6.650061	-2.627548	0.991748
H	-7.430064	-1.211749	1.754987

B3LYP energy = -1507.78850967 a.u.

(5*S*,1'*S*)-**10a**, Conf. C

C	3.612749	0.725983	1.130481
C	4.484298	1.795970	0.935170
C	4.318544	2.624012	-0.175117
C	3.277691	2.365911	-1.077545
C	2.426663	1.296432	-0.870606
C	2.580430	0.456271	0.239145
C	1.667776	-0.736318	0.462080
C	1.792412	-1.833518	-0.616612
C	3.147396	-2.506119	-0.600331
C	3.434200	-3.486393	0.353461
C	4.678293	-4.107658	0.383435
C	5.654513	-3.758551	-0.545884
C	5.377358	-2.787864	-1.503328
C	4.131833	-2.166629	-1.529407
N	0.261558	-0.290688	0.611486
C	-0.748750	-0.489253	-0.194515
N	-1.898415	0.019960	0.213294
C	-3.058169	0.125485	-0.656243
C	-3.838114	-1.019114	-0.916665
C	-3.475713	-2.417528	-0.432101
C	-4.628624	-3.109621	0.312308
C	-2.991106	-3.289668	-1.605907
C	-4.972100	-0.860420	-1.714743
C	-5.320345	0.374896	-2.237726
C	-4.507725	1.474504	-2.011009
C	-3.348613	1.374260	-1.240344
C	-2.436547	2.592386	-1.135313
C	-1.899125	2.996522	-2.520631
C	-3.126149	3.783564	-0.450146
C	-1.703243	0.706695	1.548126
C	-2.569305	0.210365	2.742888
C	-2.322856	-1.266090	3.086330
C	-4.064214	0.439387	2.461767
C	-2.181369	1.087721	3.953050
C	-0.170399	0.536266	1.754692
C	6.196964	4.001331	0.398631
O	5.102251	3.693800	-0.466824
H	3.755886	0.090101	1.996642
H	5.277900	1.968602	1.647375
H	3.160094	3.014311	-1.936180
H	1.635436	1.117105	-1.589865
H	1.924793	-1.183953	1.424638
H	1.020299	-2.587053	-0.439126
H	1.613962	-1.403652	-1.604066
H	2.675679	-3.774878	1.073044
H	4.882561	-4.869249	1.126021
H	6.622209	-4.244298	-0.526994
H	6.129325	-2.514969	-2.233472
H	3.924650	-1.412452	-2.279424
H	-0.660799	-1.017728	-1.131198
H	-2.646328	-2.332166	0.268634
H	-4.293512	-4.072070	0.704273
H	-4.987088	-2.510177	1.150263
H	-5.475445	-3.301771	-0.348485
H	-2.660423	-4.264634	-1.241620
H	-2.159514	-2.826479	-2.141478
H	-3.792647	-3.456619	-2.328277
H	-5.592252	-1.720737	-1.929660
H	-6.213394	0.475772	-2.841827
H	-4.765029	2.424915	-2.460334
H	-1.564798	2.327391	-0.537731
H	-1.186798	3.818061	-2.419632
H	-1.390778	2.163306	-3.009849
H	-2.701874	3.331688	-3.179818
H	-2.425791	4.614426	-0.343677
H	-3.495923	3.521701	0.542785
H	-3.975719	4.138992	-1.036064
H	-1.940279	1.758554	1.402877
H	-2.622622	-1.930393	2.277127
H	-1.277348	-1.472420	3.324154
H	-2.909861	-1.538302	3.965053
H	-4.269971	1.486497	2.228869
H	-4.644721	0.178851	3.348674
H	-4.431440	-0.166695	1.636157
H	-2.339052	2.148116	3.742123
H	-1.142035	0.951985	4.257566
H	-2.806155	0.822720	4.807185
H	0.364503	1.484890	1.738426
H	0.073371	0.018332	2.680071
H	6.911998	3.176048	0.439105
H	6.677245	4.875417	-0.032886
H	5.849757	4.238315	1.407397

B3LYP energy = -1507.78785478 a.u.

(5*S*,1'*S*)-**10a**, Conf. D

C	2.488857	1.353864	-0.587136
C	3.350026	2.443546	-0.668943
C	4.363023	2.596892	0.282060
C	4.492553	1.652519	1.305347
C	3.623296	0.576692	1.372846
C	2.608454	0.407435	0.427539
C	1.684169	-0.794773	0.502822
C	1.842011	-1.786768	-0.669872
C	3.193909	-2.465454	-0.676046
C	3.432011	-3.565447	0.152094
C	4.672344	-4.195037	0.159821
C	5.693690	-3.733681	-0.666237
C	5.465736	-2.641844	-1.497963
C	4.223974	-2.012977	-1.501957
N	0.274946	-0.355585	0.643649
C	-0.711556	-0.488282	-0.204156
N	-1.876026	-0.024237	0.215334
C	-3.006666	0.157548	-0.679400
C	-3.785521	-0.956531	-1.050794
C	-3.452874	-2.391555	-0.658776
C	-4.636638	-3.122646	-0.005520
C	-2.937959	-3.183587	-1.875490
C	-4.890581	-0.725109	-1.871562

C	-5.211453	0.550041	-2.310000
C	-4.398424	1.621390	-1.974451
C	-3.266734	1.449766	-1.176502
C	-2.341216	2.642125	-0.954542
C	-1.710235	3.094094	-2.284862
C	-3.046498	3.816582	-0.257536
C	-1.720612	0.554366	1.605439
C	-2.620739	-0.031373	2.732064
C	-2.377494	-1.527351	2.977506
C	-4.106505	0.212741	2.417547
C	-2.278277	0.758625	4.013888
C	-0.192905	0.371802	1.840074
C	5.180381	4.613778	-0.727428
O	5.256370	3.619720	0.295747
H	1.714434	1.255949	-1.339717
H	3.225955	3.156459	-1.471018
H	5.280168	1.778442	2.036824
H	3.741197	-0.146562	2.171336
H	1.904540	-1.335042	1.426425
H	1.064475	-2.550581	-0.586195
H	1.693500	-1.265405	-1.617707
H	2.638204	-3.940412	0.789064
H	4.838487	-5.050064	0.803608
H	6.658510	-4.225416	-0.665075
H	6.253590	-2.279900	-2.147047
H	4.055253	-1.163893	-2.153674
H	-0.594980	-0.929217	-1.182578
H	-2.645464	-2.368450	0.071833
H	-4.320939	-4.111274	0.333759
H	-5.023485	-2.577442	0.856307
H	-5.458954	-3.264954	-0.708660
H	-2.649896	-4.193081	-1.574744
H	-2.069700	-2.706343	-2.335012
H	-3.708895	-3.269820	-2.643901
H	-5.510651	-1.559543	-2.172222
H	-6.083500	0.705370	-2.932977
H	-4.633229	2.605935	-2.357861
H	-1.515407	2.329512	-0.316623
H	-0.996255	3.900851	-2.106018
H	-1.182438	2.272678	-2.773584
H	-2.466006	3.465235	-2.979305
H	-2.336074	4.624647	-0.071616
H	-3.480375	3.519132	0.698813
H	-3.850926	4.219262	-0.875498
H	-1.958226	1.614076	1.536659
H	-2.609428	-2.129909	2.100135
H	-1.349010	-1.741495	3.274774
H	-3.020962	-1.871966	3.788793
H	-4.306834	1.271392	2.239492
H	-4.713866	-0.099902	3.268783
H	-4.446226	-0.344280	1.546951
H	-2.441187	1.830104	3.875460
H	-1.246905	0.609434	4.337986
H	-2.924756	0.426727	4.827602
H	0.336585	1.320807	1.914742
H	0.030154	-0.218306	2.726586
H	4.224585	5.142953	-0.695221
H	5.986697	5.312194	-0.520250
H	5.325550	4.174740	-1.717530

B3LYP energy = -1507.78755569 a.u.

(5*S*,1'*S*)-**10a**, Conf. E

C	-2.819910	-1.129501	1.116998
C	-3.614458	-2.272316	1.089667
C	-4.161829	-2.700157	-0.121314
C	-3.906021	-1.967974	-1.286771
C	-3.119161	-0.831034	-1.238749
C	-2.555506	-0.391624	-0.034246
C	-1.666605	0.834498	0.053337
C	-2.175661	2.081696	-0.703418
C	-3.433255	2.657285	-0.089559
C	-3.363321	3.385972	1.101629
C	-4.510007	3.918065	1.681220
C	-5.749328	3.731879	1.074225
C	-5.830766	3.013543	-0.114035
C	-4.680568	2.480953	-0.690229
N	-0.275050	0.516074	-0.368590
C	0.761124	0.476398	0.427218
N	1.887434	0.068945	-0.130984
C	3.180406	0.247795	0.509757
C	3.605085	-0.674028	1.486700
C	2.731528	-1.801867	2.023440
C	3.416622	-3.176006	1.950497
C	2.285075	-1.500833	3.466966
C	4.878288	-0.495390	2.030777
C	5.693155	0.553674	1.635133
C	5.228545	1.480940	0.715581
C	3.958231	1.365815	0.149705
C	3.458390	2.484912	-0.757983
C	3.327493	3.802945	0.027437
C	4.339820	2.671402	-2.003707
C	1.644968	-0.216328	-1.598134
C	2.060996	-1.612184	-2.144847
C	1.703773	-1.610119	-3.646800
C	1.329775	-2.770817	-1.451996
C	3.582111	-1.807175	-2.022477
C	0.124039	0.094748	-1.725315
C	-5.268693	-4.576043	0.884312
O	-4.949849	-3.795361	-0.269320
H	-2.408413	-0.807494	2.067297
H	-3.802110	-2.809048	2.008306
H	-4.342600	-2.302636	-2.218890
H	-2.957519	-0.280728	-2.157325
H	-1.574789	1.103875	1.105803
H	-1.381340	2.831828	-0.676131
H	-2.352188	1.842966	-1.752898
H	-2.401773	3.550776	1.576055
H	-4.435393	4.484776	2.601304
H	-6.643093	4.148324	1.521981
H	-6.789629	2.868548	-0.596418
H	-4.756860	1.926384	-1.617802

H	0.706248	0.767227	1.467042
H	1.830384	-1.857032	1.413807
H	2.721371	-3.954927	2.270386
H	3.745058	-3.412706	0.937534
H	4.288315	-3.225963	2.605412
H	1.608060	-2.280306	3.822735
H	1.768430	-0.541479	3.541166
H	3.141311	-1.465575	4.143417
H	5.235983	-1.189151	2.780285
H	6.682330	0.662197	2.062268
H	5.855209	2.322252	0.448682
H	2.455368	2.235056	-1.101844
H	2.900195	4.579722	-0.610205
H	2.679766	3.682048	0.898184
H	4.298315	4.156411	0.379114
H	3.920391	3.445064	-2.650104
H	4.420321	1.750514	-2.584478
H	5.351103	2.979549	-1.732491
H	2.216177	0.521023	-2.159640
H	0.629793	-1.524136	-3.821644
H	2.202944	-0.793835	-4.174414
H	2.031873	-2.547266	-4.098883
H	1.556521	-2.818670	-0.387970
H	1.646063	-3.716084	-1.896206
H	0.245614	-2.711971	-1.565835
H	3.912645	-1.857639	-0.986976
H	4.129268	-0.998552	-2.512026
H	3.868504	-2.741527	-2.508597
H	-0.077071	0.894449	-2.436110
H	-0.453488	-0.780302	-2.012797
H	-4.368432	-5.000005	1.336140
H	-5.809459	-3.982920	1.625725
H	-5.907288	-5.380040	0.528495

B3LYP energy = -1507.78713539 a.u.

(5*S*,1’*S*)-**10a**, Conf. F

C	-2.347831	1.116379	1.120972
C	-2.737999	2.426491	1.331746
C	-3.080401	3.245099	0.248106
C	-3.020138	2.727260	-1.046998
C	-2.618669	1.408355	-1.240359
C	-2.276775	0.581817	-0.172580
C	-1.841188	-0.842453	-0.440397
C	-2.493683	-1.905177	0.475284
C	-3.985336	-2.027044	0.254397
C	-4.478446	-2.663140	-0.888743
C	-5.847188	-2.776874	-1.107468
C	-6.747475	-2.258186	-0.180415
C	-6.268664	-1.629021	0.964041
C	-4.897764	-1.514627	1.177969
N	-0.355030	-0.976216	-0.402322
C	0.518594	-0.094137	0.008859
N	1.783528	-0.458773	-0.110360
C	2.872303	0.494554	0.029373
C	3.319403	0.850745	1.317128
C	2.662656	0.367823	2.605067
C	3.666051	-0.262015	3.584884
C	1.891437	1.513634	3.286788
C	4.391198	1.740601	1.405092
C	4.994400	2.259466	0.270407
C	4.504316	1.931711	-0.983794
C	3.420765	1.065421	-1.136113
C	2.860623	0.841696	-2.537738
C	2.368386	2.164535	-3.153230
C	3.875722	0.156870	-3.468114
C	1.859747	-1.825805	-0.751678
C	2.643152	-2.938967	0.002164
C	4.122307	-2.546650	0.157260
C	2.582766	-4.194190	-0.895111
C	2.045014	-3.272080	1.376987
C	0.351214	-2.140743	-0.973253
C	-3.836346	5.395365	-0.499764
O	-3.456045	4.510966	0.557069
H	-2.099967	0.509033	1.982919
H	-2.794879	2.835254	2.332238
H	-3.285891	3.330152	-1.903048
H	-2.583928	1.019440	-2.251593
H	-2.107281	-1.084131	-1.472274
H	-2.028234	-2.871660	0.271767
H	-2.282181	-1.668923	1.518703
H	-3.787310	-3.083188	-1.611603
H	-6.210889	-3.276756	-1.996901
H	-7.813657	-2.348462	-0.347304
H	-6.961536	-1.227741	1.693474
H	-4.536534	-1.024572	2.074136
H	0.232074	0.870655	0.400038
H	1.935404	-0.401800	2.350119
H	3.136664	-0.662767	4.451704
H	4.224661	-1.077578	3.123660
H	4.386319	0.471669	3.951015
H	1.377771	1.145063	4.177291
H	1.145371	1.952942	2.621370
H	2.568113	2.312451	3.596849
H	4.757362	2.033444	2.380292
H	5.834015	2.936931	0.364372
H	4.956728	2.374827	-1.861464
H	1.986157	0.194994	-2.468150
H	1.900824	1.973931	-4.121571
H	1.634738	2.654814	-2.510803
H	3.192162	2.862016	-3.314078
H	3.427102	-0.025772	-4.446737
H	4.216312	-0.799319	-3.066671
H	4.756387	0.783985	-3.618731
H	2.353719	-1.696644	-1.713141
H	4.574609	-2.307949	-0.807554
H	4.260485	-1.690021	0.813606
H	4.676894	-3.384412	0.583835
H	1.565834	-4.567394	-1.030806
H	3.163426	-4.995805	-0.436440
H	3.009081	-3.997920	-1.881768
H	1.017399	-3.634795	1.310047

H	2.058182	-2.415665	2.049827
H	2.631553	-4.064018	1.845671
H	0.093036	-2.234519	-2.028220
H	0.044837	-3.048991	-0.461652
H	-4.705519	5.013531	-1.040408
H	-4.093613	6.334991	-0.018513
H	-3.009845	5.557601	-1.195891

B3LYP energy = -1507.78707965 a.u.

(5*S*,1’*S*)-**10a**, Conf. G

C	-2.413527	1.301596	0.800090
C	-2.797851	2.639487	0.846881
C	-3.075652	3.320825	-0.340225
C	-2.961601	2.646176	-1.561800
C	-2.575231	1.318959	-1.588646
C	-2.292428	0.619469	-0.408872
C	-1.855071	-0.827575	-0.492016
C	-2.483955	-1.766746	0.564058
C	-3.968908	-1.965584	0.355279
C	-4.429547	-2.758564	-0.699865
C	-5.791383	-2.945703	-0.910404
C	-6.717485	-2.343823	-0.062414
C	-6.271064	-1.558147	0.995041
C	-4.906814	-1.370665	1.200499
N	-0.368784	-0.950374	-0.463474
C	0.502252	-0.080198	-0.024493
N	1.765299	-0.463028	-0.103428
C	2.864080	0.472886	0.069832
C	3.272185	0.827100	1.371192
C	2.565901	0.357574	2.637935
C	3.524448	-0.293934	3.647963
C	1.797715	1.517710	3.298626
C	4.355729	1.698494	1.493241
C	5.007679	2.201091	0.378369
C	4.556838	1.875377	-0.891057
C	3.464246	1.027382	-1.077991
C	2.949874	0.806200	-2.497649
C	2.492544	2.133302	-3.130846
C	3.987185	0.106594	-3.391536
C	1.841323	-1.835330	-0.735843
C	2.583624	-2.956850	0.046611
C	4.063517	-2.587309	0.245084
C	2.529951	-4.215142	-0.846903
C	1.938871	-3.274962	1.404018
C	0.336499	-2.128141	-1.005041
C	-3.618003	5.362673	0.797781
O	-3.462477	4.618656	-0.412677
H	-2.213250	0.797146	1.737284
H	-2.885163	3.129834	1.805447
H	-3.188944	3.179196	-2.475846
H	-2.500460	0.811455	-2.543555
H	-2.133008	-1.202885	-1.479337
H	-1.984778	-2.736274	0.504168
H	-2.287993	-1.378341	1.564239
H	-3.717294	-3.243345	-1.358937
H	-6.129410	-3.566303	-1.731275
H	-7.778368	-2.490336	-0.222895
H	-6.984217	-1.090406	1.662729
H	-4.571563	-0.757294	2.028498
H	0.215586	0.889155	0.355570
H	1.831475	-0.396778	2.359391
H	2.959034	-0.686135	4.495778
H	4.081593	-1.119475	3.203194
H	4.246632	0.424648	4.039516
H	1.251637	1.158188	4.173414
H	1.080100	1.972848	2.612881
H	2.480668	2.302648	3.629668
H	4.693106	1.988939	2.479556
H	5.855387	2.864082	0.499106
H	5.048323	2.305820	-1.753962
H	2.066752	0.168782	-2.457031
H	2.059690	1.946810	-4.115910
H	1.739489	2.630081	-2.516372
H	3.328016	2.823262	-3.260260
H	3.568304	-0.076081	-4.383254
H	4.303178	-0.851386	-2.974338
H	4.879453	0.722710	-3.516889
H	2.366893	-1.717580	-1.681867
H	4.548552	-2.359238	-0.706459
H	4.195163	-1.730235	0.902250
H	4.591850	-3.431813	0.691256
H	1.511371	-4.571805	-1.011717
H	3.082553	-5.024100	-0.366971
H	2.989181	-4.030878	-1.820986
H	0.906471	-3.617361	1.308593
H	1.950047	-2.417751	2.075763
H	2.495815	-4.077515	1.890427
H	0.111178	-2.222485	-2.067316
H	-0.003654	-3.028718	-0.500561
H	-2.673092	5.435114	1.341999
H	-3.936461	6.355969	0.493313
H	-4.381269	4.917053	1.440048

B3LYP energy = -1507.78706555 a.u.

(5*S*,1’*S*)-**10a**, Conf. H

C	-2.715099	-1.017751	1.409719
C	-3.476091	-2.164951	1.540024
C	-4.093679	-2.728918	0.417675
C	-3.940663	-2.116133	-0.827547
C	-3.173928	-0.958507	-0.938235
C	-2.544827	-0.392190	0.168426
C	-1.670218	0.845247	0.075764
C	-2.217772	1.991366	-0.803645
C	-3.438775	2.649819	-0.199099
C	-4.724083	2.360446	-0.659860
C	-5.839440	2.965430	-0.087035
C	-5.684556	3.870243	0.958152
C	-4.407135	4.171302	1.423078
C	-3.295170	3.567026	0.846353
N	-0.290454	0.489151	-0.356587

C	0.768941	0.515173	0.409060
N	1.877205	0.053564	-0.143512
C	3.185663	0.281323	0.449521
C	3.943413	1.383840	0.007243
C	3.417084	2.441169	-0.958076
C	3.256158	3.796503	-0.244500
C	4.294037	2.584550	-2.212560
C	5.222194	1.546806	0.541513
C	5.715499	0.682011	1.505853
C	4.922244	-0.351801	1.977479
C	3.641399	-0.576702	1.469800
C	2.795244	-1.684042	2.087081
C	3.498457	-3.051027	2.070565
C	2.377318	-1.312983	3.522533
C	1.592943	-0.345284	-1.576994
C	2.012092	-1.770196	-2.039373
C	1.322805	-2.891498	-1.248589
C	3.539625	-1.936086	-1.959285
C	1.602896	-1.877077	-3.524893
C	0.064336	-0.063456	-1.677073
C	-5.475824	-4.482627	-0.461806
O	-4.817433	-3.855332	0.640469
H	-2.252465	-0.593375	2.293714
H	-3.611517	-2.637779	2.504095
H	-4.414812	-2.520642	-1.709892
H	-3.088951	-0.500433	-1.915867
H	-1.543437	1.239044	1.084560
H	-1.422458	2.733057	-0.911613
H	-2.448968	1.625685	-1.804957
H	-4.856925	1.658630	-1.474432
H	-6.828924	2.730699	-0.459808
H	-6.551445	4.342225	1.403623
H	-4.276021	4.882349	2.229623
H	-2.304251	3.821286	1.207510
H	0.745953	0.897333	1.420326
H	2.420930	2.151033	-1.289896
H	2.817774	4.529268	-0.925410
H	2.606319	3.711150	0.628865
H	4.218796	4.186341	0.091167
H	3.850206	3.307366	-2.900272
H	4.403417	1.635846	-2.741084
H	5.294594	2.940508	-1.961012
H	5.833435	2.376959	0.211979
H	6.710649	0.828200	1.907119
H	5.302530	-0.995650	2.759638
H	1.881279	-1.781434	1.502874
H	2.822862	-3.821119	2.448434
H	3.806362	-3.337338	1.063985
H	4.386467	-3.054089	2.705146
H	1.723277	-2.083526	3.935927
H	1.844136	-0.360704	3.556334
H	3.248171	-1.226185	4.175387
H	2.135239	0.356598	-2.209134
H	1.585985	-2.870890	-0.192550
H	0.234732	-2.853029	-1.327007
H	1.636748	-3.858568	-1.645184
H	4.055376	-1.155758	-2.523112
H	3.823122	-2.897610	-2.391103
H	3.908434	-1.911278	-0.935885
H	2.063477	-1.088833	-4.125237
H	0.521448	-1.826990	-3.665174
H	1.936974	-2.836157	-3.923341
H	-0.171939	0.657431	-2.457934
H	-0.513103	-0.968593	-1.850418
H	-5.980317	-5.351355	-0.047487
H	-6.213871	-3.815677	-0.913918
H	-4.757602	-4.806491	-1.219261

B3LYP energy = -1507.78701487 a.u.

(5*S*,1'*S*)-**10a**, Conf. I

C	3.193161	1.349645	-1.367472
C	4.482398	0.858722	-1.285768
C	5.081819	0.661811	-0.035101
C	4.366177	0.971034	1.122426
C	3.067382	1.467242	1.020976
C	2.457134	1.660540	-0.216021
C	1.039741	2.182638	-0.368634
C	0.620608	3.195203	0.719708
C	-0.671647	3.935149	0.452466
C	-1.785515	3.752913	1.273998
C	-2.967622	4.452660	1.041507
C	-3.051326	5.347199	-0.019860
C	-1.944648	5.542728	-0.843432
C	-0.765816	4.844760	-0.606413
N	0.069660	1.052822	-0.489985
C	0.136072	-0.100492	0.121428
N	-0.830837	-0.951961	-0.172713
C	-0.755787	-2.351467	0.214242
C	-1.167735	-2.728827	1.507450
C	-1.590301	-1.731266	2.578736
C	-2.940483	-2.083312	3.223438
C	-0.497914	-1.593646	3.656178
C	-1.124275	-4.086333	1.828726
C	-0.686262	-5.030616	0.913317
C	-0.234052	-4.627436	-0.333351
C	-0.233237	-3.282020	-0.704876
C	0.390143	-2.895351	-2.042383
C	1.885222	-3.261694	-2.073334
C	-0.351505	-3.519165	-3.236094
C	-1.710694	-0.356840	-1.252498
C	-3.252781	-0.392409	-1.058934
C	-3.727466	0.355990	0.194059
C	-3.759606	-1.845006	-1.019777
C	-3.853827	0.284554	-2.310741
C	-1.109982	1.074508	-1.378895
C	7.025935	-0.032193	1.187118
O	6.348778	0.177607	-0.054002
H	2.750052	1.503983	-2.344963
H	5.049393	0.628828	-2.178559
H	4.805004	0.841342	2.100979
H	2.544641	1.709320	1.937675

H	0.976532	2.680994	-1.336895
H	0.557233	2.689377	1.684631
H	1.438081	3.916120	0.795968
H	-1.726262	3.066182	2.110855
H	-3.819255	4.301995	1.693497
H	-3.967437	5.895835	-0.199589
H	-1.996930	6.247440	-1.664277
H	0.093896	5.021229	-1.243519
H	0.937198	-0.354148	0.800432
H	-1.700588	-0.755593	2.107281
H	-3.238783	-1.294124	3.916773
H	-3.728867	-2.193527	2.477350
H	-2.885424	-3.013492	3.791474
H	-0.782743	-0.834013	4.387346
H	0.463479	-1.306307	3.224756
H	-0.351967	-2.534732	4.190265
H	-1.436628	-4.408631	2.813237
H	-0.675670	-6.080084	1.180249
H	0.147609	-5.369017	-1.023371
H	0.340289	-1.812604	-2.153338
H	2.337390	-2.912753	-3.004056
H	2.423615	-2.804532	-1.240901
H	2.031618	-4.341535	-2.015666
H	0.089069	-3.176997	-4.174760
H	-1.409838	-3.251781	-3.240312
H	-0.285526	-4.608604	-3.217184
H	-1.502601	-0.920716	-2.161743
H	-3.364205	-0.107609	1.109482
H	-3.422305	1.403324	0.198092
H	-4.818154	0.340515	0.232192
H	-3.447189	-2.402106	-1.905965
H	-4.850896	-1.847635	-0.996712
H	-3.408482	-2.384718	-0.141919
H	-3.525881	-0.209646	-3.228543
H	-3.592749	1.342206	-2.379445
H	-4.942186	0.221002	-2.270263
H	-0.794757	1.303671	-2.395706
H	-1.798350	1.847478	-1.046420
H	8.008045	-0.416319	0.925127
H	7.136902	0.903945	1.739629
H	6.501773	-0.765790	1.804591

B3LYP energy = -1507.78652517 a.u.

(5*S*,1'*S*)-**10a**, Conf. J

C	3.305337	1.410911	-0.998352
C	4.593603	0.922443	-0.803253
C	5.043336	0.663497	0.493615
C	4.191255	0.909091	1.576410
C	2.913979	1.398930	1.363650
C	2.443239	1.653835	0.069516
C	1.045202	2.175875	-0.206548
C	0.547437	3.202332	0.835353
C	-0.700434	3.966827	0.451808
C	-0.688346	4.850765	-0.632702
C	-1.826016	5.571541	-0.977609
C	-2.997534	5.426030	-0.237779
C	-3.019103	4.559517	0.849647
C	-1.877913	3.836248	1.190069
N	0.080998	1.049418	-0.390704
C	0.119254	-0.116511	0.198480
N	-0.830573	-0.962571	-0.162090
C	-0.764572	-2.376631	0.171832
C	-0.211099	-3.266779	-0.769675
C	0.456850	-2.822784	-2.067672
C	1.953945	-3.182842	-2.061985
C	-0.238913	-3.399255	-3.311920
C	-0.224218	-4.627303	-0.459065
C	-0.720006	-5.085430	0.751524
C	-1.190229	-4.182450	1.691959
C	-1.221857	-2.811518	1.431369
C	-1.683295	-1.866892	2.533992
C	-3.078947	-2.217876	3.074763
C	-0.656940	-1.828832	3.682134
C	-1.671726	-0.337588	-1.254833
C	-3.217732	-0.347654	-1.089932
C	-3.749069	-1.791743	-1.076810
C	-3.787165	0.351984	-2.343867
C	-3.696914	0.395356	0.164571
C	-1.043104	1.084213	-1.348624
C	7.198115	-0.076000	-0.257475
O	6.274803	0.185482	0.801920
H	2.975711	1.613575	-2.011176
H	5.231944	0.757812	-1.659015
H	4.552099	0.716702	2.578431
H	2.286746	1.590294	2.224864
H	1.062665	2.662734	-1.182627
H	0.389491	2.702534	1.792593
H	1.368463	3.908394	0.981676
H	0.222100	4.989011	-1.205680
H	-1.795992	6.254860	-1.817486
H	-3.882210	5.991751	-0.502129
H	-3.921770	4.448234	1.437775
H	-1.902182	3.171314	2.046141
H	0.885642	-0.381988	0.911838
H	0.405751	-1.736572	-2.136316
H	2.434810	-2.799510	-2.964458
H	2.462606	-2.754071	-1.196481
H	2.103710	-4.263495	-2.037776
H	0.233478	-3.016715	-4.219032
H	-1.297831	-3.136007	-3.343854
H	-0.168359	-4.488290	-3.335959
H	0.181410	-5.337525	-1.168031
H	-0.718487	-6.145789	0.971462
H	-1.537132	-4.547977	2.649626
H	-1.739454	-0.861549	2.118538
H	-3.394442	-1.469245	3.804470
H	-3.825558	-2.250363	2.280032
H	-3.083042	-3.186855	3.576801
H	-0.958092	-1.095629	4.433429
H	0.341068	-1.563619	3.327232
H	-0.582881	-2.800761	4.174210

H	-1.456787	-0.893800	-2.166715
H	-3.431069	-2.342247	-1.965057
H	-3.420522	-2.348865	-0.201431
H	-4.840441	-1.776648	-1.070977
H	-3.506414	1.405307	-2.400510
H	-4.877047	0.308011	-2.320291
H	-3.454787	-0.140733	-3.260917
H	-3.379828	1.438991	0.179145
H	-3.346258	-0.079031	1.079736
H	-4.788008	0.391863	0.193244
H	-0.661488	1.305304	-2.344636
H	-1.738665	1.868399	-1.061428
H	7.434580	0.836738	-0.809652
H	8.097693	-0.451704	0.222389
H	6.808862	-0.832475	-0.943347

B3LYP energy = -1507.78652474 a.u.

(5*S*,1’*S*)-**10a**, Conf. K

C	3.405723	1.195652	-1.305341
C	4.657151	0.615135	-1.222313
C	5.214900	0.323282	0.029099
C	4.496722	0.628832	1.185698
C	3.235670	1.214580	1.082962
C	2.666837	1.503094	-0.154680
C	1.284275	2.112228	-0.310743
C	0.869071	3.067283	0.826647
C	-0.430620	3.800839	0.584755
C	-1.584898	3.471430	1.297583
C	-2.778607	4.155811	1.080640
C	-2.833987	5.181785	0.143488
C	-1.688041	5.522921	-0.571458
C	-0.497449	4.839683	-0.349687
N	0.261538	1.046963	-0.535785
C	0.058989	-0.006118	0.210261
N	-0.895421	-0.819927	-0.208978
C	-1.050247	-2.154632	0.349404
C	-0.370204	-3.228493	-0.258252
C	0.622434	-3.064024	-1.404418
C	2.039896	-3.474530	-0.965082
C	0.195763	-3.834413	-2.664702
C	-0.580276	-4.505395	0.264709
C	-1.390935	-4.706769	1.370794
C	-1.991982	-3.624472	1.994069
C	-1.837137	-2.326430	1.504708
C	-2.467160	-1.170415	2.271136
C	-3.967016	-1.378927	2.535152
C	-1.717185	-0.920869	3.592903
C	-1.398050	-0.335519	-1.556540
C	-2.928665	-0.296850	-1.821855
C	-3.697040	0.617373	-0.858343
C	-3.521158	-1.716109	-1.773681
C	-3.093082	0.236204	-3.262518
C	-0.687804	1.043556	-1.665025
C	7.082712	-0.548299	1.256117
O	6.447489	-0.244193	0.012469
H	2.995262	1.421000	-2.283617
H	5.226691	0.387178	-2.113996
H	4.905037	0.426938	2.165231
H	2.711465	1.450924	2.000264
H	1.275365	2.676261	-1.243570
H	0.805779	2.513839	1.765062
H	1.683035	3.786260	0.942516
H	-1.549300	2.681149	2.039387
H	-3.662049	3.890691	1.648429
H	-3.759562	5.718590	-0.023065
H	-1.718883	6.329987	-1.293165
H	0.392887	5.128597	-0.897853
H	0.642093	-0.217817	1.095168
H	0.679836	-2.009102	-1.669636
H	2.752093	-3.278687	-1.769255
H	2.359813	-2.915492	-0.083640
H	2.091506	-4.538049	-0.725317
H	0.905504	-3.652675	-3.474321
H	-0.795498	-3.532806	-3.008195
H	0.170054	-4.910245	-2.482125
H	-0.083520	-5.353235	-0.189092
H	-1.536810	-5.706494	1.760740
H	-2.590905	-3.789394	2.880013
H	-2.363683	-0.269267	1.667898
H	-4.392125	-0.484980	2.996039
H	-4.516602	-1.578682	1.614055
H	-4.143473	-2.212907	3.216612
H	-2.141123	-0.058078	4.111226
H	-0.655413	-0.728500	3.425270
H	-1.795467	-1.783148	4.258196
H	-0.978357	-1.017186	-2.297248
H	-3.646542	0.262059	0.169045
H	-3.338312	1.647125	-0.877415
H	-4.749863	0.638380	-1.145492
H	-3.003196	-2.388285	-2.461221
H	-4.569915	-1.681342	-2.074004
H	-3.476877	-2.153151	-0.777545
H	-2.551458	-0.382158	-3.982749
H	-2.747331	1.266541	-3.367563
H	-4.148587	0.218267	-3.537408
H	-0.145214	1.156699	-2.601477
H	-1.375217	1.881022	-1.554383
H	8.043010	-0.985829	0.996977
H	7.242901	0.355498	1.848963
H	6.498627	-1.269735	1.832924

B3LYP energy = -1507.78615385 a.u.

(5*S*,1’*R*)-**10b**, Conf. A

C	-3.464200	-0.253797	-1.472275
C	-4.668217	-0.932434	-1.457443
C	-5.591460	-0.701101	-0.429964
C	-5.283622	0.220322	0.572199
C	-4.064847	0.895889	0.540005
C	-3.136949	0.672728	-0.473581

C	-1.793768	1.380146	-0.539999
C	-1.778025	2.802209	0.057933
C	-0.524887	3.590221	-0.254281
C	-0.243212	3.986784	-1.566495
C	0.896645	4.727253	-1.859446
C	1.776595	5.088285	-0.841866
C	1.505074	4.706321	0.467310
C	0.362467	3.963690	0.756407
N	-0.735948	0.522565	0.055279
C	0.356120	0.138345	-0.551985
N	1.152266	-0.643146	0.157273
C	2.227916	-1.405152	-0.458568
C	3.487002	-0.800750	-0.639520
C	3.767432	0.667615	-0.347264
C	4.984988	0.872072	0.568581
C	3.943087	1.455608	-1.659343
C	4.508094	-1.586460	-1.177112
C	4.291264	-2.908530	-1.532001
C	3.026824	-3.461287	-1.400831
C	1.961975	-2.721323	-0.885306
C	0.570895	-3.347295	-0.885592
C	0.096537	-3.609843	-2.327059
C	0.499322	-4.630690	-0.042504
C	0.494915	-0.942761	1.488457
C	1.336687	-0.766362	2.784378
C	2.516035	-1.753998	2.802403
C	0.398784	-1.128494	3.957165
C	1.857557	0.664161	2.980436
C	-0.771056	-0.039545	1.418502
C	-7.743245	-1.203930	0.503659
O	-6.746740	-1.409782	-0.499738
H	-2.767508	-0.440027	-2.282197
H	-4.920239	-1.639744	-2.236941
H	-5.978462	0.425907	1.373562
H	-3.859286	1.610860	1.326532
H	-1.516122	1.458744	-1.591582
H	-2.647625	3.318086	-0.355550
H	-1.928295	2.761461	1.137778
H	-0.930828	3.733229	-2.366158
H	1.092092	5.032983	-2.879979
H	2.661067	5.670659	-1.067955
H	2.176907	4.992528	1.267264
H	0.152134	3.688629	1.783491
H	0.583982	0.423175	-1.569547
H	2.900499	1.085661	0.162474
H	5.089440	1.930038	0.817691
H	4.890638	0.313496	1.501051
H	5.910411	0.557386	0.083069
H	4.075673	2.517826	-1.445269
H	3.076149	1.348863	-2.314647
H	4.819962	1.109888	-2.210819
H	5.488461	-1.152778	-1.324513
H	5.102986	-3.501813	-1.934355
H	2.857278	-4.480138	-1.724018
H	-0.135487	-2.636540	-0.457843
H	-0.927454	-3.989235	-2.321907
H	0.119271	-2.695410	-2.923423
H	0.724654	-4.350146	-2.825654
H	-0.521903	-5.016910	-0.032217
H	0.804726	-4.453623	0.990372
H	1.144343	-5.411573	-0.449792
H	0.196044	-1.990423	1.454123
H	2.177025	-2.784013	2.673653
H	3.248724	-1.541503	2.026190
H	3.025489	-1.691882	3.765702
H	-0.440738	-0.436847	4.051946
H	0.958043	-1.088803	4.893069
H	-0.001701	-2.139737	3.852301
H	1.055136	1.402787	3.019105
H	2.549365	0.959156	2.193757
H	2.393975	0.725920	3.928925
H	-1.692828	-0.598463	1.563767
H	-0.736291	0.768255	2.146431
H	-8.090783	-0.168005	0.508364
H	-8.567397	-1.860967	0.239354
H	-7.368186	-1.472014	1.494413

B3LYP energy = -1507.78853843 a.u.

(5*S*,1’*R*)-**10b**, Conf. B

C	-3.959775	1.016824	0.883628
C	-5.200401	0.414353	0.999722
C	-5.679464	-0.419659	-0.016975
C	-4.893184	-0.638904	-1.150295
C	-3.646820	-0.026915	-1.248136
C	-3.157203	0.805335	-0.243729
C	-1.786637	1.439172	-0.405910
C	-1.667572	2.862913	0.181432
C	-0.419662	3.606883	-0.236905
C	-0.256442	4.027116	-1.561741
C	0.874270	4.736504	-1.951149
C	1.863285	5.042304	-1.019154
C	1.711382	4.634465	0.301491
C	0.578201	3.921798	0.687197
N	-0.736200	0.535886	0.132790
C	0.331028	0.142680	-0.511884
N	1.134179	-0.663446	0.162288
C	2.174081	-1.440661	-0.495686
C	1.867343	-2.746117	-0.928749
C	0.464693	-3.343949	-0.890827
C	-0.057167	-3.593925	-2.318027
C	0.393340	-4.628809	-0.049648
C	2.899375	-3.502449	-1.485566
C	4.171072	-2.975764	-1.648837
C	4.427451	-1.663345	-1.285083
C	3.440515	-0.862225	-0.707825
C	3.762928	0.595649	-0.408618
C	5.008000	0.762165	0.477264
C	3.921431	1.392640	-1.717176
C	0.513484	-0.966768	1.510183
C	1.395052	-0.815637	2.781989
C	0.485699	-1.172759	3.978887

C	1.944869	0.604457	2.974296
C	2.556304	-1.824185	2.756411
C	-0.738850	-0.042989	1.489183
C	-7.467616	-1.802432	-0.822115
O	-6.908265	-0.958787	0.186637
H	-3.625836	1.664055	1.684408
H	-5.820310	0.583155	1.870620
H	-5.238735	-1.268754	-1.957096
H	-3.051652	-0.201708	-2.137799
H	-1.574463	1.498099	-1.474017
H	-2.549581	3.409919	-0.158703
H	-1.728110	2.828655	1.270107
H	-1.029270	3.816016	-2.293294
H	0.977983	5.061730	-2.979085
H	2.740232	5.601867	-1.319858
H	2.469543	4.877441	1.035890
H	0.462804	3.625937	1.723594
H	0.532823	0.437731	-1.532188
H	-0.213614	-2.620066	-0.440516
H	-1.090074	-3.946960	-2.283535
H	-0.027377	-2.681183	-2.916608
H	0.537134	-4.350738	-2.832967
H	-0.635251	-4.992290	-0.004230
H	0.739955	-4.463581	0.972101
H	1.005776	-5.421966	-0.482676
H	2.697961	-4.513276	-1.815753
H	4.956892	-3.581415	-2.082967
H	5.412195	-1.248983	-1.456800
H	2.919420	1.027172	0.127841
H	5.145667	1.815185	0.731185
H	4.922595	0.199015	1.407839
H	5.912651	0.427663	-0.033472
H	4.088156	2.448600	-1.496833
H	3.032745	1.315248	-2.346888
H	4.772241	1.030245	-2.298046
H	0.197234	-2.009160	1.475636
H	-0.340676	-0.470154	4.103012
H	0.068041	-2.177378	3.878072
H	1.072095	-1.148372	4.898589
H	2.619294	0.894665	2.170846
H	2.508163	0.650268	3.907970
H	1.155253	1.354842	3.041242
H	3.269631	-1.617008	1.960953
H	2.193255	-2.846027	2.627101
H	3.094707	-1.782229	3.704888
H	-1.663833	-0.589468	1.656544
H	-0.669241	0.756076	2.224564
H	-8.443431	-2.100675	-0.448354
H	-7.588469	-1.263995	-1.765194
H	-6.852297	-2.691583	-0.980643

B3LYP energy = -1507.78846606 a.u.

(5*S*,1’*R*)-**10b**, Conf. C

C	-2.331082	1.183559	-0.798224
C	-3.144455	2.276019	-1.033044
C	-4.200386	2.569054	-0.159417
C	-4.419312	1.752293	0.950255
C	-3.584756	0.658359	1.173731
C	-2.538431	0.353968	0.310228
C	-1.663857	-0.861847	0.559216
C	-1.809033	-1.971271	-0.504706
C	-3.176964	-2.616765	-0.485017
C	-3.484503	-3.585121	0.474452
C	-4.740426	-4.182173	0.505475
C	-5.707921	-3.820109	-0.428051
C	-5.410283	-2.860980	-1.390979
C	-4.152894	-2.264272	-1.418371
N	-0.244946	-0.460707	0.715870
C	0.735855	-0.591984	-0.139487
N	1.873223	-0.019653	0.217426
C	3.118998	-0.298288	-0.479693
C	3.963960	-1.307318	0.022586
C	3.590071	-2.225531	1.181675
C	3.438202	-3.678910	0.695929
C	4.587384	-2.140513	2.348465
C	5.181697	-1.515113	-0.626505
C	5.531209	-0.785606	-1.752009
C	4.651195	0.152000	-2.268465
C	3.426066	0.417323	-1.653836
C	2.479586	1.412376	-2.315396
C	3.134392	2.783974	-2.550097
C	1.933407	0.848957	-3.641147
C	1.722166	0.567599	1.607021
C	2.208686	2.022705	1.858939
C	3.728309	2.129961	1.645504
C	1.919483	2.314460	3.347788
C	1.486218	3.062821	0.991619
C	0.205959	0.345290	1.865779
C	-6.047162	4.009393	0.360910
O	-4.943656	3.660129	-0.476882
H	-1.524335	0.979012	-1.493071
H	-2.984478	2.917524	-1.889925
H	-5.225516	1.952194	1.641049
H	-3.767978	0.031586	2.038984
H	-1.940193	-1.287214	1.525846
H	-1.621084	-1.558717	-1.497666
H	-1.050839	-2.735393	-0.313303
H	-2.732942	-3.883815	1.197211
H	-4.960796	-4.935198	1.252160
H	-6.684800	-4.287078	-0.408289
H	-6.155357	-2.578438	-2.124526
H	-3.929128	-1.519792	-2.173344
H	0.643440	-1.139267	-1.064915
H	2.614536	-1.928785	1.565199
H	3.101598	-4.315283	1.517068
H	2.709818	-3.753015	-0.114173
H	4.385960	-4.078318	0.330817
H	4.253780	-2.773157	3.173674
H	4.688023	-1.119996	2.722190
H	5.579630	-2.483448	2.050018
H	5.857869	-2.275622	-0.257773

H	6.481103	-0.964133	-2.240451
H	4.918925	0.687231	-3.170082
H	1.626091	1.565893	-1.655490
H	2.396330	3.487420	-2.940939
H	3.545454	3.201545	-1.630024
H	3.944729	2.720280	-3.278429
H	1.204870	1.539114	-4.071974
H	1.445694	-0.118482	-3.504240
H	2.734555	0.712036	-4.370026
H	2.300223	-0.071594	2.273901
H	4.268324	1.405580	2.259129
H	4.016777	1.973370	0.607654
H	4.066035	3.126099	1.936887
H	0.851750	2.306699	3.575541
H	2.298919	3.304781	3.603464
H	2.414744	1.590616	3.999672
H	0.406494	3.065887	1.149998
H	1.671388	2.908574	-0.069657
H	1.849124	4.060226	1.245797
H	0.014095	-0.197014	2.790122
H	-0.356228	1.277304	1.884462
H	-5.715422	4.241996	1.375886
H	-6.488170	4.895875	-0.087003
H	-6.789980	3.208552	0.389800

B3LYP energy = -1507.78787372 a.u.

(5*S*,1'*R*)-**10b**, Conf. D

C	-2.464603	-1.250818	0.579389
C	-3.313891	-2.348566	0.676516
C	-4.322361	-2.528296	-0.274662
C	-4.459107	-1.602026	-1.313494
C	-3.601175	-0.518337	-1.396274
C	-2.591264	-0.322259	-0.450715
C	-1.678231	0.887257	-0.546332
C	-1.845158	1.899323	0.607639
C	-3.195449	2.580877	0.581593
C	-3.423111	3.654267	-0.283788
C	-4.662262	4.284672	-0.324743
C	-5.693207	3.850554	0.504291
C	-5.475944	2.785108	1.372209
C	-4.235071	2.155301	1.409571
N	-0.263521	0.466565	-0.687841
C	0.709931	0.563003	0.179795
N	1.858142	0.029830	-0.203651
C	3.097531	0.295317	0.509339
C	3.407463	-0.462844	1.655471
C	2.463563	-1.489479	2.269403
C	3.132241	-2.855844	2.491231
C	1.871894	-0.960906	3.589900
C	4.624284	-0.206395	2.289596
C	5.494251	0.763527	1.817491
C	5.142094	1.532518	0.719470
C	3.931792	1.334791	0.053324
C	3.556156	2.293386	-1.072372
C	3.368994	3.721923	-0.529189
C	4.571302	2.272919	-2.226591
C	1.720114	-0.494119	-1.619093
C	2.211376	-1.937032	-1.927392
C	1.923212	-2.173660	-3.426330
C	1.493824	-3.014146	-1.101992
C	3.731604	-2.046055	-1.717350
C	0.205533	-0.262090	-1.881548
C	-5.120264	-4.538240	0.763826
O	-5.204743	-3.560594	-0.274390
H	-1.693058	-1.132278	1.331800
H	-3.183807	-3.047260	1.490131
H	-5.242968	-1.748626	-2.045156
H	-3.724363	0.190051	-2.207168
H	-1.904115	1.408876	-1.478981
H	-1.710085	1.395246	1.566617
H	-1.063878	2.658844	0.520012
H	-2.622343	4.007154	-0.924766
H	-4.820426	5.119025	-0.997067
H	-6.657375	4.342823	0.476814
H	-6.271514	2.444237	2.023286
H	-4.074802	1.326343	2.088738
H	0.602260	1.050999	1.136567
H	1.631850	-1.641465	1.581958
H	2.394521	-3.577550	2.848123
H	3.570965	-3.247015	1.572179
H	3.923336	-2.798494	3.240922
H	1.145746	-1.671653	3.990313
H	1.369465	-0.000660	3.454212
H	2.651186	-0.819616	4.341310
H	4.893363	-0.773299	3.171186
H	6.438161	0.935644	2.319591
H	5.810472	2.315915	0.386219
H	2.591792	1.995643	-1.482212
H	3.036708	4.387109	-1.329026
H	2.622952	3.747982	0.267604
H	4.301843	4.121909	-0.127998
H	4.236019	2.928025	-3.033296
H	4.699142	1.268913	-2.635467
H	5.551784	2.625071	-1.901324
H	2.303873	0.172324	-2.253434
H	0.855032	-2.172848	-3.652102
H	2.405985	-1.418141	-4.050919
H	2.316020	-3.147748	-3.721221
H	1.693403	-2.912827	-0.036919
H	1.848420	-3.999836	-1.408453
H	0.412180	-3.004412	-1.247773
H	4.019312	-1.928877	-0.673932
H	4.268522	-1.295793	-2.301935
H	4.074356	-3.028450	-2.046956
H	0.021299	0.337371	-2.771916
H	-0.352331	-1.192693	-1.967063
H	-5.272202	-4.085938	1.746917
H	-5.918636	-5.248306	0.565694
H	-4.158831	-5.057535	0.741321

B3LYP energy = -1507.78754569 a.u.

(5*S*,1’*R*)-**10b**, Conf. E

C	4.100373	1.371502	1.118398
C	5.353419	0.797891	0.983299
C	5.588114	-0.149025	-0.019348
C	4.547143	-0.510402	-0.879027
C	3.295611	0.079579	-0.730628
C	3.051341	1.025396	0.261875
C	1.698995	1.705447	0.401933
C	1.400736	2.655024	-0.774566
C	0.152047	3.505114	-0.651279
C	-0.052043	4.336335	0.454618
C	-1.183466	5.140137	0.541770
C	-2.130800	5.131329	-0.479209
C	-1.935120	4.316871	-1.588752
C	-0.801789	3.511592	-1.671300
N	0.632931	0.705160	0.629721
C	-0.327102	0.320250	-0.169630
N	-1.065309	-0.678513	0.284485
C	-2.321394	-1.060235	-0.338711
C	-3.527189	-0.621309	0.243573
C	-3.600417	0.345336	1.421688
C	-4.307755	1.652085	1.018859
C	-4.278116	-0.282534	2.651264
C	-4.720580	-1.057522	-0.333728
C	-4.724548	-1.854338	-1.467631
C	-3.525954	-2.213888	-2.063133
C	-2.299190	-1.828858	-1.519814
C	-1.021868	-2.215043	-2.256083
C	-0.918202	-3.727980	-2.508974
C	-0.898216	-1.439308	-3.581103
C	-0.572111	-1.093524	1.654347
C	-0.181019	-2.585060	1.865662
C	0.270059	-2.705801	3.337896
C	0.962790	-3.044588	0.949749
C	-1.404548	-3.497490	1.673097
C	0.594008	-0.088897	1.872517
C	7.153365	-1.624679	-1.082489
O	6.845138	-0.659569	-0.074975
H	3.936939	2.102859	1.901936
H	6.164106	1.068166	1.647431
H	4.697974	-1.241204	-1.660137
H	2.504441	-0.219819	-1.408642
H	1.719883	2.296974	1.319606
H	2.276629	3.304978	-0.841814
H	1.384471	2.096235	-1.713451
H	0.684331	4.372795	1.249419
H	-1.321337	5.781302	1.403736
H	-3.009706	5.760107	-0.412104
H	-2.659961	4.309598	-2.393370
H	-0.651724	2.893738	-2.550305
H	-0.527804	0.789872	-1.119354
H	-2.587321	0.625717	1.708023
H	-4.290966	2.356634	1.853014
H	-3.816935	2.125190	0.166739
H	-5.351548	1.476335	0.752954
H	-4.263946	0.418963	3.487862
H	-3.778743	-1.199918	2.968374
H	-5.320882	-0.529931	2.443842
H	-5.663189	-0.749216	0.099892
H	-5.663244	-2.178024	-1.899854
H	-3.541684	-2.806979	-2.967992
H	-0.171487	-1.932929	-1.636190
H	0.047435	-3.965285	-2.960284
H	-1.008072	-4.299794	-1.584373
H	-1.693953	-4.074749	-3.193688
H	0.046158	-1.679789	-4.074197
H	-0.934363	-0.358872	-3.425123
H	-1.708269	-1.699273	-4.265420
H	-1.375930	-0.880374	2.355948
H	1.173846	-2.131125	3.549402
H	-0.512836	-2.374770	4.024444
H	0.490602	-3.749914	3.564183
H	0.682504	-3.013732	-0.102027
H	1.223960	-4.077377	1.186979
H	1.867961	-2.447519	1.076503
H	-1.770420	-3.495067	0.647992
H	-2.228491	-3.201005	2.325864
H	-1.135657	-4.524552	1.926848
H	0.421636	0.576256	2.718557
H	1.552678	-0.583669	2.008597
H	7.014654	-1.209413	-2.083646
H	8.200388	-1.877064	-0.938004
H	6.543968	-2.524775	-0.968647

B3LYP energy = -1507.78743703 a.u.

(5*S*,1’*R*)-**10b**, Conf. F

C	3.298417	-1.149518	-1.289353
C	4.547005	-0.564286	-1.200133
C	5.130895	-0.341304	0.053691
C	4.440986	-0.719992	1.205939
C	3.182299	-1.309393	1.096900
C	2.587632	-1.530978	-0.142862
C	1.210378	-2.148894	-0.305207
C	0.831041	-3.163839	0.795775
C	-0.466563	-3.901210	0.552788
C	-0.559285	-4.867666	-0.454123
C	-1.752812	-5.542279	-0.686590
C	-2.876207	-5.264388	0.088954
C	-2.794017	-4.312938	1.100180
C	-1.596987	-3.638018	1.329150
N	0.162787	-1.094219	-0.452338
C	0.142816	0.073230	0.134522
N	-0.853976	0.865627	-0.221092
C	-1.183368	2.064828	0.532752
C	-0.475333	3.255990	0.279292
C	0.703483	3.350488	-0.681998
C	0.530915	4.469972	-1.721312
C	2.025575	3.530123	0.087699
C	-0.851983	4.394611	0.994064
C	-1.881002	4.357691	1.921816

C	-2.528476	3.161559	2.189119
C	-2.182684	1.985335	1.522619
C	-2.849772	0.681846	1.950309
C	-2.501553	0.346743	3.412155
C	-4.373098	0.703284	1.743740
C	-1.752762	0.133084	-1.195714
C	-2.153601	0.851930	-2.515346
C	-0.950820	1.215707	-3.397289
C	-2.978558	2.115880	-2.217134
C	-3.062720	-0.135761	-3.279009
C	-0.963480	-1.190907	-1.402899
C	7.019966	0.472431	1.287811
O	6.357920	0.237047	0.043009
H	2.868340	-1.322711	-2.269408
H	5.094390	-0.279903	-2.089444
H	4.868982	-0.571186	2.186610
H	2.679178	-1.600646	2.010025
H	1.192638	-2.670383	-1.263530
H	1.655377	-3.878321	0.855731
H	0.781831	-2.654652	1.759525
H	0.311764	-5.103234	-1.055893
H	-1.803899	-6.292303	-1.466324
H	-3.804137	-5.793921	-0.087415
H	-3.658222	-4.101102	1.717874
H	-1.539454	-2.908780	2.129383
H	0.882777	0.371705	0.862231
H	0.775610	2.409413	-1.225676
H	1.351485	4.438953	-2.440948
H	-0.405602	4.370840	-2.272066
H	0.541219	5.456555	-1.254943
H	2.866795	3.544575	-0.608376
H	2.193555	2.722345	0.802952
H	2.032873	4.470553	0.642442
H	-0.328459	5.325898	0.821714
H	-2.164349	5.257876	2.453008
H	-3.302319	3.135139	2.945539
H	-2.447725	-0.132187	1.348114
H	-2.924419	-0.622224	3.685480
H	-1.420844	0.302206	3.561432
H	-2.904172	1.092297	4.099965
H	-4.803262	-0.261608	2.019737
H	-4.639649	0.909139	0.705447
H	-4.846585	1.466075	2.364629
H	-2.679605	-0.078165	-0.663477
H	-0.284641	1.923550	-2.907415
H	-0.363616	0.343187	-3.689851
H	-1.305530	1.683195	-4.317381
H	-3.848825	1.887812	-1.597822
H	-3.344048	2.539948	-3.154085
H	-2.396487	2.884042	-1.711224
H	-3.931802	-0.421461	-2.681512
H	-2.537112	-1.046076	-3.573642
H	-3.427997	0.338008	-4.191327
H	-1.562058	-2.071192	-1.185685
H	-0.560189	-1.282067	-2.410957
H	7.199838	-0.463504	1.822199
H	7.970923	0.932291	1.033071
H	6.444430	1.153631	1.919403

B3LYP energy = -1507.78719991 a.u.

(5*S*,1’*R*)-**10b**, Conf. G

C	2.836380	1.236834	-1.138192
C	3.624527	2.383958	-1.111659
C	4.167300	2.816931	0.099516
C	3.912857	2.085875	1.266004
C	3.132555	0.944297	1.218780
C	2.573915	0.499507	0.013991
C	1.691183	-0.730979	-0.074939
C	2.206895	-1.978738	0.676894
C	3.464606	-2.548785	0.058433
C	3.391980	-3.288656	-1.125676
C	4.538835	-3.816559	-1.708800
C	5.780918	-3.614809	-1.112582
C	5.865115	-2.884947	0.068473
C	4.714804	-2.356579	0.648230
N	0.299631	-0.414102	0.347550
C	-0.741897	-0.409379	-0.441656
N	-1.888921	-0.098478	0.136269
C	-3.076138	0.236129	-0.634115
C	-3.366438	1.595037	-0.869487
C	-2.438083	2.741570	-0.480845
C	-1.953232	3.504561	-1.727179
C	-3.086492	3.703994	0.528319
C	-4.547497	1.895392	-1.549811
C	-5.383293	0.894585	-2.019543
C	-5.037603	-0.435427	-1.840015
C	-3.880327	-0.799738	-1.149669
C	-3.526213	-2.278555	-1.046222
C	-4.664540	-3.121438	-0.448002
C	-3.110004	-2.839966	-2.419269
C	-1.643947	0.215719	1.596971
C	-2.486346	-0.550808	2.656878
C	-2.034116	-0.020283	4.035004
C	-2.278280	-2.071524	2.607806
C	-3.980828	-0.223840	2.495636
C	-0.107636	-0.017486	1.709255
C	5.268787	4.694163	-0.909234
O	4.949219	3.916760	0.246464
H	2.427983	0.911115	-2.088515
H	3.810584	2.919984	-2.031025
H	4.345554	2.425013	2.198317
H	2.971335	0.395379	2.138283
H	1.599485	-0.997994	-1.128061
H	2.386023	-1.741519	1.726327
H	1.415476	-2.732081	0.650297
H	2.428358	-3.466042	-1.591328
H	4.462218	-4.392342	-2.623064
H	6.674761	-4.028278	-1.562931
H	6.826225	-2.727824	0.542519
H	4.793132	-1.792895	1.570128
H	-0.672187	-0.626800	-1.497974

H	-1.545992	2.327245	-0.012325
H	-1.232864	4.272474	-1.437645
H	-1.468764	2.835730	-2.441051
H	-2.779626	3.999605	-2.240093
H	-2.376536	4.482062	0.815918
H	-3.408027	3.186474	1.433998
H	-3.962134	4.195270	0.100165
H	-4.806068	2.930347	-1.733219
H	-6.293581	1.151619	-2.546855
H	-5.677720	-1.207771	-2.245753
H	-2.666926	-2.378777	-0.384352
H	-4.335705	-4.155004	-0.321961
H	-4.979258	-2.745999	0.526809
H	-5.539515	-3.133301	-1.100124
H	-2.791672	-3.879854	-2.319529
H	-2.287470	-2.273689	-2.861511
H	-3.943266	-2.811871	-3.124131
H	-1.864638	1.273659	1.728941
H	-0.990381	-0.253921	4.253285
H	-2.164757	1.061931	4.109644
H	-2.638913	-0.481877	4.816900
H	-2.609412	-2.499638	1.662762
H	-2.861036	-2.542991	3.400970
H	-1.236711	-2.359822	2.763946
H	-4.390274	-0.593873	1.557743
H	-4.159073	0.852728	2.539732
H	-4.543623	-0.685327	3.308951
H	0.427927	0.881811	2.006936
H	0.136168	-0.814372	2.407618
H	5.902742	5.502321	-0.554568
H	5.814558	4.100281	-1.646358
H	4.368382	5.112452	-1.366029

B3LYP energy = -1507.78718667 a.u.

(5*S*,1’*R*)-**10b**, Conf. H

C	-2.364390	1.297973	-0.874790
C	-2.754874	2.633137	-0.828543
C	-3.076121	3.218355	0.399231
C	-2.996261	2.451278	1.566961
C	-2.599747	1.127519	1.502398
C	-2.275489	0.524277	0.281831
C	-1.846556	-0.926562	0.257713
C	-2.570164	-1.793168	-0.798934
C	-4.048837	-1.942711	-0.515636
C	-5.003444	-1.267754	-1.277797
C	-6.361955	-1.412136	-1.009452
C	-6.785883	-2.234218	0.029441
C	-5.843217	-2.915544	0.794910
C	-4.487251	-2.771772	0.521163
N	-0.368211	-1.060322	0.093013
C	0.510970	-0.095623	-0.001316
N	1.775228	-0.478431	0.052534
C	2.859945	0.400590	-0.357432
C	3.383481	1.329364	0.562795
C	2.805204	1.560965	1.953190
C	3.860770	1.439608	3.064153
C	2.101976	2.929352	2.028647
C	4.457293	2.117294	0.143994
C	4.983744	2.001585	-1.132635
C	4.414196	1.120026	-2.038105
C	3.330123	0.316229	-1.683157
C	2.674907	-0.542623	-2.760560
C	2.021841	0.342811	-3.837954
C	3.654516	-1.541836	-3.397089
C	1.841720	-1.984587	0.181553
C	2.715659	-2.591318	1.316691
C	2.567974	-4.124105	1.197063
C	2.277190	-2.150619	2.720596
C	4.198381	-2.243886	1.099348
C	0.332776	-2.348220	0.272977
C	-3.585763	5.342281	-0.594477
O	-3.473090	4.505339	0.558713
H	-2.130077	0.868854	-1.841601
H	-2.813810	3.197879	-1.747602
H	-3.255555	2.909795	2.512347
H	-2.550781	0.548101	2.417078
H	-2.057105	-1.353617	1.241723
H	-2.415013	-1.365016	-1.790087
H	-2.115287	-2.785606	-0.809991
H	-4.685530	-0.626260	-2.091094
H	-7.088212	-0.882850	-1.614106
H	-7.842320	-2.347641	0.238661
H	-6.164145	-3.565109	1.600086
H	-3.763938	-3.320122	1.115140
H	0.225506	0.937667	-0.125007
H	2.047020	0.800577	2.136629
H	3.384290	1.531686	4.042312
H	4.378846	0.480244	3.030214
H	4.612302	2.227549	2.990104
H	1.626389	3.055391	3.003524
H	1.333520	3.033171	1.260202
H	2.814801	3.745634	1.895899
H	4.885501	2.837253	0.829182
H	5.824824	2.615651	-1.429753
H	4.805233	1.070441	-3.046102
H	1.869098	-1.119074	-2.307653
H	1.500120	-0.279021	-4.568631
H	1.299773	1.034770	-3.400026
H	2.768319	0.932075	-4.373624
H	3.131047	-2.172406	-4.118606
H	4.114946	-2.190283	-2.649739
H	4.457233	-1.028384	-3.929453
H	2.249144	-2.360413	-0.756143
H	1.548630	-4.463627	1.390753
H	2.863489	-4.478575	0.206641
H	3.215080	-4.608776	1.929532
H	2.382404	-1.076572	2.863231
H	2.903971	-2.642436	3.466360
H	1.243854	-2.423632	2.943525
H	4.395163	-1.177850	1.194334
H	4.541316	-2.563301	0.112867

H	4.805858	-2.761891	1.843643
H	0.036441	-3.053891	-0.498893
H	0.059611	-2.762003	1.243146
H	-4.325359	4.950294	-1.296728
H	-3.915397	6.309344	-0.224528
H	-2.621887	5.454765	-1.096966

B3LYP energy = -1507.78712480 a.u.

(5*S*,1’*R*)-**10b**, Conf. I

C	-2.339595	1.141839	-1.221435
C	-2.731507	2.455474	-1.406983
C	-3.105227	3.243111	-0.310950
C	-3.076046	2.690135	0.970677
C	-2.672201	1.368687	1.138790
C	-2.297432	0.572757	0.058536
C	-1.856766	-0.854201	0.301990
C	-2.469327	-1.902130	-0.657928
C	-3.952711	-2.098215	-0.437719
C	-4.405186	-2.884602	0.625838
C	-5.765729	-3.068292	0.849039
C	-6.697807	-2.469265	0.005740
C	-6.259251	-1.690238	-1.060160
C	-4.896698	-1.506570	-1.278778
N	-0.369189	-0.981159	0.301530
C	0.515926	-0.103967	-0.095310
N	1.775071	-0.431740	0.138278
C	2.882552	0.259862	-0.501413
C	3.440420	-0.302056	-1.666548
C	2.859722	-1.517814	-2.382334
C	2.342096	-1.131738	-3.780278
C	3.860785	-2.681187	-2.471383
C	4.549306	0.334086	-2.225654
C	5.057120	1.504061	-1.683345
C	4.443710	2.078240	-0.581069
C	3.343536	1.478555	0.034576
C	2.671812	2.197814	1.197969
C	3.653873	2.556215	2.325159
C	1.932474	3.456147	0.705079
C	1.830736	-1.787782	0.805963
C	2.617327	-1.908743	2.143250
C	4.106465	-1.592027	1.923263
C	2.509164	-3.387666	2.574098
C	2.052950	-1.014945	3.256850
C	0.318401	-2.134637	0.915774
C	-3.890166	5.368693	0.476298
O	-3.479289	4.515307	-0.594560
H	-2.069115	0.559368	-2.093720
H	-2.765647	2.890293	-2.397472
H	-3.367709	3.267902	1.835544
H	-2.662495	0.953047	2.139858
H	-2.151047	-1.123104	1.319344
H	-2.270752	-1.611274	-1.690054
H	-1.962081	-2.855523	-0.498453
H	-3.687127	-3.369057	1.279169
H	-6.097890	-3.685057	1.675188
H	-7.757555	-2.614027	0.175228
H	-6.977299	-1.225864	-1.724953
H	-4.567348	-0.899491	-2.113429
H	0.243167	0.818082	-0.585695
H	1.992776	-1.872685	-1.825760
H	1.868627	-1.993430	-4.255588
H	1.606156	-0.327049	-3.723363
H	3.153213	-0.796968	-4.429226
H	3.390763	-3.547420	-2.941554
H	4.220694	-2.982855	-1.485981
H	4.731187	-2.410776	-3.072108
H	5.009727	-0.081790	-3.112601
H	5.918168	1.981768	-2.134005
H	4.826040	3.012684	-0.191577
H	1.921591	1.532303	1.622697
H	3.109680	2.981369	3.171008
H	4.199622	1.681074	2.680831
H	4.385668	3.298050	2.000970
H	1.403415	3.931707	1.533696
H	1.202671	3.219639	-0.072133
H	2.631262	4.185270	0.290296
H	2.309489	-2.467057	0.102722
H	4.535648	-2.218550	1.138259
H	4.276289	-0.551869	1.652680
H	4.659218	-1.789796	2.843467
H	1.483400	-3.685761	2.799110
H	3.098253	-3.545453	3.478615
H	2.898390	-4.057186	1.803362
H	1.011542	-1.243668	3.491617
H	2.119840	0.042423	3.005919
H	2.626710	-1.170181	4.172112
H	0.068104	-3.050577	0.384570
H	-0.015732	-2.232110	1.947440
H	-3.082019	5.515412	1.196977
H	-4.140192	6.320037	0.014677
H	-4.770515	4.968392	0.984536

B3LYP energy = -1507.78710982 a.u.

(5*S*,1’*R*)-**10b**, Conf. J

C	3.241840	1.056068	0.874730
C	4.043617	2.190677	0.775473
C	4.213304	2.812223	-0.463156
C	3.576238	2.280374	-1.590333
C	2.780543	1.155643	-1.471312
C	2.593861	0.521671	-0.236614
C	1.679566	-0.687203	-0.154740
C	2.178968	-1.854498	0.727529
C	3.392099	-2.545120	0.143822
C	3.244729	-3.446798	-0.914505
C	4.350787	-4.078835	-1.472544
C	5.625777	-3.821968	-0.975552
C	5.784062	-2.933304	0.082889
C	4.674745	-2.300437	0.636849
N	0.311365	-0.282364	0.267035
C	-0.770421	-0.397740	-0.457568

N	-1.887548	-0.011199	0.132454
C	-3.131209	0.167712	-0.597598
C	-3.896147	-0.963034	-0.948025
C	-3.450195	-2.398807	-0.696226
C	-4.503436	-3.224036	0.060580
C	-3.070408	-3.093459	-2.017450
C	-5.105729	-0.744020	-1.609610
C	-5.540365	0.535967	-1.915819
C	-4.745537	1.628293	-1.605670
C	-3.515436	1.472332	-0.965467
C	-2.638095	2.703746	-0.763461
C	-2.265824	3.342567	-2.114164
C	-3.291221	3.740845	0.165204
C	-1.573706	0.480550	1.530044
C	-2.310735	-0.202697	2.718454
C	-1.812789	0.504120	3.997985
C	-2.018145	-1.706429	2.822286
C	-3.828742	0.026091	2.617781
C	-0.023579	0.332501	1.565210
C	5.653913	4.509532	0.432587
O	4.970980	3.918396	-0.674654
H	3.142576	0.591199	1.847669
H	4.531299	2.570554	1.661342
H	3.723964	2.759864	-2.549298
H	2.302662	0.756881	-2.359049
H	1.548475	-1.075026	-1.165289
H	2.400334	-1.501617	1.735516
H	1.359470	-2.572224	0.816852
H	2.255162	-3.667014	-1.300725
H	4.216724	-4.777080	-2.289708
H	6.487953	-4.315506	-1.406567
H	6.771380	-2.732618	0.480471
H	4.810171	-1.611317	1.461729
H	-0.755100	-0.768223	-1.473055
H	-2.553841	-2.375498	-0.077828
H	-4.107945	-4.217027	0.283836
H	-4.785330	-2.753295	1.003424
H	-5.410535	-3.357460	-0.531120
H	-2.703467	-4.103078	-1.820704
H	-2.291510	-2.548588	-2.555425
H	-3.933511	-3.174176	-2.680870
H	-5.717314	-1.591615	-1.890433
H	-6.489408	0.680709	-2.416861
H	-5.074789	2.620433	-1.886760
H	-1.699283	2.393624	-0.305492
H	-1.578565	4.176073	-1.955258
H	-1.780401	2.623093	-2.776097
H	-3.146108	3.730807	-2.629312
H	-2.614911	4.583201	0.324195
H	-3.539333	3.315916	1.139371
H	-4.213456	4.132701	-0.267907
H	-1.840774	1.535033	1.562990
H	-0.749513	0.340355	4.182310
H	-1.989575	1.581416	3.952986
H	-2.354109	0.114969	4.861381
H	-2.399362	-2.255146	1.962484
H	-2.507077	-2.111851	3.709719
H	-0.952023	-1.922054	2.918163
H	-4.268923	-0.466578	1.753099
H	-4.069062	1.089608	2.557888
H	-4.315647	-0.372068	3.509932
H	0.483638	1.291939	1.653263
H	0.312111	-0.313350	2.372733
H	6.370629	3.812576	0.873740
H	6.186005	5.365831	0.027026
H	4.949781	4.848232	1.196802

B3LYP energy = -1507.78701018 a.u.

(5*S*,1'*R*)-**10b**, Conf. K

C	3.460458	-1.112301	-0.944239
C	4.695888	-0.504308	-0.744797
C	5.119615	-0.215573	0.554689
C	4.295165	-0.550847	1.634540
C	3.070096	-1.158260	1.417616
C	2.625155	-1.445819	0.121078
C	1.282778	-2.093226	-0.163732
C	0.855541	-3.143532	0.887328
C	-0.380510	-3.937683	0.529959
C	-1.584355	-3.734328	1.207869
C	-2.723796	-4.463616	0.875343
C	-2.673621	-5.410190	-0.142614
C	-1.476124	-5.628999	-0.819834
C	-0.340152	-4.900825	-0.483598
N	0.215671	-1.068185	-0.369735
C	0.130078	0.105254	0.199231
N	-0.869971	0.862146	-0.219641
C	-1.284890	2.049998	0.508728
C	-0.628547	3.273025	0.271034
C	0.588191	3.418417	-0.635314
C	0.402603	4.505927	-1.705973
C	1.857111	3.692795	0.193957
C	-1.096079	4.398046	0.953111
C	-2.159568	4.316441	1.838154
C	-2.751615	3.090027	2.097084
C	-2.316605	1.927264	1.460227
C	-2.923546	0.592465	1.881542
C	-2.585482	0.285157	3.352135
C	-4.441238	0.532573	1.644809
C	-1.699357	0.086763	-1.221818
C	-2.073438	0.776672	-2.563999
C	-0.849827	1.183597	-3.397101
C	-2.963975	2.005706	-2.312035
C	-2.905279	-0.254588	-3.357546
C	-0.852200	-1.206039	-1.381655
C	7.194095	0.732264	-0.188475
O	6.300744	0.373316	0.868088
H	3.151461	-1.335704	-1.959338
H	5.314852	-0.271732	-1.599114
H	4.636732	-0.334371	2.638421
H	2.465644	-1.413717	2.278700
H	1.354868	-2.592891	-1.130855

H	1.704271	-3.820730	1.006957
H	0.704818	-2.652408	1.849932
H	-1.629575	-3.009571	2.012890
H	-3.646551	-4.298324	1.417985
H	-3.556696	-5.981733	-0.399665
H	-1.424662	-6.375541	-1.602896
H	0.589881	-5.092881	-1.007473
H	0.820713	0.437931	0.959582
H	0.743177	2.472132	-1.152741
H	1.258921	4.511252	-2.383495
H	-0.497184	4.341634	-2.300379
H	0.331015	5.499289	-1.259888
H	2.729967	3.743033	-0.460419
H	2.035540	2.912323	0.936144
H	1.782023	4.643668	0.725427
H	-0.616565	5.354341	0.789927
H	-2.512286	5.206275	2.344665
H	-3.551887	3.030226	2.823472
H	-2.469072	-0.203794	1.292227
H	-2.963419	-0.702030	3.626302
H	-1.506814	0.297394	3.521458
H	-3.037478	1.014821	4.026228
H	-4.824328	-0.454857	1.910461
H	-4.696440	0.726240	0.601405
H	-4.968812	1.266794	2.256454
H	-2.638959	-0.155157	-0.725285
H	-0.229169	1.914291	-2.881797
H	-0.221715	0.331506	-3.664472
H	-1.183647	1.637993	-4.331442
H	-3.849003	1.742699	-1.728657
H	-3.306763	2.407611	-3.267270
H	-2.438414	2.801372	-1.787148
H	-3.782143	-0.579865	-2.792678
H	-2.326349	-1.139719	-3.628215
H	-3.257104	0.199705	-4.284931
H	-1.429176	-2.107720	-1.197482
H	-0.389111	-1.281323	-2.364916
H	6.735542	1.458744	-0.863828
H	8.056485	1.182457	0.295564
H	7.512122	-0.147461	-0.753118

B3LYP energy = -1507.78695742 a.u.

(5*S*,1'*R*)-**10b**, Conf. L

C	3.227883	1.041532	1.222452
C	4.467377	0.437796	1.140314
C	5.104599	0.306048	-0.100993
C	4.476855	0.794943	-1.246319
C	3.223996	1.400071	-1.144972
C	2.577481	1.531479	0.080274
C	1.203149	2.161208	0.236007
C	0.800558	3.135329	-0.891619
C	-0.499769	3.866068	-0.645401
C	-0.558029	4.925538	0.265507
C	-1.753384	5.594991	0.504661
C	-2.913034	5.217553	-0.168199
C	-2.865969	4.171651	-1.084254
C	-1.667061	3.503112	-1.320654
N	0.164060	1.109085	0.439900
C	0.060566	-0.018385	-0.212974
N	-0.892603	-0.827807	0.213257
C	-1.318838	-1.996800	-0.536634
C	-0.520931	-3.158655	-0.525082
C	0.845986	-3.241501	0.143277
C	0.973257	-4.440040	1.097331
C	1.966543	-3.272990	-0.913587
C	-0.995672	-4.274430	-1.216435
C	-2.203399	-4.244815	-1.895520
C	-2.943692	-3.073897	-1.936016
C	-2.511950	-1.919496	-1.281576
C	-3.315621	-0.635620	-1.463693
C	-3.340824	-0.209621	-2.943524
C	-4.744478	-0.753634	-0.908513
C	-1.604865	-0.194895	1.391081
C	-1.630927	-0.993779	2.727199
C	-0.227825	-1.270413	3.287257
C	-2.392853	-2.319049	2.555215
C	-2.419057	-0.126446	3.732894
C	-0.879903	1.179743	1.481285
C	7.033798	-0.446904	-1.311317
O	6.318412	-0.300295	-0.082741
H	2.754321	1.137585	2.193311
H	4.967166	0.067202	2.025936
H	4.946341	0.717957	-2.216198
H	2.769131	1.775172	-2.052297
H	1.195629	2.717157	1.175093
H	1.615125	3.856193	-0.988676
H	0.739732	2.596479	-1.838784
H	0.341969	5.238852	0.783527
H	-1.777730	6.417854	1.208536
H	-3.842758	5.742418	0.012641
H	-3.759826	3.880903	-1.622584
H	-1.638124	2.698330	-2.047054
H	0.704951	-0.276845	-1.040483
H	0.991967	-2.340960	0.737893
H	1.935185	-4.403370	1.612539
H	0.185344	-4.441289	1.851945
H	0.924823	-5.389253	0.560961
H	2.944041	-3.269601	-0.427142
H	1.919399	-2.411222	-1.582898
H	1.899557	-4.172085	-1.529579
H	-0.407560	-5.182610	-1.222200
H	-2.557070	-5.128515	-2.411938
H	-3.864531	-3.048350	-2.504211
H	-2.816467	0.169073	-0.924924
H	-3.855335	0.747983	-3.047528
H	-2.331044	-0.098443	-3.344027
H	-3.867653	-0.938521	-3.561694
H	-5.268990	0.198219	-1.014496
H	-4.749364	-1.027909	0.147879
H	-5.317528	-1.509587	-1.448661
H	-2.640739	-0.043157	1.094653

H	0.346992	-1.931626	2.640673
H	0.352717	-0.358050	3.439807
H	-0.314355	-1.759728	4.258834
H	-3.405152	-2.151399	2.181413
H	-2.477598	-2.817321	3.522792
H	-1.891450	-3.003573	1.873813
H	-3.423381	0.096669	3.365023
H	-1.920545	0.818613	3.955789
H	-2.522939	-0.667832	4.674291
H	-1.544177	2.017418	1.278625
H	-0.406054	1.338832	2.447579
H	7.256841	0.525640	-1.756685
H	7.962550	-0.948496	-1.053264
H	6.475290	-1.059536	-2.023248

B3LYP energy = -1507.78689540 a.u.

(*R*)-**17a**, Conf. A

C	0.802542	0.524549	2.281369
C	2.321483	0.405587	2.025083
N	2.379808	-0.065437	0.616865
C	1.159626	-0.206170	0.139062
N	0.209022	0.092475	0.991631
C	3.606918	-0.329753	-0.094675
C	-1.253410	0.086283	0.787834
C	4.214601	0.722207	-0.803123
C	5.405338	0.440838	-1.475081
C	5.968442	-0.826707	-1.436776
C	5.352019	-1.844100	-0.722657
C	4.160236	-1.621038	-0.031068
C	3.621044	2.122469	-0.875694
C	3.508977	-2.764083	0.735455
C	4.594076	3.185681	-0.339720
C	3.165197	2.462649	-2.305268
C	3.001587	-3.858892	-0.219918
C	4.453046	-3.354197	1.795935
C	-1.643200	-0.736665	-0.459218
C	-1.796688	1.508471	0.778282
C	-3.134498	-0.968801	-0.550822
C	-1.357312	2.441120	-0.164191
C	-1.876434	3.730731	-0.174221
C	-2.843210	4.103226	0.757478
C	-3.284383	3.180550	1.699905
C	-2.760047	1.890192	1.710982
C	-3.925616	-0.229304	-1.430577
C	-5.299361	-0.436185	-1.512662
C	-5.882119	-1.397993	-0.700922
C	-5.123329	-2.153971	0.182364
C	-3.752171	-1.933461	0.248865
Cl	-7.628592	-1.672665	-0.796918
H	0.455448	-0.133989	3.076319
H	0.492077	1.544079	2.502451
H	2.840688	1.357701	2.115950
H	2.802280	-0.322066	2.676177
H	0.978381	-0.541302	-0.870635
H	-1.658311	-0.424044	1.663891
H	5.898350	1.223454	-2.037432
H	6.892418	-1.022811	-1.966948
H	5.803464	-2.827637	-0.702993
H	2.733907	2.152042	-0.242740
H	2.639418	-2.371382	1.263291
H	4.111203	4.165162	-0.336130
H	4.909368	2.959408	0.680716
H	5.489176	3.259264	-0.960748
H	2.689195	3.445396	-2.324020
H	2.447213	1.730259	-2.679477
H	4.011411	2.487462	-2.995202
H	2.488062	-4.640785	0.343699
H	2.303129	-3.455174	-0.955380
H	3.827683	-4.324082	-0.762254
H	3.929196	-4.113753	2.380091
H	4.811600	-2.585070	2.482621
H	5.323027	-3.829858	1.338363
H	-1.301686	-0.228792	-1.362730
H	-1.133717	-1.701840	-0.407076
H	-0.606154	2.168207	-0.896426
H	-1.527345	4.444986	-0.909576
H	-3.247067	5.107983	0.748686
H	-4.032913	3.462929	2.429737
H	-3.103898	1.176651	2.450685
H	-3.469622	0.519934	-2.065893
H	-5.902134	0.141470	-2.200010
H	-5.590369	-2.906083	0.803422
H	-3.159348	-2.531095	0.931562

B3LYP energy = -1695.55709368 a.u.

(*R*)-**17a**, Conf. B

C	-1.118527	0.769577	1.815826
C	-1.039647	-0.770358	1.930716
N	-0.737238	-1.191238	0.538040
C	-0.587433	-0.128461	-0.224586
N	-0.755387	1.014721	0.397870
C	-0.636440	-2.567897	0.117037
C	-0.714884	2.348480	-0.243523
C	0.615023	-3.205792	0.176314
C	0.674832	-4.541433	-0.225561
C	-0.459863	-5.212274	-0.657986
C	-1.684080	-4.560515	-0.699363
C	-1.804306	-3.225502	-0.310383
C	1.884003	-2.500312	0.634136
C	-3.161850	-2.539232	-0.382448
C	2.532913	-3.209870	1.834127
C	2.885146	-2.349851	-0.524618
C	-3.625495	-2.375189	-1.840529
C	-4.222955	-3.274368	0.453059
C	0.435204	3.210870	0.318664
C	-2.083337	3.015644	-0.177327
C	1.812311	2.644046	0.062159
C	-2.470715	3.824784	0.893015
C	-3.737686	4.401713	0.920325

C	-4.631146	4.180956	-0.122916
C	-4.252328	3.379045	-1.196146
C	-2.987651	2.801066	-1.220545
C	2.364555	2.668385	-1.222004
C	3.632902	2.157769	-1.472102
C	4.359278	1.618020	-0.418874
C	3.844101	1.581219	0.867732
C	2.571093	2.095893	1.097207
Cl	5.978141	0.970691	-0.725141
H	-2.119253	1.152683	2.006439
H	-0.415602	1.269307	2.478403
H	-0.240879	-1.100127	2.592901
H	-1.976356	-1.217138	2.257038
H	-0.349909	-0.196711	-1.276587
H	-0.499893	2.144749	-1.292335
H	1.622619	-5.063771	-0.201121
H	-0.389685	-6.248498	-0.965523
H	-2.559696	-5.097155	-1.041478
H	1.619385	-1.494400	0.961042
H	-3.063061	-1.537629	0.036555
H	3.393031	-2.636109	2.185368
H	1.831195	-3.312592	2.664102
H	2.886696	-4.207652	1.566698
H	3.764894	-1.795351	-0.192147
H	2.443388	-1.812139	-1.365955
H	3.219671	-3.324623	-0.886216
H	-4.574160	-1.835187	-1.875185
H	-2.895794	-1.818009	-2.431158
H	-3.774048	-3.345968	-2.318378
H	-5.158557	-2.711237	0.445371
H	-3.904473	-3.388972	1.490924
H	-4.431714	-4.268575	0.052697
H	0.345206	4.190346	-0.155614
H	0.298480	3.372125	1.388251
H	-1.790844	4.018567	1.712635
H	-4.022776	5.026917	1.757308
H	-5.614791	4.633375	-0.101964
H	-4.938425	3.206660	-2.016068
H	-2.699512	2.181417	-2.062230
H	1.806346	3.102038	-2.043663
H	4.051414	2.187710	-2.468832
H	4.422930	1.164308	1.680441
H	2.176048	2.077027	2.105879

B3LYP energy = -1695.55708698 a.u.

(*R*)-**17a**, Conf. C

C	-0.700676	0.568061	2.063715
C	-2.224882	0.334259	1.937774
N	-2.379578	-0.082027	0.519369
C	-1.199800	-0.119615	-0.064086
N	-0.197623	0.214247	0.713258
C	-3.645607	-0.391770	-0.099978
C	1.221847	0.258697	0.290747
C	-4.122678	-1.713260	-0.037492
C	-5.354099	-1.980867	-0.637751
C	-6.079784	-0.977889	-1.264444
C	-5.589121	0.319299	-1.304358
C	-4.364020	0.645825	-0.720612
C	-3.349825	-2.840244	0.633915
C	-3.854690	2.079066	-0.791509
C	-4.164104	-3.505317	1.756122
C	-2.874971	-3.881659	-0.394591
C	-3.532264	2.485366	-2.240133
C	-4.839311	3.069491	-0.147766
C	2.050438	-0.769069	1.096013
C	1.752509	1.684469	0.312107
C	3.420312	-1.005679	0.501024
C	1.823153	2.396564	-0.887473
C	2.286387	3.708159	-0.911414
C	2.682911	4.327931	0.269810
C	2.617452	3.627251	1.470535
C	2.159207	2.313315	1.491474
C	4.566744	-0.449977	1.070557
C	5.825498	-0.665362	0.516949
C	5.932003	-1.448672	-0.622470
C	4.812944	-2.020708	-1.212285
C	3.564796	-1.795316	-0.643129
Cl	7.527798	-1.732291	-1.335610
H	-0.457526	1.605298	2.284830
H	-0.243824	-0.074862	2.813091
H	-2.583261	-0.456689	2.594110
H	-2.805113	1.235957	2.122696
H	-1.073620	-0.403059	-1.099500
H	1.207811	-0.070366	-0.747733
H	-5.750368	-2.988032	-0.616506
H	-7.032854	-1.208650	-1.724498
H	-6.166550	1.090430	-1.797963
H	-2.456932	-2.416689	1.094453
H	-2.924201	2.140427	-0.226995
H	-3.552198	-4.249832	2.269743
H	-4.498191	-2.773468	2.494057
H	-5.046274	-4.015223	1.363721
H	-2.277070	-4.651558	0.097952
H	-2.262306	-3.422424	-1.172831
H	-3.721133	-4.373331	-0.879442
H	-3.113348	3.493663	-2.263646
H	-2.808043	1.805530	-2.693069
H	-4.430895	2.481128	-2.860956
H	-4.408912	4.073162	-0.143761
H	-5.065573	2.793134	0.883802
H	-5.780891	3.114744	-0.698954
H	2.149602	-0.449251	2.133453
H	1.489819	-1.706338	1.102565
H	1.517569	1.920554	-1.812188
H	2.339434	4.242756	-1.851679
H	3.044277	5.348652	0.254764
H	2.927881	4.100817	2.393604
H	2.130055	1.786397	2.436791
H	4.485938	0.158378	1.962849
H	6.706475	-0.231418	0.969871
H	4.911906	-2.637132	-2.095341

H	2.693941	-2.253390	-1.097805

B3LYP energy = -1695.55640775 a.u.

(*R*)-**17a**, Conf. D

C	0.473698	-0.709165	2.263171
C	2.007385	-0.862147	2.159959
N	2.294897	-0.339841	0.799780
C	1.167184	-0.048765	0.183884
N	0.088997	-0.257579	0.901293
C	3.619840	-0.234074	0.238017
C	-1.317939	0.069991	0.554576
C	4.344269	0.953416	0.444614
C	5.626620	1.027260	-0.101586
C	6.165367	-0.034331	-0.814431
C	5.431658	-1.197675	-0.996683
C	4.144507	-1.328529	-0.472303
C	3.781555	2.144297	1.208888
C	3.369639	-2.620604	-0.693057
C	4.673633	2.537615	2.397870
C	3.547326	3.344895	0.275347
C	3.054091	-2.837819	-2.183095
C	4.105636	-3.834540	-0.101499
C	-2.167111	-1.223290	0.525092
C	-1.405989	0.911123	-0.705816
C	-3.652691	-0.947093	0.484730
C	-1.291053	0.352713	-1.982060
C	-1.370549	1.156739	-3.114780
C	-1.574004	2.527966	-2.987810
C	-1.693495	3.092452	-1.721753
C	-1.607678	2.288070	-0.589614
C	-4.316950	-0.475193	1.620598
C	-5.682060	-0.212403	1.601742
C	-6.391158	-0.431180	0.428381
C	-5.765222	-0.906343	-0.714252
C	-4.397090	-1.159996	-0.676144
Cl	-8.131250	-0.104187	0.393349
H	-0.017837	-1.647081	2.507948
H	0.173682	0.049495	2.985925
H	2.541556	-0.277445	2.905619
H	2.329283	-1.900377	2.227486
H	1.140433	0.333597	-0.824914
H	-1.670364	0.684876	1.385770
H	6.211205	1.928711	0.030633
H	7.162424	0.045543	-1.230074
H	5.865074	-2.017261	-1.555536
H	2.810593	1.861928	1.616113
H	2.414713	-2.542088	-0.172926
H	4.203884	3.344314	2.964427
H	4.831704	1.695205	3.073966
H	5.651823	2.891611	2.066204
H	3.096354	4.170257	0.830581
H	2.880115	3.084889	-0.548663
H	4.486762	3.702179	-0.152195
H	2.448169	-3.737323	-2.311637
H	2.501992	-1.993545	-2.600626
H	3.968147	-2.963802	-2.767555
H	3.491354	-4.731342	-0.206440
H	4.319999	-3.692271	0.959457
H	5.052090	-4.018282	-0.614202
H	-1.871989	-1.835122	-0.327394
H	-1.941674	-1.803656	1.421356
H	-1.137472	-0.712016	-2.105673
H	-1.278419	0.709285	-4.096492
H	-1.641274	3.151473	-3.870567
H	-1.856136	4.157490	-1.613363
H	-1.703326	2.734395	0.393256
H	-3.769267	-0.316889	2.542535
H	-6.185067	0.149377	2.488172
H	-6.331074	-1.078926	-1.619610
H	-3.909981	-1.534440	-1.567944

B3LYP energy = -1695.55594142 a.u.

(*R*)-**17a**, Conf. E

C	1.506407	-0.077981	2.125364
C	0.818928	-1.458320	2.046480
N	0.144237	-1.411391	0.723246
C	0.446797	-0.287110	0.105292
N	1.219701	0.516440	0.796141
C	-0.666607	-2.480548	0.192131
C	1.772887	1.831431	0.421714
C	-0.055272	-3.461186	-0.610084
C	-0.866891	-4.483544	-1.104983
C	-2.220439	-4.534927	-0.804868
C	-2.794774	-3.562021	0.000642
C	-2.033713	-2.513970	0.520437
C	1.427869	-3.446867	-0.955661
C	-2.701764	-1.462664	1.396761
C	2.133772	-4.727208	-0.478370
C	1.652530	-3.221975	-2.461150
C	-3.746153	-0.655658	0.607061
C	-3.324367	-2.085808	2.657564
C	1.117928	2.435910	-0.833636
C	3.287779	1.756222	0.275449
C	-0.364993	2.738630	-0.748448
C	4.091589	2.654717	0.976585
C	5.475341	2.634631	0.822844
C	6.067931	1.709185	-0.030151
C	5.271350	0.805517	-0.729600
C	3.888964	0.829743	-0.578736
C	-0.910912	3.434710	0.333656
C	-2.263271	3.752972	0.378260
C	-3.080186	3.374646	-0.678607
C	-2.571036	2.689217	-1.770635
C	-1.214913	2.376666	-1.795488
Cl	-4.802938	3.774692	-0.629074
H	1.083416	0.560653	2.900266
H	2.581905	-0.152022	2.271304
H	1.528801	-2.283778	2.063602
H	0.083783	-1.608116	2.834011
H	0.079078	-0.063919	-0.883795

H	1.557277	2.481456	1.271690
H	-0.432686	-5.252048	-1.731811
H	-2.830541	-5.338266	-1.199397
H	-3.851778	-3.616578	0.227578
H	1.895877	-2.612162	-0.433293
H	-1.939496	-0.757147	1.727618
H	3.206395	-4.656101	-0.670676
H	1.990618	-4.885191	0.592258
H	1.760025	-5.609440	-1.001974
H	2.721497	-3.162854	-2.676878
H	1.185612	-2.295519	-2.801090
H	1.237302	-4.042814	-3.049882
H	-4.178234	0.120902	1.241709
H	-3.299233	-0.171638	-0.263112
H	-4.560746	-1.293725	0.257576
H	-3.736436	-1.302220	3.296848
H	-2.584055	-2.641728	3.236168
H	-4.136544	-2.770545	2.405073
H	1.660777	3.365907	-1.020316
H	1.322292	1.803113	-1.699768
H	3.635784	3.373874	1.647562
H	6.087673	3.337572	1.373874
H	7.144213	1.688768	-0.147950
H	5.726505	0.081214	-1.393644
H	3.283445	0.118882	-1.128240
H	-0.282248	3.749024	1.157436
H	-2.671733	4.293233	1.221334
H	-3.215834	2.401961	-2.589863
H	-0.818996	1.847700	-2.654623

B3LYP energy = -1695.55587900 a.u.

(*R*)-**17a**, Conf. F

C	-0.642569	0.169486	1.715975
C	0.371259	-0.996674	1.710180
N	1.212437	-0.691486	0.522704
C	0.730032	0.359750	-0.108448
N	-0.325344	0.892365	0.458334
C	2.351277	-1.479467	0.117415
C	-1.066140	2.101520	0.010812
C	3.614929	-1.154832	0.642384
C	4.698380	-1.938394	0.241615
C	4.530306	-2.999725	-0.636166
C	3.270944	-3.302695	-1.133246
C	2.150782	-2.554825	-0.766459
C	3.840056	0.010214	1.596904
C	0.785955	-2.914115	-1.338001
C	4.473625	-0.447798	2.921094
C	4.680945	1.116683	0.936438
C	0.732122	-2.668911	-2.856142
C	0.386073	-4.360131	-1.000271
C	-2.146771	1.749790	-1.035302
C	-0.107304	3.203068	-0.420188
C	-3.226766	0.822885	-0.526447
C	0.439923	4.033992	0.562480
C	1.331848	5.044450	0.223499
C	1.688786	5.241359	-1.108373
C	1.146396	4.423756	-2.093651
C	0.251895	3.411433	-1.752903
C	-4.221642	1.293143	0.336258
C	-5.217327	0.450529	0.816407
C	-5.217199	-0.881520	0.423757
C	-4.250702	-1.379297	-0.436912
C	-3.261627	-0.519424	-0.907178
Cl	-6.482960	-1.962274	1.026871
H	-1.673918	-0.173230	1.701150
H	-0.501378	0.845008	2.559273
H	0.988807	-1.025248	2.605059
H	-0.106237	-1.966504	1.578102
H	1.180402	0.748095	-1.010274
H	-1.569919	2.447418	0.913415
H	5.687608	-1.713545	0.619397
H	5.385466	-3.593678	-0.934855
H	3.156493	-4.133108	-1.818172
H	2.871015	0.447199	1.838617
H	0.041310	-2.261634	-0.881790
H	4.550732	0.396536	3.609317
H	3.875244	-1.224029	3.401928
H	5.479456	-0.844141	2.768282
H	4.790189	1.962817	1.618200
H	4.213455	1.477817	0.018307
H	5.681113	0.756521	0.685941
H	-0.268740	-2.885222	-3.235936
H	0.972851	-1.632966	-3.102483
H	1.437640	-3.312884	-3.385630
H	-0.627761	-4.558451	-1.354097
H	0.411543	-4.540087	0.076091
H	1.051103	-5.082087	-1.478457
H	-2.595542	2.693523	-1.352189
H	-1.675524	1.308295	-1.914305
H	0.160796	3.891487	1.600249
H	1.742017	5.681713	0.997039
H	2.380859	6.030206	-1.375562
H	1.413741	4.573258	-3.132321
H	-0.163118	2.795340	-2.539816
H	-4.230721	2.334592	0.636510
H	-5.983905	0.826913	1.479938
H	-4.267636	-2.416798	-0.741581
H	-2.511682	-0.906130	-1.586618

B3LYP energy = -1695.55529208 a.u.

Table S10. Cartesian coordinates and energies of the low-energy conformers of (5*S*,1'*S*)-**1a,** (5*S*,1'R)-**1b,** (5*S*,1'*S*)-**2a**, (5*S*,1'*R*)-**2b**, (5*S*,1'*R*)-**3a**, (5*S*,1'*R*)-**3b**, (5*S*,1'*S*)-**4a**, (5*S*,1'*R*)-**4b**, (5*R*,1'*R*)-**5a**, (5*R*,1'*S*)-**5b**, (5*S*,1'*S*)-**8a**, (5*S*,1'*R*)-**8b**, (5*S*,1'*S*)-**9a**, (5*S*,1'*R*)-**9b**, (5*S*,1'*S*)-**10a**, (5*S*,1'*R*)-**10b** and (*R*)-**17a**, calculated at the ωB97X/TZVP PCM (solvent: acetonitrile) level. Charge: +1.

(5*S*,1'*S*)-**1a**, Conf. A

C	-0.196154	-0.648123	1.363268
N	-0.680049	-0.256752	0.002839
C	0.256007	-0.495466	-0.894042
N	1.341545	-1.025368	-0.410427
C	1.227458	-1.168432	1.045173
C	-1.077223	-1.638834	2.157192
C	-1.802250	0.613013	-0.270076
C	2.565903	-1.407849	-1.125223
C	-2.926719	0.106978	-0.935875
C	-4.009956	0.962521	-1.112960
C	-3.975513	2.268614	-0.657598
C	-2.830755	2.760709	-0.056338
C	-1.712406	1.953870	0.129182
C	-0.366522	-1.876869	3.496809
C	-1.265787	-2.972782	1.434721
C	-2.442571	-1.011383	2.455119
C	-0.444206	2.578081	0.691699
C	-2.996218	-1.294392	-1.519088
C	-4.196311	-2.086913	-0.997817
C	-3.017024	-1.231824	-3.049810
C	0.067982	3.683770	-0.235840
C	-0.649154	3.106136	2.112669
C	3.715550	-0.517648	-0.687108
C	2.367614	-1.397124	-2.633290
C	4.882698	-1.081703	-0.188982
C	5.949854	-0.274216	0.186354
C	5.851554	1.104202	0.071335
C	4.684185	1.674503	-0.422844
C	3.622993	0.867140	-0.801529
H	-0.131413	0.262998	1.960444
H	0.124083	-0.254474	-1.941154
H	2.008005	-0.583052	1.532021
H	1.363838	-2.217063	1.309018
H	2.781473	-2.431251	-0.807778
H	-4.896427	0.598263	-1.619988
H	-4.836108	2.913261	-0.794088
H	-2.796687	3.798248	0.257062
H	-0.214171	-0.937172	4.034696
H	0.604390	-2.361972	3.374259
H	-0.980184	-2.526348	4.123883
H	-0.319315	-3.486302	1.250506
H	-1.878117	-3.635148	2.050153
H	-1.776051	-2.848607	0.478988
H	-3.035288	-0.857974	1.553655
H	-2.334198	-0.045466	2.956516
H	-3.009218	-1.669363	3.117562
H	0.341123	1.821254	0.724189
H	-2.093252	-1.833489	-1.231080
H	-4.178200	-3.099916	-1.405723
H	-4.192668	-2.161600	0.091730
H	-5.138648	-1.624605	-1.300242
H	-3.004275	-2.240050	-3.469327
H	-2.154115	-0.688466	-3.441694
H	-3.918961	-0.729393	-3.408066
H	1.010742	4.083393	0.144502
H	0.239697	3.302193	-1.244819
H	-0.644166	4.509491	-0.301777
H	0.285513	3.513271	2.503762
H	-0.988952	2.320595	2.792403
H	-1.395086	3.904238	2.129274
H	3.279534	-1.767469	-3.100073
H	1.543788	-2.047523	-2.932465
H	2.190118	-0.388873	-3.012980
H	4.960486	-2.159757	-0.090948
H	6.855683	-0.725237	0.574583
H	6.680895	1.735886	0.367869
H	4.600916	2.751259	-0.514792
H	2.716565	1.325184	-1.187584

ωB97X energy = -1161.81409603 a.u.

(5*S*,1'*S*)-**1a**, Conf. B

C	0.210204	-1.064665	1.018167
N	0.710413	-0.281841	-0.156071
C	-0.176160	-0.318912	-1.128612
N	-1.227591	-1.041461	-0.871411
C	-1.082273	-1.690203	0.436690
C	0.000829	-0.276335	2.332549
C	2.033093	0.273829	-0.318559
C	-2.450168	-1.111648	-1.688586
C	2.189400	1.664385	-0.417133
C	3.484434	2.162854	-0.518316
C	4.580995	1.319290	-0.519508
C	4.397178	-0.050674	-0.467580
C	3.122829	-0.604667	-0.393619
C	1.328282	0.308913	2.824815
C	-0.477334	-1.294180	3.377778
C	-1.038140	0.837369	2.193210
C	2.977838	-2.117402	-0.449055
C	1.027581	2.643798	-0.463018
C	1.138227	3.725240	0.613618
C	0.912154	3.276345	-1.853370
C	3.605641	-2.679100	-1.727740
C	3.574422	-2.790188	0.789245
C	-3.559410	-0.350438	-0.975839

C	-2.765232	-2.557453	-2.035927
C	-4.577638	-0.991839	-0.280482
C	-5.542247	-0.252430	0.394943
C	-5.497921	1.133039	0.379308
C	-4.486191	1.782031	-0.318644
C	-3.526089	1.043044	-0.990558
H	0.936980	-1.848891	1.228720
H	-0.035564	0.201827	-2.068165
H	-0.994860	-2.768108	0.301435
H	-1.961864	-1.483680	1.045011
H	-2.210492	-0.575709	-2.609397
H	3.636455	3.233375	-0.596452
H	5.581452	1.731208	-0.583644
H	5.259933	-0.706138	-0.507739
H	1.710645	1.088560	2.166210
H	2.095582	-0.465571	2.912749
H	1.185632	0.750411	3.813518
H	0.247091	-2.104277	3.498498
H	-0.588686	-0.797702	4.343548
H	-1.443784	-1.735346	3.124796
H	-2.013881	0.464376	1.869920
H	-0.719744	1.606223	1.488012
H	-1.182166	1.322335	3.160918
H	1.916473	-2.366054	-0.496194
H	0.101721	2.098248	-0.278913
H	0.250696	4.361508	0.595325
H	1.224674	3.293094	1.612952
H	2.007768	4.364615	0.446867
H	0.041562	3.934246	-1.897864
H	0.809384	2.519888	-2.635225
H	1.798175	3.872785	-2.084578
H	3.426998	-3.754695	-1.789227
H	3.177974	-2.210286	-2.616263
H	4.686004	-2.519125	-1.746746
H	3.416702	-3.869917	0.746699
H	3.125187	-2.416878	1.713064
H	4.650558	-2.610106	0.849390
H	-3.657041	-2.597171	-2.661321
H	-1.933319	-2.996678	-2.586731
H	-2.947087	-3.164630	-1.148077
H	-4.633342	-2.073961	-0.259367
H	-6.330914	-0.765445	0.933051
H	-6.251225	1.707969	0.905356
H	-4.448510	2.864953	-0.342961
H	-2.740771	1.557859	-1.537457

ωB97X energy = -1161.81378530 a.u.

(5*S*,1'*S*)-**1a**, Conf. C

C	1.033929	-1.791517	-0.132441
N	0.611121	-0.357329	-0.161036
C	-0.684298	-0.273936	-0.373210
N	-1.285525	-1.421650	-0.510948
C	-0.287288	-2.499114	-0.526915
C	1.656910	-2.302287	1.188923
C	1.467979	0.802632	-0.112805
C	-2.713455	-1.644642	-0.821445
C	1.368694	1.688857	0.970882
C	2.251827	2.763426	1.007848
C	3.195584	2.952844	0.013999
C	3.238528	2.093584	-1.069231
C	2.360629	1.018892	-1.171363
C	2.028664	-3.773347	0.954644
C	0.694147	-2.200386	2.372555
C	2.947075	-1.539637	1.506169
C	2.375222	0.179429	-2.439263
C	0.331515	1.561702	2.076274
C	0.971566	1.513271	3.465331
C	-0.692286	2.698212	1.992753
C	2.084809	1.047884	-3.666695
C	3.694975	-0.576005	-2.607875
C	-3.497685	-0.361679	-0.643567
C	-3.258966	-2.793131	0.019223
C	-4.019777	0.289547	-1.754476
C	-4.738331	1.469803	-1.606939
C	-4.931478	2.011508	-0.345362
C	-4.409631	1.367457	0.770578
C	-3.701306	0.185196	0.623030
H	1.781801	-1.935613	-0.912881
H	-1.200391	0.674853	-0.436449
H	-0.248161	-2.935420	-1.526361
H	-0.564320	-3.275882	0.181119
H	-2.754957	-1.930155	-1.876390
H	2.201593	3.464933	1.832981
H	3.885716	3.786511	0.072777
H	3.953810	2.274565	-1.863786
H	2.720479	-3.875910	0.114250
H	1.157380	-4.401739	0.757746
H	2.521518	-4.169271	1.844525
H	-0.234437	-2.751914	2.207703
H	1.166309	-2.621970	3.262397
H	0.439238	-1.164728	2.599768
H	2.765043	-0.492943	1.749747
H	3.646677	-1.574465	0.666357
H	3.437406	-2.000372	2.366456
H	1.567864	-0.551871	-2.385399
H	-0.214608	0.627690	1.940470
H	0.204839	1.339996	4.223571
H	1.711471	0.713411	3.541852
H	1.469132	2.455251	3.705968
H	-1.461947	2.568828	2.757131
H	-1.185390	2.730976	1.017745
H	-0.214200	3.667204	2.155638
H	2.027079	0.423040	-4.560551
H	1.136774	1.578814	-3.558580
H	2.871906	1.788132	-3.827332
H	3.662641	-1.199517	-3.503827
H	3.907353	-1.221569	-1.751947
H	4.531068	0.119553	-2.713709
H	-4.319199	-2.916810	-0.200459
H	-2.758191	-3.733194	-0.214036
H	-3.147324	-2.592051	1.086175

H	-3.864647	-0.128303	-2.743764
H	-5.143585	1.965870	-2.481118
H	-5.486306	2.935199	-0.228739
H	-4.556882	1.787224	1.758937
H	-3.297034	-0.306886	1.502197

ωB97X energy = -1161.81300165 a.u.

(5*S*,1’*R*)-**1b**, Conf. A

C	0.219262	-1.074245	1.049163
N	0.705723	-0.271397	-0.116326
C	-0.199594	-0.270354	-1.073237
N	-1.272223	-0.955011	-0.798626
C	-1.128075	-1.610917	0.505509
C	0.112061	-0.336463	2.404126
C	2.047326	0.228855	-0.308772
C	-2.498837	-1.087962	-1.594996
C	2.267488	1.613431	-0.355196
C	3.580583	2.054682	-0.485167
C	4.633843	1.161267	-0.564353
C	4.385625	-0.199350	-0.562657
C	3.089585	-0.695839	-0.461521
C	-0.369264	-1.376244	3.426028
C	-0.871793	0.833667	2.366013
C	1.489113	0.160825	2.855572
C	2.872160	-2.196935	-0.572515
C	1.152062	2.645146	-0.318752
C	1.353196	3.682099	0.787886
C	1.010561	3.330482	-1.681424
C	3.413669	-2.727952	-1.902790
C	3.495683	-2.948402	0.605905
C	-3.665219	-0.455623	-0.856056
C	-2.325812	-0.517174	-2.994313
C	-3.612563	0.877447	-0.456953
C	-4.689137	1.457947	0.195193
C	-5.832953	0.710991	0.451553
C	-5.891767	-0.616786	0.055411
C	-4.808952	-1.198553	-0.592957
H	0.915329	-1.900952	1.192738
H	-0.047464	0.248484	-2.010826
H	-1.123335	-2.692064	0.363191
H	-1.974006	-1.346626	1.138616
H	-2.681638	-2.162948	-1.672657
H	3.781038	3.119365	-0.524325
H	5.650257	1.527816	-0.650089
H	5.213247	-0.892364	-0.663695
H	0.324460	-2.219270	3.486456
H	-1.361948	-1.767030	3.191863
H	-0.424553	-0.915889	4.414242
H	-0.566102	1.601025	1.653402
H	-1.887911	0.521387	2.111860
H	-0.916198	1.302431	3.351298
H	1.417571	0.550341	3.873360
H	1.872090	0.960336	2.221534
H	2.224362	-0.648817	2.859196
H	1.799809	-2.397737	-0.577010
H	0.211677	2.132847	-0.115473
H	2.246725	4.284694	0.610815
H	0.497887	4.360318	0.822055
H	1.452936	3.212541	1.769001
H	0.174333	4.032725	-1.666175
H	0.835002	2.607548	-2.481819
H	1.915758	3.889243	-1.931782
H	4.497277	-2.607955	-1.970019
H	3.189834	-3.792529	-1.998675
H	2.962238	-2.206206	-2.749158
H	3.293276	-4.018187	0.522552
H	3.102813	-2.601234	1.565023
H	4.579785	-2.812376	0.624037
H	-1.500856	-0.996954	-3.523509
H	-3.241829	-0.700936	-3.554366
H	-2.163558	0.562747	-2.972002
H	-2.724311	1.471416	-0.652352
H	-4.636481	2.495745	0.503324
H	-6.675398	1.164907	0.960390
H	-6.779698	-1.205446	0.254907
H	-4.856429	-2.239773	-0.895450

ωB97X energy = -1161.81484095 a.u.

(5*S*,1’*R*)-**1b**, Conf. B

C	-0.109278	-0.412672	1.329156
N	-0.673407	-0.172056	-0.036515
C	0.243944	-0.417201	-0.949145
N	1.386863	-0.816065	-0.471897
C	1.342837	-0.839589	0.994282
C	-0.875362	-1.406344	2.231886
C	-1.893529	0.541562	-0.336674
C	2.568589	-1.198282	-1.259360
C	-2.956904	-0.137671	-0.947103
C	-4.136933	0.568504	-1.159259
C	-4.255795	1.895419	-0.785752
C	-3.172822	2.558170	-0.235872
C	-1.962372	1.906137	-0.022976
C	-0.979257	-2.803428	1.620151
C	-2.276681	-0.870628	2.542684
C	-0.098150	-1.483425	3.553649
C	-0.773220	2.710141	0.479078
C	-2.866580	-1.575643	-1.429317
C	-3.966229	-2.458165	-0.835701
C	-2.896481	-1.629891	-2.959819
C	-0.442556	3.851880	-0.486405
C	-1.003716	3.246313	1.893538
C	3.741916	-0.318798	-0.848489
C	2.801506	-2.698438	-1.159497
C	4.910553	-0.840072	-0.308841
C	5.955356	0.002792	0.056165
C	5.839367	1.372766	-0.113560
C	4.672805	1.902494	-0.653772
C	3.634896	1.061523	-1.018714
H	-0.099223	0.545005	1.851327

H	0.063678	-0.281171	-2.009027
H	2.086003	-0.147978	1.391508
H	1.574830	-1.842056	1.350828
H	2.312860	-0.957954	-2.293899
H	-4.978445	0.069616	-1.626635
H	-5.189648	2.421724	-0.946053
H	-3.262227	3.610008	0.011863
H	-1.556638	-2.801664	0.695270
H	-1.487098	-3.469039	2.321266
H	-0.001351	-3.242885	1.409383
H	-2.229836	0.131812	2.977510
H	-2.912478	-0.825253	1.658361
H	-2.763015	-1.525594	3.268833
H	0.007377	-0.495100	4.009259
H	0.899014	-1.911170	3.428167
H	-0.640002	-2.119837	4.255829
H	0.105614	2.063262	0.499685
H	-1.908144	-1.987237	-1.112074
H	-3.963715	-2.429028	0.256214
H	-4.955207	-2.144965	-1.177798
H	-3.820679	-3.494951	-1.146804
H	-2.778905	-2.659972	-3.303016
H	-2.095307	-1.030793	-3.399122
H	-3.845931	-1.251433	-3.346586
H	0.459232	4.370848	-0.154380
H	-0.269105	3.476620	-1.497192
H	-1.252012	4.583978	-0.530126
H	-0.113707	3.769051	2.249946
H	-1.836672	3.953383	1.910099
H	-1.234586	2.446107	2.601247
H	1.910256	-3.230867	-1.492527
H	3.634067	-2.988616	-1.800537
H	3.031097	-3.011839	-0.139673
H	5.023505	-1.907976	-0.167412
H	6.861064	-0.419171	0.476122
H	6.653364	2.028911	0.172172
H	4.573955	2.972647	-0.794081
H	2.728405	1.485693	-1.441064

ωB97X energy = -1161.81317719 a.u.

(5*S*,1’*R*)-**1b**, Conf. C

C	1.039181	1.686148	-0.600258
N	0.618928	0.387705	0.013587
C	-0.655094	0.437411	0.340355
N	-1.224978	1.584537	0.108344
C	-0.221454	2.551439	-0.352081
C	1.497089	1.629229	-2.077055
C	1.462168	-0.735879	0.341731
C	-2.650439	1.930161	0.282495
C	1.248966	-1.967863	-0.295871
C	2.112716	-3.013188	0.016385
C	3.146236	-2.846159	0.921004
C	3.306028	-1.635034	1.569844
C	2.453921	-0.564598	1.317048
C	2.730041	0.731417	-2.221186
C	1.909270	3.057375	-2.460935
C	0.392933	1.147650	-3.019062
C	2.608350	0.705292	2.138942
C	0.111222	-2.230462	-1.270980
C	0.612090	-2.772332	-2.611389
C	-0.923084	-3.179429	-0.657019
C	2.482098	0.408360	3.635700
C	3.929556	1.416923	1.838817
C	-3.469589	0.659290	0.400330
C	-2.814426	2.922045	1.425102
C	-3.866355	0.143086	1.628835
C	-4.570607	-1.053556	1.692488
C	-4.882747	-1.743015	0.530319
C	-4.492936	-1.231245	-0.701377
C	-3.792172	-0.037087	-0.763465
H	1.874953	2.077621	-0.020894
H	-1.178311	-0.407429	0.769193
H	-0.057041	3.303138	0.420463
H	-0.580658	3.048730	-1.251765
H	-2.933806	2.422459	-0.652280
H	1.973874	-3.976785	-0.460540
H	3.816851	-3.669832	1.136840
H	4.094149	-1.525439	2.306361
H	2.505573	-0.319519	-2.038880
H	3.523422	1.031961	-1.531060
H	3.123797	0.815256	-3.236473
H	1.073259	3.759413	-2.429319
H	2.695088	3.432208	-1.799597
H	2.299654	3.060812	-3.480349
H	0.754562	1.181046	-4.048935
H	-0.501198	1.774022	-2.970642
H	0.099900	0.117687	-2.811799
H	1.789528	1.382615	1.892348
H	-0.400002	-1.289758	-1.476676
H	1.070287	-3.757026	-2.497029
H	-0.223129	-2.873749	-3.307725
H	1.351272	-2.107455	-3.063867
H	-1.757689	-3.322926	-1.347161
H	-1.325542	-2.791116	0.282644
H	-0.481533	-4.157435	-0.450614
H	3.297043	-0.228221	3.987358
H	2.518934	1.340590	4.203406
H	1.538370	-0.092615	3.860574
H	3.994034	2.350966	2.400814
H	4.036152	1.650958	0.776588
H	4.779510	0.792720	2.125709
H	-2.279358	3.847910	1.211242
H	-3.869577	3.166025	1.549171
H	-2.434111	2.515247	2.363091
H	-3.630148	0.667794	2.547178
H	-4.875099	-1.446014	2.655736
H	-5.431305	-2.676302	0.582509
H	-4.738696	-1.760785	-1.614512
H	-3.488426	0.361671	-1.726944

ωB97X energy = -1161.81289458 a.u.

(5*S*,1'*R*)-**1b**, Conf. D

C	-0.373706	0.787311	-1.326137
N	-0.689640	0.277988	0.046596
C	0.351038	0.461560	0.832222
N	1.340392	1.107264	0.283051
C	0.986181	1.489648	-1.090071
C	-1.428412	1.670392	-2.028027
C	-1.724712	-0.680551	0.370864
C	2.602391	1.444890	0.950969
C	-2.808046	-0.284595	1.166178
C	-3.810524	-1.221038	1.401488
C	-3.736432	-2.499403	0.877907
C	-2.628414	-2.882316	0.142918
C	-1.589530	-1.991771	-0.107067
C	-2.708453	0.869956	-2.291256
C	-0.823950	2.055962	-3.386129
C	-1.766278	2.939048	-1.245744
C	-0.350233	-2.498204	-0.828730
C	-2.914334	1.083957	1.816522
C	-4.210591	1.804219	1.439646
C	-2.788504	0.966534	3.338725
C	0.313778	-3.630911	-0.041031
C	-0.662047	-2.937138	-2.260581
C	3.697180	0.427509	0.656407
C	3.013058	2.869989	0.593390
C	3.545211	-0.610198	-0.254790
C	4.578363	-1.513201	-0.479824
C	5.774167	-1.391899	0.208675
C	5.930999	-0.363342	1.130192
C	4.901974	0.537742	1.350339
H	-0.226630	-0.088366	-1.963134
H	0.385352	0.086607	1.848211
H	1.752876	1.161850	-1.790750
H	0.903989	2.574567	-1.149031
H	2.386565	1.407064	2.021103
H	-4.663705	-0.943313	2.010040
H	-4.534581	-3.208716	1.063965
H	-2.558682	-3.898984	-0.227523
H	-3.234663	0.609530	-1.372485
H	-2.494756	-0.055707	-2.832976
H	-3.386879	1.465598	-2.905694
H	0.069780	2.675452	-3.281203
H	-0.560046	1.168534	-3.967867
H	-1.554029	2.629015	-3.960628
H	-2.494129	3.525815	-1.810103
H	-0.895663	3.577897	-1.083627
H	-2.207767	2.714785	-0.275079
H	0.382747	-1.691684	-0.877898
H	-2.079045	1.693880	1.470607
H	-5.083840	1.280390	1.835123
H	-4.212343	2.812838	1.858704
H	-4.331178	1.887004	0.357269
H	-2.818386	1.957753	3.796353
H	-1.852182	0.485173	3.629715
H	-3.611151	0.379452	3.754644
H	-0.332972	-4.509527	0.013692
H	1.243857	-3.929342	-0.529881
H	0.548241	-3.318170	0.978798
H	-1.366561	-3.772133	-2.267618
H	0.250712	-3.263651	-2.763070
H	-1.102255	-2.126673	-2.846769
H	2.213983	3.572920	0.833124
H	3.895279	3.149797	1.166870
H	3.262275	2.956085	-0.465712
H	2.617277	-0.739823	-0.799639
H	4.440472	-2.314118	-1.197110
H	6.578300	-2.097458	0.035475
H	6.858530	-0.264172	1.682202
H	5.034901	1.328176	2.082050

ωB97X energy = -1161.81066697 a.u.

(5*S*,1'*S*)-**2a**, Conf. A

C	0.724461	-1.169975	1.044017
N	1.172828	-0.365964	-0.137565
C	0.316441	-0.507913	-1.126039
N	-0.684159	-1.298781	-0.865757
C	-0.515252	-1.896556	0.464320
C	0.451466	-0.389617	2.351850
C	2.436615	0.316306	-0.286401
C	-1.849909	-1.551735	-1.734226
C	3.607763	-0.452354	-0.328849
C	4.823164	0.222132	-0.385695
C	4.872878	1.603169	-0.450813
C	3.699469	2.335730	-0.482716
C	2.457060	1.714572	-0.401894
C	1.733584	0.284584	2.851014
C	0.034024	-1.432091	3.399054
C	-0.658026	0.652249	2.201097
C	1.206002	2.574834	-0.482837
C	3.609340	-1.972505	-0.367333
C	4.308349	-2.485701	-1.629398
C	4.247139	-2.572208	0.887638
C	1.170342	3.655238	0.599784
C	1.071067	3.202349	-1.873875
C	-3.110268	-1.191665	-0.960308
C	-1.790534	-2.972841	-2.274759
C	-3.388232	0.180907	-0.648382
C	-4.494760	0.481694	0.185856
C	-5.331994	-0.567142	0.647111
C	-5.074986	-1.857569	0.301068
C	-3.950694	-2.166113	-0.498047
C	-2.607055	1.267299	-1.127154
C	-2.884913	2.555747	-0.767494
C	-3.967260	2.843653	0.090515
C	-4.757995	1.828557	0.543866
H	1.506589	-1.896199	1.265247
H	0.446248	-0.013286	-2.081077
H	-0.357060	-2.970528	0.364585
H	-1.415019	-1.730491	1.053955

H	-1.741696	-0.867067	-2.577409
H	5.746913	-0.345391	-0.400906
H	5.829764	2.109789	-0.499591
H	3.748504	3.415226	-0.571215
H	2.071355	1.083010	2.190413
H	2.548234	-0.438191	2.951235
H	1.554200	0.721899	3.835522
H	0.805231	-2.197085	3.524458
H	-0.109540	-0.940458	4.363044
H	-0.903641	-1.930938	3.144739
H	-1.606673	0.213594	1.879809
H	-0.390324	1.434397	1.489908
H	-0.834857	1.134201	3.164851
H	0.332656	1.939331	-0.329987
H	2.577354	-2.321786	-0.426013
H	4.221534	-3.572789	-1.687050
H	3.861596	-2.058979	-2.529677
H	5.371807	-2.236230	-1.626844
H	4.194752	-3.662475	0.854844
H	3.748484	-2.235164	1.799842
H	5.299792	-2.288689	0.962667
H	0.218159	4.189632	0.558885
H	1.276757	3.229834	1.600005
H	1.968638	4.387426	0.458618
H	0.139644	3.769356	-1.944079
H	1.068332	2.445359	-2.661828
H	1.896760	3.888958	-2.075559
H	-2.644080	-3.159538	-2.926419
H	-0.877041	-3.102236	-2.855192
H	-1.796545	-3.717668	-1.478049
H	-6.179305	-0.318917	1.277375
H	-5.717343	-2.659216	0.645472
H	-3.758784	-3.206454	-0.728783
H	-1.790374	1.091439	-1.815694
H	-2.275023	3.366071	-1.151778
H	-4.174192	3.869509	0.371355
H	-5.606224	2.035744	1.187746

ωB97X energy = -1315.45723534 a.u.

(5*S*,1’*S*)-**2a**, Conf. B

C	1.550872	-0.702889	-1.567302
N	1.061773	-0.234658	-0.233028
C	-0.156072	-0.686274	-0.016707
N	-0.639814	-1.415140	-0.980504
C	0.319626	-1.486413	-2.087681
C	2.869352	-1.509287	-1.574934
C	1.644702	0.824444	0.559156
C	-1.942923	-2.107401	-1.011491
C	1.626586	2.127632	0.042879
C	2.268066	3.122515	0.774066
C	2.859532	2.845845	1.993613
C	2.793857	1.567250	2.518231
C	2.184965	0.529668	1.818779
C	4.026246	-0.633769	-1.083016
C	3.148951	-1.878500	-3.038715
C	2.787428	-2.783355	-0.733796
C	2.090726	-0.838479	2.473535
C	0.901950	2.513540	-1.237522
C	-0.185817	3.553942	-0.955964
C	1.873603	3.017037	-2.307309
C	3.462066	-1.390660	2.867729
C	1.162238	-0.786511	3.691157
C	-2.898314	-1.468001	-0.019334
C	-1.728912	-3.605478	-0.842199
C	-3.399935	-0.147049	-0.267855
C	-4.279921	0.437834	0.677018
C	-4.649929	-0.291642	1.837124
C	-4.164166	-1.544630	2.049816
C	-3.281777	-2.130298	1.113864
C	-3.051194	0.614794	-1.415617
C	-3.549124	1.873133	-1.606185
C	-4.424965	2.449610	-0.662023
C	-4.781305	1.744614	0.450627
H	1.716416	0.180140	-2.185751
H	-0.714996	-0.450109	0.880408
H	-0.121023	-1.036704	-2.978863
H	0.553379	-2.526981	-2.306021
H	-2.330062	-1.934137	-2.019686
H	2.286502	4.136461	0.390323
H	3.352391	3.635559	2.548795
H	3.224263	1.369407	3.493475
H	4.969183	-1.170517	-1.207537
H	3.930323	-0.371172	-0.029500
H	4.092330	0.294428	-1.657971
H	3.197024	-0.986718	-3.669587
H	4.111435	-2.388958	-3.106988
H	2.393765	-2.550196	-3.452927
H	1.991521	-3.455164	-1.065323
H	2.628128	-2.564486	0.322566
H	3.727751	-3.332408	-0.815748
H	1.649218	-1.534434	1.759764
H	0.387832	1.635485	-1.632014
H	-0.735076	3.775258	-1.874003
H	-0.899923	3.188680	-0.214068
H	0.242035	4.489022	-0.587365
H	1.338197	3.236094	-3.233432
H	2.650988	2.281364	-2.529190
H	2.369766	3.934377	-1.981521
H	3.354246	-2.397077	3.278062
H	4.139673	-1.444580	2.012787
H	3.934821	-0.770045	3.632298
H	1.053580	-1.782378	4.126008
H	0.167692	-0.419751	3.426066
H	1.565860	-0.124637	4.461415
H	-2.683597	-4.127425	-0.905321
H	-1.088141	-3.987215	-1.637514
H	-1.258541	-3.835784	0.114627
H	-5.326087	0.169182	2.549330
H	-4.445945	-2.102608	2.934704
H	-2.912971	-3.128572	1.312293
H	-2.384041	0.202455	-2.163186

H	-3.270413	2.433539	-2.491276
H	-4.813203	3.447882	-0.826357
H	-5.456495	2.173963	1.183267

ωB97X energy = -1315.45718137 a.u.

(5*S*,1'*S*)-**2a**, Conf. C

C	1.628463	-1.619371	0.640902
N	1.159020	-0.417138	-0.116538
C	-0.052047	-0.626948	-0.581492
N	-0.544062	-1.801947	-0.300961
C	0.500241	-2.630618	0.317858
C	1.891285	-1.418771	2.153169
C	1.910470	0.783786	-0.392136
C	-1.830270	-2.336010	-0.796840
C	3.052822	0.686460	-1.198064
C	3.816807	1.835112	-1.379810
C	3.433628	3.044854	-0.829305
C	2.259537	3.130614	-0.102415
C	1.472263	2.008540	0.135456
C	2.974116	-0.358884	2.381127
C	2.428725	-2.755698	2.682963
C	0.626917	-1.036442	2.924186
C	0.171848	2.184077	0.903476
C	3.462392	-0.588725	-1.918333
C	3.541100	-0.361604	-3.430614
C	4.786118	-1.142432	-1.386125
C	0.380387	2.833317	2.272561
C	-0.838290	2.983084	0.073840
C	-2.796972	-1.216516	-1.141191
C	-2.406135	-3.367509	0.169723
C	-3.270705	-0.260579	-0.180433
C	-4.206928	0.718002	-0.605578
C	-4.657622	0.728698	-1.951389
C	-4.190577	-0.184994	-2.844974
C	-3.251741	-1.154657	-2.430694
C	-2.841563	-0.217749	1.175004
C	-3.316450	0.724849	2.042919
C	-4.254746	1.687135	1.614547
C	-4.687031	1.679041	0.320737
H	2.564945	-1.953133	0.194677
H	-0.593930	0.114656	-1.154668
H	0.817778	-3.395035	-0.393761
H	0.115197	-3.121644	1.208075
H	-1.600557	-2.859664	-1.728885
H	4.716555	1.785214	-1.982600
H	4.039520	3.929556	-0.987025
H	1.946955	4.090541	0.292932
H	2.639062	0.645048	2.121429
H	3.874859	-0.576049	1.800501
H	3.253426	-0.351562	3.436915
H	3.347916	-3.042317	2.165142
H	2.657742	-2.658838	3.745811
H	1.708798	-3.570090	2.576962
H	0.871416	-0.893777	3.978707
H	-0.139796	-1.813814	2.876321
H	0.193802	-0.101764	2.562780
H	-0.270147	1.204291	1.083860
H	2.687542	-1.340764	-1.762472
H	3.748779	-1.306303	-3.937607
H	2.602248	0.036782	-3.820494
H	4.339496	0.337435	-3.689334
H	5.036307	-2.077382	-1.891790
H	4.744874	-1.337855	-0.311716
H	5.600940	-0.435962	-1.562742
H	-0.570951	2.877105	2.808347
H	1.087559	2.268473	2.884260
H	0.756112	3.854566	2.178708
H	-1.796909	3.035554	0.595815
H	-1.010146	2.527046	-0.904846
H	-0.484159	4.003074	-0.094642
H	-3.343100	-3.735899	-0.247469
H	-1.734923	-4.220266	0.277824
H	-2.612416	-2.953751	1.156075
H	-5.377263	1.482186	-2.253081
H	-4.529159	-0.175393	-3.874025
H	-2.886578	-1.872077	-3.157792
H	-2.122695	-0.939079	1.539056
H	-2.968227	0.732382	3.069722
H	-4.625751	2.427394	2.313570
H	-5.406097	2.414325	-0.024489

ωB97X energy = -1315.45630869 a.u.

(5*S*,1'*S*)-**2a**, Conf. D

C	-0.947488	-0.130371	1.504513
N	-1.391645	-0.132117	0.075652
C	-0.374132	-0.401850	-0.714526
N	0.753684	-0.595439	-0.092393
C	0.568689	-0.409434	1.351568
C	-1.680358	-1.097910	2.463144
C	-2.664443	0.332402	-0.425427
C	2.045777	-0.992270	-0.657700
C	-2.980386	1.691295	-0.289454
C	-4.244358	2.104921	-0.698746
C	-5.139635	1.216962	-1.267611
C	-4.774518	-0.103468	-1.459967
C	-3.533393	-0.577020	-1.045562
C	-3.164867	-0.732027	2.564129
C	-1.049172	-0.894018	3.847679
C	-1.539614	-2.562813	2.049534
C	-3.173750	-2.026531	-1.328385
C	-2.002081	2.735427	0.226977
C	-1.752756	3.814458	-0.830620
C	-2.478766	3.361176	1.539480
C	-4.199684	-3.002452	-0.748953
C	-3.002379	-2.253515	-2.833609
C	3.073755	0.118510	-0.495414
C	1.916435	-1.430915	-2.110483
C	4.451575	-0.206042	-0.285253
C	5.391063	0.851891	-0.177373
C	4.951592	2.196143	-0.284016

C	3.636091	2.477736	-0.494736
C	2.697271	1.428876	-0.600783
C	4.935338	-1.539875	-0.189227
C	6.261413	-1.797630	0.010776
C	7.189934	-0.740671	0.126110
C	6.761327	0.550807	0.033533
H	-1.099632	0.876472	1.895126
H	-0.476880	-0.445387	-1.790916
H	1.185328	0.421259	1.695003
H	0.885523	-1.314502	1.869170
H	2.348949	-1.853204	-0.058834
H	-4.524210	3.146703	-0.589505
H	-6.117682	1.560921	-1.583781
H	-5.468569	-0.783238	-1.941110
H	-3.633279	-1.331406	3.347695
H	-3.704557	-0.919686	1.635701
H	-3.299123	0.321324	2.825551
H	-1.137363	0.146097	4.172825
H	-1.566657	-1.518672	4.578127
H	0.007274	-1.170432	3.870729
H	-0.497860	-2.888324	2.003110
H	-2.000412	-2.757393	1.080671
H	-2.042748	-3.196065	2.783229
H	-2.214590	-2.245842	-0.858565
H	-1.038364	2.257635	0.409309
H	-0.997093	4.518072	-0.474715
H	-1.398561	3.375823	-1.765848
H	-2.661882	4.380824	-1.044192
H	-1.742659	4.079924	1.905411
H	-2.635013	2.608962	2.316730
H	-3.423636	3.891239	1.397452
H	-3.864813	-4.030422	-0.903551
H	-4.342610	-2.849645	0.323154
H	-5.171096	-2.893669	-1.236228
H	-2.697372	-3.284104	-3.027059
H	-2.247097	-1.588536	-3.259741
H	-3.940878	-2.074116	-3.363867
H	2.887105	-1.787245	-2.454635
H	1.197697	-2.245278	-2.219342
H	1.626659	-0.598087	-2.754372
H	5.682909	2.992680	-0.198154
H	3.298688	3.503809	-0.581177
H	1.655821	1.682812	-0.770546
H	4.253886	-2.376810	-0.277892
H	6.605631	-2.822933	0.081682
H	8.239070	-0.959892	0.286241
H	7.464955	1.372496	0.116704

ωB97X energy = -1315.45589428 a.u.

(5*S*,1’*S*)-**2a**, Conf. E

C	-0.676470	-0.728506	1.324233
N	-1.187810	-0.298621	-0.014684
C	-0.298269	-0.577280	-0.945675
N	0.775800	-1.167175	-0.506691
C	0.685714	-1.359428	0.945085
C	-1.592243	-1.645599	2.165935
C	-2.289749	0.608054	-0.247002
C	1.904427	-1.703910	-1.283959
C	-3.442055	0.140768	-0.894226
C	-4.505588	1.026955	-1.037012
C	-4.428530	2.323704	-0.561568
C	-3.260338	2.775577	0.025717
C	-2.158246	1.939139	0.173529
C	-2.890532	-0.916923	2.527727
C	-0.834116	-1.932977	3.469753
C	-1.915915	-2.964238	1.463955
C	-0.869777	2.523307	0.733656
C	-3.570472	-1.254715	-1.482127
C	-4.777862	-2.009109	-0.921626
C	-3.638228	-1.189082	-3.011020
C	-0.381019	3.689933	-0.129010
C	-1.031593	2.958958	2.191714
C	3.229980	-1.270590	-0.676474
C	1.776481	-1.410262	-2.772600
C	3.615904	0.100873	-0.515796
C	4.882813	0.383265	0.059757
C	5.739821	-0.680874	0.442616
C	5.355112	-1.974694	0.270986
C	4.089305	-2.260870	-0.284277
C	2.800948	1.205575	-0.888116
C	3.218002	2.493013	-0.700057
C	4.476080	2.764318	-0.122847
C	5.286057	1.730595	0.244752
H	-0.509620	0.175484	1.912228
H	-0.452638	-0.315753	-1.984832
H	1.525662	-0.867398	1.434843
H	0.733212	-2.426891	1.163020
H	1.845421	-2.788501	-1.158297
H	-5.411778	0.693636	-1.530166
H	-5.274565	2.992544	-0.669574
H	-3.195409	3.805320	0.358820
H	-3.520248	-0.729292	1.658027
H	-2.685862	0.043249	3.009639
H	-3.465538	-1.525579	3.228954
H	-0.591949	-1.006689	3.997906
H	-1.458758	-2.540660	4.127094
H	0.094549	-2.482630	3.299822
H	-1.021747	-3.552365	1.246068
H	-2.453969	-2.806003	0.529054
H	-2.551997	-3.572704	2.110201
H	-0.085607	1.764994	0.691841
H	-2.676801	-1.823824	-1.223973
H	-4.801606	-3.024926	-1.322260
H	-4.744656	-2.075929	0.168039
H	-5.714625	-1.521005	-1.199784
H	-4.529840	-0.650129	-3.340727
H	-3.680880	-2.196751	-3.429920
H	-2.766441	-0.682177	-3.431137
H	0.566449	4.068460	0.261532
H	-0.225826	3.378273	-1.164697
H	-1.095251	4.516131	-0.127068

H	-1.770426	3.759293	2.278211
H	-0.083377	3.333897	2.582496
H	-1.360709	2.134998	2.829843
H	2.619050	-1.879400	-3.279085
H	0.861517	-1.840720	-3.183142
H	1.805148	-0.344393	-2.998928
H	6.704957	-0.441848	0.876329
H	6.006664	-2.789192	0.563977
H	3.793198	-3.298305	-0.399985
H	1.832997	1.042279	-1.340461
H	2.576522	3.313513	-1.000977
H	4.793378	3.790202	0.022446
H	6.257620	1.921496	0.688106

ωB97X energy = -1315.45541486 a.u.

(5*S*,1'*R*)-**2b**, Conf. A

C	-1.584488	-1.189994	-1.258393
N	-1.105932	-0.393077	-0.086083
C	0.068538	-0.831825	0.309958
N	0.533910	-1.829764	-0.386033
C	-0.456638	-2.247746	-1.384867
C	-1.877330	-0.411584	-2.563596
C	-1.838475	0.624708	0.627501
C	1.849491	-2.483405	-0.235840
C	-2.986866	0.251548	1.337901
C	-3.727583	1.260590	1.945761
C	-3.315615	2.580366	1.896163
C	-2.135344	2.911727	1.254214
C	-1.369760	1.947196	0.606931
C	-2.403670	-1.444869	-3.569977
C	-0.636260	0.270982	-3.140852
C	-2.978841	0.630341	-2.341784
C	-0.059568	2.367591	-0.039902
C	-3.423804	-1.193380	1.522090
C	-3.476432	-1.557969	3.008646
C	-4.767873	-1.475343	0.847683
C	-0.247157	3.488512	-1.063685
C	0.963218	2.774172	1.025592
C	2.794020	-1.577919	0.536456
C	1.665104	-3.881522	0.337884
C	3.273416	-0.367953	-0.068973
C	4.133575	0.476219	0.677178
C	4.509193	0.106633	1.995217
C	4.047359	-1.048367	2.546518
C	3.183972	-1.890363	1.809108
C	2.915827	0.039034	-1.382476
C	3.382706	1.208986	-1.913068
C	4.240312	2.043520	-1.165331
C	4.608670	1.679678	0.097337
H	-2.514237	-1.678725	-0.964920
H	0.601016	-0.397289	1.146670
H	-0.811363	-3.252683	-1.157328
H	0.005946	-2.258919	-2.371552
H	2.232511	-2.586473	-1.255590
H	-4.631501	1.004318	2.486876
H	-3.904261	3.351093	2.380151
H	-1.800623	3.942991	1.254528
H	-3.308250	-1.932604	-3.197216
H	-1.667352	-2.218889	-3.797769
H	-2.653320	-0.945196	-4.507861
H	0.141037	-0.443197	-3.422863
H	-0.911239	0.815306	-4.046676
H	-0.203667	0.992245	-2.445911
H	-2.651700	1.455940	-1.709721
H	-3.865208	0.183722	-1.883269
H	-3.278283	1.048110	-3.305274
H	0.362038	1.514740	-0.572780
H	-2.673358	-1.846000	1.073527
H	-3.708441	-2.618836	3.124533
H	-2.519928	-1.362365	3.497480
H	-4.247863	-0.989045	3.532580
H	-5.040476	-2.525155	0.974173
H	-4.740777	-1.259041	-0.223153
H	-5.560844	-0.866963	1.289403
H	0.702784	3.696780	-1.561939
H	-0.979620	3.219351	-1.828023
H	-0.581913	4.412447	-0.586963
H	1.922773	3.003264	0.555625
H	1.127103	1.976467	1.755419
H	0.629100	3.660241	1.571119
H	2.631271	-4.378010	0.426056
H	1.042149	-4.482241	-0.325155
H	1.188556	-3.851842	1.318612
H	5.168781	0.762580	2.553247
H	4.332707	-1.331711	3.552532
H	2.832468	-2.800359	2.278538
H	2.268350	-0.584231	-1.987679
H	3.096513	1.495387	-2.918764
H	4.606040	2.966778	-1.599102
H	5.269067	2.310503	0.682883

ωB97X energy = -1315.45861347 a.u.

(5*S*,1'*R*)-**2b**, Conf. B

C	-0.659583	-0.753593	1.266644
N	-1.189505	-0.262989	-0.044603
C	-0.300220	-0.478373	-0.991772
N	0.783219	-1.078583	-0.592500
C	0.707053	-1.351259	0.847197
C	-1.557170	-1.721112	2.069701
C	-2.280039	0.671097	-0.216431
C	1.893618	-1.527219	-1.454516
C	-3.453933	0.249705	-0.855551
C	-4.502122	1.160807	-0.943876
C	-4.387359	2.438527	-0.425796
C	-3.197598	2.847100	0.150304
C	-2.110589	1.983750	0.245694
C	-0.772679	-2.086340	3.337992
C	-1.896285	-2.994699	1.295465
C	-2.846212	-1.014396	2.500165

C	-0.794056	2.521933	0.786187
C	-3.615958	-1.119268	-1.494649
C	-4.828839	-1.874406	-0.947748
C	-3.701081	-0.992929	-3.018806
C	-0.284801	3.675841	-0.082022
C	-0.911361	2.952265	2.249691
C	3.211566	-1.177702	-0.780693
C	1.704651	-2.996074	-1.810320
C	3.604724	0.195670	-0.647550
C	4.811257	0.497779	0.034033
C	5.610760	-0.556130	0.548153
C	5.227500	-1.852056	0.392143
C	4.018679	-2.157679	-0.273233
C	2.847046	1.280894	-1.165431
C	3.258645	2.574173	-1.004806
C	4.453067	2.867305	-0.314040
C	5.210854	1.849622	0.187438
H	-0.498754	0.122839	1.897226
H	-0.458192	-0.164106	-2.017099
H	1.545619	-0.877979	1.356882
H	0.760687	-2.426677	1.015801
H	1.805864	-0.943748	-2.373727
H	-5.424868	0.863880	-1.429534
H	-5.221092	3.127892	-0.492834
H	-3.103704	3.863972	0.514714
H	-0.500380	-1.192158	3.905301
H	0.141226	-2.642647	3.117501
H	-1.392163	-2.715183	3.980094
H	-1.005900	-3.546659	0.984994
H	-2.483661	-3.660045	1.931630
H	-2.490249	-2.783467	0.406176
H	-3.482744	-0.755571	1.653800
H	-2.628087	-0.095245	3.051278
H	-3.419436	-1.670026	3.159302
H	-0.035700	1.739327	0.726067
H	-2.728174	-1.711469	-1.270957
H	-4.874808	-2.873726	-1.386134
H	-4.783128	-1.983260	0.138068
H	-5.760860	-1.361073	-1.194865
H	-3.771431	-1.982621	-3.475232
H	-2.822674	-0.490213	-3.430071
H	-4.584205	-0.422016	-3.316143
H	0.687448	4.015083	0.283027
H	-0.168736	3.365230	-1.123097
H	-0.968643	4.527283	-0.056126
H	0.056333	3.295434	2.621472
H	-1.250459	2.133252	2.888987
H	-1.622884	3.774218	2.358988
H	2.517839	-3.334412	-2.452504
H	0.765730	-3.121084	-2.350551
H	1.675306	-3.633014	-0.924840
H	6.532507	-0.308238	1.063705
H	5.838745	-2.658613	0.779097
H	3.739891	-3.199276	-0.371449
H	1.932791	1.097956	-1.715064
H	2.664299	3.382059	-1.416247
H	4.766289	3.897444	-0.191605
H	6.137148	2.058268	0.712294

ωB97X energy = -1315.45665002 a.u.

(5*S*,1’*R*)-**2b**, Conf. C

C	0.929871	-0.520975	1.379468
N	1.412388	-0.206896	-0.002064
C	0.427227	-0.340282	-0.866228
N	-0.712073	-0.679799	-0.334058
C	-0.549592	-0.890584	1.109041
C	1.130560	0.569812	2.456691
C	2.794172	-0.081956	-0.407614
C	-1.994590	-0.922021	-1.001489
C	3.615483	-1.216048	-0.334821
C	4.963836	-1.059489	-0.640139
C	5.468193	0.161619	-1.050553
C	4.621464	1.247132	-1.186810
C	3.269442	1.152852	-0.871734
C	0.409376	1.875749	2.122054
C	2.622980	0.841643	2.671391
C	0.566120	-0.004689	3.763957
C	2.383520	2.367946	-1.089584
C	3.099175	-2.604738	0.008069
C	3.369393	-3.584588	-1.137127
C	3.692902	-3.125836	1.318821
C	2.882290	3.596260	-0.325870
C	2.255253	2.677692	-2.584097
C	-3.044283	0.073140	-0.527467
C	-1.854840	-0.916429	-2.517335
C	-4.406306	-0.335170	-0.367758
C	-5.359597	0.628451	0.052461
C	-4.950664	1.964206	0.295995
C	-3.650749	2.330940	0.121192
C	-2.698750	1.375598	-0.294571
C	-4.862056	-1.658642	-0.618966
C	-6.174469	-1.997298	-0.451578
C	-7.115996	-1.036826	-0.022947
C	-6.714650	0.243495	0.221368
H	1.472821	-1.403148	1.720794
H	0.567742	-0.190922	-1.928930
H	-0.772301	-1.931511	1.346103
H	-1.249419	-0.253844	1.648578
H	-2.278482	-1.925736	-0.680192
H	5.626968	-1.914895	-0.576017
H	6.521386	0.262606	-1.285684
H	5.018410	2.189881	-1.546056
H	0.559453	2.591704	2.932824
H	-0.669084	1.741449	2.007067
H	0.795409	2.332153	1.209698
H	3.089654	1.316236	1.808344
H	2.750874	1.508494	3.526761
H	3.167733	-0.082395	2.884563
H	1.065178	-0.939933	4.031866
H	-0.508239	-0.193447	3.708060
H	0.731134	0.707951	4.574181

H	1.383753	2.138197	-0.720679
H	2.015031	-2.558252	0.121302
H	2.922281	-4.554841	-0.910700
H	2.943584	-3.222249	-2.075084
H	4.440482	-3.737505	-1.286911
H	3.264118	-4.098843	1.567167
H	3.500575	-2.446006	2.152706
H	4.775394	-3.247886	1.233648
H	2.174965	4.420031	-0.444185
H	3.850087	3.933216	-0.704236
H	2.990822	3.394458	0.742033
H	1.574937	3.517996	-2.738041
H	1.870244	1.820921	-3.141968
H	3.224439	2.945154	-3.012118
H	-2.823390	-1.156201	-2.955594
H	-1.135456	-1.663724	-2.856574
H	-1.562760	0.068981	-2.886803
H	-5.692368	2.686524	0.619565
H	-3.336571	3.352294	0.300751
H	-1.670397	1.695469	-0.427282
H	-4.170360	-2.420938	-0.954874
H	-6.497203	-3.012897	-0.649384
H	-8.153726	-1.320388	0.107984
H	-7.428218	0.992913	0.547249

ωB97X energy = -1315.45633822 a.u.

(5*S*,1’*R*)-**2b**, Conf. D

C	0.778374	-1.167492	1.103581
N	1.191223	-0.376536	-0.098915
C	0.324187	-0.552983	-1.073457
N	-0.681992	-1.320096	-0.768732
C	-0.521622	-1.838857	0.594116
C	0.615384	-0.399070	2.435924
C	2.463304	0.277607	-0.300403
C	-1.850031	-1.670899	-1.591931
C	2.507745	1.675540	-0.407178
C	3.757714	2.272199	-0.540039
C	4.915609	1.514982	-0.566000
C	4.839998	0.134669	-0.509241
C	3.614516	-0.515151	-0.402366
C	0.208204	-1.443992	3.484995
C	-0.451798	0.693843	2.369150
C	1.951135	0.209656	2.875242
C	3.582206	-2.034375	-0.458899
C	1.270813	2.558727	-0.426368
C	1.313848	3.640173	0.655107
C	1.075057	3.187798	-1.809256
C	4.199898	-2.542688	-1.764753
C	4.274679	-2.666058	0.750435
C	-3.142240	-1.280509	-0.887628
C	-1.729220	-1.161694	-3.022143
C	-3.465120	0.066193	-0.513959
C	-4.690590	0.305339	0.162133
C	-5.577621	-0.771191	0.421945
C	-5.257944	-2.037782	0.041226
C	-4.027785	-2.285315	-0.605539
C	-2.625916	1.186583	-0.764381
C	-2.971125	2.444093	-0.358098
C	-4.185040	2.669790	0.323599
C	-5.023480	1.622695	0.570124
H	1.543902	-1.926874	1.269699
H	0.454000	-0.098510	-2.047515
H	-0.447539	-2.926099	0.559404
H	-1.394889	-1.569775	1.187093
H	-1.839835	-2.762930	-1.636617
H	3.825617	3.350977	-0.624330
H	5.879742	2.002082	-0.654684
H	5.749978	-0.451619	-0.571423
H	0.952586	-2.241527	3.558405
H	-0.760834	-1.899164	3.268108
H	0.131946	-0.965402	4.463081
H	-1.437698	0.305326	2.101568
H	-0.547016	1.166003	3.349259
H	-0.191797	1.475470	1.654878
H	2.283830	1.009792	2.213703
H	2.739604	-0.547082	2.913937
H	1.844237	0.630534	3.877302
H	2.541940	-2.363156	-0.465333
H	0.396760	1.937001	-0.228175
H	0.370113	4.190530	0.668871
H	1.471416	3.213622	1.648130
H	2.114275	4.359010	0.466538
H	0.150059	3.769116	-1.831596
H	1.020294	2.430639	-2.595074
H	1.900771	3.860743	-2.052983
H	4.089928	-3.627142	-1.831492
H	3.711759	-2.095647	-2.633201
H	5.266168	-2.311754	-1.818990
H	4.201549	-3.754508	0.701629
H	3.829270	-2.337521	1.692729
H	5.334640	-2.401418	0.774759
H	-2.588048	-1.530879	-3.581454
H	-0.827941	-1.550059	-3.499678
H	-1.733477	-0.074121	-3.095370
H	-6.512543	-0.563938	0.931441
H	-5.932461	-2.862024	0.239813
H	-3.779525	-3.305423	-0.879714
H	-1.693942	1.063097	-1.296417
H	-2.307502	3.276597	-0.565495
H	-4.447671	3.671876	0.641461
H	-5.965125	1.779597	1.085626

ωB97X energy = -1315.45576614 a.u.

(5*S*,1’*R*)-**2b**, Conf. E

C	1.490495	-0.994422	-1.410733
N	1.091019	-0.332629	-0.129023
C	-0.131833	-0.699835	0.192176
N	-0.664933	-1.580771	-0.608056
C	0.316147	-1.979329	-1.625534
C	2.887460	-1.650460	-1.485049

C	1.715625	0.834440	0.456076
C	-1.928634	-2.307533	-0.354216
C	1.611248	2.060964	-0.215000
C	2.288317	3.151339	0.322377
C	2.999072	3.041832	1.504075
C	3.024533	1.838296	2.185448
C	2.383961	0.710130	1.681950
C	3.002781	-2.244414	-2.896471
C	3.086419	-2.761320	-0.453903
C	3.987810	-0.596400	-1.323853
C	2.404400	-0.568506	2.501656
C	0.762221	2.272199	-1.459448
C	-0.311036	3.335821	-1.210754
C	1.616087	2.638514	-2.675321
C	3.829984	-1.019478	2.827658
C	1.586887	-0.399765	3.785948
C	-2.906527	-1.452126	0.430331
C	-2.512253	-2.868034	-1.647810
C	-3.406288	-0.197478	-0.053760
C	-4.358022	0.500690	0.734176
C	-4.795700	-0.050335	1.966457
C	-4.303023	-1.239867	2.407389
C	-3.349737	-1.935513	1.631813
C	-2.993323	0.401541	-1.275915
C	-3.502720	1.600581	-1.686382
C	-4.453161	2.285440	-0.900716
C	-4.867306	1.744786	0.281061
H	1.463052	-0.224164	-2.185701
H	-0.657160	-0.287268	1.045324
H	-0.111746	-1.896633	-2.622605
H	0.605397	-3.017202	-1.452125
H	-1.665226	-3.160907	0.276373
H	2.240071	4.109352	-0.182866
H	3.515622	3.904837	1.908037
H	3.550921	1.772479	3.130921
H	2.870427	-1.475388	-3.662415
H	2.269795	-3.035040	-3.073723
H	3.994324	-2.681612	-3.027805
H	2.368811	-3.576039	-0.569524
H	4.082988	-3.190677	-0.576632
H	3.013137	-2.387791	0.566862
H	4.017424	-0.172691	-0.319590
H	3.859188	0.224237	-2.034815
H	4.959258	-1.055586	-1.519144
H	1.928993	-1.358672	1.919986
H	0.229325	1.347343	-1.687735
H	-0.947078	3.435716	-2.093054
H	-0.947087	3.066076	-0.364541
H	0.134681	4.312058	-1.007690
H	0.986737	2.741593	-3.561696
H	2.374922	1.880410	-2.883664
H	2.130735	3.589344	-2.517142
H	3.809724	-1.988795	3.330630
H	4.440570	-1.117240	1.927177
H	4.327020	-0.311061	3.494183
H	1.573851	-1.335919	4.348398
H	0.553492	-0.117434	3.571888
H	2.019490	0.372375	4.427008
H	-3.416503	-3.421163	-1.394910
H	-1.820287	-3.564419	-2.122804
H	-2.780284	-2.092666	-2.364057
H	-5.526800	0.498017	2.550790
H	-4.632991	-1.660137	3.349837
H	-2.964187	-2.880468	2.000002
H	-2.261100	-0.089559	-1.901985
H	-3.171019	2.031943	-2.623832
H	-4.848930	3.235891	-1.238776
H	-5.595457	2.260129	0.898616

ωB97X energy = -1315.45447723 a.u.

(5*S*,1’*S*)-**3a**, Conf. A

C	0.491202	-0.925599	0.977334
C	-0.916816	-0.401526	1.354733
N	-1.525984	-0.192173	0.004393
C	-0.657506	-0.504087	-0.935439
N	0.487375	-0.935054	-0.489757
C	-1.690165	-1.304415	2.343238
C	-0.854542	-1.346810	3.630691
C	-1.889594	-2.726511	1.819295
C	-3.048147	-0.682819	2.684660
C	1.632601	-1.441401	-1.258278
C	-2.735567	0.546469	-0.278026
C	-3.841623	-0.125830	-0.815774
C	-5.007322	0.607069	-1.016466
C	-5.072477	1.952203	-0.699215
C	-3.950365	2.604095	-0.219384
C	-2.752172	1.924820	-0.022919
C	-1.521349	2.713020	0.397672
C	-1.195361	3.800645	-0.629862
C	-1.681360	3.316613	1.794576
C	-3.815312	-1.587916	-1.229253
C	-4.925417	-2.398531	-0.557478
C	-3.896207	-1.716772	-2.753408
C	1.352468	-1.445706	-2.753408
C	2.873992	-0.645319	-0.907924
C	3.991725	-1.280682	-0.450515
C	5.178641	-0.559557	-0.156015
C	5.190949	0.843199	-0.334760
C	4.012360	1.483160	-0.802692
C	2.889951	0.763095	-1.082324
C	6.349444	-1.204975	0.316409
C	7.476133	-0.484426	0.592831
C	7.489345	0.917747	0.411821
C	6.374852	1.565317	-0.039770
H	1.289659	-0.277345	1.337898
H	0.676154	-1.937668	1.337840
H	-0.828490	0.578097	1.827116
H	-0.885462	-0.392061	-1.987423
H	-0.698805	-0.342706	4.034388
H	0.122967	-1.810112	3.478844

H	-1.380655	-1.933456	4.386107
H	-0.944510	-3.230961	1.606177
H	-2.496654	-2.746093	0.913953
H	-2.408919	-3.320622	2.574191
H	-2.935682	0.347076	3.034952
H	-3.731900	-0.677777	1.835554
H	-3.518930	-1.256487	3.485989
H	1.781681	-2.472395	-0.925608
H	-5.880409	0.114671	-1.429571
H	-5.995871	2.499748	-0.848628
H	-3.999540	3.667719	-0.014536
H	-0.661900	2.040570	0.413882
H	-0.268106	4.307691	-0.354317
H	-1.068970	3.375602	-1.627861
H	-1.985450	4.553193	-0.678305
H	-0.765143	3.830962	2.091929
H	-1.902436	2.554061	2.545525
H	-2.495529	4.045203	1.812202
H	-2.862321	-2.018210	-0.920486
H	-4.819581	-3.456056	-0.808922
H	-4.892418	-2.304197	0.530257
H	-5.913133	-2.074869	-0.893202
H	-3.835806	-2.767108	-3.046475
H	-3.082848	-1.178677	-3.246476
H	-4.839393	-1.314082	-3.130887
H	2.214778	-1.877717	-3.259494
H	0.479499	-2.053622	-2.997374
H	1.209365	-0.435741	-3.143257
H	3.988045	-2.357268	-0.306120
H	4.019904	2.559718	-0.936992
H	1.999176	1.272695	-1.437049
H	6.334792	-2.281140	0.454238
H	8.367533	-0.984836	0.953115
H	8.391335	1.476162	0.634161
H	6.380056	2.641223	-0.179896

ωB97X energy = -1315.45992597 a.u.

(5*S*,1’*S*)-**3a**, Conf. B

C	0.397909	-1.282062	0.900597
C	-0.962179	-0.670128	1.318417
N	-1.523987	-0.263078	-0.008047
C	-0.661637	-0.540084	-0.965615
N	0.427899	-1.122864	-0.557009
C	-1.835573	-1.594484	2.195746
C	-1.024821	-1.880909	3.467845
C	-2.179549	-2.913519	1.504764
C	-3.120530	-0.870819	2.610296
C	1.587973	-1.547757	-1.351586
C	-2.624427	0.652382	-0.212888
C	-3.809735	0.188709	-0.799501
C	-4.864289	1.088915	-0.919622
C	-4.744342	2.397116	-0.485825
C	-3.543790	2.845000	0.036232
C	-2.451802	1.992067	0.162517
C	-1.125319	2.567921	0.635365
C	-0.617672	3.625201	-0.349516
C	-1.221129	3.137495	2.051724
C	-3.979095	-1.216297	-1.352807
C	-5.164193	-1.948176	-0.719818
C	-4.118985	-1.179039	-2.877735
C	1.302799	-1.486453	-2.844317
C	2.799163	-0.722849	-0.960078
C	3.939252	-1.330592	-0.520910
C	5.092375	-0.573347	-0.183494
C	5.046124	0.835329	-0.299651
C	3.845609	1.445433	-0.751042
C	2.757532	0.691409	-1.072401
C	6.285998	-1.188325	0.272143
C	7.378299	-0.433267	0.591846
C	7.332705	0.974788	0.473149
C	6.195216	1.593511	0.039015
H	1.246999	-0.761419	1.343894
H	0.476310	-2.342495	1.140647
H	-0.795768	0.242478	1.893820
H	-0.851187	-0.286123	-2.000752
H	-0.751093	-0.954344	3.979813
H	-0.109727	-2.441125	3.262493
H	-1.627002	-2.478516	4.154735
H	-1.290272	-3.482509	1.224363
H	-2.779375	-2.758023	0.607979
H	-2.762059	-3.538713	2.184702
H	-2.896868	0.077585	3.106949
H	-3.773636	-0.660857	1.763124
H	-3.678308	-1.492262	3.314300
H	1.773023	-2.587789	-1.071461
H	-5.795973	0.758724	-1.365169
H	-5.582555	3.078148	-0.577679
H	-3.444905	3.883765	0.330988
H	-0.374718	1.776521	0.644018
H	0.361853	3.990821	-0.033196
H	-0.519182	3.210195	-1.355255
H	-1.295361	4.480590	-0.399607
H	-0.246674	3.508253	2.376322
H	-1.555046	2.384017	2.769493
H	-1.926534	3.970815	2.092465
H	-3.077577	-1.787002	-1.127756
H	-5.219363	-2.969936	-1.101886
H	-5.076248	-1.998184	0.367659
H	-6.108638	-1.453577	-0.957963
H	-4.190696	-2.194206	-3.274035
H	-3.262741	-0.689467	-3.347605
H	-5.020176	-0.637676	-3.175988
H	2.164891	-1.891076	-3.372907
H	0.429731	-2.085643	-3.108053
H	1.155197	-0.461344	-3.190054
H	3.979899	-2.411684	-0.423450
H	3.807977	2.526103	-0.839347
H	1.850212	1.179855	-1.415156
H	6.317251	-2.269110	0.362784
H	8.287142	-0.910964	0.939119
H	8.207675	1.560945	0.729523

H	6.154822	2.673800	-0.053593

ωB97X energy = -1315.45991975 a.u.

(5*S*,1'*S*)-**3a**, Conf. C

C	-0.381247	-1.407081	0.573081
C	0.937792	-0.807491	1.123745
N	1.548283	-0.242575	-0.121572
C	0.718528	-0.388243	-1.132770
N	-0.399333	-0.983616	-0.831650
C	0.747107	0.171527	2.305943
C	-0.179541	1.338665	1.964094
C	2.100278	0.707385	2.784527
C	0.137743	-0.647849	3.452502
C	-1.530425	-1.221032	-1.742005
C	2.916866	0.189366	-0.285120
C	3.188051	1.537266	-0.563342
C	4.522555	1.921483	-0.652082
C	5.546616	1.008761	-0.473336
C	5.251795	-0.323722	-0.248303
C	3.935159	-0.767898	-0.175460
C	3.674479	-2.259331	-0.030168
C	4.319434	-3.040423	-1.178647
C	4.157500	-2.788840	1.321908
C	2.111919	2.581044	-0.815088
C	2.243468	3.788320	0.115406
C	2.127887	3.026083	-2.280887
C	-1.681557	-2.709950	-2.011101
C	-2.763394	-0.525745	-1.185234
C	-3.904842	-1.198616	-0.858125
C	-5.037251	-0.517226	-0.335220
C	-4.974147	0.881889	-0.145693
C	-3.777733	1.562962	-0.495013
C	-2.711954	0.882607	-0.999599
C	-6.229020	-1.205449	0.005745
C	-7.302106	-0.528143	0.511329
C	-7.237680	0.870983	0.702286
C	-6.102072	1.559056	0.381756
H	-0.395905	-2.495606	0.629012
H	-1.265515	-1.020857	1.078264
H	1.588056	-1.608017	1.476576
H	0.951295	-0.045804	-2.133861
H	-1.176648	1.009461	1.660720
H	0.227399	1.962821	1.167380
H	-0.302640	1.973107	2.844273
H	2.573350	1.361527	2.052397
H	2.794629	-0.107227	3.008928
H	1.957346	1.283517	3.701309
H	-0.849038	-1.046162	3.206890
H	0.785643	-1.485244	3.725799
H	0.023430	-0.011926	4.332336
H	-1.255753	-0.723693	-2.675788
H	4.763108	2.957311	-0.862951
H	6.578960	1.333832	-0.531597
H	6.060340	-1.039060	-0.147939
H	2.600688	-2.437481	-0.104249
H	4.060717	-4.098487	-1.100214
H	3.975135	-2.674240	-2.147984
H	5.408536	-2.961622	-1.153103
H	3.927110	-3.851895	1.418062
H	3.689358	-2.262859	2.157795
H	5.239067	-2.669431	1.421545
H	1.137143	2.130690	-0.625822
H	1.416819	4.481495	-0.054794
H	2.226835	3.493720	1.167005
H	3.173608	4.331143	-0.066914
H	1.321025	3.737970	-2.467614
H	2.001613	2.181369	-2.962290
H	3.072767	3.515329	-2.529663
H	-2.471441	-2.876058	-2.743564
H	-0.750505	-3.107689	-2.415278
H	-1.932426	-3.265782	-1.106092
H	-3.978088	-2.271952	-0.992195
H	-3.726404	2.637865	-0.357562
H	-1.808730	1.423678	-1.264908
H	-6.274850	-2.279347	-0.142419
H	-8.210159	-1.061126	0.768642
H	-8.096634	1.394950	1.105385
H	-6.047147	2.632973	0.526267

ωB97X energy = -1315.45958735 a.u.

(5*S*,1'*S*)-**3a**, Conf. D

C	-0.273201	-1.734884	0.325382
C	0.928016	-1.028413	0.999801
N	1.519043	-0.286897	-0.159702
C	0.733440	-0.400384	-1.210000
N	-0.317058	-1.145012	-1.017473
C	0.557002	-0.180081	2.238626
C	0.003284	-1.155649	3.286812
C	-0.495842	0.885128	1.928344
C	1.808847	0.477365	2.827948
C	-1.448875	-1.313218	-1.944082
C	2.835697	0.302844	-0.218747
C	2.966271	1.691754	-0.365888
C	4.252153	2.223354	-0.355350
C	5.363944	1.413453	-0.206902
C	5.210422	0.041934	-0.112785
C	3.949321	-0.545092	-0.142192
C	3.846246	-2.062069	-0.139417
C	4.604871	-2.663933	-1.325513
C	4.339926	-2.661064	1.179457
C	1.792744	2.634388	-0.579811
C	1.742783	3.750454	0.465569
C	1.826963	3.220979	-1.994506
C	-1.661556	-2.787115	-2.247426
C	-2.652025	-0.577326	-1.374668
C	-3.705936	-1.223148	-0.794631
C	-4.791210	-0.501041	-0.229381
C	-4.775363	0.912000	-0.270965
C	-3.673677	1.563500	-0.886373
C	-2.648123	0.842873	-1.418614

C	-5.888539	-1.160324	0.380826
C	-6.917550	-0.442663	0.920523
C	-6.901913	0.970394	0.877569
C	-5.857665	1.631098	0.295887
H	-0.127585	-2.812043	0.244064
H	-1.215889	-1.547268	0.837291
H	1.656597	-1.769956	1.326901
H	0.948680	0.079329	-2.157474
H	0.734254	-1.931839	3.528710
H	-0.918323	-1.643122	2.961854
H	-0.222476	-0.610932	4.205458
H	-0.134814	1.617369	1.204655
H	-0.746283	1.426399	2.843092
H	-1.424479	0.456726	1.541065
H	2.588612	-0.263463	3.026860
H	2.228182	1.239397	2.171520
H	1.556480	0.957684	3.775734
H	-1.146330	-0.807487	-2.863588
H	4.384905	3.293672	-0.465393
H	6.354913	1.851852	-0.185793
H	6.089173	-0.588601	-0.036348
H	2.799808	-2.341022	-0.271639
H	4.453066	-3.745027	-1.354937
H	4.256771	-2.243640	-2.271235
H	5.678705	-2.480106	-1.246584
H	4.203287	-3.744494	1.178420
H	3.804960	-2.251366	2.040012
H	5.403590	-2.457107	1.324731
H	0.865876	2.067099	-0.489183
H	0.853946	4.365544	0.309399
H	1.703207	3.351984	1.481660
H	2.615466	4.403322	0.393763
H	0.949958	3.849399	-2.163558
H	1.837317	2.437491	-2.755956
H	2.717060	3.837586	-2.140916
H	-2.476071	-2.899694	-2.962765
H	-0.755977	-3.210022	-2.682829
H	-1.912294	-3.360319	-1.353693
H	-3.741742	-2.306417	-0.753270
H	-3.663103	2.647405	-0.931267
H	-1.818731	1.360760	-1.891639
H	-5.897109	-2.244934	0.411704
H	-7.752989	-0.953613	1.385021
H	-7.725711	1.526759	1.309575
H	-5.841537	2.715374	0.259829

ωB97X energy = -1315.45952303 a.u.

(5*S*,1'*S*)-**3a**, Conf. E

C	-0.212786	-1.553426	-1.172003
C	1.072261	-0.729712	-1.432577
N	1.413089	-0.272980	-0.049022
C	0.564769	-0.786917	0.818200
N	-0.349705	-1.547246	0.288631
C	2.178216	-1.490499	-2.199093
C	1.586862	-1.859860	-3.566860
C	2.629870	-2.761201	-1.479672
C	3.380571	-0.572651	-2.440275
C	-1.460504	-2.232079	0.956269
C	2.299963	0.815911	0.291994
C	3.471249	0.552536	1.015142
C	4.323590	1.621692	1.273784
C	4.023391	2.900466	0.839401
C	2.834493	3.142977	0.174499
C	1.936516	2.114353	-0.092947
C	0.600537	2.456209	-0.735079
C	-0.200448	3.417316	0.147851
C	0.775993	3.025850	-2.144287
C	3.824186	-0.817085	1.570720
C	5.205634	-1.291733	1.116549
C	3.730012	-0.813578	3.099739
C	-1.243803	-2.329032	2.459454
C	-2.779096	-1.557596	0.614733
C	-2.919923	-0.199370	0.670199
C	-4.162597	0.422285	0.393586
C	-5.275458	-0.385204	0.059147
C	-5.106373	-1.791982	0.008864
C	-3.894829	-2.360565	0.275174
C	-4.322658	1.831909	0.443072
C	-5.532665	2.402829	0.174666
C	-6.646045	1.595441	-0.159668
C	-6.520539	0.237624	-0.216482
H	-1.099498	-1.102876	-1.619778
H	-0.132724	-2.582813	-1.519028
H	0.832885	0.155552	-2.022821
H	0.626006	-0.575069	1.878197
H	1.226214	-0.972683	-4.094363
H	0.761058	-2.570262	-3.487986
H	2.359049	-2.325004	-4.182437
H	1.804288	-3.451580	-1.291190
H	3.112883	-2.540752	-0.527187
H	3.357015	-3.290209	-2.099282
H	3.081061	0.344563	-2.955361
H	3.881925	-0.290667	-1.514374
H	4.109938	-1.085950	-3.070695
H	-1.470751	-3.242511	0.539369
H	5.240326	1.448791	1.826238
H	4.708882	3.715685	1.040100
H	2.588930	4.155863	-0.124867
H	0.002171	1.547351	-0.812692
H	-1.180181	3.602504	-0.299151
H	-0.353097	3.001482	1.146476
H	0.305276	4.379635	0.254592
H	-0.198230	3.203627	-2.604270
H	1.338834	2.346652	-2.789651
H	1.312254	3.977445	-2.117390
H	3.092842	-1.538056	1.204794
H	5.392945	-2.301157	1.488961
H	5.290588	-1.311343	0.027782
H	5.995134	-0.643720	1.503628
H	3.935976	-1.811235	3.493225
H	2.737184	-0.511163	3.441515

H	4.456640	-0.122510	3.534122
H	-2.051575	-2.922803	2.886056
H	-0.297650	-2.820071	2.693531
H	-1.274426	-1.346397	2.934426
H	-2.078384	0.438103	0.929925
H	-5.956426	-2.413335	-0.252470
H	-3.777512	-3.438227	0.225503
H	-3.466006	2.447246	0.700677
H	-5.648061	3.479746	0.216761
H	-7.600726	2.063601	-0.369785
H	-7.371082	-0.385323	-0.472970

ωB97X energy = -1315.45919558 energy

(5*S*,1’*S*)-**3a**, Conf. F

C	-1.158175	2.545309	-0.767782
C	-2.219630	1.532784	-0.271014
N	-1.410488	0.275900	-0.217438
C	-0.141593	0.551773	-0.422920
N	0.109345	1.812267	-0.644299
C	-2.932389	1.945047	1.039397
C	-3.736020	3.213530	0.719727
C	-1.951178	2.233551	2.176460
C	-3.924879	0.862799	1.476434
C	1.428354	2.385406	-0.989501
C	-1.906774	-1.071575	-0.077423
C	-1.550920	-1.825283	1.051834
C	-2.095330	-3.100017	1.172996
C	-2.960589	-3.606549	0.219696
C	-3.258174	-2.861220	-0.906785
C	-2.717357	-1.593045	-1.094973
C	-2.989336	-0.869318	-2.404584
C	-2.525195	-1.709332	-3.597896
C	-4.465562	-0.493113	-2.550003
C	-0.581804	-1.345316	2.121823
C	-1.185648	-1.420025	3.525782
C	0.730308	-2.133690	2.056553
C	1.541508	3.805377	-0.448391
C	2.529592	1.483195	-0.477864
C	3.372697	0.858145	-1.350745
C	4.413486	0.009431	-0.888329
C	4.564513	-0.195497	0.502618
C	3.674396	0.463672	1.392119
C	2.692391	1.280765	0.919626
C	5.297734	-0.645355	-1.782299
C	6.282375	-1.464784	-1.308507
C	6.432652	-1.670613	0.081895
C	5.594633	-1.052096	0.965578
H	-1.307516	2.827066	-1.811746
H	-1.132329	3.448026	-0.163044
H	-2.992398	1.403522	-1.028391
H	0.627048	-0.210152	-0.415636
H	-4.456309	3.031551	-0.082492
H	-3.101351	4.051716	0.424294
H	-4.292804	3.522815	1.606267
H	-1.253360	3.037893	1.932260
H	-1.371609	1.350094	2.448617
H	-2.504337	2.544943	3.064978
H	-4.616472	0.605470	0.669287
H	-3.428949	-0.052533	1.799140
H	-4.517280	1.232977	2.315917
H	1.483356	2.419149	-2.081202
H	-1.839587	-3.706704	2.034250
H	-3.387956	-4.594596	0.345362
H	-3.906187	-3.280856	-1.668085
H	-2.398053	0.047055	-2.427556
H	-2.657163	-1.146149	-4.524207
H	-1.470103	-1.975096	-3.506432
H	-3.101457	-2.633052	-3.685108
H	-4.626338	0.057814	-3.479036
H	-4.812861	0.128914	-1.721175
H	-5.093088	-1.387411	-2.577565
H	-0.338663	-0.298980	1.934864
H	-0.488290	-0.998782	4.253039
H	-2.122960	-0.863160	3.592404
H	-1.386003	-2.452746	3.818976
H	1.435736	-1.754457	2.799521
H	1.203541	-2.057773	1.073983
H	0.558859	-3.193343	2.261200
H	2.532176	4.190981	-0.687954
H	0.805779	4.468228	-0.905022
H	1.414689	3.831499	0.635469
H	3.255037	1.002061	-2.420642
H	3.791018	0.308436	2.459485
H	2.022644	1.774234	1.616834
H	5.177814	-0.485391	-2.848622
H	6.954630	-1.962440	-1.997815
H	7.217822	-2.324698	0.443107
H	5.706761	-1.207032	2.033508

ωB97X energy = -1315.45893233 a.u.

(5*S*,1’*S*)-**3a**, Conf. G

C	0.129730	-2.244910	0.311852
C	1.148645	-1.273978	0.958728
N	1.438555	-0.350334	-0.184452
C	0.644287	-0.627664	-1.195589
N	-0.148987	-1.641647	-0.996804
C	0.653963	-0.614896	2.267988
C	0.464345	-1.751795	3.282690
C	-0.668859	0.132632	2.087604
C	1.717448	0.334815	2.828043
C	-1.324811	-1.976527	-1.819414
C	2.522592	0.599350	-0.271973
C	2.233658	1.970490	-0.346776
C	3.307997	2.854462	-0.366726
C	4.614028	2.400830	-0.316703
C	4.872070	1.042048	-0.293697
C	3.836694	0.112668	-0.296709
C	4.177985	-1.366810	-0.373936
C	5.010716	-1.671468	-1.622167
C	4.896215	-1.849316	0.888062

C	0.826365	2.538351	-0.448823
C	0.523174	3.540906	0.666814
C	0.602678	3.178810	-1.822230
C	-1.352066	-3.465805	-2.119722
C	-2.568081	-1.420418	-1.138487
C	-2.874361	-0.096917	-1.303426
C	-3.969917	0.499912	-0.634371
C	-4.768723	-0.298623	0.217847
C	-4.443929	-1.669868	0.366345
C	-3.377748	-2.215842	-0.290013
C	-4.285020	1.875590	-0.788879
C	-5.345969	2.421915	-0.127550
C	-6.148280	1.622251	0.721343
C	-5.867898	0.297471	0.889809
H	0.540100	-3.244808	0.172691
H	-0.794553	-2.321456	0.882911
H	2.068673	-1.808273	1.195705
H	0.656196	-0.063350	-2.120329
H	1.396928	-2.301447	3.436576
H	-0.305399	-2.464307	2.978438
H	0.159464	-1.332302	4.243234
H	-1.476342	-0.518630	1.741152
H	-0.577649	0.960874	1.383533
H	-0.981864	0.552618	3.045712
H	2.681302	-0.170055	2.939491
H	1.867823	1.212546	2.200195
H	1.408448	0.682622	3.816179
H	-1.182811	-1.433670	-2.755691
H	3.116962	3.920228	-0.422060
H	5.433989	3.109620	-0.317705
H	5.899089	0.694217	-0.294566
H	3.251603	-1.933450	-0.478090
H	5.176679	-2.747532	-1.706339
H	4.503926	-1.331665	-2.527614
H	5.988218	-1.186063	-1.577935
H	5.083889	-2.923471	0.829488
H	4.311272	-1.654391	1.790536
H	5.859251	-1.346062	1.003850
H	0.108419	1.722930	-0.353997
H	-0.514259	3.875144	0.594274
H	0.668670	3.102636	1.656446
H	1.162379	4.423416	0.592115
H	-0.428709	3.527144	-1.912134
H	0.797371	2.474274	-2.634294
H	1.261873	4.038659	-1.963687
H	-2.224292	-3.697941	-2.730915
H	-0.454949	-3.751892	-2.668995
H	-1.404816	-4.069678	-1.212668
H	-2.270049	0.528200	-1.956560
H	-5.058788	-2.286849	1.012972
H	-3.159428	-3.269286	-0.159549
H	-3.667341	2.484480	-1.441110
H	-5.580135	3.473151	-0.249265
H	-6.989294	2.070519	1.237645
H	-6.480388	-0.318816	1.539535

ωB97X energy = -1315.45857243 a.u.

(5*S*,1’*S*)-**3a**, Conf. H

C	-1.031726	2.353764	-1.190701
C	-2.122962	1.285410	-0.928281
N	-1.368222	0.283210	-0.110451
C	-0.108168	0.650564	-0.005485
N	0.172817	1.785195	-0.578685
C	-3.431612	1.849454	-0.330970
C	-3.976523	2.849186	-1.361570
C	-3.219050	2.562073	1.004835
C	-4.468695	0.734474	-0.162550
C	1.455191	2.511993	-0.509008
C	-1.804483	-1.058479	0.206034
C	-2.054111	-1.401870	1.541612
C	-2.530157	-2.683781	1.799834
C	-2.743092	-3.590148	0.776641
C	-2.436559	-3.244218	-0.527604
C	-1.938375	-1.983044	-0.839001
C	-1.520729	-1.692605	-2.272281
C	-0.434176	-2.669226	-2.731064
C	-2.715958	-1.719554	-3.227149
C	-1.785664	-0.470998	2.711585
C	-3.027028	-0.262453	3.581324
C	-0.616934	-0.990051	3.554654
C	1.444056	3.480315	0.663869
C	2.600205	1.517889	-0.536286
C	3.419235	1.299297	0.533334
C	4.469669	0.343154	0.470223
C	4.662378	-0.391990	-0.721619
C	3.799674	-0.145033	-1.823504
C	2.804316	0.778344	-1.733435
C	5.326161	0.102619	1.574070
C	6.324686	-0.825823	1.489277
C	6.517918	-1.559028	0.296743
C	5.707402	-1.347024	-0.782284
H	-0.857145	2.524628	-2.252478
H	-1.262577	3.307715	-0.716637
H	-2.390154	0.796977	-1.867339
H	0.636330	0.046752	0.499738
H	-4.132601	2.371075	-2.332429
H	-3.311350	3.703960	-1.504018
H	-4.938134	3.236634	-1.019842
H	-2.509535	3.389775	0.932909
H	-2.865558	1.879526	1.777435
H	-4.167975	2.980611	1.346587
H	-4.632335	0.200265	-1.102715
H	-4.178913	0.005444	0.594551
H	-5.422445	1.170315	0.142874
H	1.492943	3.085475	-1.438957
H	-2.735307	-2.977264	2.823163
H	-3.128438	-4.578506	0.999008
H	-2.567060	-3.975249	-1.317695
H	-1.074611	-0.697709	-2.315143
H	-0.090790	-2.401102	-3.732501
H	0.425418	-2.648400	-2.057405

H	-0.809834	-3.694173	-2.770722
H	-2.396273	-1.473392	-4.241825
H	-3.488555	-1.005688	-2.930961
H	-3.173074	-2.711737	-3.250785
H	-1.490582	0.501747	2.317282
H	-2.817560	0.473577	4.360645
H	-3.876606	0.096265	2.996017
H	-3.327022	-1.190273	4.073592
H	-0.392770	-0.287154	4.359826
H	0.286544	-1.120395	2.954319
H	-0.858687	-1.954997	4.007198
H	2.364367	4.064643	0.674215
H	0.606208	4.172986	0.574119
H	1.350552	2.953177	1.614975
H	3.291267	1.853147	1.456911
H	3.950905	-0.700658	-2.742889
H	2.154433	0.960614	-2.583889
H	5.174661	0.667308	2.488098
H	6.974915	-1.004334	2.337887
H	7.313998	-2.292643	0.243605
H	5.851847	-1.907038	-1.700233

ωB97X energy = -1315.45817849 a.u.

(5*S*,1'*S*)-**3a**, Conf. I

C	1.020358	-2.387296	-1.113774
C	2.075945	-1.261221	-0.987194
N	1.385950	-0.308895	-0.058749
C	0.142333	-0.702984	0.129002
N	-0.157396	-1.835127	-0.438456
C	3.487572	-1.749693	-0.598706
C	3.927996	-2.712563	-1.711030
C	3.521678	-2.478984	0.743973
C	4.469424	-0.573471	-0.577158
C	-1.433939	-2.562844	-0.306908
C	1.788800	1.057517	0.196210
C	2.233758	1.416072	1.475779
C	2.674456	2.722778	1.662768
C	2.660153	3.639958	0.627008
C	2.159062	3.277232	-0.610689
C	1.692796	1.987988	-0.847944
C	1.059522	1.674793	-2.195002
C	-0.182477	2.540791	-2.424827
C	2.058221	1.832037	-3.343288
C	2.208101	0.473480	2.666947
C	3.587903	0.312708	3.308581
C	1.185494	0.947555	3.704524
C	-1.400973	-3.471958	0.911987
C	-2.580191	-1.570227	-0.367002
C	-3.426721	-1.345627	0.679187
C	-4.477757	-0.392506	0.580994
C	-4.640334	0.333824	-0.620650
C	-3.748414	0.081066	-1.697867
C	-2.755068	-0.840447	-1.575273
C	-5.363802	-0.145891	1.659767
C	-6.361284	0.780290	1.542024
C	-6.523846	1.505027	0.339892
C	-5.684385	1.286974	-0.715607
H	0.778521	-2.618647	-2.150403
H	1.327613	-3.305156	-0.610812
H	2.177135	-0.749098	-1.947317
H	-0.576420	-0.117601	0.690447
H	3.909980	-2.223094	-2.688609
H	3.297632	-3.603545	-1.761832
H	4.950398	-3.044616	-1.521363
H	2.895013	-3.373472	0.751848
H	3.204982	-1.836007	1.564791
H	4.544152	-2.800747	0.953016
H	4.449631	-0.024959	-1.523170
H	4.257672	0.132742	0.225820
H	5.484094	-0.950175	-0.431349
H	-1.486713	-3.181884	-1.206349
H	3.031060	3.028555	2.640017
H	3.019281	4.649282	0.791752
H	2.111464	4.015502	-1.403313
H	0.712330	0.640381	-2.194487
H	-0.661303	2.263608	-3.366641
H	-0.910360	2.408359	-1.620724
H	0.074382	3.601267	-2.479266
H	1.592159	1.551594	-4.290101
H	2.942517	1.205980	-3.199502
H	2.395031	2.867772	-3.430058
H	1.882073	-0.507932	2.320739
H	3.544785	-0.432581	4.105879
H	4.338668	-0.010477	2.584326
H	3.930740	1.251258	3.750186
H	1.129071	0.233448	4.529023
H	0.188285	1.046637	3.269932
H	1.468672	1.918130	4.119327
H	-2.314030	-4.065685	0.960845
H	-0.555788	-4.158446	0.847410
H	-1.305027	-2.897334	1.835090
H	-3.319526	-1.890323	1.610797
H	-3.875928	0.630171	-2.624820
H	-2.082930	-1.027195	-2.407510
H	-5.235945	-0.703867	2.581495
H	-7.034505	0.963348	2.371539
H	-7.319433	2.236855	0.260667
H	-5.805579	1.839820	-1.641247

ωB97X energy = -1315.45814150 a.u.

(5*S*,1'*S*)-**3a**, Conf. J

C	0.352468	-2.347773	0.269978
C	1.451116	-1.412754	0.840166
N	1.437652	-0.295293	-0.155339
C	0.524265	-0.524977	-1.073017
N	-0.139880	-1.634126	-0.914314
C	1.247787	-1.034070	2.325668
C	1.316309	-2.348892	3.116053
C	-0.096026	-0.352803	2.586660
C	2.387048	-0.133568	2.813694

C	-1.355893	-1.992907	-1.670990
C	2.420417	0.755838	-0.282710
C	2.036751	2.084816	-0.049862
C	3.023470	3.062899	-0.125375
C	4.335881	2.737987	-0.419683
C	4.677546	1.425714	-0.694023
C	3.725869	0.411394	-0.657775
C	4.134532	-0.993535	-1.071496
C	4.692087	-1.001751	-2.497595
C	5.140398	-1.603225	-0.092810
C	0.605814	2.509289	0.237315
C	0.488850	3.304143	1.539477
C	0.031742	3.309149	-0.936106
C	-1.410973	-3.494399	-1.900074
C	-2.580231	-1.408473	-0.988688
C	-3.108170	-0.236317	-1.454727
C	-4.216815	0.380333	-0.821776
C	-4.786352	-0.236228	0.316217
C	-4.232202	-1.457943	0.777658
C	-3.166714	-2.029209	0.145310
C	-4.766938	1.599352	-1.295133
C	-5.831962	2.171726	-0.661541
C	-6.402374	1.554900	0.476661
C	-5.893255	0.381205	0.953515
H	0.745918	-3.321016	-0.020562
H	-0.465171	-2.496801	0.974378
H	2.422959	-1.902423	0.767626
H	0.347684	0.153210	-1.899385
H	2.270155	-2.858174	2.953455
H	0.511754	-3.038709	2.851467
H	1.226913	-2.137369	4.183173
H	-0.947496	-0.963633	2.275795
H	-0.169191	0.610384	2.081735
H	-0.206265	-0.169221	3.657414
H	3.362591	-0.591532	2.628135
H	2.377929	0.846961	2.337344
H	2.291128	0.019080	3.890831
H	-1.244226	-1.498671	-2.637728
H	2.758979	4.099258	0.052207
H	5.091385	3.514184	-0.456693
H	5.700580	1.186493	-0.962269
H	3.246372	-1.627225	-1.086578
H	4.904852	-2.027067	-2.807550
H	3.978728	-0.572046	-3.203779
H	5.622323	-0.433456	-2.565601
H	5.385480	-2.625250	-0.389218
H	4.750578	-1.630039	0.927768
H	6.068067	-1.025862	-0.078398
H	-0.007318	1.614156	0.344885
H	-0.561223	3.514184	1.754300
H	0.905528	2.756615	2.387788
H	1.010602	4.261167	1.469043
H	-1.016718	3.551442	-0.748300
H	0.089458	2.751133	-1.873834
H	0.575230	4.247203	-1.073769
H	-2.286381	-3.729498	-2.504976
H	-0.520053	-3.832170	-2.430465
H	-1.489731	-4.051287	-0.965336
H	-2.681549	0.245646	-2.330186
H	-4.673748	-1.938639	1.644158
H	-2.776972	-2.971846	0.513403
H	-4.326405	2.068753	-2.168536
H	-6.247517	3.103090	-1.028499
H	-7.248415	2.020873	0.968507
H	-6.327889	-0.095483	1.825877

ωB97X energy = -1315.45806815 a.u.

(5*S*,1’*S*)-**3a**, Conf. K

C	1.228727	2.660913	0.318197
C	2.206044	1.542856	-0.120147
N	1.379777	0.325101	0.145357
C	0.147675	0.675921	0.443632
N	-0.055146	1.962886	0.474274
C	2.751686	1.699859	-1.560257
C	1.638406	1.829293	-2.601182
C	3.663501	0.523075	-1.922475
C	3.609514	2.972998	-1.566249
C	-1.316677	2.656422	0.813385
C	1.829010	-1.045372	0.173574
C	2.758923	-1.424905	1.150970
C	3.244064	-2.728540	1.112025
C	2.783467	-3.636826	0.176015
C	1.808445	-3.258793	-0.729681
C	1.309858	-1.960236	-0.756006
C	0.212342	-1.624998	-1.754760
C	0.620263	-1.942822	-3.194632
C	-1.089178	-2.346752	-1.390183
C	3.220799	-0.504055	2.269434
C	2.915460	-1.111679	3.641323
C	4.708442	-0.166246	2.147539
C	-1.566012	3.795647	-0.167501
C	-2.456997	1.660318	0.877037
C	-2.985602	1.110047	-0.260092
C	-4.023766	0.148880	-0.195470
C	-4.525804	-0.238143	1.070014
C	-3.971056	0.350920	2.233264
C	-2.964654	1.268495	2.139631
C	-4.568415	-0.443617	-1.365458
C	-5.562863	-1.372761	-1.272927
C	-6.067192	-1.757710	-0.007308
C	-5.563564	-1.204770	1.133739
H	1.506717	3.110782	1.272967
H	1.142824	3.448454	-0.425776
H	3.066700	1.519173	0.547551
H	-0.626768	-0.049794	0.652925
H	1.009015	0.938751	-2.639354
H	2.080261	1.957752	-3.591468
H	0.995858	2.694301	-2.420256
H	3.118388	-0.416203	-2.014485
H	4.451259	0.385358	-1.176445
H	4.146219	0.721226	-2.881870

H	3.029935	3.873176	-1.350798
H	4.418357	2.905092	-0.833782
H	4.059861	3.103320	-2.552050
H	-1.172357	3.078544	1.812011
H	3.979641	-3.044186	1.843303
H	3.170658	-4.649107	0.165685
H	1.428157	-3.987784	-1.436247
H	0.009253	-0.554629	-1.711217
H	-0.164500	-1.621781	-3.882958
H	1.544840	-1.432326	-3.473491
H	0.770317	-3.014766	-3.339909
H	-1.888512	-2.053224	-2.074927
H	-1.414072	-2.113517	-0.372499
H	-0.965504	-3.430324	-1.458387
H	2.652016	0.425007	2.213309
H	3.186068	-0.404767	4.428562
H	1.853865	-1.346077	3.742530
H	3.483329	-2.029588	3.808658
H	5.005604	0.528834	2.935573
H	4.945632	0.291605	1.184005
H	5.319756	-1.066734	2.245239
H	-2.516990	4.268912	0.075614
H	-0.788127	4.556560	-0.101247
H	-1.612600	3.432016	-1.195451
H	-2.607934	1.390231	-1.239439
H	-4.356256	0.058612	3.204307
H	-2.544401	1.707392	3.038383
H	-4.179240	-0.144825	-2.333355
H	-5.973715	-1.821727	-2.169769
H	-6.857682	-2.497086	0.048985
H	-5.947382	-1.497089	2.105469

ωB97X energy = -1315.45795987 a.u.

(5*S*,1’*S*)-**3a**, Conf. L

C	1.123973	-2.677942	0.056205
C	2.068254	-1.535820	0.503925
N	1.405158	-0.355480	-0.133651
C	0.229775	-0.707100	-0.604249
N	-0.061654	-1.968882	-0.445498
C	2.287067	-1.450109	2.033737
C	0.973243	-1.357839	2.811840
C	3.179563	-0.255478	2.385963
C	3.036132	-2.726473	2.441508
C	-1.284581	-2.631201	-0.948243
C	1.952413	0.968731	-0.301420
C	1.330088	2.059257	0.326697
C	1.919741	3.310472	0.176169
C	3.079892	3.476200	-0.559200
C	3.647476	2.392626	-1.204706
C	3.082902	1.124138	-1.114899
C	3.685020	-0.004691	-1.936605
C	3.702934	0.352498	-3.425525
C	5.089118	-0.373487	-1.452680
C	0.036696	1.959083	1.122454
C	0.176459	2.533639	2.533596
C	-1.113728	2.640396	0.374021
C	-1.595799	-3.869977	-0.118456
C	-2.437699	-1.645833	-0.956107
C	-2.839135	-1.021724	0.195010
C	-3.904900	-0.090206	0.190602
C	-4.570218	0.186850	-1.027879
C	-4.148633	-0.482144	-2.203224
C	-3.110286	-1.368651	-2.169479
C	-4.316118	0.584457	1.370982
C	-5.338174	1.487092	1.333170
C	-6.004484	1.763569	0.114720
C	-5.631743	1.129947	-1.034401
H	1.550435	-3.277772	-0.750113
H	0.853892	-3.337796	0.876213
H	3.048227	-1.662334	0.044908
H	-0.435486	-0.003107	-1.086828
H	0.323559	-2.219251	2.640248
H	0.414319	-0.453857	2.564122
H	1.185947	-1.323028	3.882374
H	2.688830	0.700764	2.206112
H	4.111536	-0.270270	1.813894
H	3.439749	-0.300018	3.445689
H	2.460775	-3.634291	2.248361
H	3.989877	-2.808587	1.913451
H	3.247196	-2.696032	3.512079
H	-1.080652	-2.941755	-1.976897
H	1.462332	4.172510	0.648253
H	3.531026	4.457700	-0.647318
H	4.535071	2.537688	-1.810292
H	3.046783	-0.884008	-1.841103
H	4.061439	-0.499156	-4.007619
H	2.704561	0.614292	-3.781656
H	4.367491	1.195763	-3.626325
H	5.479451	-1.215645	-2.027861
H	5.097832	-0.650875	-0.395617
H	5.776457	0.466711	-1.578885
H	-0.229522	0.907585	1.234564
H	-0.749652	2.376003	3.090717
H	0.989710	2.056471	3.084946
H	0.369813	3.608152	2.509385
H	-2.051119	2.510701	0.920984
H	-1.249832	2.225666	-0.628423
H	-0.927512	3.711687	0.265849
H	-2.509664	-4.321748	-0.503506
H	-0.800359	-4.612921	-0.188354
H	-1.756749	-3.614282	0.930793
H	-2.333191	-1.221043	1.136640
H	-4.657779	-0.272749	-3.137955
H	-2.790138	-1.865322	-3.079172
H	-3.802401	0.370431	2.302705
H	-5.645749	1.999102	2.237624
H	-6.813736	2.484458	0.101366
H	-6.138782	1.339033	-1.970466

ωB97X energy = -1315.45788268 a.u.

(5*S*,1’*S*)-**3a**, Conf. M

C	-0.020167	-1.774431	1.112592
C	1.220835	-0.868041	1.324778
N	1.420871	-0.307776	-0.049320
C	0.528761	-0.812420	-0.873134
N	-0.327266	-1.622405	-0.316667
C	1.085780	0.141106	2.488319
C	0.935365	-0.695944	3.766699
C	-0.124339	1.064401	2.342097
C	2.359745	0.982092	2.626455
C	-1.446091	-2.275303	-1.003688
C	2.576501	0.430468	-0.506403
C	2.427209	1.771189	-0.888892
C	3.574844	2.469390	-1.251836
C	4.818026	1.862662	-1.240896
C	4.929695	0.523251	-0.913441
C	3.812104	-0.228346	-0.564021
C	3.981854	-1.720192	-0.321383
C	4.568688	-2.406243	-1.558493
C	4.842547	-2.003566	0.911606
C	1.085234	2.479992	-0.975408
C	1.047576	3.755049	-0.130762
C	0.737174	2.789054	-2.434891
C	-1.515139	-3.740374	-0.581487
C	-2.757694	-1.529296	-0.797966
C	-2.833756	-0.324064	-0.160946
C	-4.069791	0.356774	-0.002248
C	-5.247100	-0.222219	-0.525847
C	-5.145531	-1.466067	-1.201856
C	-3.945937	-2.097842	-1.332467
C	-4.154333	1.603628	0.668084
C	-5.356780	2.235724	0.808781
C	-6.535121	1.654926	0.286152
C	-6.481587	0.456082	-0.366011
H	0.192746	-2.818364	1.335781
H	-0.877753	-1.460934	1.707924
H	2.090326	-1.488316	1.547897
H	0.513059	-0.580071	-1.931448
H	1.804070	-1.341775	3.920562
H	0.041166	-1.323353	3.751779
H	0.854926	-0.031285	4.628861
H	-0.102368	1.622442	1.404325
H	-0.123511	1.794257	3.154090
H	-1.072278	0.524442	2.402329
H	3.251946	0.351542	2.668517
H	2.485704	1.690910	1.807964
H	2.313600	1.553912	3.555657
H	-1.197934	-2.235899	-2.067783
H	3.492114	3.508731	-1.549013
H	5.700783	2.429823	-1.512646
H	5.901767	0.044445	-0.950449
H	2.999027	-2.166308	-0.160823
H	4.610167	-3.486066	-1.400936
H	3.961173	-2.212053	-2.444763
H	5.584689	-2.059890	-1.760868
H	4.926679	-3.079747	1.075796
H	4.423457	-1.553934	1.814965
H	5.850991	-1.603784	0.780801
H	0.311592	1.811582	-0.595328
H	0.050388	4.198893	-0.169998
H	1.288666	3.556324	0.915781
H	1.756009	4.498200	-0.503395
H	-0.252997	3.245285	-2.498139
H	0.737419	1.886018	-3.049895
H	1.457892	3.487291	-2.867484
H	-2.229309	-4.275986	-1.203849
H	-0.541389	-4.215491	-0.706262
H	-1.833828	-3.840957	0.457538
H	-1.947098	0.155099	0.239022
H	-6.042873	-1.910541	-1.619300
H	-3.899210	-3.041767	-1.862879
H	-3.248015	2.047410	1.068460
H	-5.413721	3.188418	1.322555
H	-7.482440	2.167688	0.406195
H	-7.382301	0.006428	-0.770559

ωB97X energy = -1315.45711093 a.u.

(5*S*,1’*S*)-**3a**, Conf. N

C	1.103908	-2.313988	-1.277072
C	2.028777	-1.079219	-1.162237
N	1.351162	-0.303467	-0.075578
C	0.170153	-0.840097	0.169224
N	-0.068752	-1.941200	-0.479943
C	3.524230	-1.415155	-0.986352
C	3.924496	-2.242753	-2.216406
C	3.815803	-2.220353	0.279371
C	4.358546	-0.129865	-0.987664
C	-1.294419	-2.763621	-0.425414
C	1.603851	1.088927	0.230188
C	2.175173	1.430122	1.462551
C	2.461092	2.772487	1.692118
C	2.176550	3.738270	0.742925
C	1.554313	3.382797	-0.440853
C	1.234689	2.056519	-0.714953
C	0.453842	1.738582	-1.982053
C	-0.924315	2.406784	-1.948388
C	1.227200	2.132812	-3.241731
C	2.430645	0.421168	2.568402
C	3.885916	0.429037	3.039438
C	1.480491	0.670295	3.743817
C	-1.044768	-4.033169	0.375269
C	-2.461537	-1.909615	0.040852
C	-2.967515	-0.965010	-0.812991
C	-4.020996	-0.104983	-0.423320
C	-4.568598	-0.237953	0.874886
C	-4.039275	-1.227339	1.738828
C	-3.014174	-2.037034	1.337888
C	-4.538062	0.891050	-1.293883
C	-5.551320	1.708238	-0.886364
C	-6.103557	1.571515	0.410474
C	-5.626497	0.623793	1.268029

H	0.804955	-2.521712	-2.303785
H	1.558398	-3.210178	-0.852706
H	1.949765	-0.480003	-2.073493
H	-0.549309	-0.377391	0.833203
H	3.712122	-1.704075	-3.143930
H	3.408317	-3.205013	-2.252854
H	4.996479	-2.446398	-2.184899
H	3.289742	-3.177107	0.297806
H	3.551557	-1.671068	1.182693
H	4.884617	-2.439260	0.328103
H	4.156335	0.471918	-1.878449
H	4.168294	0.490311	-0.111373
H	5.420623	-0.384540	-0.991058
H	-1.481955	-3.039208	-1.466499
H	2.908846	3.067128	2.634572
H	2.416037	4.777299	0.937666
H	1.293121	4.152686	-1.158299
H	0.270351	0.664273	-2.031931
H	-1.493678	2.133700	-2.840136
H	-1.496500	2.092894	-1.071225
H	-0.837598	3.495551	-1.925473
H	0.666466	1.846960	-4.134033
H	2.204690	1.645621	-3.282993
H	1.391157	3.212149	-3.281744
H	2.207397	-0.572844	2.180054
H	4.042553	-0.356473	3.782123
H	4.581470	0.258133	2.214758
H	4.146314	1.381895	3.506044
H	1.622906	-0.092593	4.512415
H	0.435713	0.643973	3.425999
H	1.667754	1.646125	4.199133
H	-1.947187	-4.644983	0.386674
H	-0.248044	-4.621350	-0.081101
H	-0.758779	-3.808096	1.403670
H	-2.551640	-0.855472	-1.811709
H	-4.460145	-1.334290	2.732909
H	-2.628431	-2.783322	2.021598
H	-4.111245	0.991717	-2.286512
H	-5.940032	2.467795	-1.554748
H	-6.909215	2.228021	0.718296
H	-6.046939	0.516453	2.262395

ωB97X energy = -1315.45709326 a.u.

(5*S*,1’*R*)-**3b**, Conf. A

C	-0.337252	-1.610314	0.362330
C	0.966810	-1.074266	1.003118
N	1.545212	-0.284541	-0.128871
C	0.708861	-0.274878	-1.145791
N	-0.387586	-0.948432	-0.946196
C	0.757340	-0.319816	2.336795
C	-0.216955	0.851423	2.204593
C	2.096529	0.180140	2.887777
C	0.194926	-1.345155	3.331374
C	-1.552427	-1.080200	-1.832164
C	2.896530	0.216847	-0.222464
C	3.949549	-0.706844	-0.279709
C	5.248616	-0.208417	-0.282263
C	5.493826	1.152833	-0.281902
C	4.436009	2.043962	-0.301401
C	3.117092	1.601262	-0.271567
C	2.002713	2.633642	-0.336178
C	2.106073	3.667196	0.787295
C	1.987081	3.322826	-1.704015
C	3.743874	-2.209643	-0.389453
C	4.401820	-2.758401	-1.658561
C	4.259699	-2.944626	0.849738
C	-1.273884	-0.515733	-3.216784
C	-2.765794	-0.445137	-1.180069
C	-3.881651	-1.187981	-0.922754
C	-5.037192	-0.602397	-0.340887
C	-5.019128	0.774167	-0.017073
C	-3.844213	1.524948	-0.287172
C	-2.753441	0.935814	-0.851765
C	-6.205994	-1.358480	-0.070628
C	-7.301401	-0.767071	0.491138
C	-7.283913	0.609576	0.812817
C	-6.170668	1.361023	0.565416
H	-0.320862	-2.690908	0.215672
H	-1.226941	-1.350958	0.934447
H	1.645312	-1.902224	1.209604
H	0.929833	0.243867	-2.069661
H	-1.209602	0.537601	1.871562
H	0.148182	1.608651	1.509311
H	-0.340355	1.332965	3.176935
H	2.533568	0.966146	2.272254
H	2.824058	-0.632883	2.964168
H	1.946454	0.588193	3.889660
H	-0.778206	-1.735862	3.026229
H	0.878743	-2.189387	3.454617
H	0.066089	-0.872668	4.306916
H	-1.732666	-2.154639	-1.920435
H	6.082920	-0.900409	-0.307351
H	6.513173	1.521203	-0.288906
H	4.637336	3.108534	-0.339207
H	1.045333	2.124576	-0.220815
H	1.255429	4.350877	0.744748
H	2.111546	3.194839	1.772041
H	3.016153	4.264006	0.695645
H	1.155982	4.028918	-1.761361
H	1.880347	2.602225	-2.518553
H	2.913378	3.877824	-1.871369
H	2.676233	-2.411674	-0.487514
H	4.183283	-3.823399	-1.761435
H	4.031520	-2.245767	-2.548656
H	5.487500	-2.642646	-1.627253
H	4.062281	-4.015238	0.765048
H	3.786563	-2.582168	1.765757
H	5.338393	-2.809681	0.960326
H	-2.144998	-0.702379	-3.843433
H	-0.412662	-1.000716	-3.679069

H	-1.109353	0.563648	-3.192340
H	-3.900749	-2.247021	-1.163819
H	-3.828793	2.580891	-0.038840
H	-1.867573	1.531360	-1.050765
H	-6.215936	-2.414784	-0.318378
H	-8.191707	-1.351291	0.693352
H	-8.160947	1.065459	1.257510
H	-6.152244	2.417934	0.810095

ωB97X energy = -1315.46047103 a.u.

(5*S*,1’*R*)-**3b**, Conf. B

C	-0.215967	-1.735518	0.779413
C	1.002086	-0.896806	1.238985
N	1.464297	-0.314920	-0.059711
C	0.645274	-0.666606	-1.029157
N	-0.343501	-1.423779	-0.648857
C	0.695536	0.103729	2.377722
C	-0.421770	1.084614	2.020140
C	1.959080	0.878046	2.766226
C	0.268526	-0.736877	3.589413
C	-1.449672	-1.951844	-1.454346
C	2.738317	0.320144	-0.306478
C	2.775438	1.674962	-0.667125
C	4.024720	2.262996	-0.838904
C	5.190144	1.538864	-0.661076
C	5.125389	0.191655	-0.353494
C	3.902524	-0.452782	-0.194955
C	3.891838	-1.955287	0.038547
C	4.600520	-2.690393	-1.102523
C	4.510998	-2.324887	1.388333
C	1.532488	2.511343	-0.923395
C	1.507457	3.788345	-0.081471
C	1.405875	2.843609	-2.413349
C	-1.253878	-1.672518	-2.936988
C	-2.776362	-1.416560	-0.943207
C	-2.966076	-0.081838	-0.718053
C	-4.213260	0.415946	-0.265762
C	-5.279069	-0.491148	-0.054940
C	-5.060810	-1.869841	-0.301552
C	-3.845491	-2.319499	-0.729321
C	-4.423480	1.797472	-0.015866
C	-5.635582	2.247418	0.420741
C	-6.701477	1.340431	0.631348
C	-6.527799	0.006833	0.400134
H	-0.057675	-2.807828	0.899403
H	-1.136303	-1.459741	1.292310
H	1.788606	-1.558869	1.602354
H	0.799570	-0.356942	-2.054593
H	-1.360737	0.581262	1.774925
H	-0.149772	1.724679	1.179540
H	-0.619813	1.736325	2.873742
H	2.290182	1.557791	1.981278
H	2.785513	0.200259	2.998137
H	1.758429	1.474281	3.658971
H	-0.656785	-1.288902	3.410642
H	1.046066	-1.453589	3.866978
H	0.096530	-0.080260	4.444319
H	-1.433626	-3.033687	-1.296188
H	4.085944	3.309398	-1.115657
H	6.152662	2.021480	-0.784887
H	6.043642	-0.376581	-0.255280
H	2.858217	-2.303937	0.030581
H	4.516875	-3.769569	-0.957521
H	4.157731	-2.440134	-2.068702
H	5.663171	-2.440518	-1.140567
H	4.442434	-3.402065	1.553970
H	4.010910	-1.820787	2.219209
H	5.567222	-2.046904	1.421095
H	0.655737	1.924593	-0.648240
H	0.563260	4.315663	-0.233757
H	1.606710	3.572599	0.984666
H	2.315139	4.467037	-0.364120
H	0.482964	3.397106	-2.599171
H	1.391652	1.941127	-3.029589
H	2.243355	3.461077	-2.747405
H	-2.077878	-2.130390	-3.483007
H	-0.321401	-2.101702	-3.307454
H	-1.272567	-0.600940	-3.148070
H	-2.158400	0.627722	-0.878908
H	-5.874901	-2.567842	-0.137319
H	-3.689176	-3.378749	-0.905402
H	-3.603906	2.489917	-0.178944
H	-5.788675	3.303871	0.608469
H	-7.658020	1.713224	0.979084
H	-7.341552	-0.692610	0.560840

ωB97X energy = -1315.45980851 a.u.

(5*S*,1’*R*)-**3b**, Conf. C

C	0.406249	-1.255728	0.850875
C	-0.959158	-0.656228	1.273737
N	-1.520781	-0.228562	-0.046826
C	-0.671020	-0.521948	-1.009547
N	0.413425	-1.119436	-0.609514
C	-1.834209	-1.603454	2.124975
C	-1.032748	-1.918547	3.396101
C	-2.170165	-2.905249	1.397883
C	-3.123067	-0.891913	2.547828
C	1.540557	-1.533031	-1.459034
C	-2.606363	0.706862	-0.239107
C	-2.410736	2.038388	0.153470
C	-3.488698	2.910764	0.040783
C	-4.697240	2.489711	-0.485268
C	-4.839368	1.189718	-0.936860
C	-3.799864	0.270595	-0.830200
C	-3.992272	-1.123539	-1.403380
C	-5.188133	-1.846477	-0.780669
C	-4.131999	-1.061578	-2.927467
C	-1.073801	2.584732	0.631535
C	-0.556591	3.658422	-0.330289
C	-1.153902	3.120827	2.061913

C	1.718802	-3.041720	-1.399800
C	2.760986	-0.706479	-1.082896
C	3.912679	-1.261517	-0.605909
C	5.030678	-0.454364	-0.260021
C	4.942569	0.948121	-0.410917
C	3.734952	1.504746	-0.910571
C	2.682754	0.704774	-1.235838
C	6.232039	-1.018878	0.238059
C	7.290979	-0.221087	0.567302
C	7.202598	1.181219	0.414574
C	6.057062	1.751684	-0.063100
H	1.253791	-0.713916	1.271989
H	0.498660	-2.307024	1.118772
H	-0.800449	0.245436	1.867720
H	-0.855253	-0.269133	-2.047118
H	-0.756693	-1.003192	3.926561
H	-0.119915	-2.480933	3.187415
H	-1.643139	-2.524603	4.068156
H	-1.277296	-3.455536	1.091222
H	-2.780341	-2.729224	0.511792
H	-2.739484	-3.557502	2.063327
H	-2.903450	0.041437	3.073986
H	-3.767291	-0.656335	1.700701
H	-3.688517	-1.533085	3.227600
H	1.247538	-1.260166	-2.475735
H	-3.372308	3.943655	0.349492
H	-5.524204	3.185670	-0.566514
H	-5.776912	0.881255	-1.385684
H	-3.100121	-1.711937	-1.187205
H	-5.261259	-2.859953	-1.181319
H	-5.098393	-1.918125	0.305416
H	-6.124768	-1.332060	-1.007392
H	-4.220369	-2.069438	-3.338748
H	-3.268040	-0.579210	-3.390462
H	-5.024304	-0.501184	-3.217184
H	-0.334134	1.783012	0.615324
H	0.432018	3.998440	-0.013545
H	-0.474215	3.268117	-1.347322
H	-1.219090	4.526789	-0.352172
H	-0.171907	3.466552	2.391426
H	-1.498743	2.356250	2.762683
H	-1.844586	3.965032	2.125724
H	2.499729	-3.349326	-2.095117
H	0.789308	-3.533140	-1.688004
H	1.996594	-3.384627	-0.401822
H	4.005178	-2.334519	-0.480562
H	3.664066	2.580130	-1.035466
H	1.771214	1.152221	-1.621227
H	6.296975	-2.095721	0.354283
H	8.205481	-0.660819	0.948215
H	8.050706	1.802203	0.679171
H	5.984007	2.827525	-0.182981

ωB97X energy = -1315.45922472 a.u.

(5*S*,1'*R*)-**3b**, Conf. D

C	1.193740	-2.680600	-0.014493
C	2.195602	-1.543114	0.312000
N	1.410444	-0.339782	-0.109196
C	0.182201	-0.692004	-0.419276
N	-0.050625	-1.969609	-0.326285
C	2.716960	-1.550457	1.769277
C	3.518406	-2.849584	1.935364
C	1.590699	-1.510139	2.802948
C	3.674711	-0.378247	2.006464
C	-1.337790	-2.645604	-0.579525
C	1.895020	1.009192	-0.270115
C	1.372891	2.028151	0.542103
C	1.898028	3.307247	0.387079
C	2.900192	3.566434	-0.531075
C	3.363742	2.553768	-1.351282
C	2.853629	1.262403	-1.260252
C	3.320794	0.219017	-2.263258
C	3.052039	0.682260	-3.697765
C	4.799336	-0.127195	-2.074343
C	0.247546	1.818528	1.544010
C	0.621167	2.304392	2.945722
C	-1.041607	2.491703	1.062023
C	-1.364631	-3.219363	-1.987588
C	-2.467332	-1.695512	-0.229046
C	-3.293861	-1.147644	-1.166369
C	-4.309596	-0.222986	-0.798695
C	-4.457967	0.134717	0.560834
C	-3.590482	-0.453561	1.520449
C	-2.630866	-1.339650	1.138396
C	-5.171454	0.358909	-1.762157
C	-6.131922	1.253846	-1.384389
C	-6.280009	1.610781	-0.025132
C	-5.463590	1.064527	0.924339
H	1.506207	-3.268860	-0.878252
H	1.027875	-3.354413	0.824485
H	3.066184	-1.618534	-0.338933
H	-0.570334	0.023340	-0.726245
H	4.331951	-2.906218	1.207007
H	2.900039	-3.742935	1.825236
H	3.959538	-2.879313	2.933386
H	0.894923	-2.346607	2.700935
H	1.019485	-0.582553	2.747500
H	2.018374	-1.568320	3.806033
H	4.474873	-0.360614	1.261393
H	3.168430	0.586898	1.981570
H	4.138788	-0.484460	2.989301
H	-1.362558	-3.470333	0.138332
H	1.516420	4.115836	1.000154
H	3.306822	4.566951	-0.622511
H	4.121229	2.775075	-2.094703
H	2.735356	-0.690612	-2.121904
H	3.323590	-0.107860	-4.401033
H	1.997049	0.922492	-3.844173
H	3.639973	1.567791	-3.949448
H	5.100855	-0.901621	-2.782709
H	5.007620	-0.490545	-1.065026

H	5.428869	0.749183	-2.247300
H	0.040189	0.750905	1.622945
H	-0.176639	2.057408	3.649526
H	1.542759	1.837707	3.301195
H	0.760890	3.387194	2.970337
H	-1.853640	2.298220	1.767030
H	-1.352819	2.121981	0.081057
H	-0.910725	3.573593	0.981503
H	-2.296452	-3.760978	-2.151348
H	-0.539934	-3.919171	-2.126621
H	-1.276749	-2.432410	-2.738373
H	-3.192901	-1.402374	-2.215647
H	-3.709607	-0.190897	2.566327
H	-1.976605	-1.784877	1.882295
H	-5.053834	0.083730	-2.805075
H	-6.786937	1.694517	-2.127052
H	-7.046305	2.322187	0.260227
H	-5.574552	1.333916	1.969467

ωB97X energy = -1315.45893267 a.u.

(5*S*,1’*R*)-**3b**, Conf. E

C	1.218027	-2.662124	0.425576
C	2.080692	-1.419801	0.760782
N	1.383709	-0.367835	-0.046529
C	0.241184	-0.838950	-0.497259
N	0.025551	-2.090518	-0.210360
C	2.221035	-1.128684	2.274331
C	3.007879	-2.301187	2.876479
C	0.866337	-1.008971	2.974908
C	3.036409	0.147573	2.506102
C	-1.217700	-2.852455	-0.448677
C	1.852834	0.961362	-0.351379
C	3.014573	1.099732	-1.122430
C	3.499362	2.385778	-1.340223
C	2.826180	3.495214	-0.862251
C	1.638505	3.335757	-0.170406
C	1.124294	2.072648	0.104982
C	-0.203157	1.976754	0.844432
C	-0.181210	2.726541	2.177788
C	-1.354862	2.474686	-0.035881
C	3.735754	-0.072762	-1.768555
C	3.823188	0.107434	-3.286632
C	5.127942	-0.281748	-1.168538
C	-1.008759	-3.878693	-1.551903
C	-2.358392	-1.875110	-0.662775
C	-2.930061	-1.283019	0.431521
C	-3.918974	-0.279308	0.296273
C	-4.326603	0.109139	-1.001862
C	-3.740797	-0.533313	-2.120856
C	-2.784299	-1.494855	-1.959230
C	-4.493115	0.365006	1.423487
C	-5.423346	1.349595	1.258860
C	-5.831747	1.738112	-0.039678
C	-5.300276	1.132320	-1.140966
H	1.717553	-3.333456	-0.274091
H	0.927526	-3.230407	1.307617
H	3.084960	-1.547429	0.358634
H	-0.456061	-0.232952	-1.060761
H	3.985645	-2.405480	2.398523
H	2.481404	-3.253549	2.785397
H	3.172765	-2.119769	3.940184
H	0.264501	-1.916094	2.879676
H	0.282008	-0.170668	2.592650
H	1.022515	-0.835817	4.041659
H	4.000387	0.103479	1.991573
H	2.515403	1.043709	2.169346
H	3.234728	0.261163	3.574042
H	-1.403531	-3.379030	0.491189
H	4.409292	2.520469	-1.914289
H	3.216948	4.489041	-1.047386
H	1.099900	4.213307	0.168792
H	-0.405047	0.930588	1.075260
H	-1.126136	2.572997	2.703869
H	0.627407	2.377162	2.823861
H	-0.054933	3.801213	2.030851
H	-2.309577	2.345506	0.481266
H	-1.411067	1.931390	-0.983448
H	-1.234492	3.535256	-0.269831
H	3.150196	-0.977591	-1.601243
H	4.266432	-0.780987	-3.741468
H	2.834683	0.258814	-3.724788
H	4.447105	0.963619	-3.552898
H	5.609966	-1.150462	-1.621901
H	5.087537	-0.441364	-0.088188
H	5.763155	0.588589	-1.350837
H	-1.930584	-4.438452	-1.712132
H	-0.228599	-4.586810	-1.270407
H	-0.716825	-3.403691	-2.489367
H	-2.610695	-1.565734	1.431673
H	-4.059563	-0.244579	-3.116806
H	-2.347939	-1.963928	-2.833005
H	-4.176894	0.063670	2.416755
H	-5.855981	1.839644	2.123369
H	-6.572890	2.520745	-0.153401
H	-5.611411	1.426355	-2.137841

ωB97X energy = -1315.45839885 a.u.

(5*S*,1’*R*)-**3b**, Conf. F

C	0.236574	1.477437	-0.960143
C	-1.111383	0.787136	-1.292642
N	-1.450046	0.150385	0.018577
C	-0.533942	0.454968	0.913972
N	0.421930	1.219305	0.472140
C	-2.174912	1.728208	-1.904356
C	-1.590062	2.246022	-3.226349
C	-2.509495	2.911873	-0.996883
C	-3.451897	0.947915	-2.233035
C	1.543613	1.734622	1.277307
C	-2.452770	-0.865262	0.241627
C	-2.279145	-2.118420	-0.361941

C	-3.294800	-3.057215	-0.209997
C	-4.415230	-2.781943	0.553505
C	-4.526986	-1.564721	1.201546
C	-3.552754	-0.580666	1.063590
C	-3.710289	0.715734	1.840635
C	-5.034229	1.418522	1.533835
C	-3.571281	0.461045	3.344858
C	-1.023208	-2.515243	-1.121861
C	-0.354423	-3.728683	-0.469861
C	-1.313514	-2.784006	-2.599925
C	1.461154	3.250633	1.395256
C	2.861432	1.213468	0.733107
C	3.425336	0.099980	1.291369
C	4.645426	-0.434109	0.805473
C	5.285695	0.201393	-0.283756
C	4.686800	1.357035	-0.846859
C	3.515441	1.851309	-0.352596
C	5.241643	-1.586673	1.379716
C	6.414950	-2.079691	0.886082
C	7.054019	-1.445409	-0.205234
C	6.504502	-0.333210	-0.774918
H	1.070170	1.051274	-1.518472
H	0.208164	2.551013	-1.139794
H	-0.945699	-0.012990	-2.014948
H	-0.570019	0.087390	1.932376
H	-1.327584	1.420391	-3.893414
H	-0.699917	2.861424	-3.079090
H	-2.332745	2.863829	-3.734540
H	-1.630152	3.511035	-0.748137
H	-2.976094	2.590098	-0.065506
H	-3.215894	3.571167	-1.505571
H	-3.235870	0.084425	-2.868748
H	-3.964307	0.589476	-1.340208
H	-4.143406	1.595481	-2.776288
H	1.399100	1.300991	2.268201
H	-3.195641	-4.028805	-0.681013
H	-5.193892	-3.528020	0.662358
H	-5.390637	-1.372789	1.828333
H	-2.906728	1.394200	1.553341
H	-5.074439	2.379755	2.050764
H	-5.156749	1.603722	0.464338
H	-5.886604	0.824825	1.871352
H	-3.636787	1.403052	3.893441
H	-2.615908	-0.009870	3.588513
H	-4.367370	-0.195311	3.704812
H	-0.302074	-1.698959	-1.059551
H	0.587494	-3.951443	-0.975600
H	-0.141148	-3.542385	0.584722
H	-0.987181	-4.616616	-0.536213
H	-0.389203	-3.023281	-3.129821
H	-1.772444	-1.920765	-3.088479
H	-1.995430	-3.630074	-2.714741
H	2.270721	3.606412	2.032929
H	0.510133	3.540248	1.843606
H	1.552616	3.747584	0.428561
H	2.939562	-0.395427	2.127415
H	5.178157	1.848836	-1.679668
H	3.087644	2.742805	-0.797473
H	4.748914	-2.069399	2.217248
H	6.864030	-2.961207	1.328843
H	7.984580	-1.849378	-0.586945
H	6.991850	0.157163	-1.611084

ωB97X energy = -1315.45832486 a.u.

(5*S*,1’*R*)-**3b**, Conf. G

C	1.095082	2.630822	-0.245757
C	2.192951	1.545677	-0.117513
N	1.371226	0.296901	-0.081712
C	0.091316	0.604255	-0.066074
N	-0.162114	1.881478	-0.126030
C	3.306792	1.637362	-1.184996
C	3.965619	3.011562	-0.997396
C	2.777357	1.519862	-2.614060
C	4.374106	0.564875	-0.943022
C	-1.486255	2.503982	-0.338409
C	1.847944	-1.023896	0.262074
C	2.298288	-1.254657	1.569027
C	2.840230	-2.503770	1.855095
C	2.885926	-3.500394	0.896469
C	2.360052	-3.272539	-0.362838
C	1.827507	-2.034335	-0.709031
C	1.212234	-1.859457	-2.086988
C	2.189137	-2.220672	-3.207647
C	-0.075224	-2.681100	-2.205847
C	2.167466	-0.236529	2.690975
C	1.282198	-0.783348	3.814478
C	3.531143	0.197528	3.231644
C	-1.564360	3.830794	0.406544
C	-2.588135	1.546530	0.057227
C	-3.411942	1.015461	-0.893647
C	-4.463457	0.127274	-0.544501
C	-4.648975	-0.214417	0.815069
C	-3.775795	0.344349	1.786072
C	-2.779469	1.198803	1.421514
C	-5.328752	-0.429543	-1.520141
C	-6.329380	-1.283594	-1.153537
C	-6.516135	-1.623570	0.205891
C	-5.696695	-1.102448	1.166173
H	1.165459	3.379591	0.540562
H	1.115217	3.134727	-1.213326
H	2.682101	1.637507	0.854585
H	-0.684247	-0.147902	0.006090
H	4.364449	3.124620	0.014332
H	3.271562	3.833956	-1.185996
H	4.795578	3.115686	-1.698827
H	2.055443	2.300932	-2.861471
H	2.306481	0.553815	-2.795063
H	3.608392	1.616278	-3.315941
H	4.752758	0.604699	0.082183
H	4.002052	-0.443333	-1.127923
H	5.217295	0.736270	-1.615610

H	-1.567306	2.698648	-1.411719
H	3.214723	-2.705829	2.852411
H	3.311578	-4.467225	1.139085
H	2.362194	-4.072970	-1.094023
H	0.938393	-0.811573	-2.212662
H	1.743808	-1.993337	-4.178638
H	3.125250	-1.663806	-3.123865
H	2.430430	-3.285937	-3.196272
H	-0.535775	-2.522168	-3.183326
H	-0.802325	-2.404905	-1.438254
H	0.131963	-3.748874	-2.099771
H	1.659932	0.649006	2.305940
H	1.137665	-0.019489	4.581556
H	0.301144	-1.077317	3.434786
H	1.736393	-1.654824	4.291475
H	3.407251	0.959835	4.003628
H	4.167282	0.610671	2.444898
H	4.061252	-0.647569	3.677389
H	-2.554611	4.259371	0.254116
H	-0.829986	4.543569	0.029989
H	-1.405578	3.697322	1.477747
H	-3.272678	1.270190	-1.940189
H	-3.917359	0.082997	2.829405
H	-2.123803	1.613096	2.180185
H	-5.182115	-0.166370	-2.562461
H	-6.987546	-1.704764	-1.904667
H	-7.314615	-2.302536	0.481982
H	-5.836522	-1.359898	2.210781

ωB97X energy = -1315.45828084 a.u.

(5*S*,1’*R*)-**3b**, Conf. H

C	0.068231	-1.931521	0.688175
C	-1.073251	-1.001297	1.171732
N	-1.440995	-0.294242	-0.094982
C	-0.614296	-0.652247	-1.057549
N	0.251133	-1.561702	-0.719089
C	-2.205228	-1.726596	1.932187
C	-1.549992	-2.391446	3.151503
C	-2.906282	-2.791529	1.089080
C	-3.231285	-0.711175	2.444696
C	1.372235	-2.032066	-1.550559
C	-2.204176	0.931611	-0.180042
C	-1.638534	2.103384	0.342530
C	-2.412638	3.259446	0.320101
C	-3.677384	3.262280	-0.241690
C	-4.180749	2.110063	-0.819352
C	-3.455917	0.922082	-0.808067
C	-4.020108	-0.293733	-1.522188
C	-5.403164	-0.687001	-1.000236
C	-4.060691	-0.052504	-3.034382
C	-0.214568	2.182693	0.873176
C	0.624088	3.155514	0.039022
C	-0.179067	2.559699	2.355341
C	1.330653	-3.544867	-1.691398
C	2.666940	-1.458009	-0.988944
C	2.956008	-0.139603	-1.217673
C	4.112927	0.469818	-0.676105
C	4.990182	-0.309820	0.114344
C	4.680584	-1.675191	0.329734
C	3.554018	-2.233681	-0.203718
C	4.414270	1.839824	-0.897626
C	5.534355	2.399106	-0.355294
C	6.412518	1.619037	0.435196
C	6.148935	0.299834	0.662967
H	0.998050	-1.772831	1.235020
H	-0.201298	-2.985150	0.753501
H	-0.671732	-0.249584	1.855138
H	-0.645995	-0.204714	-2.043849
H	-1.023303	-1.657575	3.767570
H	-0.840844	-3.172826	2.869134
H	-2.320255	-2.856508	3.769467
H	-2.215802	-3.548442	0.709854
H	-3.431553	-2.355789	0.239074
H	-3.646788	-3.308619	1.702883
H	-2.752437	0.061593	3.052672
H	-3.769605	-0.218039	1.634964
H	-3.966305	-1.220585	3.071636
H	1.206288	-1.585765	-2.532670
H	-2.009228	4.178355	0.730822
H	-4.263302	4.174144	-0.251458
H	-5.154728	2.133969	-1.295099
H	-3.347282	-1.134115	-1.349765
H	-5.741311	-1.601727	-1.492008
H	-5.394238	-0.865926	0.077294
H	-6.141349	0.091709	-1.205348
H	-4.420017	-0.946704	-3.548304
H	-3.071223	0.192415	-3.427908
H	-4.734261	0.771731	-3.281993
H	0.260723	1.206290	0.765888
H	1.662060	3.141000	0.381126
H	0.610383	2.882746	-1.018853
H	0.254398	4.179525	0.129689
H	0.849998	2.562068	2.720525
H	-0.755881	1.859572	2.965003
H	-0.593770	3.557890	2.515060
H	2.155076	-3.875941	-2.323176
H	0.392633	-3.848536	-2.156435
H	1.416726	-4.051772	-0.729304
H	2.291224	0.469337	-1.825739
H	5.353683	-2.276858	0.931227
H	3.345291	-3.281191	-0.022448
H	3.738506	2.433430	-1.505054
H	5.757441	3.445826	-0.526683
H	7.298248	2.078038	0.859016
H	6.819135	-0.301469	1.268256

ωB97X energy = -1315.45802171 a.u.

(5*S*,1’*R*)-**3b**, Conf. I

C	-0.013949	-1.683430	-1.259617
C	1.166294	-0.690677	-1.412641

N	1.408240	-0.288854	0.009755
C	0.499192	-0.838152	0.786480
N	-0.334462	-1.628754	0.172657
C	2.367438	-1.255153	-2.203721
C	1.836079	-1.600359	-3.602510
C	2.965863	-2.507308	-1.563789
C	3.452221	-0.184945	-2.363919
C	-1.449110	-2.337058	0.808826
C	2.216042	0.828888	0.447192
C	1.789297	2.124745	0.123330
C	2.616772	3.185617	0.477539
C	3.797461	2.972331	1.166703
C	4.157308	1.689999	1.540385
C	3.376564	0.590663	1.195544
C	3.795254	-0.785134	1.685581
C	5.204910	-1.158871	1.221930
C	3.694362	-0.866016	3.212047
C	0.455306	2.428357	-0.541901
C	-0.421205	3.294212	0.367039
C	0.637824	3.081101	-1.913344
C	-1.552529	-3.749911	0.241913
C	-2.751938	-1.556904	0.696524
C	-2.844788	-0.342909	0.079371
C	-4.075673	0.361092	0.004618
C	-5.229331	-0.205613	0.590727
C	-5.110012	-1.461930	1.241292
C	-3.915830	-2.114744	1.292188
C	-4.177818	1.619179	-0.641556
C	-5.374599	2.274587	-0.700264
C	-6.529422	1.706131	-0.115514
C	-6.458419	0.496620	0.515122
H	-0.881929	-1.397329	-1.853305
H	0.267767	-2.700856	-1.526544
H	0.830911	0.201154	-1.945934
H	0.436174	-0.631635	1.848126
H	1.390329	-0.725169	-4.083005
H	1.088268	-2.396315	-3.579975
H	2.660169	-1.944155	-4.230312
H	2.241813	-3.320135	-1.473656
H	3.372759	-2.304128	-0.573303
H	3.785102	-2.873702	-2.185933
H	3.045634	0.728631	-2.806894
H	3.922107	0.079075	-1.416345
H	4.233200	-0.557629	-3.030121
H	-1.186774	-2.410110	1.867409
H	2.322179	4.198566	0.226688
H	4.428134	3.812078	1.434735
H	5.064720	1.537523	2.113809
H	3.103811	-1.523436	1.278222
H	5.439140	-2.181463	1.525705
H	5.306547	-1.096429	0.136282
H	5.954494	-0.500131	1.666364
H	3.942518	-1.873749	3.552177
H	2.688618	-0.626081	3.564173
H	4.390037	-0.169975	3.687194
H	-0.087051	1.492916	-0.688743
H	-1.398506	3.452381	-0.095463
H	-0.576010	2.816888	1.337180
H	0.028911	4.274545	0.537910
H	-0.332258	3.237287	-2.389986
H	1.248611	2.463713	-2.576845
H	1.126976	4.053633	-1.821051
H	-2.295913	-4.318434	0.797752
H	-0.596233	-4.266994	0.331860
H	-1.856606	-3.735300	-0.806175
H	-1.976466	0.125860	-0.370275
H	-5.988949	-1.897321	1.704879
H	-3.851485	-3.067661	1.805558
H	-3.289030	2.051802	-1.091265
H	-5.445138	3.235518	-1.196678
H	-7.472859	2.236791	-0.170851
H	-7.341555	0.056579	0.966303

ωB97X energy = -1315.45711003 a.u.

(5*S*,1'*S*)-**4a**, Conf. A

C	0.029801	-1.092973	1.119258
N	0.370736	-0.223322	-0.048493
C	-0.673861	-0.109438	-0.841283
N	-1.743249	-0.729677	-0.432778
C	-1.439168	-1.475926	0.798439
C	0.244348	-0.492549	2.528087
C	1.691073	0.219041	-0.431988
C	-3.032557	-0.744124	-1.152701
C	2.627572	-0.740968	-0.838518
C	3.919366	-0.306909	-1.119837
C	4.254838	1.033420	-1.051472
C	3.291682	1.970356	-0.722124
C	1.991388	1.588807	-0.406150
C	-0.166603	-1.582151	3.528714
C	-0.590302	0.763715	2.774909
C	1.726617	-0.178770	2.757585
C	0.967226	2.668534	-0.098242
C	2.288300	-2.209528	-1.039977
C	2.550783	-2.632073	-2.488328
C	3.052025	-3.109592	-0.066523
C	1.417053	3.587111	1.039165
C	0.644459	3.481205	-1.355903
C	-3.993438	0.384465	-0.686303
C	-3.606678	-2.153979	-1.108335
C	-3.323679	1.745704	-0.897686
C	-5.251715	0.324677	-1.561235
C	-4.399319	0.252121	0.783256
H	0.659951	-1.981526	1.059747
H	-0.636035	0.445196	-1.771311
H	-1.552075	-2.543877	0.615880
H	-2.126980	-1.188377	1.590379
H	-2.774833	-0.521434	-2.192114
H	4.670048	-1.029434	-1.419949
H	5.266016	1.351627	-1.277476
H	3.554521	3.022055	-0.708476

H	0.413968	-2.496452	3.378894
H	-1.226771	-1.835339	3.457076
H	0.017300	-1.230564	4.545591
H	-1.662684	0.579071	2.680072
H	-0.415779	1.122053	3.791532
H	-0.323036	1.572039	2.093776
H	2.354452	-1.050685	2.553940
H	1.879664	0.103620	3.801397
H	2.080780	0.644055	2.136119
H	0.041385	2.187946	0.219361
H	1.220116	-2.349102	-0.867766
H	3.613215	-2.571083	-2.734518
H	2.232602	-3.665887	-2.638650
H	2.002847	-1.999531	-3.189686
H	2.769979	-4.153894	-0.215692
H	2.850417	-2.848716	0.975398
H	4.129919	-3.027484	-0.225605
H	0.624604	4.299332	1.279045
H	1.652003	3.023830	1.945185
H	2.304164	4.160181	0.760489
H	-0.130424	4.219665	-1.138684
H	0.289253	2.843186	-2.168849
H	1.528589	4.014346	-1.714049
H	-2.863249	-2.875533	-1.449728
H	-4.465536	-2.225337	-1.772727
H	-3.929299	-2.438627	-0.105488
H	-4.018536	2.544877	-0.632198
H	-2.437889	1.870638	-0.268522
H	-3.033170	1.891692	-1.942382
H	-5.870463	1.204019	-1.371525
H	-5.856882	-0.557206	-1.344553
H	-4.998845	0.315497	-2.625303
H	-5.160266	0.999556	1.018267
H	-4.822551	-0.728707	1.009323
H	-3.558176	0.434245	1.455828

ωB97X energy = -1088.02385025 a.u.

(5*S*,1’*S*)-**4a**, Conf. B

C	-0.020544	-0.461155	1.554349
N	-0.310065	-0.202484	0.108641
C	0.805674	-0.301635	-0.587525
N	1.846361	-0.673132	0.100941
C	1.444323	-0.952381	1.485310
C	-0.968903	-1.403907	2.328173
C	-1.480828	0.460214	-0.421841
C	3.193161	-0.973122	-0.422948
C	-1.670493	1.819875	-0.136872
C	-2.840807	2.419683	-0.591161
C	-3.763846	1.711536	-1.340122
C	-3.516817	0.391501	-1.671662
C	-2.372290	-0.263016	-1.225890
C	-0.392944	-1.514084	3.747689
C	-1.064551	-2.800312	1.714537
C	-2.369673	-0.791326	2.431581
C	-2.125029	-1.692527	-1.676327
C	-0.640864	2.678711	0.581158
C	-0.142281	3.799248	-0.335863
C	-1.183484	3.246125	1.893883
C	-3.300285	-2.613580	-1.341992
C	-1.817159	-1.734708	-3.176297
C	4.081799	0.298217	-0.497971
C	3.083693	-1.772234	-1.716610
C	3.544423	1.321104	-1.500664
C	4.162689	0.951808	0.885024
C	5.492936	-0.129978	-0.916006
H	-0.055195	0.505220	2.064228
H	0.839295	-0.082980	-1.647337
H	2.091918	-0.427779	2.184480
H	1.535251	-2.025552	1.661531
H	3.633174	-1.624024	0.337538
H	-3.021576	3.466509	-0.373883
H	-4.667802	2.197878	-1.688152
H	-4.225169	-0.143128	-2.294278
H	-0.290388	-0.529875	4.212701
H	0.583924	-2.003184	3.760839
H	-1.065112	-2.109690	4.368161
H	-1.701084	-3.426396	2.343427
H	-1.508457	-2.776884	0.720259
H	-0.094653	-3.296569	1.642032
H	-2.331923	0.212642	2.863411
H	-2.988889	-1.410369	3.084425
H	-2.869652	-0.726632	1.464407
H	-1.244770	-2.071202	-1.155808
H	0.230851	2.066723	0.817056
H	-0.944826	4.497089	-0.585531
H	0.650435	4.363466	0.160322
H	0.257875	3.395608	-1.268944
H	-0.409669	3.823292	2.404239
H	-1.518480	2.455015	2.569124
H	-2.031837	3.910250	1.712445
H	-3.046927	-3.647937	-1.584557
H	-3.562043	-2.567321	-0.282442
H	-4.189542	-2.347354	-1.917680
H	-1.596340	-2.758177	-3.487151
H	-0.958559	-1.108373	-3.429692
H	-2.671282	-1.381572	-3.759575
H	2.412471	-2.620685	-1.575563
H	4.059979	-2.162371	-1.998606
H	2.711997	-1.172359	-2.549429
H	4.222654	2.175967	-1.545168
H	3.468848	0.911848	-2.510242
H	2.564939	1.709121	-1.206348
H	4.861167	1.790709	0.857591
H	4.520124	0.245245	1.639431
H	3.195779	1.346275	1.207523
H	6.172092	0.721908	-0.842883
H	5.875693	-0.920659	-0.264510
H	5.524241	-0.488739	-1.946180

ωB97X energy = -1088.02352133 a.u.

(5*S*,1’*R*)-**4b**, Conf. A

C	0.123818	-1.029564	1.307898
N	0.337361	-0.243367	0.053044
C	-0.759792	-0.253466	-0.675593
N	-1.764718	-0.882647	-0.138484
C	-1.344584	-1.496115	1.128820
C	0.411533	-0.311045	2.645872
C	1.602963	0.221617	-0.464795
C	-3.095708	-1.093111	-0.740994
C	2.551585	-0.728162	-0.868460
C	3.793799	-0.263883	-1.289455
C	4.066722	1.090664	-1.356458
C	3.087926	2.009505	-1.022684
C	1.836834	1.600164	-0.571048
C	-0.483175	0.907589	2.872323
C	1.883640	0.107127	2.724449
C	0.157398	-1.342779	3.753886
C	0.792468	2.660286	-0.259446
C	2.273270	-2.222321	-0.918591
C	2.456074	-2.762698	-2.339745
C	3.145370	-2.996666	0.072002
C	1.281809	3.658438	0.791997
C	0.364009	3.390063	-1.536288
C	-4.036456	0.125781	-0.537680
C	-2.939412	-1.567676	-2.181934
C	-5.410733	-0.232571	-1.114459
C	-4.193677	0.406151	0.959563
C	-3.515267	1.389664	-1.225588
H	0.790284	-1.892451	1.267631
H	-0.801465	0.211597	-1.652444
H	-1.429732	-2.579799	1.046127
H	-1.993984	-1.158310	1.934756
H	-3.518150	-1.918459	-0.162057
H	4.553137	-0.976311	-1.591817
H	5.039081	1.432382	-1.692069
H	3.298904	3.068964	-1.114366
H	-0.205244	1.392909	3.810210
H	-1.539136	0.640134	2.953618
H	-0.378371	1.647735	2.077726
H	2.130931	0.901782	2.020647
H	2.101915	0.474238	3.729546
H	2.549078	-0.737943	2.527097
H	-0.880072	-1.684159	3.776845
H	0.375781	-0.894850	4.725159
H	0.802585	-2.217222	3.634029
H	-0.094061	2.169717	0.144844
H	1.227677	-2.393612	-0.658805
H	2.179467	-3.818535	-2.376025
H	1.830489	-2.220005	-3.051491
H	3.494547	-2.678656	-2.667801
H	2.904216	-4.061123	0.038049
H	3.003840	-2.648804	1.098325
H	4.204564	-2.884131	-0.171933
H	0.484368	4.363023	1.037849
H	1.587799	3.158624	1.713655
H	2.133007	4.235424	0.423888
H	-0.418658	4.117214	-1.309116
H	-0.022420	2.697209	-2.287812
H	1.205098	3.927786	-1.980588
H	-3.893687	-1.925509	-2.564342
H	-2.229644	-2.394806	-2.226606
H	-2.590173	-0.776753	-2.848029
H	-6.131806	0.544877	-0.854063
H	-5.388825	-0.312032	-2.202600
H	-5.777582	-1.179346	-0.707823
H	-4.922077	1.204997	1.113546
H	-3.254614	0.735791	1.410990
H	-4.548789	-0.477966	1.496475
H	-4.253113	2.187891	-1.120873
H	-2.590702	1.755950	-0.769344
H	-3.345439	1.242674	-2.294482

ωB97X energy = -1088.02377625 a.u.

(5*S*,1’*R*)-**4b**, Conf. B

C	0.059739	-0.782955	1.282919
N	-0.351169	-0.240941	-0.049722
C	0.709748	-0.159394	-0.829726
N	1.803512	-0.646764	-0.319797
C	1.508362	-1.239273	0.993765
C	-0.828441	-1.867939	1.930451
C	-1.540197	0.543320	-0.304144
C	3.105544	-0.709618	-1.013146
C	-1.633995	1.820453	0.267778
C	-2.821370	2.523379	0.089200
C	-3.855316	2.000603	-0.667349
C	-3.706714	0.771703	-1.285143
C	-2.548498	0.017769	-1.122578
C	-0.965918	-3.126636	1.073359
C	-2.217973	-1.304237	2.246459
C	-0.151112	-2.233382	3.259303
C	-2.418134	-1.297572	-1.870464
C	-0.490911	2.492040	1.014674
C	-0.065096	3.780402	0.304399
C	-0.843641	2.766215	2.477813
C	-3.566162	-2.258157	-1.553223
C	-2.328239	-1.050790	-3.379763
C	4.001386	0.514137	-0.674491
C	3.734684	-2.075181	-0.770944
C	5.322493	0.364119	-1.437618
C	3.310986	1.795371	-1.152467
C	4.293882	0.637274	0.822774
H	0.076092	0.061984	1.976665
H	0.670350	0.292829	-1.813450
H	2.211262	-0.889232	1.745338
H	1.589229	-2.323891	0.917724
H	2.865612	-0.636129	-2.077556
H	-2.930824	3.505843	0.534601
H	-4.772408	2.563980	-0.795354
H	-4.506157	0.389352	-1.909720
H	-1.609472	-3.842724	1.588921

H	-0.008822	-3.622429	0.899066
H	-1.419419	-2.914868	0.105462
H	-2.795873	-1.090817	1.346824
H	-2.778811	-2.031719	2.837217
H	-2.149280	-0.382370	2.830899
H	0.832426	-2.685364	3.110790
H	-0.767245	-2.957646	3.795449
H	-0.032849	-1.354670	3.899213
H	-1.486089	-1.773737	-1.564732
H	0.379607	1.832980	1.001066
H	0.811706	4.206924	0.796879
H	0.188397	3.590303	-0.741244
H	-0.859731	4.529435	0.328128
H	0.008562	3.213201	2.993816
H	-1.119305	1.850378	3.006502
H	-1.683726	3.460596	2.553903
H	-3.393093	-3.221367	-2.038285
H	-3.666941	-2.432244	-0.479717
H	-4.518767	-1.869303	-1.920354
H	-2.185832	-1.996651	-3.906919
H	-1.495587	-0.390431	-3.632279
H	-3.245881	-0.591116	-3.755518
H	4.601164	-2.203330	-1.416576
H	3.023563	-2.866635	-1.012052
H	4.059978	-2.204923	0.262828
H	5.901056	1.285925	-1.350336
H	5.932939	-0.448401	-1.039996
H	5.149793	0.176102	-2.501144
H	3.963042	2.654310	-0.981458
H	2.380352	1.989883	-0.611907
H	3.087578	1.753591	-2.222377
H	5.016173	1.440219	0.986017
H	3.396534	0.893650	1.391778
H	4.723650	-0.277464	1.237486

ωB97X energy = -1088.02361193 a.u.

(5*S*,1'*R*)-**4b**, Conf. C

C	0.098903	-0.152270	1.455259
N	-0.365848	-0.066127	0.037097
C	0.662994	-0.173411	-0.778230
N	1.816997	-0.298221	-0.187990
C	1.635941	-0.205251	1.267779
C	-0.485329	-1.303436	2.306525
C	-1.678947	0.342378	-0.404667
C	3.083083	-0.590751	-0.886991
C	-2.087391	1.658390	-0.147203
C	-3.385765	2.013869	-0.499256
C	-4.228420	1.115297	-1.128682
C	-3.775307	-0.154301	-1.440094
C	-2.495089	-0.570612	-1.087772
C	-0.158377	-2.683702	1.736323
C	-2.002440	-1.144016	2.447159
C	0.131578	-1.167892	3.705828
C	-2.038906	-1.960165	-1.501075
C	-1.171411	2.719760	0.441888
C	-1.007438	3.892906	-0.528343
C	-1.666746	3.205201	1.805846
C	-2.976386	-3.056580	-0.991606
C	-1.887527	-2.042616	-3.023027
C	4.137030	0.542455	-0.749939
C	3.564328	-1.980143	-0.484933
C	5.149199	0.364959	-1.889681
C	3.458892	1.904277	-0.920771
C	4.896215	0.500672	0.580388
H	-0.176923	0.778290	1.951868
H	0.554714	-0.151548	-1.856396
H	2.117418	0.698094	1.641074
H	2.089025	-1.066521	1.754537
H	2.799524	-0.619826	-1.942455
H	-3.735988	3.019900	-0.296738
H	-5.235463	1.412651	-1.397515
H	-4.429694	-0.838492	-1.968338
H	-0.549351	-3.454570	2.403654
H	0.917016	-2.854435	1.642497
H	-0.613708	-2.838382	0.757430
H	-2.525523	-1.281947	1.501164
H	-2.382707	-1.889135	3.149358
H	-2.262308	-0.155507	2.836682
H	1.213447	-1.317161	3.703541
H	-0.299512	-1.921684	4.367285
H	-0.077187	-0.184825	4.136447
H	-1.055413	-2.145764	-1.069090
H	-0.176881	2.291312	0.574704
H	-0.292650	4.613800	-0.125509
H	-0.639971	3.553173	-1.499025
H	-1.954285	4.413945	-0.687171
H	-0.968215	3.932424	2.224912
H	-1.769922	2.382659	2.518265
H	-2.641834	3.689912	1.716136
H	-2.572095	-4.039038	-1.244763
H	-3.101261	-3.011520	0.092504
H	-3.965609	-2.977283	-1.447746
H	-1.504572	-3.023655	-3.312091
H	-1.198728	-1.283010	-3.400150
H	-2.849909	-1.895761	-3.519438
H	4.465457	-2.242922	-1.037969
H	2.798777	-2.722939	-0.714044
H	3.797689	-2.042150	0.578973
H	5.900832	1.155509	-1.840355
H	5.672591	-0.591066	-1.827581
H	4.660326	0.426331	-2.865524
H	4.215321	2.690689	-0.960167
H	2.784795	2.139835	-0.093669
H	2.884675	1.947646	-1.850740
H	5.611549	1.325525	0.612510
H	4.242472	0.605159	1.448173
H	5.459927	-0.427388	0.692650

ωB97X energy = -1088.02309249 a.u.

(5*R*,1'*R*)-**5a**, Conf. A

C	0.884466	-3.647315	0.729245
C	-0.161922	-4.529906	0.492941
C	-0.425559	-4.967339	-0.796861
C	0.366538	-4.522833	-1.848079
C	1.412910	-3.643888	-1.607989
C	1.682971	-3.193633	-0.316760
C	2.808612	-2.221369	-0.064410
C	2.505805	-0.819786	-0.605136
N	1.273373	-0.294811	-0.006121
C	0.147231	-0.081354	-0.625876
N	-0.794318	0.447115	0.124974
C	-2.164011	0.589083	-0.309834
C	-3.126934	-0.279041	0.223794
C	-2.791331	-1.461932	1.119819
C	-3.343823	-1.276021	2.535144
C	-3.305631	-2.768995	0.510015
C	-4.455989	-0.063990	-0.127708
C	-4.810161	0.934861	-1.016529
C	-3.831411	1.725765	-1.590984
C	-2.489953	1.574717	-1.253437
C	-1.462639	2.452533	-1.950174
C	-1.370686	2.096198	-3.437101
C	-1.759725	3.942937	-1.771638
C	-0.277830	0.665884	1.513487
C	-0.297695	2.120407	2.040400
C	-1.733893	2.648301	2.119763
C	0.554494	3.064724	1.191550
C	0.260765	2.070285	3.469988
C	1.139976	0.048012	1.413098
C	3.621528	0.200722	-0.425568
C	4.747664	-0.047039	0.349253
C	5.731023	0.926052	0.492642
C	5.595294	2.154171	-0.133869
C	4.471431	2.409737	-0.911408
C	3.494836	1.438432	-1.055716
H	1.090579	-3.322782	1.744794
H	-0.769131	-4.878423	1.320804
H	-1.241240	-5.656695	-0.982758
H	0.173434	-4.867377	-2.857659
H	2.034506	-3.312031	-2.434464
H	3.020995	-2.174513	1.006016
H	3.721518	-2.568500	-0.553655
H	2.293418	-0.905240	-1.673558
H	-0.003643	-0.322048	-1.671531
H	-1.705413	-1.561385	1.184194
H	-3.058017	-2.119745	3.166867
H	-2.973108	-0.360889	3.003261
H	-4.435115	-1.220776	2.521056
H	-3.004313	-3.613446	1.133639
H	-2.896692	-2.926356	-0.490483
H	-4.395615	-2.777534	0.440727
H	-5.225762	-0.705871	0.285391
H	-5.851196	1.083084	-1.279211
H	-4.114879	2.480068	-2.316239
H	-0.482559	2.262233	-1.511410
H	-0.595744	2.694265	-3.921223
H	-1.130249	1.040889	-3.585932
H	-2.317429	2.295572	-3.944993
H	-0.958638	4.538455	-2.214842
H	-1.842299	4.216242	-0.717398
H	-2.693016	4.222620	-2.265156
H	-0.891906	0.070511	2.189220
H	-2.371021	1.982357	2.708378
H	-1.733103	3.624335	2.609910
H	-2.190393	2.771087	1.137789
H	0.161196	3.167792	0.179669
H	0.554832	4.058585	1.643973
H	1.596497	2.742332	1.119121
H	1.304122	1.749266	3.502965
H	-0.325327	1.394665	4.099003
H	0.211828	3.066434	3.913609
H	1.927176	0.746317	1.693725
H	1.240231	-0.851420	2.021120
H	4.877208	-0.999305	0.848758
H	6.605016	0.717531	1.098699
H	6.362089	2.911729	-0.020744
H	4.358447	3.366095	-1.408614
H	2.621218	1.645452	-1.667172

ωB97X energy = -1392.87444377 a.u.

(5*R*,1'*R*)-**5a**, Conf. B

C	0.857567	3.651618	-0.723461
C	-0.181894	4.537921	-0.470937
C	-0.409586	4.993864	0.819316
C	0.411641	4.564296	1.854371
C	1.450879	3.681553	1.598014
C	1.684753	3.212541	0.306363
C	2.800802	2.233717	0.037097
C	2.498126	0.835492	0.587140
N	1.265600	0.304569	-0.008598
C	0.139946	0.095198	0.613084
N	-0.797972	-0.449423	-0.131166
C	-2.168947	-0.587796	0.300229
C	-3.133137	0.261161	-0.261196
C	-2.797961	1.421910	-1.185749
C	-3.346830	1.199473	-2.597115
C	-3.315155	2.742867	-0.609297
C	-4.463240	0.049017	0.088013
C	-4.817918	-0.928148	1.000360
C	-3.838562	-1.698788	1.600832
C	-2.495758	-1.549795	1.267765
C	-1.468963	-2.403839	1.994494
C	-1.395558	-2.015199	3.474559
C	-1.752861	-3.900146	1.845986
C	-0.273776	-0.697834	-1.511788
C	-0.267103	-2.167808	-1.994340
C	0.597707	-3.070671	-1.113648
C	0.294795	-2.153366	-3.423419
C	-1.694577	-2.720366	-2.061759
C	1.132464	-0.053641	-1.424072

C	3.612532	-0.185946	0.415599
C	3.574107	-1.344934	1.189602
C	4.544247	-2.323684	1.046917
C	5.569771	-2.155036	0.123920
C	5.617212	-1.004598	-0.647353
C	4.642602	-0.023572	-0.503056
H	1.035445	3.312121	-1.739500
H	-0.811915	4.875075	-1.286316
H	-1.219404	5.686537	1.017951
H	0.246648	4.923844	2.863714
H	2.095288	3.360730	2.411269
H	2.992444	2.185387	-1.036806
H	3.725540	2.573902	0.508800
H	2.284977	0.927408	1.654499
H	-0.013414	0.351750	1.654466
H	-1.712160	1.521369	-1.250157
H	-3.059345	2.026244	-3.250066
H	-2.974969	0.272327	-3.040154
H	-4.438137	1.144464	-2.584708
H	-3.016309	3.571610	-1.254822
H	-2.906021	2.926313	0.386677
H	-4.405113	2.750484	-0.539469
H	-5.233435	0.676202	-0.346410
H	-5.859742	-1.074963	1.260651
H	-4.122733	-2.435834	2.343357
H	-0.485592	-2.215935	1.562106
H	-0.619020	-2.594729	3.978297
H	-1.168145	-0.954533	3.604712
H	-2.344814	-2.214854	3.977639
H	-0.954657	-4.479297	2.315314
H	-1.816898	-4.198807	0.797364
H	-2.691853	-4.174841	2.331399
H	-0.895155	-0.133540	-2.206961
H	0.199872	-3.152156	-0.101506
H	0.620635	-4.077087	-1.536808
H	1.632807	-2.725537	-1.044706
H	1.334090	-1.820388	-3.464120
H	0.259664	-3.162858	-3.837161
H	-0.298067	-1.504360	-4.073742
H	-2.339228	-2.082148	-2.672577
H	-2.152747	-2.819520	-1.077953
H	-1.677432	-3.710829	-2.521709
H	1.930658	-0.739738	-1.702922
H	1.213652	0.842473	-2.040222
H	2.777473	-1.481642	1.915247
H	4.503054	-3.217598	1.658380
H	6.330585	-2.918619	0.010878
H	6.414351	-0.864869	-1.368228
H	4.701773	0.867648	-1.116160

ωB97X energy = -1392.87439587 a.u.

(5*R*,1’*R*)-**5a**, Conf. C

C	0.922896	3.115518	-1.572440
C	0.183857	4.252939	-1.266764
C	0.482947	4.992553	-0.132846
C	1.532335	4.595287	0.687416
C	2.267916	3.461469	0.378847
C	1.967633	2.702327	-0.752003
C	2.749233	1.447202	-1.066102
C	2.708687	0.413980	0.067205
N	1.332654	0.066938	0.435138
C	0.305111	-0.085264	-0.351761
N	-0.803110	-0.434858	0.266145
C	-1.974903	-0.932147	-0.418033
C	-2.227741	-2.310444	-0.365759
C	-1.257657	-3.320209	0.228233
C	-1.860923	-4.046202	1.432746
C	-0.795927	-4.320361	-0.835414
C	-3.409034	-2.775006	-0.935122
C	-4.283063	-1.912886	-1.572367
C	-3.971272	-0.569286	-1.679137
C	-2.811586	-0.047355	-1.113723
C	-2.494261	1.423203	-1.334769
C	-2.257548	1.693723	-2.824273
C	-3.583558	2.350275	-0.792289
C	-0.561916	-0.500937	1.742212
C	-1.362329	0.510406	2.594785
C	-2.862634	0.225890	2.476048
C	-1.066322	1.959401	2.204886
C	-0.960861	0.277341	4.057947
C	0.972657	-0.318429	1.803872
C	3.534080	-0.823846	-0.235694
C	4.767154	-0.992218	0.383856
C	5.556712	-2.097329	0.093020
C	5.115334	-3.046386	-0.817089
C	3.882526	-2.886106	-1.437015
C	3.097823	-1.779911	-1.148579
H	0.688954	2.551625	-2.471246
H	-0.624733	4.562287	-1.920262
H	-0.092128	5.879020	0.108327
H	1.780114	5.173263	1.570483
H	3.088854	3.169634	1.026648
H	2.394565	1.000588	-1.999101
H	3.803659	1.682940	-1.227663
H	3.123060	0.881971	0.962092
H	0.350113	0.048123	-1.425050
H	-0.360530	-2.796409	0.561195
H	-2.727951	-4.640583	1.134538
H	-1.126518	-4.723534	1.873466
H	-2.189861	-3.348287	2.206980
H	-1.627627	-4.927406	-1.200240
H	-0.049550	-4.996309	-0.412440
H	-0.348318	-3.809072	-1.690578
H	-3.637540	-3.834159	-0.895223
H	-5.198495	-2.293803	-2.010064
H	-4.642117	0.090471	-2.217860
H	-1.568890	1.668370	-0.810977
H	-1.957965	2.733218	-2.977116
H	-1.477803	1.047616	-3.234841
H	-3.168531	1.522133	-3.402340
H	-3.288755	3.392493	-0.936630

H	-3.758042	2.194398	0.274059
H	-4.530593	2.197091	-1.314896
H	-0.835284	-1.498924	2.083669
H	-3.088409	-0.810326	2.743710
H	-3.413143	0.875033	3.160369
H	-3.244164	0.403450	1.471431
H	-1.348694	2.171257	1.171807
H	-1.638654	2.633974	2.845114
H	-0.010266	2.215957	2.324498
H	0.089171	0.507272	4.249446
H	-1.144010	-0.758624	4.356174
H	-1.559225	0.922419	4.704096
H	1.276382	0.468638	2.491899
H	1.498152	-1.237917	2.068938
H	5.113160	-0.255315	1.101710
H	6.515278	-2.217948	0.584139
H	5.727996	-3.911628	-1.042030
H	3.530805	-3.624253	-2.148349
H	2.138508	-1.676452	-1.646229

ωB97X energy = -1392.87361413 a.u.

(5*R*,1’*R*)-**5a**, Conf. D

C	1.194034	3.207342	0.666281
C	0.368570	4.311371	0.845140
C	0.008483	5.097688	-0.238753
C	0.472195	4.768067	-1.505990
C	1.285079	3.659239	-1.682851
C	1.663093	2.866357	-0.599272
C	2.600325	1.700724	-0.847268
C	2.645813	0.613410	0.224065
N	1.340328	-0.012339	0.446032
C	0.265189	0.061558	-0.287724
N	-0.772291	-0.576806	0.208704
C	-1.983185	-0.818995	-0.540631
C	-2.201771	-2.108662	-1.045023
C	-1.161919	-3.216773	-0.987759
C	-1.628167	-4.392202	-0.125979
C	-0.793802	-3.689466	-2.396952
C	-3.417602	-2.360979	-1.672054
C	-4.359325	-1.360698	-1.835383
C	-4.084813	-0.076678	-1.399803
C	-2.893041	0.227433	-0.748581
C	-2.629702	1.671867	-0.353023
C	-2.514583	2.553220	-1.600642
C	-3.699295	2.221864	0.592751
C	-0.410164	-1.210694	1.515498
C	-1.238895	-0.771649	2.744661
C	-2.711830	-1.153271	2.562847
C	-1.118706	0.726988	3.024702
C	-0.697386	-1.563655	3.942945
C	1.097807	-0.874526	1.607760
C	3.695124	-0.437675	-0.092441
C	3.459145	-1.433183	-1.035717
C	4.446097	-2.360727	-1.335797
C	5.678861	-2.298199	-0.698610
C	5.919048	-1.307028	0.242130
C	4.928538	-0.382762	0.545667
H	1.467703	2.618957	1.535501
H	0.011372	4.556280	1.839120
H	-0.632895	5.960229	-0.098778
H	0.193340	5.372479	-2.361704
H	1.638231	3.405214	-2.677540
H	2.382565	1.249727	-1.820411
H	3.622796	2.079096	-0.928980
H	2.915273	1.071778	1.179695
H	0.219939	0.588381	-1.232323
H	-0.245595	-2.815365	-0.551522
H	-2.516746	-4.860518	-0.556121
H	-0.845575	-5.151190	-0.065080
H	-1.879078	-4.079820	0.891027
H	0.005088	-4.432254	-2.343890
H	-0.448079	-2.858108	-3.014923
H	-1.646984	-4.152651	-2.897586
H	-3.621874	-3.353487	-2.057808
H	-5.301005	-1.576454	-2.326730
H	-4.812109	0.709312	-1.569183
H	-1.672503	1.729461	0.167800
H	-2.280050	3.580226	-1.313391
H	-1.724452	2.204087	-2.270095
H	-3.452181	2.562224	-2.161968
H	-3.438045	3.238449	0.896148
H	-3.797398	1.614831	1.495134
H	-4.676169	2.261626	0.105384
H	-0.543022	-2.287207	1.407692
H	-2.819485	-2.212694	2.312726
H	-3.248816	-0.976406	3.497250
H	-3.203070	-0.571959	1.783124
H	-1.454220	1.329177	2.178691
H	-1.742398	0.989166	3.881874
H	-0.095894	1.023919	3.269110
H	0.351388	-1.343043	4.153427
H	-0.793834	-2.640351	3.778966
H	-1.270626	-1.308038	4.836130
H	1.355317	-0.332673	2.517352
H	1.731053	-1.760043	1.540249
H	2.498705	-1.496913	-1.538689
H	4.251713	-3.134471	-2.069420
H	6.448479	-3.024360	-0.933091
H	6.876848	-1.254959	0.746591
H	5.116736	0.386513	1.287905

ωB97X energy = -1392.87329219 a.u.

(5*R*,1’*R*)-**5a**, Conf. E

C	-1.088673	-3.029725	-1.310577
C	-0.373628	-4.184694	-1.018023
C	-0.747352	-4.979232	0.055393
C	-1.842161	-4.615301	0.830565
C	-2.554998	-3.462536	0.533918
C	-2.184307	-2.653183	-0.539128
C	-2.945689	-1.388595	-0.856679

C	-2.783328	-0.300616	0.213085
N	-1.362413	-0.093128	0.539316
C	-0.438323	0.295790	-0.291578
N	0.778368	0.307213	0.210632
C	1.868412	0.969755	-0.470862
C	2.130625	2.312893	-0.169009
C	1.257744	3.151168	0.752321
C	2.027279	3.626678	1.986125
C	0.648136	4.334285	-0.004834
C	3.220941	2.913090	-0.791395
C	3.990837	2.220809	-1.709519
C	3.668270	0.916487	-2.040600
C	2.600500	0.262501	-1.433753
C	2.258214	-1.152253	-1.868183
C	1.718694	-1.166427	-3.301515
C	3.450132	-2.102130	-1.734420
C	0.720184	-0.026457	1.671223
C	1.736030	-1.045934	2.231834
C	3.165759	-0.514660	2.087251
C	1.629789	-2.426753	1.585229
C	1.424017	-1.166952	3.731053
C	-0.759306	-0.453053	1.826775
C	-3.444538	1.032712	-0.117181
C	-3.245810	2.111474	0.745967
C	-3.838414	3.337490	0.497368
C	-4.645548	3.505585	-0.622224
C	-4.850621	2.440354	-1.483038
C	-4.252899	1.208940	-1.233229
H	-0.797243	-2.420723	-2.162026
H	0.476056	-4.461728	-1.632909
H	-0.192716	-5.881385	0.286248
H	-2.146176	-5.235896	1.665826
H	-3.416820	-3.195978	1.138760
H	-2.617138	-1.003057	-1.825468
H	-4.011796	-1.607607	-0.947736
H	-3.219327	-0.671085	1.143908
H	-0.645148	0.606096	-1.309576
H	0.418061	2.544476	1.095752
H	2.854815	4.281425	1.702749
H	1.367402	4.189510	2.649554
H	2.443917	2.788680	2.550439
H	-0.032752	4.884639	0.648256
H	0.085942	3.995877	-0.878161
H	1.418926	5.029281	-0.345844
H	3.457519	3.947823	-0.569886
H	4.834295	2.707601	-2.185403
H	4.255996	0.395132	-2.787890
H	1.462443	-1.524491	-1.222126
H	1.447973	-2.185358	-3.589988
H	0.832198	-0.535960	-3.407389
H	2.470740	-0.806623	-4.008096
H	3.138210	-3.125766	-1.955276
H	3.870832	-2.086151	-0.726520
H	4.246689	-1.840502	-2.434700
H	0.888750	0.908973	2.210670
H	3.266630	0.481867	2.526624
H	3.853844	-1.180032	2.613154
H	3.487476	-0.459757	1.046803
H	1.852875	-2.399103	0.519204
H	2.351773	-3.098766	2.054508
H	0.642649	-2.878575	1.707338
H	0.430655	-1.583833	3.913945
H	1.486186	-0.195828	4.229695
H	2.150232	-1.832668	4.201101
H	-0.873153	-1.527419	1.980555
H	-1.261811	0.072751	2.637769
H	-2.619037	1.994446	1.624443
H	-3.673302	4.163066	1.180005
H	-5.111341	4.464178	-0.818952
H	-5.476901	2.560920	-2.359362
H	-4.429909	0.393895	-1.923783

ωB97X energy = -1392.87234090 a.u.

(5*R*,1’*R*)-**5a**, Conf. F

C	-4.420312	-2.433764	0.279793
C	-5.780209	-2.708491	0.301286
C	-6.632540	-2.073849	-0.593029
C	-6.116427	-1.166734	-1.508211
C	-4.754937	-0.893381	-1.526297
C	-3.894043	-1.522265	-0.632472
C	-2.418439	-1.205860	-0.621859
C	-2.068640	-0.233943	0.513163
N	-0.618179	-0.075634	0.655779
C	0.319615	-0.400260	-0.186991
N	1.538632	-0.101466	0.212112
C	2.669557	-0.128808	-0.688084
C	3.093920	1.078166	-1.261398
C	2.346752	2.392455	-1.096114
C	3.185788	3.433798	-0.353107
C	1.882496	2.929608	-2.452734
C	4.232889	1.049574	-2.060122
C	4.893924	-0.137656	-2.320960
C	4.409420	-1.326315	-1.804783
C	3.288348	-1.351774	-0.980708
C	2.770586	-2.692280	-0.487884
C	2.213355	-3.514982	-1.653689
C	3.840480	-3.483175	0.267483
C	1.487812	0.587184	1.539858
C	2.351662	-0.009061	2.674280
C	3.839358	0.099833	2.324374
C	2.000021	-1.462583	2.989390
C	2.093761	0.858624	3.914654
C	-0.034380	0.586800	1.827426
C	-2.723377	1.125203	0.345210
C	-2.300718	1.997016	-0.654575
C	-2.925670	3.222440	-0.826913
C	-3.982821	3.586832	-0.001575
C	-4.407276	2.722683	0.996792
C	-3.776242	1.497178	1.171168
H	-3.758941	-2.939309	0.977218
H	-6.174237	-3.422898	1.015175

H	-7.695084	-2.287883	-0.578190
H	-6.774832	-0.669458	-2.211412
H	-4.357239	-0.181181	-2.242533
H	-1.848816	-2.129258	-0.484932
H	-2.133148	-0.772069	-1.583799
H	-2.411758	-0.671561	1.454785
H	0.128466	-0.865271	-1.145429
H	1.440422	2.212437	-0.516077
H	2.609550	4.349125	-0.203403
H	3.509191	3.070612	0.625775
H	4.082002	3.691286	-0.922777
H	1.289900	3.836088	-2.311036
H	1.266002	2.196169	-2.977173
H	2.729771	3.181495	-3.094489
H	4.596901	1.970069	-2.502721
H	5.777729	-0.139460	-2.948447
H	4.911769	-2.255982	-2.046970
H	1.945880	-2.511313	0.201954
H	1.792971	-4.453149	-1.285147
H	1.427260	-2.976510	-2.188462
H	2.999555	-3.757285	-2.372982
H	3.408815	-4.400119	0.674696
H	4.258849	-2.908464	1.096834
H	4.662793	-3.768570	-0.392420
H	1.832845	1.611340	1.388191
H	4.116475	1.131376	2.089062
H	4.438052	-0.217808	3.180833
H	4.115906	-0.526641	1.475861
H	2.232972	-2.127127	2.157410
H	2.584524	-1.798083	3.848654
H	0.946132	-1.593787	3.244764
H	1.064775	0.779634	4.272500
H	2.306193	1.912216	3.713599
H	2.747398	0.534151	4.726446
H	-0.296278	0.019993	2.720838
H	-0.443866	1.592999	1.920258
H	-1.477184	1.725120	-1.309258
H	-2.587889	3.894980	-1.606877
H	-4.471558	4.544732	-0.136438
H	-5.228659	3.002274	1.646295
H	-4.109073	0.823506	1.954347

ωB97X energy = -1392.87230332 a.u.

(5*R*,1'*R*)-**5a**, Conf. G

C	3.040516	-1.694564	1.217904
C	3.399207	-2.989583	0.863745
C	4.336955	-3.202403	-0.135869
C	4.920059	-2.114172	-0.774652
C	4.562211	-0.823051	-0.416212
C	3.614251	-0.598573	0.581295
C	3.199699	0.806858	0.940825
C	2.336885	1.464570	-0.141049
N	1.157055	0.635822	-0.451931
C	0.106689	0.501620	0.305732
N	-0.807912	-0.320239	-0.167363
C	-2.151612	-0.332195	0.368955
C	-3.093952	0.554734	-0.169889
C	-2.749077	1.625558	-1.193961
C	-3.520646	1.435350	-2.500841
C	-2.984881	3.023191	-0.614048
C	-4.399011	0.477758	0.306368
C	-4.743531	-0.413907	1.306853
C	-3.777215	-1.227206	1.872297
C	-2.460280	-1.201066	1.423313
C	-1.423958	-2.061422	2.125632
C	-1.194919	-1.564109	3.556260
C	-1.804541	-3.543112	2.125147
C	-0.417696	-0.748731	-1.550245
C	-0.594139	-2.232792	-1.936000
C	-2.075382	-2.623052	-1.894361
C	0.219699	-3.186730	-1.062180
C	-0.115246	-2.350113	-3.390533
C	1.039549	-0.234789	-1.627465
C	1.890523	2.888732	0.162031
C	2.133752	3.512411	1.378898
C	1.687917	4.811338	1.604937
C	0.996802	5.495202	0.618929
C	0.753130	4.878901	-0.604010
C	1.198569	3.588090	-0.828274
H	2.308941	-1.536191	2.005134
H	2.941647	-3.832605	1.369180
H	4.617334	-4.211833	-0.413938
H	5.660473	-2.272344	-1.550536
H	5.029468	0.021503	-0.913928
H	2.652864	0.795094	1.887226
H	4.083518	1.432390	1.085551
H	2.907774	1.481315	-1.072363
H	-0.022852	1.038601	1.238564
H	-1.684454	1.568477	-1.426372
H	-3.215094	2.186331	-3.232182
H	-3.345222	0.447226	-2.933644
H	-4.596029	1.542604	-2.340368
H	-2.667148	3.783223	-1.331515
H	-2.419218	3.168782	0.309402
H	-4.042016	3.190068	-0.394591
H	-5.153945	1.142320	-0.098936
H	-5.765544	-0.458162	1.665249
H	-4.049676	-1.893202	2.683343
H	-0.476661	-1.958386	1.594842
H	-0.411312	-2.153096	4.038203
H	-0.892170	-0.514592	3.573755
H	-2.104649	-1.660619	4.154152
H	-1.004266	-4.134023	2.576317
H	-1.973326	-3.918915	1.113557
H	-2.713906	-3.719226	2.704284
H	-1.042577	-0.173170	-2.238996
H	-2.684744	-1.931311	-2.483052
H	-2.199198	-3.620495	-2.321547
H	-2.474297	-2.644842	-0.879857
H	-0.038399	-3.097826	-0.006874
H	0.015837	-4.215798	-1.366315

H	1.295832	-3.028655	-1.158376
H	0.947593	-2.119283	-3.495906
H	-0.679165	-1.683445	-4.048689
H	-0.264825	-3.372765	-3.741915
H	1.772618	-1.039381	-1.549149
H	1.233408	0.333746	-2.535847
H	2.672164	2.999905	2.166697
H	1.883551	5.284244	2.560266
H	0.648714	6.505887	0.797751
H	0.218375	5.407973	-1.384389
H	1.004617	3.114924	-1.786336

ωB97X energy = -1392.87189851 a.u.

(5*R*,1’*R*)-**5a**, Conf. H

C	-5.319297	-1.287417	-0.693833
C	-6.585512	-1.455889	-0.148044
C	-6.733849	-2.046054	1.098937
C	-5.609476	-2.470889	1.795601
C	-4.346836	-2.302557	1.246417
C	-4.186426	-1.706551	-0.003242
C	-2.803504	-1.515762	-0.578427
C	-2.031936	-0.409762	0.150870
N	-0.628990	-0.392566	-0.292033
C	0.414300	-0.463957	0.485621
N	1.560905	-0.251296	-0.123926
C	2.844278	-0.479426	0.497185
C	3.617508	-1.562033	0.054671
C	3.124016	-2.590184	-0.951613
C	3.917675	-2.527249	-2.258924
C	3.169982	-4.001882	-0.360296
C	4.887095	-1.718669	0.602263
C	5.353246	-0.865946	1.586545
C	4.542660	0.151294	2.057212
C	3.274546	0.369798	1.528090
C	2.423509	1.479416	2.125569
C	2.038490	1.142502	3.569526
C	3.114497	2.842778	2.056965
C	1.314735	0.098984	-1.558907
C	1.728560	1.525907	-1.991063
C	3.238782	1.720236	-1.820764
C	0.973724	2.614002	-1.224709
C	1.409727	1.641983	-3.488510
C	-0.201117	-0.188015	-1.681222
C	-2.615749	0.987987	0.030251
C	-3.144379	1.472449	-1.163241
C	-3.608811	2.777936	-1.248426
C	-3.555728	3.611643	-0.139948
C	-3.037224	3.134368	1.055918
C	-2.569818	1.830606	1.137392
H	-5.212492	-0.826224	-1.670336
H	-7.457905	-1.125402	-0.700370
H	-7.721257	-2.178569	1.526036
H	-5.716928	-2.940067	2.766983
H	-3.474482	-2.648927	1.792889
H	-2.230447	-2.441006	-0.477870
H	-2.872062	-1.297941	-1.646203
H	-1.995169	-0.666826	1.211510
H	0.344687	-0.688004	1.543385
H	2.075604	-2.387256	-1.175128
H	3.521798	-3.246777	-2.978699
H	3.879264	-1.534554	-2.714692
H	4.969147	-2.769006	-2.085452
H	2.736791	-4.714506	-1.065389
H	2.607032	-4.058354	0.573485
H	4.195930	-4.317023	-0.157740
H	5.514054	-2.536548	0.265314
H	6.343308	-1.007778	2.004043
H	4.902884	0.793398	2.852998
H	1.497372	1.559375	1.555850
H	1.371768	1.908727	3.970648
H	1.530775	0.177437	3.638736
H	2.923565	1.097324	4.208669
H	2.446197	3.617548	2.438909
H	3.388067	3.106903	1.033207
H	4.022912	2.859015	2.663252
H	1.875535	-0.601129	-2.176672
H	3.800945	0.937676	-2.338383
H	3.531711	2.680040	-2.251641
H	3.545763	1.718907	-0.775293
H	1.192732	2.588396	-0.156301
H	1.276768	3.595494	-1.595211
H	-0.110392	2.541614	-1.351466
H	0.341286	1.560515	-3.699272
H	1.931084	0.872905	-4.064783
H	1.740354	2.615799	-3.854435
H	-0.748865	0.644705	-2.117552
H	-0.409032	-1.087887	-2.260858
H	-3.204521	0.836723	-2.039898
H	-4.017374	3.142391	-2.183722
H	-3.922697	4.629224	-0.207161
H	-2.998719	3.776534	1.928150
H	-2.164396	1.462281	2.074986

ωB97X energy = -1392.87141632 a.u.

(5*R*,1’*R*)-**5a**, Conf. I

C	-5.199824	-0.341391	-0.979190
C	-6.541163	-0.161050	-0.668557
C	-7.096510	-0.812561	0.423882
C	-6.304497	-1.649269	1.199820
C	-4.965380	-1.830370	0.883759
C	-4.397606	-1.176090	-0.207314
C	-2.934010	-1.352969	-0.527728
C	-2.042122	-0.596810	0.469888
N	-0.620129	-0.809366	0.131664
C	0.349146	0.028985	0.379724
N	1.547262	-0.414603	0.074444
C	2.751346	0.322534	0.374104
C	3.620062	-0.185421	1.349566
C	3.300401	-1.402173	2.204069
C	4.249465	-2.566603	1.911943

C	3.324075	-1.046187	3.693203
C	4.813381	0.494683	1.571545
C	5.108690	1.663390	0.893084
C	4.200138	2.184423	-0.010823
C	3.005176	1.530565	-0.294185
C	2.041316	2.177041	-1.277806
C	1.451487	3.465259	-0.695368
C	2.696196	2.448966	-2.633777
C	1.447884	-1.786983	-0.512912
C	1.902333	-1.936351	-1.985333
C	3.381555	-1.565282	-2.132057
C	1.062104	-1.096594	-2.948552
C	1.756848	-3.422706	-2.341229
C	-0.049189	-2.103097	-0.271280
C	-2.350531	0.884778	0.520948
C	-2.208511	1.682294	-0.614039
C	-2.503835	3.035043	-0.561369
C	-2.953345	3.604286	0.624877
C	-3.101061	2.815625	1.755428
C	-2.796520	1.460290	1.703401
H	-4.770720	0.172303	-1.833592
H	-7.153426	0.489693	-1.282570
H	-8.143391	-0.672239	0.667503
H	-6.732026	-2.165962	2.051659
H	-4.355548	-2.493321	1.490339
H	-2.686006	-2.416321	-0.493223
H	-2.727534	-0.998358	-1.540586
H	-2.203874	-1.023887	1.464439
H	0.185655	1.008040	0.810790
H	2.282195	-1.725814	1.983948
H	3.974509	-3.438728	2.508871
H	4.228880	-2.856555	0.858455
H	5.279748	-2.300359	2.160612
H	3.010631	-1.908097	4.286164
H	2.648881	-0.216610	3.912322
H	4.327050	-0.764163	4.021236
H	5.513884	0.114253	2.306405
H	6.041422	2.181179	1.084317
H	4.424706	3.119523	-0.511343
H	1.208999	1.495767	-1.456118
H	0.721686	3.891284	-1.387680
H	0.947755	3.287217	0.258415
H	2.232067	4.210616	-0.524640
H	1.953091	2.834577	-3.335141
H	3.128748	1.542602	-3.063227
H	3.490460	3.193929	-2.549667
H	2.069638	-2.453660	0.084411
H	4.005808	-2.133099	-1.436562
H	3.714686	-1.800941	-3.145099
H	3.565080	-0.504742	-1.961270
H	1.163538	-0.028379	-2.753163
H	1.399648	-1.271935	-3.972136
H	0.000297	-1.351756	-2.911644
H	0.721955	-3.768433	-2.297403
H	2.353890	-4.048633	-1.672734
H	2.112609	-3.587148	-3.360071
H	-0.540054	-2.471151	-1.168170
H	-0.198013	-2.826799	0.531927
H	-1.859999	1.247403	-1.546213
H	-2.386347	3.646632	-1.448461
H	-3.185634	4.662123	0.665492
H	-3.451124	3.253350	2.682959
H	-2.908761	0.846851	2.591365

ωB97X energy = -1392.87128947 a.u.

(5*R*,1’*S*)-**5b**, Conf. A

C	1.524867	3.519382	-1.502836
C	0.500165	4.426147	-1.729859
C	-0.334356	4.811687	-0.687472
C	-0.137751	4.284427	0.580069
C	0.893254	3.379713	0.803671
C	1.735324	2.986287	-0.231336
C	2.848359	1.997570	0.012801
C	2.577997	0.628497	-0.623818
N	1.358298	0.025280	-0.068170
C	0.189592	0.010388	-0.644143
N	-0.771723	-0.516547	0.083942
C	-2.059930	-0.870290	-0.467728
C	-3.038345	0.121881	-0.617182
C	-2.786114	1.599992	-0.369770
C	-2.864783	2.377598	-1.687162
C	-3.753009	2.189598	0.658921
C	-4.295659	-0.275857	-1.062763
C	-4.568392	-1.600058	-1.355251
C	-3.566058	-2.549022	-1.259632
C	-2.285351	-2.202955	-0.840721
C	-1.196104	-3.264663	-0.863205
C	-1.476250	-4.393755	0.130584
C	-1.013572	-3.823117	-2.277492
C	-0.207818	-1.025012	1.374422
C	-0.932335	-0.619098	2.677515
C	-0.954904	0.893099	2.899994
C	-0.161918	-1.290774	3.823011
C	-2.365434	-1.162535	2.693011
C	1.262233	-0.542943	1.281103
C	3.722366	-0.366314	-0.519257
C	3.759462	-1.434921	-1.413289
C	4.768549	-2.381950	-1.341359
C	5.757072	-2.270972	-0.370908
C	5.728343	-1.210213	0.520923
C	4.715411	-0.261366	0.447599
H	2.179635	3.233988	-2.320938
H	0.355889	4.839237	-2.721718
H	-1.131737	5.524706	-0.863906
H	-0.782027	4.581175	1.400406
H	1.053243	2.987012	1.802796
H	3.782988	2.371120	-0.412248
H	3.009615	1.889936	1.087490
H	2.364559	0.781366	-1.684089
H	0.024230	0.383378	-1.648092

H	-1.774210	1.726667	0.019659
H	-2.651273	3.433908	-1.512544
H	-2.144135	2.003626	-2.418757
H	-3.861590	2.300560	-2.128425
H	-3.486326	3.229027	0.865540
H	-3.728571	1.639719	1.602338
H	-4.781045	2.178386	0.289352
H	-5.076092	0.467069	-1.182319
H	-5.559954	-1.889584	-1.683751
H	-3.776747	-3.576717	-1.533776
H	-0.245944	-2.801098	-0.592446
H	-0.661277	-5.120271	0.117478
H	-1.583230	-4.021625	1.152260
H	-2.398560	-4.919569	-0.128057
H	-0.184616	-4.533922	-2.293027
H	-0.795926	-3.027202	-2.992706
H	-1.909402	-4.347892	-2.616892
H	-0.235261	-2.115303	1.325957
H	-1.394661	1.425508	2.055174
H	-1.553606	1.120722	3.784402
H	0.042481	1.300789	3.077119
H	-0.147233	-2.377653	3.705121
H	0.870366	-0.939074	3.890859
H	-0.649128	-1.062740	4.772901
H	-3.009307	-0.666443	1.966479
H	-2.798973	-1.005167	3.682917
H	-2.387763	-2.236287	2.488003
H	1.492863	0.228853	2.016698
H	1.978169	-1.355921	1.393283
H	2.992181	-1.524003	-2.176494
H	4.787102	-3.204421	-2.046957
H	6.548233	-3.009555	-0.314057
H	6.496609	-1.115965	1.279494
H	4.714942	0.559823	1.154406

ωB97X energy = -1392.87521777 a.u.

(5*R*,1’*S*)-**5b**, Conf. B

C	1.564421	3.445300	-1.505488
C	0.579477	4.391721	-1.745120
C	-0.205737	4.862157	-0.699273
C	0.001208	4.379648	0.584128
C	0.992364	3.434162	0.820309
C	1.783663	2.953943	-0.218211
C	2.863492	1.930912	0.038964
C	2.577804	0.574384	-0.617335
N	1.351937	-0.026087	-0.070894
C	0.178717	-0.000405	-0.636969
N	-0.785136	-0.538378	0.079164
C	-2.091082	-0.835315	-0.462849
C	-3.029995	0.196423	-0.600183
C	-2.717720	1.662428	-0.347047
C	-2.773515	2.449629	-1.659953
C	-3.652645	2.285491	0.691478
C	-4.306067	-0.147969	-1.036446
C	-4.635113	-1.458728	-1.331948
C	-3.671532	-2.447909	-1.247908
C	-2.374575	-2.156286	-0.837889
C	-1.331227	-3.262476	-0.870095
C	-1.647116	-4.375755	0.131097
C	-1.187904	-3.833034	-2.283982
C	-0.227458	-1.079148	1.358680
C	-0.909144	-0.628348	2.670581
C	-0.835402	0.882801	2.887237
C	-0.168806	-1.345190	3.808484
C	-2.372383	-1.083053	2.704496
C	1.266153	-0.679345	1.240726
C	3.712516	-0.432356	-0.526835
C	3.750949	-1.478359	-1.446791
C	4.751551	-2.435408	-1.387197
C	5.729765	-2.356757	-0.403377
C	5.699172	-1.318601	0.514788
C	4.694680	-0.360206	0.454068
H	2.181564	3.096110	-2.328135
H	0.428007	4.769189	-2.750053
H	-0.972890	5.605135	-0.885788
H	-0.603480	4.742939	1.407686
H	1.161265	3.078666	1.831520
H	3.815938	2.286609	-0.361996
H	2.999923	1.808304	1.115638
H	2.367264	0.743393	-1.675665
H	0.011165	0.412302	-1.624839
H	-1.699511	1.747323	0.037326
H	-2.526999	3.497621	-1.479547
H	-2.064956	2.058228	-2.394399
H	-3.772542	2.405863	-2.100637
H	-3.342111	3.312264	0.900121
H	-3.642847	1.731273	1.632603
H	-4.683129	2.317588	0.330062
H	-5.056992	0.626241	-1.145513
H	-5.640494	-1.705960	-1.652837
H	-3.925655	-3.465092	-1.524438
H	-0.359961	-2.837423	-0.612154
H	-0.860434	-5.132730	0.116020
H	-1.733382	-3.995172	1.151726
H	-2.590621	-4.867098	-0.118774
H	-0.386736	-4.574798	-2.306393
H	-0.949032	-3.048818	-3.005241
H	-2.106884	-4.325228	-2.610099
H	-0.317261	-2.165709	1.315079
H	-1.270581	1.438408	2.054933
H	-1.390688	1.147902	3.789421
H	0.189681	1.231188	3.028845
H	-0.230840	-2.430940	3.695734
H	0.886237	-1.066500	3.860936
H	-0.625911	-1.080676	4.763837
H	-2.993844	-0.546869	1.987291
H	-2.782820	-0.901273	3.700097
H	-2.463680	-2.152956	2.497994
H	1.574208	0.018453	2.019386
H	1.932221	-1.541015	1.264978
H	2.990992	-1.541438	-2.219775

H	4.771490	-3.240302	-2.112721
H	6.514523	-3.102813	-0.356349
H	6.459592	-1.249847	1.283909
H	4.691550	0.442819	1.181483

ωB97X energy = -1392.87510317 a.u.

(5*R*,1'*S*)-**5b**, Conf. C

C	-4.387035	2.600430	0.428926
C	-5.744505	2.885808	0.462250
C	-6.605945	2.277233	-0.441449
C	-6.101260	1.384833	-1.377206
C	-4.742156	1.100768	-1.407184
C	-3.872070	1.704011	-0.504565
C	-2.398526	1.377597	-0.508834
C	-2.048747	0.380321	0.603270
N	-0.598062	0.209530	0.733163
C	0.335578	0.521748	-0.119595
N	1.543073	0.128414	0.227530
C	2.734470	0.571774	-0.459806
C	3.056592	0.015482	-1.706180
C	2.164477	-0.965615	-2.448614
C	1.576680	-0.314653	-3.704173
C	2.898672	-2.259491	-2.806074
C	4.244133	0.421901	-2.306812
C	5.078434	1.342190	-1.698174
C	4.712859	1.913231	-0.492308
C	3.524363	1.562332	0.140443
C	3.123526	2.300470	1.408038
C	4.085539	2.012430	2.562764
C	3.018538	3.806570	1.152516
C	1.493239	-0.583033	1.543373
C	2.054843	-2.023207	1.583500
C	3.550830	-2.027354	1.251807
C	1.316154	-2.976494	0.643385
C	1.892998	-2.507231	3.031754
C	-0.012930	-0.478944	1.889161
C	-2.716042	-0.970308	0.416096
C	-2.331712	-1.812028	-0.624064
C	-2.961901	-3.033028	-0.808184
C	-3.986672	-3.422787	0.045913
C	-4.373871	-2.588376	1.083927
C	-3.737227	-1.367359	1.269743
H	-3.718475	3.085746	1.133853
H	-6.129448	3.588668	1.192375
H	-7.666560	2.499805	-0.417546
H	-6.766803	0.908118	-2.087893
H	-4.353560	0.400677	-2.140161
H	-2.121438	0.962290	-1.481466
H	-1.822361	2.294176	-0.356248
H	-2.379416	0.801462	1.556629
H	0.150721	1.070145	-1.034179
H	1.328498	-1.233903	-1.801956
H	2.365426	-0.053133	-4.413852
H	0.891375	-1.004364	-4.201423
H	1.028151	0.599942	-3.466397
H	2.204163	-2.968221	-3.262380
H	3.336147	-2.733654	-1.924767
H	3.702144	-2.075033	-3.522493
H	4.520317	0.006491	-3.269359
H	6.006740	1.632152	-2.176578
H	5.353518	2.663517	-0.042693
H	2.126683	1.971396	1.705380
H	3.745029	2.511339	3.472427
H	4.164935	0.942561	2.771124
H	5.088998	2.379909	2.334498
H	2.652899	4.310984	2.049463
H	2.328372	4.021609	0.334142
H	3.989799	4.238036	0.900430
H	2.076156	0.003965	2.254156
H	4.102118	-1.330523	1.889274
H	3.957381	-3.026384	1.423170
H	3.749524	-1.763690	0.212962
H	0.247422	-3.042238	0.862377
H	1.430100	-2.687888	-0.401985
H	1.729750	-3.981444	0.751029
H	2.407612	-1.842018	3.730494
H	2.329846	-3.502374	3.133511
H	0.845896	-2.577562	3.334461
H	-0.486553	-1.453565	2.002621
H	-0.200113	0.105690	2.789862
H	-1.534214	-1.519511	-1.301668
H	-2.653796	-3.681969	-1.619721
H	-4.479547	-4.377258	-0.097888
H	-5.170068	-2.887785	1.755539
H	-4.040395	-0.717102	2.084166

ωB97X energy = -1392.87282190 a.u.

(5*R*,1'*S*)-**5b**, Conf. D

C	4.525245	-1.176015	0.310267
C	4.747051	-2.491739	0.690705
C	4.007434	-3.518338	0.116193
C	3.055968	-3.221608	-0.849639
C	2.839793	-1.903228	-1.231207
C	3.562771	-0.866131	-0.648478
C	3.264293	0.569222	-1.002173
C	2.447106	1.281506	0.084411
N	1.230029	0.511922	0.407159
C	0.122460	0.523079	-0.277081
N	-0.824983	-0.250837	0.205629
C	-2.028573	-0.550766	-0.533599
C	-3.038719	0.417157	-0.623096
C	-2.903442	1.823755	-0.063022
C	-2.793553	2.848268	-1.196557
C	-4.051404	2.183370	0.881988
C	-4.203141	0.071670	-1.302043
C	-4.358266	-1.180820	-1.869360
C	-3.325155	-2.099377	-1.810345
C	-2.129100	-1.797020	-1.167066
C	-0.985177	-2.797664	-1.234974
C	-1.315162	-4.096968	-0.498234

C	-0.595839	-3.079580	-2.688858
C	-0.337854	-0.950826	1.435655
C	-1.173083	-0.762349	2.723317
C	-1.265914	0.698508	3.164068
C	-0.470449	-1.582079	3.815027
C	-2.581408	-1.336497	2.537975
C	1.117541	-0.424050	1.533563
C	2.074746	2.723915	-0.227289
C	1.504888	3.493654	0.788361
C	1.121664	4.802725	0.554413
C	1.307312	5.367266	-0.703159
C	1.878507	4.613560	-1.714774
C	2.260179	3.296125	-1.479421
H	5.105406	-0.379092	0.765703
H	5.499383	-2.717299	1.437808
H	4.178511	-4.546078	0.414714
H	2.482201	-4.018619	-1.310579
H	2.100556	-1.678525	-1.995507
H	4.192648	1.128628	-1.139202
H	2.721829	0.601154	-1.949988
H	3.027314	1.275412	1.010397
H	-0.010786	1.116576	-1.174045
H	-1.977842	1.882308	0.510623
H	-2.639288	3.848568	-0.785743
H	-1.959589	2.622937	-1.866268
H	-3.706603	2.866132	-1.796870
H	-3.875345	3.165170	1.326835
H	-4.149899	1.457020	1.692000
H	-5.005307	2.226642	0.351617
H	-5.003132	0.798321	-1.387726
H	-5.280071	-1.433653	-2.380336
H	-3.440500	-3.062616	-2.294691
H	-0.103863	-2.358916	-0.764360
H	-0.461069	-4.777331	-0.531128
H	-1.565598	-3.917840	0.550295
H	-2.165851	-4.602924	-0.961064
H	0.275339	-3.738789	-2.721870
H	-0.347166	-2.156904	-3.218123
H	-1.405162	-3.573362	-3.231300
H	-0.326803	-2.020381	1.221780
H	-1.780714	1.314750	2.426364
H	-1.833786	0.760047	4.094817
H	-0.286355	1.142691	3.355088
H	-0.391118	-2.634540	3.529380
H	0.533364	-1.211517	4.034507
H	-1.048192	-1.527823	4.739594
H	-3.176737	-0.761567	1.828263
H	-3.105353	-1.325357	3.496077
H	-2.545945	-2.372687	2.189936
H	1.309026	0.110124	2.463800
H	1.856280	-1.218807	1.429438
H	1.360240	3.062344	1.774298
H	0.682336	5.386772	1.354728
H	1.010632	6.393035	-0.888511
H	2.030648	5.046586	-2.696522
H	2.706721	2.729442	-2.287166

ωB97X energy = -1392.87275048 a.u.

(5*S*,1'*S*)-**8a**, Conf. A

C	-3.564335	-0.788265	0.661573
C	-4.664802	-1.582070	0.362739
C	-5.635544	-1.110238	-0.513566
C	-5.516780	0.141365	-1.089458
C	-4.411402	0.928784	-0.787076
C	-3.427313	0.485194	0.082433
C	-2.194400	1.301028	0.418240
C	-2.253814	2.775915	0.007243
C	-1.014249	3.529989	0.421662
C	-0.088687	3.954880	-0.526973
C	1.065586	4.626081	-0.143426
C	1.310663	4.877128	1.198775
C	0.391911	4.460236	2.153978
C	-0.761488	3.793223	1.766848
N	-1.014963	0.656647	-0.173263
C	0.009886	0.198322	0.484711
N	0.890390	-0.437262	-0.259358
C	2.199554	-0.823332	0.209756
C	3.311992	-0.116891	-0.269110
C	3.208715	1.135819	-1.126098
C	3.887564	2.321668	-0.434986
C	3.793727	0.918938	-2.523745
C	4.574887	-0.565791	0.104874
C	4.728530	-1.637701	0.965176
C	3.612902	-2.265401	1.489300
C	2.326717	-1.876151	1.128251
C	1.147544	-2.580187	1.781868
C	1.174370	-4.093339	1.555972
C	1.102815	-2.258752	3.279268
C	0.404205	-0.504737	-1.673398
C	0.131638	-1.922826	-2.228033
C	-0.946971	-2.662939	-1.434779
C	1.422194	-2.748001	-2.246222
C	-0.330406	-1.745978	-3.681163
C	-0.845800	0.406279	-1.607781
C	-2.671914	-2.434404	2.140825
O	-2.566798	-1.166932	1.504698
H	-4.776935	-2.563254	0.803680
H	-6.490201	-1.736984	-0.740710
H	-6.273605	0.508368	-1.771618
H	-4.325529	1.907406	-1.243386
H	-2.036373	1.249491	1.497488
H	-2.390204	2.865644	-1.072743
H	-3.138142	3.212204	0.477362
H	-0.276933	3.772358	-1.580827
H	1.773263	4.954348	-0.896330
H	2.211462	5.399438	1.500096
H	0.571653	4.659595	3.204329
H	-1.478711	3.480946	2.520103
H	0.134396	0.337258	1.551644
H	2.155994	1.405950	-1.231355

H	3.756264	3.226140	-1.033094
H	3.455349	2.503801	0.551452
H	4.959951	2.153639	-0.313808
H	4.864577	0.708541	-2.467175
H	3.659920	1.815227	-3.132941
H	3.319679	0.081116	-3.041395
H	5.453691	-0.052092	-0.268370
H	5.720962	-1.970309	1.246669
H	3.742659	-3.078412	2.194656
H	0.224206	-2.202328	1.339766
H	0.275767	-4.549700	1.977865
H	1.212965	-4.343676	0.493662
H	2.038390	-4.552645	2.041385
H	0.250793	-2.752619	3.752503
H	1.017516	-1.184646	3.460080
H	2.007452	-2.610449	3.780528
H	1.160445	-0.049202	-2.311957
H	-0.649447	-2.825413	-0.397204
H	-1.119598	-3.643647	-1.882890
H	-1.904213	-2.134149	-1.434683
H	2.220476	-2.225982	-2.781577
H	1.784560	-2.978171	-1.244760
H	1.240629	-3.694830	-2.759189
H	0.420318	-1.213137	-4.271244
H	-0.480011	-2.726732	-4.136508
H	-1.274543	-1.202822	-3.759420
H	-0.696162	1.352308	-2.130062
H	-1.739713	-0.071290	-2.005207
H	-3.581017	-2.498864	2.743009
H	-1.803881	-2.515653	2.791028
H	-2.656699	-3.244083	1.405847

ωB97X energy = -1507.41562724 a.u.

(5*S*,1'*S*)-**8a**, Conf. B

C	-3.470734	1.061422	0.823228
C	-4.498927	1.990679	0.925287
C	-5.578735	1.926898	0.052474
C	-5.640971	0.946104	-0.920920
C	-4.607460	0.022223	-1.016591
C	-3.519000	0.057477	-0.156912
C	-2.438052	-0.997853	-0.286857
C	-2.367220	-1.964764	0.902808
C	-1.337377	-3.056424	0.721393
C	-0.303754	-3.213872	1.639012
C	0.658785	-4.202223	1.467380
C	0.599268	-5.047920	0.370396
C	-0.434814	-4.906911	-0.547612
C	-1.394548	-3.921751	-0.371322
N	-1.130358	-0.410235	-0.607845
C	-0.196698	-0.017388	0.211807
N	0.868599	0.477920	-0.383669
C	1.905828	1.202500	0.312330
C	1.950618	2.596120	0.161231
C	0.866450	3.395660	-0.545013
C	0.216152	4.389703	0.421575
C	1.400067	4.115962	-1.785004
C	3.015749	3.278171	0.740797
C	3.972598	2.611421	1.484904
C	3.859139	1.248019	1.689223
C	2.826168	0.512992	1.115158
C	2.728515	-0.969920	1.434902
C	3.985908	-1.742629	1.031578
C	2.431797	-1.171816	2.924479
C	0.708100	0.395758	-1.869155
C	1.723392	-0.509624	-2.605164
C	1.367016	-0.456388	-4.097327
C	1.670073	-1.958308	-2.117515
C	3.141847	0.046647	-2.446672
C	-0.762134	-0.069397	-1.985297
C	-2.286162	2.056771	2.648347
O	-2.379633	1.061051	1.636138
H	-4.470747	2.764954	1.679817
H	-6.373457	2.658284	0.143247
H	-6.480197	0.897328	-1.603709
H	-4.647173	-0.750130	-1.778132
H	-2.683066	-1.590164	-1.170821
H	-2.180656	-1.415620	1.826990
H	-3.364659	-2.401100	0.997115
H	-0.254398	-2.564313	2.508313
H	1.454528	-4.313118	2.196205
H	1.349281	-5.818308	0.233582
H	-0.495642	-5.570874	-1.402480
H	-2.201029	-3.832307	-1.092887
H	-0.289747	-0.071429	1.286742
H	0.075290	2.713403	-0.859887
H	0.925949	5.154192	0.745221
H	-0.620169	4.894396	-0.067101
H	-0.162668	3.882463	1.311854
H	1.857309	3.422778	-2.495584
H	2.156859	4.854464	-1.509770
H	0.589822	4.640850	-2.295421
H	3.085960	4.353799	0.623405
H	4.795490	3.159730	1.928622
H	4.589443	0.740005	2.308988
H	1.893063	-1.397453	0.879211
H	3.841684	-2.809109	1.221210
H	4.217651	-1.616885	-0.028018
H	4.854702	-1.416424	1.607899
H	2.316582	-2.235176	3.146681
H	1.515903	-0.658382	3.228335
H	3.247699	-0.789071	3.542075
H	0.817640	1.400632	-2.276473
H	0.386244	-0.885653	-4.311589
H	2.102957	-1.027485	-4.666510
H	1.379265	0.571572	-4.469912
H	1.944550	-2.047236	-1.064832
H	0.681312	-2.406938	-2.244545
H	2.375744	-2.562948	-2.691302
H	3.494755	0.006225	-1.416647
H	3.832388	-0.538753	-3.057741
H	3.198408	1.086136	-2.782132

H	-1.425444	0.714926	-2.354987
H	-0.876243	-0.948531	-2.617760
H	-2.292411	3.059429	2.213747
H	-1.336747	1.884421	3.150298
H	-3.101962	1.961192	3.368491

ωB97X energy = -1507.41347711 a.u.

(5*S*,1'*S*)-**8a**, Conf. C

C	1.785019	3.114532	0.207898
C	1.572842	4.437705	-0.154470
C	1.834367	4.841416	-1.459581
C	2.304758	3.939439	-2.395831
C	2.516350	2.615355	-2.024383
C	2.262619	2.184181	-0.732277
C	2.477996	0.754434	-0.274100
C	3.187485	-0.155737	-1.281923
C	3.399185	-1.547082	-0.738042
C	2.602318	-2.607706	-1.158130
C	2.770851	-3.878114	-0.620586
C	3.740181	-4.101607	0.346410
C	4.545471	-3.050206	0.768225
C	4.375954	-1.784066	0.227993
C	1.192720	0.166988	0.147503
N	0.089764	0.177691	-0.538631
C	-0.944000	-0.362404	0.078407
N	-2.293604	-0.131686	-0.385253
C	-2.974931	1.002490	0.078008
C	-2.321099	2.088488	0.920459
C	-2.299183	3.415313	0.155011
C	-3.004101	2.250142	2.279592
C	-4.301134	1.162702	-0.311678
C	-4.909700	0.258661	-1.164660
C	-4.189438	-0.809111	-1.670491
C	-2.865951	-1.025660	-1.299121
C	-2.097196	-2.170098	-1.936246
C	-2.789865	-3.518836	-1.732723
C	-1.871413	-1.899477	-3.426933
C	-0.557894	-0.694487	1.488173
C	-0.997289	-2.058689	2.061194
C	-2.525493	-2.132862	2.144411
C	-0.441642	-2.117168	3.491560
C	-0.463422	-3.250053	1.266797
C	0.973719	-0.479837	1.444128
C	1.122156	3.519755	2.468883
C	1.552518	2.622719	1.453095
O	1.208153	5.158697	0.564941
H	1.665652	5.876128	-1.734645
H	2.509019	4.255689	-3.411268
H	2.889154	1.916120	-2.763012
H	3.069064	0.782243	0.644116
H	2.608014	-0.207223	-2.207135
H	4.148612	0.302807	-1.525165
H	1.842610	-2.441914	-1.916744
H	2.140525	-4.693209	-0.958126
H	3.872421	-5.091459	0.767761
H	5.310521	-3.218618	1.517608
H	5.013611	-0.969305	0.558254
H	0.008720	0.619231	-1.525280
H	-1.277694	1.822219	1.102363
H	-1.780508	4.179999	0.738845
H	-1.782052	3.311318	-0.801864
H	-3.310616	3.777680	-0.042257
H	-3.012584	1.312993	2.841796
H	-4.039725	2.577761	2.161719
H	-2.482769	3.000675	2.878105
H	-4.860705	2.021456	0.042095
H	-5.943727	0.402019	-1.456392
H	-4.662419	-1.486629	-2.372559
H	-1.114081	-2.222912	-1.466513
H	-2.167260	-4.322730	-2.131725
H	-2.973158	-3.725129	-0.675867
H	-3.749803	-3.554062	-2.252931
H	-1.277811	-2.702493	-3.869578
H	-1.344278	-0.956553	-3.588778
H	-2.822316	-1.848579	-3.963346
H	-1.014676	0.069267	2.123923
H	-2.996070	-2.160011	1.161155
H	-2.933915	-1.278113	2.691091
H	-2.816978	-3.040250	2.677777
H	0.650879	-2.113965	3.509802
H	-0.798040	-1.274678	4.090726
H	-0.775522	-3.037344	3.974723
H	-0.808228	-4.175869	1.732721
H	-0.820153	-3.247922	0.236744
H	0.627825	-3.285055	1.244534
H	1.327995	0.176160	2.237518
H	1.529095	-1.418851	1.473546
H	1.859425	4.309063	2.633394
H	1.020628	2.922535	3.371837
H	0.155907	3.966059	2.217857

ωB97X energy = -1507.41283784 a.u.

(5*S*,1'*S*)-**8a**, Conf. D

C	-3.471835	-1.120648	0.217980
C	-4.211374	-2.225983	-0.180364
C	-4.713524	-2.286357	-1.475708
C	-4.484254	-1.255740	-2.367876
C	-3.739027	-0.152896	-1.962224
C	-3.221980	-0.066362	-0.679222
C	-2.395913	1.111778	-0.193680
C	-2.281356	2.277110	-1.183074
C	-1.383111	3.380386	-0.678570
C	-1.785846	4.207678	0.368648
C	-0.942824	5.197817	0.852645
C	0.316211	5.378523	0.292409
C	0.724050	4.564960	-0.754575
C	-0.121301	3.572423	-1.234307
N	-1.058518	0.648342	0.218024
C	-0.231407	-0.037913	-0.510649
N	0.898472	-0.352519	0.094818

C	1.772067	-1.375139	-0.434724
C	2.793848	-1.010354	-1.320701
C	2.959098	0.398867	-1.862682
C	4.364857	0.952985	-1.625011
C	2.605769	0.443445	-3.352397
C	3.656725	-2.010579	-1.757969
C	3.501740	-3.321408	-1.342225
C	2.446757	-3.666053	-0.515367
C	1.546258	-2.707350	-0.061289
C	0.342112	-3.156663	0.754029
C	-0.539129	-4.102715	-0.067345
C	0.756114	-3.801147	2.078110
C	0.816817	0.057925	1.534441
C	2.040530	0.755045	2.165751
C	2.420174	2.059125	1.465051
C	3.244539	-0.192598	2.185177
C	1.652381	1.058622	3.620397
C	-0.473439	0.910422	1.535030
C	-3.146756	-2.011746	2.412084
O	-2.942965	-0.974618	1.460908
H	-4.405051	-3.039416	0.505925
H	-5.289966	-3.152903	-1.778406
H	-4.878646	-1.301334	-3.375480
H	-3.566976	0.649024	-2.669458
H	-2.843791	1.482247	0.731259
H	-1.895167	1.912159	-2.137783
H	-3.287604	2.660628	-1.366810
H	-2.770207	4.079548	0.808938
H	-1.271608	5.834815	1.665911
H	0.974068	6.153274	0.669029
H	1.702924	4.700547	-1.200685
H	0.204014	2.943576	-2.058393
H	-0.453556	-0.353202	-1.523931
H	2.250969	1.047790	-1.345711
H	4.417075	1.993611	-1.953142
H	4.641455	0.916705	-0.568911
H	5.113312	0.390473	-2.187924
H	1.589147	0.087057	-3.534435
H	3.288505	-0.179410	-3.935949
H	2.680389	1.466975	-3.726777
H	4.460499	-1.758529	-2.440644
H	4.192365	-4.083302	-1.684853
H	2.307880	-4.704036	-0.234127
H	-0.275896	-2.286994	0.987072
H	-1.431924	-4.375942	0.501148
H	-0.861624	-3.633036	-0.999571
H	-0.009475	-5.024997	-0.316619
H	-0.127946	-4.066182	2.662705
H	1.372299	-3.128143	2.679642
H	1.329299	-4.715544	1.907893
H	0.649656	-0.857371	2.109585
H	2.678196	1.900045	0.418109
H	3.293492	2.491962	1.958119
H	1.623785	2.805616	1.503205
H	2.988956	-1.150553	2.647155
H	3.633811	-0.392550	1.186484
H	4.049799	0.255531	2.771283
H	1.368872	0.147226	4.154118
H	2.504614	1.500783	4.139906
H	0.823517	1.767510	3.686508
H	-1.177271	0.608077	2.308077
H	-0.269448	1.978531	1.627741
H	-4.210037	-2.154428	2.618005
H	-2.640405	-1.689812	3.318926
H	-2.710075	-2.952600	2.064951

ωB97X energy = -1507.41273029 a.u.

(5*S*,1’*S*)-**8a**, Conf. E

C	-2.163449	2.011795	-0.359170
C	-2.605760	3.267712	0.037182
C	-3.421227	3.385824	1.156507
C	-3.795379	2.266380	1.878169
C	-3.349724	1.013641	1.472153
C	-2.541012	0.863266	0.356524
C	-1.980458	-0.471931	-0.094662
C	-2.732027	-1.721705	0.372891
C	-4.107095	-1.842785	-0.242041
C	-5.252524	-1.836284	0.547714
C	-6.512275	-1.960759	-0.024423
C	-6.642870	-2.093666	-1.398984
C	-5.505706	-2.107536	-2.197083
C	-4.249816	-1.985032	-1.621544
N	-0.572362	-0.549303	0.322589
C	0.461753	-0.465283	-0.464858
N	1.613027	-0.356843	0.166110
C	2.896369	-0.535875	-0.471699
C	3.401423	0.458809	-1.322085
C	2.621638	1.694087	-1.747889
C	3.341134	2.994015	-1.380968
C	2.341069	1.648164	-3.253753
C	4.679959	0.274829	-1.840686
C	5.425523	-0.850076	-1.537654
C	4.880920	-1.844206	-0.745108
C	3.599548	-1.722455	-0.217101
C	3.013911	-2.896579	0.552402
C	2.977203	-4.152857	-0.322789
C	3.777614	-3.165651	1.850974
C	1.375937	-0.272161	1.642265
C	1.794725	1.056706	2.313200
C	3.308242	1.256748	2.190404
C	1.462905	0.926389	3.806346
C	1.053191	2.259966	1.728019
C	-0.137809	-0.578781	1.723816
C	-1.007390	2.913248	-2.244546
O	-1.347212	1.800719	-1.425977
H	-2.318194	4.155553	-0.509522
H	-3.760896	4.369274	1.460417
H	-4.428624	2.359879	2.751708
H	-3.644861	0.142499	2.044139
H	-1.954585	-0.475197	-1.185119
H	-2.127601	-2.584793	0.081021

H	-2.807332	-1.748808	1.461958
H	-5.162139	-1.739236	1.624886
H	-7.392827	-1.954907	0.608014
H	-7.624712	-2.191525	-1.847614
H	-5.597347	-2.221105	-3.271309
H	-3.368955	-2.013370	-2.256111
H	0.381989	-0.493805	-1.544346
H	1.657439	1.698391	-1.236359
H	2.724428	3.850692	-1.662786
H	3.541602	3.061766	-0.309937
H	4.293748	3.082358	-1.908129
H	3.272450	1.623967	-3.823883
H	1.789072	2.538362	-3.562724
H	1.757225	0.766678	-3.530733
H	5.099827	1.029004	-2.496663
H	6.424516	-0.963891	-1.942346
H	5.453780	-2.743760	-0.549190
H	1.976878	-2.670096	0.805059
H	2.479205	-4.965071	0.211089
H	2.434928	-3.971088	-1.252899
H	3.984321	-4.490203	-0.577689
H	3.312968	-3.987450	2.399728
H	3.798588	-2.289157	2.503636
H	4.812989	-3.444994	1.641473
H	1.942509	-1.069404	2.121965
H	3.852267	0.401403	2.601040
H	3.606250	2.143577	2.754031
H	3.629797	1.395087	1.158933
H	0.391469	0.828573	3.992926
H	1.966847	0.062505	4.248396
H	1.805174	1.819531	4.332451
H	-0.031500	2.177143	1.843724
H	1.364811	3.168962	2.246669
H	1.269884	2.398351	0.666979
H	-0.341217	-1.561922	2.149626
H	-0.687673	0.168650	2.292972
H	-1.903581	3.407540	-2.626147
H	-0.438016	2.507349	-3.076786
H	-0.393126	3.632233	-1.694767

ωB97X energy = -1507.41180169 a.u.

(5*S*,1'*S*)-**8a**, Conf. F

C	2.370553	1.913713	-0.330601
C	3.023610	3.139981	-0.287370
C	3.956278	3.391297	0.712725
C	4.238820	2.434474	1.671222
C	3.576918	1.213673	1.622921
C	2.648132	0.931889	0.633060
C	1.976390	-0.425619	0.602102
C	2.336519	-1.281270	-0.621902
C	3.816545	-1.571131	-0.661633
C	4.666209	-0.816945	-1.465692
C	6.033214	-1.060104	-1.475170
C	6.567086	-2.063158	-0.677512
C	5.727071	-2.823285	0.125897
C	4.361007	-2.577372	0.132303
N	0.523725	-0.287789	0.758916
C	-0.395897	-0.408797	-0.154567
N	-1.616685	-0.158735	0.270200
C	-2.756934	-0.091413	-0.612133
C	-3.261196	-1.271089	-1.180001
C	-2.616492	-2.636833	-1.004125
C	-3.598871	-3.673334	-0.454130
C	-2.006581	-3.119698	-2.323518
C	-4.398743	-1.170287	-1.975067
C	-5.015887	0.048063	-2.194901
C	-4.472223	1.202662	-1.661355
C	-3.318915	1.164023	-0.884017
C	-2.707960	2.475744	-0.416800
C	-2.416882	3.396649	-1.605417
C	-3.599614	3.188092	0.603178
C	-1.589773	0.208474	1.720874
C	-2.388322	-0.703164	2.682546
C	-3.879635	-0.683570	2.331700
C	-2.230273	-0.099115	4.085145
C	-1.879812	-2.145013	2.687678
C	-0.065651	0.245229	1.991400
C	1.188474	2.514649	-2.319373
O	1.447975	1.584726	-1.275007
H	2.815535	3.902956	-1.025230
H	4.460203	4.350693	0.733887
H	4.964948	2.631135	2.450256
H	3.791807	0.455582	2.369390
H	2.305505	-0.971648	1.490772
H	1.778676	-2.220159	-0.561854
H	2.042867	-0.766279	-1.537144
H	4.252998	-0.031557	-2.091350
H	6.681866	-0.464804	-2.107785
H	7.634036	-2.254710	-0.684655
H	6.135823	-3.612225	0.747104
H	3.708893	-3.179228	0.758311
H	-0.186844	-0.683554	-1.179356
H	-1.801512	-2.549936	-0.285108
H	-3.079314	-4.614986	-0.264065
H	-4.054574	-3.342664	0.481729
H	-4.401688	-3.876463	-1.166189
H	-1.271243	-2.410796	-2.711963
H	-2.778586	-3.249339	-3.085984
H	-1.509509	-4.081356	-2.179632
H	-4.812224	-2.065050	-2.426318
H	-5.912565	0.100261	-2.801539
H	-4.940704	2.157889	-1.869890
H	-1.744726	2.265369	0.052017
H	-1.877936	4.284050	-1.265909
H	-1.807739	2.892081	-2.357944
H	-3.338336	3.731692	-2.086729
H	-3.120608	4.106117	0.949852
H	-3.808520	2.564042	1.475434
H	-4.558428	3.458152	0.153629
H	-2.004849	1.211743	1.816168
H	-4.267512	0.338198	2.293904

H	-4.439046	-1.222820	3.099234
H	-4.089471	-1.158815	1.373660
H	-1.195388	-0.110683	4.433811
H	-2.588556	0.933523	4.112345
H	-2.821139	-0.677674	4.797707
H	-0.827962	-2.218227	2.973326
H	-2.452361	-2.729335	3.411116
H	-2.001782	-2.622875	1.715026
H	0.302363	1.259138	2.156128
H	0.233381	-0.373516	2.836203
H	0.757496	3.438613	-1.925265
H	0.470594	2.030714	-2.977667
H	2.100077	2.740120	-2.877971

ωB97X energy = -1507.41152226 a.u.

(5*S*,1'*R*)-**8b**, Conf. A

C	-3.752078	-0.893765	-0.784113
C	-4.882518	-1.664125	-0.539941
C	-5.833502	-1.219515	0.371658
C	-5.666757	-0.017968	1.035987
C	-4.532407	0.746552	0.786945
C	-3.567135	0.328348	-0.115104
C	-2.310937	1.122638	-0.406777
C	-2.338437	2.582161	0.062262
C	-1.088129	3.331710	-0.327351
C	-0.169216	3.731795	0.636991
C	0.996021	4.399679	0.278891
C	1.253130	4.679141	-1.054832
C	0.340158	4.288347	-2.027304
C	-0.819641	3.619468	-1.665450
N	-1.149962	0.421077	0.156794
C	-0.079552	0.086993	-0.502741
N	0.849686	-0.497631	0.224216
C	2.002740	-1.143145	-0.358560
C	1.969643	-2.531538	-0.549662
C	0.720080	-3.375650	-0.348870
C	0.327001	-4.070208	-1.656155
C	0.891391	-4.400189	0.774576
C	3.130976	-3.149757	-1.002130
C	4.263761	-2.413393	-1.301016
C	4.241900	-1.034964	-1.184690
C	3.113599	-0.367444	-0.717564
C	3.129958	1.151706	-0.675020
C	4.273841	1.697948	0.181852
C	3.206996	1.718455	-2.096161
C	0.342863	-0.704789	1.617380
C	1.260116	-0.280767	2.786458
C	2.566421	-1.082649	2.771103
C	0.502756	-0.640109	4.072687
C	1.576134	1.215307	2.787413
C	-1.014669	0.041973	1.566430
C	-2.935808	-2.437654	-2.410274
O	-2.770932	-1.244005	-1.655698
H	-5.032049	-2.606053	-1.050252
H	-6.712088	-1.827090	0.555742
H	-6.409402	0.327974	1.744232
H	-4.409934	1.687918	1.308837
H	-2.149970	1.112180	-1.486818
H	-3.216570	3.051693	-0.387348
H	-2.469681	2.633636	1.145293
H	-0.370779	3.532461	1.684865
H	1.699945	4.704543	1.045317
H	2.159083	5.203476	-1.337194
H	0.531187	4.508200	-3.071598
H	-1.531262	3.327542	-2.432139
H	0.035534	0.261744	-1.565749
H	-0.111850	-2.718669	-0.087150
H	-0.619467	-4.600401	-1.528157
H	0.208991	-3.347357	-2.466481
H	1.079655	-4.800314	-1.961707
H	1.686173	-5.110779	0.535290
H	-0.032868	-4.964644	0.914269
H	1.144925	-3.925133	1.725256
H	3.142245	-4.224818	-1.143063
H	5.158861	-2.913622	-1.652279
H	5.119576	-0.462937	-1.463985
H	2.193743	1.503525	-0.237578
H	5.245493	1.477342	-0.266292
H	4.186113	2.783862	0.267950
H	4.264434	1.276563	1.189406
H	3.180587	2.809647	-2.067035
H	2.370444	1.375838	-2.710421
H	4.133510	1.413172	-2.588801
H	0.163108	-1.776171	1.733109
H	3.104983	-0.908592	3.705093
H	3.224464	-0.795158	1.950953
H	2.376258	-2.156401	2.691765
H	-0.445858	-0.104907	4.158736
H	0.297316	-1.712776	4.123107
H	1.109954	-0.373485	4.939956
H	0.695510	1.824595	2.999995
H	2.308594	1.429813	3.568501
H	1.996990	1.548476	1.837711
H	-1.850030	-0.590786	1.863726
H	-1.019375	0.939197	2.187017
H	-3.846195	-2.398440	-3.013322
H	-2.963345	-3.313960	-1.757257
H	-2.069741	-2.500531	-3.064493

ωB97X energy = -1507.41493765 a.u.

(5*S*,1'*R*)-**8b**, Conf. B

C	3.288191	-1.690497	0.135564
C	3.804450	-2.918659	-0.256231
C	4.116796	-3.137437	-1.593666
C	3.922797	-2.143704	-2.535150
C	3.406465	-0.915284	-2.134849
C	3.080995	-0.670934	-0.810218
C	2.507467	0.644253	-0.323317
C	2.582293	1.801137	-1.327874

C	1.988245	3.071076	-0.770607
C	0.719371	3.491707	-1.158990
C	0.139953	4.619669	-0.590925
C	0.828702	5.345597	0.370564
C	2.102484	4.943260	0.753441
C	2.676390	3.814660	0.186256
N	1.118352	0.453036	0.136163
C	0.179503	-0.174982	-0.506966
N	-0.969925	-0.242130	0.133258
C	-2.187405	-0.667479	-0.516742
C	-2.425697	-2.036644	-0.699432
C	-1.419538	-3.118120	-0.341815
C	-2.011773	-4.166970	0.601618
C	-0.865030	-3.780834	-1.606574
C	-3.635090	-2.408964	-1.278909
C	-4.567171	-1.462261	-1.664967
C	-4.284478	-0.115216	-1.522226
C	-3.080952	0.312637	-0.970805
C	-2.773384	1.802170	-0.943910
C	-2.753881	2.377594	-2.362631
C	-3.749834	2.574579	-0.055286
C	-0.869893	0.488505	1.435896
C	-1.304184	-0.272467	2.708970
C	-0.494699	-1.546275	2.949994
C	-2.796689	-0.613605	2.648822
C	-1.086536	0.696777	3.880060
C	0.617237	0.926979	1.430026
C	3.214410	-2.346043	2.429409
O	2.951950	-1.383727	1.415213
H	3.968672	-3.706776	0.466131
H	4.518909	-4.099208	-1.890599
H	4.169181	-2.314340	-3.575792
H	3.264312	-0.139844	-2.877724
H	3.049297	0.933423	0.579657
H	3.633644	1.952255	-1.583045
H	2.058710	1.529108	-2.247346
H	0.177986	2.933814	-1.918368
H	-0.850296	4.933865	-0.903394
H	0.378992	6.226022	0.814736
H	2.651190	5.511635	1.495768
H	3.672220	3.507409	0.491530
H	0.322128	-0.606733	-1.490784
H	-0.577275	-2.651857	0.170674
H	-1.238296	-4.875569	0.905799
H	-2.428538	-3.711574	1.502862
H	-2.807660	-4.734466	0.114080
H	-1.657743	-4.287495	-2.162672
H	-0.110904	-4.524956	-1.340838
H	-0.402108	-3.051478	-2.275681
H	-3.849752	-3.460715	-1.431394
H	-5.508625	-1.775274	-2.101398
H	-5.003713	0.619377	-1.866858
H	-1.769314	1.948090	-0.542453
H	-3.736882	2.313270	-2.834501
H	-2.468335	3.432336	-2.335113
H	-2.039930	1.845668	-2.995662
H	-3.757819	2.189911	0.967564
H	-4.768959	2.507774	-0.443688
H	-3.473568	3.630823	-0.018975
H	-1.512049	1.368526	1.363948
H	-0.813662	-2.005442	3.888020
H	0.576322	-1.349935	3.031637
H	-0.643709	-2.280798	2.158433
H	-3.400305	0.277536	2.455007
H	-3.025579	-1.351039	1.878750
H	-3.113461	-1.027384	3.608718
H	-1.641790	1.627308	3.733828
H	-1.441021	0.236507	4.804228
H	-0.032363	0.946322	4.021314
H	0.733779	2.008639	1.499939
H	1.201851	0.453458	2.218652
H	4.276716	-2.598712	2.463677
H	2.622945	-3.252095	2.274454
H	2.925129	-1.879262	3.368063

ωB97X energy = -1507.41324842 a.u.

(5*S*,1'*R*)-**8b**, Conf. C

C	0.972063	3.358574	-0.185949
C	0.435367	4.570766	0.228913
C	0.686639	5.030385	1.516949
C	1.466259	4.292314	2.387742
C	1.995018	3.076460	1.967595
C	1.759489	2.590259	0.690933
C	2.304385	1.262518	0.198968
C	3.289860	0.578754	1.156254
C	3.764415	-0.754927	0.636404
C	3.208393	-1.938564	1.114756
C	3.587705	-3.165964	0.586564
C	4.536285	-3.223999	-0.425346
C	5.109008	-2.049790	-0.898300
C	4.725288	-0.825273	-0.369846
N	1.193146	0.350325	-0.131416
C	0.045965	0.294468	0.477694
N	-0.838944	-0.494325	-0.094473
C	-2.076136	-0.844759	0.560474
C	-2.147679	-2.055275	1.261998
C	-0.946364	-2.960096	1.489928
C	-0.657739	-3.109819	2.986312
C	-1.123683	-4.329714	0.831963
C	-3.372789	-2.404658	1.821450
C	-4.466116	-1.560775	1.731939
C	-4.346217	-0.335376	1.099481
C	-3.151211	0.052306	0.501393
C	-3.053291	1.428165	-0.137101
C	-4.138735	1.655431	-1.190626
C	-3.095972	2.525850	0.930523
C	-0.211913	-1.191818	-1.260687
C	-0.999819	-1.238471	-2.589718
C	-1.256480	0.143986	-3.190186
C	-2.333797	-1.970940	-2.410305
C	-0.134194	-2.054712	-3.560594

C	1.151322	-0.462815	-1.351699
C	-0.001743	3.573177	-2.352943
O	0.776839	2.829926	-1.422562
H	-0.174882	5.164393	-0.438086
H	0.263083	5.977338	1.830965
H	1.664375	4.651495	3.390003
H	2.603896	2.505747	2.657458
H	2.805107	1.433383	-0.757917
H	4.137312	1.252844	1.299998
H	2.812890	0.436498	2.128589
H	2.467351	-1.900215	1.908241
H	3.142312	-4.077653	0.969129
H	4.834074	-4.180295	-0.839584
H	5.858331	-2.087835	-1.680727
H	5.176711	0.087786	-0.746286
H	-0.170740	0.849643	1.382597
H	-0.063375	-2.489812	1.053517
H	0.253679	-3.694010	3.133474
H	-0.520262	-2.136252	3.461752
H	-1.472495	-3.625733	3.499548
H	-1.974194	-4.863148	1.263290
H	-0.231128	-4.939853	0.986582
H	-1.294096	-4.244709	-0.243875
H	-3.466553	-3.343495	2.355916
H	-5.410465	-1.849018	2.179090
H	-5.197712	0.335181	1.070805
H	-2.087110	1.507745	-0.637737
H	-3.977661	2.613085	-1.691255
H	-4.135004	0.870271	-1.950069
H	-5.132637	1.681570	-0.738370
H	-2.969850	3.506961	0.465878
H	-2.303493	2.399074	1.672698
H	-4.052782	2.521644	1.458880
H	-0.047230	-2.229223	-0.958184
H	-1.846614	0.035575	-4.102580
H	-0.332202	0.655628	-3.464988
H	-1.815126	0.789328	-2.511679
H	-2.195685	-2.944446	-1.932473
H	-3.045581	-1.399187	-1.813898
H	-2.784637	-2.142716	-3.390094
H	0.049353	-3.061531	-3.175864
H	-0.648670	-2.149696	-4.518684
H	0.831070	-1.579155	-3.750961
H	1.990381	-1.156049	-1.374995
H	1.215277	0.209779	-2.208509
H	-0.002396	2.996794	-3.274570
H	0.440730	4.554368	-2.539161
H	-1.028964	3.692493	-1.995749

ωB97X energy = -1507.41302653 a.u.

(5*S*,1’*R*)-**8b**, Conf. D

C	2.436912	-1.650577	-0.140541
C	3.137160	-2.844274	-0.006392
C	4.051959	-2.998664	1.028634
C	4.270683	-1.976437	1.934559
C	3.559380	-0.790646	1.798982
C	2.644709	-0.605403	0.773121
C	1.908496	0.714835	0.662495
C	2.245973	1.526690	-0.596064
C	3.719338	1.845261	-0.656587
C	4.246987	2.897010	0.087914
C	5.607606	3.170819	0.060717
C	6.458447	2.393620	-0.714337
C	5.941022	1.344740	-1.462450
C	4.579712	1.073728	-1.432326
N	0.462312	0.503558	0.800796
C	-0.465716	0.651800	-0.098802
N	-1.660531	0.248325	0.283933
C	-2.870220	0.560476	-0.440650
C	-3.141907	-0.093195	-1.651563
C	-2.172728	-1.042612	-2.337082
C	-2.790206	-2.418401	-2.596215
C	-1.662639	-0.426078	-3.643844
C	-4.354510	0.180545	-2.276492
C	-5.260942	1.070308	-1.728875
C	-4.945312	1.744623	-0.563144
C	-3.737169	1.525736	0.091629
C	-3.401803	2.375936	1.307469
C	-3.401895	3.864105	0.946207
C	-4.353675	2.101768	2.474027
C	-1.583952	-0.346633	1.654626
C	-1.968624	-1.840799	1.768665
C	-1.819675	-2.222645	3.248097
C	-1.077008	-2.743023	0.914068
C	-3.436873	-2.044004	1.381387
C	-0.117916	-0.032004	2.037427
C	1.328695	-2.445472	-2.097631
O	1.533357	-1.420762	-1.132819
H	2.981929	-3.657517	-0.701992
H	4.591849	-3.934139	1.119008
H	4.983526	-2.095792	2.741068
H	3.719868	0.015872	2.507498
H	2.193914	1.316934	1.529943
H	1.957942	0.967765	-1.487416
H	1.671279	2.456609	-0.572516
H	3.586286	3.512298	0.691460
H	6.003457	3.995506	0.642578
H	7.520969	2.607111	-0.738183
H	6.598026	0.735905	-2.073178
H	4.179082	0.252354	-2.018756
H	-0.288577	1.086174	-1.073287
H	-1.310415	-1.189567	-1.685094
H	-2.038031	-3.093556	-3.011533
H	-3.177974	-2.868456	-1.679910
H	-3.610657	-2.356010	-3.314599
H	-2.485667	-0.257369	-4.342139
H	-0.949897	-1.094620	-4.132577
H	-1.169427	0.533966	-3.473593
H	-4.593478	-0.314233	-3.211151
H	-6.206762	1.255622	-2.224605
H	-5.642931	2.472329	-0.164022

H	-2.387156	2.141237	1.632336
H	-4.398258	4.204362	0.655232
H	-3.086008	4.456291	1.807792
H	-2.717924	4.070110	0.120360
H	-4.059616	2.686223	3.348325
H	-4.360054	1.046065	2.757029
H	-5.378160	2.379383	2.214335
H	-2.263795	0.209207	2.299903
H	-2.143025	-3.255557	3.390539
H	-2.441585	-1.585928	3.883234
H	-0.788388	-2.152373	3.600152
H	-1.187936	-2.531873	-0.150772
H	-0.017846	-2.650905	1.170065
H	-1.357577	-3.786453	1.071932
H	-3.622780	-1.839754	0.327194
H	-3.723572	-3.079955	1.575071
H	-4.094698	-1.400128	1.972024
H	-0.037668	0.717654	2.825793
H	0.436221	-0.917850	2.343304
H	0.910807	-3.343742	-1.634630
H	2.260062	-2.693451	-2.611704
H	0.619472	-2.043637	-2.817251

ωB97X energy = -1507.41301894 a.u.

(5*S*,1'*S*)-**9a**, Conf. A

C	0.609825	0.716313	1.547829
C	-0.481330	-0.381549	1.641503
N	-0.830338	-0.578478	0.198468
C	-0.120885	0.237954	-0.549249
N	0.719940	0.982155	0.110594
C	-0.052656	-1.629922	2.447330
C	-1.196325	-2.646715	2.520389
C	0.230108	-1.136849	3.874209
C	1.200471	-2.304289	1.886655
C	1.729028	1.869465	-0.478153
C	-1.953466	-1.324866	-0.319172
C	-3.247002	-0.849531	-0.066403
C	-4.316130	-1.627856	-0.500049
C	-4.108612	-2.802490	-1.201019
C	-2.821161	-3.209219	-1.504468
C	-1.715716	-2.482674	-1.073838
C	-0.326868	-2.948264	-1.477489
C	-0.059154	-4.400224	-1.076981
C	-0.106179	-2.754520	-2.980818
C	-3.532234	0.484439	0.606570
C	-4.369440	1.386141	-0.304660
C	-4.217512	0.300806	1.962530
C	1.487105	3.316360	-0.037596
C	3.114139	1.308650	-0.179609
C	4.060181	1.996527	0.558610
C	5.301643	1.407383	0.804699
C	5.593414	0.147096	0.331571
C	4.637144	-0.552101	-0.410923
C	3.407126	0.032480	-0.670793
C	0.104052	3.792608	-0.409198
C	-0.882103	3.952762	0.559847
C	-2.166121	4.350431	0.208778
C	-2.479485	4.594923	-1.120418
C	-1.500680	4.446498	-2.094956
C	-0.219499	4.048945	-1.740378
O	4.999741	-1.787673	-0.836027
C	4.041830	-2.561030	-1.543697
H	0.323067	1.625493	2.075027
H	1.577124	0.385756	1.926284
H	-1.366701	0.024801	2.133384
H	-0.231899	0.289249	-1.625981
H	-1.421536	-3.092759	1.551382
H	-2.111740	-2.188101	2.904299
H	-0.919023	-3.454059	3.201617
H	-0.647028	-0.644467	4.302951
H	1.068634	-0.438131	3.915909
H	0.481218	-1.987852	4.509883
H	1.024408	-2.735191	0.900882
H	2.050930	-1.621900	1.811690
H	1.498514	-3.119715	2.549052
H	1.566708	1.813812	-1.557540
H	-5.329828	-1.297569	-0.303052
H	-4.955369	-3.392310	-1.532540
H	-2.670523	-4.111354	-2.086632
H	0.405985	-2.326695	-0.961705
H	0.979579	-4.660895	-1.294037
H	-0.234849	-4.565518	-0.011634
H	-0.696745	-5.089819	-1.634461
H	0.916839	-3.029328	-3.249040
H	-0.270809	-1.718065	-3.284739
H	-0.787649	-3.383027	-3.559484
H	-2.586788	1.006607	0.769916
H	-4.512527	2.360885	0.166698
H	-3.875525	1.545663	-1.265856
H	-5.356004	0.957076	-0.493511
H	-4.369562	1.269340	2.443649
H	-3.627258	-0.324835	2.636465
H	-5.195322	-0.172288	1.842769
H	2.243318	3.937113	-0.522807
H	1.638297	3.414534	1.039792
H	3.855931	2.984790	0.949720
H	6.044212	1.946801	1.381109
H	6.551917	-0.320369	0.523273
H	2.666005	-0.494966	-1.259308
H	-0.644599	3.778909	1.605016
H	-2.921244	4.471504	0.977514
H	-3.481341	4.903730	-1.396264
H	-1.733963	4.644944	-3.134893
H	0.541042	3.944565	-2.508625
H	4.525120	-3.509013	-1.765497
H	3.152658	-2.742839	-0.930931
H	3.750132	-2.074978	-2.478865

ωB97X energy = -1507.41240571 a.u.

(5*S*,1'*S*)-**9a**, Conf. B

C	0.782831	0.157640	1.268926
C	-0.543813	-0.616293	1.477039
N	-1.165973	-0.508963	0.118785
C	-0.349573	0.117672	-0.700226
N	0.775894	0.488389	-0.158944
C	-0.362593	-2.041325	2.050040
C	0.275292	-1.871442	3.436393
C	0.537291	-2.917289	1.177361
C	-1.721408	-2.723090	2.241865
C	1.898840	1.129315	-0.851948
C	-2.534755	-0.819603	-0.221586
C	-2.803069	-1.870633	-1.110995
C	-4.136708	-2.180891	-1.358657
C	-5.162383	-1.480095	-0.749683
C	-4.870667	-0.414250	0.082554
C	-3.555415	-0.042409	0.343197
C	-3.298270	1.206020	1.173298
C	-3.975809	2.425426	0.542133
C	-3.751896	1.028974	2.624127
C	-1.725624	-2.652620	-1.844839
C	-1.832600	-4.158377	-1.594788
C	-1.769532	-2.350856	-3.346023
C	2.109116	2.553070	-0.327249
C	3.113906	0.214866	-0.777446
C	3.061891	-1.007841	-1.452769
C	4.133244	-1.876195	-1.386101
C	5.274190	-1.554245	-0.655115
C	5.326205	-0.338514	0.012915
C	4.242228	0.542969	-0.050142
C	0.866508	3.399061	-0.457601
C	0.162878	3.806857	0.671892
C	-0.997451	4.561392	0.553330
C	-1.470509	4.916367	-0.701462
C	-0.773098	4.519688	-1.835652
C	0.386835	3.768207	-1.713270
O	6.379364	0.085556	0.754572
C	7.508024	-0.770721	0.860506
H	0.819836	1.073274	1.859749
H	1.664965	-0.439064	1.497619
H	-1.181304	-0.069435	2.172559
H	-0.595855	0.312125	-1.737533
H	-0.348507	-1.249149	4.083732
H	1.270343	-1.423802	3.388454
H	0.379638	-2.849046	3.910530
H	1.533702	-2.490229	1.037952
H	0.103246	-3.090872	0.192240
H	0.667172	-3.891491	1.653318
H	-2.393089	-2.108068	2.847214
H	-2.219977	-2.936393	1.296439
H	-1.579800	-3.672194	2.763266
H	1.592134	1.193945	-1.898255
H	-4.375860	-2.988636	-2.041204
H	-6.194051	-1.749887	-0.943561
H	-5.681685	0.155280	0.522255
H	-2.226405	1.415764	1.171472
H	-3.726033	3.325118	1.108784
H	-3.643541	2.573708	-0.487728
H	-5.063153	2.321284	0.540075
H	-3.515163	1.922628	3.205487
H	-3.269588	0.173569	3.103724
H	-4.831935	0.870825	2.676674
H	-0.749552	-2.330020	-1.480394
H	-0.999938	-4.676183	-2.075866
H	-1.807499	-4.394620	-0.528698
H	-2.758816	-4.564466	-2.007309
H	-0.958450	-2.872251	-3.858872
H	-1.667037	-1.281505	-3.545579
H	-2.713854	-2.682908	-3.784150
H	2.930644	2.992121	-0.897959
H	2.426642	2.529290	0.717433
H	2.183300	-1.275484	-2.031011
H	4.093678	-2.823225	-1.911749
H	6.100346	-2.251492	-0.620236
H	4.326592	1.480570	0.485278
H	0.532187	3.544627	1.658819
H	-1.530253	4.872789	1.444882
H	-2.378007	5.501568	-0.796582
H	-1.131527	4.800520	-2.819529
H	0.929398	3.472659	-2.606545
H	8.221911	-0.248887	1.492765
H	7.239027	-1.722921	1.325494
H	7.957136	-0.954868	-0.119053

ωB97X energy = -1507.41179842 a.u.

(5*S*,1'*S*)-**9a**, Conf. C

C	0.567629	0.638263	1.523048
C	-0.481450	-0.499718	1.603200
N	-0.894974	-0.625092	0.169257
C	-0.207214	0.217186	-0.570774
N	0.654031	0.936535	0.090749
C	0.030276	-1.783887	2.296051
C	1.270610	-2.366835	1.617561
C	-1.077106	-2.841090	2.354995
C	0.374835	-1.388933	3.739498
C	1.618630	1.879357	-0.485635
C	-2.043398	-1.349092	-0.324437
C	-3.320183	-0.899033	0.038734
C	-4.412581	-1.664915	-0.356924
C	-4.248733	-2.800394	-1.129820
C	-2.983493	-3.176082	-1.544534
C	-1.853774	-2.463505	-1.154515
C	-0.497835	-2.905223	-1.680355
C	-0.186102	-4.361776	-1.329384
C	-0.409944	-2.687966	-3.194229
C	-3.570310	0.401815	0.786261
C	-4.468960	1.330628	-0.035124
C	-4.171203	0.157713	2.172337
C	1.310494	3.307675	-0.025402
C	3.030703	1.389048	-0.190105
C	3.921980	2.099733	0.608808

C	5.185787	1.581487	0.848875
C	5.581808	0.364153	0.313083
C	4.688367	-0.346047	-0.485528
C	3.420294	0.172732	-0.731736
C	-0.087254	3.739548	-0.394696
C	-1.066548	3.897647	0.581608
C	-2.358434	4.275567	0.238066
C	-2.687924	4.497756	-1.091059
C	-1.717375	4.346538	-2.073420
C	-0.427340	3.971870	-1.726256
O	4.960480	-1.542360	-1.063495
C	6.246579	-2.108456	-0.851594
H	0.252150	1.527776	2.068352
H	1.548781	0.336355	1.888006
H	-1.351745	-0.158668	2.165183
H	-0.351906	0.309541	-1.640568
H	2.106248	-1.662537	1.592431
H	1.064689	-2.682653	0.594455
H	1.606149	-3.246871	2.170122
H	-1.338083	-3.230141	1.370946
H	-1.985664	-2.437867	2.811137
H	-0.742683	-3.681945	2.966522
H	1.185907	-0.659440	3.791912
H	-0.495508	-0.970381	4.252190
H	0.695074	-2.274767	4.291146
H	1.462880	1.828790	-1.565844
H	-5.411618	-1.354038	-0.072599
H	-5.113018	-3.381140	-1.430698
H	-2.868426	-4.043859	-2.184202
H	0.273011	-2.285535	-1.221147
H	0.826414	-4.613933	-1.651969
H	-0.254363	-4.544317	-0.254772
H	-0.874313	-5.045974	-1.830747
H	0.584382	-2.958331	-3.556151
H	-0.599316	-1.646789	-3.465112
H	-1.139896	-3.307689	-3.720800
H	-2.619901	0.925753	0.911659
H	-4.592652	2.283934	0.482894
H	-4.034419	1.534774	-1.016256
H	-5.460978	0.897969	-0.182605
H	-4.302869	1.105361	2.698884
H	-3.537641	-0.487351	2.786278
H	-5.150481	-0.320538	2.091795
H	2.044599	3.966569	-0.494493
H	1.450698	3.395493	1.054313
H	3.646908	3.051531	1.044145
H	5.880748	2.134938	1.469754
H	6.573706	-0.015096	0.519083
H	2.747088	-0.401590	-1.361119
H	-0.816053	3.739163	1.626202
H	-3.106974	4.399248	1.012724
H	-3.696142	4.790718	-1.360877
H	-1.963572	4.527029	-3.113649
H	0.326922	3.868531	-2.500732
H	6.253960	-3.048996	-1.396583
H	7.033053	-1.456427	-1.240610
H	6.422628	-2.302399	0.209724

ωB97X energy = -1507.41164426 a.u.

(5*S*,1’*S*)-**9a**, Conf. D

C	-0.832098	-0.126611	-1.278095
C	0.486441	-0.929818	-1.390100
N	1.194875	-0.502094	-0.141910
C	0.386628	0.217585	0.606227
N	-0.790036	0.420436	0.082502
C	0.286717	-2.452996	-1.577435
C	-0.560382	-3.074408	-0.466135
C	1.638478	-3.169027	-1.661665
C	-0.422766	-2.637476	-2.926577
C	-1.905548	1.141058	0.709495
C	2.592776	-0.700398	0.160006
C	3.546384	-0.073579	-0.654695
C	4.887955	-0.350551	-0.411328
C	5.272388	-1.172591	0.632394
C	4.315169	-1.718287	1.468684
C	2.957936	-1.499485	1.254159
C	1.962565	-2.101524	2.233248
C	2.118362	-3.618372	2.360508
C	2.085070	-1.430167	3.604669
C	3.192907	0.917999	-1.752589
C	3.898086	2.258454	-1.530046
C	3.520806	0.366255	-3.142387
C	-2.084567	2.521453	0.067299
C	-3.137602	0.249278	0.698146
C	-3.152919	-0.870283	1.533799
C	-4.243885	-1.717376	1.526123
C	-5.336859	-1.473493	0.698613
C	-5.321841	-0.358220	-0.128646
C	-4.217745	0.499282	-0.127183
C	-0.885849	3.407874	0.292986
C	-0.683613	4.006816	1.535560
C	0.429035	4.801455	1.767156
C	1.357832	5.011138	0.755066
C	1.164151	4.423347	-0.486497
C	0.050008	3.624401	-0.713912
O	-6.325849	-0.015705	-0.973436
C	-7.477091	-0.847232	-1.010797
H	-0.881222	0.689222	-2.000873
H	-1.718191	-0.747496	-1.395462
H	1.065469	-0.573217	-2.241269
H	0.677490	0.612161	1.572555
H	-1.548748	-2.614342	-0.383496
H	-0.071843	-3.002465	0.506683
H	-0.713287	-4.135297	-0.674818
H	2.184175	-3.143532	-0.718820
H	2.275773	-2.728493	-2.433420
H	1.476537	-4.217132	-1.922436
H	-1.419057	-2.190872	-2.943242
H	0.160426	-2.201089	-3.741943
H	-0.540122	-3.703279	-3.130948
H	-1.609977	1.286303	1.751044

H	5.646673	0.103099	-1.038807
H	6.323293	-1.371293	0.807882
H	4.627686	-2.332750	2.305302
H	0.952651	-1.911430	1.869002
H	1.342661	-4.017682	3.017466
H	2.032143	-4.117164	1.392650
H	3.086134	-3.883836	2.791512
H	1.332778	-1.828153	4.288956
H	1.948007	-0.348058	3.539010
H	3.069453	-1.613642	4.042016
H	2.121462	1.123783	-1.705696
H	3.569787	2.978982	-2.282655
H	3.670103	2.668300	-0.543793
H	4.982164	2.159587	-1.618015
H	3.208391	1.073126	-3.913861
H	3.024647	-0.588166	-3.334433
H	4.596327	0.205126	-3.250116
H	-2.972957	2.975328	0.511328
H	-2.278872	2.418635	-1.002874
H	-2.311046	-1.072550	2.188158
H	-4.258147	-2.584460	2.176363
H	-6.179388	-2.151665	0.713561
H	-4.245538	1.356435	-0.789118
H	-1.411285	3.856966	2.327700
H	0.568856	5.264175	2.737490
H	2.225958	5.635341	0.933030
H	1.880460	4.587959	-1.283257
H	-0.098597	3.176953	-1.692525
H	-8.149577	-0.397565	-1.736909
H	-7.222072	-1.860940	-1.331070
H	-7.968226	-0.884692	-0.034827

ωB97X energy = -1507.41163315 a.u.

(5*S*,1'*S*)-**9a**, Conf. E

C	0.420735	0.636024	1.608378
C	-0.713262	-0.419891	1.635684
N	-0.945614	-0.648100	0.175113
C	-0.152858	0.129582	-0.530040
N	0.648608	0.871446	0.179663
C	-0.397838	-1.660023	2.502424
C	-1.585735	-2.627716	2.508677
C	-0.205260	-1.142934	3.935339
C	0.864820	-2.394867	2.051573
C	1.700364	1.745994	-0.356951
C	-2.067066	-1.346662	-0.410174
C	-1.842430	-2.537461	-1.115384
C	-2.954129	-3.215299	-1.606322
C	-4.234784	-2.730235	-1.409375
C	-4.424584	-1.524552	-0.757987
C	-3.346995	-0.793460	-0.267471
C	-3.606930	0.575112	0.343612
C	-4.325196	1.488373	-0.653761
C	-4.399877	0.473800	1.648593
C	-0.458535	-3.093136	-1.407663
C	-0.297352	-4.533840	-0.918167
C	-0.145527	-2.995673	-2.904030
C	1.475295	3.196078	0.085553
C	3.066813	1.176002	-0.014296
C	3.730235	1.490095	1.171871
C	4.952696	0.899509	1.445566
C	5.534807	0.000519	0.560133
C	4.873312	-0.309174	-0.624032
C	3.641161	0.280941	-0.902296
C	0.127020	3.723249	-0.341995
C	-0.880021	3.952101	0.591196
C	-2.129486	4.409826	0.191402
C	-2.386699	4.645644	-1.151153
C	-1.387436	4.425993	-2.090887
C	-0.141224	3.968353	-1.687639
O	5.344329	-1.165438	-1.563848
C	6.604787	-1.779947	-1.333796
H	0.130377	1.563769	2.100419
H	1.342315	0.273921	2.063346
H	-1.621281	0.028806	2.041556
H	-0.173501	0.155159	-1.613035
H	-1.754289	-3.088291	1.535086
H	-2.508200	-2.122526	2.808566
H	-1.397211	-3.428239	3.227355
H	-1.093656	-0.610084	4.284979
H	0.653129	-0.473480	4.026746
H	-0.035032	-1.986661	4.606643
H	0.753838	-2.819194	1.053641
H	1.748892	-1.752311	2.050706
H	1.066939	-3.219852	2.737875
H	1.580678	1.701444	-1.441150
H	-2.813359	-4.141292	-2.152397
H	-5.087712	-3.282296	-1.786907
H	-5.428717	-1.131345	-0.646189
H	-2.649833	1.054483	0.561468
H	-4.442239	2.486079	-0.225423
H	-3.758123	1.584575	-1.582444
H	-5.319848	1.108749	-0.898189
H	-4.532373	1.464492	2.088757
H	-3.898898	-0.160748	2.383923
H	-5.391226	0.050687	1.468599
H	0.278019	-2.482712	-0.884132
H	0.730720	-4.867648	-1.074981
H	-0.526038	-4.630528	0.145502
H	-0.953398	-5.213997	-1.465936
H	0.871030	-3.344089	-3.098855
H	-0.230377	-1.969269	-3.268970
H	-0.831482	-3.614511	-3.487797
H	2.276897	3.790619	-0.358619
H	1.581400	3.287952	1.168014
H	3.306974	2.190688	1.881543
H	5.470151	1.140677	2.366889
H	6.491976	-0.442092	0.800653
H	3.146294	0.024751	-1.833806
H	-0.684731	3.786679	1.646421
H	-2.901097	4.584832	0.932959
H	-3.360796	5.002981	-1.465275

H	-1.577335	4.616087	-3.141145
H	0.636358	3.810108	-2.429101
H	6.792576	-2.412503	-2.197642
H	7.398192	-1.032423	-1.251430
H	6.585464	-2.395312	-0.430379

ωB97X energy = -1507.41141397 a.u.

(5*S*,1’*S*)-**9a**, Conf. F

C	0.370134	0.517155	-1.971445
C	-0.769259	-0.518396	-1.823062
N	-0.793223	-0.712330	-0.337923
C	0.007582	0.157305	0.239311
N	0.648790	0.930337	-0.591542
C	-2.109411	-0.089751	-2.464655
C	-2.631483	1.230773	-1.895677
C	-3.155673	-1.193635	-2.283266
C	-1.854601	0.065216	-3.970384
C	1.607804	1.990472	-0.280717
C	-1.372078	-1.838012	0.359865
C	-0.805506	-3.102811	0.146744
C	-1.421012	-4.197267	0.745942
C	-2.526009	-4.038201	1.562691
C	-3.018297	-2.770845	1.818391
C	-2.453399	-1.642562	1.231514
C	-3.002408	-0.275529	1.608128
C	-4.490347	-0.129475	1.283727
C	-2.751120	0.009709	3.092449
C	0.470098	-3.328208	-0.650766
C	1.563541	-3.928984	0.237268
C	0.224506	-4.201940	-1.882533
C	1.183131	2.800118	0.953363
C	3.030093	1.467649	-0.167555
C	4.073840	2.278287	-0.613667
C	5.381319	1.848813	-0.477185
C	5.677231	0.614931	0.092434
C	4.634914	-0.192641	0.532834
C	3.314133	0.239730	0.401822
C	-0.199949	3.394788	0.821699
C	-1.208583	3.066590	1.721997
C	-2.488759	3.589957	1.579432
C	-2.775203	4.452198	0.532120
C	-1.771417	4.797790	-0.364964
C	-0.495957	4.274465	-0.219371
O	4.796886	-1.414242	1.099471
C	6.119997	-1.909978	1.249948
H	1.274401	0.090355	-2.409114
H	0.076616	1.387974	-2.555438
H	-0.479019	-1.464638	-2.279791
H	0.131011	0.201028	1.313736
H	-1.924051	2.053730	-2.029076
H	-2.861370	1.156817	-0.831450
H	-3.553427	1.509771	-2.410200
H	-3.409442	-1.361334	-1.237364
H	-2.803557	-2.141302	-2.700678
H	-4.072612	-0.917805	-2.808556
H	-1.165311	0.880050	-4.200337
H	-1.450595	-0.855726	-4.399492
H	-2.797316	0.284127	-4.475313
H	1.568120	2.656385	-1.145275
H	-1.013554	-5.189391	0.587033
H	-2.990733	-4.903287	2.021304
H	-3.859048	-2.653060	2.492777
H	-2.468567	0.486571	1.038875
H	-4.821989	0.885508	1.516010
H	-4.698206	-0.317828	0.228743
H	-5.094937	-0.820237	1.875923
H	-3.087298	1.018322	3.344977
H	-1.691944	-0.072742	3.348041
H	-3.300470	-0.691663	3.724800
H	0.851183	-2.364336	-0.991863
H	2.495642	-4.016343	-0.325396
H	1.750893	-3.301924	1.111852
H	1.289450	-4.926561	0.588181
H	1.144638	-4.305687	-2.461257
H	-0.543409	-3.779252	-2.535490
H	-0.102868	-5.203165	-1.592353
H	1.927737	3.590490	1.073378
H	1.251567	2.182137	1.852504
H	3.859523	3.239292	-1.068696
H	6.193052	2.476970	-0.825466
H	6.708191	0.300040	0.181163
H	2.535145	-0.425332	0.758916
H	-0.992342	2.404662	2.555846
H	-3.261762	3.324458	2.292701
H	-3.772307	4.861440	0.418310
H	-1.982963	5.481872	-1.178881
H	0.279636	4.562938	-0.922465
H	6.022669	-2.885874	1.718718
H	6.612291	-2.019637	0.280096
H	6.716159	-1.256178	1.891951

ωB97X energy = -1507.41116766 a.u.

(5*S*,1’*S*)-**9a**, Conf. G

C	0.881946	0.118521	1.183574
C	-0.448217	-0.628812	1.448782
N	-1.135132	-0.484410	0.126549
C	-0.322022	0.079032	-0.740681
N	0.844366	0.396696	-0.255403
C	-0.270302	-2.071331	1.977968
C	0.420901	-1.950493	3.343475
C	0.580817	-2.937478	1.048025
C	-1.633398	-2.734129	2.201211
C	1.961628	0.972205	-1.015439
C	-2.534051	-0.720284	-0.141384
C	-3.481053	0.101229	0.486413
C	-4.826087	-0.194592	0.287755
C	-5.217481	-1.228946	-0.543159
C	-4.263700	-1.976107	-1.210834
C	-2.904361	-1.742764	-1.027292
C	-1.910160	-2.571174	-1.825567

C	-2.089816	-4.073378	-1.597011
C	-2.008915	-2.237007	-3.317174
C	-3.115114	1.315917	1.325986
C	-3.774079	2.582379	0.772749
C	-3.483865	1.118915	2.798502
C	2.259222	2.398872	-0.539304
C	3.142226	0.014406	-0.981399
C	3.186053	-1.016854	-1.908523
C	4.225229	-1.940499	-1.868042
C	5.215418	-1.841693	-0.910792
C	5.176390	-0.805088	0.021390
C	4.140864	0.124071	-0.013986
C	1.049490	3.296635	-0.628486
C	0.424124	3.761824	0.524729
C	-0.705746	4.566125	0.446385
C	-1.227434	4.912897	-0.791289
C	-0.608836	4.458049	-1.949093
C	0.521376	3.657321	-1.866987
O	6.187090	-0.780383	0.924944
C	6.195365	0.256862	1.895129
H	0.944173	1.056801	1.736271
H	1.759433	-0.482061	1.417031
H	-1.035440	-0.084669	2.188027
H	-0.601982	0.268654	-1.770323
H	-0.168881	-1.337363	4.030275
H	1.420417	-1.516182	3.271354
H	0.527352	-2.942389	3.786436
H	1.580443	-2.524651	0.887635
H	0.108850	-3.073310	0.073875
H	0.708228	-3.927928	1.489764
H	-2.275381	-2.119956	2.838862
H	-2.164653	-2.920043	1.268127
H	-1.491871	-3.695694	2.699242
H	1.606817	1.024923	-2.046524
H	-5.581536	0.410976	0.775492
H	-6.270804	-1.438309	-0.689369
H	-4.580777	-2.759380	-1.890061
H	-0.900053	-2.313872	-1.504649
H	-1.317136	-4.626448	-2.135480
H	-2.015978	-4.333716	-0.538881
H	-3.059701	-4.418566	-1.961934
H	-1.257401	-2.795323	-3.879397
H	-1.852593	-1.171832	-3.503534
H	-2.992233	-2.502828	-3.712600
H	-2.037100	1.478985	1.258700
H	-3.434586	3.454062	1.336536
H	-3.515078	2.738349	-0.276699
H	-4.862301	2.535084	0.854144
H	-3.169776	1.984030	3.386361
H	-3.014564	0.229118	3.225582
H	-4.564767	1.008239	2.915163
H	3.070392	2.781795	-1.162883
H	2.628096	2.392207	0.488149
H	2.412914	-1.101718	-2.665093
H	4.261802	-2.743244	-2.595242
H	6.030807	-2.554002	-0.868294
H	4.115248	0.928851	0.707712
H	0.832623	3.507168	1.498057
H	-1.176541	4.923405	1.355413
H	-2.110572	5.538290	-0.854731
H	-1.005532	4.731871	-2.920134
H	1.003358	3.316764	-2.778699
H	7.078072	0.088097	2.506620
H	6.264868	1.239944	1.421756
H	5.303515	0.215163	2.526581

ωB97X energy = -1507.41116476 a.u.

(5*S*,1’*S*)-**9a**, Conf. H

C	-0.905258	-0.218750	-1.202174
C	0.462213	-0.922272	-1.373549
N	1.180797	-0.467330	-0.141123
C	0.349105	0.178509	0.647764
N	-0.855491	0.307099	0.166279
C	0.364914	-2.452503	-1.582954
C	-0.395481	-3.150050	-0.454119
C	1.760277	-3.068474	-1.727633
C	-0.376796	-2.665623	-2.910209
C	-1.997790	0.931173	0.847583
C	2.599576	-0.568260	0.104874
C	3.471732	0.145221	-0.729557
C	4.838504	-0.042951	-0.549357
C	5.323029	-0.863729	0.452975
C	4.442395	-1.494798	1.312970
C	3.065318	-1.365160	1.161516
C	2.158112	-2.053721	2.168924
C	2.414052	-3.560092	2.248949
C	2.307506	-1.407830	3.550011
C	3.003141	1.138736	-1.781875
C	3.623610	2.518466	-1.545850
C	3.311920	0.651036	-3.199776
C	-2.294850	2.312103	0.250012
C	-3.166709	-0.042129	0.850512
C	-3.158337	-1.083286	1.767719
C	-4.190722	-2.014683	1.765842
C	-5.227352	-1.911896	0.858893
C	-5.240940	-0.864601	-0.061601
C	-4.209242	0.069982	-0.067764
C	-1.156142	3.280048	0.452674
C	-0.289000	3.598525	-0.588575
C	0.766736	4.479537	-0.387699
C	0.971163	5.048227	0.861030
C	0.112052	4.735972	1.907536
C	-0.942956	3.859329	1.702743
O	-6.296175	-0.835280	-0.912713
C	-6.366452	0.220115	-1.860645
H	-1.037619	0.602908	-1.907827
H	-1.747626	-0.900194	-1.302186
H	0.983998	-0.510216	-2.236254
H	0.642797	0.573554	1.613065
H	-1.416841	-2.776268	-0.342429
H	0.111260	-3.041761	0.505952

H	-0.464360	-4.218813	-0.667040
H	2.335501	-3.023031	-0.803424
H	2.337328	-2.568488	-2.510494
H	1.663328	-4.120033	-2.006311
H	-1.401430	-2.288825	-2.885396
H	0.146629	-2.177912	-3.737090
H	-0.427750	-3.733855	-3.128924
H	-1.679044	1.068435	1.883014
H	5.536336	0.479673	-1.193644
H	6.391467	-0.994585	0.579081
H	4.832173	-2.106307	2.118854
H	1.120903	-1.919288	1.860715
H	1.701090	-4.022014	2.935313
H	2.306728	-4.042463	1.274950
H	3.418416	-3.772694	2.621563
H	1.606369	-1.858273	4.255750
H	2.115390	-0.332712	3.516142
H	3.317871	-1.551835	3.940364
H	1.922710	1.267840	-1.685775
H	3.219139	3.234842	-2.264620
H	3.406100	2.884889	-0.540248
H	4.707881	2.496519	-1.675372
H	2.931170	1.361525	-3.936466
H	2.865809	-0.324728	-3.406382
H	4.390421	0.557377	-3.349111
H	-3.197712	2.688207	0.736155
H	-2.519735	2.225466	-0.815238
H	-2.349893	-1.168854	2.486426
H	-4.187141	-2.825651	2.484806
H	-6.039461	-2.629112	0.848263
H	-4.218149	0.881017	-0.782837
H	-0.447964	3.168842	-1.573513
H	1.427600	4.725050	-1.211155
H	1.792053	5.738588	1.017613
H	0.259975	5.183064	2.883972
H	-1.617740	3.629564	2.522050
H	-7.284598	0.059315	-2.419909
H	-6.409961	1.193936	-1.365481
H	-5.515117	0.194128	-2.546447

ωB97X energy = -1507.41106329 a.u.

(5*S*,1’*S*)-**9a**, Conf. I

C	-0.526014	-0.165039	1.791082
C	0.988054	-0.475722	1.728866
N	1.222561	-0.489526	0.249185
C	0.133574	-0.103232	-0.380295
N	-0.874857	0.158475	0.403783
C	1.872767	0.495157	2.544090
C	1.451223	0.351703	4.013303
C	1.701152	1.949176	2.101548
C	3.344135	0.081458	2.441895
C	-2.213242	0.613135	0.029148
C	2.359994	-1.077824	-0.421790
C	3.236859	-0.265271	-1.155493
C	4.354716	-0.871483	-1.720933
C	4.591863	-2.226008	-1.571879
C	3.683044	-3.014362	-0.889399
C	2.537962	-2.465241	-0.321288
C	1.524798	-3.397493	0.325023
C	1.001582	-4.420357	-0.687320
C	2.101441	-4.097644	1.557581
C	3.013250	1.217874	-1.405377
C	4.173116	2.076935	-0.897021
C	2.771404	1.480332	-2.895226
C	-2.163246	1.651933	-1.101742
C	-3.154238	-0.540771	-0.279508
C	-2.716142	-1.745272	-0.802285
C	-3.643057	-2.742637	-1.101146
C	-4.989420	-2.543880	-0.882373
C	-5.432936	-1.329857	-0.351837
C	-4.515330	-0.332614	-0.048713
C	-1.316198	2.854430	-0.754507
C	-1.628295	3.641456	0.353800
C	-0.837946	4.726797	0.698785
C	0.280051	5.046788	-0.062507
C	0.591853	4.280204	-1.174926
C	-0.202895	3.191754	-1.517174
O	-6.769895	-1.218973	-0.158374
C	-7.269583	-0.003338	0.380995
H	-1.118863	-1.019168	2.122866
H	-0.755083	0.687994	2.427869
H	1.179110	-1.479909	2.106481
H	0.084222	-0.034391	-1.459994
H	1.546886	-0.683484	4.352103
H	0.423802	0.675977	4.190490
H	2.099886	0.969363	4.637297
H	2.314899	2.596868	2.731164
H	0.667805	2.295071	2.191125
H	2.017356	2.103591	1.068749
H	3.483629	-0.959533	2.747567
H	3.740839	0.190834	1.433260
H	3.945629	0.707093	3.104932
H	-2.589510	1.116452	0.922365
H	5.053121	-0.268968	-2.290653
H	5.477027	-2.672935	-2.009376
H	3.855204	-4.082051	-0.813079
H	0.659195	-2.813841	0.641696
H	0.219433	-5.029828	-0.229431
H	0.580216	-3.927109	-1.566052
H	1.794614	-5.092401	-1.023007
H	1.337741	-4.716961	2.032564
H	2.469844	-3.383181	2.298247
H	2.935289	-4.747551	1.281582
H	2.115759	1.533586	-0.871924
H	3.943506	3.135194	-1.043423
H	4.363482	1.917634	0.166122
H	5.095256	1.859027	-1.440743
H	2.561954	2.539542	-3.063274
H	1.929101	0.899589	-3.279039
H	3.651805	1.218736	-3.486916
H	-3.195282	1.956470	-1.289734

H	-1.812246	1.186729	-2.026632
H	-1.663690	-1.934570	-0.979088
H	-3.300604	-3.686720	-1.508868
H	-5.717636	-3.313608	-1.108377
H	-4.841688	0.610542	0.372419
H	-2.503219	3.409843	0.953590
H	-1.096518	5.327592	1.563307
H	0.899180	5.894489	0.207259
H	1.455021	4.530230	-1.782637
H	0.042206	2.607743	-2.399514
H	-6.856736	0.185916	1.375541
H	-7.045742	0.841079	-0.276491
H	-8.346758	-0.128346	0.455613

ωB97X energy = -1507.41080505 a.u.

(5*S*,1'*S*)-**9a**, Conf. J

C	0.514833	0.400547	-2.018526
C	-0.766639	-0.450803	-1.865905
N	-0.822099	-0.625741	-0.379075
C	0.097416	0.122014	0.193415
N	0.847396	0.783696	-0.642447
C	-2.025011	0.168914	-2.517182
C	-1.744908	0.267742	-4.023338
C	-2.340642	1.559488	-1.963560
C	-3.228940	-0.759601	-2.330547
C	1.957237	1.698627	-0.350807
C	-1.592066	-1.625218	0.325597
C	-2.622434	-1.234762	1.193476
C	-3.389019	-2.240077	1.775631
C	-3.142413	-3.576036	1.515763
C	-2.081768	-3.935830	0.703936
C	-1.269200	-2.973384	0.113453
C	-0.048654	-3.428343	-0.671630
C	0.890065	-4.246115	0.220344
C	-0.433854	-4.222199	-1.921860
C	-2.917935	0.208689	1.569192
C	-4.356818	0.614389	1.242841
C	-2.619674	0.442344	3.053759
C	1.691646	2.541131	0.902260
C	3.286172	0.966212	-0.305150
C	4.308192	1.358063	-1.152189
C	5.541369	0.711163	-1.090856
C	5.749206	-0.318319	-0.198249
C	4.717242	-0.718227	0.655112
C	3.487508	-0.074999	0.603037
C	0.411069	3.341157	0.824519
C	0.199982	4.248536	-0.213758
C	-0.983059	4.966232	-0.299336
C	-1.977007	4.792350	0.656600
C	-1.772480	3.903489	1.700602
C	-0.585419	3.184416	1.782276
O	5.009798	-1.739038	1.497994
C	3.995954	-2.188944	2.383799
H	1.347406	-0.159810	-2.448024
H	0.354938	1.299041	-2.612177
H	-0.619801	-1.432919	-2.314268
H	0.216549	0.161468	1.268617
H	-1.493304	-0.709649	-4.443834
H	-0.933737	0.960023	-4.257809
H	-2.639124	0.631000	-4.533251
H	-3.211794	1.968819	-2.479384
H	-1.517799	2.264741	-2.108279
H	-2.575941	1.532737	-0.898134
H	-3.021473	-1.757894	-2.726369
H	-3.518082	-0.866171	-1.285705
H	-4.086870	-0.357516	-2.873776
H	1.983017	2.374561	-1.207569
H	-4.196944	-1.969259	2.446055
H	-3.764394	-4.340237	1.967210
H	-1.867472	-4.986233	0.541287
H	0.516384	-2.548739	-0.984835
H	1.809050	-4.483089	-0.320199
H	1.156180	-3.692689	1.123978
H	0.429493	-5.187828	0.526981
H	0.459827	-4.492677	-2.488056
H	-1.095493	-3.653584	-2.580012
H	-0.951501	-5.145813	-1.651804
H	-2.259106	0.865758	0.999801
H	-4.503301	1.674179	1.464927
H	-4.595104	0.455322	0.189238
H	-5.073624	0.046650	1.840304
H	-2.790794	1.489409	3.313755
H	-1.585436	0.192031	3.303151
H	-3.269811	-0.167944	3.684843
H	2.549962	3.209108	1.006566
H	1.692309	1.910282	1.795533
H	4.150097	2.163989	-1.860202
H	6.342928	1.015658	-1.753672
H	6.701216	-0.832596	-0.141717
H	2.689332	-0.379228	1.268757
H	0.969908	4.404345	-0.963141
H	-1.128589	5.668302	-1.112431
H	-2.902404	5.352540	0.589887
H	-2.537074	3.771528	2.458503
H	-0.430855	2.501068	2.612264
H	3.686596	-1.394538	3.068395
H	3.125979	-2.557995	1.832458
H	4.433483	-3.005402	2.952528

ωB97X energy = -1507.41080005 a.u.

(5*S*,1'*S*)-**9a**, Conf. K

C	0.660977	0.039654	1.720921
C	-0.760064	0.648980	1.724245
N	-1.098830	0.605508	0.265964
C	-0.168473	-0.056561	-0.388782
N	0.819473	-0.468475	0.354495
C	-1.757785	-0.066032	2.664401
C	-1.911744	-1.549470	2.324130
C	-3.119261	0.633812	2.614979
C	-1.212532	0.082501	4.091724

C	2.016405	-1.209591	-0.053203
C	-2.119556	1.399349	-0.380064
C	-1.955939	2.792032	-0.387868
C	-2.991368	3.563749	-0.905206
C	-4.124888	2.975439	-1.436445
C	-4.224015	1.596776	-1.493085
C	-3.225032	0.776365	-0.977260
C	-3.360030	-0.728176	-1.150507
C	-4.625849	-1.289052	-0.499829
C	-3.323796	-1.088534	-2.639870
C	-0.687021	3.490300	0.076660
C	-0.017362	4.218609	-1.092418
C	-0.953072	4.448916	1.238746
C	1.716343	-2.212053	-1.173921
C	3.155571	-0.267823	-0.400439
C	2.997807	0.734380	-1.359074
C	4.068399	1.549764	-1.675007
C	5.304752	1.388370	-1.056473
C	5.459164	0.389577	-0.102774
C	4.378223	-0.433426	0.222822
C	0.616072	-3.187383	-0.823054
C	-0.534842	-3.271169	-1.599874
C	-1.559405	-4.148552	-1.263320
C	-1.444955	-4.953974	-0.140917
C	-0.294492	-4.886505	0.636385
C	0.726436	-4.012385	0.296284
O	6.610327	0.140271	0.568264
C	7.743699	0.942957	0.268046
H	1.439689	0.777139	1.924402
H	0.767997	-0.783595	2.425233
H	-0.721115	1.695310	2.027475
H	-0.224645	-0.224723	-1.456463
H	-0.963717	-2.091399	2.381891
H	-2.324387	-1.699829	1.324635
H	-2.596531	-2.018100	3.034046
H	-3.599131	0.547193	1.641042
H	-3.022138	1.697716	2.849467
H	-3.786788	0.187896	3.355537
H	-0.266234	-0.441383	4.240408
H	-1.064720	1.134958	4.348837
H	-1.930879	-0.336711	4.798803
H	2.305012	-1.774800	0.835138
H	-2.898628	4.644039	-0.909724
H	-4.922524	3.593383	-1.832252
H	-5.096399	1.145393	-1.952104
H	-2.506056	-1.216714	-0.678285
H	-4.654014	-2.374570	-0.621891
H	-4.668922	-1.069454	0.568720
H	-5.525328	-0.878893	-0.964703
H	-3.349635	-2.172917	-2.768056
H	-2.424012	-0.705768	-3.127592
H	-4.187038	-0.671666	-3.163749
H	0.028135	2.739694	0.415492
H	0.922295	4.667572	-0.763224
H	0.201116	3.529595	-1.911497
H	-0.653508	5.017453	-1.480457
H	-0.017522	4.894552	1.582894
H	-1.420280	3.940380	2.085891
H	-1.617265	5.260569	0.932505
H	2.651425	-2.744084	-1.363914
H	1.477208	-1.683935	-2.100959
H	2.049060	0.884707	-1.863150
H	3.949755	2.329132	-2.418883
H	6.124315	2.041463	-1.324272
H	4.520990	-1.202580	0.974422
H	-0.631407	-2.653183	-2.488262
H	-2.447731	-4.204451	-1.882953
H	-2.242772	-5.637844	0.124467
H	-0.191113	-5.520771	1.509415
H	1.623189	-3.979383	0.907565
H	8.545233	0.581188	0.907001
H	8.036357	0.834519	-0.779666
H	7.551347	1.996447	0.487725

ωB97X energy = -1507.41079226 a.u.

(5*S*,1’*S*)-**9a**, Conf. L

C	-0.481546	1.028941	-1.520273
C	-0.827024	-0.479168	-1.539689
N	-0.548939	-0.864034	-0.117201
C	0.084671	0.123904	0.481690
N	0.192174	1.208703	-0.229453
C	-2.213844	-0.808293	-2.131316
C	-3.367673	-0.151857	-1.374331
C	-2.422774	-2.325231	-2.186329
C	-2.193311	-0.280121	-3.573439
C	0.858105	2.452450	0.202373
C	-0.449478	-2.219490	0.380050
C	0.632125	-3.006501	-0.039402
C	0.670459	-4.330728	0.386344
C	-0.300354	-4.838235	1.231904
C	-1.314354	-4.017307	1.693063
C	-1.409141	-2.690293	1.285002
C	-2.496172	-1.811106	1.878838
C	-3.895277	-2.389450	1.659071
C	-2.238676	-1.577304	3.370830
C	1.788774	-2.469513	-0.869966
C	3.092264	-2.522214	-0.066387
C	1.931336	-3.208201	-2.201554
C	-0.017244	3.232515	1.188433
C	2.260392	2.120261	0.693661
C	2.659345	2.308660	2.004254
C	3.951195	1.943500	2.388832
C	4.830680	1.396293	1.481701
C	4.430276	1.207692	0.154817
C	3.151728	1.573088	-0.236295
C	-1.389279	3.525902	0.633909
C	-2.503868	2.830618	1.092472
C	-3.762133	3.071004	0.553900
C	-3.918993	4.011931	-0.453314
C	-2.812943	4.716597	-0.913913
C	-1.559007	4.476084	-0.371997

O	5.356541	0.664997	-0.673909
C	5.015542	0.502289	-2.042779
H	0.185290	1.314815	-2.332355
H	-1.369545	1.662821	-1.550785
H	-0.095040	-1.012671	-2.153027
H	0.508120	0.023726	1.474759
H	-3.303862	0.938326	-1.373993
H	-3.419845	-0.485099	-0.337903
H	-4.311030	-0.420558	-1.854781
H	-2.520036	-2.769816	-1.195527
H	-1.594135	-2.821085	-2.700020
H	-3.338240	-2.545601	-2.739617
H	-2.091715	0.807219	-3.613364
H	-1.374384	-0.722968	-4.146946
H	-3.129928	-0.540288	-4.069998
H	0.952165	3.041207	-0.713084
H	1.486147	-4.970065	0.067656
H	-0.251193	-5.872136	1.553595
H	-2.044683	-4.412283	2.390297
H	-2.452868	-0.837476	1.388984
H	-4.650048	-1.692789	2.030685
H	-4.097013	-2.574250	0.601649
H	-4.022666	-3.333112	2.194373
H	-2.993557	-0.903067	3.781548
H	-1.255484	-1.134040	3.543720
H	-2.286563	-2.516581	3.927563
H	1.609993	-1.416263	-1.094268
H	3.908461	-2.073907	-0.638765
H	2.999042	-1.976326	0.875668
H	3.374033	-3.552002	0.164989
H	2.741411	-2.772907	-2.790711
H	1.013380	-3.154200	-2.792098
H	2.163969	-4.263674	-2.042469
H	0.502111	4.163778	1.426110
H	-0.115781	2.670062	2.120147
H	1.988921	2.735137	2.739018
H	4.264937	2.091298	3.415611
H	5.834456	1.108498	1.771075
H	2.827226	1.435704	-1.260855
H	-2.389151	2.095183	1.883543
H	-4.620073	2.520091	0.922609
H	-4.899119	4.199925	-0.876120
H	-2.928899	5.459919	-1.694442
H	-0.701237	5.035289	-0.733700
H	5.890392	0.069905	-2.521460
H	4.165390	-0.176489	-2.162231
H	4.782854	1.463815	-2.508187

ωB97X energy = -1507.41004090 a.u.

(5*S*,1’*S*)-**9a**, Conf. M

C	-0.104978	0.147442	1.936814
C	-1.629185	0.203452	1.668394
N	-1.687949	-0.213354	0.232505
C	-0.485090	-0.532488	-0.195763
N	0.454379	-0.407898	0.698308
C	-2.485625	-0.615782	2.661988
C	-2.265954	0.012943	4.045207
C	-2.091875	-2.092268	2.704327
C	-3.973228	-0.485929	2.319237
C	1.889456	-0.677507	0.562952
C	-2.819619	-0.072495	-0.654055
C	-3.262149	1.219452	-0.970066
C	-4.408411	1.337326	-1.750054
C	-5.058618	0.219682	-2.241465
C	-4.555549	-1.042258	-1.979812
C	-3.428788	-1.219593	-1.183063
C	-2.897367	-2.627943	-0.972842
C	-3.956976	-3.570184	-0.398364
C	-2.331061	-3.186518	-2.281945
C	-2.531212	2.485361	-0.548896
C	-2.106142	3.300740	-1.773676
C	-3.375064	3.336762	0.402147
C	2.198988	-1.421860	-0.743158
C	2.676668	0.612188	0.705973
C	3.724272	0.685664	1.606106
C	4.471526	1.858909	1.696796
C	4.169223	2.946787	0.905273
C	3.104662	2.875623	0.002537
C	2.360373	1.707714	-0.098383
C	3.647702	-1.839285	-0.807603
C	4.091412	-2.947567	-0.090432
C	5.427709	-3.320661	-0.120962
C	6.337648	-2.588510	-0.872666
C	5.903442	-1.484607	-1.593537
C	4.565844	-1.113232	-1.559612
O	2.872086	3.992685	-0.730193
C	1.789969	3.973826	-1.648638
H	0.325845	1.133376	2.115289
H	0.156084	-0.502612	2.771007
H	-1.976819	1.235166	1.727928
H	-0.305639	-0.856827	-1.212497
H	-2.535329	1.072602	4.044382
H	-1.232425	-0.076453	4.386822
H	-2.896813	-0.493007	4.778362
H	-2.701813	-2.609327	3.448016
H	-1.046524	-2.239528	2.985467
H	-2.258684	-2.586009	1.746379
H	-4.227458	-0.968270	1.375386
H	-4.568769	-0.957278	3.103984
H	-4.276362	0.562986	2.255568
H	2.148370	-1.332628	1.399321
H	-4.786754	2.323714	-1.993988
H	-5.948548	0.333424	-2.849557
H	-5.049182	-1.910389	-2.401780
H	-2.075841	-2.588185	-0.257127
H	-3.512895	-4.545812	-0.189636
H	-4.380975	-3.184198	0.531304
H	-4.776485	-3.723438	-1.103970
H	-1.903860	-4.177808	-2.116548
H	-1.548599	-2.542201	-2.690238
H	-3.114365	-3.277598	-3.038409

H	-1.610913	2.206424	-0.031946
H	-1.548184	4.185204	-1.457249
H	-1.469803	2.712403	-2.438793
H	-2.970627	3.643636	-2.346315
H	-2.811103	4.213142	0.728087
H	-3.677475	2.776846	1.290607
H	-4.283939	3.687505	-0.092609
H	1.968707	-0.782426	-1.599728
H	1.562350	-2.309218	-0.802658
H	3.965912	-0.165862	2.232572
H	5.294448	1.920273	2.399423
H	4.737464	3.867105	0.970322
H	1.534106	1.642077	-0.796536
H	3.384328	-3.529007	0.493755
H	5.758233	-4.187894	0.439327
H	7.381361	-2.880023	-0.899335
H	6.606671	-0.909998	-2.185584
H	4.231366	-0.247433	-2.122589
H	1.936759	3.211529	-2.418998
H	0.840426	3.795645	-1.135076
H	1.770030	4.956534	-2.112846

ωB97X energy = -1507.40999171 a.u.

(5*S*,1'*S*)-**9a**, Conf. N

C	-0.677846	0.276072	1.644540
C	0.702329	-0.425008	1.695762
N	0.990013	-0.610447	0.237358
C	0.045907	-0.042892	-0.482390
N	-0.895615	0.523946	0.215748
C	1.771594	0.332496	2.517434
C	3.087050	-0.452421	2.530794
C	1.251865	0.393913	3.960898
C	2.014103	1.751719	2.002455
C	-2.075083	1.245877	-0.290267
C	1.985301	-1.489874	-0.331626
C	1.829439	-2.871425	-0.152559
C	2.835684	-3.703787	-0.632826
C	3.927810	-3.189847	-1.308589
C	4.020375	-1.828510	-1.537225
C	3.055351	-0.947671	-1.058403
C	3.192504	0.531129	-1.382184
C	4.521076	1.111968	-0.894434
C	3.019568	0.774164	-2.884498
C	0.601376	-3.501549	0.485817
C	-0.097648	-4.445761	-0.496518
C	0.946232	-4.229445	1.786871
C	-1.677585	2.302281	-1.328289
C	-3.143678	0.277880	-0.767168
C	-3.035827	-0.371365	-1.988520
C	-4.020082	-1.274431	-2.376032
C	-5.105356	-1.528531	-1.560749
C	-5.219790	-0.873654	-0.334768
C	-4.237562	0.029577	0.060936
C	-0.639785	3.270877	-0.811338
C	0.613384	3.360601	-1.409227
C	1.576182	4.234953	-0.918526
C	1.294300	5.033974	0.179288
C	0.042546	4.959687	0.778491
C	-0.915160	4.085770	0.286220
O	-6.315751	-1.176422	0.403132
C	-6.480631	-0.527119	1.655959
H	-1.482761	-0.351500	2.030483
H	-0.687881	1.225643	2.178250
H	0.600486	-1.415591	2.139864
H	0.055836	-0.070013	-1.565246
H	3.545451	-0.519264	1.544494
H	2.936501	-1.469736	2.903318
H	3.798419	0.042705	3.195389
H	1.053635	-0.607343	4.353041
H	0.338143	0.984643	4.054155
H	2.006879	0.858899	4.597643
H	2.439546	1.757388	0.998494
H	1.102928	2.355638	1.982496
H	2.723386	2.259829	2.659457
H	-2.461669	1.767200	0.586705
H	2.750800	-4.775572	-0.493031
H	4.699057	-3.855758	-1.678025
H	4.861886	-1.439222	-2.099202
H	2.394538	1.074509	-0.875325
H	4.545731	2.188820	-1.077890
H	4.667504	0.948231	0.175275
H	5.367263	0.666047	-1.422046
H	3.075045	1.843814	-3.101834
H	2.058521	0.400306	-3.247256
H	3.806959	0.276263	-3.455496
H	-0.118931	-2.715545	0.717204
H	-1.016694	-4.832607	-0.051133
H	-0.357291	-3.929605	-1.423325
H	0.535230	-5.299455	-0.749456
H	0.041955	-4.637739	2.242873
H	1.422836	-3.565031	2.512219
H	1.631165	-5.059826	1.598762
H	-2.593291	2.831562	-1.602188
H	-1.315492	1.824470	-2.241655
H	-2.200823	-0.181721	-2.653193
H	-3.938364	-1.781784	-3.330187
H	-5.878654	-2.228108	-1.855000
H	-4.313300	0.548283	1.008469
H	0.838624	2.751812	-2.280398
H	2.546818	4.291868	-1.399463
H	2.042632	5.717601	0.563211
H	-0.190020	5.588394	1.630478
H	-1.892428	4.045655	0.757947
H	-7.408821	-0.909955	2.072526
H	-5.655726	-0.761519	2.334192
H	-6.558059	0.556312	1.531646

ωB97X energy = -1507.40988076 a.u.

(5*S*,1'*S*)-**9a**, Conf. O

C	-0.927324	0.925452	-1.465997

C	-0.974913	-0.620432	-1.489125
N	-0.475092	-0.951419	-0.114753
C	0.035072	0.135421	0.429816
N	-0.140348	1.222976	-0.263134
C	-2.325988	-1.212687	-1.940395
C	-3.495866	-0.810543	-1.042893
C	-2.234368	-2.740160	-2.022165
C	-2.572907	-0.675084	-3.357813
C	0.361823	2.561821	0.103032
C	-0.046456	-2.263813	0.321161
C	1.120755	-2.803225	-0.237961
C	1.476718	-4.095808	0.134417
C	0.728644	-4.805379	1.057579
C	-0.375214	-4.219382	1.651990
C	-0.783308	-2.935000	1.304817
C	-1.952631	-2.305056	2.041318
C	-3.221607	-3.154681	1.952028
C	-1.583044	-2.042513	3.504612
C	2.041657	-2.026485	-1.167646
C	3.410750	-1.812040	-0.514106
C	2.183693	-2.703716	-2.531778
C	-0.565954	3.243209	1.114161
C	1.817222	2.441763	0.534711
C	2.234092	2.660948	1.844396
C	3.568185	2.472465	2.176067
C	4.497470	2.064886	1.230129
C	4.079461	1.852472	-0.082253
C	2.744032	2.047001	-0.420602
C	-1.985772	3.349245	0.615558
C	-2.976614	2.505965	1.109236
C	-4.273792	2.569933	0.615050
C	-4.594280	3.479713	-0.382073
C	-3.613622	4.331877	-0.876108
C	-2.320424	4.267025	-0.378738
O	4.894459	1.457485	-1.091558
C	6.254073	1.183700	-0.782067
H	-0.437748	1.341655	-2.344859
H	-1.917349	1.373233	-1.364951
H	-0.222839	-0.996129	-2.189334
H	0.586677	0.112797	1.362819
H	-3.652434	0.269927	-1.017213
H	-3.362874	-1.156206	-0.017811
H	-4.413520	-1.261723	-1.426678
H	-2.124578	-3.204641	-1.041828
H	-1.388822	-3.054088	-2.640979
H	-3.145304	-3.134958	-2.477156
H	-2.701600	0.409986	-3.370585
H	-1.749992	-0.932323	-4.030326
H	-3.485503	-1.118057	-3.761106
H	0.331666	3.127733	-0.831004
H	2.367028	-4.546490	-0.289879
H	1.022391	-5.811478	1.334040
H	-0.927959	-4.767741	2.406527
H	-2.161236	-1.337009	1.584154
H	-4.055701	-2.632698	2.426296
H	-3.498112	-3.363681	0.916211
H	-3.094940	-4.110907	2.464876
H	-2.408343	-1.542931	4.016822
H	-0.696237	-1.410194	3.587959
H	-1.378297	-2.978964	4.029660
H	1.625063	-1.032298	-1.336149
H	4.037362	-1.185158	-1.153557
H	3.312764	-1.312913	0.453616
H	3.926951	-2.761849	-0.355626
H	2.810410	-2.097735	-3.189267
H	1.213823	-2.841729	-3.016554
H	2.651373	-3.686392	-2.435759
H	-0.159909	4.237249	1.315716
H	-0.551877	2.689247	2.055741
H	1.535823	2.975476	2.609153
H	3.894164	2.641512	3.195809
H	5.529351	1.920555	1.520833
H	2.444962	1.873313	-1.449747
H	-2.733662	1.790515	1.889416
H	-5.033498	1.904406	1.009373
H	-5.605041	3.530655	-0.770139
H	-3.858810	5.052218	-1.648202
H	-1.561864	4.940929	-0.765574
H	6.715595	0.864338	-1.713014
H	6.762991	2.077596	-0.412399
H	6.334691	0.382810	-0.041796

ωB97X energy = -1507.40984795 a.u.

(5*S*,1’*S*)-**9a**, Conf. P

C	0.253970	-0.939296	-1.800439
C	0.810158	0.498669	-1.664417
N	0.636288	0.744117	-0.195375
C	-0.092379	-0.221021	0.323845
N	-0.380942	-1.185987	-0.501111
C	2.211025	0.698329	-2.281316
C	2.644439	2.163452	-2.164805
C	2.065051	0.363356	-3.773430
C	3.279082	-0.206845	-1.668440
C	-1.109210	-2.415236	-0.153161
C	0.763658	2.021207	0.472681
C	-0.200779	3.005934	0.220144
C	-0.028740	4.247240	0.824986
C	1.034085	4.484822	1.678989
C	1.931357	3.471295	1.968007
C	1.813031	2.215908	1.379789
C	2.772741	1.111480	1.787042
C	4.236769	1.508733	1.592338
C	2.517401	0.685097	3.235848
C	-1.447832	2.759585	-0.615547
C	-2.705544	2.846746	0.254761
C	-1.534780	3.709383	-1.810782
C	-0.320836	-3.247699	0.870322
C	-2.534957	-2.074209	0.274753
C	-3.158882	-2.720919	1.339712
C	-4.458074	-2.380135	1.683593
C	-5.157730	-1.401416	0.991690

C	-4.536777	-0.759965	-0.076439
C	-3.233965	-1.102333	-0.427908
C	1.139437	-3.403027	0.520350
C	2.116943	-2.754605	1.270540
C	3.462866	-2.880622	0.949606
C	3.848424	-3.659836	-0.131130
C	2.881742	-4.313519	-0.886063
C	1.538504	-4.186146	-0.561769
O	-5.115014	0.207123	-0.832050
C	-6.420287	0.640510	-0.476117
H	-0.479522	-1.029575	-2.600453
H	1.039846	-1.680506	-1.955967
H	0.142370	1.196945	-2.177023
H	-0.442661	-0.193448	1.349283
H	2.828032	2.462460	-1.132354
H	1.891041	2.836638	-2.583820
H	3.571449	2.312349	-2.722659
H	1.298610	0.982820	-4.247005
H	1.808947	-0.686202	-3.937690
H	3.011814	0.552504	-4.282659
H	3.438247	0.002044	-0.611109
H	3.042925	-1.268961	-1.766401
H	4.228263	-0.039258	-2.182351
H	-1.159080	-2.975565	-1.089807
H	-0.752345	5.033669	0.641448
H	1.149656	5.458018	2.141961
H	2.735203	3.655538	2.671904
H	2.576832	0.241222	1.159532
H	4.885740	0.656759	1.808741
H	4.436103	1.834586	0.569016
H	4.521574	2.320649	2.265459
H	3.173788	-0.146691	3.503998
H	1.483119	0.365953	3.386790
H	2.717190	1.506933	3.928101
H	-1.418520	1.742147	-1.007797
H	-3.588193	2.585679	-0.334449
H	-2.649394	2.155782	1.099752
H	-2.844785	3.855581	0.650809
H	-2.413747	3.474152	-2.414494
H	-0.651563	3.633509	-2.449917
H	-1.622694	4.748041	-1.483698
H	-0.794035	-4.229565	0.935659
H	-0.410111	-2.786494	1.857325
H	-2.645763	-3.484276	1.909301
H	-4.938110	-2.881996	2.515765
H	-6.167915	-1.152767	1.287959
H	-2.787678	-0.580533	-1.267457
H	1.825115	-2.155430	2.129160
H	4.208934	-2.368485	1.547924
H	4.897597	-3.762733	-0.382949
H	3.175827	-4.930522	-1.727524
H	0.792762	-4.710439	-1.151954
H	-6.683205	1.417944	-1.189028
H	-7.143061	-0.176339	-0.548736
H	-6.435385	1.054464	0.535830

ωB97X energy = -1507.40975963 a.u.

(5*S*,1'*S*)-**9a**, Conf. Q

C	-0.179368	-0.140297	1.969981
C	-1.674728	0.117137	1.660668
N	-1.749424	-0.267044	0.216756
C	-0.560365	-0.637275	-0.213108
N	0.369551	-0.625038	0.698343
C	-2.656696	-0.585210	2.624895
C	-2.482130	-2.103526	2.649545
C	-4.102579	-0.234895	2.259569
C	-2.372526	-0.013250	4.021100
C	1.800576	-0.909398	0.550520
C	-2.821594	0.072497	-0.692135
C	-3.028167	1.424672	-1.000115
C	-4.111941	1.744067	-1.812109
C	-4.928388	0.758053	-2.336958
C	-4.658156	-0.573680	-2.076856
C	-3.600535	-0.948268	-1.253565
C	-3.319480	-2.427154	-1.046851
C	-4.532822	-3.176207	-0.492287
C	-2.839938	-3.070553	-2.351587
C	-2.106182	2.543098	-0.538326
C	-1.490598	3.272334	-1.735805
C	-2.825106	3.526109	0.388036
C	2.097000	-1.660807	-0.753595
C	2.596257	0.377709	0.683955
C	3.622187	0.469531	1.619682
C	4.360363	1.638637	1.710510
C	4.089477	2.724762	0.887859
C	3.059382	2.630219	-0.044563
C	2.318342	1.454411	-0.141809
C	3.540854	-2.096408	-0.814950
C	4.467880	-1.388642	-1.573486
C	5.801248	-1.775747	-1.602577
C	6.222224	-2.877230	-0.870397
C	5.303287	-3.591340	-0.112202
C	3.971488	-3.202465	-0.086270
O	2.703982	3.623809	-0.896930
C	3.429458	4.844166	-0.838661
H	0.348284	0.764735	2.272256
H	-0.030834	-0.902861	2.734263
H	-1.884993	1.186070	1.724000
H	-0.383979	-0.908316	-1.246249
H	-1.475680	-2.404841	2.948555
H	-2.696873	-2.553551	1.679765
H	-3.177095	-2.535783	3.372488
H	-4.407069	-0.664908	1.305447
H	-4.246926	0.847794	2.203596
H	-4.775900	-0.621417	3.027710
H	-1.366978	-0.252988	4.374149
H	-2.489531	1.073876	4.033339
H	-3.080152	-0.434211	4.737803
H	2.058846	-1.563856	1.387306
H	-4.307328	2.782829	-2.053808
H	-5.765655	1.027932	-2.970183

H	-5.281365	-1.340508	-2.522904
H	-2.510839	-2.526427	-0.322322
H	-4.269509	-4.217056	-0.291832
H	-4.893675	-2.733190	0.438547
H	-5.358912	-3.175687	-1.206930
H	-2.597566	-4.122319	-2.185153
H	-1.949546	-2.573538	-2.743436
H	-3.615518	-3.021563	-3.119902
H	-1.270526	2.110587	0.014200
H	-0.773544	4.019542	-1.388253
H	-0.965288	2.576836	-2.394140
H	-2.252531	3.787932	-2.324749
H	-3.639117	4.032434	-0.136450
H	-2.128971	4.288899	0.742788
H	-3.254338	3.025301	1.259696
H	1.877599	-1.021042	-1.612329
H	1.446374	-2.537828	-0.810684
H	3.842749	-0.369359	2.270551
H	5.160210	1.714792	2.437975
H	4.677241	3.627825	0.983499
H	1.525573	1.414055	-0.882896
H	4.143900	-0.524883	-2.145536
H	6.511480	-1.215073	-2.199632
H	7.262557	-3.180931	-0.893107
H	5.623459	-4.456585	0.457107
H	3.257367	-3.769772	0.503228
H	2.994256	5.489040	-1.597886
H	3.327373	5.317054	0.141662
H	4.487887	4.684766	-1.060439

ωB97X energy = -1507.40974216 a.u.

(5*S*,1'*S*)-**9a**, Conf. R

C	0.157083	-2.134713	0.852426
C	1.618212	-1.649119	1.023977
N	1.687601	-0.581323	-0.022295
C	0.486321	-0.347297	-0.498338
N	-0.454377	-1.098025	0.007570
C	1.982341	-1.217692	2.465598
C	3.422016	-0.697163	2.528538
C	1.907991	-2.482057	3.333368
C	1.028359	-0.159265	3.022101
C	-1.872763	-1.054477	-0.404632
C	2.870326	0.073781	-0.526491
C	3.818638	-0.697329	-1.212110
C	4.989533	-0.068578	-1.623845
C	5.188912	1.283618	-1.412634
C	4.204722	2.038155	-0.799324
C	3.026690	1.456705	-0.341228
C	1.975369	2.355305	0.293609
C	2.527407	3.140901	1.484992
C	1.370664	3.305275	-0.745508
C	3.609439	-2.161330	-1.565539
C	3.717742	-2.383315	-3.076663
C	4.587068	-3.069664	-0.816397
C	-2.767305	-1.590125	0.723561
C	-2.235863	0.358545	-0.815411
C	-2.706699	0.612472	-2.089889
C	-3.040309	1.917949	-2.452998
C	-2.892090	2.954856	-1.557920
C	-2.409241	2.699977	-0.270306
C	-2.089964	1.402556	0.103767
C	-4.219289	-1.599618	0.313661
C	-4.694841	-2.567962	-0.567758
C	-6.022098	-2.565848	-0.972844
C	-6.893817	-1.594332	-0.497640
C	-6.430128	-0.628205	0.384445
C	-5.100387	-0.631259	0.784881
O	-2.282796	3.780206	0.539540
C	-1.801671	3.573931	1.859110
H	0.097347	-3.097534	0.342030
H	-0.365078	-2.209330	1.802249
H	2.308133	-2.443412	0.741268
H	0.295634	0.403970	-1.253745
H	3.548929	0.249202	2.003603
H	4.126447	-1.417496	2.103827
H	3.700731	-0.536393	3.572142
H	2.191525	-2.235625	4.358322
H	2.597190	-3.249148	2.970203
H	0.905339	-2.913214	3.367586
H	-0.007287	-0.505835	3.058662
H	1.319510	0.090946	4.044340
H	1.059870	0.764131	2.441342
H	-1.984722	-1.713414	-1.270479
H	5.748872	-0.645278	-2.139974
H	6.105380	1.756065	-1.746502
H	4.355622	3.103740	-0.668878
H	1.162220	1.733367	0.670657
H	1.727132	3.717483	1.955582
H	2.956902	2.480224	2.241180
H	3.301092	3.845750	1.173198
H	0.570376	3.899037	-0.295513
H	0.950948	2.764356	-1.597700
H	2.125530	3.995333	-1.129860
H	2.593196	-2.444459	-1.287777
H	3.478823	-3.421578	-3.316634
H	3.027167	-1.737860	-3.622975
H	4.728437	-2.183673	-3.439438
H	4.390365	-4.117280	-1.053502
H	4.511665	-2.947111	0.266760
H	5.618372	-2.848114	-1.102084
H	-2.635148	-0.977776	1.619042
H	-2.458438	-2.608342	0.970840
H	-2.814691	-0.197059	-2.802858
H	-3.413358	2.120537	-3.450114
H	-3.137758	3.974566	-1.829811
H	-1.719148	1.191671	1.099967
H	-4.021186	-3.335612	-0.937277
H	-6.377602	-3.326865	-1.658287
H	-7.931478	-1.593203	-0.811173
H	-7.105198	0.131122	0.762705
H	-4.741941	0.128714	1.472104

H	-2.468148	2.918610	2.426346
H	-0.792931	3.147982	1.851023
H	-1.773038	4.554511	2.327004

ωB97X energy = -1507.40973913 a.u.

(5*S*,1’*S*)-**9a**, Conf. S

C	-0.137391	0.778156	1.555139
C	-1.661412	0.944259	1.337272
N	-1.906111	-0.010938	0.212250
C	-0.773150	-0.572416	-0.157001
N	0.261137	-0.210576	0.546931
C	-2.512315	0.759150	2.614010
C	-2.055551	1.846226	3.597303
C	-2.329509	-0.616621	3.253946
C	-3.994311	0.996947	2.306277
C	1.658780	-0.632805	0.409128
C	-3.095232	-0.059784	-0.608378
C	-3.385621	1.041843	-1.425386
C	-4.577898	1.019061	-2.141999
C	-5.423423	-0.074936	-2.089193
C	-5.075400	-1.181665	-1.335178
C	-3.906080	-1.202632	-0.580933
C	-3.547133	-2.466078	0.182648
C	-4.646352	-2.886258	1.160130
C	-3.225564	-3.605193	-0.789668
C	-2.444588	2.223063	-1.606436
C	-2.035177	2.372366	-3.074499
C	-3.053316	3.523613	-1.077729
C	1.794026	-1.819885	-0.553614
C	2.511170	0.555176	0.001264
C	2.193617	1.296167	-1.137888
C	3.003774	2.352075	-1.510472
C	4.132978	2.689608	-0.769293
C	4.443469	1.951500	0.366375
C	3.624949	0.885459	0.748520
C	3.206078	-2.351891	-0.574219
C	3.642550	-3.223763	0.419768
C	4.948979	-3.692639	0.425582
C	5.835882	-3.293477	-0.565684
C	5.408445	-2.426697	-1.562356
C	4.101149	-1.959672	-1.565126
O	5.510435	2.189285	1.168321
C	6.372638	3.265991	0.828582
H	0.412143	1.705778	1.394769
H	0.109133	0.393631	2.544717
H	-1.871812	1.946774	0.961808
H	-0.720743	-1.265470	-0.986788
H	-2.168886	2.843440	3.163380
H	-1.014124	1.721691	3.902104
H	-2.667857	1.801524	4.499890
H	-1.291965	-0.814004	3.532467
H	-2.662857	-1.418698	2.594981
H	-2.925050	-0.673193	4.167561
H	-4.148445	1.967847	1.826830
H	-4.414171	0.229562	1.655697
H	-4.563780	0.991233	3.238141
H	1.970783	-0.961535	1.404578
H	-4.838501	1.864284	-2.769240
H	-6.346903	-0.074487	-2.656642
H	-5.725336	-2.049570	-1.332609
H	-2.644571	-2.274020	0.763574
H	-4.321887	-3.756811	1.734463
H	-4.890172	-2.088118	1.864866
H	-5.562957	-3.159976	0.632826
H	-2.908269	-4.493308	-0.239022
H	-2.427209	-3.329688	-1.482801
H	-4.104018	-3.872442	-1.382395
H	-1.524146	2.030229	-1.052518
H	-1.296232	3.170296	-3.175359
H	-1.595952	1.449122	-3.458349
H	-2.890576	2.628044	-3.703842
H	-2.340000	4.343965	-1.180499
H	-3.331719	3.445233	-0.023711
H	-3.953113	3.788006	-1.638471
H	1.506542	-1.511945	-1.562331
H	1.111740	-2.612606	-0.234737
H	1.318270	1.052534	-1.731624
H	2.762259	2.930689	-2.394680
H	4.749109	3.520670	-1.084980
H	3.886235	0.323759	1.639227
H	2.951484	-3.544793	1.193445
H	5.273551	-4.374805	1.203122
H	6.855791	-3.660755	-0.563334
H	6.094525	-2.113179	-2.340977
H	3.772202	-1.279358	-2.344395
H	7.147413	3.281981	1.590842
H	6.830707	3.110620	-0.151828
H	5.836338	4.218666	0.835666

ωB97X energy = -1507.40966062 a.u.

(5*S*,1’*S*)-**9a**, Conf. T

C	-0.226532	0.777102	-1.981802
C	-1.350843	-0.231353	-1.645967
N	-1.025387	-0.572171	-0.223060
C	0.140981	-0.053535	0.098247
N	0.670094	0.698016	-0.824336
C	-2.773541	0.274409	-1.971766
C	-3.811494	-0.813168	-1.676238
C	-2.785977	0.543979	-3.484148
C	-3.146456	1.557982	-1.230890
C	1.912561	1.477380	-0.698342
C	-1.622717	-1.635563	0.553214
C	-1.360025	-2.961857	0.185337
C	-1.998675	-3.969038	0.902264
C	-2.827626	-3.671357	1.969620
C	-3.013623	-2.355800	2.356443
C	-2.414326	-1.309265	1.662270
C	-2.602379	0.112459	2.162319
C	-4.078585	0.486737	2.305993
C	-1.859376	0.320406	3.485107

C	-0.374312	-3.348167	-0.906638
C	0.786286	-4.158467	-0.322114
C	-1.055006	-4.107862	-2.046486
C	1.796860	2.521318	0.419611
C	3.106905	0.537332	-0.585052
C	3.234787	-0.499935	-1.515393
C	4.312746	-1.358695	-1.445466
C	5.288175	-1.211412	-0.461820
C	5.163437	-0.180297	0.457897
C	4.070315	0.690605	0.393555
C	0.530073	3.339061	0.345462
C	-0.448900	3.204622	1.325524
C	-1.625776	3.940605	1.264491
C	-1.837392	4.824704	0.217219
C	-0.865114	4.970438	-0.765419
C	0.309154	4.233908	-0.700758
O	6.044751	0.066616	1.459020
C	7.177648	-0.782737	1.572894
H	0.311183	0.513757	-2.891816
H	-0.594665	1.800101	-2.074081
H	-1.205293	-1.146454	-2.225599
H	0.623034	-0.257197	1.047861
H	-3.911314	-1.014737	-0.609046
H	-3.557734	-1.751816	-2.177023
H	-4.787605	-0.492371	-2.046589
H	-2.514880	-0.352007	-4.049290
H	-2.101937	1.347873	-3.766646
H	-3.789465	0.843941	-3.791619
H	-3.190670	1.411249	-0.152333
H	-2.453840	2.379455	-1.428202
H	-4.135974	1.885584	-1.557647
H	1.993849	2.005672	-1.651029
H	-1.826403	-5.005572	0.634579
H	-3.313359	-4.470971	2.516922
H	-3.633865	-2.135834	3.218072
H	-2.158377	0.793289	1.435323
H	-4.172112	1.546116	2.556837
H	-4.632149	0.308002	1.381348
H	-4.560087	-0.085455	3.102186
H	-1.977345	1.352082	3.826269
H	-0.790756	0.115259	3.381598
H	-2.252068	-0.338342	4.263643
H	0.065957	-2.441316	-1.324645
H	1.522551	-4.372799	-1.100015
H	1.286172	-3.610010	0.479580
H	0.440477	-5.111633	0.084496
H	-0.332028	-4.337552	-2.831782
H	-1.868077	-3.528068	-2.490276
H	-1.475321	-5.051759	-1.691338
H	2.669301	3.175184	0.352415
H	1.847224	2.025962	1.392633
H	2.490853	-0.633615	-2.293256
H	4.409534	-2.160406	-2.168528
H	6.122887	-1.898390	-0.429652
H	4.017848	1.478576	1.134226
H	-0.282638	2.526386	2.158069
H	-2.376001	3.822438	2.039161
H	-2.754073	5.400746	0.166029
H	-1.019441	5.666071	-1.582436
H	1.066329	4.365135	-1.468351
H	7.745937	-0.413050	2.422625
H	6.878528	-1.817814	1.758607
H	7.797343	-0.736784	0.673312

ωB97X energy = -1507.40964435 a.u.

(5*S*,1’*R*)-**9b**, Conf. A

C	-0.897321	0.383153	1.470430
C	0.247051	-0.657217	1.549095
N	0.827831	-0.564788	0.171417
C	0.060552	0.195301	-0.580273
N	-0.941669	0.741901	0.048793
C	1.204446	-0.464924	2.745372
C	2.276073	-1.560928	2.762339
C	0.346337	-0.624858	4.008352
C	1.873046	0.910327	2.762051
C	-1.904970	1.683969	-0.531815
C	1.798958	-1.475579	-0.392033
C	1.419525	-2.807964	-0.607604
C	2.397406	-3.695355	-1.045151
C	3.687433	-3.266754	-1.304675
C	4.012515	-1.929325	-1.164750
C	3.077911	-1.002075	-0.713211
C	3.474559	0.463507	-0.647012
C	4.685758	0.697448	0.258087
C	3.745944	1.003553	-2.054626
C	-0.007961	-3.310346	-0.449040
C	-0.544590	-3.836164	-1.783860
C	-0.129301	-4.376007	0.641633
C	-1.609504	3.117492	-0.074366
C	-3.316839	1.186491	-0.251809
C	-4.273265	1.966344	0.373152
C	-5.550563	1.446698	0.588993
C	-5.868443	0.164897	0.197778
C	-4.901313	-0.627137	-0.427986
C	-3.634096	-0.113544	-0.657535
C	-0.201830	3.546452	-0.408208
C	0.747962	3.714282	0.593790
C	2.053396	4.079106	0.286195
C	2.422086	4.287407	-1.034358
C	1.480152	4.129140	-2.044137
C	0.179781	3.761301	-1.732657
O	-5.290961	-1.878568	-0.776950
C	-4.317504	-2.747362	-1.336180
H	-1.857466	-0.024870	1.783010
H	-0.685441	1.276111	2.060490
H	-0.179451	-1.658670	1.645914
H	0.236562	0.334082	-1.640374
H	2.999892	-1.452805	1.954214
H	1.830358	-2.556442	2.685912
H	2.823018	-1.513307	3.706333

H	-0.127104	-1.609649	4.043933
H	-0.436176	0.134856	4.074794
H	0.977710	-0.523607	4.893098
H	2.378121	1.134810	1.821734
H	1.161537	1.713137	2.964319
H	2.621161	0.938707	3.556954
H	-1.740552	1.632322	-1.611355
H	2.140467	-4.736641	-1.204362
H	4.435407	-3.974149	-1.643565
H	5.013630	-1.595557	-1.413716
H	2.639470	1.036090	-0.238736
H	4.876387	1.769210	0.353727
H	4.530484	0.290716	1.259681
H	5.584675	0.235225	-0.156682
H	3.986863	2.067506	-2.007126
H	2.877244	0.882514	-2.706238
H	4.589274	0.484965	-2.517512
H	-0.649005	-2.472319	-0.169369
H	-1.588745	-4.138991	-1.673762
H	-0.489689	-3.071496	-2.561644
H	0.018563	-4.708003	-2.123925
H	-1.172327	-4.678796	0.757342
H	0.226963	-4.011044	1.607953
H	0.451911	-5.265394	0.386677
H	-2.326748	3.773312	-0.572425
H	-1.783165	3.212272	1.000198
H	-4.050745	2.974418	0.697395
H	-6.300828	2.058105	1.076787
H	-6.854988	-0.249762	0.367550
H	-2.879382	-0.713282	-1.151781
H	0.462914	3.572359	1.631512
H	2.779628	4.204929	1.081587
H	3.438269	4.576813	-1.277189
H	1.758537	4.297655	-3.078274
H	-0.550882	3.650876	-2.528586
H	-4.821624	-3.694703	-1.509676
H	-3.935329	-2.360613	-2.285095
H	-3.485197	-2.900083	-0.641484

ωB97X energy = -1507.41290728 a.u.

(5*S*,1’*R*)-**9b**, Conf. B

C	-0.908467	-0.178316	1.194417
C	0.471134	-0.858508	1.387045
N	1.138949	-0.553753	0.081708
C	0.279841	0.021571	-0.731649
N	-0.889724	0.253981	-0.206597
C	1.210372	-0.450239	2.680389
C	1.451714	1.056125	2.780557
C	2.544903	-1.195056	2.798844
C	0.313996	-0.899380	3.843165
C	-1.995663	0.954621	-0.868940
C	2.382273	-1.124743	-0.383871
C	2.430466	-2.502291	-0.639406
C	3.662052	-3.053922	-0.977345
C	4.789806	-2.261915	-1.103973
C	4.695938	-0.892854	-0.928440
C	3.492993	-0.290734	-0.571142
C	3.441597	1.224145	-0.454378
C	4.438151	1.760760	0.575321
C	3.686483	1.870870	-1.821323
C	1.203352	-3.401513	-0.624168
C	0.972457	-4.021424	-2.005492
C	1.302452	-4.488050	0.448448
C	-2.162033	2.369914	-0.304335
C	-3.238615	0.078306	-0.821704
C	-3.212197	-1.140815	-1.505103
C	-4.316873	-1.968370	-1.476422
C	-5.466016	-1.608928	-0.776033
C	-5.491398	-0.397397	-0.099228
C	-4.373769	0.443095	-0.123858
C	-0.893354	3.182450	-0.398957
C	-0.239924	3.609989	0.751666
C	0.946146	4.330557	0.673472
C	1.492096	4.635216	-0.563980
C	0.842874	4.222068	-1.721672
C	-0.339671	3.502712	-1.638980
O	-6.548983	0.061124	0.615379
C	-7.714375	-0.748975	0.675403
H	-1.740191	-0.861507	1.361830
H	-1.032893	0.692033	1.839957
H	0.338800	-1.941712	1.430967
H	0.520045	0.261351	-1.760595
H	0.522413	1.617305	2.893529
H	1.977089	1.448210	1.908755
H	2.062718	1.267239	3.660754
H	3.279186	-0.856093	2.067714
H	2.413483	-2.272959	2.671075
H	2.964281	-1.026819	3.793024
H	-0.656651	-0.397679	3.835166
H	0.142301	-1.978911	3.815779
H	0.799225	-0.660401	4.791364
H	-1.692065	1.041033	-1.914758
H	3.736132	-4.119663	-1.162760
H	5.740801	-2.711167	-1.365850
H	5.576100	-0.276364	-1.072711
H	2.441151	1.520267	-0.131798
H	4.304233	2.838838	0.693846
H	4.304631	1.293566	1.553355
H	5.468796	1.586012	0.257636
H	3.611139	2.957078	-1.739708
H	2.956802	1.536055	-2.562419
H	4.683317	1.627054	-2.196904
H	0.321602	-2.795305	-0.409875
H	0.045650	-4.599266	-2.004446
H	0.894906	-3.251557	-2.776134
H	1.786431	-4.695971	-2.280675
H	0.392447	-5.091550	0.459842
H	1.440606	-4.063811	1.445904
H	2.144730	-5.155402	0.251163
H	-2.964717	2.851395	-0.868170
H	-2.488721	2.327227	0.737018

H	-2.326783	-1.435762	-2.058880
H	-4.297743	-2.912544	-2.008379
H	-6.318893	-2.274211	-0.770801
H	-4.438706	1.379623	0.416020
H	-0.668311	3.388165	1.724241
H	1.440676	4.653882	1.582606
H	2.417944	5.195676	-0.629022
H	1.260007	4.462433	-2.693120
H	-0.842001	3.195281	-2.551520
H	-8.426980	-0.203861	1.289204
H	-8.137910	-0.906018	-0.320205
H	-7.500228	-1.715591	1.139192

ωB97X energy = -1507.41261620 a.u.

(5*S*,1'*R*)-**9b**, Conf. C

C	-0.801865	0.339449	1.483247
C	0.328115	-0.718477	1.538452
N	0.891592	-0.623924	0.154222
C	0.132176	0.161492	-0.579304
N	-0.849762	0.722459	0.068259
C	1.305040	-0.549811	2.722674
C	1.987111	0.818752	2.746139
C	2.366948	-1.655387	2.710927
C	0.464024	-0.717888	3.996048
C	-1.792502	1.700394	-0.487131
C	1.844932	-1.540294	-0.429248
C	3.122772	-1.076328	-0.768685
C	4.040931	-2.008963	-1.242242
C	3.700949	-3.342424	-1.385555
C	2.411794	-3.761089	-1.106071
C	1.449848	-2.867812	-0.645586
C	0.020864	-3.357081	-0.462990
C	-0.545076	-3.875617	-1.788442
C	-0.088383	-4.424475	0.627504
C	3.534294	0.385055	-0.698401
C	4.762473	0.601403	0.187956
C	3.786742	0.933728	-2.106193
C	-1.455690	3.116873	-0.005562
C	-3.215518	1.239671	-0.203462
C	-4.086822	1.954912	0.613255
C	-5.367345	1.470856	0.834121
C	-5.800039	0.283746	0.260253
C	-4.926409	-0.431004	-0.556032
C	-3.640872	0.052876	-0.782344
C	-0.049704	3.528742	-0.366200
C	0.304911	3.762745	-1.694890
C	1.602961	4.119086	-2.028790
C	2.569392	4.246004	-1.037875
C	2.227907	4.017475	0.286620
C	0.924791	3.664443	0.616815
O	-5.236261	-1.598842	-1.172546
C	-6.541352	-2.127673	-0.982233
H	-1.766101	-0.059835	1.794620
H	-0.573870	1.219018	2.087247
H	-0.112064	-1.713786	1.634091
H	0.299401	0.310021	-1.639396
H	1.286838	1.625090	2.972326
H	2.477338	1.051601	1.799818
H	2.749427	0.829416	3.527841
H	3.083788	-1.540105	1.897630
H	1.912187	-2.645754	2.622489
H	2.923644	-1.627941	3.650073
H	-0.314973	0.043767	4.080316
H	-0.012276	-1.701442	4.028875
H	1.108038	-0.627117	4.872800
H	-1.638631	1.664446	-1.568293
H	5.040815	-1.682876	-1.506035
H	4.436523	-4.054182	-1.742080
H	2.142340	-4.798862	-1.267538
H	-0.608132	-2.514182	-0.171680
H	-1.596172	-4.144916	-1.664031
H	-0.476883	-3.116909	-2.570913
H	-0.009601	-4.764517	-2.129421
H	-1.130000	-4.725192	0.757691
H	0.282768	-4.062864	1.589556
H	0.487066	-5.314704	0.362393
H	2.712004	0.962209	-0.271046
H	4.964867	1.670746	0.286833
H	4.619222	0.190231	1.189541
H	5.650039	0.133068	-0.243988
H	4.036784	1.995371	-2.054706
H	2.906599	0.824414	-2.744391
H	4.618113	0.411917	-2.586744
H	-2.177356	3.795882	-0.465477
H	-1.594673	3.191270	1.075335
H	-3.783369	2.884537	1.076704
H	-6.046955	2.027962	1.468551
H	-6.804961	-0.068240	0.450942
H	-2.982408	-0.522266	-1.426053
H	-0.444771	3.677174	-2.475974
H	1.860436	4.302921	-3.065726
H	3.583755	4.526332	-1.298420
H	2.973366	4.118461	1.067617
H	0.661053	3.506570	1.657858
H	-7.304019	-1.437726	-1.352844
H	-6.578210	-3.050168	-1.556118
H	-6.728093	-2.348117	0.072085

ωB97X energy = -1507.41239874 a.u.

(5*S*,1'*R*)-**9b**, Conf. D

C	-0.720165	0.313465	1.569909
C	0.452152	-0.700922	1.587465
N	0.918814	-0.640040	0.166400
C	0.102426	0.113962	-0.537744
N	-0.850875	0.671413	0.153447
C	1.500218	-0.450490	2.694914
C	2.133108	0.938885	2.616676
C	2.600191	-1.517290	2.651581
C	0.754126	-0.594106	4.028942
C	-1.851118	1.607025	-0.378056

C	1.879771	-1.526916	-0.447746
C	3.098139	-1.005323	-0.903566
C	4.034159	-1.903417	-1.408098
C	3.768713	-3.259856	-1.466726
C	2.533127	-3.740011	-1.069597
C	1.553301	-2.884024	-0.576944
C	0.174954	-3.448748	-0.267674
C	-0.445016	-4.072643	-1.521449
C	0.211614	-4.459766	0.879812
C	3.424627	0.479030	-0.923861
C	4.714069	0.799589	-0.165657
C	3.509108	0.982944	-2.368092
C	-1.566757	3.039843	0.089190
C	-3.245482	1.089137	-0.065843
C	-3.976317	1.520780	1.039554
C	-5.227687	0.977875	1.283099
C	-5.770537	0.010730	0.446822
C	-5.039089	-0.418805	-0.656663
C	-3.780242	0.124399	-0.904916
C	-0.191687	3.511630	-0.317294
C	0.789322	3.748169	0.639490
C	2.067867	4.147023	0.267401
C	2.377554	4.320797	-1.072949
C	1.402431	4.097635	-2.038199
C	0.129544	3.695820	-1.662453
O	-5.465691	-1.353077	-1.542259
C	-6.748116	-1.929398	-1.336667
H	-1.651653	-0.117778	1.933654
H	-0.499547	1.211147	2.149442
H	0.057868	-1.706375	1.750233
H	0.207678	0.244135	-1.608208
H	1.415531	1.733762	2.827236
H	2.577179	1.134701	1.639923
H	2.925731	1.017299	3.363822
H	3.245252	-1.416861	1.778229
H	2.178774	-2.526092	2.647607
H	3.227171	-1.422985	3.540713
H	-0.039251	0.148892	4.141089
H	0.309850	-1.588073	4.129595
H	1.454317	-0.453781	4.854620
H	-1.721097	1.574933	-1.461531
H	4.988603	-1.531680	-1.763620
H	4.518452	-3.943427	-1.848080
H	2.317727	-4.798435	-1.164889
H	-0.484579	-2.630160	0.024426
H	-1.464574	-4.401619	-1.309350
H	-0.481159	-3.353976	-2.342782
H	0.124162	-4.942781	-1.856201
H	-0.795657	-4.821047	1.097064
H	0.620203	-4.023415	1.794363
H	0.828380	-5.323387	0.619649
H	2.613468	1.025828	-0.439023
H	4.860830	1.881648	-0.123105
H	4.687032	0.419901	0.858176
H	5.585106	0.365101	-0.661649
H	3.703256	2.057450	-2.379937

H	2.578188	0.801822	-2.911221
H	4.316893	0.484884	-2.910080
H	-2.336670	3.678124	-0.350920
H	-1.674498	3.118826	1.172770
H	-3.583203	2.274364	1.710552
H	-5.798547	1.311856	2.141777
H	-6.750982	-0.392664	0.661454
H	-3.231034	-0.224607	-1.773555
H	0.550562	3.630932	1.692004
H	2.819580	4.324743	1.028369
H	3.373267	4.633816	-1.366484
H	1.633890	4.240020	-3.087764
H	-0.626462	3.532820	-2.424921
H	-7.532939	-1.169650	-1.378895
H	-6.890255	-2.642144	-2.145035
H	-6.796739	-2.453188	-0.378341

ωB97X energy = -1507.41224407 a.u.

(5*S*,1'*R*)-**9b**, Conf. E

C	-1.013735	-0.585791	1.025102
C	0.431603	-1.087902	1.275506
N	1.144367	-0.564661	0.067715
C	0.290847	0.044723	-0.727024
N	-0.930834	0.087831	-0.276058
C	1.011894	-0.707651	2.657013
C	0.114876	-1.386343	3.702034
C	1.022560	0.801445	2.900502
C	2.429515	-1.266542	2.821466
C	-2.059764	0.765957	-0.928466
C	2.477715	-0.933509	-0.348788
C	2.718737	-2.263729	-0.720344
C	4.031016	-2.627975	-1.004942
C	5.055186	-1.698449	-0.971089
C	4.776152	-0.374615	-0.683273
C	3.485077	0.040839	-0.370608
C	3.237838	1.520353	-0.124593
C	4.088563	2.069497	1.022131
C	3.490108	2.316438	-1.408871
C	1.619821	-3.303324	-0.880203
C	1.583788	-3.836000	-2.315638
C	1.770575	-4.452442	0.118799
C	-2.333796	2.126128	-0.274546
C	-3.248075	-0.181472	-0.980243
C	-3.245181	-1.189704	-1.933377
C	-4.299229	-2.094571	-1.986010
C	-5.352513	-1.997248	-1.097754
C	-5.360317	-0.983034	-0.141090
C	-4.306363	-0.075429	-0.079807
C	-1.180507	3.089477	-0.418799
C	-0.839328	3.600742	-1.671185
C	0.215224	4.490368	-1.811086
C	0.949651	4.882110	-0.698056
C	0.622332	4.377983	0.551630
C	-0.437938	3.489517	0.687794
O	-6.433031	-0.957221	0.688308

C	-6.502154	0.070382	1.666280
H	-1.735236	-1.400324	0.971445
H	-1.346578	0.122101	1.784273
H	0.453210	-2.177102	1.214431
H	0.579338	0.460922	-1.685142
H	0.099272	-2.471096	3.565161
H	-0.914473	-1.021883	3.665996
H	0.500671	-1.179750	4.702131
H	0.014932	1.217405	2.965004
H	1.563000	1.339839	2.120151
H	1.516097	1.012198	3.851563
H	2.465858	-2.336859	2.601100
H	3.153954	-0.765188	2.179659
H	2.751963	-1.129435	3.855795
H	-1.735450	0.940870	-1.956446
H	4.251294	-3.654546	-1.275737
H	6.071426	-2.001982	-1.194583
H	5.578089	0.354702	-0.701530
H	2.190002	1.666912	0.144312
H	3.829102	3.114492	1.208754
H	3.933096	1.511613	1.948091
H	5.153295	2.029774	0.780725
H	3.274619	3.373923	-1.243722
H	2.857788	1.965591	-2.228187
H	4.531821	2.226736	-1.726616
H	0.654653	-2.824482	-0.706983
H	0.744309	-4.523905	-2.436930
H	1.468427	-3.024180	-3.036636
H	2.498731	-4.379746	-2.561417
H	0.954288	-5.167950	-0.000072
H	1.763429	-4.099133	1.152797
H	2.710413	-4.986075	-0.041945
H	-3.225870	2.538972	-0.751857
H	-2.571639	1.998071	0.783853
H	-2.424141	-1.268574	-2.638109
H	-4.299818	-2.880028	-2.732736
H	-6.182096	-2.693583	-1.129209
H	-4.310414	0.710350	0.662919
H	-1.415265	3.313416	-2.546049
H	0.460308	4.885548	-2.790383
H	1.770449	5.582110	-0.806063
H	1.186610	4.680581	1.426826
H	-0.698293	3.116234	1.673074
H	-7.437254	-0.085069	2.198312
H	-5.668871	0.003501	2.371140
H	-6.510619	1.059243	1.199969

ωB97X energy = -1507.41180990 a.u.

(5*S*,1’*R*)-**9b**, Conf. F

C	0.518514	0.398320	2.053500
C	-0.543106	-0.713078	1.857467
N	-0.839149	-0.582813	0.396078
C	-0.105530	0.374435	-0.128773
N	0.692968	0.966080	0.712384
C	-0.105760	-2.106772	2.363267
C	-1.228350	-3.128292	2.155316
C	0.125005	-1.960988	3.874346
C	1.177168	-2.608333	1.699041
C	1.757993	1.929420	0.420535
C	-1.955830	-1.168401	-0.309481
C	-1.712921	-2.125281	-1.305297
C	-2.815331	-2.703651	-1.926117
C	-4.105284	-2.345209	-1.575937
C	-4.315949	-1.360962	-0.626735
C	-3.248894	-0.732826	0.008201
C	-3.533343	0.417512	0.961050
C	-4.270133	1.550224	0.240540
C	-4.313296	-0.045083	2.193408
C	-0.319286	-2.516087	-1.769236
C	-0.086372	-4.026701	-1.710020
C	-0.052676	-1.981594	-3.179949
C	1.480712	2.731048	-0.855685
C	3.104985	1.228446	0.377878
C	4.141454	1.679516	1.175529
C	5.382588	1.047831	1.113038
C	5.582064	-0.026146	0.271972
C	4.533902	-0.486284	-0.529773
C	3.297937	0.144179	-0.480288
C	0.177190	3.497074	-0.839975
C	-0.763780	3.317832	-1.849763
C	-1.962429	4.021127	-1.839913
C	-2.234978	4.915262	-0.815528
C	-1.299267	5.107681	0.193683
C	-0.103436	4.405741	0.179475
O	4.815842	-1.551039	-1.320847
C	3.777171	-2.076587	-2.132309
H	0.187379	1.180414	2.736654
H	1.474176	0.009063	2.404936
H	-1.454213	-0.452271	2.397976
H	-0.183442	0.646443	-1.173138
H	-1.414023	-3.334544	1.100978
H	-2.165062	-2.785045	2.603664
H	-0.955404	-4.070677	2.634886
H	-0.773583	-1.593178	4.376949
H	0.948885	-1.283086	4.108108
H	0.372065	-2.935120	4.300271
H	1.035599	-2.798192	0.634702
H	2.012777	-1.912894	1.811315
H	1.477433	-3.551435	2.160214
H	1.760284	2.616860	1.269185
H	-2.660578	-3.448176	-2.698749
H	-4.950285	-2.819237	-2.061701
H	-5.329724	-1.058605	-0.389295
H	-2.585404	0.838729	1.299834
H	-4.410007	2.394116	0.920013
H	-3.703113	1.902208	-0.624686
H	-5.256294	1.230179	-0.103633
H	-4.459677	0.788102	2.883828
H	-3.791397	-0.842792	2.728355
H	-5.298611	-0.424757	1.912176
H	0.406808	-2.048806	-1.102488

H	0.949956	-4.255355	-1.970382
H	-0.280349	-4.427198	-0.712515
H	-0.728267	-4.557154	-2.416564
H	0.968112	-2.219424	-3.489404
H	-0.180732	-0.897496	-3.234522
H	-0.736272	-2.433095	-3.903119
H	2.319385	3.423051	-0.962602
H	1.524020	2.074705	-1.729464
H	3.987820	2.517262	1.846625
H	6.196968	1.397552	1.736771
H	6.539298	-0.531044	0.219540
H	2.484507	-0.203622	-1.106765
H	-0.555863	2.628796	-2.663571
H	-2.682836	3.867913	-2.635459
H	-3.170079	5.463211	-0.803984
H	-1.499764	5.812459	0.992582
H	0.622335	4.578023	0.968114
H	3.420946	-1.333658	-2.851767
H	4.208550	-2.918937	-2.666961
H	2.939811	-2.428088	-1.520153

ωB97X energy = -1507.41143628 a.u.

(5*S*,1’*R*)-**9b**, Conf. G

C	-0.749788	1.024232	1.515353
C	-1.070247	-0.491268	1.428550
N	-0.417965	-0.858189	0.131359
C	0.133189	0.203846	-0.411783
N	0.023621	1.291941	0.295406
C	-0.663157	-1.302590	2.680479
C	0.821436	-1.173126	3.024524
C	-1.023453	-2.781580	2.505829
C	-1.502799	-0.745755	3.839156
C	0.587533	2.606443	-0.059672
C	-0.567219	-2.099520	-0.591080
C	-1.823039	-2.412936	-1.126814
C	-1.966897	-3.645803	-1.755509
C	-0.893514	-4.508454	-1.891588
C	0.354904	-4.140581	-1.420609
C	0.549209	-2.930247	-0.762564
C	1.950355	-2.554328	-0.308257
C	2.572366	-3.618586	0.597592
C	2.856447	-2.268704	-1.509846
C	-3.001878	-1.452540	-1.106412
C	-3.438329	-1.101764	-2.531566
C	-4.176903	-2.000184	-0.294860
C	-0.342134	3.349324	-1.031084
C	2.022046	2.436939	-0.548978
C	2.468845	2.981697	-1.739633
C	3.793413	2.785223	-2.135064
C	4.660710	2.047303	-1.361485
C	4.212956	1.494540	-0.157739
C	2.903716	1.698230	0.249812
C	-1.770191	3.381247	-0.548440
C	-2.165168	4.265816	0.452596
C	-3.458940	4.235357	0.954042
C	-4.377700	3.317860	0.459613
C	-3.998833	2.442961	-0.549305
C	-2.704418	2.479632	-1.051765
O	5.127068	0.777969	0.542182
C	4.710051	0.157668	1.748849
H	-1.650076	1.638604	1.525858
H	-0.144903	1.277319	2.386202
H	-2.145003	-0.631659	1.300381
H	0.635262	0.169787	-1.371333
H	1.112642	-0.144133	3.247902
H	1.460760	-1.543948	2.222570
H	1.038807	-1.766524	3.914992
H	-0.405805	-3.276865	1.756231
H	-2.070926	-2.905295	2.216980
H	-0.877105	-3.302992	3.454157
H	-1.292341	0.307751	4.037483
H	-2.572198	-0.848573	3.636415
H	-1.279206	-1.301819	4.751509
H	0.611962	3.157702	0.884312
H	-2.930822	-3.924750	-2.166490
H	-1.025220	-5.462790	-2.388147
H	1.197158	-4.807149	-1.567727
H	1.886822	-1.630946	0.268573
H	3.538242	-3.271584	0.972922
H	1.935504	-3.841380	1.456699
H	2.744814	-4.551350	0.056174
H	3.843279	-1.946510	-1.167862
H	2.447431	-1.482911	-2.149837
H	2.986310	-3.164088	-2.122714
H	-2.684101	-0.516084	-0.644961
H	-4.229092	-0.347703	-2.506614
H	-2.604399	-0.704196	-3.114347
H	-3.830573	-1.976399	-3.055201
H	-4.987894	-1.268989	-0.266611
H	-3.889420	-2.227299	0.734783
H	-4.567945	-2.917661	-0.741401
H	0.040353	4.365227	-1.149206
H	-0.298381	2.866929	-2.010517
H	1.810257	3.559158	-2.374508
H	4.141184	3.213904	-3.067680
H	5.687612	1.880979	-1.664365
H	2.552953	1.282648	1.186383
H	-1.451835	4.983550	0.846449
H	-3.751239	4.930141	1.733081
H	-5.386634	3.290651	0.854527
H	-4.714145	1.731563	-0.948442
H	-2.416316	1.797409	-1.847094
H	3.904539	-0.561009	1.564882
H	5.579784	-0.368076	2.134202
H	4.378352	0.897211	2.482529

ωB97X energy = -1507.41082368 a.u.

(5*S*,1’*R*)-**9b**, Conf. H

C	0.168973	-0.769391	-1.597797
C	1.663583	-1.022714	-1.282774

N	1.920489	0.011985	-0.232807
C	0.805831	0.650906	0.055465
N	-0.230090	0.258890	-0.629806
C	1.996344	-2.486363	-0.909462
C	3.496506	-2.643776	-0.641942
C	1.646824	-3.337715	-2.138607
C	1.199658	-2.982762	0.297619
C	-1.625786	0.695133	-0.520250
C	3.214866	0.451925	0.234417
C	4.066778	1.100569	-0.670443
C	5.342015	1.440186	-0.229321
C	5.737749	1.194288	1.073216
C	4.851137	0.621995	1.967779
C	3.573482	0.237383	1.573086
C	2.631269	-0.343358	2.614622
C	3.229583	-1.559094	3.324962
C	2.225168	0.730425	3.628464
C	3.651186	1.493199	-2.079754
C	3.775423	3.005794	-2.283387
C	4.453323	0.736373	-3.140860
C	-1.759895	1.940097	0.365568
C	-2.491335	-0.459318	-0.048892
C	-2.210999	-1.115019	1.150749
C	-3.033011	-2.143552	1.571924
C	-4.138153	-2.535575	0.821281
C	-4.412972	-1.880827	-0.373261
C	-3.581680	-0.844208	-0.804872
C	-3.169464	2.478780	0.336939
C	-3.598190	3.257239	-0.735237
C	-4.902337	3.728131	-0.790917
C	-5.795149	3.425869	0.229050
C	-5.375319	2.654095	1.303860
C	-4.069948	2.183544	1.355844
O	-5.455348	-2.176228	-1.188195
C	-6.329754	-3.225692	-0.798023
H	0.008973	-0.391423	-2.608122
H	-0.448156	-1.655548	-1.455904
H	2.272021	-0.768954	-2.151107
H	0.772715	1.442658	0.792963
H	3.820644	-2.106921	0.249827
H	4.090382	-2.284917	-1.487339
H	3.729627	-3.700758	-0.496271
H	2.192711	-2.998071	-3.022990
H	0.579323	-3.322580	-2.369024
H	1.924755	-4.376546	-1.951406
H	1.465292	-2.446075	1.208998
H	0.119729	-2.893622	0.154442
H	1.417352	-4.039380	0.466870
H	-1.929371	0.962693	-1.536258
H	6.029602	1.926136	-0.912467
H	6.734003	1.470406	1.398901
H	5.157597	0.468877	2.996287
H	1.721054	-0.675504	2.114771
H	2.494398	-1.990433	4.007811
H	3.529750	-2.333825	2.615944
H	4.107194	-1.284140	3.914214
H	1.510160	0.322247	4.345996
H	1.763949	1.592745	3.140795
H	3.094493	1.089625	4.184751
H	2.595362	1.253303	-2.214307
H	3.398923	3.280076	-3.271121
H	3.200750	3.553940	-1.533986
H	4.815409	3.333270	-2.220063
H	4.109533	1.008017	-4.141107
H	4.356212	-0.346647	-3.030737
H	5.516024	0.981297	-3.072397
H	-1.482347	1.694315	1.394070
H	-1.071331	2.708178	0.003429
H	-1.357058	-0.824807	1.754891
H	-2.820782	-2.656330	2.502987
H	-4.764304	-3.342892	1.176370
H	-3.814145	-0.347839	-1.741149
H	-2.902708	3.503110	-1.532204
H	-5.220910	4.335655	-1.630393
H	-6.813683	3.794582	0.187677
H	-6.065871	2.416582	2.105133
H	-3.747494	1.577029	2.196393
H	-7.084322	-3.293227	-1.577561
H	-5.796584	-4.177340	-0.724819
H	-6.813135	-3.002648	0.156793

ωB97X energy = -1507.41020066 a.u.

(5*S*,1’*R*)-**9b**, Conf. I

C	0.229061	-0.282843	2.017860
C	1.682813	0.117073	1.664112
N	1.760575	-0.303736	0.229949
C	0.594752	-0.764450	-0.171165
N	-0.328683	-0.763100	0.747968
C	2.029387	1.591409	1.978570
C	3.486805	1.892713	1.614790
C	1.882738	1.754144	3.498551
C	1.105332	2.582647	1.269869
C	-1.760234	-1.048705	0.609400
C	2.961067	-0.418423	-0.565005
C	3.915418	-1.377641	-0.199844
C	5.104376	-1.414091	-0.921705
C	5.316204	-0.568834	-1.995994
C	4.326502	0.316669	-2.383412
C	3.129333	0.414541	-1.680979
C	2.066330	1.377707	-2.184288
C	2.591950	2.809323	-2.306332
C	1.493758	0.896929	-3.521185
C	3.694384	-2.404437	0.899851
C	3.788007	-3.827034	0.341065
C	4.671946	-2.212678	2.061370
C	-2.062084	-1.813403	-0.685567
C	-2.556826	0.238653	0.737093
C	-3.571203	0.340132	1.684575
C	-4.305214	1.511775	1.776038
C	-4.041283	2.591646	0.942881
C	-3.022954	2.487749	-0.001292

C	-2.287132	1.308703	-0.099768
C	-3.508586	-2.241091	-0.741040
C	-3.945278	-3.336316	0.000267
C	-5.279564	-3.717019	-0.019789
C	-6.194917	-3.005592	-0.784762
C	-5.767872	-1.915052	-1.529718
C	-4.432085	-1.536044	-1.506425
O	-2.672896	3.474852	-0.862912
C	-3.393085	4.698401	-0.803493
H	0.179879	-1.082872	2.756903
H	-0.363867	0.558483	2.374514
H	2.383261	-0.501213	2.226554
H	0.435534	-1.124133	-1.179494
H	3.662760	1.867830	0.539277
H	4.169573	1.180242	2.086063
H	3.749822	2.890979	1.971185
H	2.537413	1.062508	4.035679
H	0.858570	1.590654	3.841139
H	2.163208	2.770069	3.782700
H	1.214562	2.536993	0.185766
H	0.050959	2.424180	1.510662
H	1.358442	3.598794	1.579621
H	-2.012497	-1.694967	1.454168
H	5.869103	-2.133185	-0.650475
H	6.248126	-0.615986	-2.547400
H	4.486824	0.948071	-3.249999
H	1.243961	1.394498	-1.468365
H	1.779535	3.481319	-2.591382
H	3.013408	3.167855	-1.364583
H	3.367610	2.884058	-3.071495
H	0.701284	1.570063	-3.855286
H	1.075353	-0.109654	-3.444122
H	2.267610	0.877479	-4.292358
H	2.680213	-2.293014	1.286522
H	3.553653	-4.551371	1.124003
H	3.086151	-3.974593	-0.482290
H	4.793119	-4.046040	-0.026178
H	4.467968	-2.938930	2.850932
H	4.600304	-1.211630	2.494005
H	5.703241	-2.357958	1.730811
H	-1.839282	-1.185204	-1.552230
H	-1.416926	-2.694954	-0.732602
H	-3.785425	-0.493167	2.344692
H	-5.095996	1.595115	2.512564
H	-4.624933	3.497230	1.039794
H	-1.502658	1.262403	-0.849317
H	-3.234139	-3.901603	0.595279
H	-5.604475	-4.573756	0.559619
H	-7.237216	-3.302797	-0.802731
H	-6.475269	-1.356588	-2.132187
H	-4.103379	-0.680830	-2.088536
H	-4.454293	4.541814	-1.013725
H	-3.279154	5.175735	0.173335
H	-2.963110	5.337723	-1.570336

ωB97X energy = -1507.41018148 a.u.

(5*S*,1'*R*)-**9b**, Conf. J

C	-0.598435	1.280437	1.599809
C	-1.688202	0.224271	1.282249
N	-1.022150	-0.573647	0.204556
C	0.171865	-0.083777	-0.047075
N	0.504717	0.934287	0.693688
C	-2.203621	-0.551103	2.516694
C	-3.292303	-1.549021	2.108369
C	-2.844864	0.494063	3.440903
C	-1.092131	-1.281629	3.270225
C	1.785601	1.661572	0.636441
C	-1.650599	-1.549487	-0.654042
C	-2.629309	-1.111310	-1.555964
C	-3.279406	-2.074570	-2.320945
C	-2.935420	-3.411803	-2.230284
C	-1.912148	-3.807644	-1.386944
C	-1.245385	-2.889424	-0.581564
C	-0.094892	-3.370629	0.287364
C	-0.509922	-4.512819	1.216250
C	1.099950	-3.781743	-0.578420
C	-2.962611	0.355287	-1.781663
C	-2.657486	0.760939	-3.226370
C	-4.412616	0.678250	-1.417492
C	1.763587	2.713630	-0.482237
C	2.938344	0.668163	0.561116
C	3.051027	-0.309676	1.556313
C	4.077329	-1.230946	1.501424
C	5.015839	-1.204353	0.471934
C	4.909699	-0.228531	-0.508397
C	3.867799	0.703837	-0.460459
C	0.549371	3.604988	-0.418434
C	-0.529046	3.385012	-1.270915
C	-1.684507	4.149118	-1.167782
C	-1.770995	5.151801	-0.212032
C	-0.692856	5.392039	0.631057
C	0.458437	4.623973	0.527452
O	5.760189	-0.095474	-1.555978
C	6.823670	-1.030132	-1.672875
H	-0.935777	2.297640	1.403167
H	-0.244948	1.223348	2.629185
H	-2.554754	0.714380	0.834770
H	0.818801	-0.496713	-0.812416
H	-2.900762	-2.373810	1.512458
H	-4.084114	-1.061010	1.532988
H	-3.747818	-1.974928	3.004997
H	-3.651909	1.028448	2.932309
H	-2.123273	1.229834	3.803364
H	-3.270587	-0.004379	4.313719
H	-0.610951	-2.040124	2.652478
H	-0.320367	-0.603238	3.640712
H	-1.517852	-1.788381	4.138768
H	1.849893	2.179517	1.597008
H	-4.052422	-1.768914	-3.017315
H	-3.451504	-4.146144	-2.837907
H	-1.624490	-4.852418	-1.352724

H	0.236303	-2.542720	0.915147
H	0.317833	-4.774612	1.878958
H	-1.366025	-4.237628	1.836596
H	-0.778082	-5.408568	0.651753
H	1.936509	-4.086151	0.054716
H	1.437864	-2.960682	-1.215873
H	0.842274	-4.622700	-1.227100
H	-2.315285	0.966036	-1.150511
H	-2.837560	1.830371	-3.362275
H	-1.615967	0.554667	-3.483056
H	-3.293088	0.224939	-3.934721
H	-4.608374	1.743417	-1.559849
H	-4.633984	0.428313	-0.376873
H	-5.108773	0.121462	-2.049302
H	2.676215	3.307611	-0.397507
H	1.790001	2.210605	-1.451842
H	2.332522	-0.347854	2.368410
H	4.162304	-1.988166	2.272280
H	5.809713	-1.938730	0.453595
H	3.825924	1.444409	-1.248761
H	-0.463925	2.609060	-2.028882
H	-2.516796	3.963604	-1.838506
H	-2.671407	5.749293	-0.129057
H	-0.748871	6.180607	1.372721
H	1.295044	4.816603	1.192476
H	7.492702	-0.977587	-0.809885
H	6.441871	-2.049017	-1.779346
H	7.369820	-0.753081	-2.570999

ωB97X energy = -1507.41014515 a.u.

(5*S*,1'*R*)-**9b**, Conf. K

C	0.941760	1.015541	-1.509412
C	1.203929	-0.512423	-1.428509
N	0.494653	-0.866484	-0.158054
C	-0.063879	0.203177	0.359712
N	0.101286	1.291404	-0.336436
C	0.805102	-1.296526	-2.700856
C	-0.658050	-1.092704	-3.095856
C	1.087173	-2.792197	-2.526113
C	1.711620	-0.772902	-3.824084
C	-0.474841	2.607112	-0.008041
C	0.553751	-2.119307	0.555613
C	1.771418	-2.504167	1.131000
C	1.825468	-3.745975	1.756424
C	0.701827	-4.548270	1.850311
C	-0.507432	-4.109696	1.339398
C	-0.612681	-2.887690	0.682642
C	-1.976038	-2.435840	0.182527
C	-2.618025	-3.461595	-0.753308
C	-2.908736	-2.114020	1.354516
C	3.001877	-1.610283	1.157200
C	3.403102	-1.285059	2.598715
C	4.173682	-2.223219	0.388651
C	0.405976	3.343957	1.012107
C	-1.931338	2.434599	0.413175
C	-2.435742	2.976298	1.592386
C	-3.763568	2.761101	1.932474
C	-4.603823	2.006457	1.127408
C	-4.099898	1.467319	-0.054163
C	-2.773385	1.694138	-0.406937
C	1.854156	3.385724	0.594983
C	2.767616	2.478396	1.125385
C	4.083527	2.452828	0.681442
C	4.504783	3.344952	-0.295281
C	3.605990	4.267257	-0.816644
C	2.290652	4.286345	-0.373954
O	-4.819112	0.708107	-0.918066
C	-6.178957	0.443523	-0.600649
H	1.862083	1.596538	-1.450667
H	0.404166	1.304024	-2.412429
H	2.267952	-0.693768	-1.267730
H	-0.616697	0.175433	1.290918
H	-0.890233	-0.048460	-3.316834
H	-1.344676	-1.439986	-2.323006
H	-0.870833	-1.664896	-4.001248
H	0.419916	-3.261470	-1.802753
H	2.116565	-2.968787	-2.202180
H	0.948978	-3.299528	-3.483279
H	1.556689	0.290084	-4.022824
H	2.767094	-0.925728	-3.583442
H	1.496397	-1.313199	-4.747872
H	-0.451462	3.160971	-0.950227
H	2.757726	-4.080406	2.197817
H	0.763555	-5.510543	2.345205
H	-1.389804	-4.729417	1.452895
H	-1.851767	-1.513059	-0.384920
H	-3.550567	-3.062459	-1.158683
H	-1.962625	-3.709529	-1.591406
H	-2.854530	-4.388449	-0.225952
H	-3.862007	-1.732461	0.979501
H	-2.481103	-1.359191	2.019438
H	-3.111702	-3.006886	1.951084
H	2.755568	-0.656366	0.688018
H	4.238355	-0.580332	2.603835
H	2.572340	-0.834900	3.146916
H	3.719309	-2.181131	3.137575
H	5.025902	-1.540028	0.394706
H	3.913188	-2.430603	-0.652313
H	4.493493	-3.162716	0.845716
H	0.014849	4.357518	1.122676
H	0.319276	2.851723	1.983945
H	-1.809882	3.561859	2.252497
H	-4.153809	3.183533	2.851151
H	-5.631546	1.846979	1.424689
H	-2.417836	1.265235	-1.338289
H	2.445738	1.780250	1.893481
H	4.780600	1.735281	1.100960
H	5.530763	3.326990	-0.644245
H	3.930556	4.974621	-1.571223
H	1.593447	5.008093	-0.788893
H	-6.561592	-0.172663	-1.410237

H	-6.265282	-0.101871	0.343159
H	-6.757337	1.369286	-0.543158

ωB97X energy = -1507.41008966 a.u.

(5*S*,1’*R*)-**9b**, Conf. L

C	0.178748	-0.930029	-1.493285
C	1.694399	-1.086032	-1.217061
N	1.926820	0.012628	-0.228360
C	0.789753	0.610365	0.059397
N	-0.244492	0.129199	-0.570000
C	2.119820	-2.508938	-0.786044
C	3.634179	-2.570648	-0.563044
C	1.780116	-3.436462	-1.961561
C	1.390874	-2.988567	0.469396
C	-1.651282	0.524827	-0.465592
C	3.210217	0.541068	0.173581
C	4.000307	1.180634	-0.792102
C	5.269953	1.604314	-0.412447
C	5.718663	1.448147	0.886775
C	4.889742	0.883173	1.839234
C	3.620536	0.417697	1.508812
C	2.743416	-0.150745	2.612499
C	3.417748	-1.310822	3.347687
C	2.339021	0.950805	3.597025
C	3.523358	1.477142	-2.205297
C	3.559181	2.981342	-2.489469
C	4.333985	0.709234	-3.251838
C	-1.821130	1.794272	0.380876
C	-2.488953	-0.633678	0.046156
C	-2.132208	-1.321307	1.195788
C	-2.952134	-2.343414	1.664471
C	-4.117380	-2.673177	0.999947
C	-4.476970	-1.979792	-0.155869
C	-3.656169	-0.965023	-0.637096
C	-3.261283	2.245388	0.397531
C	-3.809147	2.888728	-0.709317
C	-5.146602	3.259197	-0.721853
C	-5.952952	2.992267	0.377297
C	-5.412775	2.359443	1.488368
C	-4.074810	1.988174	1.496397
O	-5.636019	-2.363496	-0.745633
C	-6.060772	-1.660464	-1.904393
H	-0.034176	-0.619862	-2.517010
H	-0.382920	-1.838630	-1.280568
H	2.260933	-0.845034	-2.116802
H	0.736590	1.439804	0.752339
H	3.954514	-1.982216	0.296849
H	4.179038	-2.211854	-1.440809
H	3.932701	-3.606188	-0.386356
H	2.277820	-3.110432	-2.878931
H	0.706660	-3.490817	-2.155496
H	2.122214	-4.448331	-1.736549
H	1.640056	-2.382628	1.341520
H	0.304141	-2.983861	0.351582
H	1.686866	-4.016154	0.690715
H	-1.967051	0.752416	-1.487847
H	5.910353	2.085550	-1.143169
H	6.709699	1.789070	1.163181
H	5.235839	0.798817	2.863173
H	1.826660	-0.537762	2.166674
H	2.730202	-1.736283	4.081911
H	3.715609	-2.106250	2.660991
H	4.309546	-0.977103	3.882586
H	1.666091	0.549251	4.357628
H	1.830469	1.776766	3.093657
H	3.215651	1.361095	4.104229
H	2.478822	1.175294	-2.294619
H	3.140839	3.182431	-3.478000
H	2.976135	3.536996	-1.752168
H	4.581062	3.366812	-2.474878
H	3.938607	0.899730	-4.251733
H	4.309405	-0.369195	-3.076394
H	5.380875	1.022098	-3.238897
H	-1.489564	1.601826	1.404674
H	-1.193465	2.586441	-0.036642
H	-1.222848	-1.070352	1.732351
H	-2.677442	-2.885627	2.561897
H	-4.765118	-3.464388	1.358434
H	-3.916916	-0.417233	-1.534056
H	-3.183423	3.105146	-1.570148
H	-5.559465	3.759456	-1.590501
H	-6.997805	3.280776	0.368962
H	-6.034273	2.151907	2.352056
H	-3.658899	1.487846	2.365213
H	-7.003497	-2.112918	-2.201344
H	-6.218578	-0.600097	-1.686760
H	-5.336798	-1.763833	-2.717179

ωB97X energy = -1507.40988643 a.u.

(5*S*,1’*S*)-**10a**, Conf. A

C	3.277237	-0.482547	-1.121505
C	4.465555	-1.167149	-0.996121
C	5.521736	-0.608310	-0.270404
C	5.362632	0.638745	0.318868
C	4.151681	1.314168	0.184951
C	3.097120	0.772246	-0.530318
C	1.756647	1.470206	-0.690693
C	1.708223	2.909172	-0.164965
C	0.360163	3.556378	-0.363458
C	-0.103789	3.856231	-1.643250
C	-1.351342	4.435024	-1.827366
C	-2.153505	4.727339	-0.731334
C	-1.698886	4.438779	0.547108
C	-0.451371	3.855027	0.727324
N	0.706454	0.659399	-0.059806
C	-0.353988	0.183491	-0.648168
N	-1.119161	-0.555877	0.125376
C	-2.422647	-1.030187	-0.275186
C	-2.521524	-2.057349	-1.225766

C	-1.329864	-2.648344	-1.962420
C	-1.242614	-4.167136	-1.796921
C	-1.371836	-2.270346	-3.446237
C	-3.792799	-2.533293	-1.531290
C	-4.920295	-2.016153	-0.919280
C	-4.799206	-0.969863	-0.022741
C	-3.555268	-0.434692	0.297465
C	-3.497660	0.786191	1.202917
C	-4.337362	1.929856	0.627178
C	-3.945096	0.457332	2.629305
C	-0.520948	-0.648568	1.495180
C	-0.166353	-2.066541	2.002055
C	0.868735	-2.766136	1.120227
C	-1.425920	-2.932180	2.109672
C	0.402299	-1.890723	3.417362
C	0.696683	0.300461	1.361588
C	7.757849	-0.821032	0.514770
O	6.651926	-1.352160	-0.201722
H	2.469208	-0.932096	-1.691388
H	4.602627	-2.138315	-1.456609
H	6.162132	1.098991	0.883618
H	4.058446	2.284414	0.657359
H	1.503757	1.486586	-1.753920
H	1.970647	2.931040	0.895264
H	2.479241	3.474197	-0.694041
H	0.520326	3.644506	-2.506466
H	-1.695731	4.664463	-2.829401
H	-3.127151	5.181871	-0.874200
H	-2.314130	4.670592	1.409316
H	-0.099099	3.644459	1.732638
H	-0.589443	0.380615	-1.687317
H	-0.414535	-2.224631	-1.547931
H	-0.335474	-4.542655	-2.275359
H	-1.216192	-4.459028	-0.744751
H	-2.094490	-4.667010	-2.263166
H	-0.486009	-2.653336	-3.957281
H	-1.406263	-1.187542	-3.587762
H	-2.252458	-2.697195	-3.932370
H	-3.900470	-3.327430	-2.261301
H	-5.898943	-2.416072	-1.158093
H	-5.691412	-0.543946	0.422085
H	-2.468694	1.151114	1.239167
H	-4.224012	2.822523	1.246050
H	-4.021414	2.180718	-0.387745
H	-5.398957	1.673962	0.601886
H	-3.854364	1.338885	3.267442
H	-3.350705	-0.345549	3.072379
H	-4.990634	0.139440	2.642686
H	-1.240070	-0.227865	2.198306
H	0.494881	-2.935080	0.109787
H	1.805643	-2.207862	1.046453
H	1.107415	-3.742583	1.546982
H	-2.195421	-2.440892	2.711693
H	-1.175524	-3.876696	2.597523
H	-1.858296	-3.166406	1.137116
H	-0.313958	-1.384563	4.070299
H	1.334989	-1.322508	3.427988
H	0.612694	-2.871361	3.848036
H	0.585391	1.200411	1.967391
H	1.639398	-0.179048	1.620714
H	8.104457	0.114553	0.067839
H	7.505162	-0.654187	1.565237
H	8.545247	-1.567433	0.447342

ωB97X energy = -1507.41208871 a.u.

(5*S*,1'*S*)-**10a**, Conf. B

C	3.373716	-0.354642	-1.238906
C	4.570619	-1.030668	-1.149798
C	5.560993	-0.586167	-0.269085
C	5.329600	0.539855	0.510447
C	4.112284	1.208721	0.408682
C	3.122636	0.778722	-0.459341
C	1.774214	1.464257	-0.580588
C	1.697340	2.867593	0.032056
C	0.377779	3.542737	-0.246769
C	0.070284	3.980609	-1.534454
C	-1.147327	4.586915	-1.805173
C	-2.077215	4.768190	-0.788548
C	-1.779899	4.339856	0.496853
C	-0.561460	3.727152	0.763302
N	0.735359	0.596965	-0.005162
C	-0.382606	0.252437	-0.579876
N	-1.135763	-0.552172	0.139595
C	-2.481428	-0.927004	-0.225448
C	-2.691083	-1.782748	-1.317718
C	-1.585228	-2.270666	-2.240547
C	-1.524228	-3.797920	-2.315062
C	-1.750652	-1.670418	-3.640112
C	-3.997920	-2.178036	-1.586464
C	-5.054852	-1.748125	-0.804810
C	-4.827235	-0.866002	0.235899
C	-3.544829	-0.414526	0.531764
C	-3.374207	0.632750	1.621779
C	-4.237162	1.864799	1.336571
C	-3.694708	0.067545	3.008063
C	-0.444723	-0.875820	1.428516
C	-0.078665	-2.362359	1.653847
C	0.887221	-2.894966	0.594274
C	-1.341510	-3.229192	1.689739
C	0.581957	-2.446887	3.037248
C	0.773463	0.076969	1.365584
C	7.741612	-0.900862	0.630147
O	6.704957	-1.311540	-0.250383
H	2.616177	-0.711401	-1.930922
H	4.765074	-1.906944	-1.756750
H	6.077284	0.909389	1.199198
H	3.959843	2.083982	1.028918
H	1.527627	1.542286	-1.642035
H	1.867375	2.818591	1.110146
H	2.515214	3.453520	-0.393267
H	0.796584	3.855827	-2.332276

H	-1.368361	4.926018	-2.810890
H	-3.027079	5.246874	-0.997278
H	-2.496803	4.483711	1.297060
H	-0.333935	3.403660	1.774680
H	-0.669393	0.602962	-1.564486
H	-0.625152	-1.927561	-1.853641
H	-0.680288	-4.106806	-2.935743
H	-1.400151	-4.248238	-1.327843
H	-2.432098	-4.210091	-2.760830
H	-0.920717	-1.973944	-4.281679
H	-1.779533	-0.578670	-3.611496
H	-2.678019	-2.014727	-4.104106
H	-4.189945	-2.841037	-2.422540
H	-6.062249	-2.084552	-1.020866
H	-5.666173	-0.503147	0.818823
H	-2.337669	0.977223	1.618148
H	-4.048226	2.631312	2.091535
H	-4.010981	2.290189	0.356566
H	-5.302091	1.624528	1.369051
H	-3.517502	0.823937	3.775535
H	-3.087800	-0.808763	3.248455
H	-4.743745	-0.232629	3.068789
H	-1.104377	-0.579538	2.243113
H	0.448950	-2.872333	-0.404460
H	1.827508	-2.338091	0.565503
H	1.134213	-3.935110	0.817075
H	-2.060159	-2.854579	2.424004
H	-1.073292	-4.247896	1.977906
H	-1.842621	-3.280178	0.723607
H	-0.085377	-2.067669	3.815910
H	1.520690	-1.891619	3.090596
H	0.806662	-3.490052	3.267480
H	0.688225	0.903248	2.073544
H	1.720764	-0.429784	1.538565
H	8.092371	0.105104	0.384931
H	7.409014	-0.929758	1.671185
H	8.552640	-1.611062	0.490386

ωB97X energy = -1507.41180317 a.u.

(5*S*,1'*S*)-**10a**, Conf. C

C	3.430736	-0.011160	-1.085179
C	4.661945	-0.631160	-0.925315
C	5.546906	-0.153339	0.038233
C	5.185493	0.942882	0.819826
C	3.953915	1.546284	0.647848
C	3.054380	1.077036	-0.307931
C	1.672728	1.673655	-0.505824
C	1.487055	3.092882	0.042794
C	0.127469	3.656062	-0.288631
C	-0.185672	4.016604	-1.598435
C	-1.444738	4.503526	-1.917494
C	-2.411163	4.639955	-0.928453
C	-2.107991	4.290187	0.379280
C	-0.846927	3.799399	0.694539
N	0.666027	0.767480	0.067019
C	-0.354986	0.257793	-0.560832
N	-1.073246	-0.577018	0.159401
C	-2.332901	-1.123760	-0.284536
C	-2.350647	-2.064424	-1.325689
C	-1.119796	-2.480348	-2.116021
C	-0.899400	-3.994301	-2.091460
C	-1.211904	-1.973219	-3.558674
C	-3.579085	-2.616353	-1.675445
C	-4.742855	-2.253553	-1.021611
C	-4.704892	-1.287076	-0.033037
C	-3.508355	-0.681787	0.338436
C	-3.547067	0.446877	1.357311
C	-4.474695	1.572279	0.891062
C	-3.963911	-0.055601	2.741758
C	-0.461249	-0.733358	1.517588
C	0.037588	-2.151110	1.885731
C	0.599241	-2.060427	3.311817
C	1.129142	-2.650959	0.938459
C	-1.128466	-3.144780	1.906991
C	0.653813	0.340150	1.469463
C	7.169833	-1.818295	-0.472567
O	6.770138	-0.682416	0.281842
H	2.750189	-0.393376	-1.840756
H	4.916727	-1.473506	-1.554233
H	5.884188	1.308220	1.562996
H	3.703583	2.394839	1.273886
H	1.462632	1.693064	-1.577716
H	1.636506	3.104992	1.124761
H	2.271896	3.715078	-0.393319
H	0.566692	3.924162	-2.376231
H	-1.670756	4.782203	-2.940502
H	-3.394337	5.023497	-1.176076
H	-2.853569	4.399776	1.158618
H	-0.615885	3.538906	1.723135
H	-0.596250	0.501414	-1.588749
H	-0.239046	-2.019282	-1.667857
H	0.028622	-4.243665	-2.610532
H	-0.829419	-4.377434	-1.071005
H	-1.712909	-4.522060	-2.593964
H	-0.301548	-2.226157	-4.106273
H	-1.345169	-0.889452	-3.600189
H	-2.056823	-2.431049	-4.078706
H	-3.623445	-3.348349	-2.473903
H	-5.685848	-2.710457	-1.298547
H	-5.627182	-0.980357	0.447178
H	-2.550065	0.885541	1.435052
H	-4.445167	2.397163	1.606551
H	-4.169554	1.958587	-0.083987
H	-5.510403	1.233467	0.816758
H	-3.945697	0.762847	3.464378
H	-3.303025	-0.845773	3.106803
H	-4.978811	-0.460095	2.717537
H	-1.211563	-0.453558	2.256477
H	1.473169	-1.409001	3.379576
H	-0.156151	-1.692913	4.011689
H	0.907275	-3.053855	3.643236

H	0.758671	-2.771685	-0.080291
H	1.483734	-3.627662	1.274327
H	1.995765	-1.984725	0.908985
H	-1.552878	-3.315056	0.917740
H	-1.931817	-2.800486	2.564342
H	-0.777544	-4.106318	2.288007
H	0.431060	1.194118	2.110847
H	1.632702	-0.051000	1.740422
H	7.227003	-1.585091	-1.539030
H	8.158220	-2.087227	-0.108669
H	6.484387	-2.655558	-0.316443

ωB97X energy = -1507.41179187 a.u.

(5*S*,1’*S*)-**10a**, Conf. D

C	-4.120106	1.432335	0.966222
C	-5.366872	0.855366	0.820541
C	-5.590061	-0.080953	-0.188208
C	-4.549967	-0.427842	-1.046785
C	-3.305140	0.166396	-0.887600
C	-3.069513	1.098141	0.113815
C	-1.718724	1.765177	0.278973
C	-1.342743	2.674132	-0.897801
C	-0.017086	3.377987	-0.717046
C	0.997999	3.230745	-1.656708
C	2.227536	3.855894	-1.483380
C	2.456808	4.640631	-0.363724
C	1.445085	4.806370	0.574865
C	0.219695	4.182125	0.397791
N	-0.662057	0.784691	0.568539
C	0.057801	0.099577	-0.273848
N	0.918277	-0.721667	0.290687
C	1.652430	-1.731829	-0.435542
C	1.269984	-3.070999	-0.271777
C	0.029368	-3.501753	0.495589
C	-0.942089	-4.250427	-0.421452
C	0.382133	-4.350070	1.719441
C	2.045656	-4.047085	-0.889263
C	3.128475	-3.706932	-1.679840
C	3.436721	-2.374941	-1.890046
C	2.709402	-1.357897	-1.279089
C	3.070578	0.083626	-1.600152
C	4.523202	0.415845	-1.252892
C	2.789982	0.384924	-3.075958
C	0.848463	-0.585370	1.780672
C	2.131275	-0.065664	2.471526
C	2.537575	1.324039	1.978633
C	3.282646	-1.055100	2.266123
C	1.831430	-0.008886	3.976074
C	-0.380299	0.340415	1.937439
C	-7.120001	-1.543400	-1.279335
O	-6.840951	-0.598081	-0.255863
H	-3.961014	2.158295	1.757201
H	-6.182781	1.115979	1.484123
H	-4.692813	-1.151998	-1.837414
H	-2.515355	-0.120618	-1.575232
H	-1.762249	2.383660	1.177447
H	-1.343530	2.106946	-1.832584
H	-2.151002	3.403684	-0.989124
H	0.825540	2.631303	-2.545666
H	3.005713	3.730895	-2.228806
H	3.414980	5.127667	-0.224663
H	1.611109	5.426867	1.448189
H	-0.561890	4.329409	1.136843
H	-0.042061	0.174497	-1.349131
H	-0.499542	-2.611265	0.838288
H	-1.859082	-4.488736	0.121807
H	-1.207974	-3.647138	-1.292177
H	-0.511503	-5.188869	-0.778070
H	-0.521947	-4.603545	2.276917
H	1.064429	-3.828832	2.395624
H	0.863870	-5.283912	1.419815
H	1.785082	-5.092023	-0.763440
H	3.719844	-4.482028	-2.153344
H	4.262619	-2.117006	-2.543404
H	2.438443	0.747289	-1.008979
H	4.714860	1.475782	-1.437309
H	4.747030	0.211201	-0.203906
H	5.219969	-0.160420	-1.865666
H	2.999443	1.434644	-3.294657
H	1.749065	0.181456	-3.340180
H	3.422163	-0.223649	-3.726771
H	0.633406	-1.568242	2.198770
H	2.778516	1.328164	0.914137
H	1.759625	2.072974	2.148492
H	3.428823	1.655233	2.515902
H	3.007110	-2.058117	2.604528
H	4.147529	-0.732861	2.850078
H	3.593643	-1.125900	1.224401
H	1.521663	-0.986799	4.354593
H	1.052920	0.715418	4.224036
H	2.734204	0.287386	4.513559
H	-1.254244	-0.179982	2.333784
H	-0.176409	1.207835	2.563373
H	-8.165152	-1.816327	-1.157725
H	-6.971577	-1.106494	-2.270418
H	-6.497273	-2.435798	-1.174466

ωB97X energy = -1507.41073688 a.u.

(5*S*,1’*S*)-**10a**, Conf. E

C	3.280170	0.297513	1.327761
C	4.475423	-0.354379	1.544636
C	5.485072	-0.311029	0.579586
C	5.277419	0.392584	-0.600482
C	4.063672	1.042173	-0.802630
C	3.053428	1.006709	0.145422
C	1.743052	1.718110	-0.120689
C	1.304921	2.666828	1.001902
C	0.026519	3.407799	0.686002
C	-1.107652	3.244075	1.474797
C	-2.292225	3.902704	1.165887

C	-2.354839	4.739015	0.061525
C	-1.223819	4.921443	-0.725276
C	-0.044461	4.261757	-0.414337
N	0.672815	0.765634	-0.468545
C	-0.058816	0.053428	0.339219
N	-0.937534	-0.720910	-0.262238
C	-1.685553	-1.748420	0.424995
C	-2.743312	-1.389883	1.274139
C	-3.103191	0.046369	1.618317
C	-4.556922	0.380285	1.276868
C	-2.820879	0.333482	3.096434
C	-3.476324	-2.417514	1.859912
C	-3.174064	-3.745452	1.617423
C	-2.093416	-4.071837	0.817938
C	-1.311144	-3.084971	0.226310
C	-0.073221	-3.502134	-0.552669
C	0.887380	-4.295295	0.337912
C	-0.433254	-4.301228	-1.807204
C	-0.855457	-0.527553	-1.745335
C	-2.129431	0.016294	-2.433659
C	-1.806854	0.130114	-3.930320
C	-2.547906	1.385679	-1.896321
C	-3.281267	-0.982192	-2.281828
C	0.375983	0.402680	-1.858094
C	7.678758	-0.965116	-0.068162
O	6.623716	-0.979540	0.883390
H	2.521215	0.251196	2.102233
H	4.652951	-0.902028	2.462521
H	6.040565	0.446021	-1.365065
H	3.913785	1.589565	-1.727869
H	1.868458	2.313029	-1.027081
H	1.198242	2.125417	1.945081
H	2.126933	3.371470	1.148065
H	-1.064550	2.605663	2.352612
H	-3.166188	3.763361	1.793375
H	-3.277195	5.253696	-0.181569
H	-1.259780	5.584026	-1.582592
H	0.834061	4.421919	-1.032093
H	0.044625	0.071473	1.416681
H	-2.471563	0.715366	1.033175
H	-4.750426	1.438109	1.470617
H	-4.782784	0.183577	0.226788
H	-5.251460	-0.202027	1.886447
H	-3.037875	1.379702	3.326110
H	-1.777890	0.135612	3.356784
H	-3.447254	-0.285856	3.742804
H	-4.302119	-2.171383	2.517945
H	-3.769349	-4.529065	2.071625
H	-1.839341	-5.114868	0.665683
H	0.466652	-2.605697	-0.861424
H	1.803808	-4.521846	-0.211238
H	1.156255	-3.728731	1.232007
H	0.446326	-5.242461	0.656425
H	0.469141	-4.546895	-2.370920
H	-1.106042	-3.746505	-2.465944
H	-0.928864	-5.238458	-1.542710
H	-0.636320	-1.495251	-2.196040
H	-1.028643	0.867475	-4.138269
H	-1.485459	-0.831269	-4.340246
H	-2.702846	0.441722	-4.470476
H	-2.821696	1.346347	-0.841204
H	-3.421549	1.741051	-2.447005
H	-1.764758	2.140134	-2.009860
H	-3.600767	-1.098831	-1.246649
H	-3.001528	-1.968837	-2.662241
H	-4.141757	-0.635569	-2.858278
H	1.243183	-0.096610	-2.294104
H	0.169092	1.306322	-2.429457
H	8.029280	0.053740	-0.252931
H	8.484334	-1.550694	0.367446
H	7.365647	-1.422073	-1.010673

ωB97X energy = -1507.41071224 a.u.

(5*S*,1’*S*)-**10a**, Conf. F

C	3.312252	0.382252	-1.206332
C	4.526703	-0.249315	-1.058872
C	5.337651	0.039367	0.043124
C	4.908337	0.966307	0.982076
C	3.673746	1.592232	0.818999
C	2.858894	1.314388	-0.265420
C	1.509854	1.984954	-0.479780
C	1.102443	2.964357	0.628253
C	-0.282156	3.540437	0.457245
C	-1.295115	3.215015	1.354839
C	-2.571471	3.745534	1.212097
C	-2.851454	4.607939	0.162743
C	-1.848324	4.938472	-0.740954
C	-0.573886	4.410835	-0.592295
N	0.465990	0.964321	-0.688705
C	0.129715	0.035615	0.159126
N	-0.893386	-0.702908	-0.216719
C	-1.216749	-1.944828	0.448272
C	-2.044145	-1.919123	1.578477
C	-2.535348	-0.632953	2.220560
C	-4.056892	-0.602642	2.374208
C	-1.850196	-0.413378	3.572694
C	-2.382342	-3.138655	2.156778
C	-1.911892	-4.332359	1.637811
C	-1.051028	-4.325538	0.554262
C	-0.664651	-3.132754	-0.049030
C	0.370181	-3.170156	-1.163069
C	1.684417	-3.774442	-0.660611
C	-0.141556	-3.918751	-2.394955
C	-1.306053	-0.318200	-1.604981
C	-2.811246	-0.118877	-1.888183
C	-3.443836	0.986638	-1.044017
C	-3.575710	-1.430321	-1.679041
C	-2.914157	0.260636	-3.372817
C	-0.440665	0.942840	-1.842128
C	7.373146	-0.367384	1.205112
O	6.514083	-0.630044	0.104478

H	2.701569	0.145104	-2.072136
H	4.872988	-0.970832	-1.789406
H	5.512503	1.213839	1.844488
H	3.370578	2.310093	1.570676
H	1.542579	2.528474	-1.427244
H	1.160325	2.461108	1.596773
H	1.840939	3.769364	0.644349
H	-1.080068	2.549455	2.186443
H	-3.345981	3.482597	1.924069
H	-3.845281	5.025173	0.048790
H	-2.057640	5.617726	-1.559487
H	0.205251	4.688762	-1.295994
H	0.656332	-0.135037	1.091225
H	-2.250620	0.201110	1.577631
H	-4.373171	0.369998	2.758404
H	-4.563402	-0.771399	1.421282
H	-4.401387	-1.363816	3.077894
H	-2.172299	0.535415	4.008444
H	-0.762391	-0.390912	3.472118
H	-2.105271	-1.210307	4.275784
H	-3.023318	-3.153330	3.031075
H	-2.198402	-5.272088	2.095709
H	-0.654149	-5.263719	0.182607
H	0.601379	-2.146996	-1.464433
H	2.441585	-3.726968	-1.446421
H	2.060785	-3.231930	0.209622
H	1.558858	-4.822569	-0.379717
H	0.606956	-3.893880	-3.189553
H	-1.063688	-3.478577	-2.782812
H	-0.345913	-4.966150	-2.160344
H	-0.968610	-1.120714	-2.266057
H	-3.383806	0.772652	0.022946
H	-2.985434	1.963357	-1.214256
H	-4.501224	1.077006	-1.302612
H	-3.115070	-2.254159	-2.231523
H	-4.598377	-1.315075	-2.044614
H	-3.629757	-1.717591	-0.628493
H	-2.480311	-0.512521	-4.012776
H	-2.415192	1.207840	-3.591873
H	-3.964801	0.373003	-3.646843
H	0.131678	0.887089	-2.767076
H	-1.028835	1.861578	-1.846738
H	8.246897	-0.997980	1.061739
H	7.680892	0.681515	1.223411
H	6.890617	-0.624096	2.151936

ωB97X energy = -1507.40984394 a.u.

(5*S*,1’*S*)-**10a**, Conf. G

C	3.165055	1.907855	0.983001
C	4.456182	1.396783	1.101029
C	5.025789	0.716344	0.033627
C	4.298408	0.558255	-1.150400
C	3.026092	1.074183	-1.251028
C	2.432979	1.756190	-0.182201
C	1.015001	2.274031	-0.347348
C	0.548507	3.233191	0.752211
C	-0.837980	3.777501	0.516285
C	-1.069691	4.709711	-0.494236
C	-2.349171	5.185838	-0.737931
C	-3.417735	4.739682	0.031260
C	-3.195963	3.819595	1.045533
C	-1.912894	3.341501	1.284048
N	0.085830	1.133599	-0.495580
C	0.118000	0.031597	0.196411
N	-0.798506	-0.854533	-0.139036
C	-0.686048	-2.237451	0.272100
C	-1.270824	-2.644931	1.477977
C	-1.917659	-1.681233	2.457744
C	-3.357030	-2.075327	2.794550
C	-1.076067	-1.572681	3.732994
C	-1.198124	-3.995450	1.806124
C	-0.558391	-4.900806	0.978035
C	0.064408	-4.461837	-0.176962
C	0.034405	-3.120178	-0.544683
C	0.825009	-2.678484	-1.767120
C	2.319506	-2.949603	-1.571863
C	0.309567	-3.337762	-3.047734
C	-1.538840	-0.368756	-1.347571
C	-3.076111	-0.507291	-1.360458
C	-3.547273	0.094543	-2.692337
C	-3.758950	0.227534	-0.207789
C	-3.476829	-1.986498	-1.342112
C	-1.023333	1.086957	-1.457270
C	7.050660	0.325119	1.218826
O	6.268386	0.177561	0.041771
H	2.748794	2.432399	1.834367
H	4.994469	1.538514	2.028395
H	4.755477	0.031622	-1.979855
H	2.475846	0.945889	-2.178837
H	0.953338	2.794078	-1.306753
H	0.579870	2.724097	1.718438
H	1.268075	4.054343	0.794804
H	-0.239394	5.069815	-1.094558
H	-2.513194	5.911927	-1.526049
H	-4.417409	5.114250	-0.156209
H	-4.022814	3.469851	1.653474
H	-1.745323	2.618122	2.076424
H	0.858553	-0.161176	0.963800
H	-1.938734	-0.691386	2.000063
H	-3.811543	-1.317818	3.436956
H	-3.972176	-2.172063	1.897135
H	-3.392466	-3.027303	3.329285
H	-1.514427	-0.839074	4.413223
H	-0.051478	-1.264517	3.512767
H	-1.032194	-2.531810	4.255057
H	-1.645963	-4.343377	2.730153
H	-0.522801	-5.950259	1.246878
H	0.602540	-5.171579	-0.795450
H	0.724181	-1.598672	-1.886355
H	2.881645	-2.577797	-2.431609
H	2.698342	-2.449829	-0.677113

H	2.520421	-4.019001	-1.475149
H	0.858581	-2.962150	-3.913617
H	-0.753375	-3.138313	-3.205420
H	0.442493	-4.421580	-3.011277
H	-1.168931	-0.950963	-2.195933
H	-3.341291	1.165727	-2.757509
H	-3.069257	-0.399432	-3.542720
H	-4.626243	-0.038596	-2.790957
H	-3.445209	-0.155524	0.763207
H	-4.839617	0.088263	-0.283302
H	-3.568575	1.302617	-0.225091
H	-3.237129	-2.472202	-0.395747
H	-2.980948	-2.542037	-2.143281
H	-4.554702	-2.072658	-1.495717
H	-0.657747	1.326876	-2.454969
H	-1.778867	1.819586	-1.170565
H	6.567826	-0.151897	2.075857
H	7.996788	-0.169810	1.015263
H	7.232304	1.380150	1.440383

ωB97X energy = -1507.40977219 a.u.

(5*S*,1'*S*)-**10a**, Conf. H

C	3.687801	0.497106	1.100759
C	4.628367	1.507750	0.927588
C	4.459777	2.426345	-0.101591
C	3.350954	2.323322	-0.946069
C	2.432518	1.310827	-0.763166
C	2.590605	0.380290	0.265164
C	1.615053	-0.763284	0.446161
C	1.743302	-1.837695	-0.642159
C	3.115592	-2.464671	-0.628638
C	3.436312	-3.424601	0.327845
C	4.706248	-3.982246	0.373092
C	5.672532	-3.586197	-0.542521
C	5.360324	-2.633318	-1.502573
C	4.089087	-2.076136	-1.543581
N	0.244060	-0.244779	0.528046
C	-0.791939	-0.560880	-0.195371
N	-1.915151	0.015662	0.180302
C	-3.101753	0.007110	-0.644575
C	-3.932789	-1.121663	-0.641754
C	-3.596653	-2.409469	0.091128
C	-4.704263	-2.833648	1.057465
C	-3.295462	-3.530186	-0.908897
C	-5.102723	-1.062571	-1.392875
C	-5.431648	0.066988	-2.121236
C	-4.565834	1.145816	-2.152410
C	-3.372327	1.131001	-1.437245
C	-2.407646	2.296539	-1.594336
C	-1.997371	2.467613	-3.059869
C	-2.989791	3.598392	-1.039593
C	-1.658182	0.933973	1.332762
C	-2.538727	0.755019	2.590178
C	-2.411411	-0.636352	3.209923
C	-4.007454	1.045328	2.264113
C	-2.062787	1.809650	3.599434
C	-0.146692	0.704439	1.576853
C	6.452084	3.596458	0.468250
O	5.307610	3.451866	-0.360641
H	3.827088	-0.215529	1.907454
H	5.473748	1.565125	1.599853
H	3.230266	3.050883	-1.739938
H	1.576207	1.250609	-1.429253
H	1.813736	-1.234044	1.414096
H	0.992709	-2.614271	-0.470243
H	1.551667	-1.393422	-1.622517
H	2.683180	-3.742941	1.042446
H	4.940682	-4.730177	1.122004
H	6.664302	-4.022484	-0.509693
H	6.108106	-2.321818	-2.223010
H	3.850848	-1.328576	-2.293637
H	-0.748289	-1.228527	-1.046089
H	-2.690848	-2.248748	0.676544
H	-4.395838	-3.723138	1.611252
H	-4.935371	-2.048243	1.780495
H	-5.624753	-3.078596	0.522876
H	-2.991779	-4.436164	-0.380077
H	-2.494140	-3.251349	-1.597298
H	-4.179211	-3.769118	-1.505783
H	-5.768793	-1.918035	-1.408171
H	-6.356195	0.097175	-2.686152
H	-4.811960	2.009093	-2.760593
H	-1.490999	2.073300	-1.045057
H	-1.245231	3.254702	-3.146440
H	-1.574758	1.544805	-3.462710
H	-2.849335	2.750265	-3.682286
H	-2.260892	4.406544	-1.128676
H	-3.266908	3.505395	0.013440
H	-3.885370	3.890679	-1.593118
H	-1.821582	1.952368	0.975723
H	-2.736576	-1.418351	2.523532
H	-1.390537	-0.863014	3.524838
H	-3.043148	-0.695695	4.098765
H	-4.122954	2.023623	1.789048
H	-4.588654	1.053649	3.188703
H	-4.444913	0.296372	1.603684
H	-2.146821	2.817964	3.184992
H	-1.027249	1.651968	3.909231
H	-2.683623	1.762515	4.496076
H	0.436864	1.619306	1.480820
H	0.061195	0.258478	2.549884
H	7.095841	2.714686	0.410010
H	6.989534	4.462048	0.089306
H	6.166873	3.773297	1.508818

ωB97X energy = -1507.40966697 a.u.

(5*S*,1'*S*)-**10a**, Conf. I

C	2.365435	1.294432	-0.476746
C	3.221405	2.384967	-0.563083
C	4.292283	2.487859	0.321377

C	4.487914	1.499027	1.285056
C	3.623664	0.423814	1.358344
C	2.552601	0.303757	0.476353
C	1.643543	-0.907121	0.541952
C	1.821080	-1.861158	-0.646268
C	3.220900	-2.422478	-0.693746
C	3.603564	-3.435021	0.182752
C	4.897027	-3.937269	0.162004
C	5.825633	-3.432465	-0.739302
C	5.452362	-2.425132	-1.618139
C	4.157716	-1.923688	-1.593371
N	0.245112	-0.481717	0.677819
C	-0.723119	-0.592312	-0.185292
N	-1.872517	-0.090127	0.218254
C	-2.965160	0.165672	-0.693046
C	-3.802187	-0.887805	-1.085721
C	-3.558519	-2.339328	-0.707788
C	-4.762786	-2.969561	-0.005702
C	-3.170640	-3.154488	-1.945838
C	-4.880473	-0.580836	-1.910104
C	-5.113353	0.714924	-2.335743
C	-4.238092	1.726253	-1.981891
C	-3.133751	1.471404	-1.174785
C	-2.146595	2.597426	-0.906756
C	-1.537780	3.107633	-2.215870
C	-2.789011	3.741292	-0.119537
C	-1.724669	0.487908	1.590156
C	-2.666758	-0.073583	2.679070
C	-2.497242	-1.578650	2.886738
C	-4.124352	0.244384	2.331652
C	-2.323136	0.667337	3.978970
C	-0.217666	0.253839	1.860167
C	5.030460	4.532801	-0.647959
O	5.185203	3.507927	0.323767
H	1.536580	1.234730	-1.177049
H	3.043127	3.138628	-1.318142
H	5.321520	1.592625	1.970860
H	3.786718	-0.338210	2.113786
H	1.871829	-1.459864	1.457697
H	1.100928	-2.679223	-0.555257
H	1.614512	-1.333396	-1.581121
H	2.880921	-3.839488	0.885436
H	5.179575	-4.727179	0.848671
H	6.835268	-3.826559	-0.758604
H	6.170220	-2.028709	-2.327254
H	3.871398	-1.135647	-2.282756
H	-0.605925	-1.044239	-1.161444
H	-2.716283	-2.380015	-0.016489
H	-4.527934	-3.994686	0.288933
H	-5.045001	-2.416166	0.892579
H	-5.632265	-3.005271	-0.666030
H	-2.936906	-4.182995	-1.662782
H	-2.298464	-2.732170	-2.450701
H	-3.991103	-3.181941	-2.667110
H	-5.549333	-1.373074	-2.227151
H	-5.967355	0.933202	-2.966495
H	-4.403235	2.730149	-2.357003
H	-1.315457	2.207020	-0.317383
H	-0.785113	3.870373	-2.004409
H	-1.057275	2.297186	-2.768494
H	-2.296451	3.555473	-2.861635
H	-2.047593	4.512112	0.100513
H	-3.211846	3.394843	0.826973
H	-3.595641	4.205242	-0.692252
H	-1.926632	1.557455	1.519393
H	-2.754710	-2.144337	1.990373
H	-1.479961	-1.848960	3.179748
H	-3.162624	-1.911842	3.685918
H	-4.266748	1.315897	2.164730
H	-4.770113	-0.052277	3.161032
H	-4.465541	-0.282840	1.441042
H	-2.430116	1.748477	3.855799
H	-1.307767	0.460920	4.324013
H	-3.007213	0.350968	4.768630
H	0.342914	1.184939	1.949976
H	-0.035005	-0.348078	2.749708
H	4.070918	5.044001	-0.533206
H	5.838243	5.238622	-0.471860
H	5.113347	4.130800	-1.661245

ωB97X energy = -1507.40960794 a.u.

(5*S*,1’*S*)-**10a**, Conf. J

C	3.157750	1.302312	-0.936902
C	4.446778	0.829017	-0.745743
C	5.039992	0.951589	0.509519
C	4.331742	1.552971	1.547102
C	3.044434	2.015014	1.339237
C	2.433872	1.893731	0.093065
C	1.008688	2.343480	-0.182569
C	0.381347	3.212977	0.912380
C	-1.028637	3.637516	0.585166
C	-2.114629	3.051598	1.228383
C	-3.414957	3.414896	0.899548
C	-3.643148	4.369449	-0.080909
C	-2.565072	4.964735	-0.725522
C	-1.268377	4.601986	-0.392528
N	0.164927	1.164105	-0.466633
C	0.077399	0.102314	0.280226
N	-0.754949	-0.816493	-0.168159
C	-0.717668	-2.168449	0.345688
C	-1.542759	-2.513635	1.423578
C	-2.399250	-1.507372	2.172727
C	-3.870620	-1.922515	2.224456
C	-1.850129	-1.285005	3.585396
C	-1.524448	-3.837718	1.851480
C	-0.709153	-4.776279	1.243777
C	0.140975	-4.395489	0.220458
C	0.172308	-3.081675	-0.236600
C	1.201467	-2.694962	-1.288216
C	2.621861	-2.907647	-0.755962
C	0.988158	-3.449178	-2.601661

C	-1.241164	-0.415118	-1.528677
C	-2.734011	-0.618404	-1.863354
C	-3.672984	0.160744	-0.942746
C	-3.090245	-2.108321	-1.830324
C	-2.916105	-0.119733	-3.304579
C	-0.759648	1.053151	-1.601120
C	7.045752	-0.102465	-0.219486
O	6.289780	0.522528	0.808575
H	2.708997	1.200775	-1.920513
H	4.973065	0.375374	-1.574894
H	4.805113	1.649221	2.516913
H	2.524398	2.476728	2.169869
H	1.001874	2.908463	-1.117487
H	0.384580	2.666589	1.858856
H	1.019793	4.089887	1.043025
H	-1.943823	2.305374	1.999217
H	-4.250227	2.948109	1.409418
H	-4.656832	4.653444	-0.339087
H	-2.735572	5.717672	-1.486601
H	-0.431229	5.077090	-0.895231
H	0.659333	-0.033443	1.184872
H	-2.337959	-0.551294	1.651236
H	-4.462174	-1.141292	2.707051
H	-4.280772	-2.089241	1.225896
H	-4.003577	-2.841776	2.799524
H	-2.441713	-0.527253	4.104091
H	-0.810638	-0.949768	3.563251
H	-1.893482	-2.206081	4.172011
H	-2.156188	-4.137401	2.680097
H	-0.718133	-5.804609	1.586384
H	0.808948	-5.128268	-0.218483
H	1.111061	-1.628565	-1.500382
H	3.352402	-2.560108	-1.490290
H	2.783492	-2.355308	0.172940
H	2.818384	-3.964392	-0.560527
H	1.713570	-3.119284	-3.348125
H	-0.014245	-3.283336	-3.004342
H	1.117540	-4.524950	-2.461666
H	-0.677382	-1.017120	-2.247254
H	-3.541359	-0.112208	0.104295
H	-3.540185	1.241150	-1.026942
H	-4.707840	-0.059941	-1.213661
H	-2.410909	-2.693085	-2.457280
H	-4.102007	-2.247442	-2.217267
H	-3.059755	-2.522957	-0.822322
H	-2.263895	-0.659301	-3.996764
H	-2.710525	0.949131	-3.400789
H	-3.948382	-0.284793	-3.619091
H	-0.239733	1.275805	-2.531615
H	-1.573565	1.768272	-1.468906
H	6.547109	-1.004963	-0.583218
H	7.219428	0.582644	-1.053456
H	7.997803	-0.372151	0.230450

ωB97X energy = -1507.40943414 a.u.

(5*S*,1’*S*)-**10a**, Conf. K

C	-2.181267	0.990379	0.909552
C	-2.640945	2.282007	1.045411
C	-3.169841	2.958561	-0.058391
C	-3.224655	2.322958	-1.292672
C	-2.746319	1.020886	-1.413067
C	-2.224054	0.339385	-0.327283
C	-1.725938	-1.078823	-0.480424
C	-2.461344	-2.074756	0.430793
C	-3.944622	-2.084362	0.155819
C	-4.446596	-2.733060	-0.970222
C	-5.806648	-2.722700	-1.246943
C	-6.685229	-2.063735	-0.396717
C	-6.195374	-1.419089	0.730787
C	-4.833744	-1.429207	1.002705
N	-0.271739	-1.143473	-0.220585
C	0.560312	-0.141877	-0.304400
N	1.822413	-0.462417	-0.127494
C	2.899673	0.484551	-0.286675
C	3.030544	1.540871	0.628148
C	2.041766	1.825930	1.748887
C	2.720176	1.881933	3.119368
C	1.274807	3.123622	1.474323
C	4.114926	2.398288	0.471368
C	5.034410	2.217327	-0.546143
C	4.856167	1.196733	-1.462699
C	3.774201	0.326529	-1.370706
C	3.568777	-0.706340	-2.467762
C	3.451410	-0.032017	-3.837578
C	4.681390	-1.756824	-2.476047
C	1.942704	-1.927629	0.146634
C	2.502598	-2.323288	1.534605
C	3.920572	-1.774068	1.721361
C	2.591485	-3.855768	1.548707
C	1.612107	-1.851865	2.685274
C	0.489097	-2.397179	-0.112574
C	-4.135878	4.955786	-0.918470
O	-3.599233	4.221546	0.173514
H	-1.776483	0.483995	1.780898
H	-2.604199	2.790076	2.001765
H	-3.629559	2.820159	-2.163741
H	-2.790436	0.534471	-2.382048
H	-1.876037	-1.390902	-1.518725
H	-2.062403	-3.077026	0.260593
H	-2.272006	-1.819459	1.476394
H	-3.767620	-3.259632	-1.634496
H	-6.181955	-3.234044	-2.126191
H	-7.748071	-2.056723	-0.609837
H	-6.874551	-0.905835	1.402131
H	-4.456971	-0.922103	1.885274
H	0.241720	0.871064	-0.511935
H	1.307424	1.020997	1.788510
H	1.969627	2.022938	3.900117
H	3.266830	0.961837	3.337625
H	3.424333	2.714363	3.181256
H	0.526115	3.290909	2.251979

H	0.760411	3.096662	0.510226
H	1.951571	3.981447	1.465628
H	4.243570	3.222535	1.163770
H	5.880584	2.888359	-0.637864
H	5.560137	1.086013	-2.279913
H	2.618653	-1.213744	-2.295896
H	3.217273	-0.777666	-4.600340
H	2.660793	0.720922	-3.840896
H	4.385841	0.455542	-4.124361
H	4.489971	-2.506306	-3.246842
H	4.765302	-2.271492	-1.515656
H	5.648829	-1.295308	-2.688681
H	2.610684	-2.354995	-0.600854
H	4.571534	-2.059153	0.890284
H	3.940852	-0.688036	1.808398
H	4.350235	-2.187961	2.636158
H	1.616713	-4.336182	1.440680
H	3.014840	-4.186176	2.499080
H	3.241358	-4.218876	0.747907
H	0.607360	-2.278414	2.638768
H	1.520097	-0.765039	2.711730
H	2.050975	-2.163992	3.635241
H	0.390236	-2.955744	-1.045098
H	0.104714	-3.005841	0.701540
H	-5.024212	4.465639	-1.325364
H	-4.410698	5.928663	-0.519092
H	-3.392262	5.083981	-1.709512

ωB97X energy = -1507.40916792 a.u.

(5*S*,1’*S*)-**10a**, Conf. L

C	-3.156419	0.764860	0.958622
C	-3.883628	1.949887	0.924690
C	-3.941821	2.685585	-0.254339
C	-3.270484	2.224157	-1.388085
C	-2.552250	1.047863	-1.333882
C	-2.481075	0.296555	-0.159146
C	-1.625689	-0.955276	-0.136596
C	-2.172818	-2.122912	0.694272
C	-3.458148	-2.669242	0.120929
C	-3.439610	-3.384822	-1.075290
C	-4.612485	-3.883234	-1.622798
C	-5.825416	-3.675720	-0.977939
C	-5.854364	-2.970422	0.216496
C	-4.677671	-2.470756	0.760289
N	-0.263194	-0.615489	0.306816
C	0.810292	-0.665834	-0.428555
N	1.887368	-0.176448	0.148658
C	3.215712	-0.333216	-0.394941
C	3.579703	0.376921	-1.548962
C	2.622042	1.246072	-2.349128
C	3.137515	2.676967	-2.516038
C	2.337027	0.613345	-3.714848
C	4.887363	0.239768	-2.004441
C	5.798049	-0.566468	-1.345650
C	5.400695	-1.292546	-0.237498
C	4.098580	-1.213185	0.246741
C	3.694194	-2.111717	1.405509
C	3.924316	-3.585582	1.059987
C	4.429896	-1.737612	2.694459
C	1.534251	0.375249	1.494751
C	1.718342	1.900920	1.672579
C	1.330978	2.225468	3.122148
C	0.842211	2.713633	0.717511
C	3.189452	2.284662	1.485906
C	0.072898	-0.111915	1.643916
C	-5.316850	4.359061	0.731121
O	-4.622068	3.848102	-0.398209
H	-3.132840	0.211852	1.891276
H	-4.396428	2.279622	1.818093
H	-3.328856	2.801483	-2.303106
H	-2.035675	0.702588	-2.224316
H	-1.509662	-1.299723	-1.166391
H	-1.411207	-2.906812	0.702132
H	-2.326514	-1.819666	1.732013
H	-2.495021	-3.563742	-1.580719
H	-4.579201	-4.439270	-2.552918
H	-6.742477	-4.066762	-1.403421
H	-6.795664	-2.808441	0.729373
H	-4.710256	-1.924520	1.697429
H	0.821775	-1.084419	-1.427557
H	1.672159	1.305889	-1.817318
H	2.397544	3.278943	-3.047785
H	3.330377	3.153028	-1.552257
H	4.063346	2.701397	-3.094993
H	1.612193	1.216286	-4.265910
H	1.934636	-0.397789	-3.616580
H	3.248552	0.551328	-4.314180
H	5.198757	0.777037	-2.893066
H	6.815456	-0.646088	-1.710734
H	6.110204	-1.951453	0.250240
H	2.622617	-2.001755	1.578471
H	3.558779	-4.219956	1.870264
H	3.398715	-3.863293	0.144095
H	4.985839	-3.801640	0.920668
H	4.088977	-2.364246	3.521226
H	4.266871	-0.692439	2.969327
H	5.506703	-1.885704	2.582418
H	2.166680	-0.114119	2.234663
H	0.277859	2.024880	3.330737
H	1.935877	1.652234	3.829871
H	1.504740	3.285899	3.313921
H	1.097492	2.531042	-0.327424
H	0.990149	3.778851	0.906713
H	-0.224099	2.507491	0.847816
H	3.532008	2.139223	0.461899
H	3.838081	1.700295	2.144628
H	3.325227	3.339404	1.734676
H	-0.024768	-0.918620	2.371336
H	-0.609023	0.690433	1.917478
H	-4.628389	4.573685	1.552793
H	-5.786319	5.282578	0.402040

H	-6.086612	3.660666	1.069687

ωB97X energy = -1507.40911284 a.u.

(5*S*,1’*S*)-**10a**, Conf. M

C	-2.563033	1.176638	-1.047048
C	-3.231906	2.390573	-0.945698
C	-3.828649	2.741947	0.260804
C	-3.751767	1.868697	1.346806
C	-3.084869	0.666127	1.225827
C	-2.474276	0.299959	0.024706
C	-1.669403	-0.976420	-0.127315
C	-2.237505	-2.209922	0.584564
C	-3.553292	-2.646515	-0.012427
C	-4.757369	-2.427110	0.649148
C	-5.961614	-2.822044	0.080280
C	-5.975709	-3.442482	-1.160717
C	-4.778696	-3.669094	-1.828511
C	-3.578342	-3.274389	-1.256539
N	-0.281716	-0.741796	0.306504
C	0.749438	-0.630894	-0.480782
N	1.855081	-0.246502	0.120695
C	3.149994	-0.277606	-0.517957
C	4.060636	-1.270271	-0.129703
C	3.712158	-2.398483	0.828336
C	3.911698	-3.763111	0.163187
C	4.516406	-2.307439	2.127244
C	5.333091	-1.237306	-0.691267
C	5.672580	-0.289640	-1.639919
C	4.731961	0.633482	-2.060373
C	3.453098	0.664202	-1.512673
C	2.457861	1.681512	-2.048323
C	2.973443	3.116663	-1.920285
C	2.094724	1.367880	-3.503017
C	1.582934	0.008128	1.570448
C	1.818860	1.453389	2.068993
C	1.497182	1.454844	3.570228
C	0.929505	2.473156	1.355310
C	3.291729	1.841579	1.906547
C	0.116081	-0.474209	1.693319
C	-4.611288	4.816681	-0.603326
O	-4.504539	3.896313	0.473888
H	-2.101093	0.915797	-1.994518
H	-3.280427	3.042645	-1.807383
H	-4.226185	2.150246	2.279306
H	-3.049896	0.008429	2.087429
H	-1.598172	-1.204504	-1.193035
H	-1.504052	-3.014479	0.488071
H	-2.358071	-2.016528	1.652511
H	-4.756057	-1.945745	1.621776
H	-6.890844	-2.645454	0.610076
H	-6.914555	-3.752258	-1.605198
H	-4.779768	-4.159789	-2.795225
H	-2.645831	-3.464923	-1.779874
H	0.705645	-0.845016	-1.541973
H	2.651783	-2.331631	1.076003
H	3.576118	-4.556219	0.834728
H	3.343324	-3.837273	-0.766088
H	4.963902	-3.945084	-0.066727
H	4.211212	-3.095784	2.818464
H	4.379336	-1.345108	2.626978
H	5.584620	-2.428050	1.930954
H	6.064222	-1.981344	-0.395671
H	6.667377	-0.283854	-2.070109
H	4.996684	1.349795	-2.829854
H	1.538600	1.614179	-1.465728
H	2.207104	3.819361	-2.254517
H	3.231133	3.363544	-0.888109
H	3.861387	3.276128	-2.535952
H	1.343944	2.074293	-3.863583
H	1.691739	0.358038	-3.611775
H	2.970924	1.448090	-4.150983
H	2.235434	-0.642702	2.151994
H	0.449126	1.226723	3.775621
H	2.116270	0.731055	4.107123
H	1.702296	2.443571	3.984829
H	1.151430	2.533074	0.289027
H	1.101069	3.464391	1.780073
H	-0.136183	2.254347	1.468739
H	3.590438	1.919163	0.861420
H	3.949260	1.115516	2.393067
H	3.465131	2.813014	2.374560
H	0.025661	-1.387697	2.281499
H	-0.537448	0.284175	2.120766
H	-5.181290	5.660039	-0.222006
H	-3.625514	5.161238	-0.926974
H	-5.140986	4.373035	-1.450436

ωB97X energy = -1507.40898799 a.u.

(5*S*,1’*S*)-**10a**, Conf. N

C	-2.668131	0.942310	-1.715328
C	-3.090057	2.256318	-1.775223
C	-3.079888	3.047233	-0.626582
C	-2.648204	2.502206	0.580877
C	-2.236889	1.177100	0.623488
C	-2.235377	0.381155	-0.515775
C	-1.777610	-1.058503	-0.472581
C	-2.522731	-1.898773	0.577218
C	-4.001488	-1.967622	0.285511
C	-4.908851	-1.164820	0.970719
C	-6.265981	-1.223580	0.682475
C	-6.733129	-2.086695	-0.299249
C	-5.836178	-2.892165	-0.988703
C	-4.480810	-2.832235	-0.696143
N	-0.320121	-1.136799	-0.236076
C	0.528184	-0.147015	-0.281484
N	1.784544	-0.494879	-0.112712
C	2.875727	0.441488	-0.233142
C	3.749937	0.315593	-1.321439
C	3.531678	-0.667692	-2.460571
C	3.422106	0.065062	-3.800763

C	4.631536	-1.730277	-2.514958
C	4.844336	1.173188	-1.376044
C	5.034923	2.153149	-0.418482
C	4.115625	2.306041	0.603933
C	3.018950	1.458321	0.723539
C	2.029218	1.710408	1.851331
C	2.703503	1.702699	3.224918
C	1.277847	3.026465	1.625366
C	1.882470	-1.970924	0.102942
C	2.444547	-2.433464	1.468888
C	1.574318	-1.988856	2.645233
C	3.876214	-1.924382	1.665004
C	2.499022	-3.967002	1.420441
C	0.419482	-2.405894	-0.163829
C	-3.505342	5.170397	0.362683
O	-3.502370	4.324381	-0.779316
H	-2.677544	0.340555	-2.618220
H	-3.430630	2.691345	-2.707181
H	-2.630471	3.090696	1.488338
H	-1.903404	0.772232	1.574435
H	-1.951968	-1.506643	-1.455932
H	-2.118062	-2.912866	0.578783
H	-2.347969	-1.477571	1.569944
H	-4.550495	-0.486886	1.738800
H	-6.959605	-0.594397	1.228775
H	-7.792699	-2.135491	-0.522888
H	-6.193129	-3.574239	-1.752074
H	-3.787350	-3.473584	-1.232226
H	0.229410	0.878313	-0.453675
H	2.575817	-1.170695	-2.309259
H	3.181562	-0.645836	-4.594111
H	2.638718	0.825032	-3.773112
H	4.361484	0.555233	-4.066137
H	4.429116	-2.444904	-3.315418
H	4.711023	-2.284993	-1.576726
H	5.604055	-1.272059	-2.710890
H	5.548520	1.086147	-2.195938
H	5.890797	2.815133	-0.481444
H	4.254121	3.100068	1.328974
H	1.285497	0.913053	1.856086
H	1.952134	1.818724	4.009004
H	3.239762	0.768404	3.405594
H	3.416321	2.524190	3.323665
H	0.530708	3.173610	2.408643
H	0.764084	3.041160	0.660408
H	1.964374	3.876185	1.648509
H	2.537885	-2.378800	-0.666387
H	1.518371	-0.902204	2.723262
H	0.555751	-2.379496	2.584086
H	2.006248	-2.360263	3.576892
H	4.514894	-2.196474	0.820159
H	4.301353	-2.379750	2.562027
H	3.923522	-0.842709	1.789320
H	3.128570	-4.311854	0.595646
H	1.512293	-4.421345	1.308423
H	2.928523	-4.344867	2.350120
H	0.306438	-2.937602	-1.110160
H	0.027138	-3.029685	0.634727
H	-2.497888	5.285910	0.771441
H	-3.869099	6.135437	0.019184
H	-4.173917	4.785072	1.136953

ωB97X energy = -1507.40889045 a.u.

(5*S*,1’*R*)-**10b**, Conf. A

C	-4.278331	1.233946	0.089034
C	-5.508795	0.589089	0.194640
C	-5.683060	-0.657085	-0.391778
C	-4.622159	-1.246441	-1.085938
C	-3.414545	-0.592247	-1.184030
C	-3.219492	0.661438	-0.594993
C	-1.862401	1.329061	-0.735519
C	-1.780747	2.758404	-0.186194
C	-0.407404	3.365565	-0.335691
C	0.140096	3.599618	-1.597394
C	1.410916	4.138629	-1.728910
C	2.157194	4.453534	-0.599085
C	1.621162	4.229821	0.659757
C	0.346517	3.690282	0.786864
N	-0.834644	0.477715	-0.120984
C	0.269297	0.090407	-0.694201
N	1.078862	-0.593992	0.085805
C	2.199216	-1.345100	-0.432685
C	3.416011	-0.689468	-0.662648
C	3.595888	0.815136	-0.545574
C	4.713183	1.193768	0.428482
C	3.858911	1.423578	-1.926503
C	4.498163	-1.465029	-1.068536
C	4.373948	-2.830888	-1.250799
C	3.143965	-3.441943	-1.081868
C	2.025350	-2.711230	-0.694094
C	0.676085	-3.411628	-0.634209
C	0.306485	-3.985421	-2.005323
C	0.641003	-4.502584	0.437640
C	0.429921	-0.808308	1.418257
C	1.276376	-0.528175	2.680043
C	2.491784	-1.460577	2.733477
C	0.375175	-0.857538	3.878609
C	1.741679	0.924956	2.779401
C	-0.838857	0.070293	1.287866
C	-7.944206	-0.809224	0.333606
O	-6.832670	-1.372922	-0.348492
H	-4.173518	2.204710	0.557975
H	-6.311285	1.072438	0.735281
H	-4.771148	-2.216997	-1.544025
H	-2.602238	-1.064205	-1.729148
H	-1.606569	1.357827	-1.797876
H	-2.518228	3.356076	-0.727324
H	-2.071585	2.773300	0.866716
H	-0.436333	3.369352	-2.488611
H	1.821036	4.315800	-2.716765

H	3.150194	4.876357	-0.702512
H	2.191938	4.476911	1.547962
H	-0.071953	3.531312	1.775883
H	0.494654	0.294937	-1.734275
H	2.668155	1.252649	-0.171017
H	4.744225	2.278960	0.553763
H	4.564941	0.743078	1.412270
H	5.689218	0.874432	0.055564
H	3.960197	2.507623	-1.845852
H	3.043174	1.209833	-2.621493
H	4.781417	1.027884	-2.358717
H	5.455829	-0.989119	-1.246787
H	5.233983	-3.417811	-1.552057
H	3.045369	-4.504613	-1.273334
H	-0.093745	-2.676650	-0.392929
H	-0.692171	-4.425907	-1.968403
H	0.310344	-3.208618	-2.772813
H	1.005716	-4.766982	-2.311049
H	-0.347203	-4.965683	0.473314
H	0.864500	-4.105272	1.430747
H	1.371057	-5.286141	0.221133
H	0.140927	-1.860814	1.463581
H	2.201260	-2.505722	2.596981
H	3.238192	-1.216448	1.977349
H	2.969053	-1.372417	3.711753
H	-0.513456	-0.222445	3.915466
H	0.929084	-0.700632	4.806144
H	0.049706	-1.901175	3.853461
H	0.913628	1.616796	2.944903
H	2.276505	1.248298	1.885205
H	2.420408	1.029287	3.628613
H	-1.754107	-0.474038	1.515998
H	-0.790696	0.959246	1.919047
H	-8.254150	0.131716	-0.128703
H	-8.748010	-1.536148	0.248896
H	-7.716455	-0.641487	1.389647

ωB97X energy = -1507.41295679 a.u.

(5*S*,1’*R*)-**10b**, Conf. B

C	-3.502300	-0.381860	-1.408858
C	-4.718030	-1.028514	-1.370657
C	-5.688328	-0.638384	-0.443391
C	-5.417209	0.401955	0.436350
C	-4.181856	1.042498	0.384229
C	-3.212861	0.666883	-0.531116
C	-1.850047	1.326946	-0.610913
C	-1.752342	2.707175	0.051820
C	-0.441493	3.399784	-0.231047
C	-0.143189	3.843903	-1.519496
C	1.058293	4.481396	-1.789762
C	1.984412	4.684045	-0.773519
C	1.700318	4.244963	0.510910
C	0.493216	3.609464	0.777925
N	-0.832719	0.414162	-0.065671
C	0.314261	0.141416	-0.619099
N	1.095464	-0.639275	0.098091
C	2.265438	-1.286603	-0.450887
C	3.466958	-0.572006	-0.551776
C	3.597918	0.911631	-0.246946
C	4.637514	1.189617	0.840913
C	3.939299	1.688272	-1.522449
C	4.590153	-1.261501	-0.999640
C	4.522890	-2.599707	-1.343813
C	3.310600	-3.264447	-1.295434
C	2.153006	-2.619206	-0.872558
C	0.831386	-3.369053	-0.944102
C	0.528104	-3.791696	-2.384477
C	0.809337	-4.578729	-0.007452
C	0.393346	-1.038724	1.358173
C	1.149810	-0.814157	2.686402
C	2.429381	-1.656493	2.724013
C	0.223010	-1.317087	3.802172
C	1.488949	0.655975	2.932056
C	-0.937479	-0.253235	1.236868
C	-7.874939	-0.962837	0.437729
O	-6.854525	-1.326910	-0.481518
H	-2.760087	-0.693737	-2.137935
H	-4.943786	-1.837182	-2.055603
H	-6.148757	0.727173	1.163543
H	-3.997600	1.851053	1.081861
H	-1.594884	1.439694	-1.667185
H	-2.579744	3.308669	-0.332074
H	-1.896927	2.619452	1.131146
H	-0.865821	3.703586	-2.318115
H	1.270028	4.827665	-2.795032
H	2.922366	5.185488	-0.983349
H	2.415781	4.400641	1.310858
H	0.272163	3.285804	1.789988
H	0.595351	0.509552	-1.598880
H	2.637320	1.287019	0.111332
H	4.653969	2.257219	1.073633
H	4.419149	0.646342	1.762760
H	5.640055	0.905509	0.512345
H	3.998829	2.756750	-1.306281
H	3.182798	1.542389	-2.297414
H	4.902548	1.370573	-1.928942
H	5.536473	-0.738742	-1.081282
H	5.414208	-3.119631	-1.675515
H	3.257505	-4.300487	-1.610767
H	0.025904	-2.695608	-0.647090
H	-0.456162	-4.262083	-2.436339
H	0.532739	-2.932122	-3.058031
H	1.262395	-4.513045	-2.750163
H	-0.164547	-5.070769	-0.048665
H	1.003530	-4.295193	1.030086
H	1.566891	-5.310350	-0.298303
H	0.192467	-2.109227	1.288263
H	2.219903	-2.708029	2.508584
H	3.179072	-1.310276	2.012739
H	2.868658	-1.602333	3.722443
H	-0.714756	-0.758472	3.848548

H	0.720136	-1.203256	4.767398
H	-0.015337	-2.375881	3.669716
H	0.594985	1.268065	3.070298
H	2.071482	1.085143	2.115087
H	2.081234	0.744918	3.845191
H	-1.810559	-0.904490	1.246020
H	-1.054000	0.492682	2.023343
H	-8.190065	0.072989	0.286688
H	-8.710795	-1.628222	0.237012
H	-7.542269	-1.098283	1.470282

ωB97X energy = -1507.41278551 a.u.

(5*S*,1'*R*)-**10b**, Conf. C

C	3.680490	-0.019068	1.275900
C	4.934376	-0.602838	1.151138
C	5.740647	-0.263194	0.068354
C	5.278037	0.657235	-0.871783
C	4.028051	1.227708	-0.731217
C	3.207018	0.898086	0.347329
C	1.817437	1.478292	0.524863
C	1.628974	2.883326	-0.063338
C	0.272753	3.466683	0.245849
C	-0.692822	3.592506	-0.747616
C	-1.949624	4.111492	-0.460032
C	-2.252649	4.517272	0.831148
C	-1.295559	4.398323	1.831978
C	-0.044135	3.875974	1.540975
N	0.814689	0.546287	-0.015639
C	-0.250429	0.133933	0.610499
N	-1.038375	-0.642728	-0.103401
C	-2.113812	-1.408270	0.484569
C	-3.349740	-0.788806	0.713733
C	-3.594445	0.698454	0.520560
C	-4.753867	0.977321	-0.437724
C	-3.841436	1.372594	1.873612
C	-4.388911	-1.582174	1.191047
C	-4.206350	-2.930666	1.440030
C	-2.958448	-3.502864	1.266457
C	-1.880124	-2.751603	0.809841
C	-0.507205	-3.403171	0.744866
C	-0.075082	-3.888086	2.131912
C	-0.461905	-4.549796	-0.267002
C	-0.414763	-0.900725	-1.439423
C	-1.304287	-0.724363	-2.690317
C	-2.477692	-1.710285	-2.664022
C	-0.420202	-1.074937	-3.895729
C	-1.836946	0.700033	-2.850504
C	0.816258	0.038490	-1.391798
C	7.496848	-1.700214	0.787217
O	6.978873	-0.767396	-0.151224
H	3.062648	-0.289752	2.126874
H	5.266809	-1.309127	1.899932
H	5.916113	0.915836	-1.708413
H	3.700700	1.940908	-1.479053
H	1.608265	1.528603	1.595829
H	2.416756	3.513120	0.356407
H	1.781071	2.867036	-1.144550
H	-0.456193	3.296379	-1.764824
H	-2.688426	4.203595	-1.248534
H	-3.228985	4.930586	1.057537
H	-1.523469	4.719074	2.842196
H	0.700856	3.797714	2.327339
H	-0.463128	0.389760	1.641832
H	-2.697471	1.151305	0.093948
H	-4.839716	2.052374	-0.613976
H	-4.610938	0.485992	-1.402708
H	-5.703425	0.632342	-0.021859
H	-3.978443	2.447231	1.737918
H	-3.001750	1.221792	2.556548
H	-4.739518	0.972906	2.351042
H	-5.359429	-1.133937	1.371387
H	-5.034220	-3.533192	1.795653
H	-2.813443	-4.549956	1.507651
H	0.225100	-2.653718	0.440570
H	0.939477	-4.289796	2.087252
H	-0.089389	-3.073091	2.858568
H	-0.732193	-4.680113	2.498198
H	0.542693	-4.975431	-0.311306
H	-0.733632	-4.218538	-1.272235
H	-1.151705	-5.348056	0.017279
H	-0.078836	-1.940216	-1.438271
H	-2.136743	-2.733513	-2.484814
H	-3.215235	-1.461386	-1.900867
H	-2.982612	-1.692846	-3.632129
H	0.434071	-0.400446	-3.993019
H	-1.005921	-0.995802	-4.813561
H	-0.042354	-2.098451	-3.824407
H	-1.045557	1.415844	-3.080974
H	-2.359175	1.047238	-1.957731
H	-2.544839	0.729609	-3.681454
H	1.750398	-0.482691	-1.595034
H	0.723075	0.874552	-2.086588
H	6.873327	-2.596757	0.838215
H	8.486803	-1.969104	0.427524
H	7.580660	-1.253586	1.781461

ωB97X energy = -1507.41271148 a.u.

(5*S*,1'*R*)-**10b**, Conf. D

C	4.085465	1.577886	1.030961
C	5.344035	1.025501	0.893843
C	5.567133	0.018057	-0.044494
C	4.515695	-0.424747	-0.842931
C	3.258209	0.146125	-0.694375
C	3.024673	1.147573	0.236993
C	1.656367	1.784323	0.381869
C	1.306018	2.683391	-0.807827
C	-0.021044	3.403504	-0.703462
C	-0.356822	4.136156	0.434008
C	-1.570871	4.802473	0.515406
C	-2.469951	4.749893	-0.542368

C	-2.144280	4.029194	-1.681752
C	-0.928381	3.361299	-1.758810
N	0.639156	0.755289	0.622105
C	-0.289094	0.324368	-0.181970
N	-0.999738	-0.676779	0.294082
C	-2.249068	-1.087579	-0.304367
C	-3.443516	-0.650027	0.283845
C	-3.495257	0.350114	1.428424
C	-4.241683	1.618230	1.004461
C	-4.116004	-0.256780	2.688114
C	-4.638450	-1.119968	-0.252689
C	-4.647998	-1.949780	-1.359739
C	-3.457004	-2.303176	-1.969303
C	-2.233112	-1.879636	-1.460887
C	-0.961337	-2.240632	-2.209613
C	-0.813231	-3.748083	-2.421555
C	-0.901105	-1.496422	-3.547373
C	-0.512378	-1.048525	1.658469
C	-0.045190	-2.509347	1.849873
C	0.397023	-2.635670	3.314642
C	1.118773	-2.884878	0.931953
C	-1.215168	-3.472669	1.627234
C	0.593039	0.012007	1.885951
C	7.105380	-1.501741	-1.041550
O	6.830174	-0.469955	-0.104446
H	3.924226	2.355972	1.770269
H	6.169444	1.357099	1.512493
H	4.658927	-1.205955	-1.577175
H	2.451459	-0.215505	-1.325689
H	1.662385	2.393246	1.289901
H	2.116732	3.412846	-0.875641
H	1.350034	2.106665	-1.736529
H	0.338347	4.199129	1.265114
H	-1.814213	5.368025	1.407680
H	-3.419202	5.268970	-0.477258
H	-2.836874	3.984098	-2.514461
H	-0.680653	2.805959	-2.659221
H	-0.487198	0.755439	-1.153915
H	-2.478289	0.663297	1.670039
H	-4.215230	2.354333	1.811274
H	-3.783459	2.069275	0.120560
H	-5.289381	1.408149	0.777239
H	-4.092546	0.465673	3.506501
H	-3.584141	-1.155617	3.010526
H	-5.158912	-0.532645	2.514738
H	-5.578674	-0.812095	0.191128
H	-5.589218	-2.303099	-1.764819
H	-3.477427	-2.919454	-2.861007
H	-0.107307	-1.909679	-1.618442
H	0.145483	-3.964128	-2.897921
H	-0.852300	-4.294518	-1.476618
H	-1.600542	-4.138726	-3.070077
H	0.042921	-1.706419	-4.054759
H	-0.981946	-0.414719	-3.411965
H	-1.715371	-1.810148	-4.205305
H	-1.333386	-0.885178	2.357814
H	1.266279	-2.015590	3.544610
H	-0.411018	-2.358063	3.996916
H	0.670211	-3.671694	3.523016
H	0.835504	-2.850009	-0.120893
H	1.437756	-3.906235	1.150082
H	1.989281	-2.238107	1.069955
H	-1.551663	-3.486480	0.590494
H	-2.070356	-3.209255	2.256419
H	-0.907560	-4.487208	1.889845
H	0.358441	0.700614	2.698293
H	1.569022	-0.433093	2.076894
H	6.919253	-1.165975	-2.065089
H	8.160064	-1.737895	-0.925166
H	6.508570	-2.393843	-0.833486

ωB97X energy = -1507.41077054 a.u.

(5*S*,1’*R*)-**10b**, Conf. E

C	3.174793	1.130147	1.234254
C	4.420027	0.550340	1.141889
C	5.083663	0.508661	-0.088428
C	4.480916	1.061449	-1.210308
C	3.219218	1.642560	-1.098489
C	2.548293	1.684532	0.112235
C	1.153074	2.260018	0.270539
C	0.685143	3.158622	-0.882617
C	-0.716811	3.674731	-0.676014
C	-0.970418	4.687609	0.246718
C	-2.268969	5.106304	0.499026
C	-3.334685	4.515869	-0.169245
C	-3.091839	3.516657	-1.101522
C	-1.789758	3.103403	-1.354794
N	0.185631	1.165989	0.499691
C	0.124887	0.055638	-0.176086
N	-0.812753	-0.776040	0.223524
C	-1.195701	-1.937045	-0.543945
C	-0.364553	-3.065836	-0.545607
C	0.992422	-3.108519	0.137816
C	1.117444	-4.281448	1.111518
C	2.116733	-3.145460	-0.902286
C	-0.796550	-4.182630	-1.254281
C	-2.001455	-4.179097	-1.934788
C	-2.777657	-3.033695	-1.960374
C	-2.380699	-1.880958	-1.290018
C	-3.209996	-0.615228	-1.445510
C	-3.306649	-0.205103	-2.917591
C	-4.602423	-0.761402	-0.828810
C	-1.557231	-0.189538	1.381333
C	-1.573992	-1.020410	2.685271
C	-0.175573	-1.254594	3.257712
C	-2.268973	-2.366371	2.455611
C	-2.413030	-0.222017	3.693128
C	-0.876170	1.197571	1.513889
C	7.010515	-0.157575	-1.313602
O	6.302254	-0.082200	-0.084044
H	2.674208	1.155607	2.197763

H	4.903824	0.123432	2.012359
H	4.971203	1.049975	-2.174333
H	2.776930	2.066499	-1.991940
H	1.126135	2.842184	1.194972
H	1.391922	3.987908	-0.961651
H	0.729577	2.602364	-1.821786
H	-0.142993	5.151698	0.775121
H	-2.450425	5.894566	1.220742
H	-4.349565	4.839574	0.030571
H	-3.917400	3.057544	-1.634930
H	-1.606450	2.326849	-2.092521
H	0.789014	-0.170329	-1.001782
H	1.120427	-2.192041	0.714692
H	2.078964	-4.235751	1.627360
H	0.327419	-4.266218	1.865613
H	1.066077	-5.239728	0.589953
H	3.089704	-3.112773	-0.406832
H	2.058290	-2.299660	-1.592453
H	2.070339	-4.061937	-1.495853
H	-0.176931	-5.072143	-1.273137
H	-2.325728	-5.065833	-2.467029
H	-3.700022	-3.026838	-2.530496
H	-2.700004	0.204117	-0.935795
H	-3.836618	0.746747	-3.005801
H	-2.315675	-0.087425	-3.361926
H	-3.855178	-0.944796	-3.504966
H	-5.152593	0.178321	-0.915445
H	-4.552931	-1.030034	0.229460
H	-5.177229	-1.536441	-1.341640
H	-2.596100	-0.061098	1.075009
H	0.446014	-1.847740	2.585824
H	0.350953	-0.322316	3.475204
H	-0.256557	-1.804557	4.197622
H	-3.257423	-2.233329	2.006789
H	-2.403723	-2.872986	3.413705
H	-1.691552	-3.027623	1.809333
H	-3.424632	-0.050723	3.315282
H	-1.968960	0.747136	3.931115
H	-2.493936	-0.782916	4.626094
H	-1.561214	2.019886	1.306644
H	-0.428453	1.354888	2.494438
H	7.218441	0.839343	-1.710978
H	7.947879	-0.660757	-1.090675
H	6.452814	-0.737785	-2.053585

ωB97X energy = -1507.41055980 a.u.

(5*S*,1'*R*)-**10b**, Conf. F

C	-2.397110	1.121940	-0.455915
C	-3.270892	2.195599	-0.570728
C	-4.306256	2.338032	0.349766
C	-4.450578	1.403553	1.374940
C	-3.568385	0.345301	1.475933
C	-2.530500	0.187657	0.560737
C	-1.593321	-0.998567	0.666982
C	-1.769681	-2.012020	-0.470804
C	-3.169320	-2.575617	-0.491559
C	-3.549576	-3.550340	0.427481
C	-4.843912	-4.051195	0.432626
C	-5.775446	-3.582799	-0.485036
C	-5.404220	-2.613957	-1.407066
C	-4.108844	-2.114115	-1.408368
N	-0.202897	-0.533707	0.754598
C	0.778749	-0.736202	-0.077306
N	1.894341	-0.110623	0.236809
C	3.151084	-0.367016	-0.427111
C	3.351194	0.104969	-1.732756
C	2.275943	0.792974	-2.558246
C	2.721304	2.168274	-3.059685
C	1.847576	-0.095088	-3.730647
C	4.597988	-0.108656	-2.312315
C	5.606612	-0.762531	-1.627686
C	5.367945	-1.261889	-0.359958
C	4.131198	-1.099762	0.256941
C	3.893545	-1.762539	1.604878
C	4.108640	-3.275890	1.513666
C	4.773352	-1.154960	2.699464
C	1.695974	0.700081	1.478869
C	1.914357	2.225805	1.347595
C	3.362697	2.527923	0.949359
C	1.679260	2.821799	2.742698
C	0.955273	2.872910	0.347809
C	0.256352	0.292888	1.877516
C	-5.099411	4.324630	-0.694459
O	-5.211510	3.346852	0.330279
H	-1.596590	1.028775	-1.184496
H	-3.133351	2.906176	-1.374500
H	-5.258020	1.526781	2.086871
H	-3.690567	-0.373501	2.279900
H	-1.793420	-1.510162	1.613155
H	-1.560720	-1.530226	-1.429948
H	-1.050791	-2.825275	-0.338592
H	-2.824574	-3.925782	1.143552
H	-5.124864	-4.811509	1.152495
H	-6.785860	-3.975392	-0.483456
H	-6.124307	-2.246284	-2.129281
H	-3.824169	-1.355795	-2.130871
H	0.702803	-1.372150	-0.949605
H	1.396548	0.945595	-1.931995
H	1.898018	2.656949	-3.585271
H	3.031844	2.818435	-2.238850
H	3.557334	2.085047	-3.757682
H	1.033588	0.378329	-4.283783
H	1.504342	-1.075357	-3.391084
H	2.677225	-0.255493	-4.423357
H	4.782347	0.246657	-3.319879
H	6.575368	-0.902867	-2.092952
H	6.151804	-1.807052	0.153872
H	2.849624	-1.618524	1.886291
H	3.857844	-3.745851	2.467025
H	3.480138	-3.718197	0.738079
H	5.149348	-3.517723	1.286926

H	4.553474	-1.618764	3.663296
H	4.617956	-0.077543	2.797266
H	5.831863	-1.318236	2.482934
H	2.403133	0.340587	2.226371
H	4.070622	2.042279	1.627038
H	3.592363	2.205251	-0.065943
H	3.536345	3.604839	1.002996
H	0.656286	2.674367	3.095413
H	1.862575	3.897493	2.712733
H	2.360773	2.385681	3.478176
H	-0.093811	2.732112	0.620411
H	1.096687	2.483901	-0.661525
H	1.138531	3.948819	0.311314
H	0.224551	-0.299116	2.793128
H	-0.408693	1.147381	1.992265
H	-5.213725	3.873975	-1.683906
H	-5.906673	5.032513	-0.524358
H	-4.140784	4.847193	-0.637933

ωB97X energy = -1507.41036358 a.u.

(5*S*,1'*R*)-**10b**, Conf. G

C	3.129758	1.201373	0.893440
C	4.406102	0.697285	0.695797
C	5.022774	0.865570	-0.542928
C	4.349671	1.541298	-1.557637
C	3.074462	2.033167	-1.343995
C	2.440799	1.868508	-0.114427
C	1.028300	2.355130	0.164084
C	0.421236	3.241017	-0.933190
C	-1.002207	3.637398	-0.632104
C	-2.064140	2.986989	-1.254931
C	-3.377871	3.288869	-0.918574
C	-3.644338	4.254858	0.041953
C	-2.591832	4.923285	0.655343
C	-1.280936	4.616070	0.319677
N	0.149346	1.201071	0.442605
C	0.114898	0.094120	-0.240892
N	-0.741635	-0.797714	0.207607
C	-1.089947	-1.981832	-0.540677
C	-0.189325	-3.054880	-0.588660
C	1.203477	-3.007563	0.018166
C	1.454101	-4.163012	0.988713
C	2.269416	-2.982475	-1.081840
C	-0.587799	-4.199261	-1.272499
C	-1.825962	-4.274414	-1.886007
C	-2.673246	-3.180284	-1.870082
C	-2.314349	-2.002560	-1.222106
C	-3.226850	-0.790721	-1.335078
C	-3.418064	-0.389930	-2.800443
C	-4.576818	-1.020528	-0.653158
C	-1.456761	-0.262248	1.407850
C	-1.374233	-1.105189	2.701191
C	0.058851	-1.282364	3.203526
C	-2.020781	-2.478169	2.489883
C	-2.196634	-0.353350	3.757303
C	-0.839663	1.155463	1.527088
C	6.982298	-0.292778	0.154751
O	6.263323	0.414072	-0.846515
H	2.662762	1.062486	1.864111
H	4.905168	0.185323	1.507620
H	4.840053	1.671231	-2.514973
H	2.582338	2.552515	-2.157064
H	1.035754	2.921059	1.099168
H	1.052452	4.127285	-1.030467
H	0.453499	2.710835	-1.888084
H	-1.862324	2.235455	-2.013542
H	-4.194652	2.770613	-1.409806
H	-4.668393	4.491850	0.306169
H	-2.793223	5.686364	1.398492
H	-0.463410	5.140039	0.805712
H	0.737669	-0.081864	-1.109993
H	1.305844	-2.080156	0.582630
H	2.433669	-4.049520	1.458185
H	0.701161	-4.196632	1.779370
H	1.443280	-5.125638	0.472670
H	3.263141	-2.875848	-0.640585
H	2.114899	-2.153209	-1.776984
H	2.254685	-3.908826	-1.661605
H	0.085572	-5.047324	-1.325408
H	-2.121721	-5.181665	-2.399963
H	-3.623050	-3.233642	-2.390391
H	-2.744542	0.059206	-0.848965
H	-4.007960	0.528117	-2.862810
H	-2.457736	-0.213266	-3.290159
H	-3.947434	-1.163124	-3.361782
H	-5.189541	-0.118452	-0.717055
H	-4.460941	-1.277550	0.402796
H	-5.124422	-1.834168	-1.134961
H	-2.513914	-0.180989	1.151584
H	0.678700	-1.820769	2.485708
H	0.545520	-0.330170	3.427196
H	0.049628	-1.863278	4.128221
H	-3.030917	-2.383937	2.081373
H	-2.096408	-2.994692	3.449098
H	-1.442787	-3.111673	1.816700
H	-3.231490	-0.219909	3.430470
H	-1.781231	0.630665	3.986139
H	-2.209248	-0.927086	4.685902
H	-1.579464	1.943872	1.388544
H	-0.334171	1.315648	2.478890
H	6.451048	-1.199801	0.455418
H	7.933852	-0.564092	-0.295255
H	7.161409	0.335592	1.031097

ωB97X energy = -1507.41026233 a.u.

(5*S*,1'*R*)-**10b**, Conf. H

C	-3.457997	-0.597857	-1.258243
C	-4.248156	-1.720626	-1.025876
C	-4.022248	-2.491874	0.107762
C	-3.007994	-2.130429	0.999402

C	-2.239061	-1.013687	0.753822
C	-2.453482	-0.228482	-0.380727
C	-1.611995	1.005969	-0.636637
C	-1.788985	2.093472	0.430087
C	-3.201776	2.623927	0.440780
C	-4.127254	2.168582	1.375060
C	-5.433335	2.639564	1.365073
C	-5.829364	3.573389	0.417490
C	-4.912382	4.034916	-0.518056
C	-3.607605	3.562110	-0.505006
N	-0.200208	0.623172	-0.778583
C	0.764162	0.747155	0.088337
N	1.878630	0.130278	-0.246040
C	3.122009	0.328611	0.461997
C	4.106494	1.134932	-0.125296
C	3.888472	1.923252	-1.407287
C	4.088514	3.422225	-1.166814
C	4.794912	1.431020	-2.537659
C	5.327292	1.248637	0.532501
C	5.544966	0.628652	1.749911
C	4.531360	-0.102185	2.343841
C	3.300193	-0.270195	1.717914
C	2.211005	-1.044458	2.441854
C	2.666214	-2.447465	2.847921
C	1.715776	-0.263124	3.662662
C	1.706972	-0.565478	-1.559645
C	1.961453	-2.091029	-1.583630
C	1.733070	-2.539654	-3.034613
C	1.024171	-2.867684	-0.658049
C	3.417271	-2.400778	-1.220796
C	0.261503	-0.154550	-1.933850
C	-5.763996	-4.015221	-0.446822
O	-4.725778	-3.603787	0.431566
H	-3.640963	-0.003535	-2.147923
H	-5.023986	-1.976888	-1.734632
H	-2.842663	-2.740757	1.879217
H	-1.459951	-0.751753	1.464406
H	-1.896620	1.427768	-1.604211
H	-1.550738	1.690538	1.417986
H	-1.090849	2.908485	0.221857
H	-3.822683	1.438470	2.118296
H	-6.142402	2.277465	2.100860
H	-6.847937	3.944264	0.409944
H	-5.212550	4.768693	-1.257444
H	-2.893371	3.932073	-1.234577
H	0.678186	1.318309	1.003824
H	2.851526	1.799123	-1.723056
H	3.852888	3.979278	-2.076130
H	3.440814	3.784322	-0.365820
H	5.122398	3.648075	-0.896532
H	4.584876	1.979764	-3.458098
H	4.655063	0.365877	-2.737802
H	5.847451	1.585558	-2.288050
H	6.114268	1.851879	0.094032
H	6.501590	0.733745	2.248437
H	4.699154	-0.554329	3.314854
H	1.362662	-1.162463	1.767183
H	1.830182	-2.997165	3.285770
H	3.035226	-3.016539	1.991713
H	3.462882	-2.407464	3.594052
H	0.895757	-0.798419	4.146342
H	1.357369	0.732621	3.389549
H	2.515904	-0.137645	4.396301
H	2.410276	-0.117594	-2.262461
H	0.701466	-2.390363	-3.360761
H	2.390297	-2.001008	-3.722719
H	1.953355	-3.604883	-3.125797
H	1.194096	-2.624963	0.391524
H	1.204336	-3.938119	-0.777493
H	-0.031614	-2.691356	-0.879839
H	3.645083	-2.171730	-0.179555
H	4.112251	-1.839610	-1.851556
H	3.610491	-3.464189	-1.377193
H	0.219726	0.469806	-2.826546
H	-0.397595	-1.010508	-2.075565
H	-6.191346	-4.912924	-0.007404
H	-6.537800	-3.247466	-0.529821
H	-5.370324	-4.248105	-1.439781

ωB97X energy = -1507.41020266 a.u.

(*R*)-**17a**, Conf. A

C	1.395502	-0.269715	1.662579
C	0.458158	0.938375	1.822045
N	0.164585	1.297875	0.422355
C	0.582885	0.344773	-0.375473
N	1.230887	-0.611238	0.238141
C	-0.641909	2.427111	0.050867
C	1.789850	-1.807761	-0.404162
C	-2.014078	2.246472	-0.158562
C	-2.763416	3.373006	-0.487170
C	-2.171368	4.619045	-0.597900
C	-0.811255	4.767673	-0.383686
C	-0.017337	3.675753	-0.050716
C	-2.705044	0.896801	-0.055529
C	1.475095	3.861496	0.157698
C	-3.754400	0.889651	1.058141
C	-3.327822	0.500588	-1.396847
C	2.165346	4.206320	-1.163981
C	1.770079	4.914063	1.227321
C	1.157898	-3.078748	0.182488
C	3.306799	-1.789914	-0.353311
C	-0.348249	-3.068213	0.103034
C	4.012761	-2.187339	0.779248
C	5.399599	-2.130074	0.799031
C	6.095944	-1.681240	-0.314526
C	5.399893	-1.288525	-1.449297
C	4.013570	-1.341688	-1.465713
C	-0.994910	-3.205274	-1.123719
C	-2.377588	-3.168598	-1.212587
C	-3.120387	-2.993825	-0.055954

C	-2.508648	-2.856184	1.176564
C	-1.122612	-2.896276	1.245880
Cl	-4.867620	-2.944988	-0.156313
H	2.439874	-0.018080	1.852535
H	1.107530	-1.104550	2.298711
H	-0.475499	0.680583	2.326719
H	0.926630	1.773133	2.340249
H	0.405613	0.352075	-1.444071
H	1.488225	-1.734922	-1.451075
H	-3.829132	3.272229	-0.659404
H	-2.774737	5.481897	-0.855834
H	-0.358800	5.748231	-0.480493
H	-1.963105	0.135079	0.198831
H	1.895888	2.915604	0.504263
H	-4.201627	-0.104177	1.144406
H	-3.313847	1.151754	2.022619
H	-4.557696	1.600334	0.848797
H	-3.805438	-0.478053	-1.316960
H	-2.575851	0.451637	-2.187606
H	-4.091383	1.220018	-1.702388
H	3.242635	4.305088	-1.013997
H	1.993448	3.431137	-1.914322
H	1.792381	5.152631	-1.564257
H	2.845838	4.977252	1.404035
H	1.282457	4.667660	2.173220
H	1.426412	5.904169	0.918476
H	1.564298	-3.922139	-0.379896
H	1.469424	-3.217531	1.219258
H	3.490954	-2.548172	1.658795
H	5.936358	-2.440462	1.687904
H	7.178907	-1.640169	-0.298554
H	5.935866	-0.941497	-2.325032
H	3.474752	-1.034171	-2.356401
H	-0.414223	-3.353019	-2.028691
H	-2.873168	-3.281496	-2.168826
H	-3.102947	-2.726694	2.072590
H	-0.644944	-2.803550	2.215669

ωB97X energy = -1695.21419165 a.u.

(*R*)-**17a**, Conf. B

C	1.501517	-0.090162	1.732714
C	0.601606	1.153322	1.875336
N	-0.003289	1.274727	0.536457
C	0.433191	0.317742	-0.245848
N	1.278591	-0.494447	0.332915
C	-0.862839	2.359560	0.150469
C	1.926529	-1.653232	-0.291929
C	-0.276868	3.519936	-0.369490
C	-1.125981	4.560555	-0.731245
C	-2.496864	4.450176	-0.568317
C	-3.047336	3.295385	-0.039379
C	-2.242439	2.223608	0.335400
C	1.222277	3.664297	-0.566370
C	-2.876580	0.968167	0.906730
C	1.574884	3.694219	-2.055580
C	1.770369	4.898039	0.152815
C	-3.662036	1.271868	2.184139
C	-3.764255	0.282769	-0.133961
C	1.343623	-2.959692	0.260444
C	3.436155	-1.512777	-0.163524
C	-0.133768	-3.085586	-0.011288
C	4.069483	-0.500091	-0.883068
C	5.438058	-0.312127	-0.784658
C	6.195556	-1.139357	0.036715
C	5.574533	-2.150478	0.752169
C	4.199889	-2.336495	0.653958
C	-1.070234	-2.863071	0.993189
C	-2.431667	-2.960437	0.740119
C	-2.854038	-3.282810	-0.537199
C	-1.946222	-3.507207	-1.559063
C	-0.590943	-3.407138	-1.287513
Cl	-4.567449	-3.413454	-0.868721
H	2.557489	0.129828	1.885782
H	1.205860	-0.893657	2.406750
H	-0.180728	1.022785	2.622877
H	1.164331	2.058053	2.106128
H	0.124030	0.216758	-1.279322
H	1.673313	-1.590835	-1.353056
H	-0.709038	5.470668	-1.147720
H	-3.142297	5.272075	-0.856420
H	-4.122291	3.224076	0.083321
H	1.715568	2.791548	-0.132436
H	-2.081686	0.265948	1.170185
H	2.657054	3.760627	-2.187057
H	1.222402	2.796008	-2.567611
H	1.122342	4.560098	-2.545583
H	2.856301	4.941026	0.046065
H	1.532346	4.876327	1.218505
H	1.359166	5.818899	-0.267203
H	-4.053283	0.346076	2.611818
H	-3.032124	1.755548	2.933978
H	-4.510851	1.929526	1.981220
H	-4.213461	-0.619786	0.286192
H	-3.190347	-0.005619	-1.018462
H	-4.574792	0.941982	-0.454834
H	1.881560	-3.784140	-0.212426
H	1.528478	-3.033939	1.334228
H	3.484584	0.148436	-1.528892
H	5.916192	0.478338	-1.351425
H	7.266749	-0.994750	0.115702
H	6.158277	-2.800884	1.393103
H	3.737051	-3.133954	1.222753
H	-0.739768	-2.623056	1.998836
H	-3.153644	-2.792694	1.530000
H	-2.293881	-3.763704	-2.552017
H	0.121018	-3.594749	-2.084937

ωB97X energy = -1695.21379848 a.u.

(*R*)-**17a**, Conf. C

C	1.516952	0.026798	2.140956

C	0.781771	-1.325853	2.082461
N	0.183969	-1.308797	0.735072
C	0.475465	-0.188048	0.120162
N	1.210973	0.626374	0.830311
C	-0.604597	-2.384849	0.202299
C	1.767199	1.924299	0.444797
C	0.050673	-3.415398	-0.481296
C	-0.731867	-4.450552	-0.982455
C	-2.104662	-4.458291	-0.799404
C	-2.724644	-3.430186	-0.109565
C	-1.987841	-2.371030	0.411514
C	1.552540	-3.421942	-0.706820
C	-2.695189	-1.251778	1.154900
C	2.197100	-4.706984	-0.184935
C	1.881118	-3.203328	-2.185730
C	-3.591428	-0.450779	0.207574
C	-3.492018	-1.784856	2.346886
C	1.073828	2.520141	-0.781470
C	3.267732	1.820317	0.231143
C	-0.427028	2.691544	-0.686978
C	4.117933	2.727589	0.850982
C	5.488430	2.669582	0.631774
C	6.017381	1.698591	-0.205697
C	5.172044	0.786075	-0.824828
C	3.803902	0.848198	-0.608688
C	-1.064374	3.041866	0.500532
C	-2.439087	3.219673	0.549958
C	-3.182725	3.050637	-0.604982
C	-2.578917	2.706330	-1.800876
C	-1.204095	2.525977	-1.830653
Cl	-4.919800	3.251593	-0.545004
H	1.144246	0.677252	2.931955
H	2.595962	-0.085280	2.247326
H	1.455129	-2.177801	2.176314
H	-0.005378	-1.411320	2.831642
H	0.126062	0.028563	-0.881420
H	1.597533	2.579094	1.303353
H	-0.260750	-5.262924	-1.524458
H	-2.696967	-5.273934	-1.198136
H	-3.800234	-3.451017	0.026608
H	1.990268	-2.590324	-0.150111
H	-1.942010	-0.564776	1.547602
H	3.281647	-4.654110	-0.300705
H	1.973740	-4.863425	0.872573
H	1.846462	-5.581417	-0.738133
H	2.962925	-3.163551	-2.331120
H	1.452275	-2.270013	-2.558065
H	1.488533	-4.020460	-2.796453
H	-4.067267	0.375035	0.742238
H	-3.018963	-0.029963	-0.623150
H	-4.380762	-1.081525	-0.209576
H	-3.936953	-0.955347	2.900444
H	-2.855100	-2.350433	3.030464
H	-4.302977	-2.440182	2.020559
H	1.541364	3.494894	-0.943374
H	1.320582	1.928183	-1.667601
H	3.708249	3.484732	1.511973
H	6.142456	3.381617	1.121558
H	7.086855	1.648592	-0.374086
H	5.579323	0.024051	-1.479124
H	3.156252	0.127160	-1.099399
H	-0.496010	3.184428	1.412939
H	-2.926112	3.487057	1.479444
H	-3.172176	2.573307	-2.696925
H	-0.729776	2.251267	-2.767161

ωB97X energy = -1695.21225979 a.u.

(*R*)-**17a**, Conf. D

C	0.792925	0.429483	2.296889
C	2.307800	0.300033	2.051344
N	2.368750	-0.112683	0.637508
C	1.159272	-0.291885	0.162573
N	0.211169	-0.043239	1.027407
C	3.596064	-0.290372	-0.088102
C	-1.237998	-0.039729	0.813631
C	4.145593	0.820313	-0.739721
C	5.334749	0.631564	-1.435658
C	5.950526	-0.608828	-1.469848
C	5.390560	-1.686475	-0.805414
C	4.201823	-1.550718	-0.094513
C	3.472148	2.181532	-0.734272
C	3.608026	-2.750514	0.621617
C	4.433497	3.296975	-0.323192
C	2.840127	2.473870	-2.097573
C	3.158393	-3.817819	-0.379154
C	4.587044	-3.333404	1.642535
C	-1.617938	-0.831134	-0.445502
C	-1.766117	1.383340	0.792221
C	-3.111656	-1.012968	-0.548275
C	-1.235287	2.324846	-0.085204
C	-1.753396	3.609878	-0.132233
C	-2.809658	3.965661	0.698027
C	-3.339168	3.033170	1.577624
C	-2.815900	1.747018	1.625676
C	-3.882528	-0.174631	-1.346862
C	-5.259688	-0.324352	-1.420059
C	-5.865315	-1.326402	-0.682056
C	-5.124005	-2.176589	0.121006
C	-3.748665	-2.012537	0.181555
Cl	-7.602597	-1.526737	-0.769299
H	0.441808	-0.197109	3.115546
H	0.482081	1.459650	2.473610
H	2.841169	1.240346	2.187398
H	2.772140	-0.464958	2.674510
H	0.982714	-0.620602	-0.853372
H	-1.663668	-0.553044	1.680091
H	5.785638	1.465952	-1.960827
H	6.877089	-0.735793	-2.017965
H	5.884828	-2.650847	-0.839931
H	2.666035	2.164054	0.002607
H	2.722400	-2.422151	1.169914

H	3.894447	4.243475	-0.246344
H	4.894721	3.087482	0.644212
H	5.230697	3.430503	-1.058079
H	2.325661	3.437276	-2.078745
H	2.114946	1.705296	-2.375814
H	3.603894	2.512246	-2.878614
H	2.695764	-4.656754	0.145167
H	2.433276	-3.418689	-1.091964
H	4.009342	-4.204304	-0.945808
H	4.114524	-4.152547	2.188732
H	4.904658	-2.578725	2.365238
H	5.479750	-3.731216	1.153901
H	-1.255312	-0.310044	-1.335216
H	-1.133344	-1.810158	-0.406357
H	-0.410026	2.061644	-0.741551
H	-1.333880	4.335258	-0.819668
H	-3.216405	4.969526	0.659496
H	-4.159580	3.305460	2.231422
H	-3.231280	1.019122	2.315349
H	-3.404553	0.610974	-1.922696
H	-5.854511	0.329674	-2.045250
H	-5.613164	-2.959341	0.687158
H	-3.164488	-2.681636	0.805154

ωB97X energy = -1695.21147599 a.u.

(*R*)-**17a**, Conf. E

C	-0.584721	0.541585	1.829462
C	0.097360	-0.840210	1.836861
N	0.780924	-0.868412	0.529891
C	0.666141	0.293547	-0.065064
N	-0.055837	1.157779	0.598056
C	1.584464	-1.969009	0.077444
C	-0.396243	2.517470	0.146915
C	2.957908	-1.951542	0.344068
C	3.710292	-3.029916	-0.111455
C	3.112188	-4.080557	-0.786966
C	1.747125	-4.076930	-1.023090
C	0.951120	-3.020250	-0.593904
C	3.635150	-0.806185	1.077364
C	-0.538172	-3.013731	-0.890891
C	4.458514	-1.301842	2.267190
C	4.496581	0.022123	0.120070
C	-0.788931	-2.771934	-2.381504
C	-1.224519	-4.294971	-0.417252
C	-1.477611	2.477860	-0.942354
C	0.872976	3.268532	-0.235030
C	-2.674054	1.654373	-0.538840
C	1.904574	3.352778	0.701920
C	3.079486	4.020963	0.403872
C	3.241476	4.622167	-0.839830
C	2.221282	4.548678	-1.773031
C	1.041357	3.874543	-1.473345
C	-3.612205	2.152659	0.361953
C	-4.681850	1.377330	0.782188
C	-4.811487	0.087514	0.292956
C	-3.900895	-0.432324	-0.610080
C	-2.838221	0.359844	-1.021833
Cl	-6.157959	-0.898285	0.819471
H	-1.670867	0.469524	1.764110
H	-0.315670	1.149179	2.692356
H	0.837835	-0.942607	2.631459
H	-0.613854	-1.662251	1.905993
H	1.136730	0.511294	-1.016608
H	-0.820343	2.997945	1.032397
H	4.779832	-3.049561	0.065378
H	3.716331	-4.911533	-1.132741
H	1.294820	-4.907350	-1.553495
H	2.863365	-0.144070	1.477368
H	-0.993908	-2.183001	-0.346988
H	4.866463	-0.451806	2.817905
H	3.847408	-1.892901	2.952568
H	5.298473	-1.919794	1.941986
H	4.948873	0.863879	0.649494
H	3.908542	0.421326	-0.710499
H	5.301846	-0.585195	-0.301210
H	-1.861997	-2.717482	-2.582046
H	-0.329281	-1.839104	-2.717954
H	-0.375057	-3.585373	-2.983013
H	-2.302122	-4.220746	-0.578793
H	-1.050078	-4.468756	0.646643
H	-0.866534	-5.168440	-0.967111
H	-1.781257	3.506058	-1.149929
H	-1.052156	2.072978	-1.863853
H	1.787740	2.886791	1.675683
H	3.869789	4.076276	1.143556
H	4.160829	5.145254	-1.076007
H	2.337468	5.014969	-2.744437
H	0.260279	3.833461	-2.222232
H	-3.510084	3.162606	0.745764
H	-5.408496	1.770826	1.481916
H	-4.020184	-1.439345	-0.991677
H	-2.129548	-0.043166	-1.739380

ωB97X energy = -1695.21094645 a.u.

Table S11. Cartesian coordinates and energies of the low-energy conformers of (5*S*,1'*S*)-**8a** and (5*S*,1'*R*)-**8b** calculated at the ωB97X/TZVP PCM (solvent: methanol) level. Charge: +1.

(5*S*,1'*S*)-**8a**, Conf. A

C	-3.564603	-0.788032	0.661442
C	-4.665271	-1.581604	0.362761
C	-5.636010	-1.109599	-0.513437
C	-5.517054	0.141965	-1.089371
C	-4.411512	0.929170	-0.787108
C	-3.427416	0.485392	0.082299
C	-2.194394	1.301066	0.418039
C	-2.253613	2.775943	0.007043
C	-1.013980	3.529866	0.421468
C	-0.088444	3.954801	-0.527156
C	1.065796	4.626053	-0.143594
C	1.310889	4.877012	1.198607
C	0.392193	4.460004	2.153809
C	-0.761194	3.792998	1.766665
N	-1.015010	0.656568	-0.173453
C	0.009978	0.198531	0.484502
N	0.890401	-0.437300	-0.259454
C	2.199552	-0.823269	0.209764
C	3.312044	-0.117036	-0.269272
C	3.208896	1.135603	-1.126364
C	3.887391	2.321518	-0.435047
C	3.794418	0.918719	-2.523814
C	4.574890	-0.565937	0.104829
C	4.728433	-1.637643	0.965395
C	3.612763	-2.265114	1.489690
C	2.326625	-1.875868	1.128525
C	1.147370	-2.579676	1.782222
C	1.174117	-4.092877	1.556695
C	1.102495	-2.257812	3.279520
C	0.404105	-0.505124	-1.673416
C	0.131816	-1.923361	-2.227812
C	1.422508	-2.748317	-2.245852
C	-0.330257	-1.746816	-3.680966
C	-0.946646	-2.663576	-1.434440
C	-0.846045	0.405718	-1.607905
C	-2.672142	-2.434561	2.140188
O	-2.567037	-1.166886	1.504485
H	-4.777620	-2.562733	0.803768
H	-6.490847	-1.736147	-0.740435
H	-6.273899	0.509110	-1.771430
H	-4.325552	1.907797	-1.243394
H	-2.036301	1.249496	1.497280
H	-2.389958	2.865665	-1.072950
H	-3.137905	3.212380	0.477105
H	-0.276771	3.772404	-1.581023
H	1.773406	4.954489	-0.896484
H	2.211630	5.399410	1.499942
H	0.571948	4.659357	3.204158
H	-1.478445	3.480722	2.519895
H	0.134669	0.337860	1.551366
H	2.156184	1.405629	-1.232016
H	3.756466	3.225920	-1.033342
H	3.454667	2.503802	0.551144
H	4.959699	2.153465	-0.313235
H	4.865222	0.708215	-2.466870
H	3.660960	1.815045	-3.133034
H	3.320482	0.080951	-3.041649
H	5.453721	-0.052387	-0.268550
H	5.720837	-1.970252	1.246975
H	3.742467	-3.077942	2.195264
H	0.224103	-2.201861	1.339921
H	0.275436	-4.549095	1.978580
H	1.212839	-4.343468	0.494451
H	2.038036	-4.552146	2.042319
H	0.250423	-2.751521	3.752841
H	1.017175	-1.183653	3.460004
H	2.007061	-2.609327	3.781025
H	1.160215	-0.049571	-2.312125
H	2.220760	-2.226288	-2.781238
H	1.241114	-3.695250	-2.758683
H	1.784887	-2.978282	-1.244349
H	-1.274570	-1.203965	-3.759303
H	0.420320	-1.213825	-4.271115
H	-0.479551	-2.727645	-4.136235
H	-1.903929	-2.134858	-1.434181
H	-1.119265	-3.644276	-1.882563
H	-0.648939	-2.826075	-0.396920
H	-0.696620	1.351615	-2.130515
H	-1.739963	-0.072099	-2.005027
H	-3.580927	-2.499002	2.742855
H	-1.803773	-2.516309	2.789882
H	-2.657523	-3.243968	1.404900

ωB97X energy = -1507.41544365 a.u.

(5*S*,1'*S*)-**8a**, Conf. B

C	-3.471250	-1.060957	-0.822743
C	-4.500065	-1.989542	-0.924587
C	-5.580047	-1.924583	-0.052085
C	-5.641852	-0.943252	0.920783
C	-4.607750	-0.020027	1.016212
C	-3.519078	-0.056455	0.156832
C	-2.437687	0.998458	0.286387
C	-2.366482	1.964935	-0.903619
C	-1.336404	3.056367	-0.722275
C	-1.393948	3.922207	0.370014
C	-0.433994	4.907117	0.546450
C	0.600703	5.047331	-0.370979
C	0.660591	4.201139	-1.467551
C	-0.302181	3.213047	-1.639338
N	-1.130177	0.410602	0.607652

C	-0.196585	0.017302	-0.211866
N	0.868765	-0.477701	0.383741
C	1.905567	-1.203143	-0.312055
C	2.826316	-0.514433	-1.115087
C	2.729527	0.968444	-1.435234
C	3.987416	1.740520	-1.032231
C	2.432821	1.170152	-2.924831
C	3.858809	-1.250290	-1.688950
C	3.971399	-2.613701	-1.484239
C	3.014145	-3.279635	-0.739932
C	1.949463	-2.596743	-0.160538
C	0.864816	-3.395421	0.545956
C	0.213869	-4.389304	-0.420366
C	1.398075	-4.115706	1.786109
C	0.708389	-0.394856	1.869203
C	1.724030	0.510494	2.604762
C	1.367637	0.458114	4.096947
C	1.671238	1.958960	2.116408
C	3.142235	-0.046471	2.446534
C	-0.761670	0.070894	1.985296
C	-2.287105	-2.058044	-2.647156
O	-2.379912	-1.061797	-1.635405
H	-4.472290	-2.764159	-1.678781
H	-6.375258	-2.655452	-0.142715
H	-6.481220	-0.893543	1.603327
H	-4.647214	0.752782	1.777306
H	-2.682518	1.591243	1.170099
H	-2.179968	1.415452	-1.827620
H	-3.363805	2.401477	-0.998186
H	-2.200976	3.833422	1.091055
H	-0.495153	5.571551	1.400927
H	1.350875	5.817544	-0.234075
H	1.456795	4.311482	-2.195958
H	-0.252572	2.563137	-2.508365
H	-0.289722	0.070785	-1.286823
H	1.894354	1.396587	-0.879582
H	3.843832	2.807024	-1.222206
H	4.219162	1.614986	0.027385
H	4.855972	1.413593	-1.608498
H	1.516609	0.657176	-3.228525
H	3.248436	0.786790	-3.542419
H	2.318215	2.233523	-3.147297
H	4.589435	-0.742914	-2.308859
H	4.793943	-3.162664	-1.927791
H	3.083668	-4.355273	-0.622208
H	0.074071	-2.712617	0.860712
H	-0.622745	-4.893400	0.068424
H	-0.164640	-3.882051	-1.310775
H	0.923181	-5.154299	-0.743871
H	0.587615	-4.640156	2.296635
H	1.855653	-3.422561	2.496508
H	2.154558	-4.854577	1.511038
H	0.817657	-1.399610	2.276921
H	0.387014	0.887816	4.311035
H	2.103765	1.029231	4.665861
H	1.379559	-0.569655	4.470074
H	1.945855	2.047343	1.063723
H	0.682622	2.408015	2.243094
H	2.377028	2.563642	2.690000
H	3.495049	-0.007026	1.416442
H	3.833110	0.539079	3.057080
H	3.198372	-1.085724	2.782799
H	-1.425181	-0.712799	2.355960
H	-0.875232	0.950718	2.616916
H	-2.294469	-3.060523	-2.212153
H	-1.337368	-1.886865	-3.148908
H	-3.102582	-1.961969	-3.367599

ωB97X energy = -1507.41328580 a.u.

(5*S*,1’*S*)-**8a**, Conf. C

C	-1.788499	-3.112891	0.208204
C	-1.577446	-4.436105	-0.154637
C	-1.840134	-4.839319	-1.459667
C	-2.310565	-3.936801	-2.395366
C	-2.520889	-2.612645	-2.023478
C	-2.265945	-2.181942	-0.731451
C	-2.479515	-0.752009	-0.272955
C	-3.187544	0.159329	-1.280790
C	-3.396342	1.551249	-0.737278
C	-4.373787	1.790890	0.227388
C	-4.540724	3.057536	0.767255
C	-3.732093	4.106764	0.346467
C	-2.762001	3.880629	-0.619147
C	-2.596074	2.609741	-1.156324
N	-1.193428	-0.166402	0.148670
C	-0.090814	-0.177950	-0.537984
N	0.943793	0.360737	0.078774
C	2.292828	0.129639	-0.386268
C	2.864612	1.023765	-1.300316
C	2.095749	2.168884	-1.936119
C	2.789131	3.517209	-1.732347
C	1.868568	1.899203	-3.426767
C	4.187622	0.806749	-1.673092
C	4.907871	-0.261608	-1.168474
C	4.299749	-1.165766	-0.315296
C	2.974040	-1.005068	0.075811
C	2.320404	-2.090980	0.918502
C	2.297483	-3.417738	0.153013
C	3.004180	-2.252914	2.277212
C	0.558810	0.692231	1.488977
C	1.000567	2.055239	2.062941
C	0.467046	3.248072	1.270520
C	2.528975	2.127524	2.144279
C	0.446814	2.112765	3.494071
C	-0.973131	0.479761	1.445415
C	-1.124702	-3.519162	2.468731
O	-1.554929	-2.621557	1.453408
H	-1.212711	-5.157537	0.564316
H	-1.672299	-5.874071	-1.735107
H	-2.515787	-4.252670	-3.410725
H	-2.893709	-1.913001	-2.761714

H	-3.070658	-0.779086	0.645243
H	-2.608239	0.209359	-2.206206
H	-4.149629	-0.297386	-1.523675
H	-5.014127	0.977871	0.556748
H	-5.306391	3.228069	1.515523
H	-3.862387	5.097010	0.767495
H	-2.129061	4.694020	-0.955901
H	-1.835760	2.441990	-1.913927
H	-0.010725	-0.619110	-1.524878
H	1.113019	2.221864	-1.465536
H	2.166685	4.321596	-2.130601
H	2.973121	3.722879	-0.675494
H	3.748789	3.552253	-2.253080
H	1.340887	0.956592	-3.588694
H	2.818962	1.848140	-3.964051
H	1.274976	2.702737	-3.868486
H	4.660261	1.484370	-2.375287
H	5.941532	-0.405303	-1.461325
H	4.859262	-2.024950	0.037518
H	1.277182	-1.824381	1.101005
H	1.778407	-4.182072	0.736944
H	1.780167	-3.313353	-0.803720
H	3.308612	-3.780791	-0.044544
H	2.482959	-3.003254	2.876064
H	3.013422	-1.315755	2.839398
H	4.039604	-2.580916	2.158675
H	1.014760	-0.072781	2.123820
H	0.820252	3.245226	0.239240
H	0.815712	4.172955	1.735423
H	-0.624170	3.285790	1.251972
H	2.937232	1.271244	2.688687
H	2.998319	2.156146	1.160475
H	2.822150	3.033508	2.679092
H	0.803473	1.269499	4.091992
H	0.781897	3.032271	3.977670
H	-0.645691	2.110245	3.513743
H	-1.328019	-0.176005	2.238723
H	-1.527202	1.419542	1.475266
H	-1.862364	-4.308092	2.633302
H	-1.022505	-2.922289	3.371846
H	-0.158802	-3.965946	2.217206

ωB97X energy = -1507.41264740 a.u.

(5*S*,1'*S*)-**8a**, Conf. D

C	-2.162971	2.011700	-0.359159
C	-2.604863	3.267715	0.037304
C	-3.420162	3.385997	1.156718
C	-3.794579	2.266621	1.878337
C	-3.349341	1.013781	1.472219
C	-2.540745	0.863249	0.356523
C	-1.980545	-0.472068	-0.094721
C	-2.732414	-1.721582	0.372954
C	-4.107471	-1.842429	-0.242058
C	-5.252869	-1.836161	0.547729
C	-6.512626	-1.960584	-0.024394
C	-6.643244	-2.093212	-1.398968
C	-5.506110	-2.106819	-2.197095
C	-4.250216	-1.984358	-1.621569
N	-0.572416	-0.549783	0.322389
C	0.461760	-0.465292	-0.464950
N	1.612978	-0.357045	0.166125
C	2.896344	-0.535748	-0.471774
C	3.401265	0.459237	-1.321888
C	2.621305	1.694497	-1.747414
C	3.340567	2.994468	-1.380192
C	2.340732	1.648846	-3.253285
C	4.679829	0.275564	-1.840476
C	5.425478	-0.849399	-1.537837
C	4.880961	-1.843855	-0.745681
C	3.599612	-1.722344	-0.217567
C	3.014138	-2.896763	0.551580
C	2.977610	-4.152824	-0.323913
C	3.777871	-3.166027	1.850090
C	1.375811	-0.272763	1.642257
C	1.794601	1.055922	2.313597
C	3.308095	1.256098	2.190808
C	1.462866	0.925059	3.806725
C	1.053025	2.259371	1.728871
C	-0.137957	-0.579381	1.723630
C	-1.007301	2.912960	-2.244805
O	-1.346794	1.800488	-1.426028
H	-2.317118	4.155513	-0.509374
H	-3.759484	4.369520	1.460766
H	-4.427764	2.360266	2.751901
H	-3.644871	0.142704	2.044101
H	-1.954746	-0.475315	-1.185174
H	-2.128201	-2.584937	0.081413
H	-2.807858	-1.748447	1.462020
H	-5.162462	-1.739322	1.624919
H	-7.393156	-1.954896	0.608070
H	-7.625087	-2.191082	-1.847588
H	-5.597779	-2.220152	-3.271341
H	-3.369423	-2.012493	-2.256243
H	0.382047	-0.493362	-1.544456
H	1.657098	1.698583	-1.235885
H	2.723707	3.851106	-1.661783
H	3.541047	3.061958	-0.309150
H	4.293161	3.083141	-1.907325
H	3.272105	1.624891	-3.823425
H	1.788597	2.539010	-3.562108
H	1.757066	0.767310	-3.530490
H	5.099672	1.030017	-2.496140
H	6.424477	-0.963006	-1.942567
H	5.453888	-2.743440	-0.550114
H	1.977071	-2.670450	0.804280
H	2.479824	-4.965281	0.209787
H	2.435265	-3.970942	-1.253962
H	3.984758	-4.489923	-0.578994
H	3.313529	-3.988186	2.398563
H	3.798511	-2.289734	2.503022
H	4.813356	-3.444904	1.640558

H	1.942359	-1.070140	2.121788
H	3.852250	0.400662	2.601068
H	3.606063	2.142704	2.754804
H	3.629608	1.394916	1.159388
H	0.391464	0.826908	3.993343
H	1.967041	0.061162	4.248483
H	1.804922	1.818090	4.333145
H	-0.031663	2.176682	1.844734
H	1.364828	3.168192	2.247707
H	1.269558	2.398005	0.667834
H	-0.341447	-1.562533	2.149398
H	-0.687881	0.168043	2.292740
H	-1.903639	3.407196	-2.626108
H	-0.438213	2.507028	-3.077227
H	-0.392782	3.631972	-1.695334

ωB97X energy = -1507.41159477 a.u.

(5*S*,1'*S*)-**8a**, Conf. E

C	2.369032	1.914404	-0.330989
C	3.021424	3.141026	-0.288188
C	3.954565	3.392869	0.711327
C	4.238231	2.436214	1.669646
C	3.576973	1.215062	1.621781
C	2.647703	0.932754	0.632525
C	1.976659	-0.425112	0.602068
C	2.336930	-1.280873	-0.621820
C	3.816988	-1.570599	-0.661458
C	4.666768	-0.815816	-1.464818
C	6.033778	-1.058888	-1.474156
C	6.567532	-2.062448	-0.677071
C	5.727404	-2.823164	0.125646
C	4.361330	-2.577334	0.131918
N	0.523927	-0.288034	0.759191
C	-0.395690	-0.408921	-0.154353
N	-1.616455	-0.158768	0.270346
C	-2.756675	-0.091550	-0.612047
C	-3.260280	-1.271193	-1.180567
C	-2.615110	-2.636761	-1.004997
C	-3.597309	-3.673930	-0.455951
C	-2.004276	-3.118786	-2.324270
C	-4.397618	-1.170513	-1.975933
C	-5.015248	0.047660	-2.195342
C	-4.472305	1.202233	-1.661022
C	-3.319185	1.163765	-0.883405
C	-2.709136	2.475559	-0.415178
C	-2.419235	3.397962	-1.602921
C	-3.601137	3.186172	0.605728
C	-1.589612	0.207863	1.721171
C	-2.387351	-0.704862	2.682496
C	-3.878639	-0.686679	2.331484
C	-2.230086	-0.101079	4.085296
C	-1.877363	-2.146194	2.687231
C	-0.065480	0.245580	1.991414
C	1.185345	2.514692	-2.319058
O	1.446009	1.584912	-1.274857
H	2.812491	3.903858	-1.025955
H	4.458006	4.352523	0.732151
H	4.964779	2.633261	2.448186
H	3.792840	0.457087	2.368082
H	2.306300	-0.970765	1.490776
H	1.779220	-2.219851	-0.561683
H	2.043218	-0.766013	-1.537129
H	4.253665	-0.030008	-2.090017
H	6.682534	-0.463121	-2.106221
H	7.634492	-2.253934	-0.684119
H	6.136075	-3.612511	0.746387
H	3.709124	-3.179667	0.757378
H	-0.186597	-0.683666	-1.179135
H	-1.800578	-2.549831	-0.285465
H	-3.077463	-4.615470	-0.266131
H	-4.053556	-3.343907	0.479868
H	-4.399733	-3.877056	-1.168447
H	-1.268873	-2.409514	-2.711941
H	-2.775782	-3.248189	-3.087275
H	-1.507081	-4.080414	-2.180630
H	-4.810573	-2.065243	-2.427724
H	-5.911772	0.099748	-2.802213
H	-4.941223	2.157333	-1.869140
H	-1.745564	2.265517	0.053152
H	-1.880686	4.285329	-1.262698
H	-1.810198	2.894676	-2.356387
H	-3.341127	3.732996	-2.083382
H	-3.122855	4.104272	0.953207
H	-3.809405	2.561098	1.477398
H	-4.560262	3.455855	0.156624
H	-2.005392	1.210780	1.816985
H	-4.267592	0.334684	2.293991
H	-4.437587	-1.226762	3.098764
H	-4.087896	-1.161806	1.373267
H	-1.195235	-0.111605	4.434102
H	-2.589531	0.931149	4.112776
H	-2.820397	-0.680481	4.797627
H	-0.825464	-2.218425	2.972971
H	-2.449358	-2.731337	3.410437
H	-1.998799	-2.623905	1.714430
H	0.302068	1.259809	2.155268
H	0.233977	-0.372334	2.836673
H	0.754218	3.438476	-1.924685
H	0.467205	2.030410	-2.976824
H	2.096432	2.740578	-2.878329

ωB97X energy = -1507.41131539 a.u.

(5*S*,1'*R*)-**8b**, Conf. A

C	-3.752548	0.892950	0.783570
C	-4.883267	1.662870	0.539355
C	-5.834047	1.217898	-0.372270
C	-5.666821	0.016417	-1.036592
C	-4.532199	-0.747663	-0.787521
C	-3.567104	-0.329068	0.114540

C	-2.310722	-1.122976	0.406335
C	-2.337715	-2.582540	-0.062613
C	-1.087334	-3.331665	0.327533
C	-0.167757	-3.731369	-0.636335
C	0.997527	-4.398817	-0.277624
C	1.254022	-4.678215	1.056236
C	0.340403	-4.287792	2.028226
C	-0.819462	-3.619344	1.665753
N	-1.149878	-0.421079	-0.157114
C	-0.079379	-0.087289	0.502428
N	0.849946	0.497311	-0.224423
C	2.002626	1.143314	0.358623
C	1.968642	2.531593	0.550328
C	0.718654	3.375110	0.349647
C	0.325012	4.069186	1.657002
C	0.889666	4.399979	-0.773563
C	3.129571	3.150327	1.003129
C	4.262824	2.414558	1.301623
C	4.241874	1.036170	1.184623
C	3.113988	0.368149	0.717261
C	3.131192	-1.150968	0.674232
C	4.275132	-1.696340	-0.183096
C	3.208969	-1.718052	2.095206
C	0.343057	0.704810	-1.617524
C	1.260405	0.281227	-2.786673
C	1.576845	-1.214762	-2.787829
C	2.566474	1.083489	-2.771146
C	0.502993	0.640553	-4.072863
C	-1.014389	-0.042101	-1.566759
C	-2.937723	2.436383	2.410883
O	-2.771531	1.243630	1.655179
H	-5.033197	2.604727	1.049680
H	-6.712874	1.825113	-0.556370
H	-6.409345	-0.329800	-1.744825
H	-4.409413	-1.689036	-1.309324
H	-2.149830	-1.112414	1.486396
H	-3.215862	-3.052279	0.386749
H	-2.468605	-2.634088	-1.145687
H	-0.368880	-3.532165	-1.684320
H	1.701954	-4.703469	-1.043669
H	2.159972	-5.202302	1.339051
H	0.530896	-4.507687	3.072606
H	-1.531650	-3.327772	2.432054
H	0.035736	-0.262351	1.565384
H	-0.112925	2.717778	0.087642
H	-0.621676	4.598973	1.528962
H	0.207210	3.346112	2.467162
H	1.077245	4.799578	1.962883
H	1.684053	5.110897	-0.533967
H	-0.034819	4.964039	-0.913364
H	1.143693	3.925236	-1.724269
H	3.140146	4.225324	1.144558
H	5.157609	2.915197	1.653099
H	5.119948	0.464593	1.463571
H	2.195027	-1.503181	0.236989
H	4.188095	-2.782299	-0.269299
H	4.265125	-1.274836	-1.190591
H	5.246787	-1.475157	0.264753
H	3.182705	-2.809240	2.065834
H	2.372661	-1.375685	2.709944
H	4.135647	-1.412739	2.587512
H	0.163184	1.776210	-1.732917
H	2.309567	-1.428893	-3.568764
H	0.696469	-1.824273	-3.000805
H	1.997531	-1.548004	-1.838077
H	2.375968	2.157182	-2.691773
H	3.224548	0.796173	-1.950966
H	3.105154	0.909675	-3.705109
H	0.297380	1.713189	-4.123195
H	1.110254	0.374150	-4.940152
H	-0.445519	0.105180	-4.159009
H	-1.849790	0.590459	-1.864370
H	-1.018798	-0.939416	-2.187227
H	-2.072040	2.499325	3.065610
H	-3.848382	2.395823	3.013423
H	-2.965642	3.313351	1.758765

ωB97X energy = -1507.41475154 a.u.

(5*S*,1’*R*)-**8b**, Conf. B

C	3.288086	1.690710	-0.135593
C	3.804524	2.918817	0.256095
C	4.117209	3.137553	1.593455
C	3.923383	2.143817	2.534960
C	3.406899	0.915435	2.134760
C	3.081053	0.671133	0.810209
C	2.507541	-0.644093	0.323396
C	2.582444	-1.801003	1.327930
C	1.988399	-3.070913	0.770616
C	0.719264	-3.491230	1.158462
C	0.139884	-4.619138	0.590272
C	0.828930	-5.345329	-0.370803
C	2.102970	-4.943312	-0.753126
C	2.676839	-3.814761	-0.185811
N	1.118368	-0.452996	-0.136018
C	0.179510	0.175044	0.507079
N	-0.969903	0.242236	-0.133155
C	-2.187386	0.667421	0.516997
C	-3.080693	-0.312814	0.971301
C	-2.773149	-1.802350	0.944010
C	-2.754186	-2.378459	2.362447
C	-3.749414	-2.574303	0.054785
C	-4.284079	0.114906	1.523104
C	-4.566908	1.461912	1.665875
C	-3.635117	2.408730	1.279422
C	-2.425849	2.036565	0.699595
C	-1.420043	3.118195	0.341429
C	-2.012791	4.166592	-0.602201
C	-0.865374	3.781486	1.605808
C	-0.869898	-0.488532	-1.435723
C	-1.304323	0.272219	-2.708872
C	-0.494915	1.546038	-2.950091

C	-2.796831	0.613299	-2.648717
C	-1.086649	-0.697193	-3.879818
C	0.617258	-0.926919	-1.429895
C	3.213951	2.346203	-2.429468
O	2.951506	1.383981	-1.415185
H	3.968720	3.706901	-0.466309
H	4.519469	4.099281	1.890317
H	4.170092	2.314411	3.575530
H	3.265023	0.139938	2.877627
H	3.049313	-0.933254	-0.579620
H	3.633796	-1.952109	1.583096
H	2.058874	-1.529027	2.247434
H	0.177645	-2.933182	1.917571
H	-0.850544	-4.933134	0.902370
H	0.379277	-6.225763	-0.815008
H	2.651940	-5.511947	-1.495053
H	3.672913	-3.507802	-0.490577
H	0.322140	0.606816	1.490900
H	-1.768941	-1.948154	0.542825
H	-2.468786	-3.433225	2.334492
H	-2.040356	-1.846984	2.995991
H	-3.737300	-2.314268	2.834079
H	-4.768654	-2.507397	0.442863
H	-3.473382	-3.630603	0.018214
H	-3.757035	-2.189303	-0.967936
H	-5.003118	-0.619773	1.867956
H	-5.508270	1.774816	2.102578
H	-3.849939	3.460455	1.431853
H	-0.577806	2.651959	-0.171140
H	-1.239576	4.875284	-0.906829
H	-2.429703	3.710813	-1.503179
H	-2.808673	4.734063	-0.114633
H	-0.111695	4.525900	1.339623
H	-0.401833	3.052533	2.274925
H	-1.658107	4.287905	2.162083
H	-1.511998	-1.368581	-1.363588
H	-0.813844	2.005031	-3.888212
H	0.576129	1.349757	-3.031628
H	-0.644047	2.280706	-2.158684
H	-3.400465	-0.277852	-2.455052
H	-3.025789	1.350613	-1.878554
H	-3.113597	1.027193	-3.608563
H	-1.641766	-1.627779	-3.733396
H	-1.441278	-0.237150	-4.804038
H	-0.032454	-0.946624	-4.021134
H	0.733892	-2.008567	-1.499846
H	1.201841	-0.453297	-2.218496
H	2.924244	1.879501	-3.368034
H	4.276316	2.598567	-2.464072
H	2.622799	3.252440	-2.274377

ωB97X energy = -1507.41306447 a.u.

(5*S*,1’*R*)-**8b**, Conf. C

C	2.437060	-1.651062	-0.140711
C	3.137384	-2.844715	-0.006581
C	4.052455	-2.998948	1.028207
C	4.271387	-1.976600	1.933938
C	3.559971	-0.790895	1.798435
C	2.644972	-0.605824	0.772833
C	1.908637	0.714348	0.662440
C	2.246039	1.526515	-0.595944
C	3.719294	1.845664	-0.656148
C	4.246198	2.898047	0.087968
C	5.606704	3.172428	0.061141
C	6.458154	2.395176	-0.713172
C	5.941467	1.345672	-1.460904
C	4.580276	1.074084	-1.431146
N	0.462464	0.502948	0.800757
C	-0.465728	0.652016	-0.098554
N	-1.660533	0.248496	0.284085
C	-2.870238	0.560863	-0.440391
C	-3.141513	-0.091945	-1.651859
C	-2.172008	-1.040636	-2.337887
C	-2.789314	-2.416239	-2.598412
C	-1.661458	-0.422854	-3.643871
C	-4.354084	0.181902	-2.276781
C	-5.260886	1.070923	-1.728593
C	-4.945675	1.744407	-0.562277
C	-3.737568	1.525429	0.092516
C	-3.402658	2.374830	1.309038
C	-3.403145	3.863271	0.948895
C	-4.354612	2.099496	2.475257
C	-1.583811	-0.347527	1.654282
C	-1.968743	-1.841710	1.767276
C	-1.819856	-2.224485	3.246467
C	-1.077297	-2.743583	0.912125
C	-3.437014	-2.044423	1.379825
C	-0.117630	-0.033482	2.037073
C	1.327888	-2.446865	-2.096807
O	1.533342	-1.421422	-1.132946
H	2.982052	-3.658043	-0.702058
H	4.592423	-3.934382	1.118533
H	4.984503	-2.095800	2.740224
H	3.720678	0.015723	2.506786
H	2.194032	1.316311	1.530000
H	1.958297	0.967629	-1.487430
H	1.671069	2.456274	-0.572339
H	3.585021	3.513407	0.690926
H	6.001985	3.997611	0.642681
H	7.520592	2.609107	-0.736735
H	6.598960	0.736793	-2.071057
H	4.180237	0.252200	-2.017260
H	-0.288640	1.086874	-1.072840
H	-1.309925	-1.188154	-1.685733
H	-2.036966	-3.091053	-3.013965
H	-3.177452	-2.867011	-1.682614
H	-3.609472	-2.353312	-3.317076
H	-2.484240	-0.253372	-4.342265
H	-0.948599	-1.090947	-4.133043
H	-1.168268	0.537016	-3.472528
H	-4.592733	-0.312236	-3.211859

H	-6.206688	1.256326	-2.224320
H	-5.643621	2.471534	-0.162677
H	-2.387970	2.140185	1.633853
H	-4.399590	4.203489	0.658176
H	-3.087430	4.454931	1.810904
H	-2.719269	4.070083	0.123168
H	-4.060897	2.683391	3.350047
H	-4.360763	1.043573	2.757461
H	-5.379141	2.376989	2.215621
H	-2.263477	0.207920	2.300083
H	-2.143141	-3.257501	3.388270
H	-2.441856	-1.588223	3.881979
H	-0.788591	-2.154377	3.598631
H	-1.188273	-2.531847	-0.152593
H	-0.018106	-2.651803	1.168125
H	-1.358033	-3.787055	1.069424
H	-3.622817	-1.839614	0.325722
H	-3.723933	-3.080411	1.572965
H	-4.094792	-1.400736	1.970714
H	-0.037026	0.715512	2.826050
H	0.436395	-0.919704	2.342098
H	0.910124	-3.344679	-1.632797
H	2.258886	-2.695465	-2.611254
H	0.618313	-2.045547	-2.816378

ωB97X energy = -1507.41281419 a.u.
